# Supplementary material for: The role of children in the spread of COVID-19: Using household data from Bnei Brak, Israel, to estimate the relative susceptibility and infectivity of children
Source: PLoS Comput Biol. 2021 Feb 11;17(2):e1008559. doi: 10.1371/journal.pcbi.1008559 (PMC7877572; doi:10.1371/journal.pcbi.1008559)
Supplement: S2 Text — The time-line plots for all households in the study. (PDF) [file pcbi.1008559.s002.pdf]

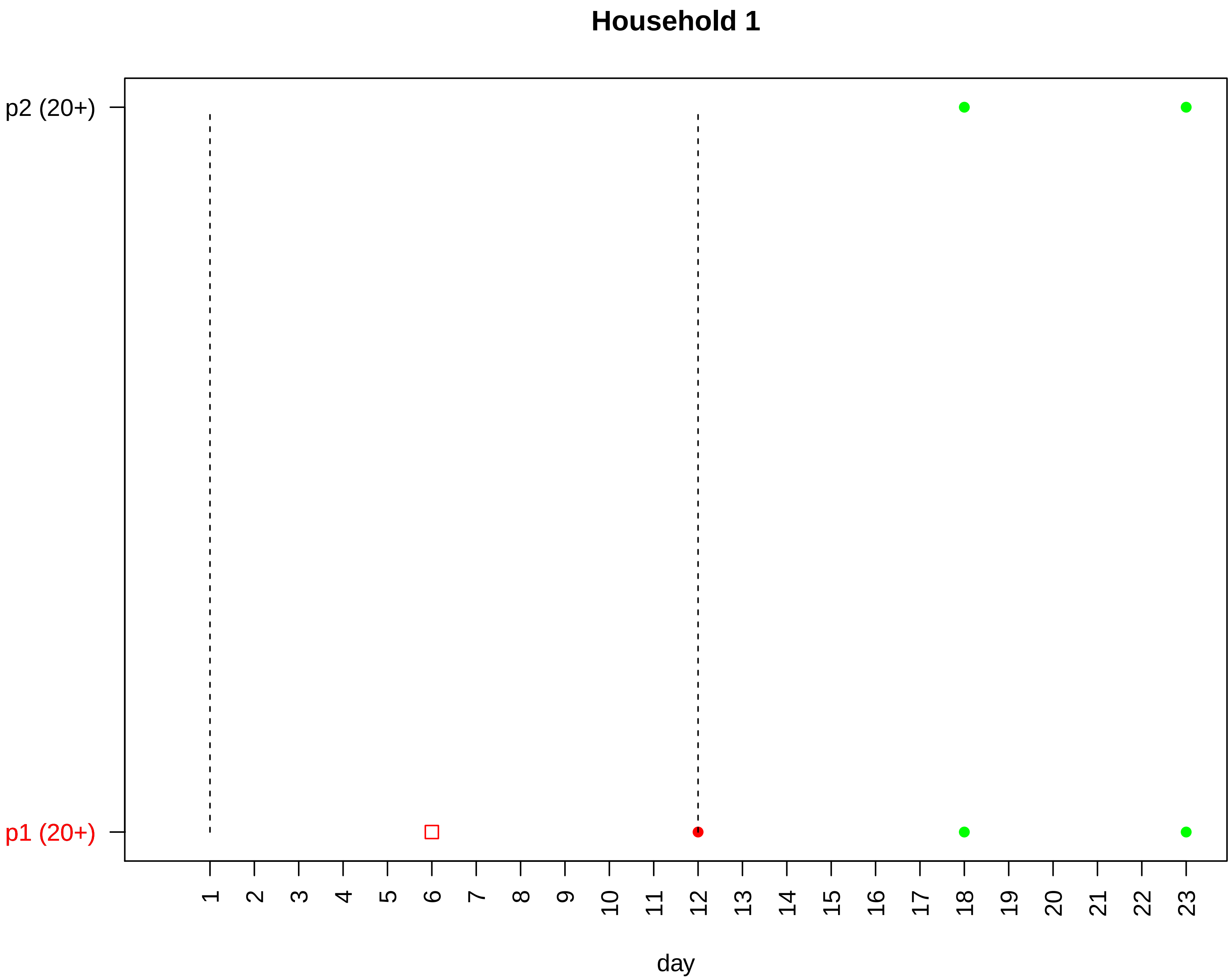

Household 1
p2 (20+)
p1 (20+)
1
2
3
4
5
6
7
8
9
10
11
12
13
14
15
16
17
18
19
20
21
22
23
day

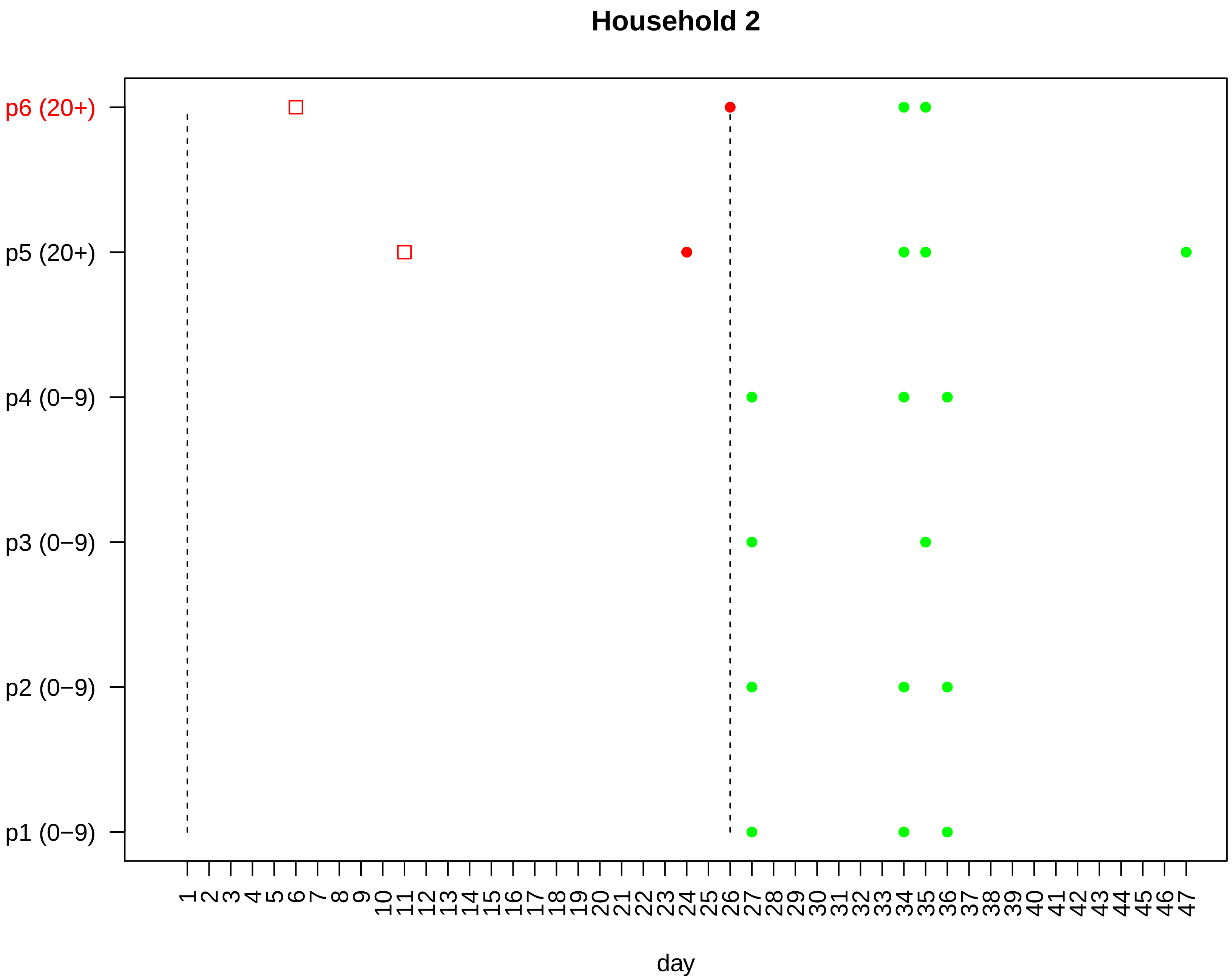

Household 2
p6 (20+)
p5 (20+)
p4 (0−9)
p3 (0−9)
p2 (0−9)
p1 (0−9)
1 2 3 4 5 6 7 8 9 10 11 12 13 14 15 16 17 18 19 20 21 22 23 24 25 26 27 28 29 30 31 32 33 34 35 36 37 38 39 40 41 42 43 44 45 46 47
day

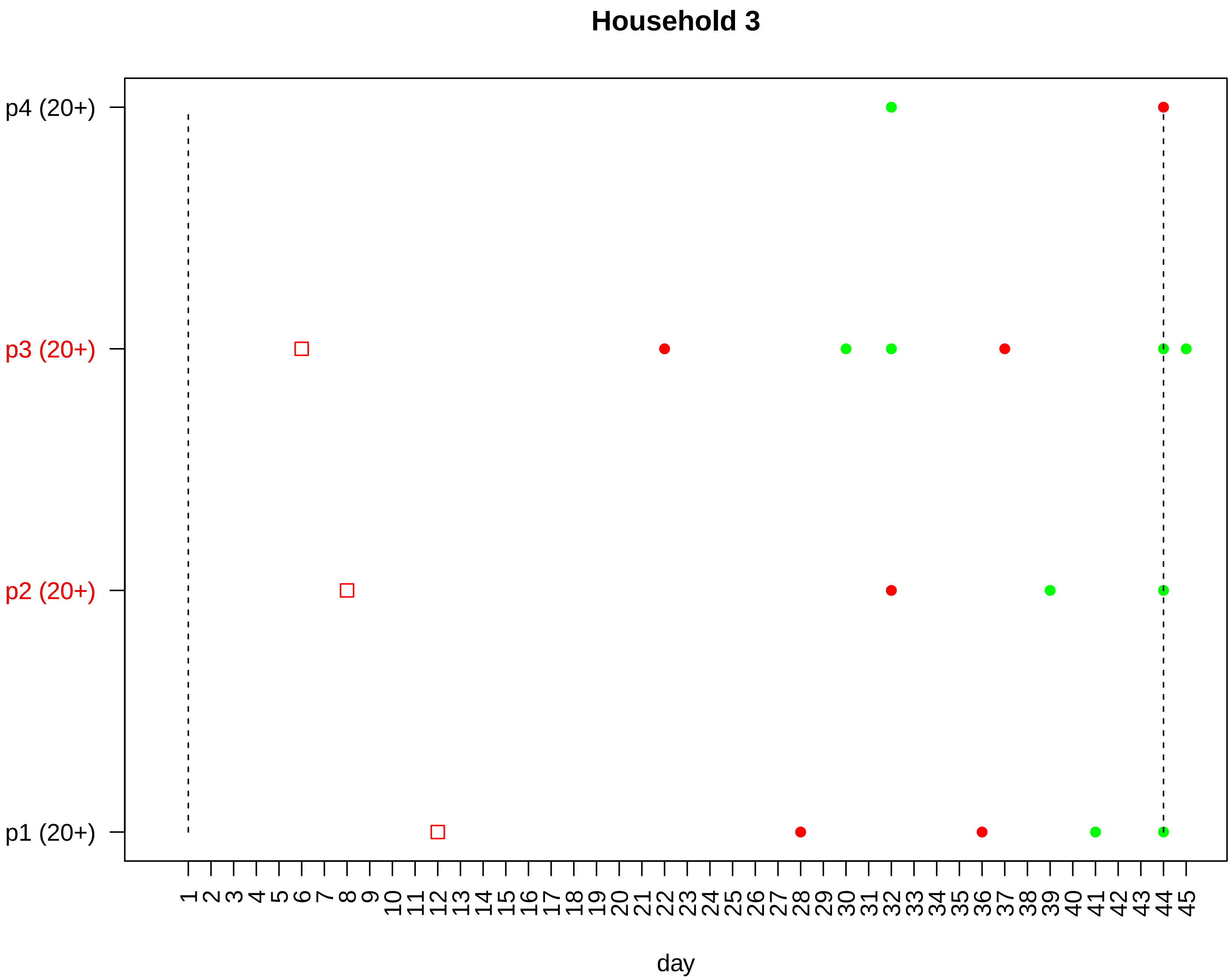
Household 3
p4 (20+)
p3 (20+)
p2 (20+)
p1 (20+)
1 2 3 4 5 6 7 8 9 10 11 12 13 14 15 16 17 18 19 20 21 22 23 24 25 26 27 28 29 30 31 32 33 34 35 36 37 38 39 40 41 42 43 44 45
day

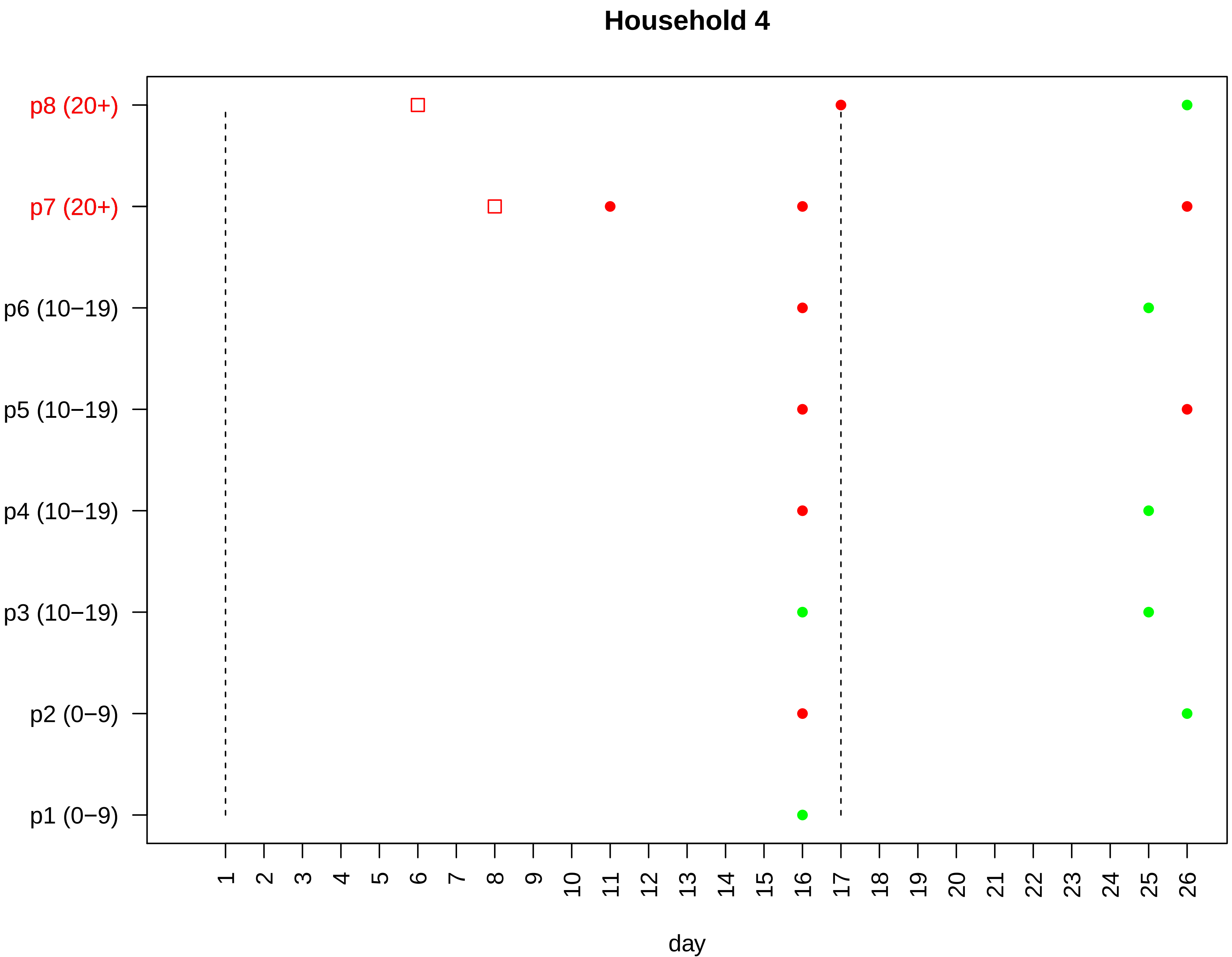
Household 4
p8 (20+)
p7 (20+)
p6 (10−19)
p5 (10−19)
p4 (10−19)
p3 (10−19)
p2 (0−9)
p1 (0−9)
1 2 3 4 5 6 7 8 9 10 11 12 13 14 15 16 17 18 19 20 21 22 23 24 25 26
day

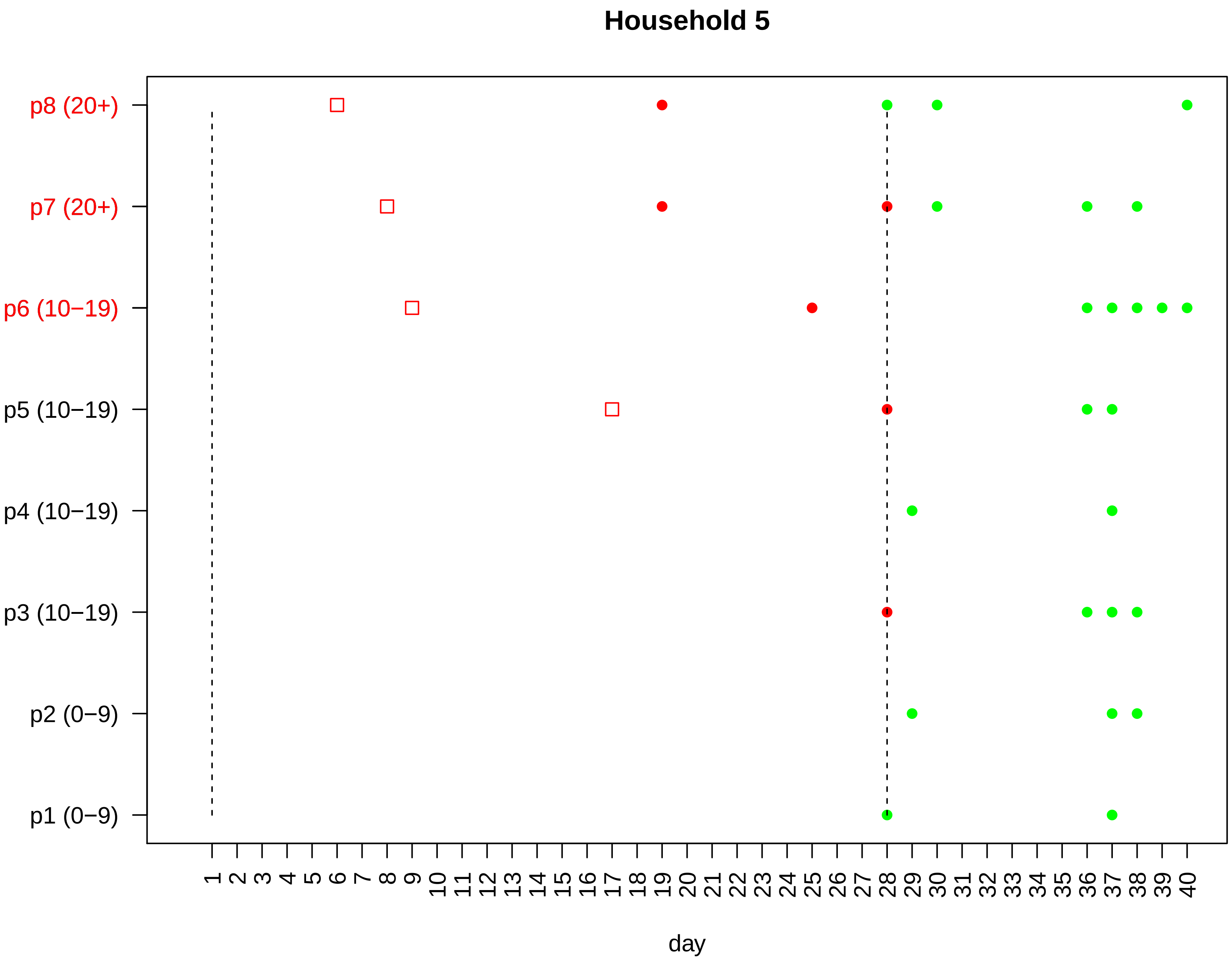

Household 5
p8 (20+)
p7 (20+)
p6 (10−19)
p5 (10−19)
p4 (10−19)
p3 (10−19)
p2 (0−9)
p1 (0−9)
1 2 3 4 5 6 7 8 9 10 11 12 13 14 15 16 17 18 19 20 21 22 23 24 25 26 27 28 29 30 31 32 33 34 35 36 37 38 39 40
day

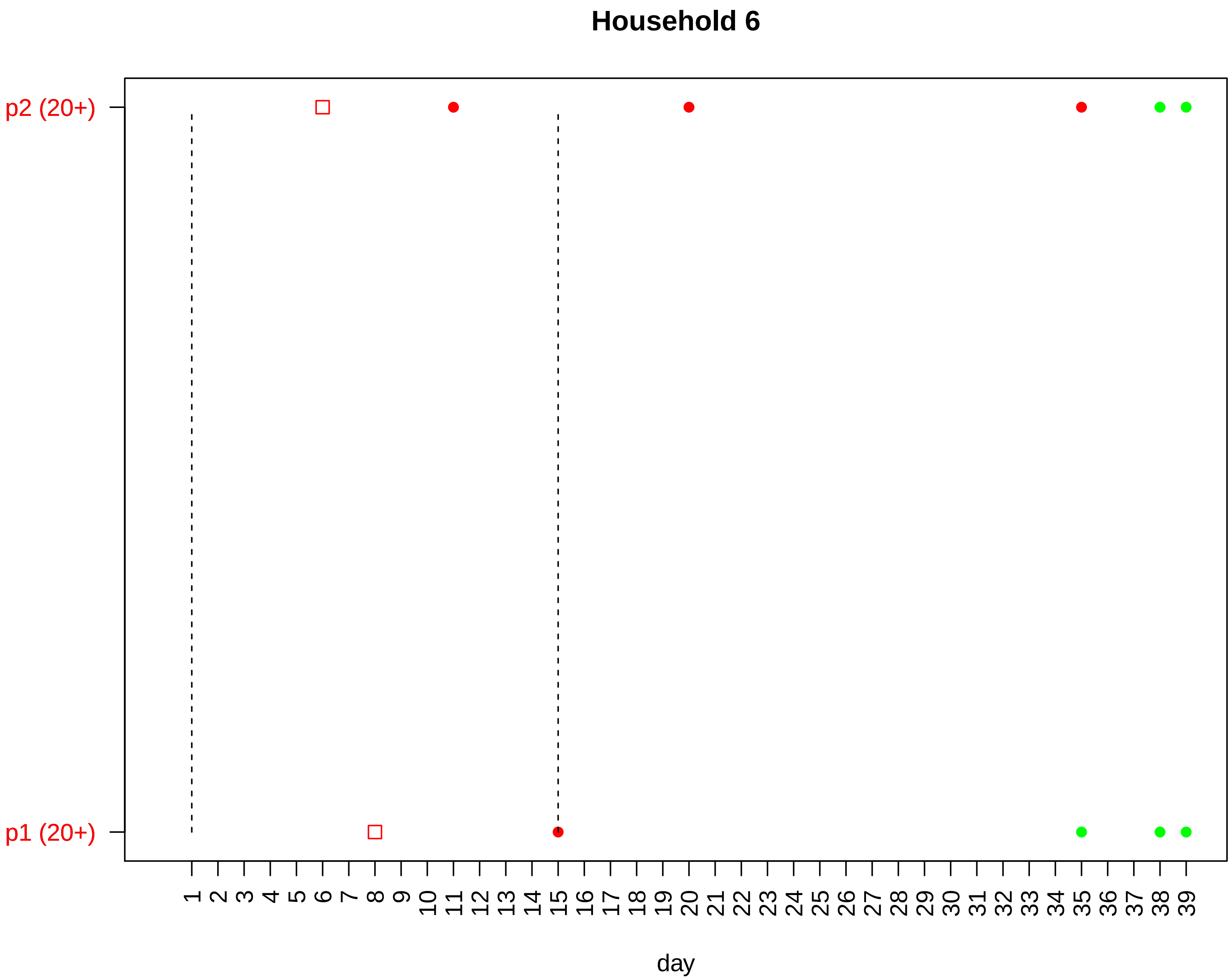

Household 6
p2 (20+)
p1 (20+)
1
2
3
4
5
6
7
8
9
10
11
12
13
14
15
16
17
18
19
20
21
22
23
24
25
26
27
28
29
30
31
32
33
34
35
36
37
38
39
day

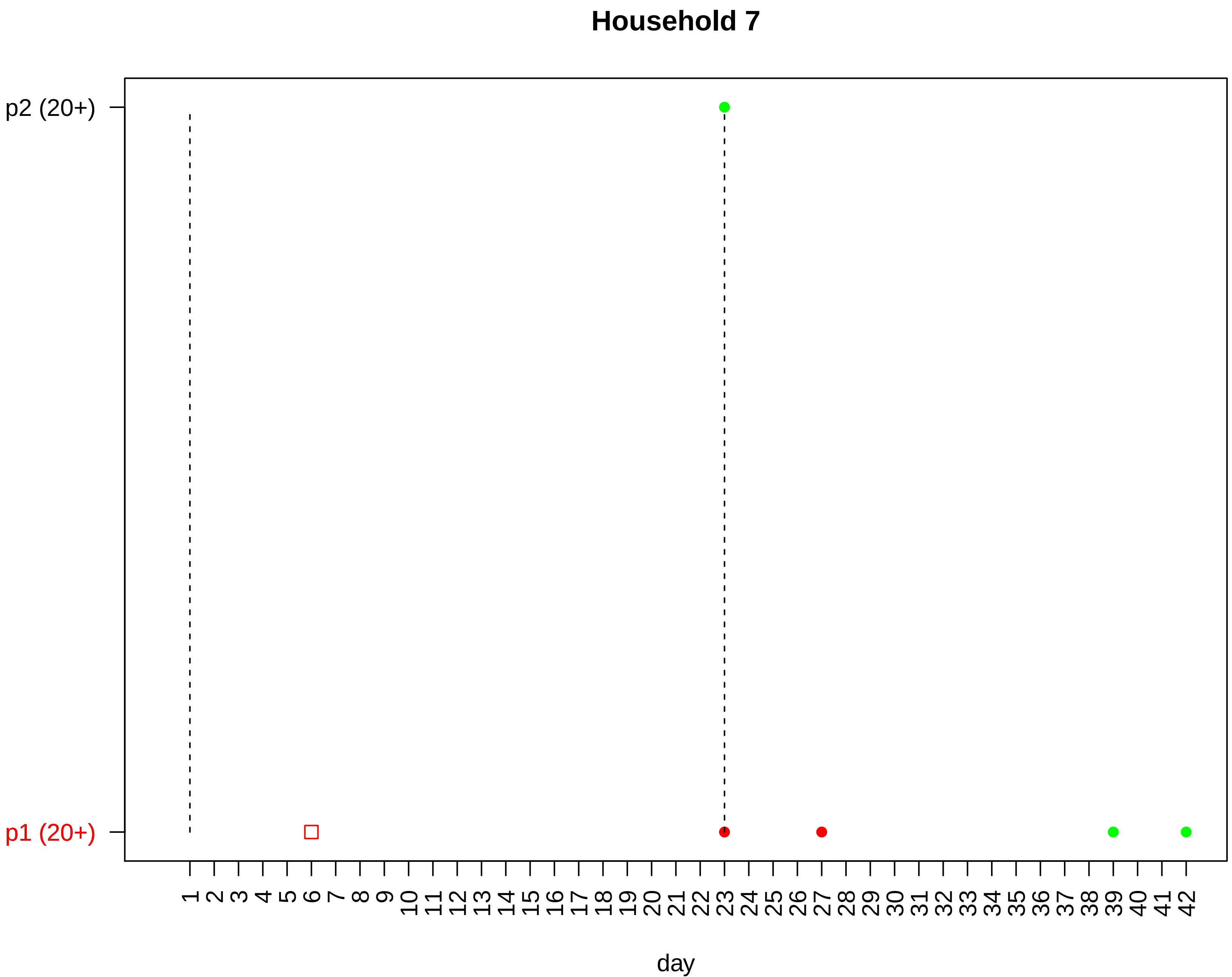

Household 7
p2 (20+)
p1 (20+)
1 2 3 4 5 6 7 8 9 10 11 12 13 14 15 16 17 18 19 20 21 22 23 24 25 26 27 28 29 30 31 32 33 34 35 36 37 38 39 40 41 42
day

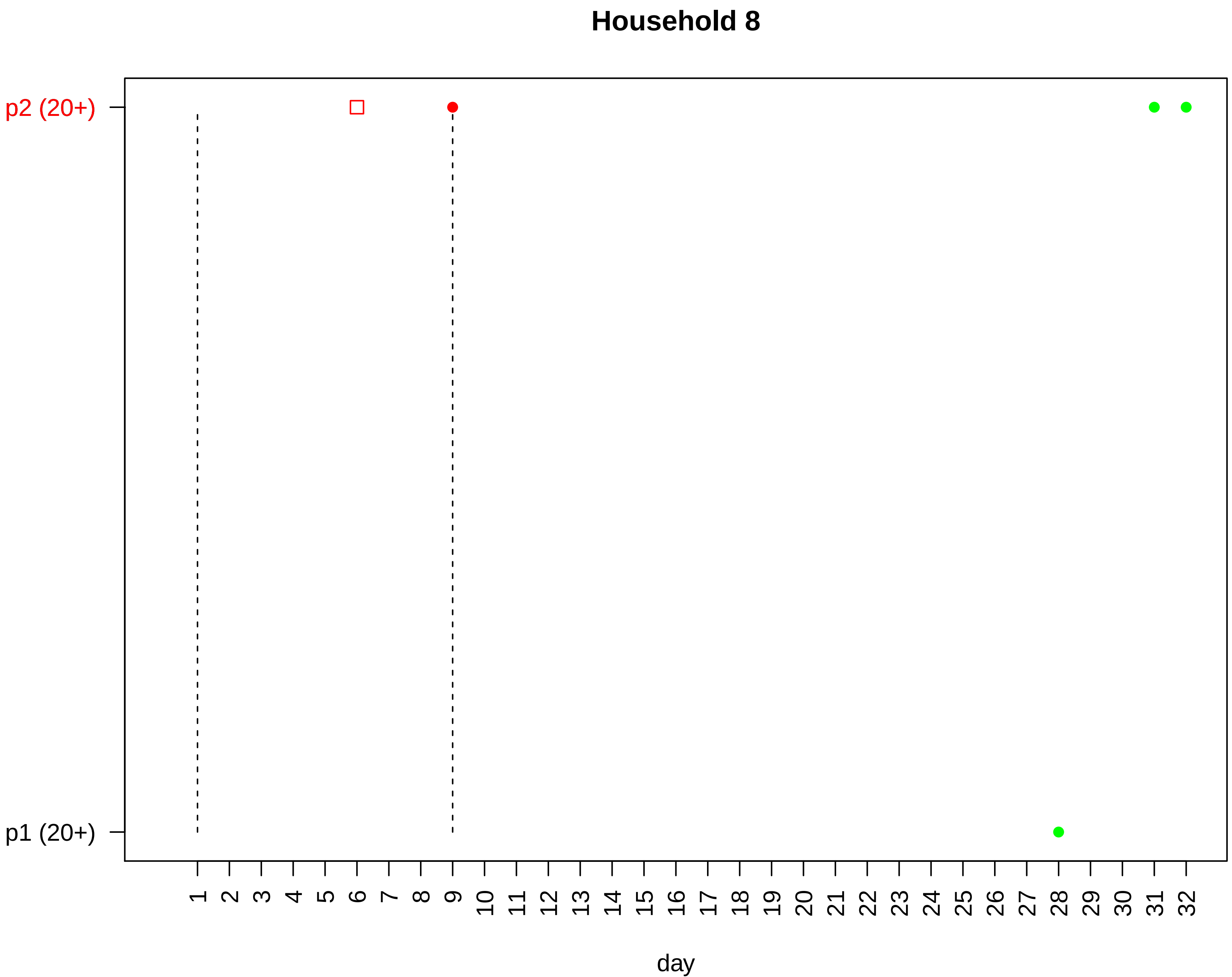

Household 8
p2 (20+)
p1 (20+)
1 2 3 4 5 6 7 8 9 10 11 12 13 14 15 16 17 18 19 20 21 22 23 24 25 26 27 28 29 30 31 32
day

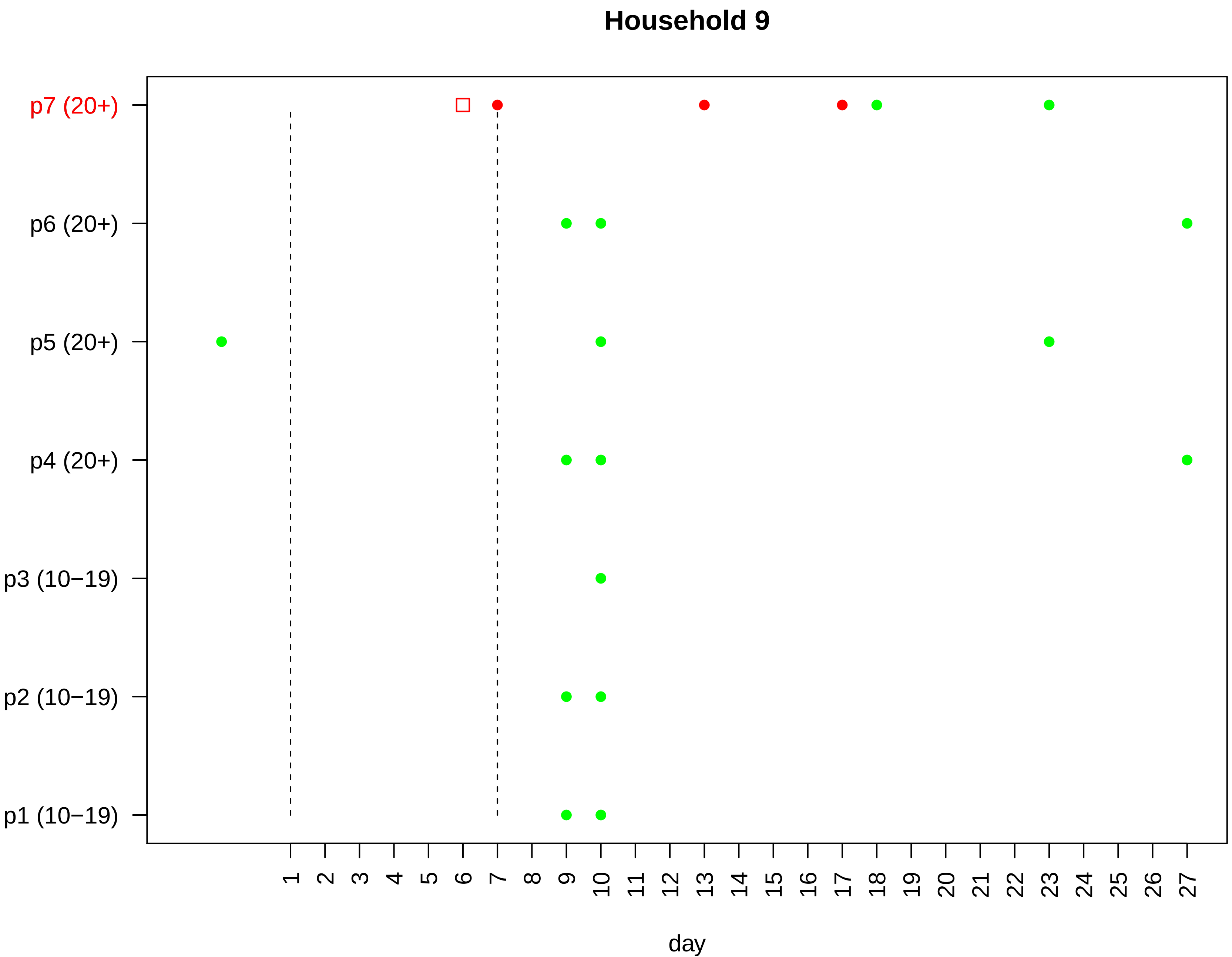

Household 9
p7 (20+)
p6 (20+)
p5 (20+)
p4 (20+)
p3 (10−19)
p2 (10−19)
p1 (10−19)
1 2 3 4 5 6 7 8 9 10 11 12 13 14 15 16 17 18 19 20 21 22 23 24 25 26 27
day

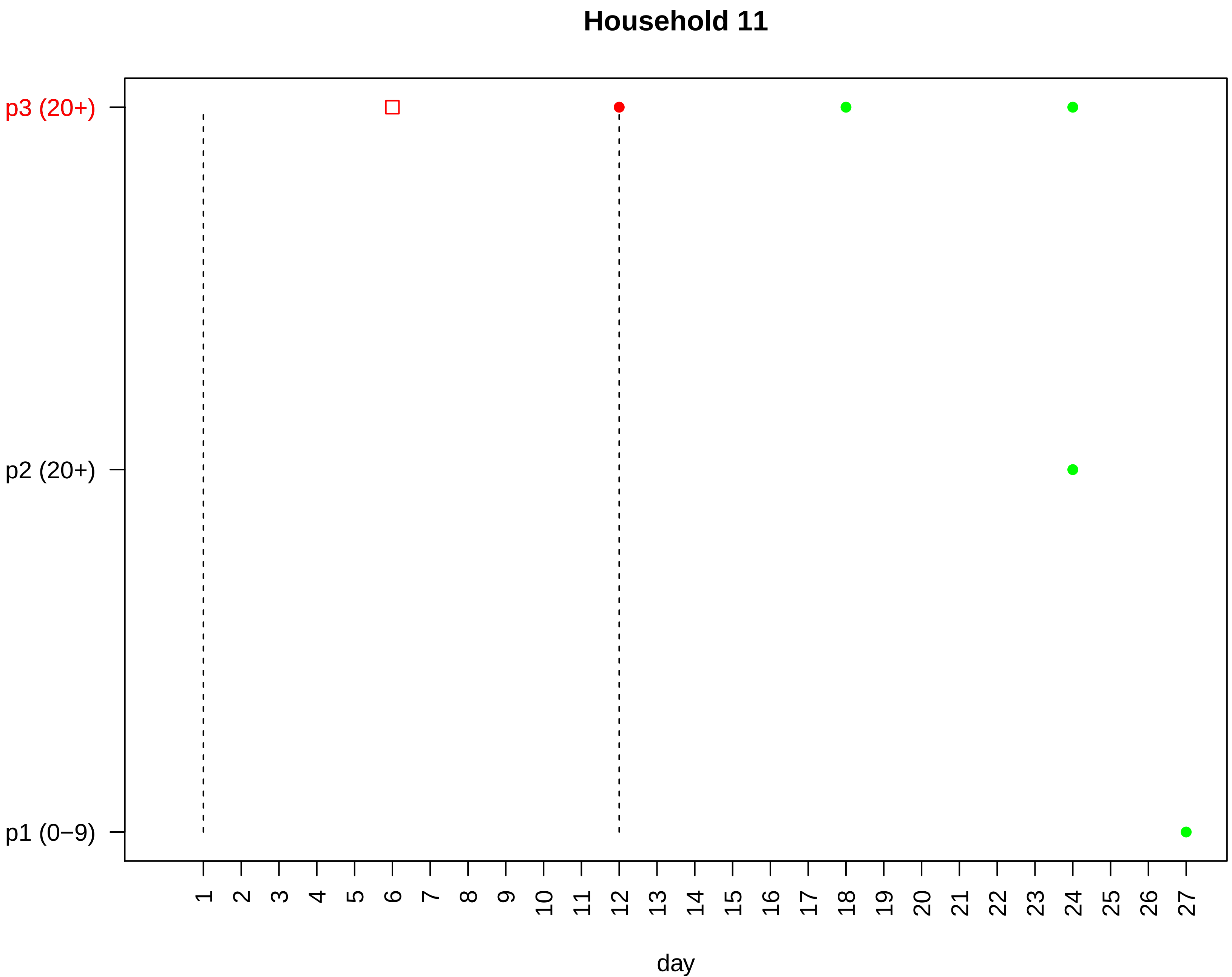

Household 11
p3 (20+)
p2 (20+)
p1 (0−9)
1 2 3 4 5 6 7 8 9 10 11 12 13 14 15 16 17 18 19 20 21 22 23 24 25 26 27
day

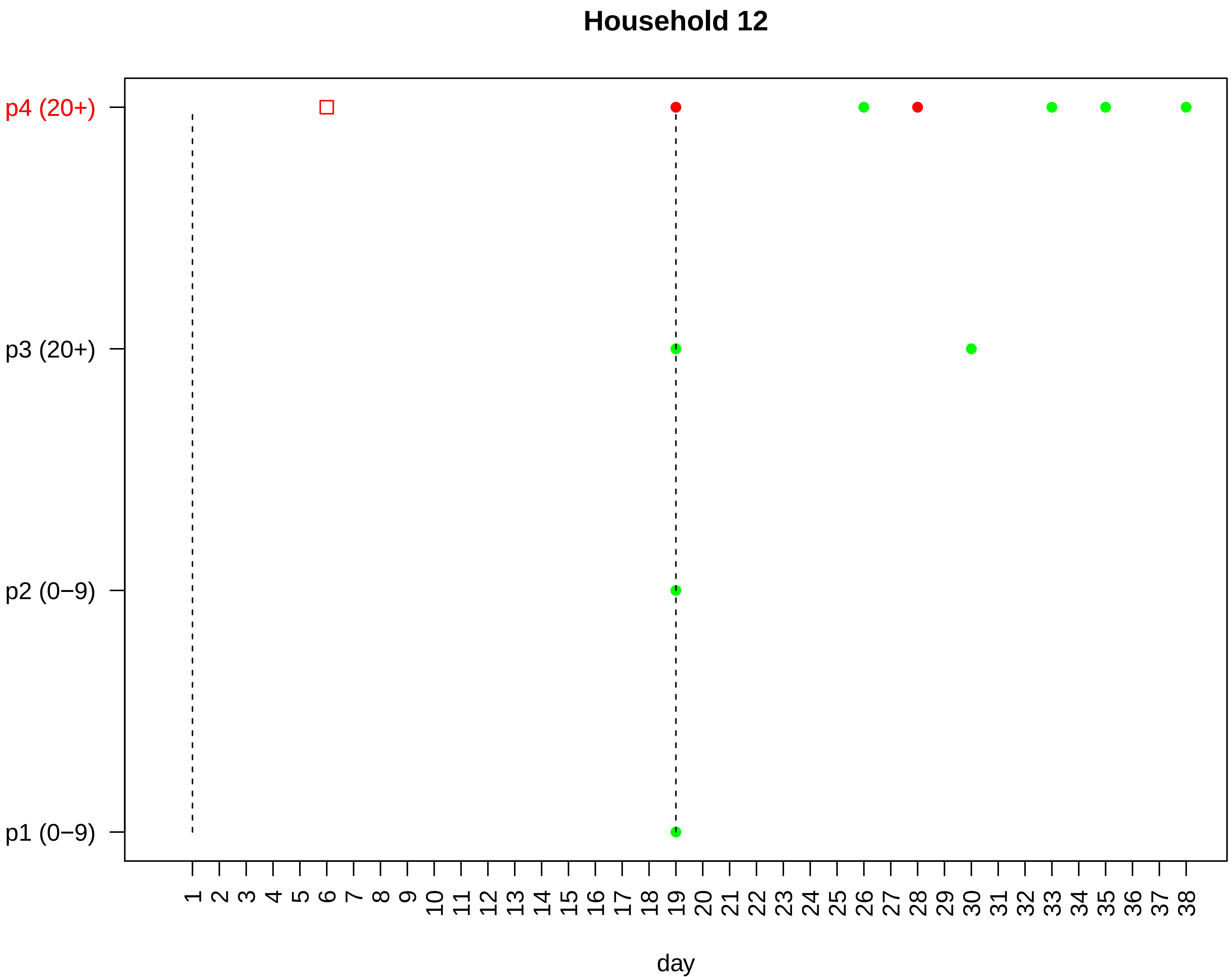
Household 12
p4 (20+)
p3 (20+)
p2 (0−9)
p1 (0−9)
1 2 3 4 5 6 7 8 9 10 11 12 13 14 15 16 17 18 19 20 21 22 23 24 25 26 27 28 29 30 31 32 33 34 35 36 37 38
day

Household 13

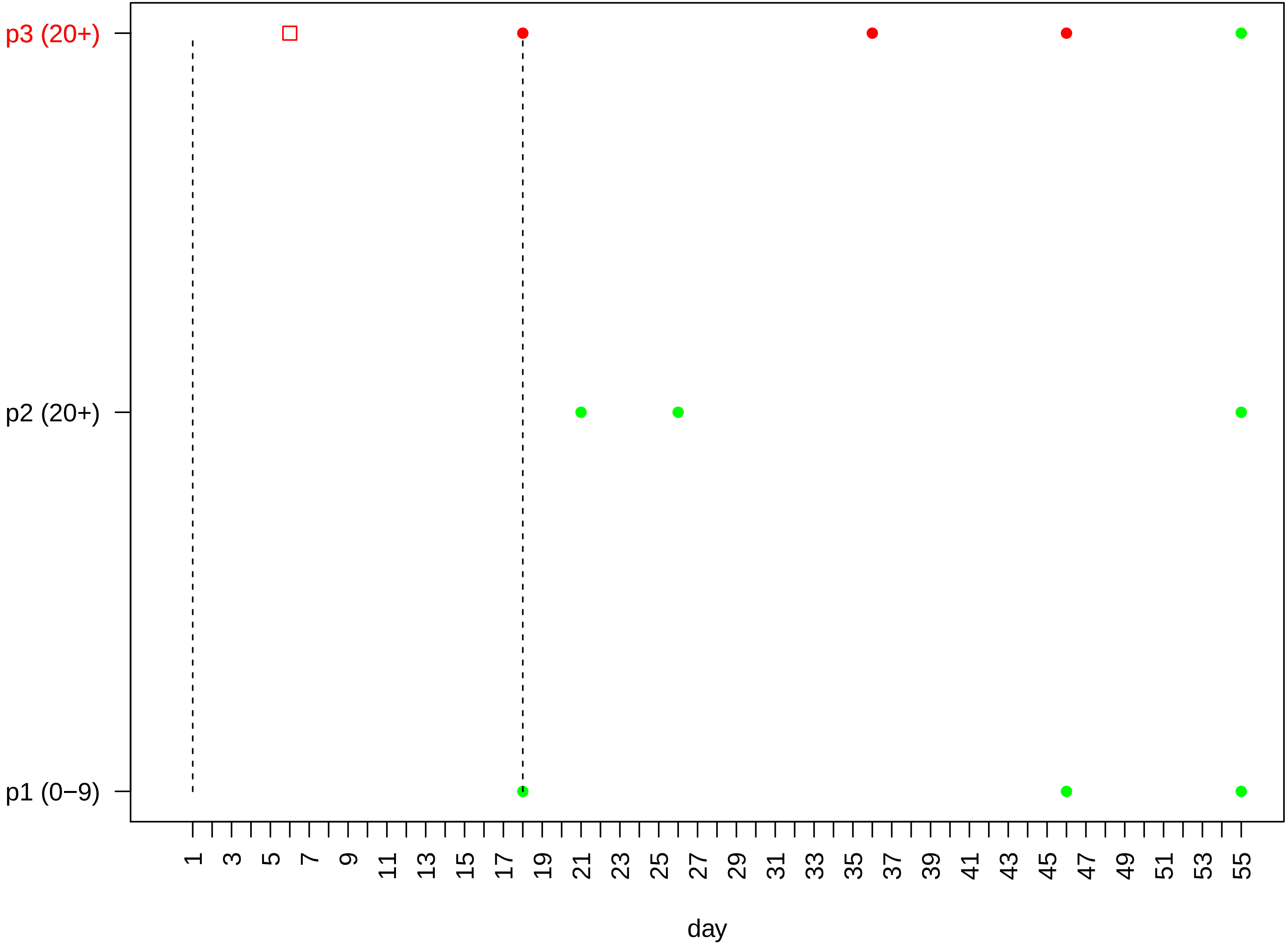

Household 14

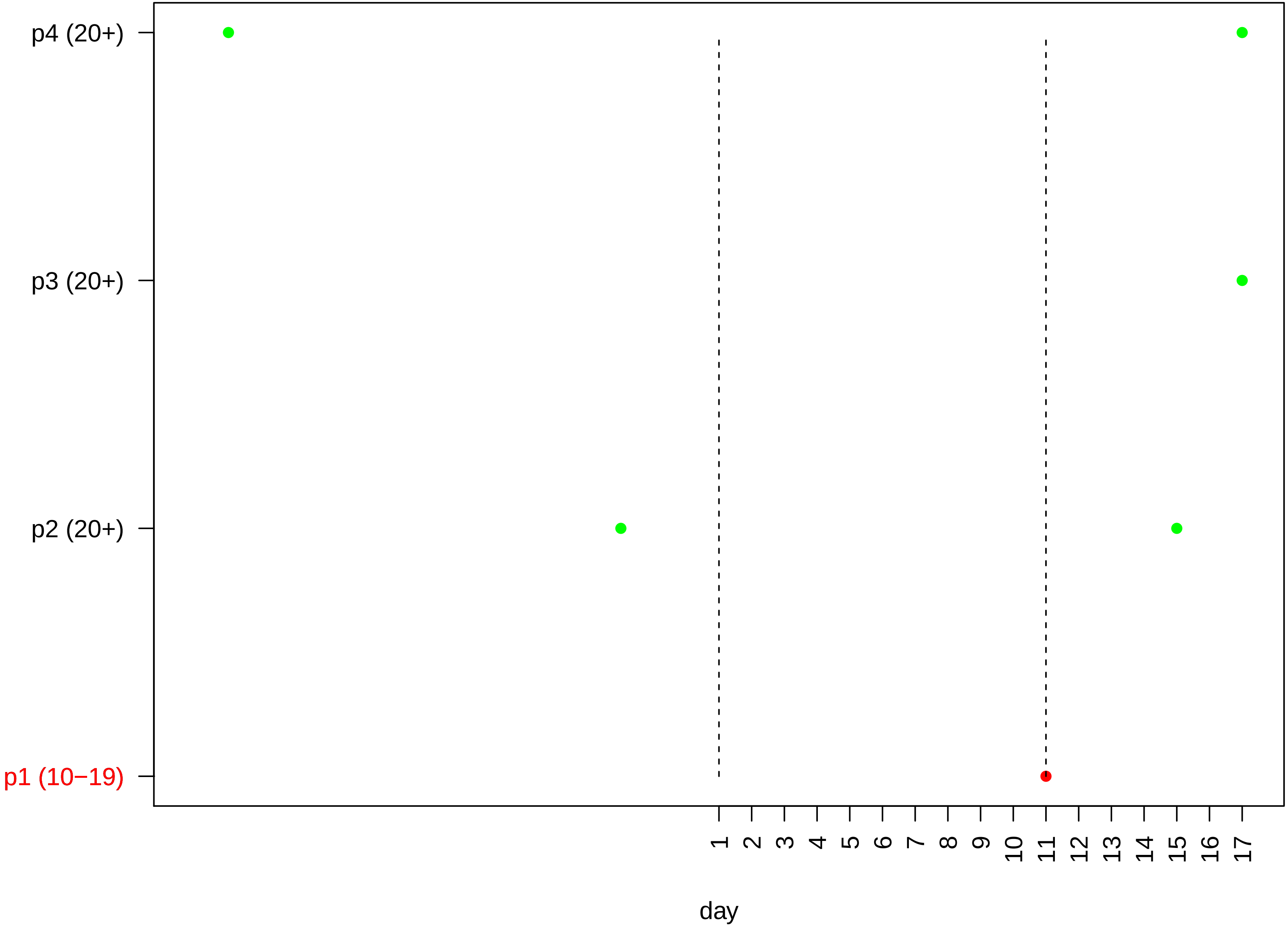

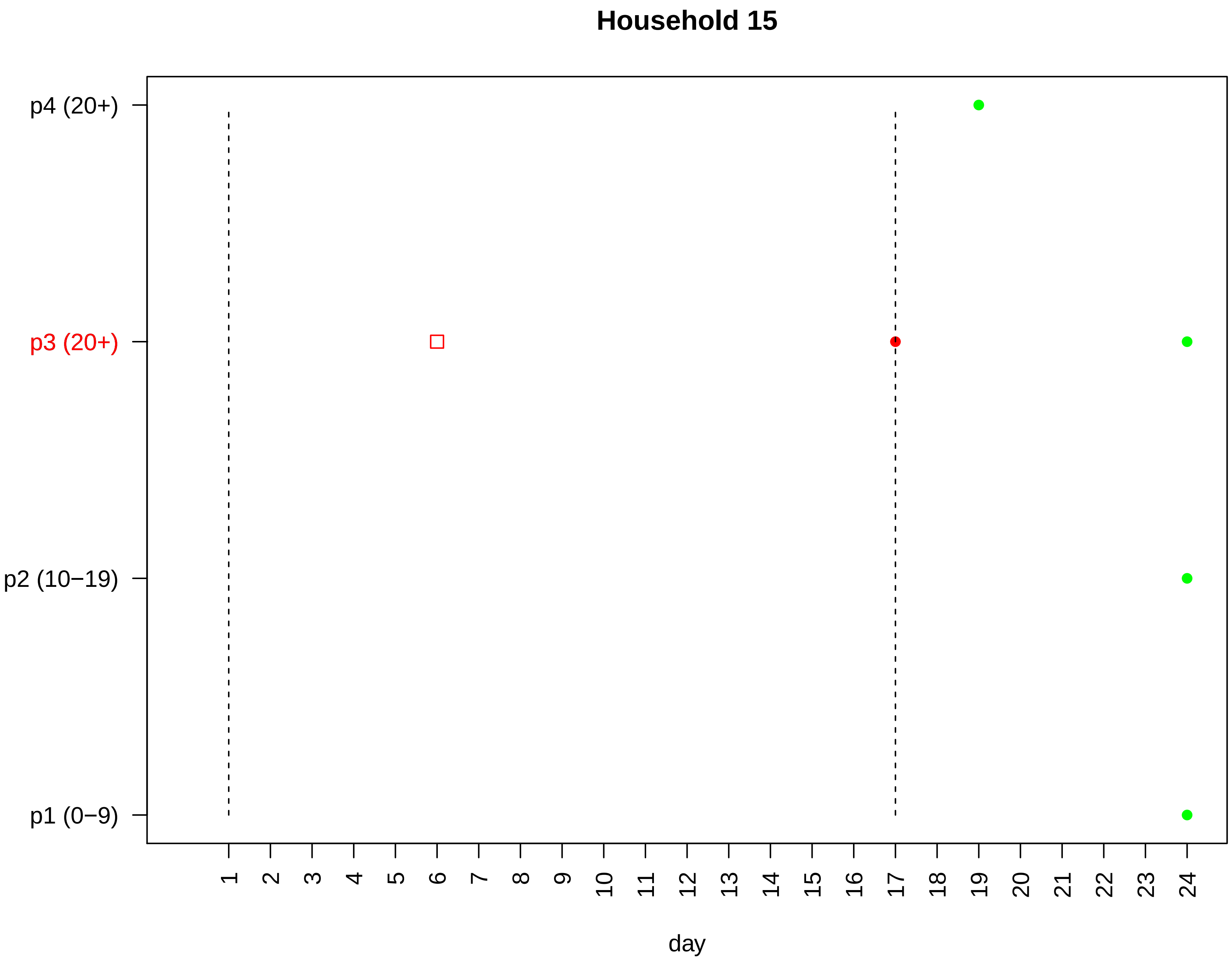

Household 15
p4 (20+)
p3 (20+)
p2 (10−19)
p1 (0−9)
1
2
3
4
5
6
7
8
9
10
11
12
13
14
15
16
17
18
19
20
21
22
23
24
day

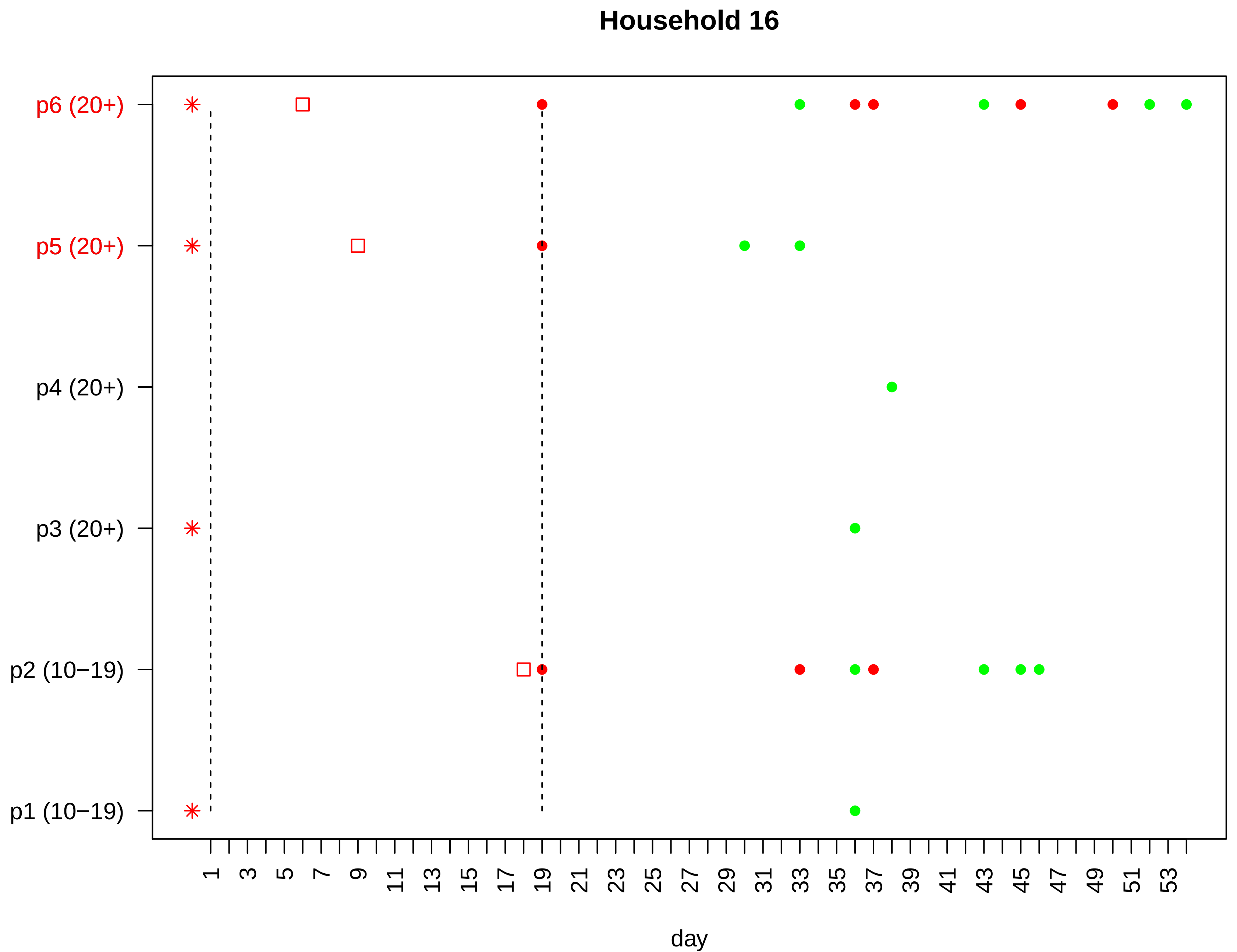

Household 16
p6 (20+)
p5 (20+)
p4 (20+)
p3 (20+)
p2 (10−19)
p1 (10−19)
1 3 5 7 9 11 13 15 17 19 21 23 25 27 29 31 33 35 37 39 41 43 45 47 49 51 53
day

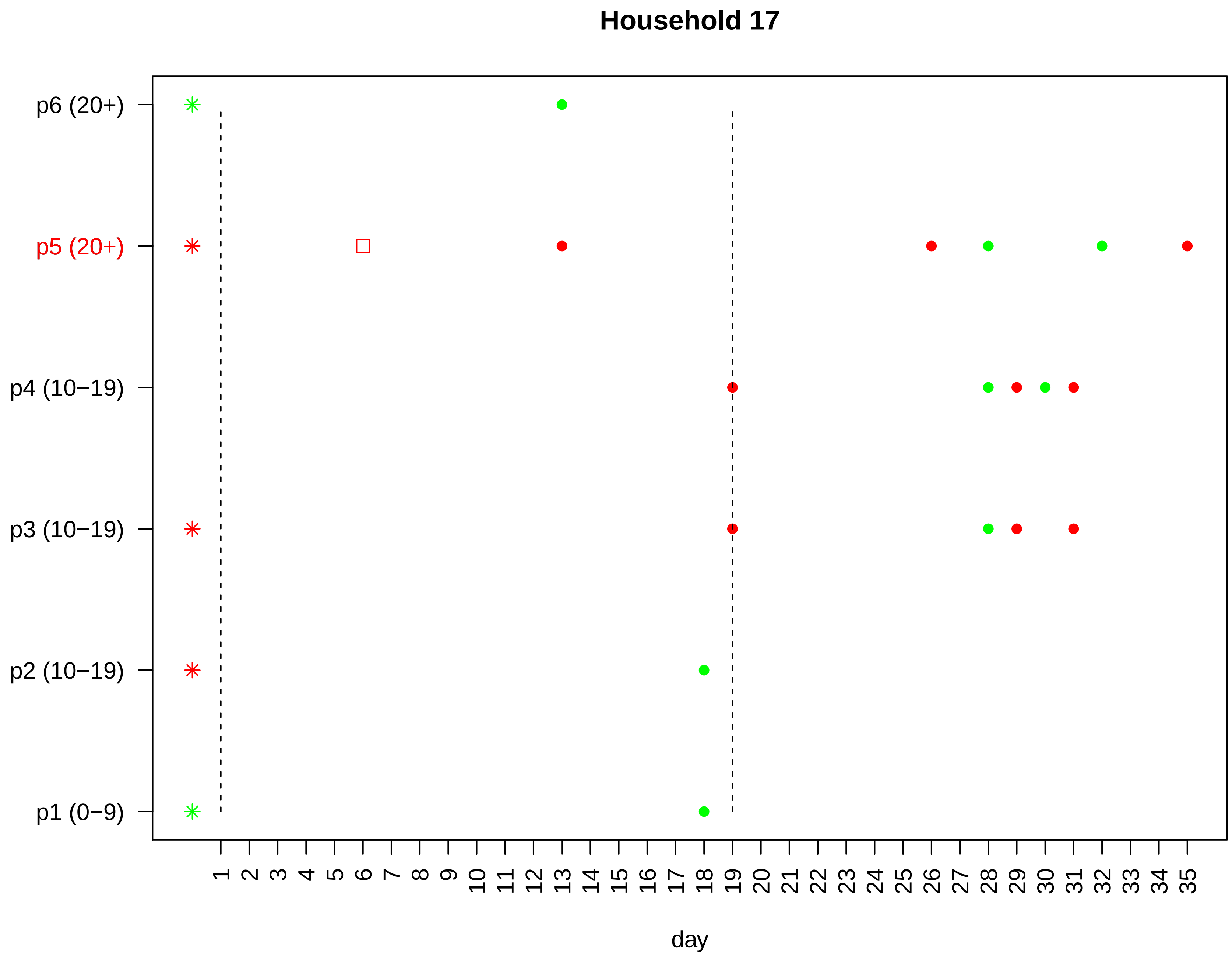
Household 17
p6 (20+)
p5 (20+)
p4 (10−19)
p3 (10−19)
p2 (10−19)
p1 (0−9)
1 2 3 4 5 6 7 8 9 10 11 12 13 14 15 16 17 18 19 20 21 22 23 24 25 26 27 28 29 30 31 32 33 34 35
day

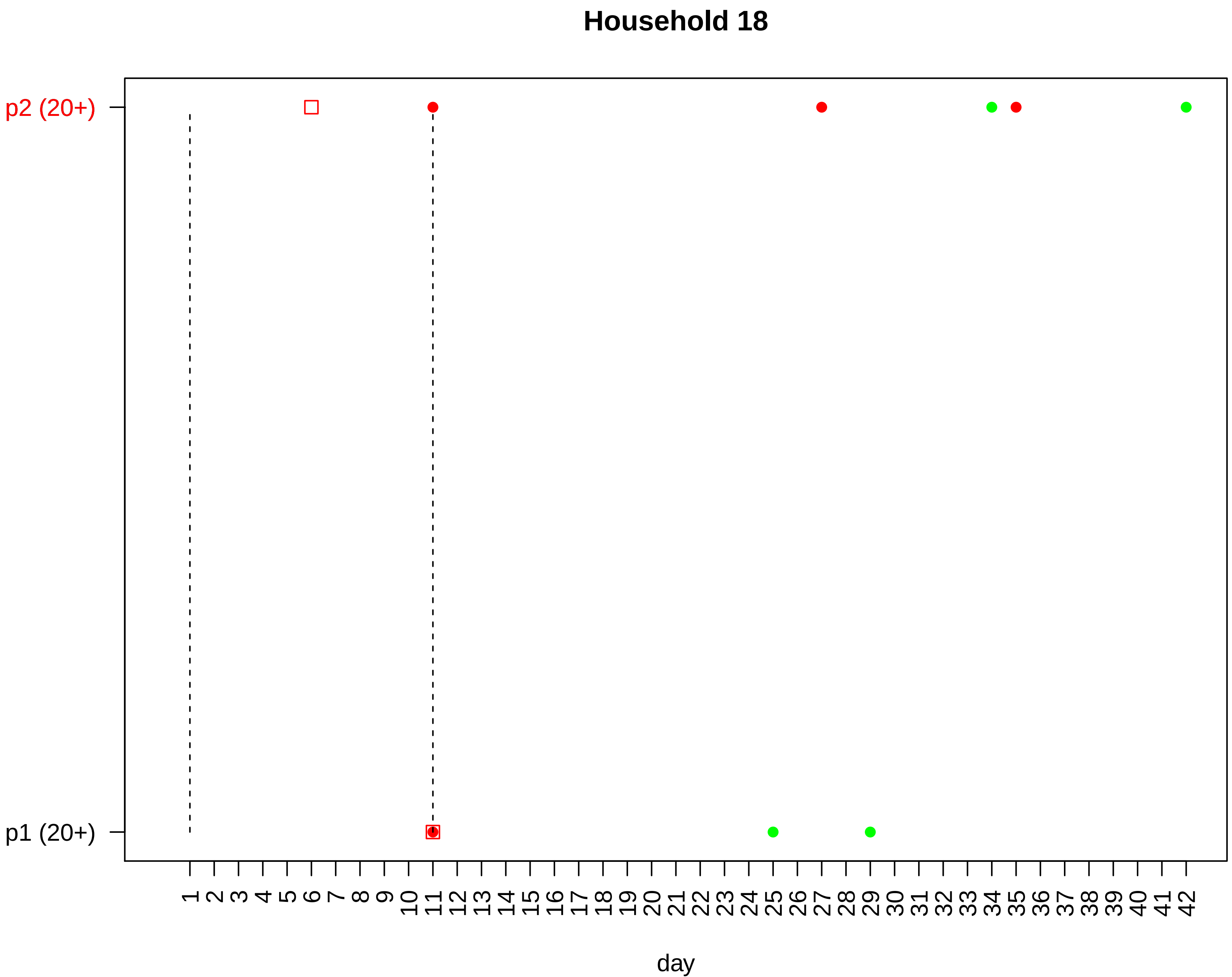

Household 18
p2 (20+)
p1 (20+)
1
2
3
4
5
6
7
8
9
10
11
12
13
14
15
16
17
18
19
20
21
22
23
24
25
26
27
28
29
30
31
32
33
34
35
36
37
38
39
40
41
42
day

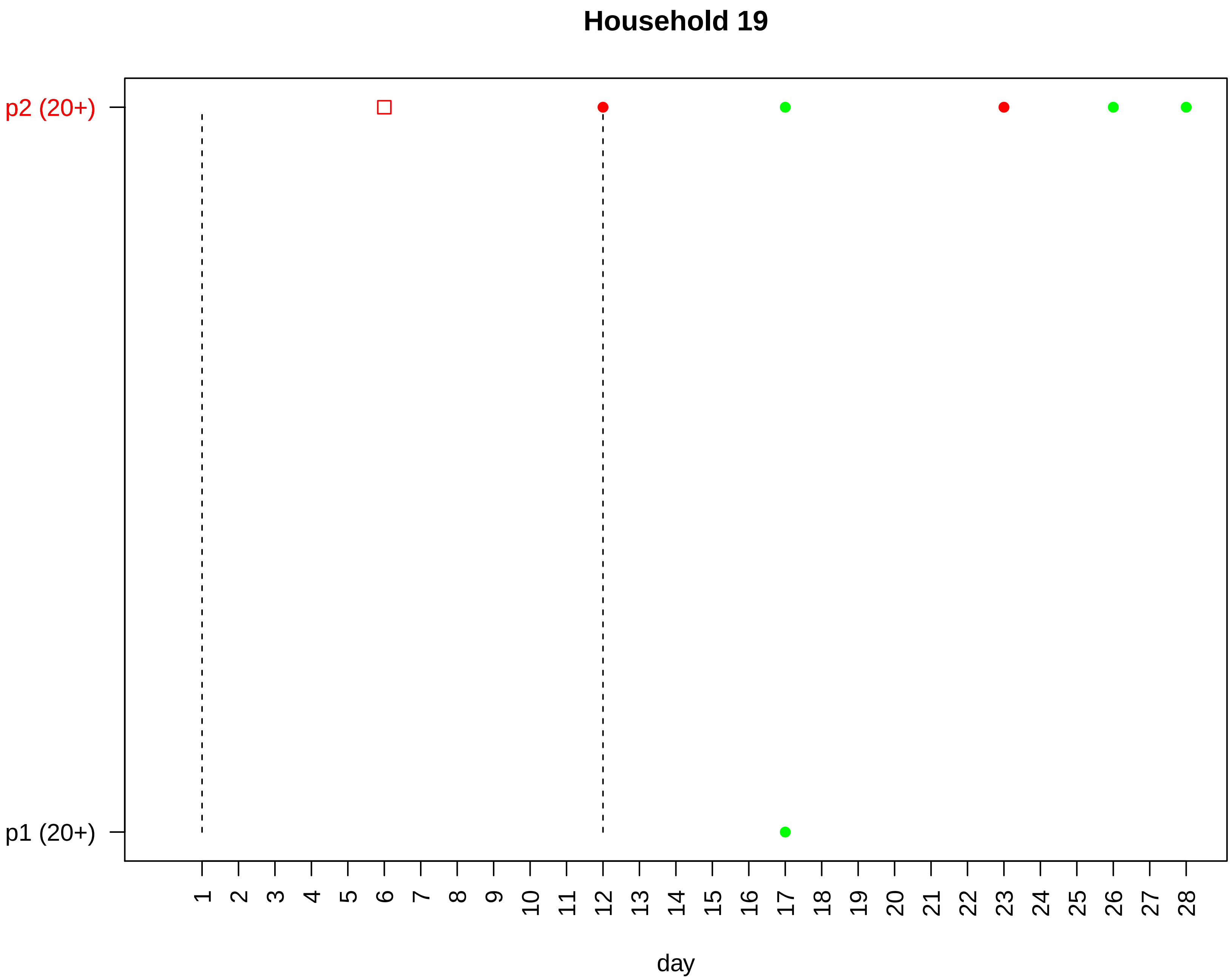

Household 19
p2 (20+)
p1 (20+)
1
2
3
4
5
6
7
8
9
10
11
12
13
14
15
16
17
18
19
20
21
22
23
24
25
26
27
28
day

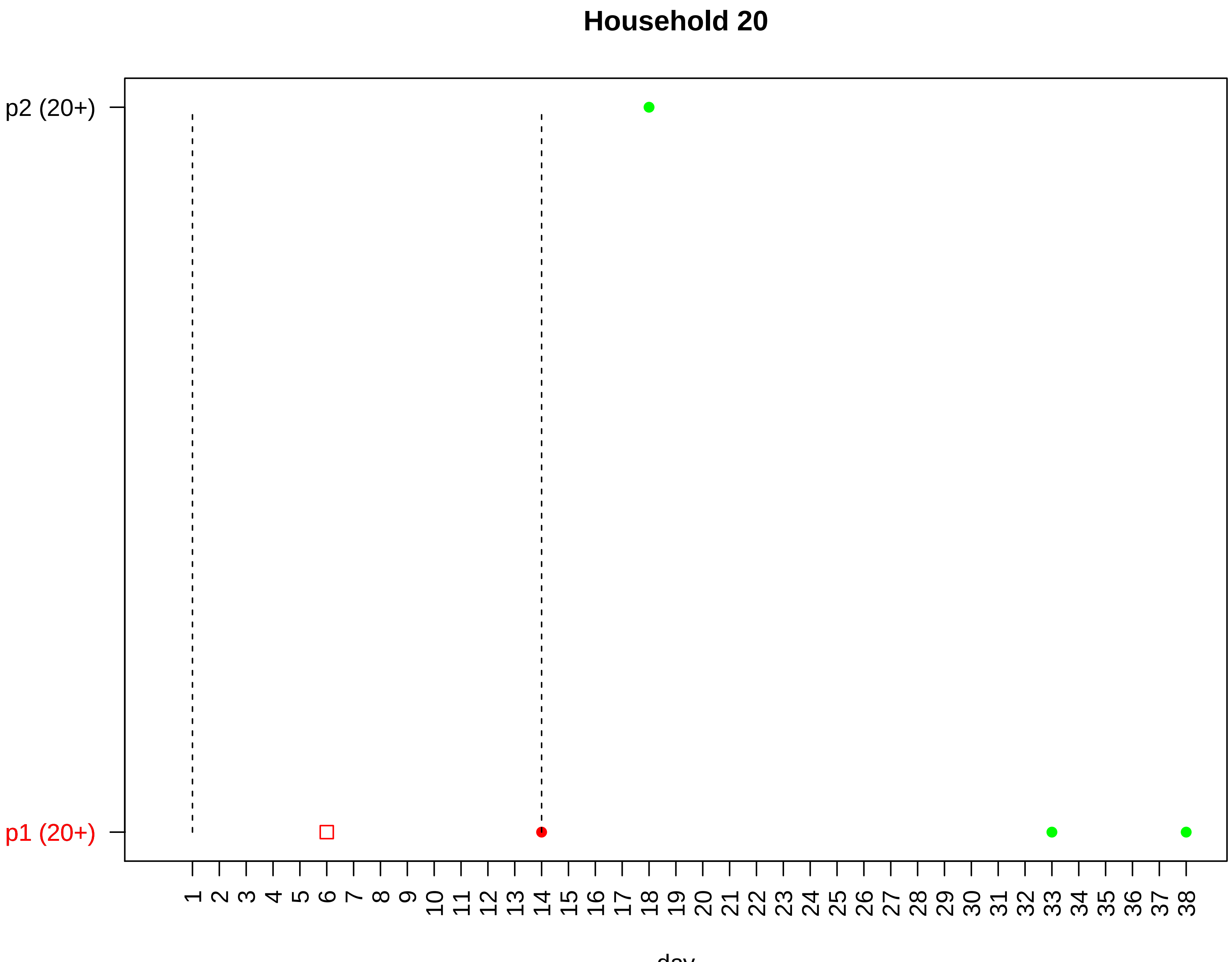
Household 20
p2 (20+)
p1 (20+)
1 2 3 4 5 6 7 8 9 10 11 12 13 14 15 16 17 18 19 20 21 22 23 24 25 26 27 28 29 30 31 32 33 34 35 36 37 38
day

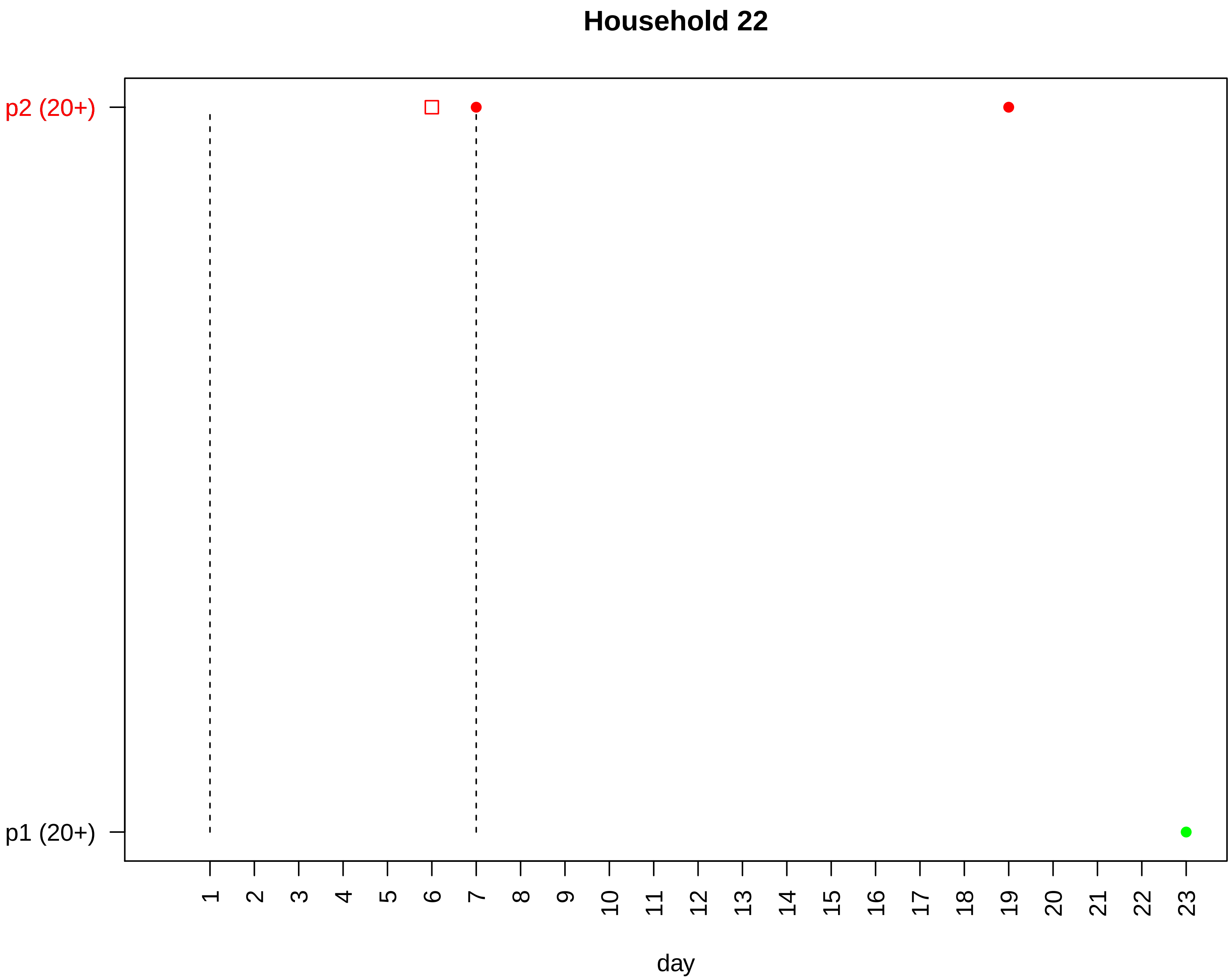

Household 22
p2 (20+)
p1 (20+)
1
2
3
4
5
6
7
8
9
10
11
12
13
14
15
16
17
18
19
20
21
22
23
day

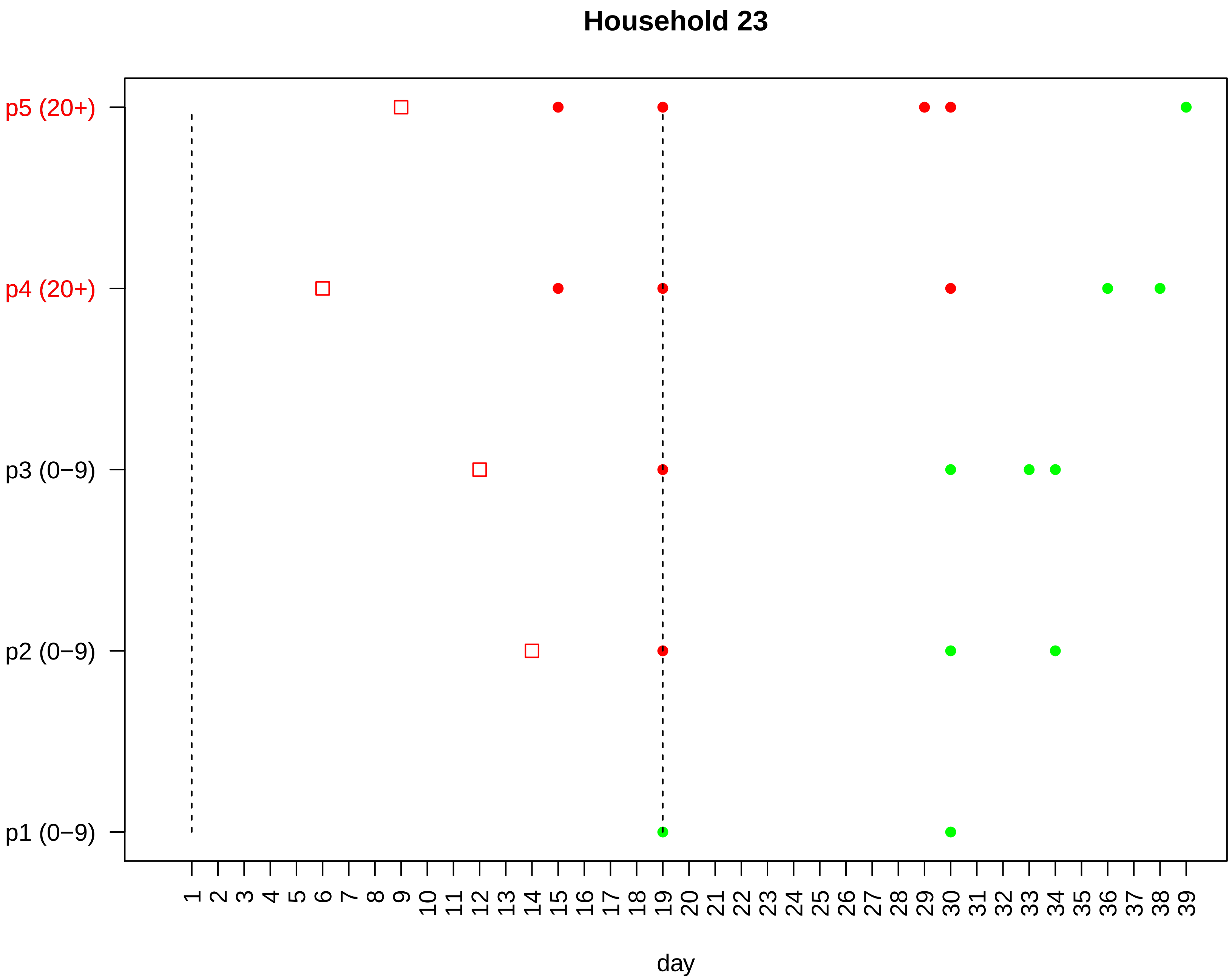
Household 23
p5 (20+)
p4 (20+)
p3 (0−9)
p2 (0−9)
p1 (0−9)
1 2 3 4 5 6 7 8 9 10 11 12 13 14 15 16 17 18 19 20 21 22 23 24 25 26 27 28 29 30 31 32 33 34 35 36 37 38 39
day

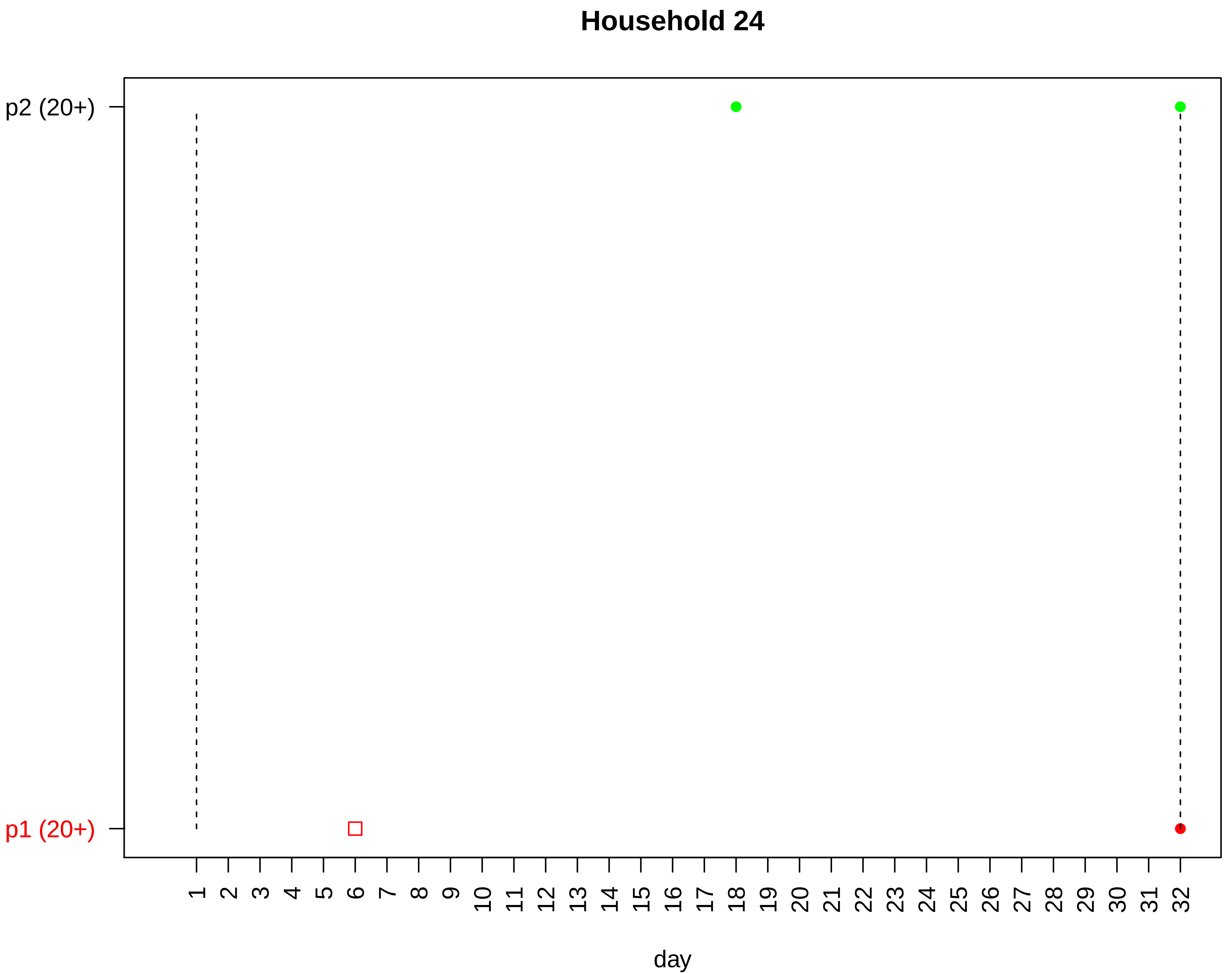

Household 24
p2 (20+)
p1 (20+)
1
2
3
4
5
6
7
8
9
10
11
12
13
14
15
16
17
18
19
20
21
22
23
24
25
26
27
28
29
30
31
32
day

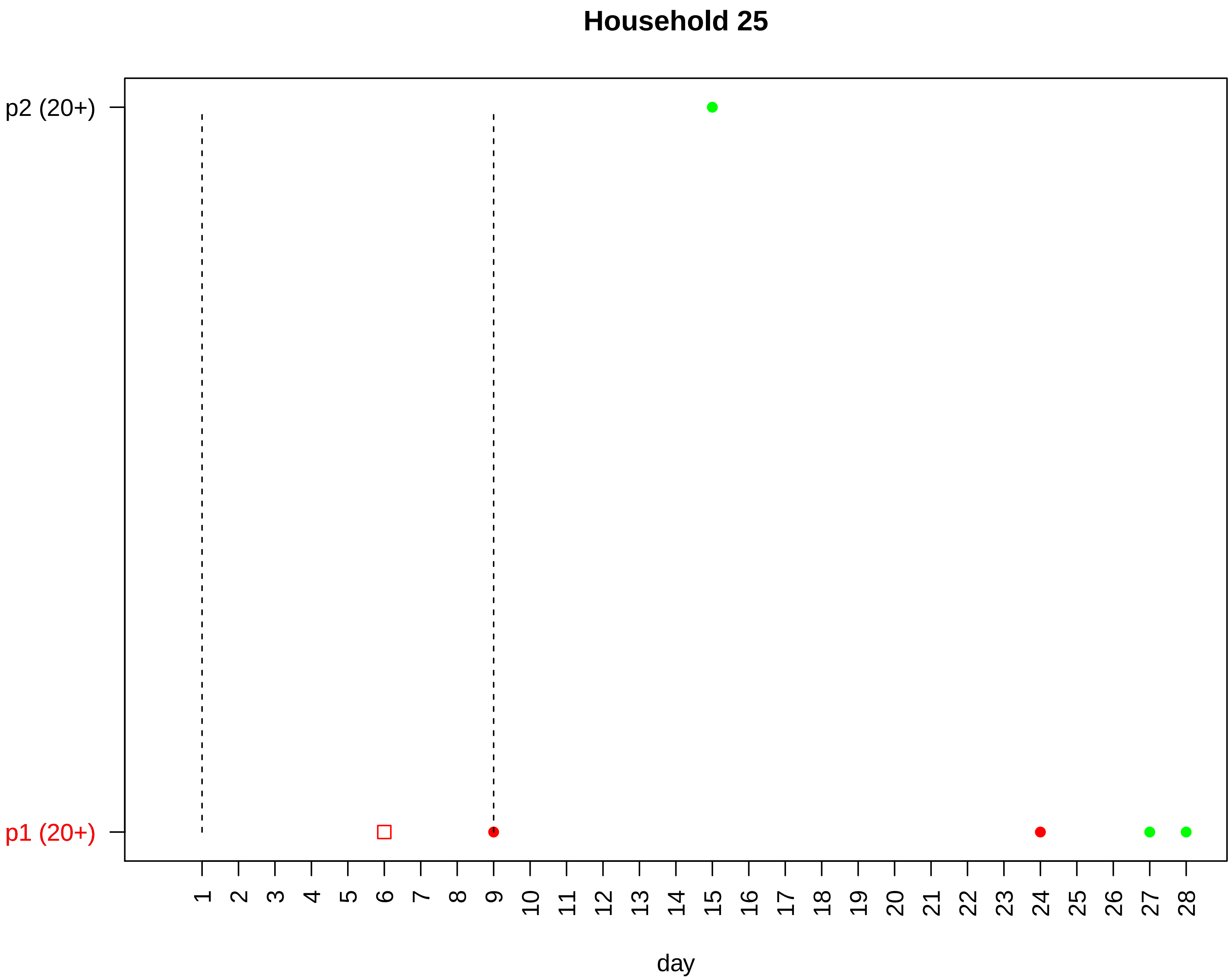

Household 25
p2 (20+)
p1 (20+)
1
2
3
4
5
6
7
8
9
10
11
12
13
14
15
16
17
18
19
20
21
22
23
24
25
26
27
28
day

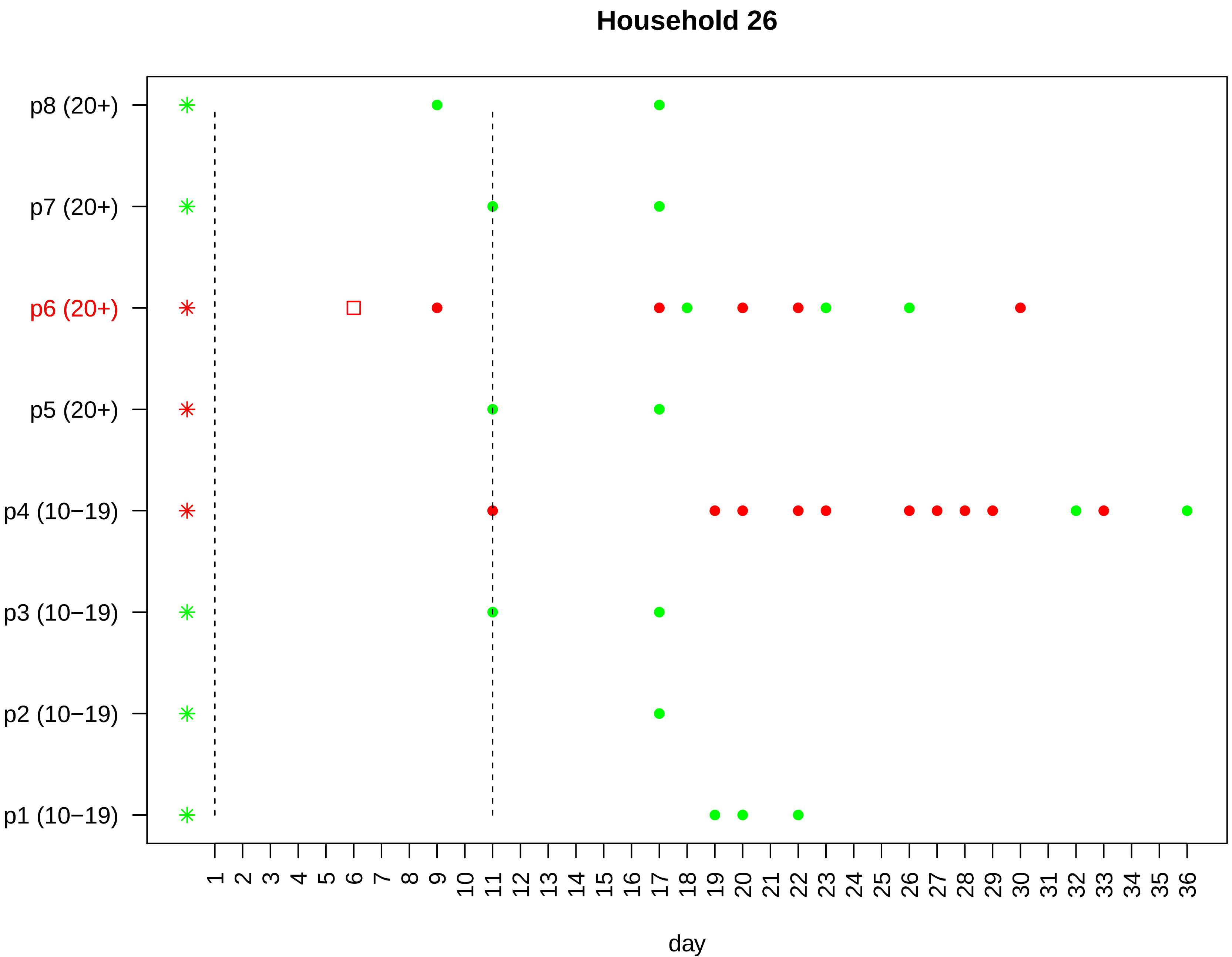

Household 26
p8 (20+)
p7 (20+)
p6 (20+)
p5 (20+)
p4 (10−19)
p3 (10−19)
p2 (10−19)
p1 (10−19)
1 2 3 4 5 6 7 8 9 10 11 12 13 14 15 16 17 18 19 20 21 22 23 24 25 26 27 28 29 30 31 32 33 34 35 36
day

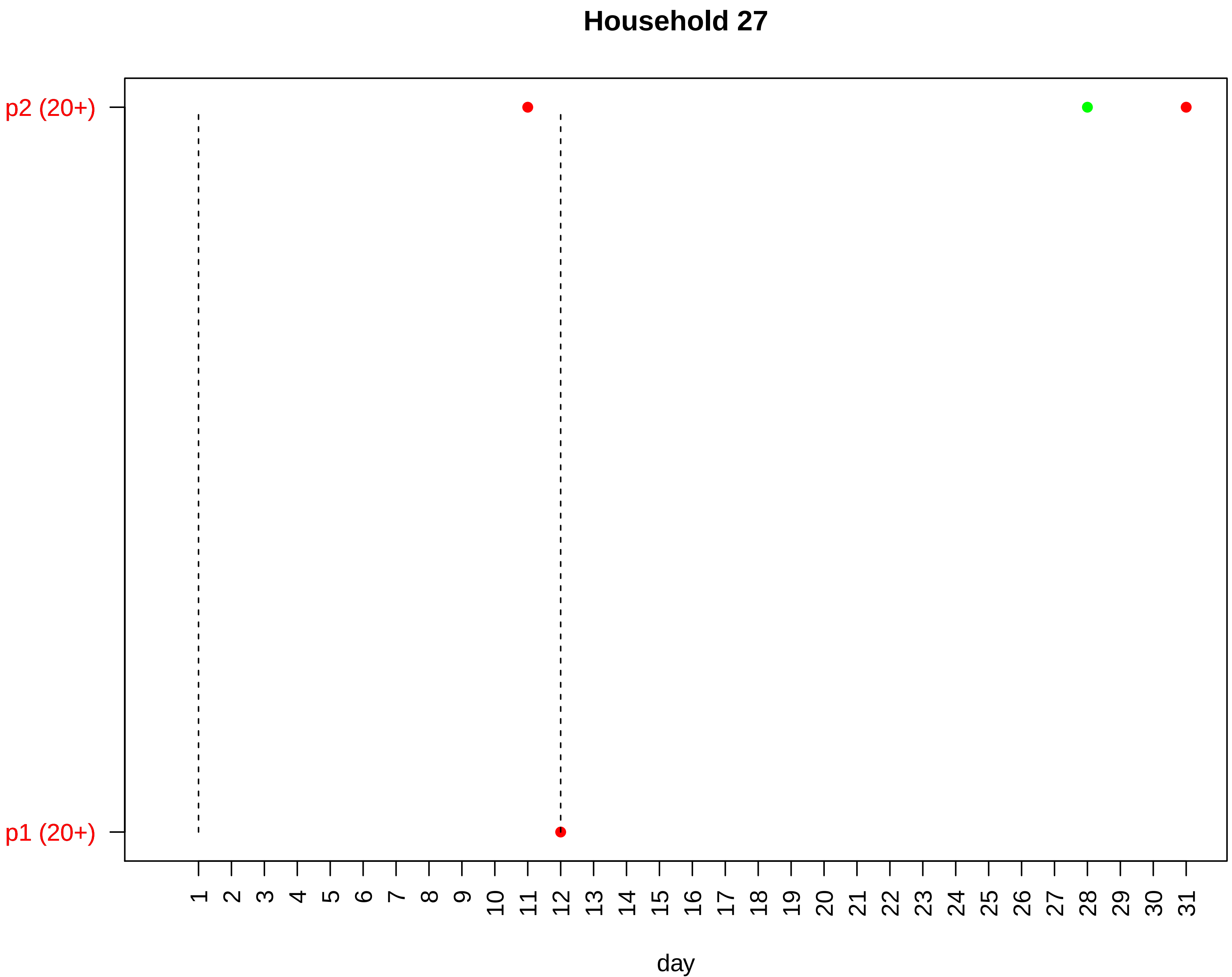

Household 27
p2 (20+)
p1 (20+)
1
2
3
4
5
6
7
8
9
10
11
12
13
14
15
16
17
18
19
20
21
22
23
24
25
26
27
28
29
30
31
day

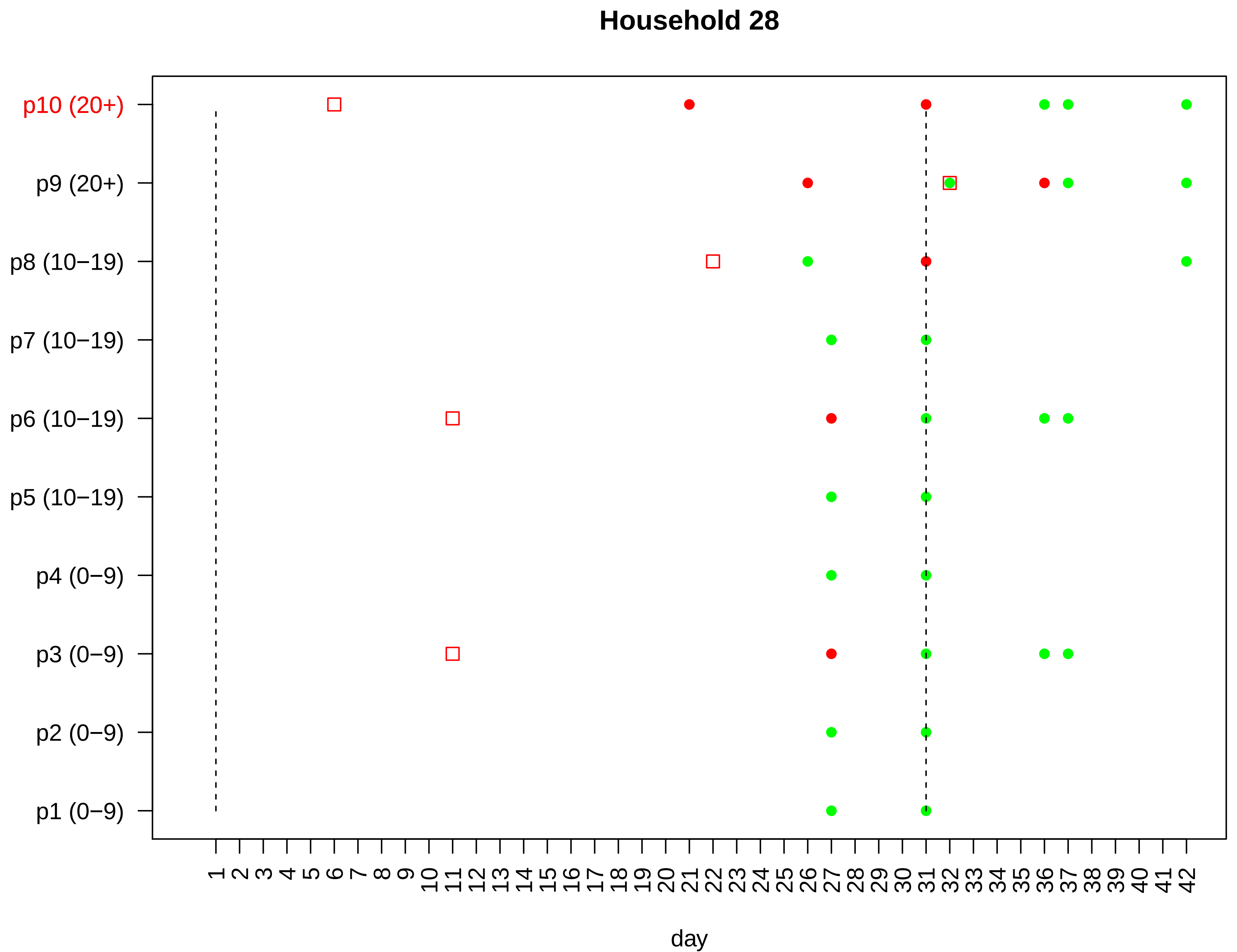

Household 28
p10 (20+)
p9 (20+)
p8 (10−19)
p7 (10−19)
p6 (10−19)
p5 (10−19)
p4 (0−9)
p3 (0−9)
p2 (0−9)
p1 (0−9)
1 2 3 4 5 6 7 8 9 10 11 12 13 14 15 16 17 18 19 20 21 22 23 24 25 26 27 28 29 30 31 32 33 34 35 36 37 38 39 40 41 42
day

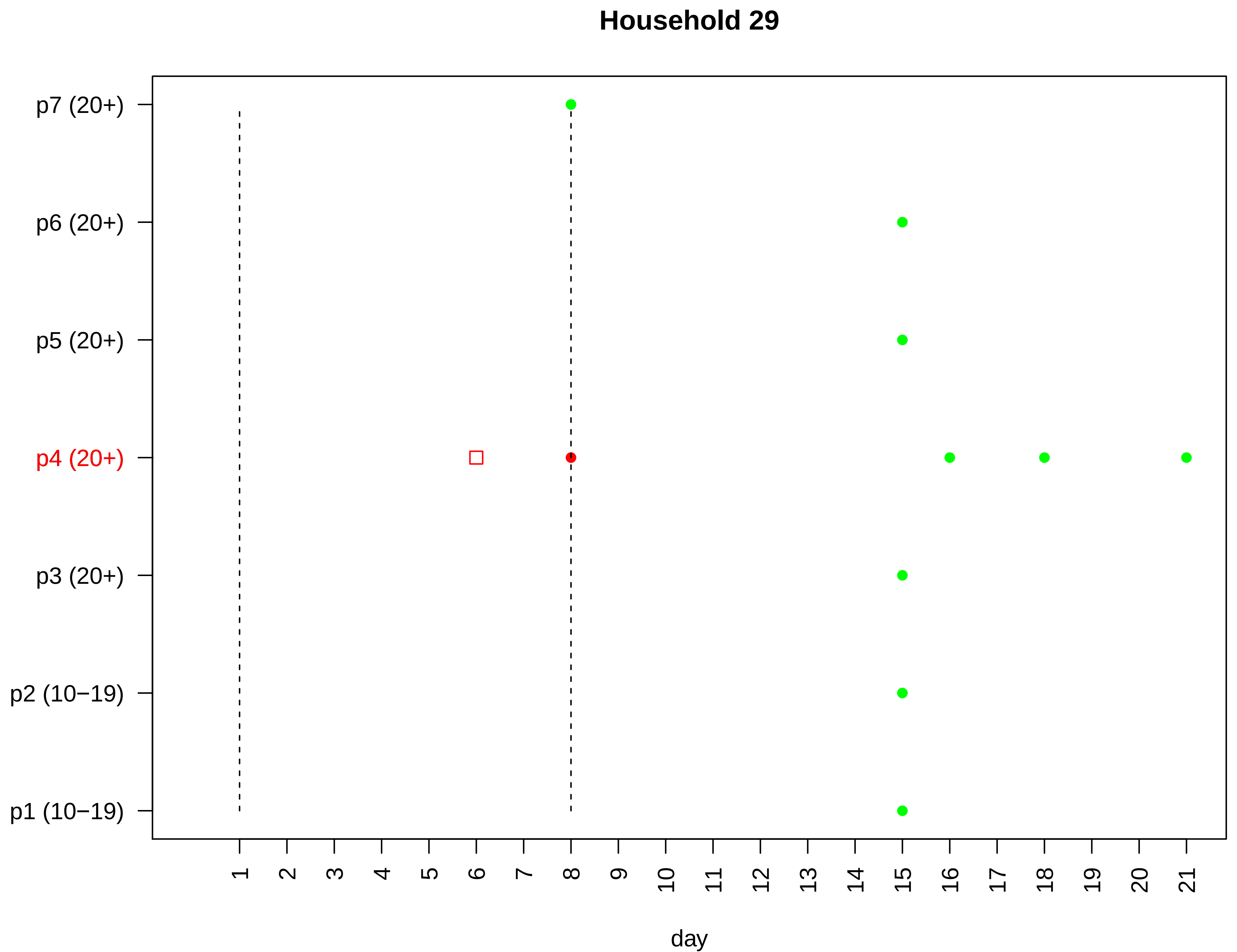

Household 29
p7 (20+)
p6 (20+)
p5 (20+)
p4 (20+)
p3 (20+)
p2 (10−19)
p1 (10−19)
1
2
3
4
5
6
7
8
9
10
11
12
13
14
15
16
17
18
19
20
21
day

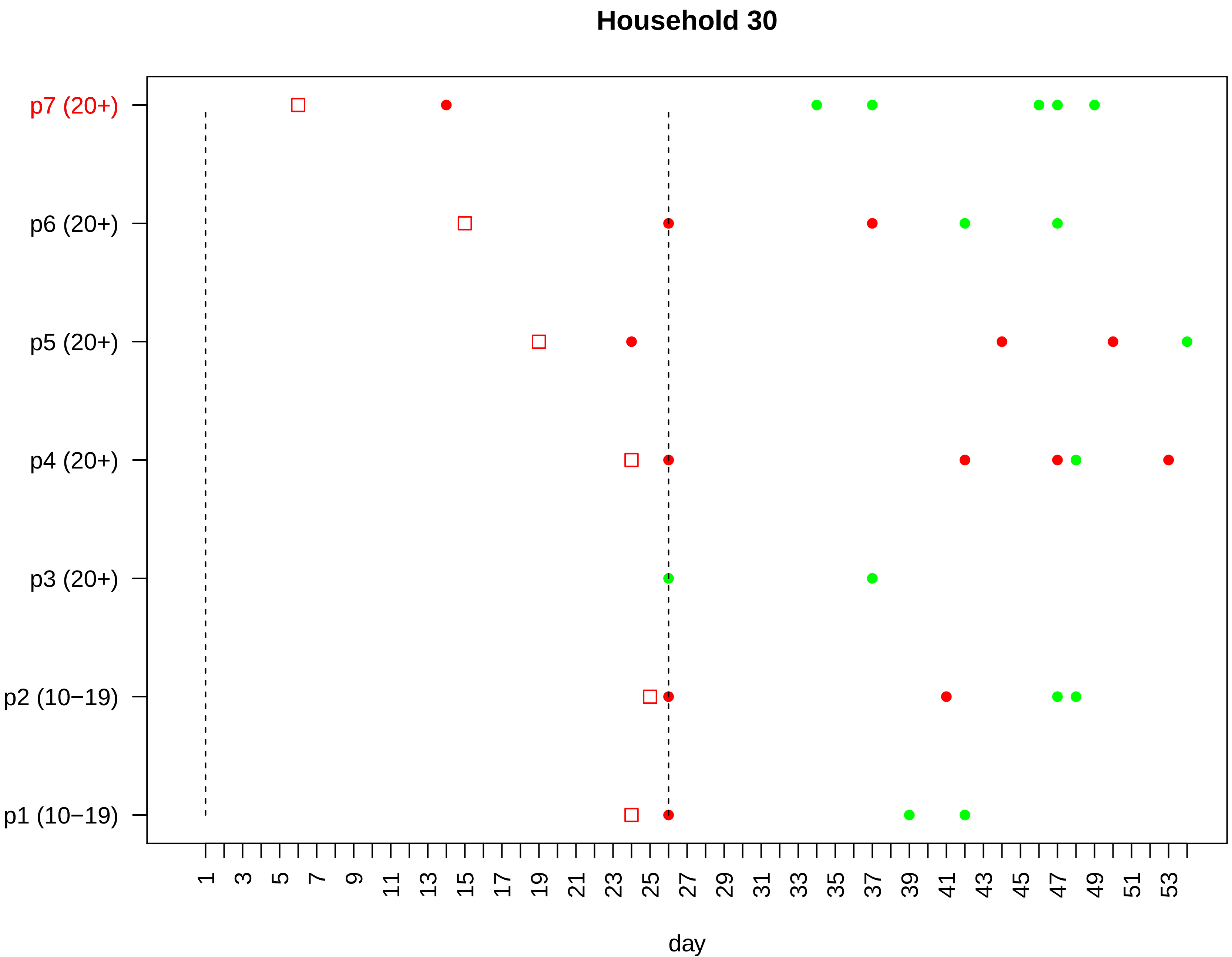

Household 30
p7 (20+)
p6 (20+)
p5 (20+)
p4 (20+)
p3 (20+)
p2 (10−19)
p1 (10−19)
1 3 5 7 9 11 13 15 17 19 21 23 25 27 29 31 33 35 37 39 41 43 45 47 49 51 53
day

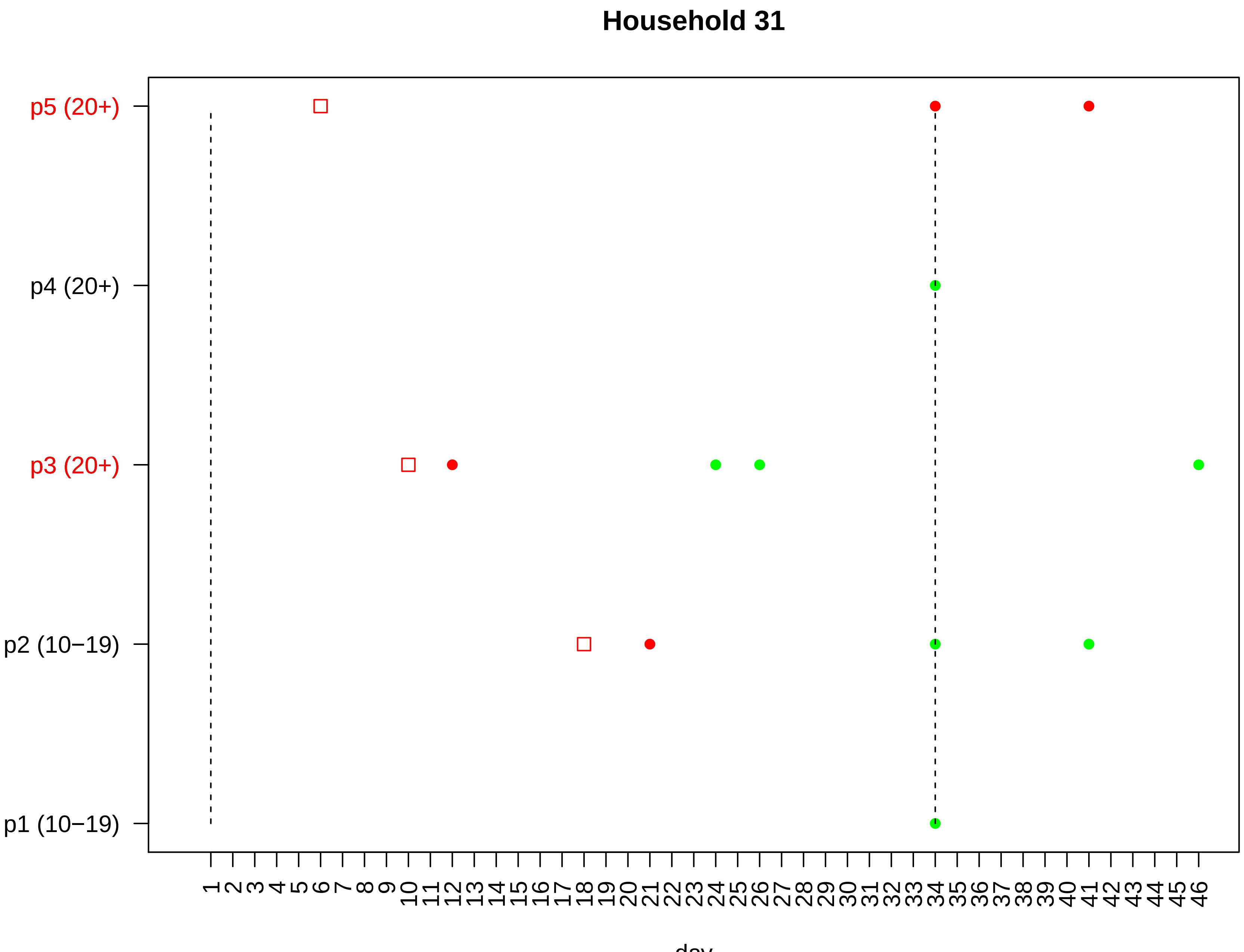

Household 31
p5 (20+)
p4 (20+)
p3 (20+)
p2 (10−19)
p1 (10−19)
1 2 3 4 5 6 7 8 9 10 11 12 13 14 15 16 17 18 19 20 21 22 23 24 25 26 27 28 29 30 31 32 33 34 35 36 37 38 39 40 41 42 43 44 45 46
day

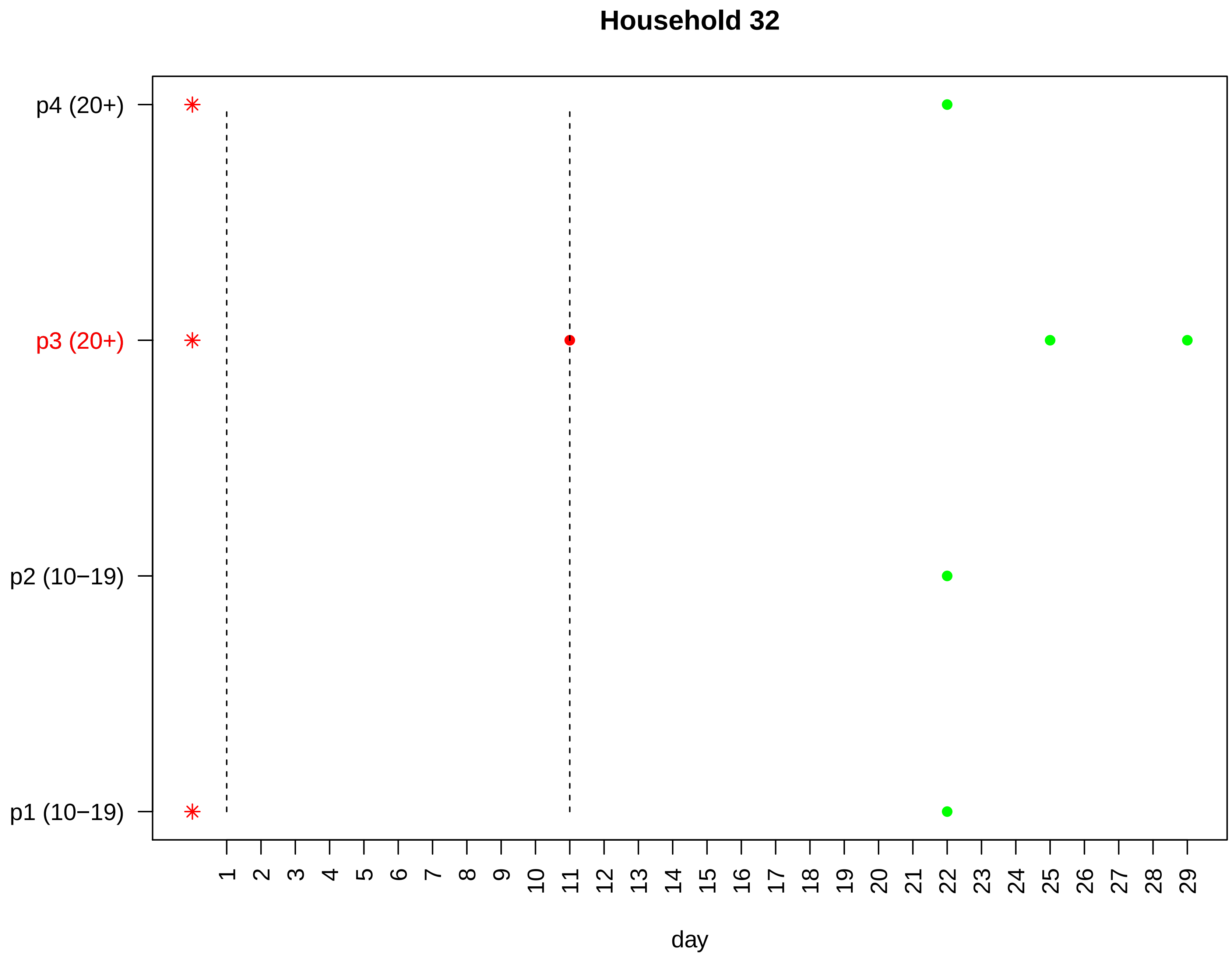

Household 32
p4 (20+)
p3 (20+)
p2 (10−19)
p1 (10−19)
1 2 3 4 5 6 7 8 9 10 11 12 13 14 15 16 17 18 19 20 21 22 23 24 25 26 27 28 29
day

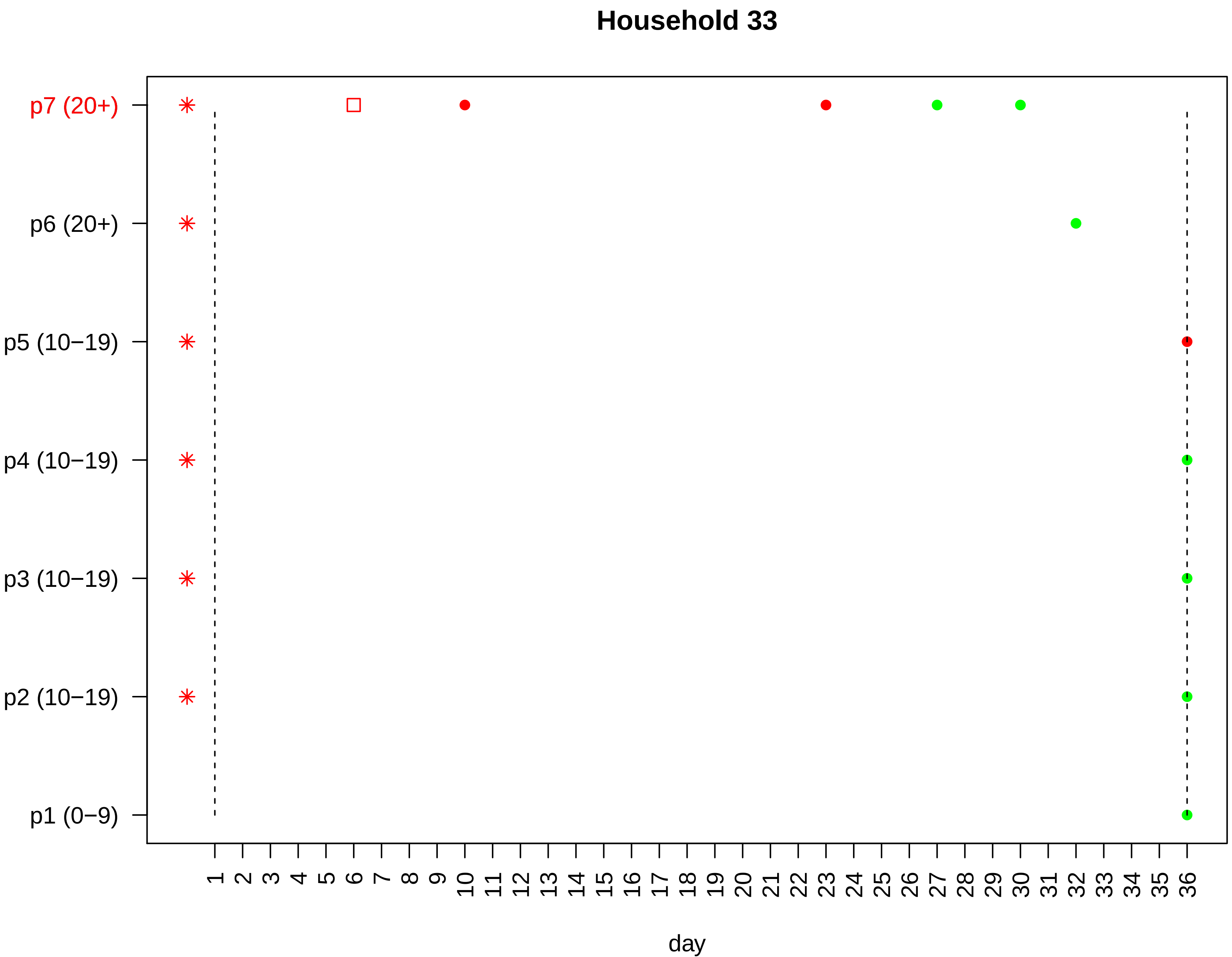

Household 33
p7 (20+)
p6 (20+)
p5 (10−19)
p4 (10−19)
p3 (10−19)
p2 (10−19)
p1 (0−9)
1 2 3 4 5 6 7 8 9 10 11 12 13 14 15 16 17 18 19 20 21 22 23 24 25 26 27 28 29 30 31 32 33 34 35 36
day

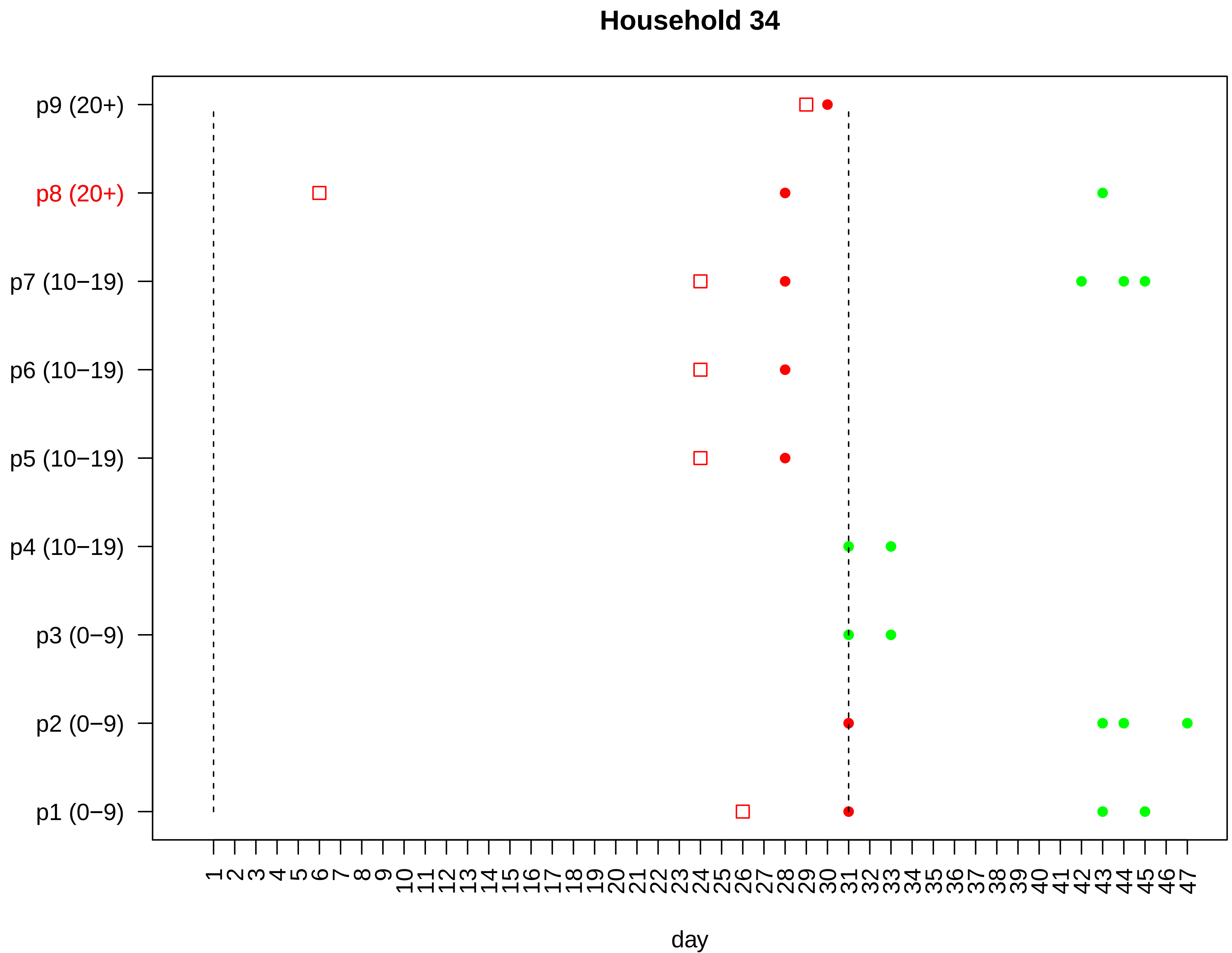

Household 34
p9 (20+)
p8 (20+)
p7 (10−19)
p6 (10−19)
p5 (10−19)
p4 (10−19)
p3 (0−9)
p2 (0−9)
p1 (0−9)
1 2 3 4 5 6 7 8 9 10 11 12 13 14 15 16 17 18 19 20 21 22 23 24 25 26 27 28 29 30 31 32 33 34 35 36 37 38 39 40 41 42 43 44 45 46 47
day

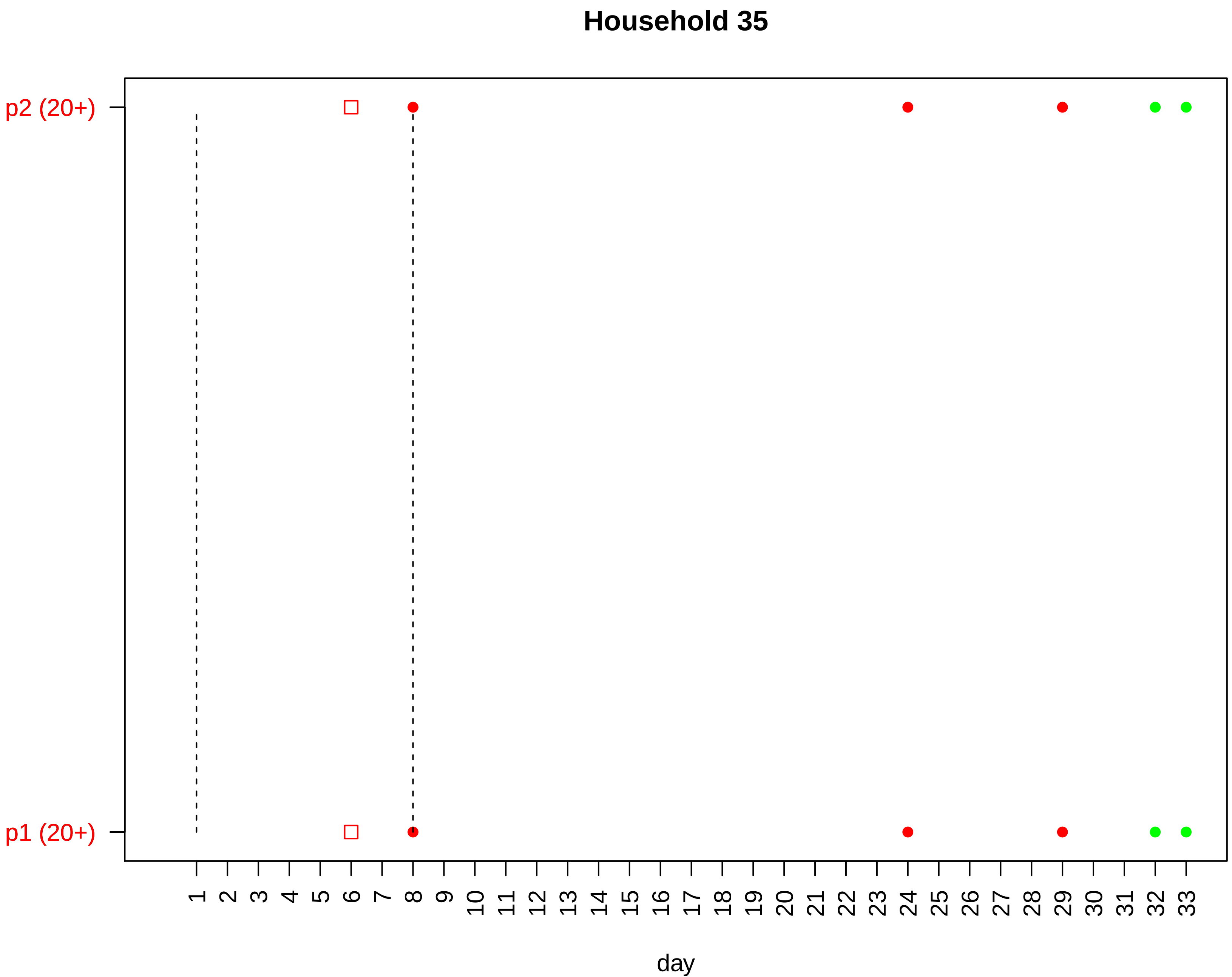

Household 35
p2 (20+)
p1 (20+)
1
2
3
4
5
6
7
8
9
10
11
12
13
14
15
16
17
18
19
20
21
22
23
24
25
26
27
28
29
30
31
32
33
day

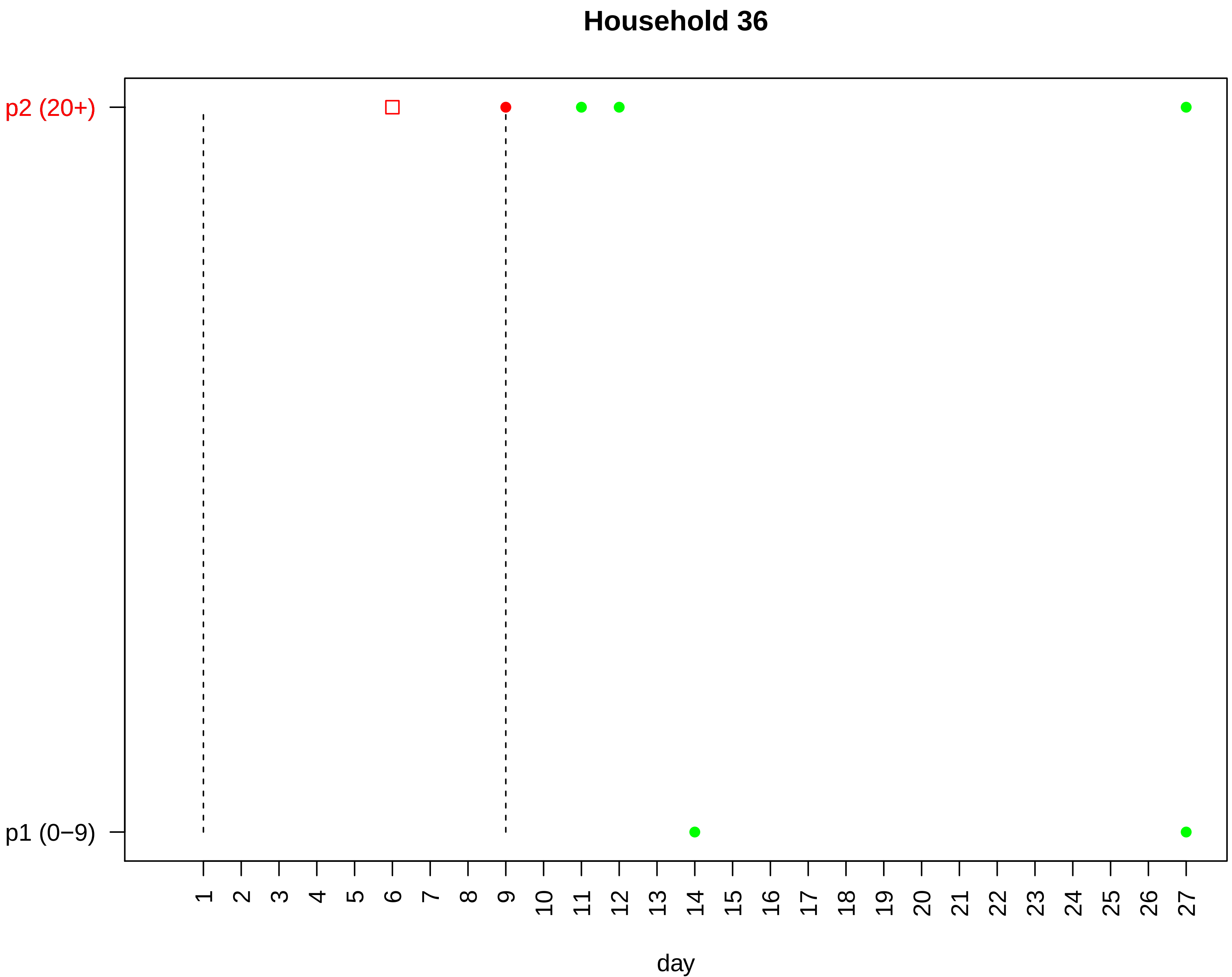

Household 36
p2 (20+)
p1 (0−9)
1
2
3
4
5
6
7
8
9
10
11
12
13
14
15
16
17
18
19
20
21
22
23
24
25
26
27
day

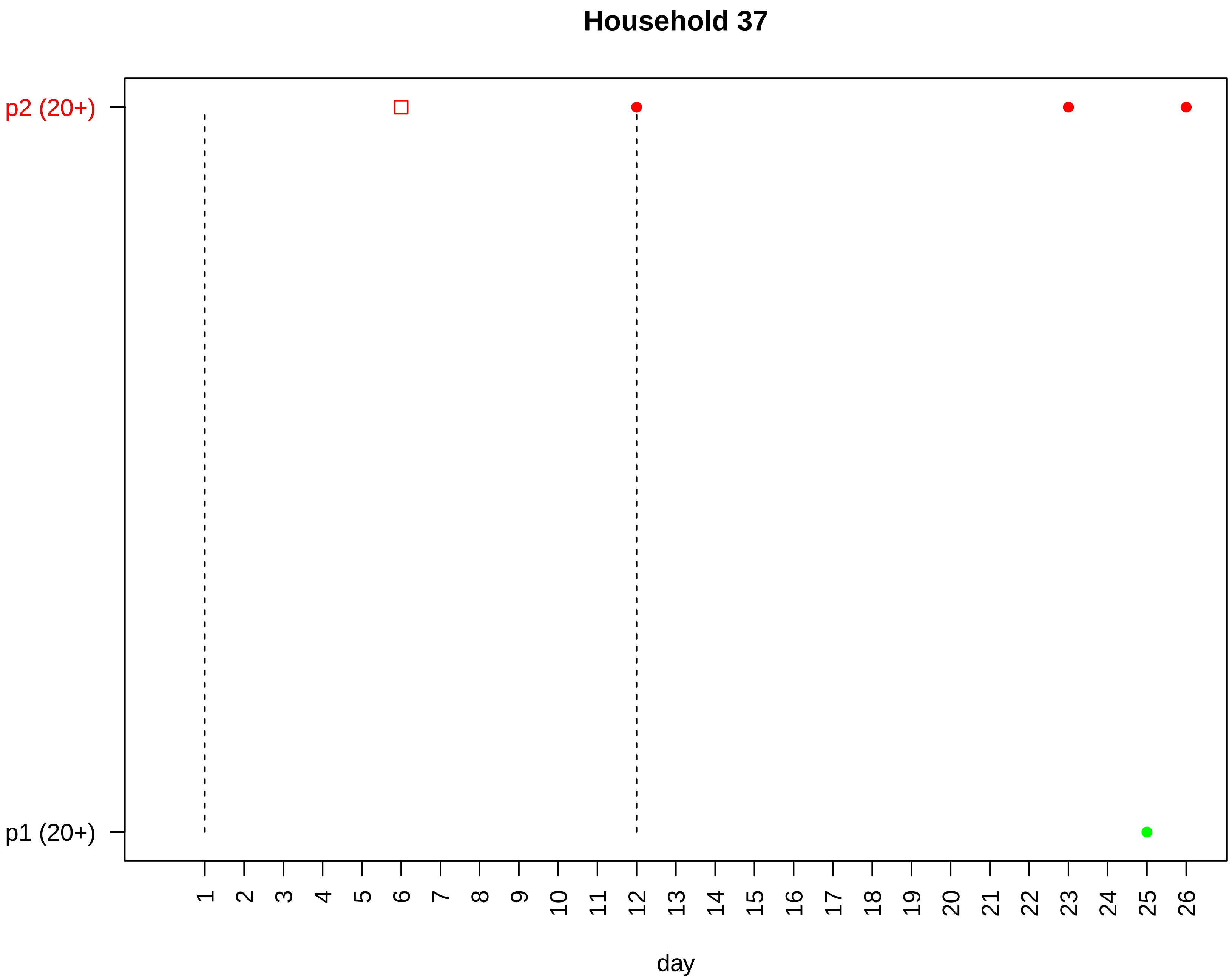

Household 37
p2 (20+)
p1 (20+)
1
2
3
4
5
6
7
8
9
10
11
12
13
14
15
16
17
18
19
20
21
22
23
24
25
26
day

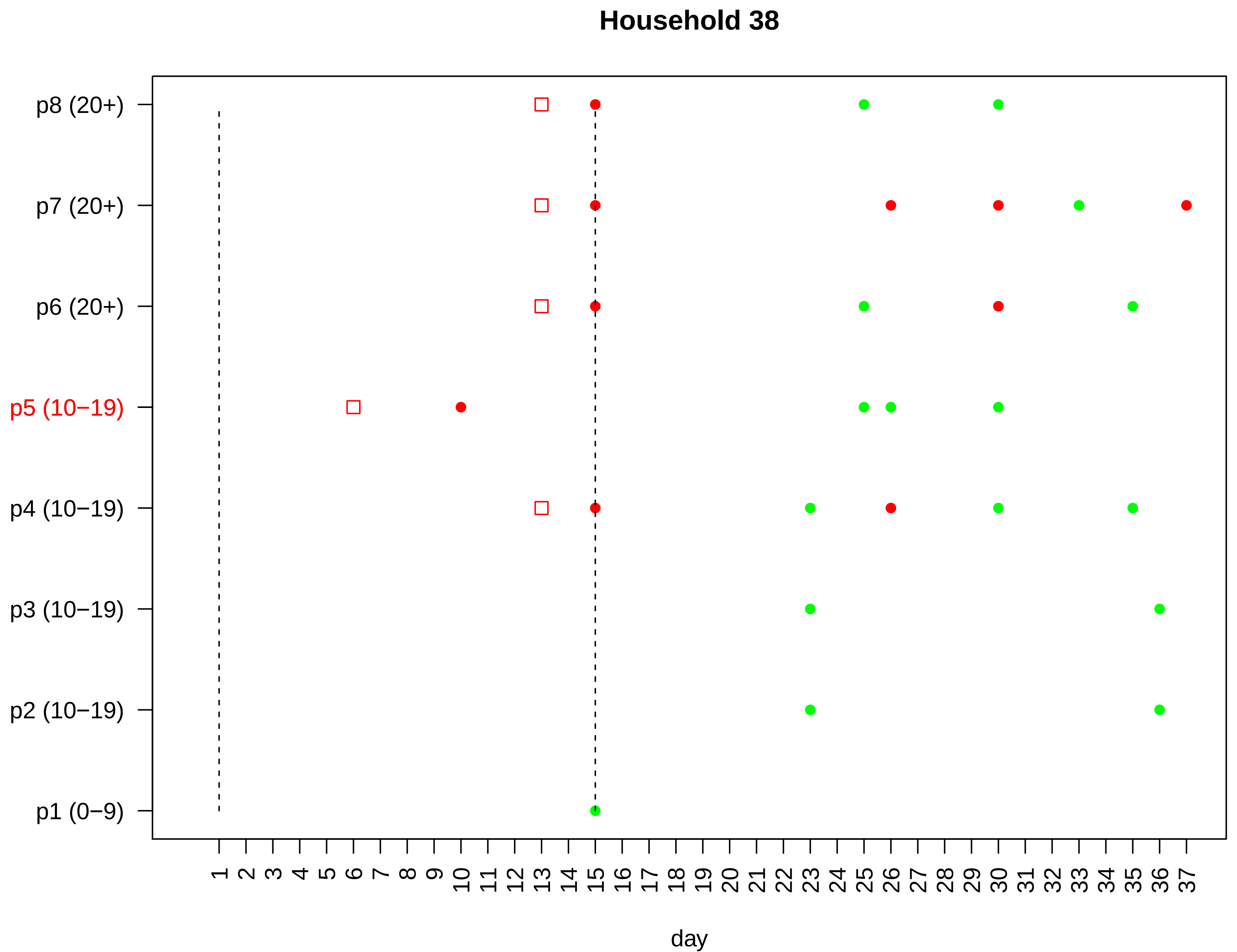

Household 38
p8 (20+)
p7 (20+)
p6 (20+)
p5 (10−19)
p4 (10−19)
p3 (10−19)
p2 (10−19)
p1 (0−9)
1 2 3 4 5 6 7 8 9 10 11 12 13 14 15 16 17 18 19 20 21 22 23 24 25 26 27 28 29 30 31 32 33 34 35 36 37
day

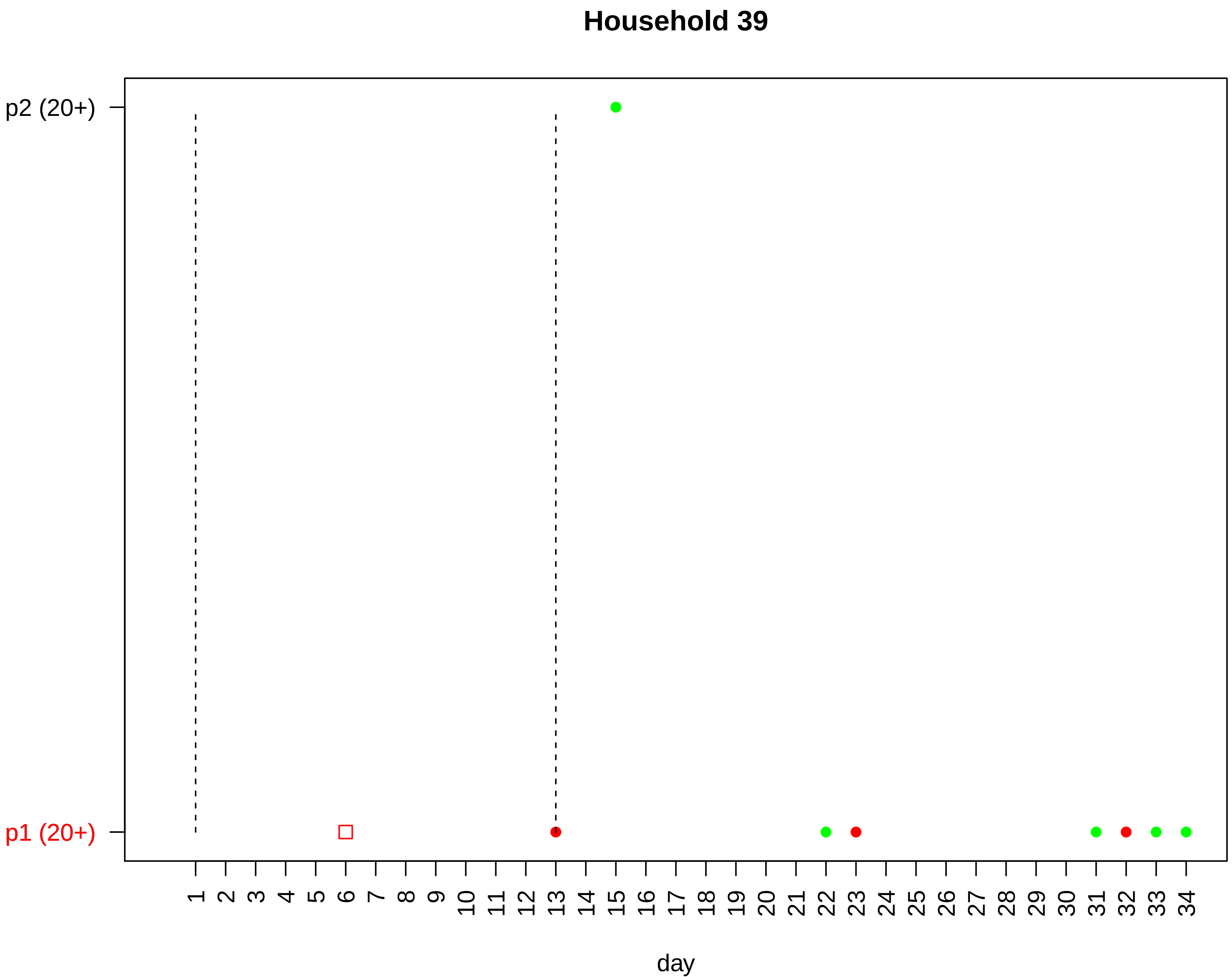
Household 39
p2 (20+)
p1 (20+)
1 2 3 4 5 6 7 8 9 10 11 12 13 14 15 16 17 18 19 20 21 22 23 24 25 26 27 28 29 30 31 32 33 34
day

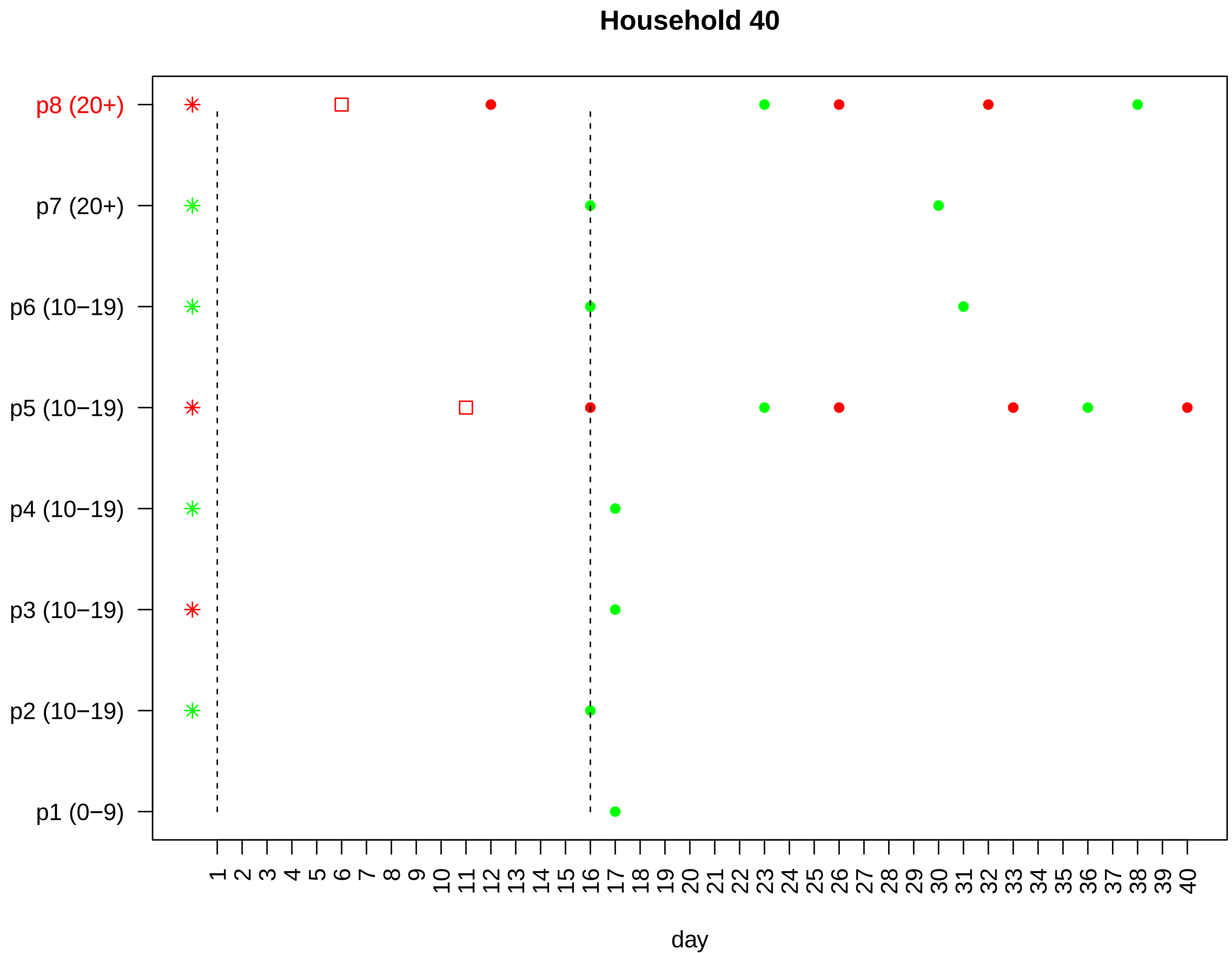
Household 40
p8 (20+)
p7 (20+)
p6 (10−19)
p5 (10−19)
p4 (10−19)
p3 (10−19)
p2 (10−19)
p1 (0−9)
1 2 3 4 5 6 7 8 9 10 11 12 13 14 15 16 17 18 19 20 21 22 23 24 25 26 27 28 29 30 31 32 33 34 35 36 37 38 39 40
day

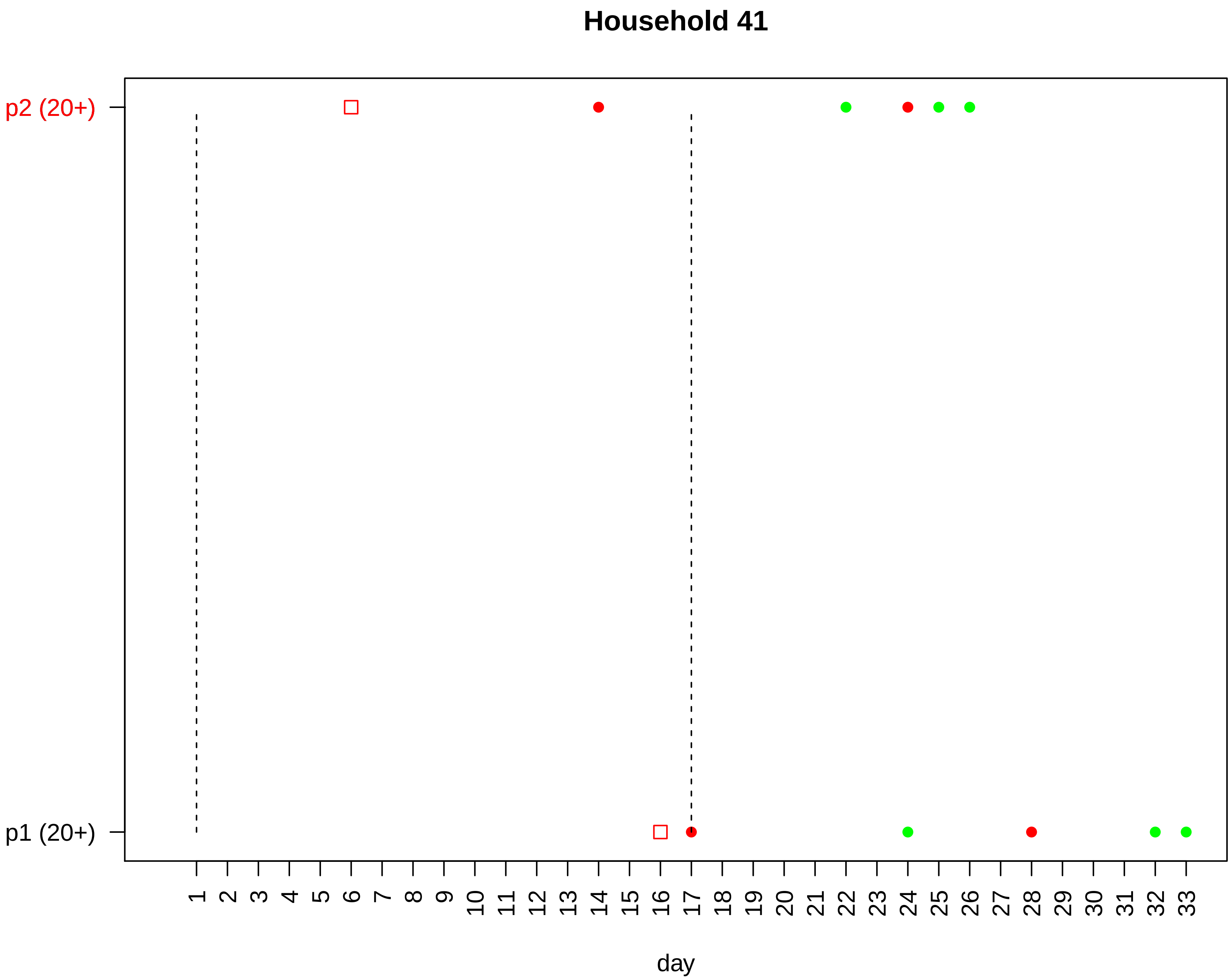

Household 41
p2 (20+)
p1 (20+)
1 2 3 4 5 6 7 8 9 10 11 12 13 14 15 16 17 18 19 20 21 22 23 24 25 26 27 28 29 30 31 32 33
day

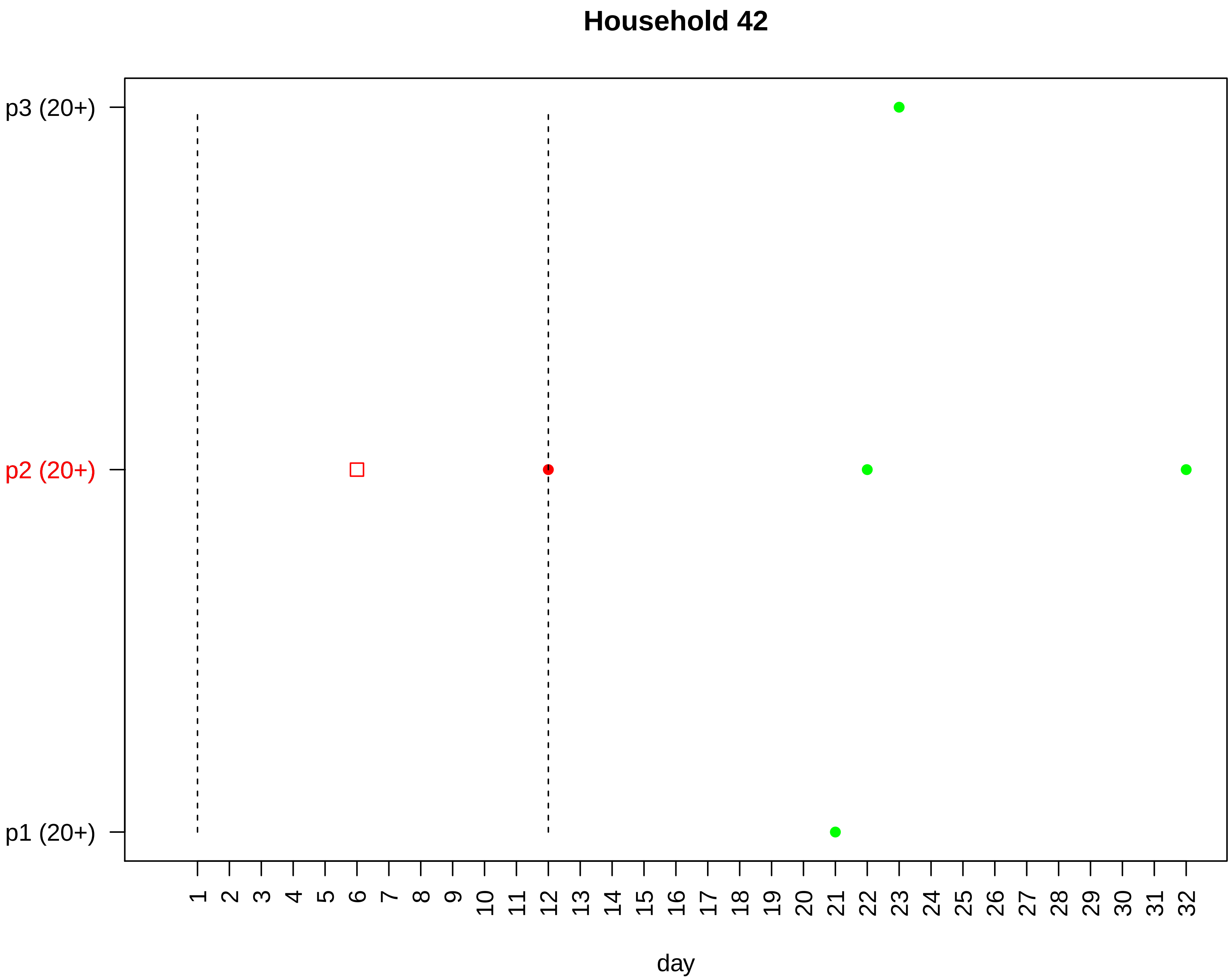

Household 42
p3 (20+)
p2 (20+)
p1 (20+)
1 2 3 4 5 6 7 8 9 10 11 12 13 14 15 16 17 18 19 20 21 22 23 24 25 26 27 28 29 30 31 32
day

Household 43

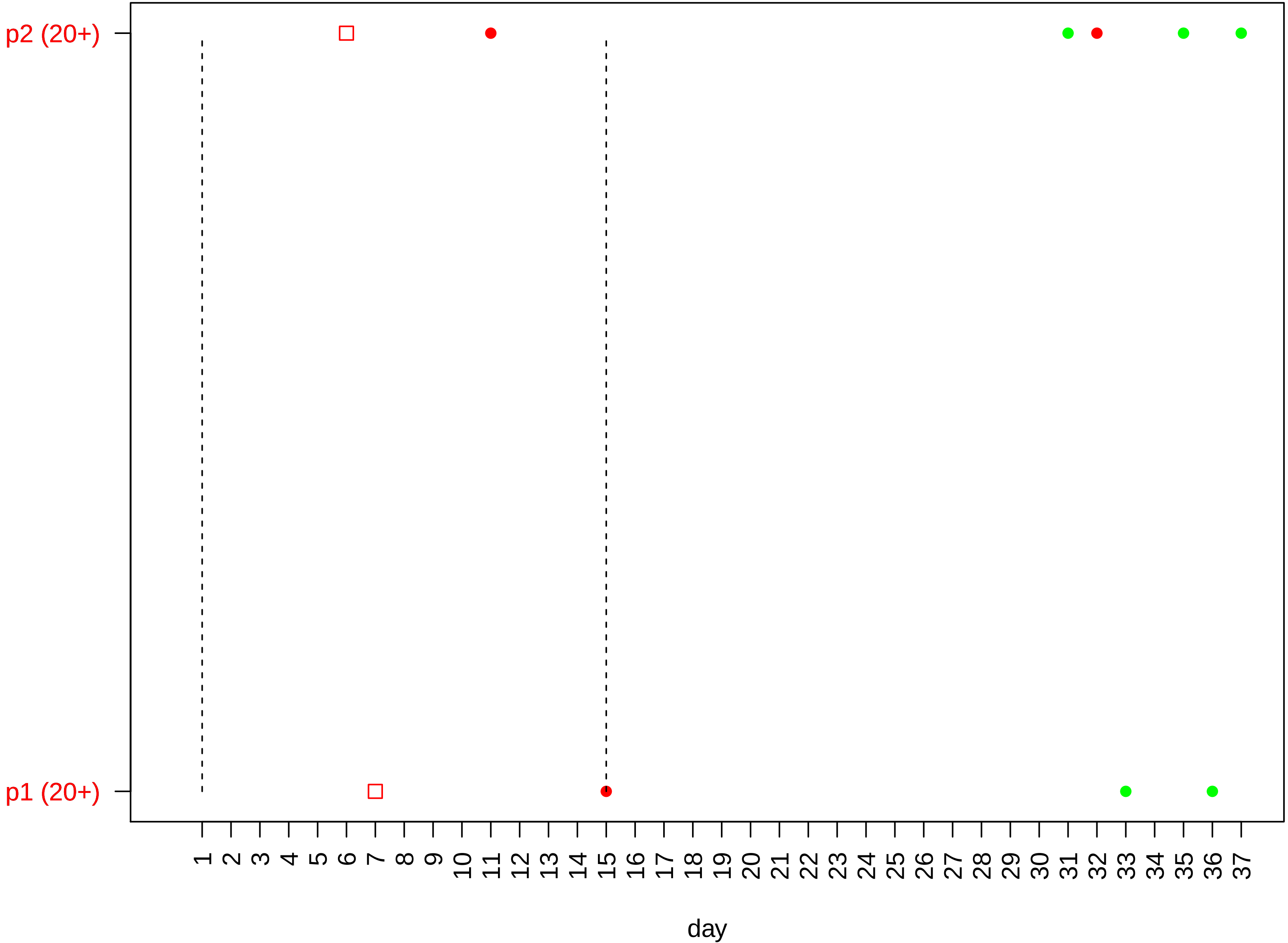

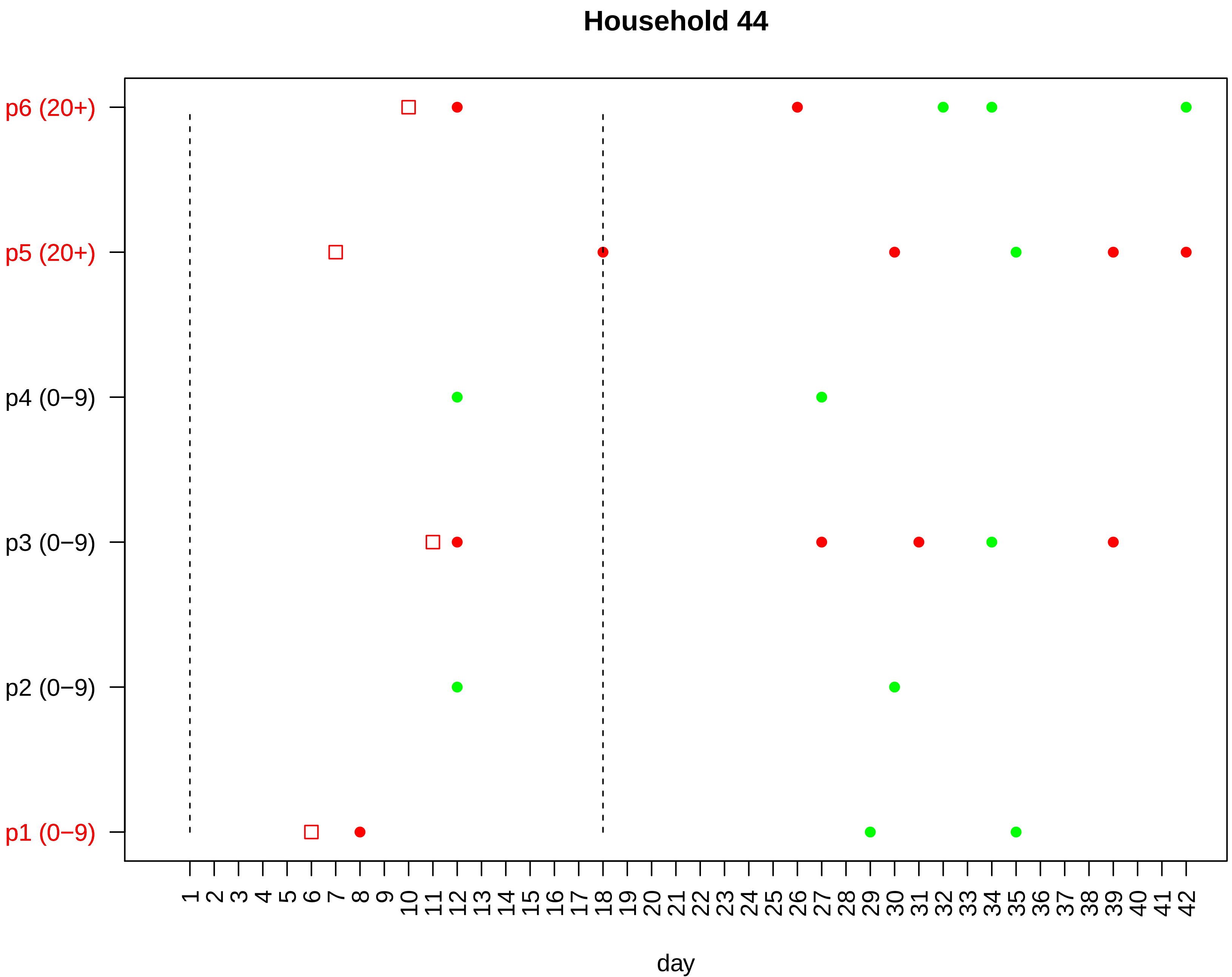

Household 44
p6 (20+)
p5 (20+)
p4 (0−9)
p3 (0−9)
p2 (0−9)
p1 (0−9)
1 2 3 4 5 6 7 8 9 10 11 12 13 14 15 16 17 18 19 20 21 22 23 24 25 26 27 28 29 30 31 32 33 34 35 36 37 38 39 40 41 42
day

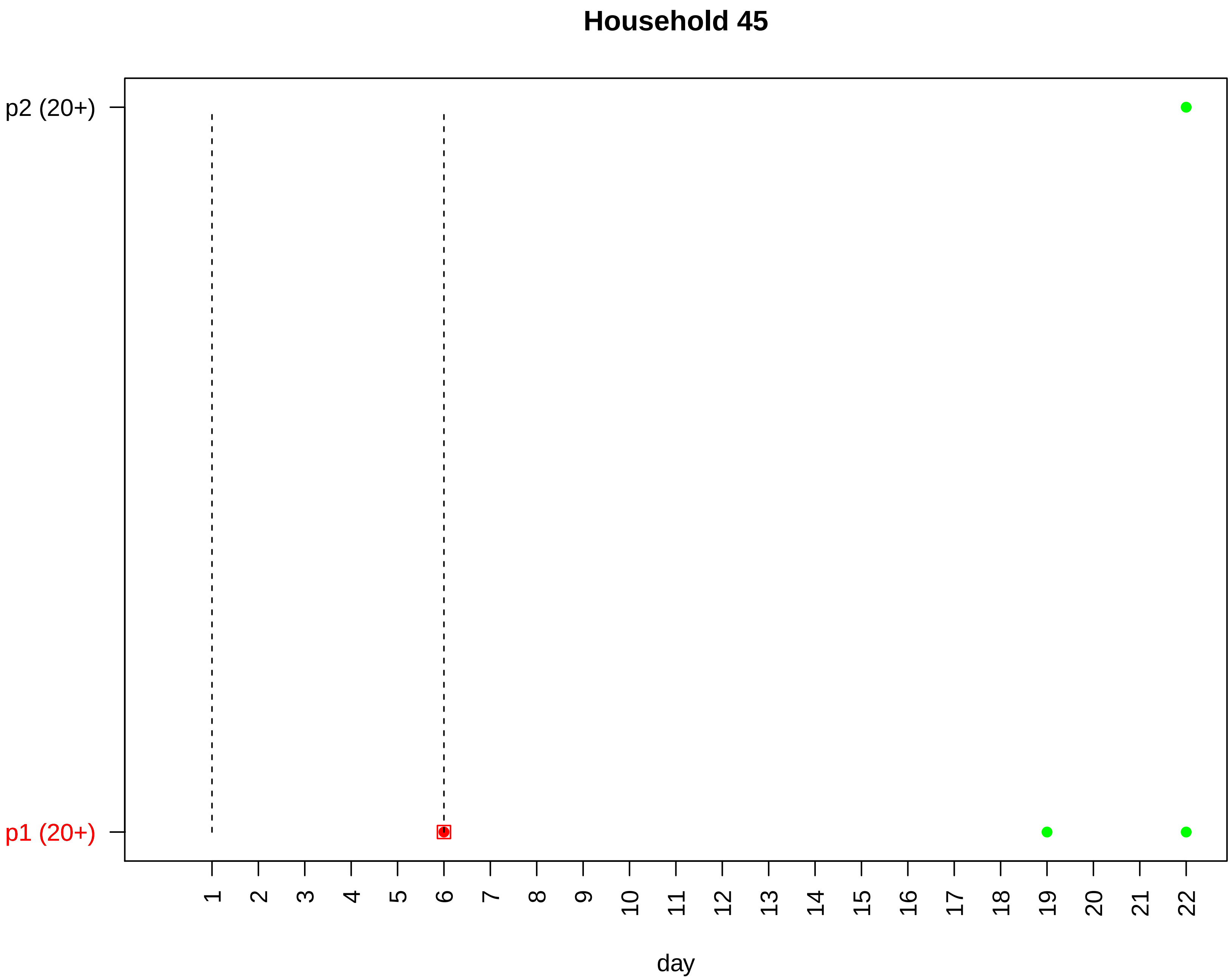

Household 45
p2 (20+)
p1 (20+)
1
2
3
4
5
6
7
8
9
10
11
12
13
14
15
16
17
18
19
20
21
22
day

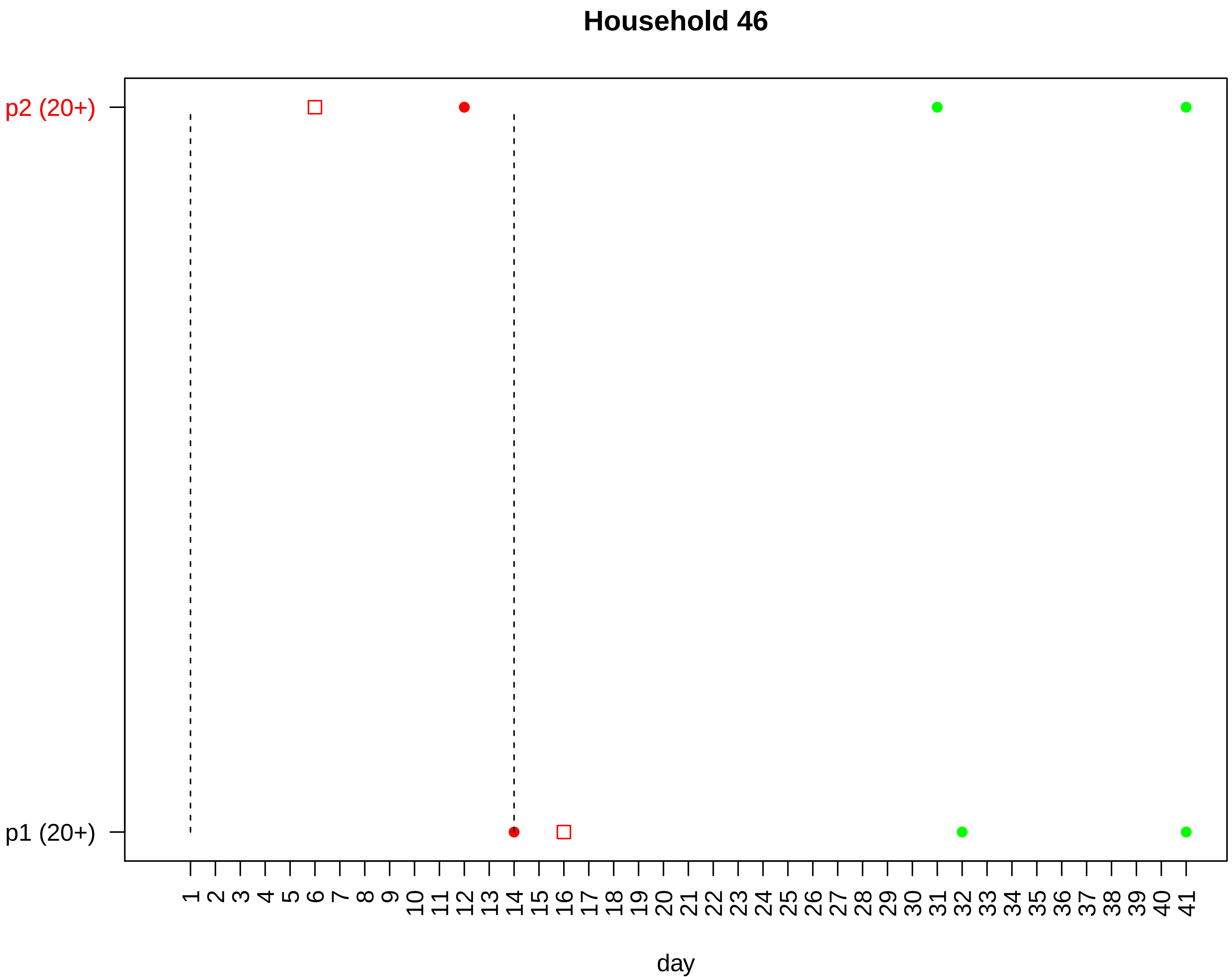

Household 46
p2 (20+)
p1 (20+)
1
2
3
4
5
6
7
8
9
10
11
12
13
14
15
16
17
18
19
20
21
22
23
24
25
26
27
28
29
30
31
32
33
34
35
36
37
38
39
40
41
day

Household 47

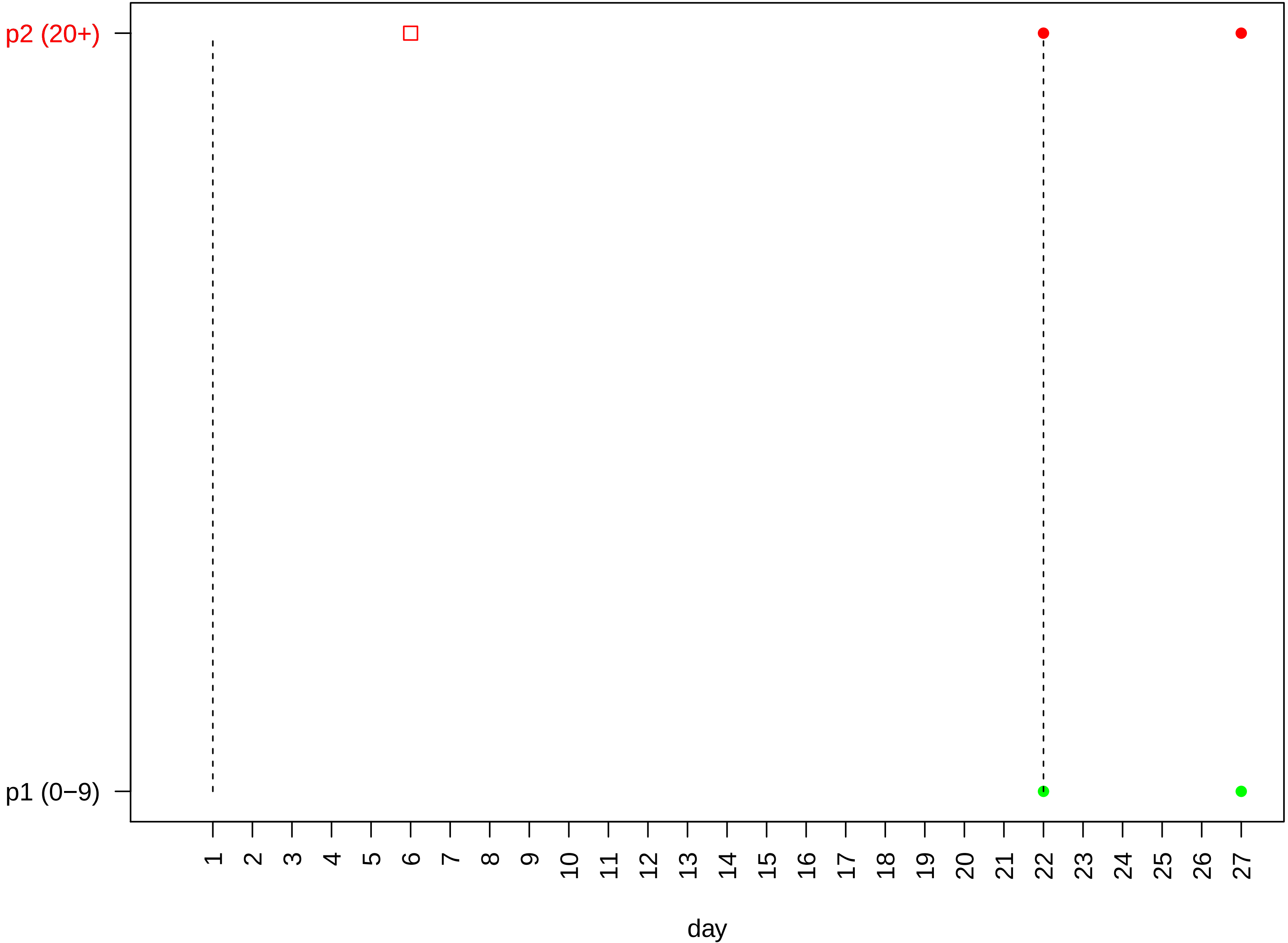

Household 48

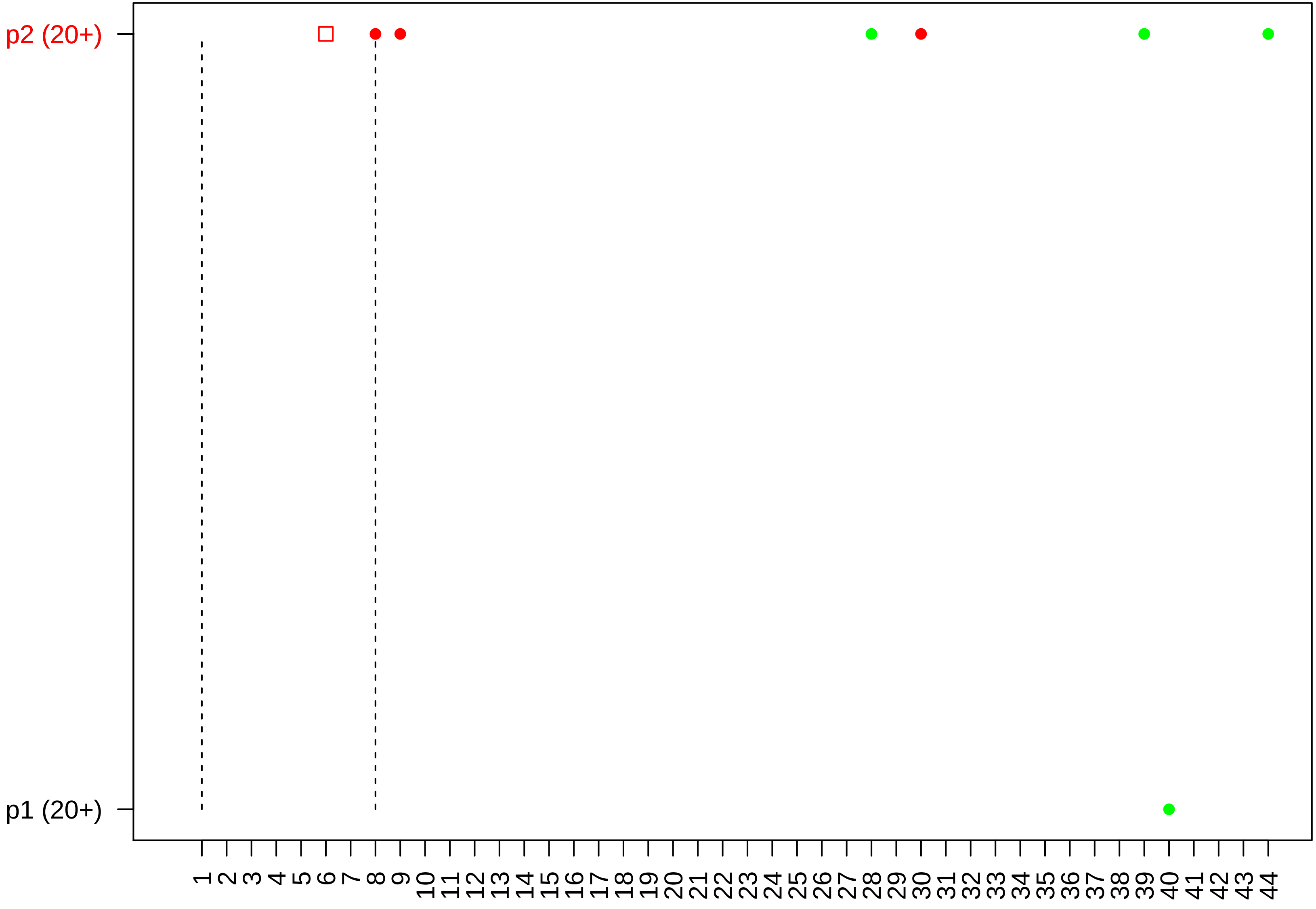

Household 49

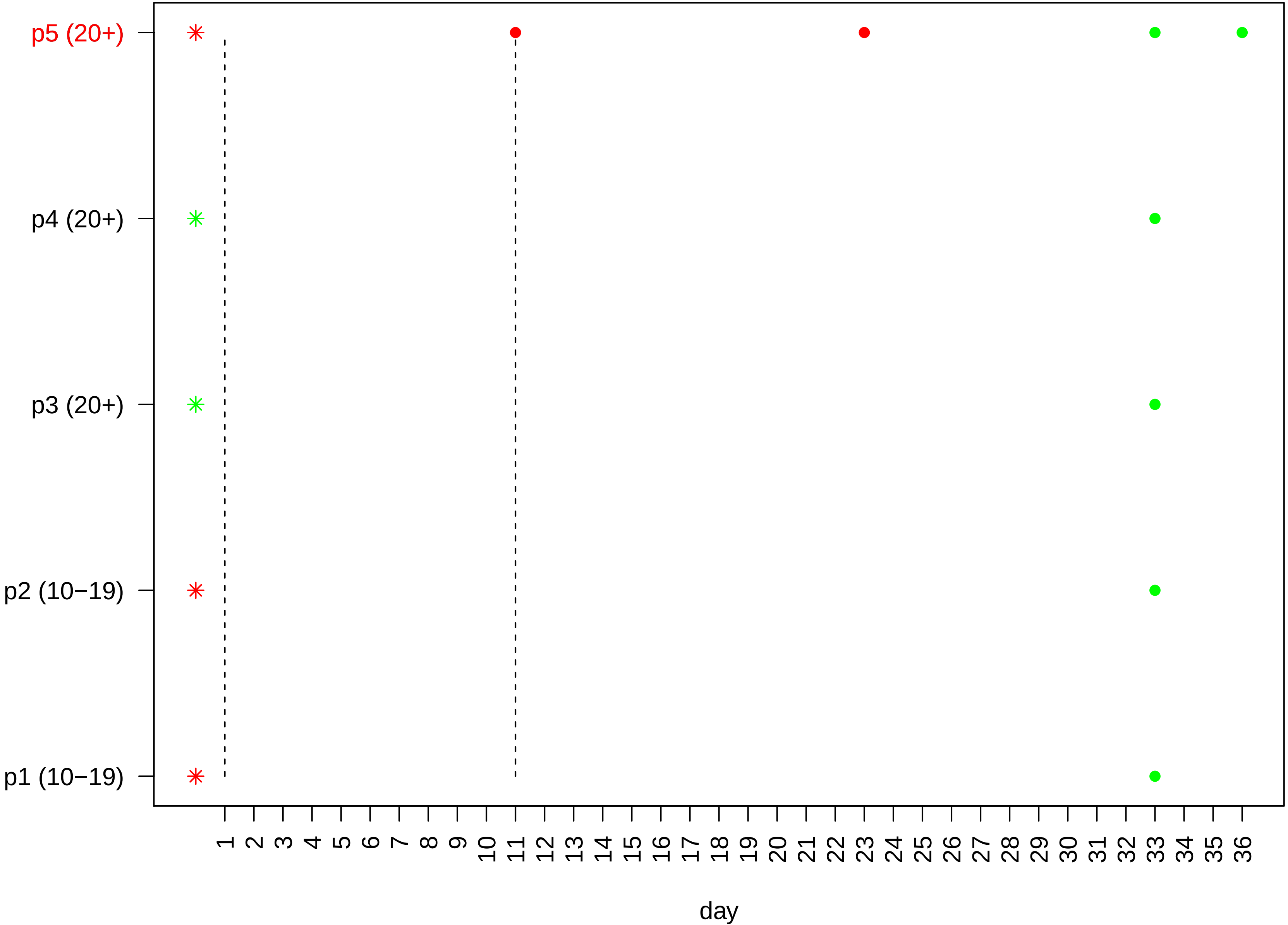

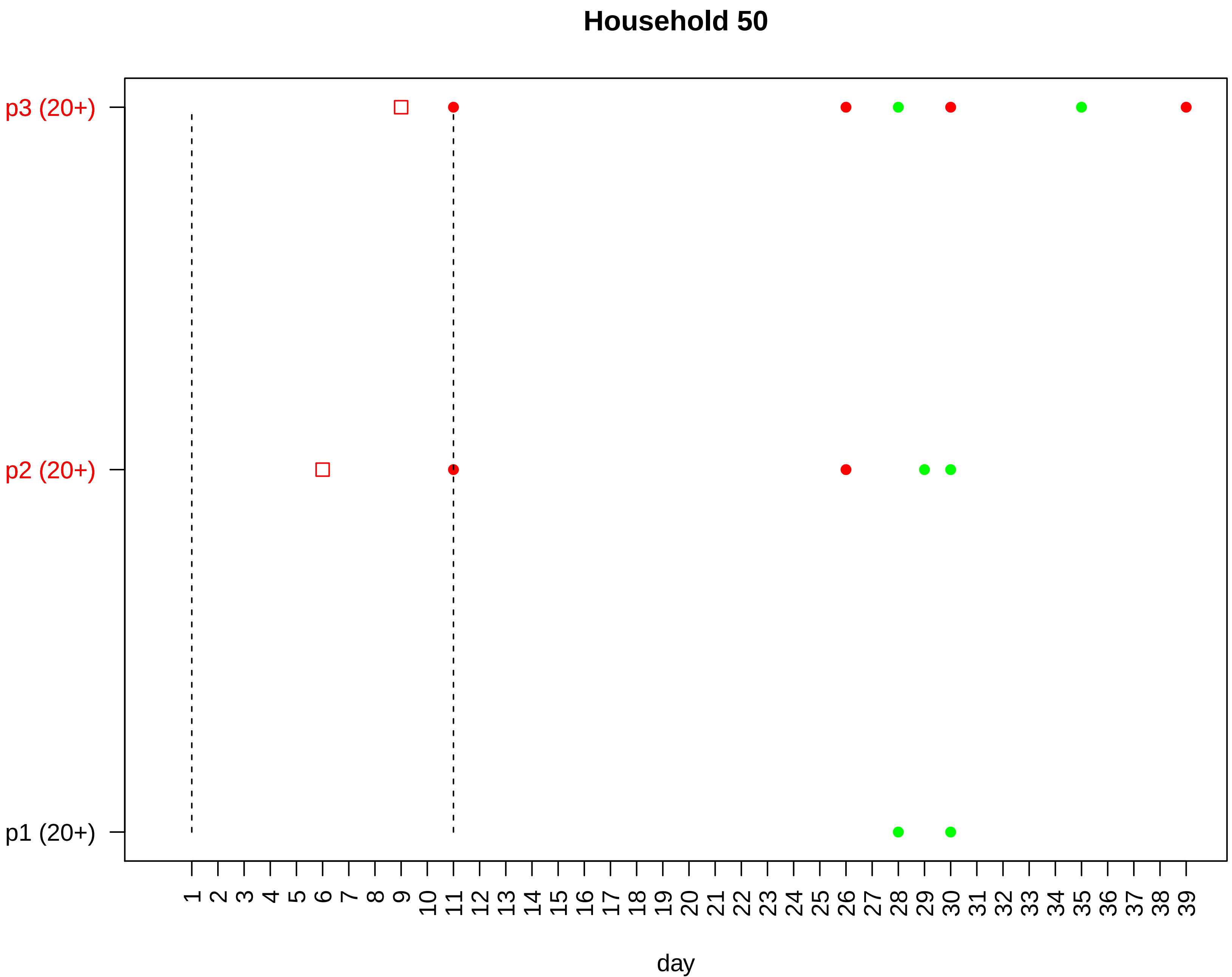

Household 50
p3 (20+)
p2 (20+)
p1 (20+)
1 2 3 4 5 6 7 8 9 10 11 12 13 14 15 16 17 18 19 20 21 22 23 24 25 26 27 28 29 30 31 32 33 34 35 36 37 38 39
day

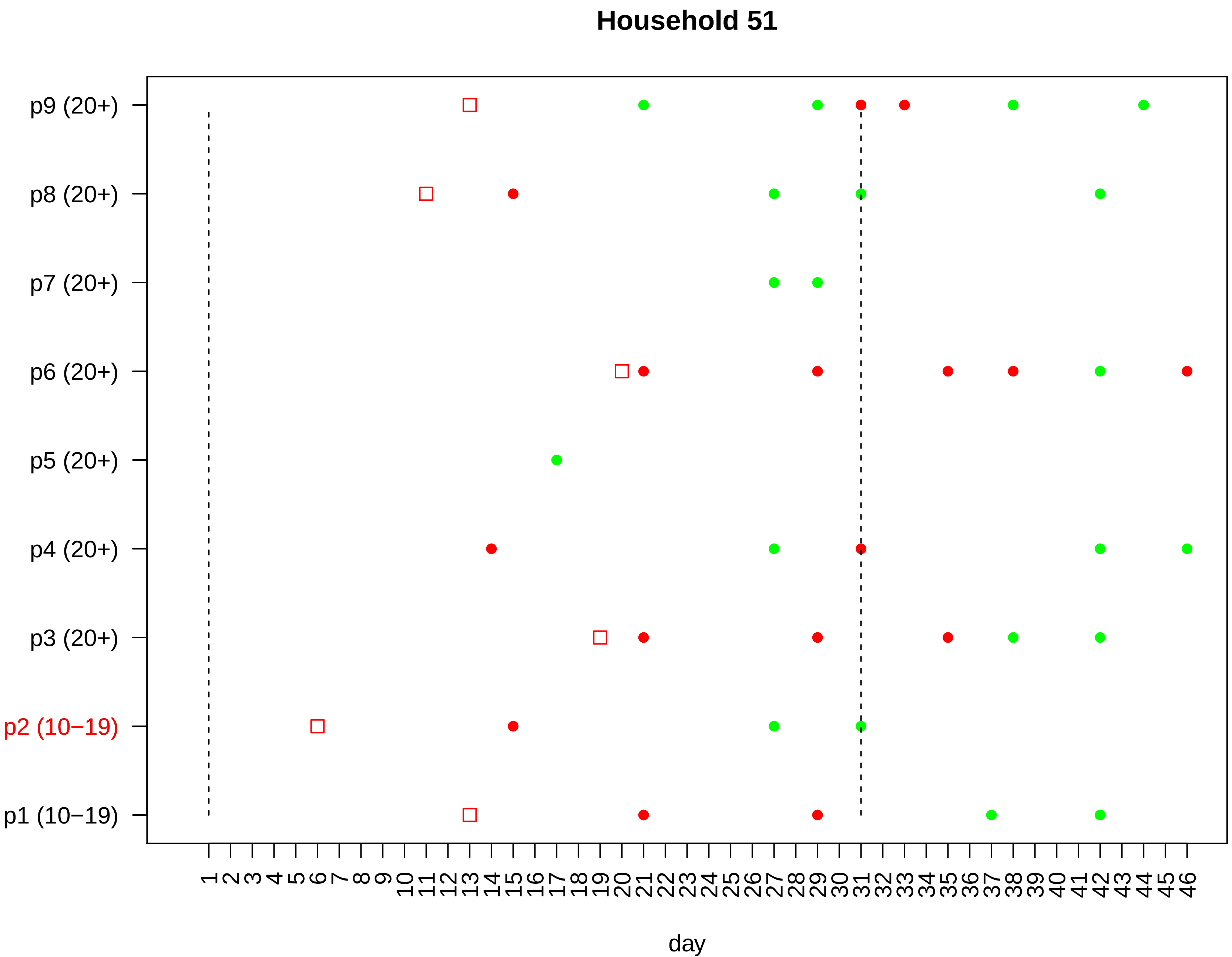

Household 51
p9 (20+)
p8 (20+)
p7 (20+)
p6 (20+)
p5 (20+)
p4 (20+)
p3 (20+)
p2 (10−19)
p1 (10−19)
1 2 3 4 5 6 7 8 9 10 11 12 13 14 15 16 17 18 19 20 21 22 23 24 25 26 27 28 29 30 31 32 33 34 35 36 37 38 39 40 41 42 43 44 45 46
day

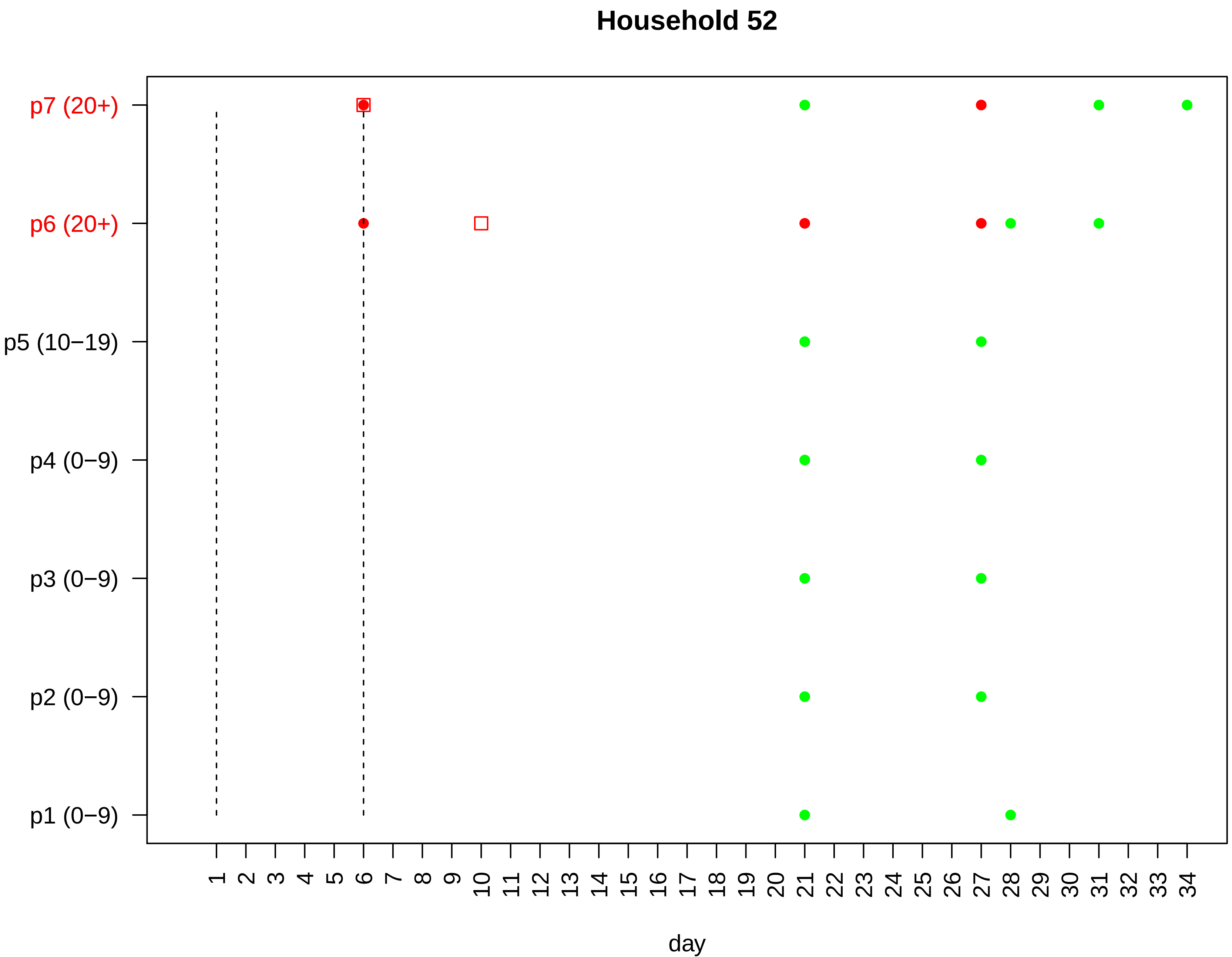

Household 52
p7 (20+)
p6 (20+)
p5 (10−19)
p4 (0−9)
p3 (0−9)
p2 (0−9)
p1 (0−9)
1 2 3 4 5 6 7 8 9 10 11 12 13 14 15 16 17 18 19 20 21 22 23 24 25 26 27 28 29 30 31 32 33 34
day

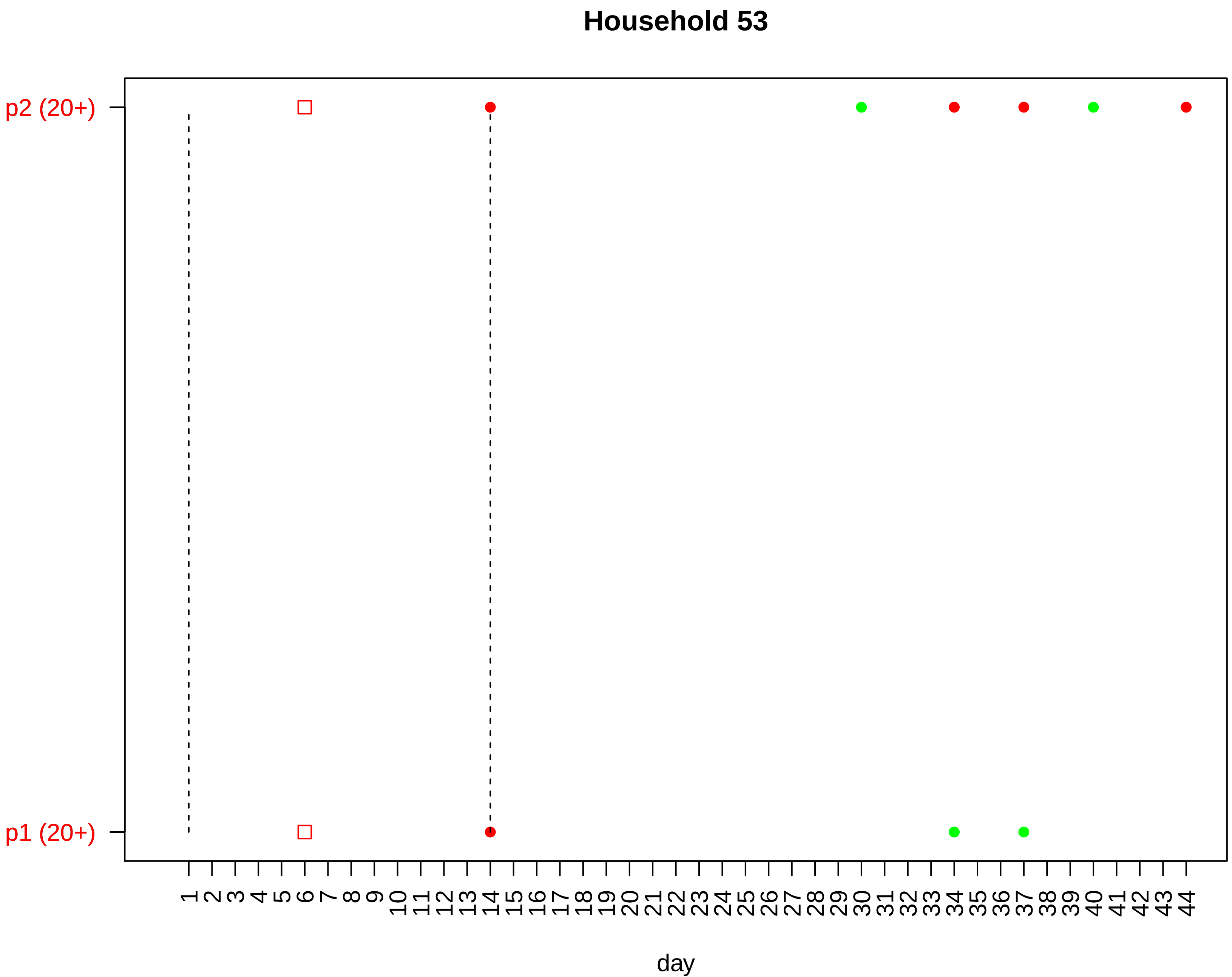

Household 53
p2 (20+)
p1 (20+)
1 2 3 4 5 6 7 8 9 10 11 12 13 14 15 16 17 18 19 20 21 22 23 24 25 26 27 28 29 30 31 32 33 34 35 36 37 38 39 40 41 42 43 44
day

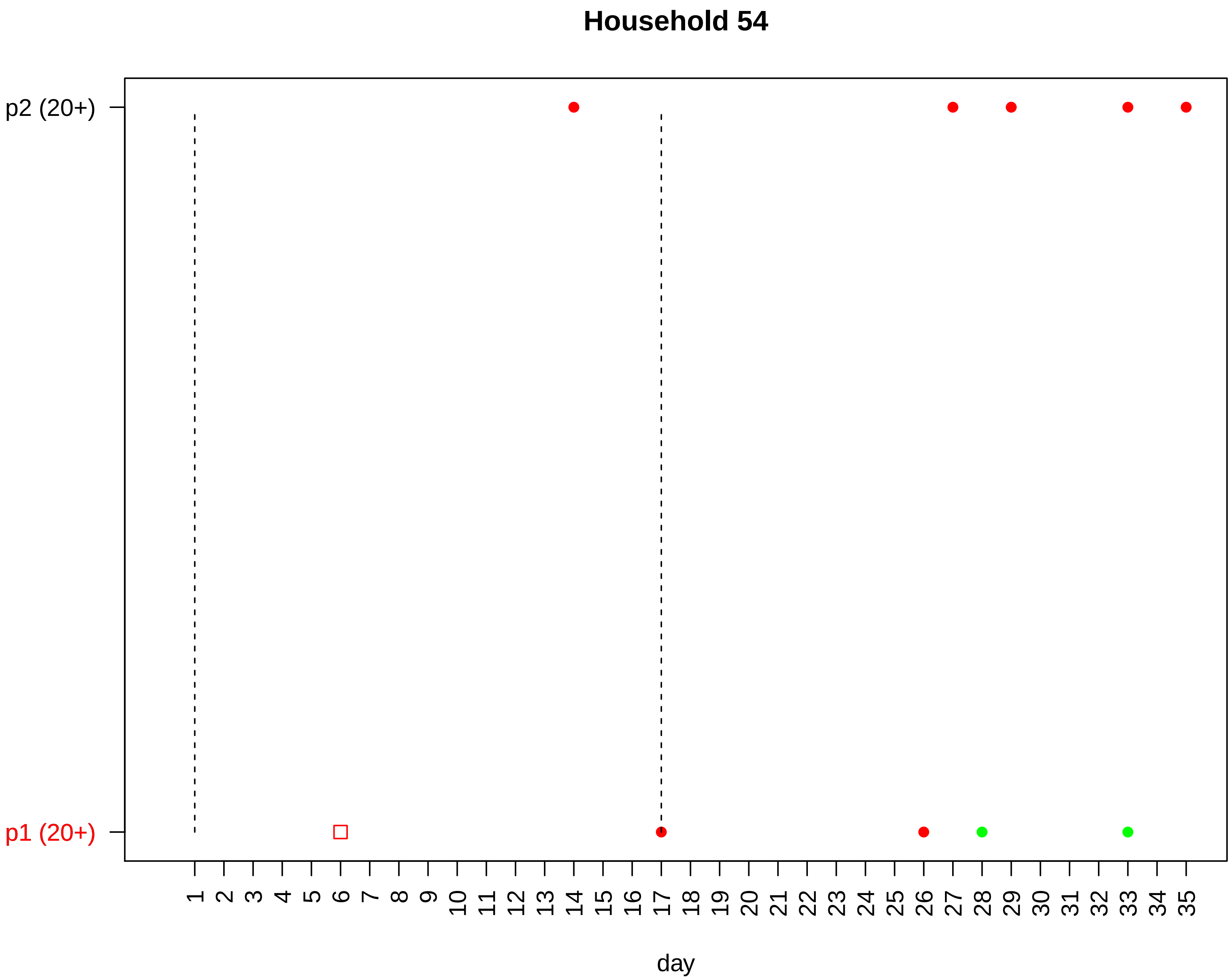

Household 54
p2 (20+)
p1 (20+)
1 2 3 4 5 6 7 8 9 10 11 12 13 14 15 16 17 18 19 20 21 22 23 24 25 26 27 28 29 30 31 32 33 34 35
day

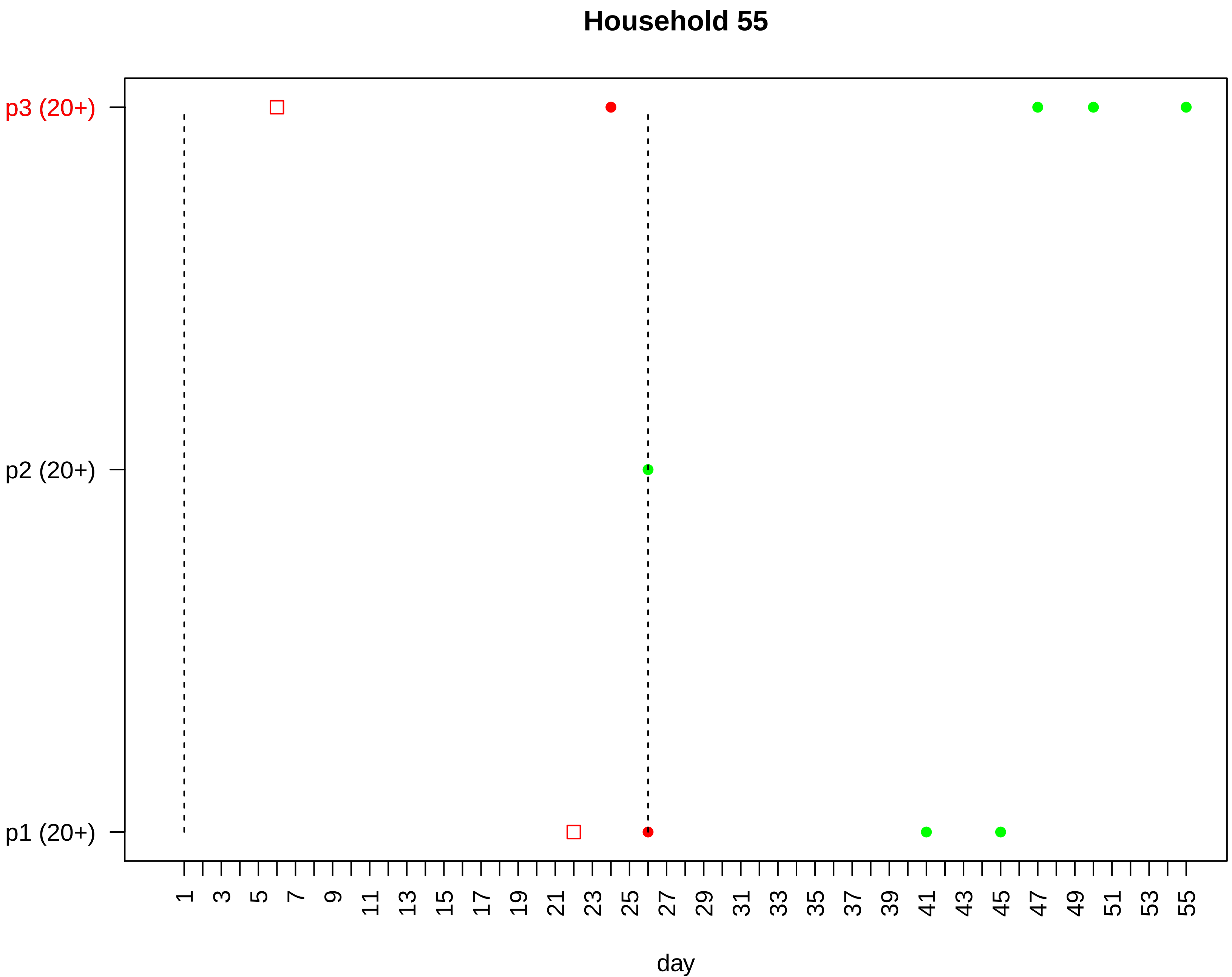

Household 55
p3 (20+)
p2 (20+)
p1 (20+)
1
3
5
7
9
11
13
15
17
19
21
23
25
27
29
31
33
35
37
39
41
43
45
47
49
51
53
55
day

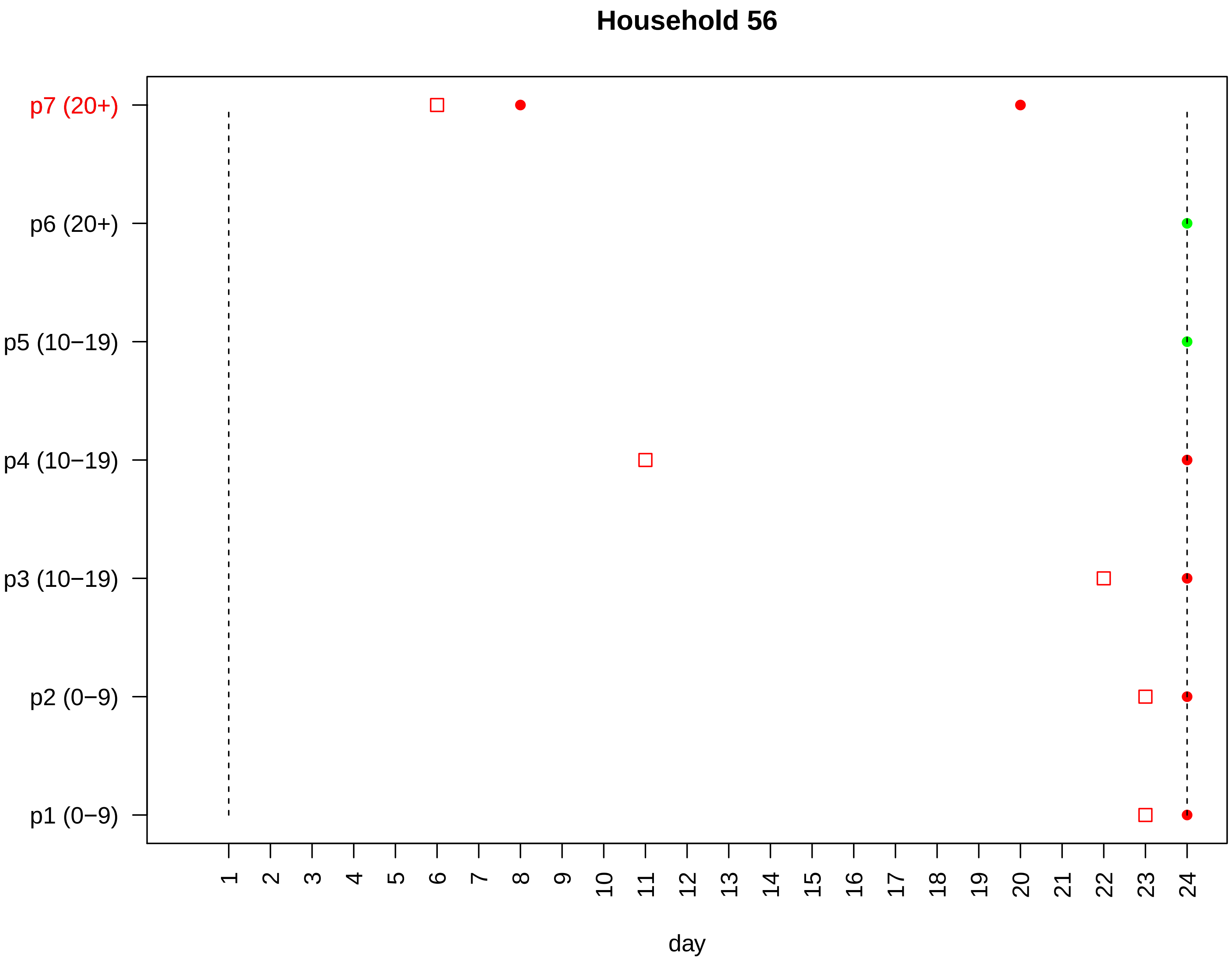

Household 56
p7 (20+)
p6 (20+)
p5 (10−19)
p4 (10−19)
p3 (10−19)
p2 (0−9)
p1 (0−9)
1 2 3 4 5 6 7 8 9 10 11 12 13 14 15 16 17 18 19 20 21 22 23 24
day

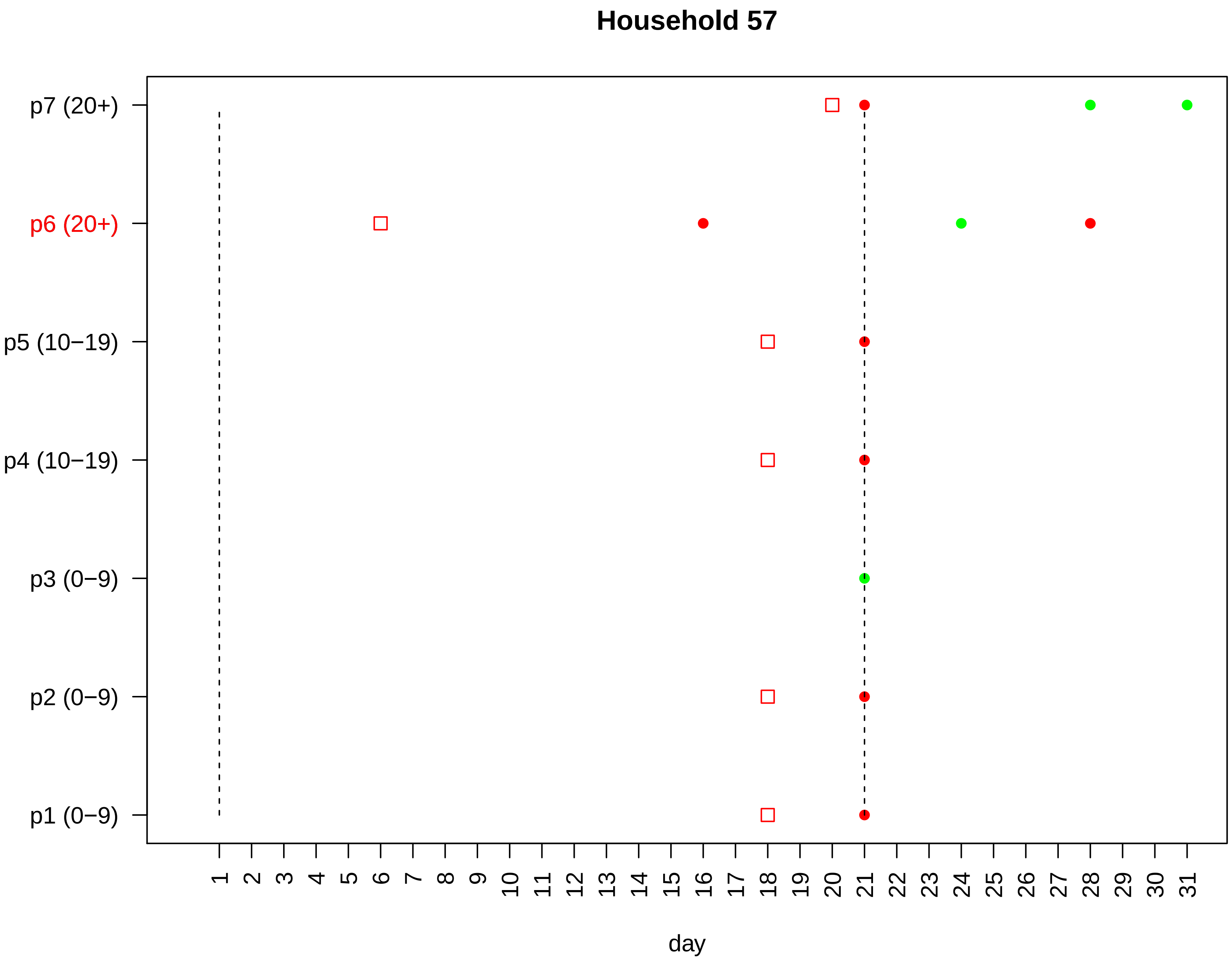

Household 57
p7 (20+)
p6 (20+)
p5 (10−19)
p4 (10−19)
p3 (0−9)
p2 (0−9)
p1 (0−9)
1 2 3 4 5 6 7 8 9 10 11 12 13 14 15 16 17 18 19 20 21 22 23 24 25 26 27 28 29 30 31
day

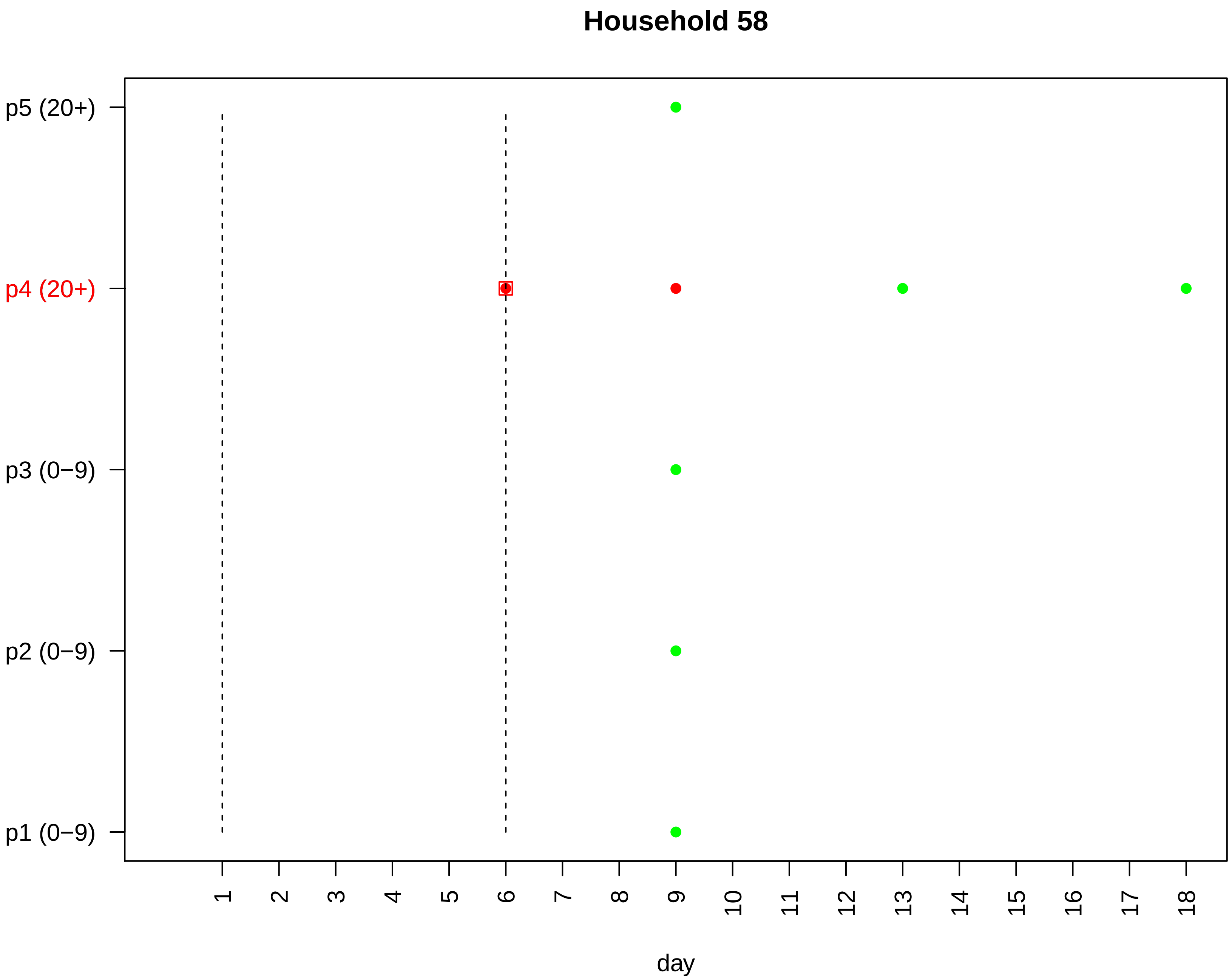

Household 58
p5 (20+)
p4 (20+)
p3 (0−9)
p2 (0−9)
p1 (0−9)
1
2
3
4
5
6
7
8
9
10
11
12
13
14
15
16
17
18
day

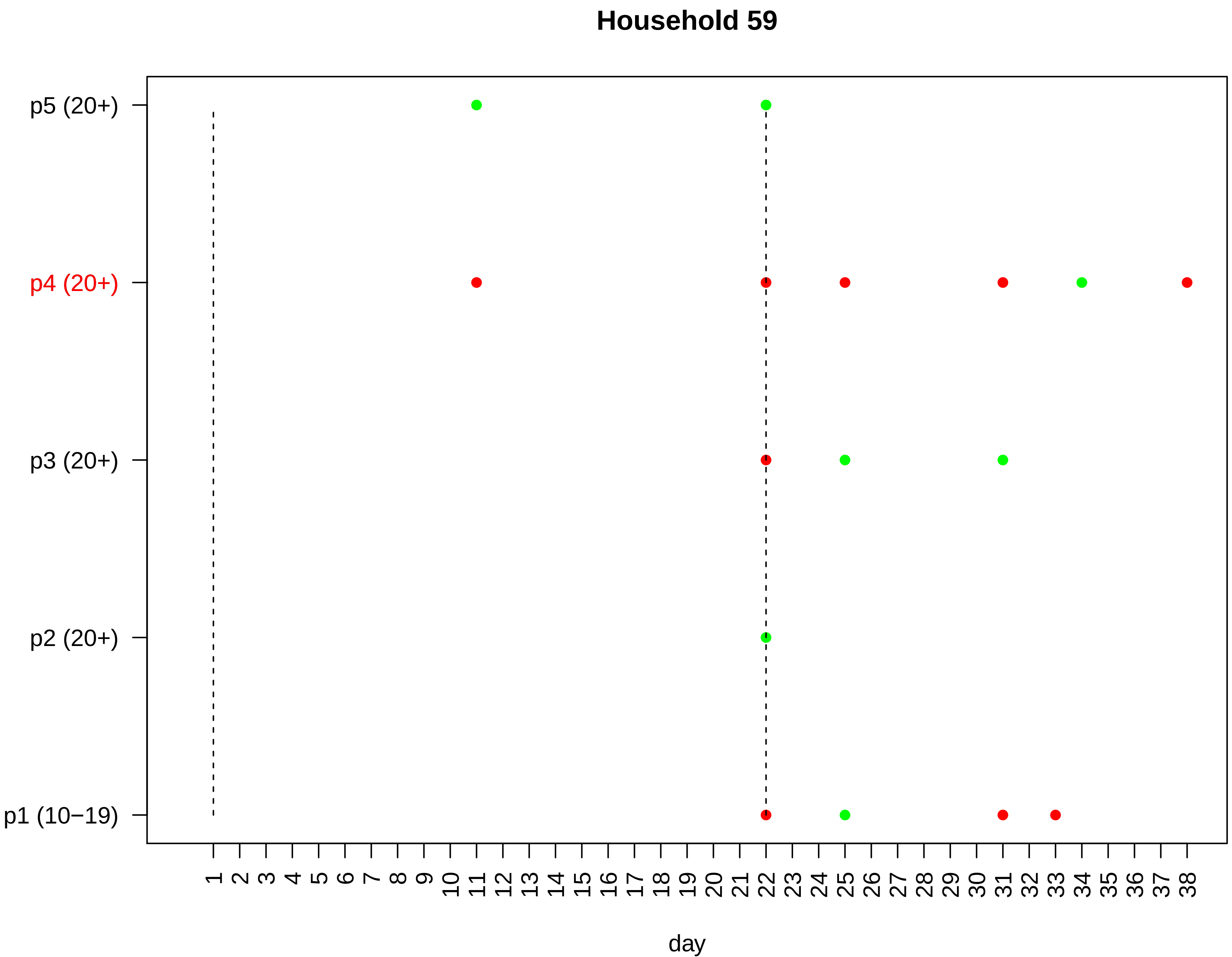
Household 59
p5 (20+)
p4 (20+)
p3 (20+)
p2 (20+)
p1 (10−19)
1 2 3 4 5 6 7 8 9 10 11 12 13 14 15 16 17 18 19 20 21 22 23 24 25 26 27 28 29 30 31 32 33 34 35 36 37 38
day

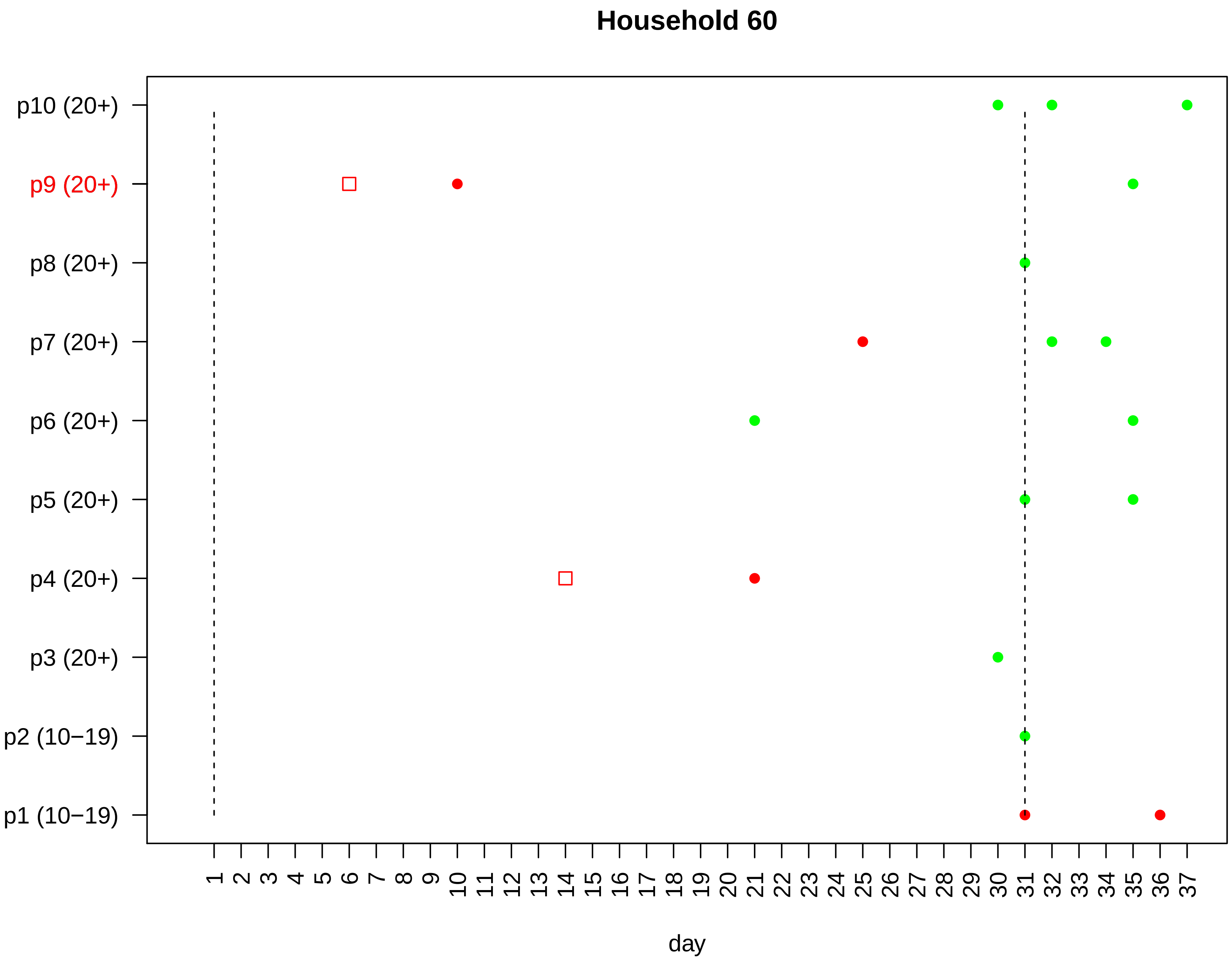

Household 60
p10 (20+)
p9 (20+)
p8 (20+)
p7 (20+)
p6 (20+)
p5 (20+)
p4 (20+)
p3 (20+)
p2 (10−19)
p1 (10−19)
1 2 3 4 5 6 7 8 9 10 11 12 13 14 15 16 17 18 19 20 21 22 23 24 25 26 27 28 29 30 31 32 33 34 35 36 37
day

Household 61

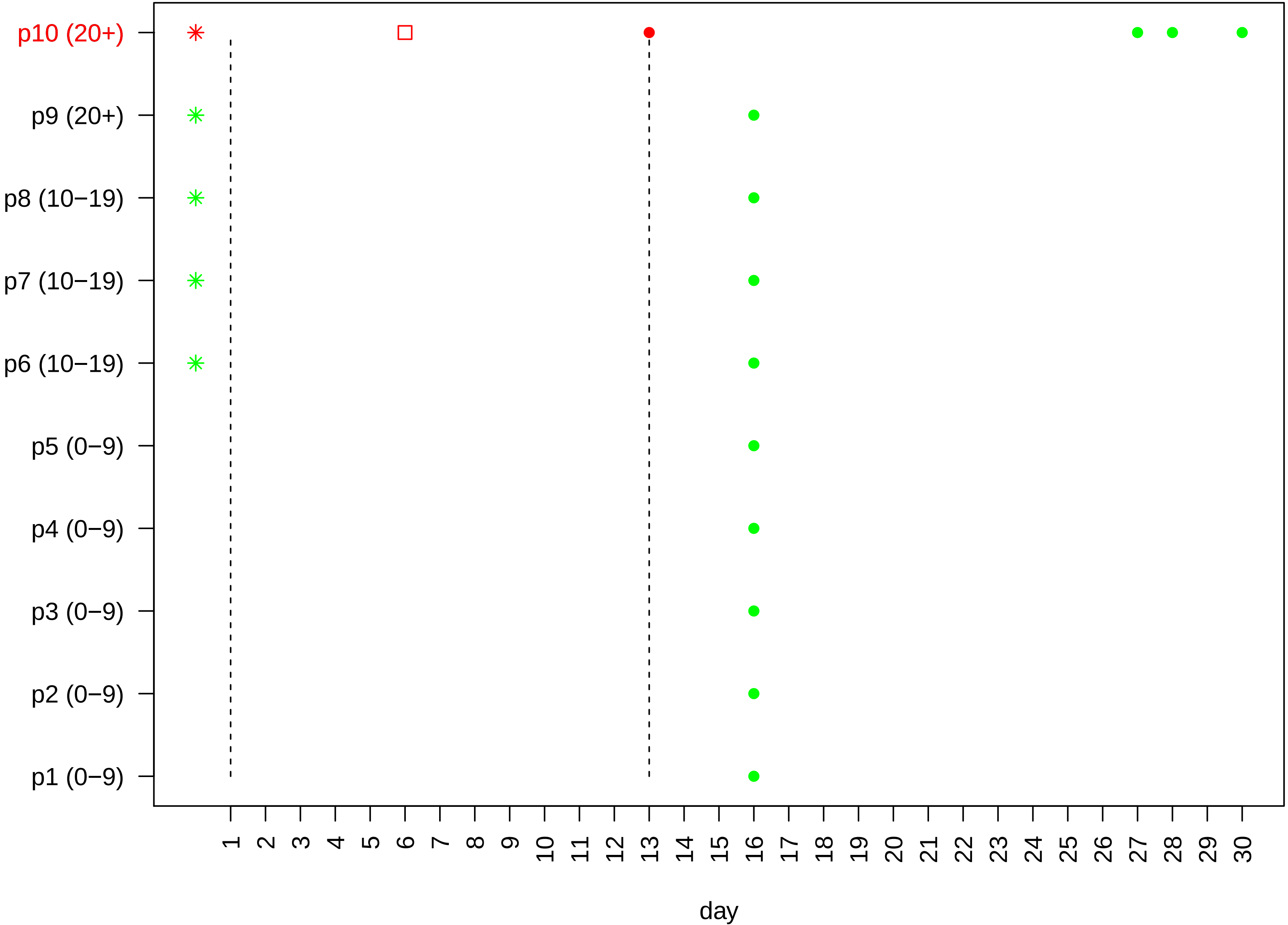

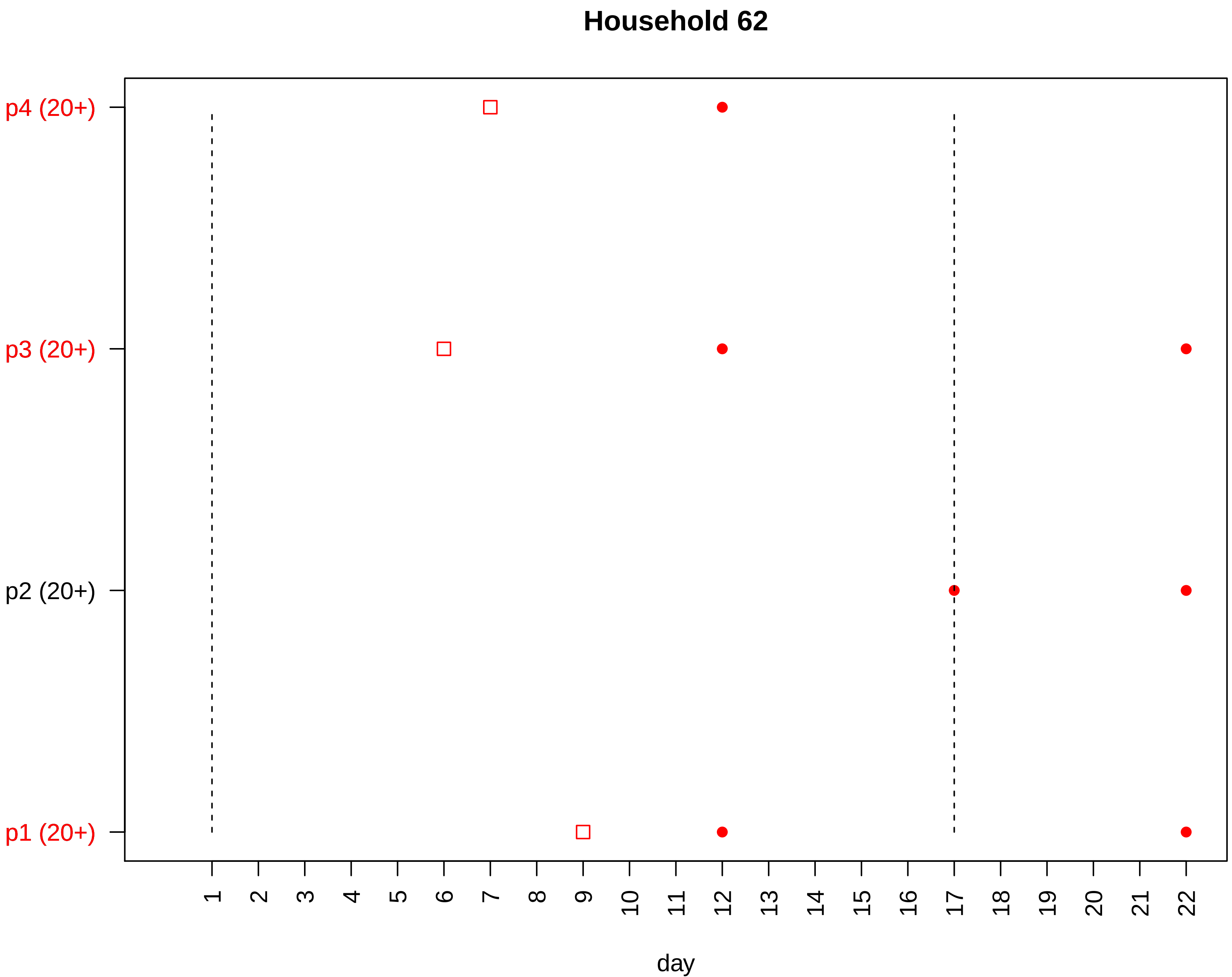
Household 62
p4 (20+)
p3 (20+)
p2 (20+)
p1 (20+)
1
2
3
4
5
6
7
8
9
10
11
12
13
14
15
16
17
18
19
20
21
22
day

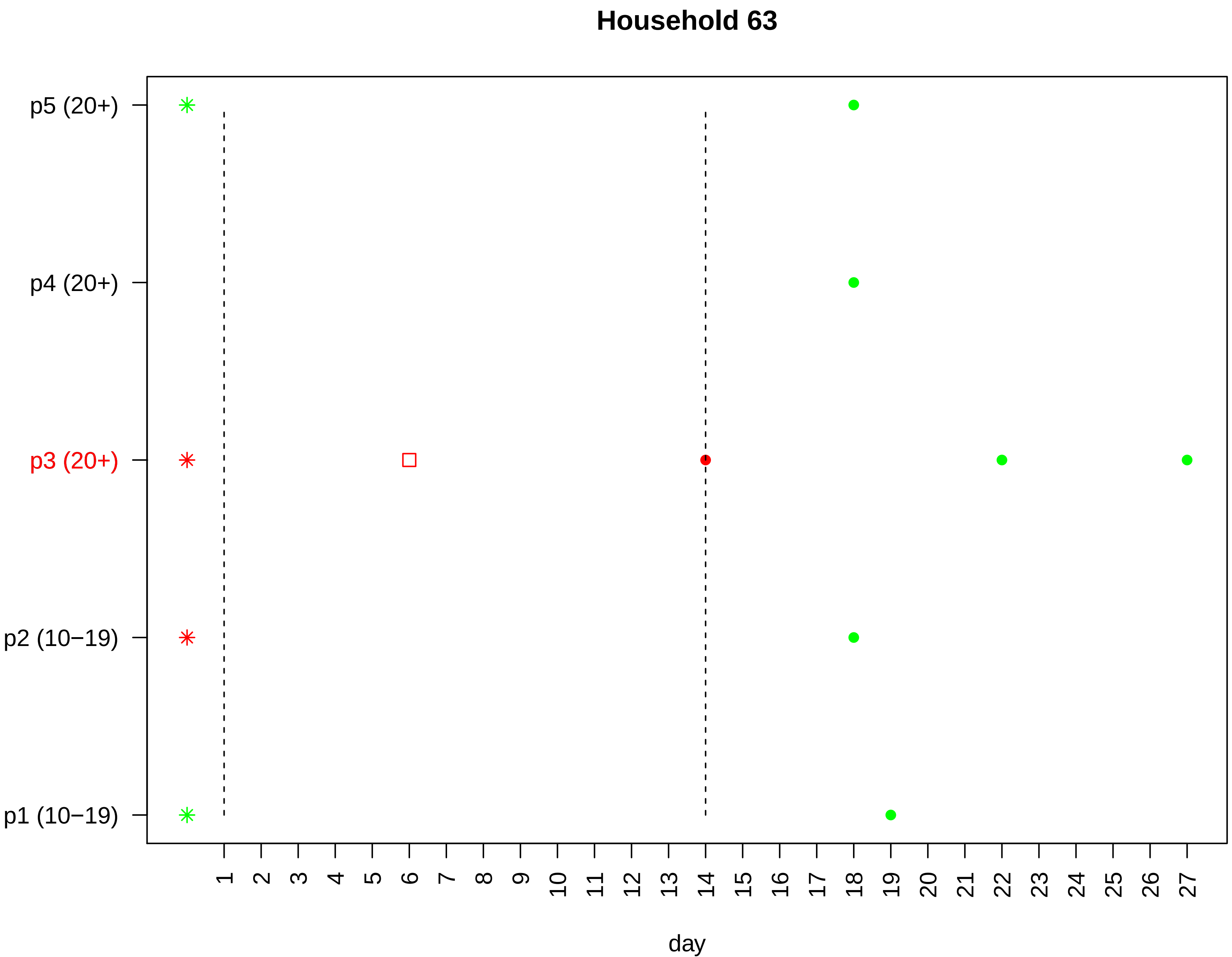
Household 63
p5 (20+)
p4 (20+)
p3 (20+)
p2 (10−19)
p1 (10−19)
1 2 3 4 5 6 7 8 9 10 11 12 13 14 15 16 17 18 19 20 21 22 23 24 25 26 27
day

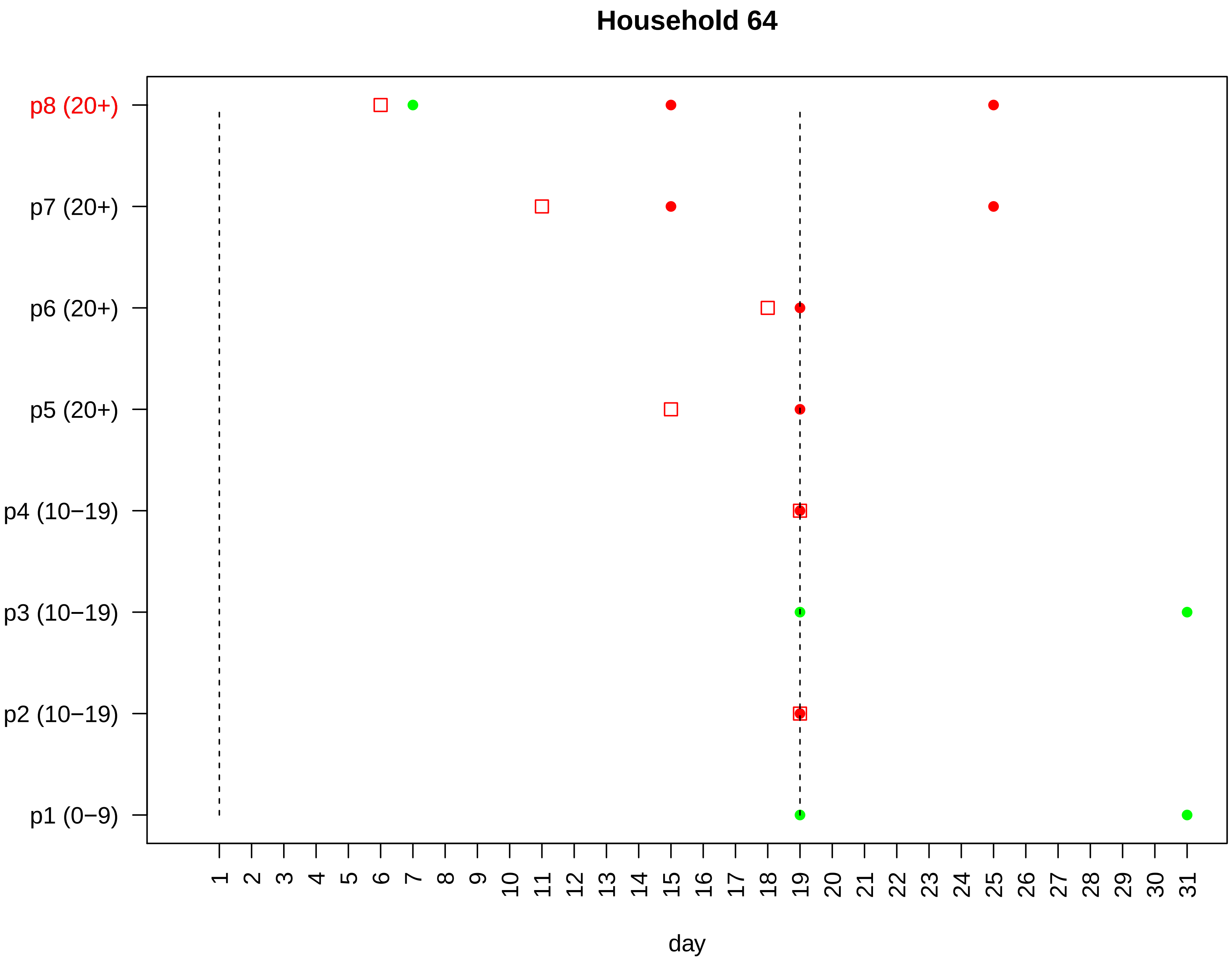

Household 64
p8 (20+)
p7 (20+)
p6 (20+)
p5 (20+)
p4 (10−19)
p3 (10−19)
p2 (10−19)
p1 (0−9)
1 2 3 4 5 6 7 8 9 10 11 12 13 14 15 16 17 18 19 20 21 22 23 24 25 26 27 28 29 30 31
day

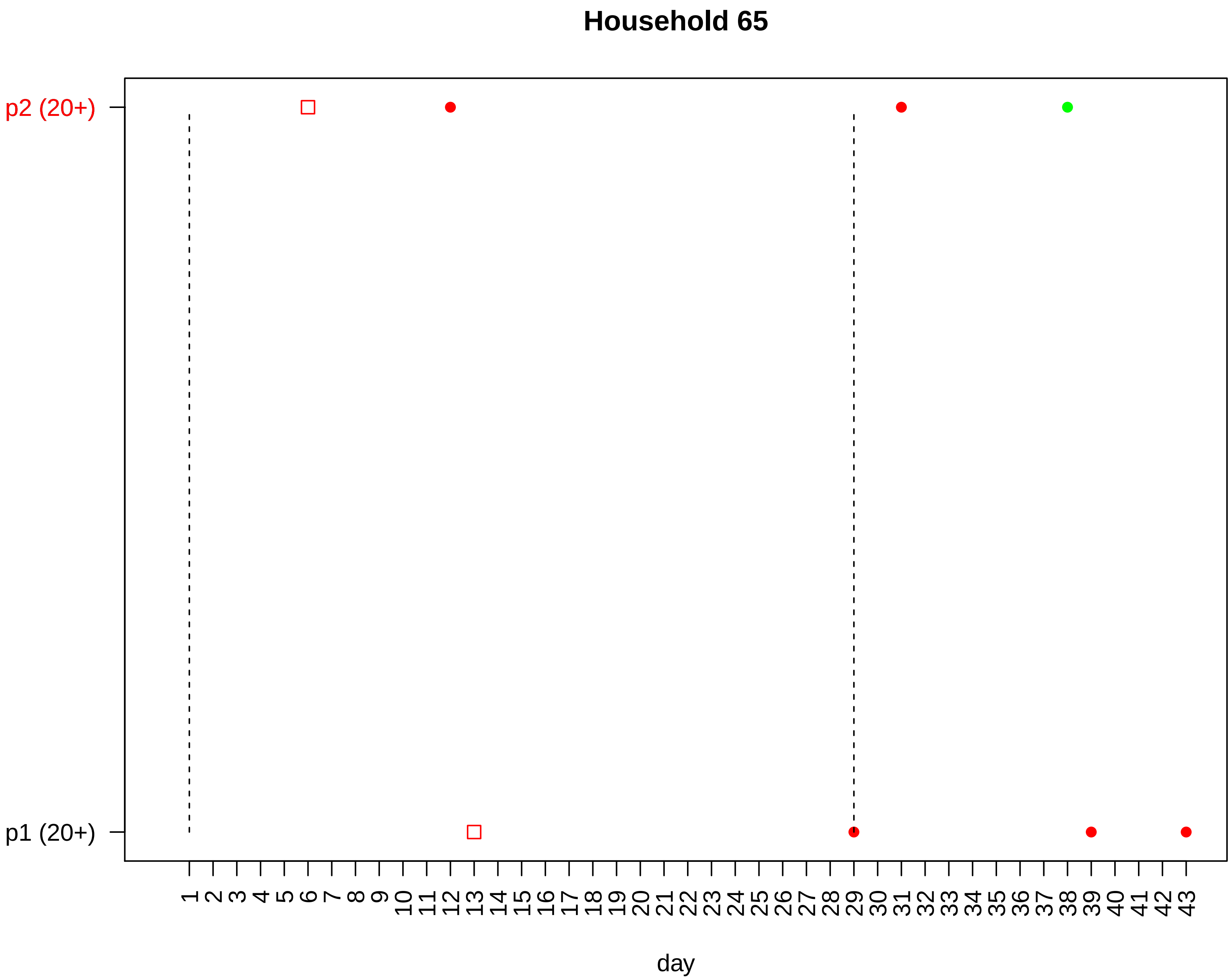
Household 65
p2 (20+)
p1 (20+)
1 2 3 4 5 6 7 8 9 10 11 12 13 14 15 16 17 18 19 20 21 22 23 24 25 26 27 28 29 30 31 32 33 34 35 36 37 38 39 40 41 42 43
day

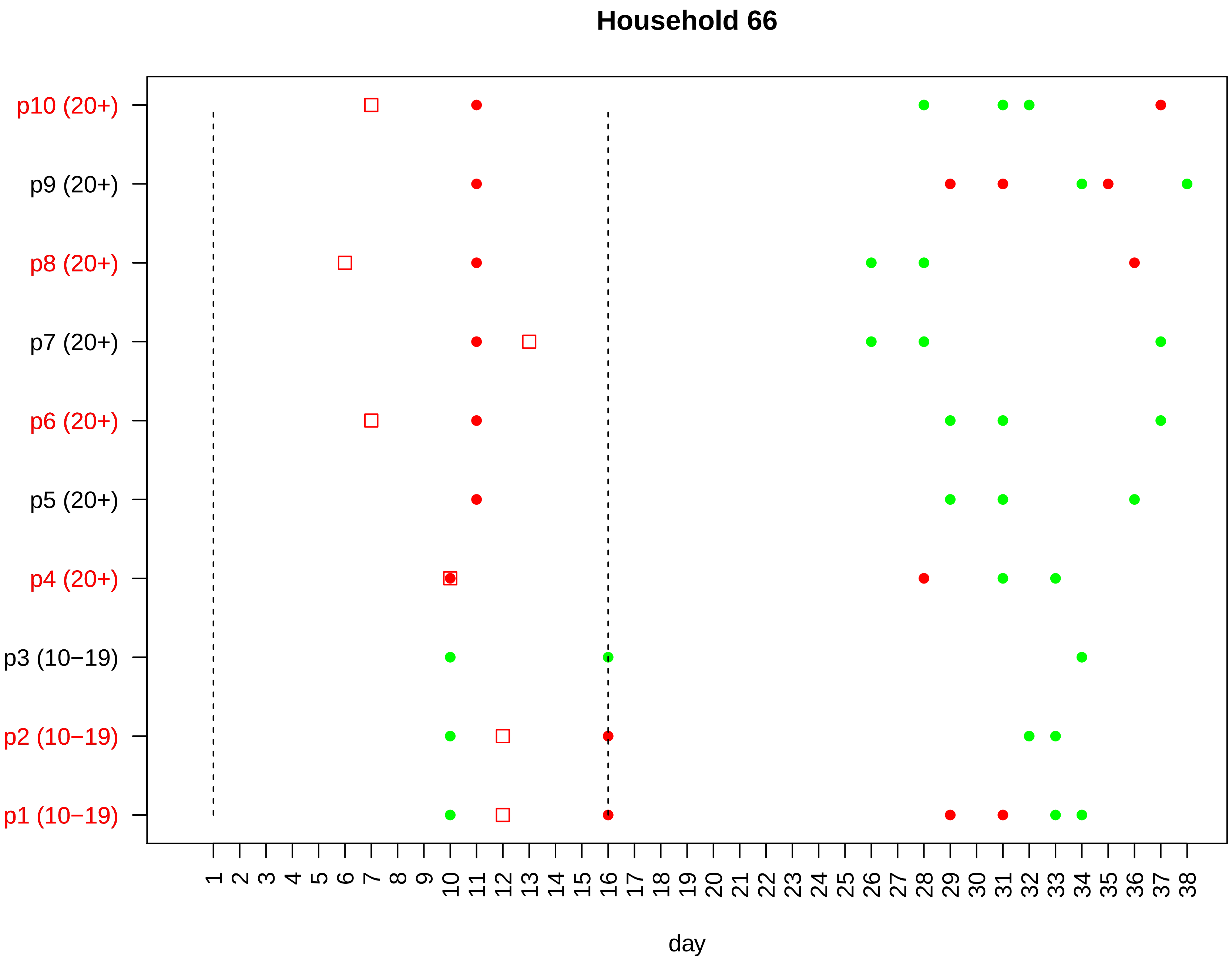

Household 66
p10 (20+)
p9 (20+)
p8 (20+)
p7 (20+)
p6 (20+)
p5 (20+)
p4 (20+)
p3 (10−19)
p2 (10−19)
p1 (10−19)
1 2 3 4 5 6 7 8 9 10 11 12 13 14 15 16 17 18 19 20 21 22 23 24 25 26 27 28 29 30 31 32 33 34 35 36 37 38
day

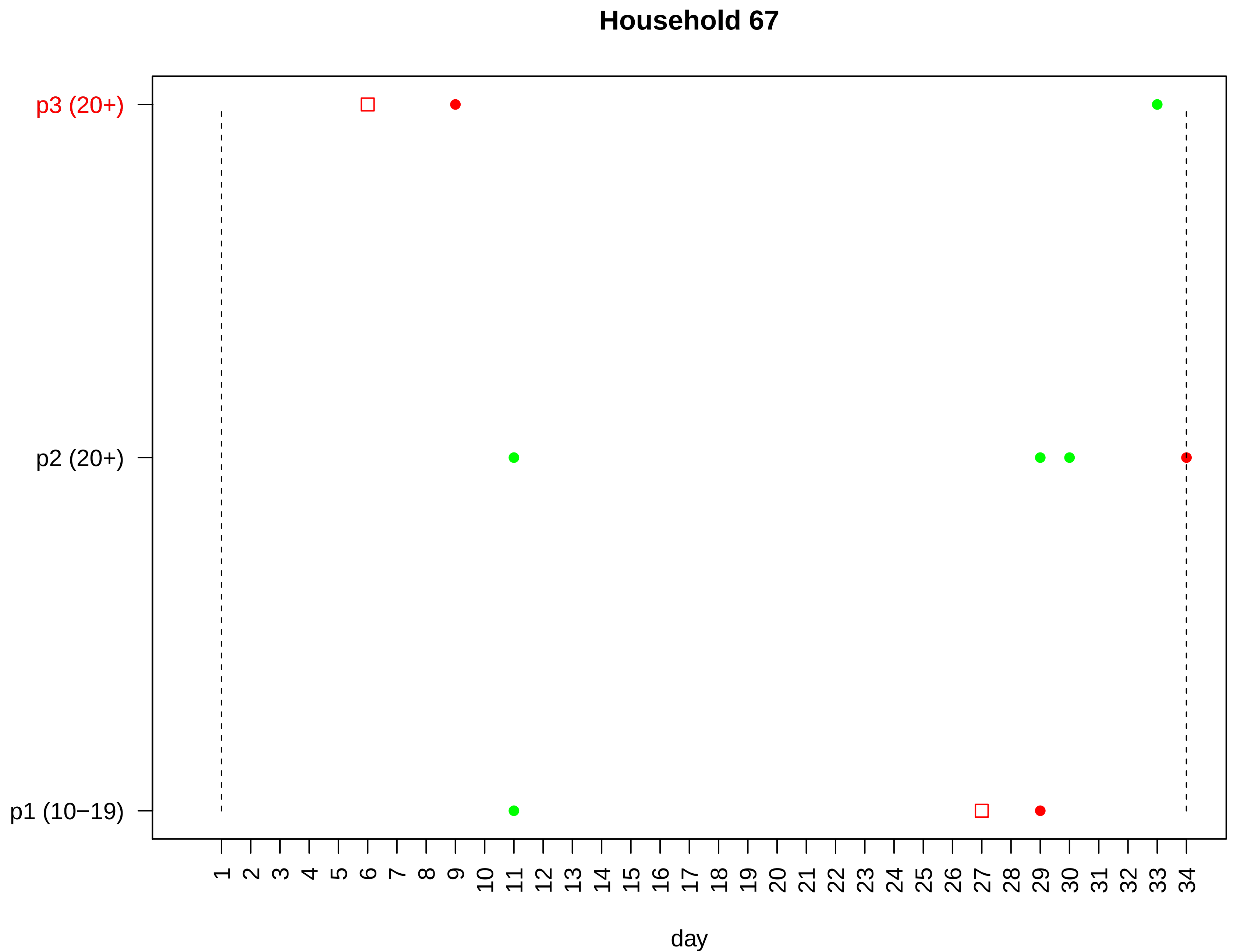

Household 67
p3 (20+)
p2 (20+)
p1 (10−19)
1 2 3 4 5 6 7 8 9 10 11 12 13 14 15 16 17 18 19 20 21 22 23 24 25 26 27 28 29 30 31 32 33 34
day

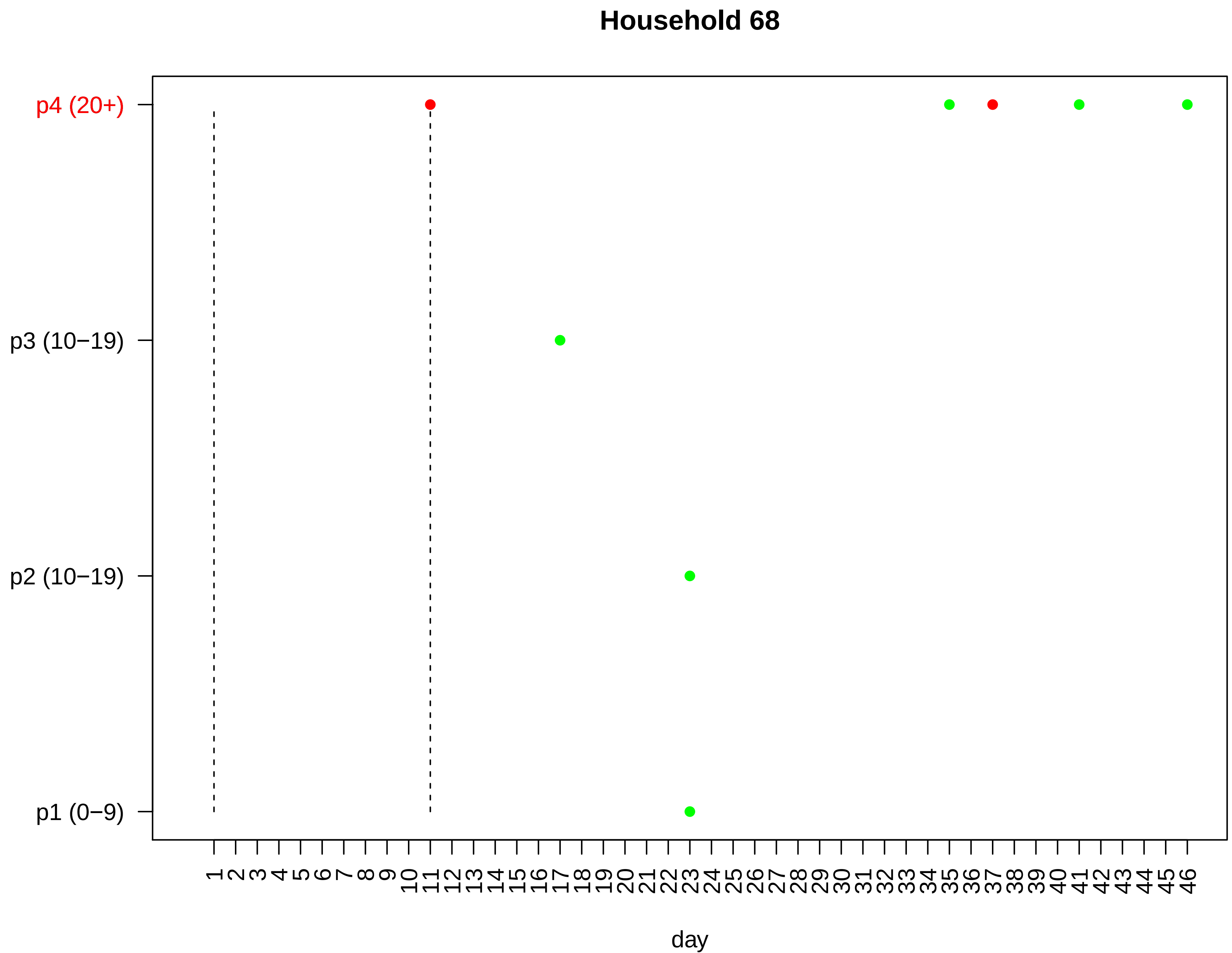

Household 68
p4 (20+)
p3 (10−19)
p2 (10−19)
p1 (0−9)
1 2 3 4 5 6 7 8 9 10 11 12 13 14 15 16 17 18 19 20 21 22 23 24 25 26 27 28 29 30 31 32 33 34 35 36 37 38 39 40 41 42 43 44 45 46
day

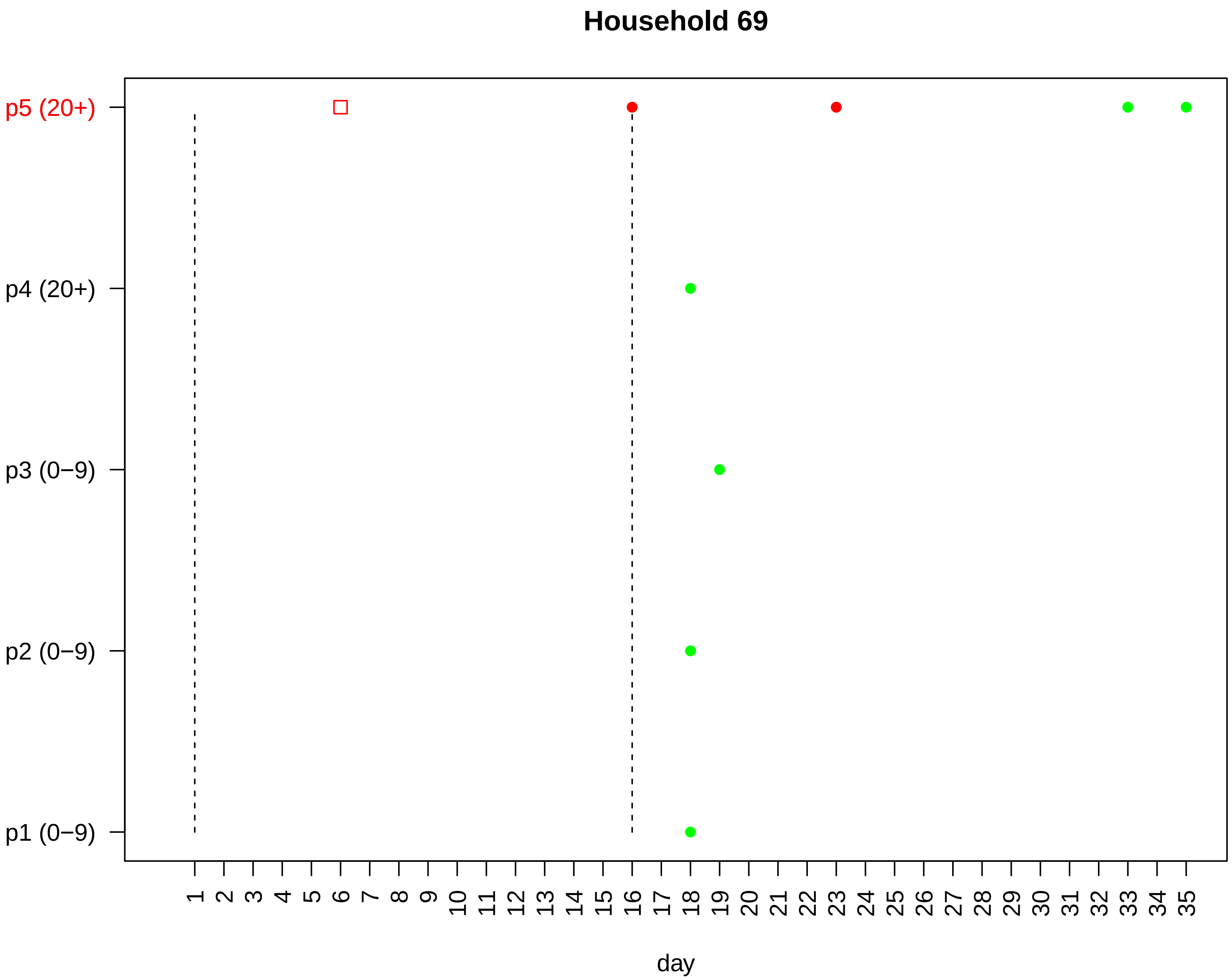

Household 69
p5 (20+)
p4 (20+)
p3 (0−9)
p2 (0−9)
p1 (0−9)
1 2 3 4 5 6 7 8 9 10 11 12 13 14 15 16 17 18 19 20 21 22 23 24 25 26 27 28 29 30 31 32 33 34 35
day

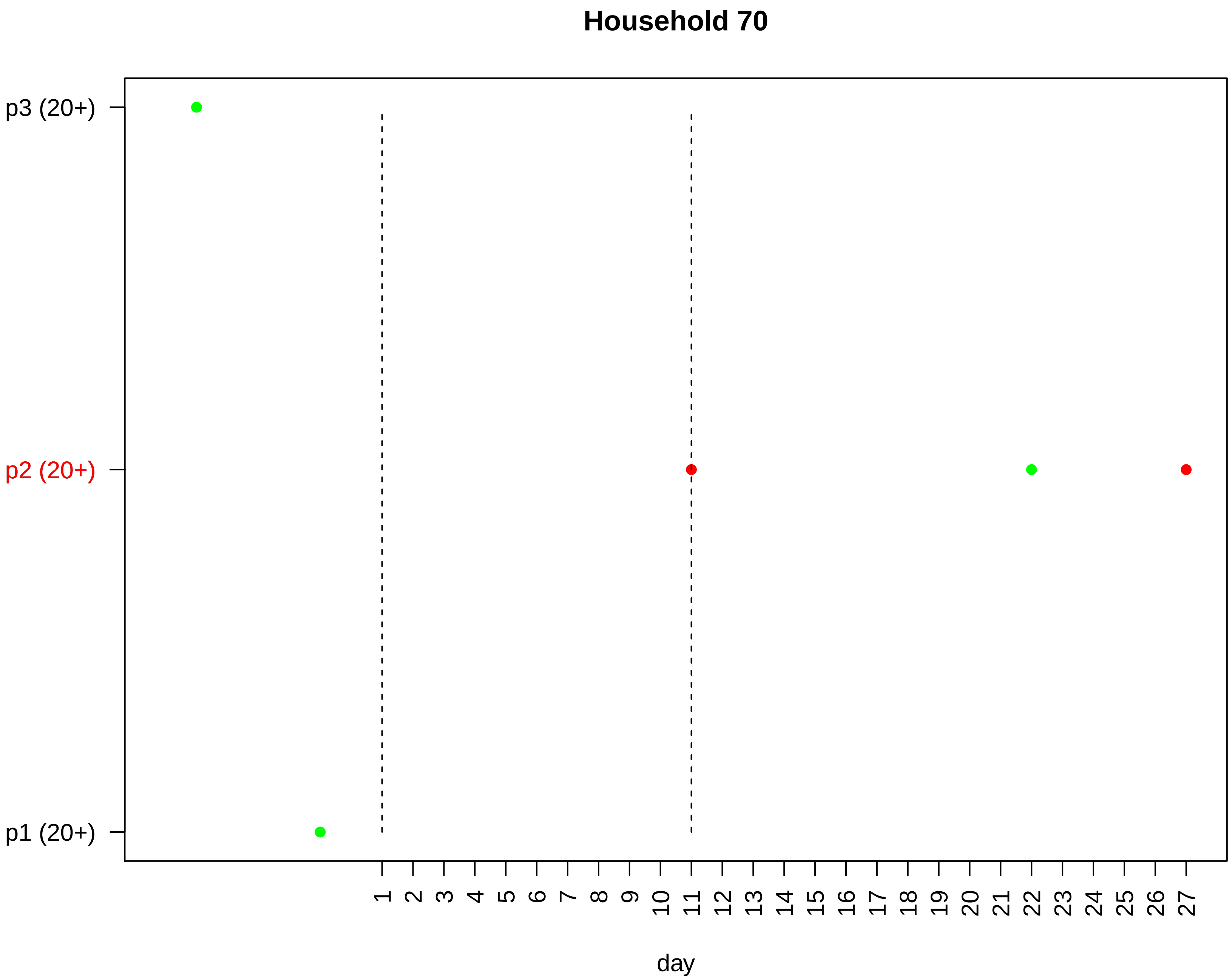

Household 70
p3 (20+)
p2 (20+)
p1 (20+)
1 2 3 4 5 6 7 8 9 10 11 12 13 14 15 16 17 18 19 20 21 22 23 24 25 26 27
day

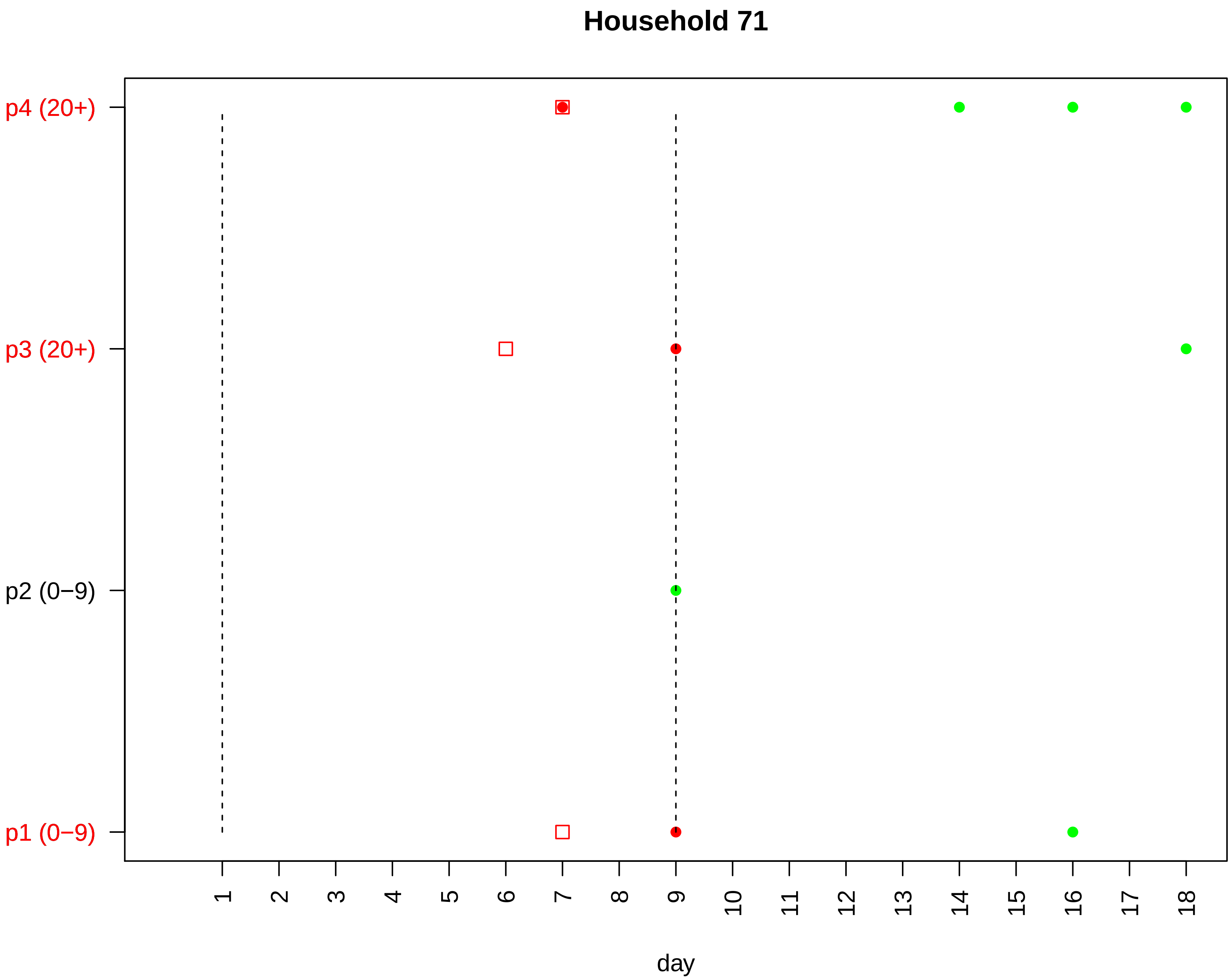

Household 71
p4 (20+)
p3 (20+)
p2 (0−9)
p1 (0−9)
1
2
3
4
5
6
7
8
9
10
11
12
13
14
15
16
17
18
day

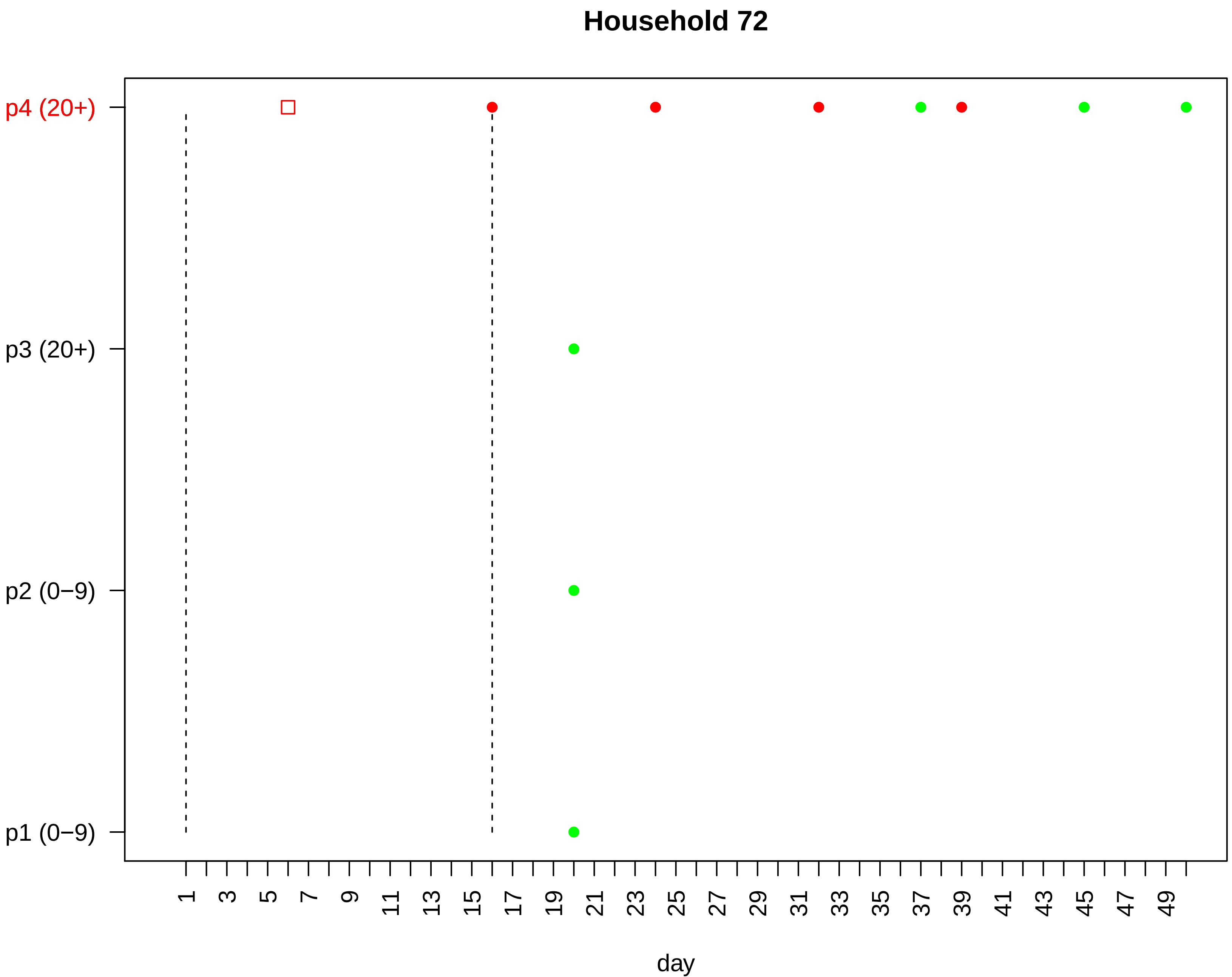

Household 72
p4 (20+)
p3 (20+)
p2 (0−9)
p1 (0−9)
1
3
5
7
9
11
13
15
17
19
21
23
25
27
29
31
33
35
37
39
41
43
45
47
49
day

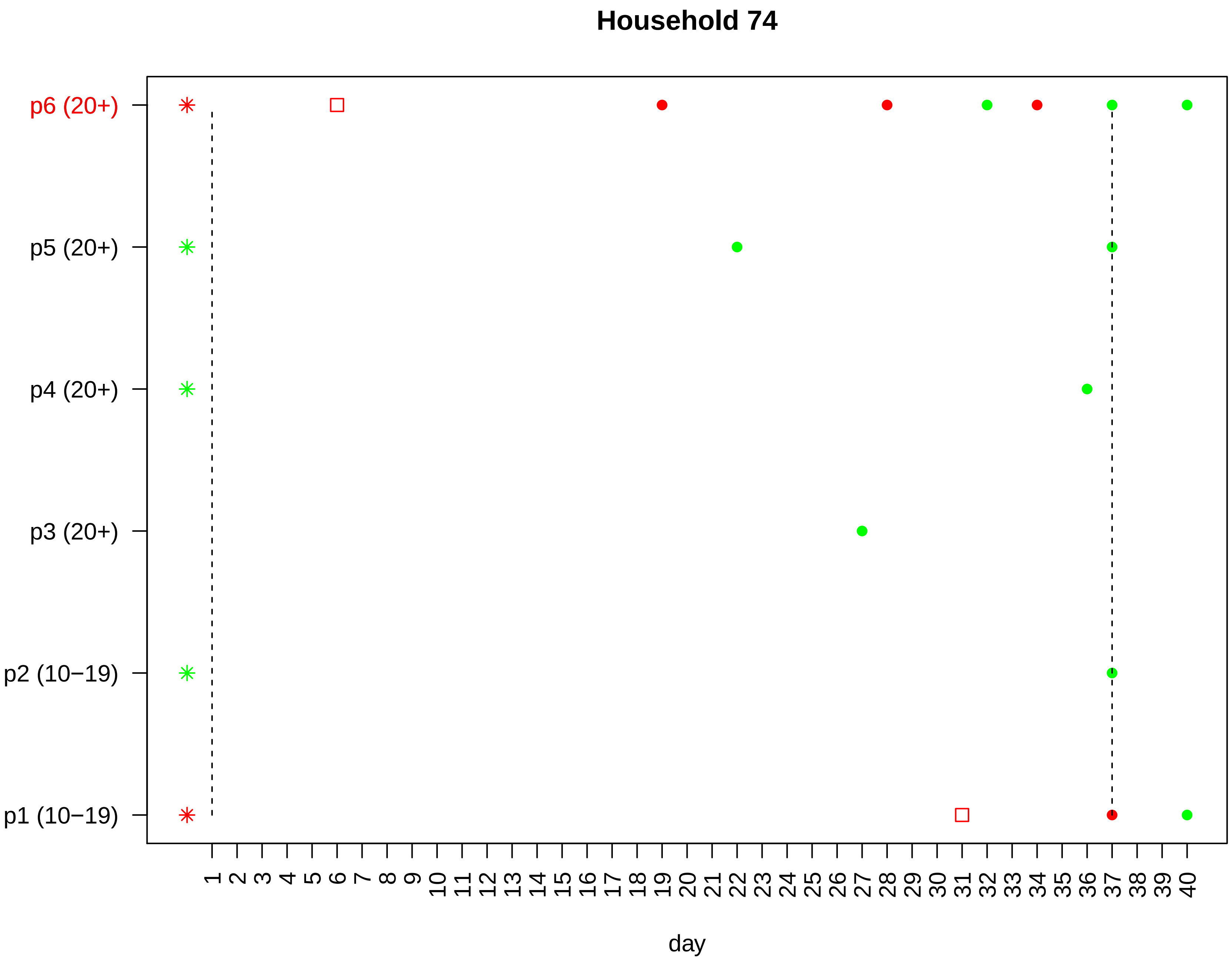

Household 74
p6 (20+)
p5 (20+)
p4 (20+)
p3 (20+)
p2 (10−19)
p1 (10−19)
1 2 3 4 5 6 7 8 9 10 11 12 13 14 15 16 17 18 19 20 21 22 23 24 25 26 27 28 29 30 31 32 33 34 35 36 37 38 39 40
day

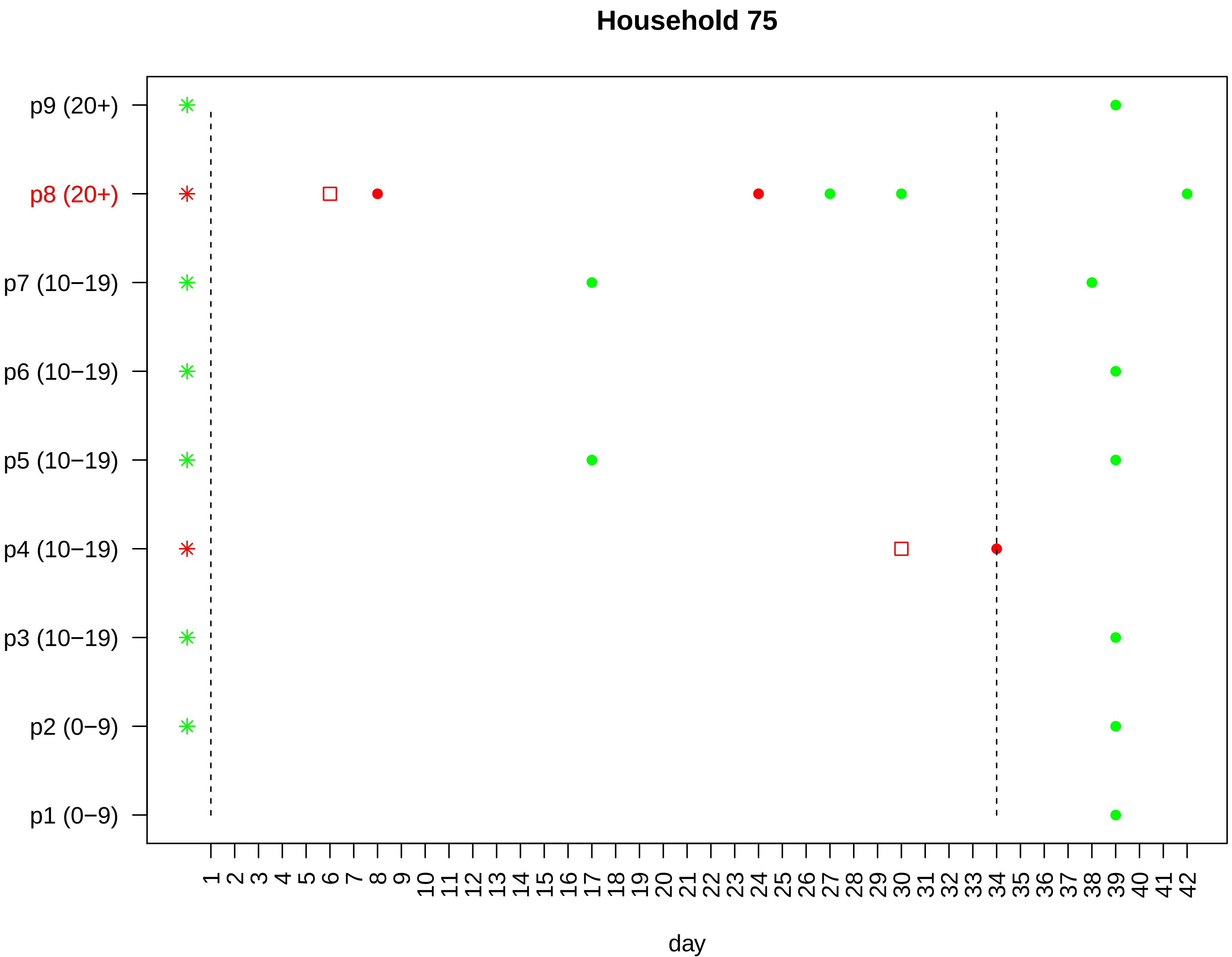

Household 75
p9 (20+)
p8 (20+)
p7 (10−19)
p6 (10−19)
p5 (10−19)
p4 (10−19)
p3 (10−19)
p2 (0−9)
p1 (0−9)
1 2 3 4 5 6 7 8 9 10 11 12 13 14 15 16 17 18 19 20 21 22 23 24 25 26 27 28 29 30 31 32 33 34 35 36 37 38 39 40 41 42
day

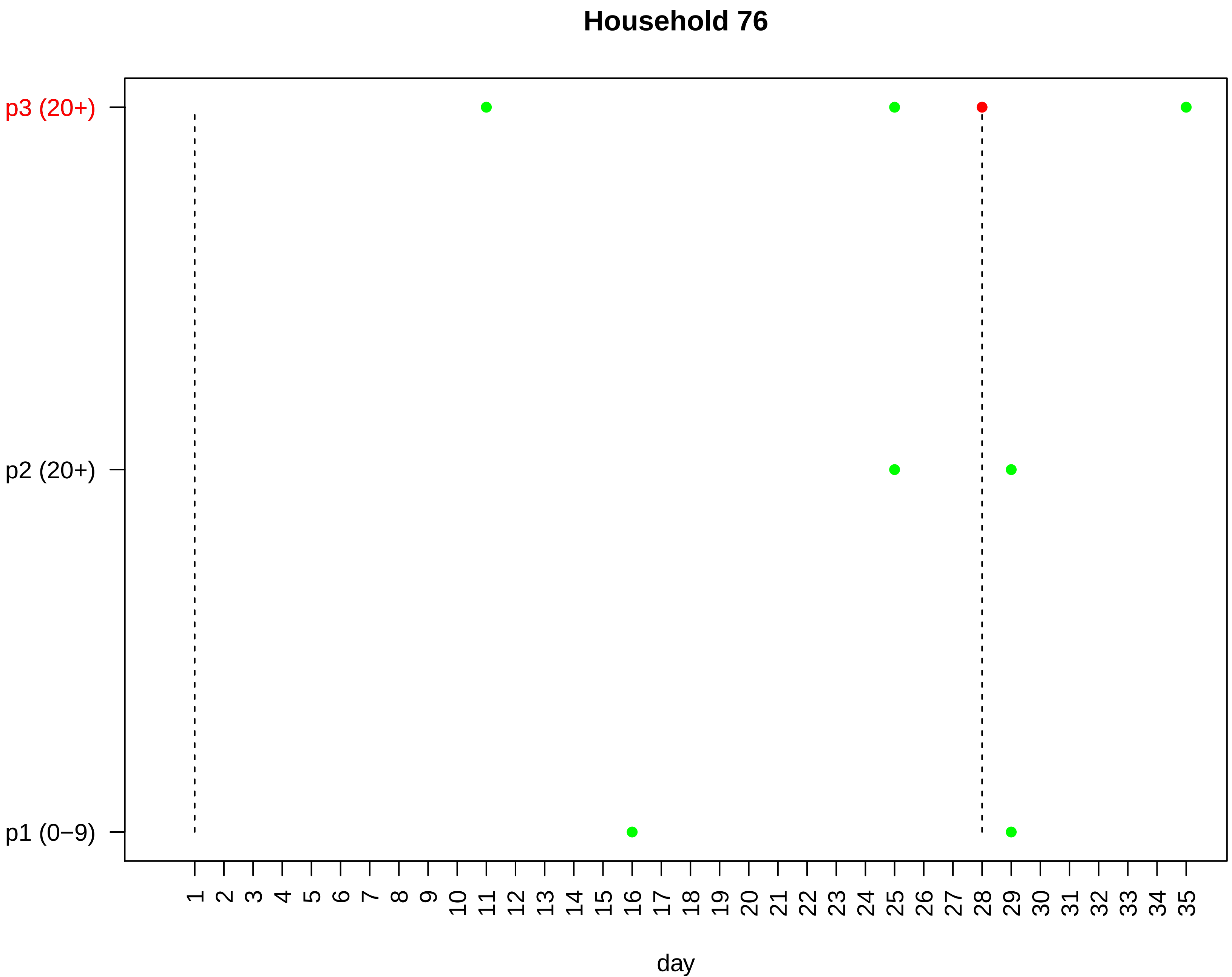

Household 76
p3 (20+)
p2 (20+)
p1 (0−9)
1 2 3 4 5 6 7 8 9 10 11 12 13 14 15 16 17 18 19 20 21 22 23 24 25 26 27 28 29 30 31 32 33 34 35
day

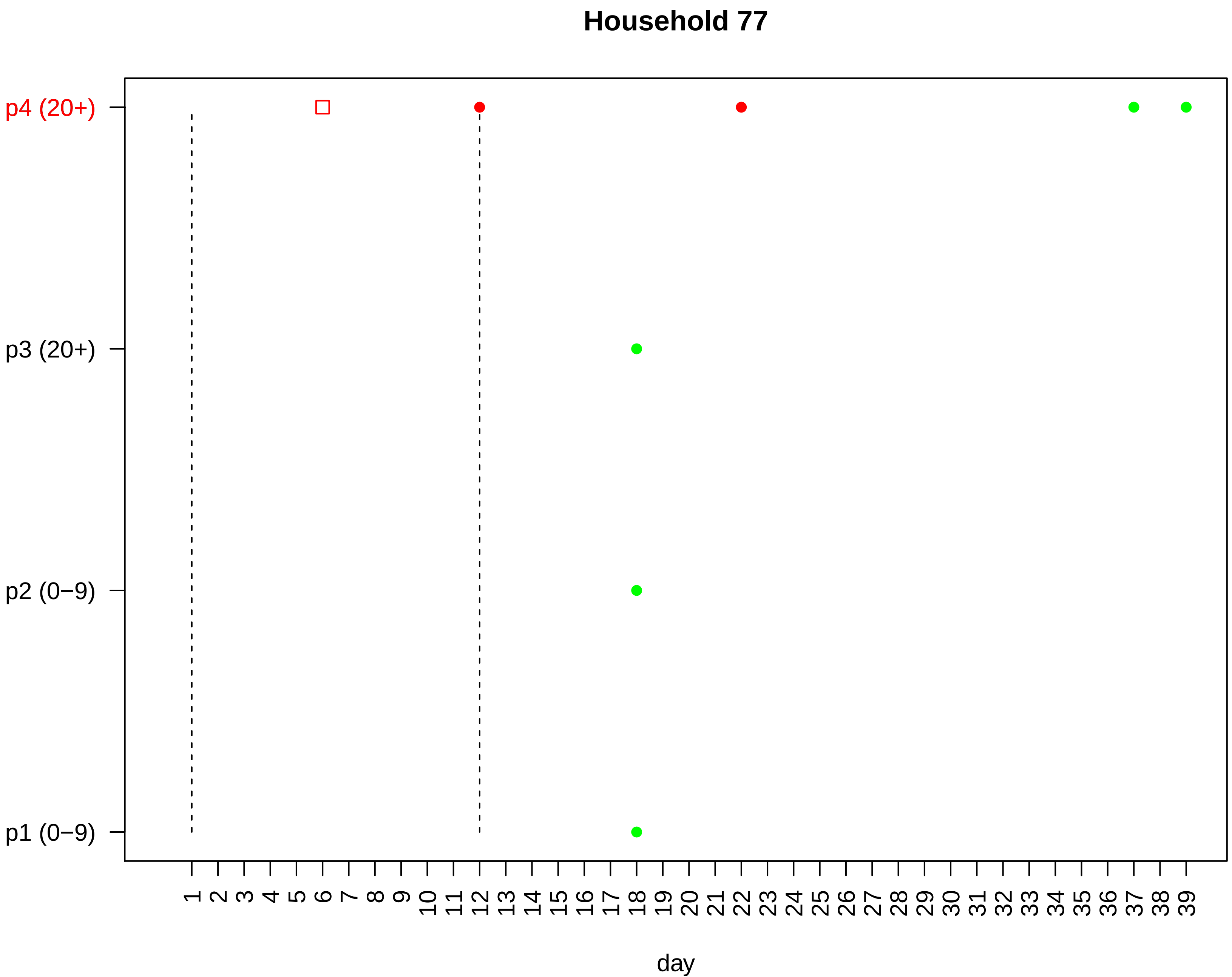

Household 77
p4 (20+)
p3 (20+)
p2 (0−9)
p1 (0−9)
1 2 3 4 5 6 7 8 9 10 11 12 13 14 15 16 17 18 19 20 21 22 23 24 25 26 27 28 29 30 31 32 33 34 35 36 37 38 39
day

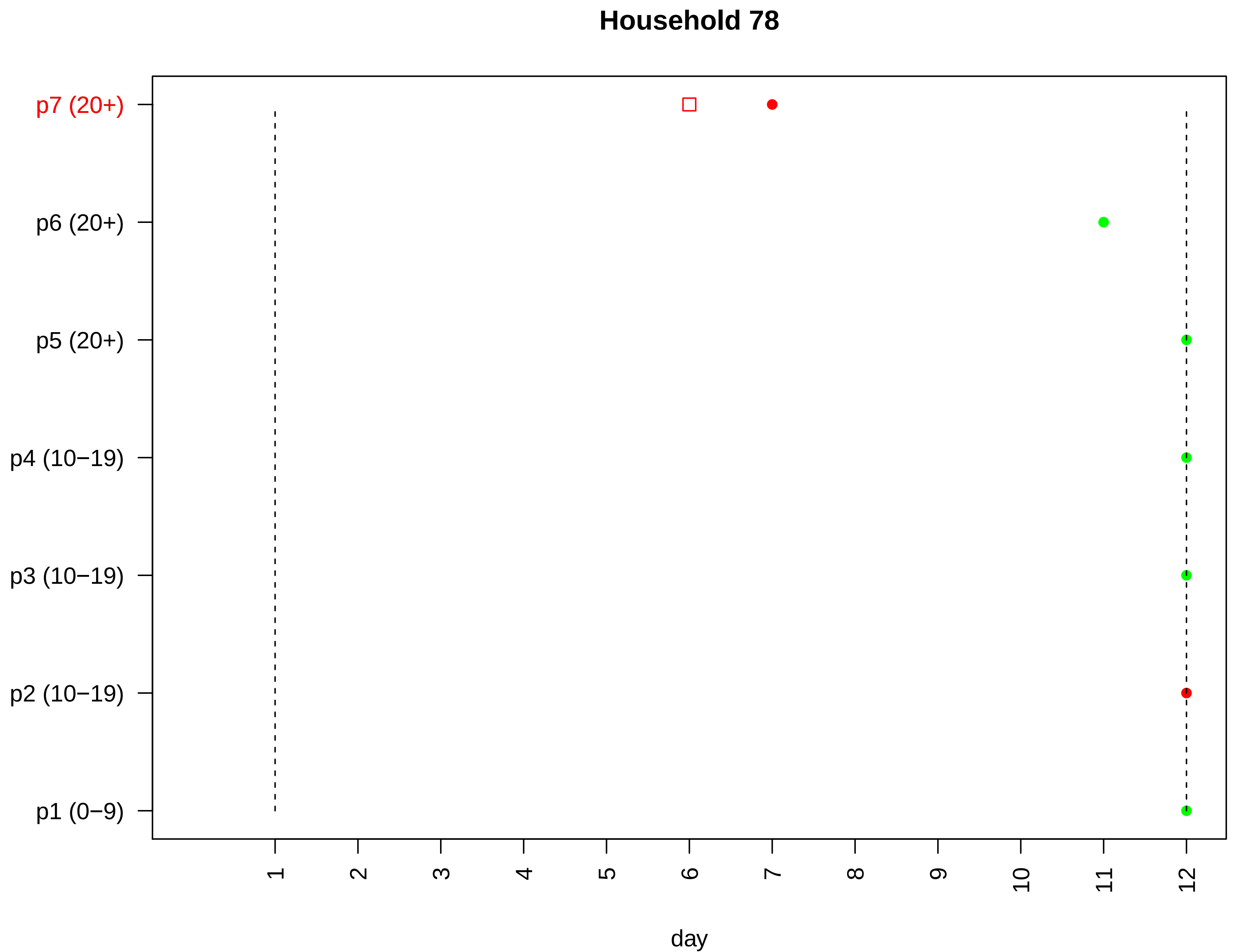

Household 78
p7 (20+)
p6 (20+)
p5 (20+)
p4 (10−19)
p3 (10−19)
p2 (10−19)
p1 (0−9)
1
2
3
4
5
6
7
8
9
10
11
12
day

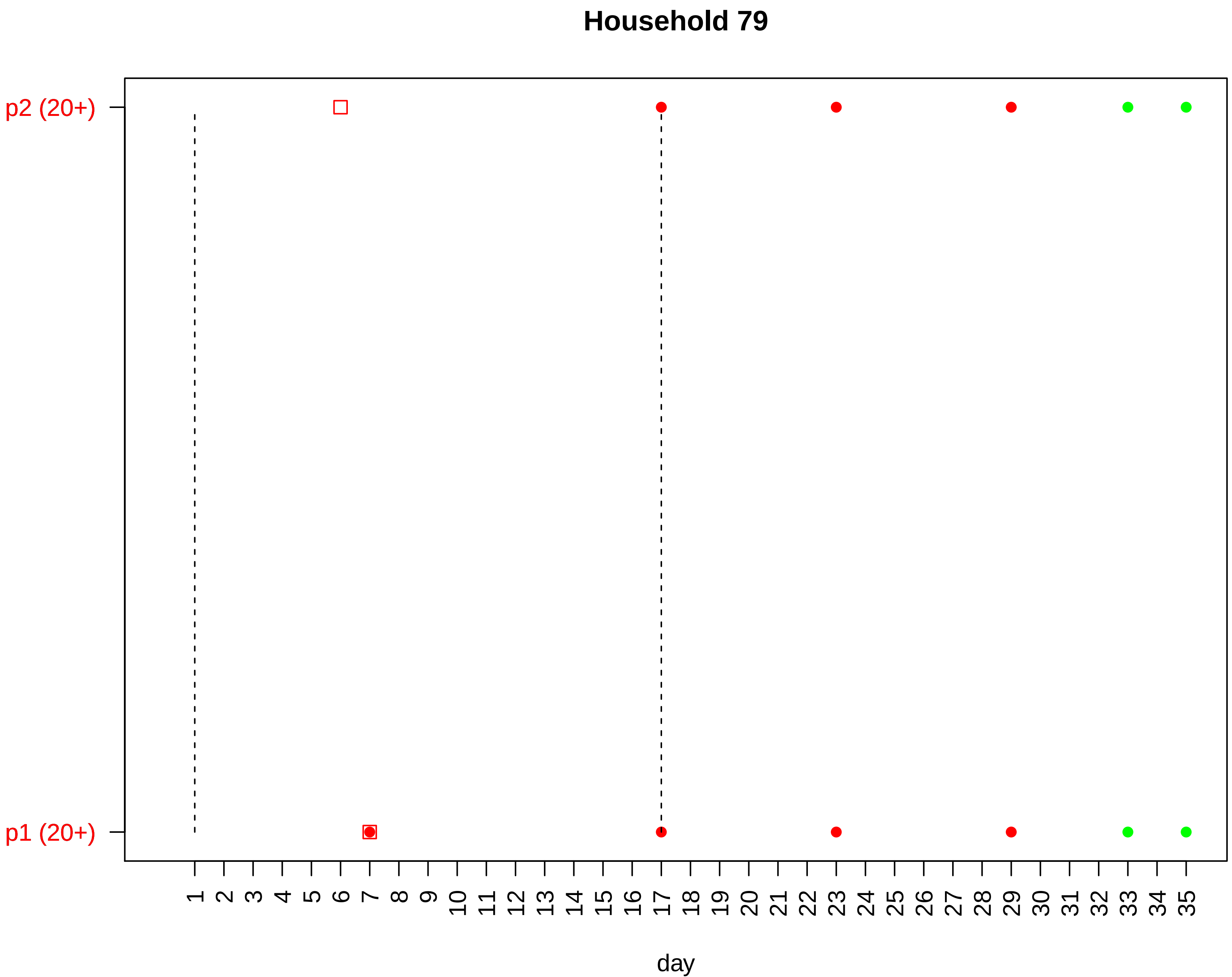
Household 79
p2 (20+)
p1 (20+)
1 2 3 4 5 6 7 8 9 10 11 12 13 14 15 16 17 18 19 20 21 22 23 24 25 26 27 28 29 30 31 32 33 34 35
day

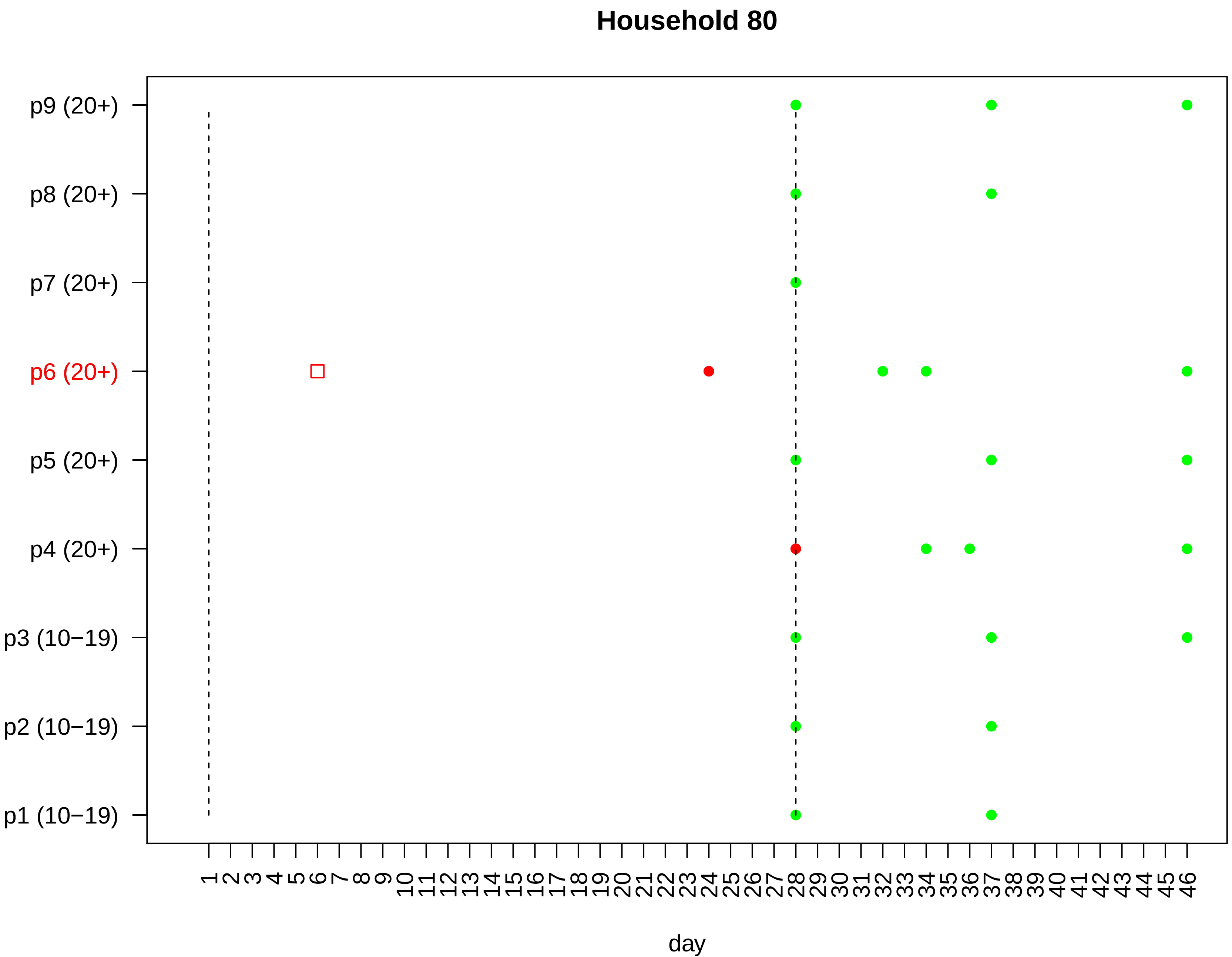

Household 80
p9 (20+)
p8 (20+)
p7 (20+)
p6 (20+)
p5 (20+)
p4 (20+)
p3 (10−19)
p2 (10−19)
p1 (10−19)
1 2 3 4 5 6 7 8 9 10 11 12 13 14 15 16 17 18 19 20 21 22 23 24 25 26 27 28 29 30 31 32 33 34 35 36 37 38 39 40 41 42 43 44 45 46
day

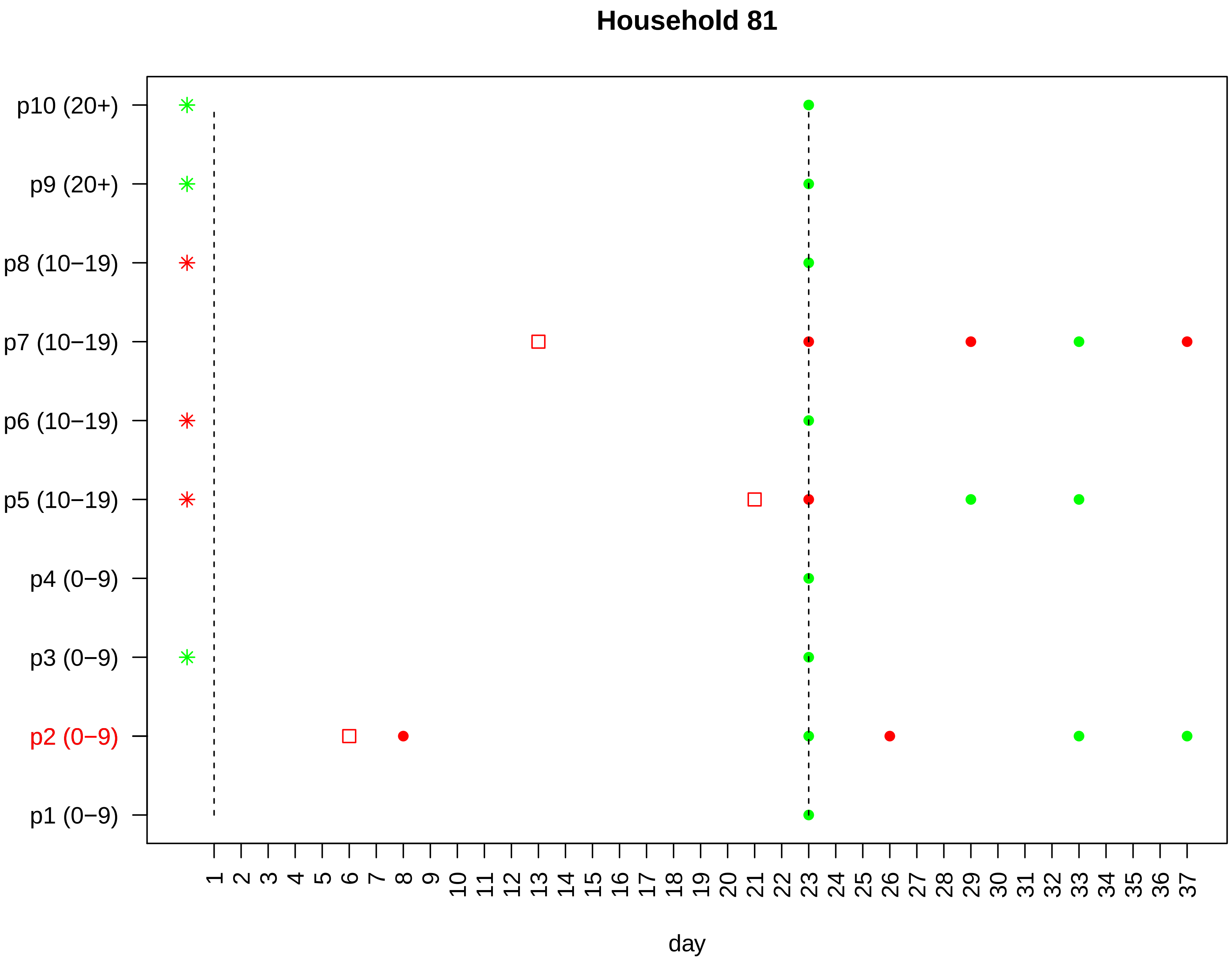

Household 81
p10 (20+)
p9 (20+)
p8 (10−19)
p7 (10−19)
p6 (10−19)
p5 (10−19)
p4 (0−9)
p3 (0−9)
p2 (0−9)
p1 (0−9)
1 2 3 4 5 6 7 8 9 10 11 12 13 14 15 16 17 18 19 20 21 22 23 24 25 26 27 28 29 30 31 32 33 34 35 36 37
day

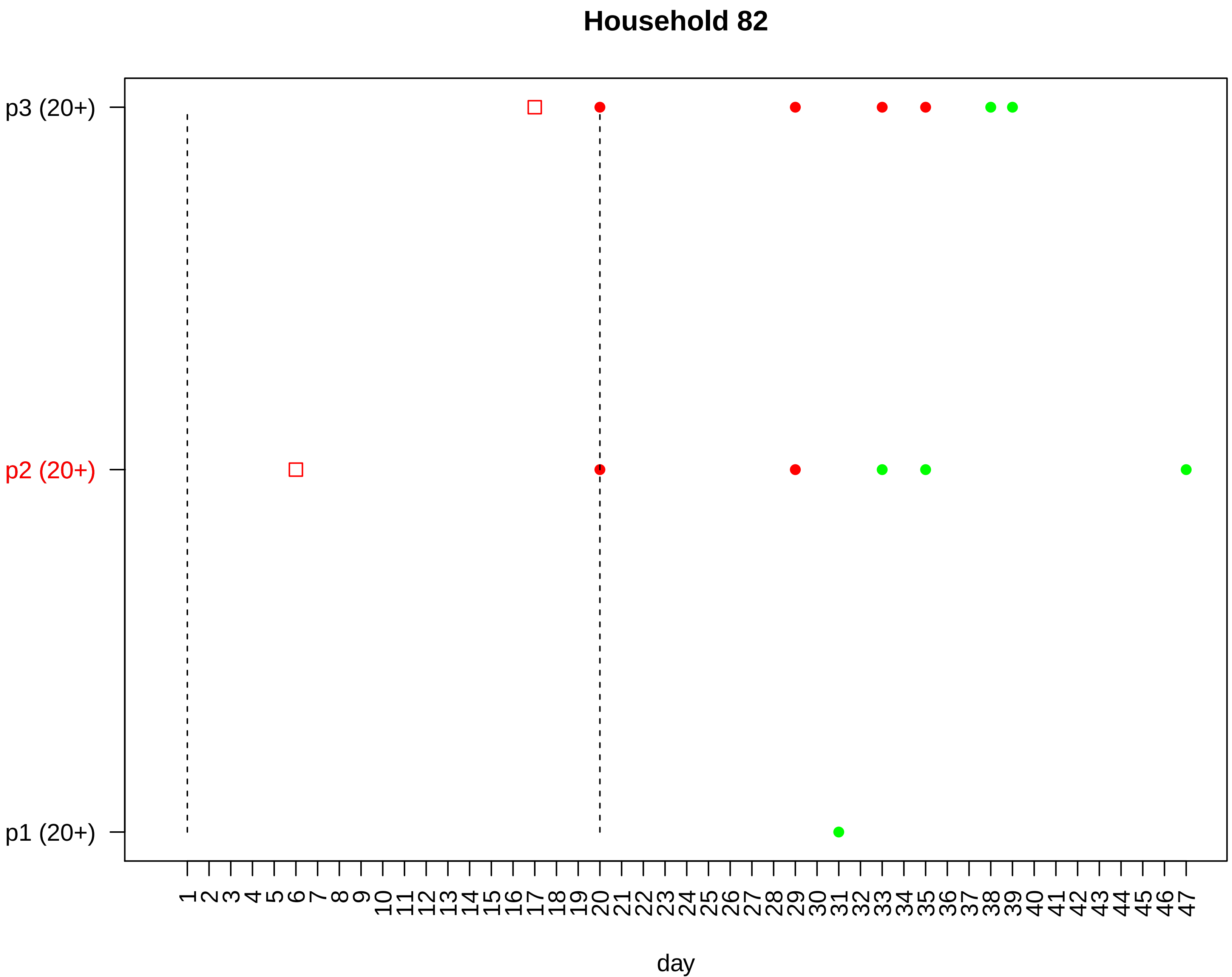

Household 82
p3 (20+)
p2 (20+)
p1 (20+)
1 2 3 4 5 6 7 8 9 10 11 12 13 14 15 16 17 18 19 20 21 22 23 24 25 26 27 28 29 30 31 32 33 34 35 36 37 38 39 40 41 42 43 44 45 46 47
day

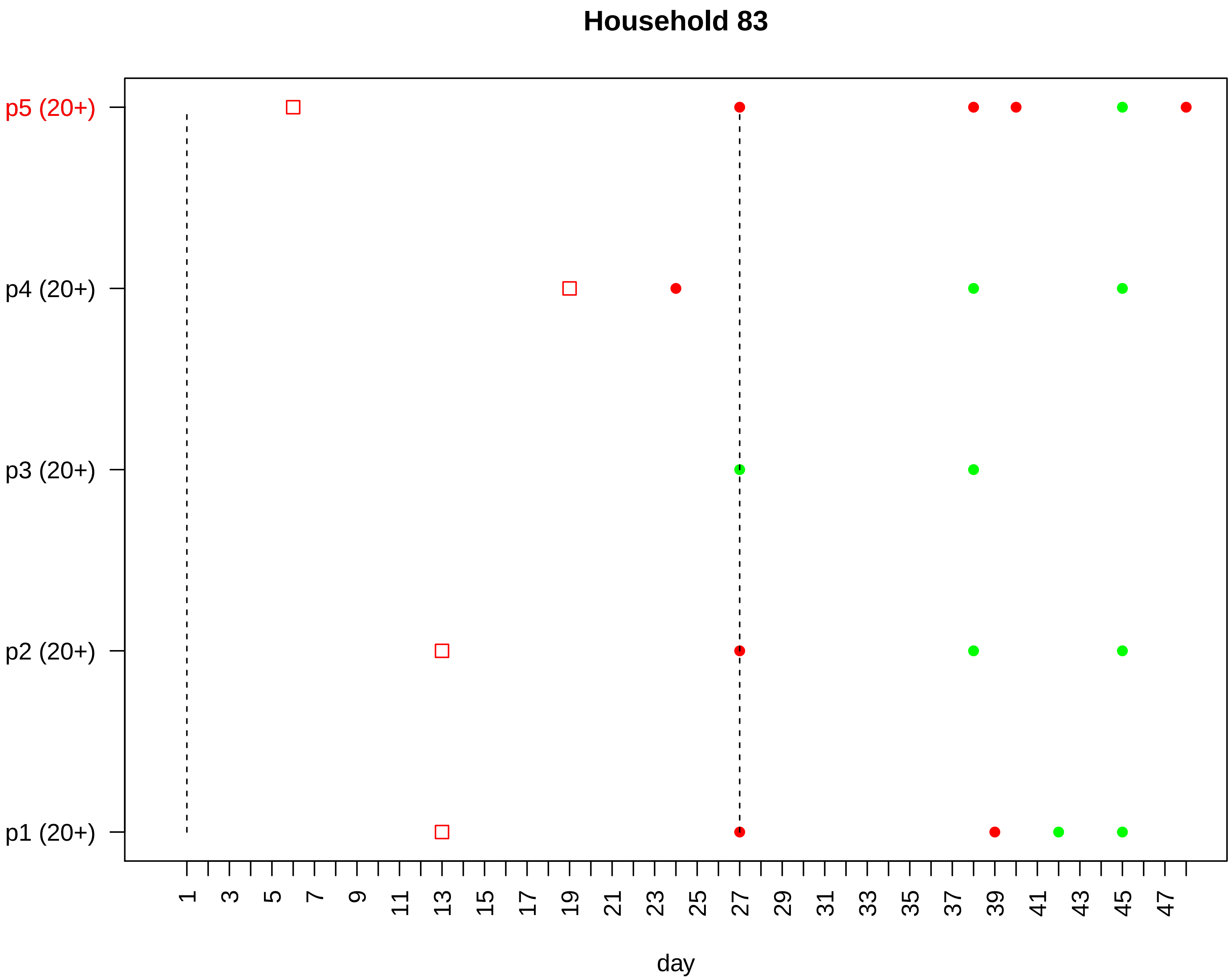
Household 83
p5 (20+)
p4 (20+)
p3 (20+)
p2 (20+)
p1 (20+)
1 3 5 7 9 11 13 15 17 19 21 23 25 27 29 31 33 35 37 39 41 43 45 47
day

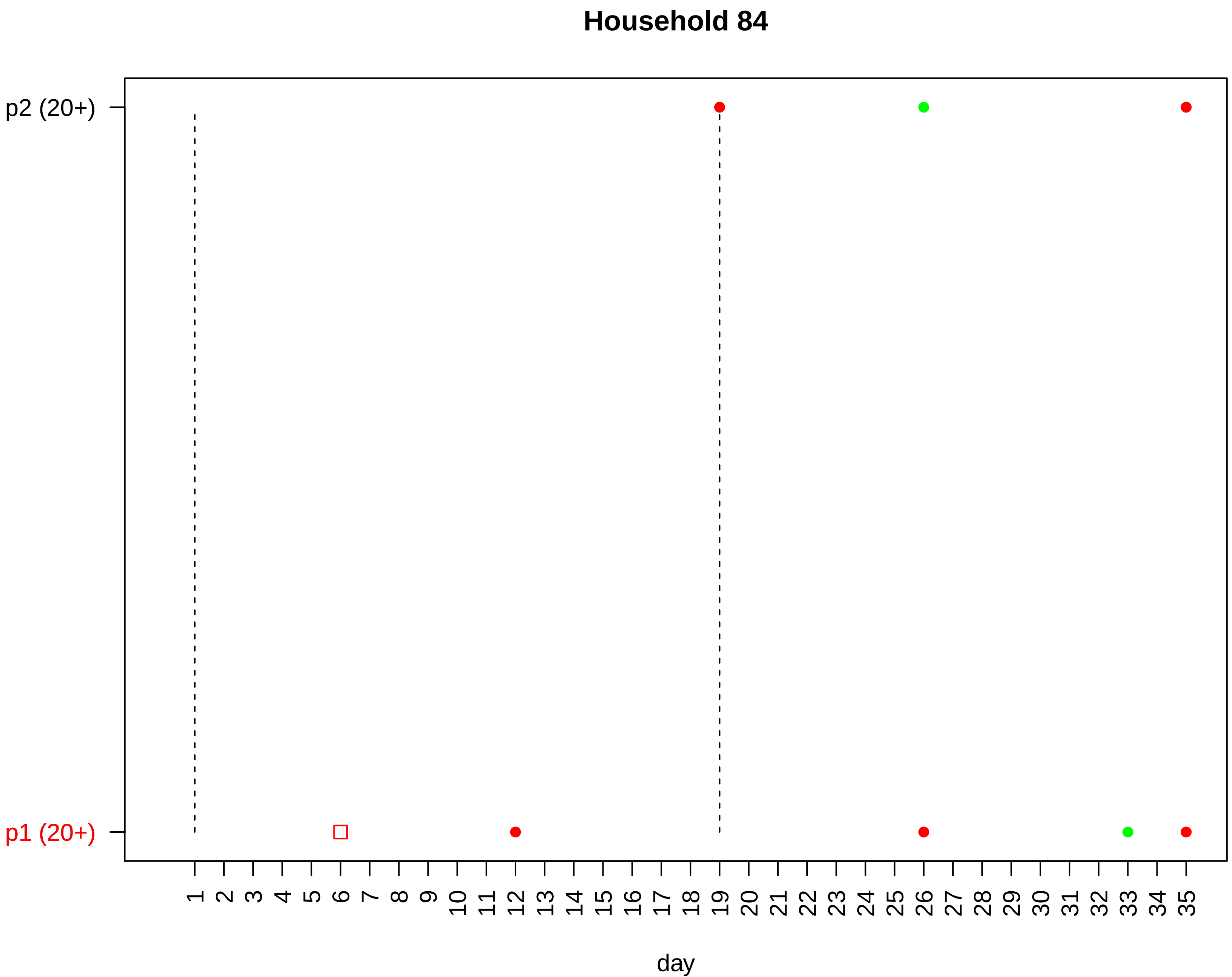
Household 84
p2 (20+)
p1 (20+)
1 2 3 4 5 6 7 8 9 10 11 12 13 14 15 16 17 18 19 20 21 22 23 24 25 26 27 28 29 30 31 32 33 34 35
day

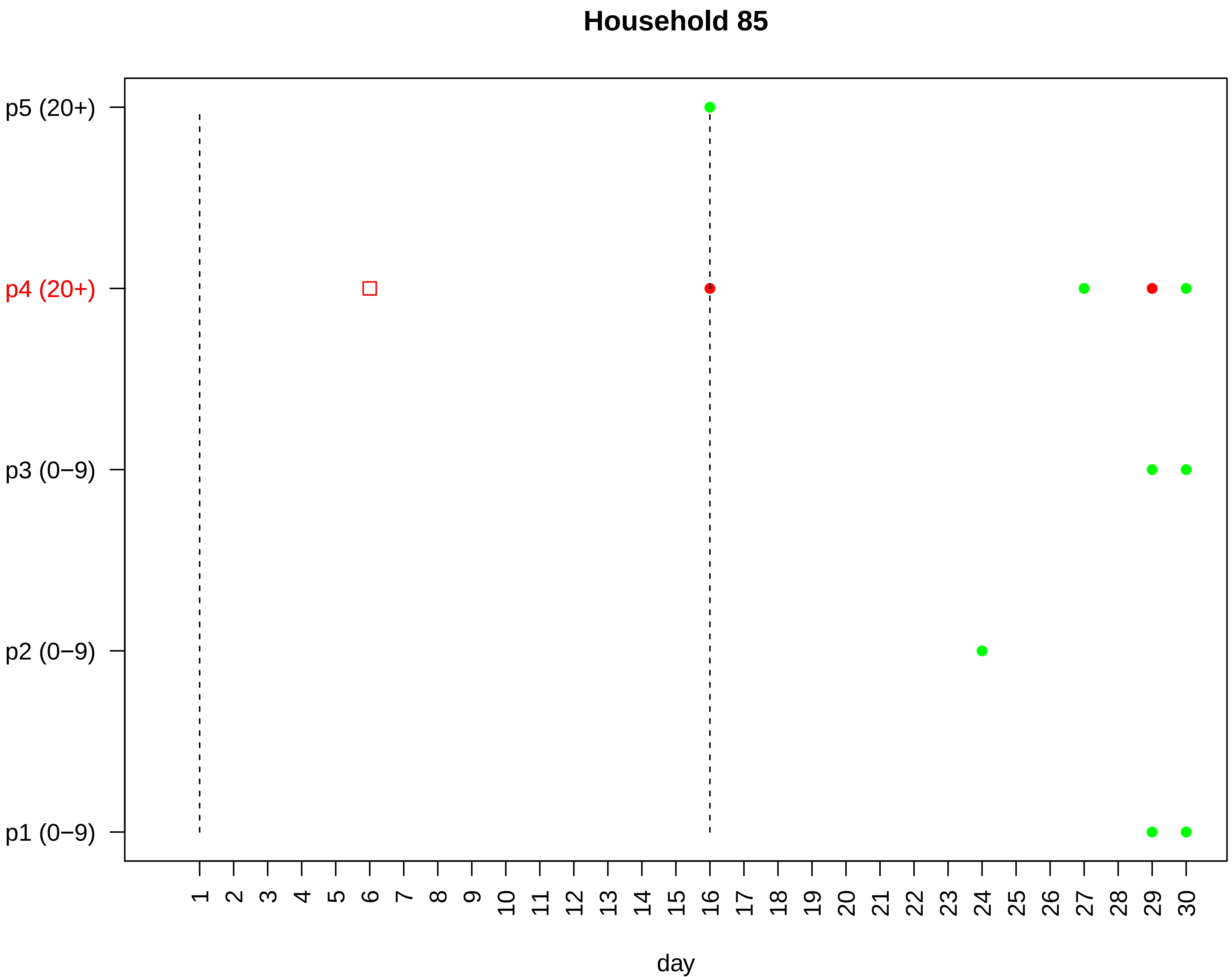
Household 85
p5 (20+)
p4 (20+)
p3 (0−9)
p2 (0−9)
p1 (0−9)
1
2
3
4
5
6
7
8
9
10
11
12
13
14
15
16
17
18
19
20
21
22
23
24
25
26
27
28
29
30
day

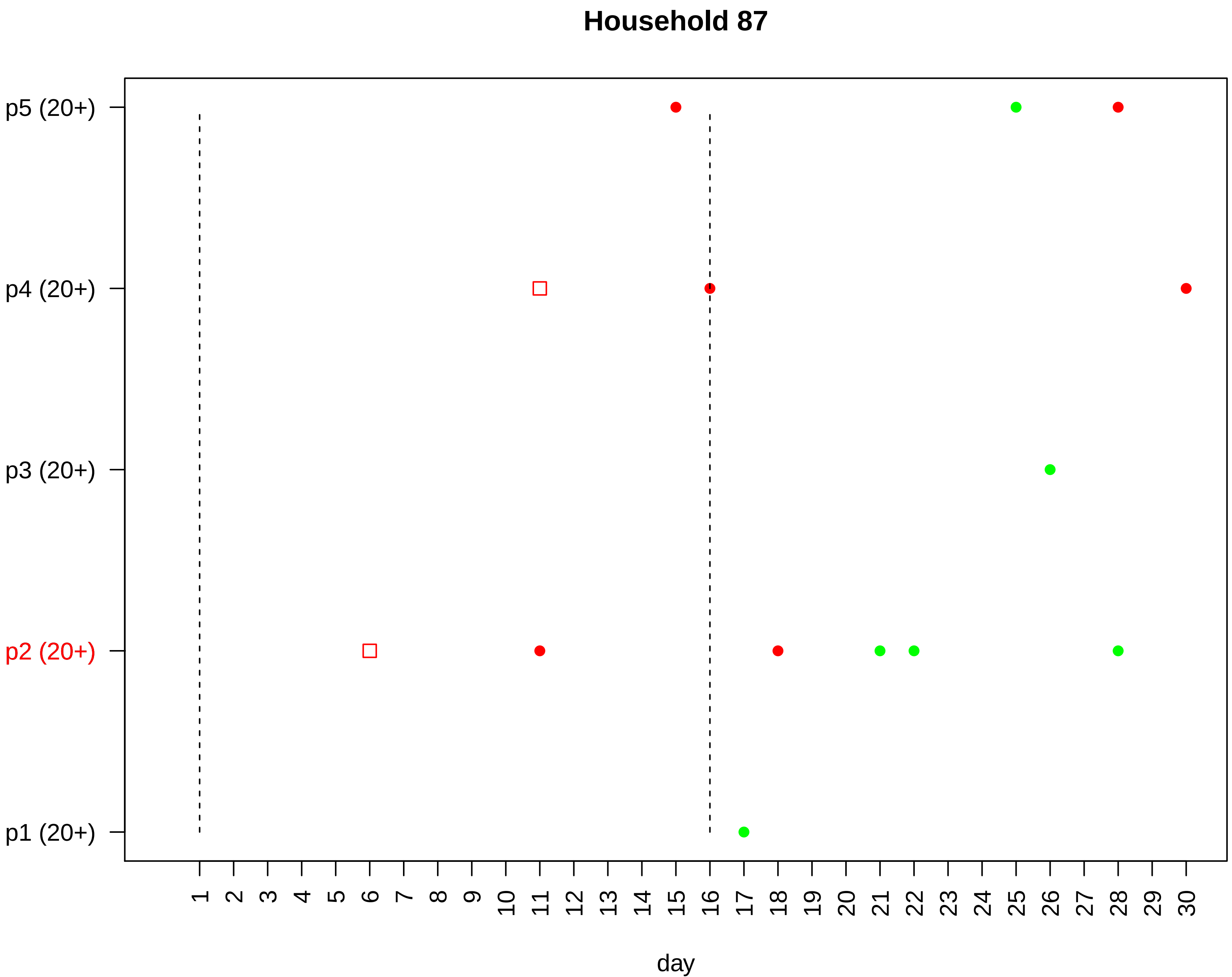

Household 87
p5 (20+)
p4 (20+)
p3 (20+)
p2 (20+)
p1 (20+)
1
2
3
4
5
6
7
8
9
10
11
12
13
14
15
16
17
18
19
20
21
22
23
24
25
26
27
28
29
30
day

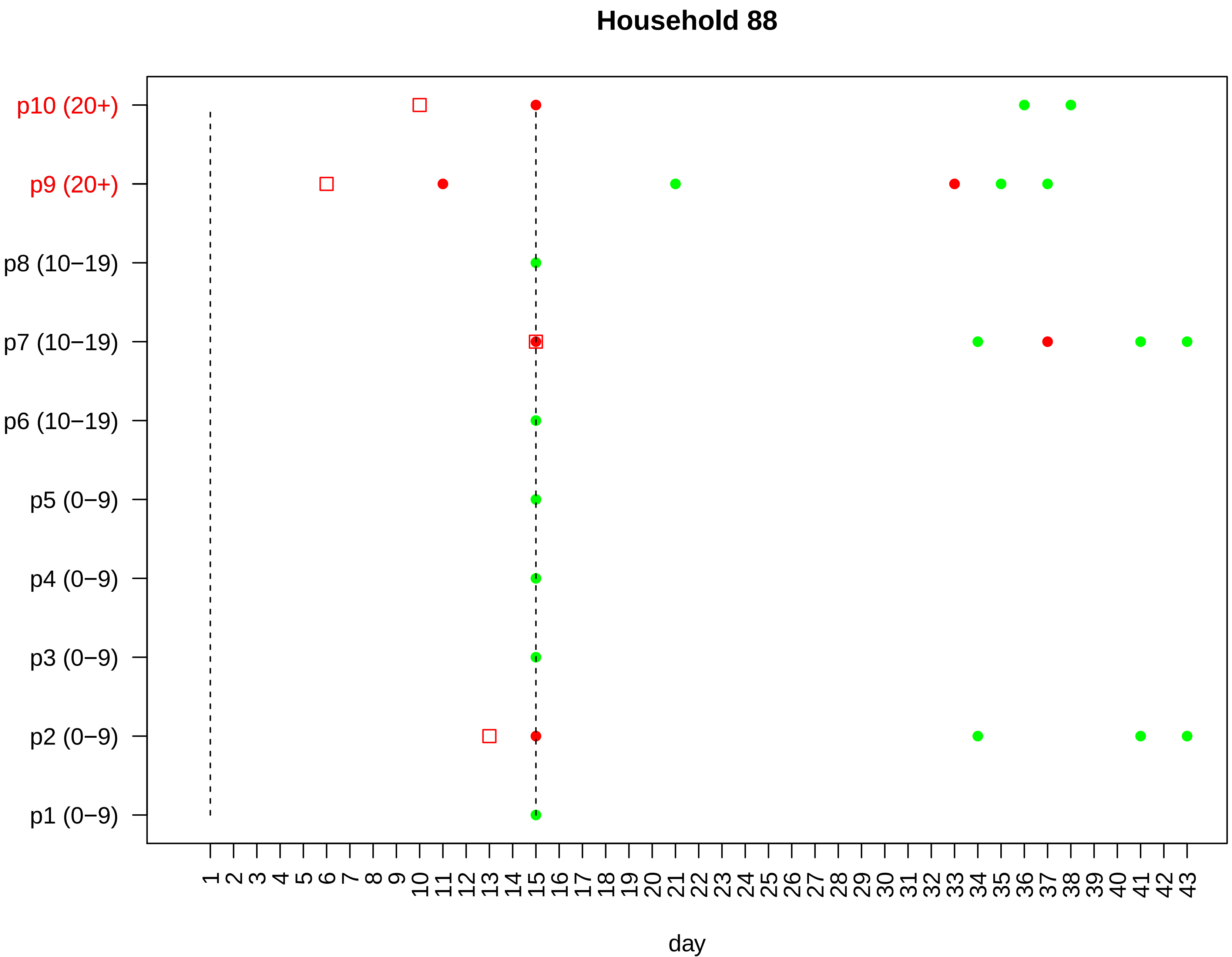

Household 88
p10 (20+)
p9 (20+)
p8 (10−19)
p7 (10−19)
p6 (10−19)
p5 (0−9)
p4 (0−9)
p3 (0−9)
p2 (0−9)
p1 (0−9)
1 2 3 4 5 6 7 8 9 10 11 12 13 14 15 16 17 18 19 20 21 22 23 24 25 26 27 28 29 30 31 32 33 34 35 36 37 38 39 40 41 42 43
day

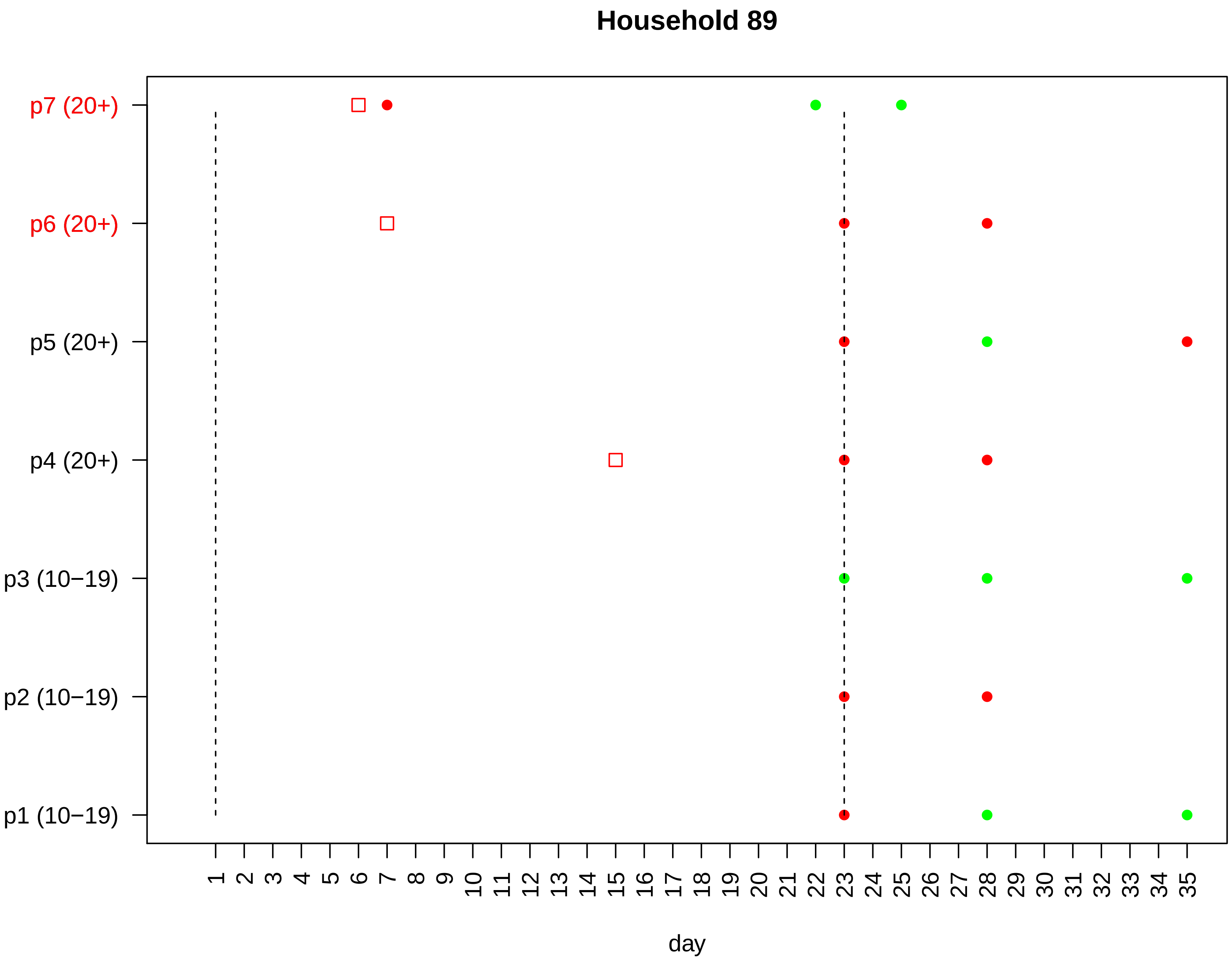

Household 89
p7 (20+)
p6 (20+)
p5 (20+)
p4 (20+)
p3 (10−19)
p2 (10−19)
p1 (10−19)
1 2 3 4 5 6 7 8 9 10 11 12 13 14 15 16 17 18 19 20 21 22 23 24 25 26 27 28 29 30 31 32 33 34 35
day

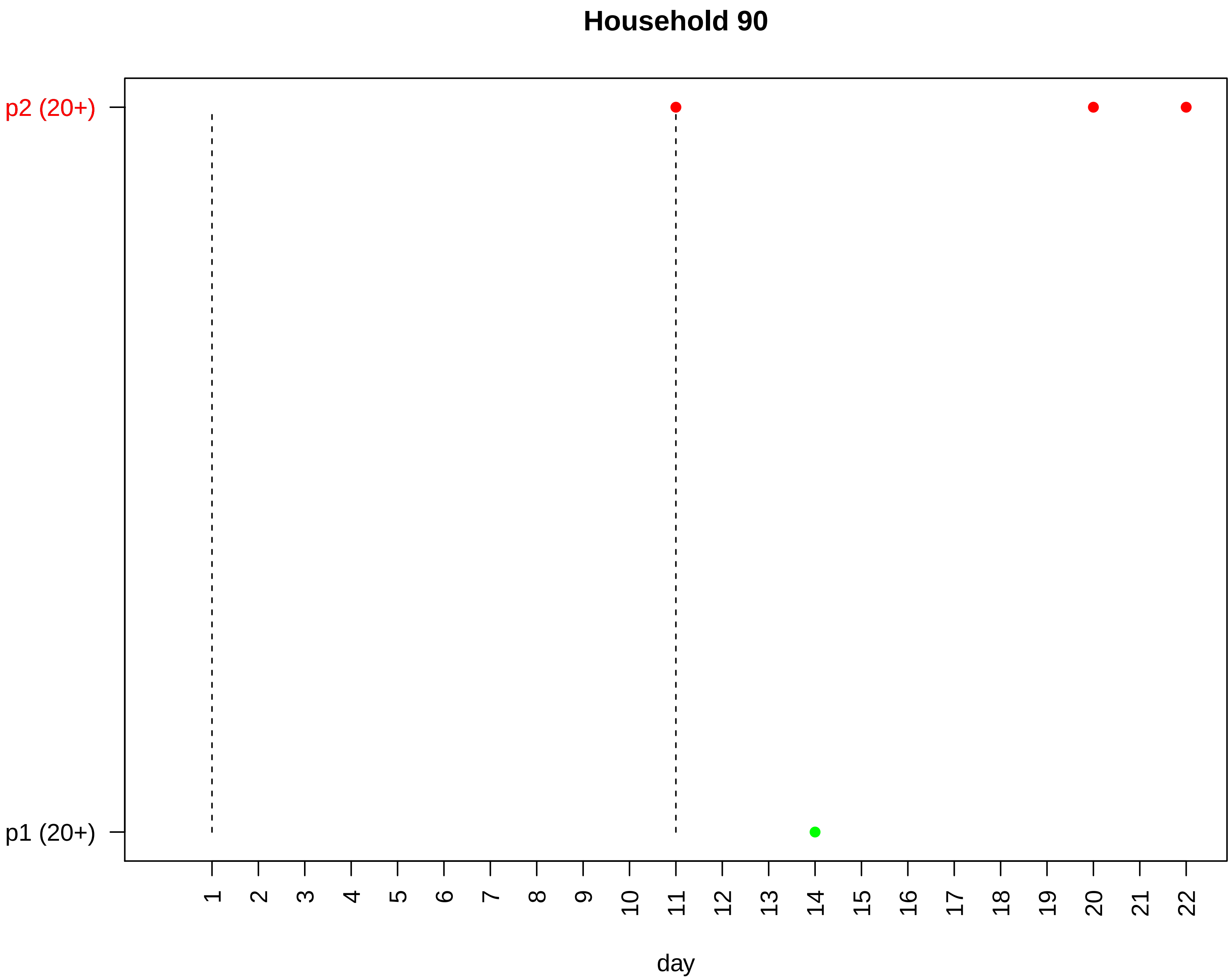

Household 90
p2 (20+)
p1 (20+)
1
2
3
4
5
6
7
8
9
10
11
12
13
14
15
16
17
18
19
20
21
22
day

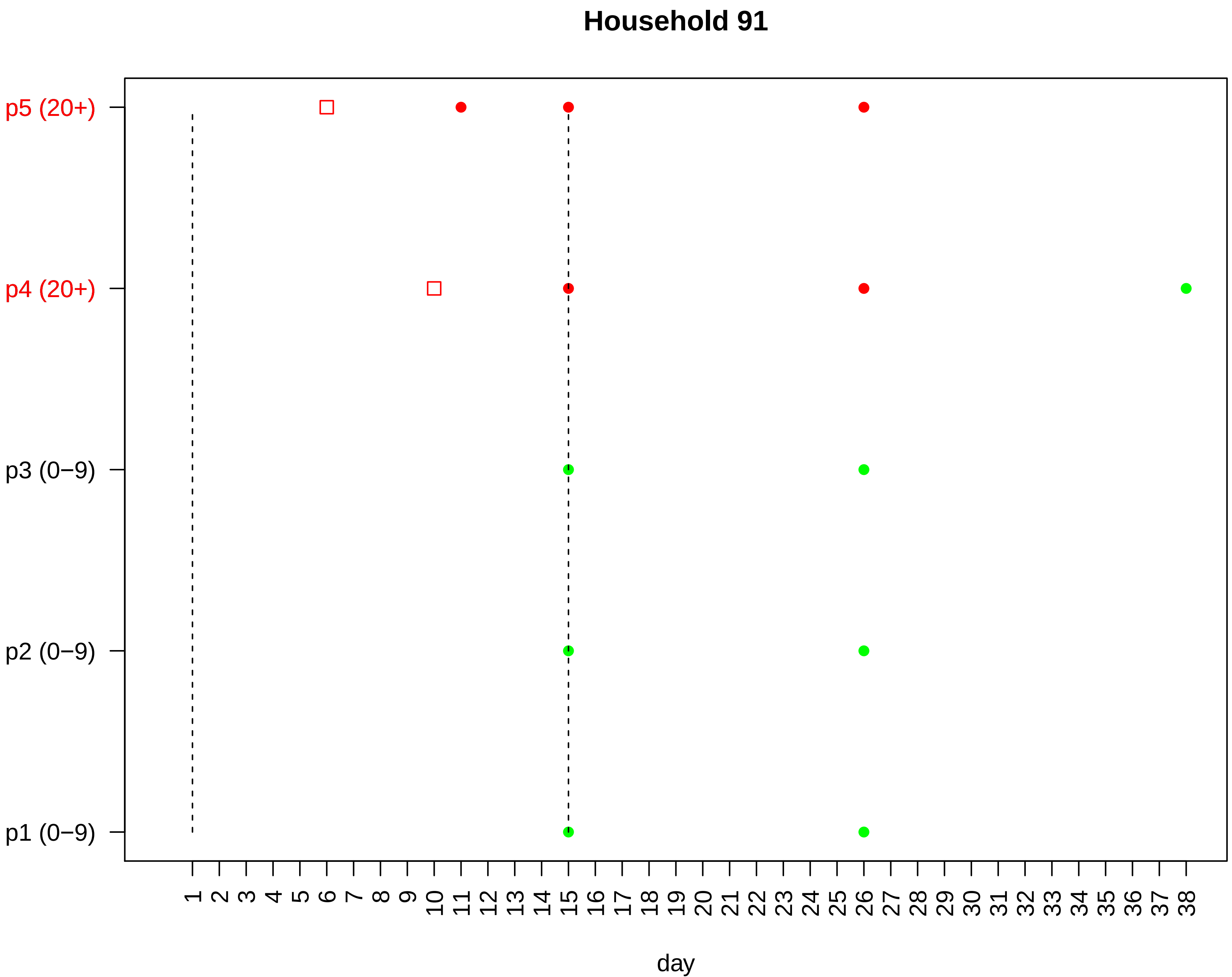
Household 91
p5 (20+)
p4 (20+)
p3 (0−9)
p2 (0−9)
p1 (0−9)
1 2 3 4 5 6 7 8 9 10 11 12 13 14 15 16 17 18 19 20 21 22 23 24 25 26 27 28 29 30 31 32 33 34 35 36 37 38
day

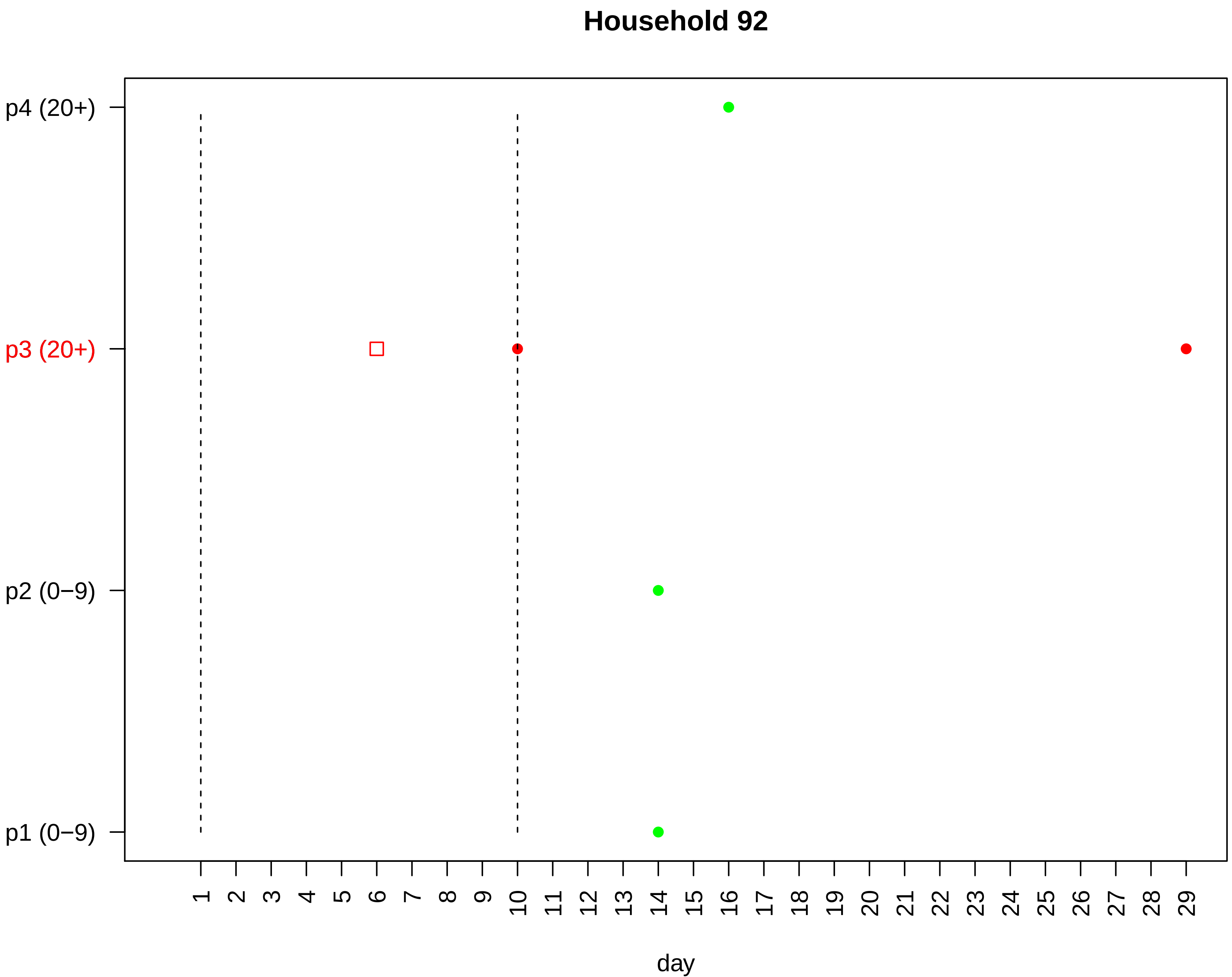

Household 92
p4 (20+)
p3 (20+)
p2 (0−9)
p1 (0−9)
1
2
3
4
5
6
7
8
9
10
11
12
13
14
15
16
17
18
19
20
21
22
23
24
25
26
27
28
29
day

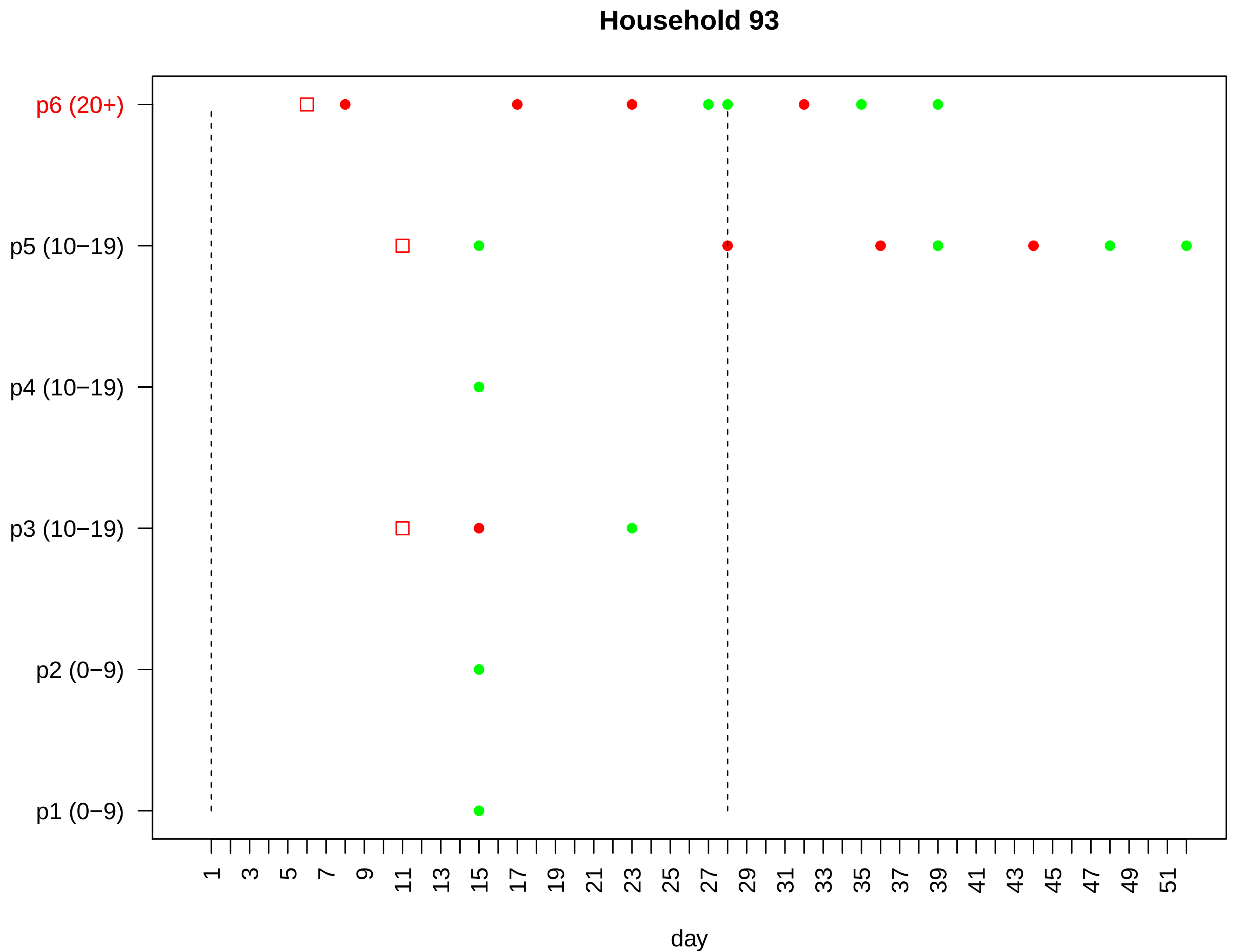

Household 93
p6 (20+)
p5 (10−19)
p4 (10−19)
p3 (10−19)
p2 (0−9)
p1 (0−9)
1 3 5 7 9 11 13 15 17 19 21 23 25 27 29 31 33 35 37 39 41 43 45 47 49 51
day

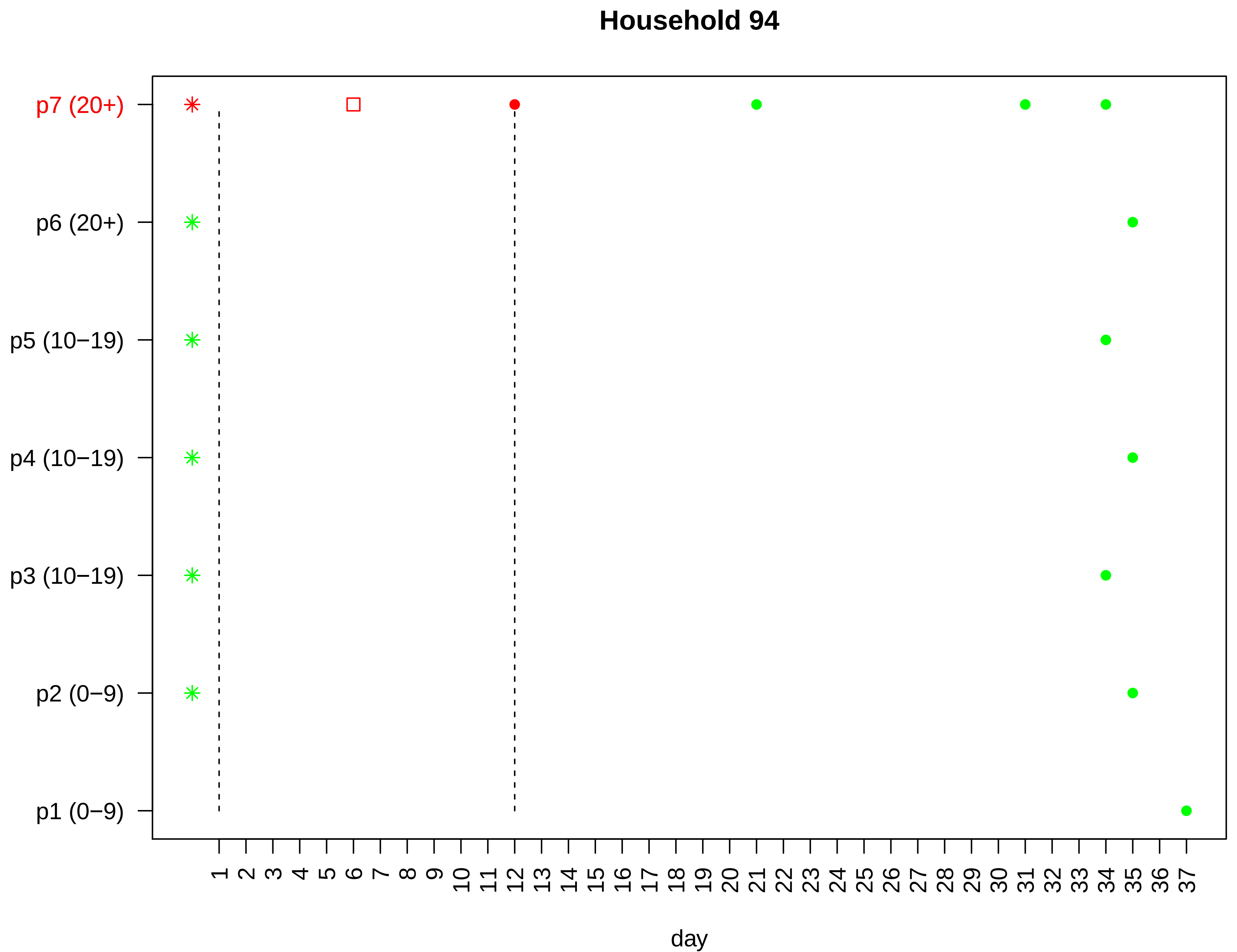

Household 94
p7 (20+)
p6 (20+)
p5 (10−19)
p4 (10−19)
p3 (10−19)
p2 (0−9)
p1 (0−9)
1 2 3 4 5 6 7 8 9 10 11 12 13 14 15 16 17 18 19 20 21 22 23 24 25 26 27 28 29 30 31 32 33 34 35 36 37
day

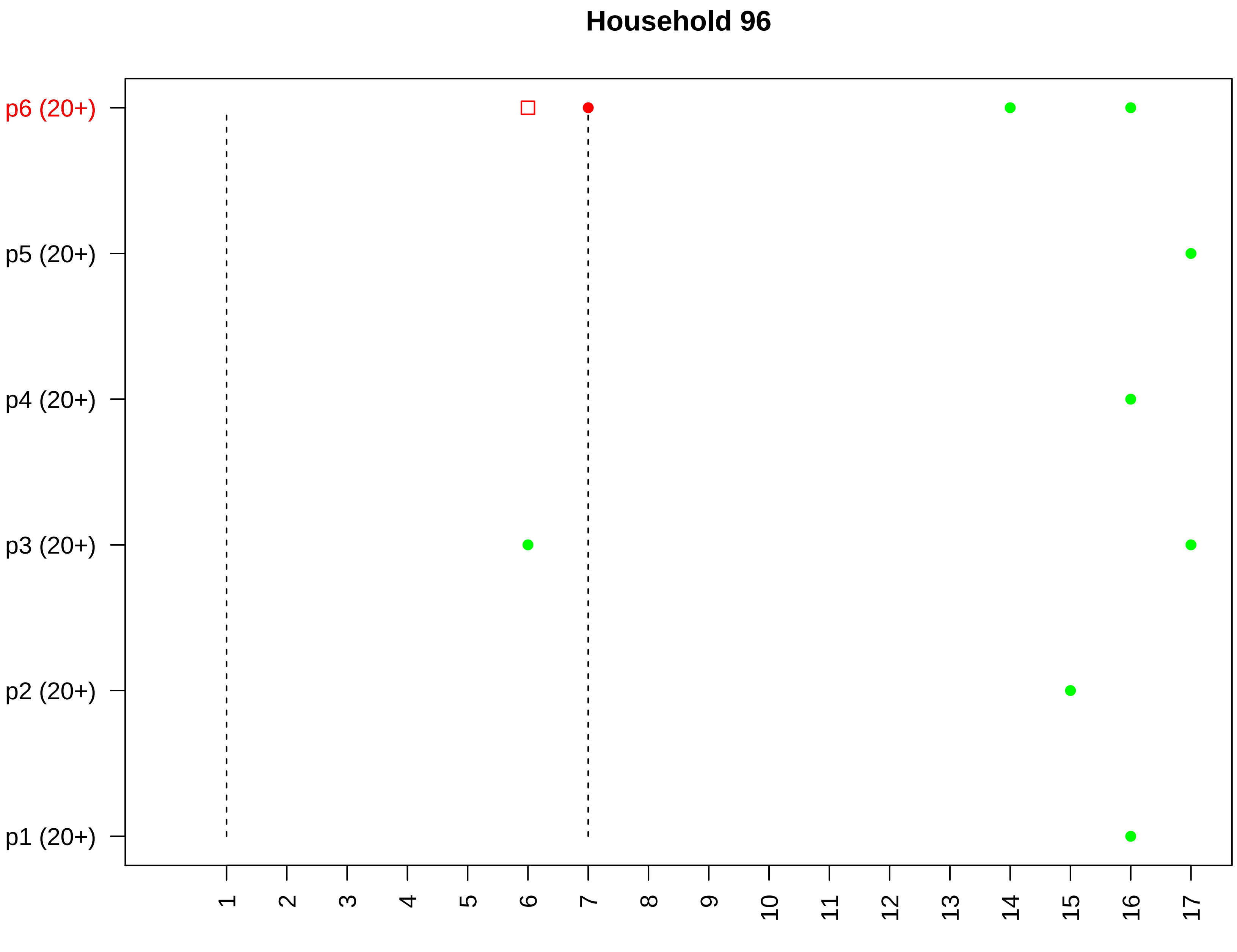
Household 96
p6 (20+)
p5 (20+)
p4 (20+)
p3 (20+)
p2 (20+)
p1 (20+)
1
2
3
4
5
6
7
8
9
10
11
12
13
14
15
16
17
day

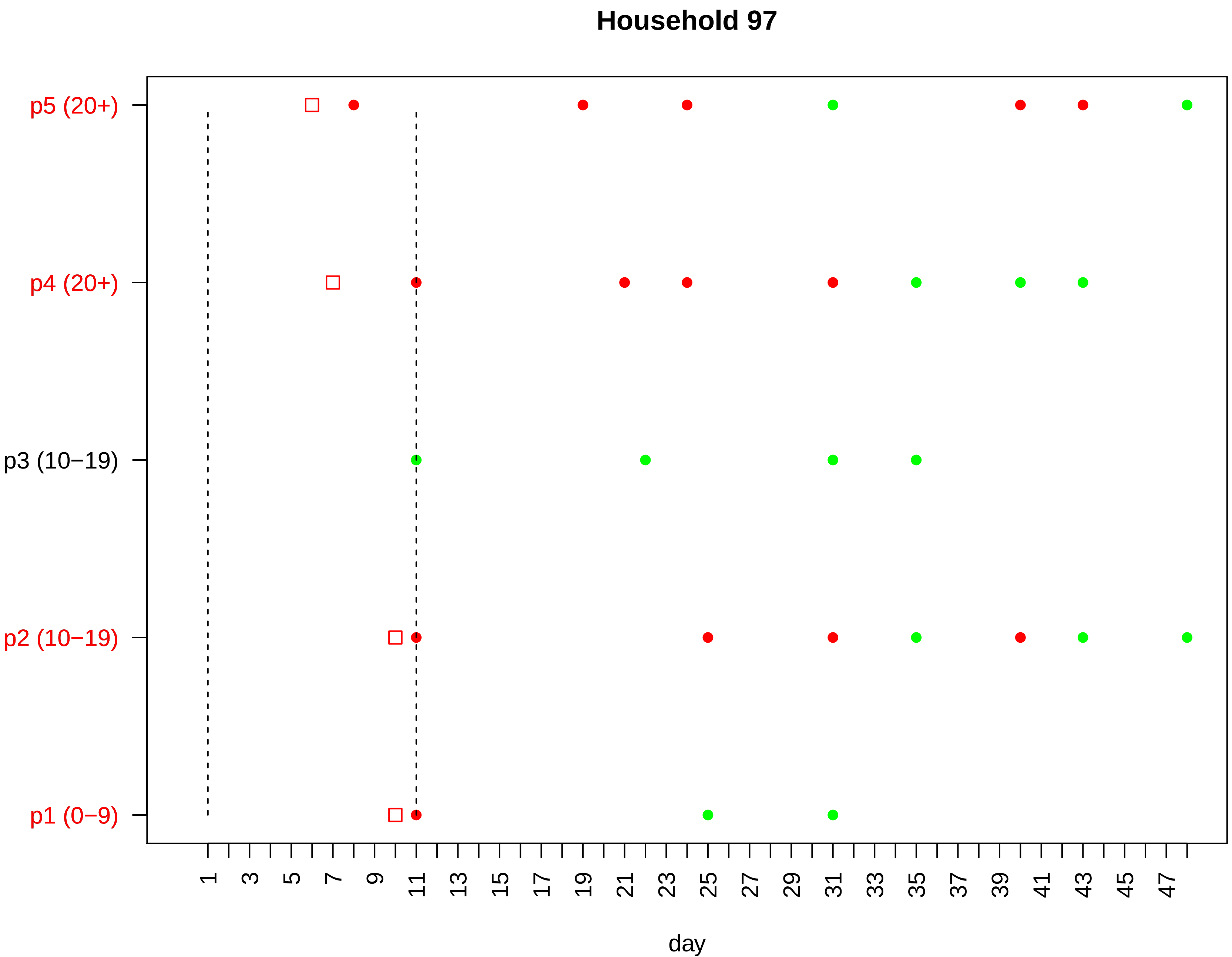
Household 97
p5 (20+)
p4 (20+)
p3 (10−19)
p2 (10−19)
p1 (0−9)
1
3
5
7
9
11
13
15
17
19
21
23
25
27
29
31
33
35
37
39
41
43
45
47
day

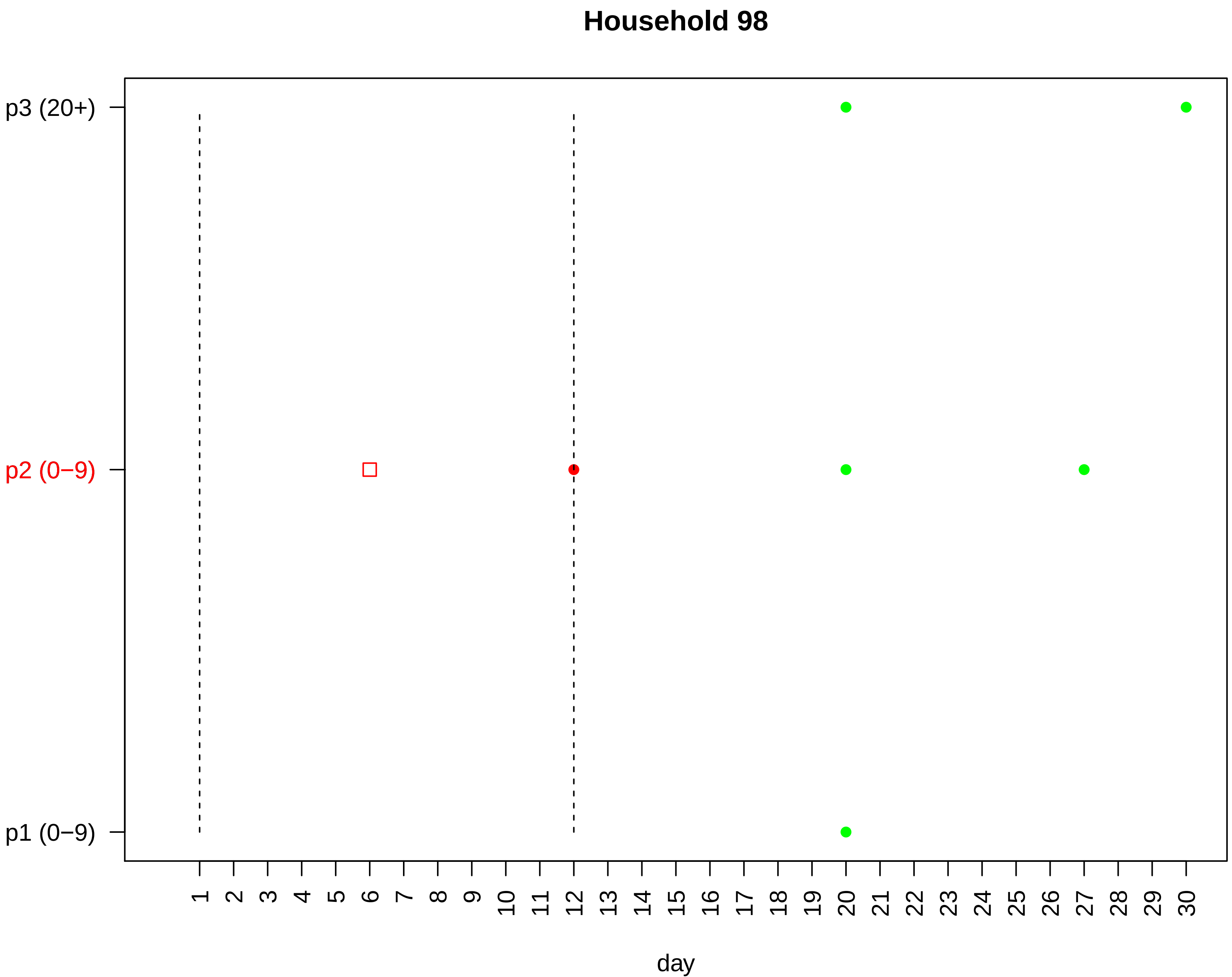

Household 98
p3 (20+)
p2 (0−9)
p1 (0−9)
1 2 3 4 5 6 7 8 9 10 11 12 13 14 15 16 17 18 19 20 21 22 23 24 25 26 27 28 29 30
day

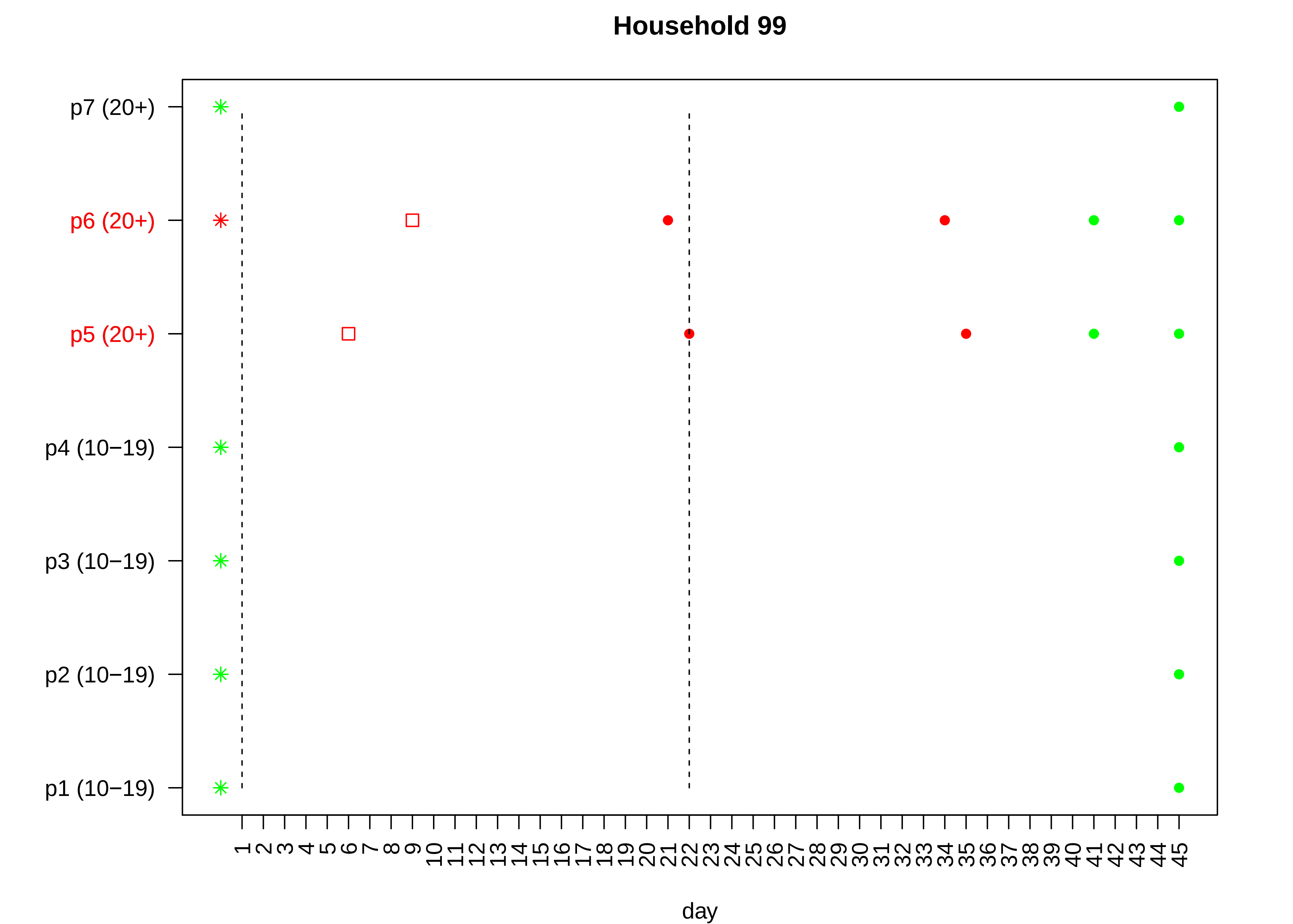

Household 99
p7 (20+)
p6 (20+)
p5 (20+)
p4 (10−19)
p3 (10−19)
p2 (10−19)
p1 (10−19)
1 2 3 4 5 6 7 8 9 10 11 12 13 14 15 16 17 18 19 20 21 22 23 24 25 26 27 28 29 30 31 32 33 34 35 36 37 38 39 40 41 42 43 44 45
day

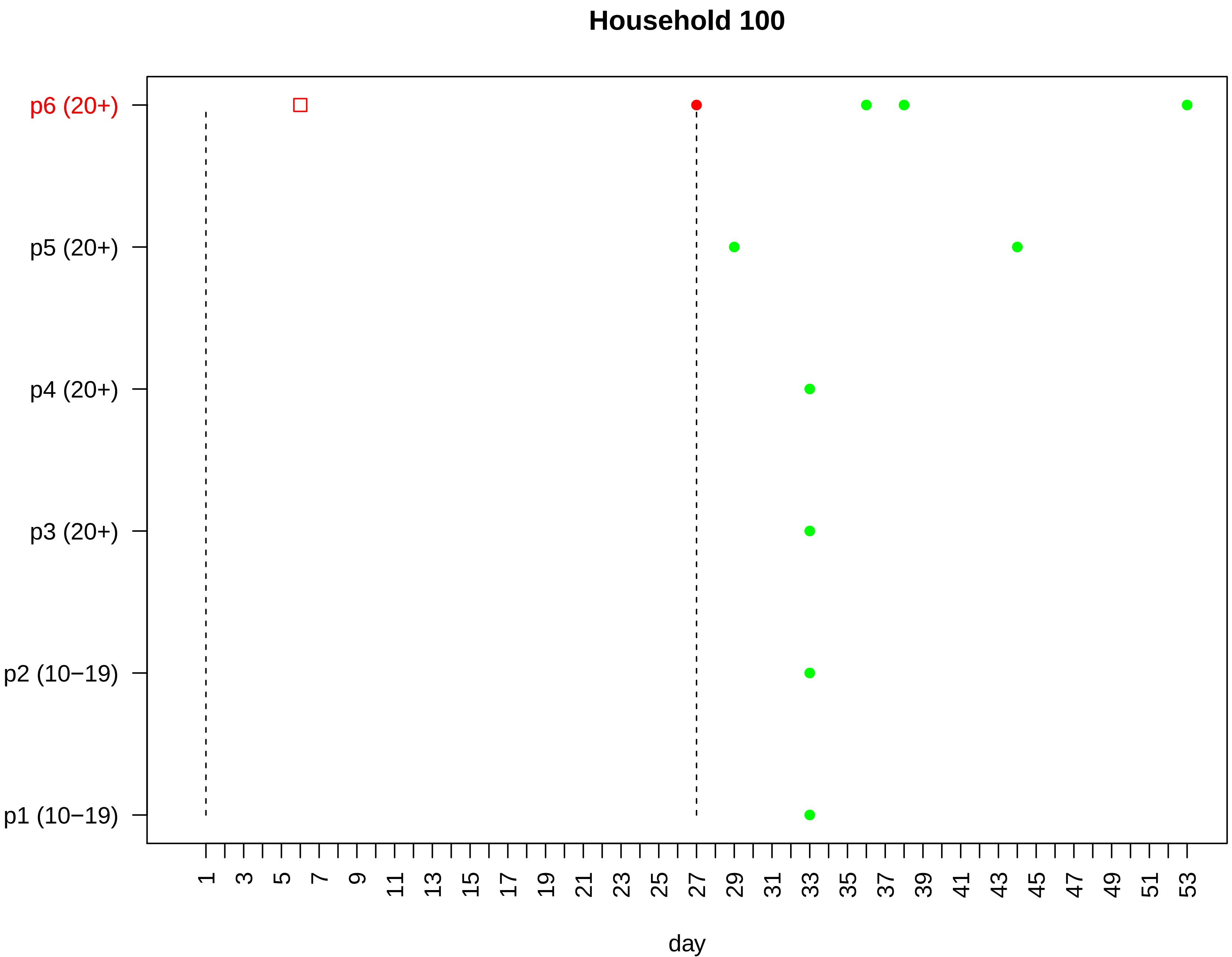

Household 100
p6 (20+)
p5 (20+)
p4 (20+)
p3 (20+)
p2 (10−19)
p1 (10−19)
1 3 5 7 9 11 13 15 17 19 21 23 25 27 29 31 33 35 37 39 41 43 45 47 49 51 53
day

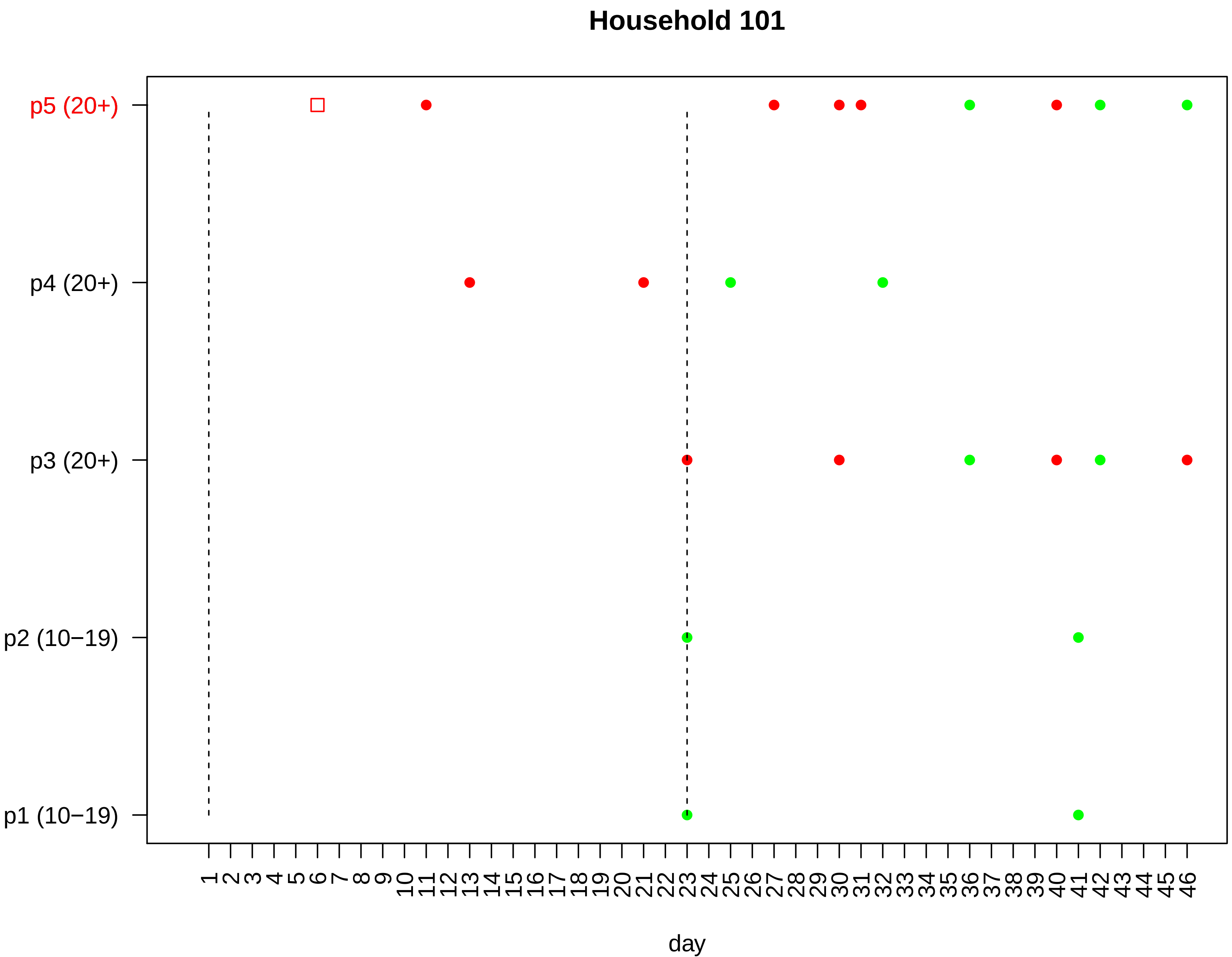

Household 101
p5 (20+)
p4 (20+)
p3 (20+)
p2 (10−19)
p1 (10−19)
1 2 3 4 5 6 7 8 9 10 11 12 13 14 15 16 17 18 19 20 21 22 23 24 25 26 27 28 29 30 31 32 33 34 35 36 37 38 39 40 41 42 43 44 45 46
day

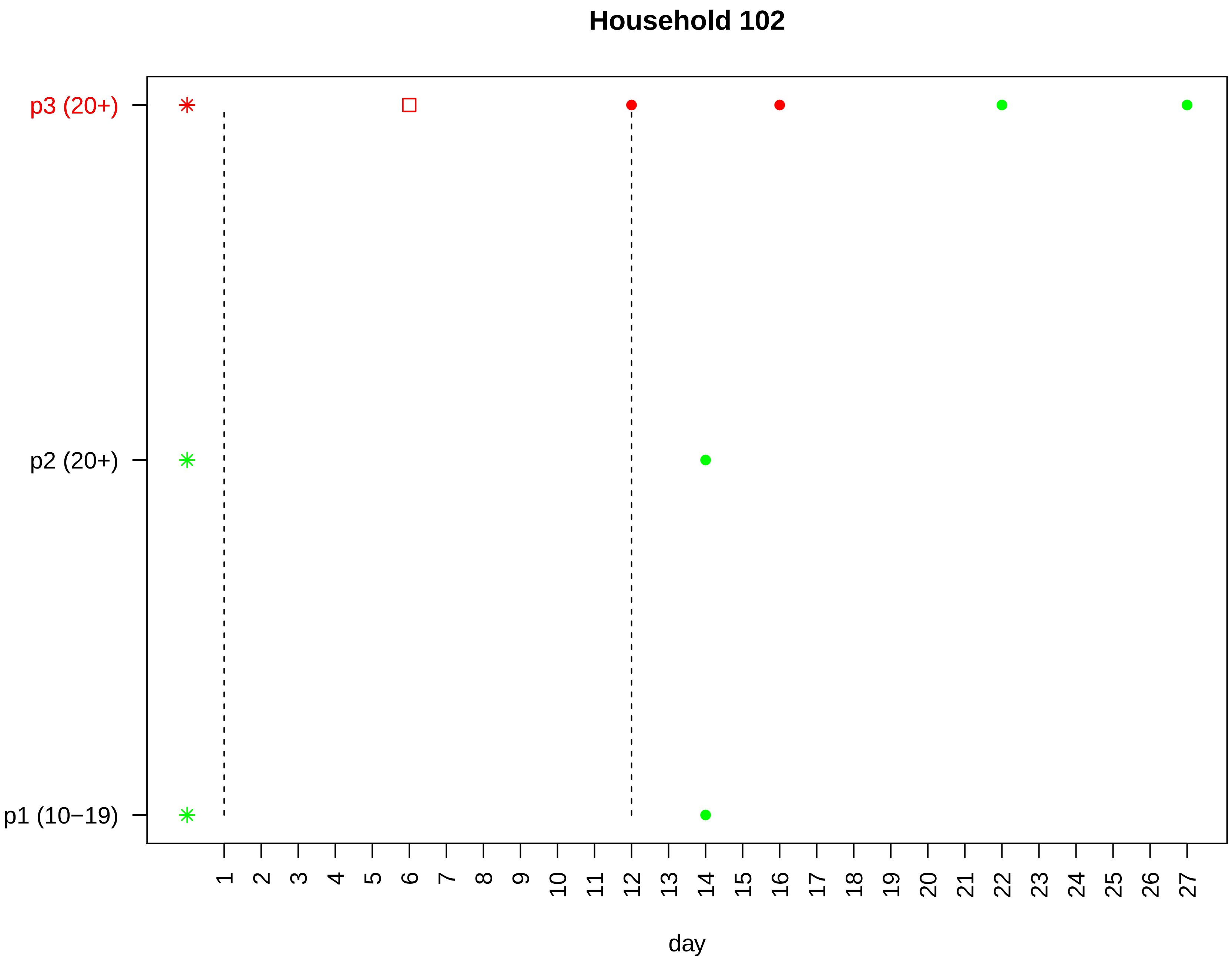

Household 102
p3 (20+)
p2 (20+)
p1 (10−19)
1
2
3
4
5
6
7
8
9
10
11
12
13
14
15
16
17
18
19
20
21
22
23
24
25
26
27
day

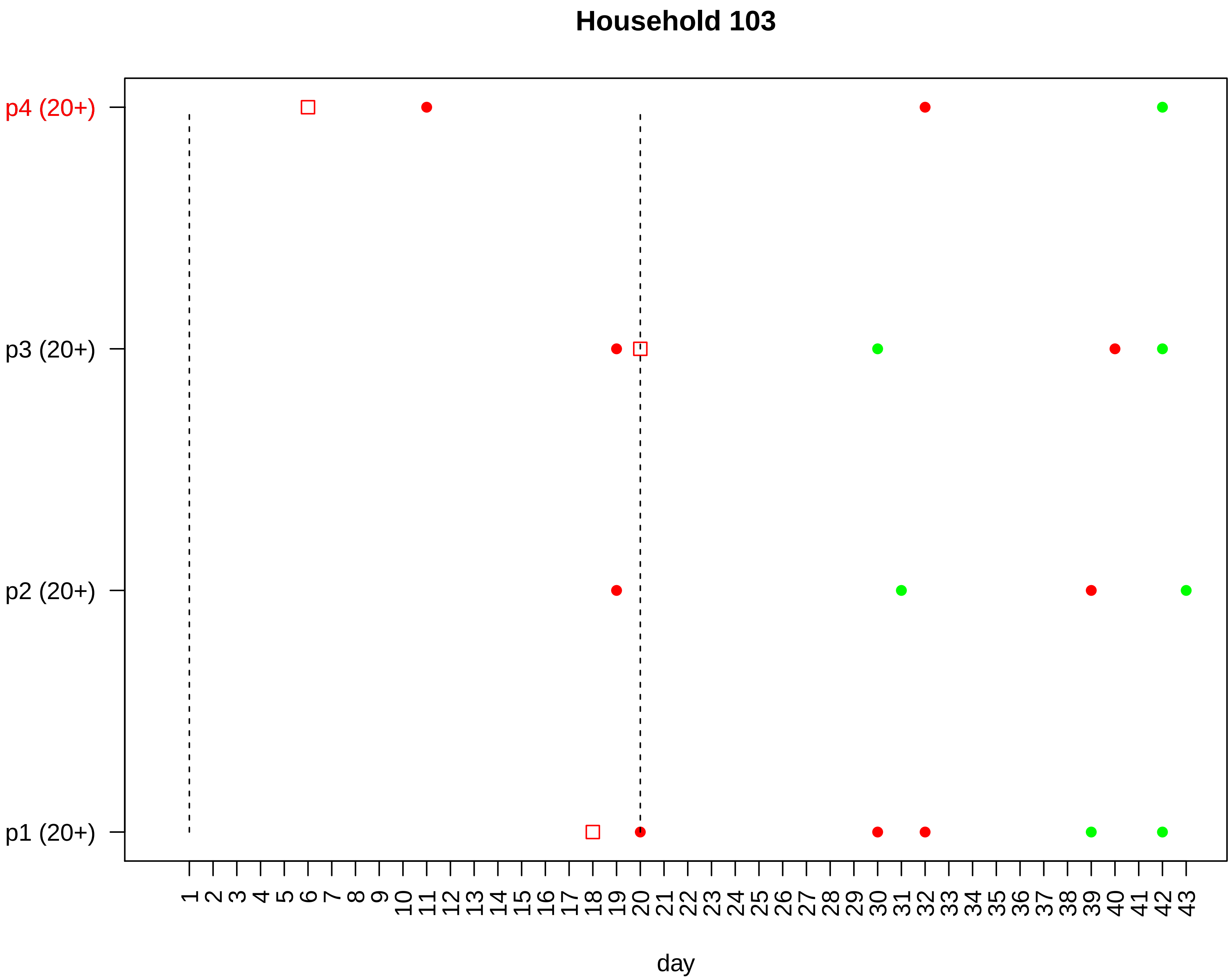

Household 103
p4 (20+)
p3 (20+)
p2 (20+)
p1 (20+)
1 2 3 4 5 6 7 8 9 10 11 12 13 14 15 16 17 18 19 20 21 22 23 24 25 26 27 28 29 30 31 32 33 34 35 36 37 38 39 40 41 42 43
day

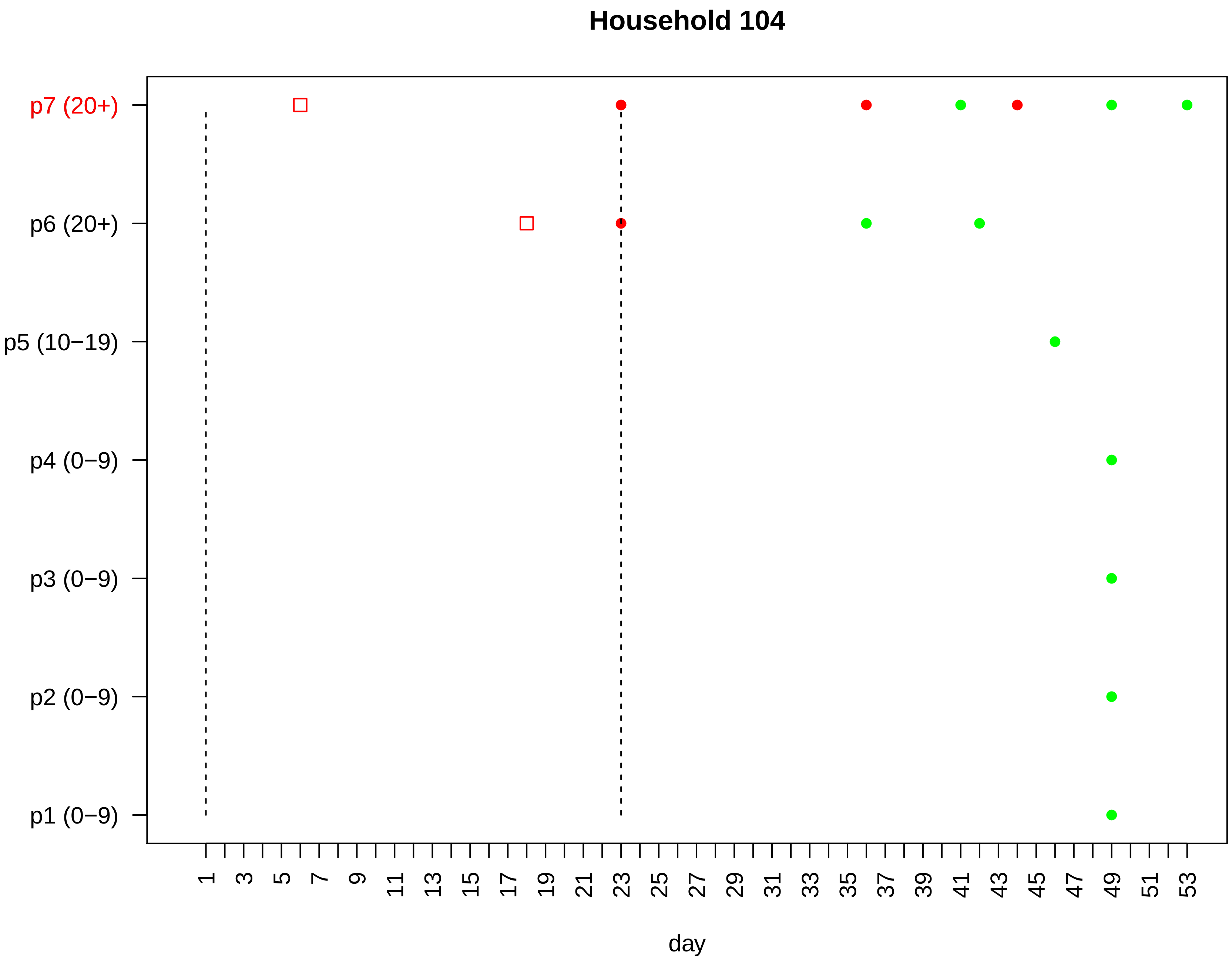

Household 104
p7 (20+)
p6 (20+)
p5 (10−19)
p4 (0−9)
p3 (0−9)
p2 (0−9)
p1 (0−9)
1 3 5 7 9 11 13 15 17 19 21 23 25 27 29 31 33 35 37 39 41 43 45 47 49 51 53
day

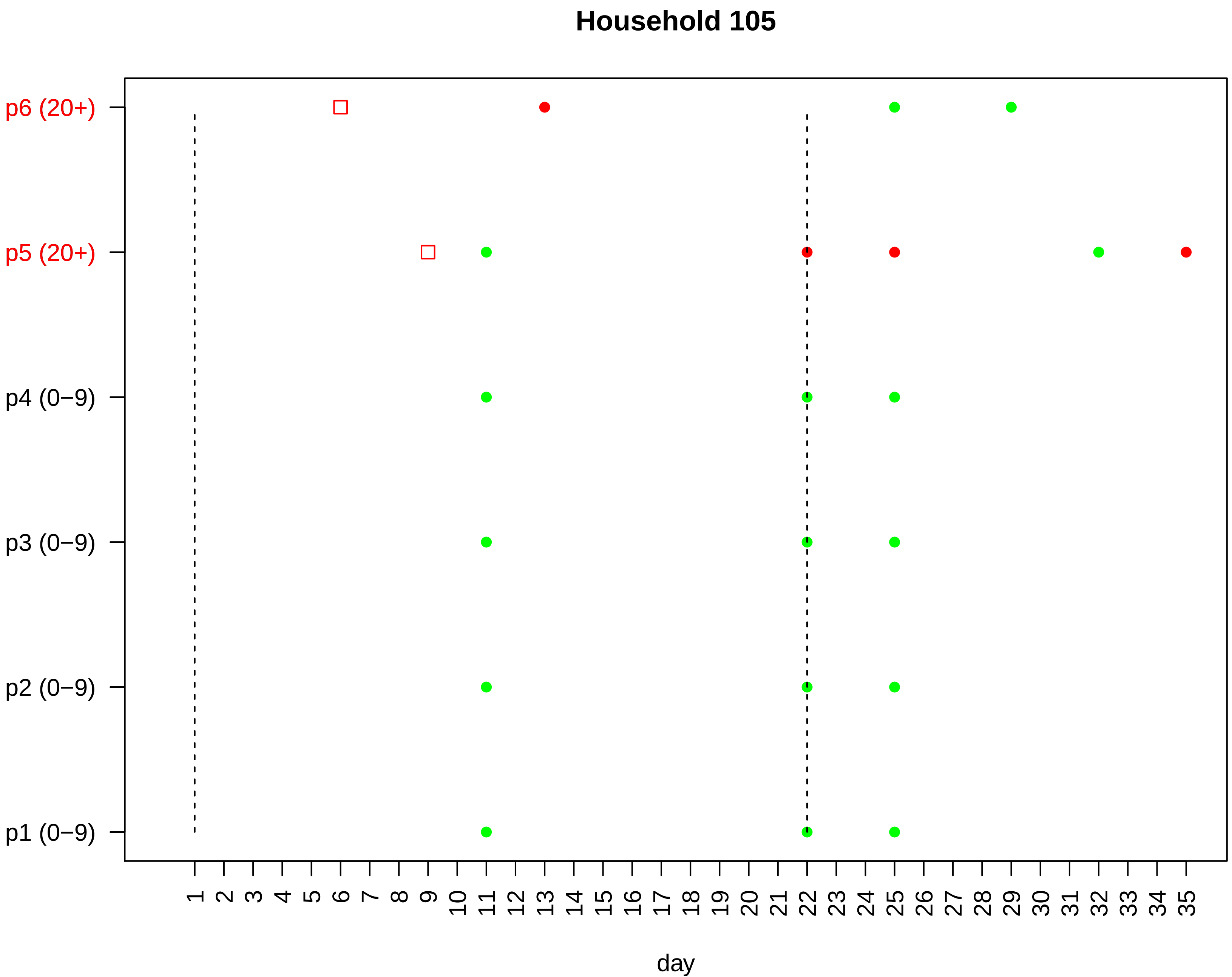
Household 105
p6 (20+)
p5 (20+)
p4 (0−9)
p3 (0−9)
p2 (0−9)
p1 (0−9)
1 2 3 4 5 6 7 8 9 10 11 12 13 14 15 16 17 18 19 20 21 22 23 24 25 26 27 28 29 30 31 32 33 34 35
day

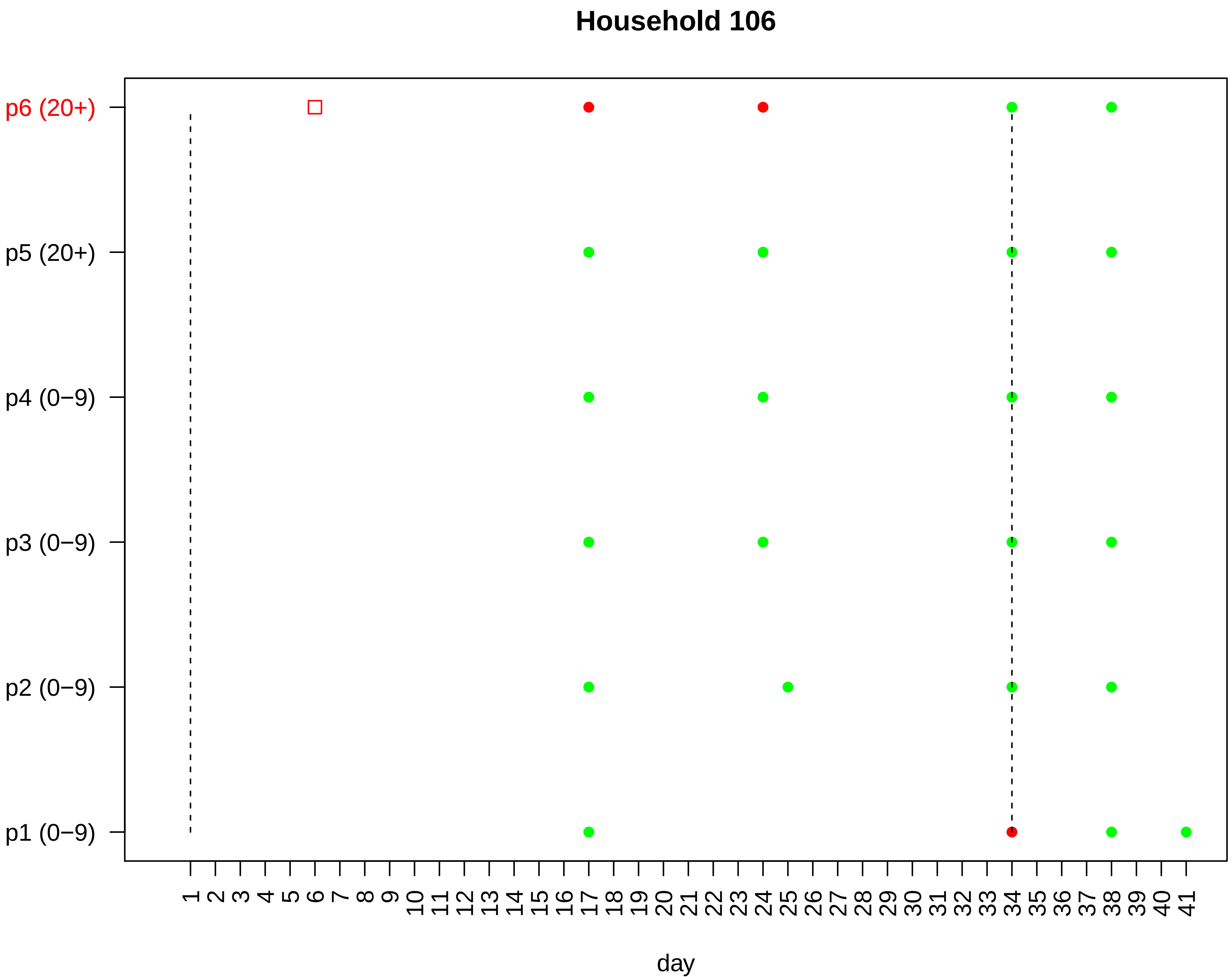

Household 106
p6 (20+)
p5 (20+)
p4 (0−9)
p3 (0−9)
p2 (0−9)
p1 (0−9)
1 2 3 4 5 6 7 8 9 10 11 12 13 14 15 16 17 18 19 20 21 22 23 24 25 26 27 28 29 30 31 32 33 34 35 36 37 38 39 40 41
day

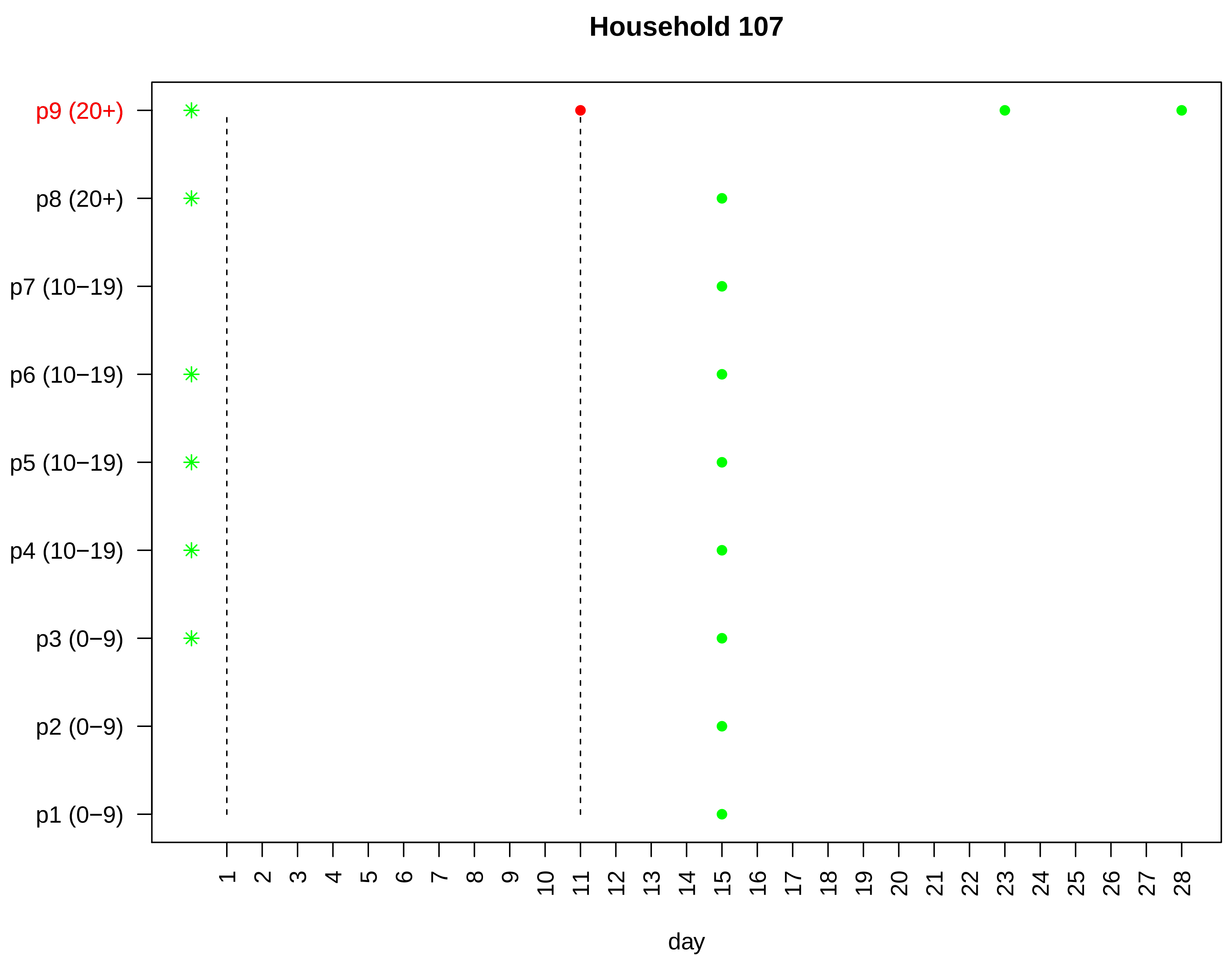

Household 107
p9 (20+)
p8 (20+)
p7 (10−19)
p6 (10−19)
p5 (10−19)
p4 (10−19)
p3 (0−9)
p2 (0−9)
p1 (0−9)
1 2 3 4 5 6 7 8 9 10 11 12 13 14 15 16 17 18 19 20 21 22 23 24 25 26 27 28
day

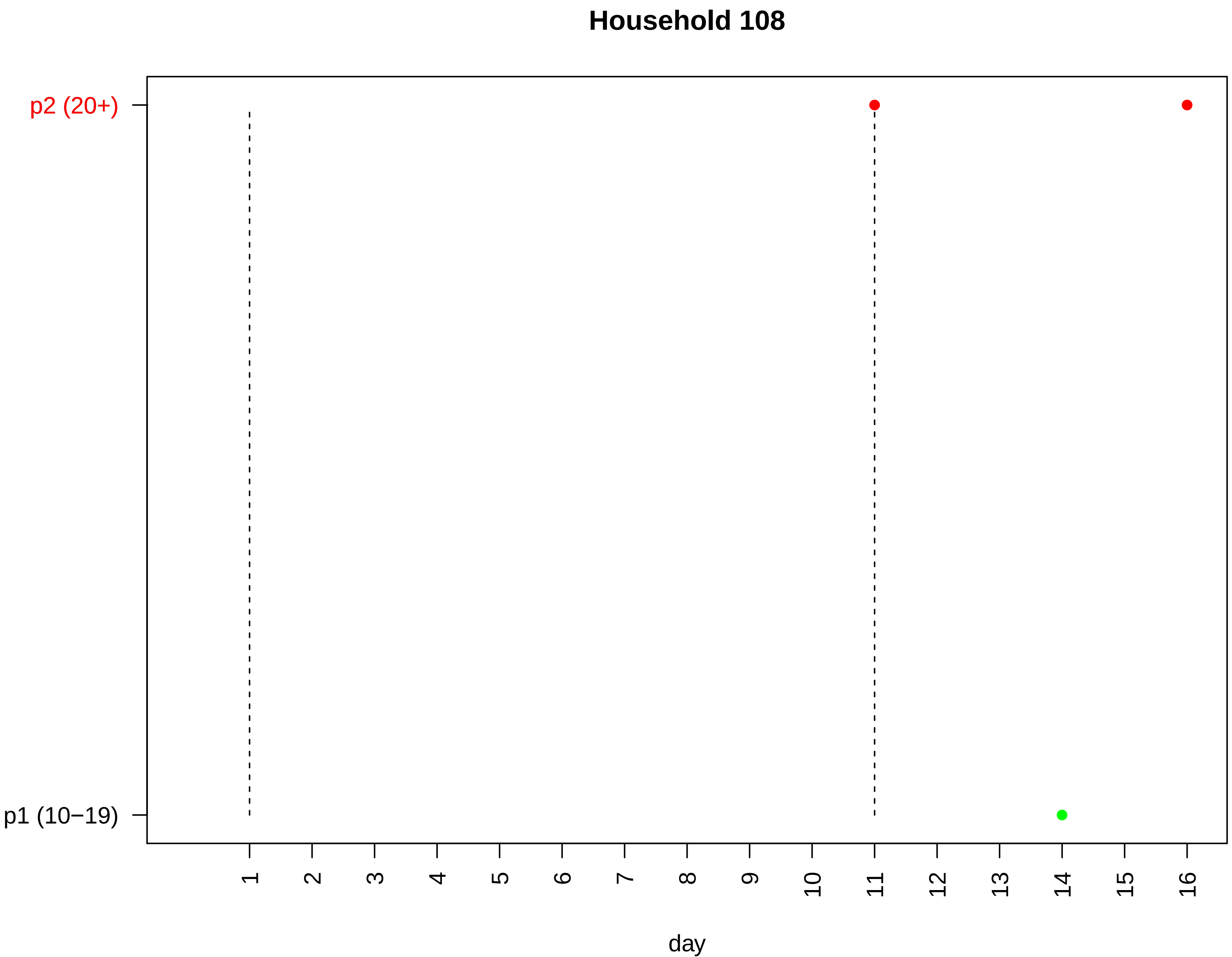

Household 108
p2 (20+)
p1 (10−19)
1
2
3
4
5
6
7
8
9
10
11
12
13
14
15
16
day

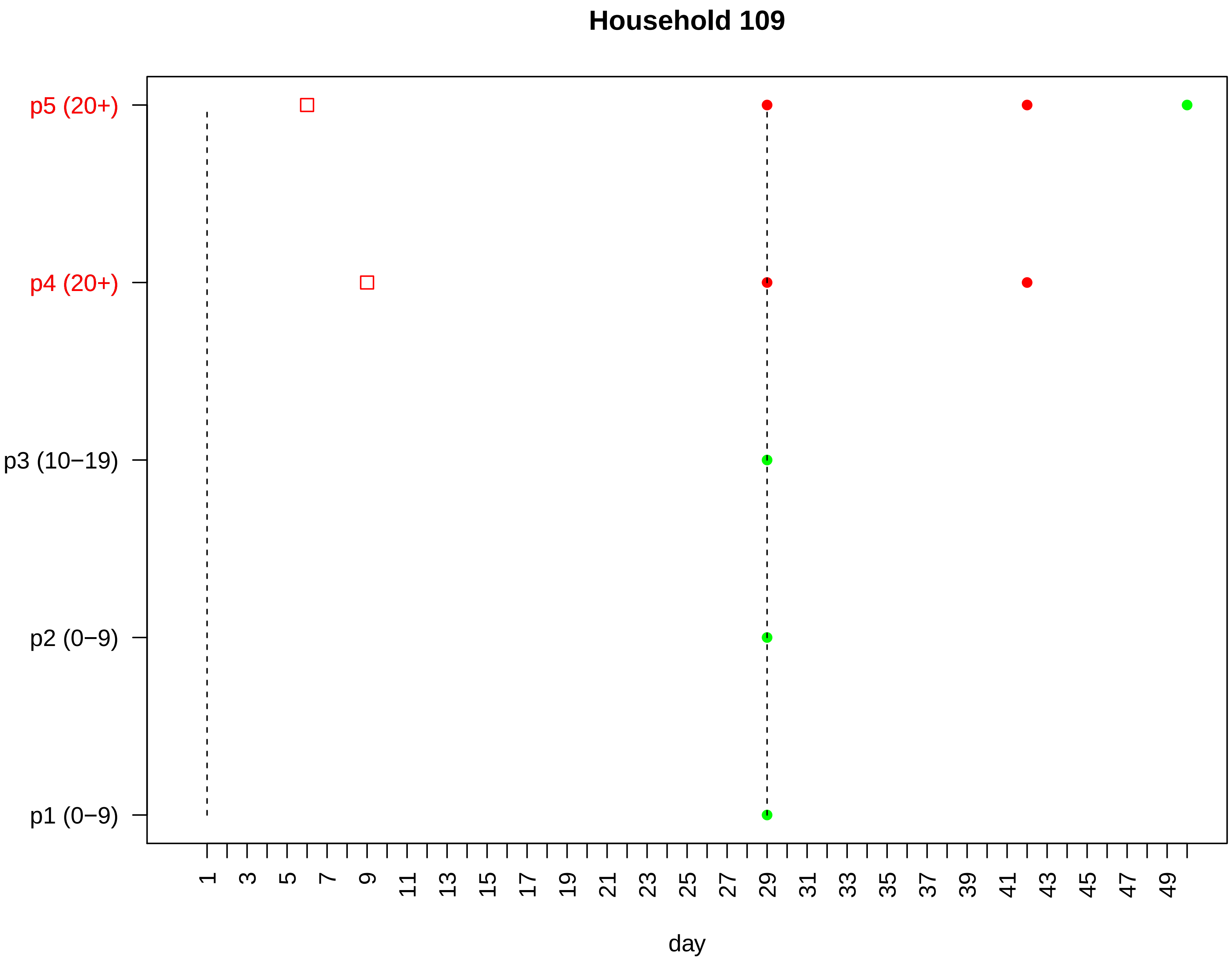

Household 109
p5 (20+)
p4 (20+)
p3 (10−19)
p2 (0−9)
p1 (0−9)
1
3
5
7
9
11
13
15
17
19
21
23
25
27
29
31
33
35
37
39
41
43
45
47
49
day

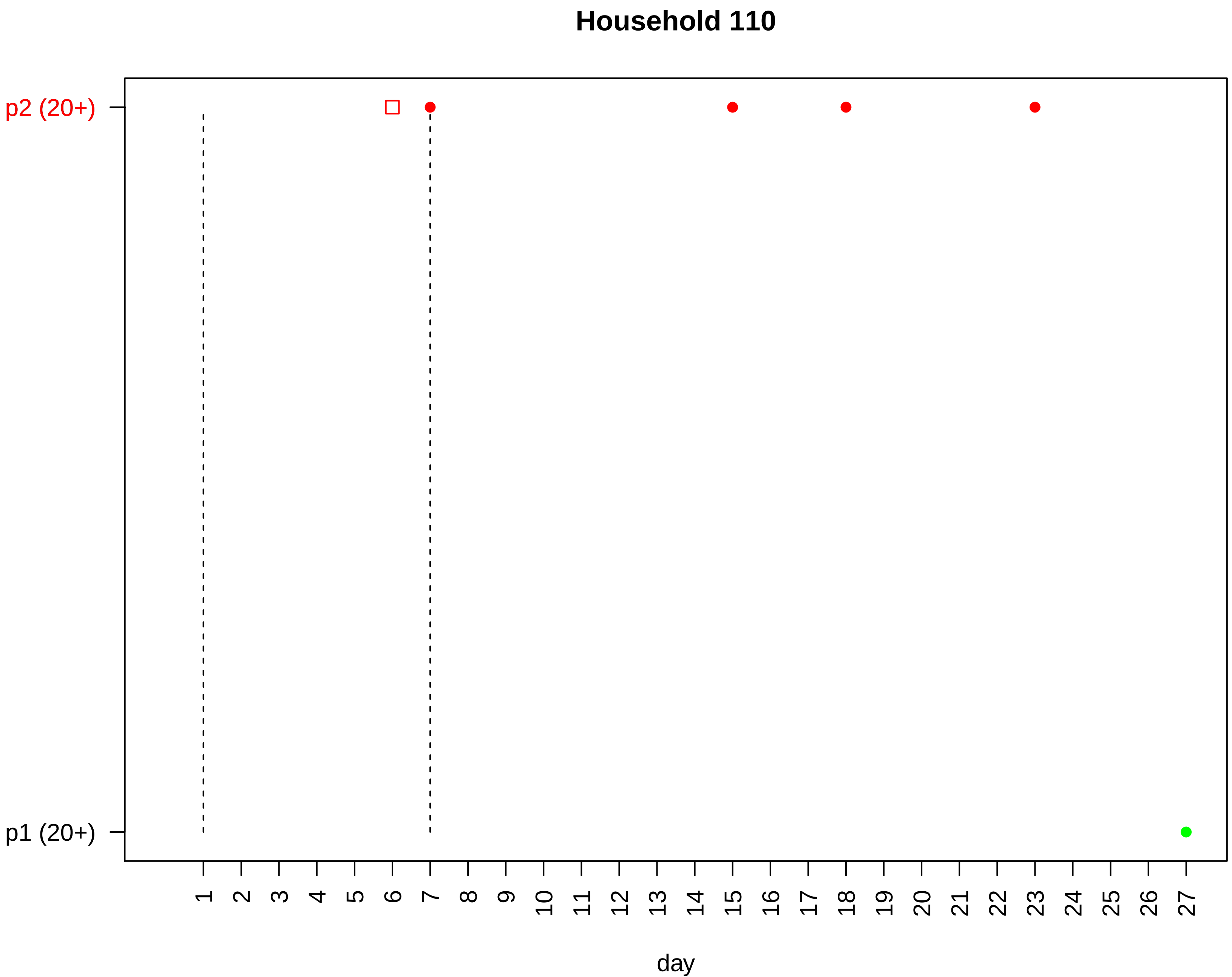
Household 110
p2 (20+)
p1 (20+)
1
2
3
4
5
6
7
8
9
10
11
12
13
14
15
16
17
18
19
20
21
22
23
24
25
26
27
day

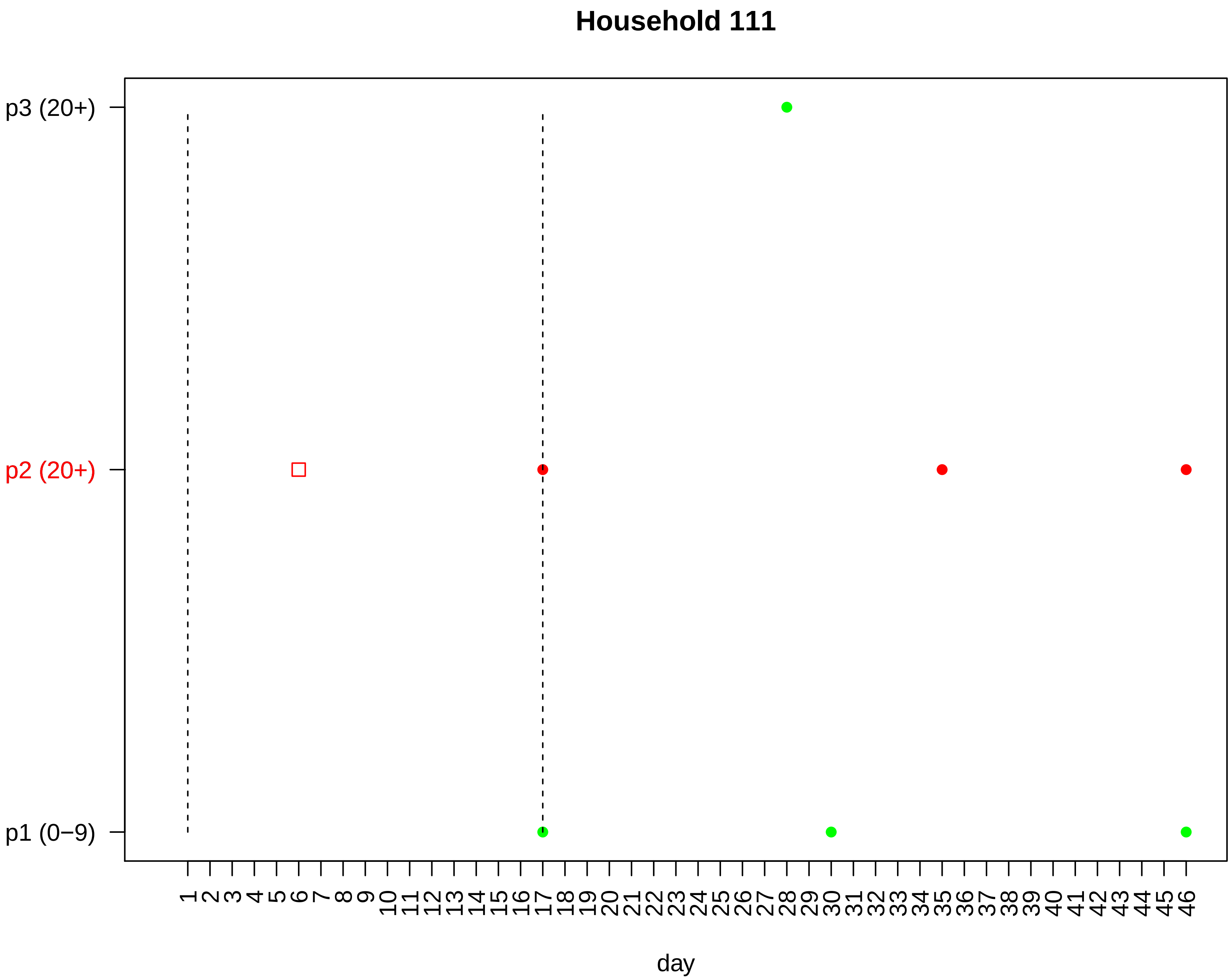

Household 111
p3 (20+)
p2 (20+)
p1 (0−9)
1 2 3 4 5 6 7 8 9 10 11 12 13 14 15 16 17 18 19 20 21 22 23 24 25 26 27 28 29 30 31 32 33 34 35 36 37 38 39 40 41 42 43 44 45 46
day

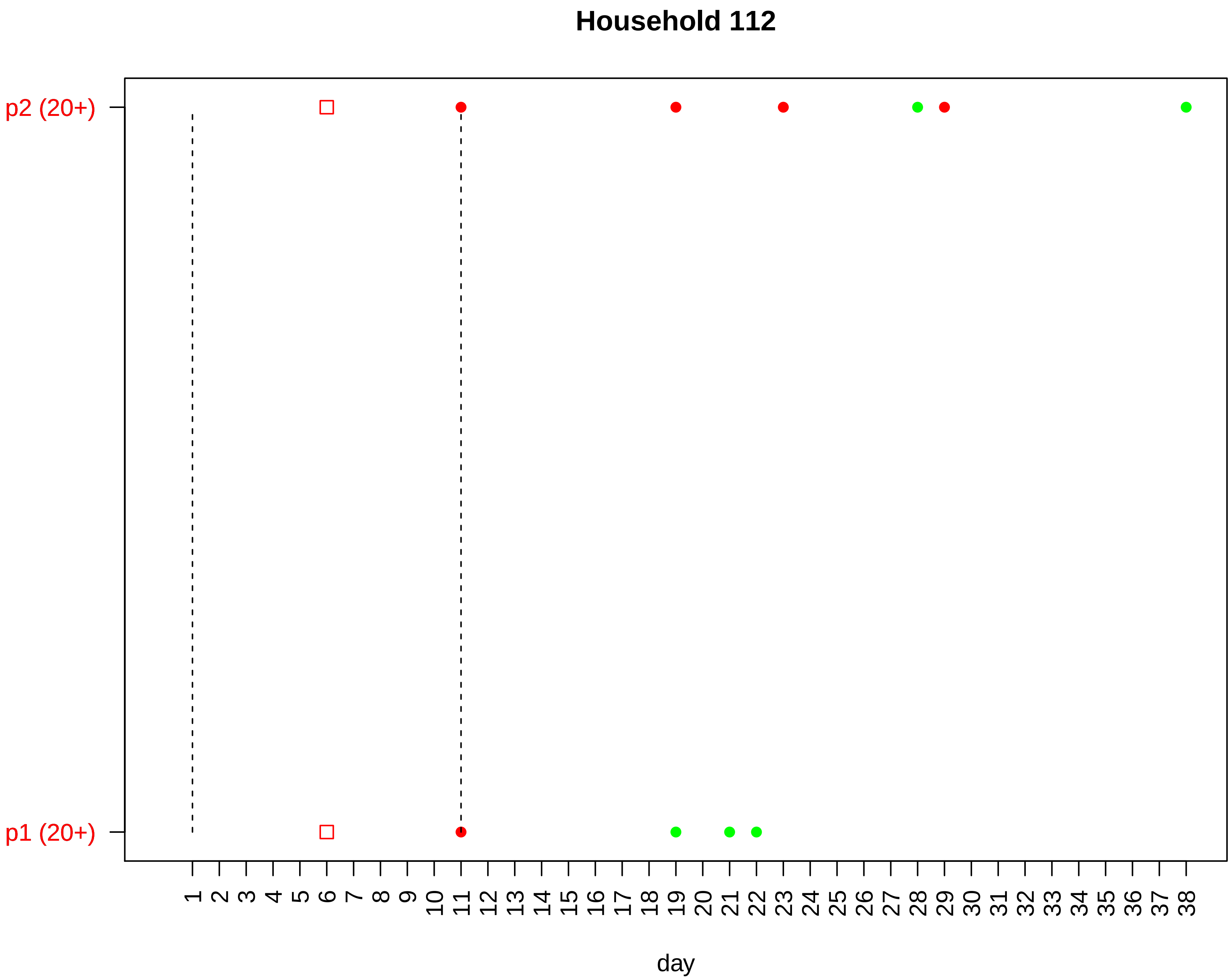

Household 112
p2 (20+)
p1 (20+)
1 2 3 4 5 6 7 8 9 10 11 12 13 14 15 16 17 18 19 20 21 22 23 24 25 26 27 28 29 30 31 32 33 34 35 36 37 38
day

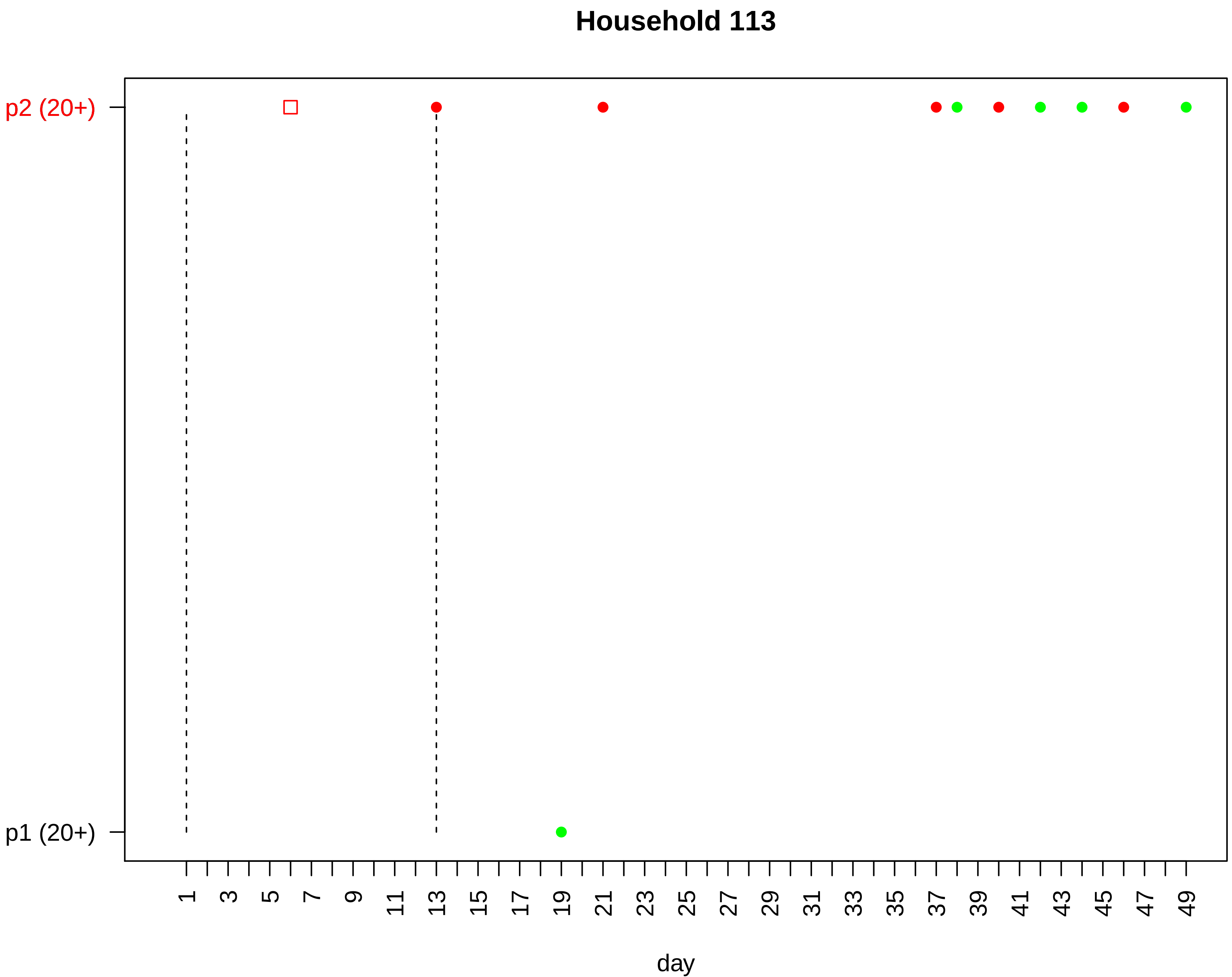

Household 113
p2 (20+)
p1 (20+)
1
3
5
7
9
11
13
15
17
19
21
23
25
27
29
31
33
35
37
39
41
43
45
47
49
day

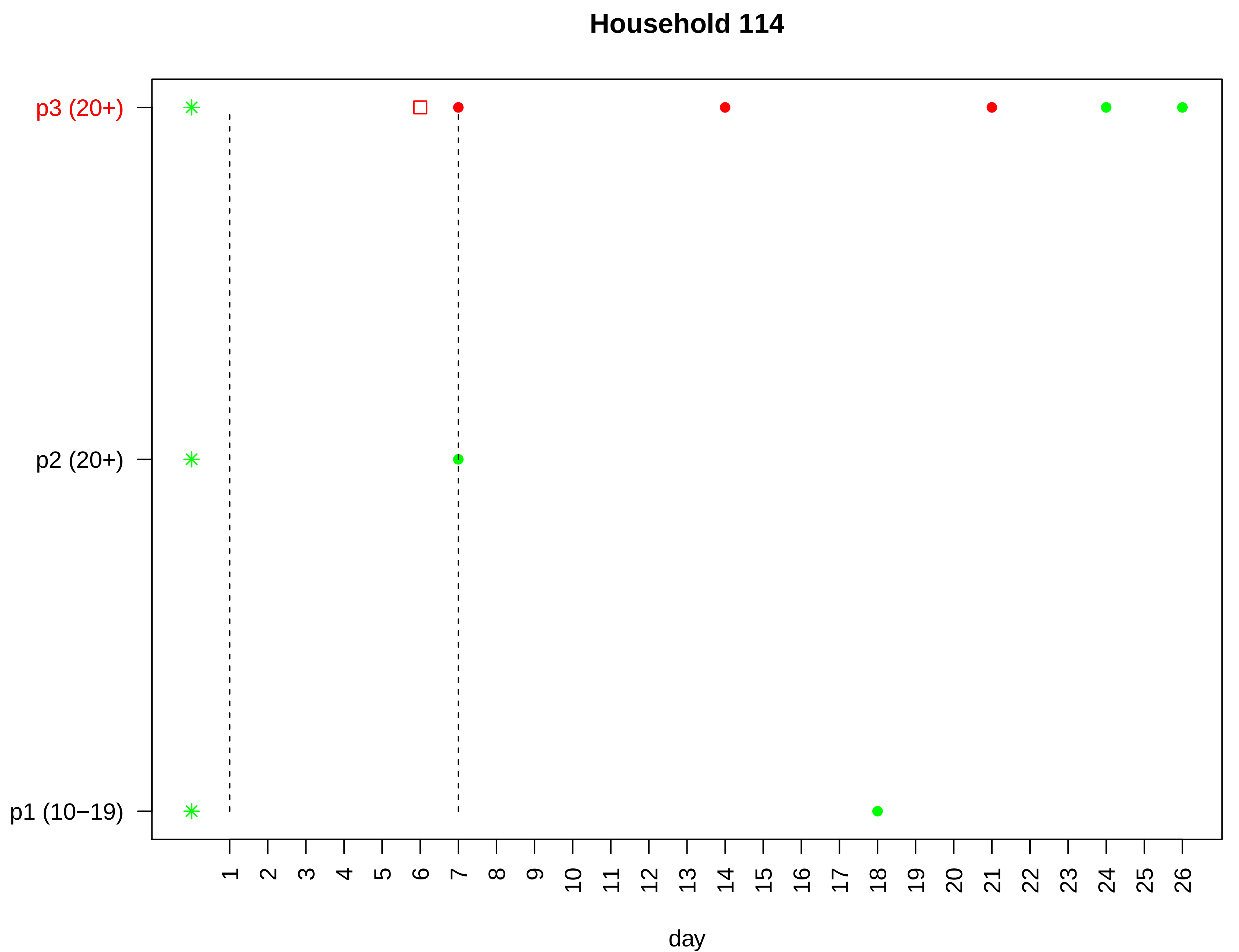

Household 114
p3 (20+)
p2 (20+)
p1 (10−19)
1 2 3 4 5 6 7 8 9 10 11 12 13 14 15 16 17 18 19 20 21 22 23 24 25 26
day

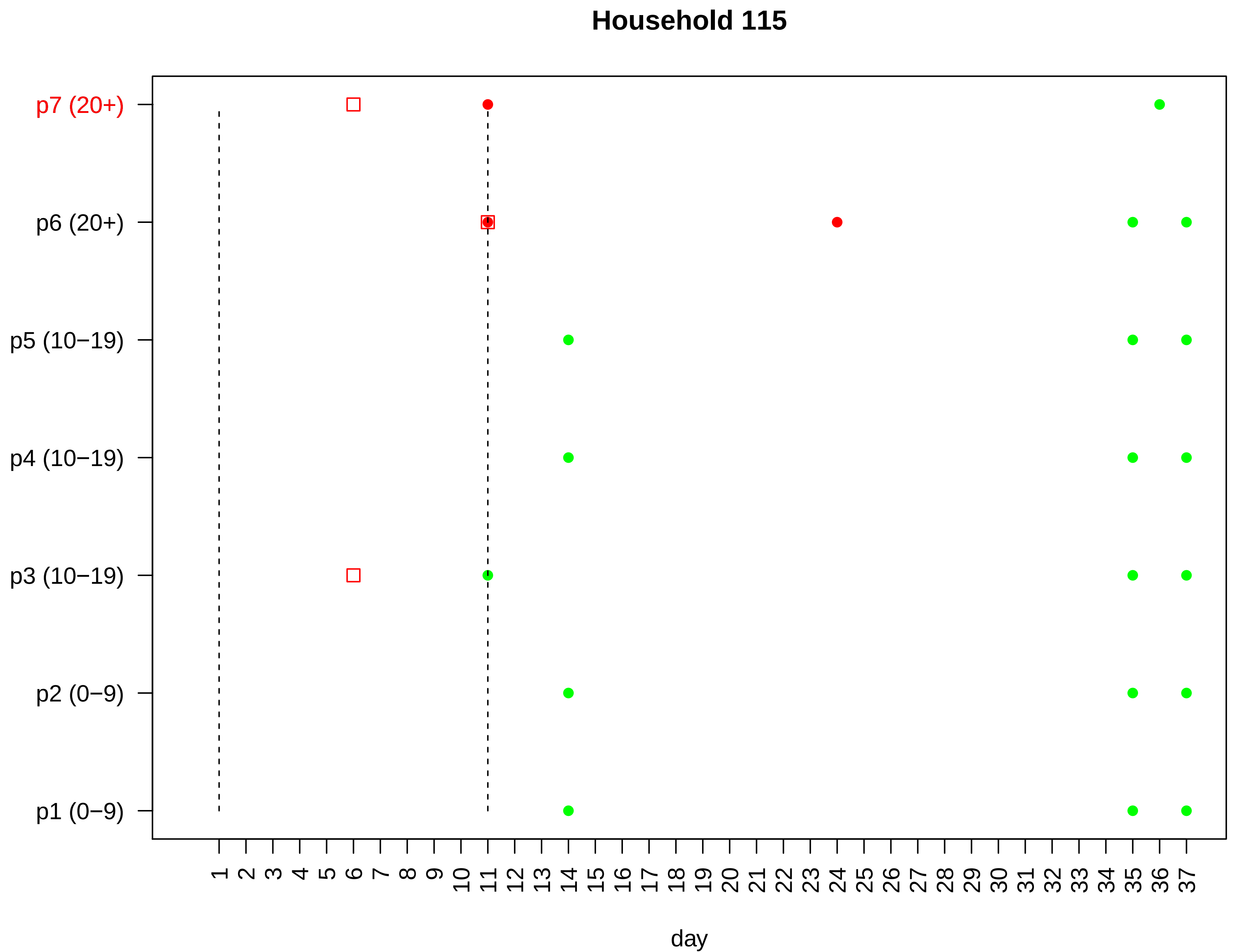

Household 115
p7 (20+)
p6 (20+)
p5 (10−19)
p4 (10−19)
p3 (10−19)
p2 (0−9)
p1 (0−9)
1 2 3 4 5 6 7 8 9 10 11 12 13 14 15 16 17 18 19 20 21 22 23 24 25 26 27 28 29 30 31 32 33 34 35 36 37
day

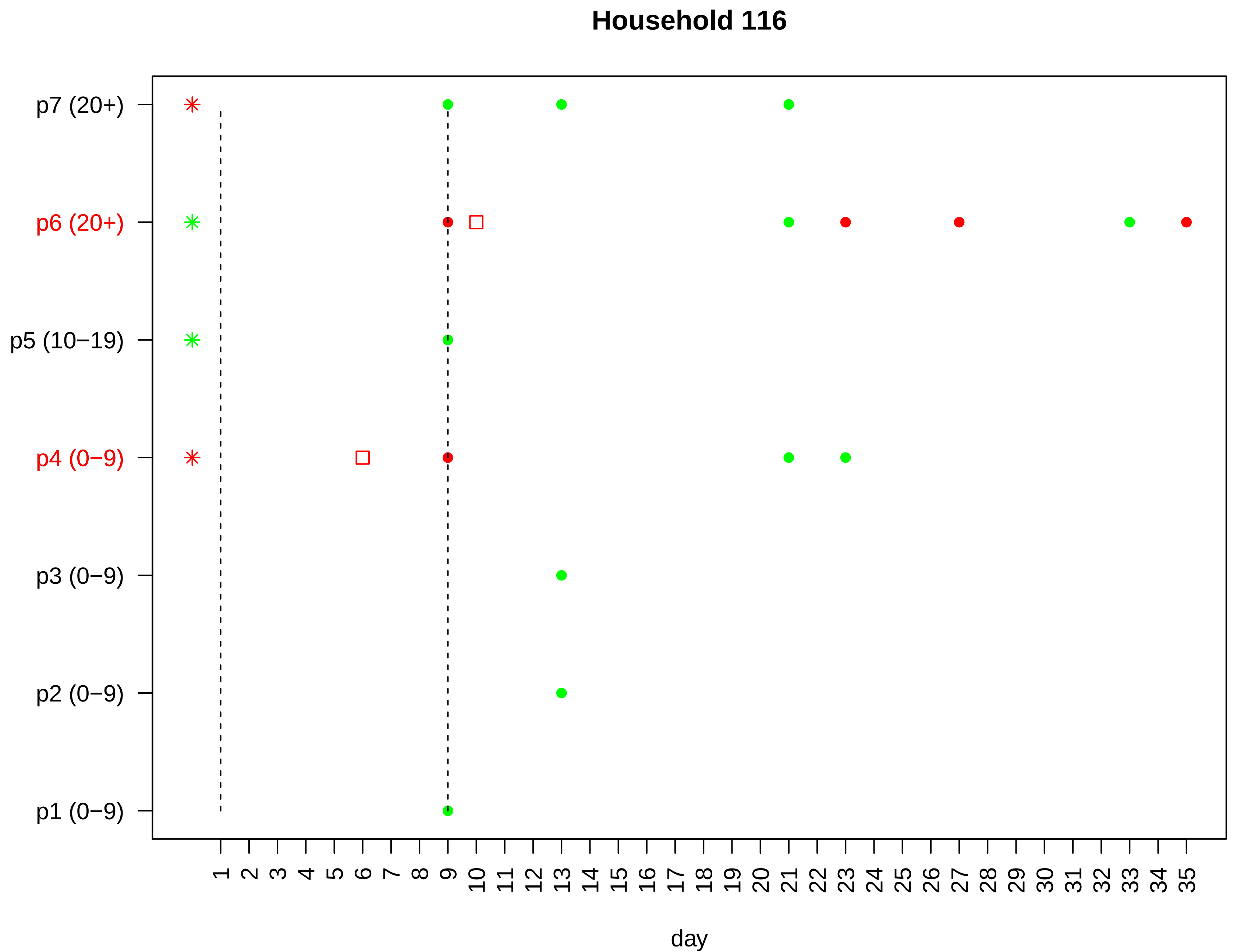
Household 116
p7 (20+)
p6 (20+)
p5 (10−19)
p4 (0−9)
p3 (0−9)
p2 (0−9)
p1 (0−9)
1 2 3 4 5 6 7 8 9 10 11 12 13 14 15 16 17 18 19 20 21 22 23 24 25 26 27 28 29 30 31 32 33 34 35
day

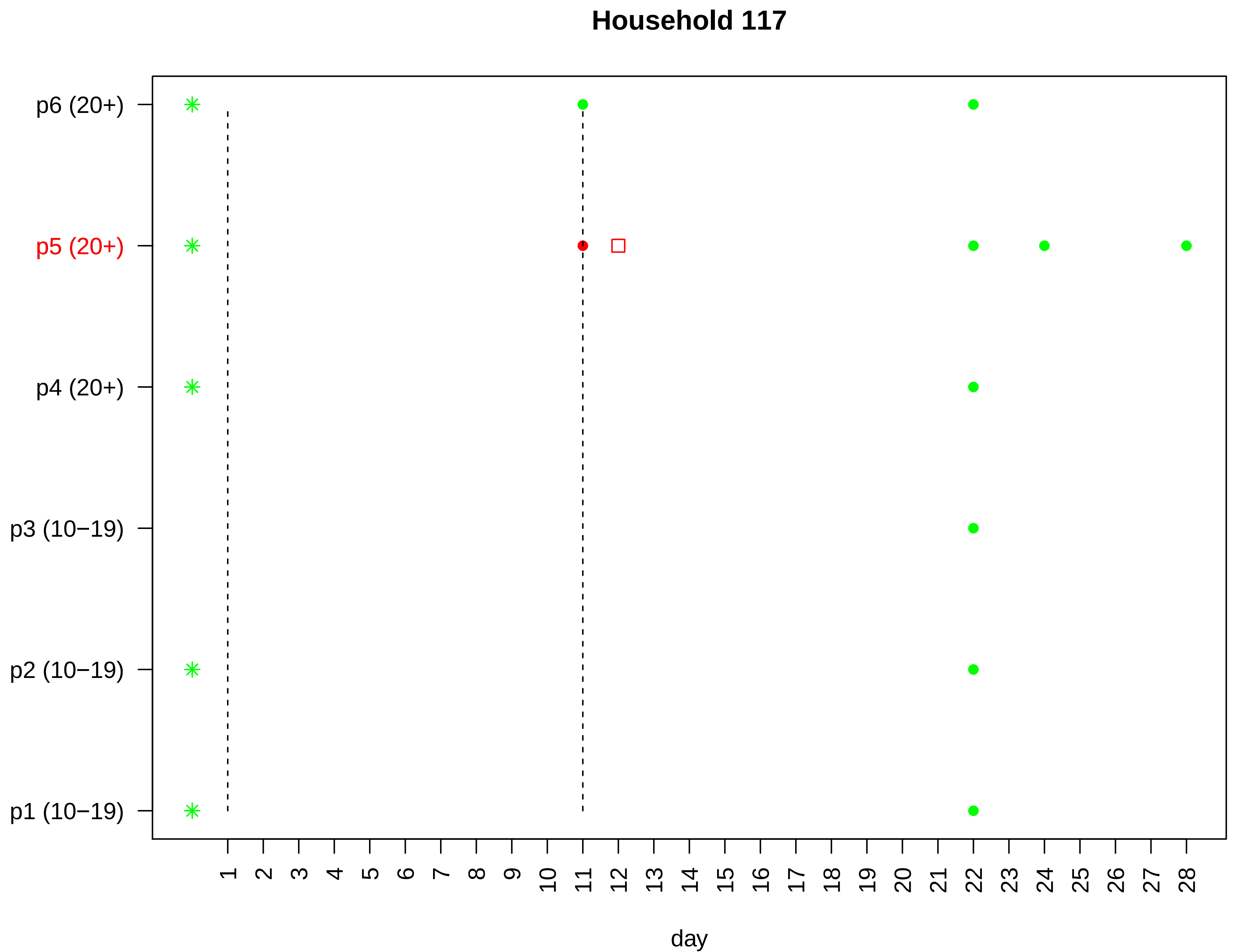

Household 117
p6 (20+)
p5 (20+)
p4 (20+)
p3 (10−19)
p2 (10−19)
p1 (10−19)
1
2
3
4
5
6
7
8
9
10
11
12
13
14
15
16
17
18
19
20
21
22
23
24
25
26
27
28
day

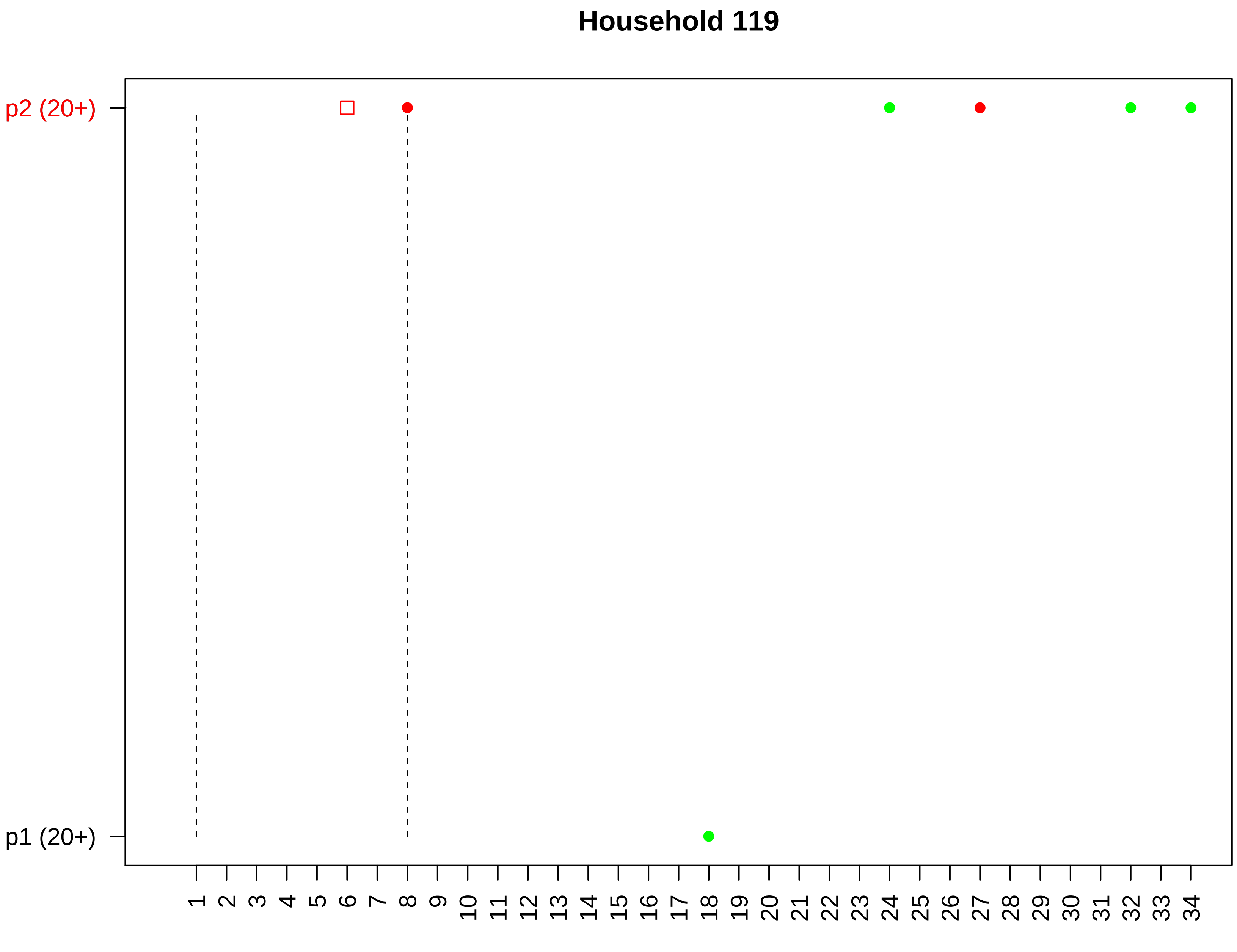
Household 119
p2 (20+)
p1 (20+)
1
2
3
4
5
6
7
8
9
10
11
12
13
14
15
16
17
18
19
20
21
22
23
24
25
26
27
28
29
30
31
32
33
34
day

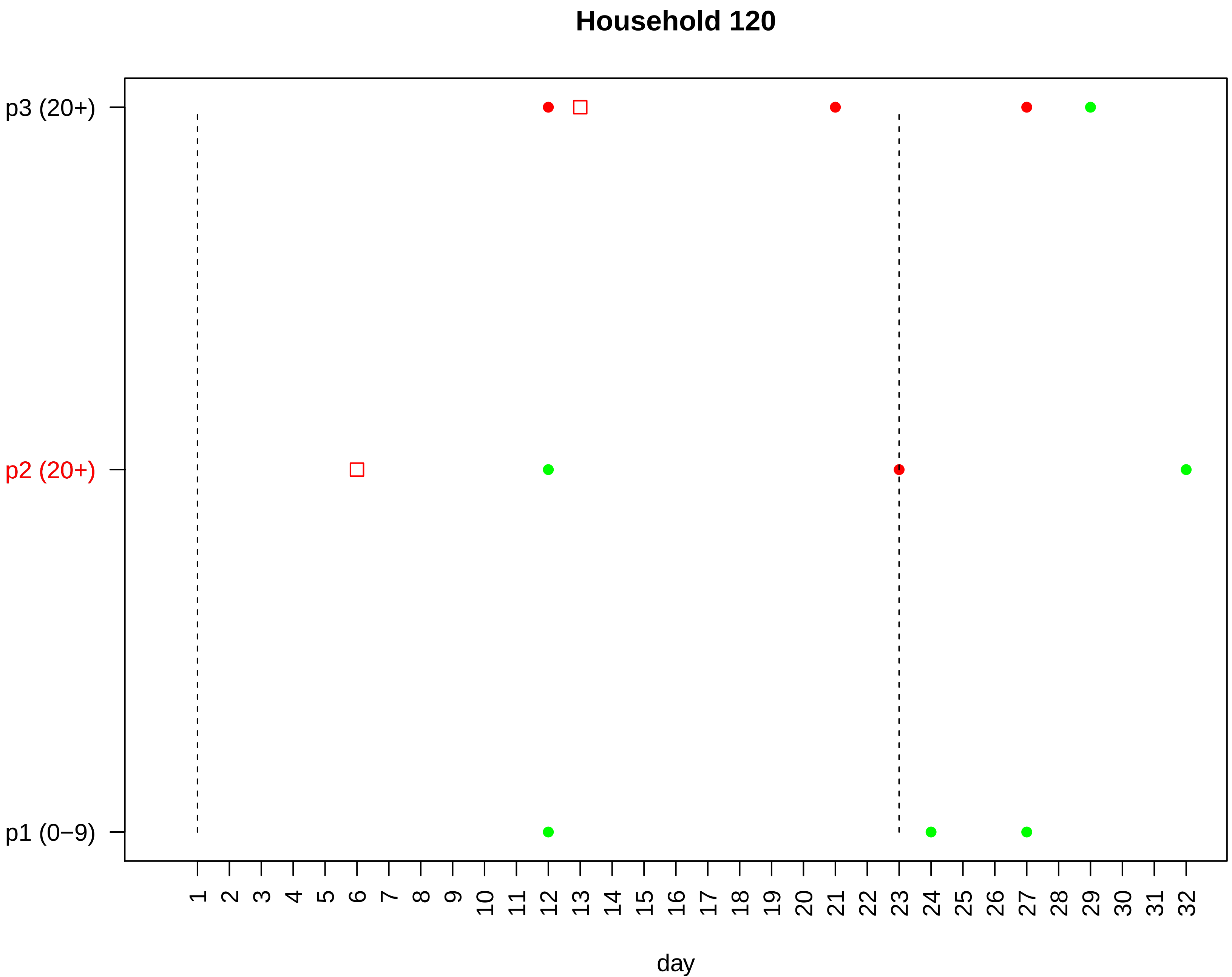

Household 120
p3 (20+)
p2 (20+)
p1 (0−9)
1
2
3
4
5
6
7
8
9
10
11
12
13
14
15
16
17
18
19
20
21
22
23
24
25
26
27
28
29
30
31
32
day

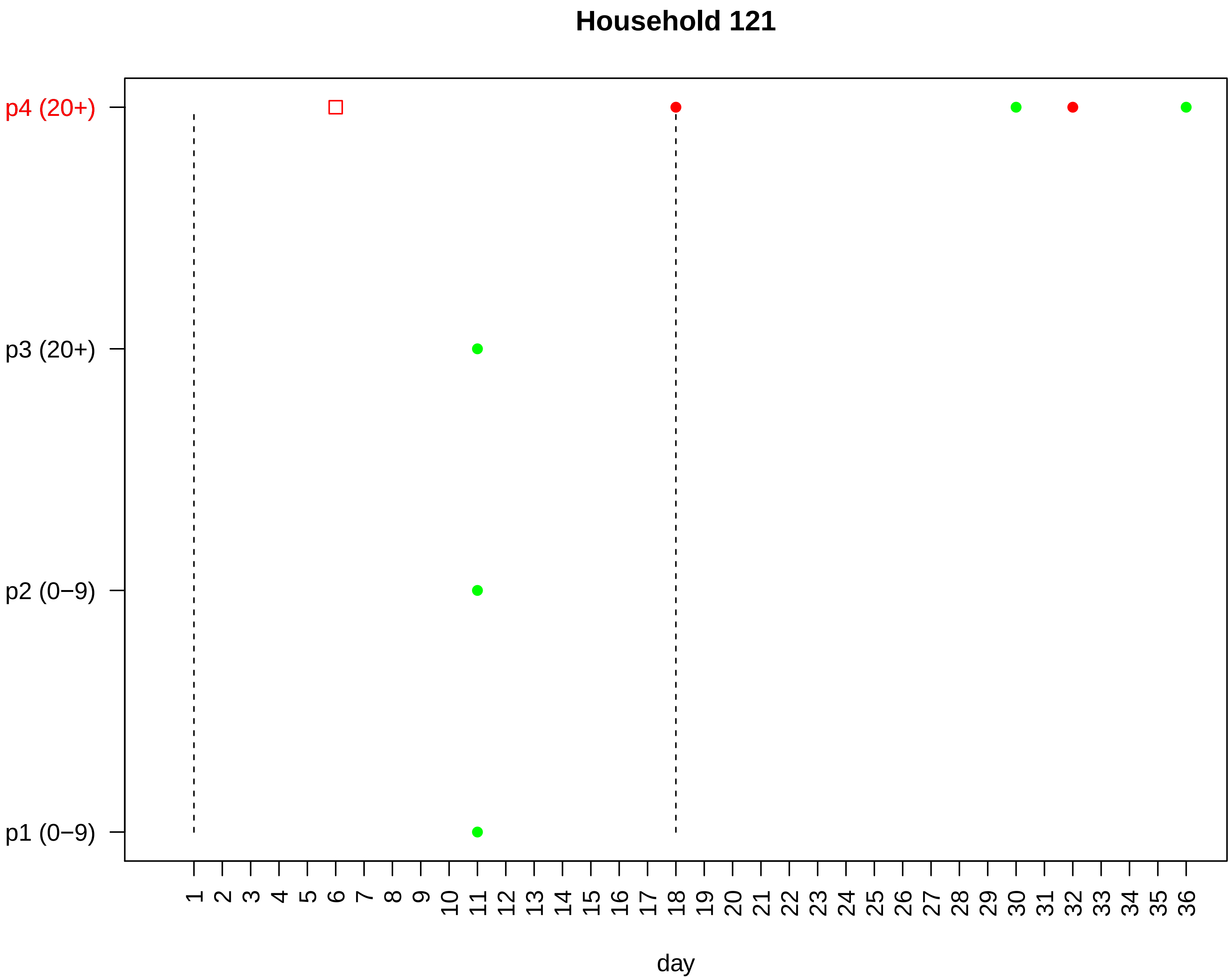
Household 121
p4 (20+)
p3 (20+)
p2 (0−9)
p1 (0−9)
1 2 3 4 5 6 7 8 9 10 11 12 13 14 15 16 17 18 19 20 21 22 23 24 25 26 27 28 29 30 31 32 33 34 35 36
day

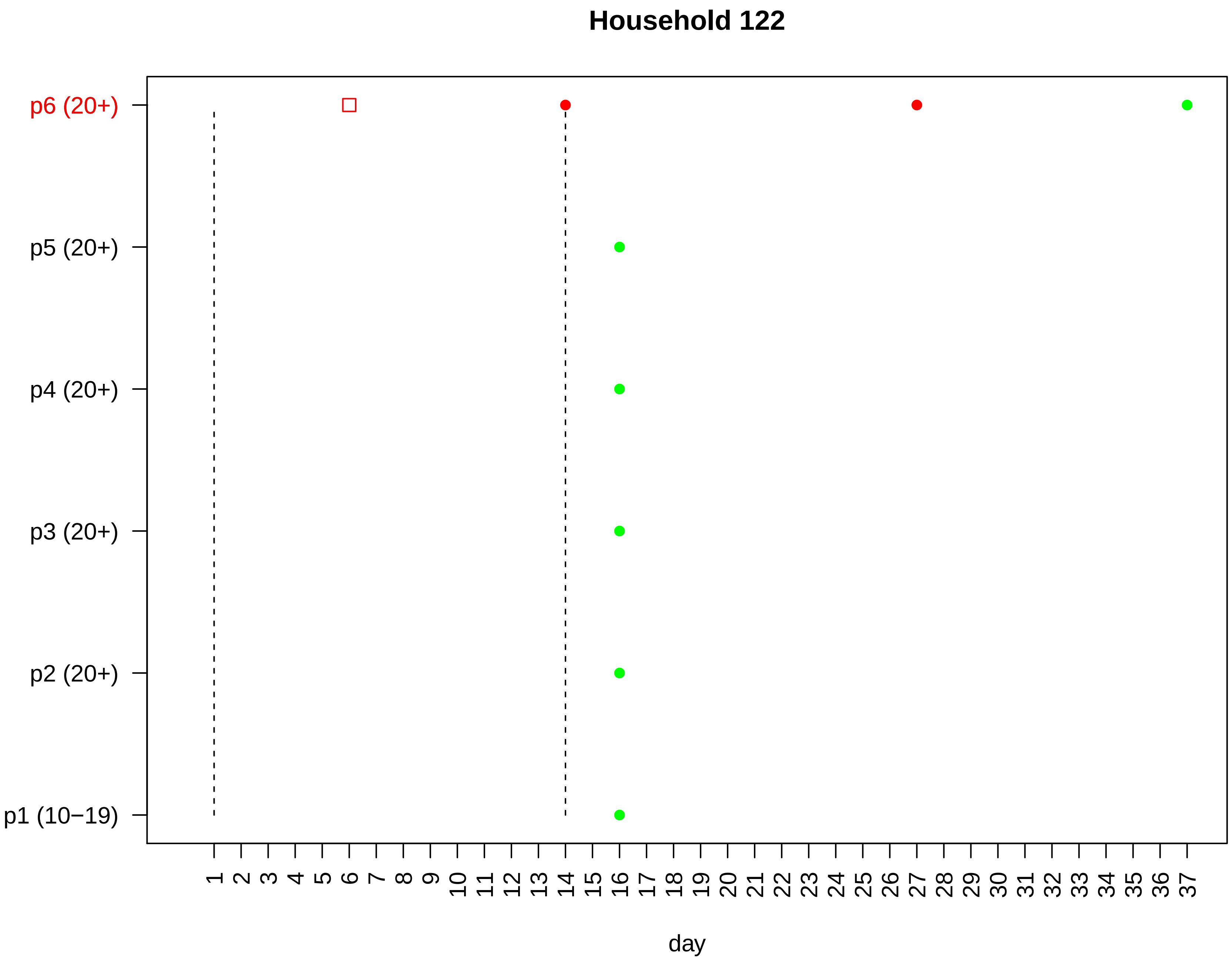
Household 122
p6 (20+)
p5 (20+)
p4 (20+)
p3 (20+)
p2 (20+)
p1 (10−19)
1 2 3 4 5 6 7 8 9 10 11 12 13 14 15 16 17 18 19 20 21 22 23 24 25 26 27 28 29 30 31 32 33 34 35 36 37
day

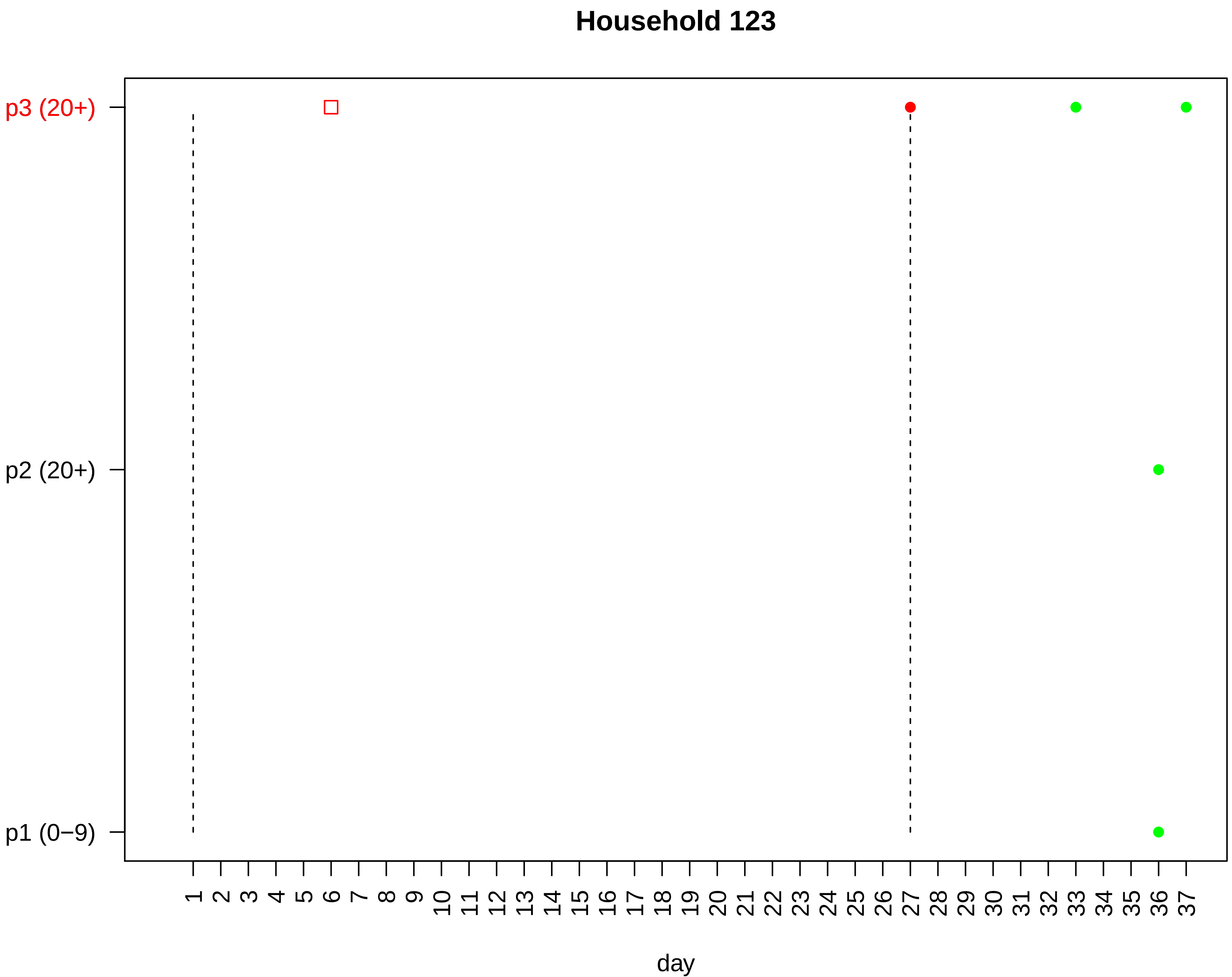

Household 123
p3 (20+)
p2 (20+)
p1 (0−9)
1 2 3 4 5 6 7 8 9 10 11 12 13 14 15 16 17 18 19 20 21 22 23 24 25 26 27 28 29 30 31 32 33 34 35 36 37
day

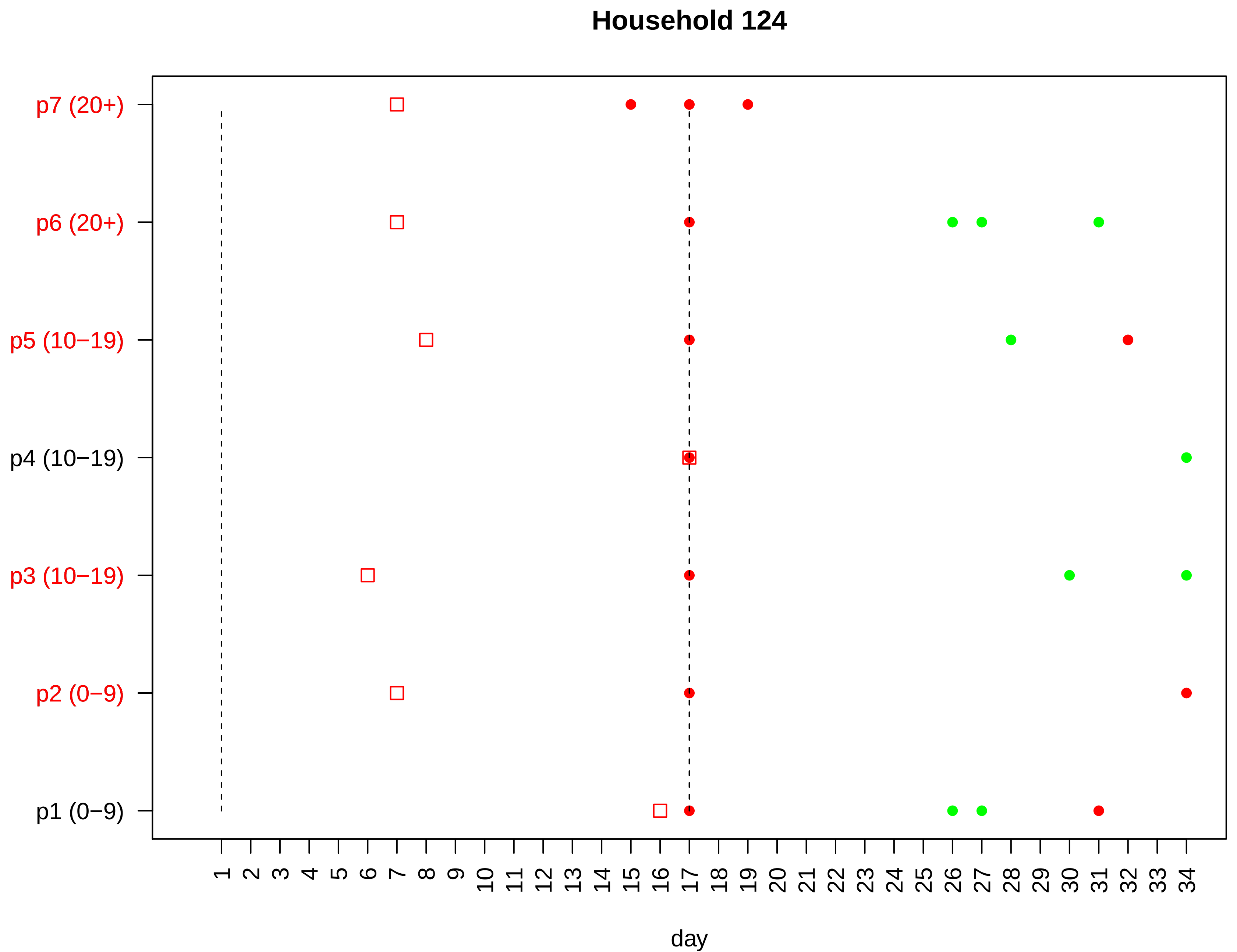

Household 124
p7 (20+)
p6 (20+)
p5 (10−19)
p4 (10−19)
p3 (10−19)
p2 (0−9)
p1 (0−9)
1 2 3 4 5 6 7 8 9 10 11 12 13 14 15 16 17 18 19 20 21 22 23 24 25 26 27 28 29 30 31 32 33 34
day

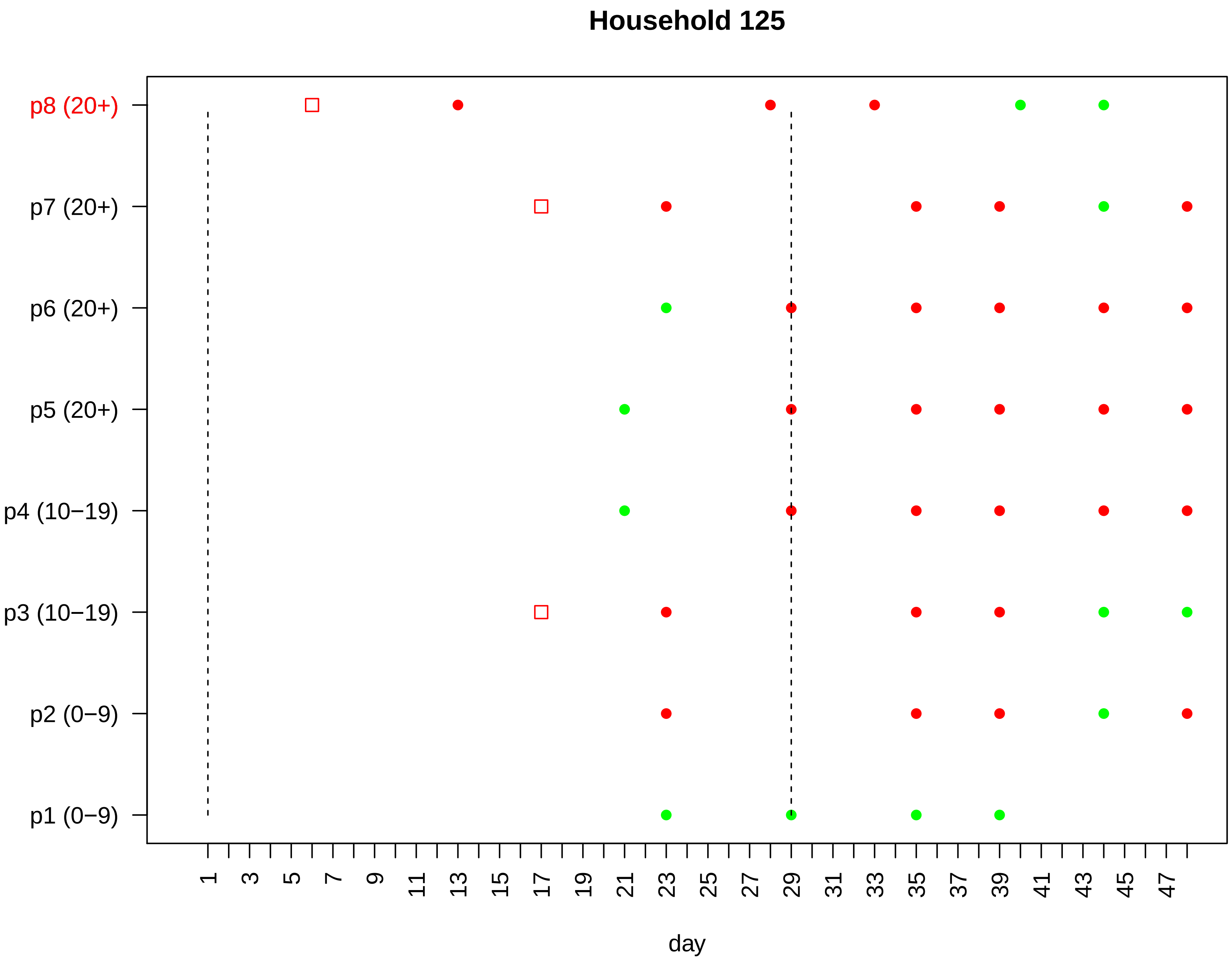

Household 125
p8 (20+)
p7 (20+)
p6 (20+)
p5 (20+)
p4 (10−19)
p3 (10−19)
p2 (0−9)
p1 (0−9)
1
3
5
7
9
11
13
15
17
19
21
23
25
27
29
31
33
35
37
39
41
43
45
47
day

Household 126

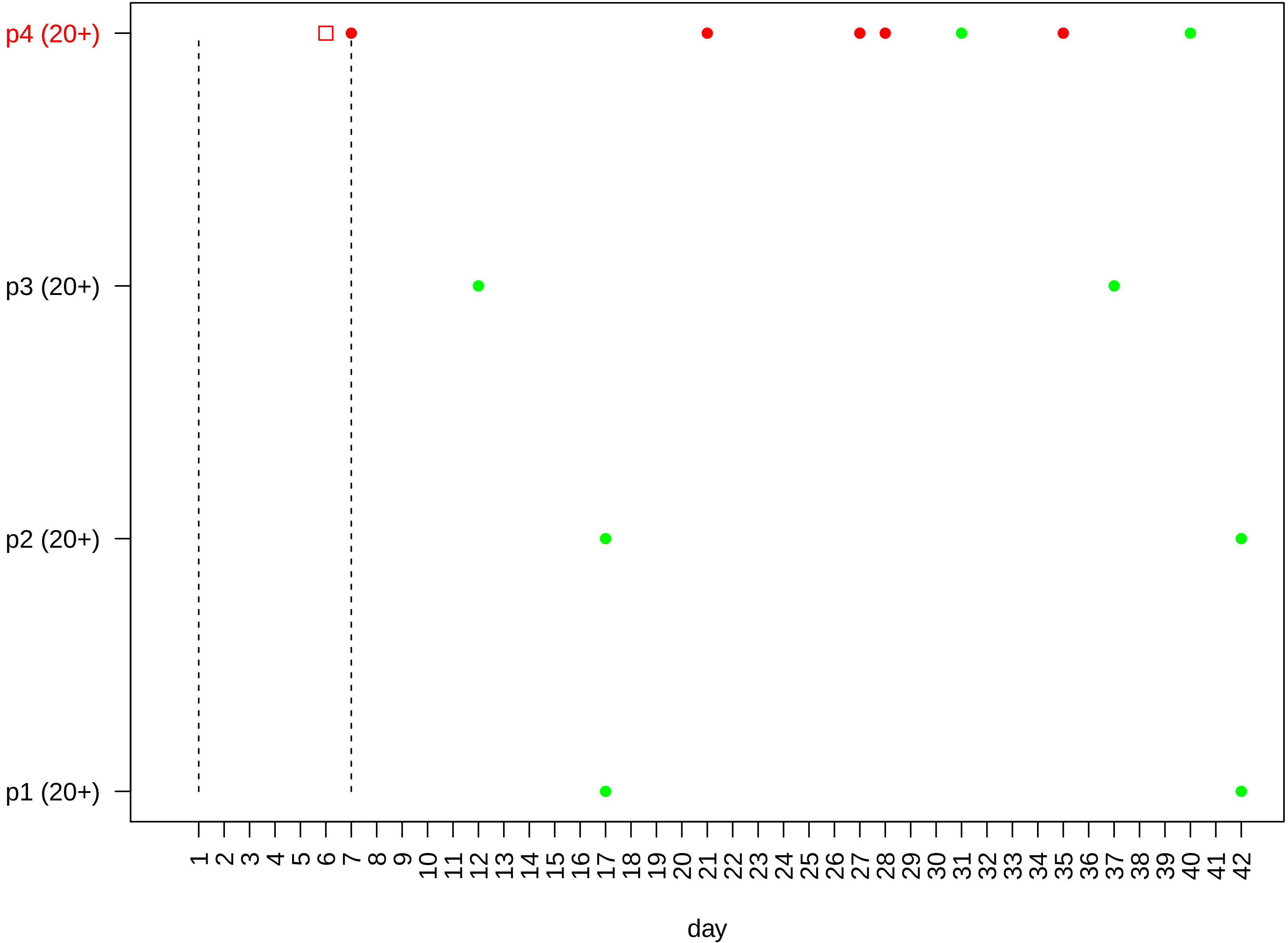

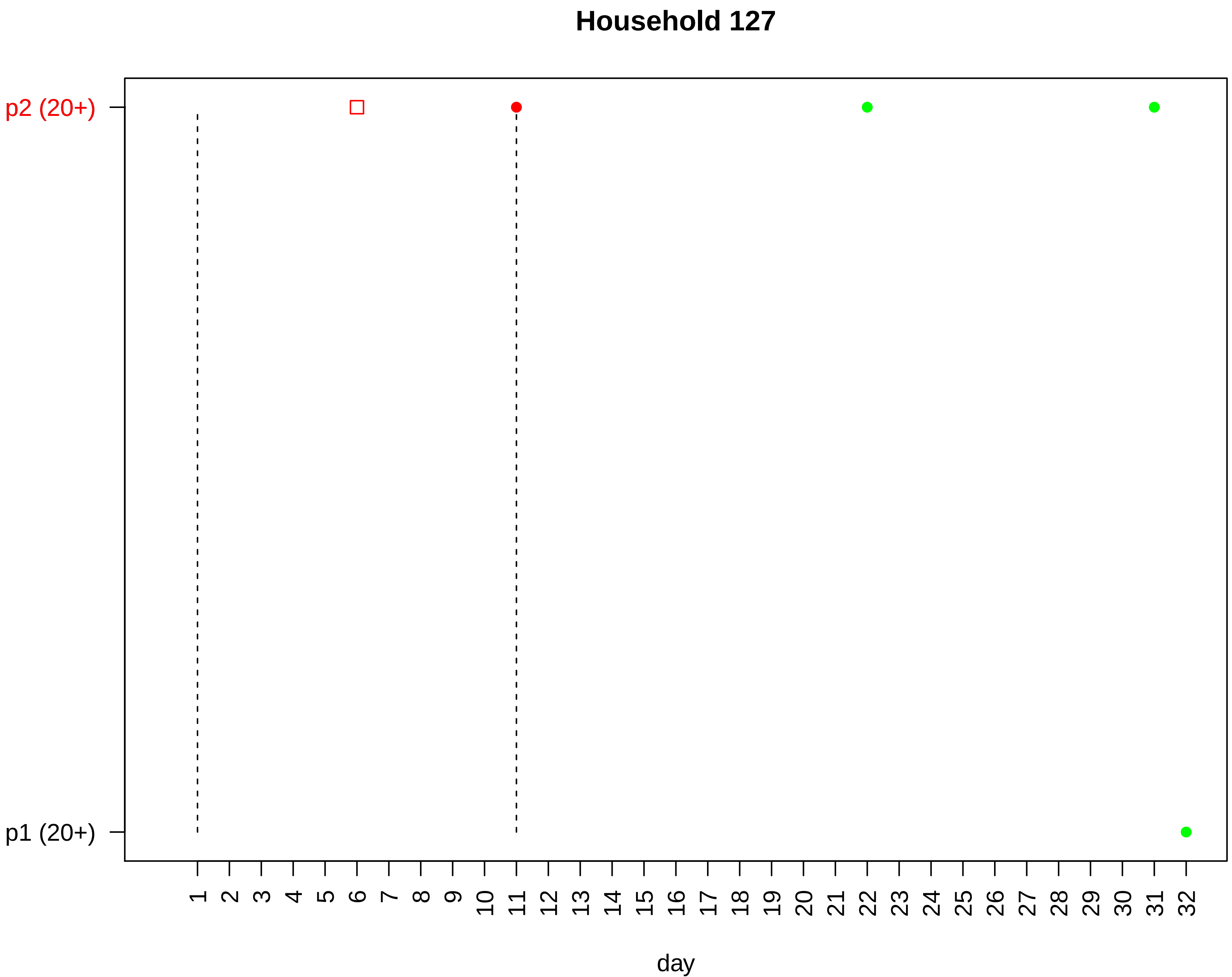

Household 127
p2 (20+)
p1 (20+)
1
2
3
4
5
6
7
8
9
10
11
12
13
14
15
16
17
18
19
20
21
22
23
24
25
26
27
28
29
30
31
32
day

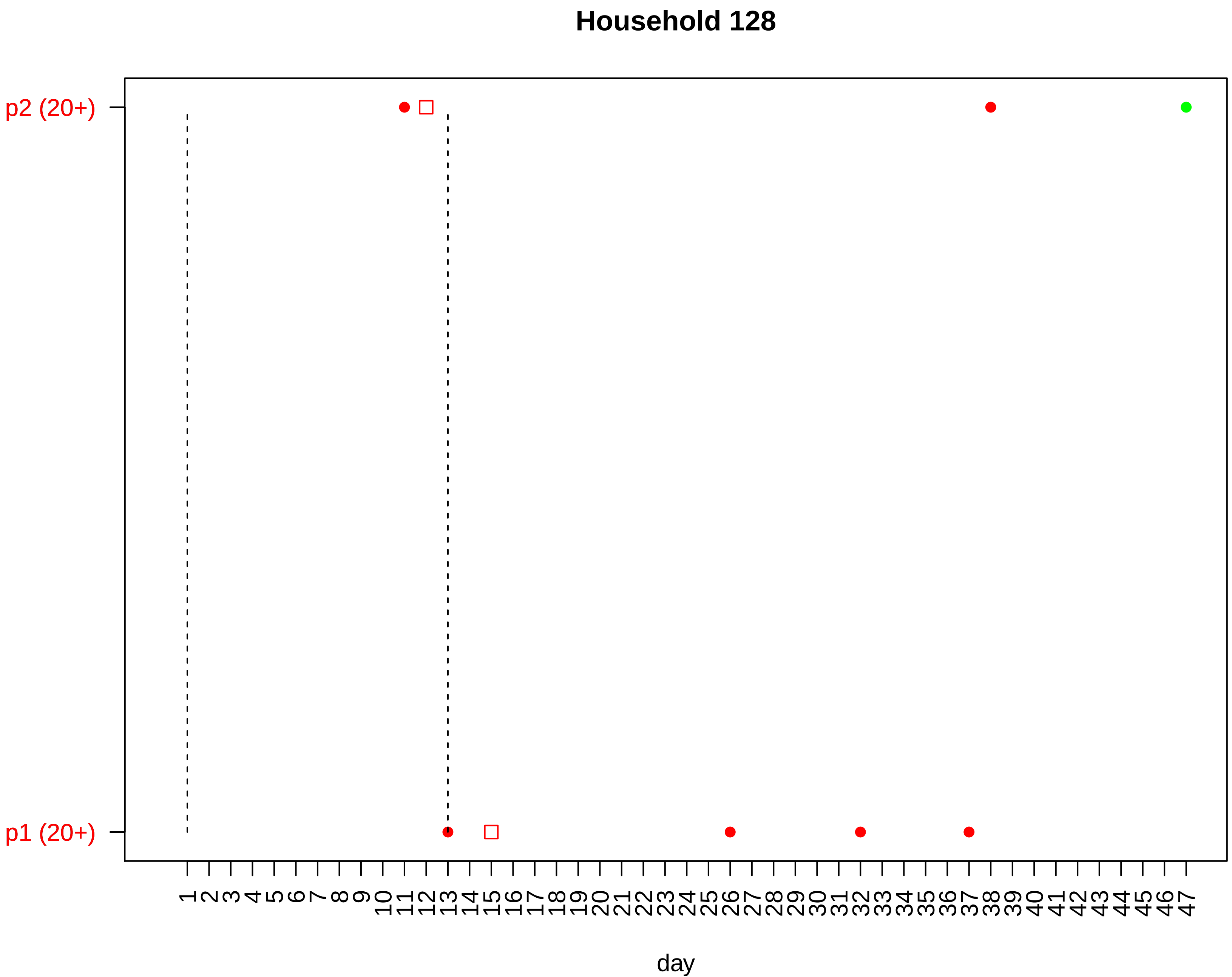

Household 128
p2 (20+)
p1 (20+)
1 2 3 4 5 6 7 8 9 10 11 12 13 14 15 16 17 18 19 20 21 22 23 24 25 26 27 28 29 30 31 32 33 34 35 36 37 38 39 40 41 42 43 44 45 46 47
day

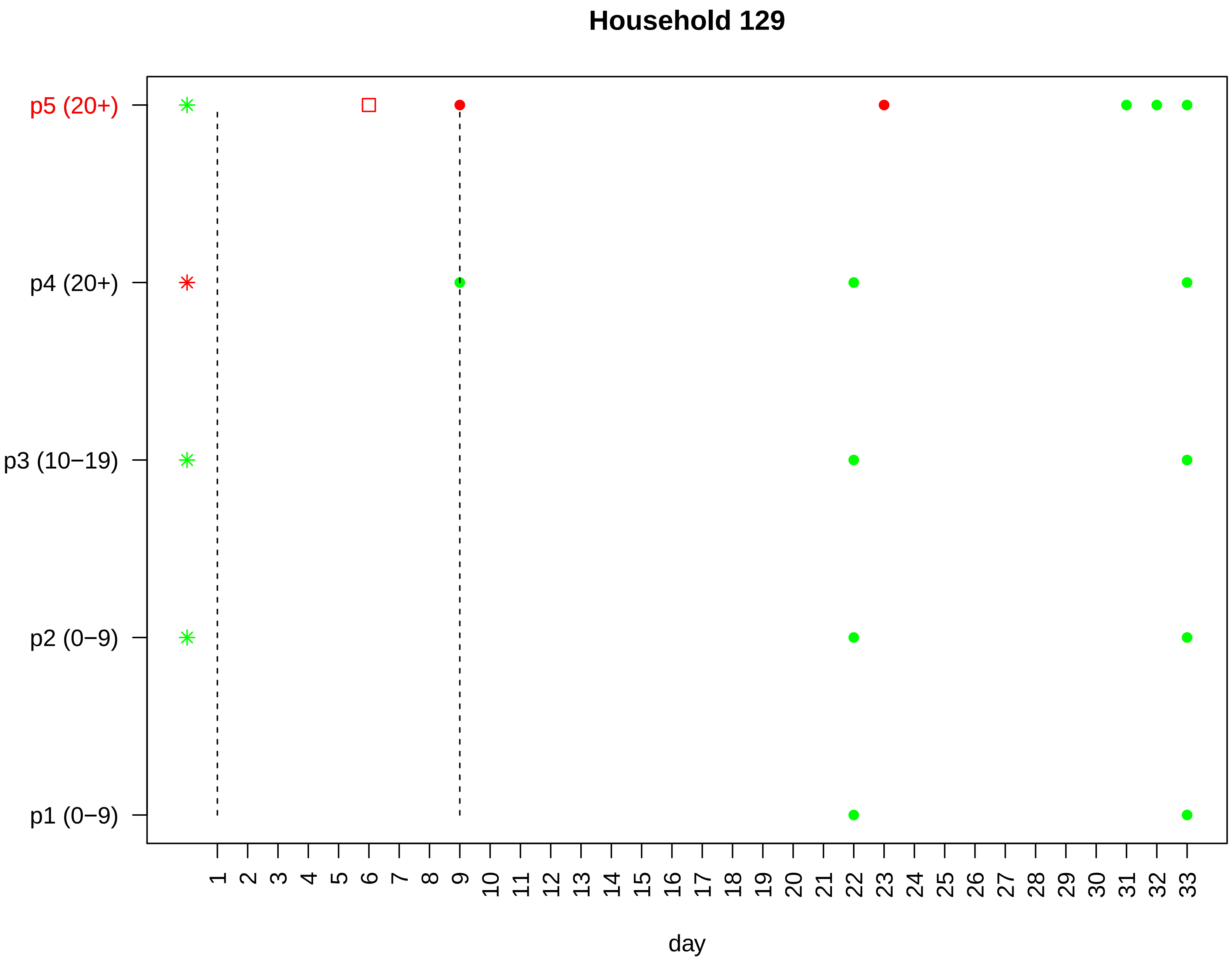

Household 129
p5 (20+)
p4 (20+)
p3 (10−19)
p2 (0−9)
p1 (0−9)
1 2 3 4 5 6 7 8 9 10 11 12 13 14 15 16 17 18 19 20 21 22 23 24 25 26 27 28 29 30 31 32 33
day

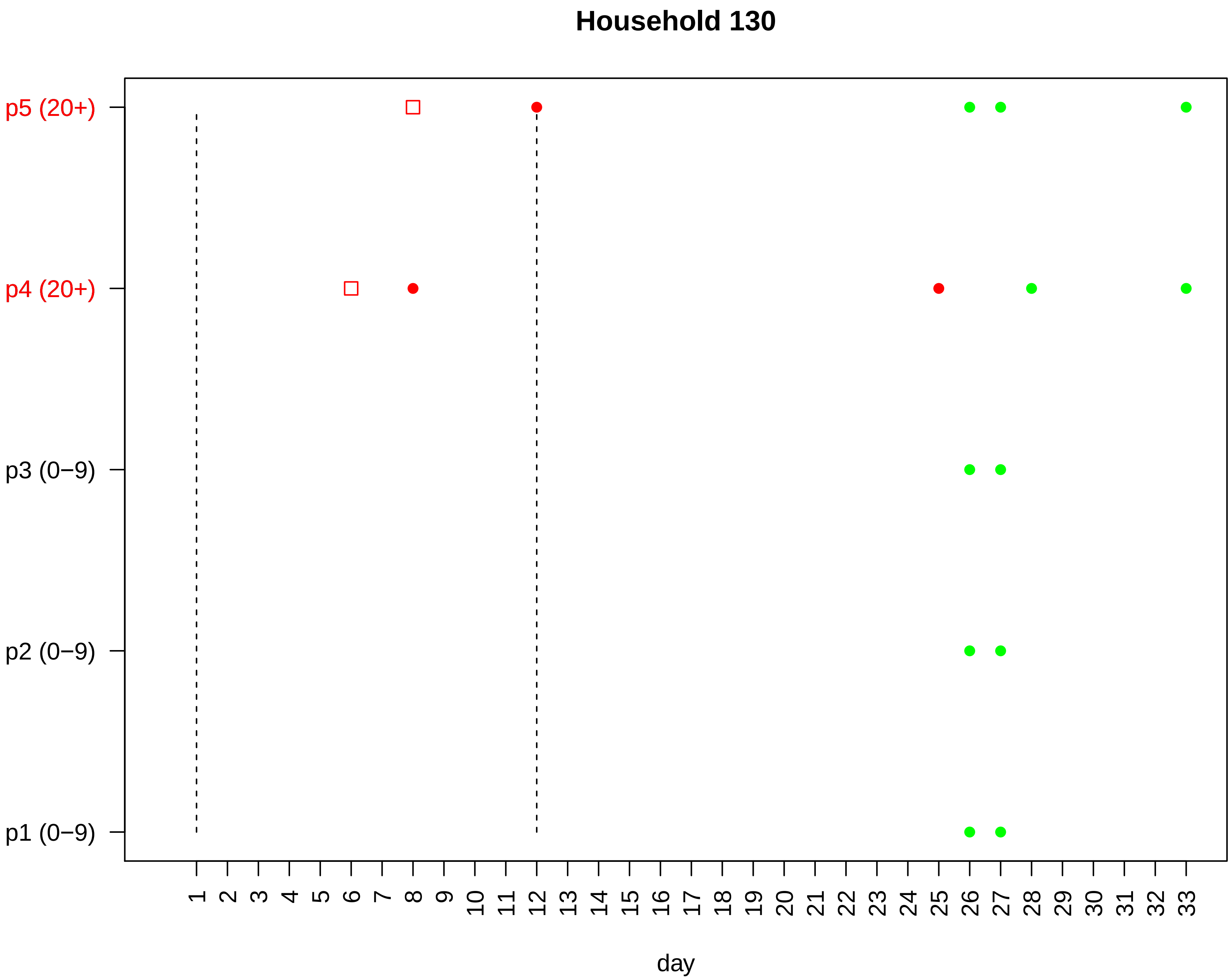

Household 130
p5 (20+)
p4 (20+)
p3 (0−9)
p2 (0−9)
p1 (0−9)
1 2 3 4 5 6 7 8 9 10 11 12 13 14 15 16 17 18 19 20 21 22 23 24 25 26 27 28 29 30 31 32 33
day

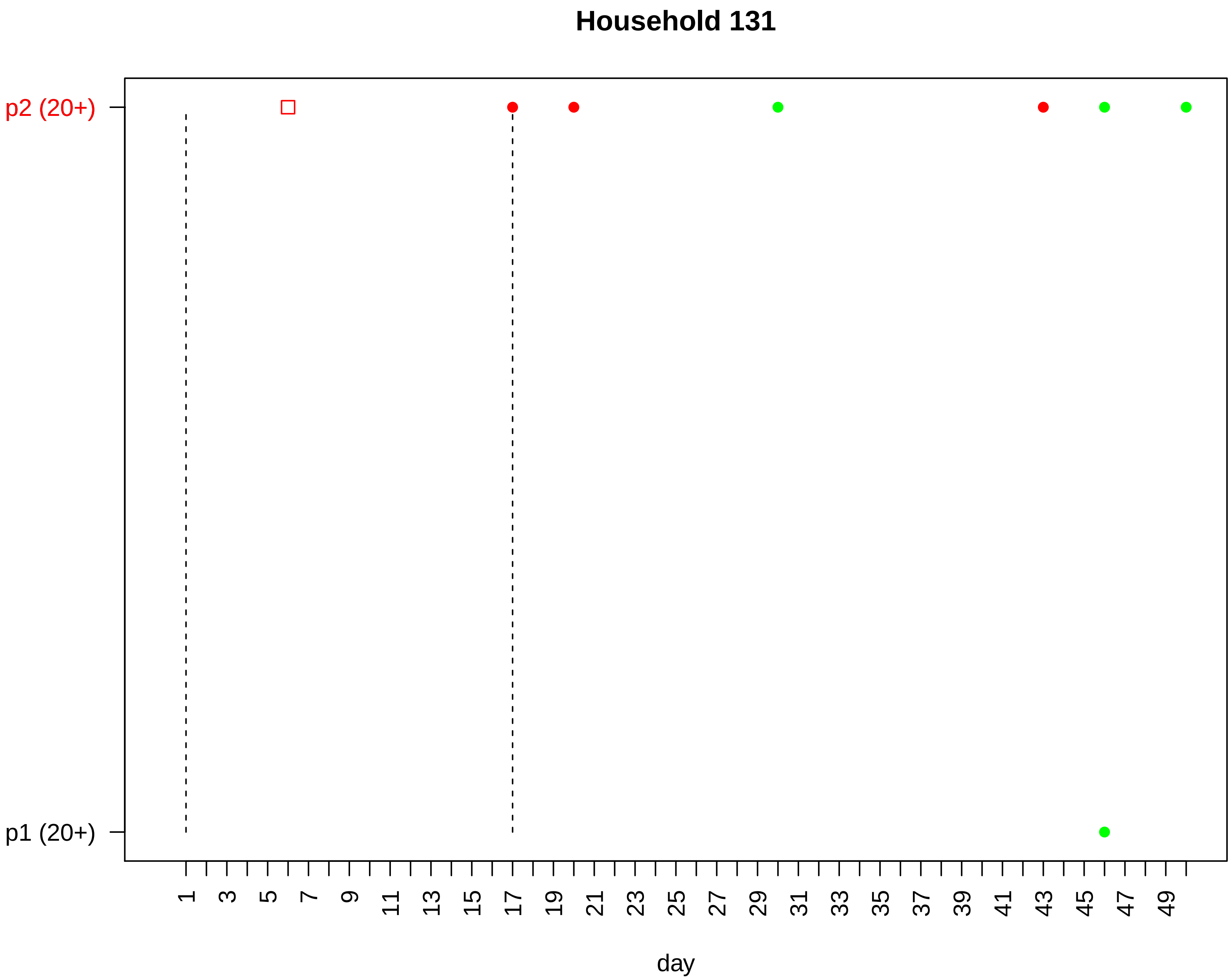
Household 131
p2 (20+)
p1 (20+)
1 3 5 7 9 11 13 15 17 19 21 23 25 27 29 31 33 35 37 39 41 43 45 47 49
day

Household 132

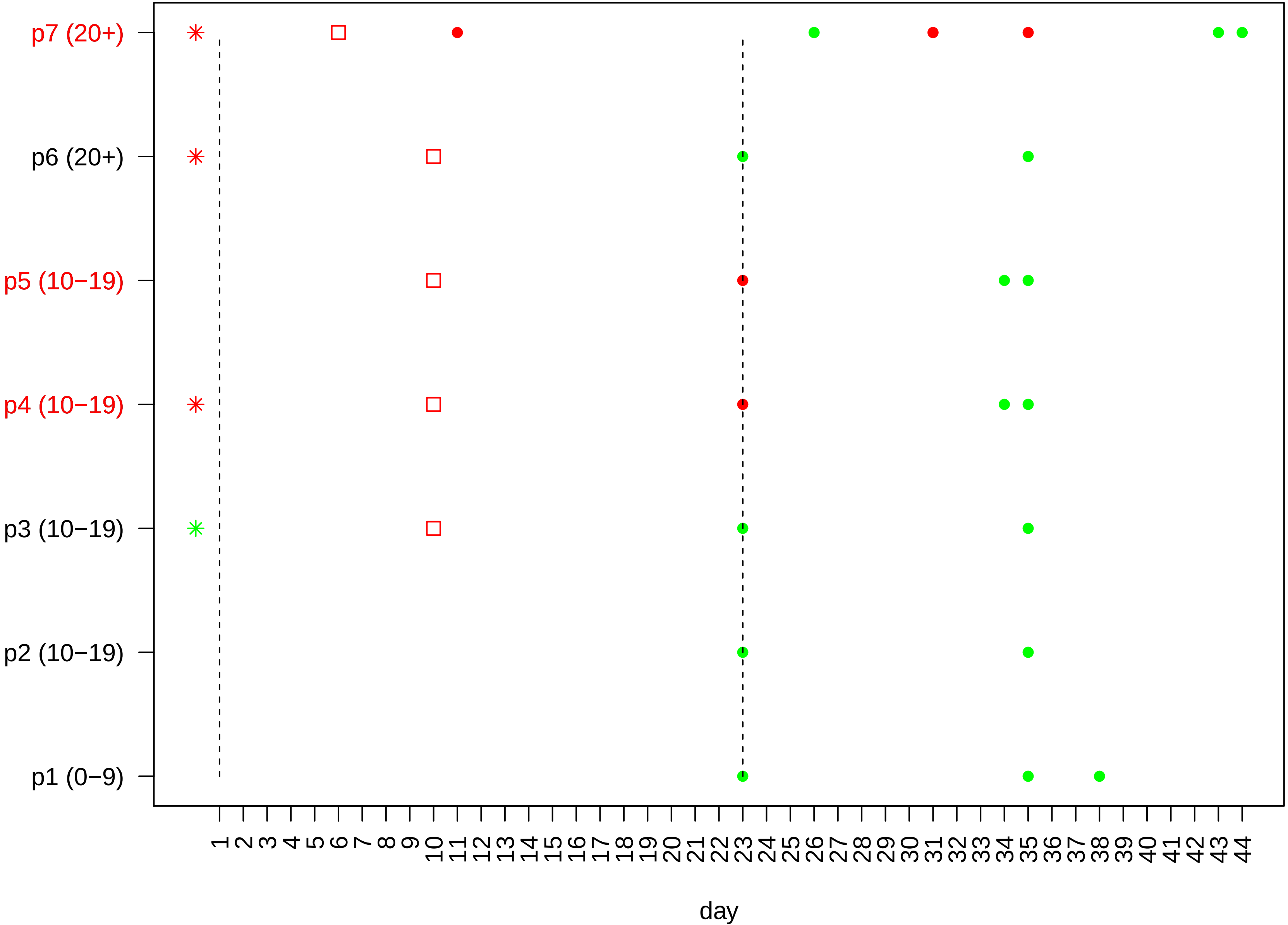

Household 133

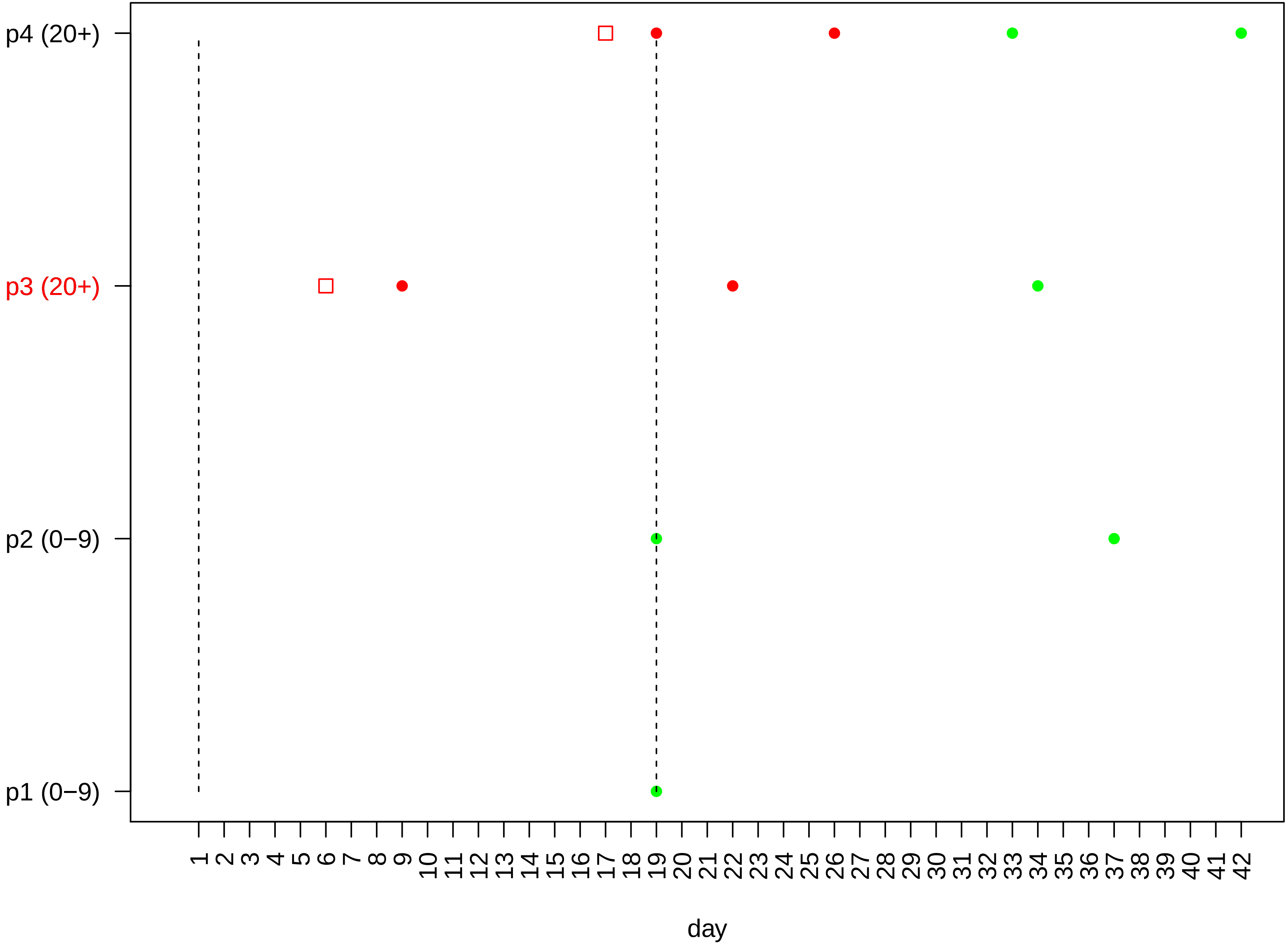

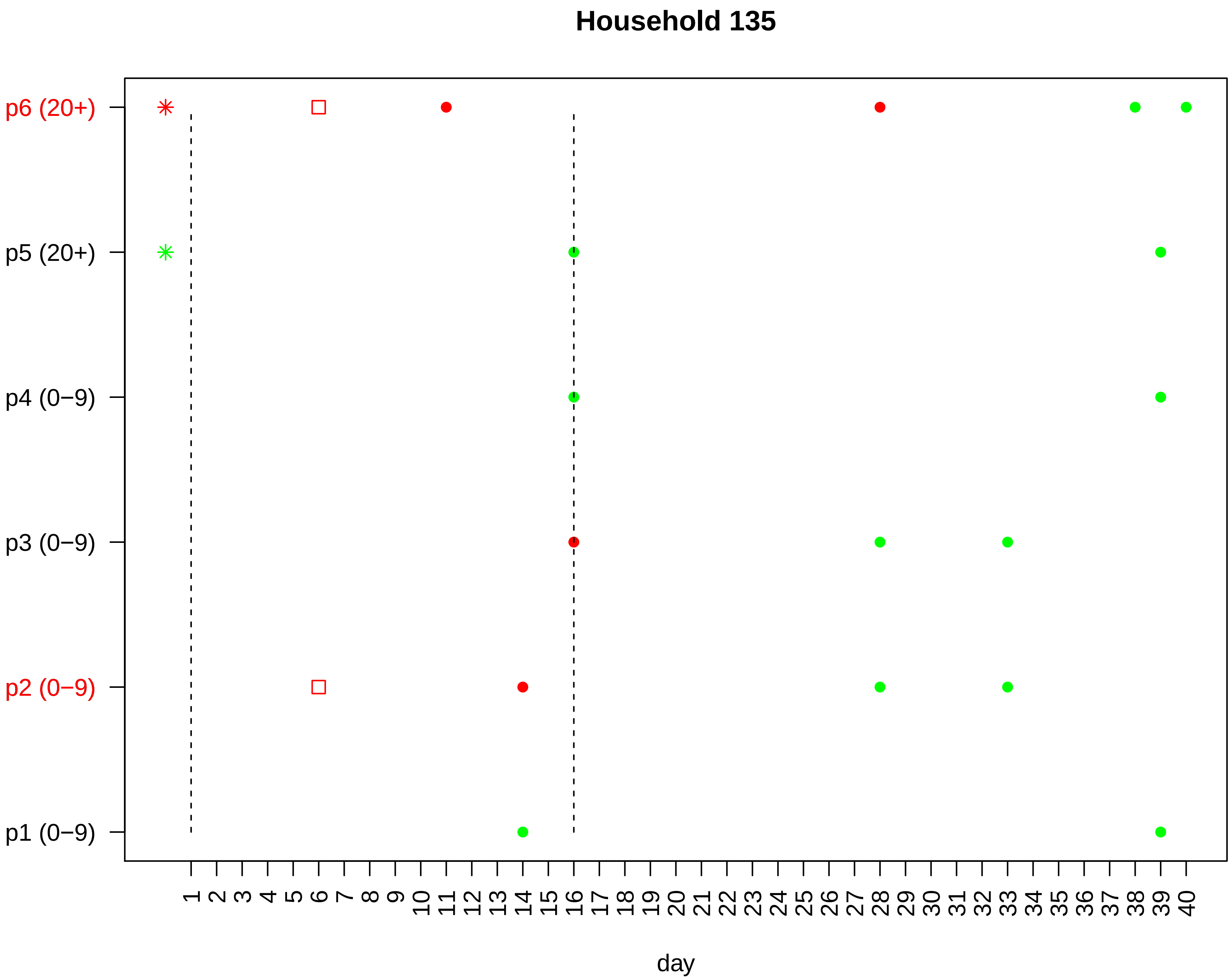

Household 135
p6 (20+)
p5 (20+)
p4 (0−9)
p3 (0−9)
p2 (0−9)
p1 (0−9)
1 2 3 4 5 6 7 8 9 10 11 12 13 14 15 16 17 18 19 20 21 22 23 24 25 26 27 28 29 30 31 32 33 34 35 36 37 38 39 40
day

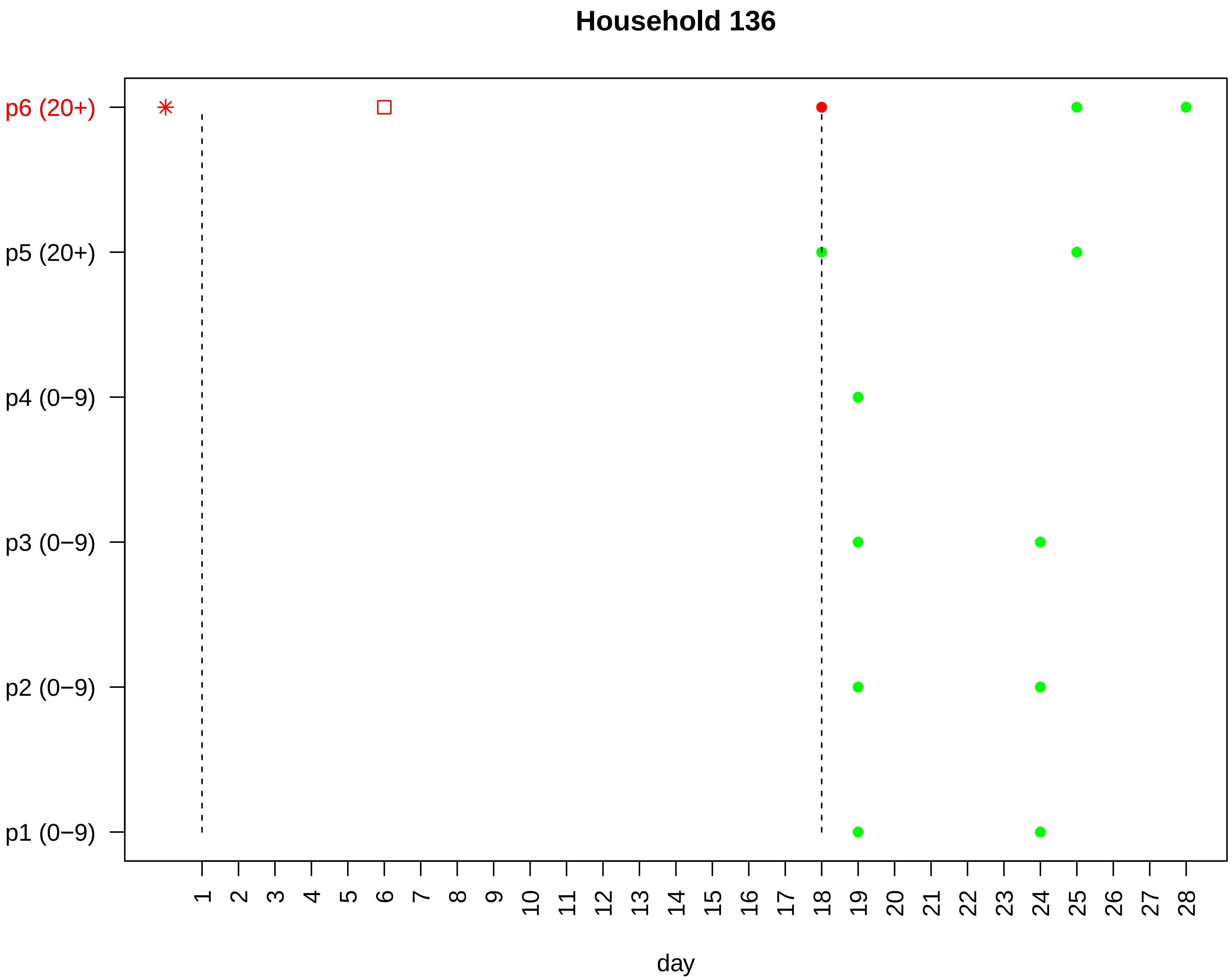

Household 136
p6 (20+)
p5 (20+)
p4 (0−9)
p3 (0−9)
p2 (0−9)
p1 (0−9)
1 2 3 4 5 6 7 8 9 10 11 12 13 14 15 16 17 18 19 20 21 22 23 24 25 26 27 28
day

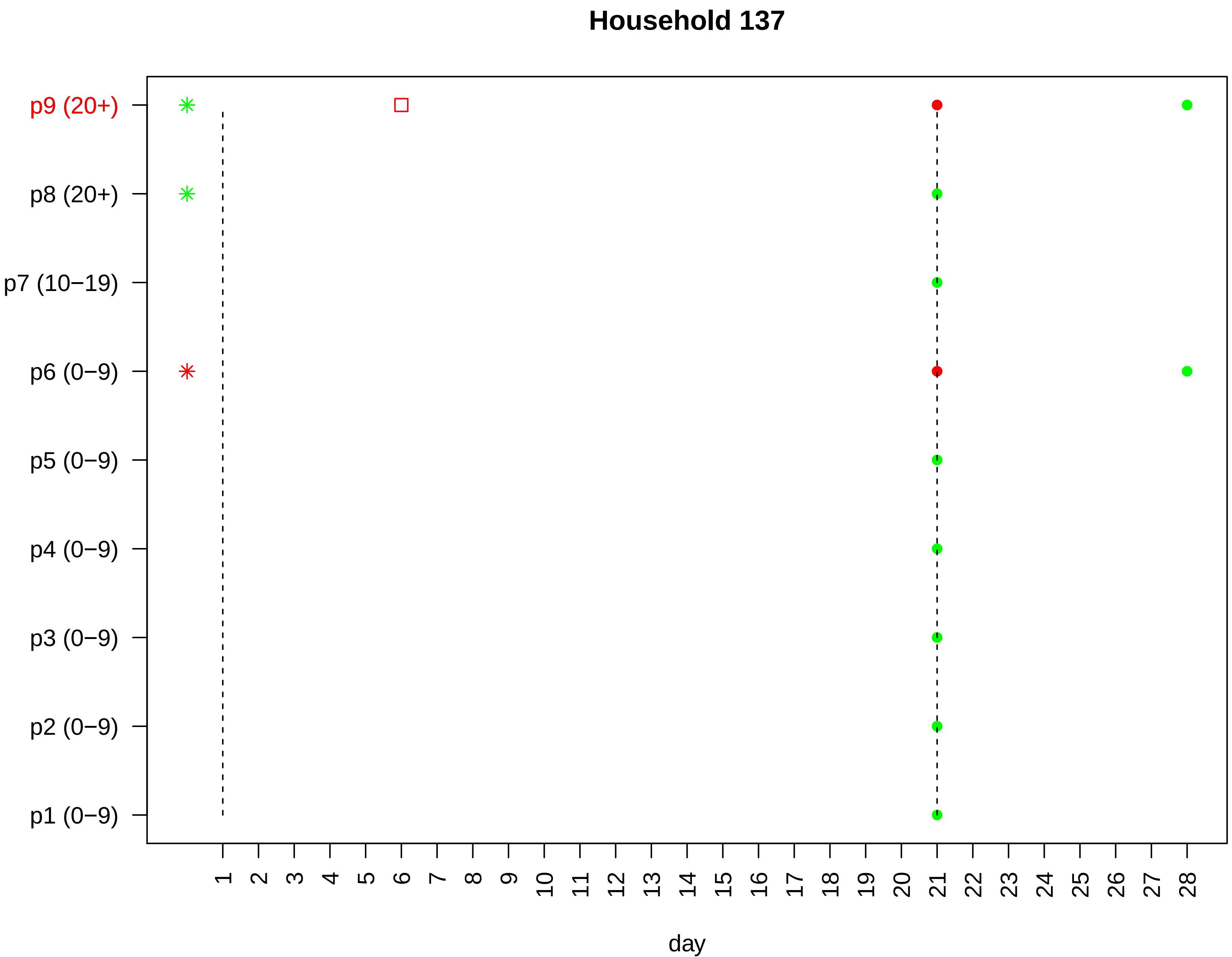

Household 137
p9 (20+)
p8 (20+)
p7 (10−19)
p6 (0−9)
p5 (0−9)
p4 (0−9)
p3 (0−9)
p2 (0−9)
p1 (0−9)
1 2 3 4 5 6 7 8 9 10 11 12 13 14 15 16 17 18 19 20 21 22 23 24 25 26 27 28
day

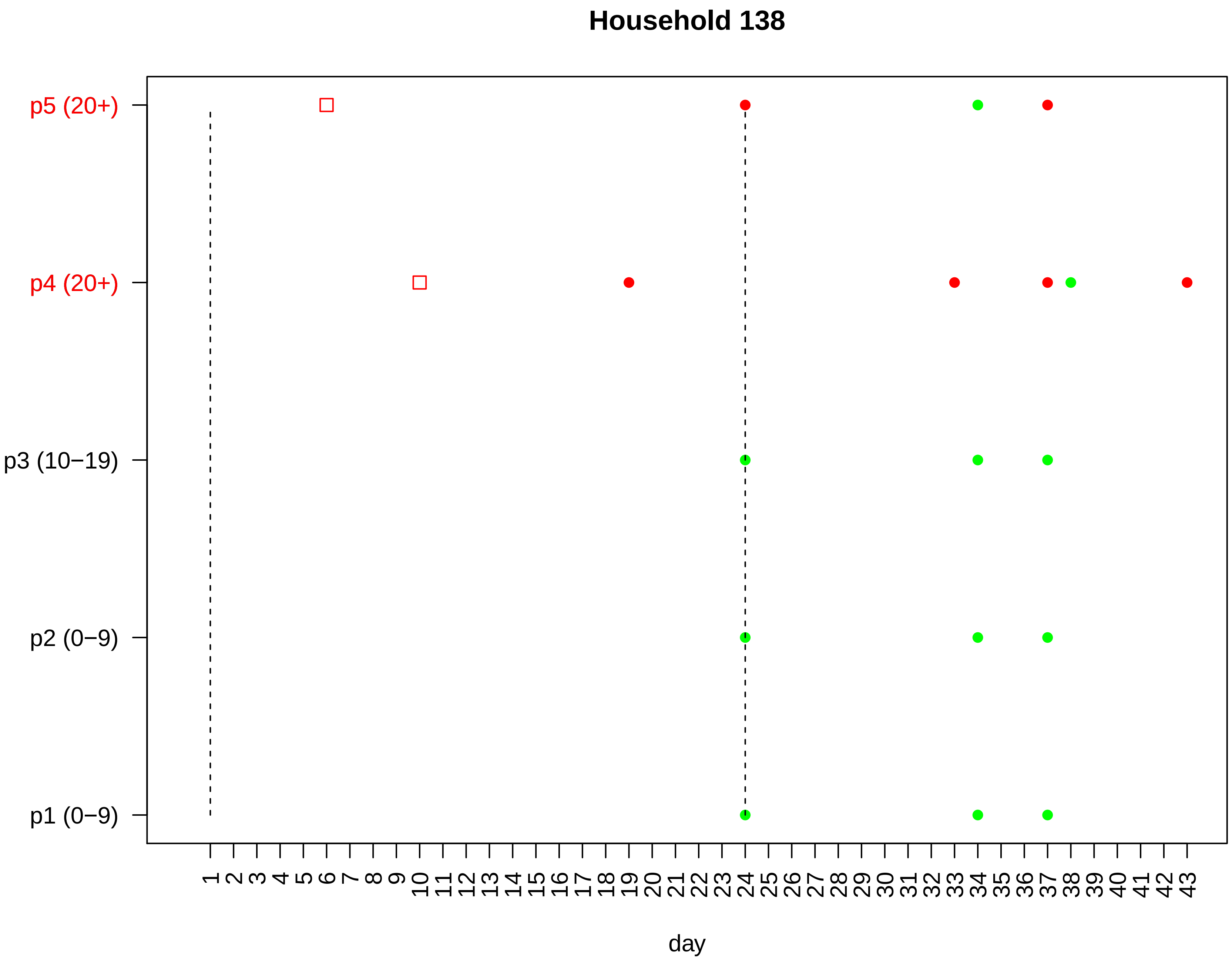

Household 138
p5 (20+)
p4 (20+)
p3 (10−19)
p2 (0−9)
p1 (0−9)
1 2 3 4 5 6 7 8 9 10 11 12 13 14 15 16 17 18 19 20 21 22 23 24 25 26 27 28 29 30 31 32 33 34 35 36 37 38 39 40 41 42 43
day

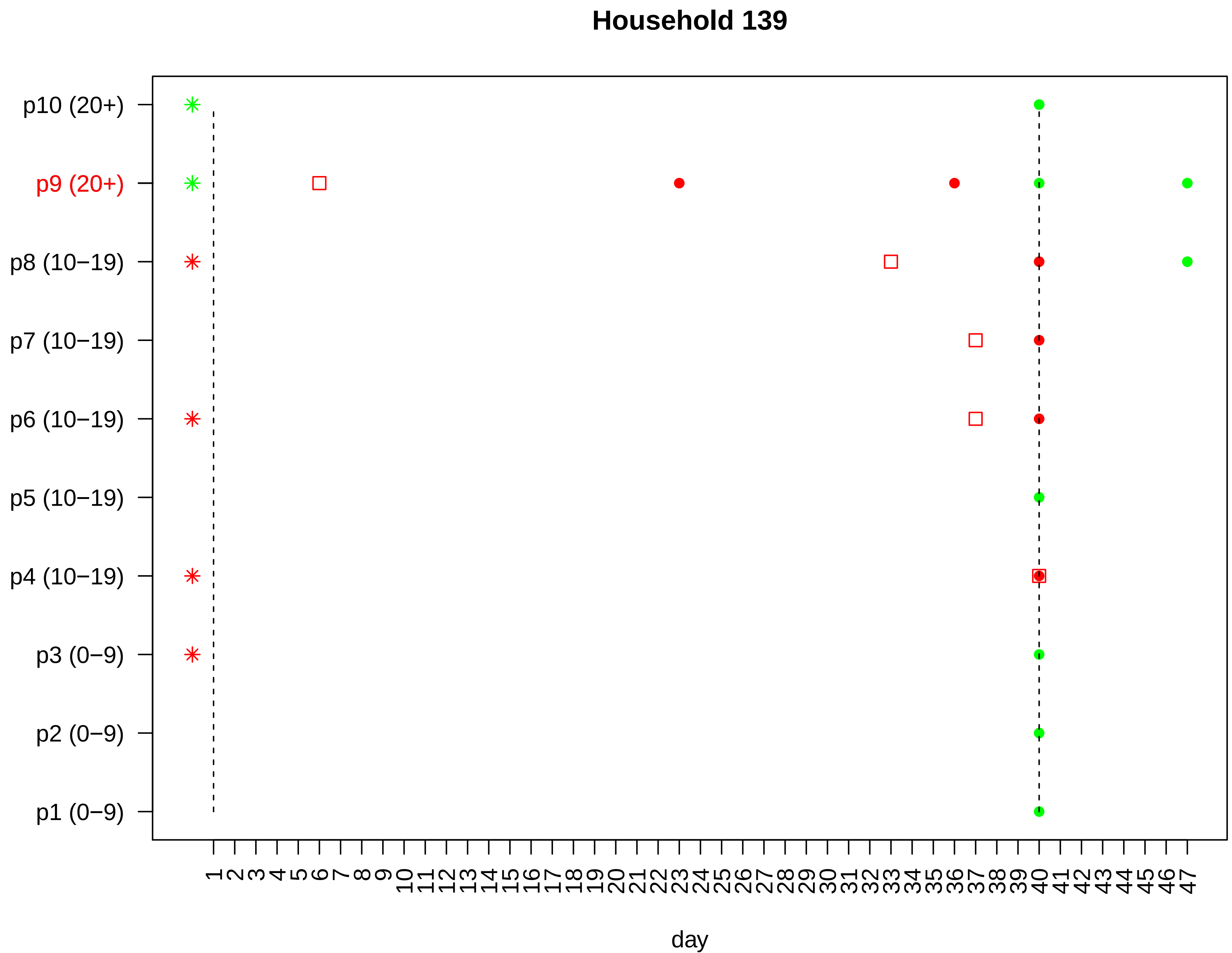

Household 139
p10 (20+)
p9 (20+)
p8 (10−19)
p7 (10−19)
p6 (10−19)
p5 (10−19)
p4 (10−19)
p3 (0−9)
p2 (0−9)
p1 (0−9)
1 2 3 4 5 6 7 8 9 10 11 12 13 14 15 16 17 18 19 20 21 22 23 24 25 26 27 28 29 30 31 32 33 34 35 36 37 38 39 40 41 42 43 44 45 46 47
day

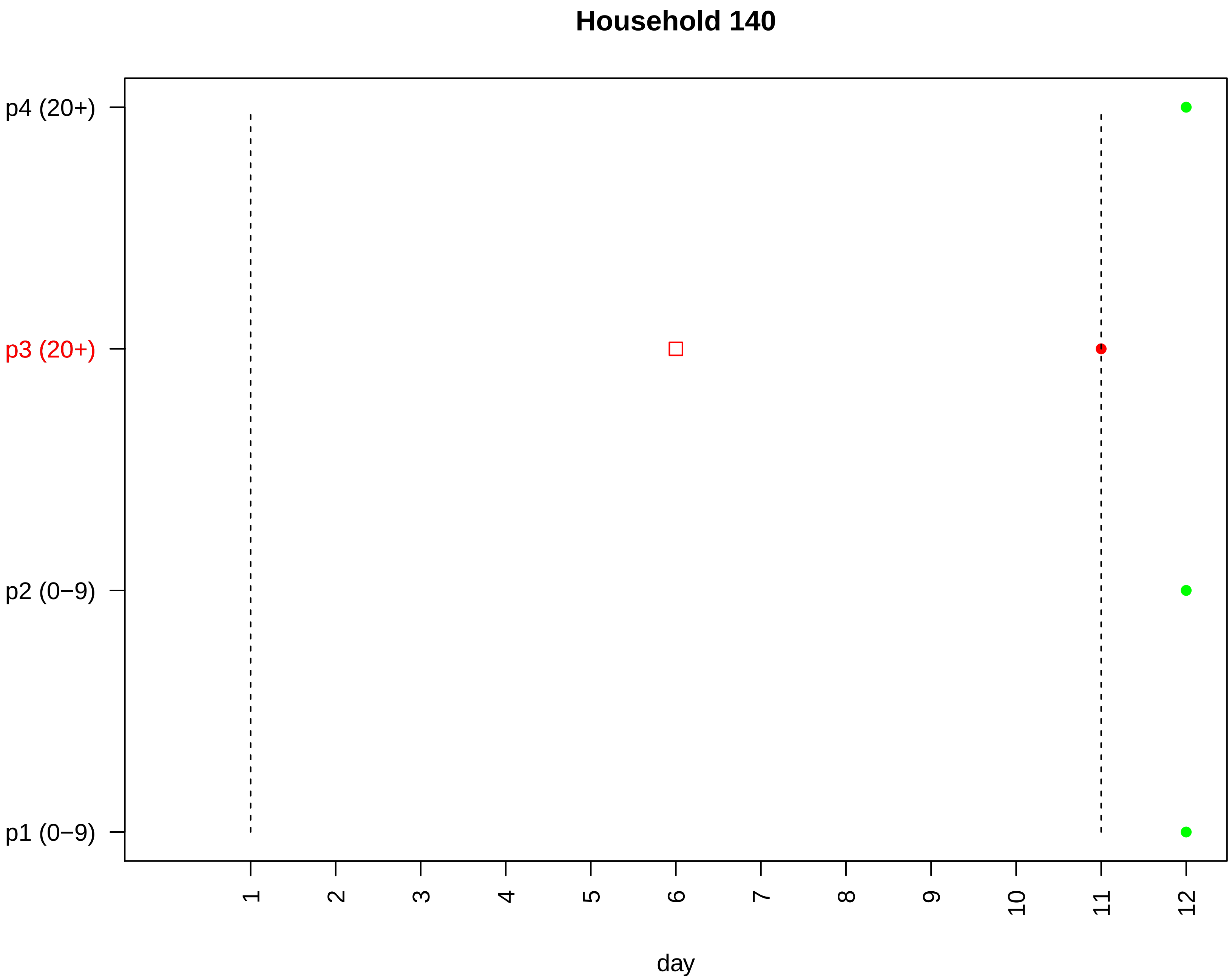

Household 140
p4 (20+)
p3 (20+)
p2 (0−9)
p1 (0−9)
1
2
3
4
5
6
7
8
9
10
11
12
day

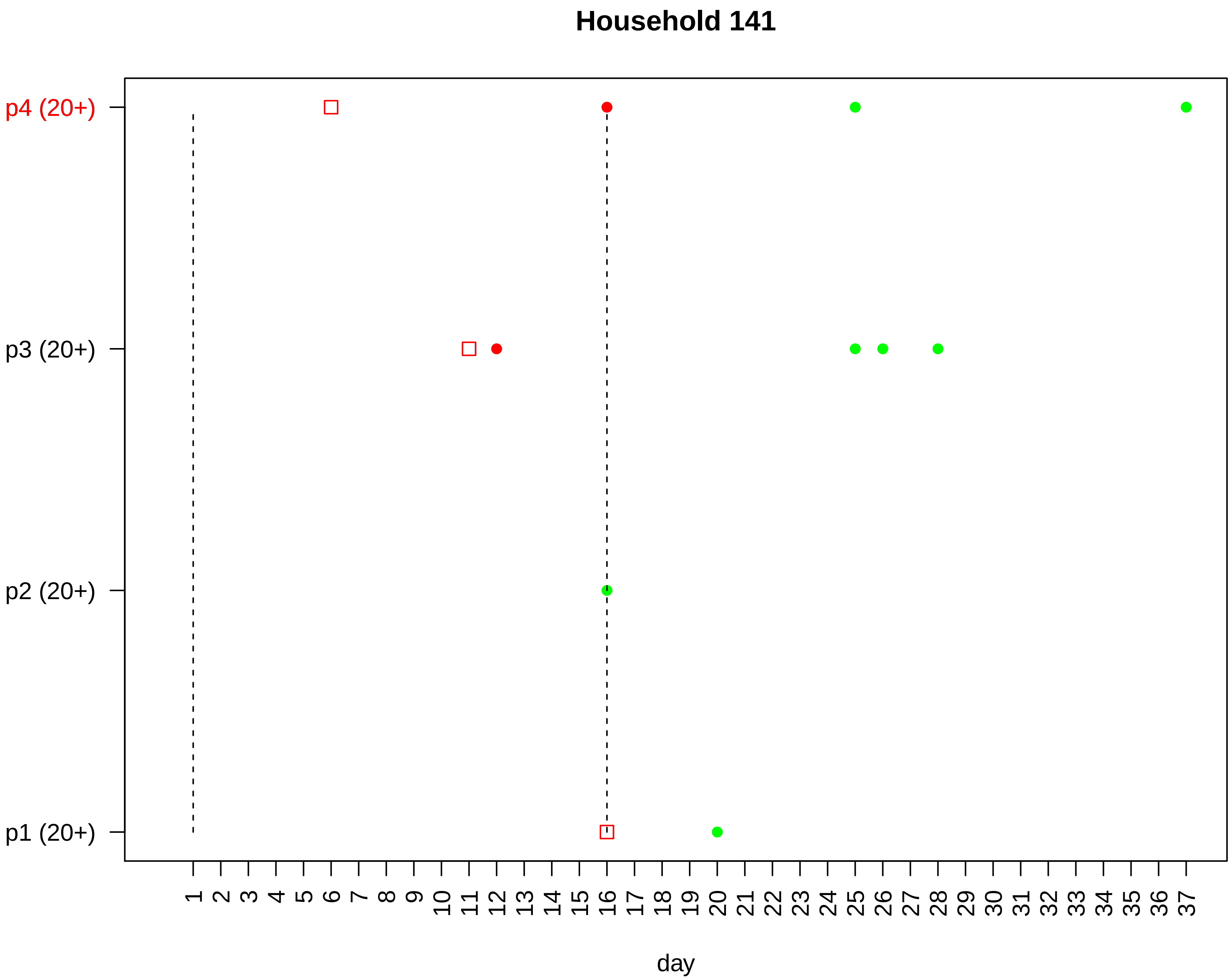

Household 141
p4 (20+)
p3 (20+)
p2 (20+)
p1 (20+)
1 2 3 4 5 6 7 8 9 10 11 12 13 14 15 16 17 18 19 20 21 22 23 24 25 26 27 28 29 30 31 32 33 34 35 36 37
day

Household 142

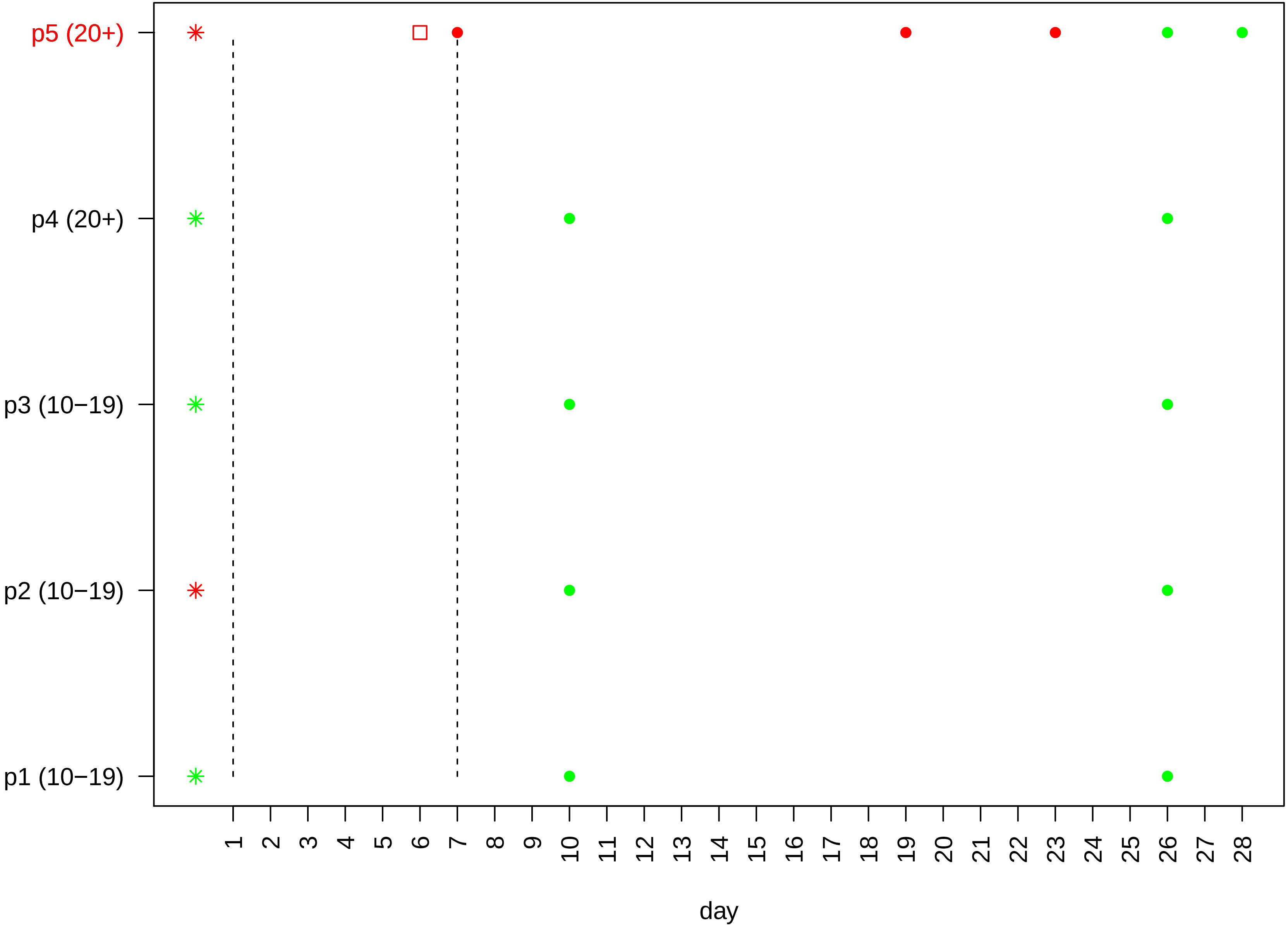

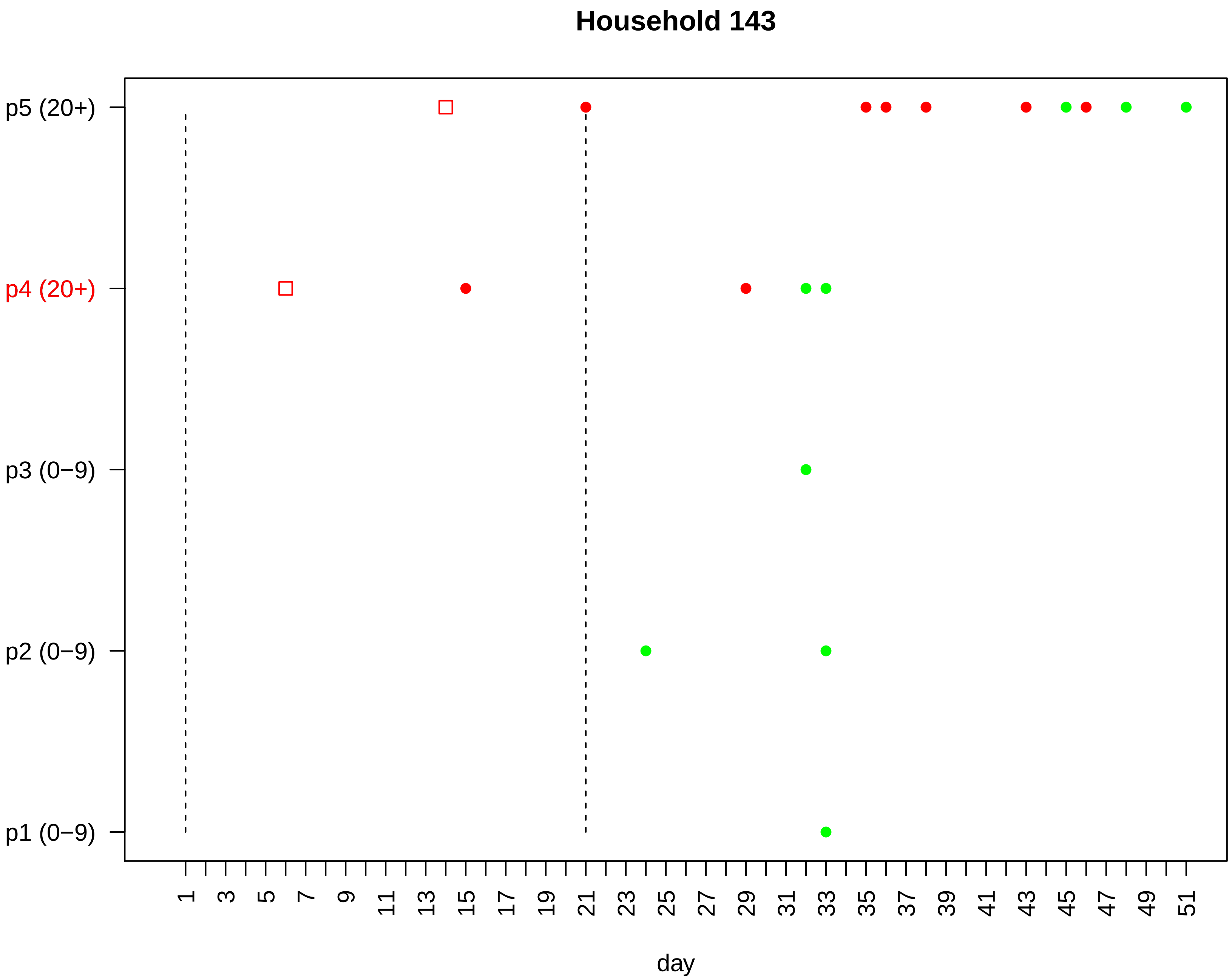

Household 143
p5 (20+)
p4 (20+)
p3 (0−9)
p2 (0−9)
p1 (0−9)
1
3
5
7
9
11
13
15
17
19
21
23
25
27
29
31
33
35
37
39
41
43
45
47
49
51
day

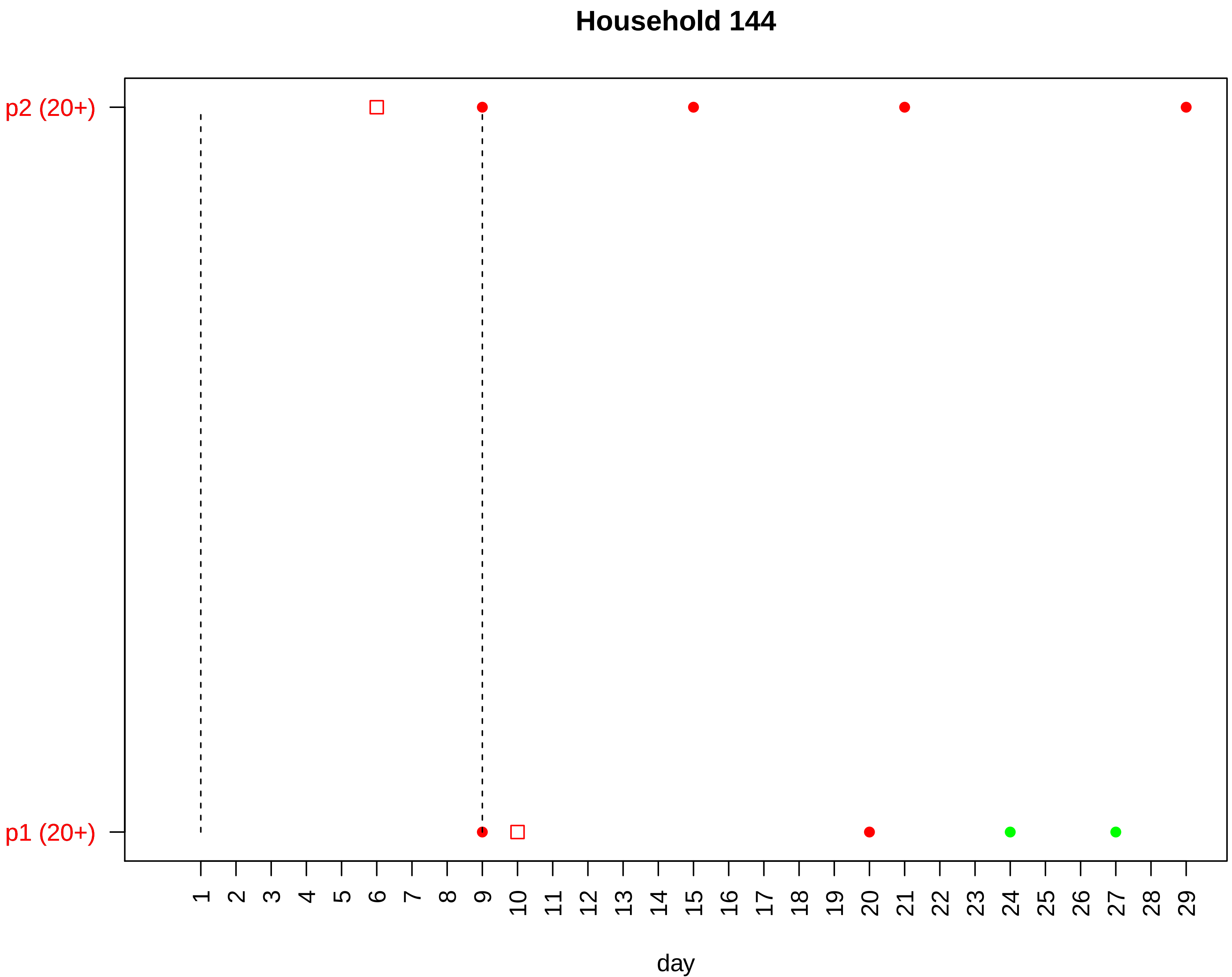

Household 144
p2 (20+)
p1 (20+)
1
2
3
4
5
6
7
8
9
10
11
12
13
14
15
16
17
18
19
20
21
22
23
24
25
26
27
28
29
day

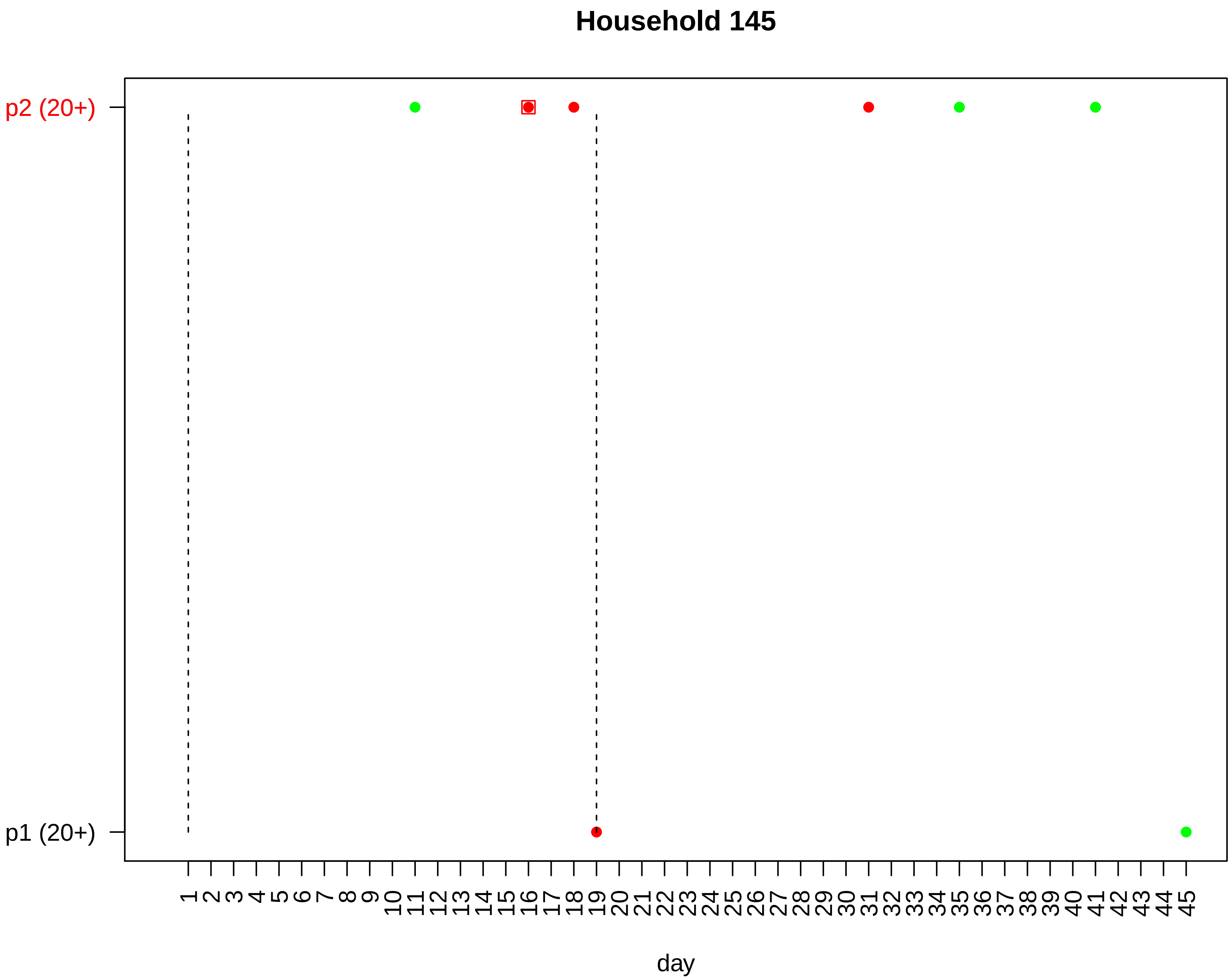

Household 145
p2 (20+)
p1 (20+)
1 2 3 4 5 6 7 8 9 10 11 12 13 14 15 16 17 18 19 20 21 22 23 24 25 26 27 28 29 30 31 32 33 34 35 36 37 38 39 40 41 42 43 44 45
day

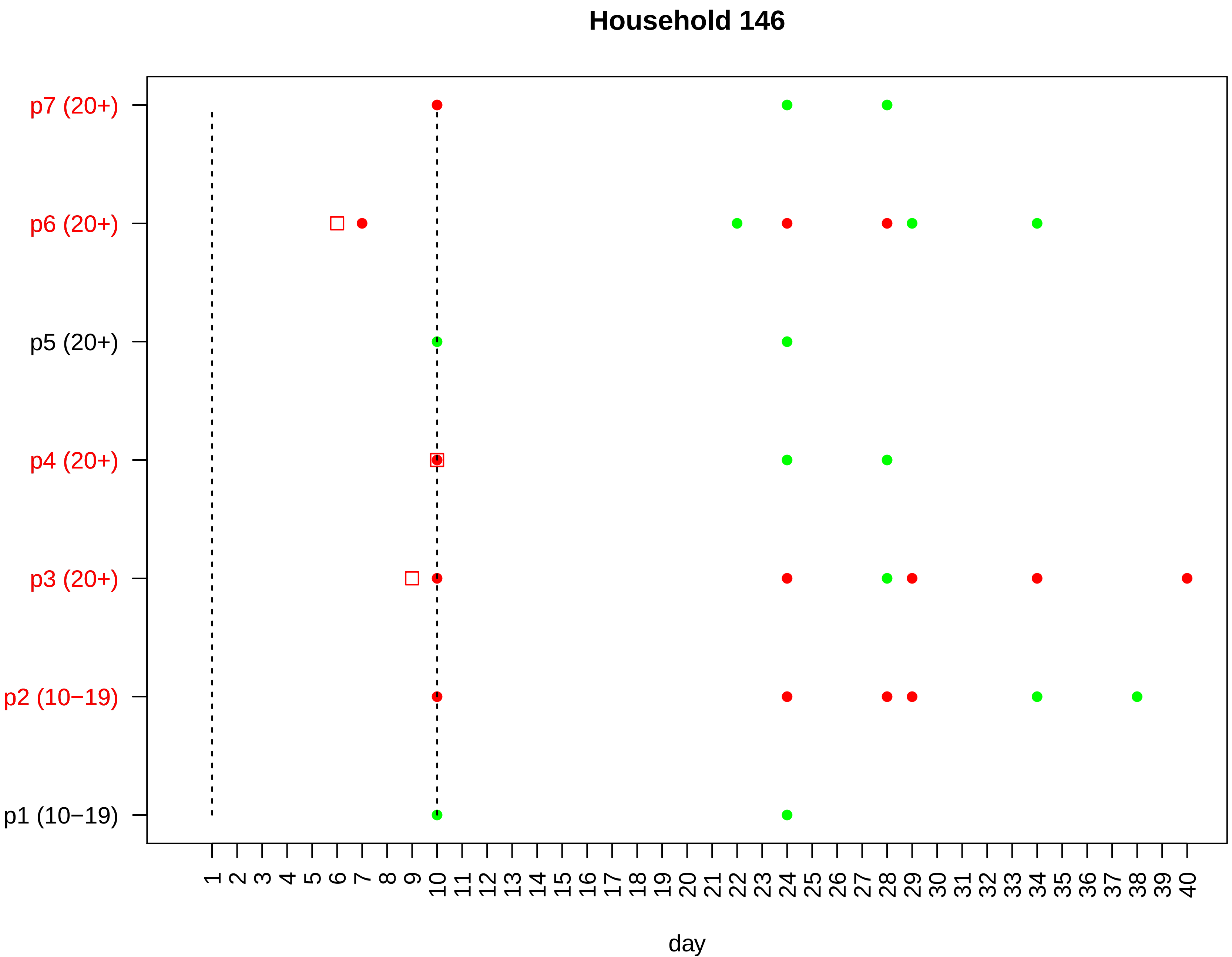

Household 146
p7 (20+)
p6 (20+)
p5 (20+)
p4 (20+)
p3 (20+)
p2 (10−19)
p1 (10−19)
1 2 3 4 5 6 7 8 9 10 11 12 13 14 15 16 17 18 19 20 21 22 23 24 25 26 27 28 29 30 31 32 33 34 35 36 37 38 39 40
day

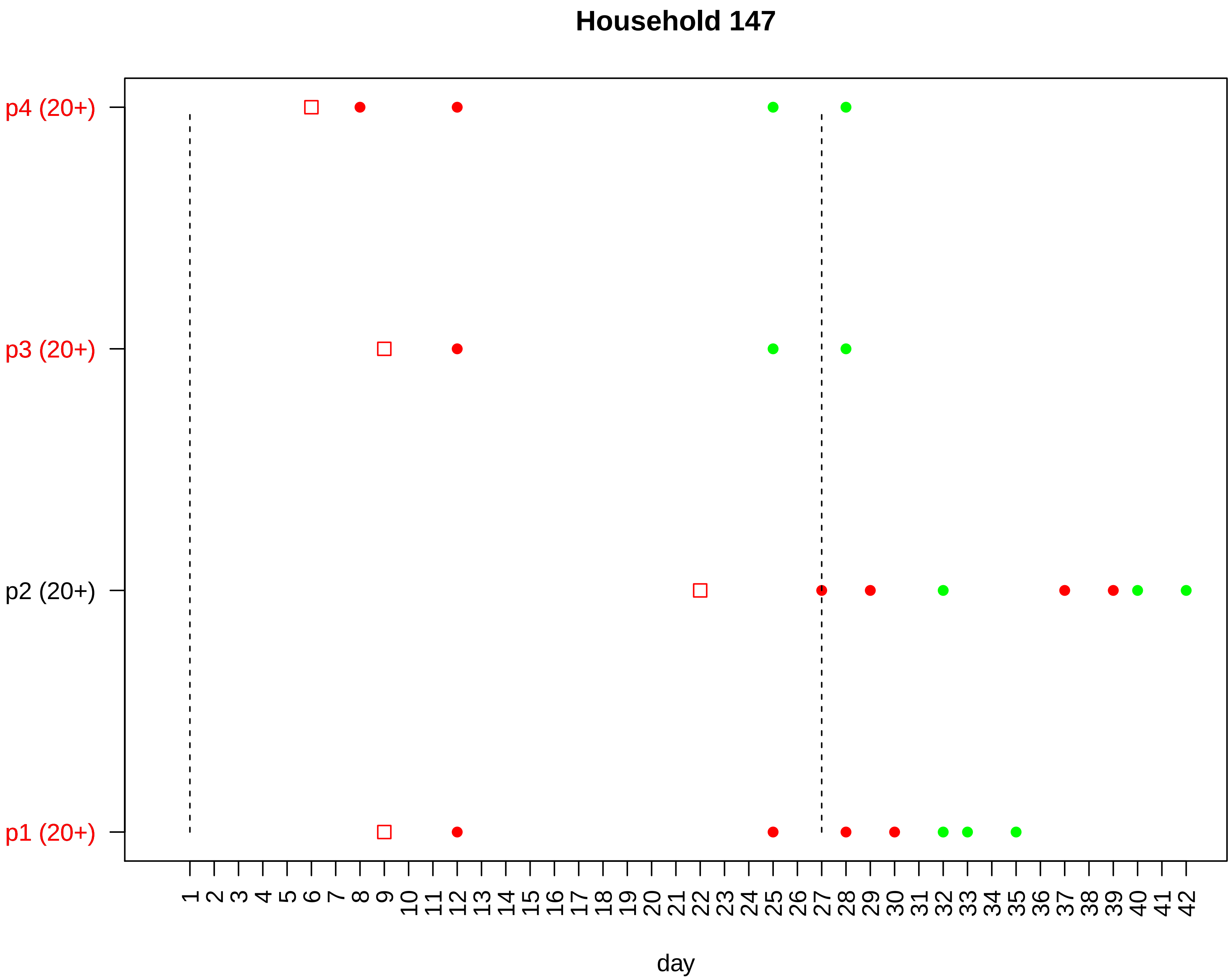

Household 147
p4 (20+)
p3 (20+)
p2 (20+)
p1 (20+)
1 2 3 4 5 6 7 8 9 10 11 12 13 14 15 16 17 18 19 20 21 22 23 24 25 26 27 28 29 30 31 32 33 34 35 36 37 38 39 40 41 42
day

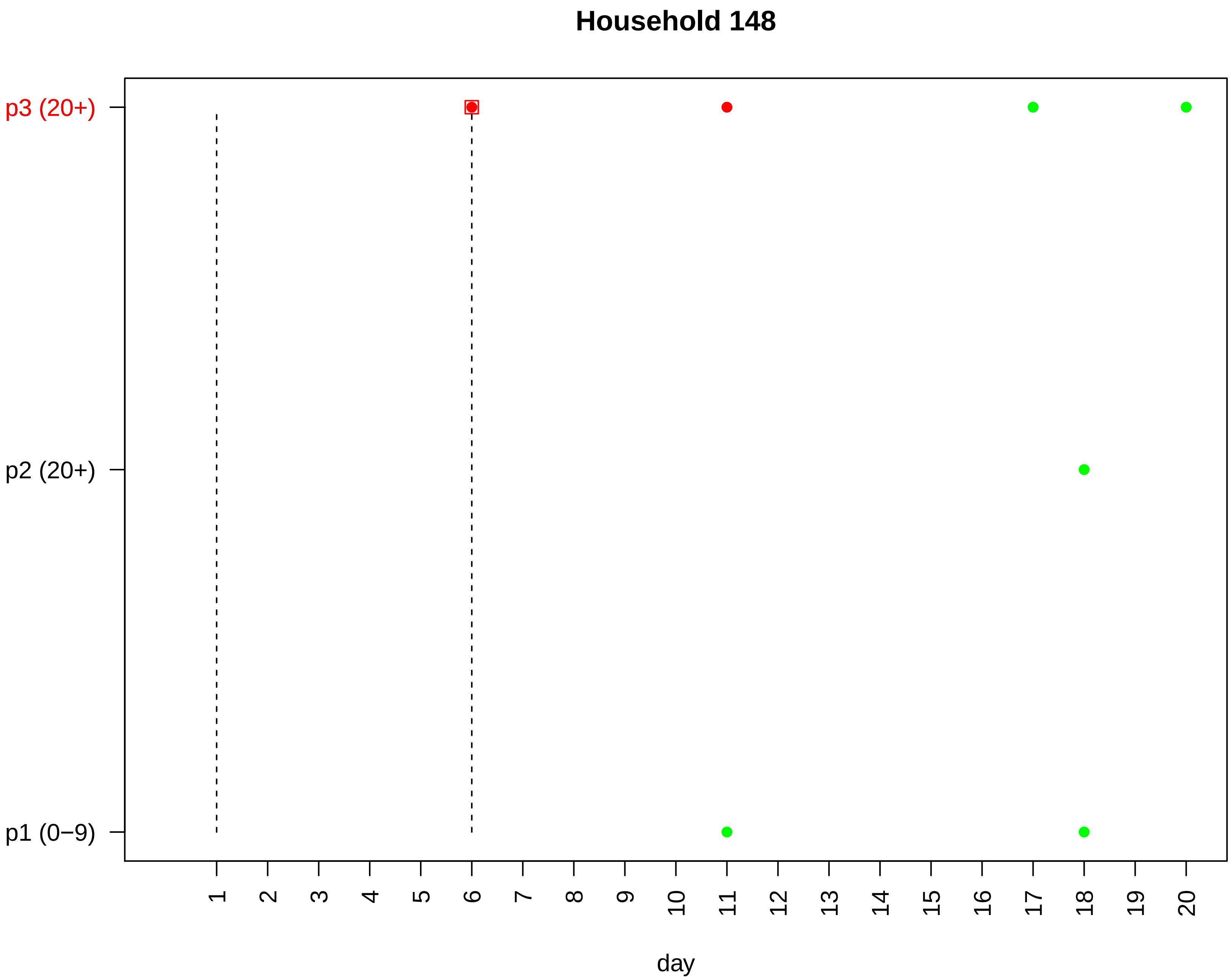

Household 148
p3 (20+)
p2 (20+)
p1 (0−9)
1
2
3
4
5
6
7
8
9
10
11
12
13
14
15
16
17
18
19
20
day

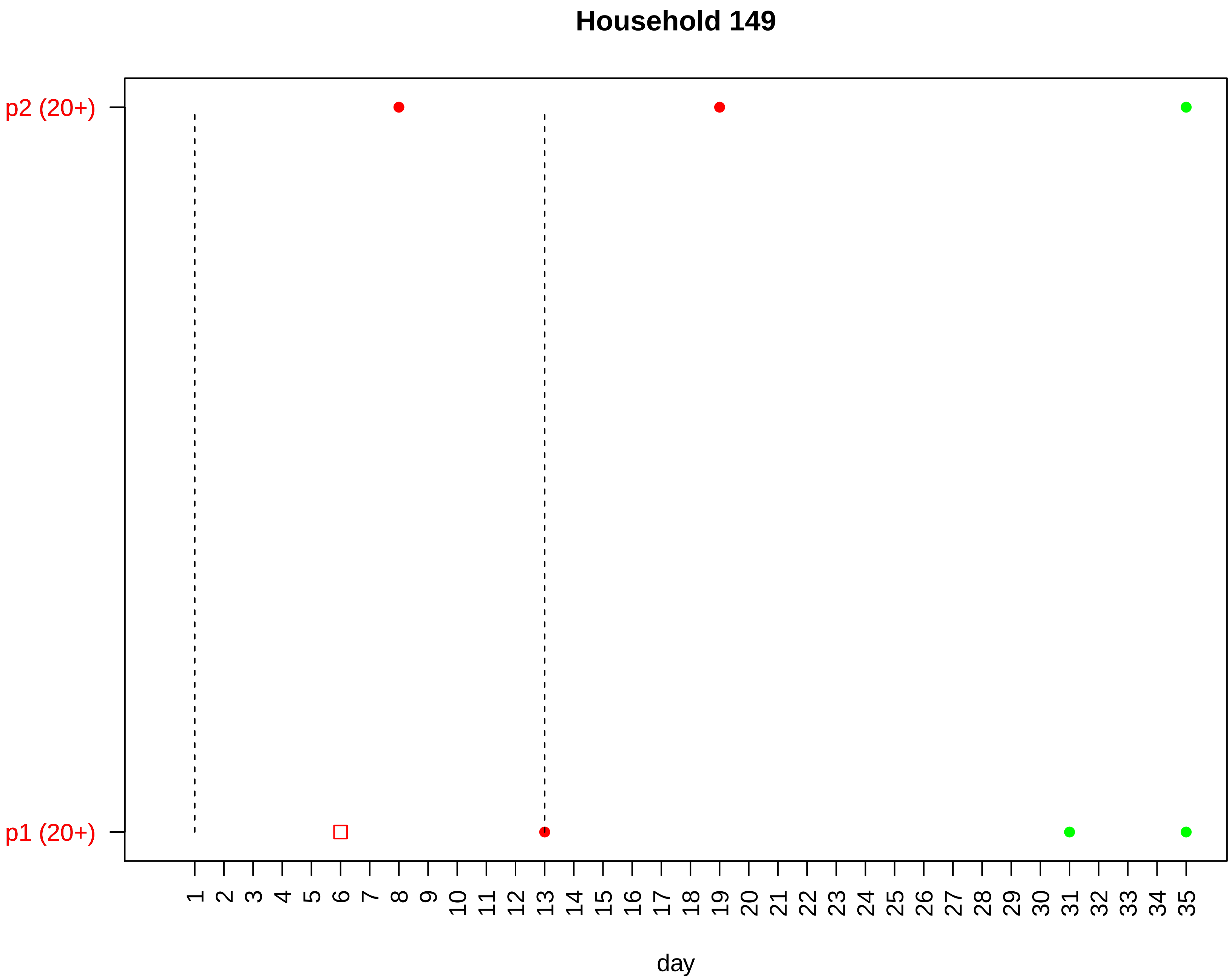
Household 149
p2 (20+)
p1 (20+)
1 2 3 4 5 6 7 8 9 10 11 12 13 14 15 16 17 18 19 20 21 22 23 24 25 26 27 28 29 30 31 32 33 34 35
day

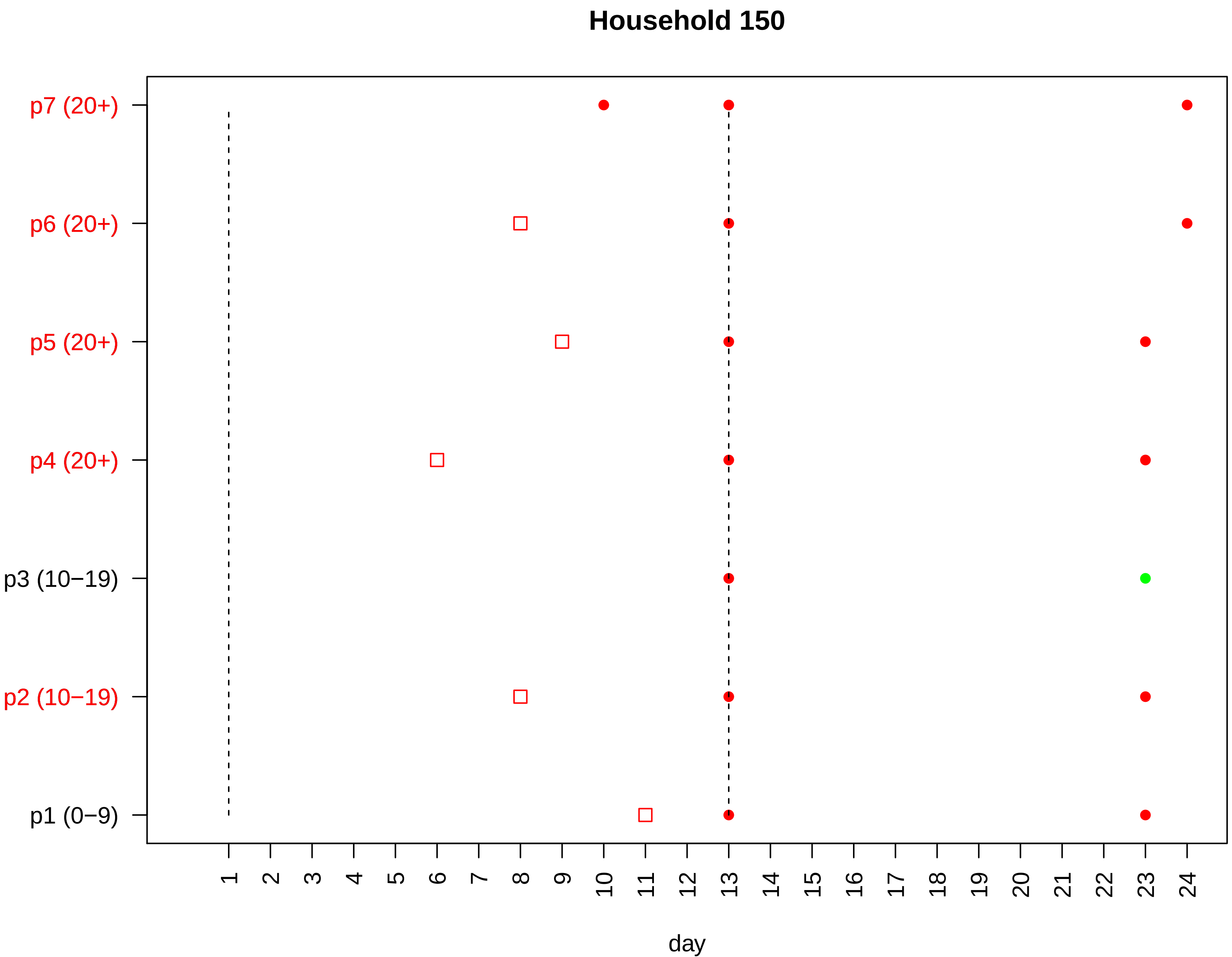

Household 150
p7 (20+)
p6 (20+)
p5 (20+)
p4 (20+)
p3 (10−19)
p2 (10−19)
p1 (0−9)
1 2 3 4 5 6 7 8 9 10 11 12 13 14 15 16 17 18 19 20 21 22 23 24
day

Household 151

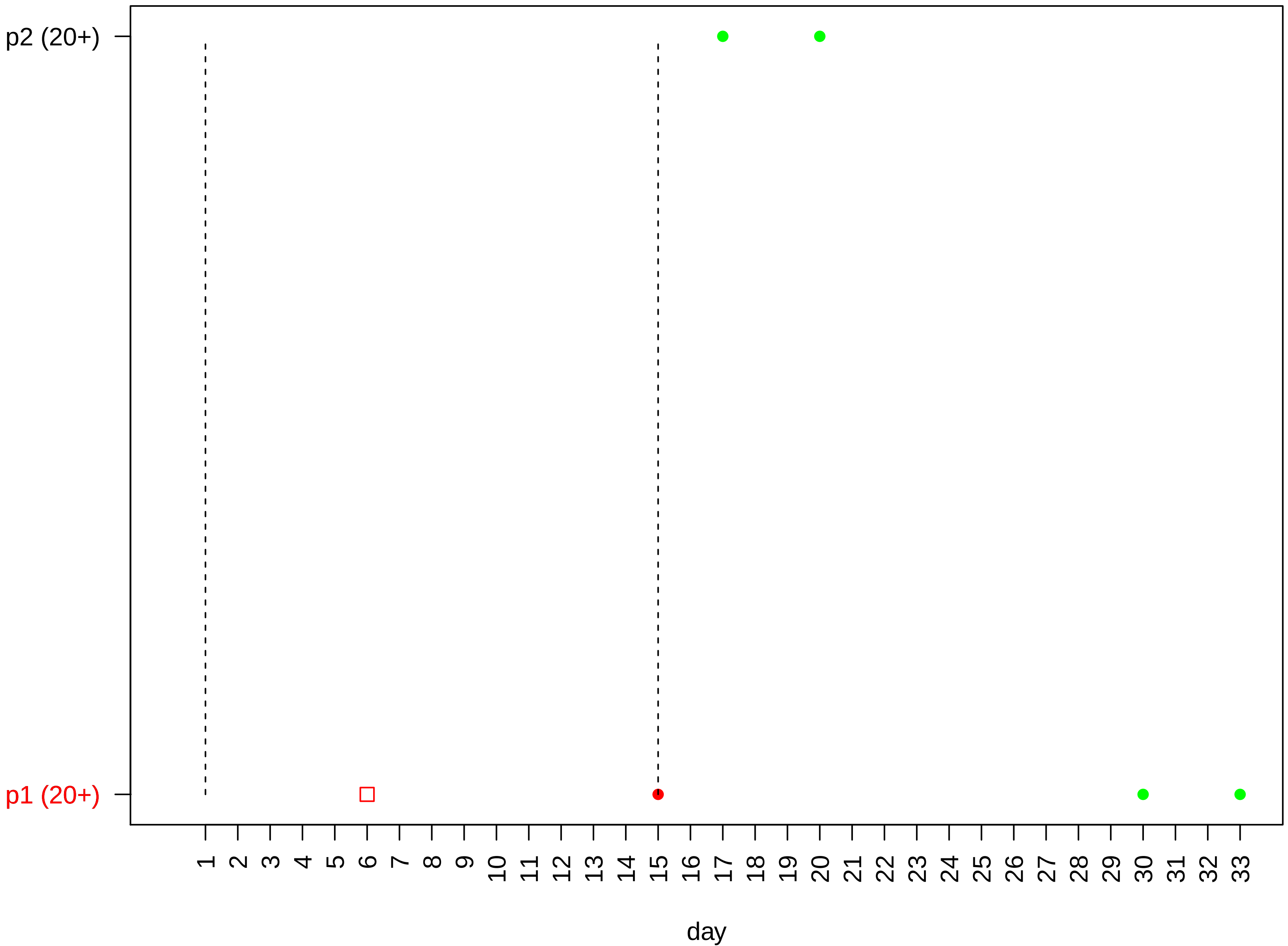

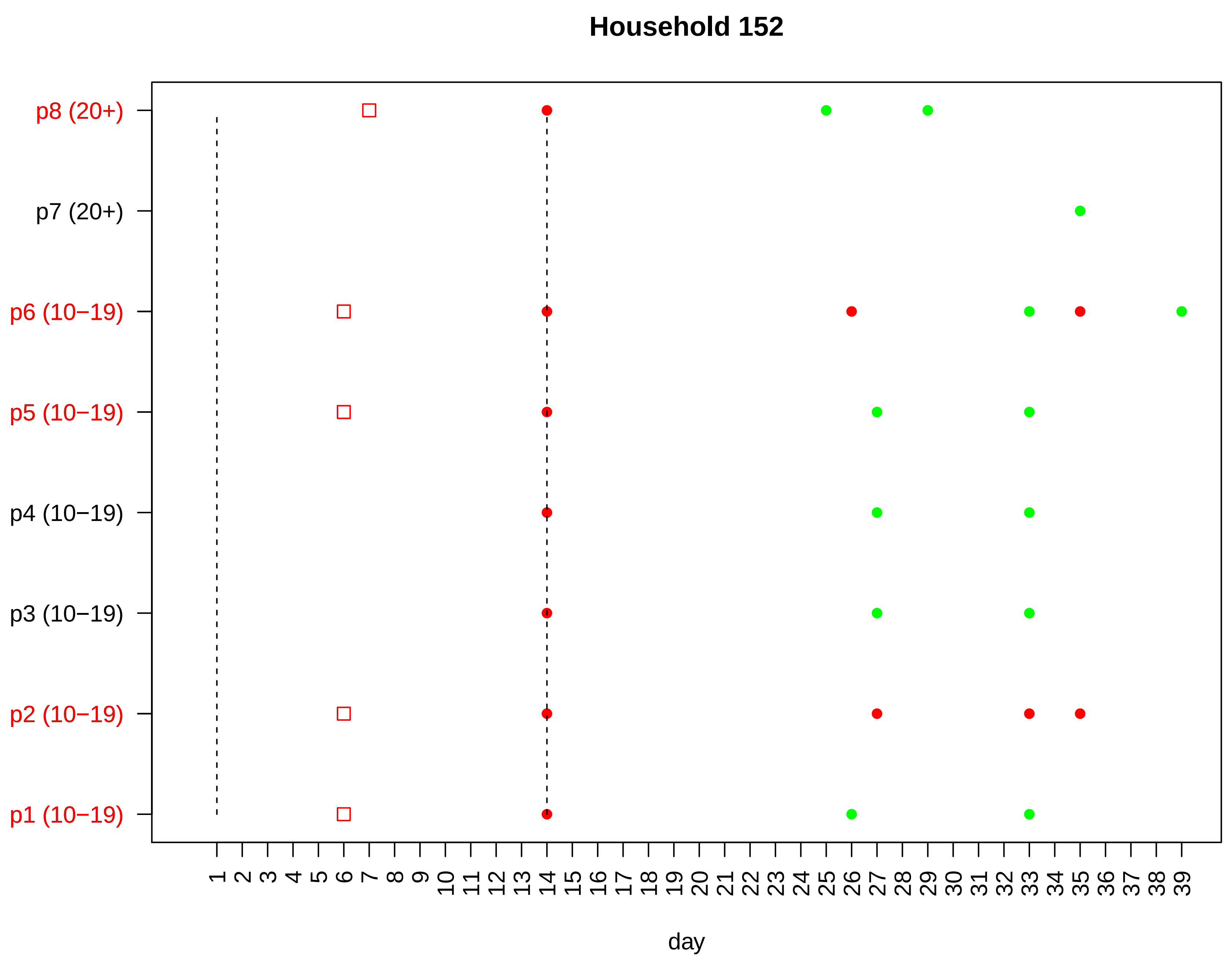

Household 152
p8 (20+)
p7 (20+)
p6 (10−19)
p5 (10−19)
p4 (10−19)
p3 (10−19)
p2 (10−19)
p1 (10−19)
1 2 3 4 5 6 7 8 9 10 11 12 13 14 15 16 17 18 19 20 21 22 23 24 25 26 27 28 29 30 31 32 33 34 35 36 37 38 39
day

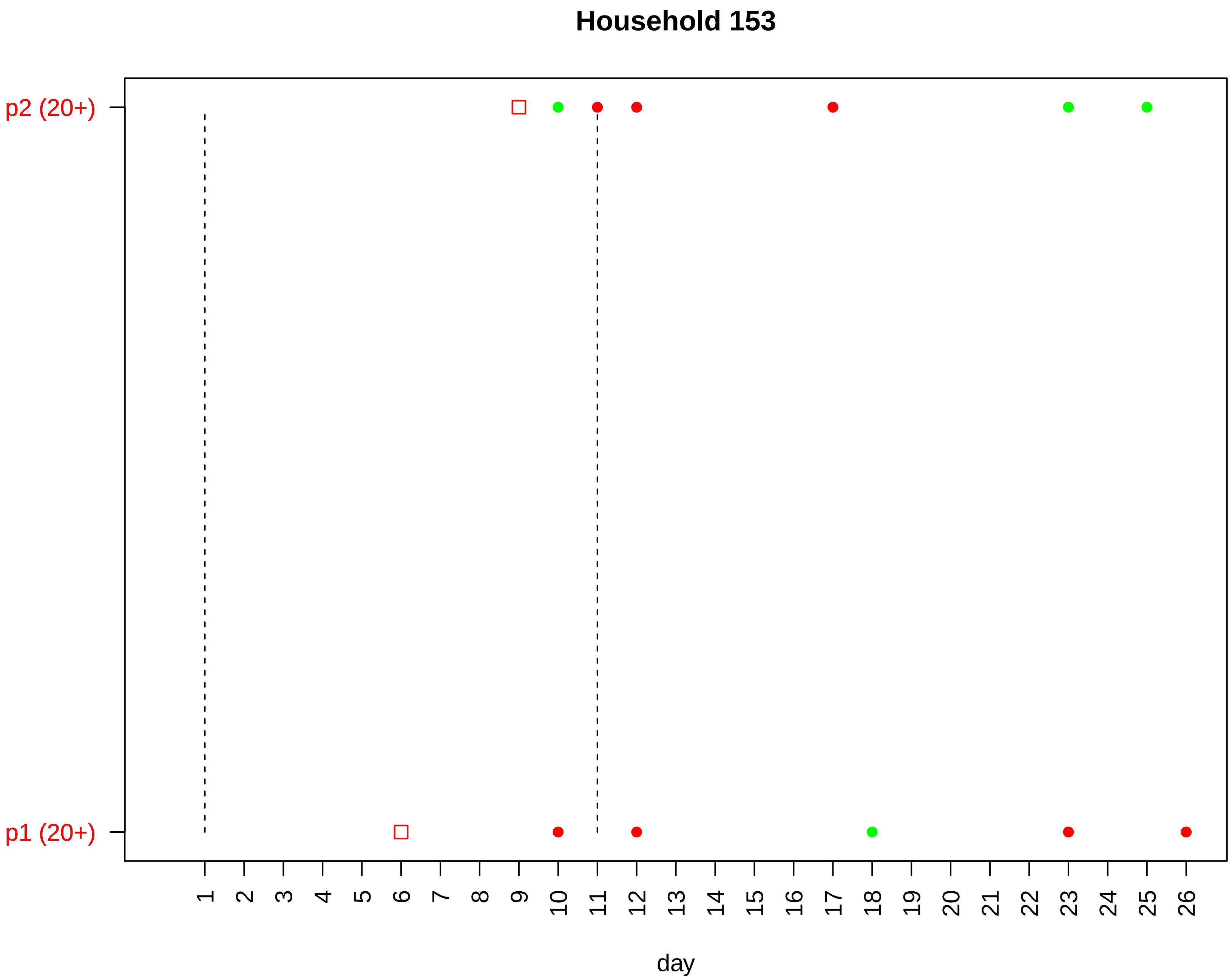

Household 153
p2 (20+)
p1 (20+)
1
2
3
4
5
6
7
8
9
10
11
12
13
14
15
16
17
18
19
20
21
22
23
24
25
26
day

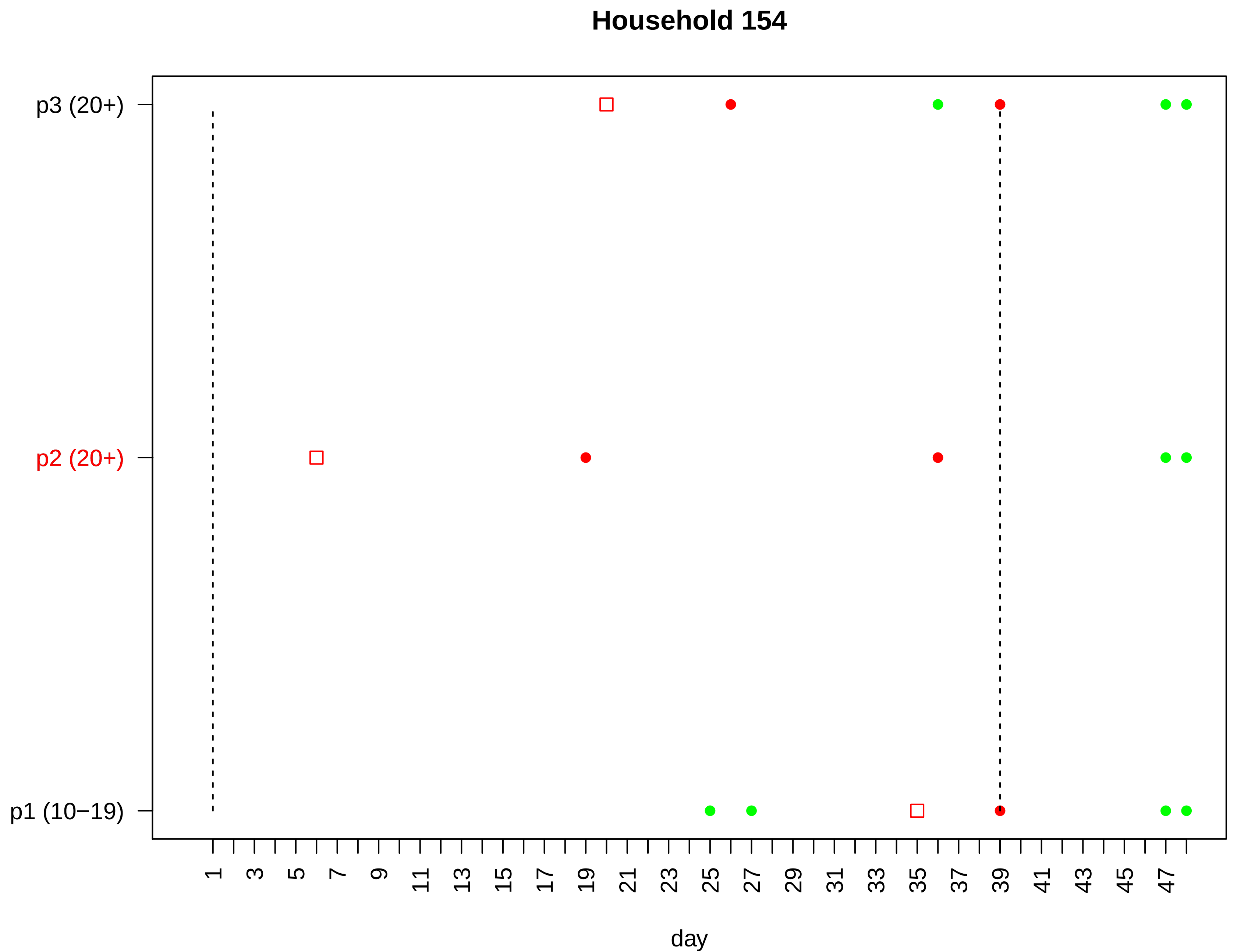

Household 154
p3 (20+)
p2 (20+)
p1 (10−19)
1
3
5
7
9
11
13
15
17
19
21
23
25
27
29
31
33
35
37
39
41
43
45
47
day

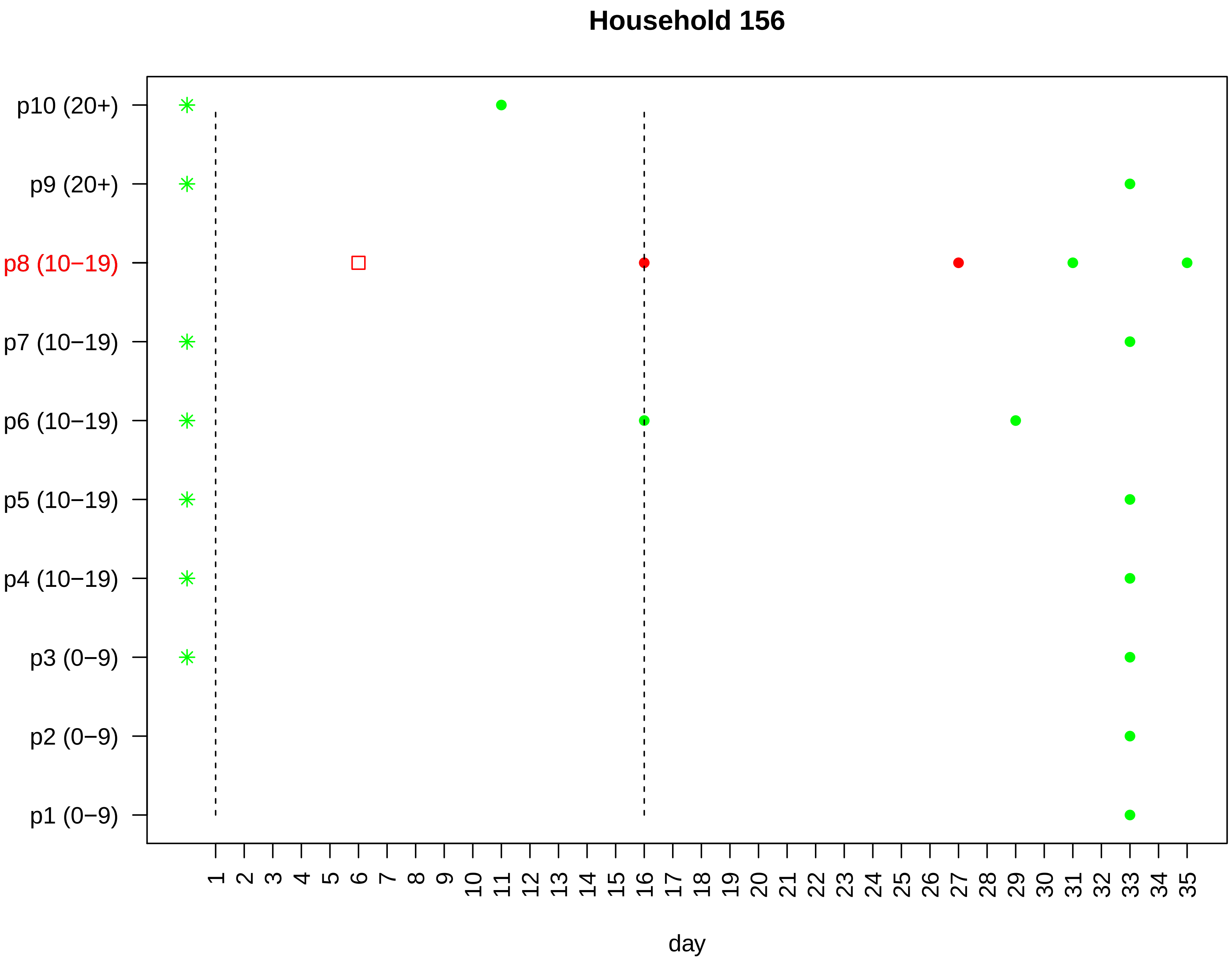

Household 156
p10 (20+)
p9 (20+)
p8 (10−19)
p7 (10−19)
p6 (10−19)
p5 (10−19)
p4 (10−19)
p3 (0−9)
p2 (0−9)
p1 (0−9)
1 2 3 4 5 6 7 8 9 10 11 12 13 14 15 16 17 18 19 20 21 22 23 24 25 26 27 28 29 30 31 32 33 34 35
day

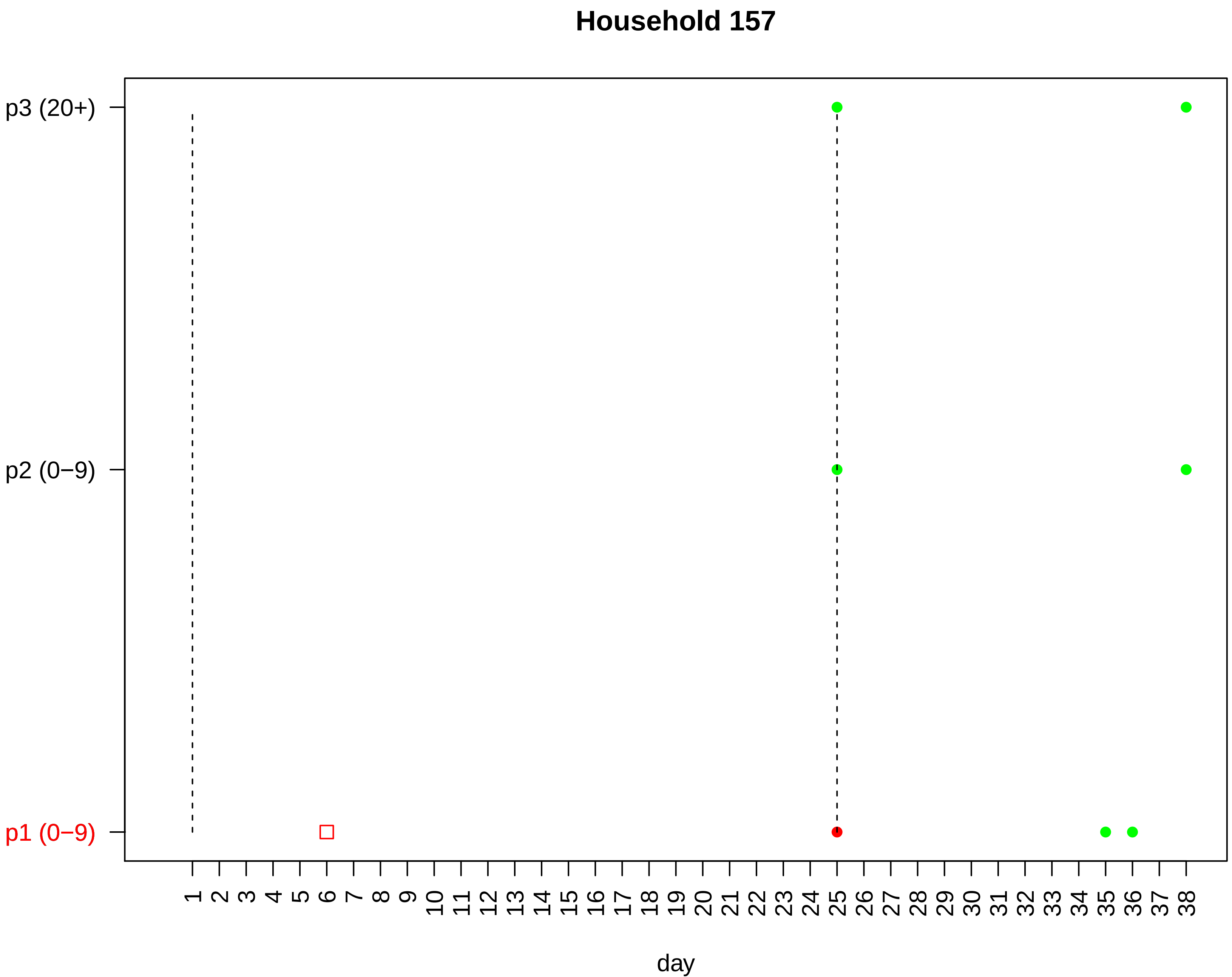

Household 157
p3 (20+)
p2 (0−9)
p1 (0−9)
1 2 3 4 5 6 7 8 9 10 11 12 13 14 15 16 17 18 19 20 21 22 23 24 25 26 27 28 29 30 31 32 33 34 35 36 37 38
day

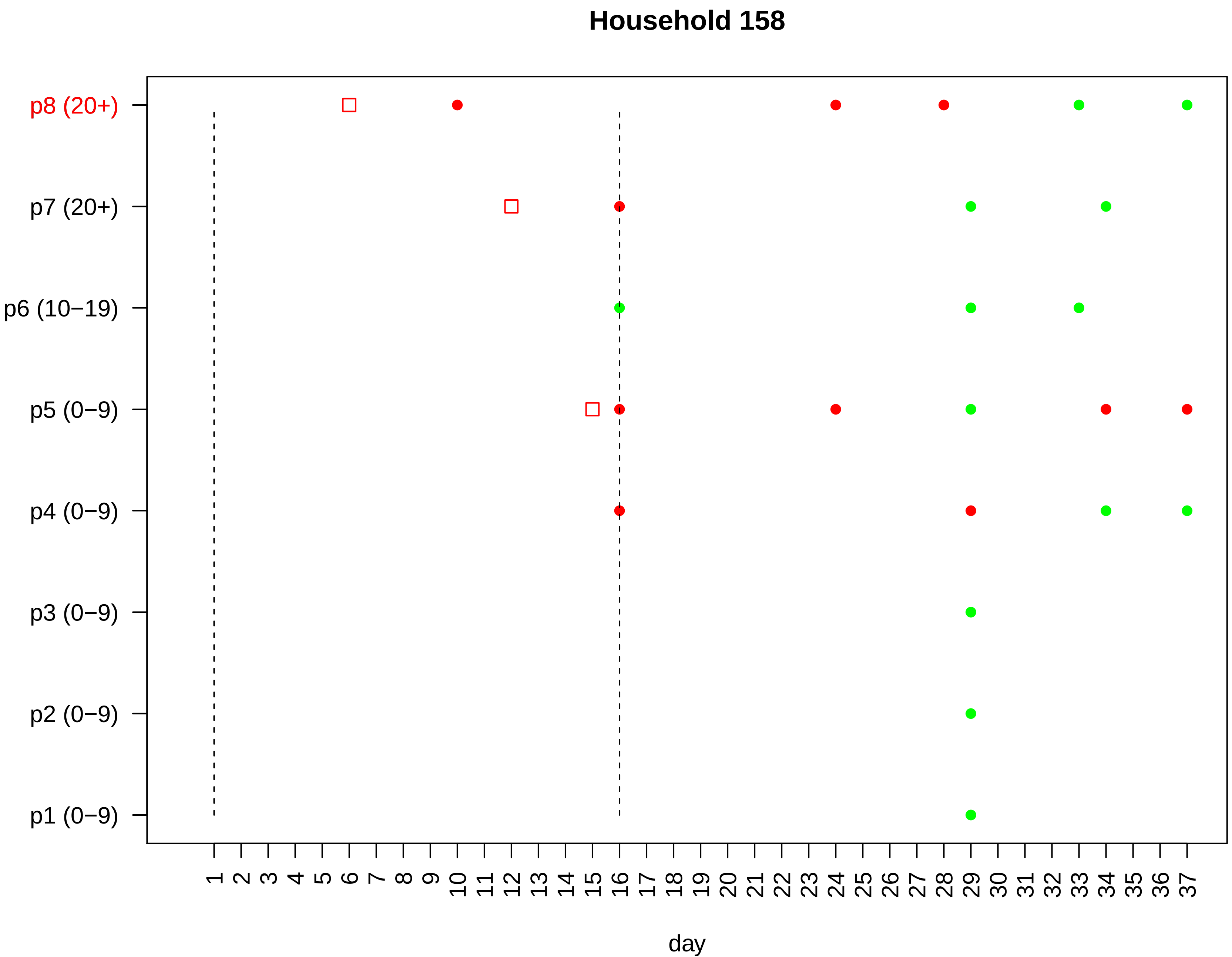

Household 158
p8 (20+)
p7 (20+)
p6 (10−19)
p5 (0−9)
p4 (0−9)
p3 (0−9)
p2 (0−9)
p1 (0−9)
1 2 3 4 5 6 7 8 9 10 11 12 13 14 15 16 17 18 19 20 21 22 23 24 25 26 27 28 29 30 31 32 33 34 35 36 37
day

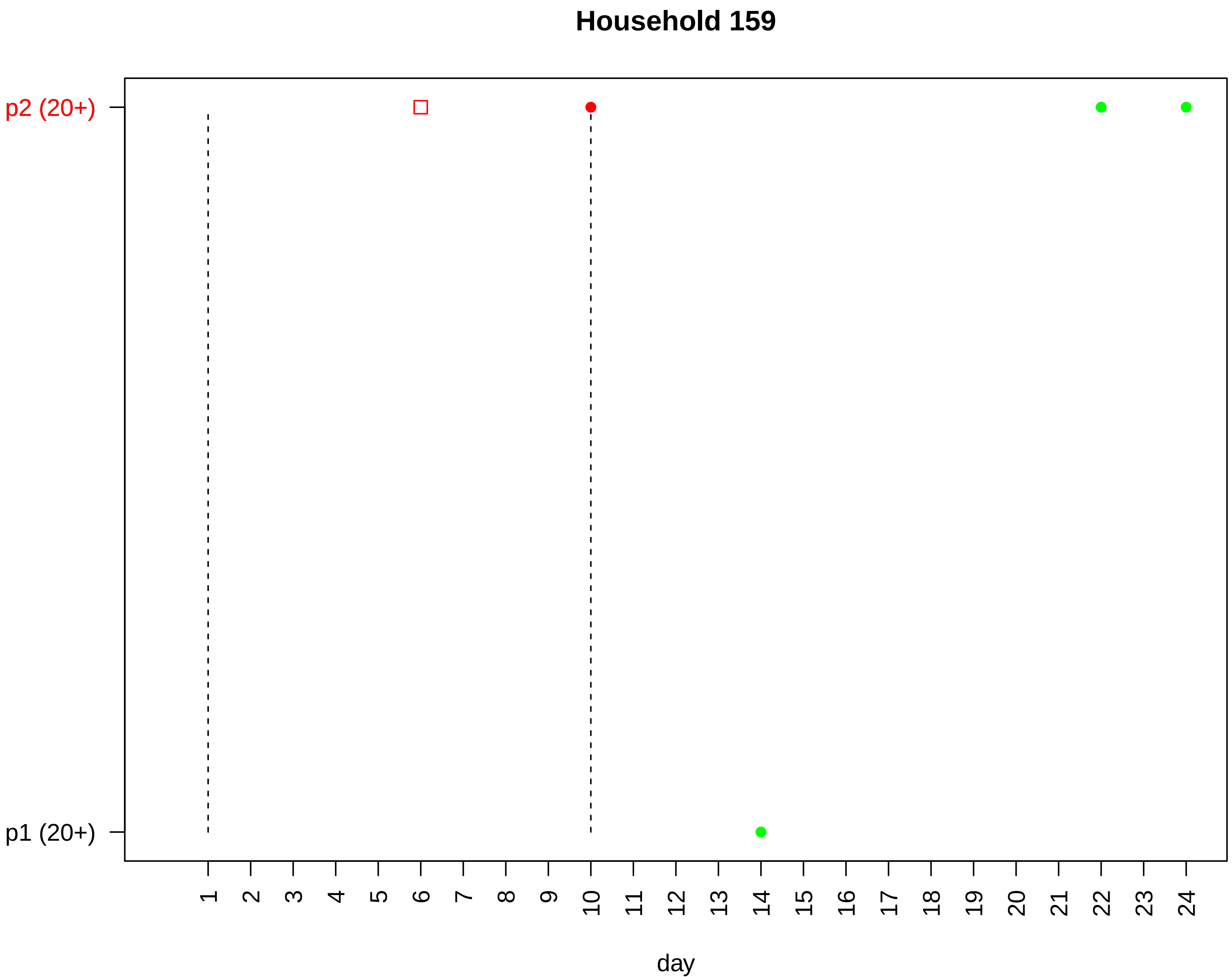
Household 159
p2 (20+)
p1 (20+)
1
2
3
4
5
6
7
8
9
10
11
12
13
14
15
16
17
18
19
20
21
22
23
24
day

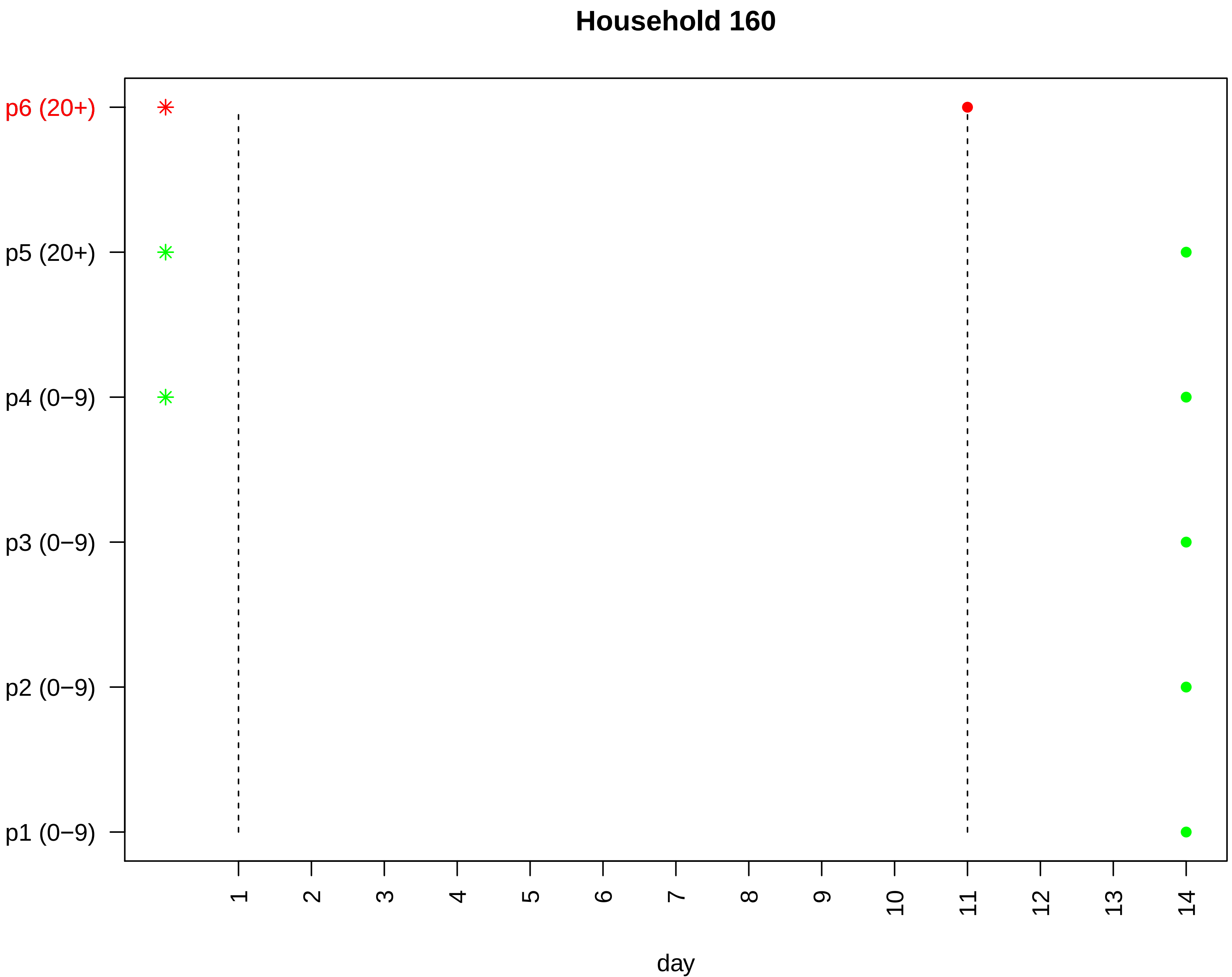

Household 160
p6 (20+)
p5 (20+)
p4 (0−9)
p3 (0−9)
p2 (0−9)
p1 (0−9)
1
2
3
4
5
6
7
8
9
10
11
12
13
14
day

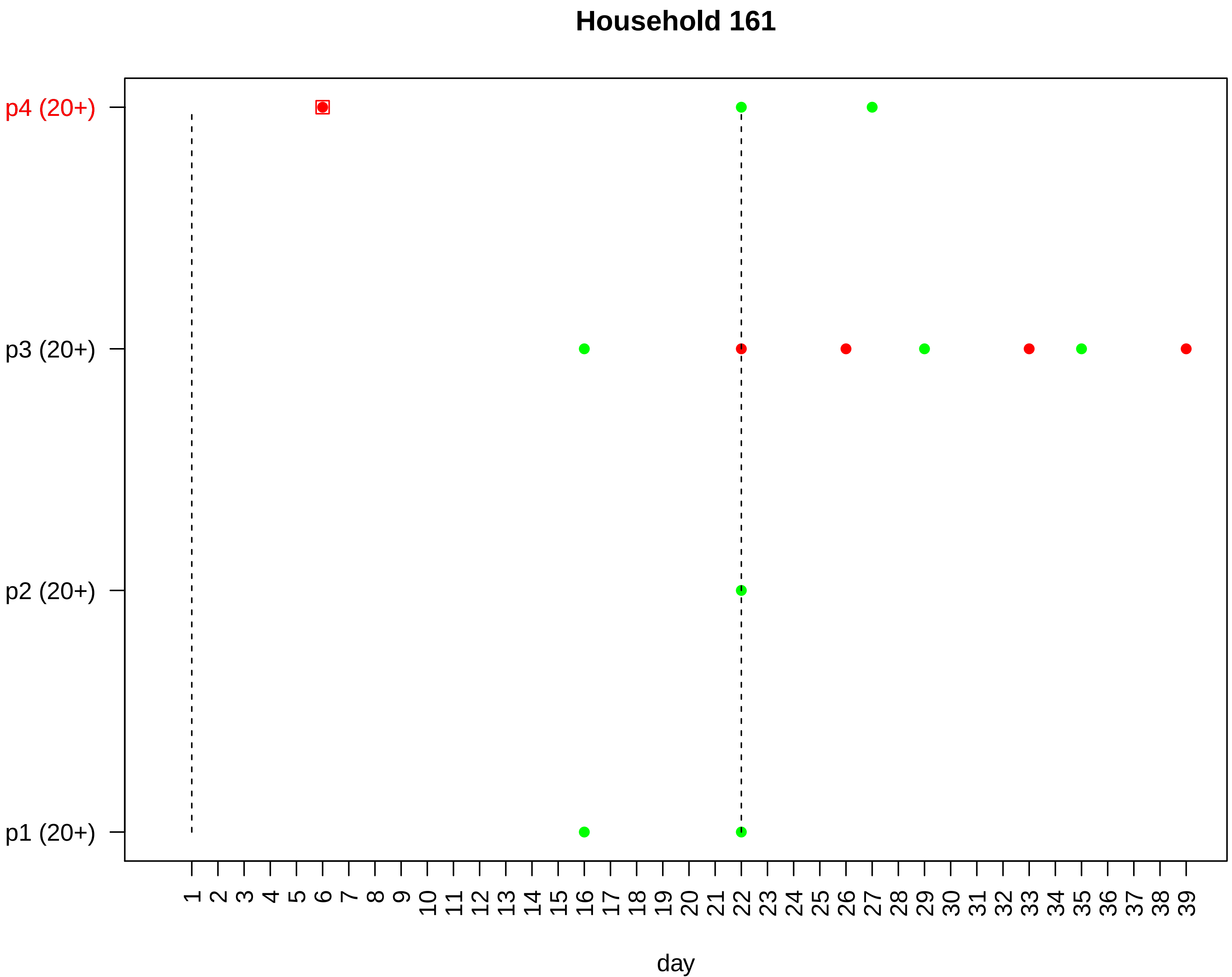

Household 161
p4 (20+)
p3 (20+)
p2 (20+)
p1 (20+)
1 2 3 4 5 6 7 8 9 10 11 12 13 14 15 16 17 18 19 20 21 22 23 24 25 26 27 28 29 30 31 32 33 34 35 36 37 38 39
day

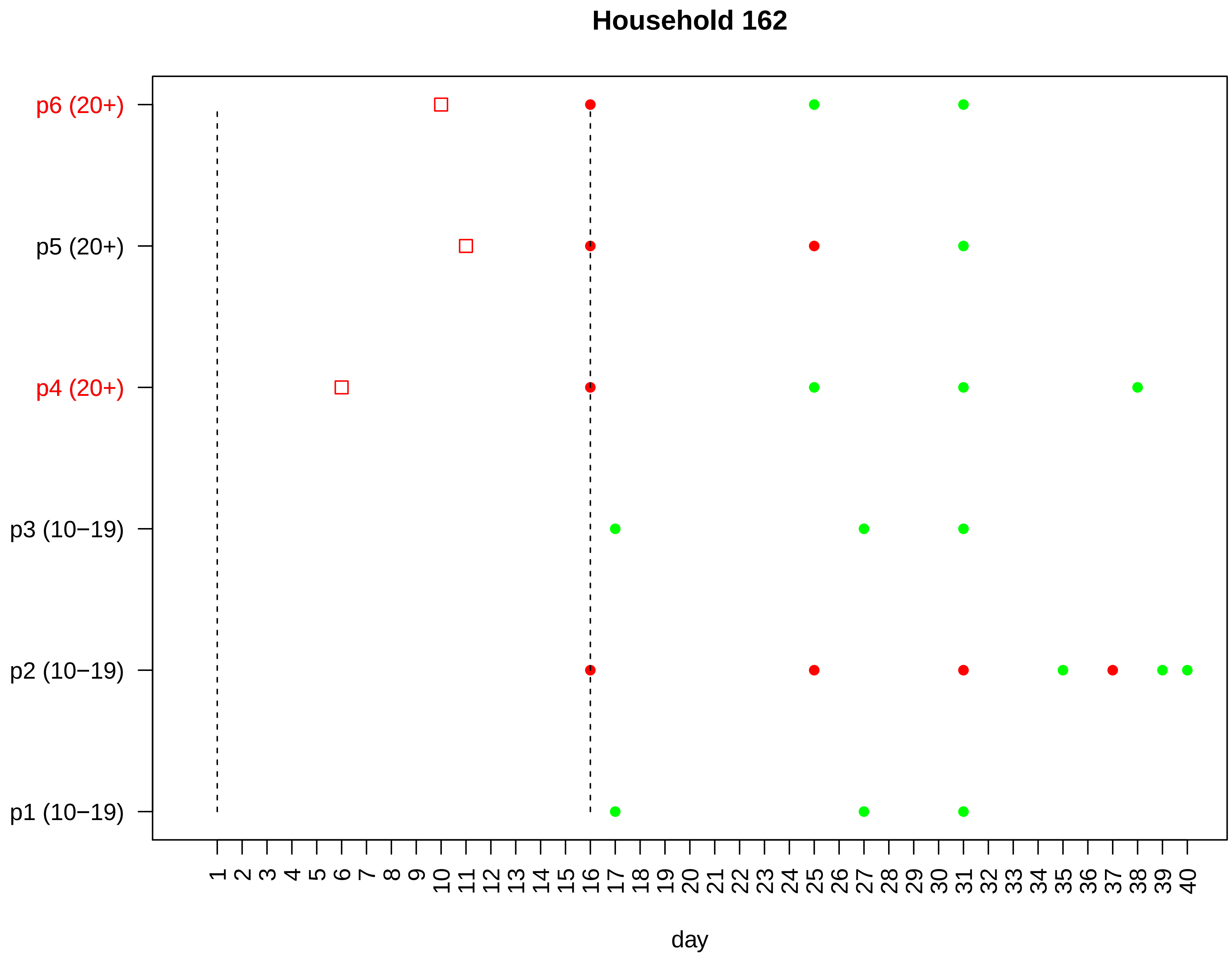
Household 162
p6 (20+)
p5 (20+)
p4 (20+)
p3 (10−19)
p2 (10−19)
p1 (10−19)
1 2 3 4 5 6 7 8 9 10 11 12 13 14 15 16 17 18 19 20 21 22 23 24 25 26 27 28 29 30 31 32 33 34 35 36 37 38 39 40
day

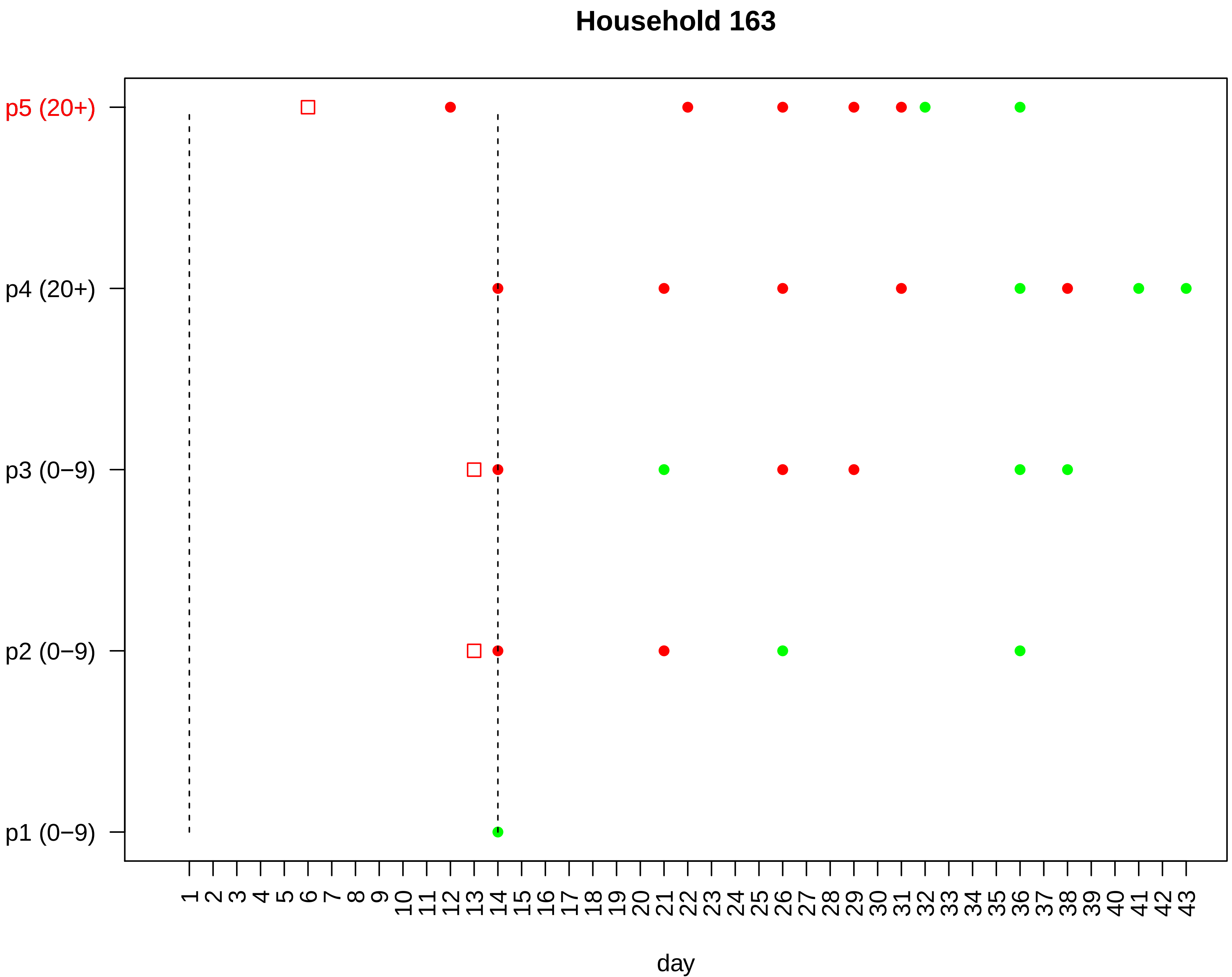

Household 163
p5 (20+)
p4 (20+)
p3 (0−9)
p2 (0−9)
p1 (0−9)
1 2 3 4 5 6 7 8 9 10 11 12 13 14 15 16 17 18 19 20 21 22 23 24 25 26 27 28 29 30 31 32 33 34 35 36 37 38 39 40 41 42 43
day

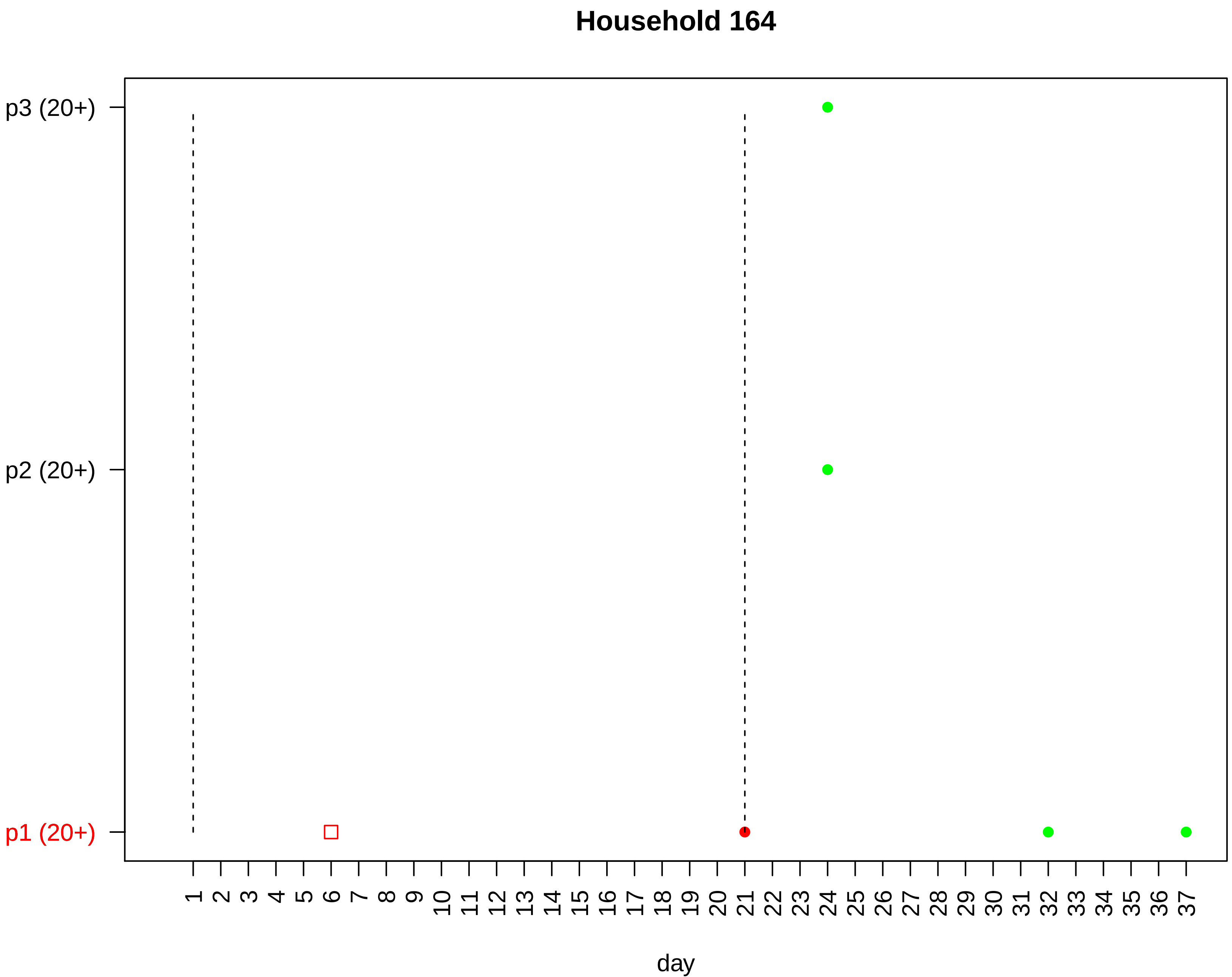

Household 164
p3 (20+)
p2 (20+)
p1 (20+)
1 2 3 4 5 6 7 8 9 10 11 12 13 14 15 16 17 18 19 20 21 22 23 24 25 26 27 28 29 30 31 32 33 34 35 36 37
day

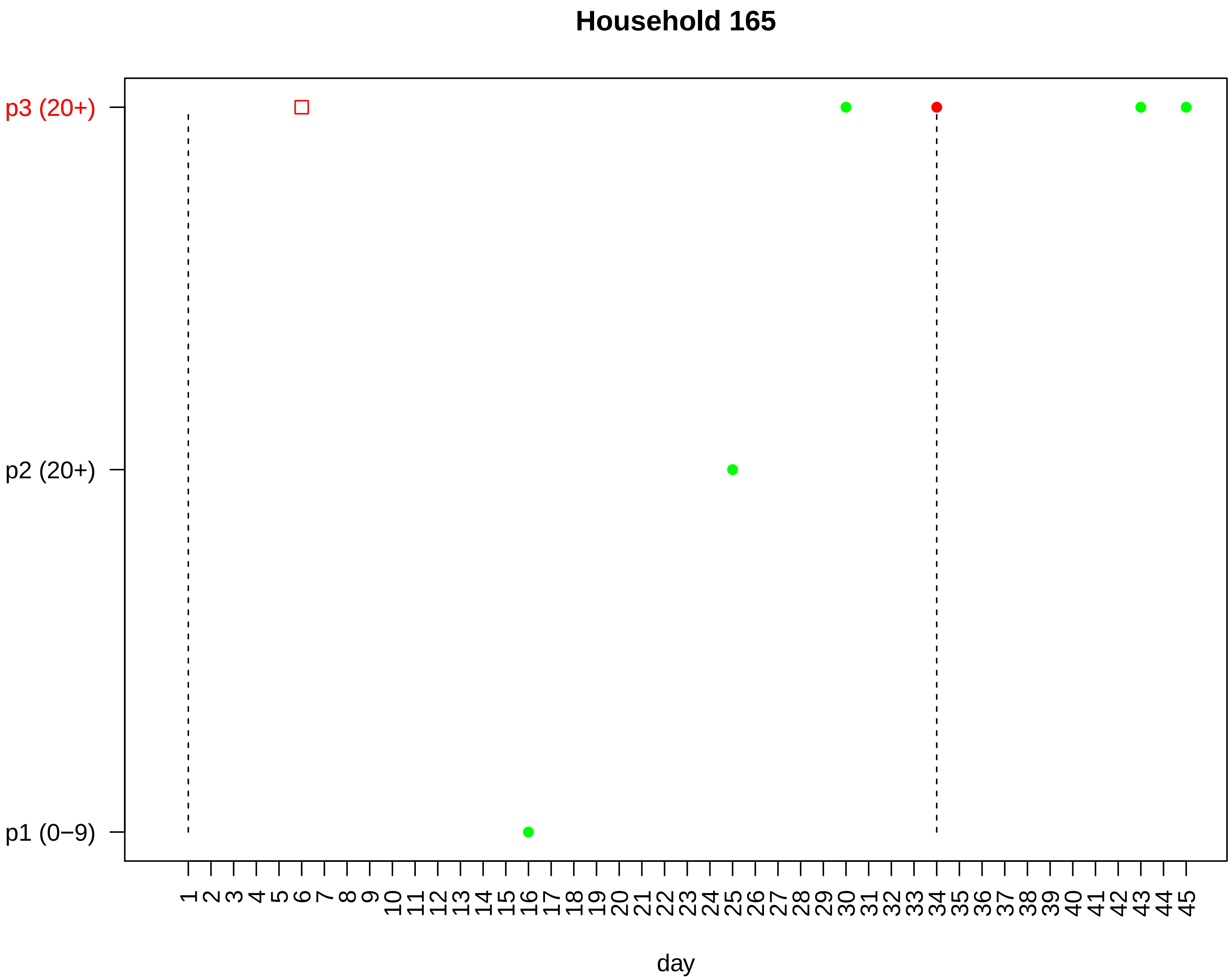

Household 165
p3 (20+)
p2 (20+)
p1 (0−9)
1
2
3
4
5
6
7
8
9
10
11
12
13
14
15
16
17
18
19
20
21
22
23
24
25
26
27
28
29
30
31
32
33
34
35
36
37
38
39
40
41
42
43
44
45
day

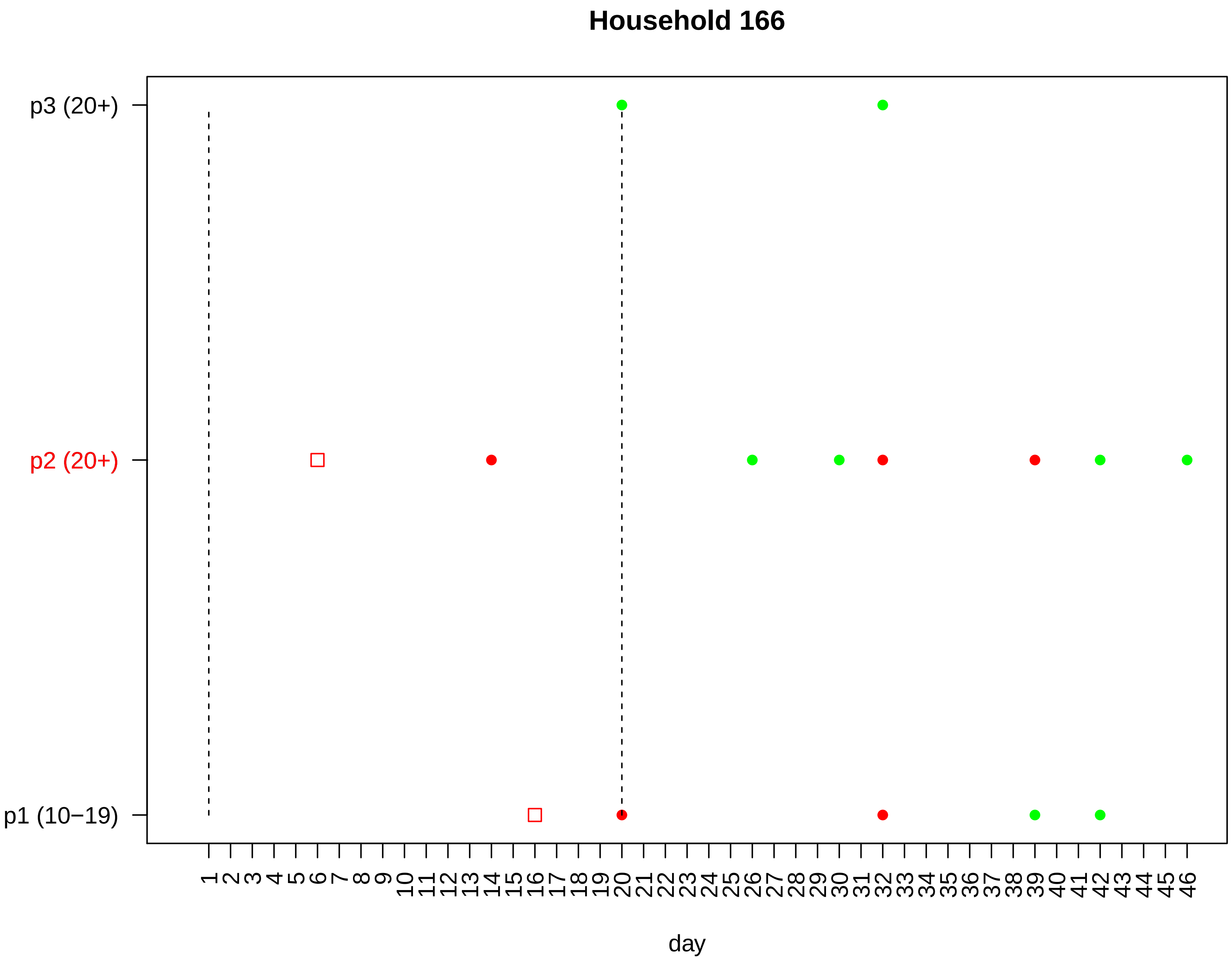

Household 166
p3 (20+)
p2 (20+)
p1 (10−19)
1 2 3 4 5 6 7 8 9 10 11 12 13 14 15 16 17 18 19 20 21 22 23 24 25 26 27 28 29 30 31 32 33 34 35 36 37 38 39 40 41 42 43 44 45 46
day

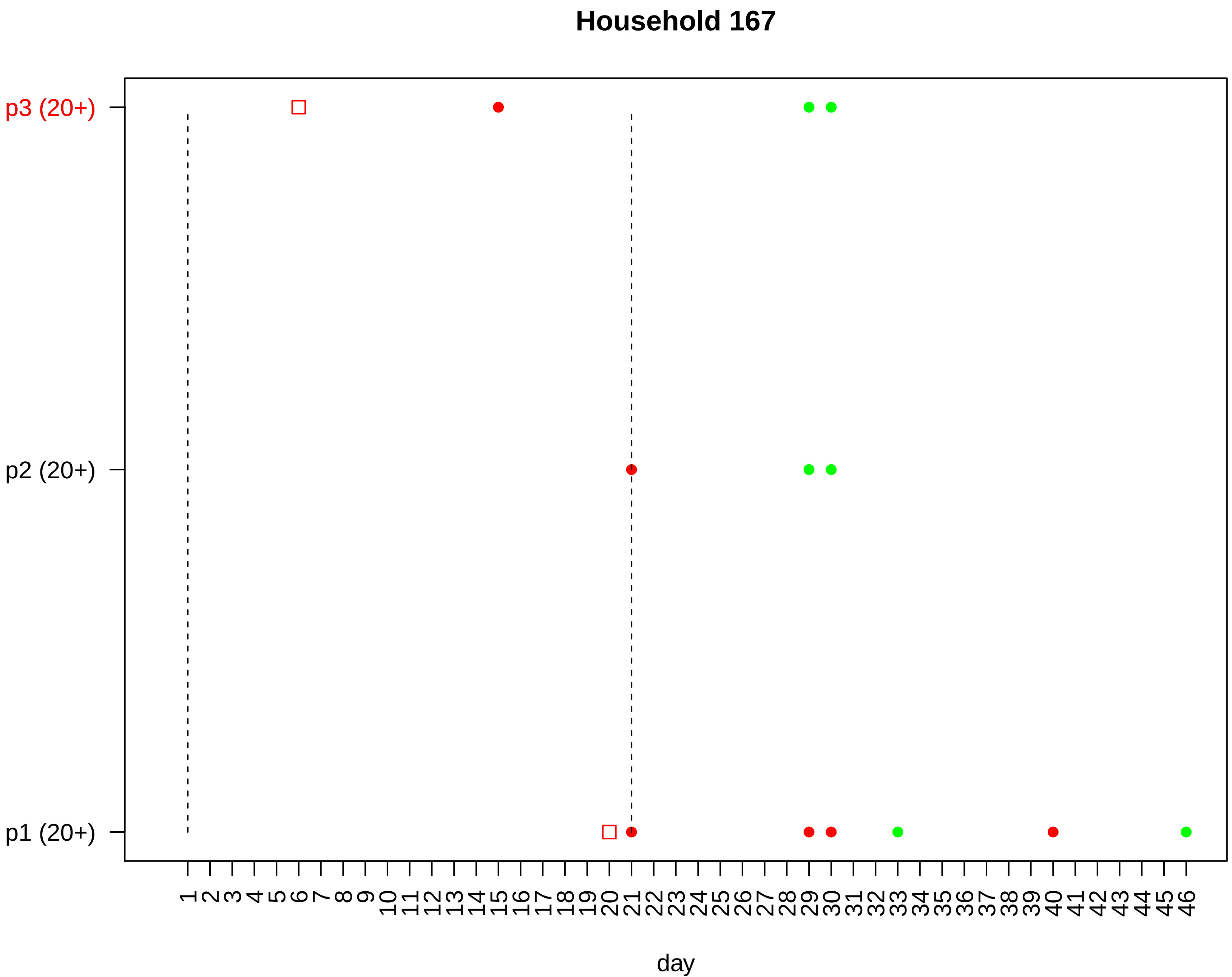

Household 167
p3 (20+)
p2 (20+)
p1 (20+)
1 2 3 4 5 6 7 8 9 10 11 12 13 14 15 16 17 18 19 20 21 22 23 24 25 26 27 28 29 30 31 32 33 34 35 36 37 38 39 40 41 42 43 44 45 46
day

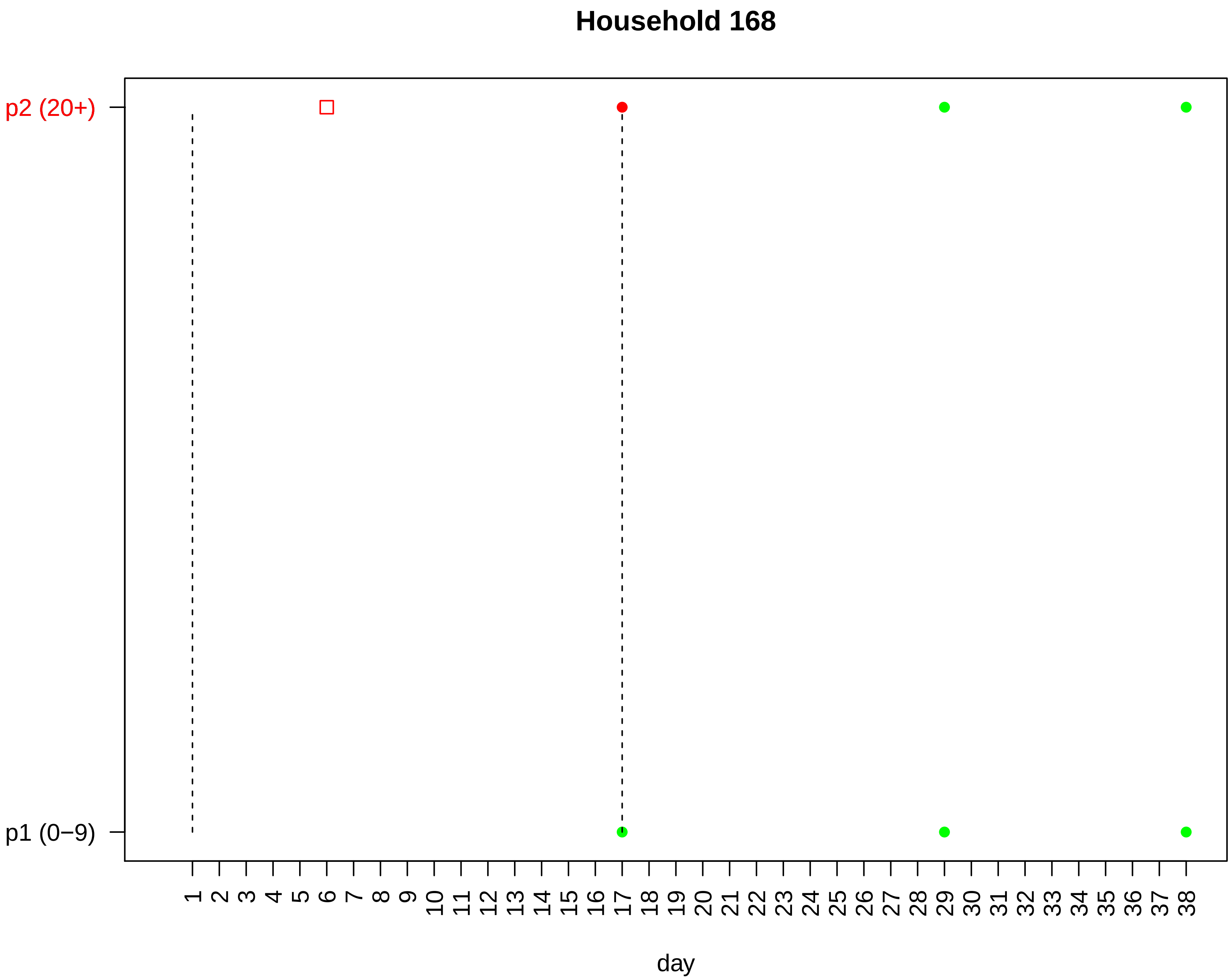
Household 168
p2 (20+)
p1 (0−9)
1
2
3
4
5
6
7
8
9
10
11
12
13
14
15
16
17
18
19
20
21
22
23
24
25
26
27
28
29
30
31
32
33
34
35
36
37
38
day

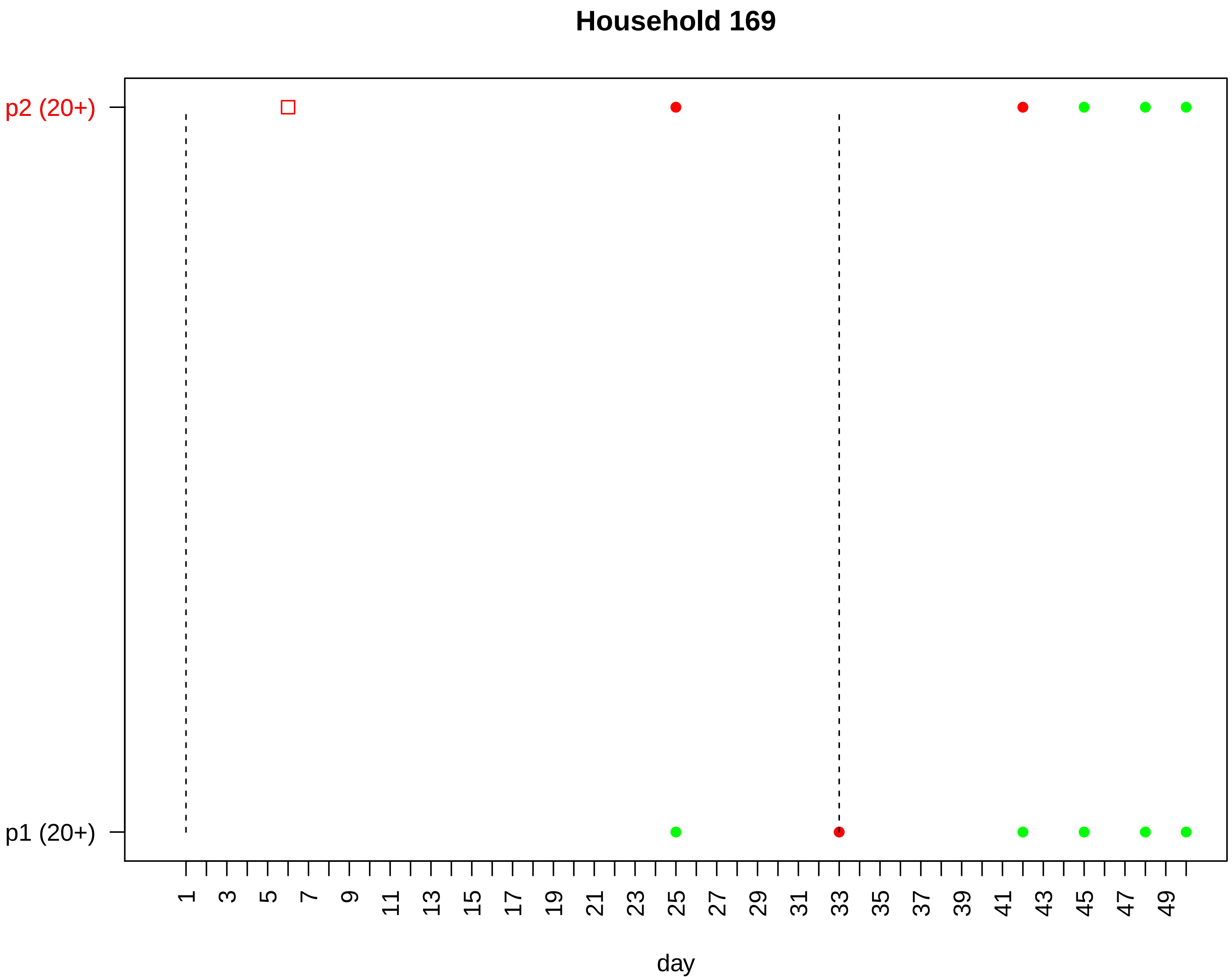

Household 169
p2 (20+)
p1 (20+)
1 3 5 7 9 11 13 15 17 19 21 23 25 27 29 31 33 35 37 39 41 43 45 47 49
day

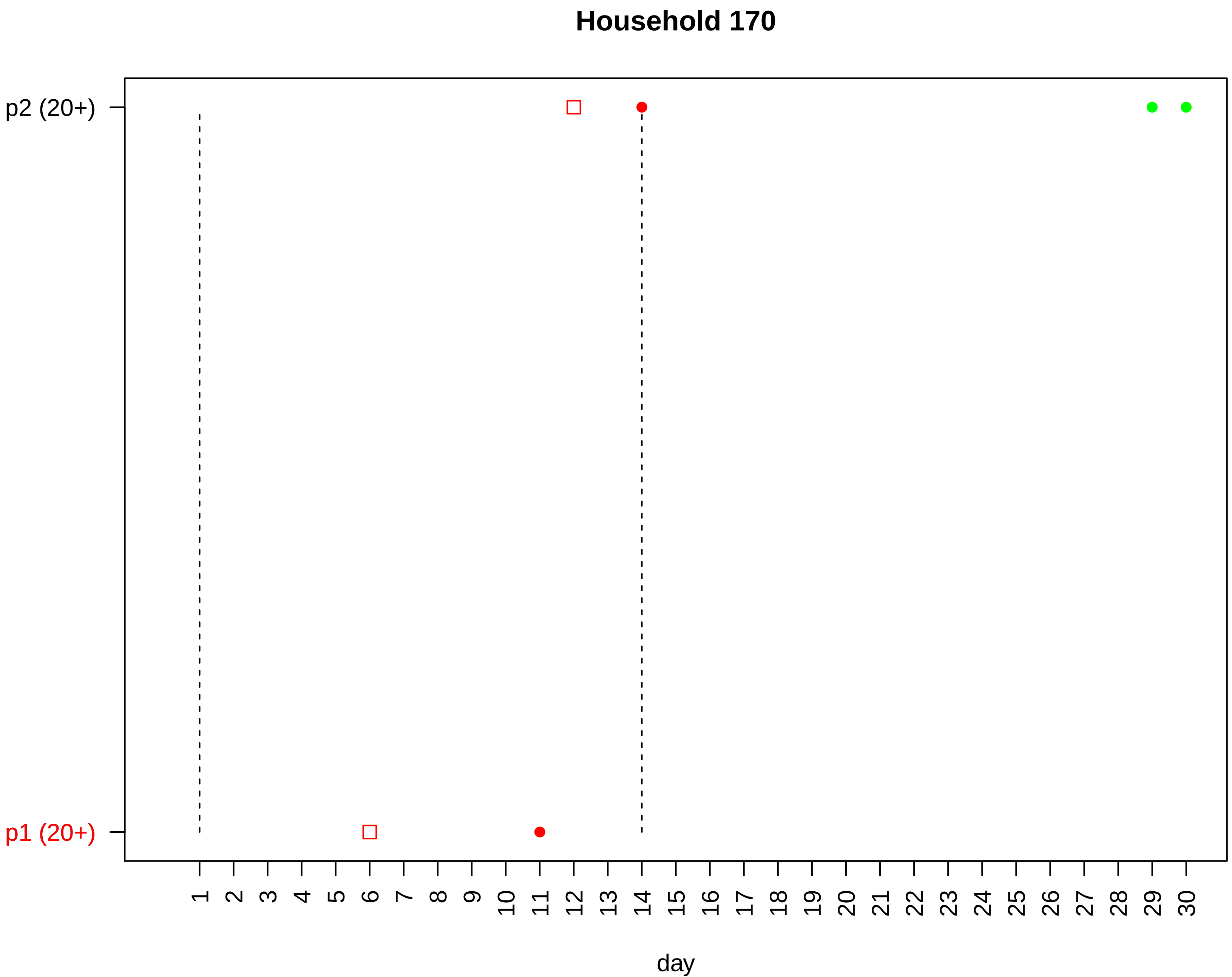
Household 170
p2 (20+)
p1 (20+)
1
2
3
4
5
6
7
8
9
10
11
12
13
14
15
16
17
18
19
20
21
22
23
24
25
26
27
28
29
30
day

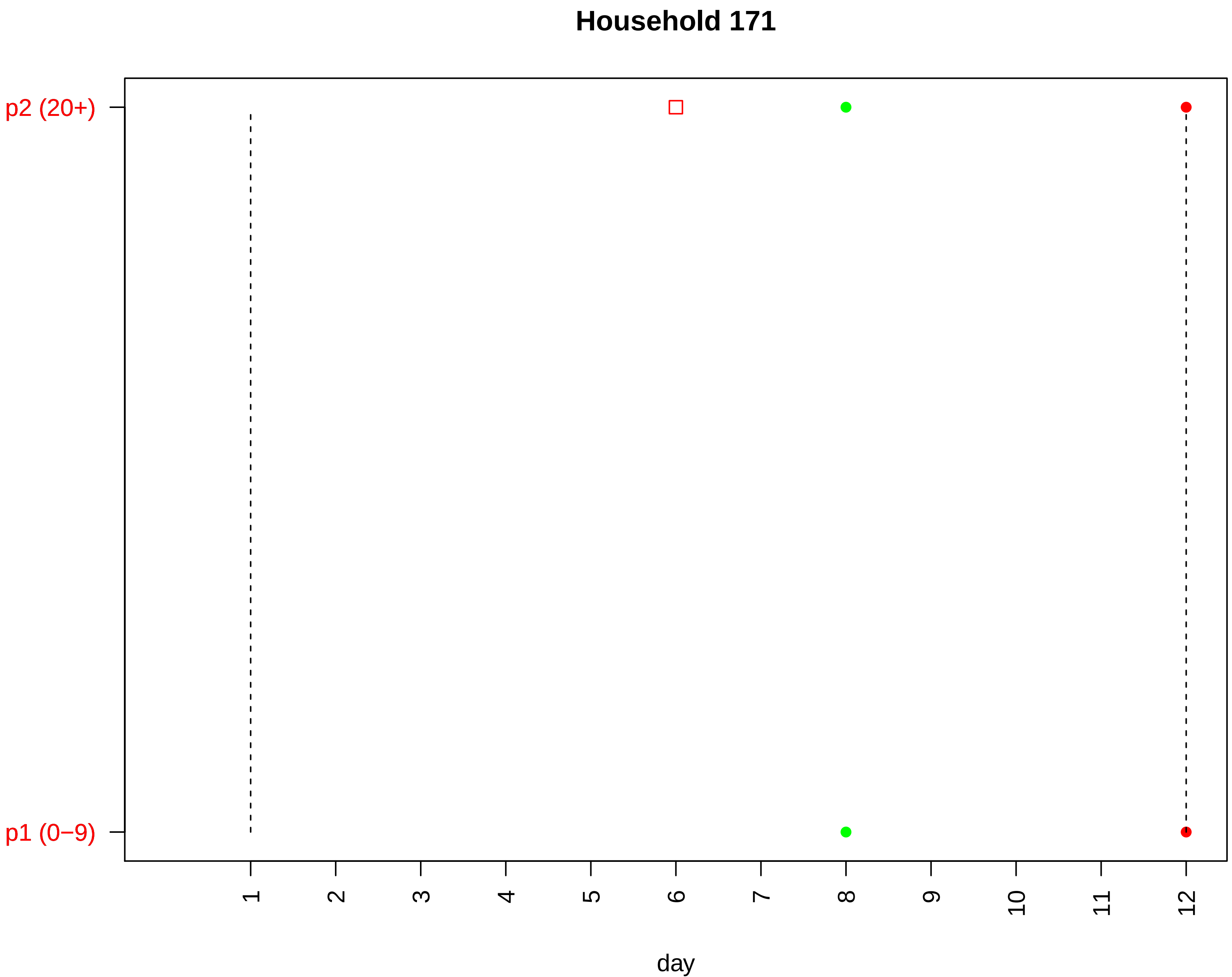

Household 171
p2 (20+)
p1 (0−9)
1
2
3
4
5
6
7
8
9
10
11
12
day

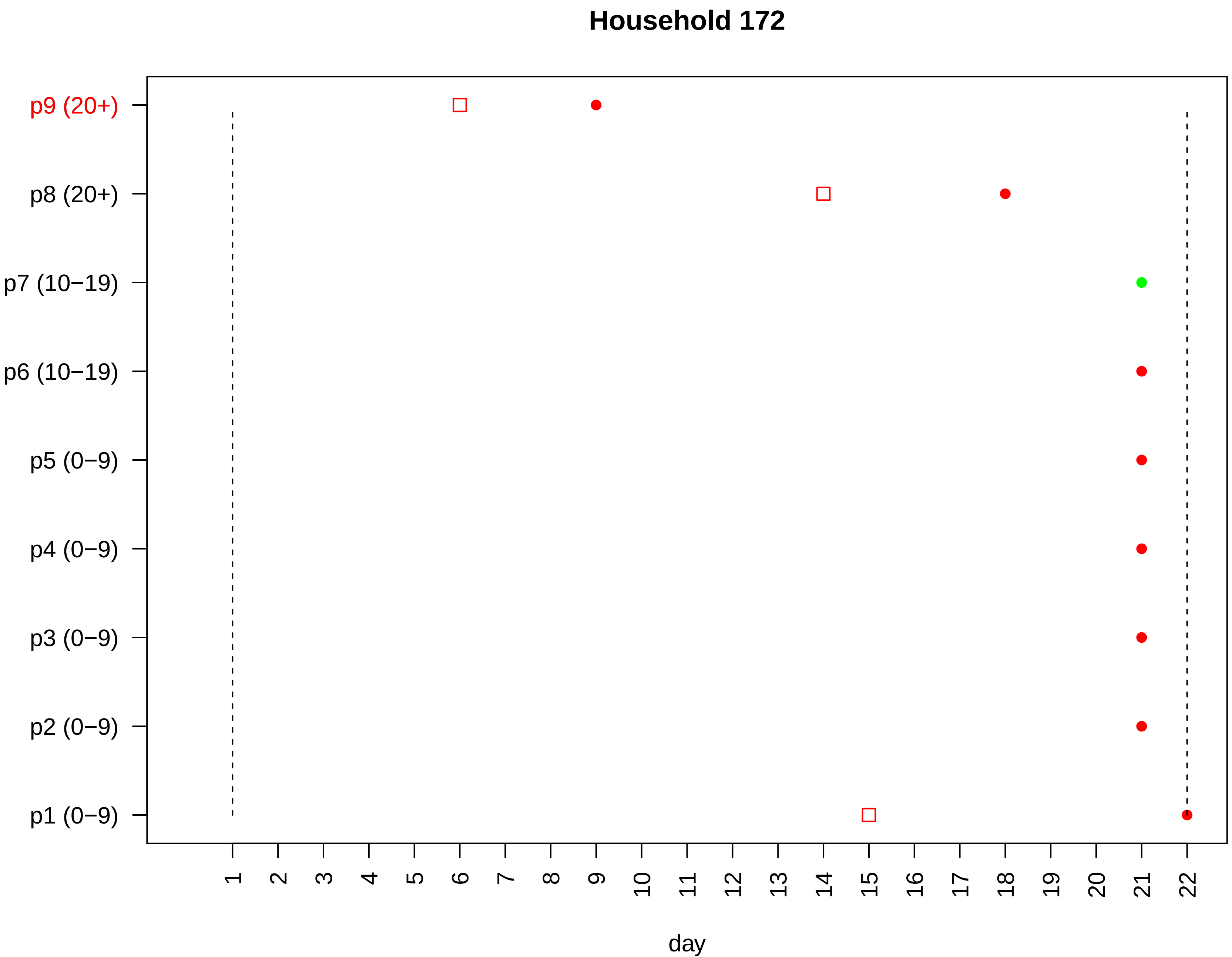

Household 172
p9 (20+)
p8 (20+)
p7 (10−19)
p6 (10−19)
p5 (0−9)
p4 (0−9)
p3 (0−9)
p2 (0−9)
p1 (0−9)
1
2
3
4
5
6
7
8
9
10
11
12
13
14
15
16
17
18
19
20
21
22
day

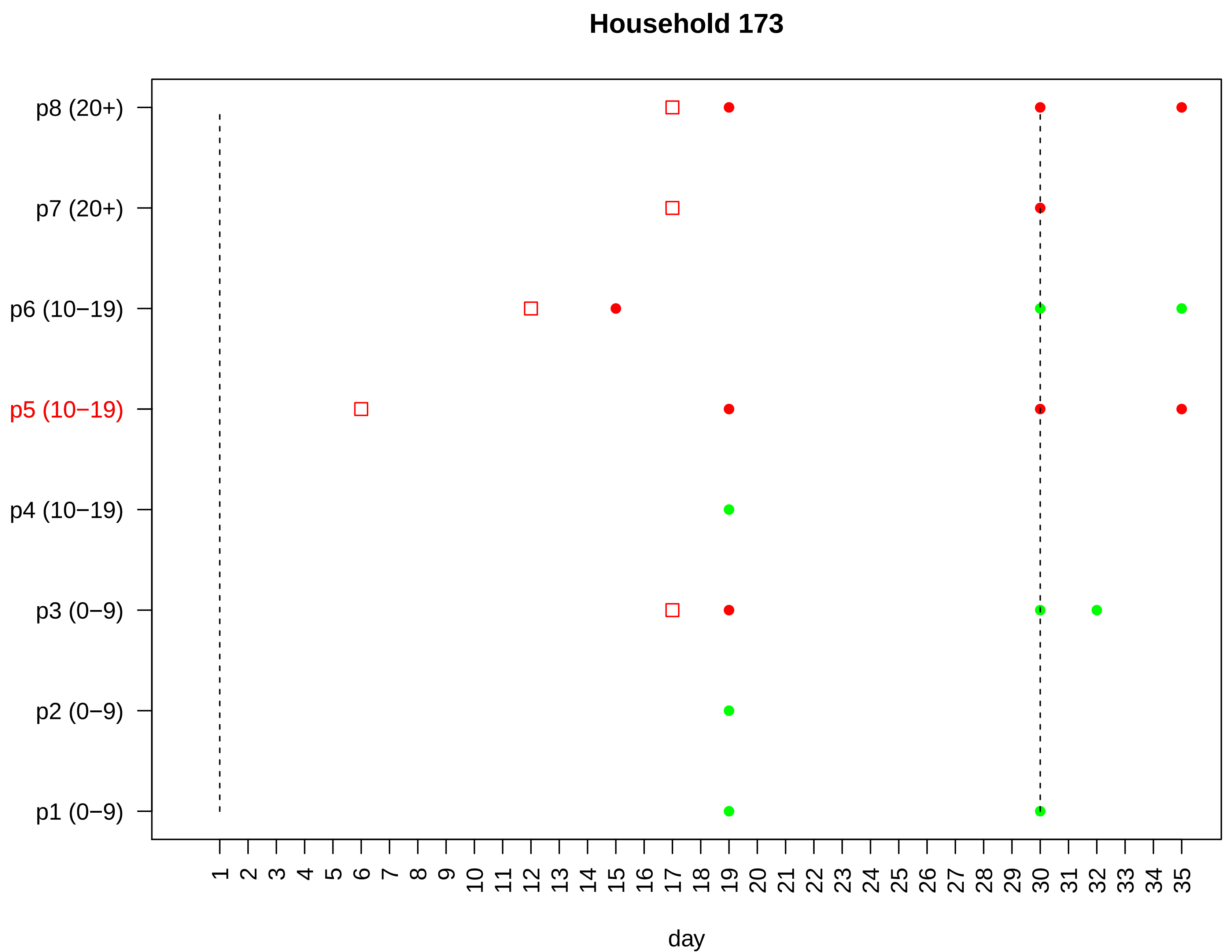

Household 173
p8 (20+)
p7 (20+)
p6 (10−19)
p5 (10−19)
p4 (10−19)
p3 (0−9)
p2 (0−9)
p1 (0−9)
1
2
3
4
5
6
7
8
9
10
11
12
13
14
15
16
17
18
19
20
21
22
23
24
25
26
27
28
29
30
31
32
33
34
35
day

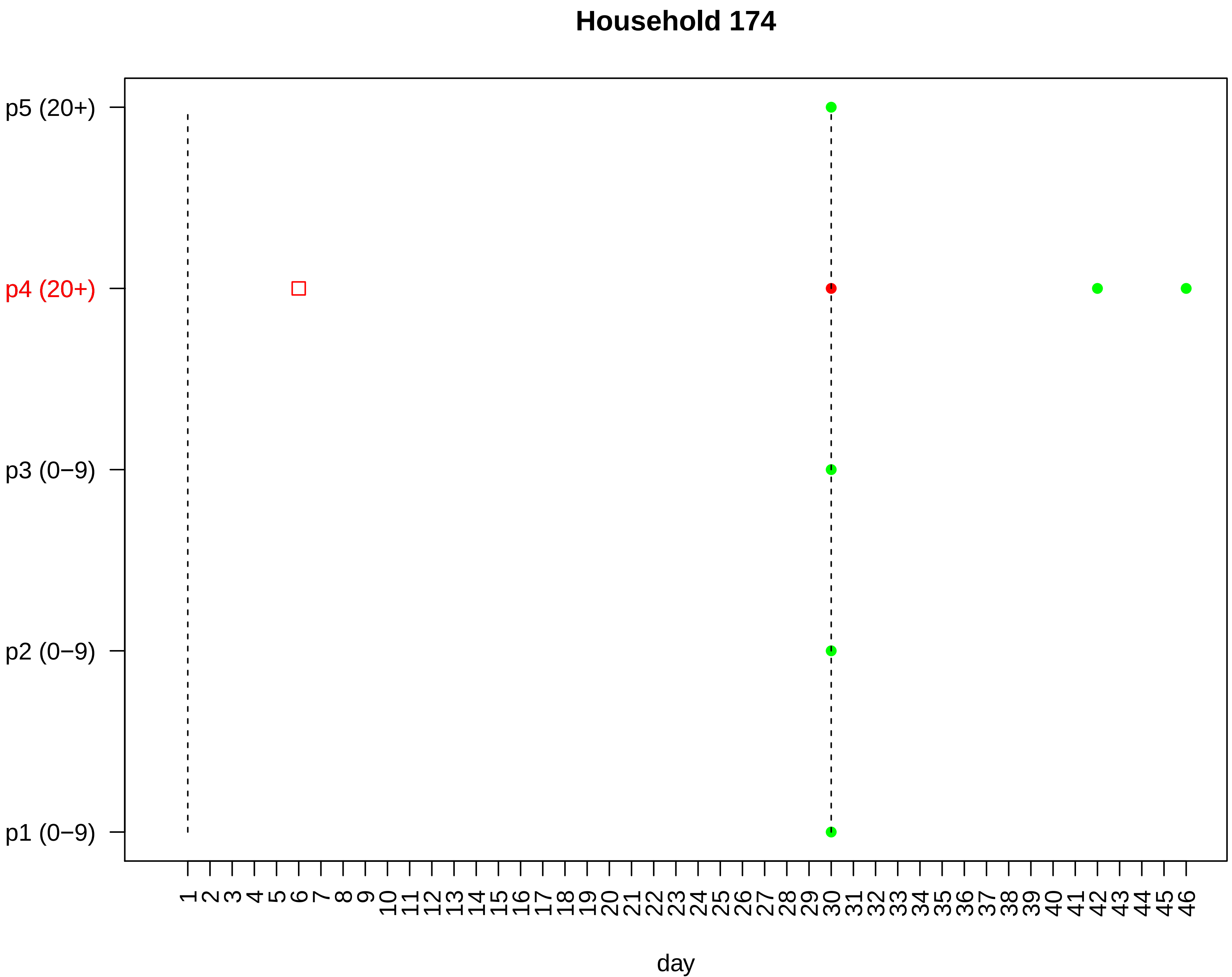

Household 174
p5 (20+)
p4 (20+)
p3 (0−9)
p2 (0−9)
p1 (0−9)
1 2 3 4 5 6 7 8 9 10 11 12 13 14 15 16 17 18 19 20 21 22 23 24 25 26 27 28 29 30 31 32 33 34 35 36 37 38 39 40 41 42 43 44 45 46
day

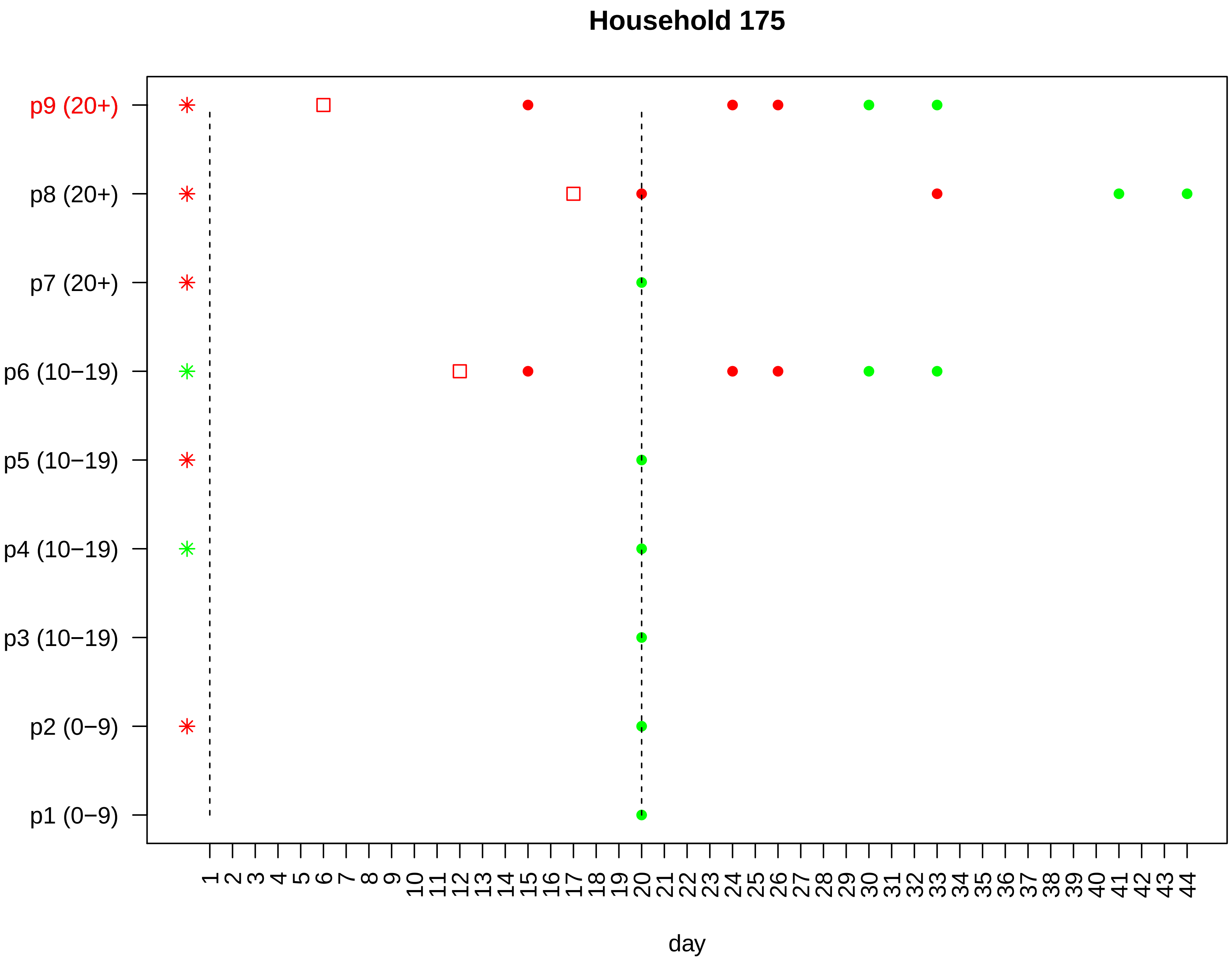

Household 175
p9 (20+)
p8 (20+)
p7 (20+)
p6 (10−19)
p5 (10−19)
p4 (10−19)
p3 (10−19)
p2 (0−9)
p1 (0−9)
1 2 3 4 5 6 7 8 9 10 11 12 13 14 15 16 17 18 19 20 21 22 23 24 25 26 27 28 29 30 31 32 33 34 35 36 37 38 39 40 41 42 43 44
day

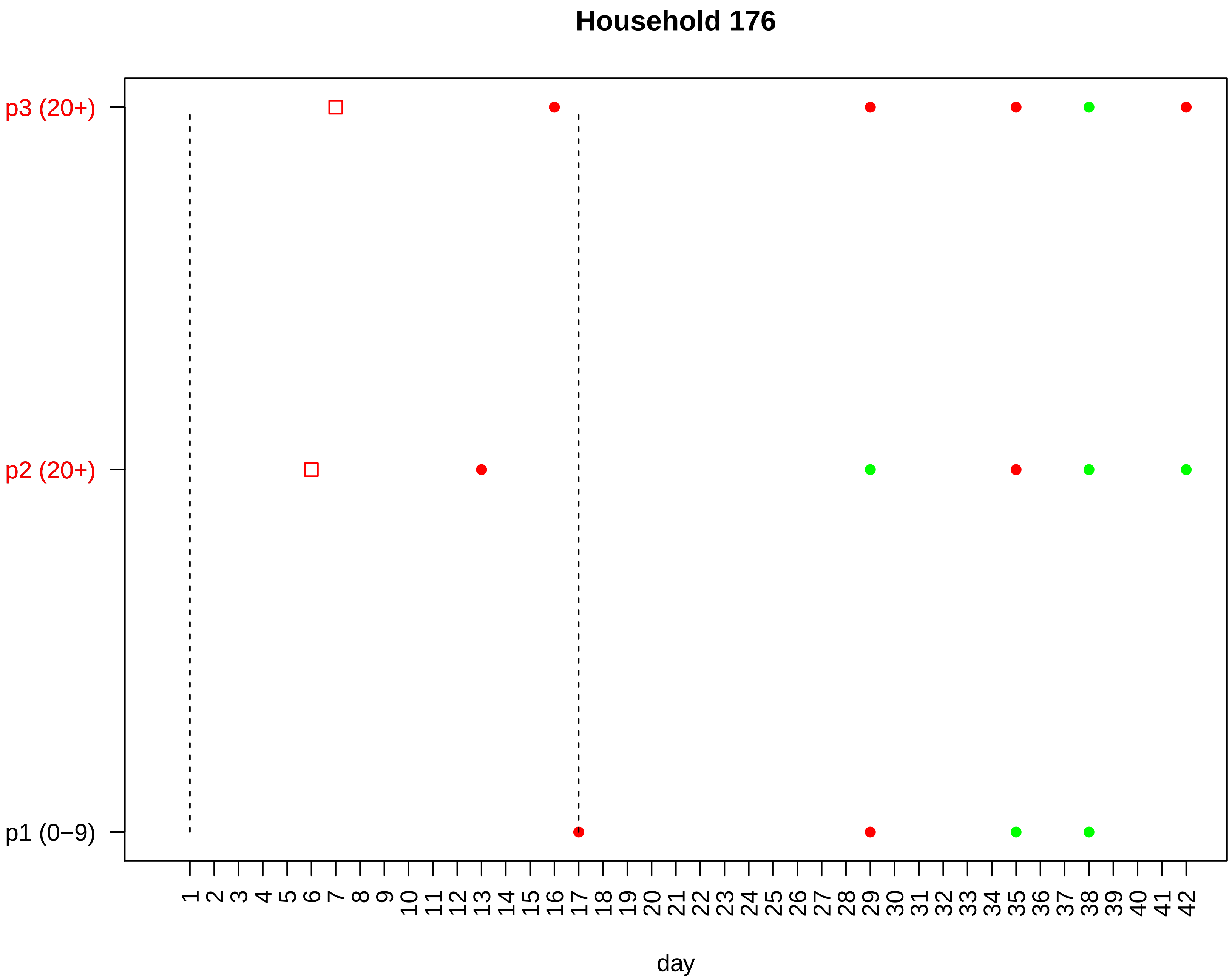

Household 176
p3 (20+)
p2 (20+)
p1 (0−9)
1
2
3
4
5
6
7
8
9
10
11
12
13
14
15
16
17
18
19
20
21
22
23
24
25
26
27
28
29
30
31
32
33
34
35
36
37
38
39
40
41
42
day

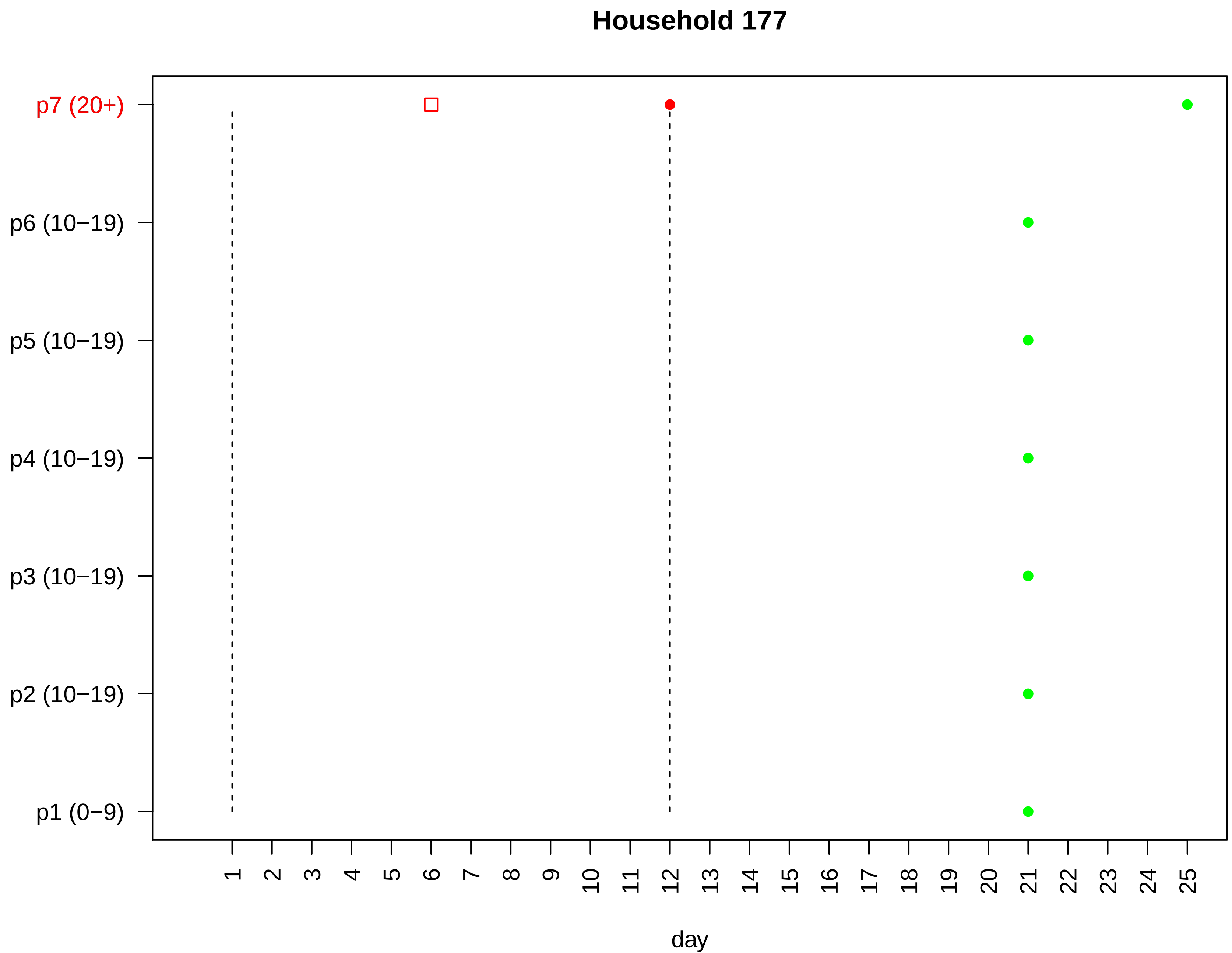

Household 177
p7 (20+)
p6 (10−19)
p5 (10−19)
p4 (10−19)
p3 (10−19)
p2 (10−19)
p1 (0−9)
1
2
3
4
5
6
7
8
9
10
11
12
13
14
15
16
17
18
19
20
21
22
23
24
25
day

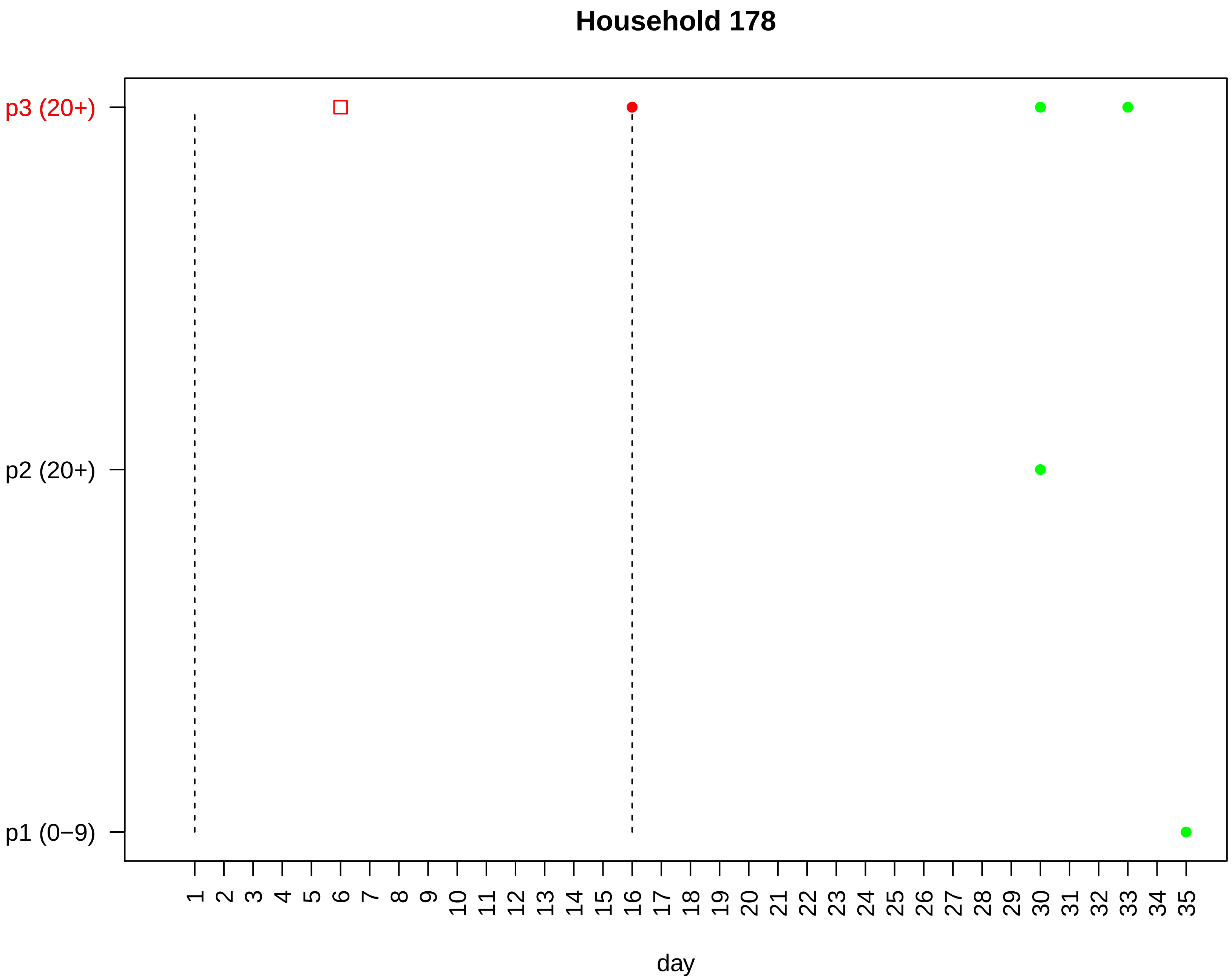

Household 178
p3 (20+)
p2 (20+)
p1 (0−9)
1 2 3 4 5 6 7 8 9 10 11 12 13 14 15 16 17 18 19 20 21 22 23 24 25 26 27 28 29 30 31 32 33 34 35
day

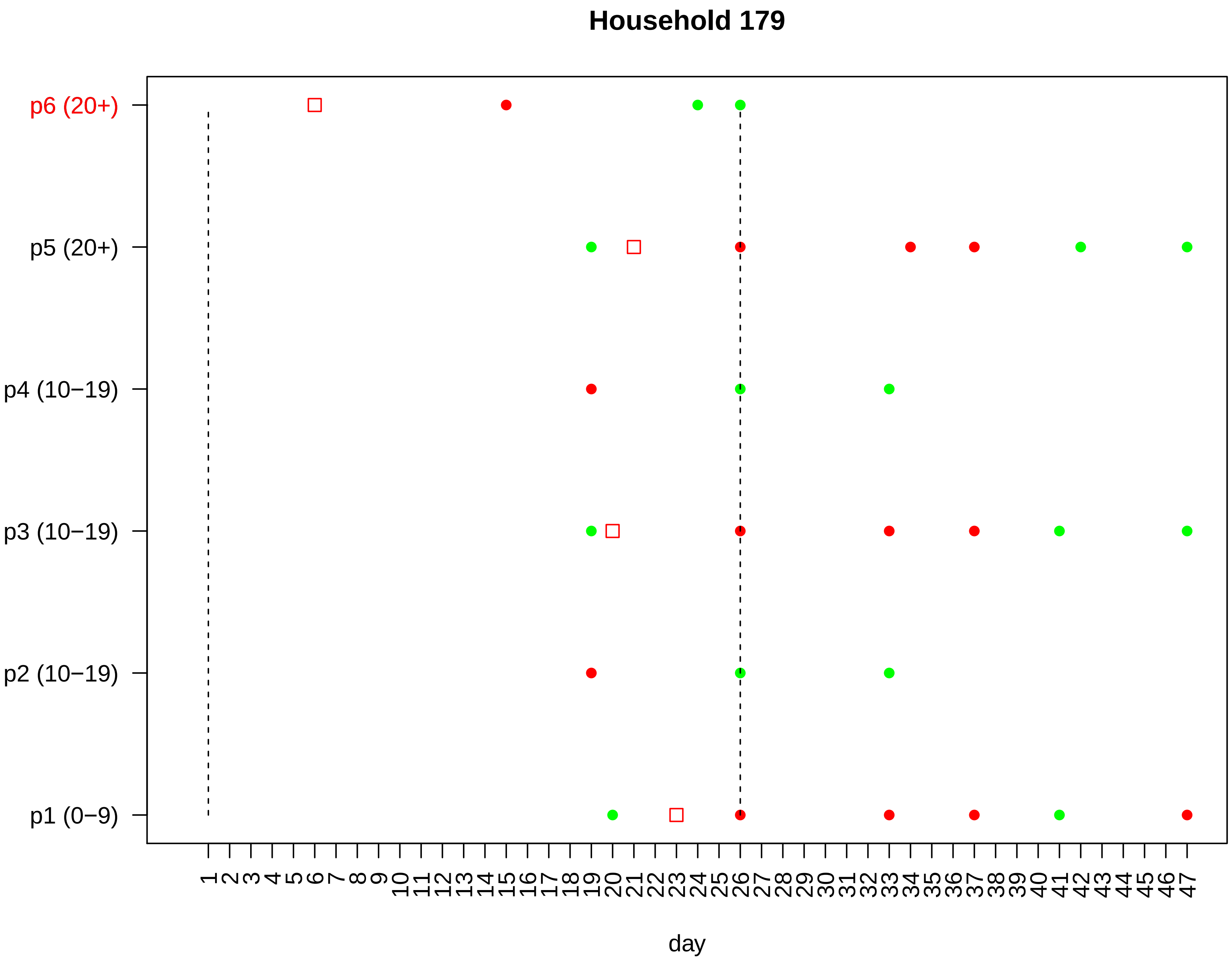
Household 179
p6 (20+)
p5 (20+)
p4 (10−19)
p3 (10−19)
p2 (10−19)
p1 (0−9)
1 2 3 4 5 6 7 8 9 10 11 12 13 14 15 16 17 18 19 20 21 22 23 24 25 26 27 28 29 30 31 32 33 34 35 36 37 38 39 40 41 42 43 44 45 46 47
day

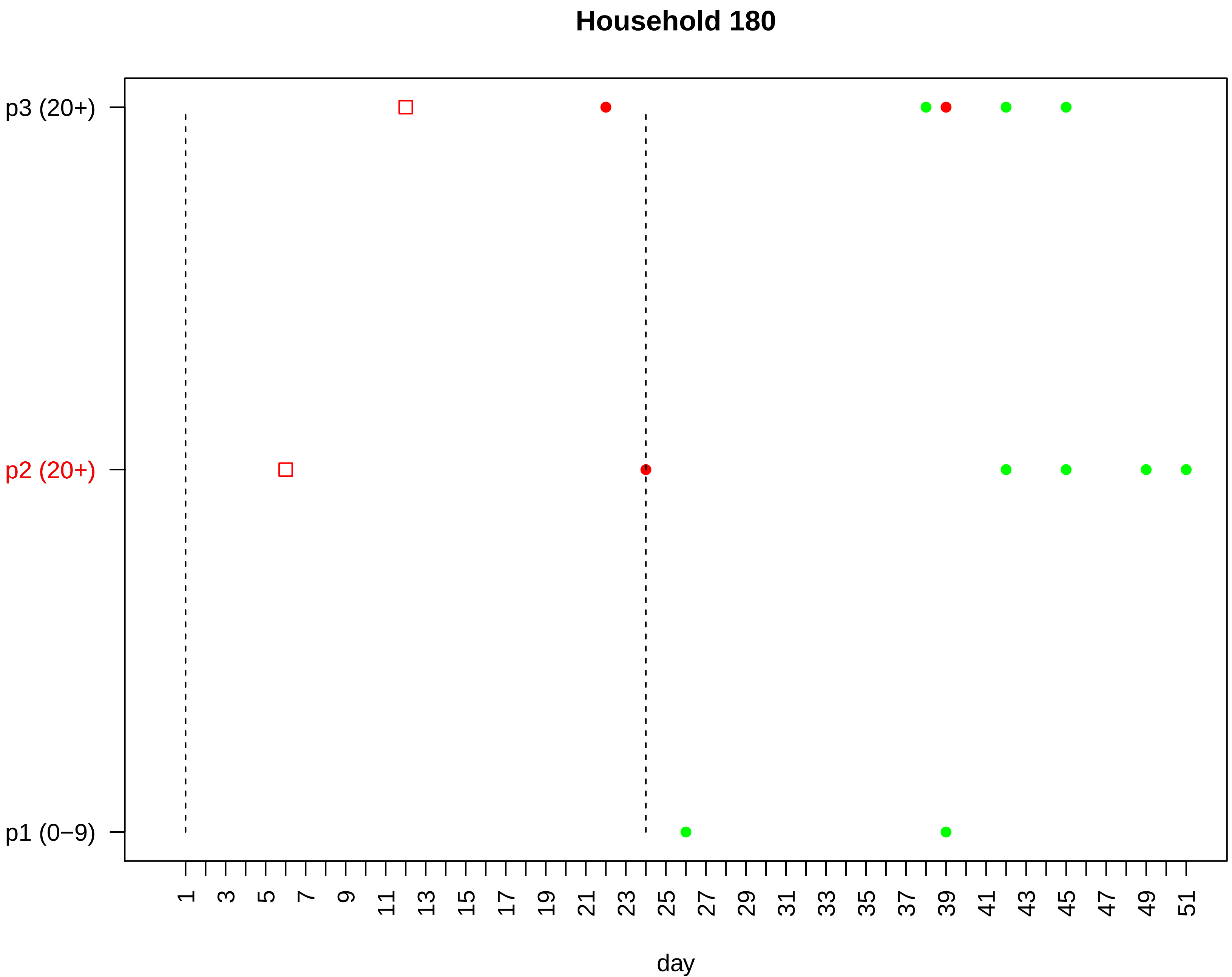

Household 180
p3 (20+)
p2 (20+)
p1 (0−9)
1
3
5
7
9
11
13
15
17
19
21
23
25
27
29
31
33
35
37
39
41
43
45
47
49
51
day

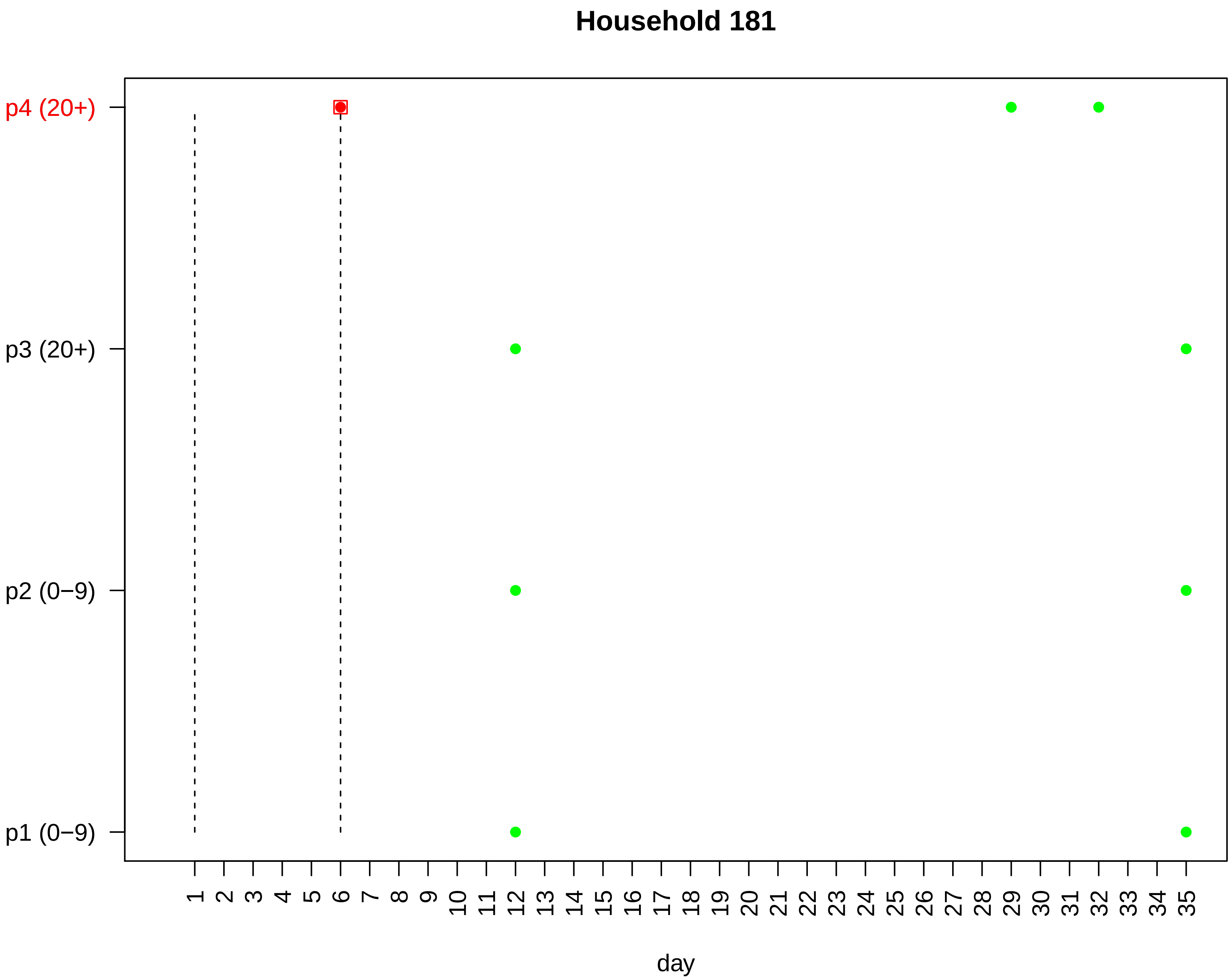

Household 181
p4 (20+)
p3 (20+)
p2 (0−9)
p1 (0−9)
1 2 3 4 5 6 7 8 9 10 11 12 13 14 15 16 17 18 19 20 21 22 23 24 25 26 27 28 29 30 31 32 33 34 35
day

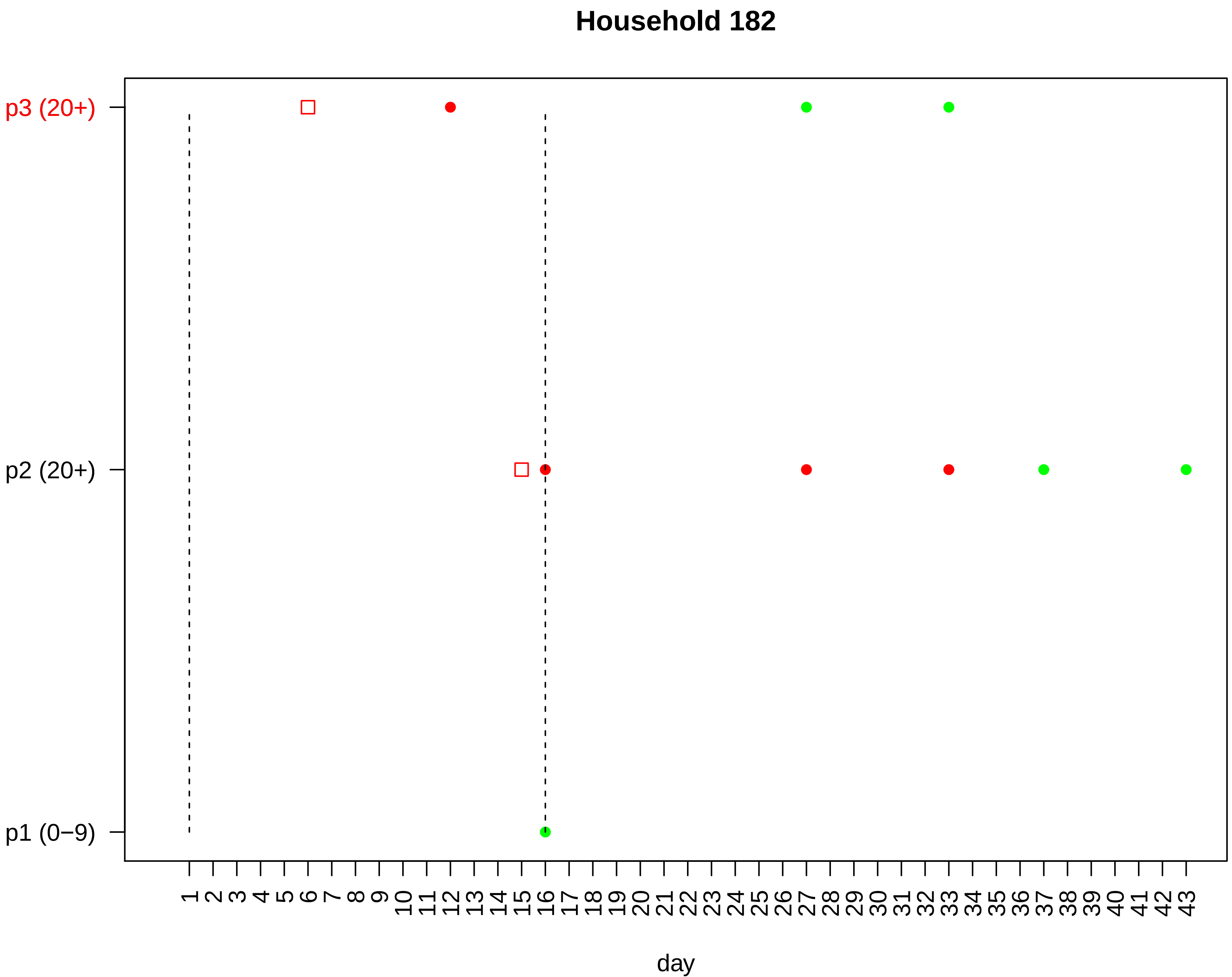

Household 182
p3 (20+)
p2 (20+)
p1 (0−9)
1 2 3 4 5 6 7 8 9 10 11 12 13 14 15 16 17 18 19 20 21 22 23 24 25 26 27 28 29 30 31 32 33 34 35 36 37 38 39 40 41 42 43
day

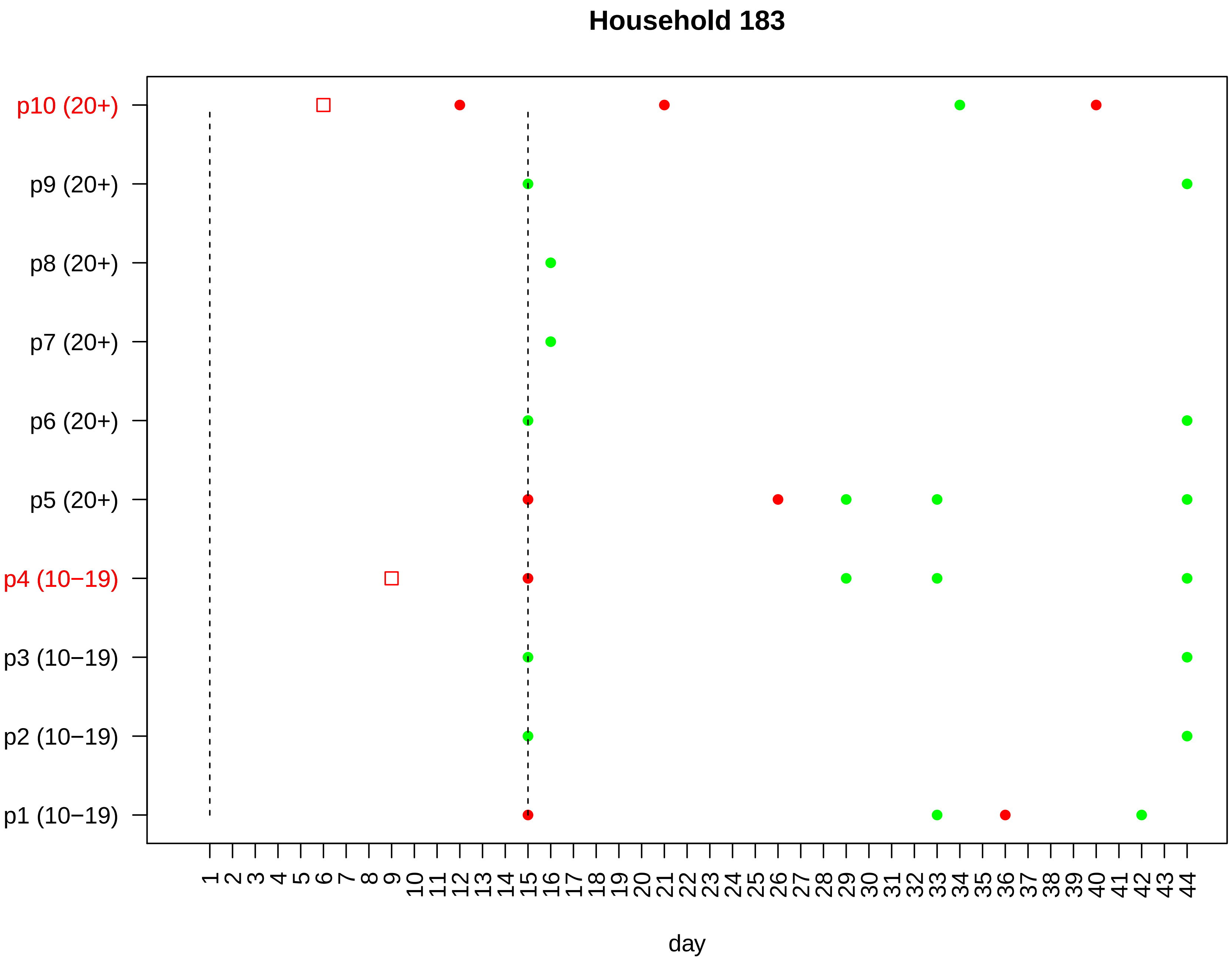

Household 183
p10 (20+)
p9 (20+)
p8 (20+)
p7 (20+)
p6 (20+)
p5 (20+)
p4 (10−19)
p3 (10−19)
p2 (10−19)
p1 (10−19)
1 2 3 4 5 6 7 8 9 10 11 12 13 14 15 16 17 18 19 20 21 22 23 24 25 26 27 28 29 30 31 32 33 34 35 36 37 38 39 40 41 42 43 44
day

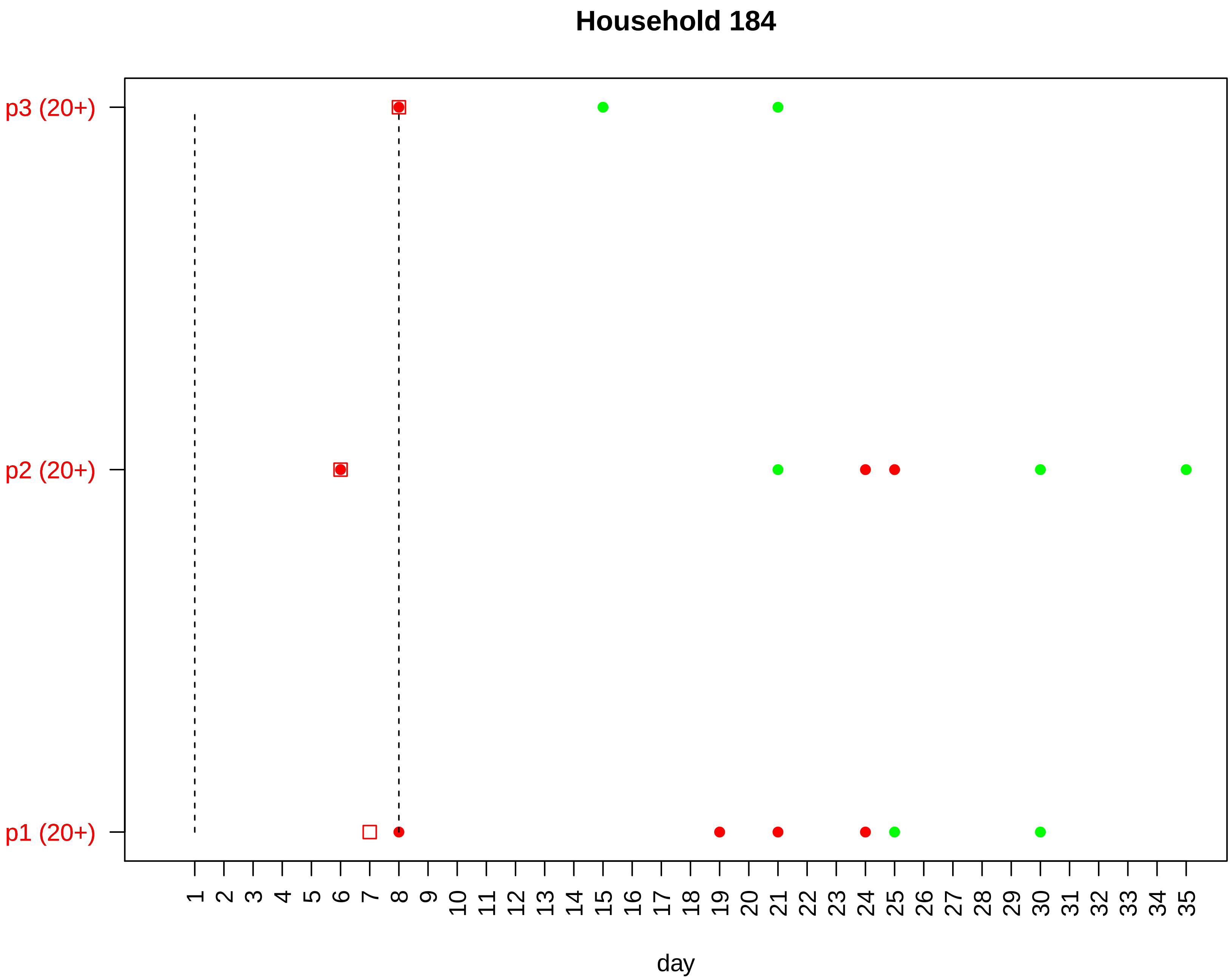

Household 184
p3 (20+)
p2 (20+)
p1 (20+)
1 2 3 4 5 6 7 8 9 10 11 12 13 14 15 16 17 18 19 20 21 22 23 24 25 26 27 28 29 30 31 32 33 34 35
day

Household 185

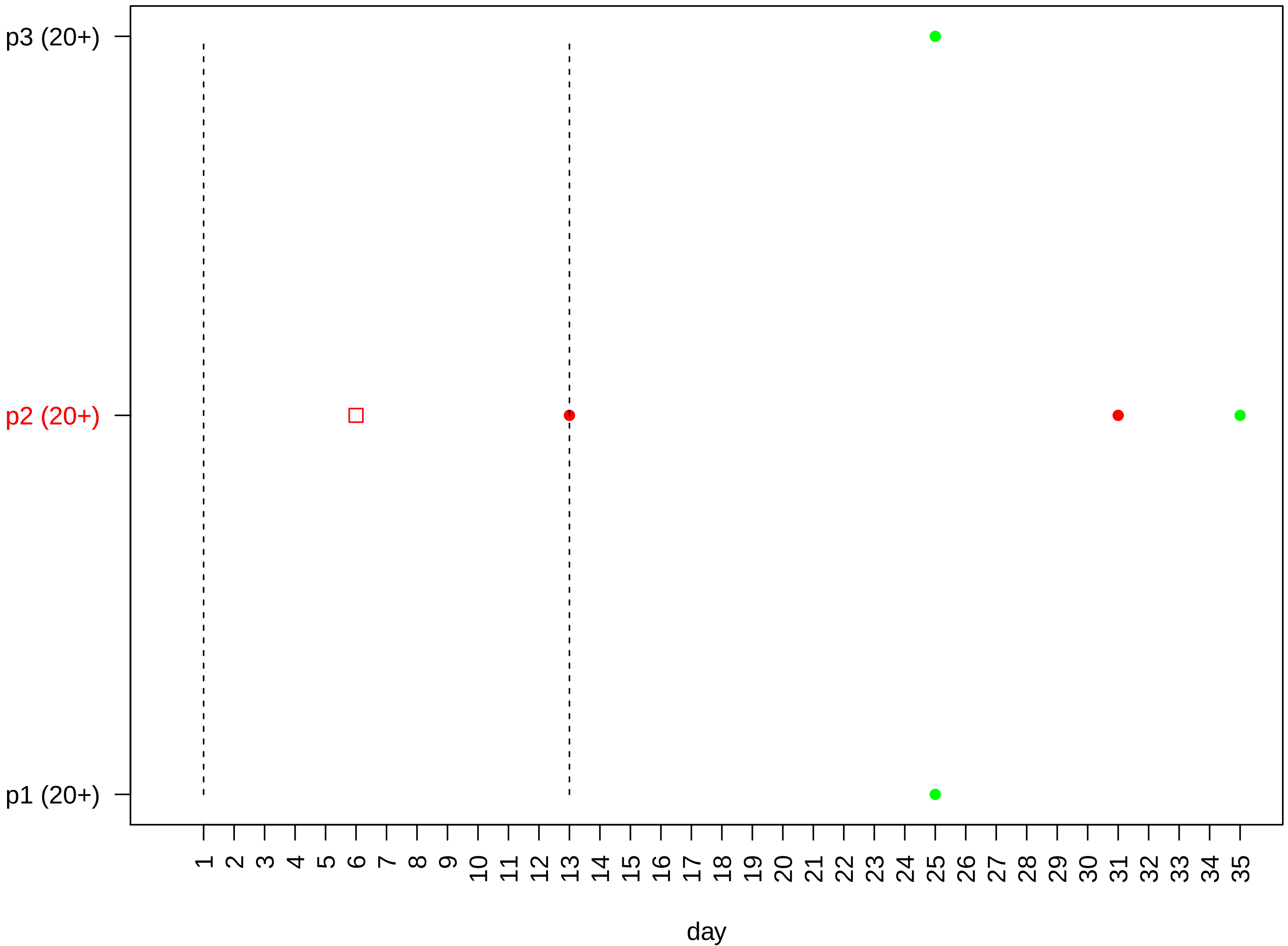

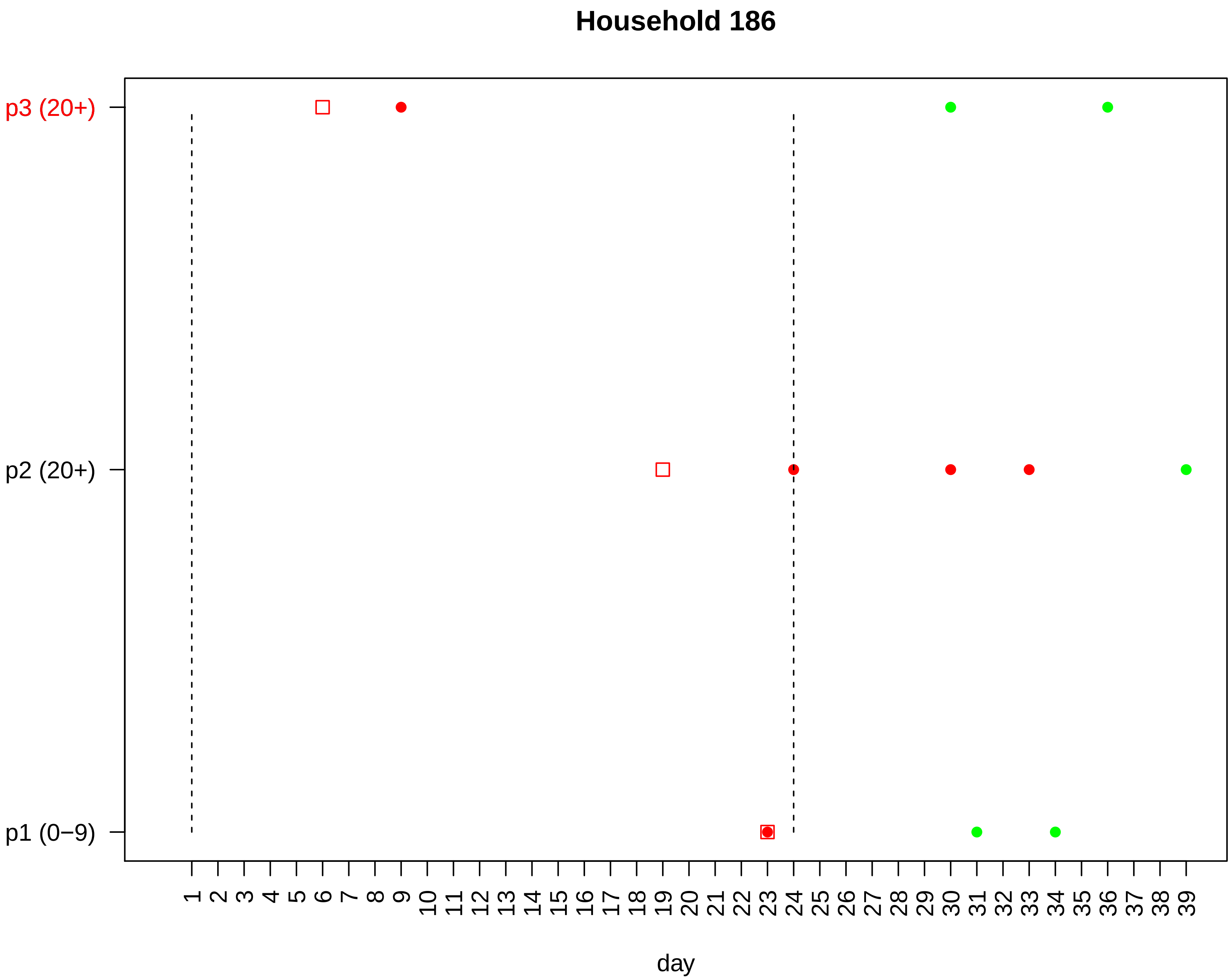
Household 186
p3 (20+)
p2 (20+)
p1 (0−9)
1 2 3 4 5 6 7 8 9 10 11 12 13 14 15 16 17 18 19 20 21 22 23 24 25 26 27 28 29 30 31 32 33 34 35 36 37 38 39
day

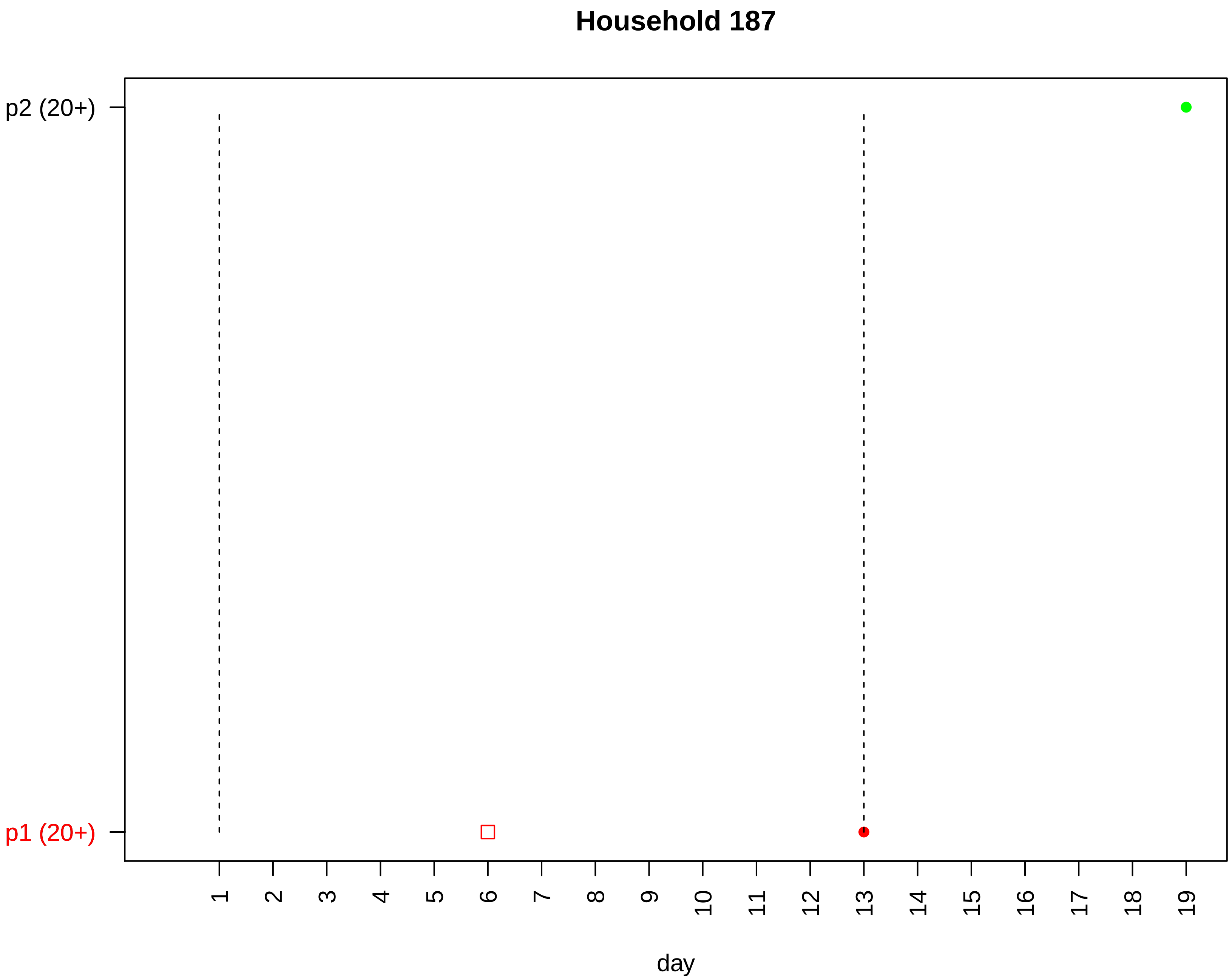
Household 187
p2 (20+)
p1 (20+)
1
2
3
4
5
6
7
8
9
10
11
12
13
14
15
16
17
18
19
day

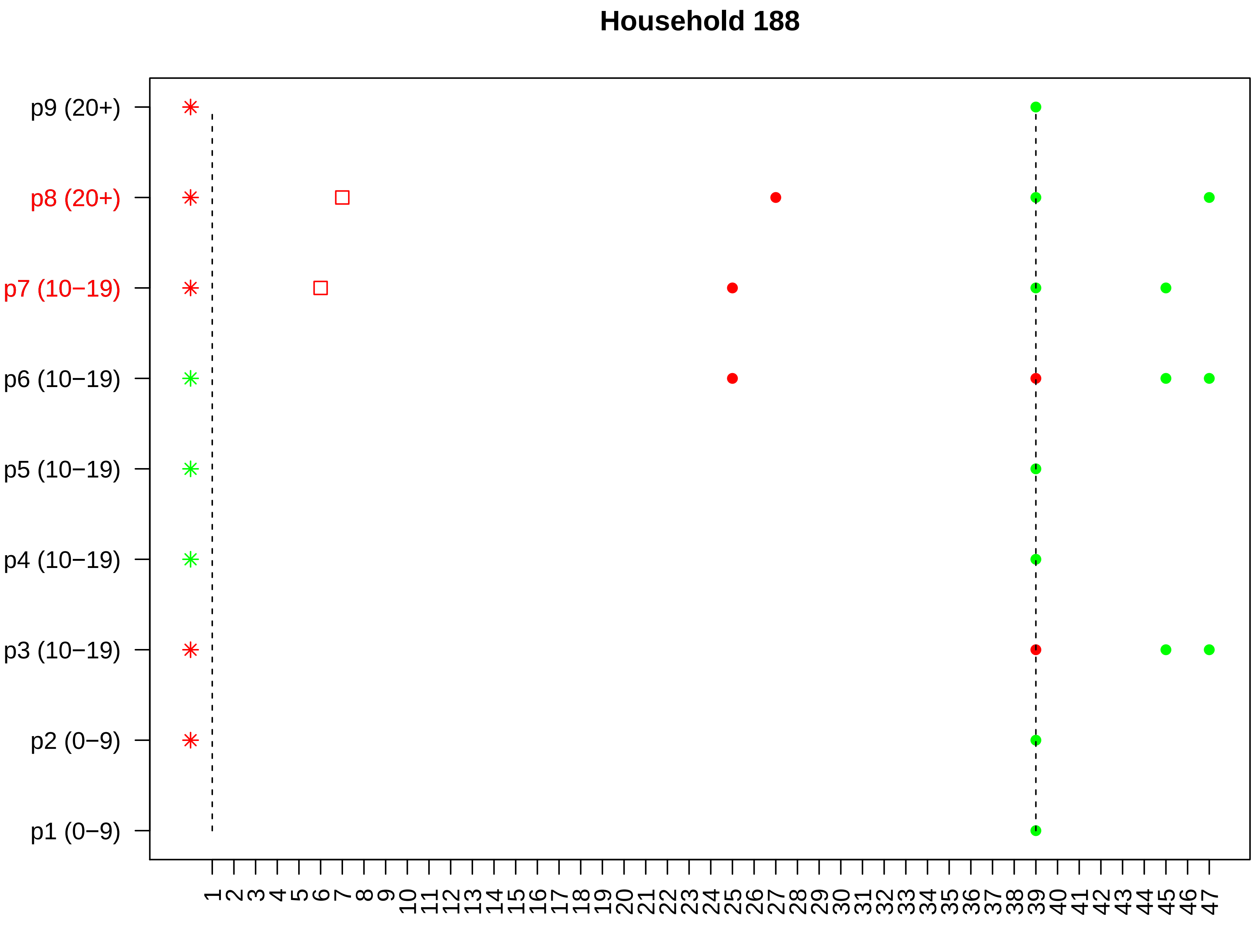
Household 188
p9 (20+)
p8 (20+)
p7 (10−19)
p6 (10−19)
p5 (10−19)
p4 (10−19)
p3 (10−19)
p2 (0−9)
p1 (0−9)
1 2 3 4 5 6 7 8 9 10 11 12 13 14 15 16 17 18 19 20 21 22 23 24 25 26 27 28 29 30 31 32 33 34 35 36 37 38 39 40 41 42 43 44 45 46 47
day

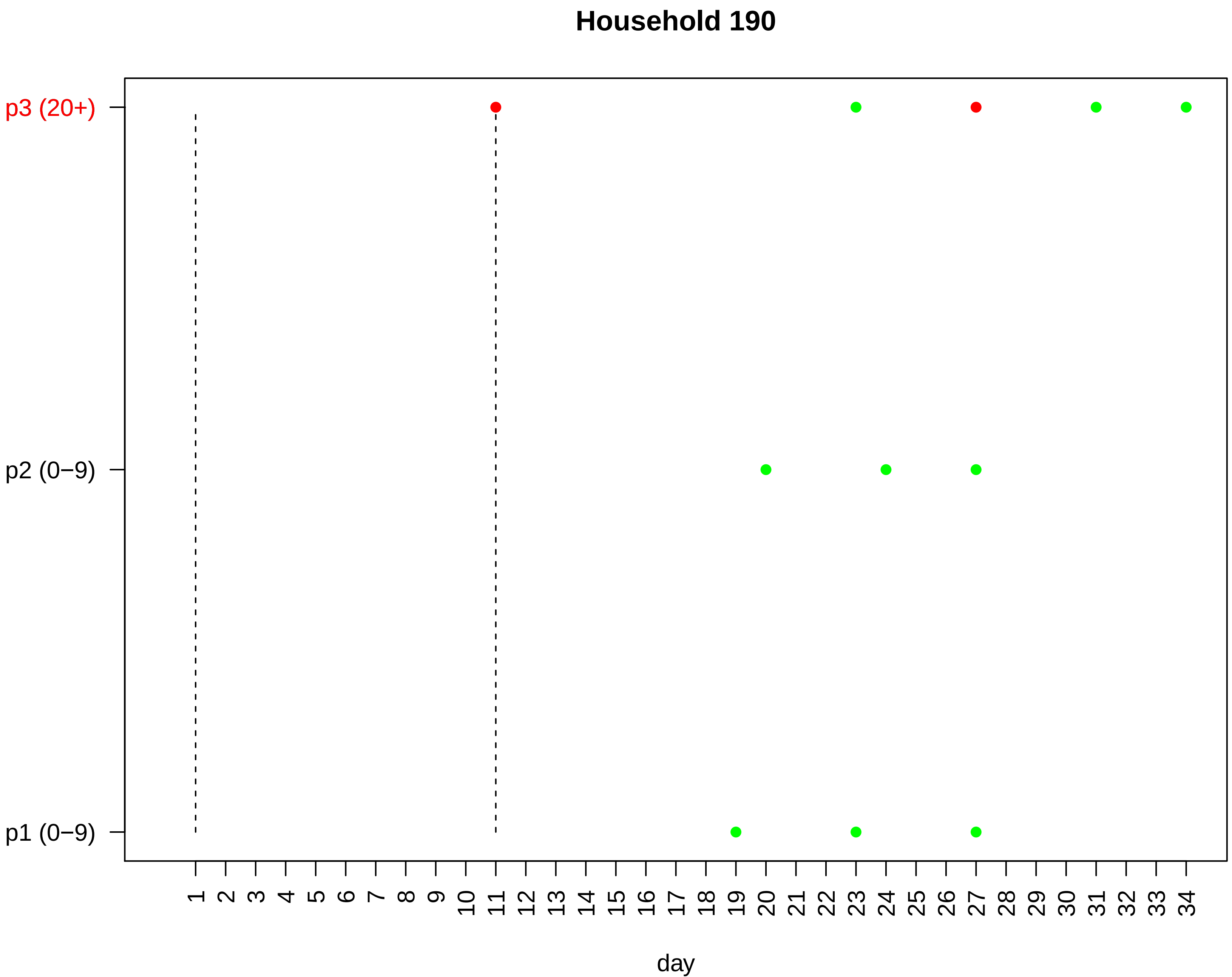

Household 190
p3 (20+)
p2 (0−9)
p1 (0−9)
1
2
3
4
5
6
7
8
9
10
11
12
13
14
15
16
17
18
19
20
21
22
23
24
25
26
27
28
29
30
31
32
33
34
day

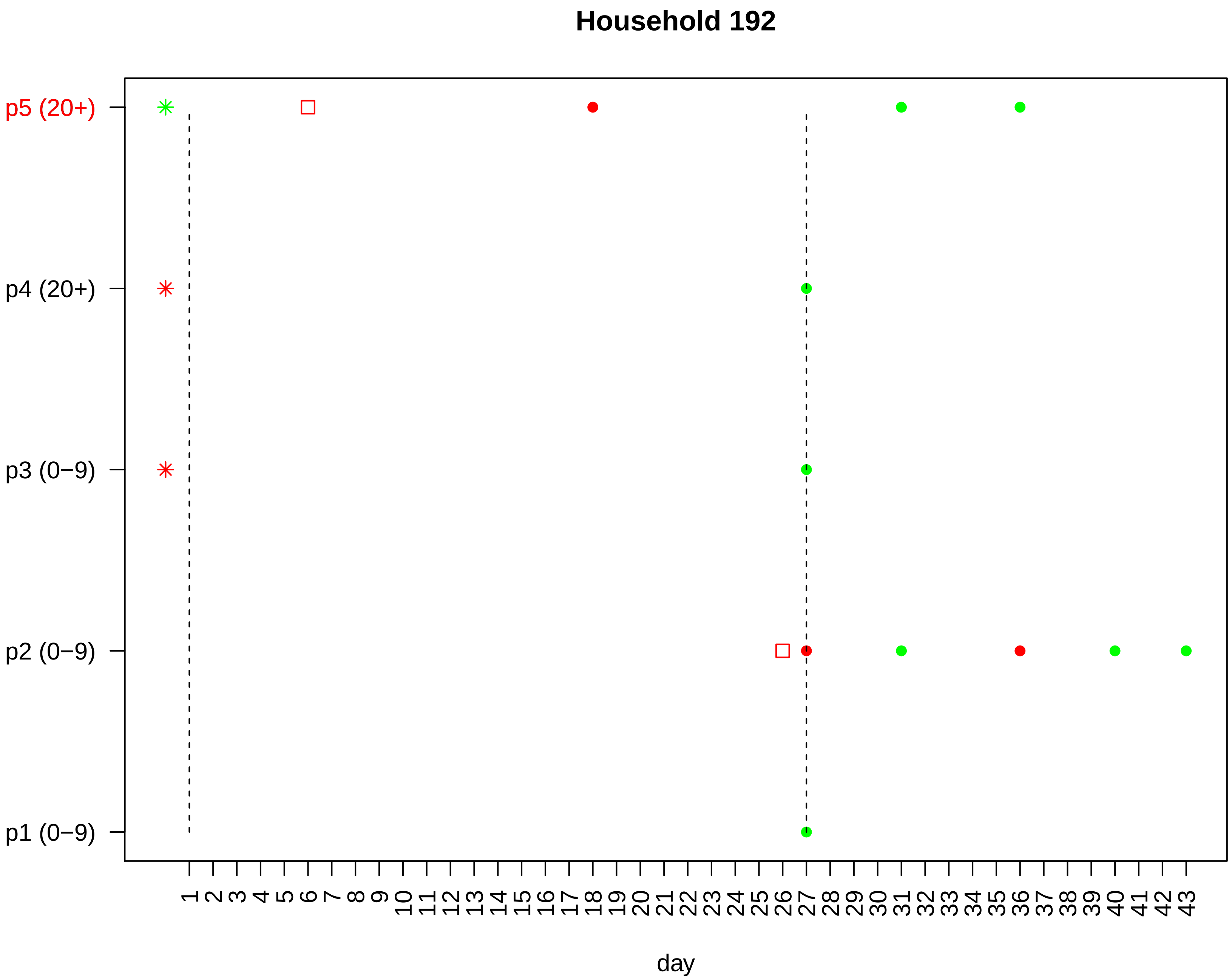

Household 192
p5 (20+)
p4 (20+)
p3 (0−9)
p2 (0−9)
p1 (0−9)
1 2 3 4 5 6 7 8 9 10 11 12 13 14 15 16 17 18 19 20 21 22 23 24 25 26 27 28 29 30 31 32 33 34 35 36 37 38 39 40 41 42 43
day

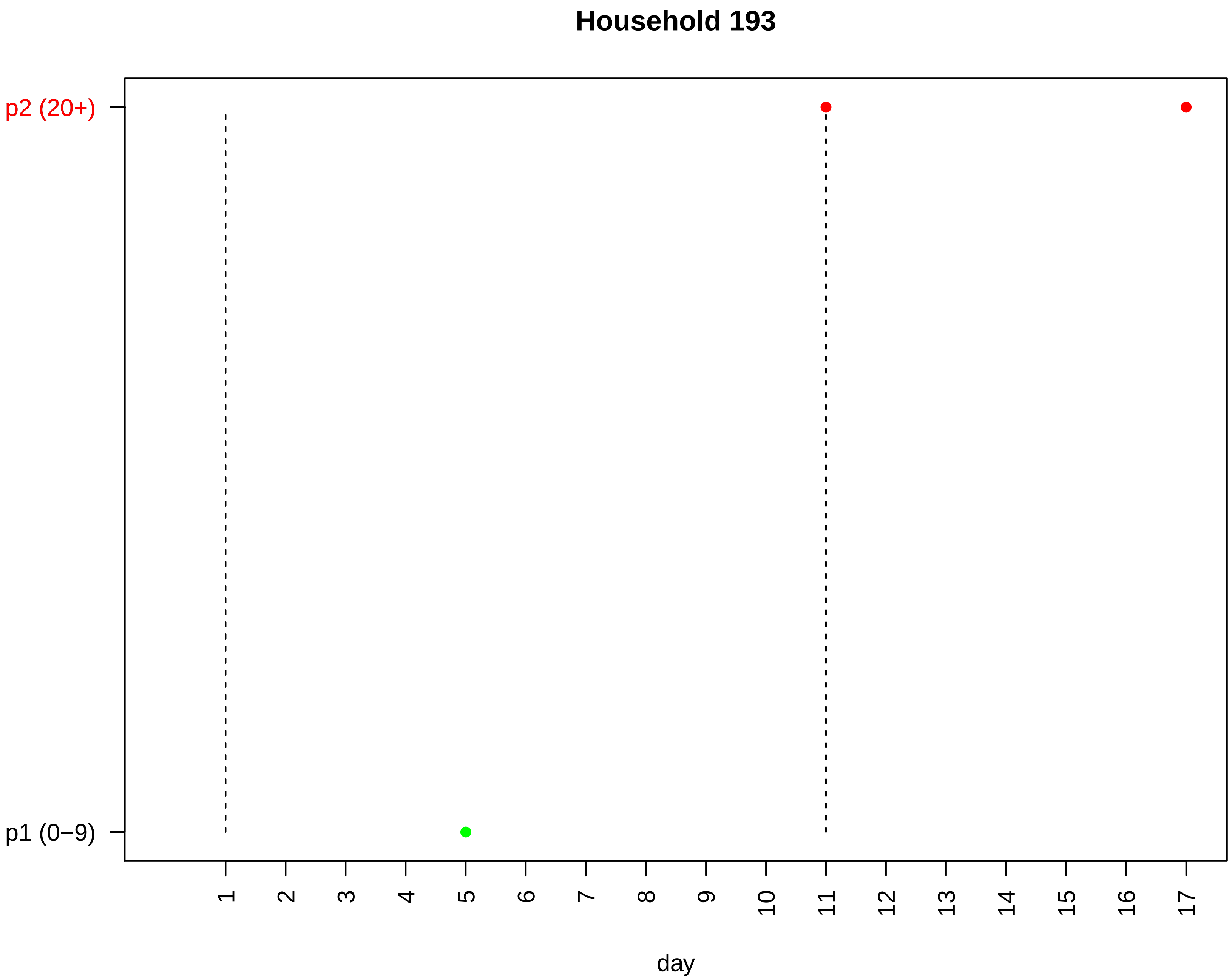

Household 193
p2 (20+)
p1 (0−9)
1
2
3
4
5
6
7
8
9
10
11
12
13
14
15
16
17
day

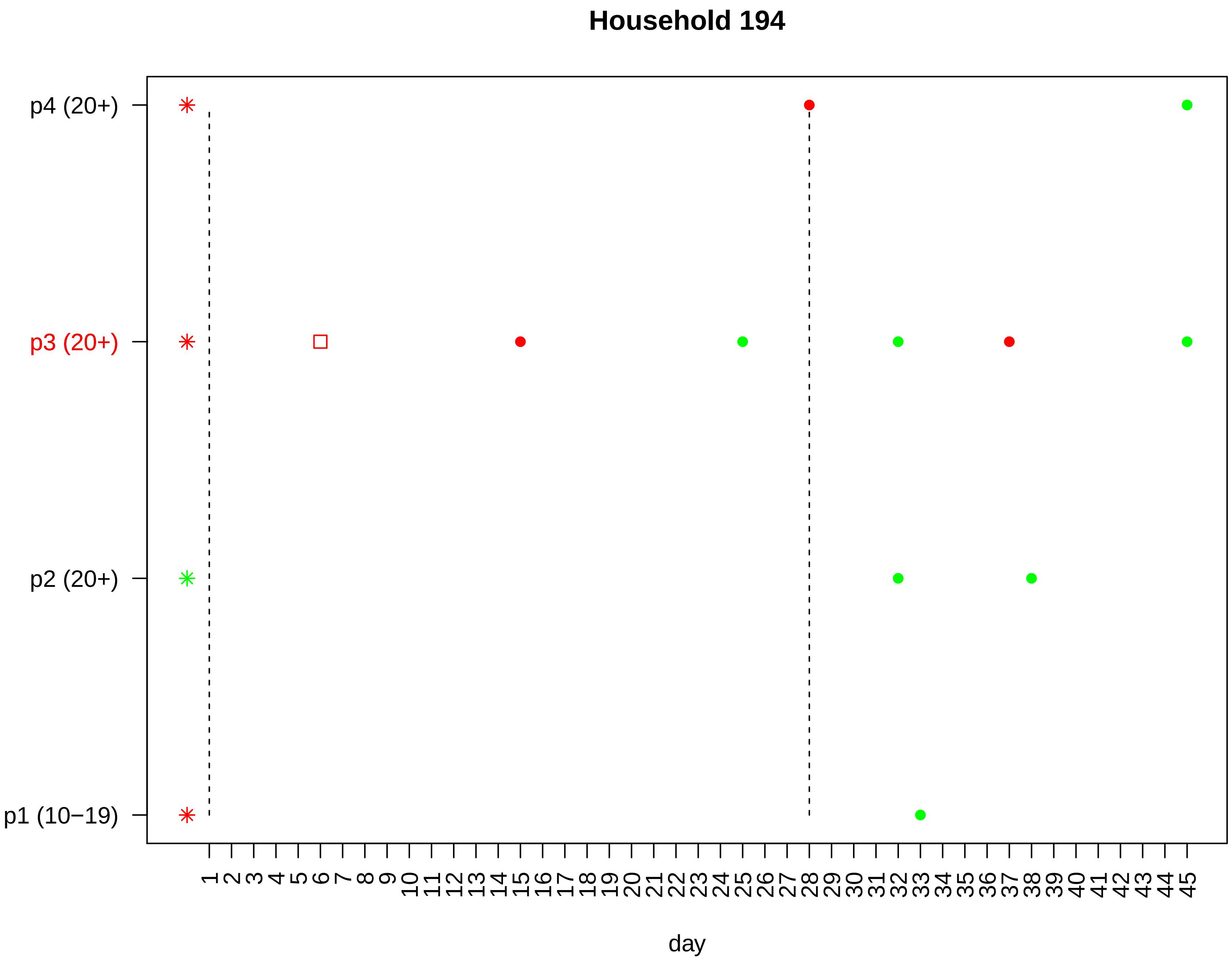
Household 194
p4 (20+)
p3 (20+)
p2 (20+)
p1 (10−19)
1 2 3 4 5 6 7 8 9 10 11 12 13 14 15 16 17 18 19 20 21 22 23 24 25 26 27 28 29 30 31 32 33 34 35 36 37 38 39 40 41 42 43 44 45
day

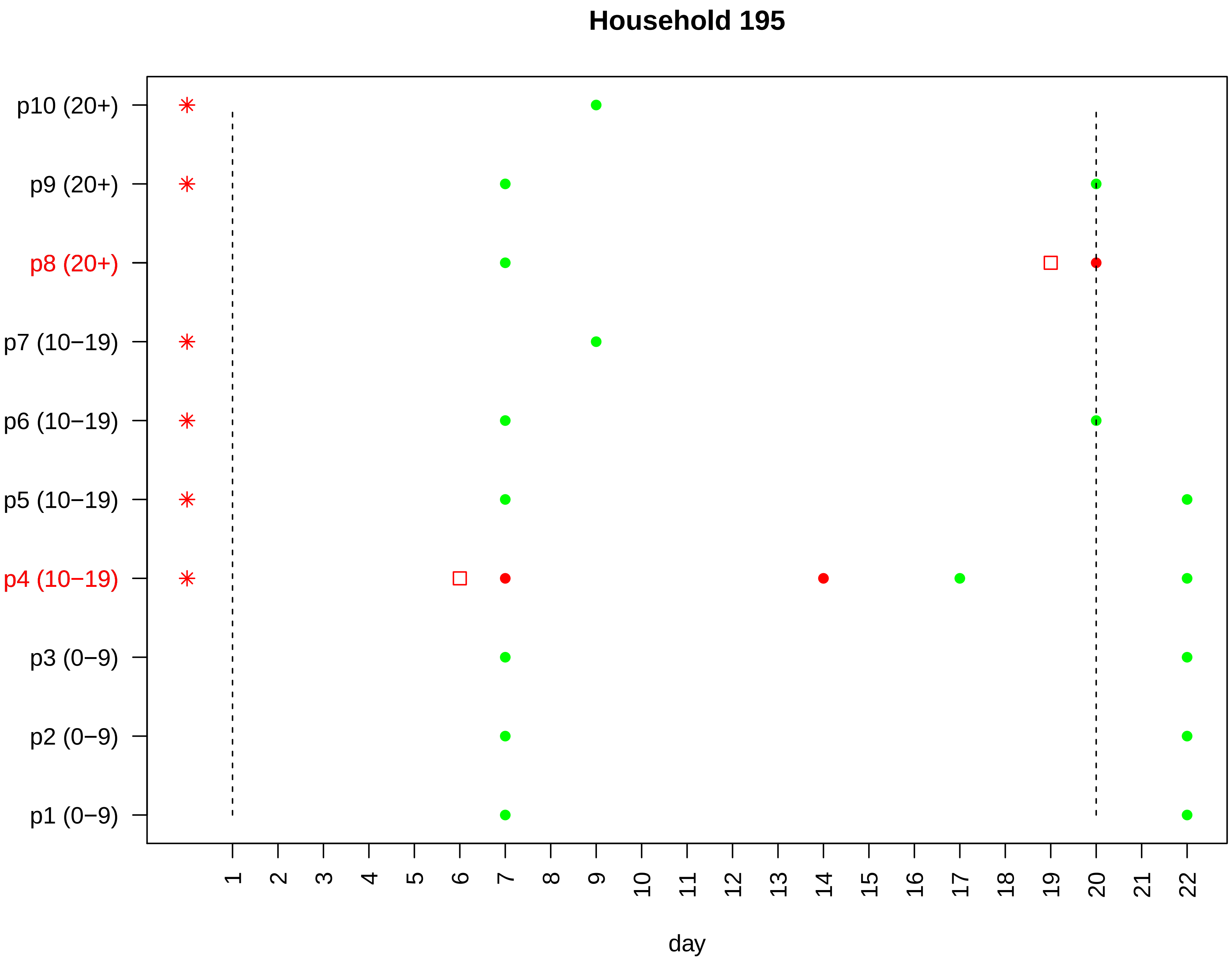

Household 195
p10 (20+)
p9 (20+)
p8 (20+)
p7 (10−19)
p6 (10−19)
p5 (10−19)
p4 (10−19)
p3 (0−9)
p2 (0−9)
p1 (0−9)
1 2 3 4 5 6 7 8 9 10 11 12 13 14 15 16 17 18 19 20 21 22
day

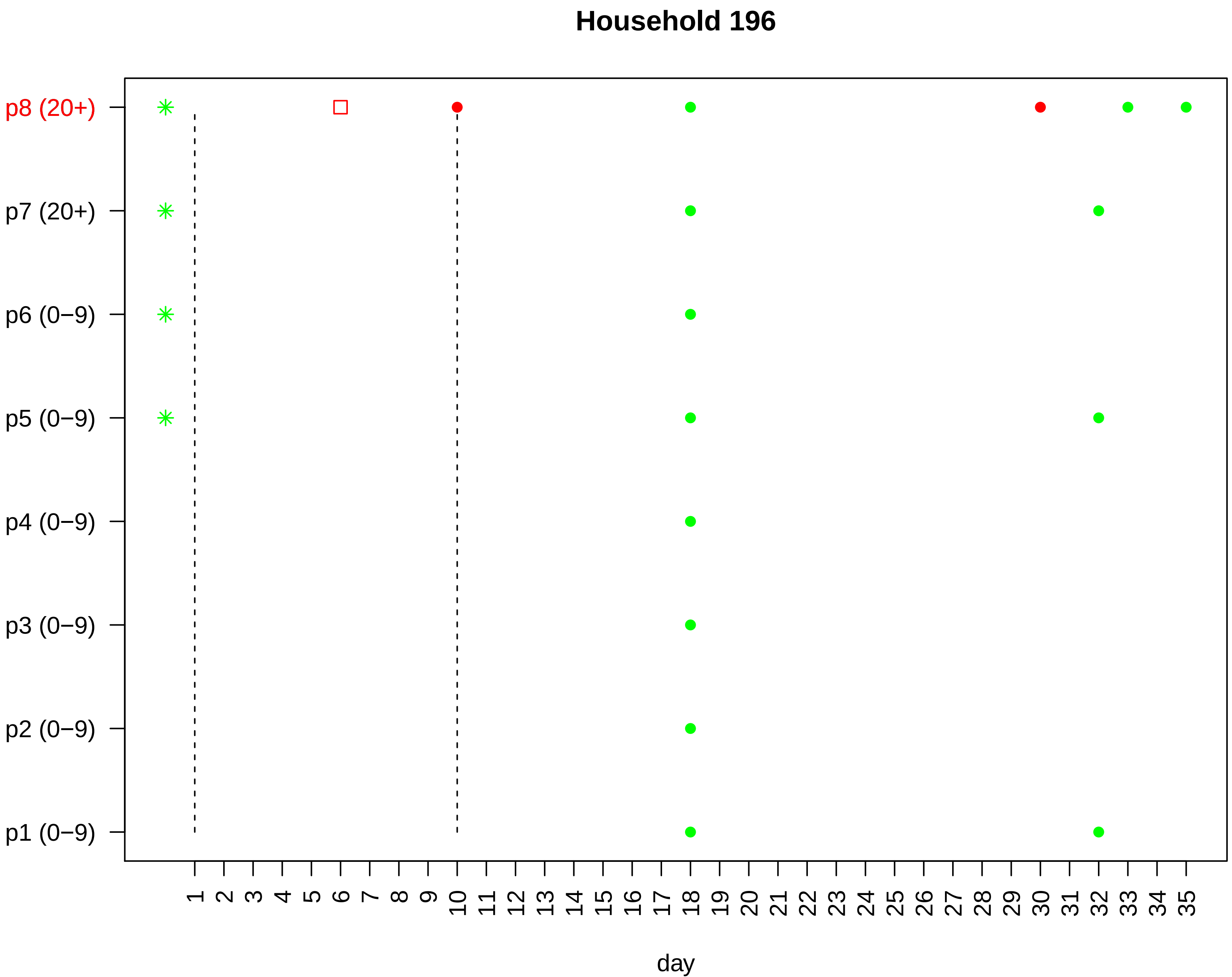
Household 196
p8 (20+)
p7 (20+)
p6 (0−9)
p5 (0−9)
p4 (0−9)
p3 (0−9)
p2 (0−9)
p1 (0−9)
1 2 3 4 5 6 7 8 9 10 11 12 13 14 15 16 17 18 19 20 21 22 23 24 25 26 27 28 29 30 31 32 33 34 35
day

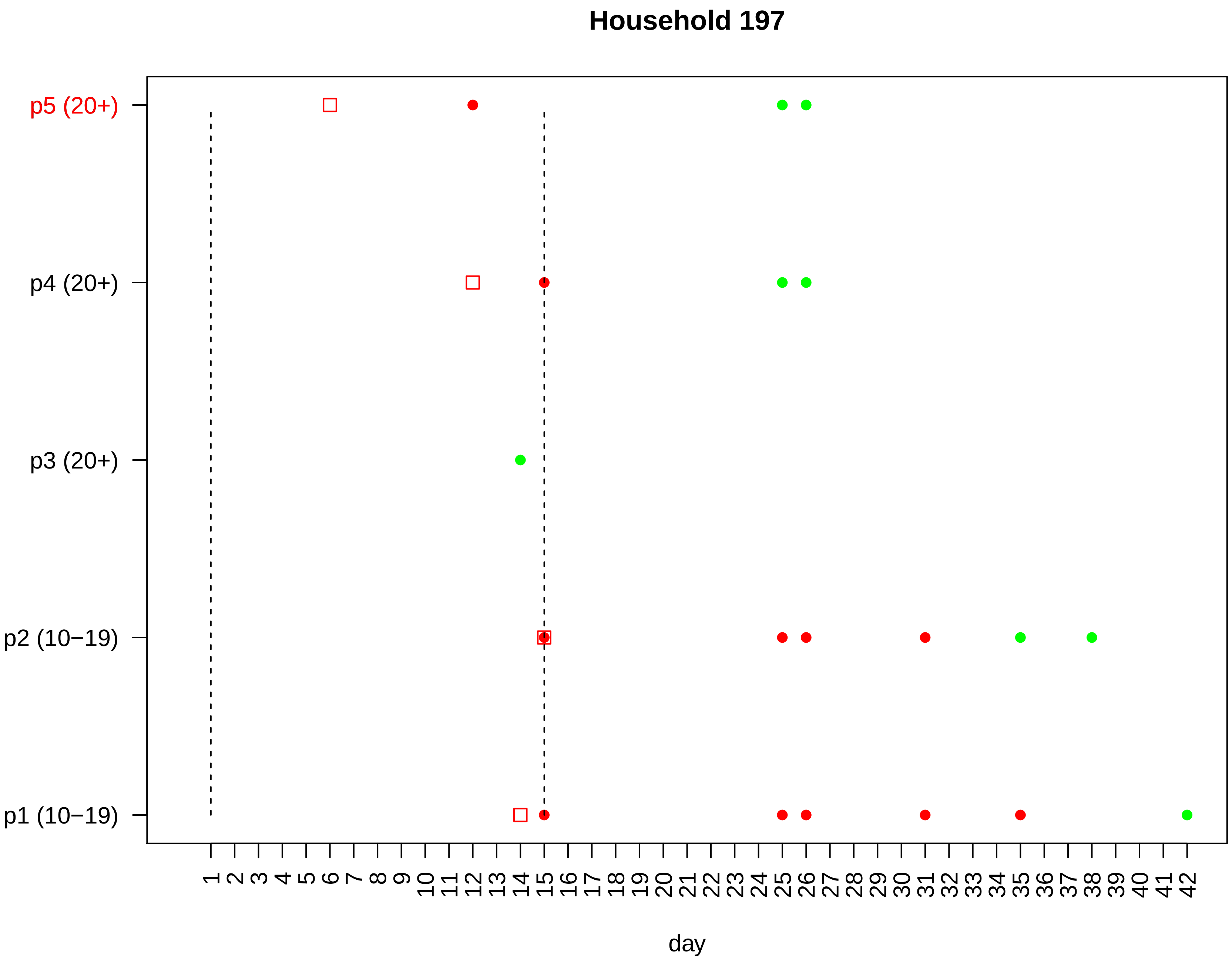

Household 197
p5 (20+)
p4 (20+)
p3 (20+)
p2 (10−19)
p1 (10−19)
1
2
3
4
5
6
7
8
9
10
11
12
13
14
15
16
17
18
19
20
21
22
23
24
25
26
27
28
29
30
31
32
33
34
35
36
37
38
39
40
41
42
day

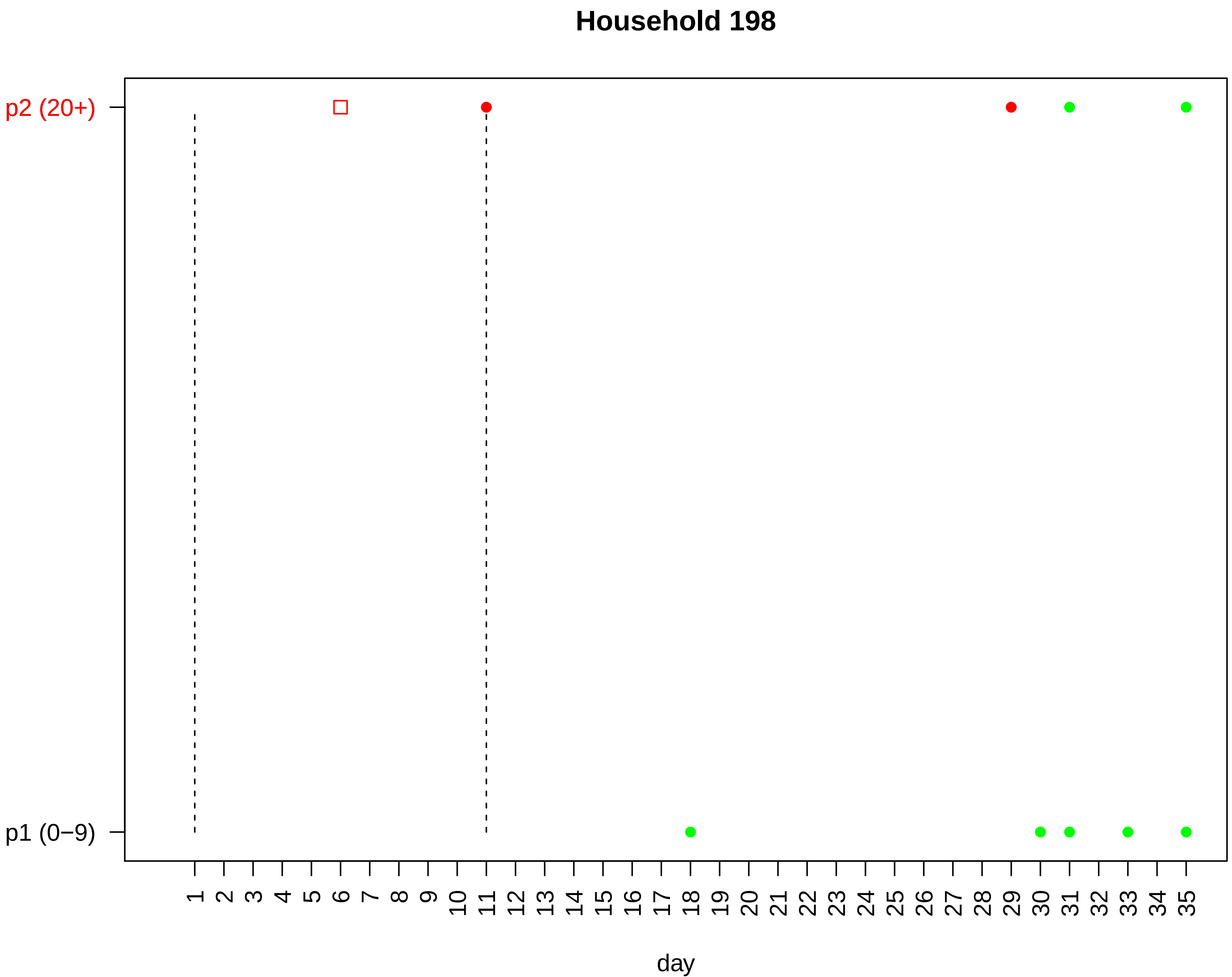

Household 198
p2 (20+)
p1 (0−9)
1
2
3
4
5
6
7
8
9
10
11
12
13
14
15
16
17
18
19
20
21
22
23
24
25
26
27
28
29
30
31
32
33
34
35
day

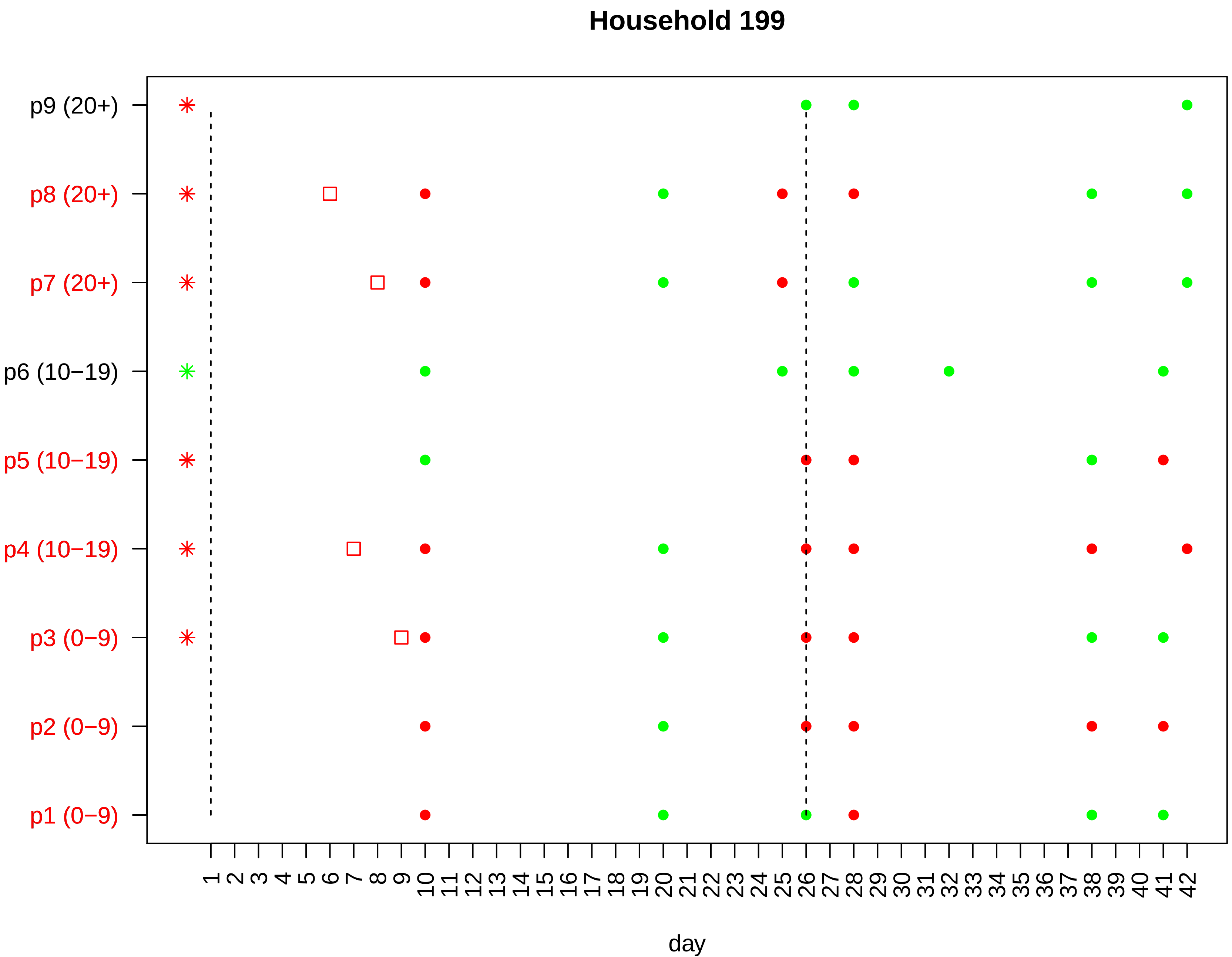
Household 199
p9 (20+)
p8 (20+)
p7 (20+)
p6 (10−19)
p5 (10−19)
p4 (10−19)
p3 (0−9)
p2 (0−9)
p1 (0−9)
1 2 3 4 5 6 7 8 9 10 11 12 13 14 15 16 17 18 19 20 21 22 23 24 25 26 27 28 29 30 31 32 33 34 35 36 37 38 39 40 41 42
day

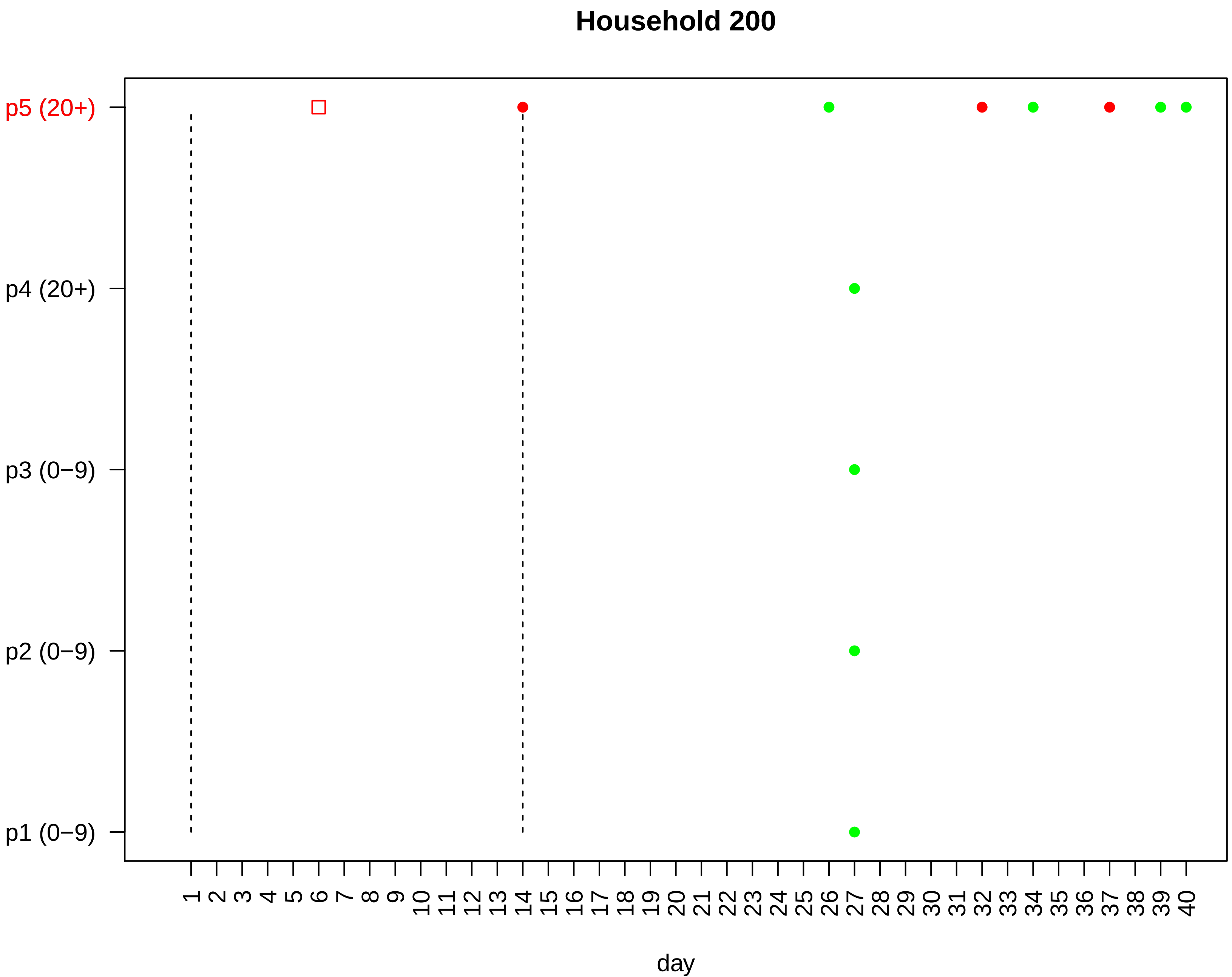

Household 200
p5 (20+)
p4 (20+)
p3 (0−9)
p2 (0−9)
p1 (0−9)
1 2 3 4 5 6 7 8 9 10 11 12 13 14 15 16 17 18 19 20 21 22 23 24 25 26 27 28 29 30 31 32 33 34 35 36 37 38 39 40
day

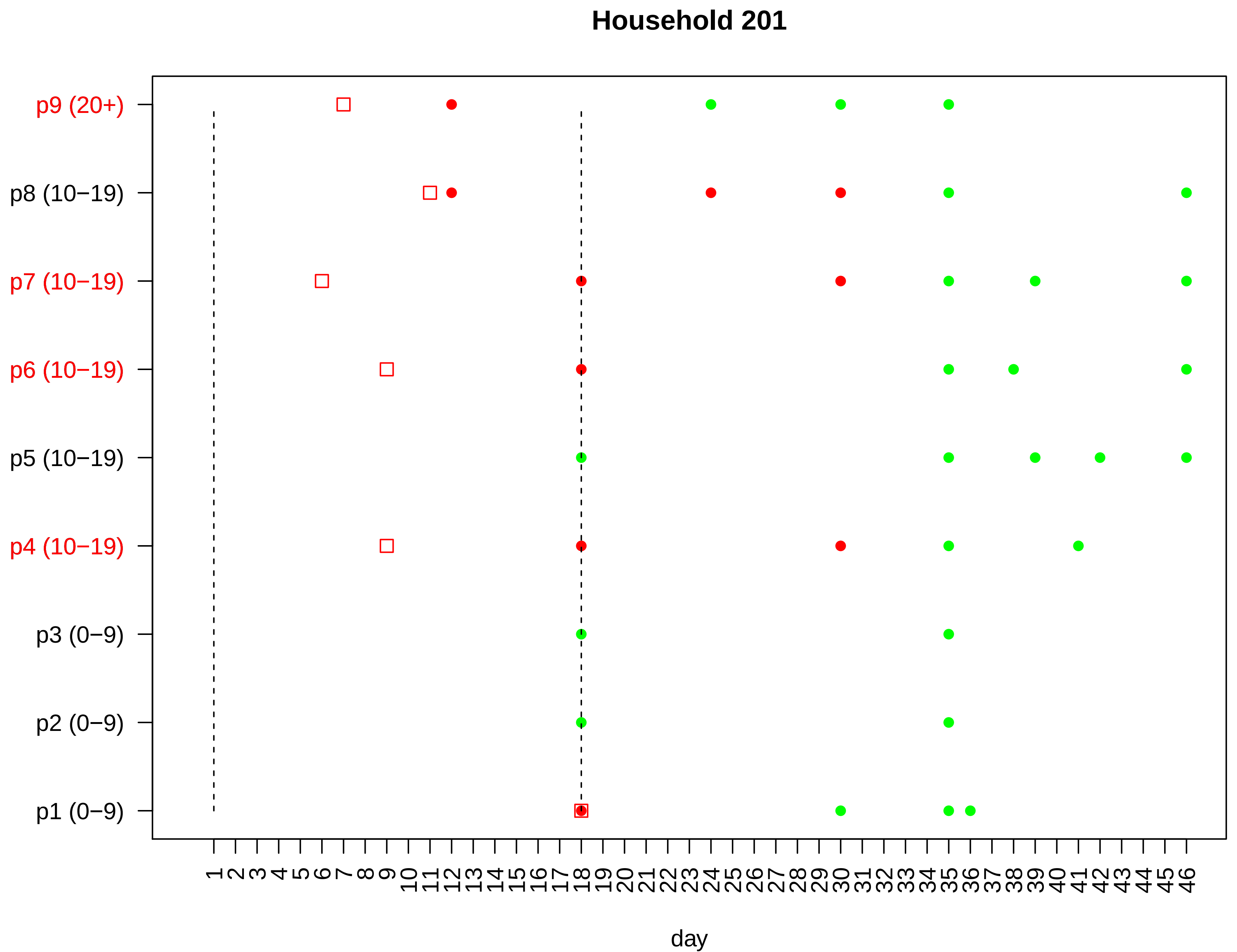
Household 201
p9 (20+)
p8 (10−19)
p7 (10−19)
p6 (10−19)
p5 (10−19)
p4 (10−19)
p3 (0−9)
p2 (0−9)
p1 (0−9)
1 2 3 4 5 6 7 8 9 10 11 12 13 14 15 16 17 18 19 20 21 22 23 24 25 26 27 28 29 30 31 32 33 34 35 36 37 38 39 40 41 42 43 44 45 46
day

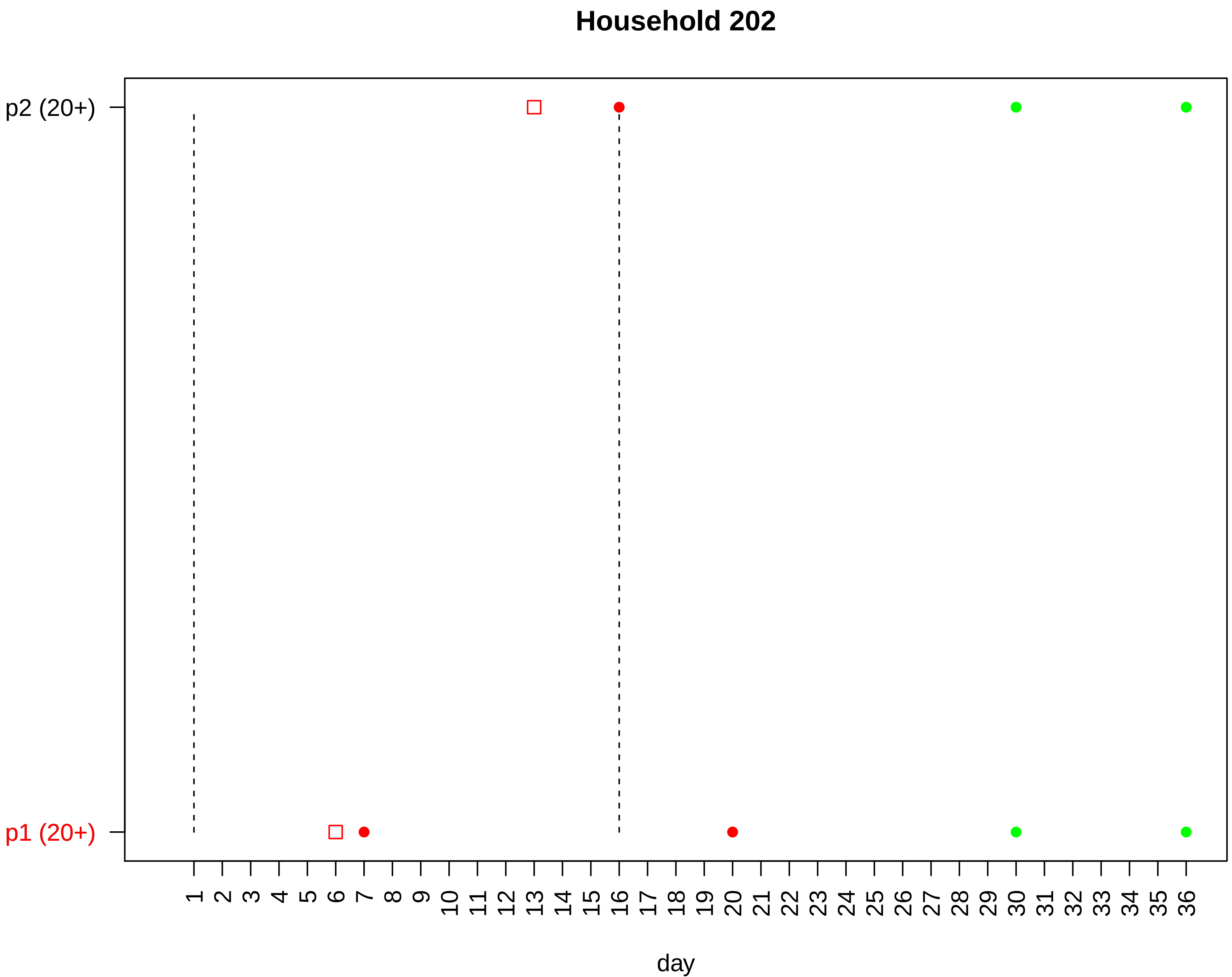
Household 202
p2 (20+)
p1 (20+)
1
2
3
4
5
6
7
8
9
10
11
12
13
14
15
16
17
18
19
20
21
22
23
24
25
26
27
28
29
30
31
32
33
34
35
36
day

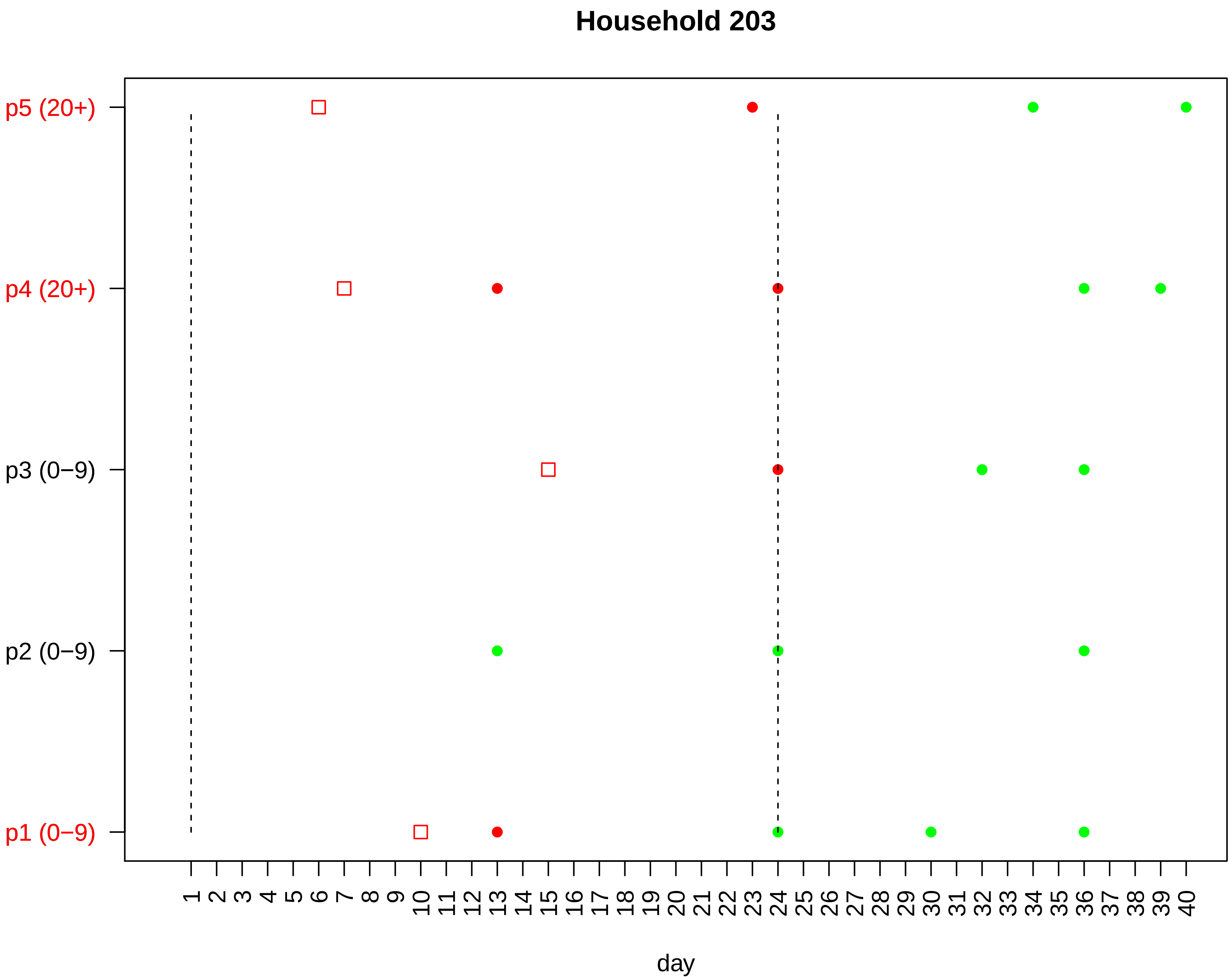

Household 203
p5 (20+)
p4 (20+)
p3 (0−9)
p2 (0−9)
p1 (0−9)
1 2 3 4 5 6 7 8 9 10 11 12 13 14 15 16 17 18 19 20 21 22 23 24 25 26 27 28 29 30 31 32 33 34 35 36 37 38 39 40
day

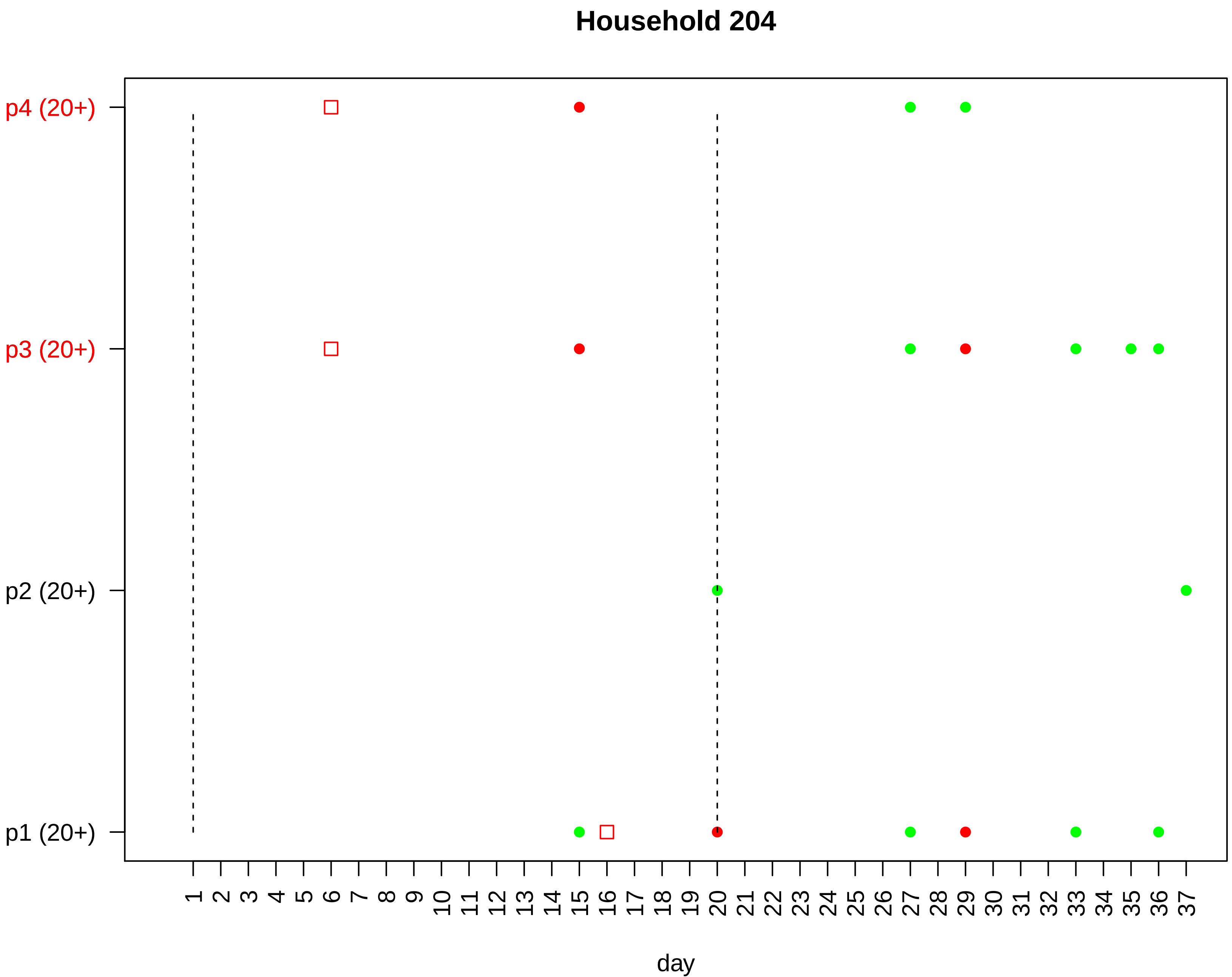

Household 204
p4 (20+)
p3 (20+)
p2 (20+)
p1 (20+)
1 2 3 4 5 6 7 8 9 10 11 12 13 14 15 16 17 18 19 20 21 22 23 24 25 26 27 28 29 30 31 32 33 34 35 36 37
day

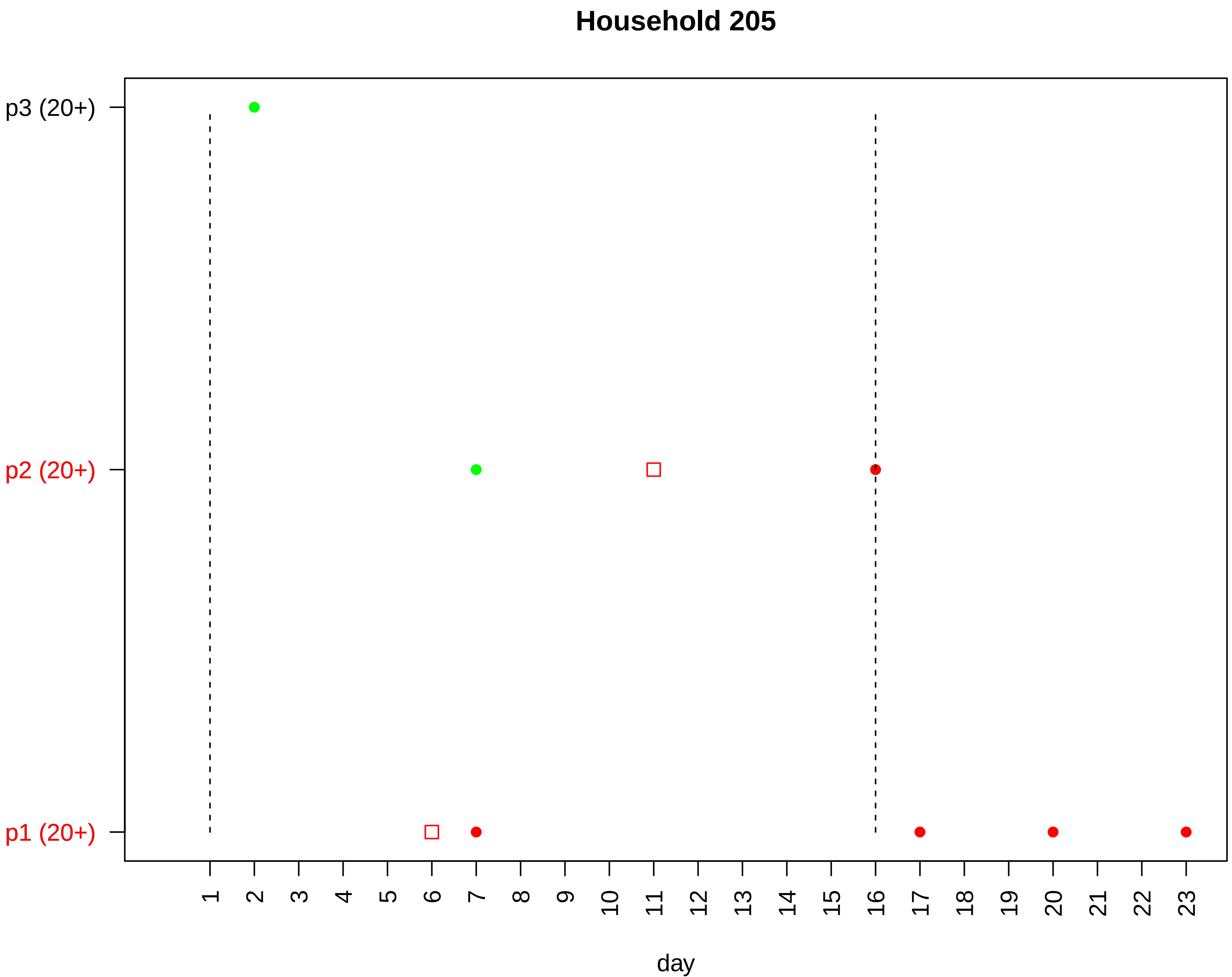

Household 205
p3 (20+)
p2 (20+)
p1 (20+)
1
2
3
4
5
6
7
8
9
10
11
12
13
14
15
16
17
18
19
20
21
22
23
day

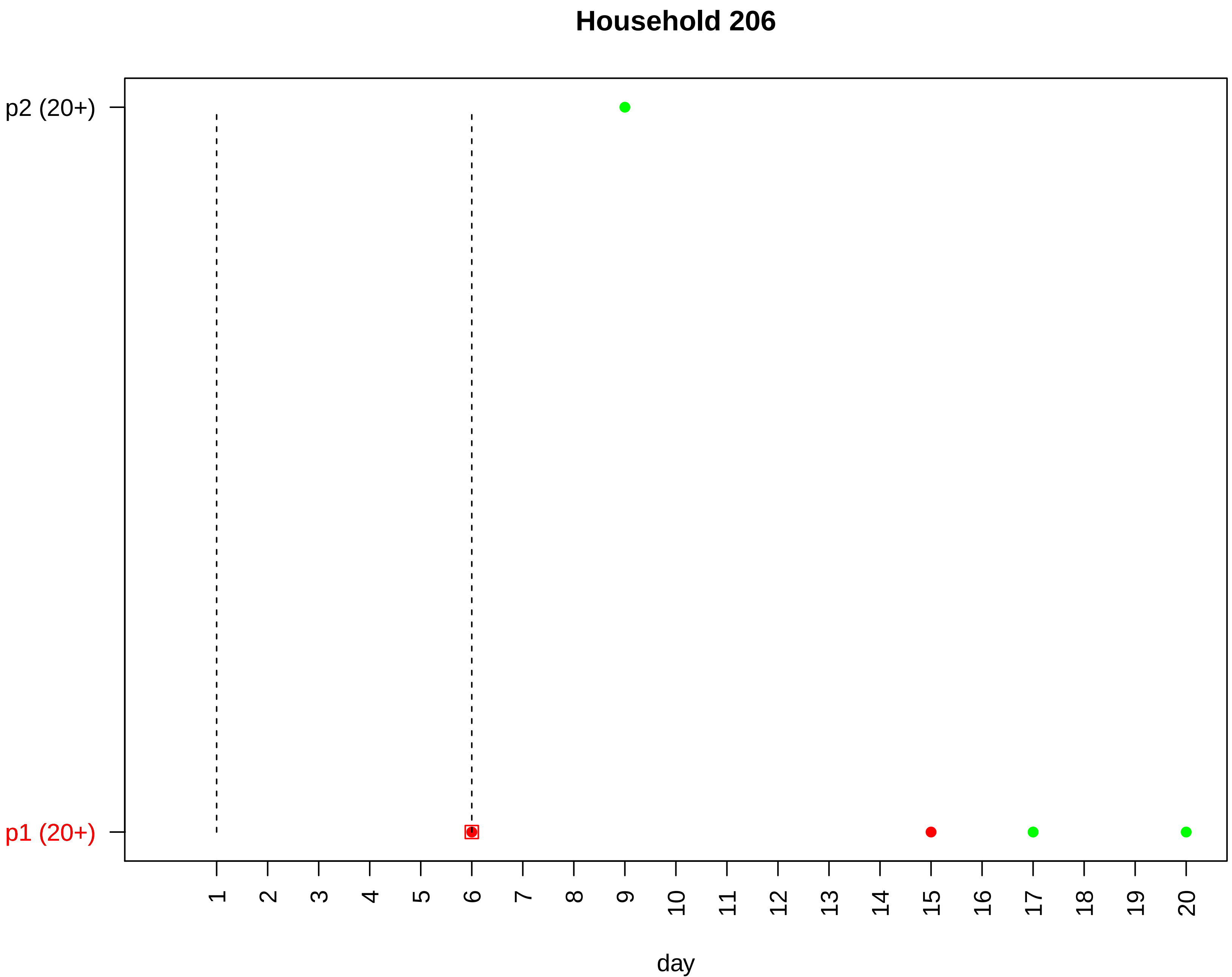

Household 206
p2 (20+)
p1 (20+)
1
2
3
4
5
6
7
8
9
10
11
12
13
14
15
16
17
18
19
20
day

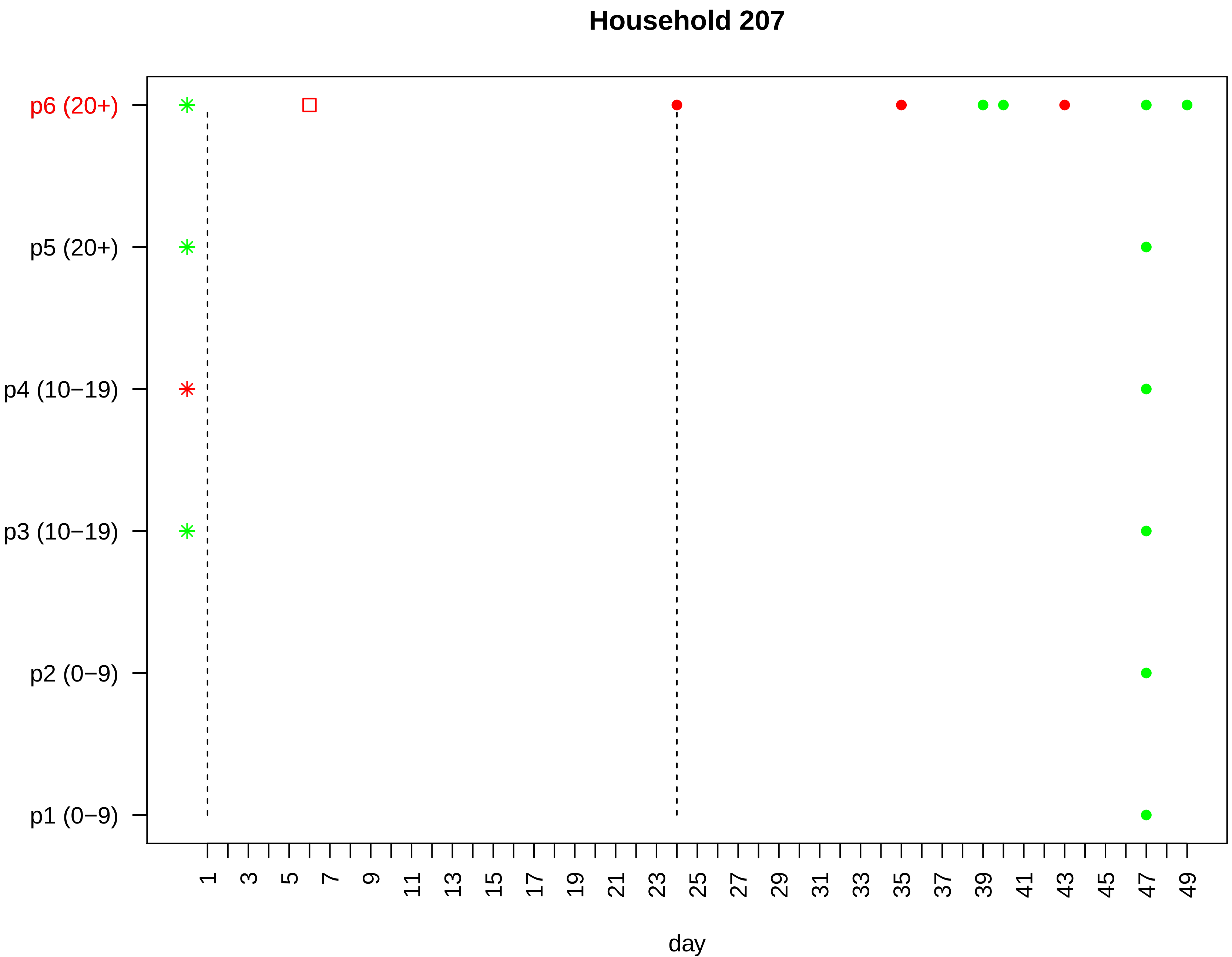

Household 207
p6 (20+)
p5 (20+)
p4 (10−19)
p3 (10−19)
p2 (0−9)
p1 (0−9)
1 3 5 7 9 11 13 15 17 19 21 23 25 27 29 31 33 35 37 39 41 43 45 47 49
day

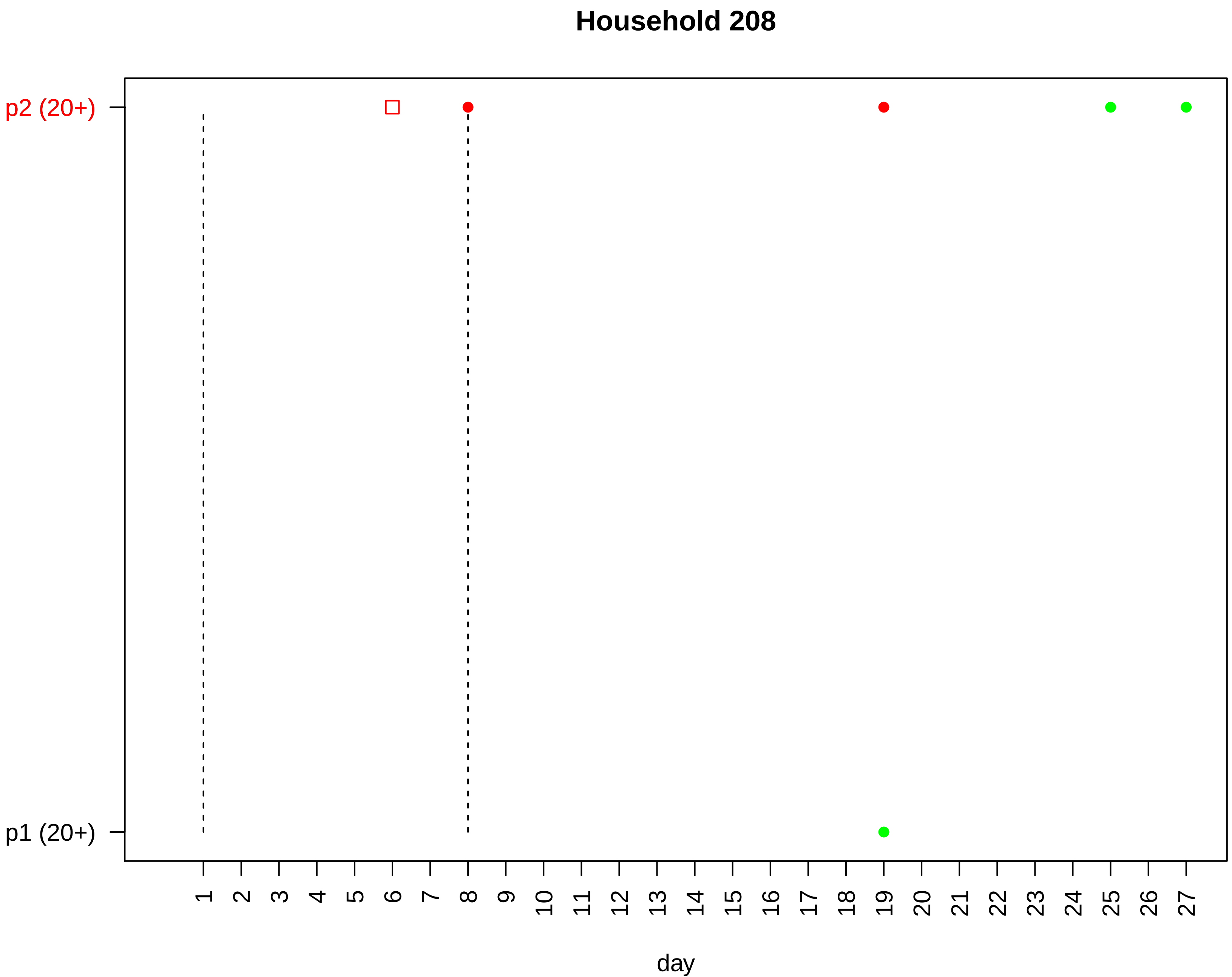
Household 208
p2 (20+)
p1 (20+)
1
2
3
4
5
6
7
8
9
10
11
12
13
14
15
16
17
18
19
20
21
22
23
24
25
26
27
day

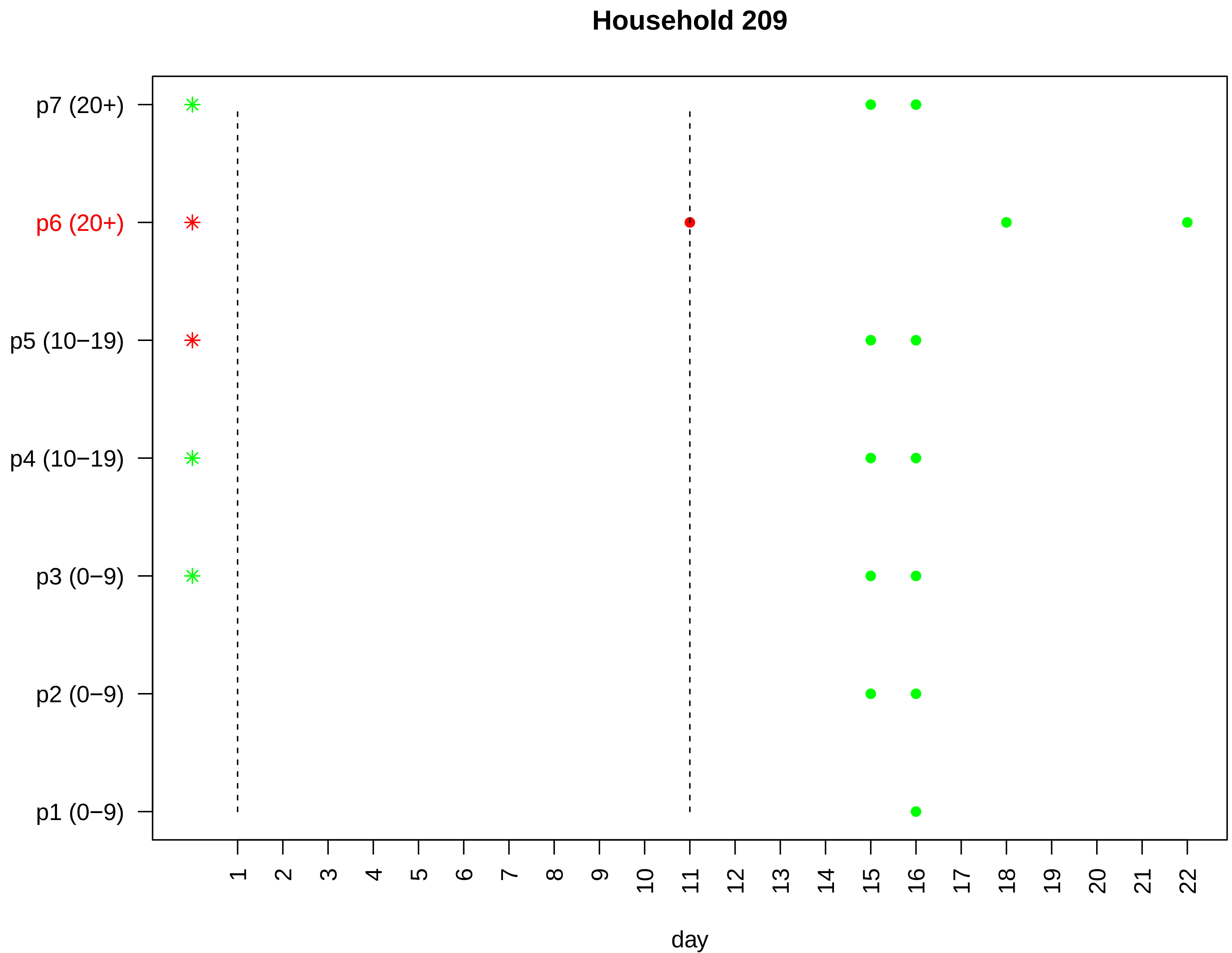

Household 209
p7 (20+)
p6 (20+)
p5 (10−19)
p4 (10−19)
p3 (0−9)
p2 (0−9)
p1 (0−9)
1
2
3
4
5
6
7
8
9
10
11
12
13
14
15
16
17
18
19
20
21
22
day

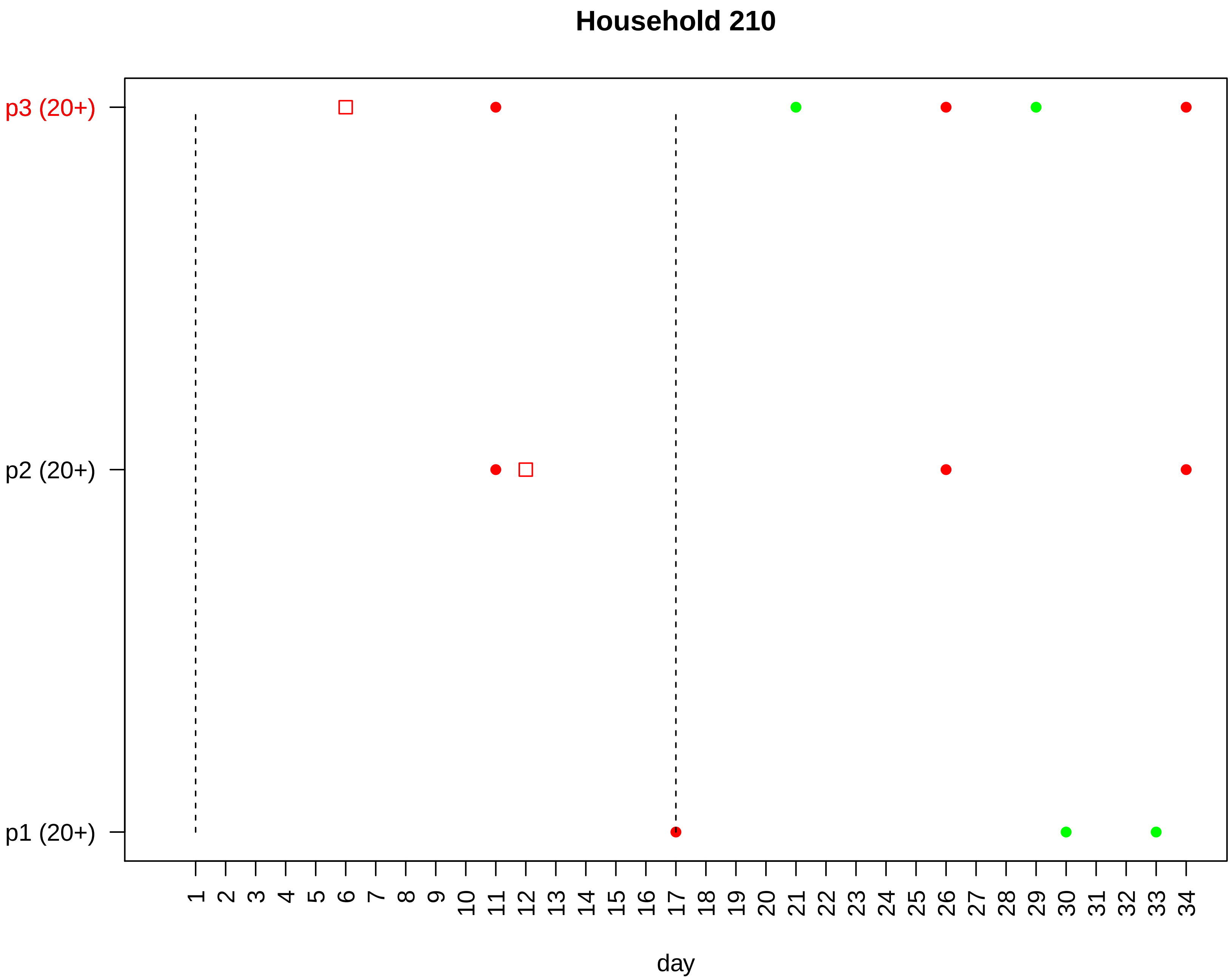
Household 210
p3 (20+)
p2 (20+)
p1 (20+)
1 2 3 4 5 6 7 8 9 10 11 12 13 14 15 16 17 18 19 20 21 22 23 24 25 26 27 28 29 30 31 32 33 34
day

Household 211

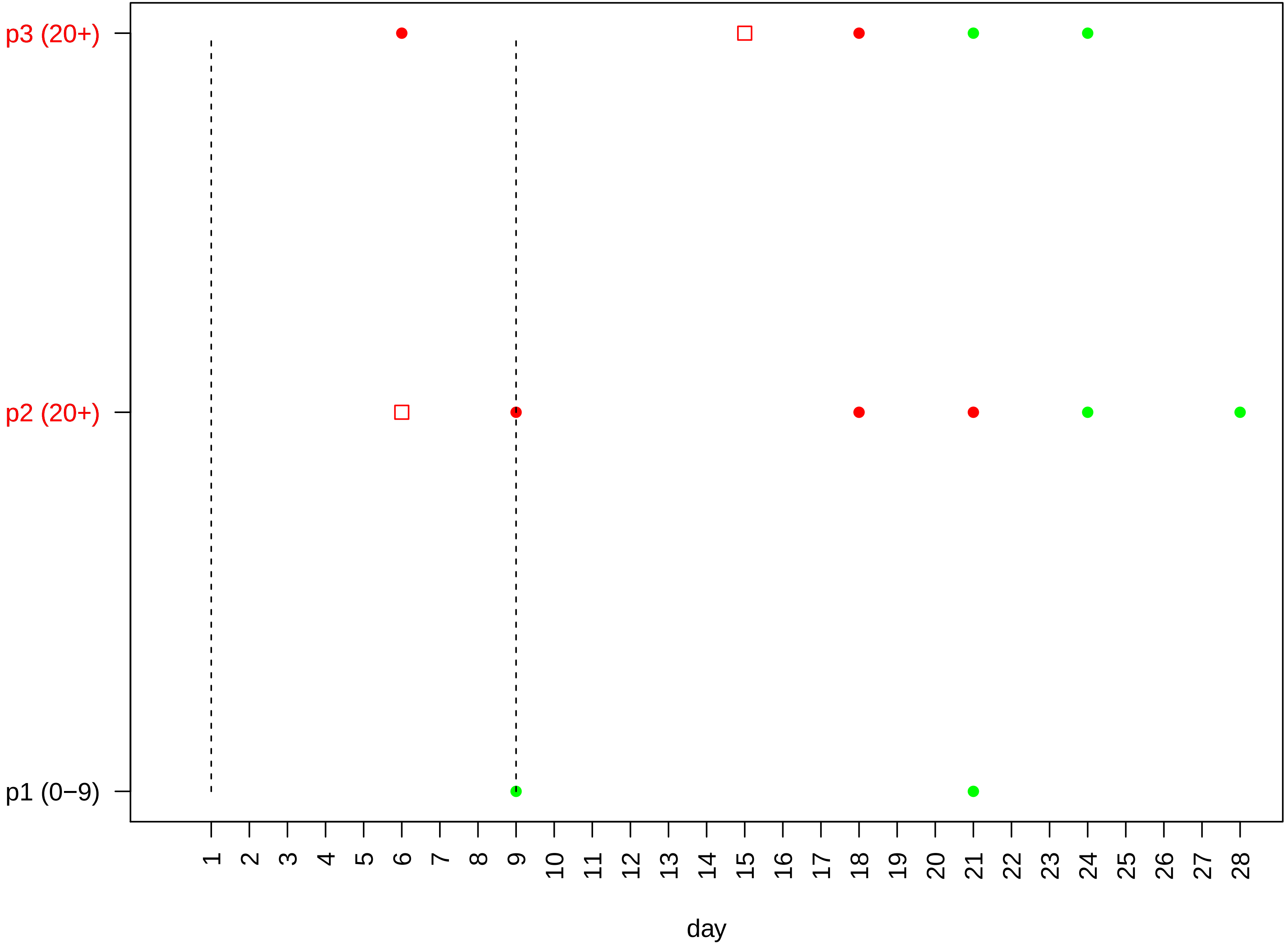

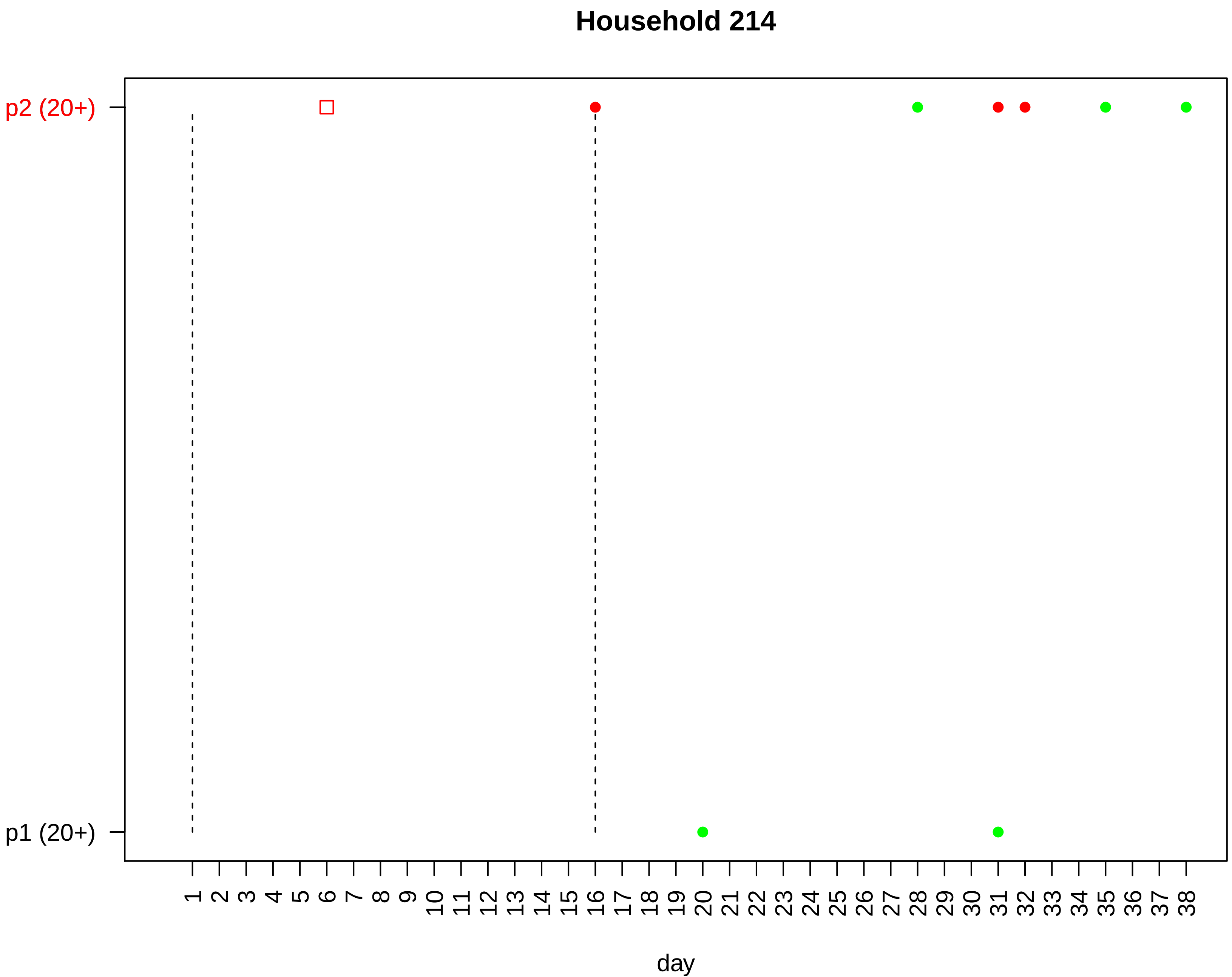

Household 214
p2 (20+)
p1 (20+)
1 2 3 4 5 6 7 8 9 10 11 12 13 14 15 16 17 18 19 20 21 22 23 24 25 26 27 28 29 30 31 32 33 34 35 36 37 38
day

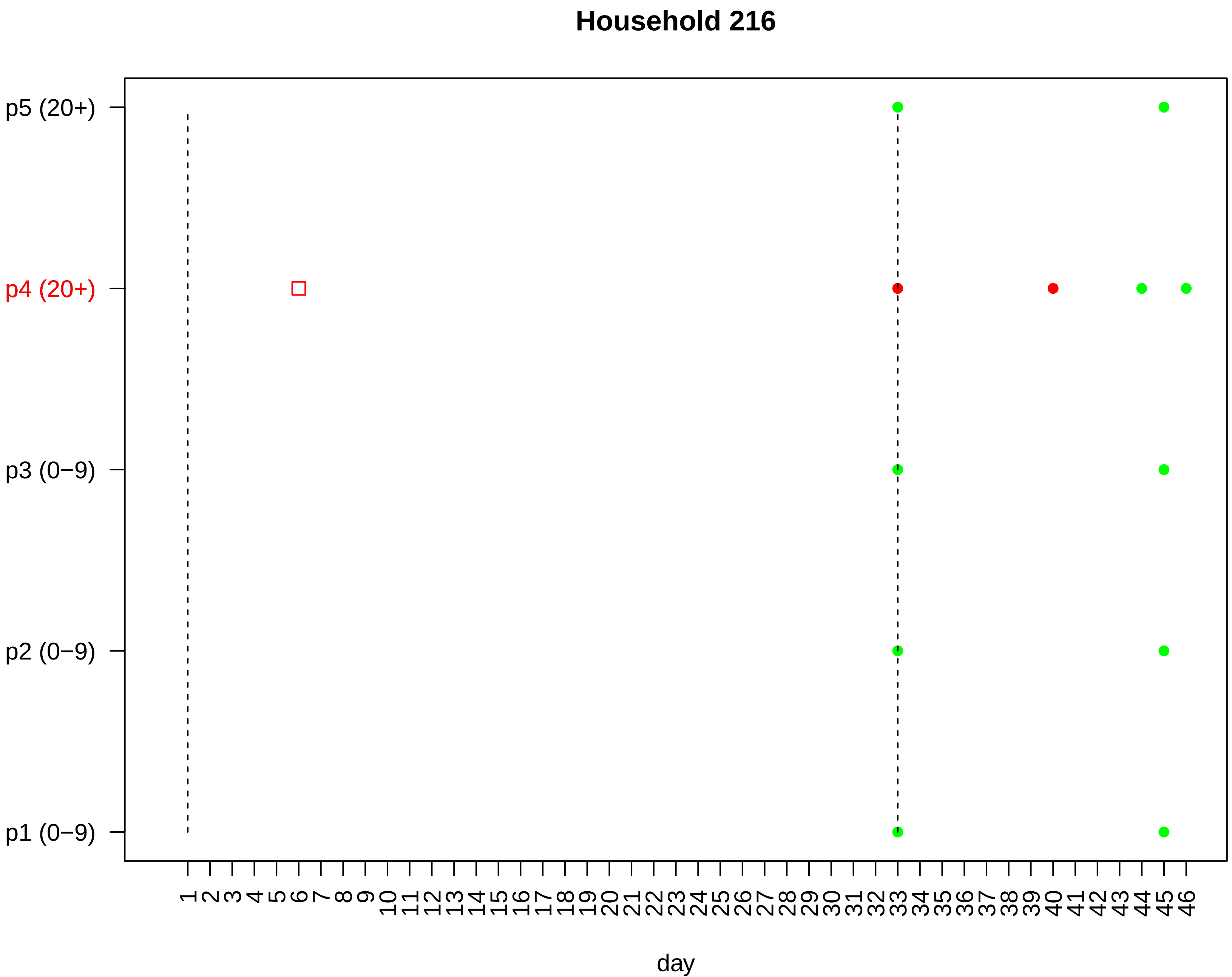

Household 216
p5 (20+)
p4 (20+)
p3 (0−9)
p2 (0−9)
p1 (0−9)
1 2 3 4 5 6 7 8 9 10 11 12 13 14 15 16 17 18 19 20 21 22 23 24 25 26 27 28 29 30 31 32 33 34 35 36 37 38 39 40 41 42 43 44 45 46
day

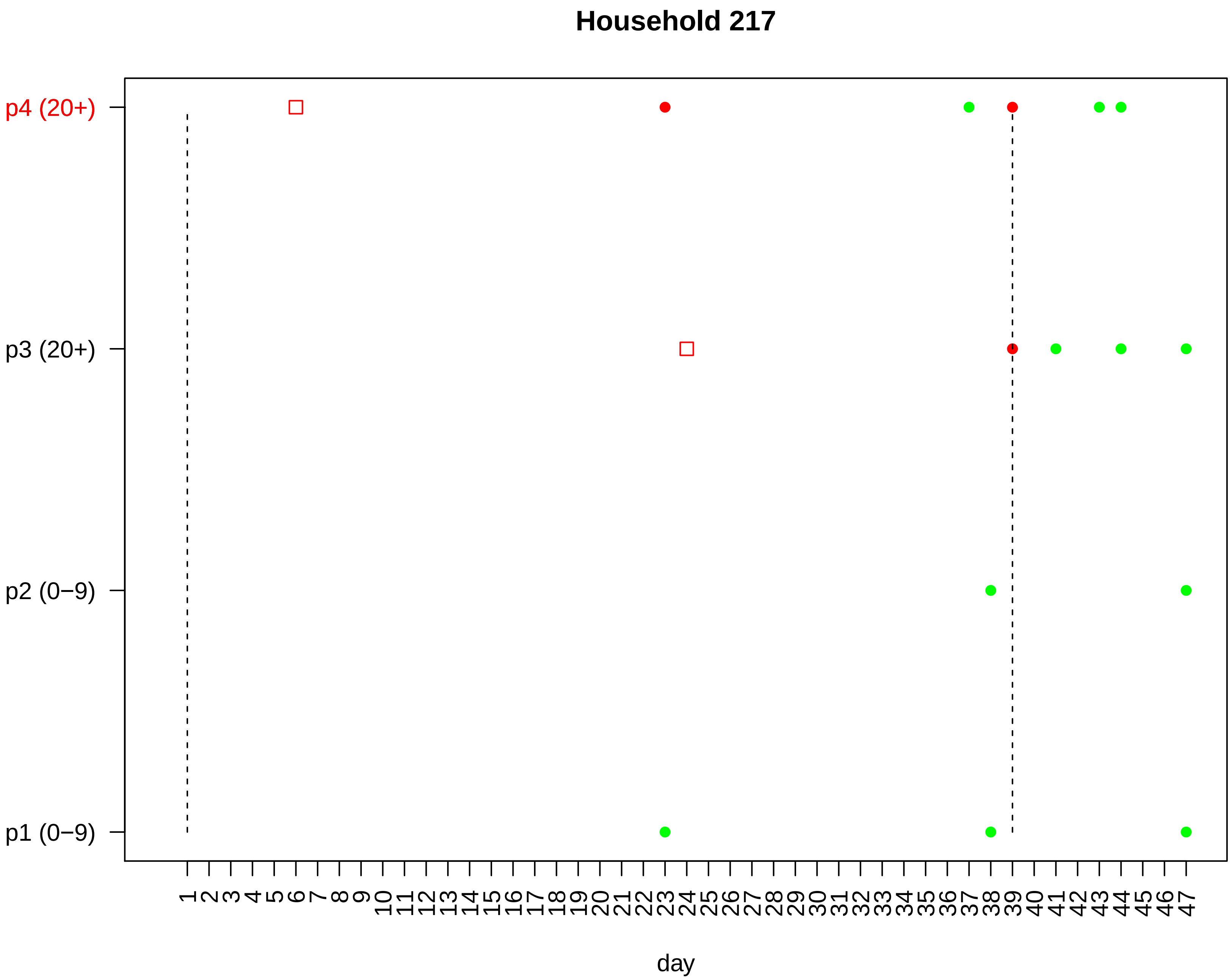
Household 217
p4 (20+)
p3 (20+)
p2 (0−9)
p1 (0−9)
1 2 3 4 5 6 7 8 9 10 11 12 13 14 15 16 17 18 19 20 21 22 23 24 25 26 27 28 29 30 31 32 33 34 35 36 37 38 39 40 41 42 43 44 45 46 47
day

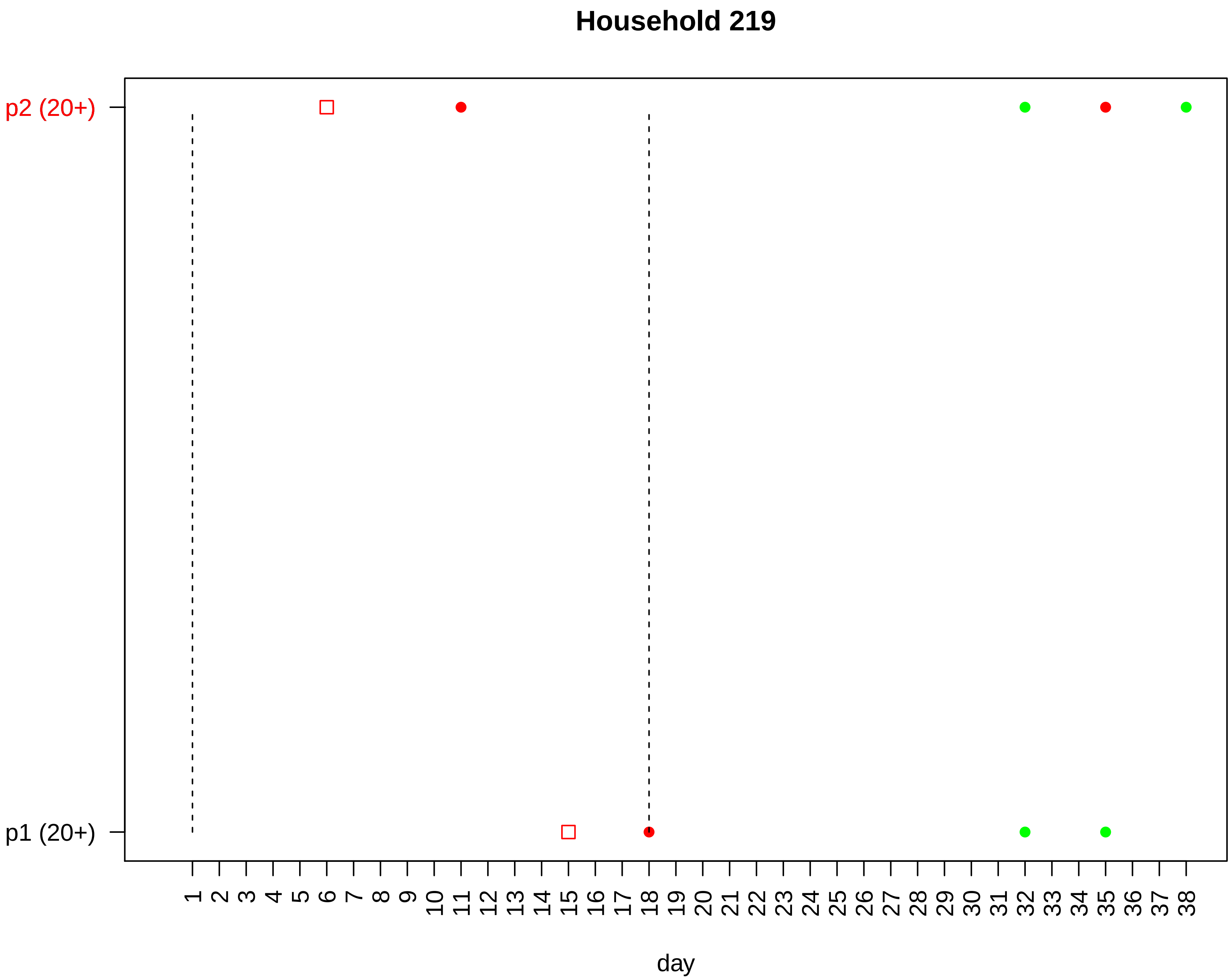

Household 219
p2 (20+)
p1 (20+)
1 2 3 4 5 6 7 8 9 10 11 12 13 14 15 16 17 18 19 20 21 22 23 24 25 26 27 28 29 30 31 32 33 34 35 36 37 38
day

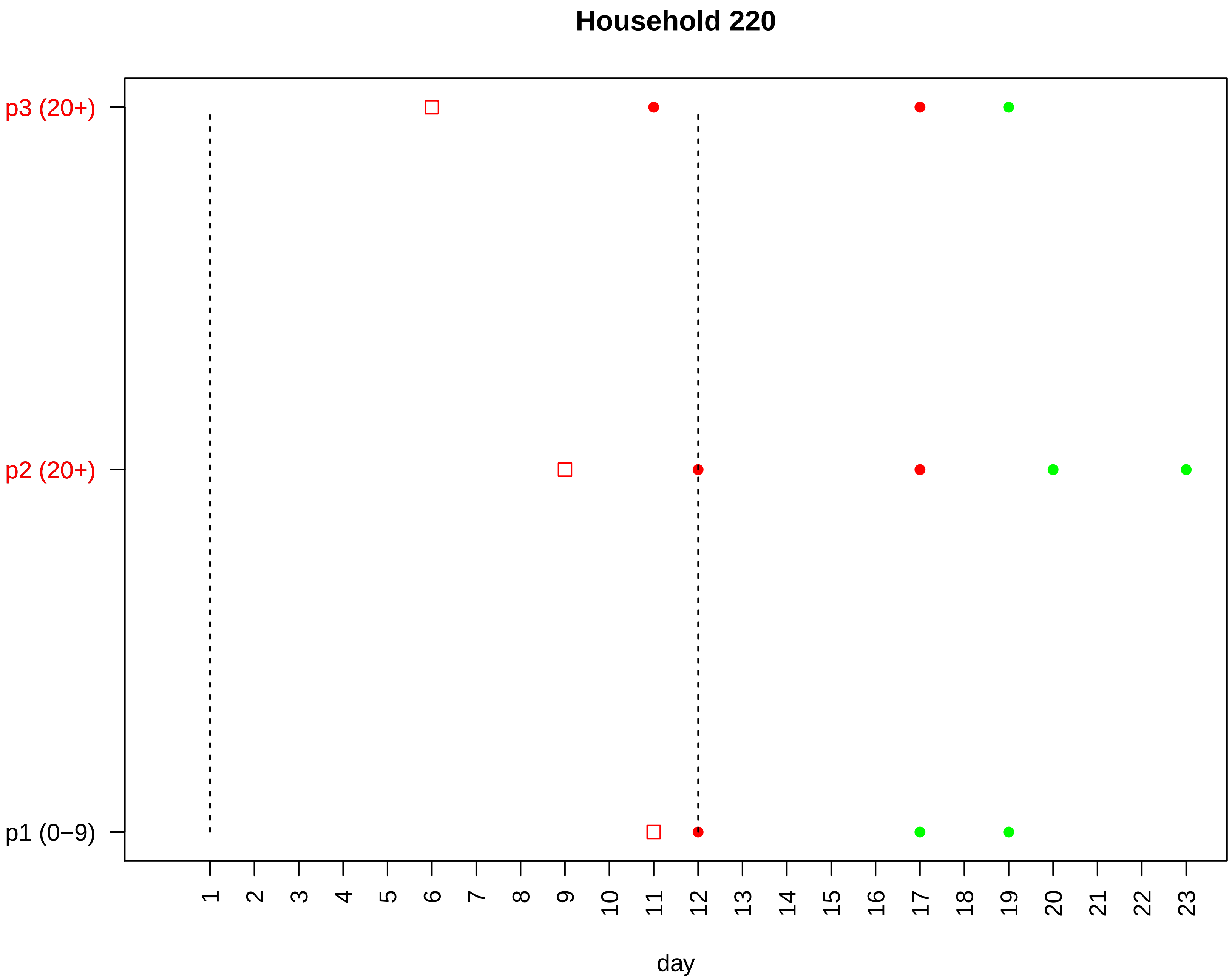
Household 220
p3 (20+)
p2 (20+)
p1 (0−9)
1
2
3
4
5
6
7
8
9
10
11
12
13
14
15
16
17
18
19
20
21
22
23
day

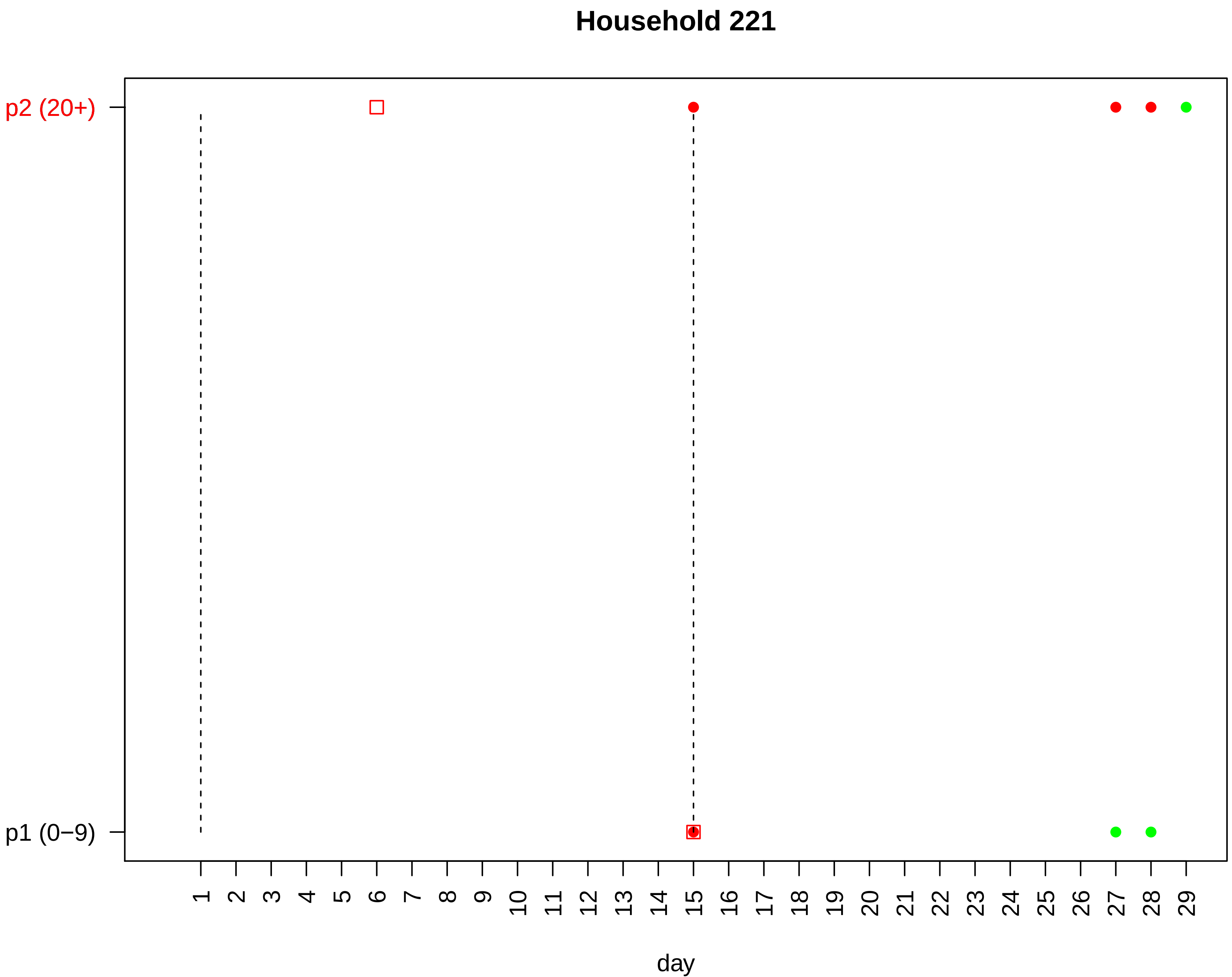

Household 221
p2 (20+)
p1 (0−9)
1
2
3
4
5
6
7
8
9
10
11
12
13
14
15
16
17
18
19
20
21
22
23
24
25
26
27
28
29
day

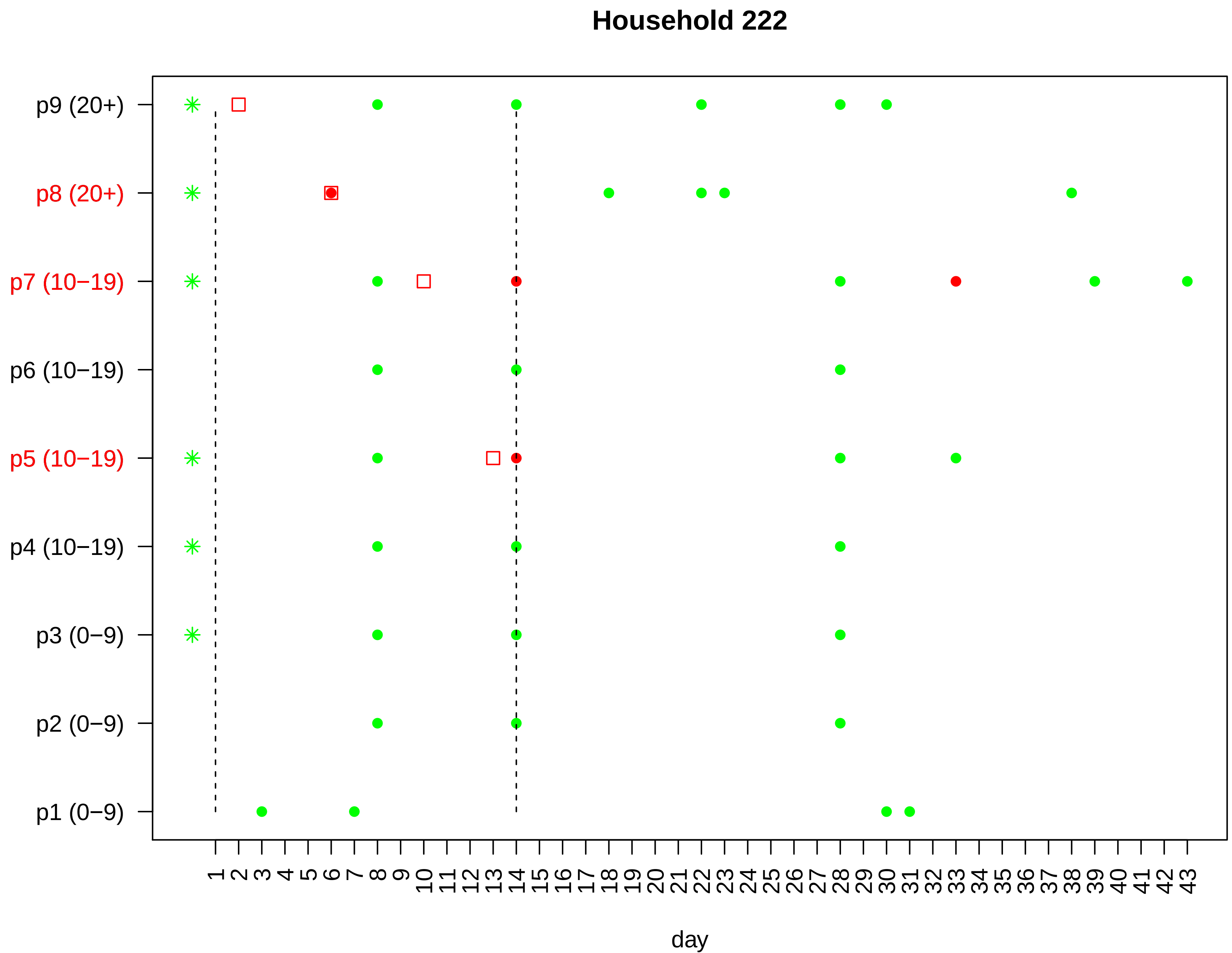
Household 222
p9 (20+)
p8 (20+)
p7 (10−19)
p6 (10−19)
p5 (10−19)
p4 (10−19)
p3 (0−9)
p2 (0−9)
p1 (0−9)
1 2 3 4 5 6 7 8 9 10 11 12 13 14 15 16 17 18 19 20 21 22 23 24 25 26 27 28 29 30 31 32 33 34 35 36 37 38 39 40 41 42 43
day

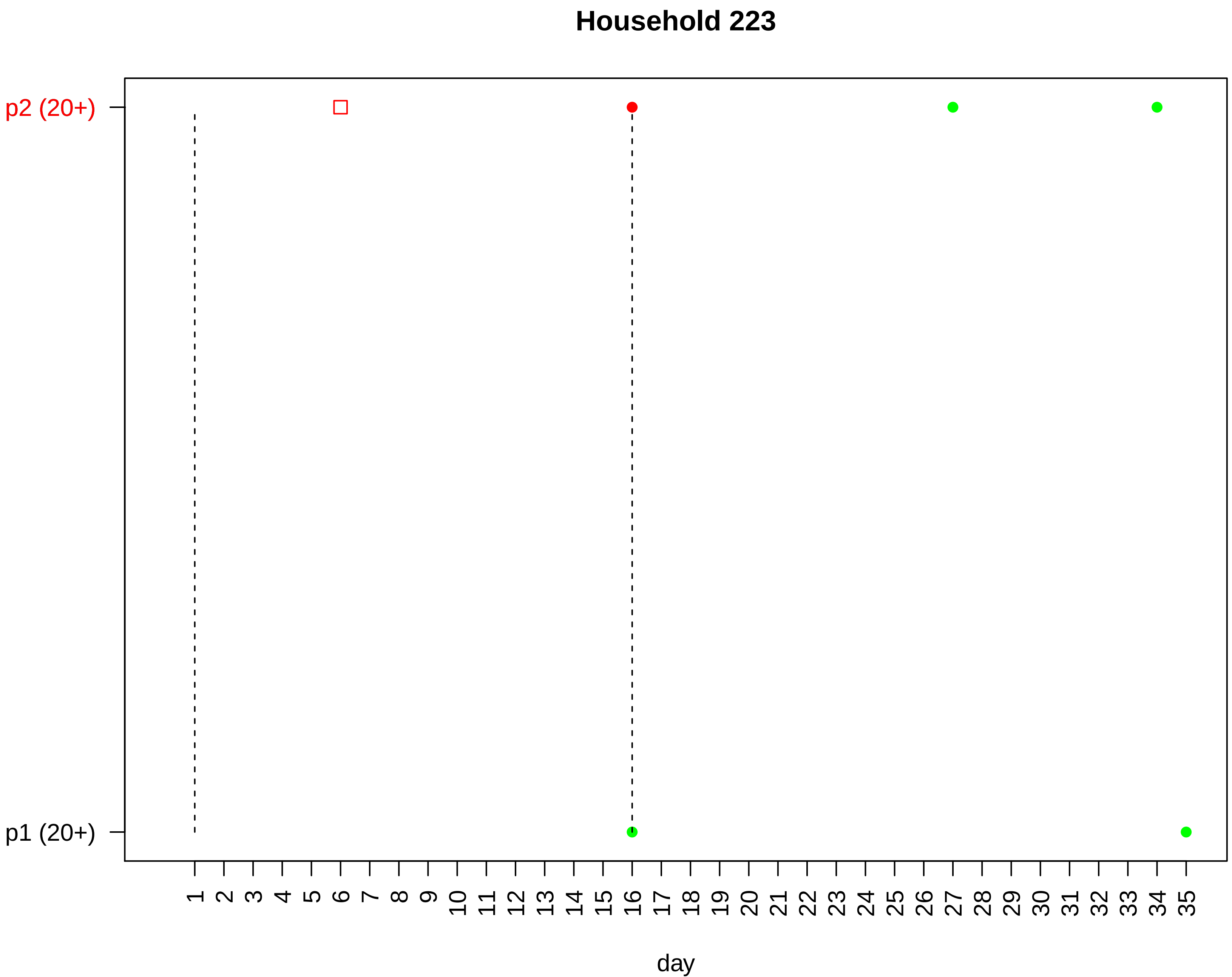

Household 223
p2 (20+)
p1 (20+)
1
2
3
4
5
6
7
8
9
10
11
12
13
14
15
16
17
18
19
20
21
22
23
24
25
26
27
28
29
30
31
32
33
34
35
day

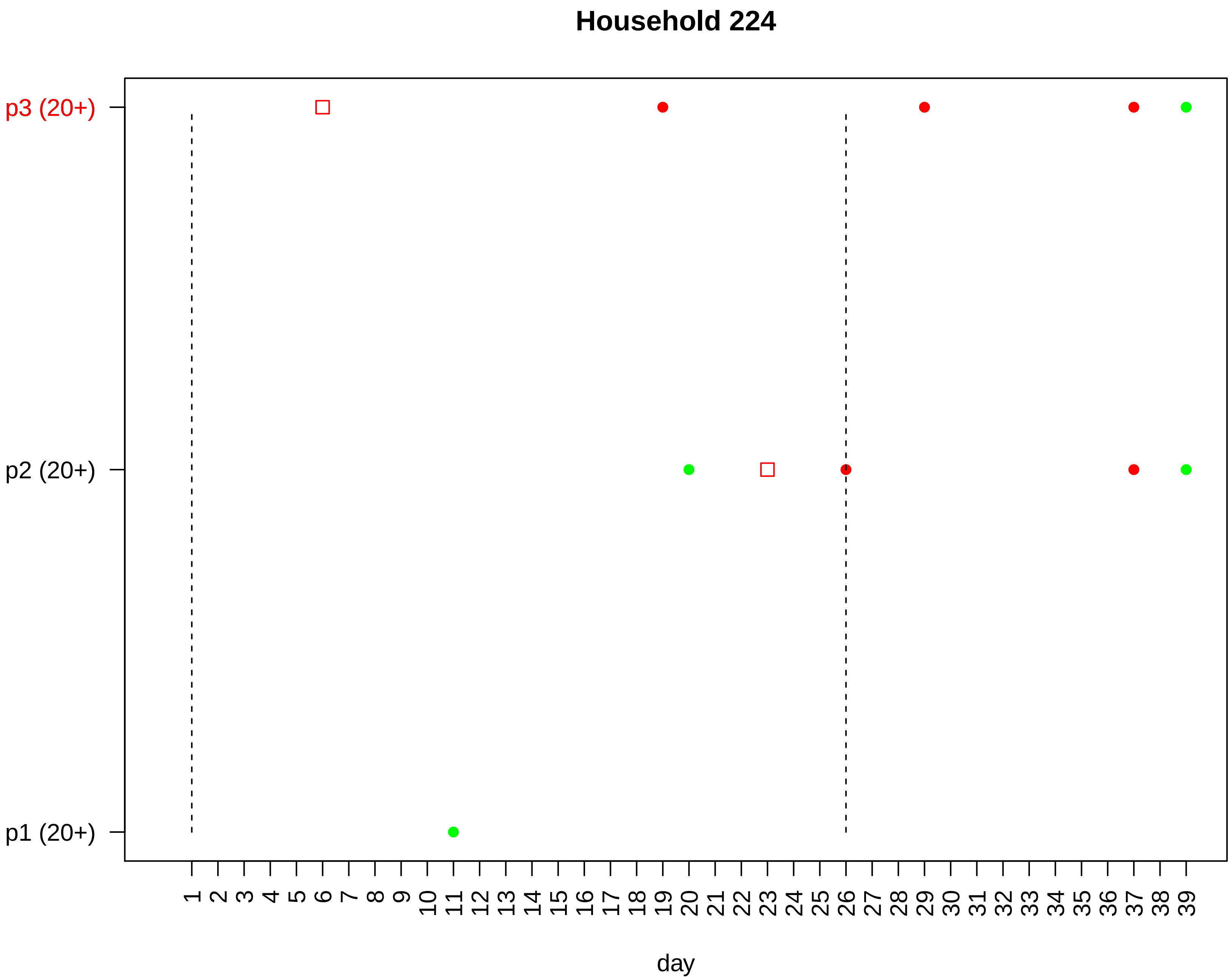
Household 224
p3 (20+)
p2 (20+)
p1 (20+)
1
2
3
4
5
6
7
8
9
10
11
12
13
14
15
16
17
18
19
20
21
22
23
24
25
26
27
28
29
30
31
32
33
34
35
36
37
38
39
day

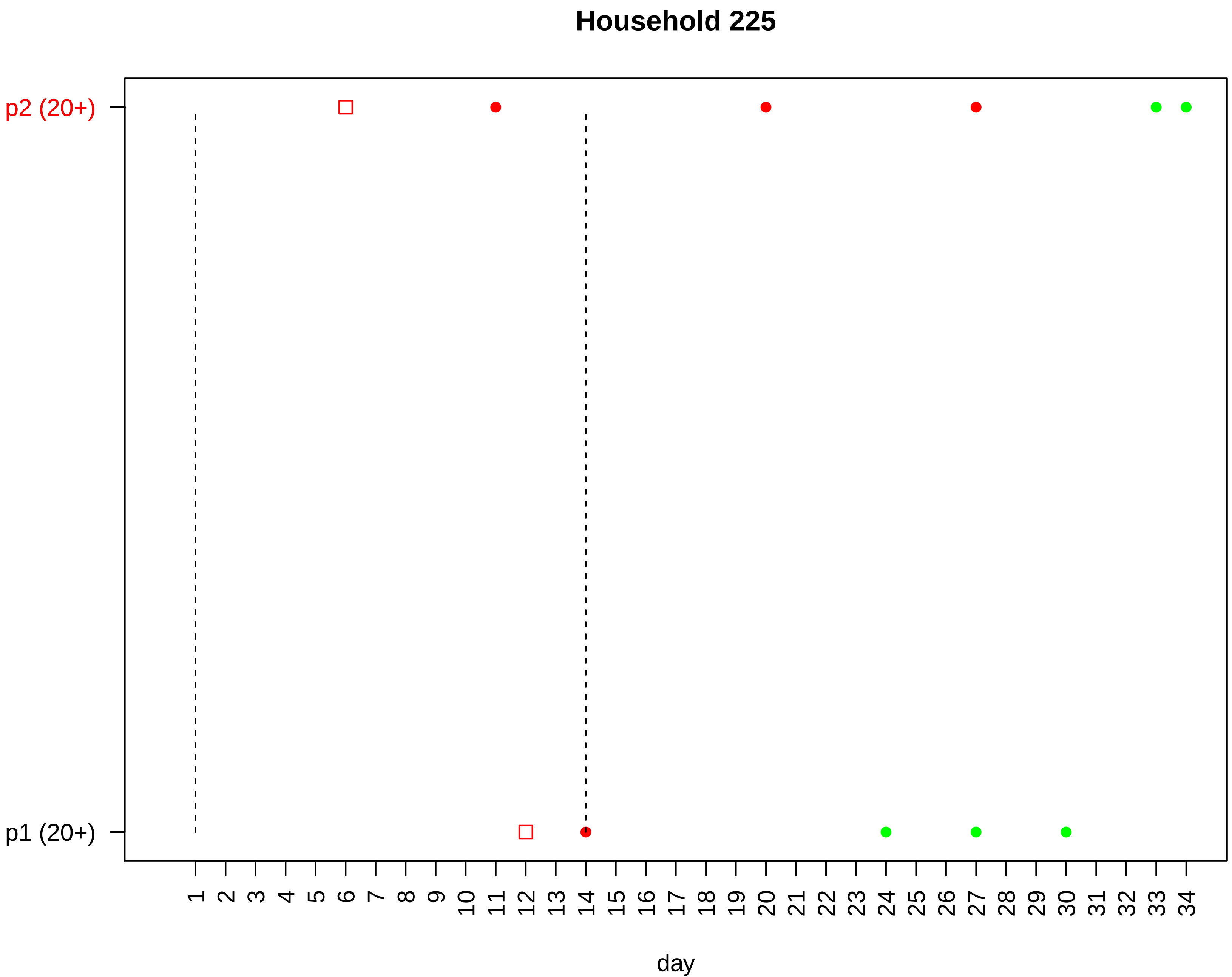

Household 225
p2 (20+)
p1 (20+)
1
2
3
4
5
6
7
8
9
10
11
12
13
14
15
16
17
18
19
20
21
22
23
24
25
26
27
28
29
30
31
32
33
34
day

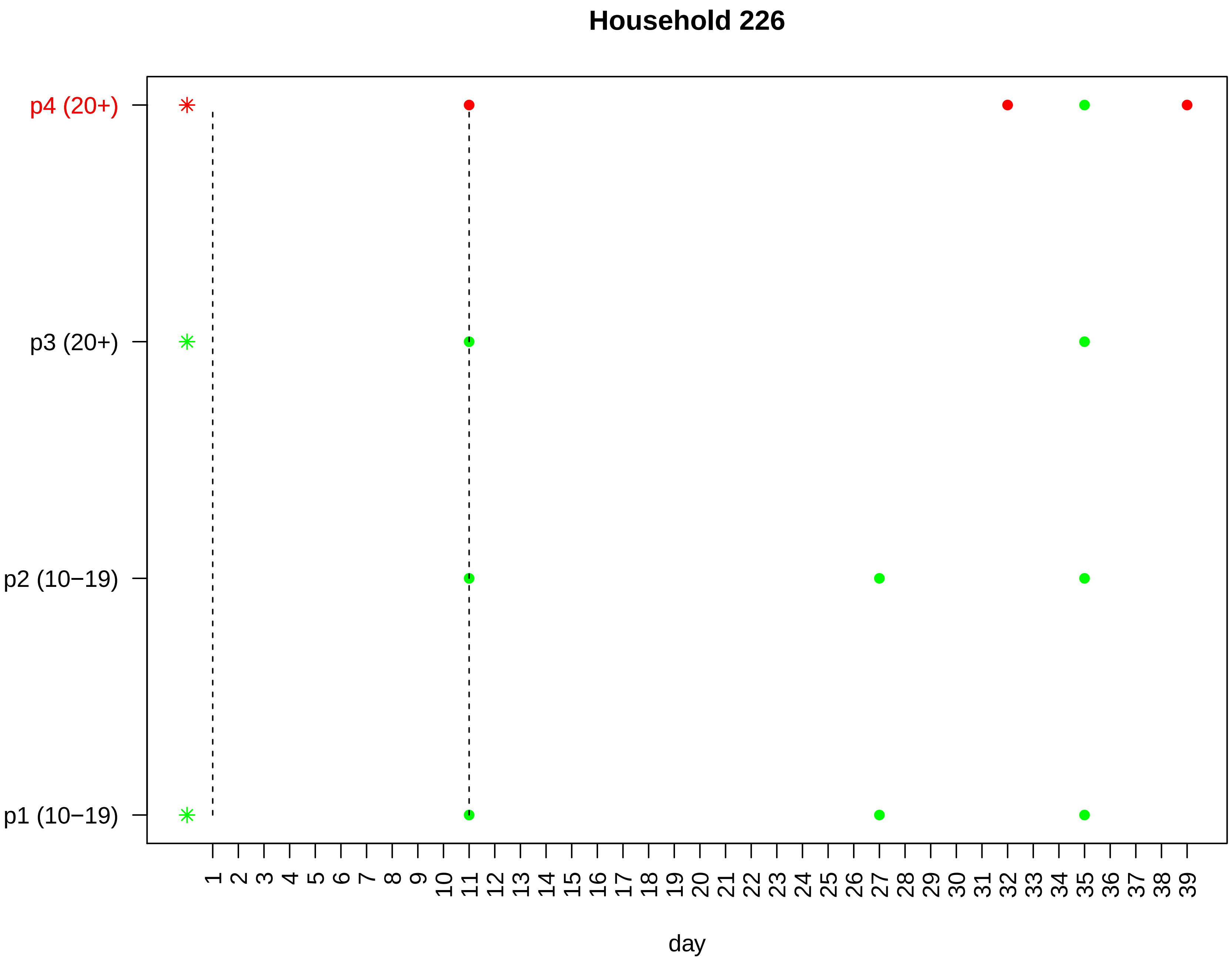
Household 226
p4 (20+)
p3 (20+)
p2 (10−19)
p1 (10−19)
1 2 3 4 5 6 7 8 9 10 11 12 13 14 15 16 17 18 19 20 21 22 23 24 25 26 27 28 29 30 31 32 33 34 35 36 37 38 39
day

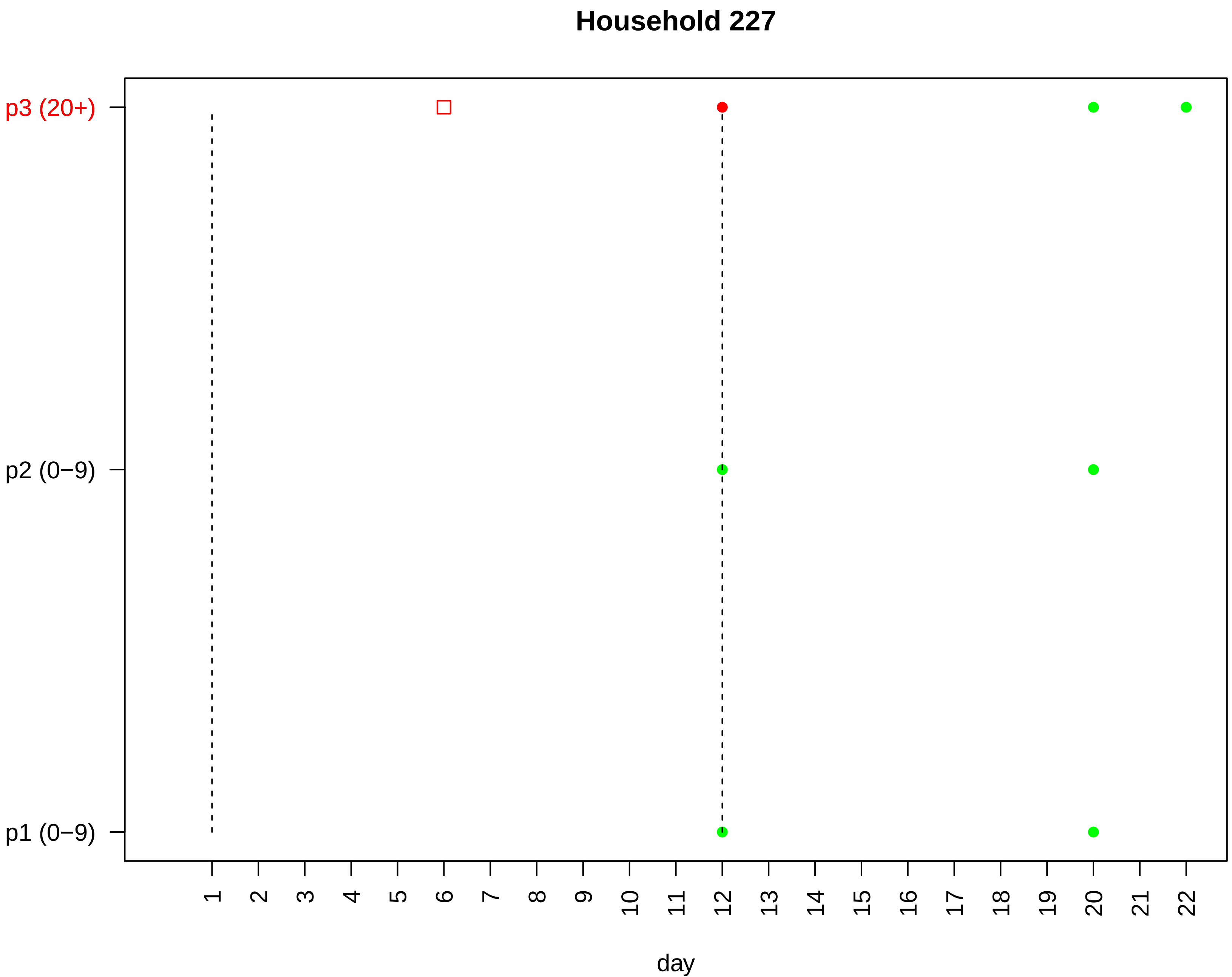
Household 227
p3 (20+)
p2 (0−9)
p1 (0−9)
1
2
3
4
5
6
7
8
9
10
11
12
13
14
15
16
17
18
19
20
21
22
day

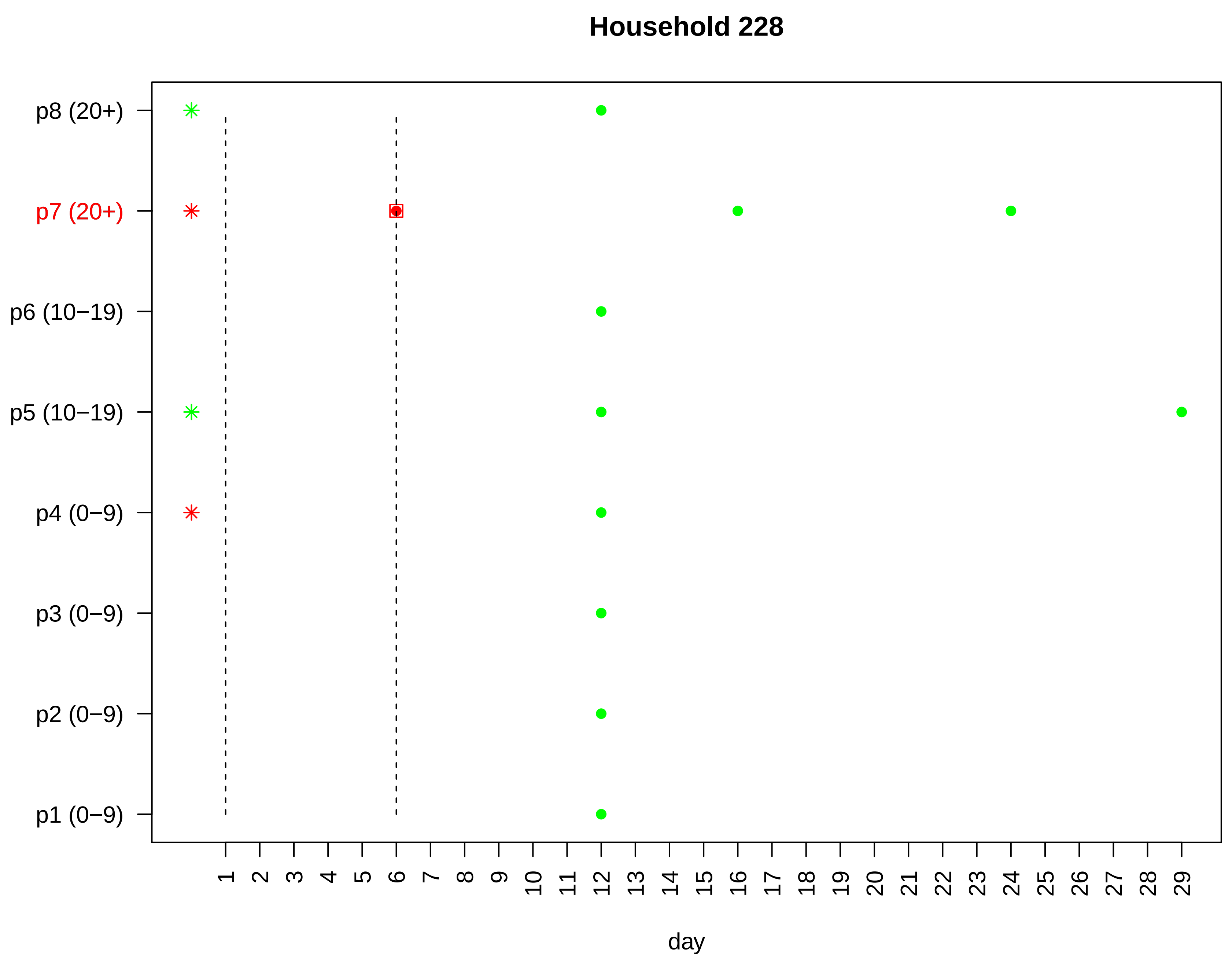

Household 228
p8 (20+)
p7 (20+)
p6 (10−19)
p5 (10−19)
p4 (0−9)
p3 (0−9)
p2 (0−9)
p1 (0−9)
1 2 3 4 5 6 7 8 9 10 11 12 13 14 15 16 17 18 19 20 21 22 23 24 25 26 27 28 29
day

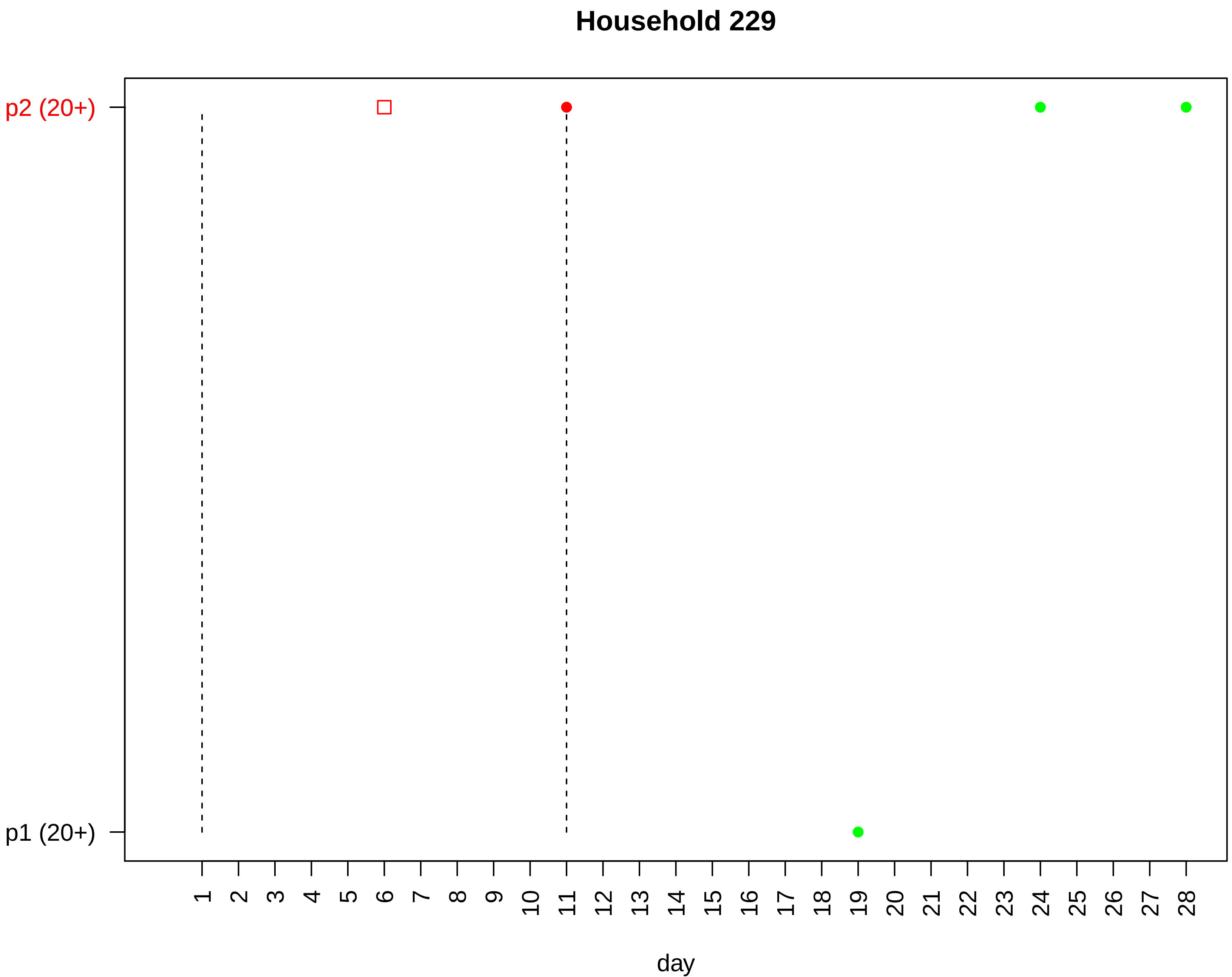

Household 229
p2 (20+)
p1 (20+)
1
2
3
4
5
6
7
8
9
10
11
12
13
14
15
16
17
18
19
20
21
22
23
24
25
26
27
28
day

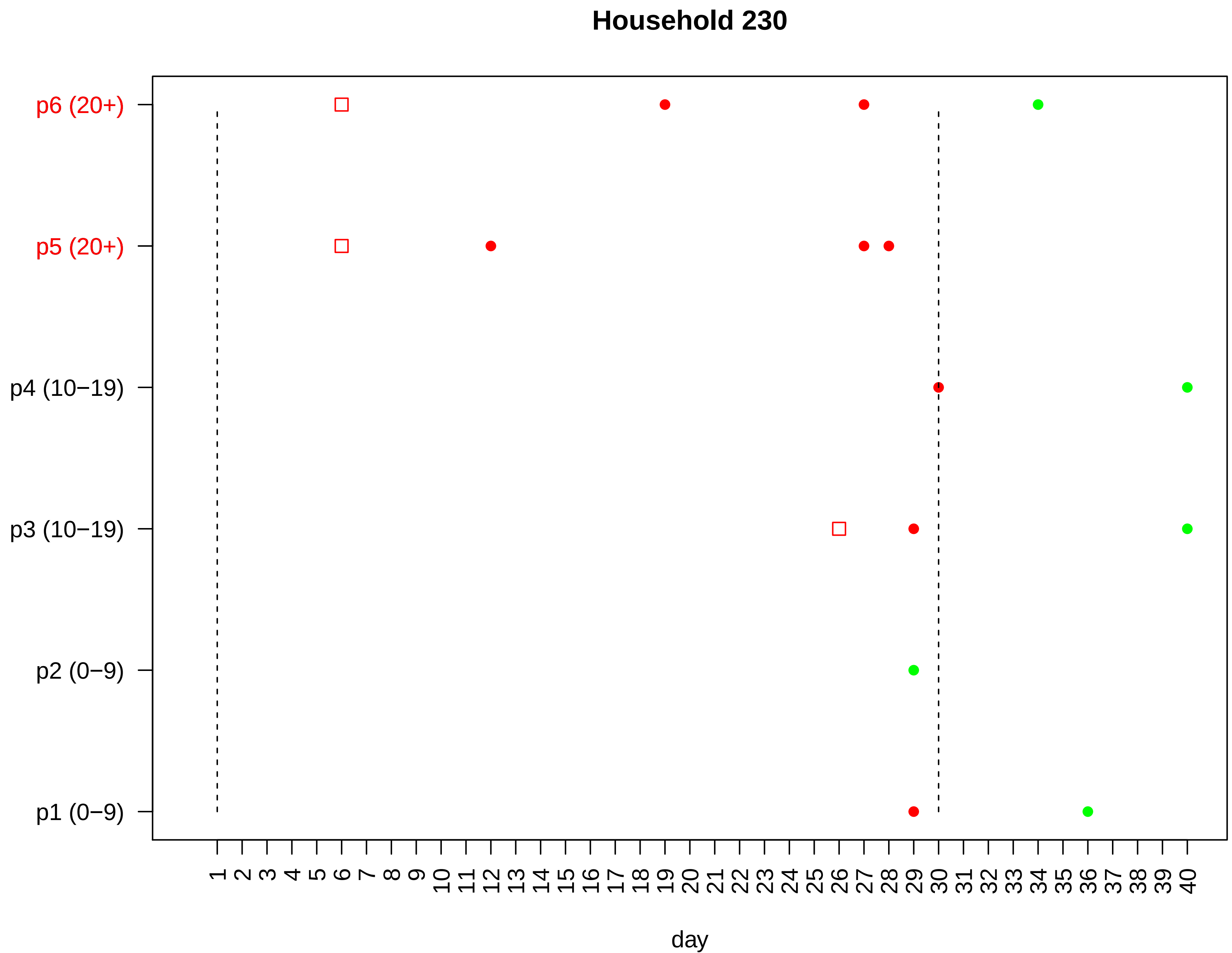

Household 230
p6 (20+)
p5 (20+)
p4 (10−19)
p3 (10−19)
p2 (0−9)
p1 (0−9)
1 2 3 4 5 6 7 8 9 10 11 12 13 14 15 16 17 18 19 20 21 22 23 24 25 26 27 28 29 30 31 32 33 34 35 36 37 38 39 40
day

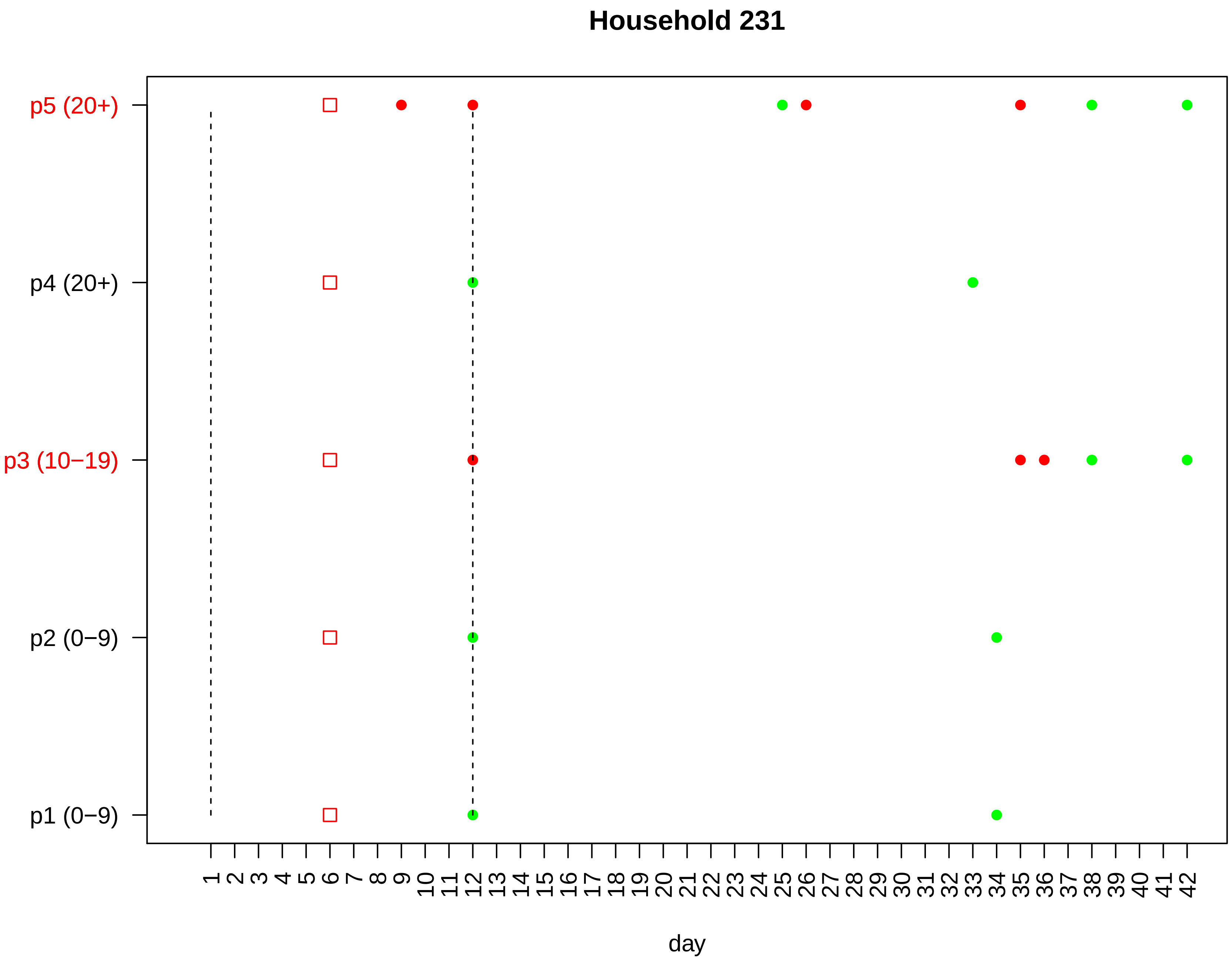
Household 231
p5 (20+)
p4 (20+)
p3 (10−19)
p2 (0−9)
p1 (0−9)
1 2 3 4 5 6 7 8 9 10 11 12 13 14 15 16 17 18 19 20 21 22 23 24 25 26 27 28 29 30 31 32 33 34 35 36 37 38 39 40 41 42
day

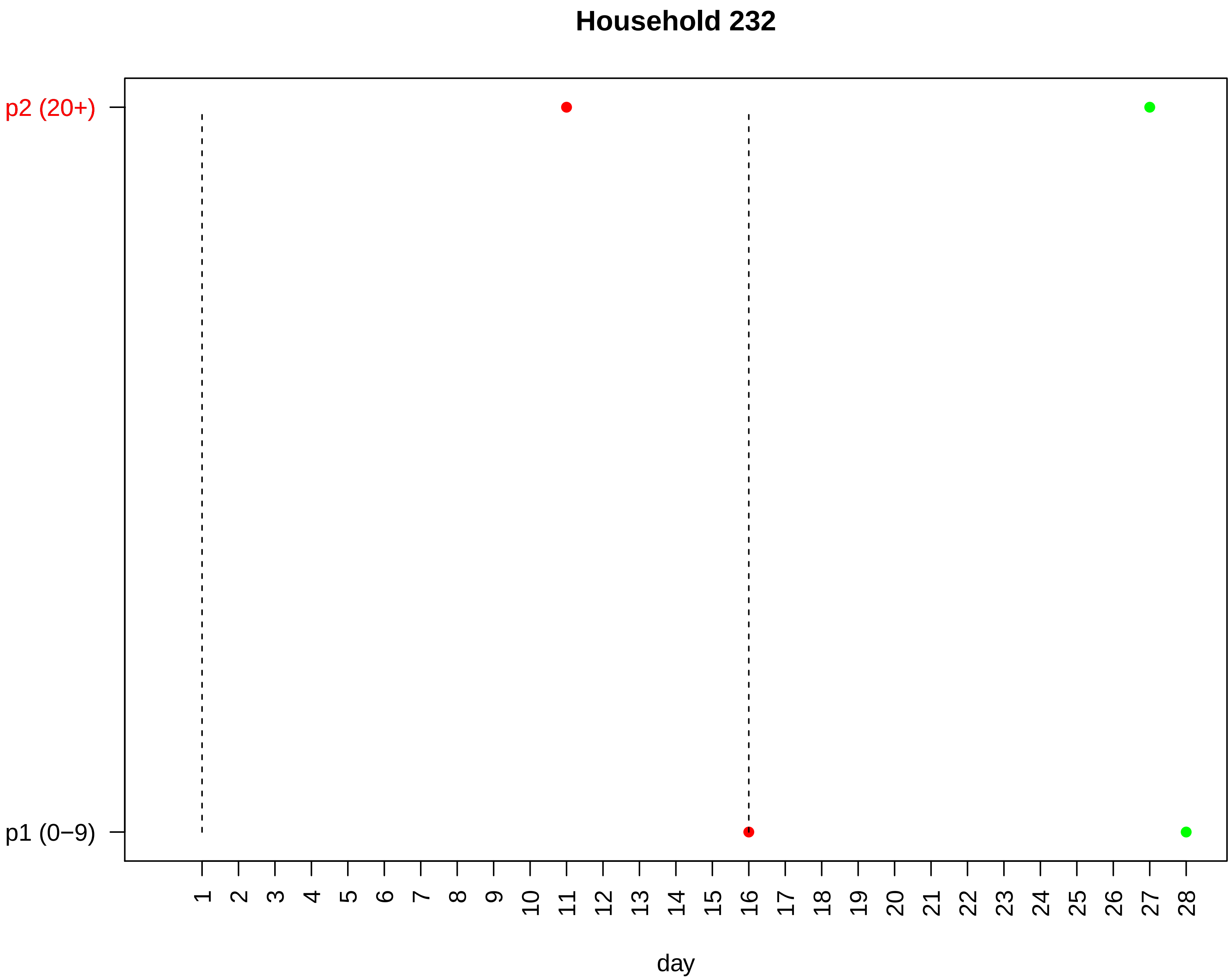
Household 232
p2 (20+)
p1 (0−9)
1
2
3
4
5
6
7
8
9
10
11
12
13
14
15
16
17
18
19
20
21
22
23
24
25
26
27
28
day

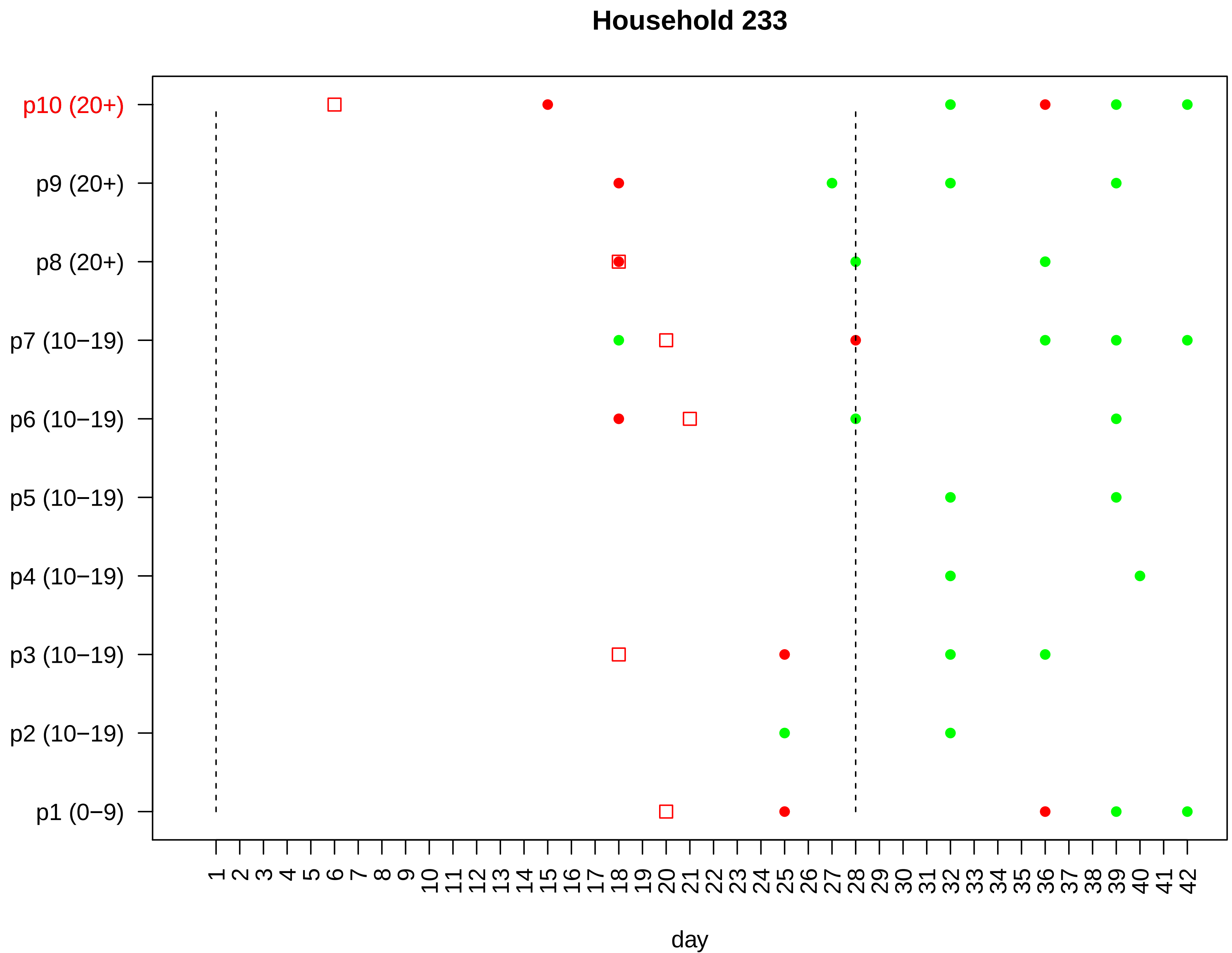

Household 233
p10 (20+)
p9 (20+)
p8 (20+)
p7 (10−19)
p6 (10−19)
p5 (10−19)
p4 (10−19)
p3 (10−19)
p2 (10−19)
p1 (0−9)
1 2 3 4 5 6 7 8 9 10 11 12 13 14 15 16 17 18 19 20 21 22 23 24 25 26 27 28 29 30 31 32 33 34 35 36 37 38 39 40 41 42
day

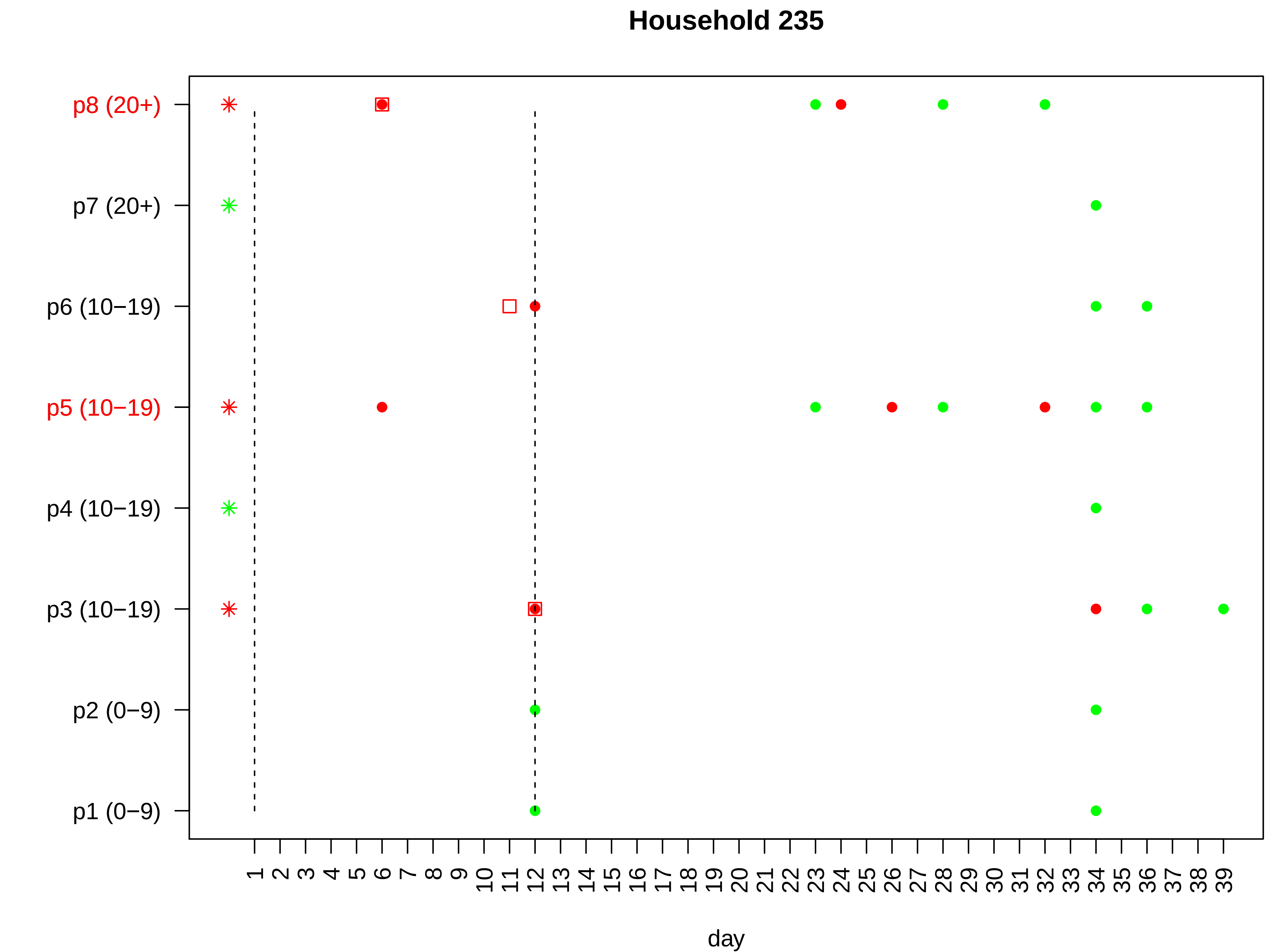

Household 235
p8 (20+)
p7 (20+)
p6 (10−19)
p5 (10−19)
p4 (10−19)
p3 (10−19)
p2 (0−9)
p1 (0−9)
1 2 3 4 5 6 7 8 9 10 11 12 13 14 15 16 17 18 19 20 21 22 23 24 25 26 27 28 29 30 31 32 33 34 35 36 37 38 39
day

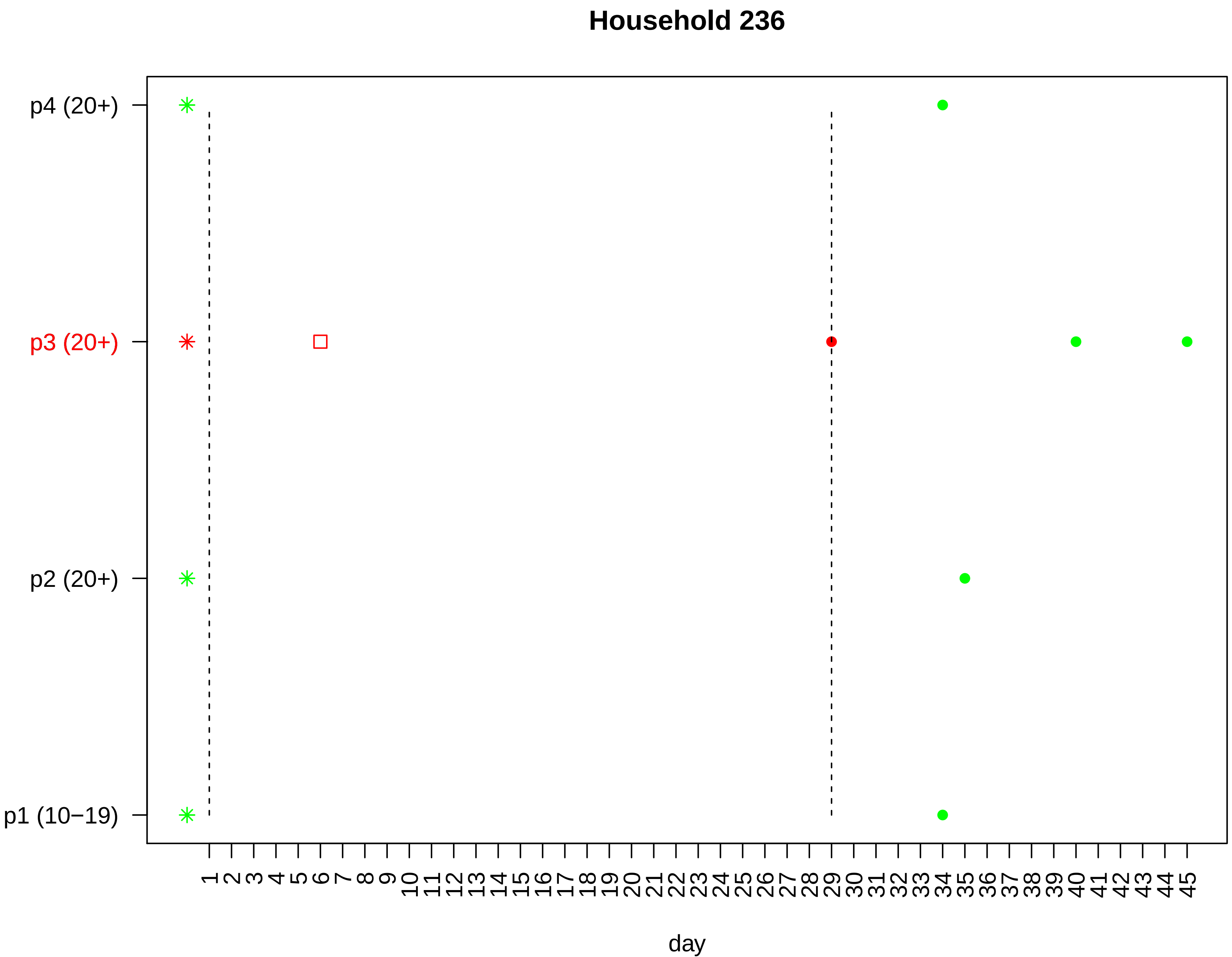

Household 236
p4 (20+)
p3 (20+)
p2 (20+)
p1 (10−19)
1 2 3 4 5 6 7 8 9 10 11 12 13 14 15 16 17 18 19 20 21 22 23 24 25 26 27 28 29 30 31 32 33 34 35 36 37 38 39 40 41 42 43 44 45
day

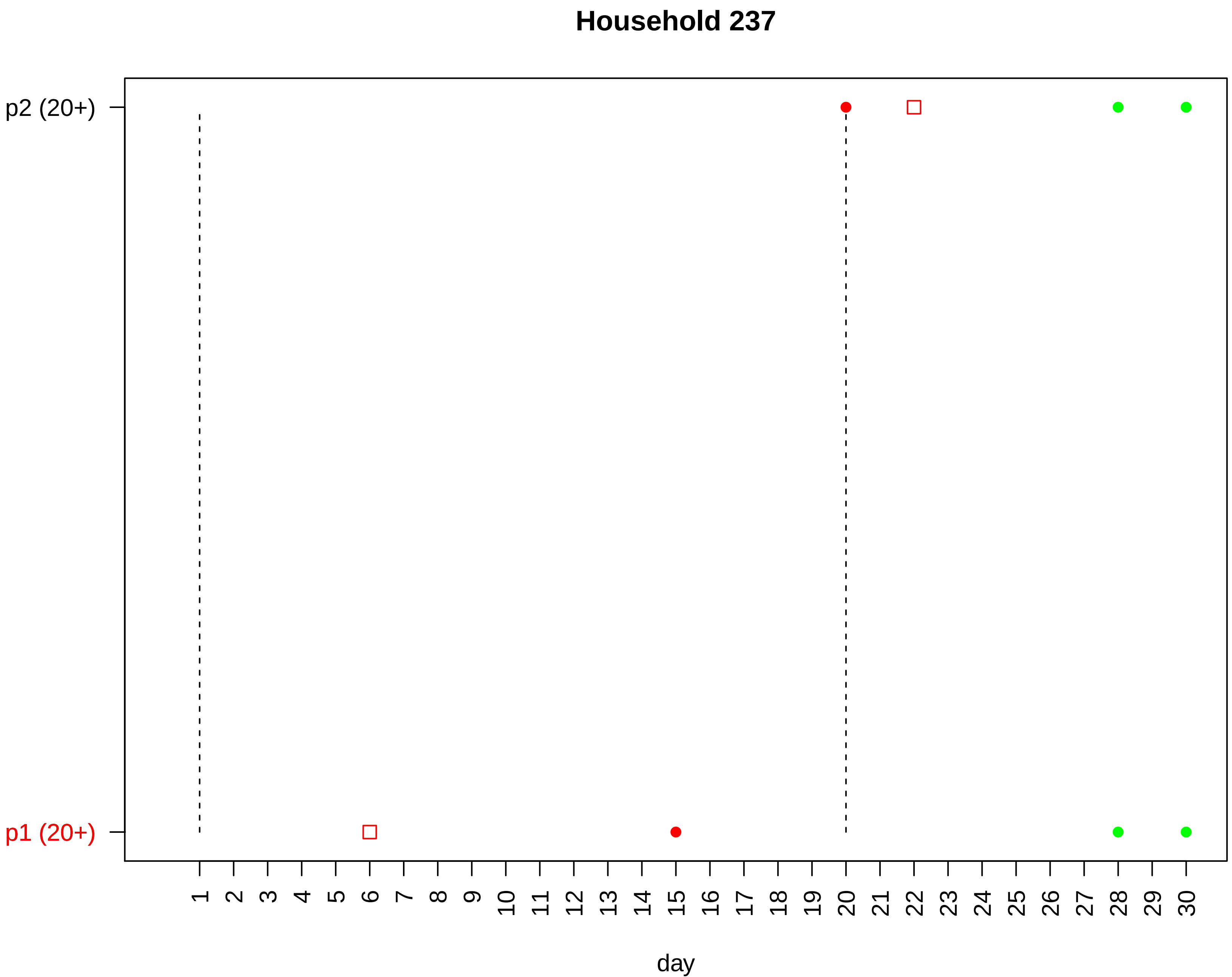
Household 237
p2 (20+)
p1 (20+)
1
2
3
4
5
6
7
8
9
10
11
12
13
14
15
16
17
18
19
20
21
22
23
24
25
26
27
28
29
30
day

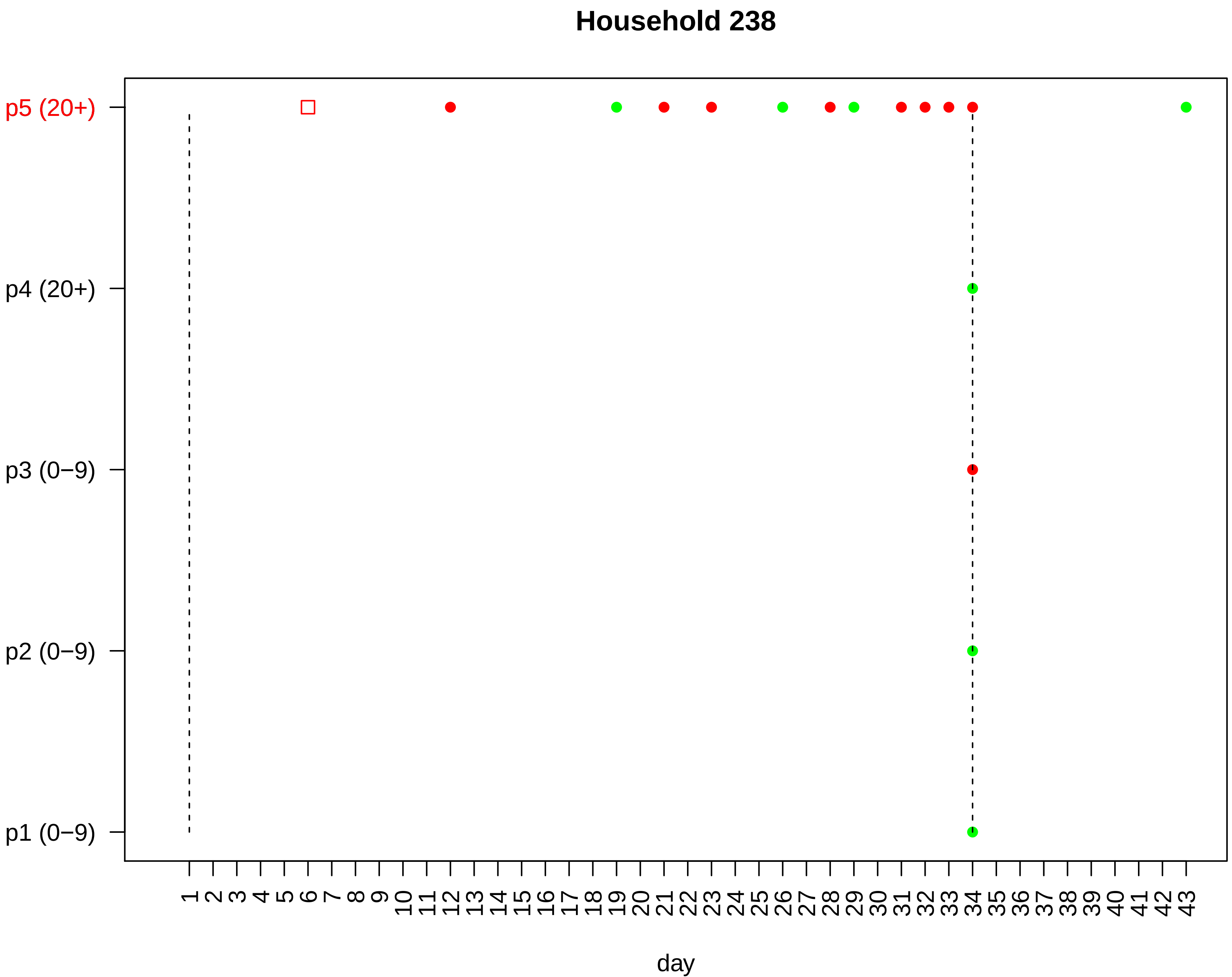

Household 238
p5 (20+)
p4 (20+)
p3 (0−9)
p2 (0−9)
p1 (0−9)
1 2 3 4 5 6 7 8 9 10 11 12 13 14 15 16 17 18 19 20 21 22 23 24 25 26 27 28 29 30 31 32 33 34 35 36 37 38 39 40 41 42 43
day

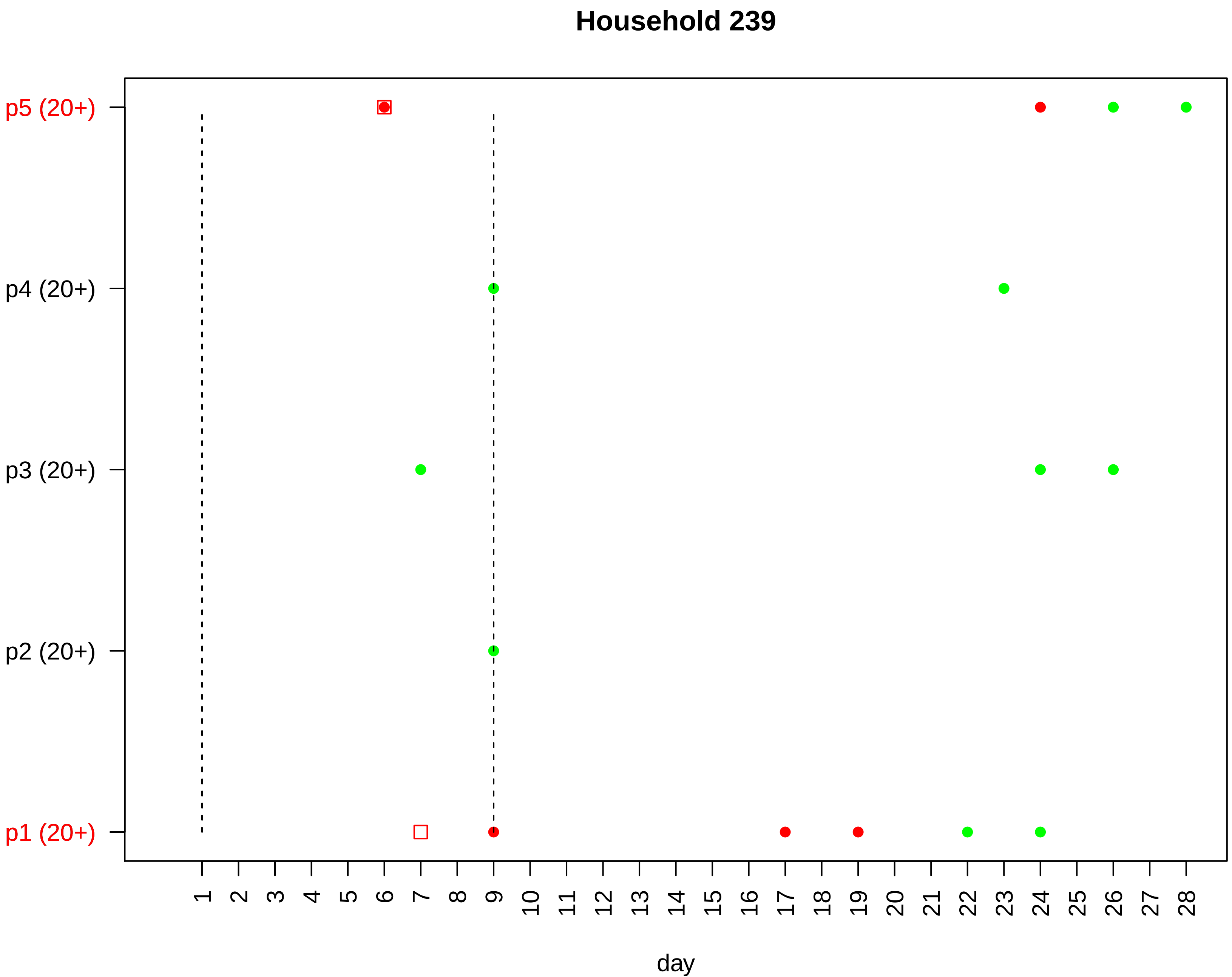
Household 239
p5 (20+)
p4 (20+)
p3 (20+)
p2 (20+)
p1 (20+)
1 2 3 4 5 6 7 8 9 10 11 12 13 14 15 16 17 18 19 20 21 22 23 24 25 26 27 28
day

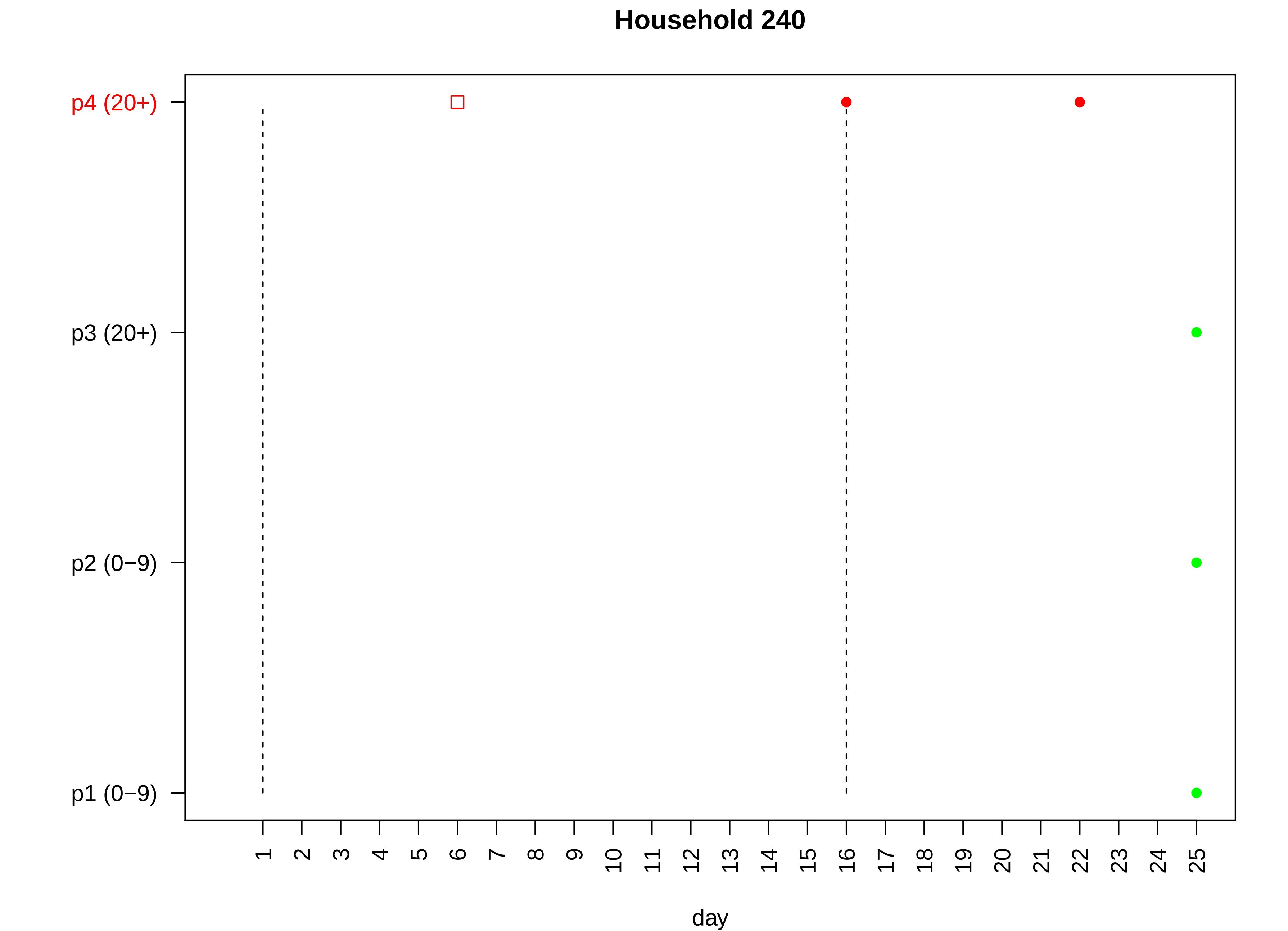
Household 240
p4 (20+)
p3 (20+)
p2 (0−9)
p1 (0−9)
1
2
3
4
5
6
7
8
9
10
11
12
13
14
15
16
17
18
19
20
21
22
23
24
25
day

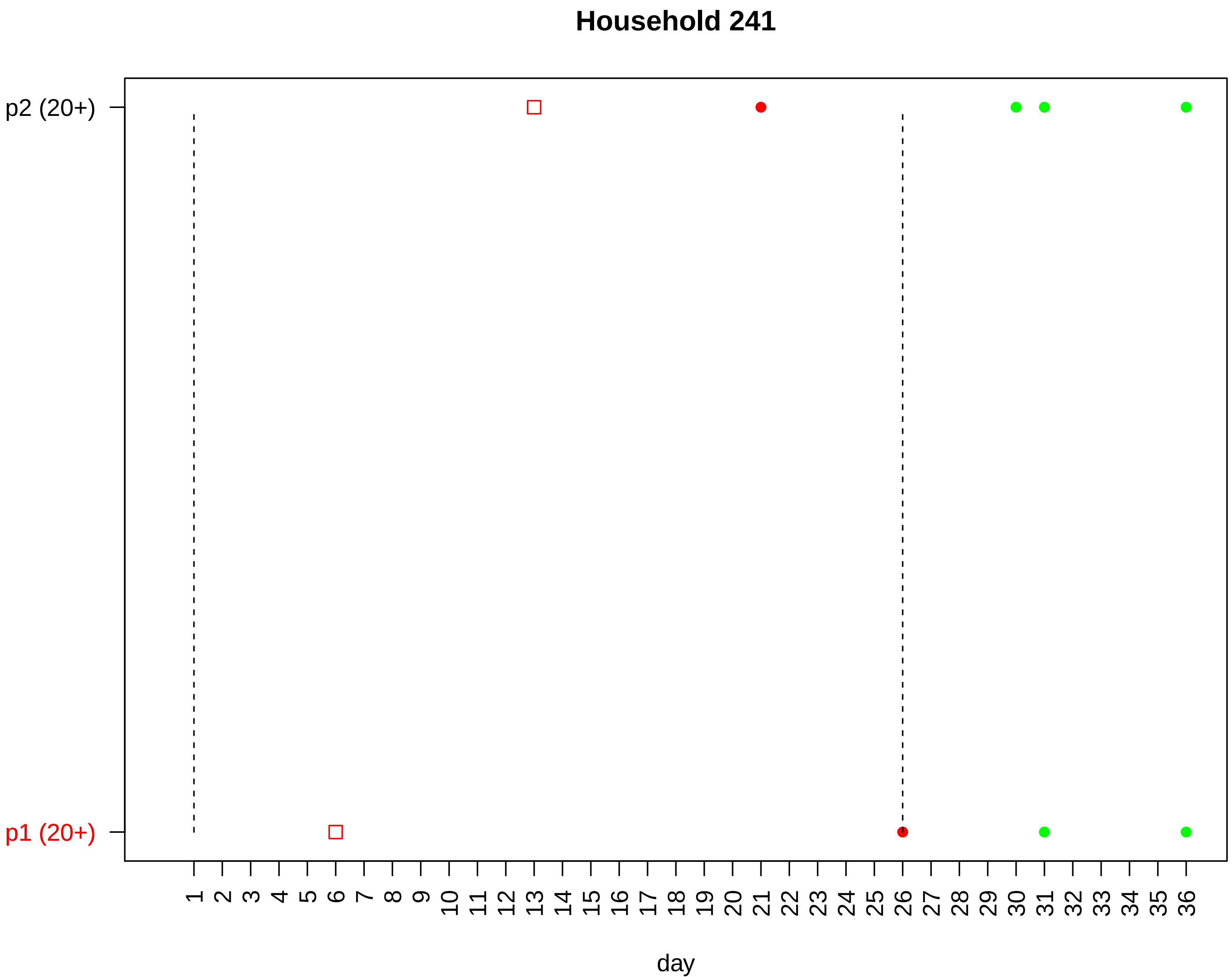

Household 241
p2 (20+)
p1 (20+)
1
2
3
4
5
6
7
8
9
10
11
12
13
14
15
16
17
18
19
20
21
22
23
24
25
26
27
28
29
30
31
32
33
34
35
36
day

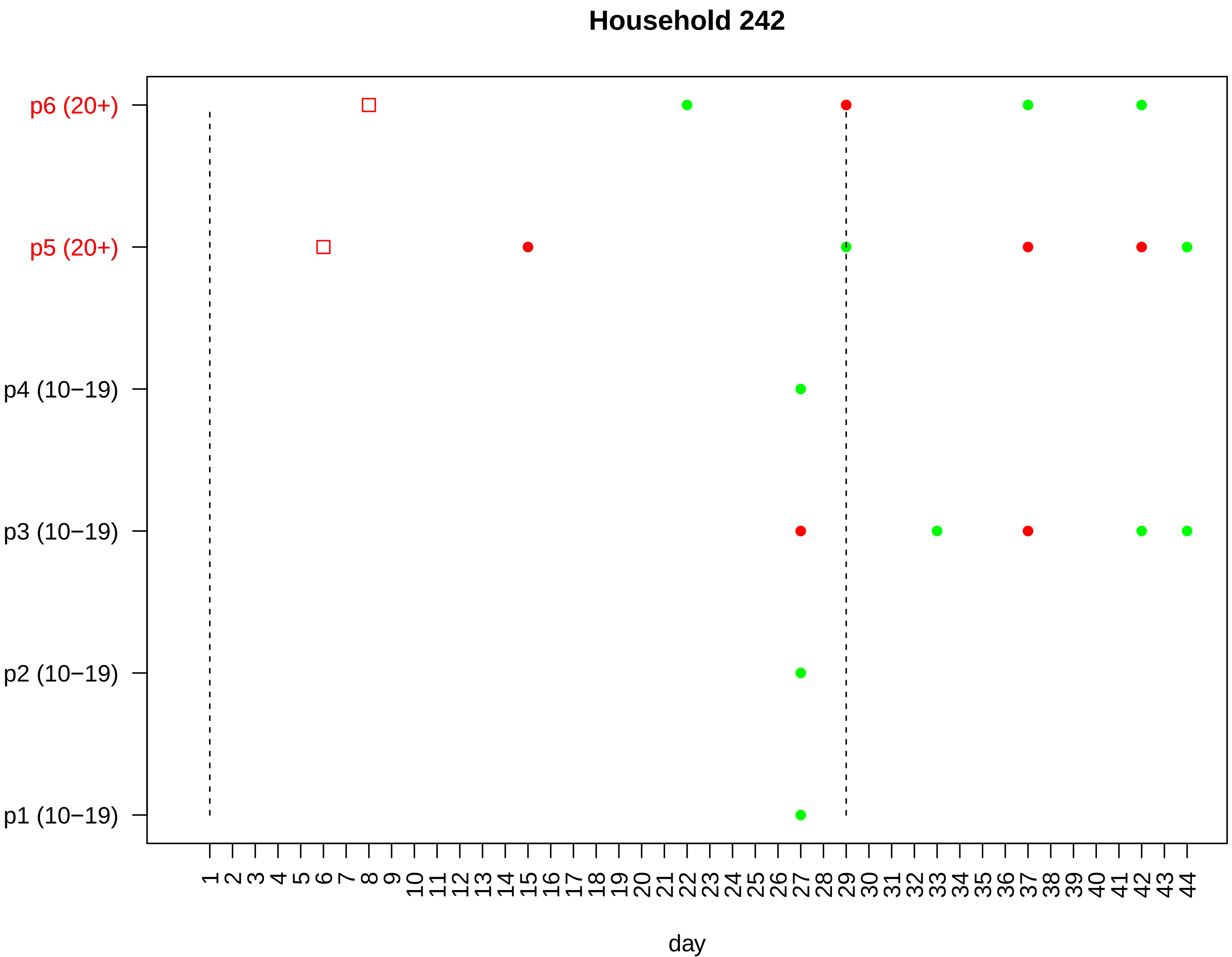

Household 242
p6 (20+)
p5 (20+)
p4 (10−19)
p3 (10−19)
p2 (10−19)
p1 (10−19)
1 2 3 4 5 6 7 8 9 10 11 12 13 14 15 16 17 18 19 20 21 22 23 24 25 26 27 28 29 30 31 32 33 34 35 36 37 38 39 40 41 42 43 44
day

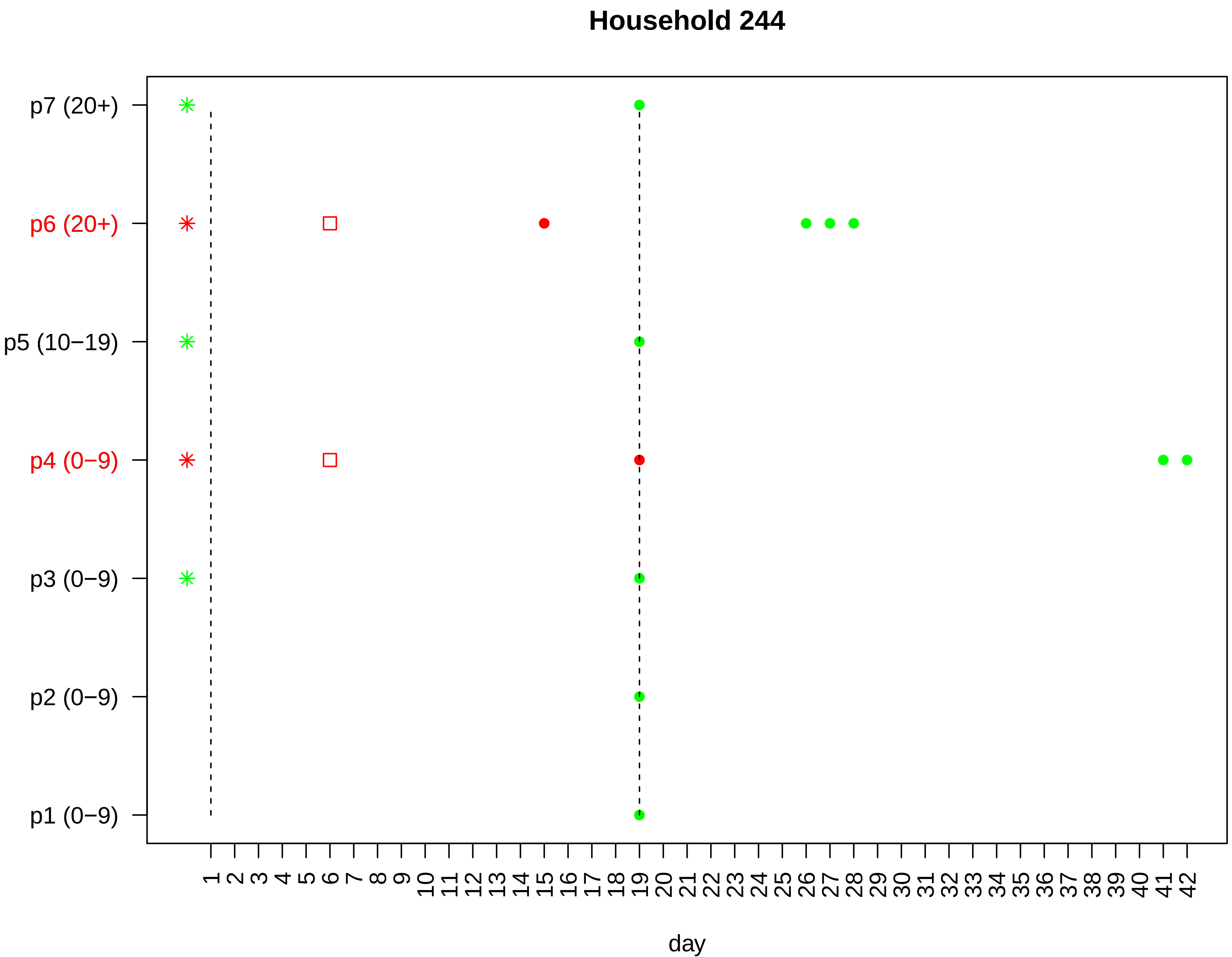

Household 244
p7 (20+)
p6 (20+)
p5 (10−19)
p4 (0−9)
p3 (0−9)
p2 (0−9)
p1 (0−9)
1 2 3 4 5 6 7 8 9 10 11 12 13 14 15 16 17 18 19 20 21 22 23 24 25 26 27 28 29 30 31 32 33 34 35 36 37 38 39 40 41 42
day

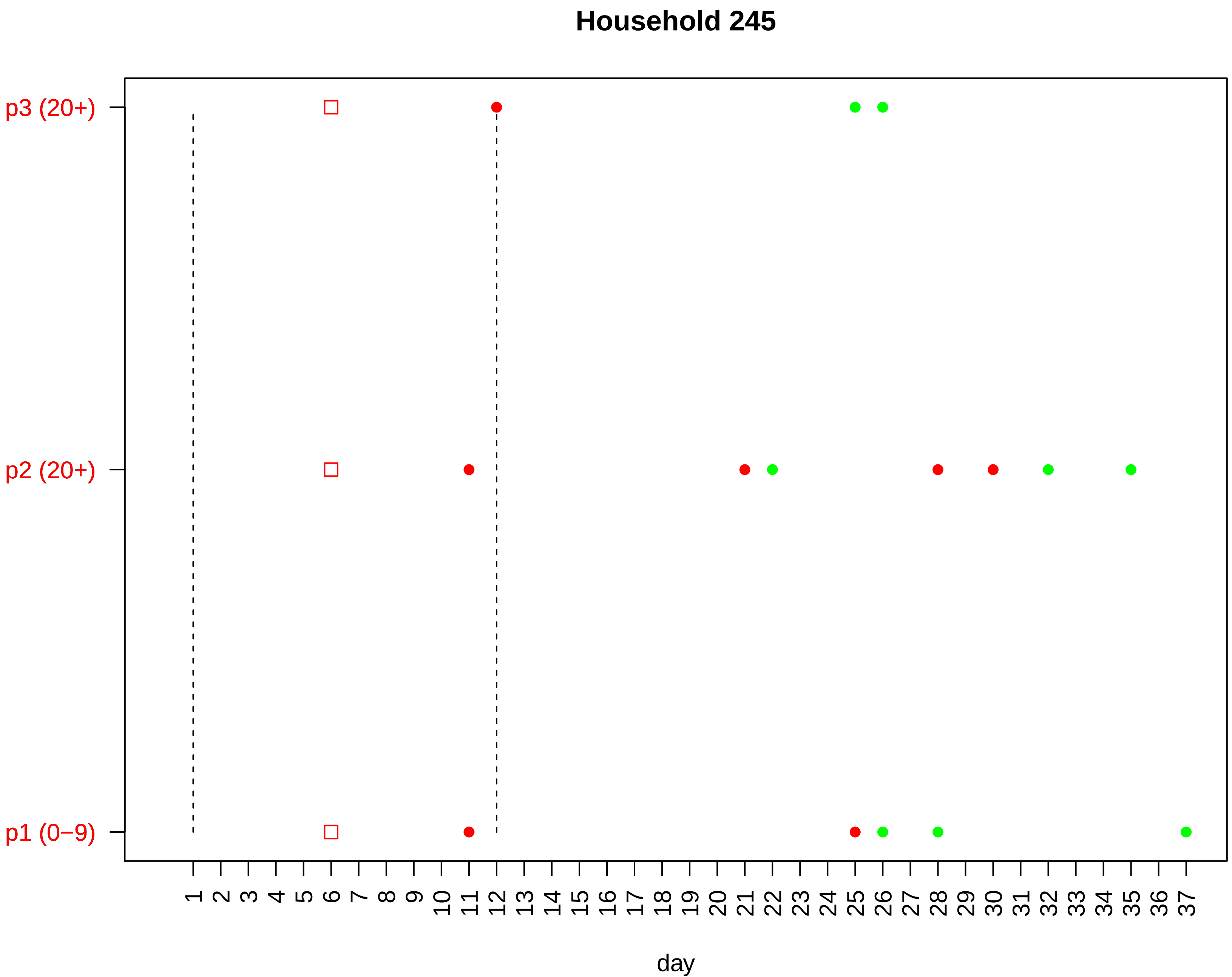

Household 245
p3 (20+)
p2 (20+)
p1 (0−9)
1 2 3 4 5 6 7 8 9 10 11 12 13 14 15 16 17 18 19 20 21 22 23 24 25 26 27 28 29 30 31 32 33 34 35 36 37
day

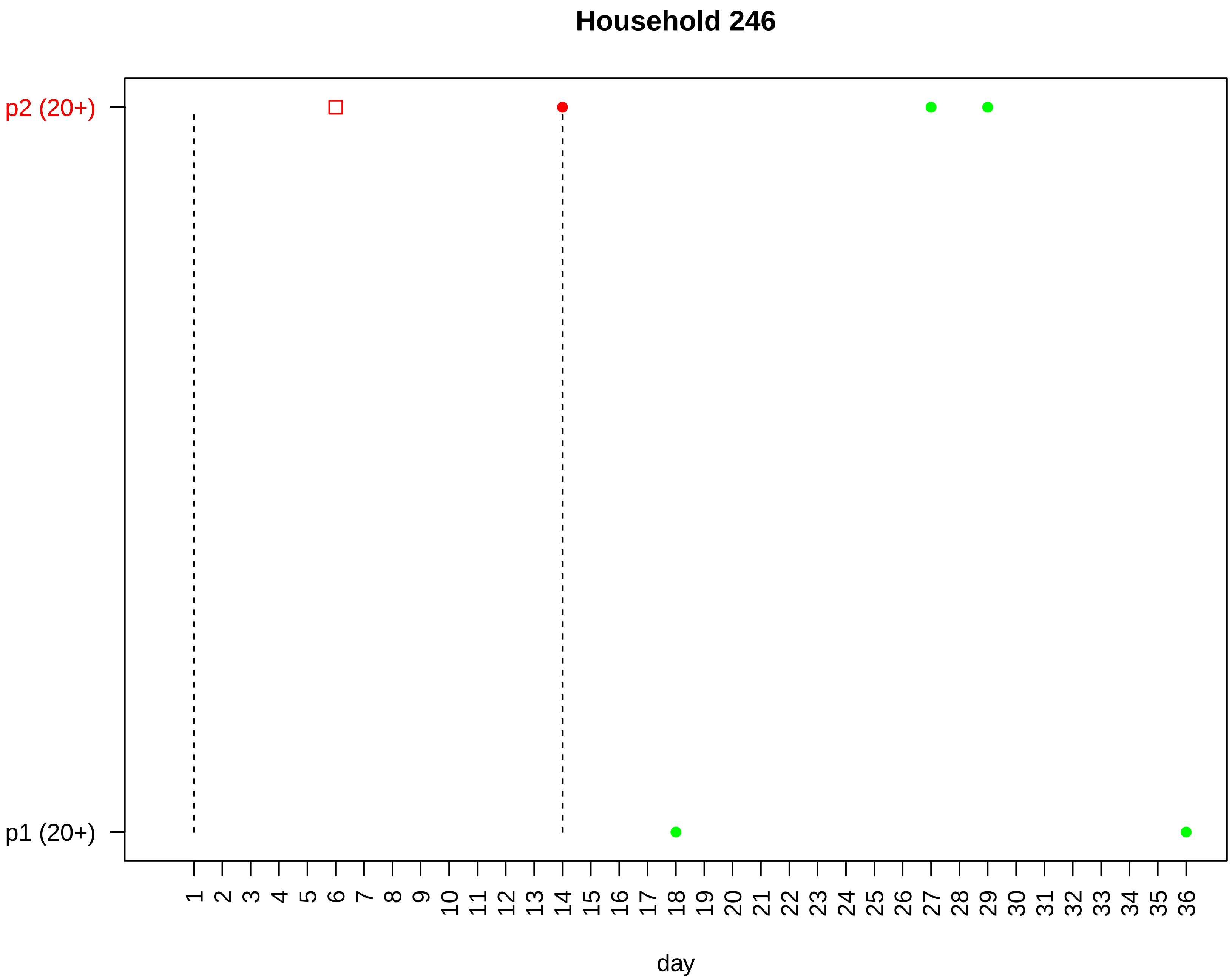

Household 246
p2 (20+)
p1 (20+)
1
2
3
4
5
6
7
8
9
10
11
12
13
14
15
16
17
18
19
20
21
22
23
24
25
26
27
28
29
30
31
32
33
34
35
36
day

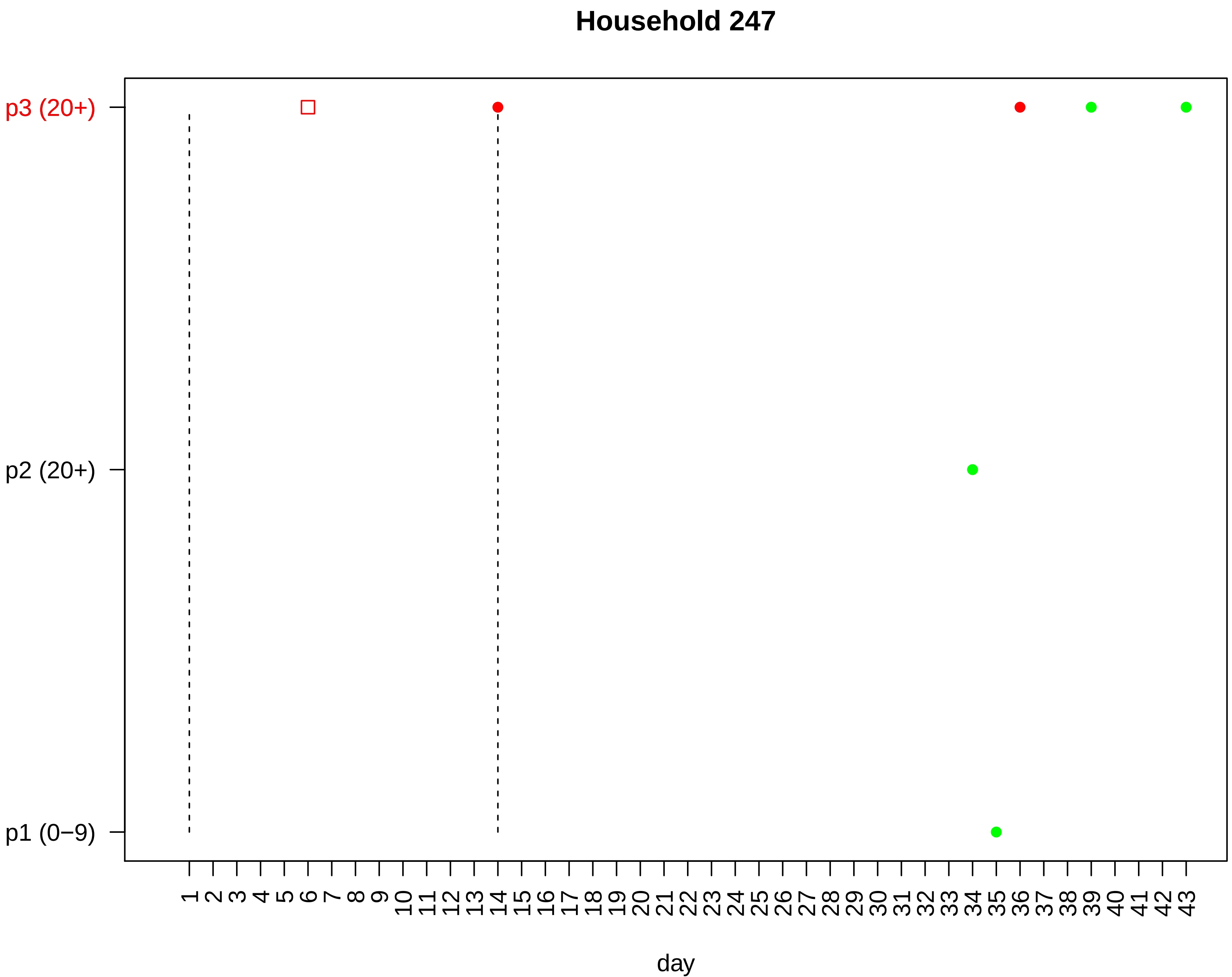
Household 247
p3 (20+)
p2 (20+)
p1 (0−9)
1 2 3 4 5 6 7 8 9 10 11 12 13 14 15 16 17 18 19 20 21 22 23 24 25 26 27 28 29 30 31 32 33 34 35 36 37 38 39 40 41 42 43
day

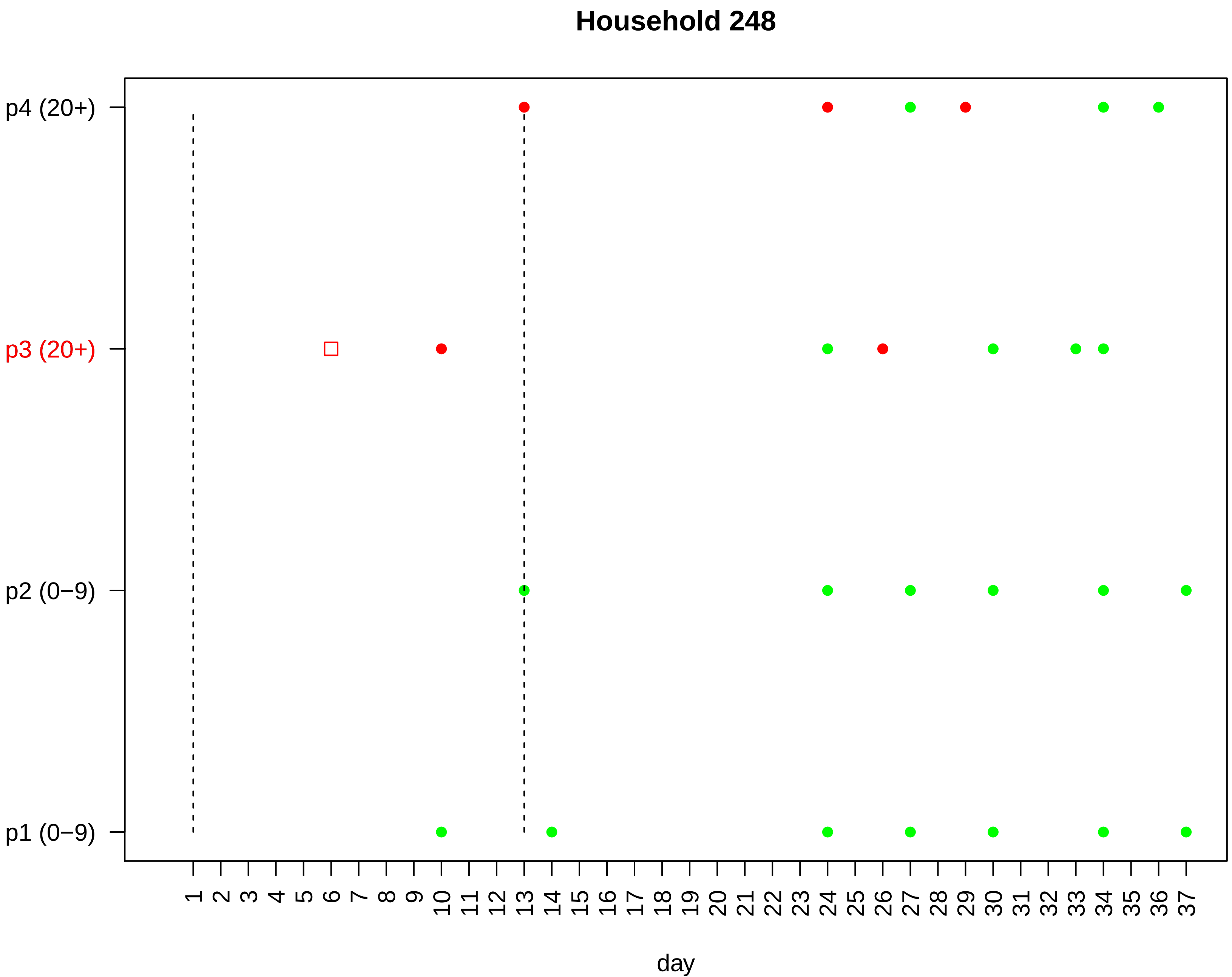

Household 248
p4 (20+)
p3 (20+)
p2 (0−9)
p1 (0−9)
1 2 3 4 5 6 7 8 9 10 11 12 13 14 15 16 17 18 19 20 21 22 23 24 25 26 27 28 29 30 31 32 33 34 35 36 37
day

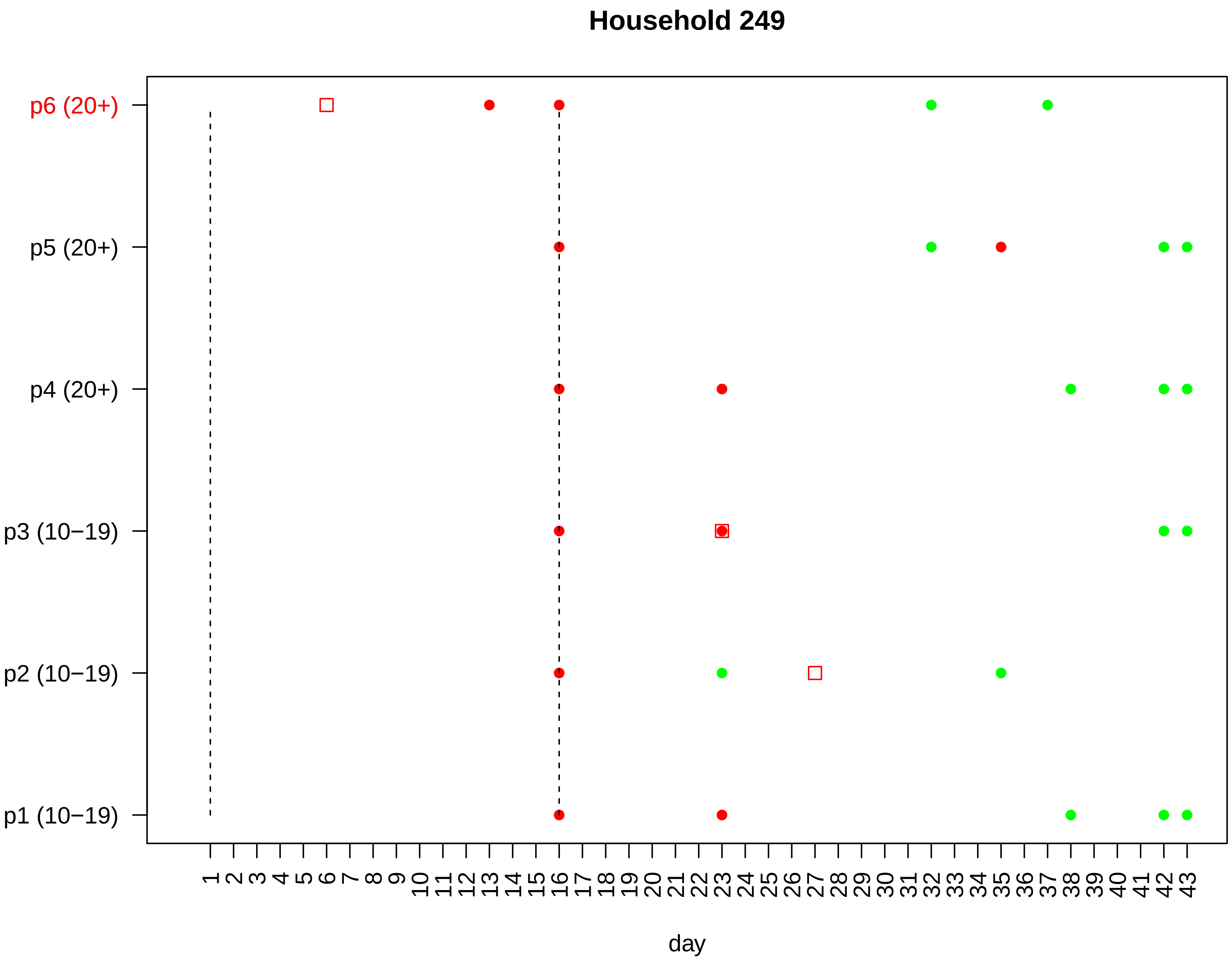

Household 249
p6 (20+)
p5 (20+)
p4 (20+)
p3 (10−19)
p2 (10−19)
p1 (10−19)
1 2 3 4 5 6 7 8 9 10 11 12 13 14 15 16 17 18 19 20 21 22 23 24 25 26 27 28 29 30 31 32 33 34 35 36 37 38 39 40 41 42 43
day

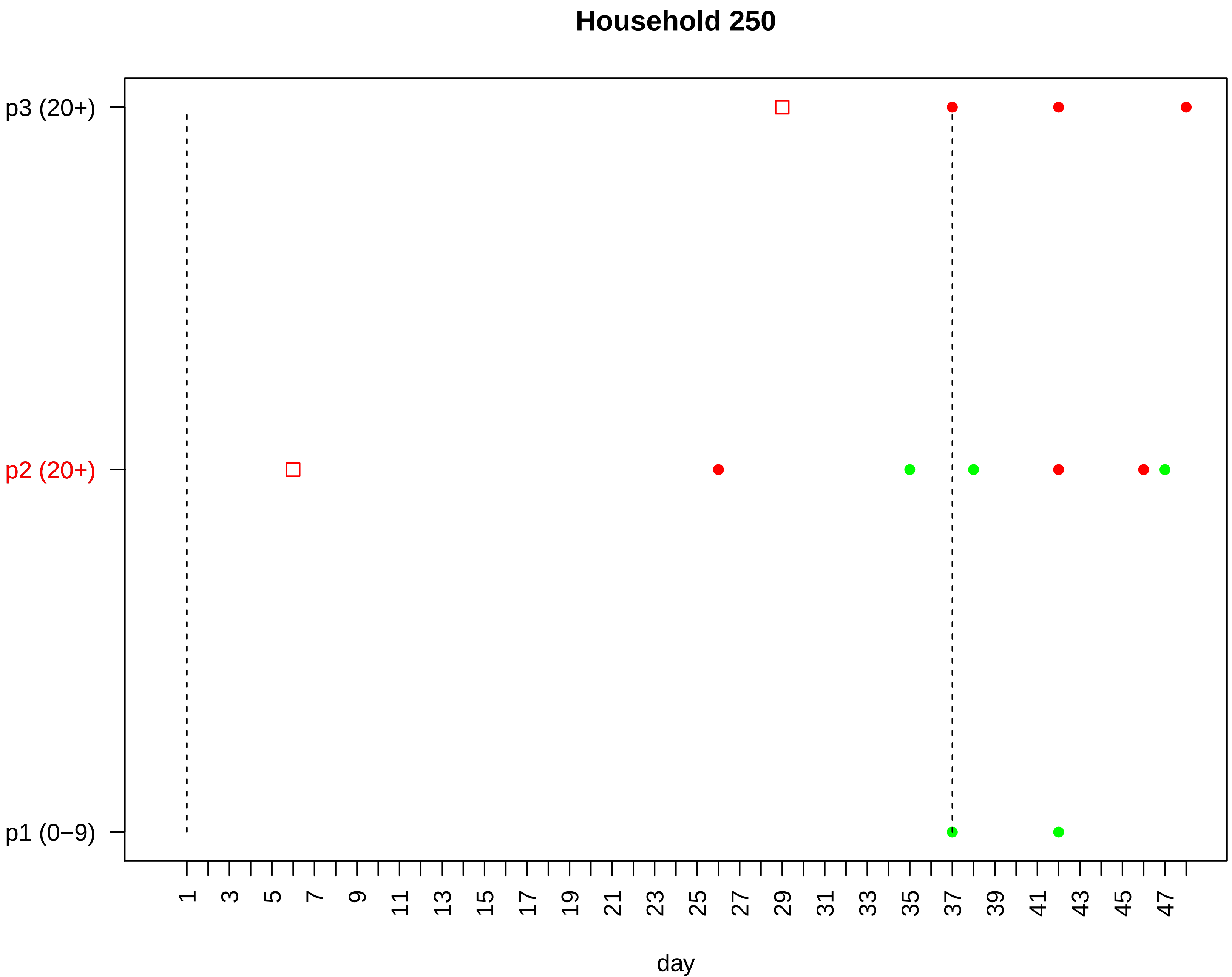

Household 250
p3 (20+)
p2 (20+)
p1 (0−9)
1
3
5
7
9
11
13
15
17
19
21
23
25
27
29
31
33
35
37
39
41
43
45
47
day

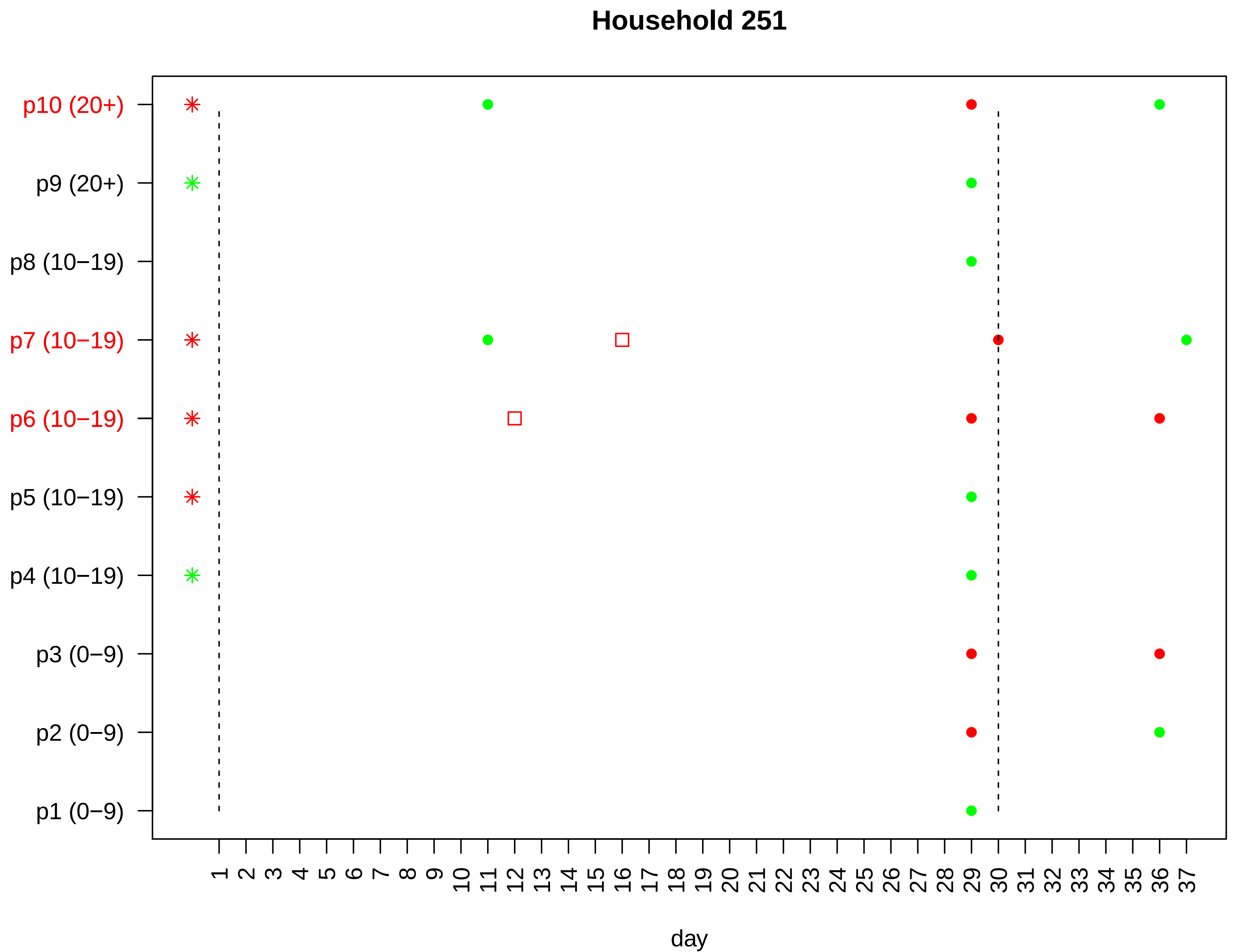

Household 251
p10 (20+)
p9 (20+)
p8 (10−19)
p7 (10−19)
p6 (10−19)
p5 (10−19)
p4 (10−19)
p3 (0−9)
p2 (0−9)
p1 (0−9)
1 2 3 4 5 6 7 8 9 10 11 12 13 14 15 16 17 18 19 20 21 22 23 24 25 26 27 28 29 30 31 32 33 34 35 36 37
day

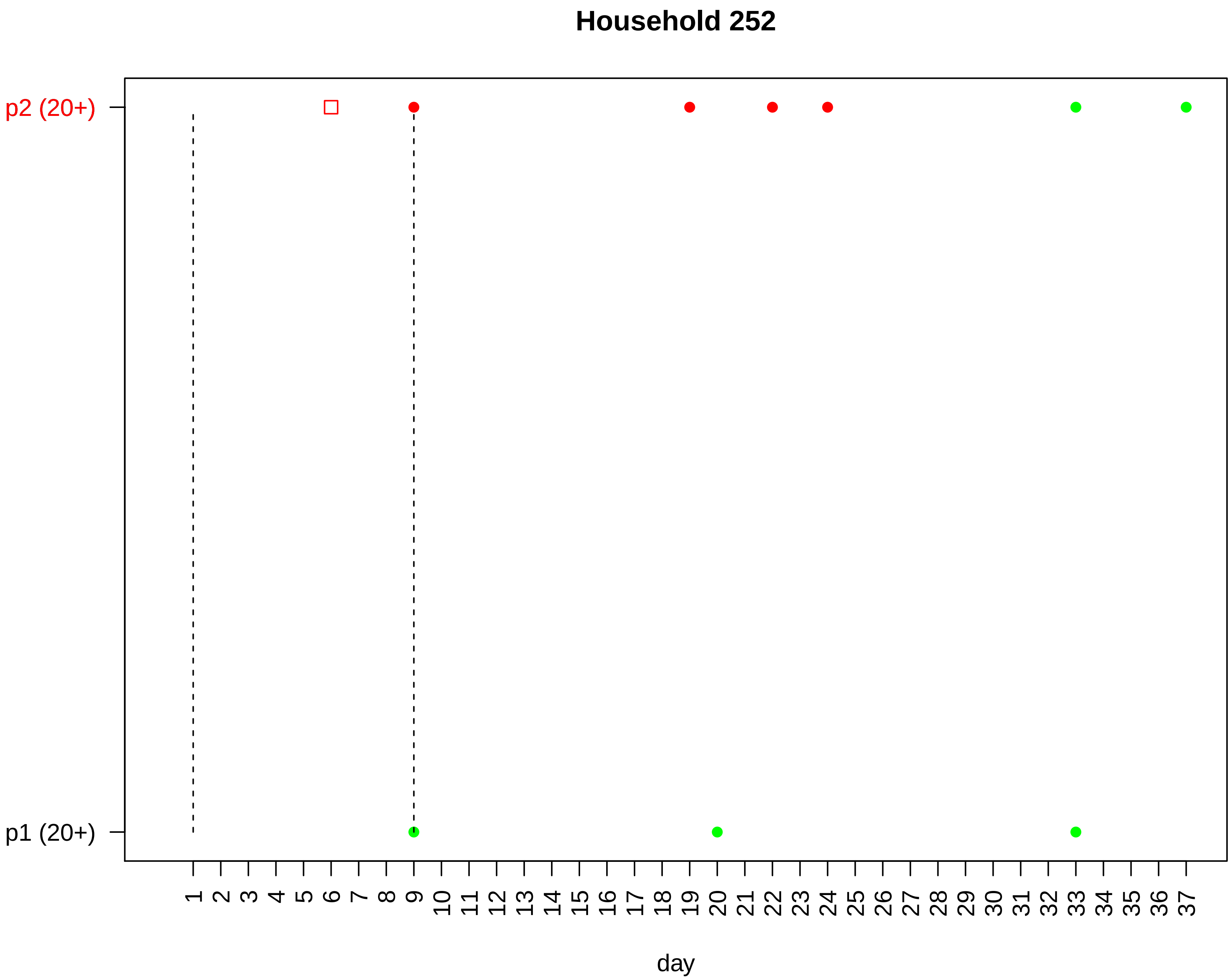

Household 252
p2 (20+)
p1 (20+)
1
2
3
4
5
6
7
8
9
10
11
12
13
14
15
16
17
18
19
20
21
22
23
24
25
26
27
28
29
30
31
32
33
34
35
36
37
day

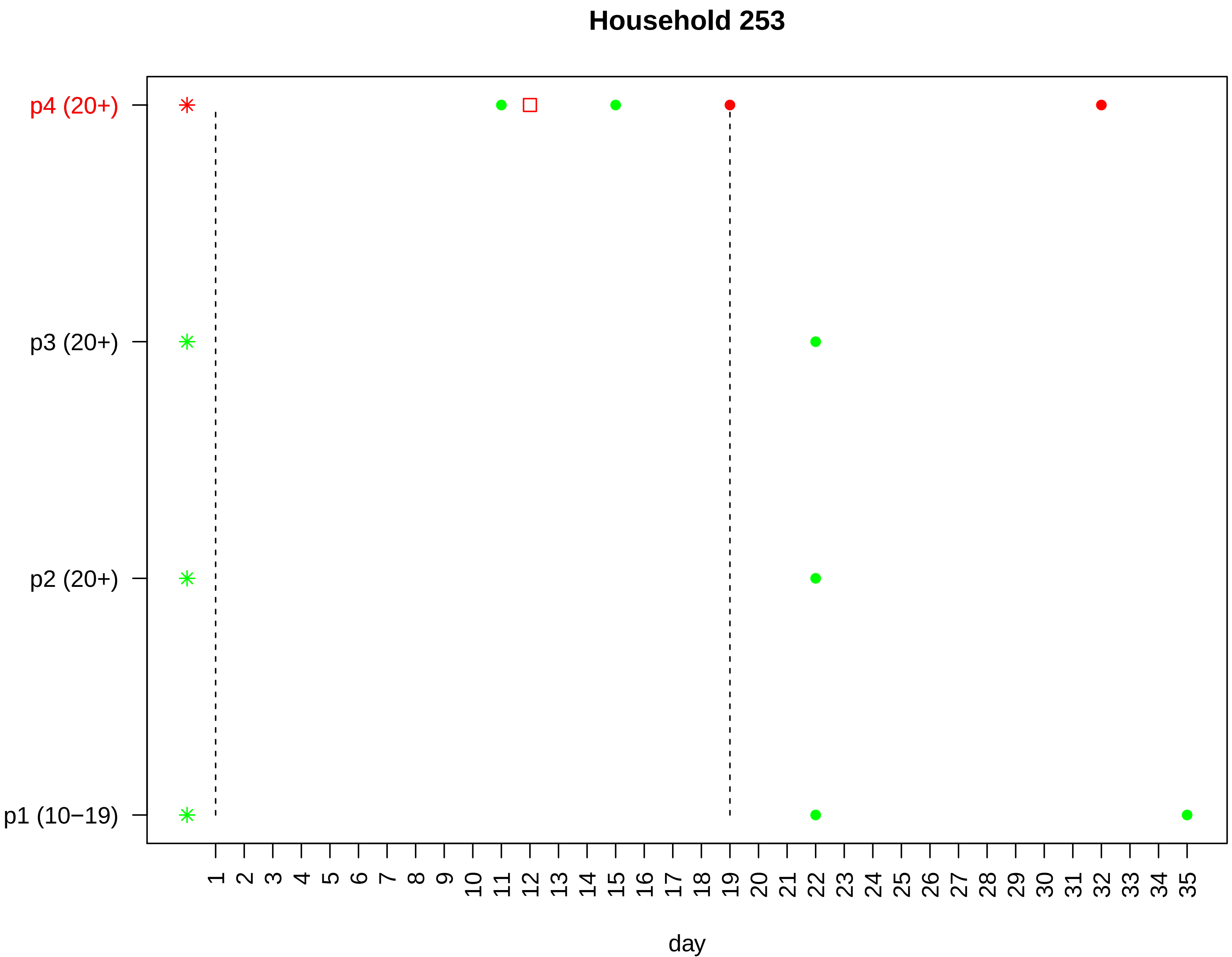

Household 253
p4 (20+)
p3 (20+)
p2 (20+)
p1 (10−19)
1 2 3 4 5 6 7 8 9 10 11 12 13 14 15 16 17 18 19 20 21 22 23 24 25 26 27 28 29 30 31 32 33 34 35
day

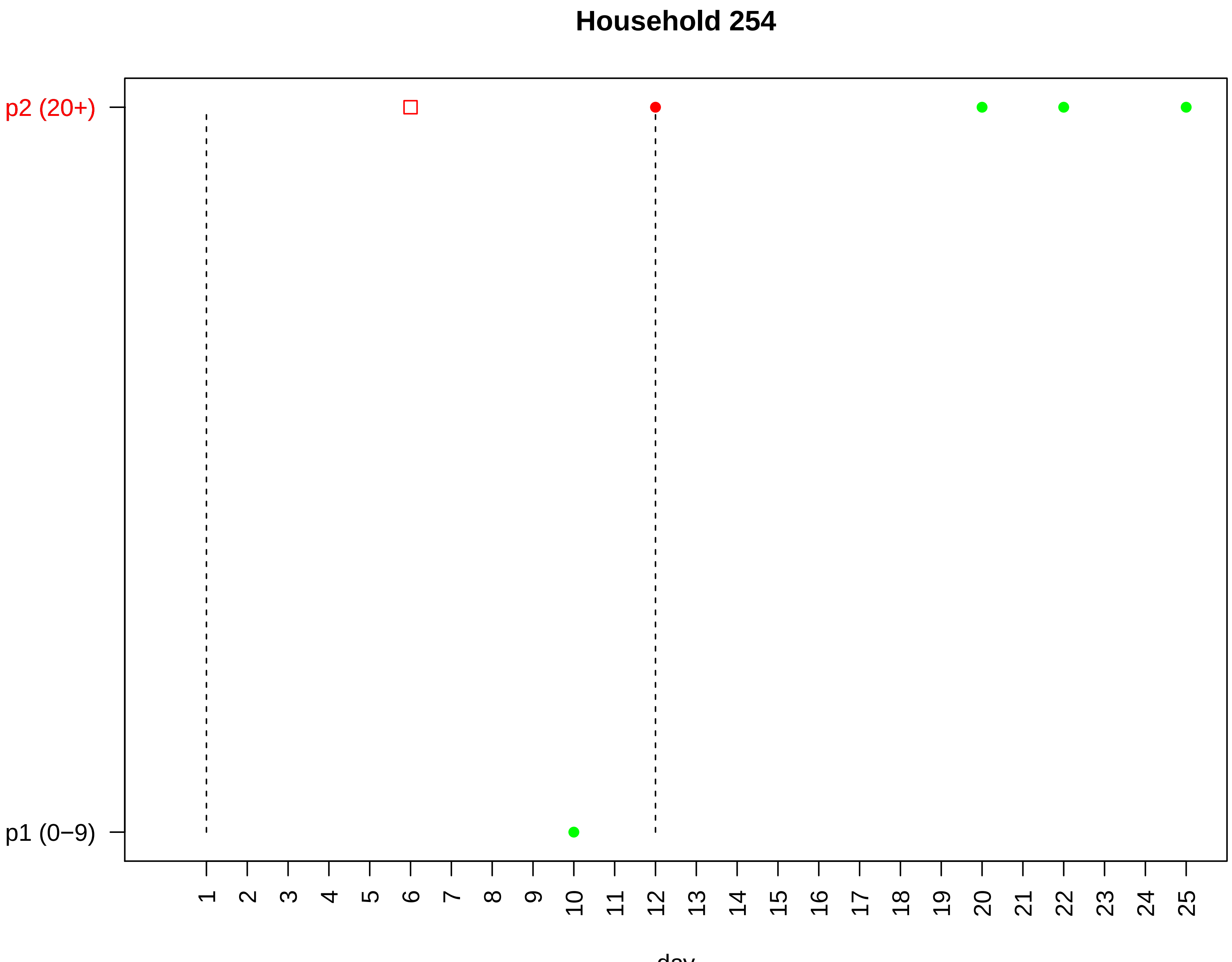
Household 254
p2 (20+)
p1 (0−9)
1
2
3
4
5
6
7
8
9
10
11
12
13
14
15
16
17
18
19
20
21
22
23
24
25
day

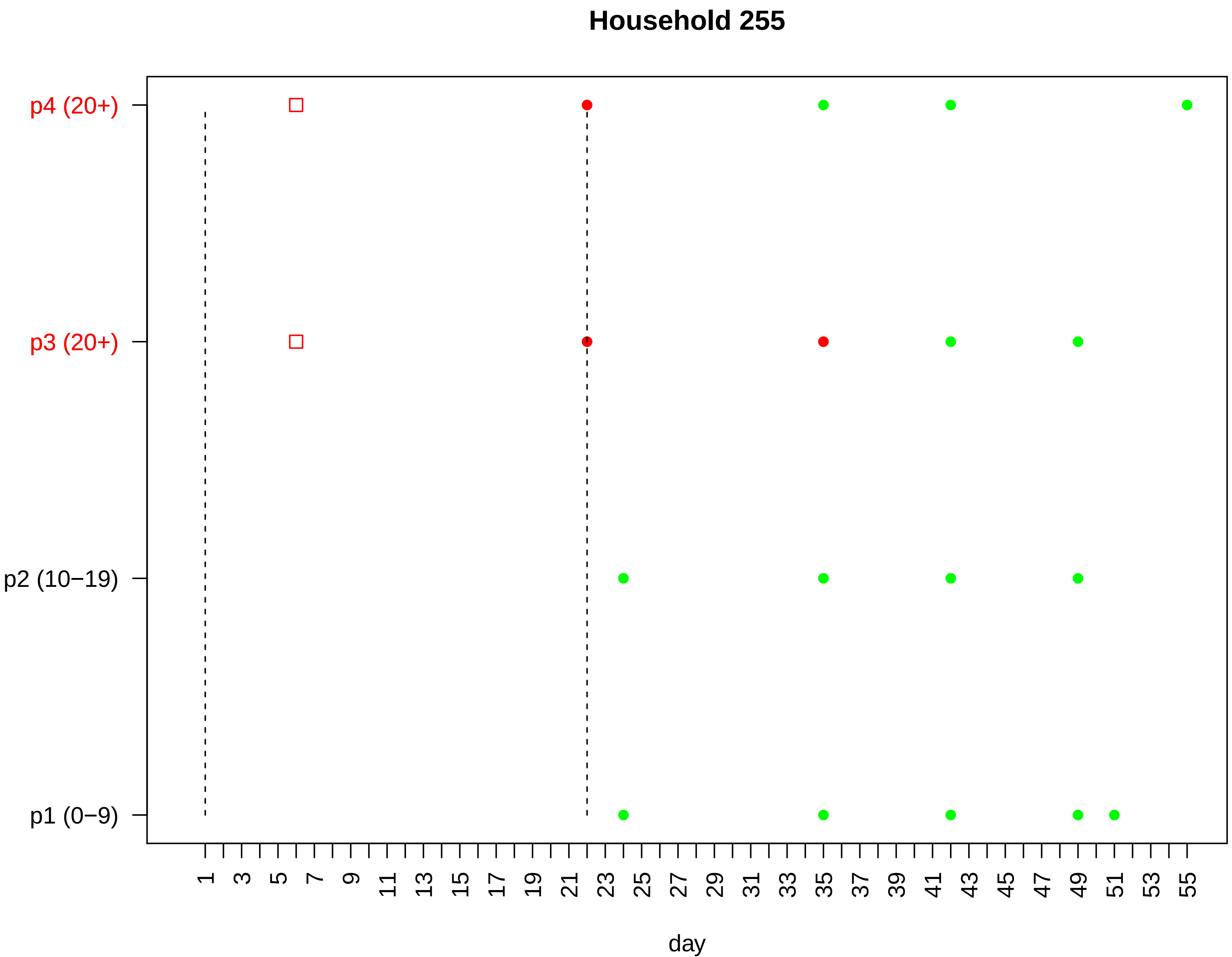

Household 255
p4 (20+)
p3 (20+)
p2 (10−19)
p1 (0−9)
1
3
5
7
9
11
13
15
17
19
21
23
25
27
29
31
33
35
37
39
41
43
45
47
49
51
53
55
day

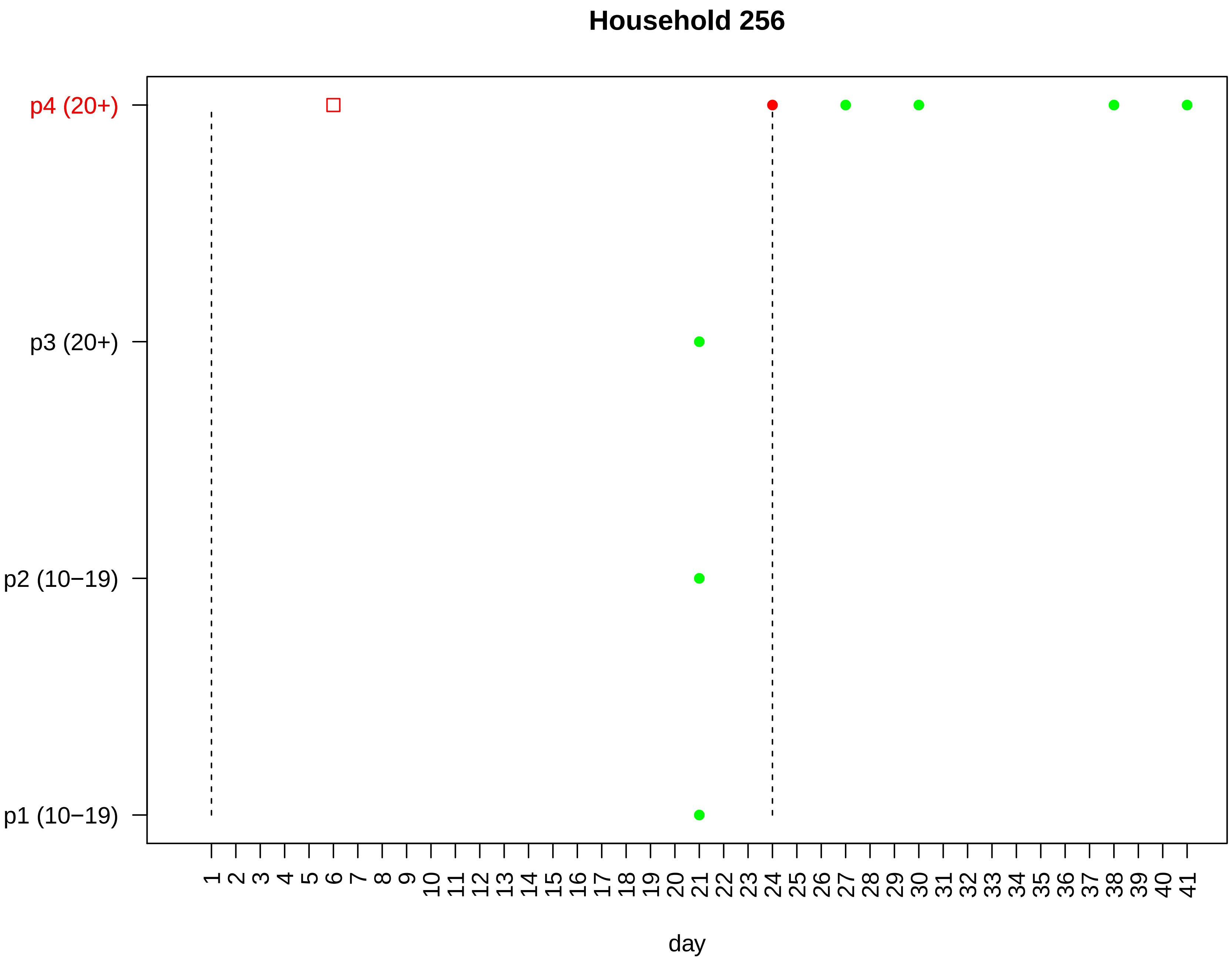

Household 256
p4 (20+)
p3 (20+)
p2 (10−19)
p1 (10−19)
1 2 3 4 5 6 7 8 9 10 11 12 13 14 15 16 17 18 19 20 21 22 23 24 25 26 27 28 29 30 31 32 33 34 35 36 37 38 39 40 41
day

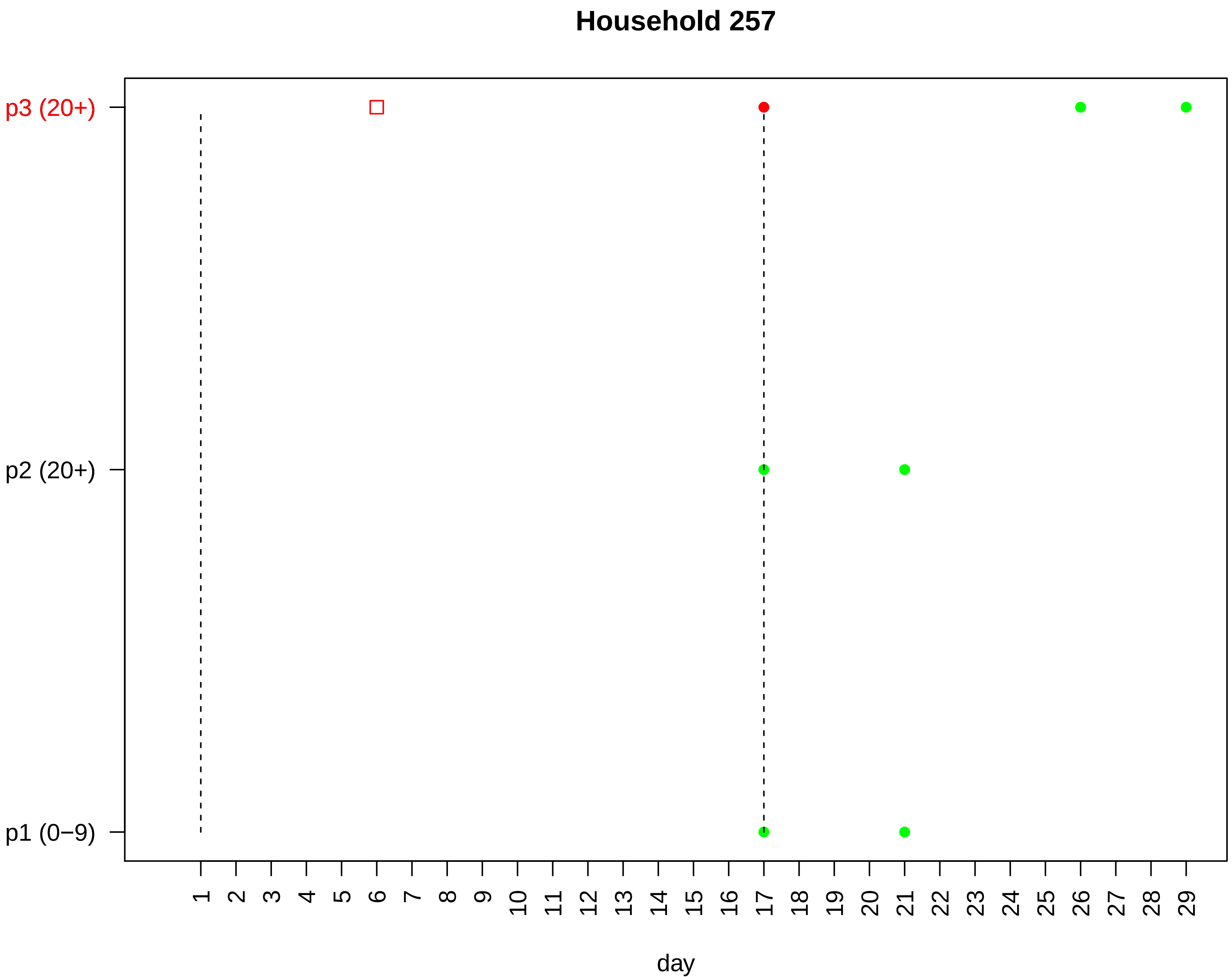

Household 257
p3 (20+)
p2 (20+)
p1 (0−9)
1
2
3
4
5
6
7
8
9
10
11
12
13
14
15
16
17
18
19
20
21
22
23
24
25
26
27
28
29
day

Household 258

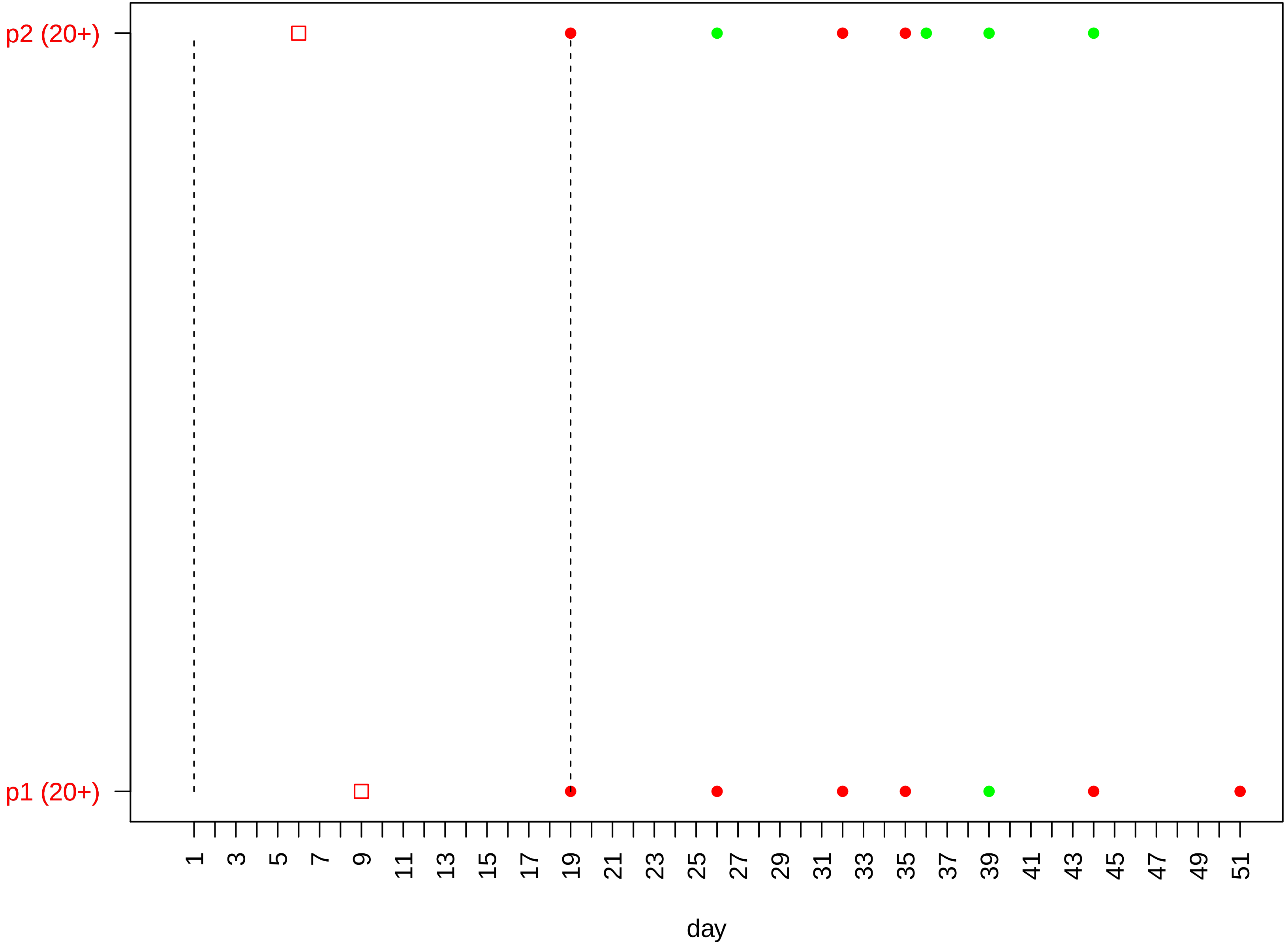

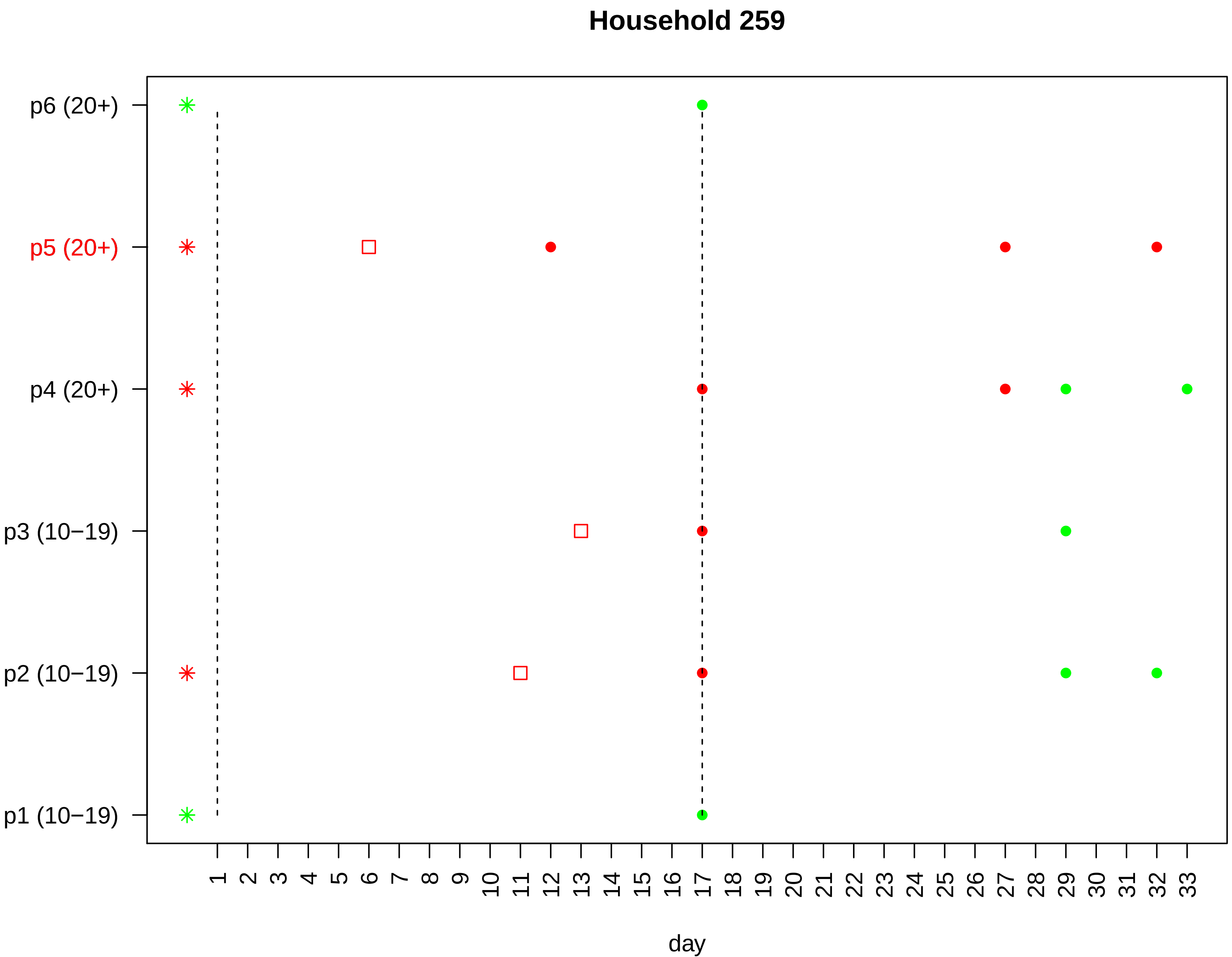
Household 259
p6 (20+)
p5 (20+)
p4 (20+)
p3 (10−19)
p2 (10−19)
p1 (10−19)
1 2 3 4 5 6 7 8 9 10 11 12 13 14 15 16 17 18 19 20 21 22 23 24 25 26 27 28 29 30 31 32 33
day

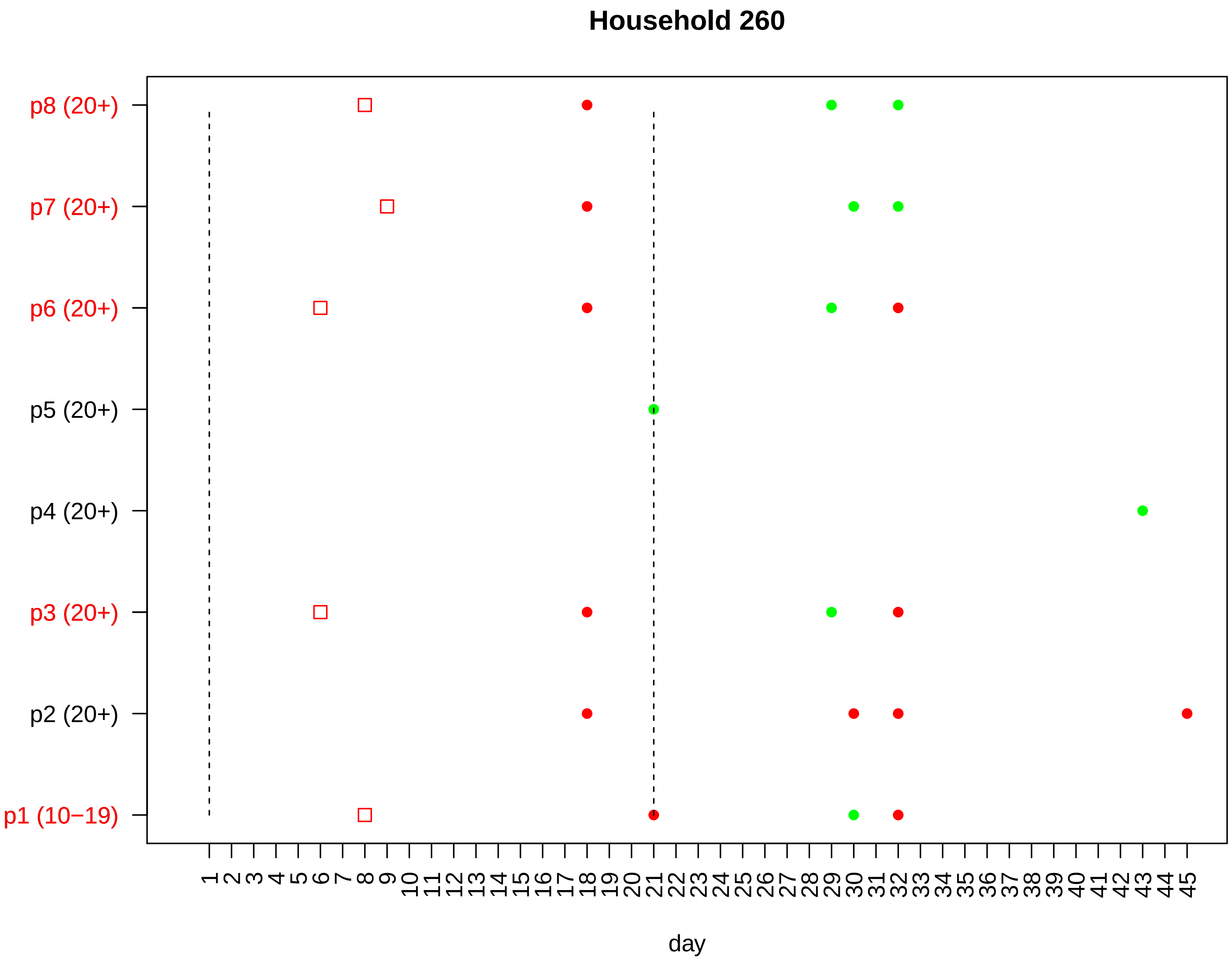

Household 260
p8 (20+)
p7 (20+)
p6 (20+)
p5 (20+)
p4 (20+)
p3 (20+)
p2 (20+)
p1 (10−19)
1 2 3 4 5 6 7 8 9 10 11 12 13 14 15 16 17 18 19 20 21 22 23 24 25 26 27 28 29 30 31 32 33 34 35 36 37 38 39 40 41 42 43 44 45
day

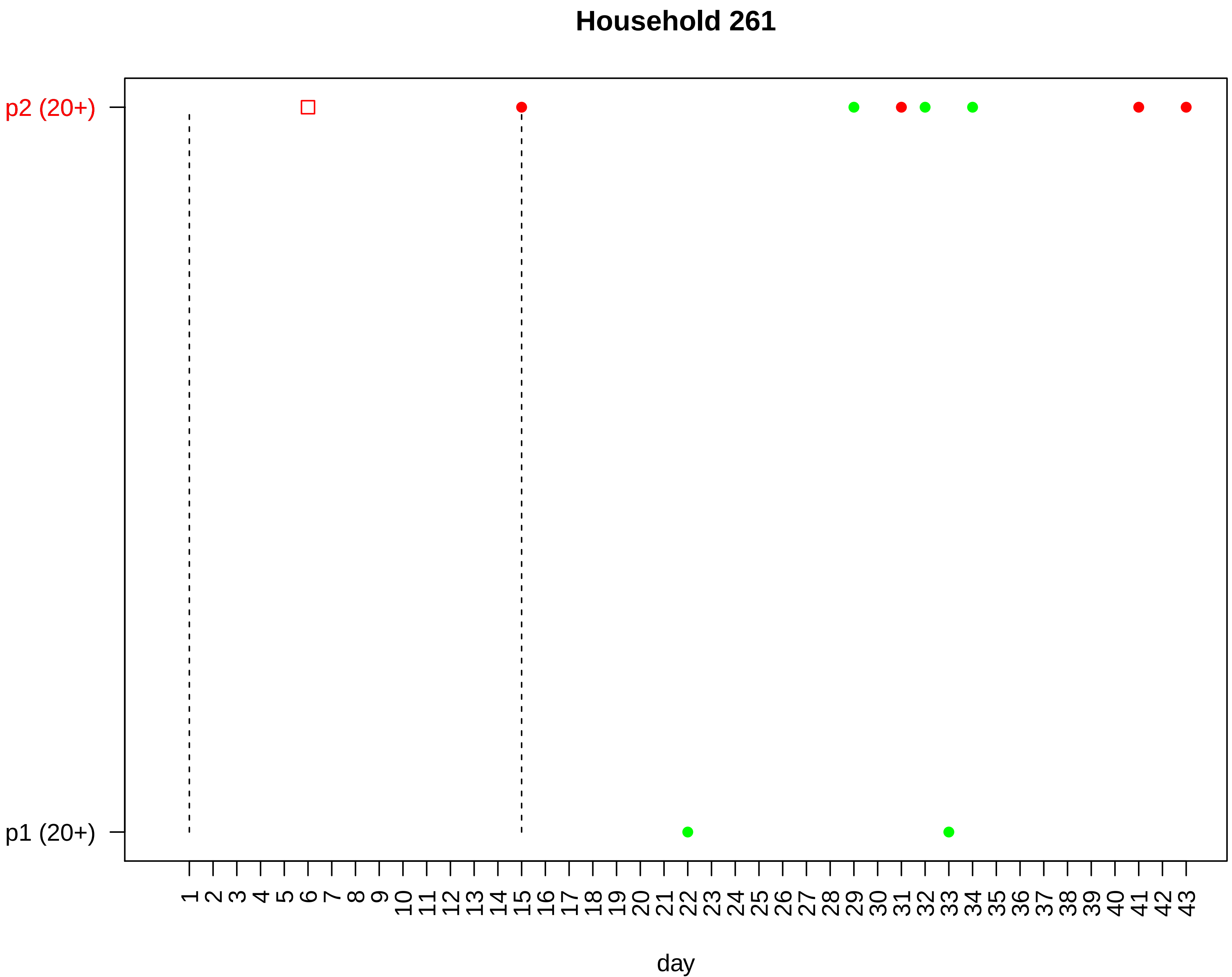
Household 261
p2 (20+)
p1 (20+)
1 2 3 4 5 6 7 8 9 10 11 12 13 14 15 16 17 18 19 20 21 22 23 24 25 26 27 28 29 30 31 32 33 34 35 36 37 38 39 40 41 42 43
day

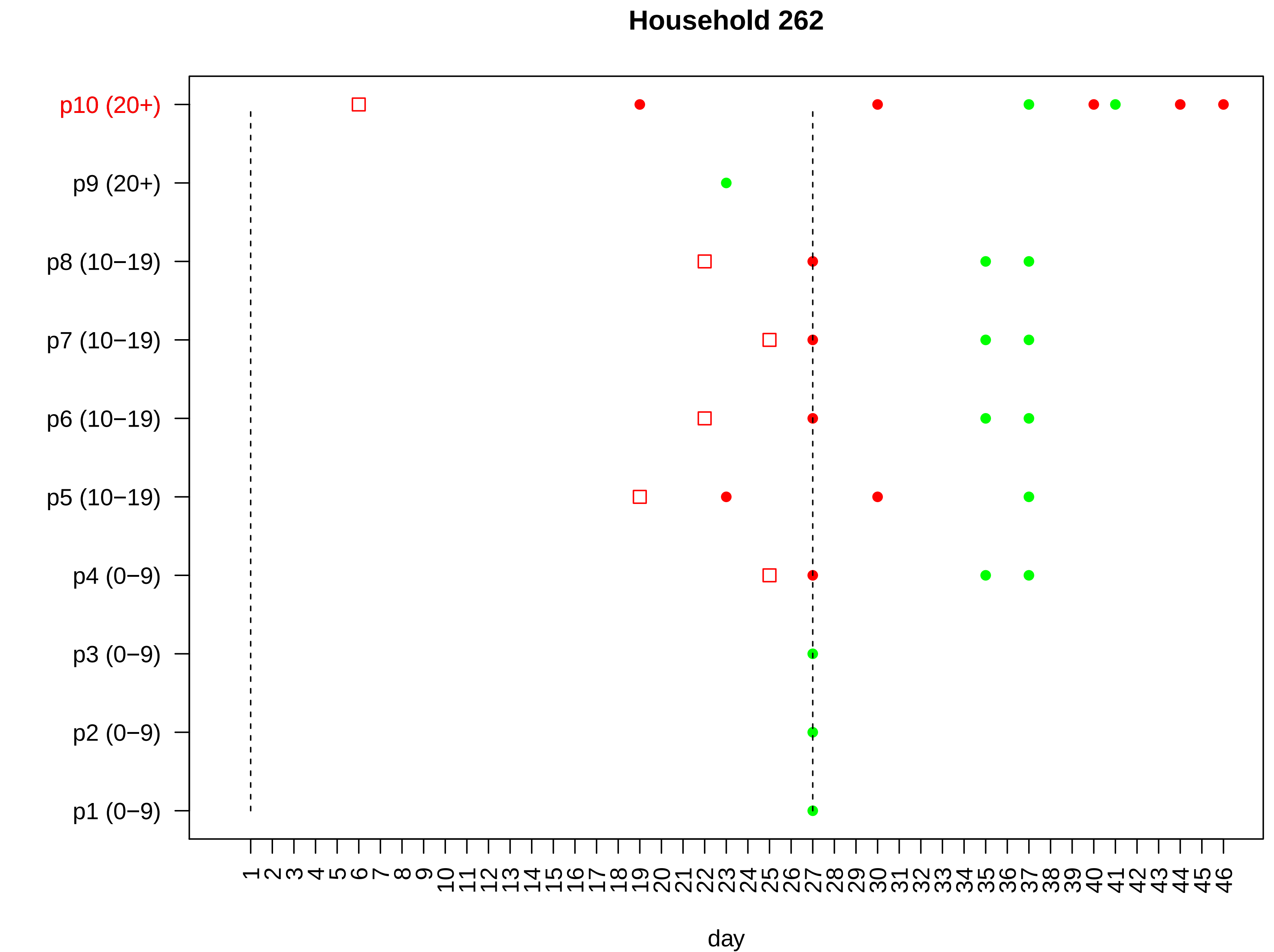

Household 262
p10 (20+)
p9 (20+)
p8 (10−19)
p7 (10−19)
p6 (10−19)
p5 (10−19)
p4 (0−9)
p3 (0−9)
p2 (0−9)
p1 (0−9)
1 2 3 4 5 6 7 8 9 10 11 12 13 14 15 16 17 18 19 20 21 22 23 24 25 26 27 28 29 30 31 32 33 34 35 36 37 38 39 40 41 42 43 44 45 46
day

Household 263

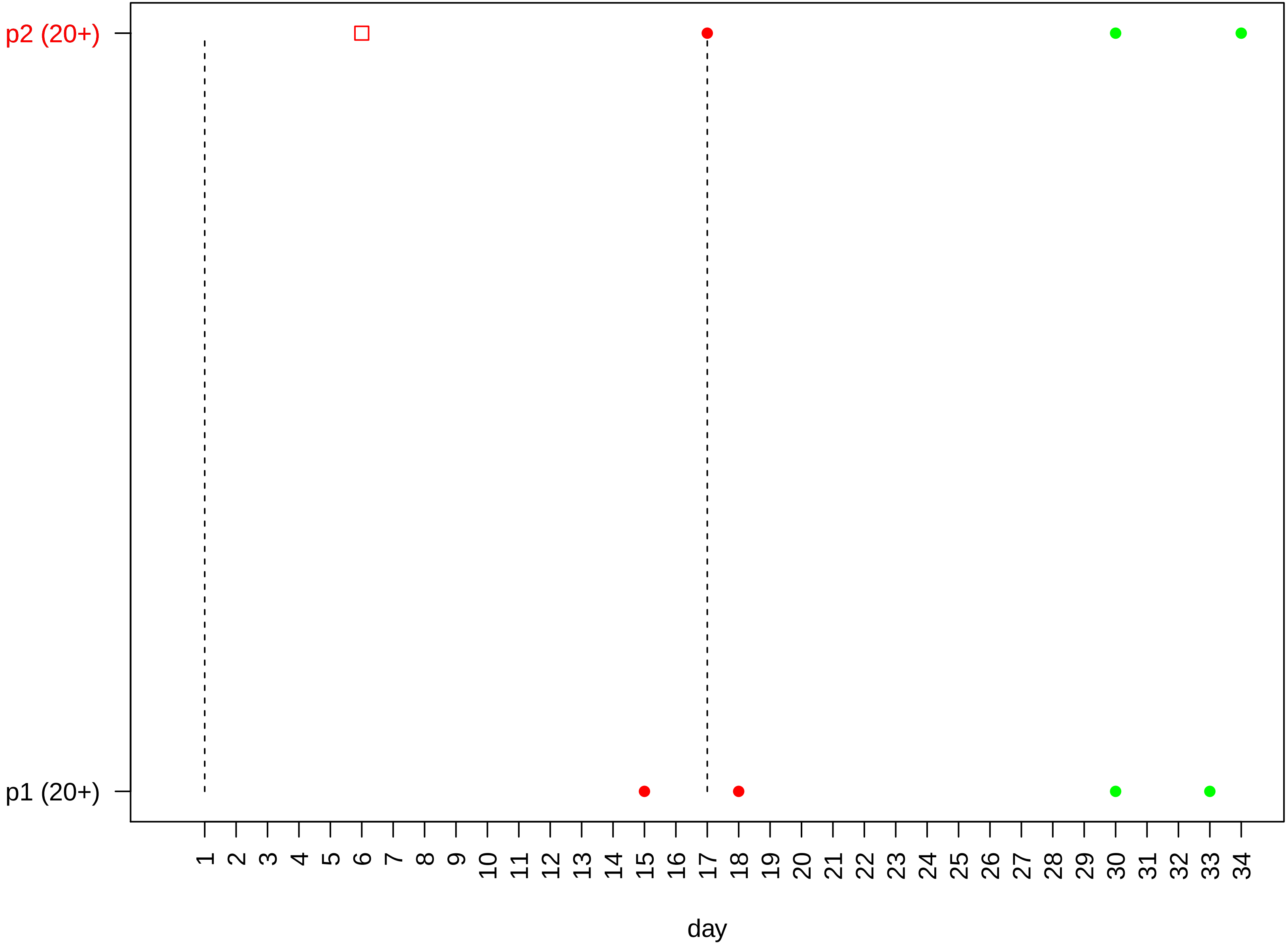

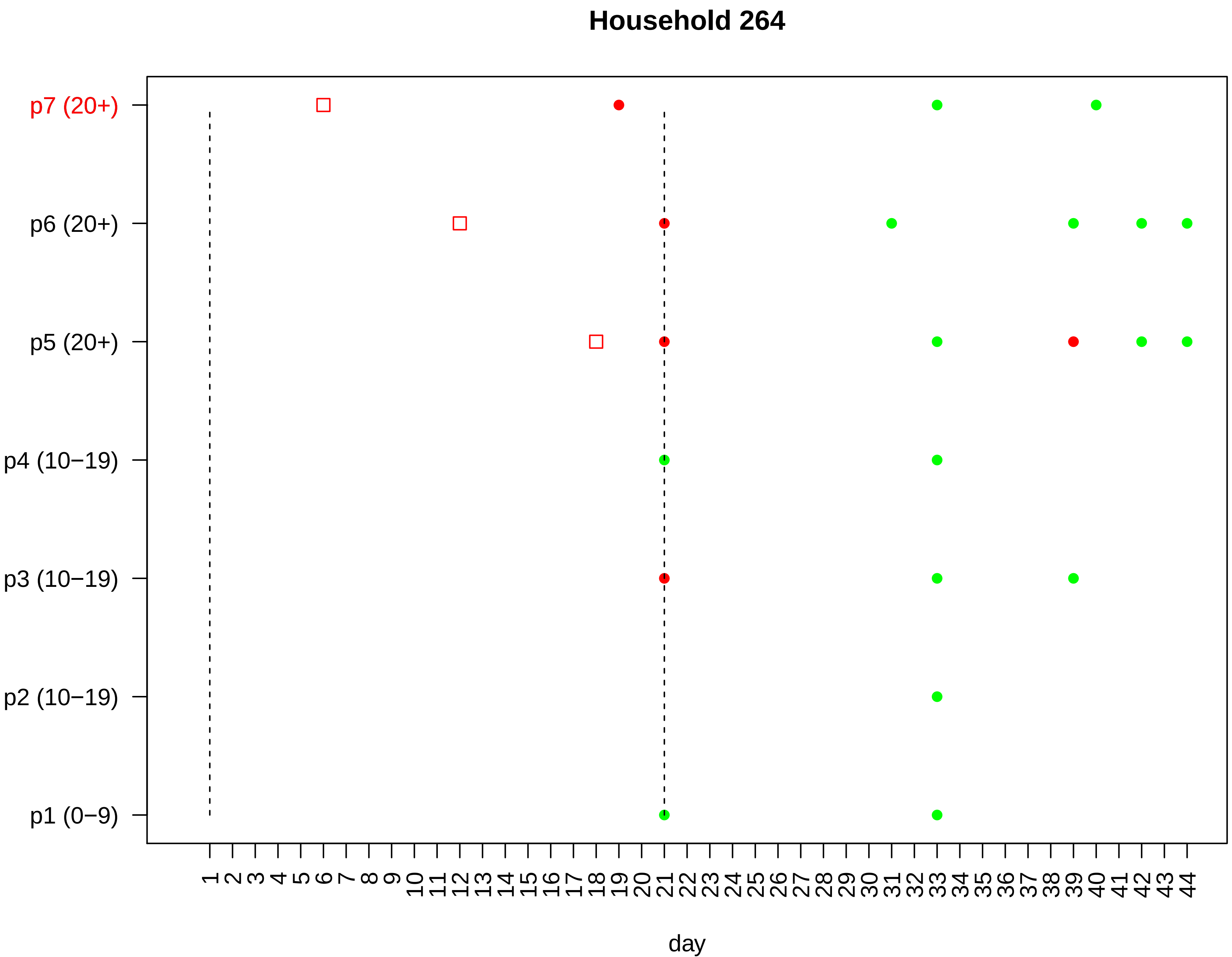

Household 264
p7 (20+)
p6 (20+)
p5 (20+)
p4 (10−19)
p3 (10−19)
p2 (10−19)
p1 (0−9)
1 2 3 4 5 6 7 8 9 10 11 12 13 14 15 16 17 18 19 20 21 22 23 24 25 26 27 28 29 30 31 32 33 34 35 36 37 38 39 40 41 42 43 44
day

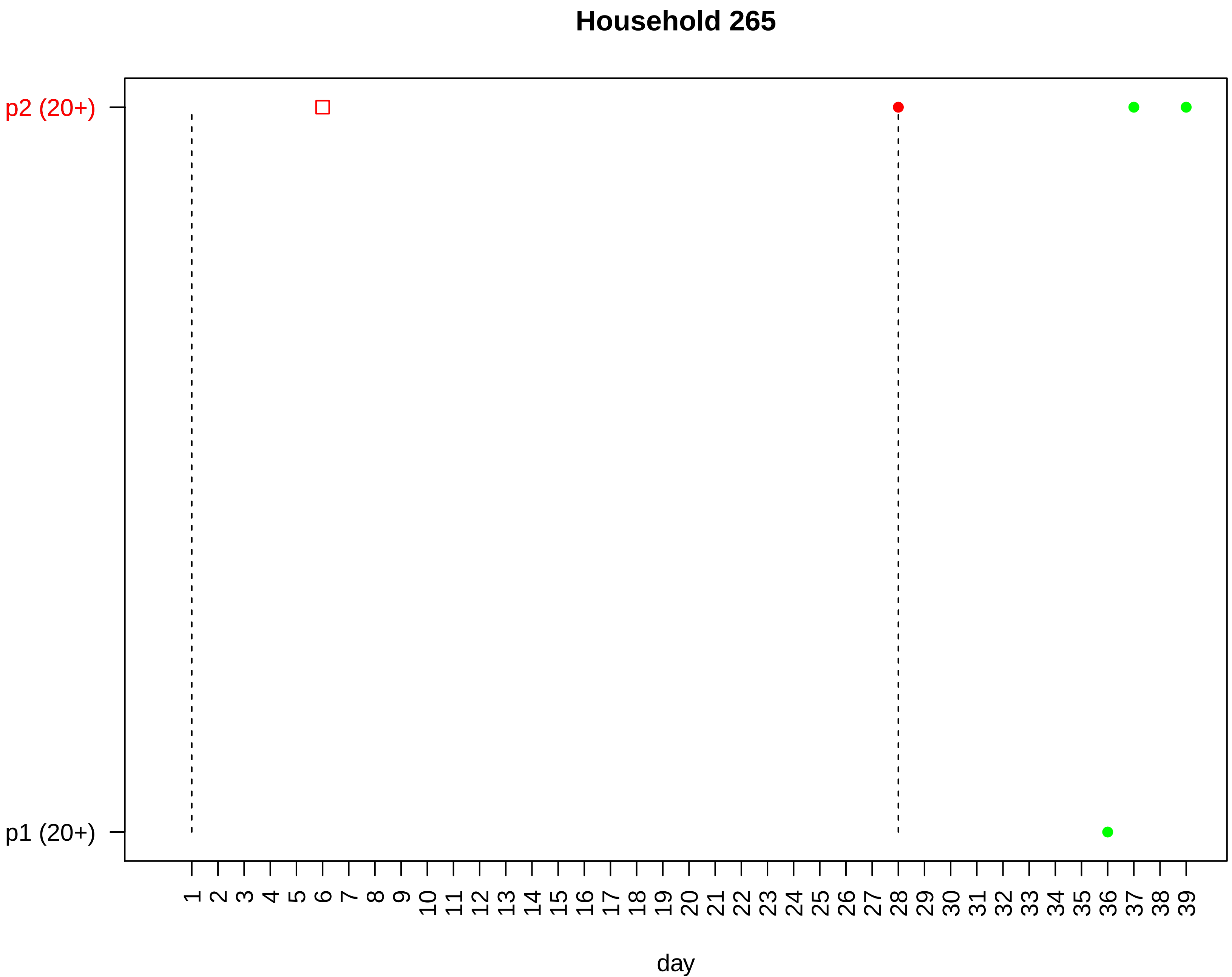

Household 265
p2 (20+)
p1 (20+)
1
2
3
4
5
6
7
8
9
10
11
12
13
14
15
16
17
18
19
20
21
22
23
24
25
26
27
28
29
30
31
32
33
34
35
36
37
38
39
day

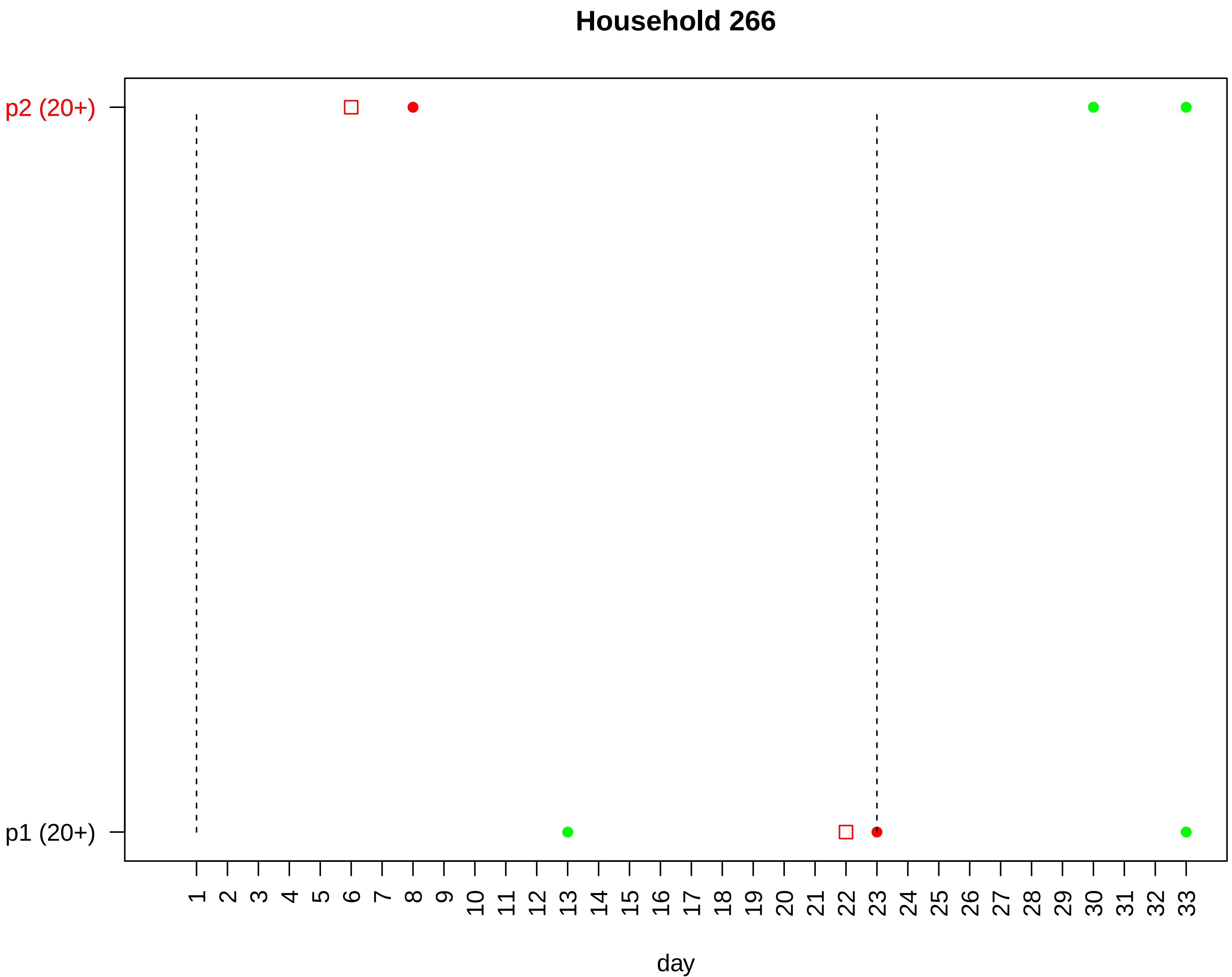

Household 266
p2 (20+)
p1 (20+)
1 2 3 4 5 6 7 8 9 10 11 12 13 14 15 16 17 18 19 20 21 22 23 24 25 26 27 28 29 30 31 32 33
day

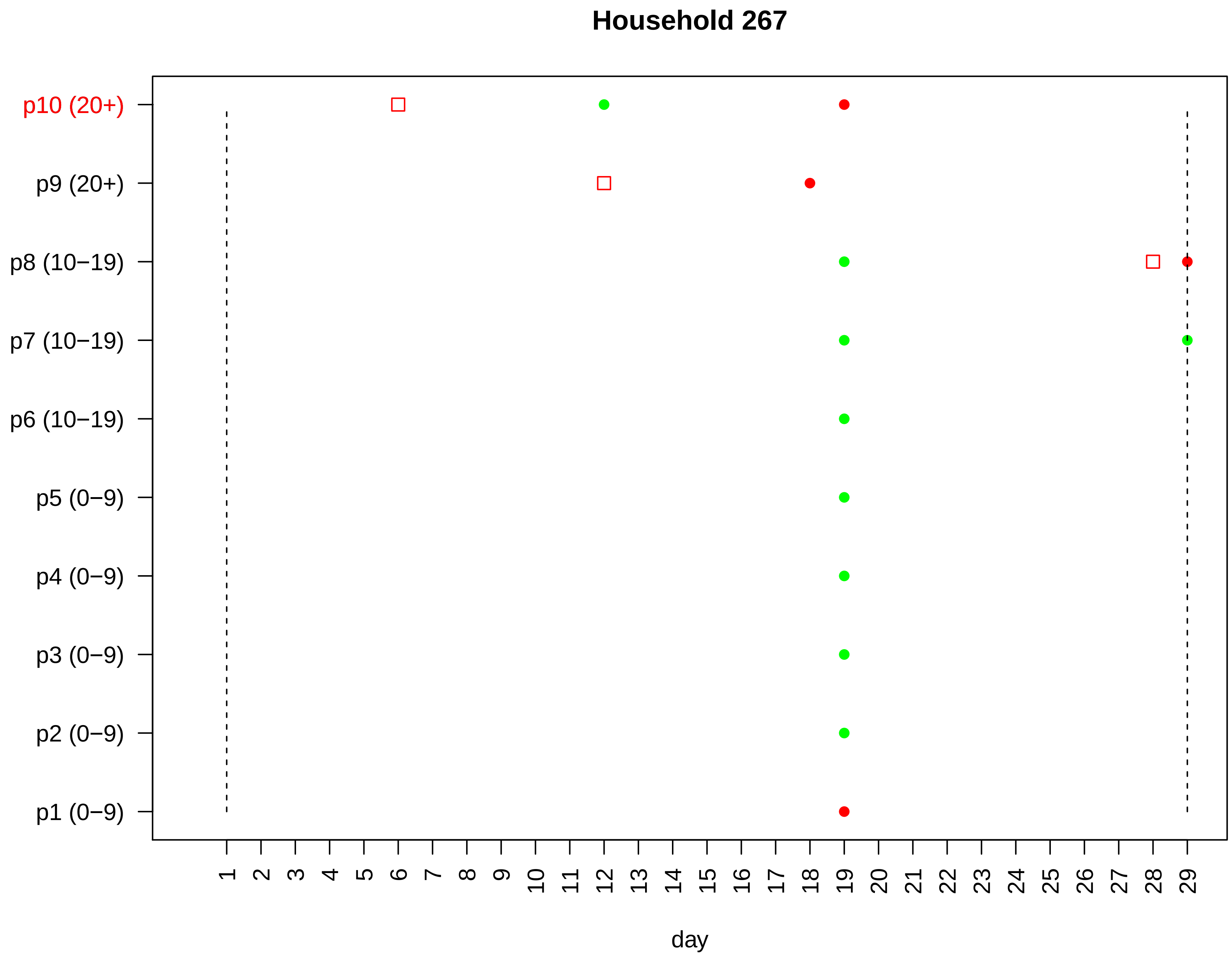
Household 267
p10 (20+)
p9 (20+)
p8 (10−19)
p7 (10−19)
p6 (10−19)
p5 (0−9)
p4 (0−9)
p3 (0−9)
p2 (0−9)
p1 (0−9)
1 2 3 4 5 6 7 8 9 10 11 12 13 14 15 16 17 18 19 20 21 22 23 24 25 26 27 28 29
day

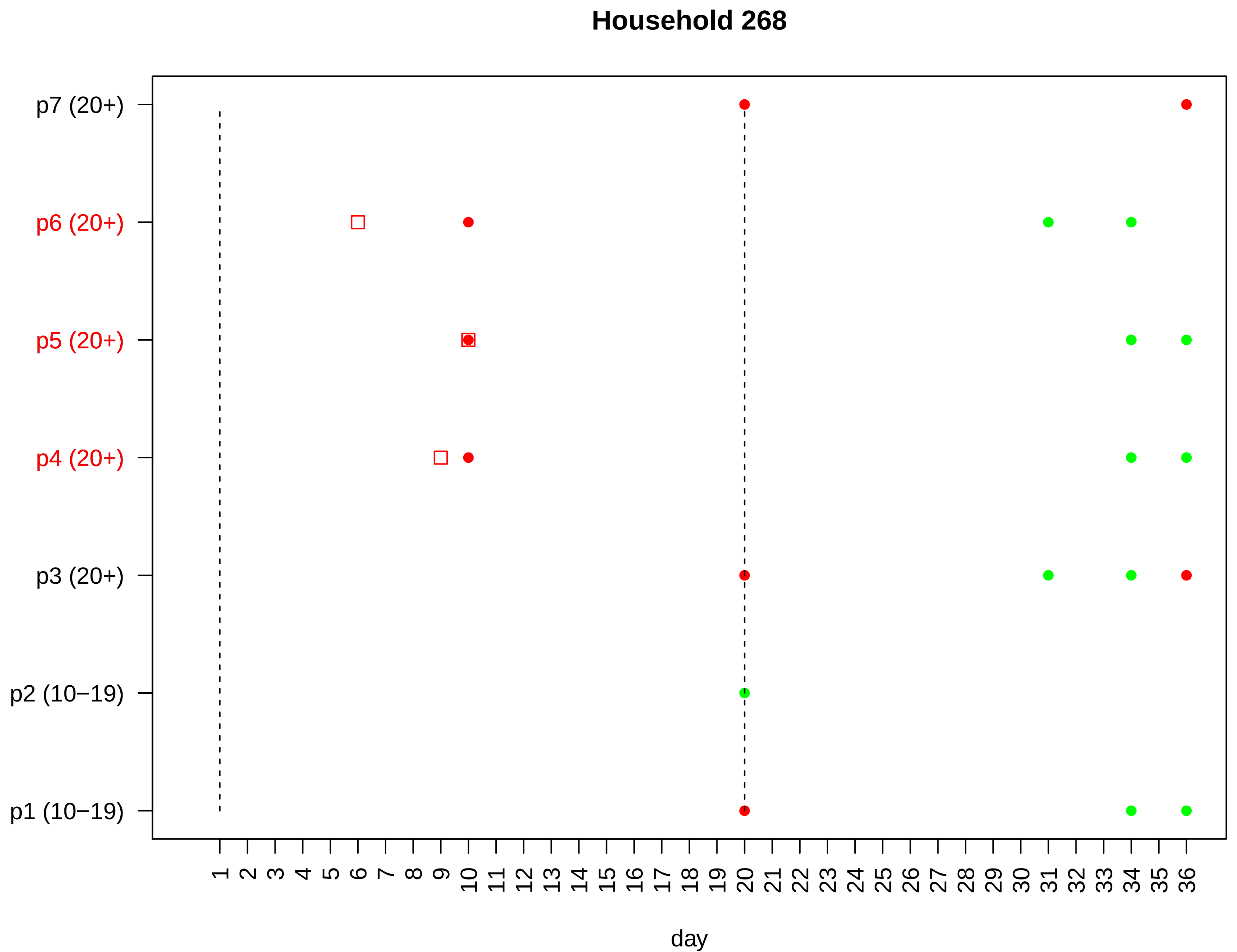

Household 268
p7 (20+)
p6 (20+)
p5 (20+)
p4 (20+)
p3 (20+)
p2 (10−19)
p1 (10−19)
1 2 3 4 5 6 7 8 9 10 11 12 13 14 15 16 17 18 19 20 21 22 23 24 25 26 27 28 29 30 31 32 33 34 35 36
day

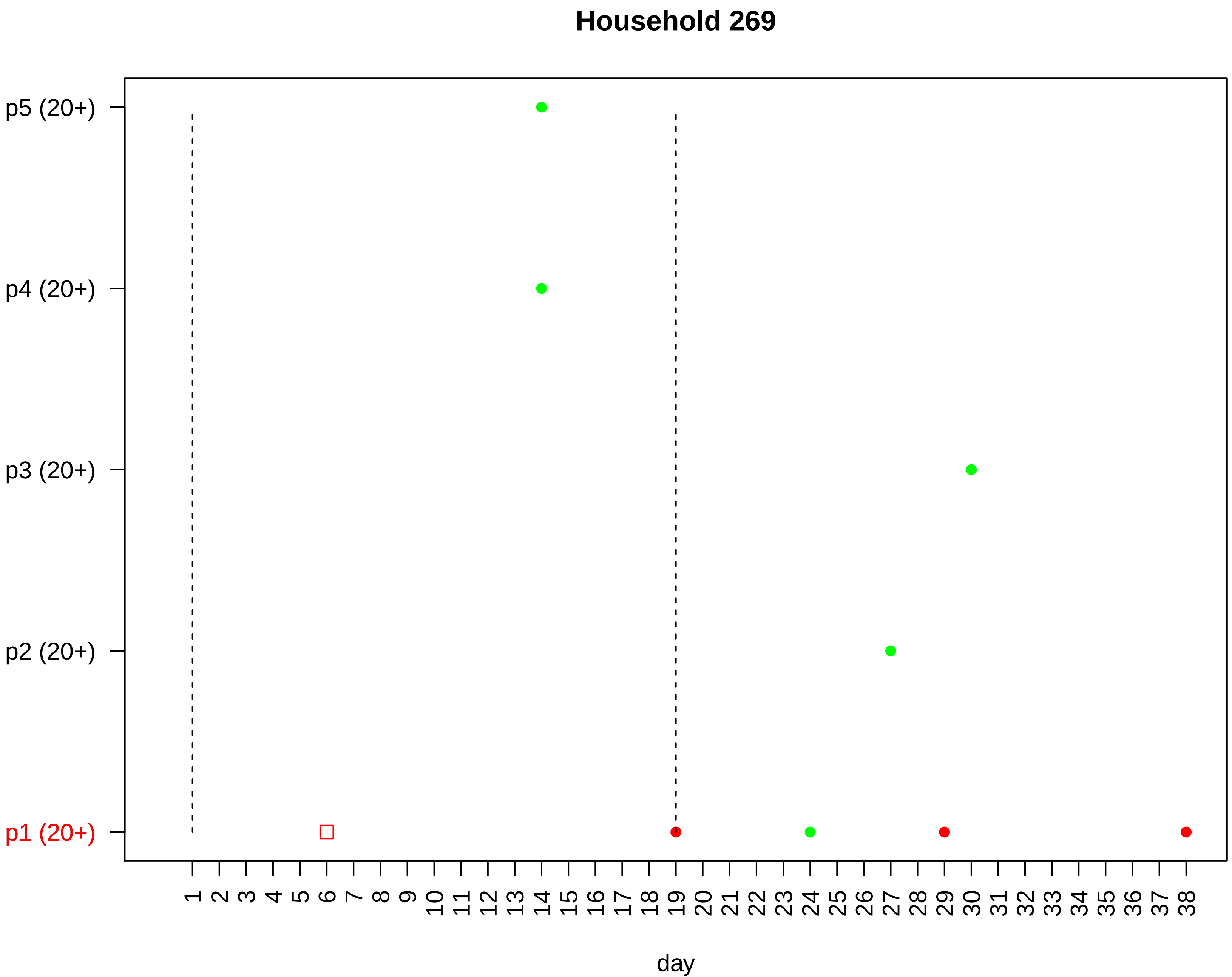
Household 269
p5 (20+)
p4 (20+)
p3 (20+)
p2 (20+)
p1 (20+)
1 2 3 4 5 6 7 8 9 10 11 12 13 14 15 16 17 18 19 20 21 22 23 24 25 26 27 28 29 30 31 32 33 34 35 36 37 38
day

Household 271

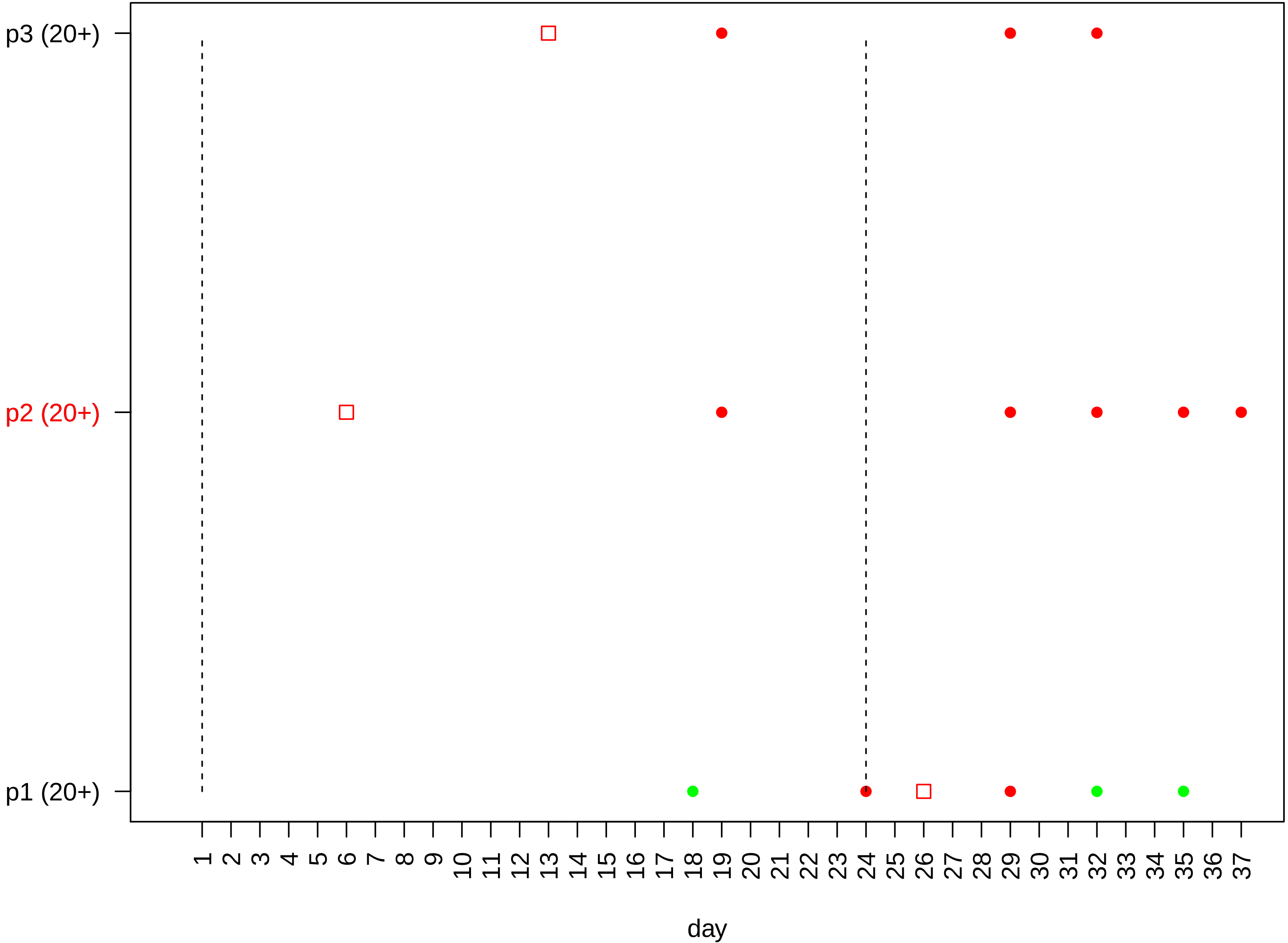

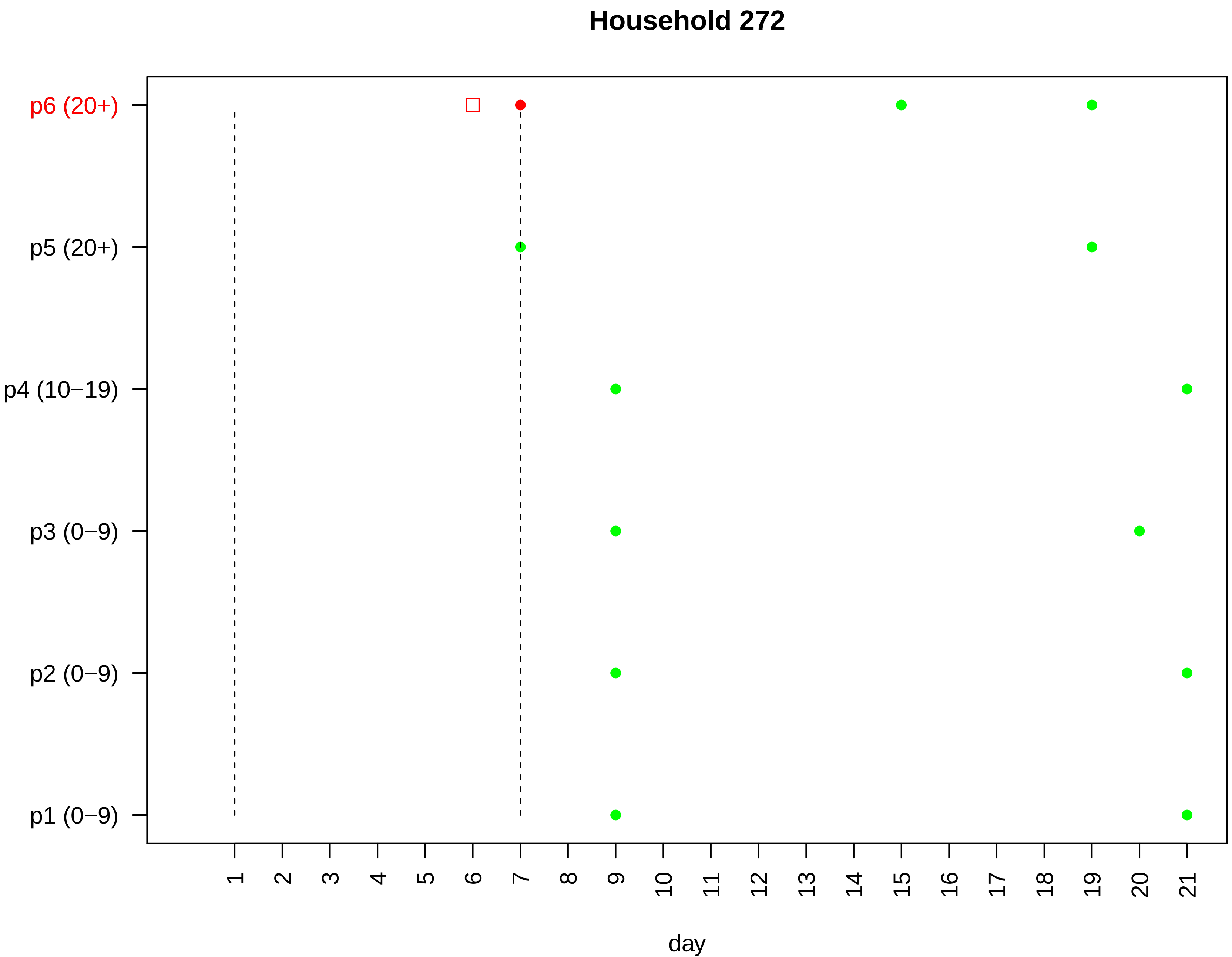
Household 272
p6 (20+)
p5 (20+)
p4 (10−19)
p3 (0−9)
p2 (0−9)
p1 (0−9)
1 2 3 4 5 6 7 8 9 10 11 12 13 14 15 16 17 18 19 20 21
day

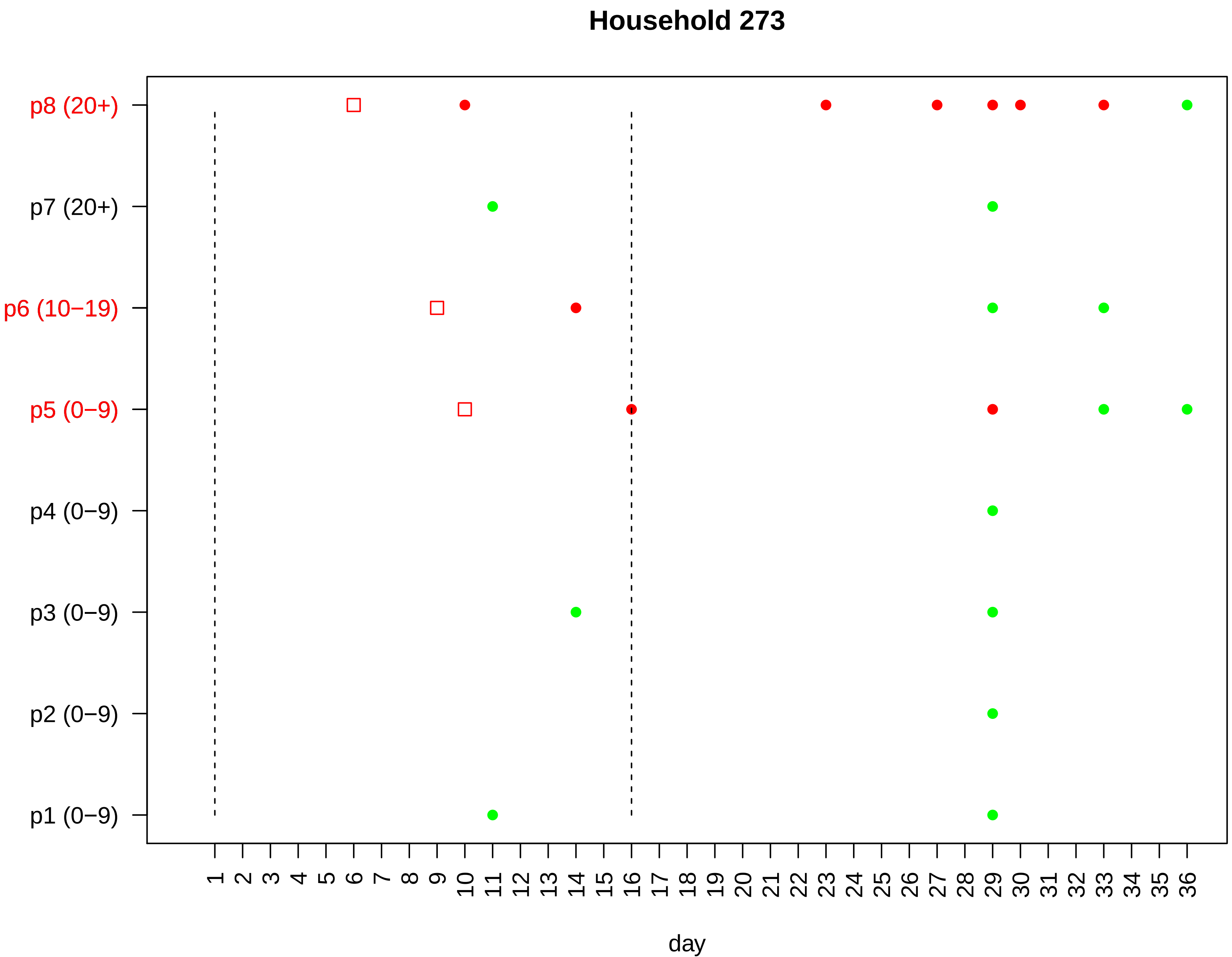

Household 273
p8 (20+)
p7 (20+)
p6 (10−19)
p5 (0−9)
p4 (0−9)
p3 (0−9)
p2 (0−9)
p1 (0−9)
1 2 3 4 5 6 7 8 9 10 11 12 13 14 15 16 17 18 19 20 21 22 23 24 25 26 27 28 29 30 31 32 33 34 35 36
day

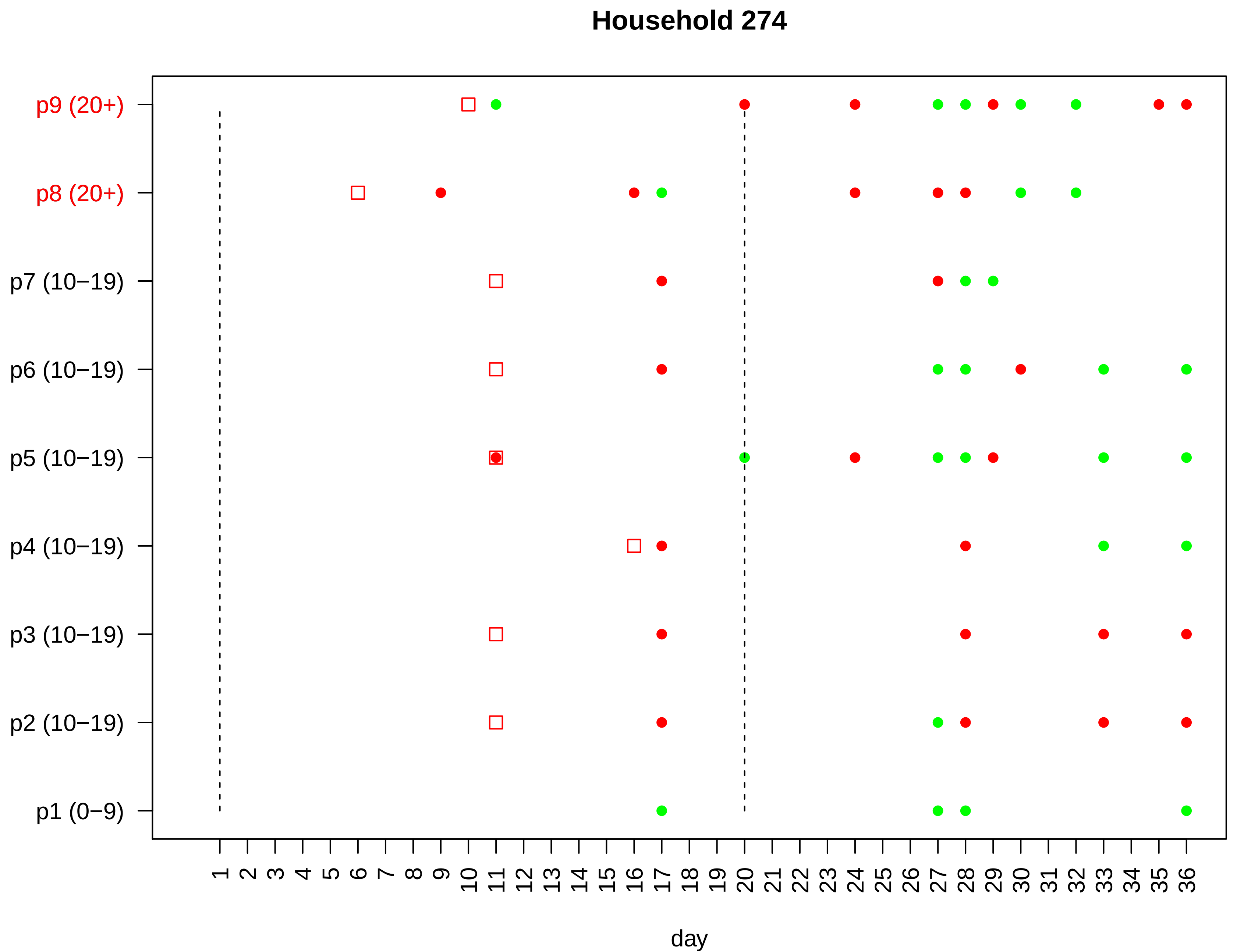

Household 274
p9 (20+)
p8 (20+)
p7 (10−19)
p6 (10−19)
p5 (10−19)
p4 (10−19)
p3 (10−19)
p2 (10−19)
p1 (0−9)
1 2 3 4 5 6 7 8 9 10 11 12 13 14 15 16 17 18 19 20 21 22 23 24 25 26 27 28 29 30 31 32 33 34 35 36
day

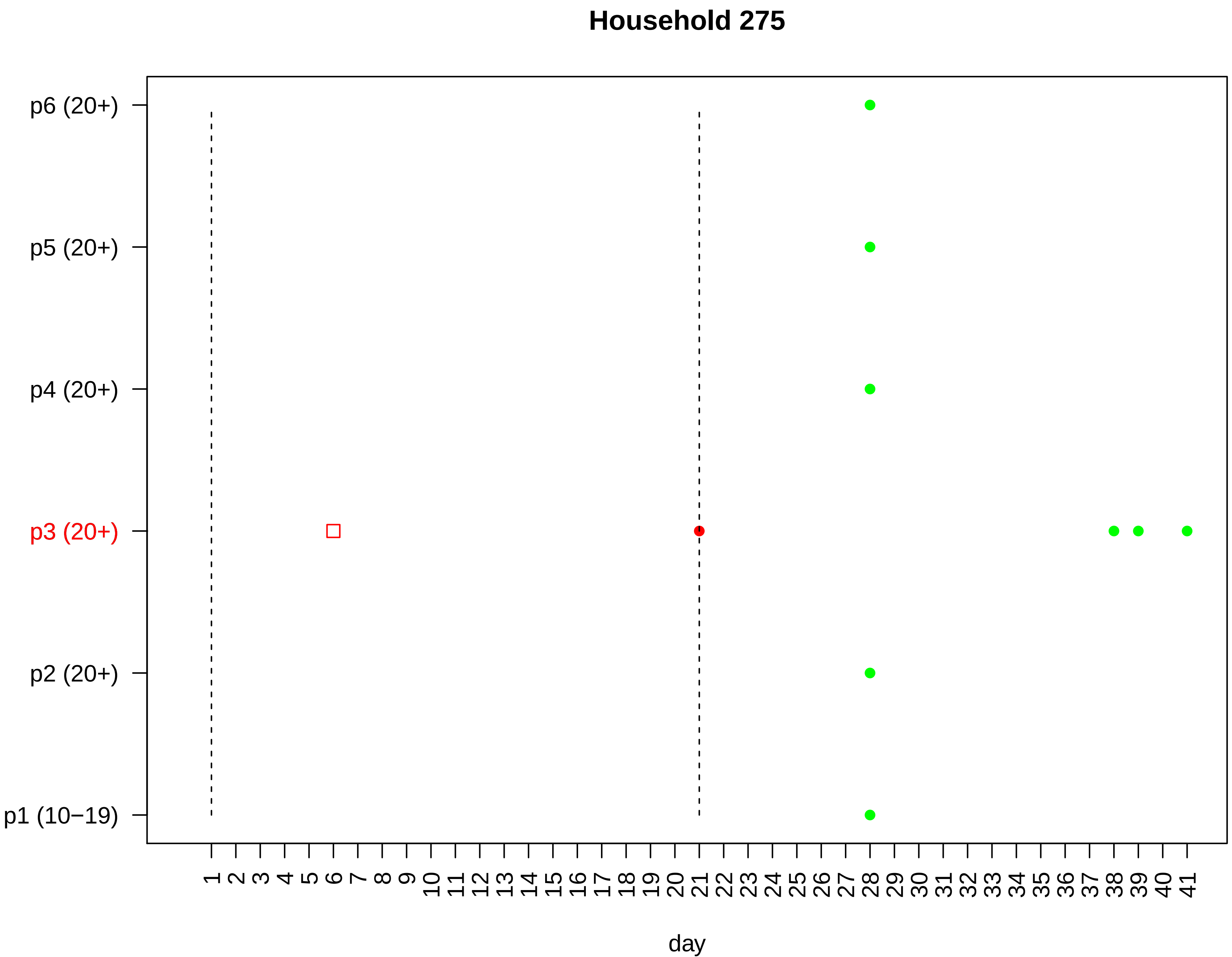
Household 275
p6 (20+)
p5 (20+)
p4 (20+)
p3 (20+)
p2 (20+)
p1 (10−19)
1 2 3 4 5 6 7 8 9 10 11 12 13 14 15 16 17 18 19 20 21 22 23 24 25 26 27 28 29 30 31 32 33 34 35 36 37 38 39 40 41
day

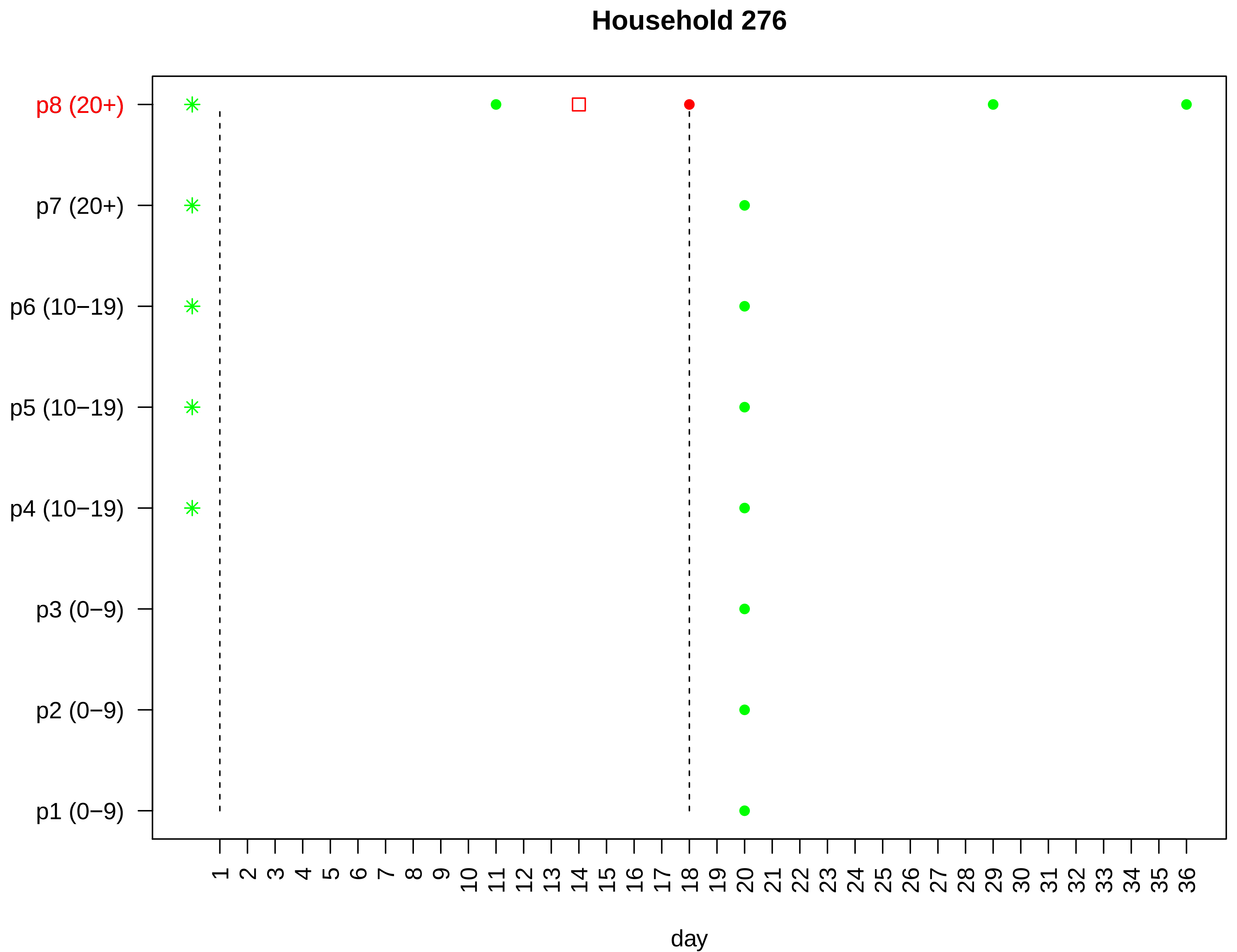

Household 276
p8 (20+)
p7 (20+)
p6 (10−19)
p5 (10−19)
p4 (10−19)
p3 (0−9)
p2 (0−9)
p1 (0−9)
1 2 3 4 5 6 7 8 9 10 11 12 13 14 15 16 17 18 19 20 21 22 23 24 25 26 27 28 29 30 31 32 33 34 35 36
day

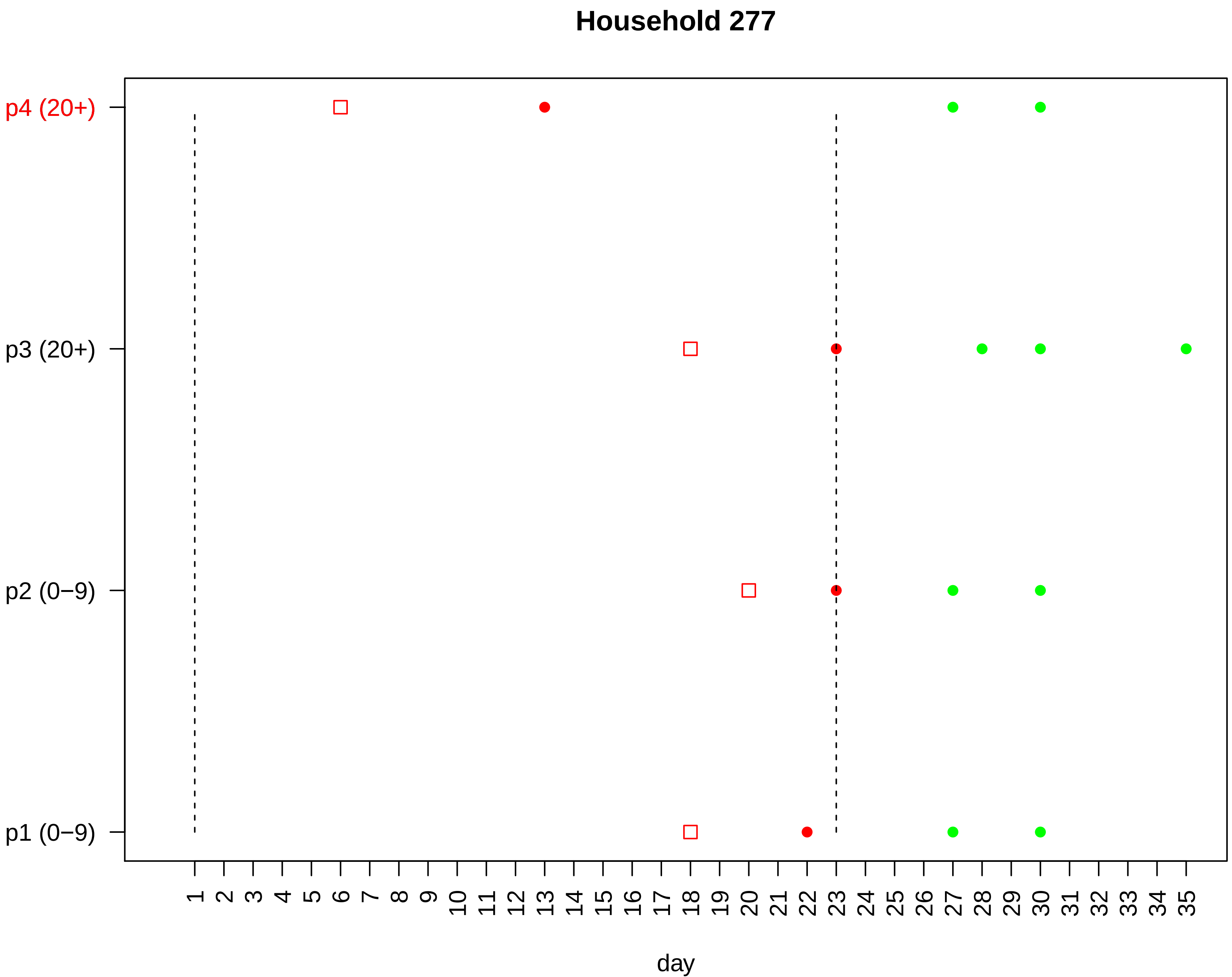

Household 277
p4 (20+)
p3 (20+)
p2 (0−9)
p1 (0−9)
1 2 3 4 5 6 7 8 9 10 11 12 13 14 15 16 17 18 19 20 21 22 23 24 25 26 27 28 29 30 31 32 33 34 35
day

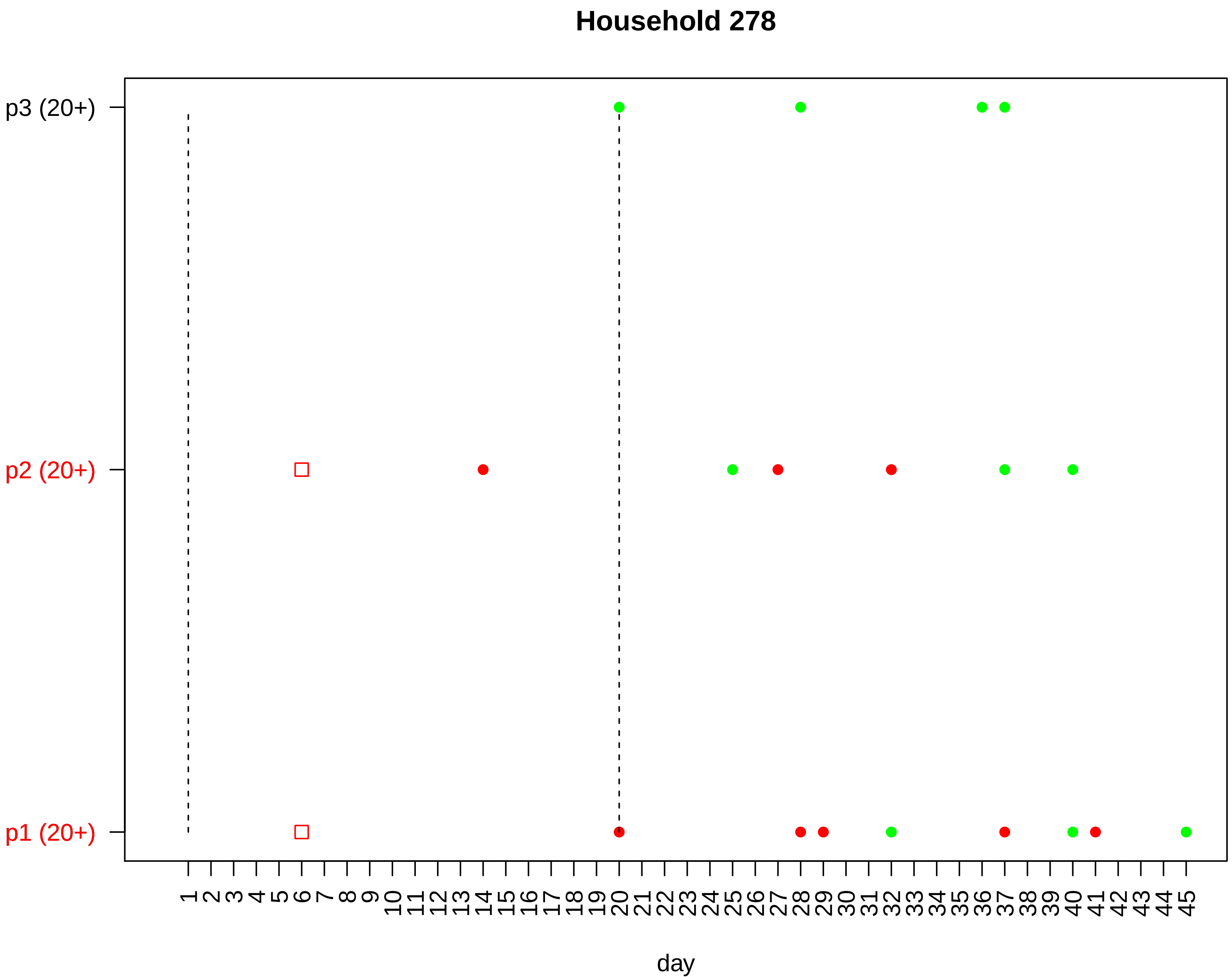

Household 278
p3 (20+)
p2 (20+)
p1 (20+)
1 2 3 4 5 6 7 8 9 10 11 12 13 14 15 16 17 18 19 20 21 22 23 24 25 26 27 28 29 30 31 32 33 34 35 36 37 38 39 40 41 42 43 44 45
day

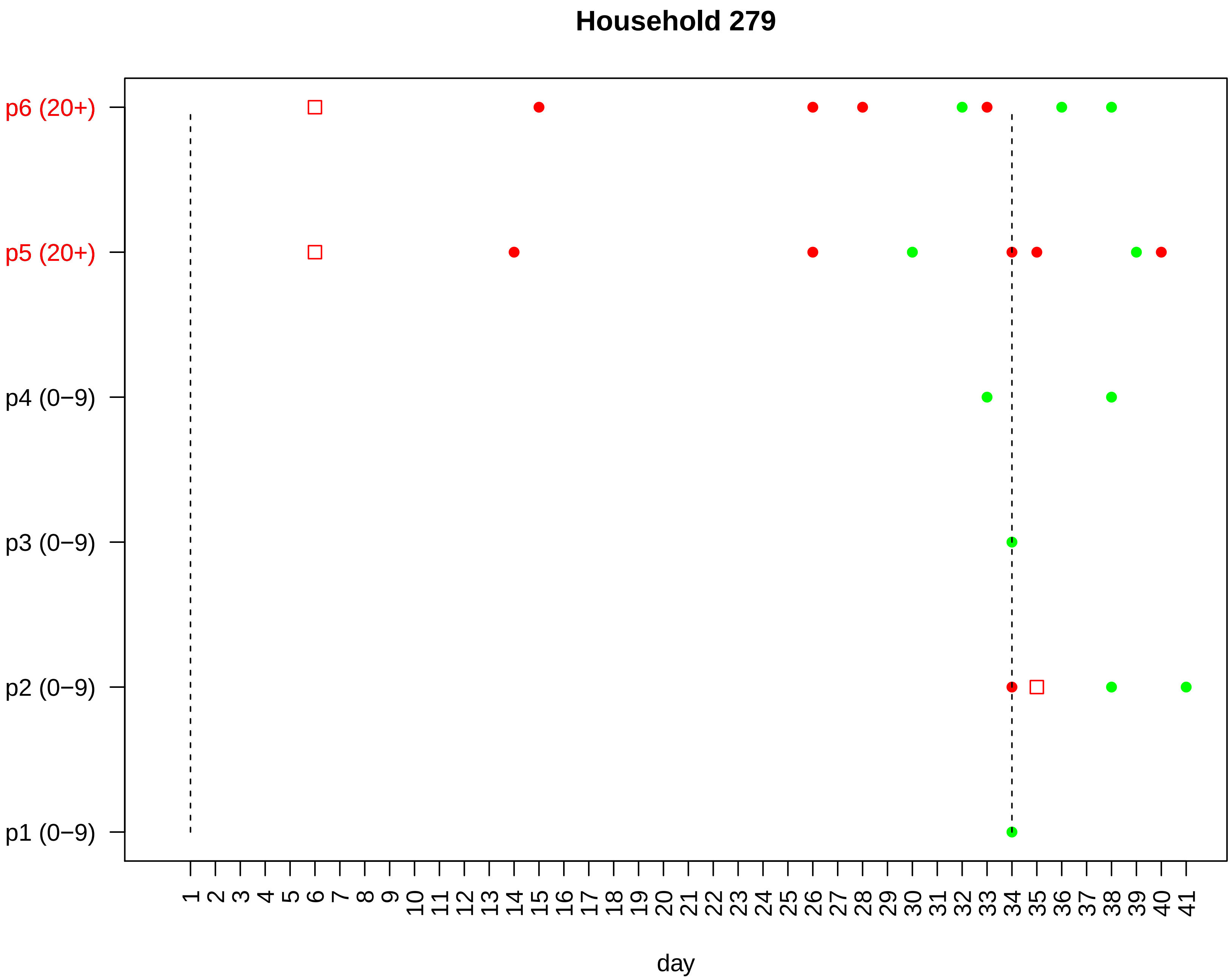

Household 279
p6 (20+)
p5 (20+)
p4 (0−9)
p3 (0−9)
p2 (0−9)
p1 (0−9)
1 2 3 4 5 6 7 8 9 10 11 12 13 14 15 16 17 18 19 20 21 22 23 24 25 26 27 28 29 30 31 32 33 34 35 36 37 38 39 40 41
day

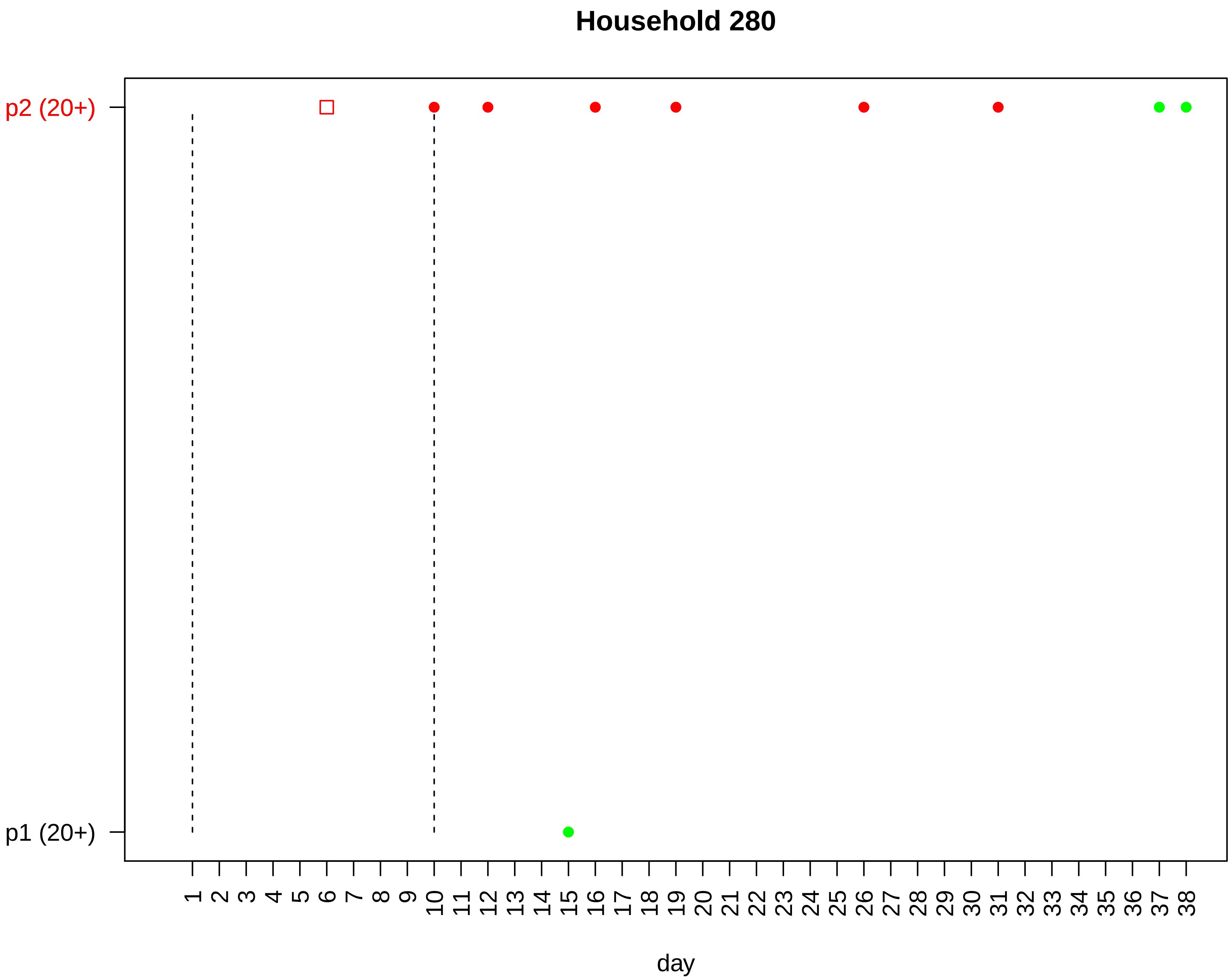

Household 280
p2 (20+)
p1 (20+)
1 2 3 4 5 6 7 8 9 10 11 12 13 14 15 16 17 18 19 20 21 22 23 24 25 26 27 28 29 30 31 32 33 34 35 36 37 38
day

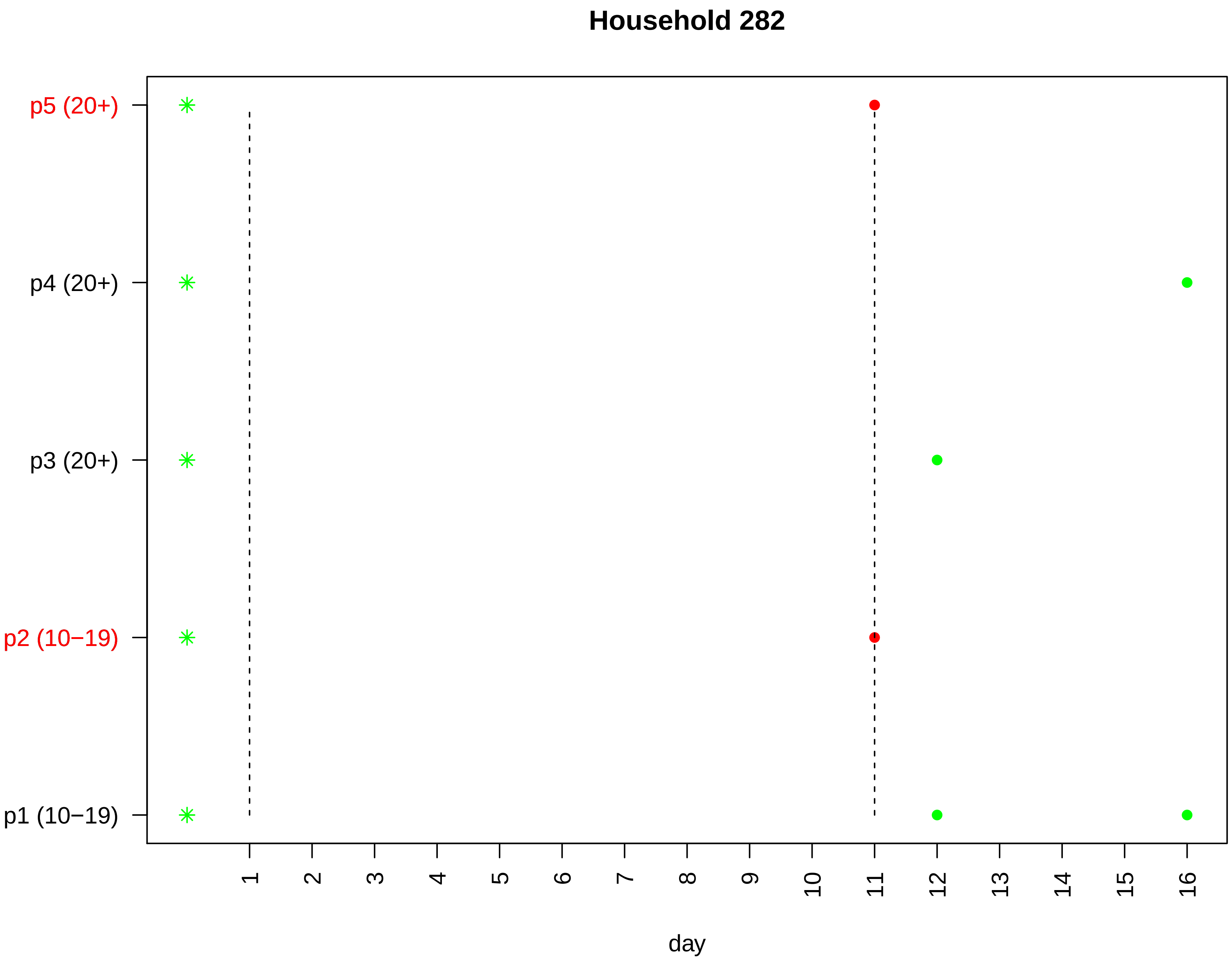

Household 282
p5 (20+)
p4 (20+)
p3 (20+)
p2 (10−19)
p1 (10−19)
1
2
3
4
5
6
7
8
9
10
11
12
13
14
15
16
day

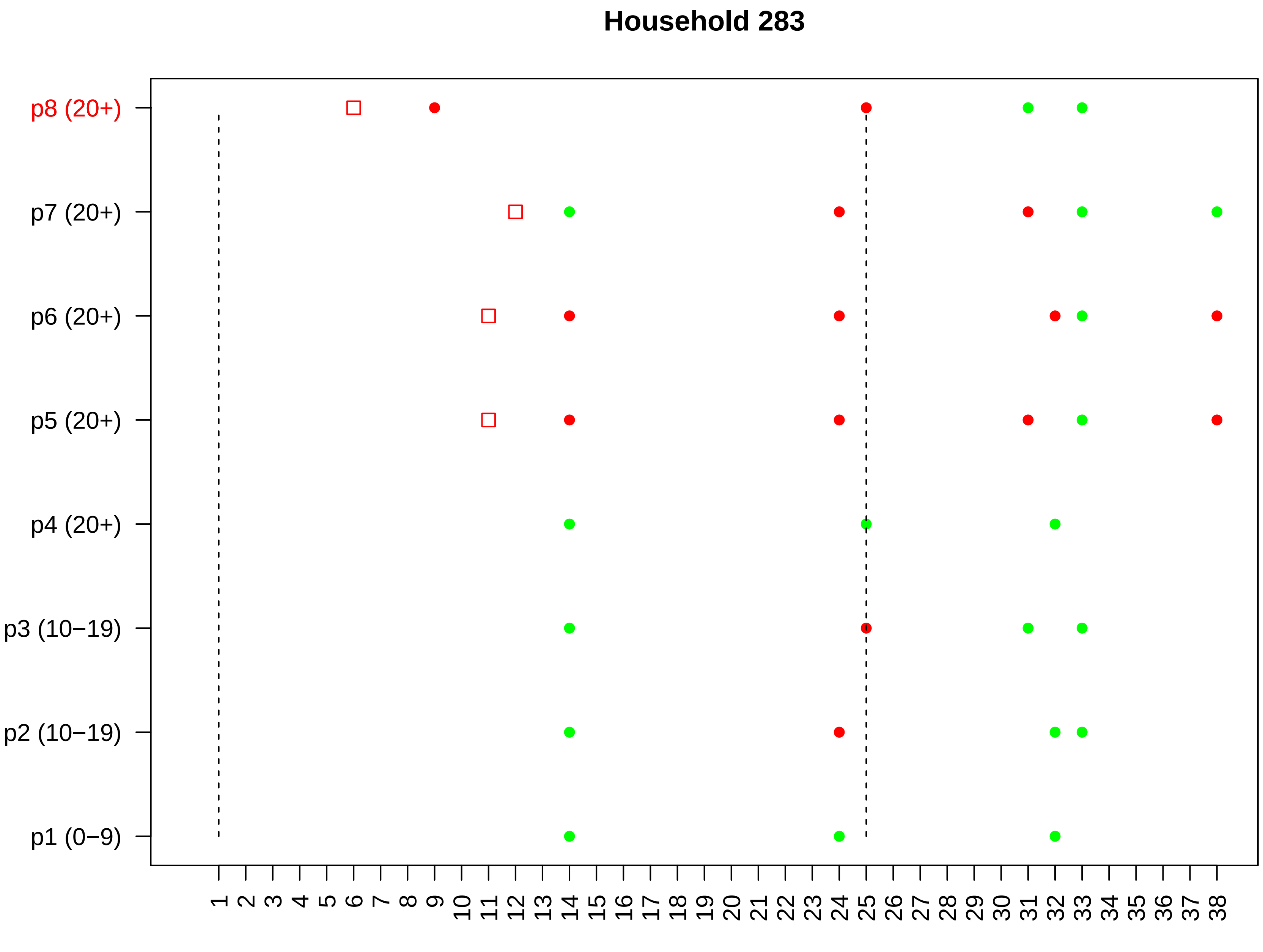

Household 283
p8 (20+)
p7 (20+)
p6 (20+)
p5 (20+)
p4 (20+)
p3 (10−19)
p2 (10−19)
p1 (0−9)
1 2 3 4 5 6 7 8 9 10 11 12 13 14 15 16 17 18 19 20 21 22 23 24 25 26 27 28 29 30 31 32 33 34 35 36 37 38
day

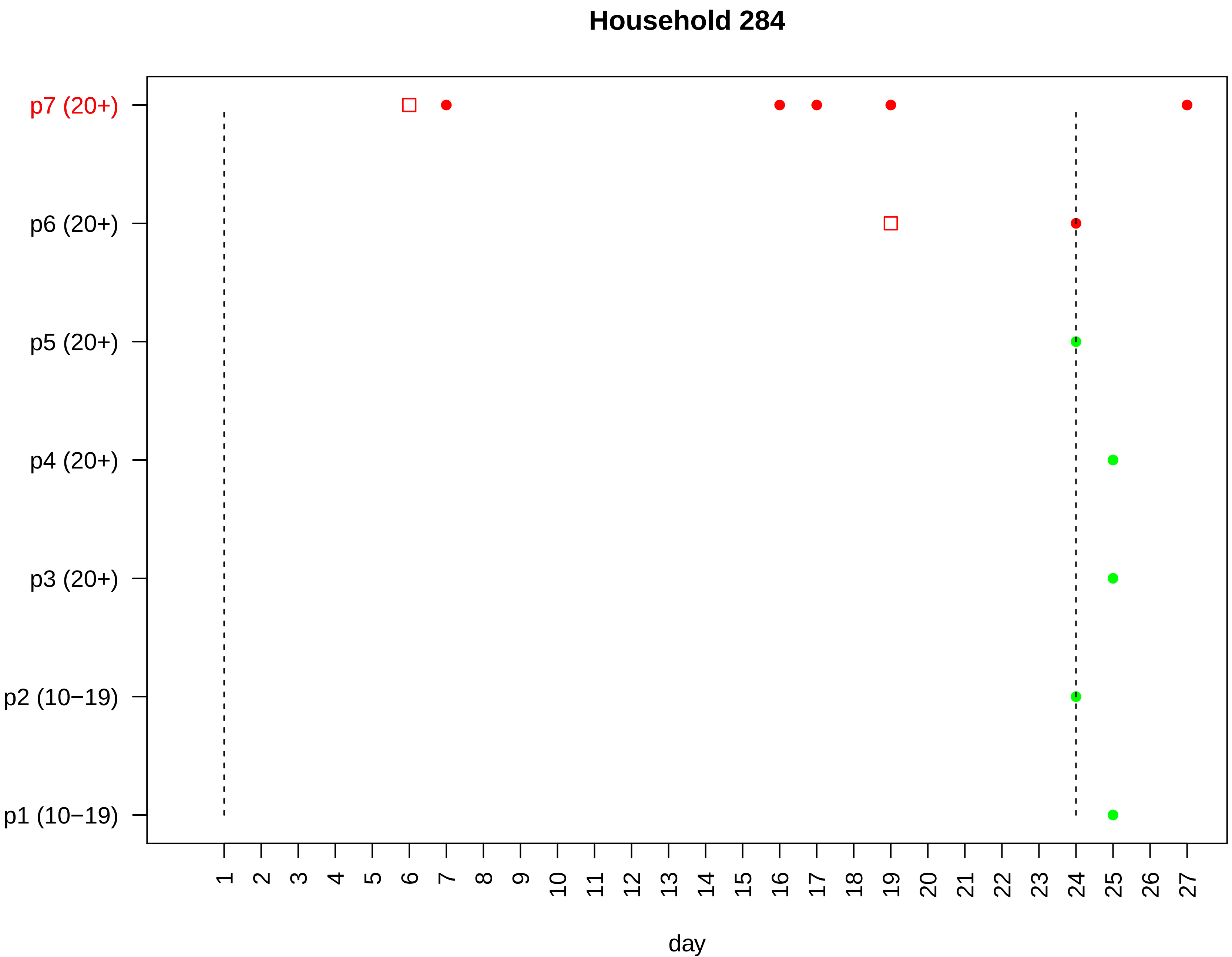
Household 284
p7 (20+)
p6 (20+)
p5 (20+)
p4 (20+)
p3 (20+)
p2 (10−19)
p1 (10−19)
1 2 3 4 5 6 7 8 9 10 11 12 13 14 15 16 17 18 19 20 21 22 23 24 25 26 27
day

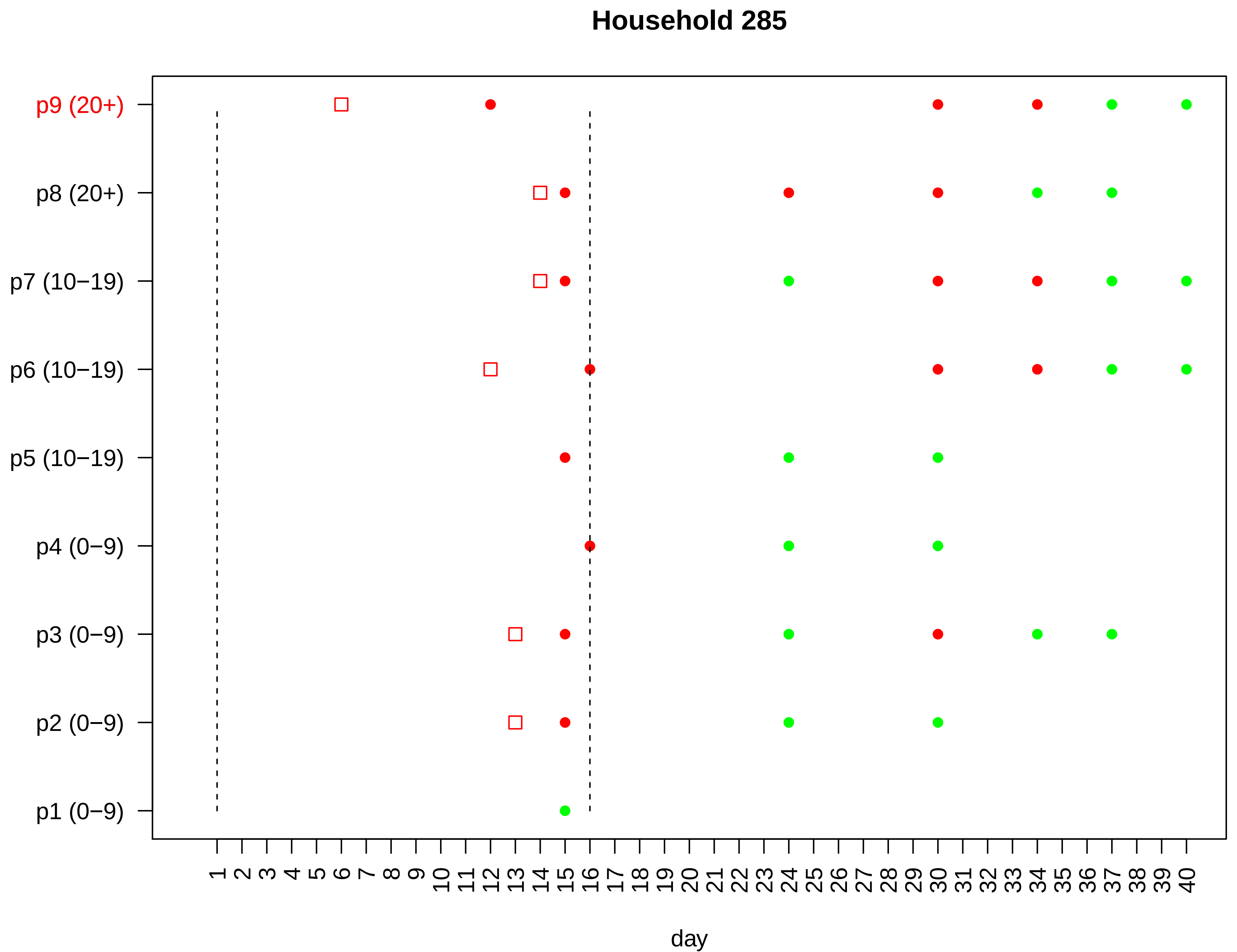

Household 285
p9 (20+)
p8 (20+)
p7 (10−19)
p6 (10−19)
p5 (10−19)
p4 (0−9)
p3 (0−9)
p2 (0−9)
p1 (0−9)
1 2 3 4 5 6 7 8 9 10 11 12 13 14 15 16 17 18 19 20 21 22 23 24 25 26 27 28 29 30 31 32 33 34 35 36 37 38 39 40
day

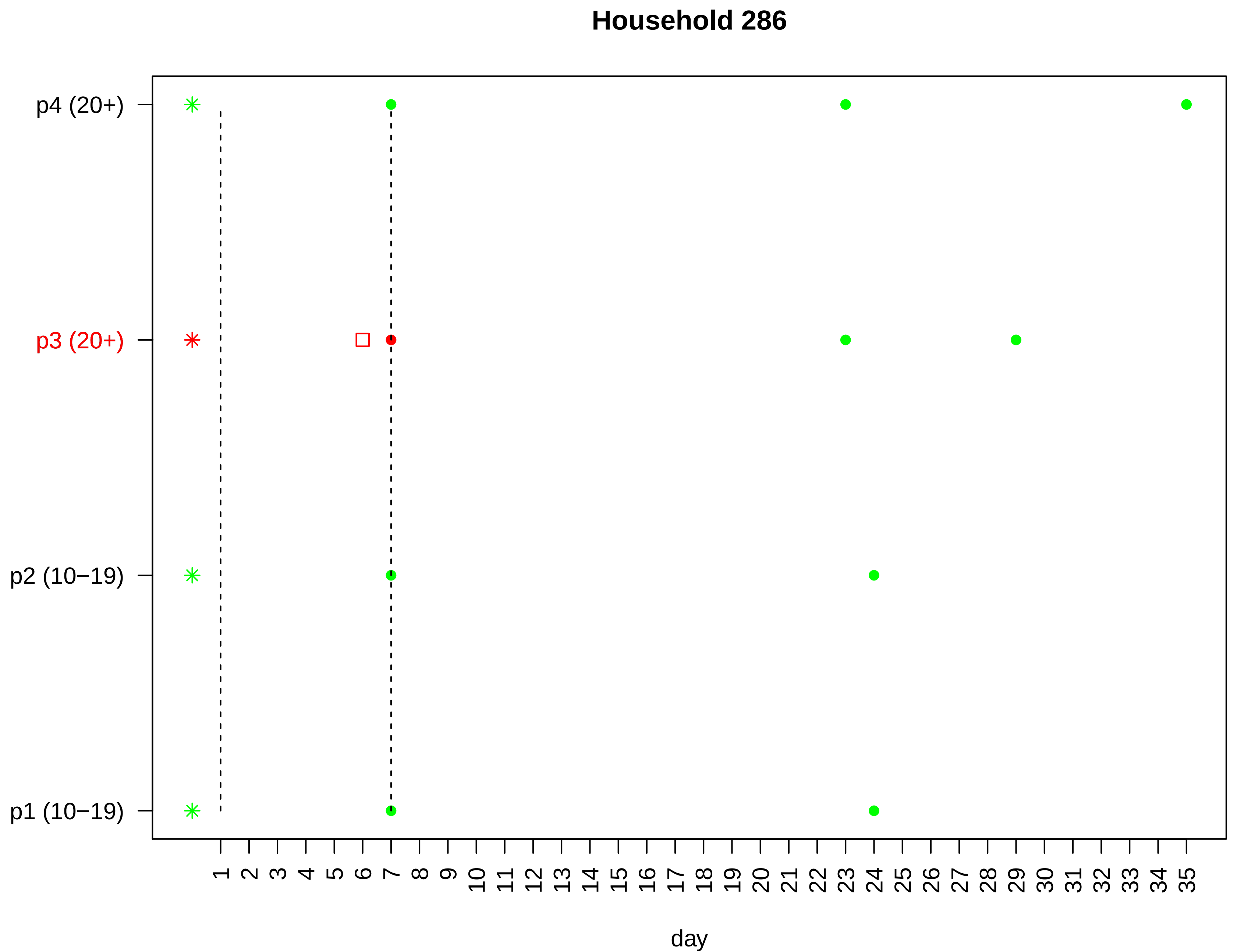

Household 286
p4 (20+)
p3 (20+)
p2 (10−19)
p1 (10−19)
1
2
3
4
5
6
7
8
9
10
11
12
13
14
15
16
17
18
19
20
21
22
23
24
25
26
27
28
29
30
31
32
33
34
35
day

Household 287

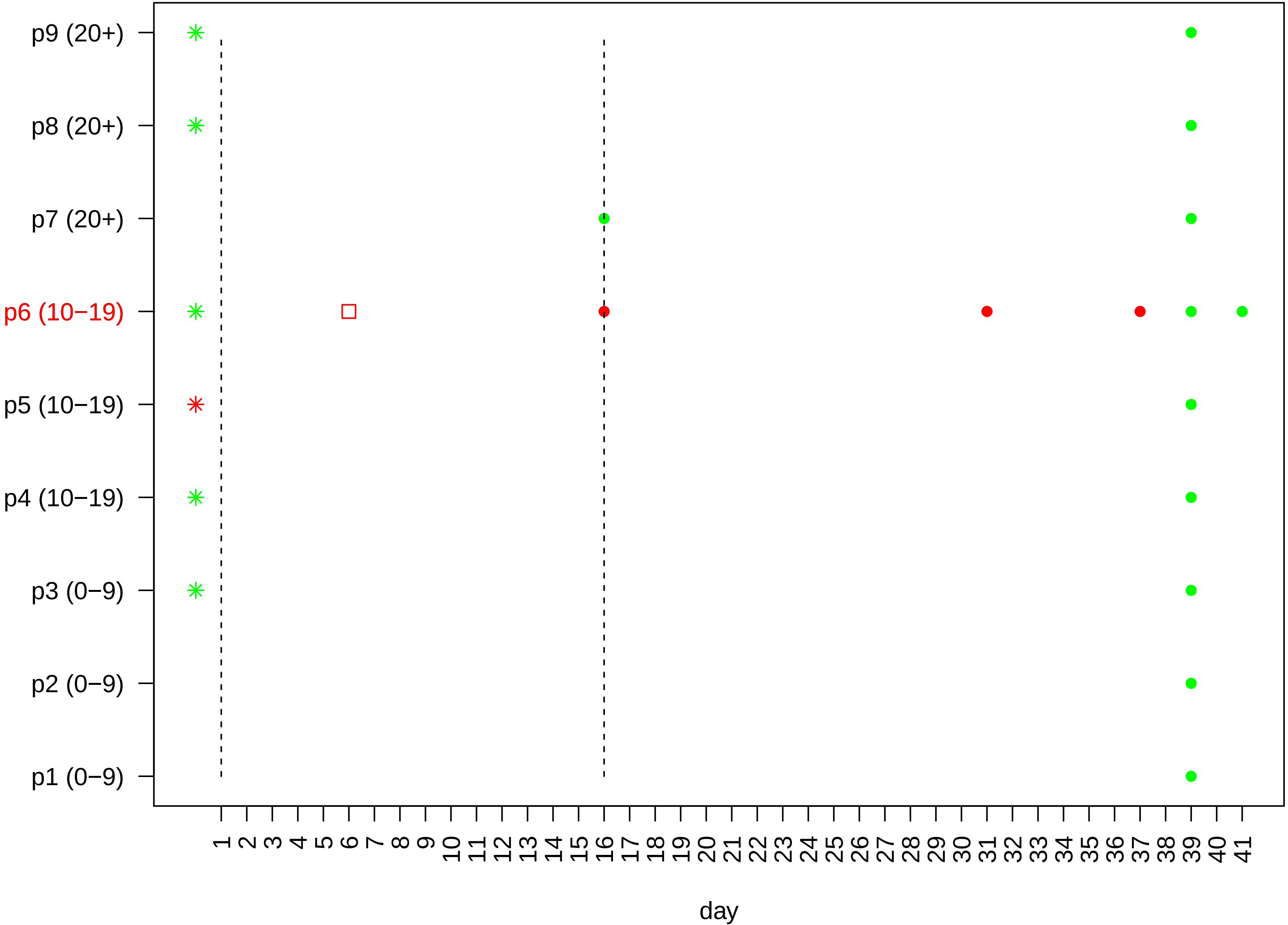

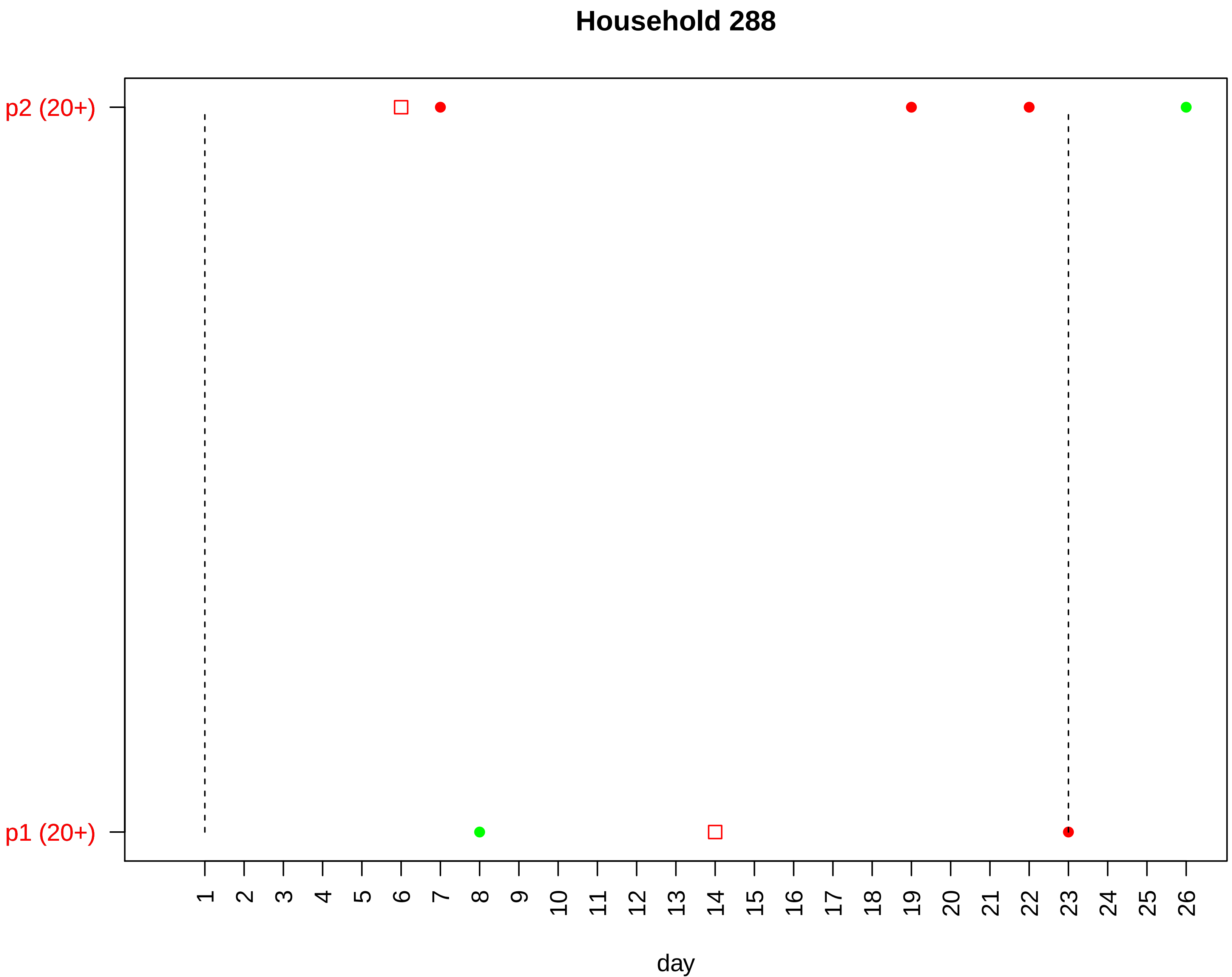

Household 288
p2 (20+)
p1 (20+)
1
2
3
4
5
6
7
8
9
10
11
12
13
14
15
16
17
18
19
20
21
22
23
24
25
26
day

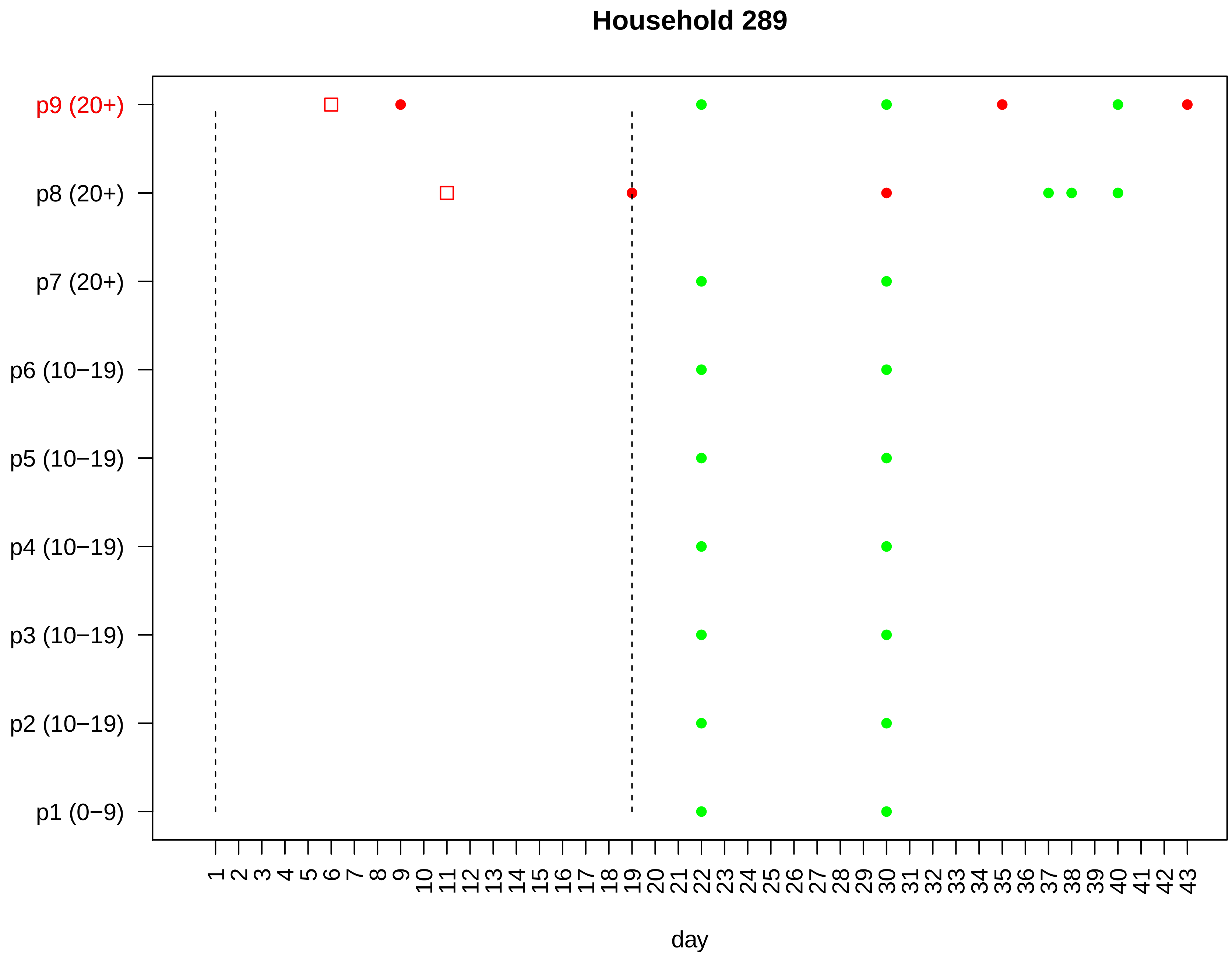

Household 289
p9 (20+)
p8 (20+)
p7 (20+)
p6 (10−19)
p5 (10−19)
p4 (10−19)
p3 (10−19)
p2 (10−19)
p1 (0−9)
1 2 3 4 5 6 7 8 9 10 11 12 13 14 15 16 17 18 19 20 21 22 23 24 25 26 27 28 29 30 31 32 33 34 35 36 37 38 39 40 41 42 43
day

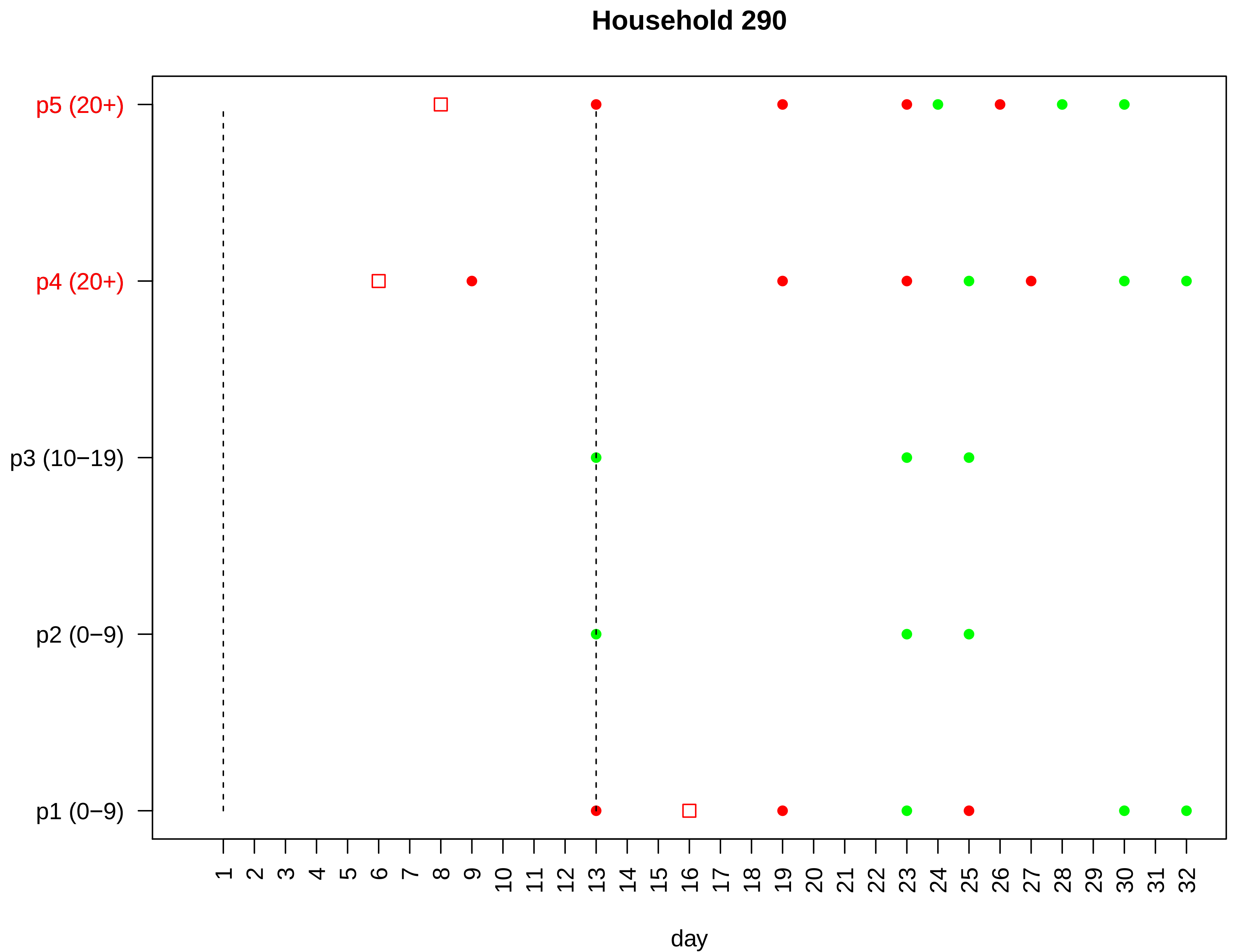
Household 290
p5 (20+)
p4 (20+)
p3 (10−19)
p2 (0−9)
p1 (0−9)
1 2 3 4 5 6 7 8 9 10 11 12 13 14 15 16 17 18 19 20 21 22 23 24 25 26 27 28 29 30 31 32
day

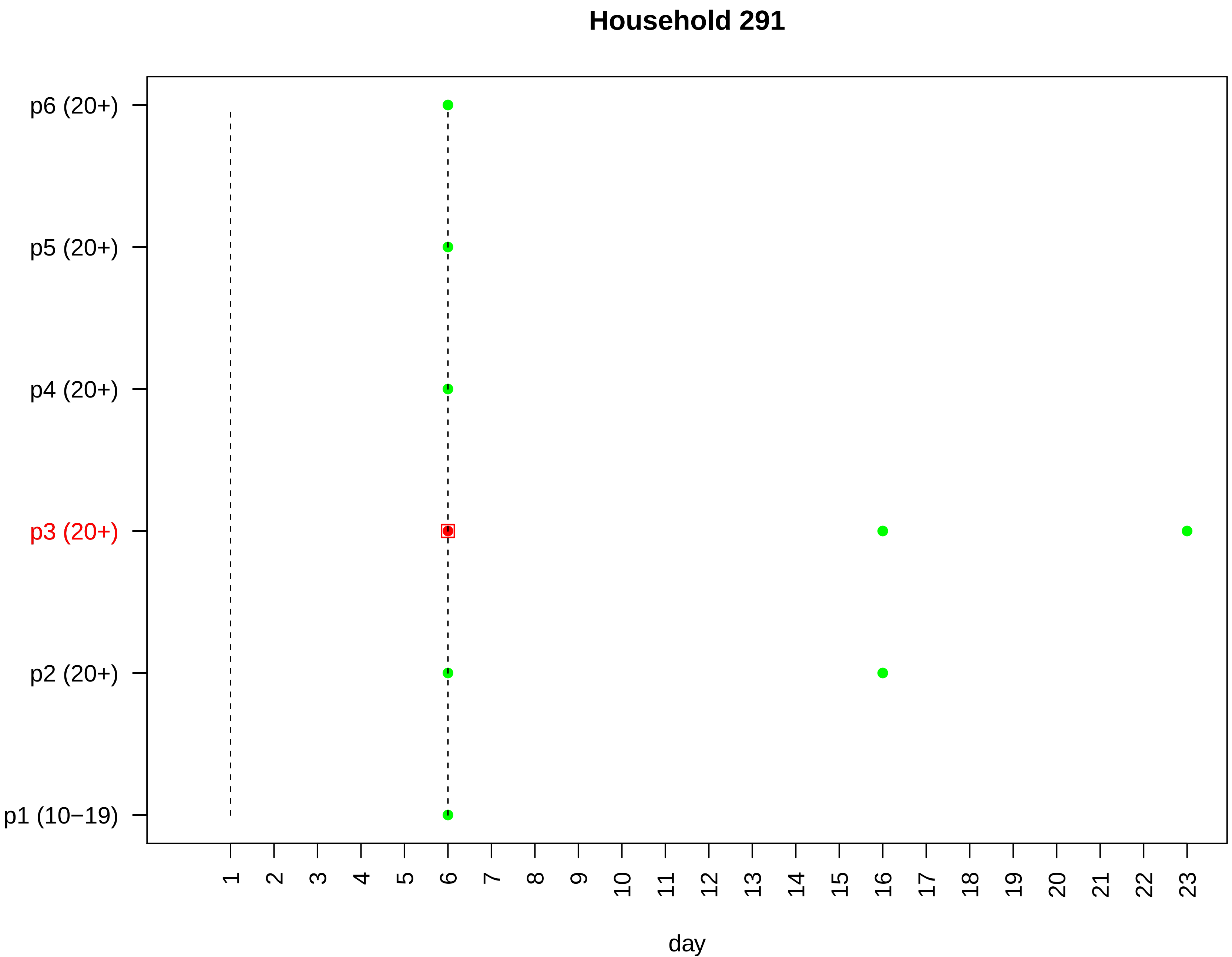
Household 291
p6 (20+)
p5 (20+)
p4 (20+)
p3 (20+)
p2 (20+)
p1 (10−19)
1
2
3
4
5
6
7
8
9
10
11
12
13
14
15
16
17
18
19
20
21
22
23
day

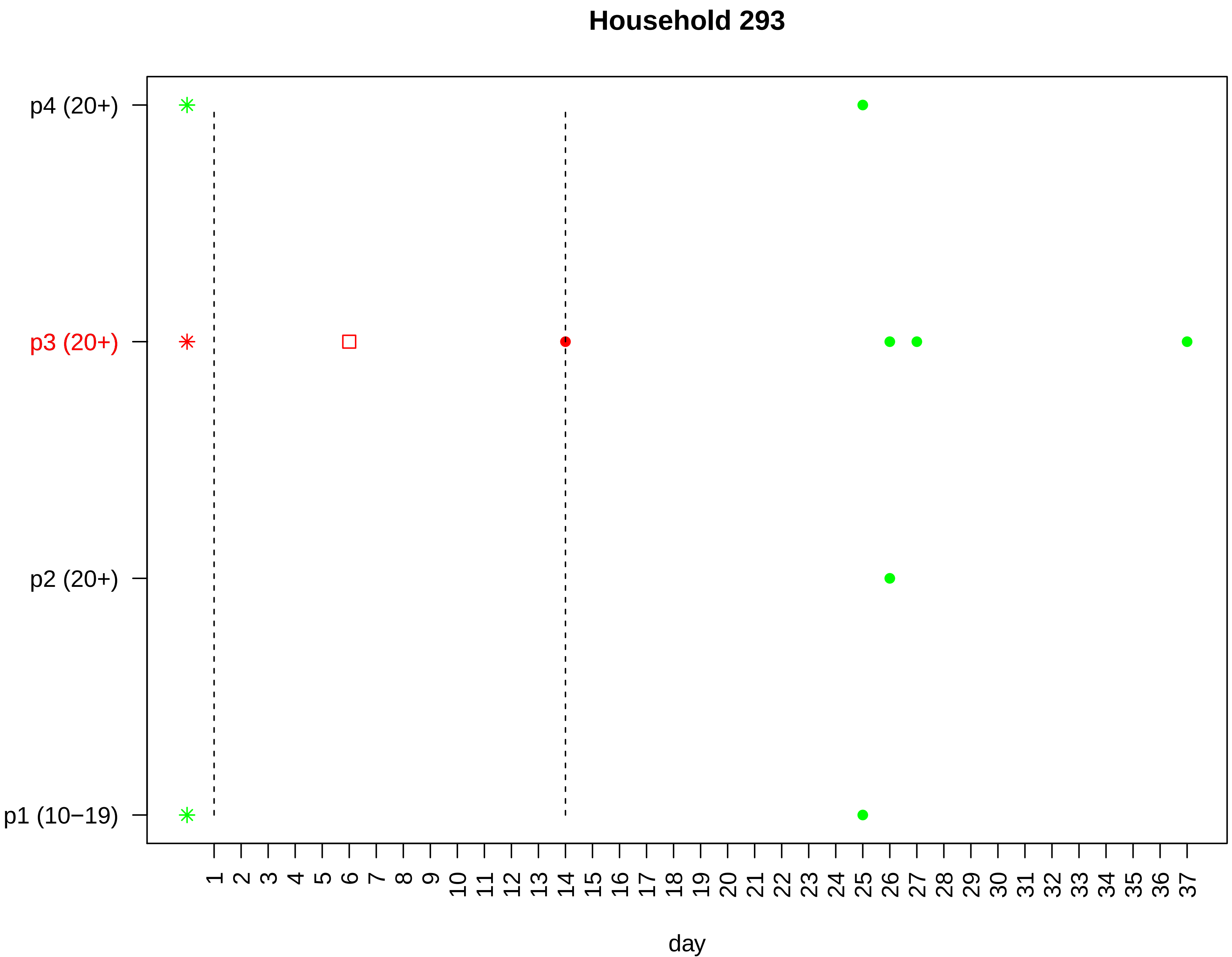

Household 293
p4 (20+)
p3 (20+)
p2 (20+)
p1 (10−19)
1 2 3 4 5 6 7 8 9 10 11 12 13 14 15 16 17 18 19 20 21 22 23 24 25 26 27 28 29 30 31 32 33 34 35 36 37
day

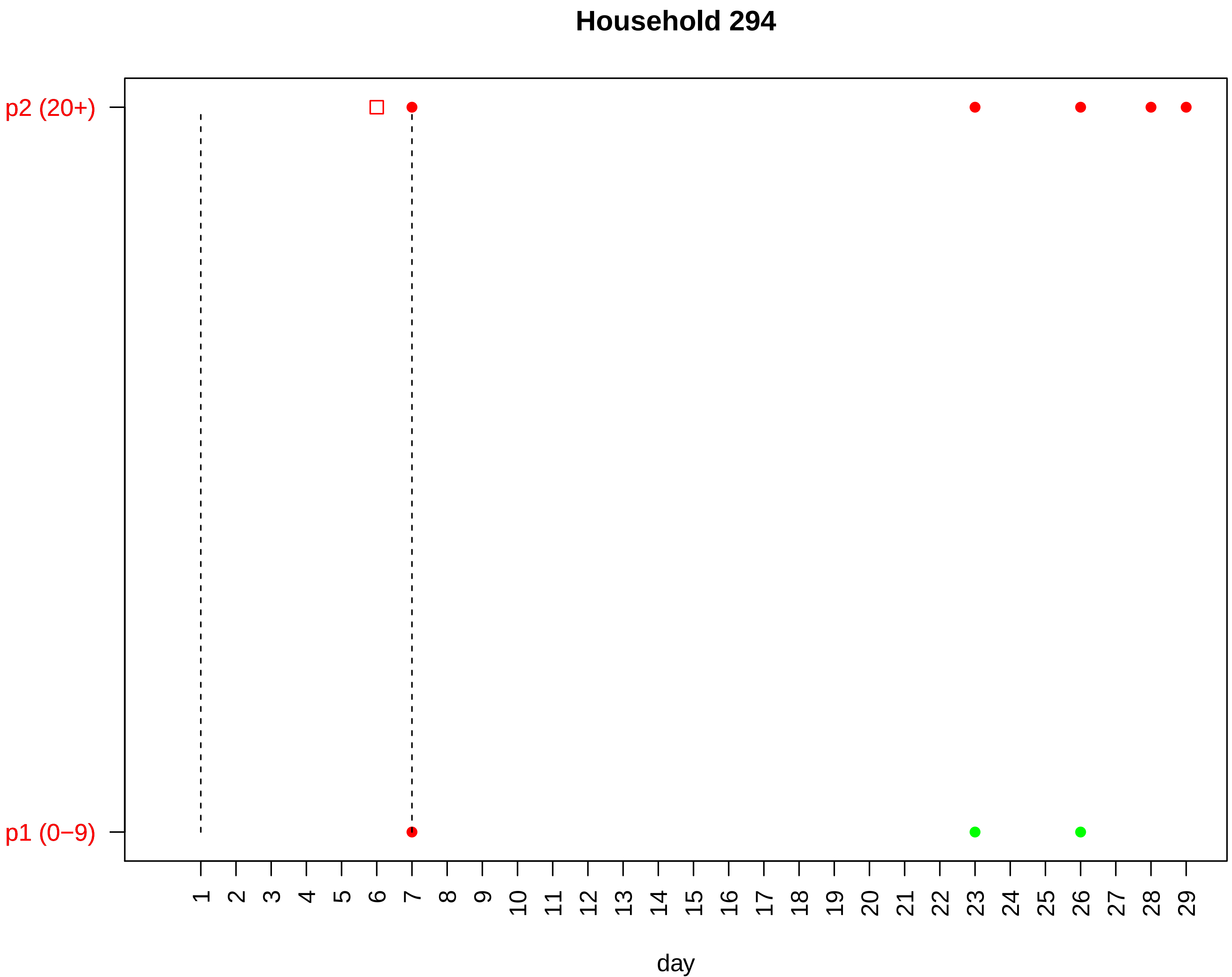

Household 294
p2 (20+)
p1 (0−9)
1
2
3
4
5
6
7
8
9
10
11
12
13
14
15
16
17
18
19
20
21
22
23
24
25
26
27
28
29
day

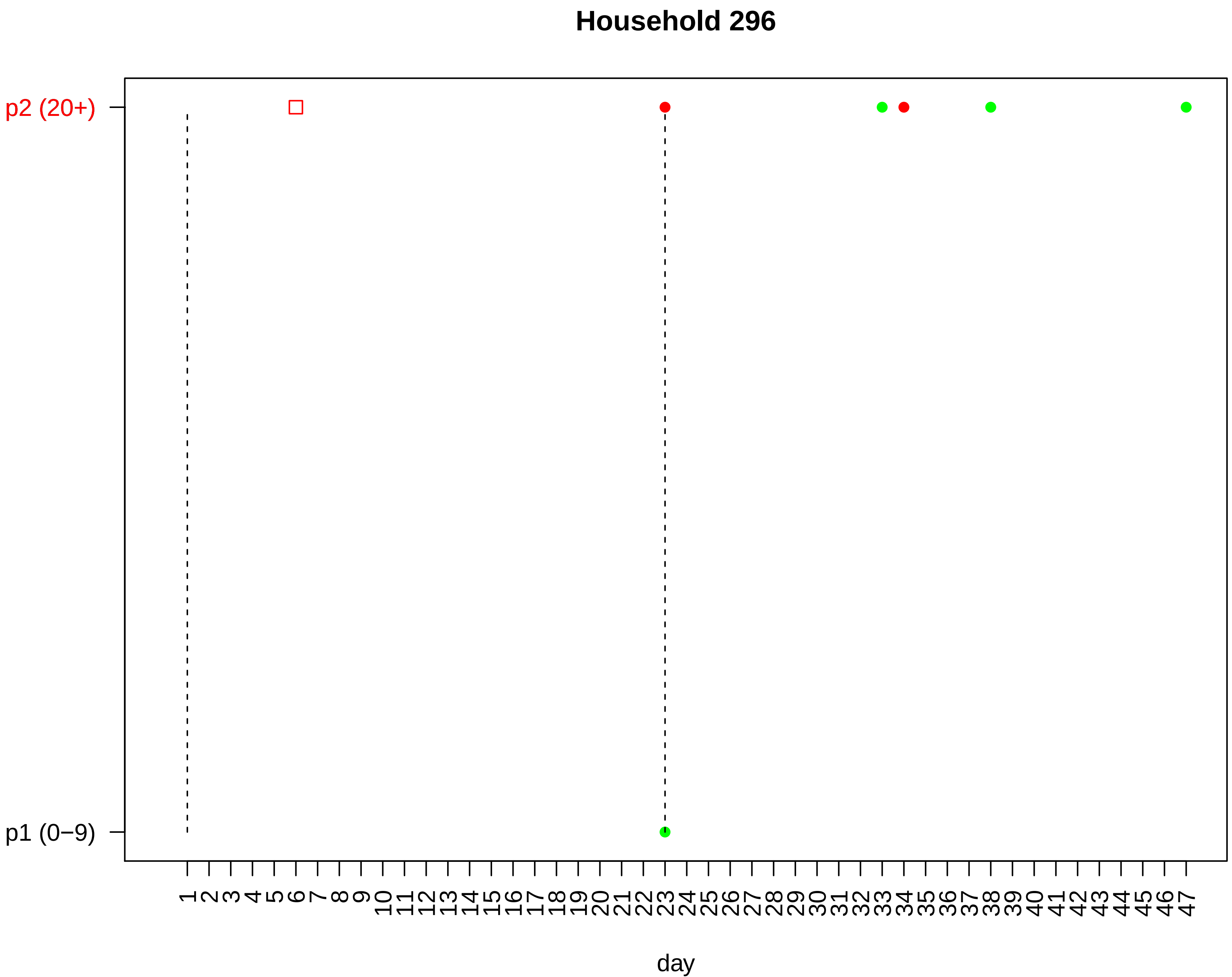

Household 296
p2 (20+)
p1 (0−9)
1 2 3 4 5 6 7 8 9 10 11 12 13 14 15 16 17 18 19 20 21 22 23 24 25 26 27 28 29 30 31 32 33 34 35 36 37 38 39 40 41 42 43 44 45 46 47
day

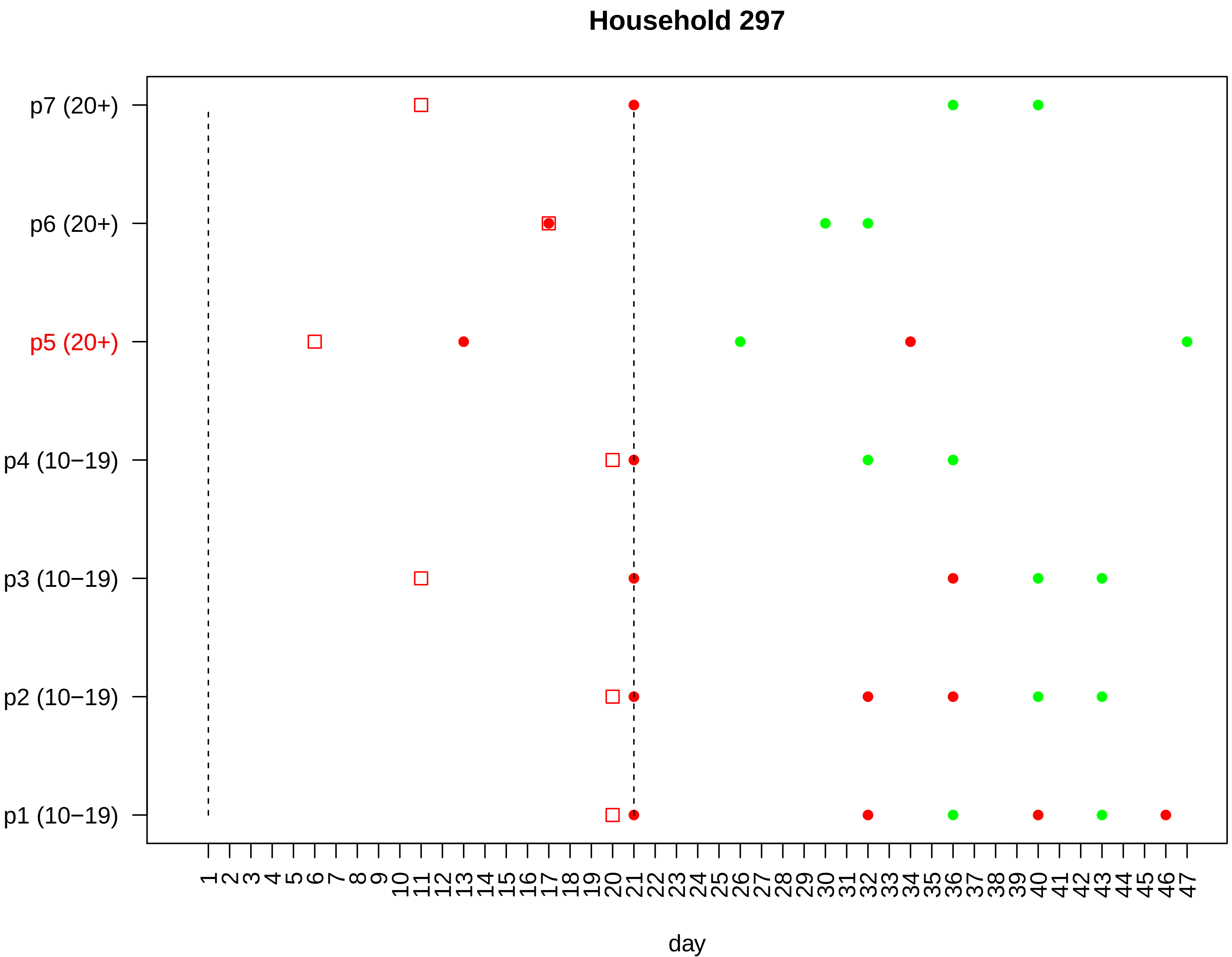

Household 297
p7 (20+)
p6 (20+)
p5 (20+)
p4 (10−19)
p3 (10−19)
p2 (10−19)
p1 (10−19)
1 2 3 4 5 6 7 8 9 10 11 12 13 14 15 16 17 18 19 20 21 22 23 24 25 26 27 28 29 30 31 32 33 34 35 36 37 38 39 40 41 42 43 44 45 46 47
day

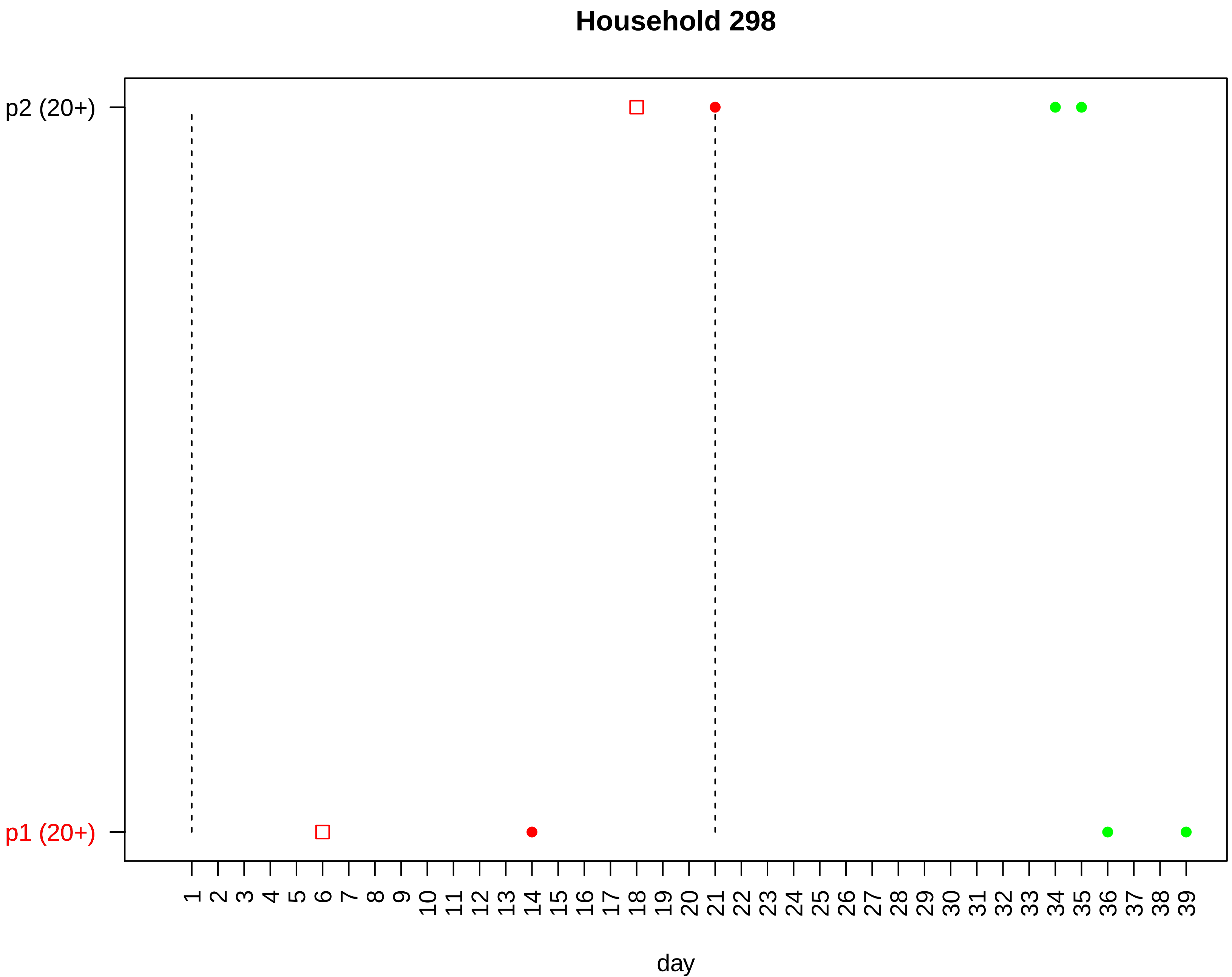

Household 298
p2 (20+)
p1 (20+)
1 2 3 4 5 6 7 8 9 10 11 12 13 14 15 16 17 18 19 20 21 22 23 24 25 26 27 28 29 30 31 32 33 34 35 36 37 38 39
day

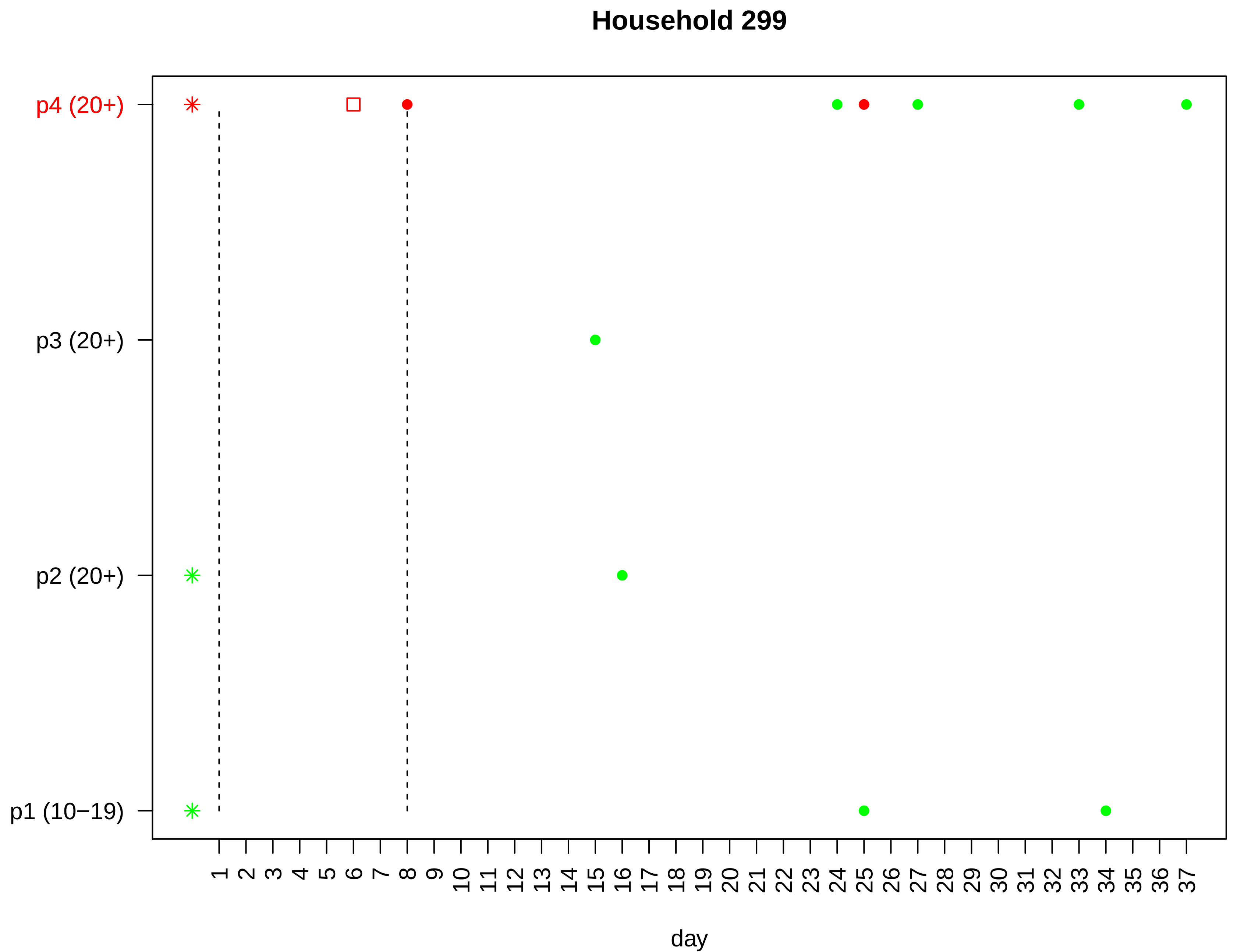
Household 299
p4 (20+)
p3 (20+)
p2 (20+)
p1 (10−19)
1 2 3 4 5 6 7 8 9 10 11 12 13 14 15 16 17 18 19 20 21 22 23 24 25 26 27 28 29 30 31 32 33 34 35 36 37
day

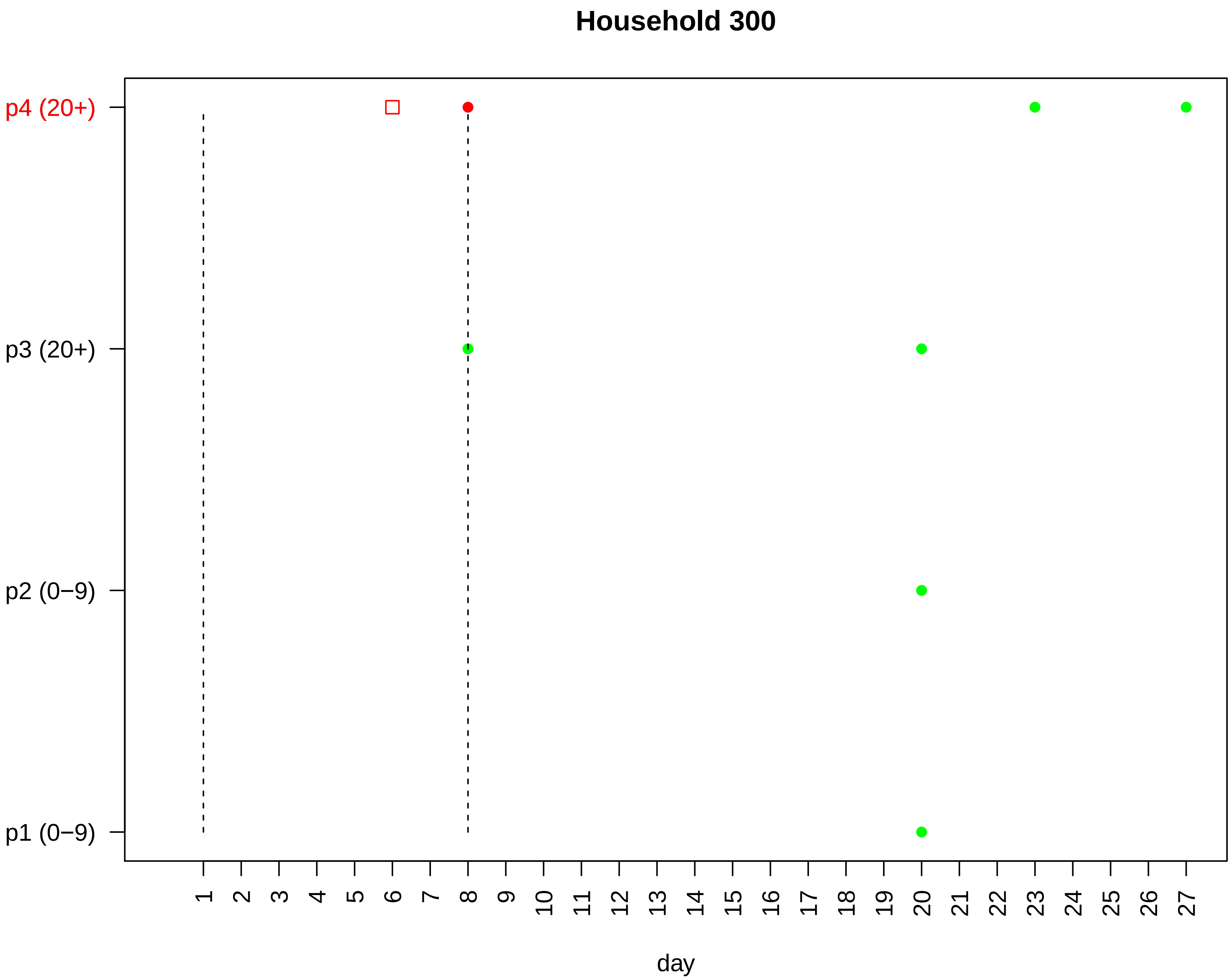

Household 300
p4 (20+)
p3 (20+)
p2 (0−9)
p1 (0−9)
1 2 3 4 5 6 7 8 9 10 11 12 13 14 15 16 17 18 19 20 21 22 23 24 25 26 27
day

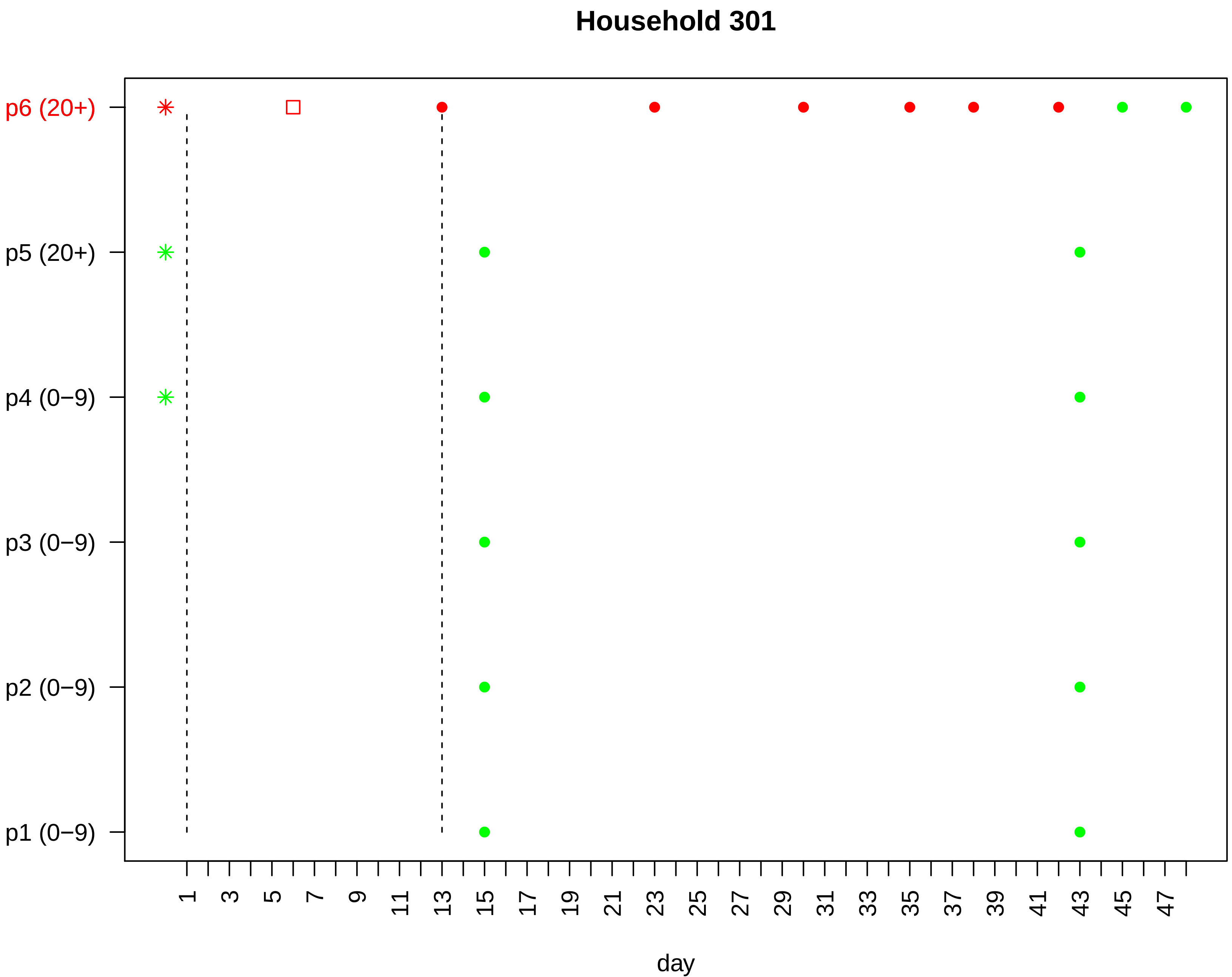
Household 301
p6 (20+)
p5 (20+)
p4 (0−9)
p3 (0−9)
p2 (0−9)
p1 (0−9)
1 3 5 7 9 11 13 15 17 19 21 23 25 27 29 31 33 35 37 39 41 43 45 47
day

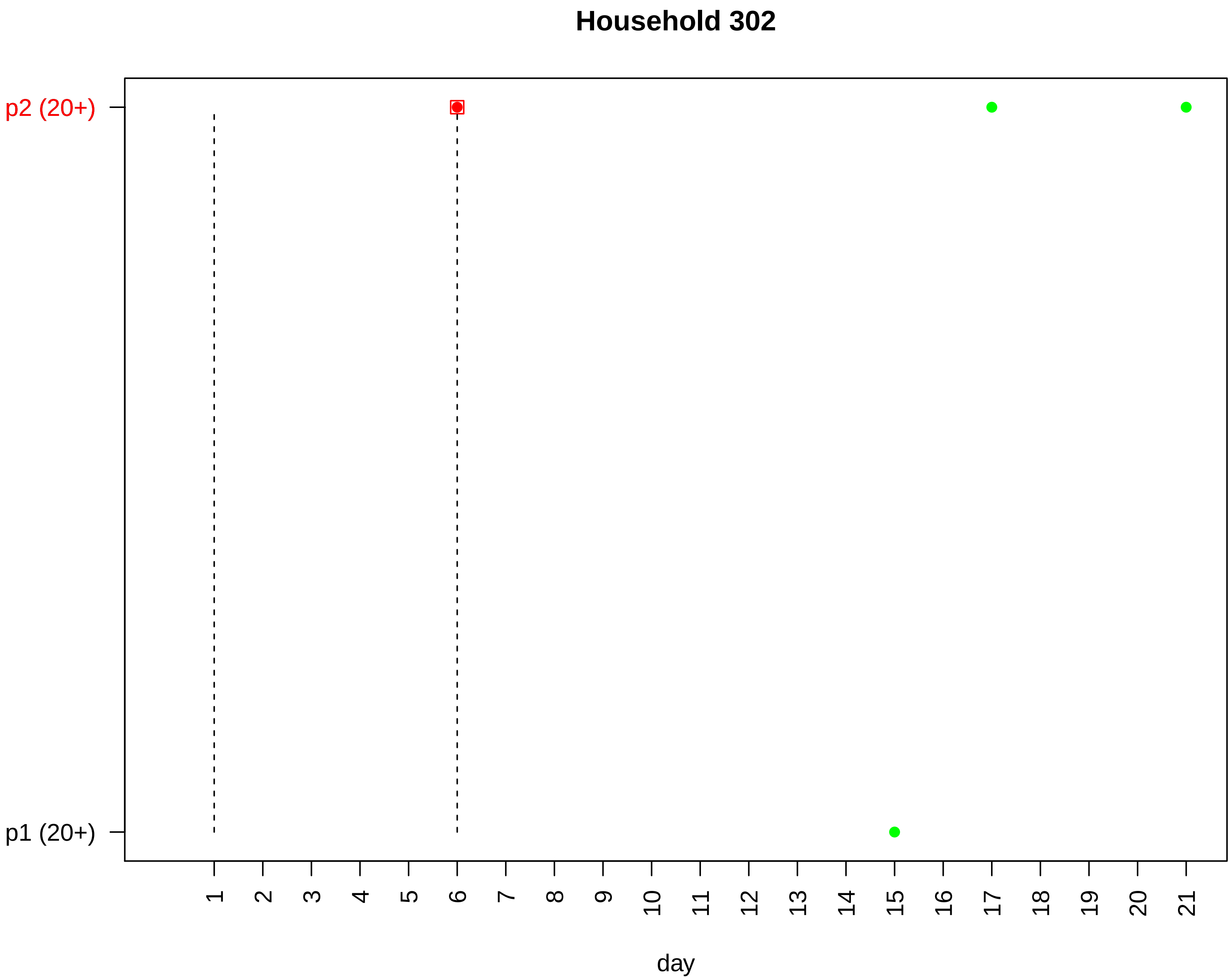
Household 302
p2 (20+)
p1 (20+)
1
2
3
4
5
6
7
8
9
10
11
12
13
14
15
16
17
18
19
20
21
day

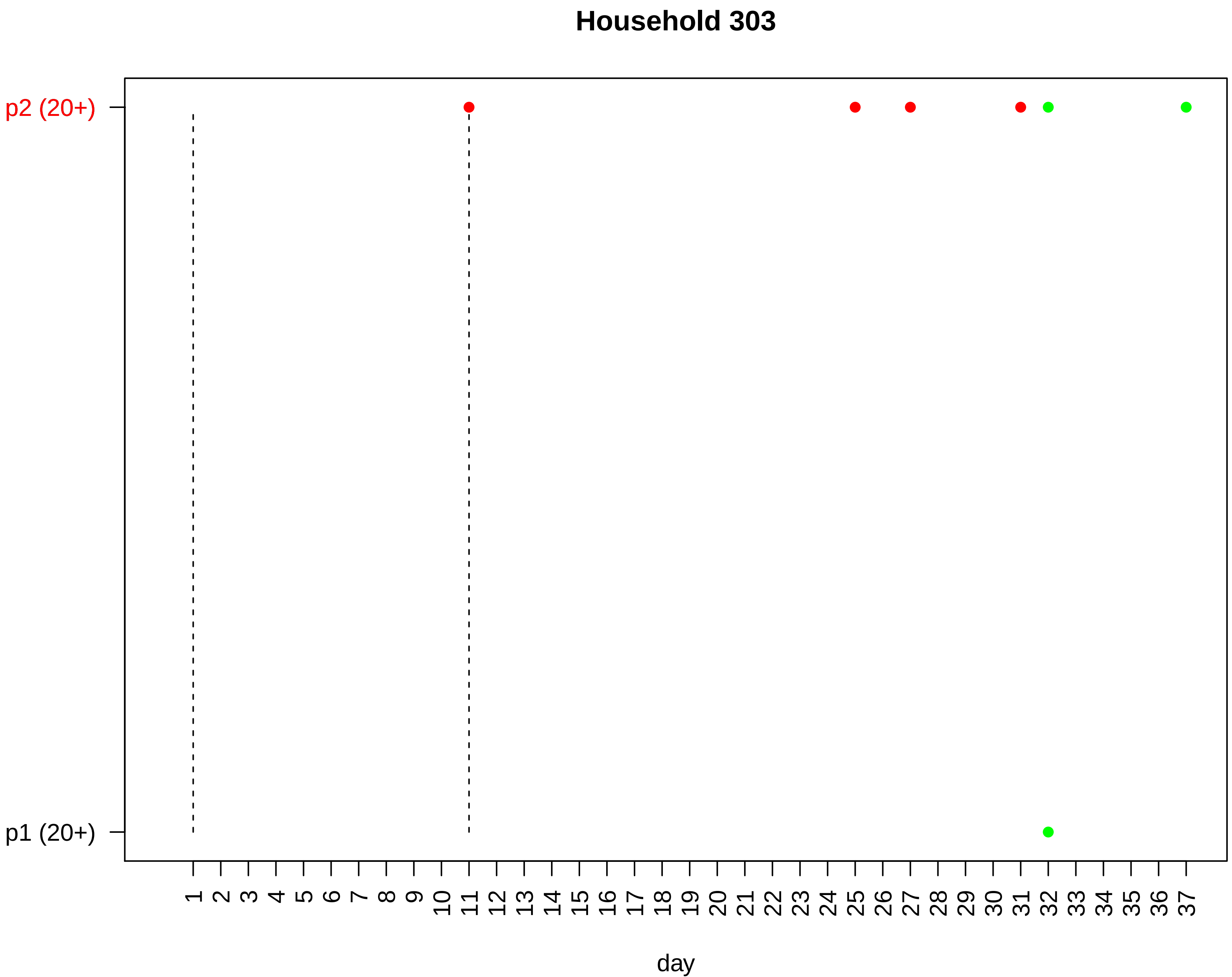

Household 303
p2 (20+)
p1 (20+)
1
2
3
4
5
6
7
8
9
10
11
12
13
14
15
16
17
18
19
20
21
22
23
24
25
26
27
28
29
30
31
32
33
34
35
36
37
day

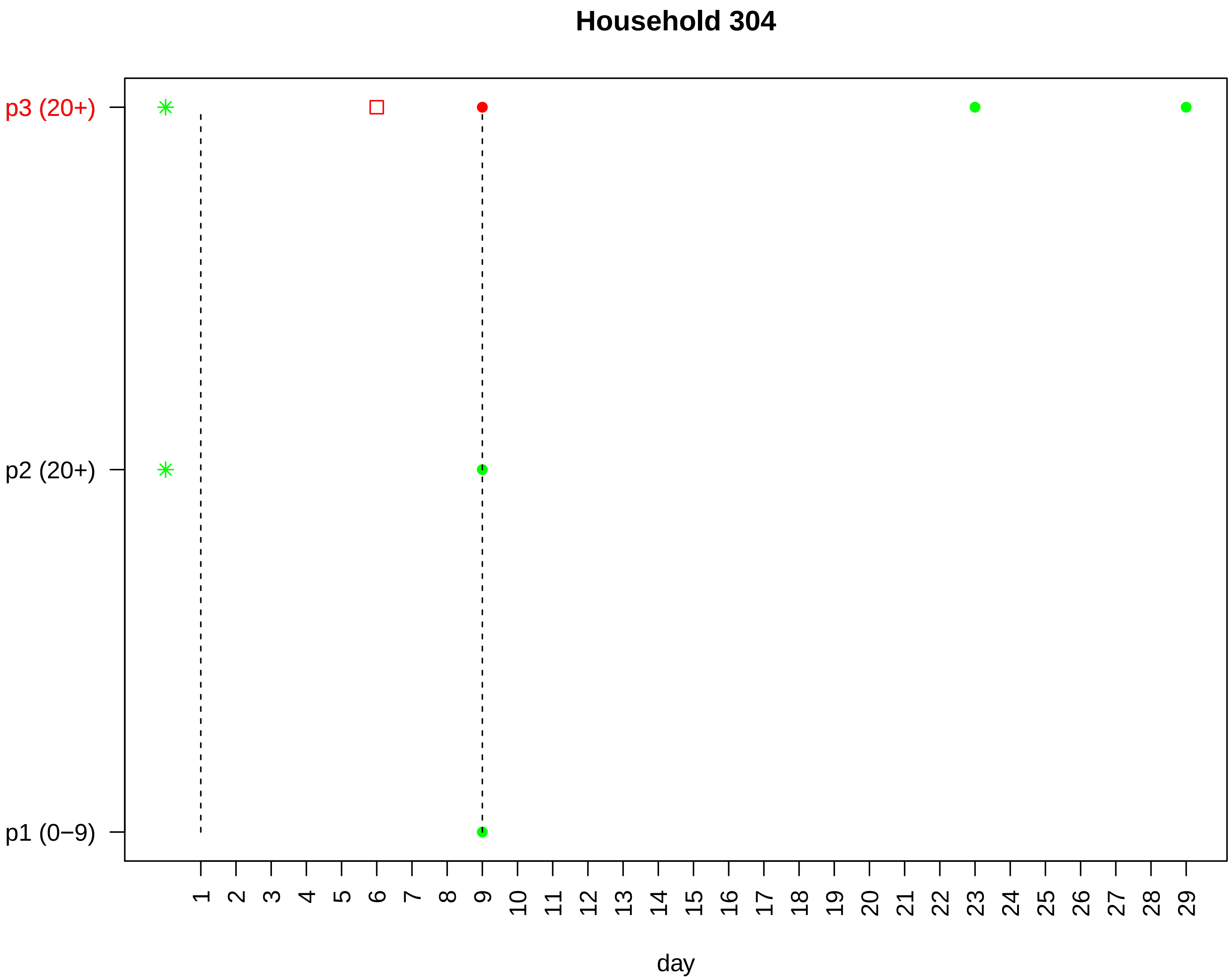

Household 304
p3 (20+)
p2 (20+)
p1 (0−9)
1 2 3 4 5 6 7 8 9 10 11 12 13 14 15 16 17 18 19 20 21 22 23 24 25 26 27 28 29
day

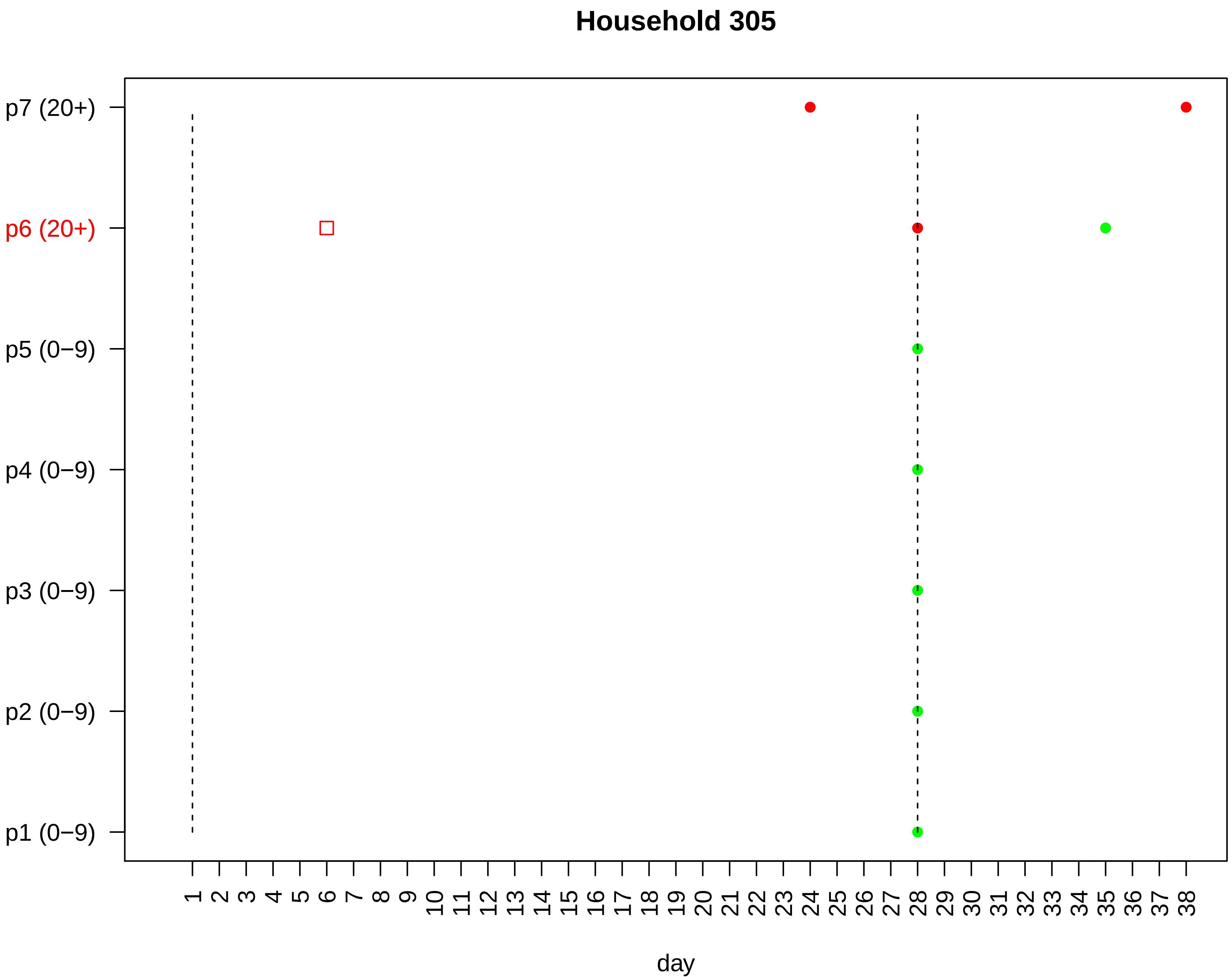

Household 305
p7 (20+)
p6 (20+)
p5 (0−9)
p4 (0−9)
p3 (0−9)
p2 (0−9)
p1 (0−9)
1 2 3 4 5 6 7 8 9 10 11 12 13 14 15 16 17 18 19 20 21 22 23 24 25 26 27 28 29 30 31 32 33 34 35 36 37 38
day

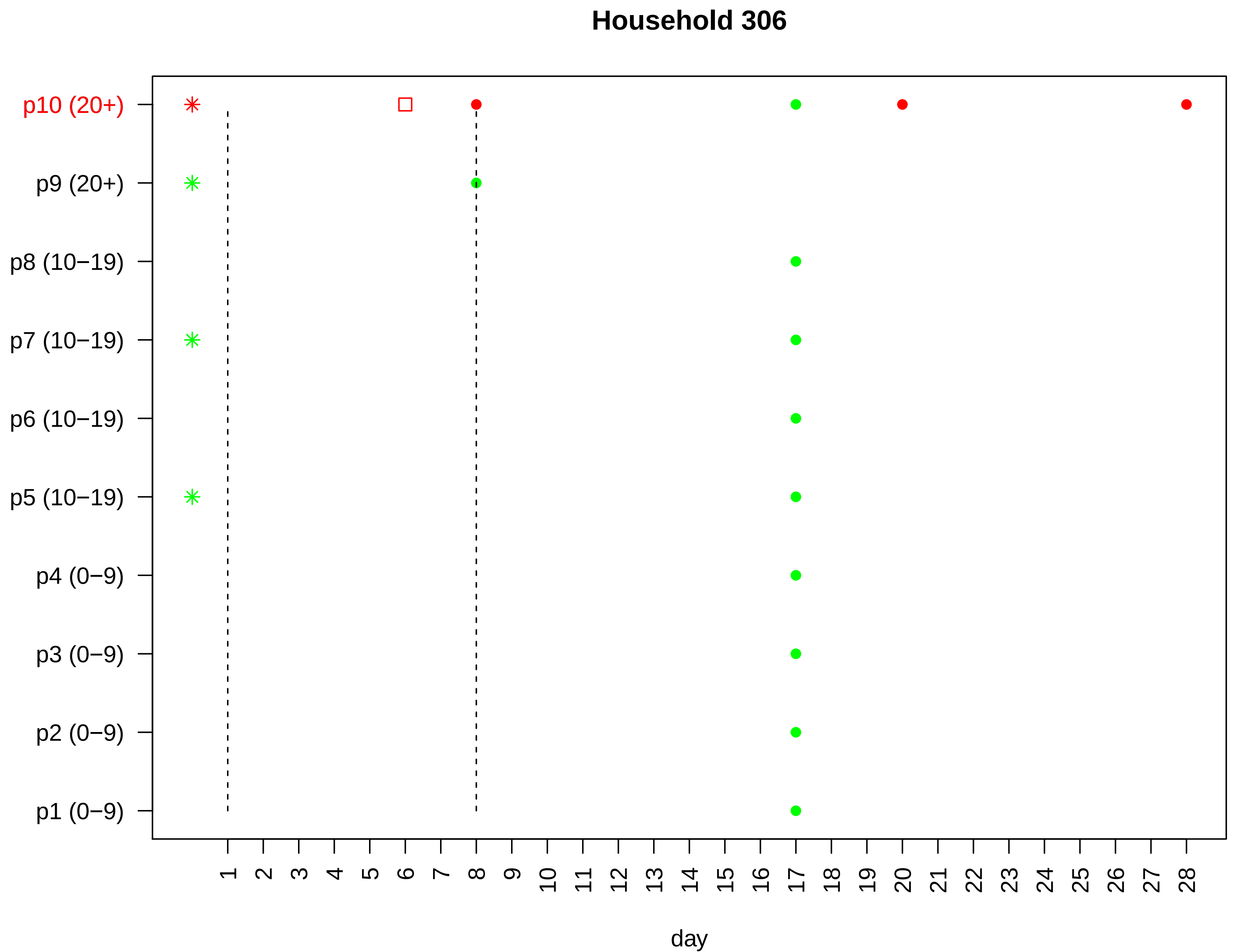

Household 306
p10 (20+)
p9 (20+)
p8 (10−19)
p7 (10−19)
p6 (10−19)
p5 (10−19)
p4 (0−9)
p3 (0−9)
p2 (0−9)
p1 (0−9)
1 2 3 4 5 6 7 8 9 10 11 12 13 14 15 16 17 18 19 20 21 22 23 24 25 26 27 28
day

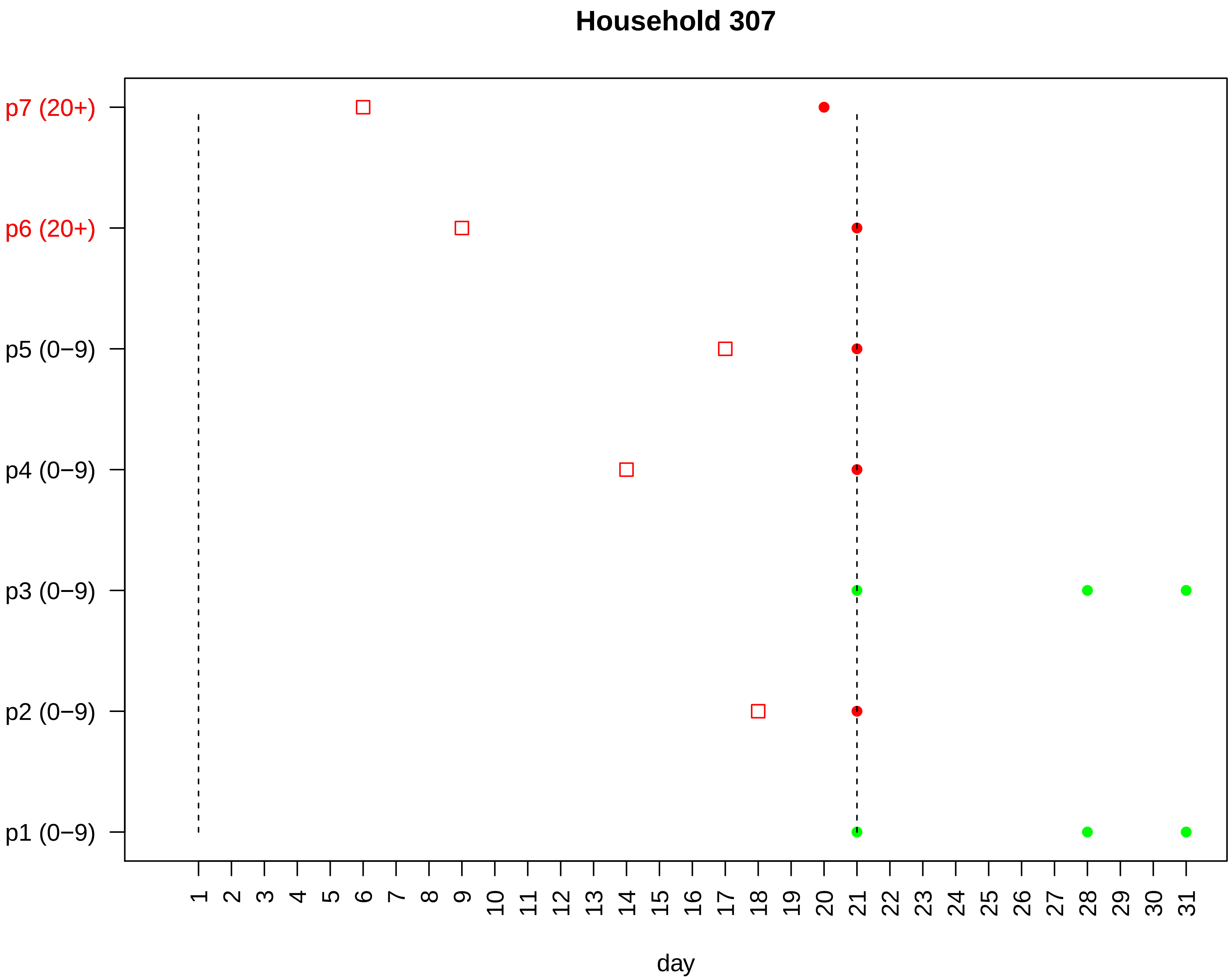
Household 307
p7 (20+)
p6 (20+)
p5 (0−9)
p4 (0−9)
p3 (0−9)
p2 (0−9)
p1 (0−9)
1 2 3 4 5 6 7 8 9 10 11 12 13 14 15 16 17 18 19 20 21 22 23 24 25 26 27 28 29 30 31
day

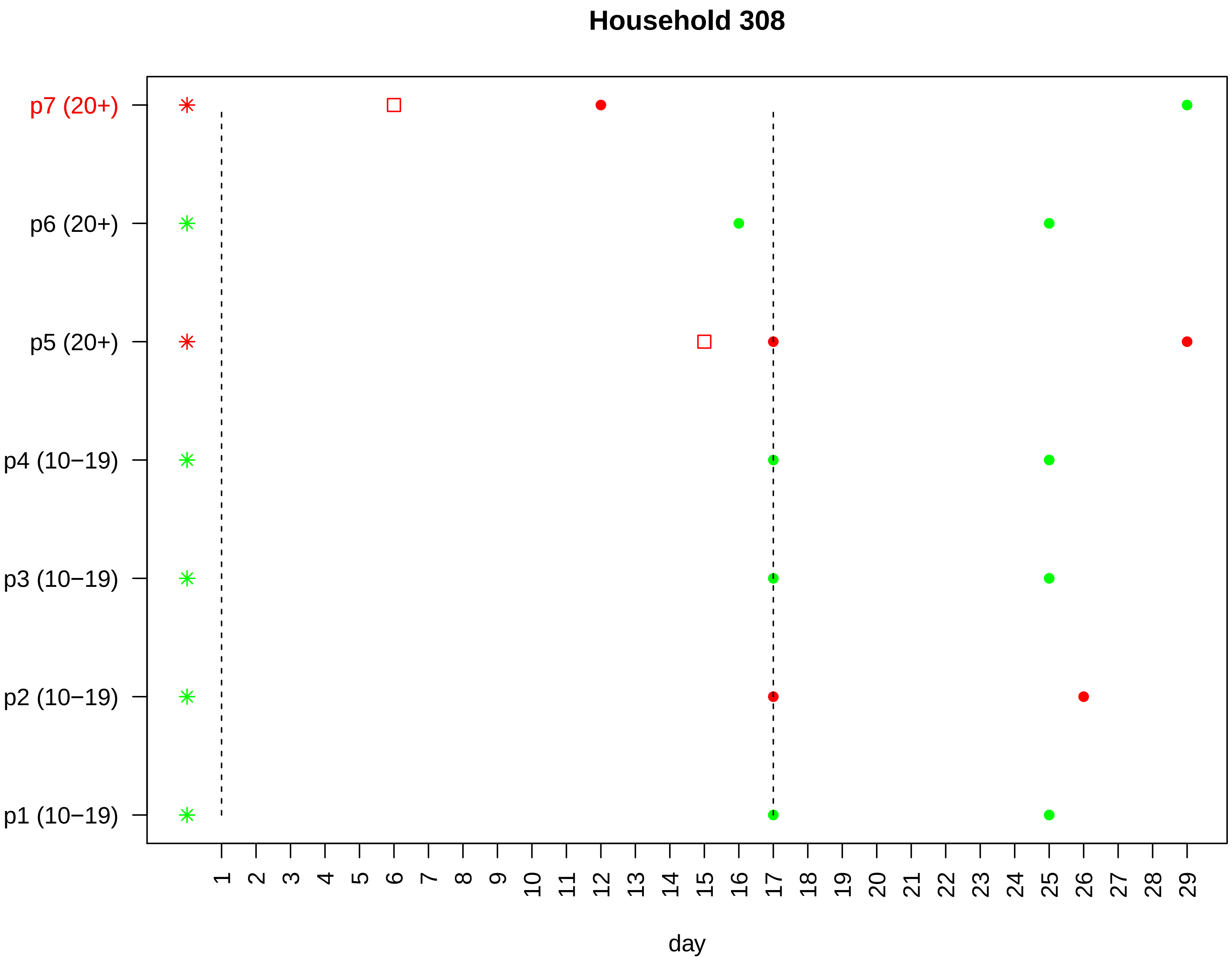

Household 308
p7 (20+)
p6 (20+)
p5 (20+)
p4 (10−19)
p3 (10−19)
p2 (10−19)
p1 (10−19)
1 2 3 4 5 6 7 8 9 10 11 12 13 14 15 16 17 18 19 20 21 22 23 24 25 26 27 28 29
day

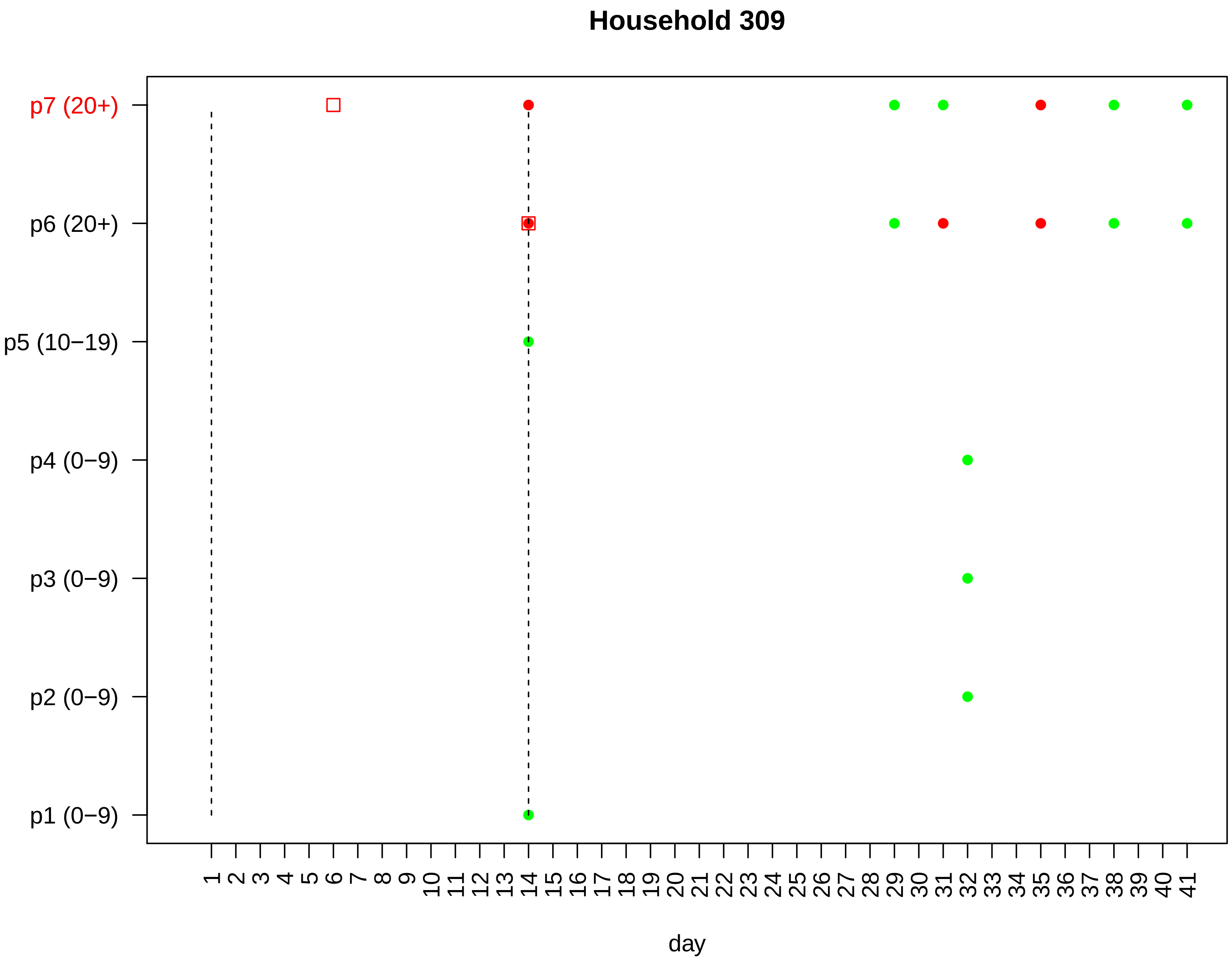
Household 309
p7 (20+)
p6 (20+)
p5 (10−19)
p4 (0−9)
p3 (0−9)
p2 (0−9)
p1 (0−9)
1 2 3 4 5 6 7 8 9 10 11 12 13 14 15 16 17 18 19 20 21 22 23 24 25 26 27 28 29 30 31 32 33 34 35 36 37 38 39 40 41
day

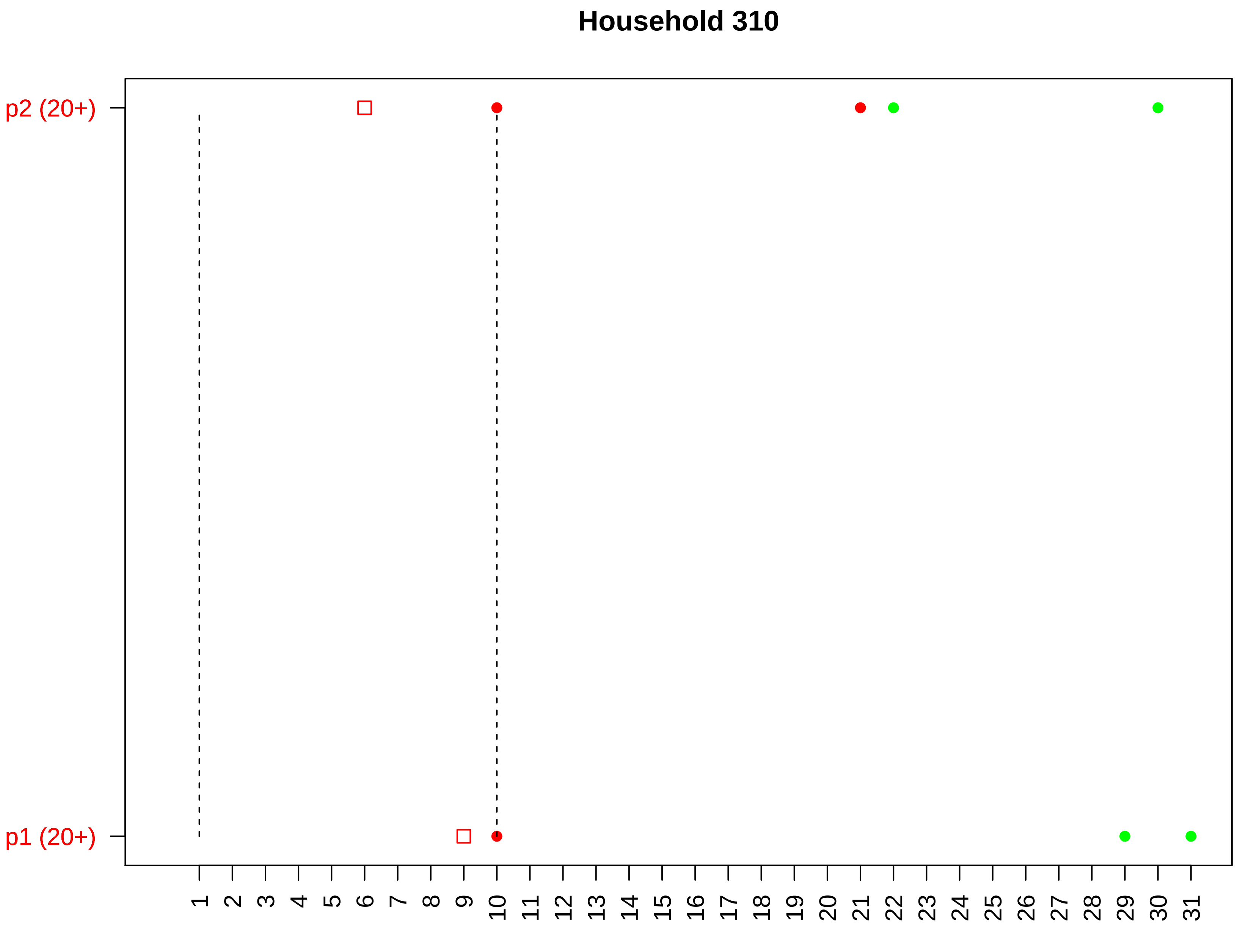

Household 310
p2 (20+)
p1 (20+)
1 2 3 4 5 6 7 8 9 10 11 12 13 14 15 16 17 18 19 20 21 22 23 24 25 26 27 28 29 30 31
day

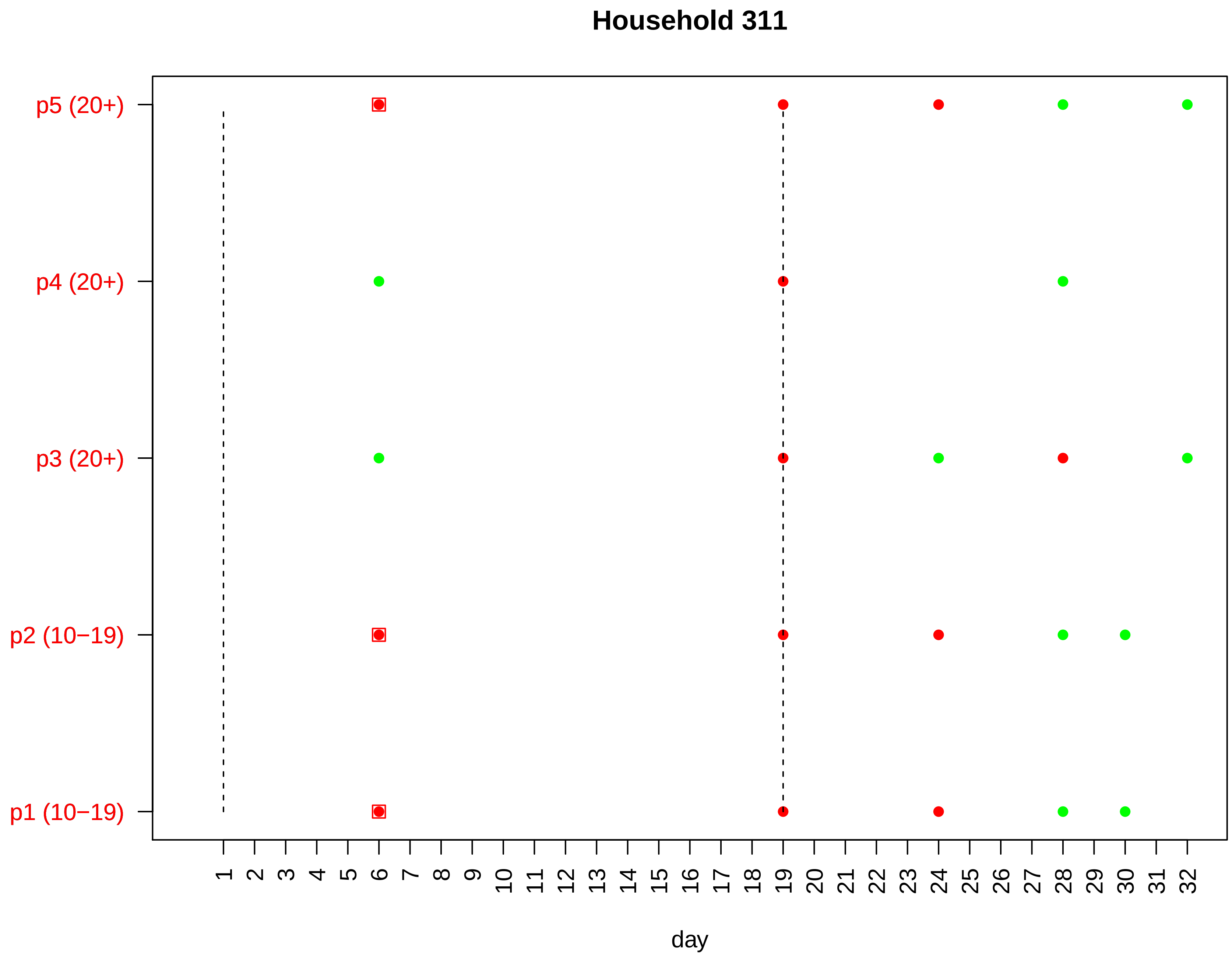
Household 311
p5 (20+)
p4 (20+)
p3 (20+)
p2 (10−19)
p1 (10−19)
1 2 3 4 5 6 7 8 9 10 11 12 13 14 15 16 17 18 19 20 21 22 23 24 25 26 27 28 29 30 31 32
day

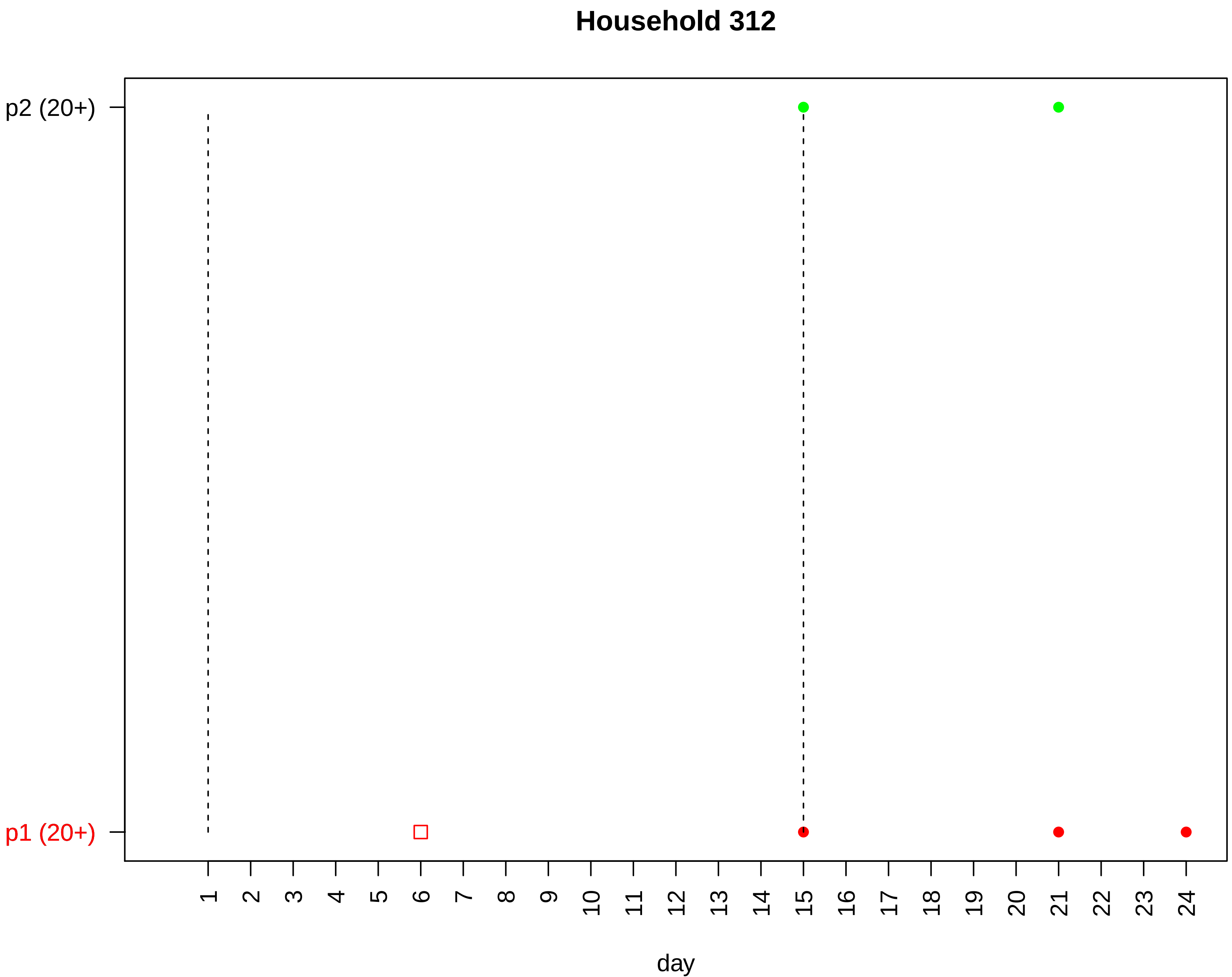
Household 312
p2 (20+)
p1 (20+)
1
2
3
4
5
6
7
8
9
10
11
12
13
14
15
16
17
18
19
20
21
22
23
24
day

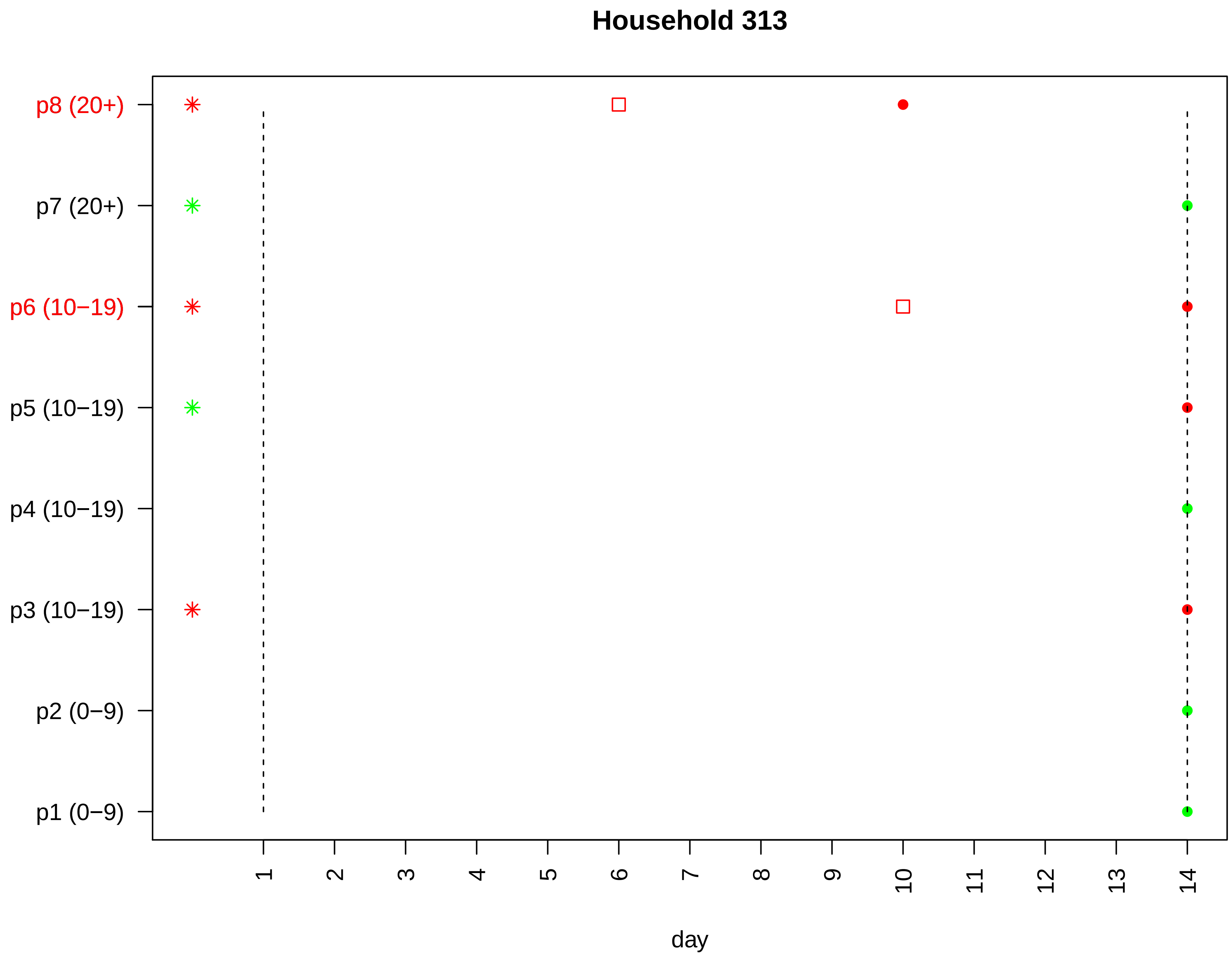

Household 313
p8 (20+)
p7 (20+)
p6 (10−19)
p5 (10−19)
p4 (10−19)
p3 (10−19)
p2 (0−9)
p1 (0−9)
1
2
3
4
5
6
7
8
9
10
11
12
13
14
day

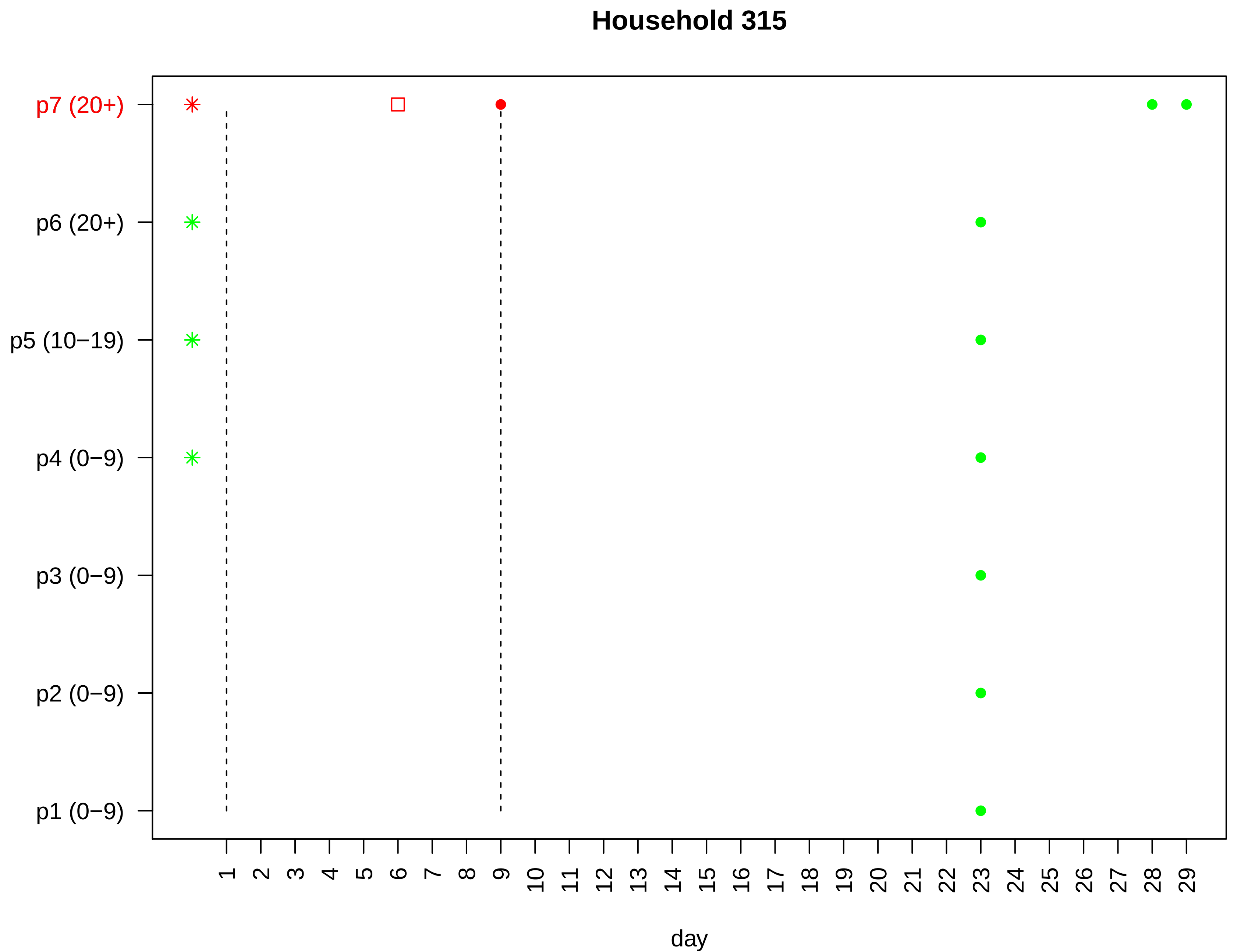
Household 315
p7 (20+)
p6 (20+)
p5 (10−19)
p4 (0−9)
p3 (0−9)
p2 (0−9)
p1 (0−9)
1 2 3 4 5 6 7 8 9 10 11 12 13 14 15 16 17 18 19 20 21 22 23 24 25 26 27 28 29
day

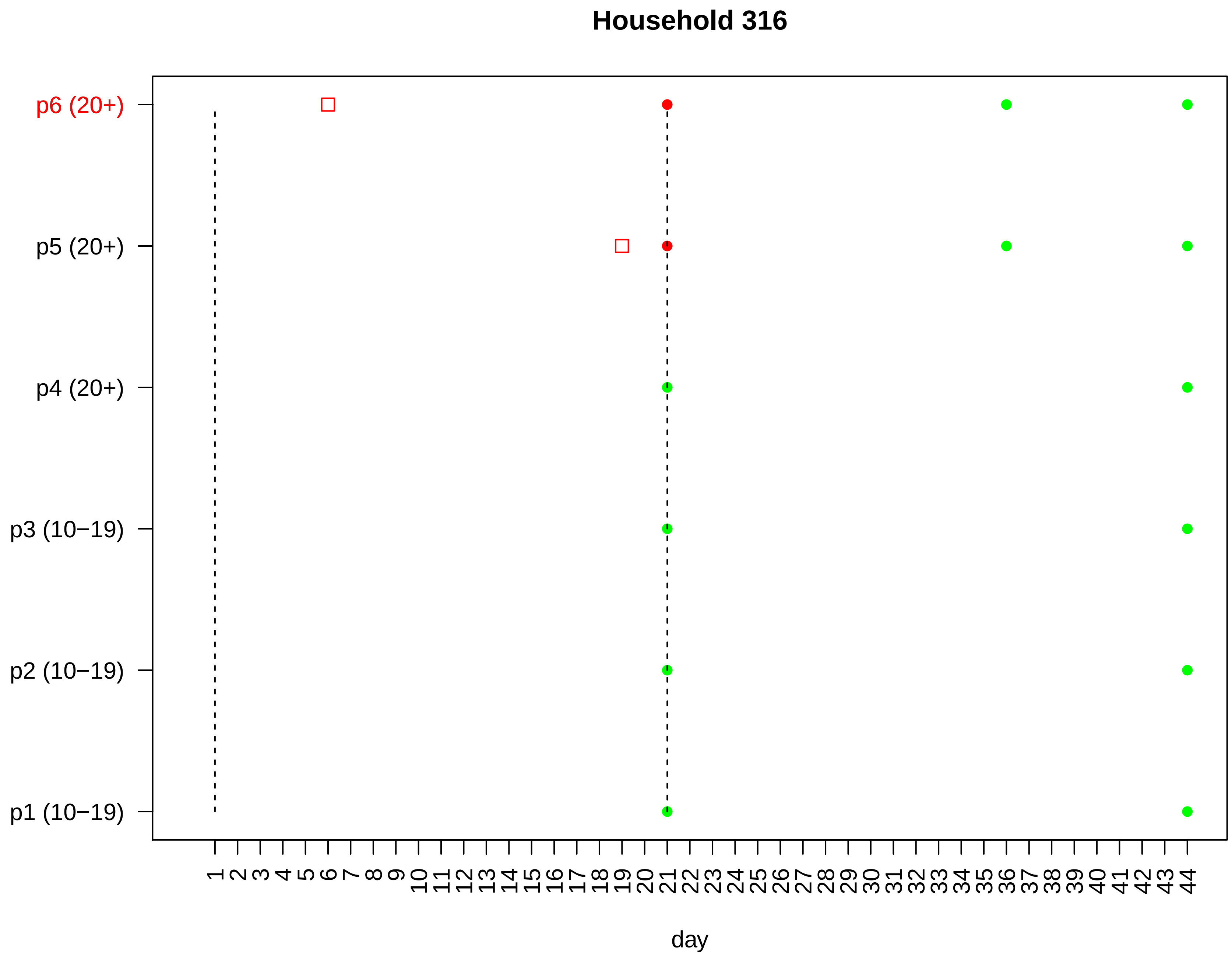
Household 316
p6 (20+)
p5 (20+)
p4 (20+)
p3 (10−19)
p2 (10−19)
p1 (10−19)
1 2 3 4 5 6 7 8 9 10 11 12 13 14 15 16 17 18 19 20 21 22 23 24 25 26 27 28 29 30 31 32 33 34 35 36 37 38 39 40 41 42 43 44
day

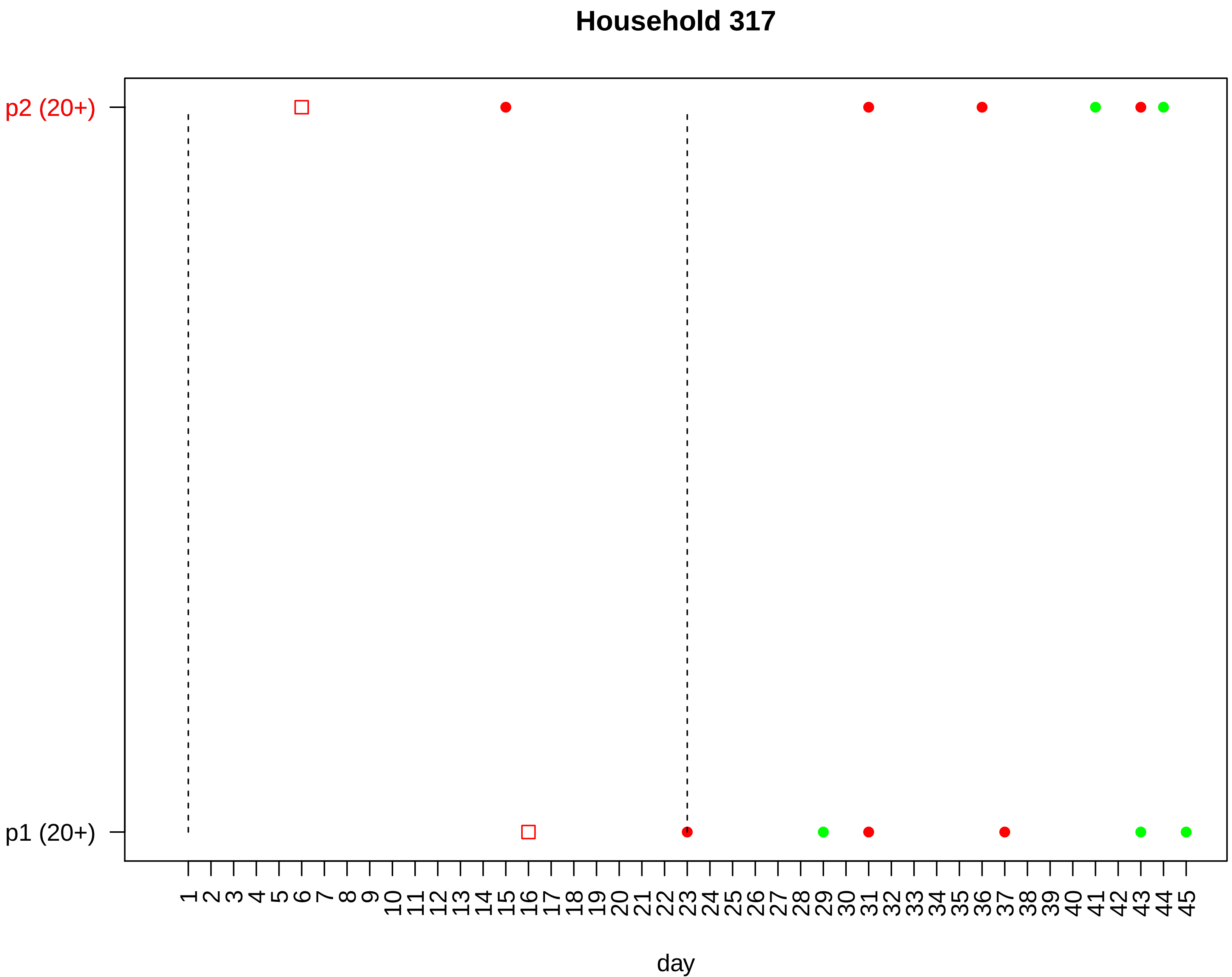

Household 317
p2 (20+)
p1 (20+)
1 2 3 4 5 6 7 8 9 10 11 12 13 14 15 16 17 18 19 20 21 22 23 24 25 26 27 28 29 30 31 32 33 34 35 36 37 38 39 40 41 42 43 44 45
day

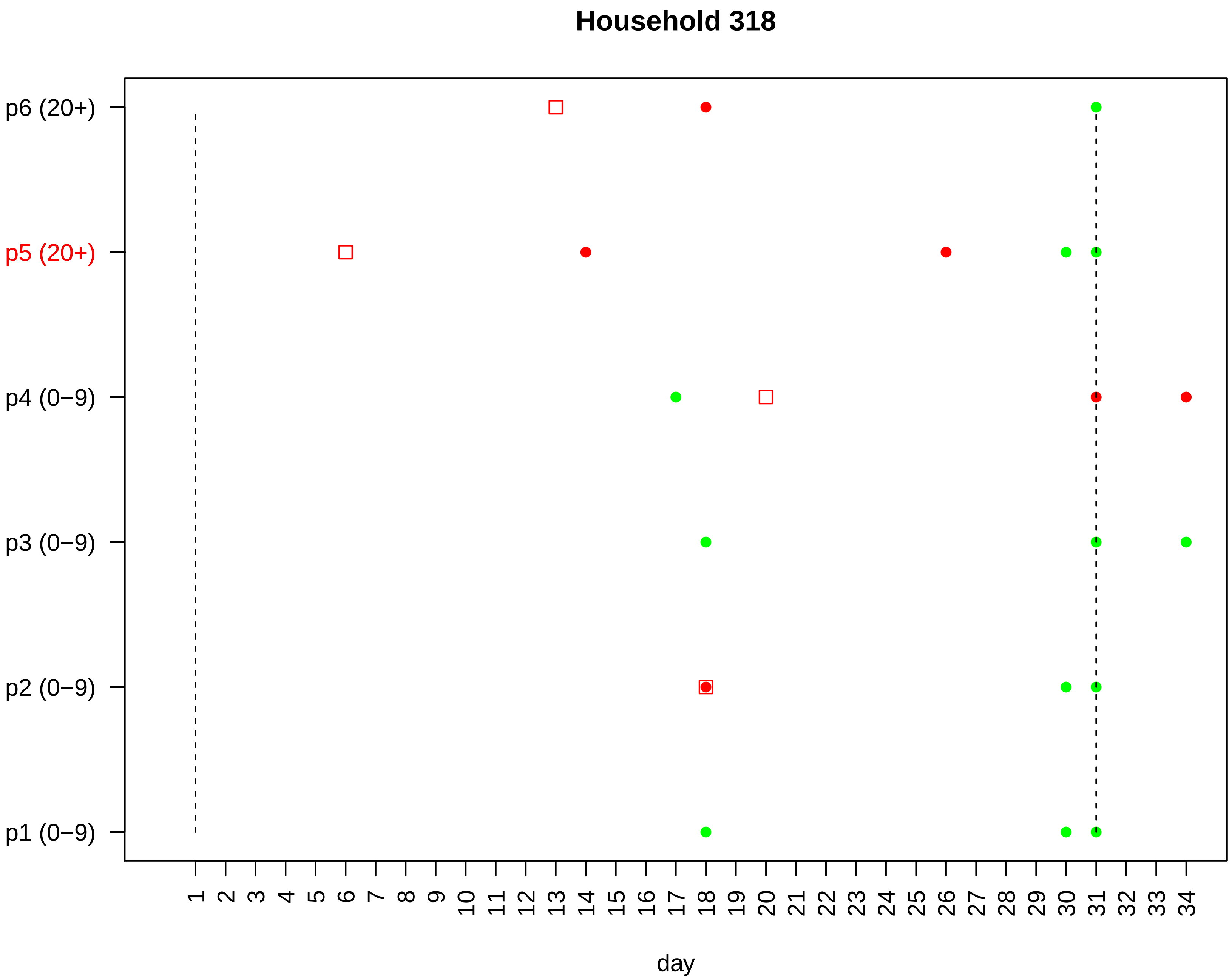

Household 318
p6 (20+)
p5 (20+)
p4 (0−9)
p3 (0−9)
p2 (0−9)
p1 (0−9)
1 2 3 4 5 6 7 8 9 10 11 12 13 14 15 16 17 18 19 20 21 22 23 24 25 26 27 28 29 30 31 32 33 34
day

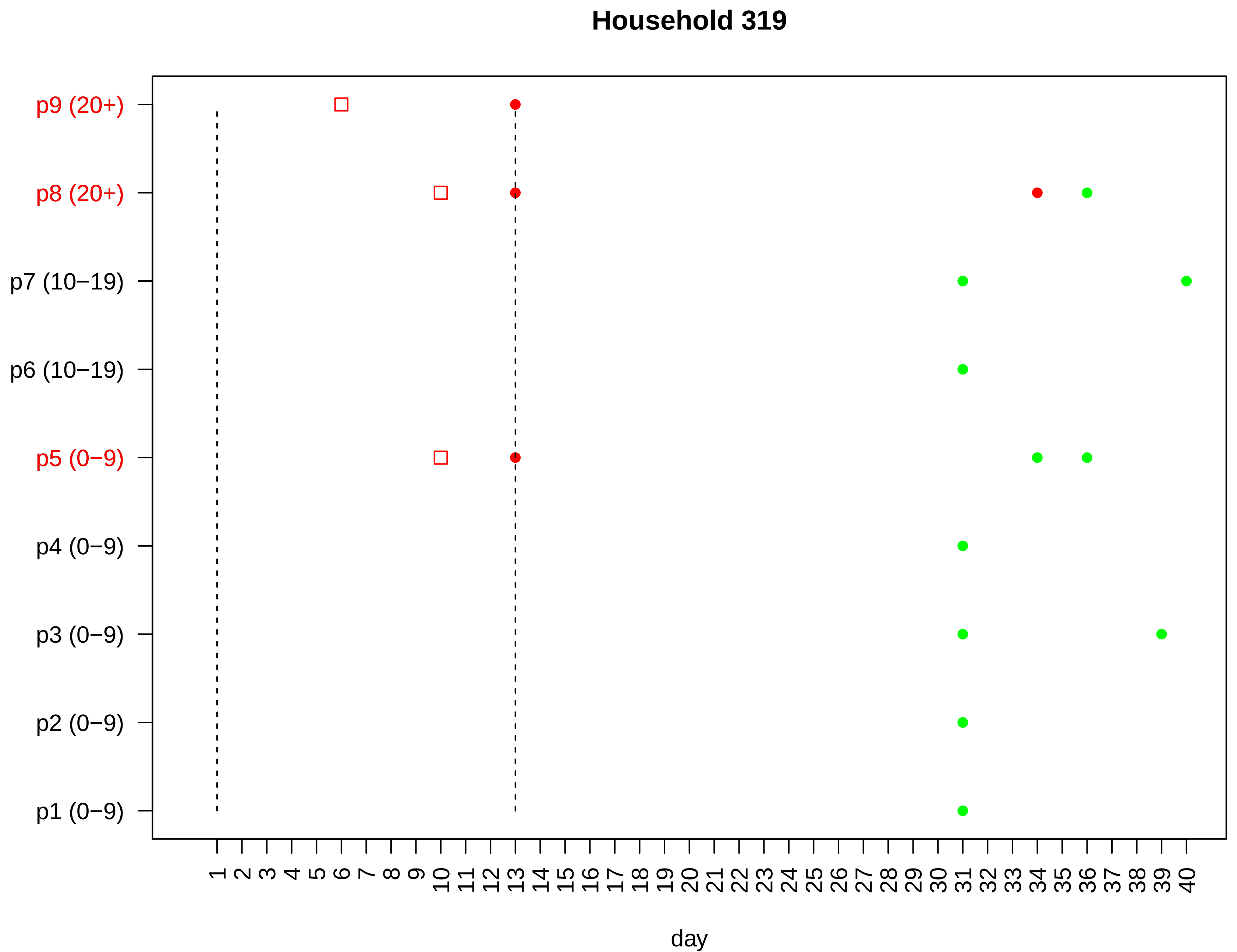
Household 319
p9 (20+)
p8 (20+)
p7 (10−19)
p6 (10−19)
p5 (0−9)
p4 (0−9)
p3 (0−9)
p2 (0−9)
p1 (0−9)
1 2 3 4 5 6 7 8 9 10 11 12 13 14 15 16 17 18 19 20 21 22 23 24 25 26 27 28 29 30 31 32 33 34 35 36 37 38 39 40
day

Household 320

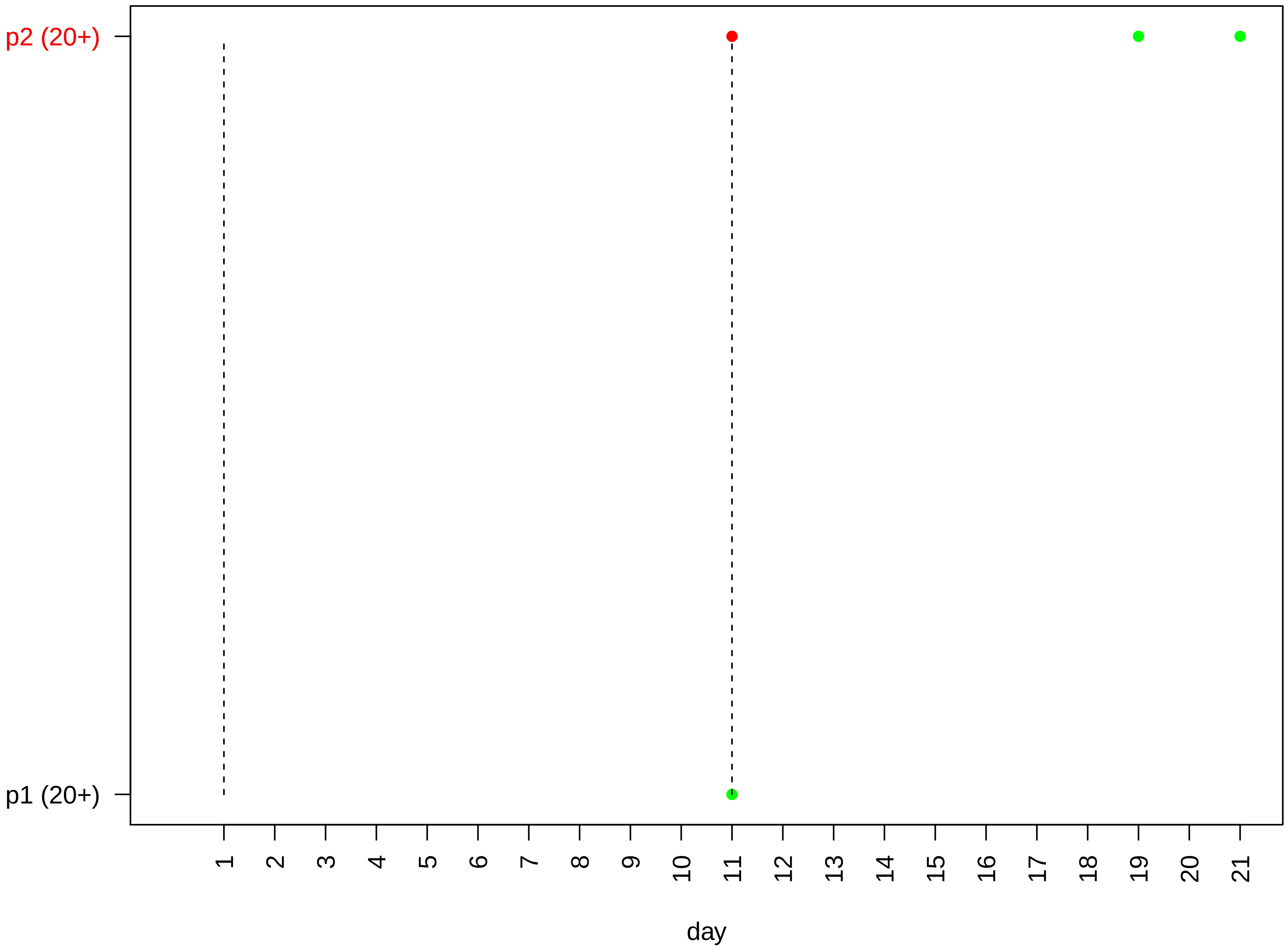

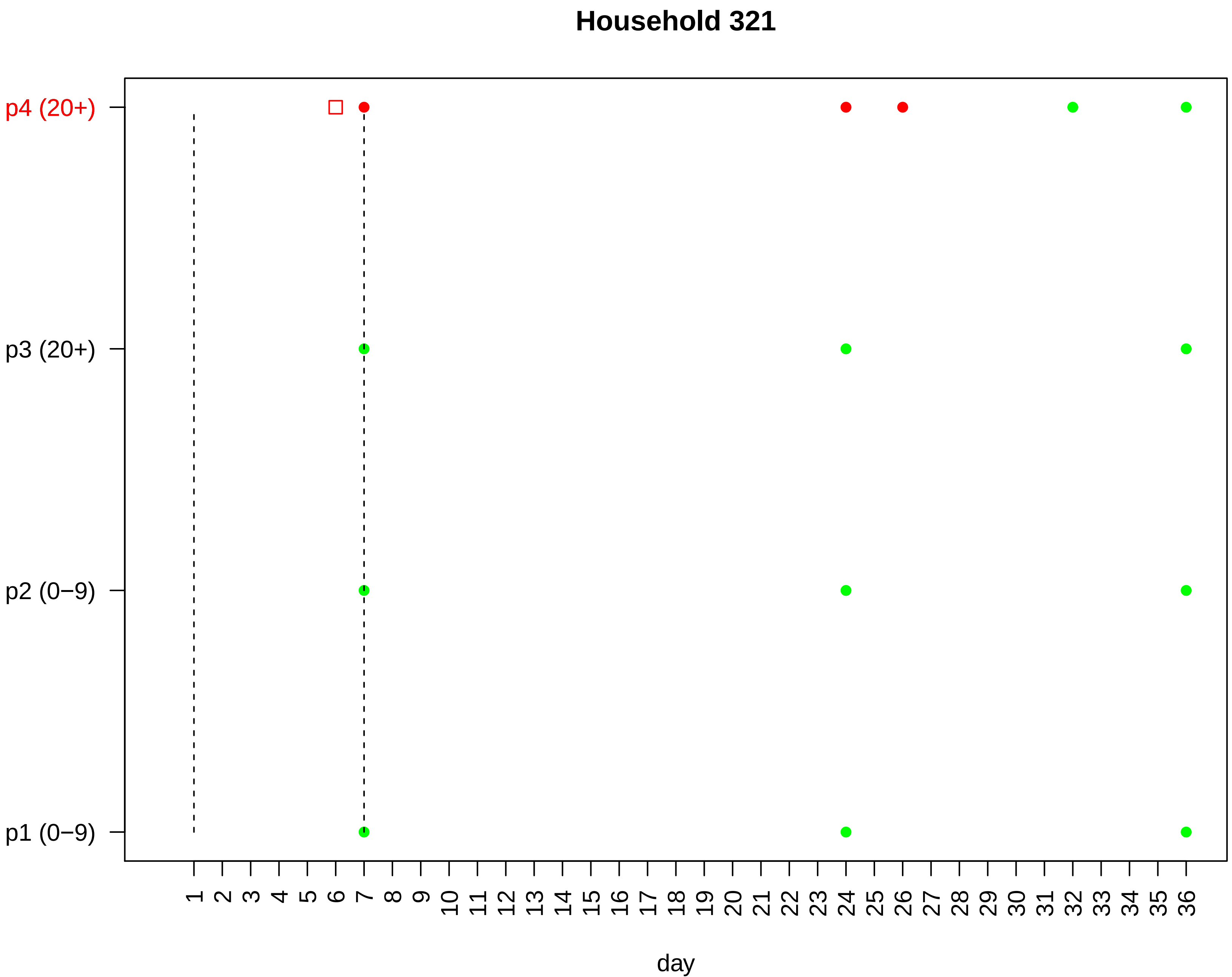

Household 321
p4 (20+)
p3 (20+)
p2 (0−9)
p1 (0−9)
1 2 3 4 5 6 7 8 9 10 11 12 13 14 15 16 17 18 19 20 21 22 23 24 25 26 27 28 29 30 31 32 33 34 35 36
day

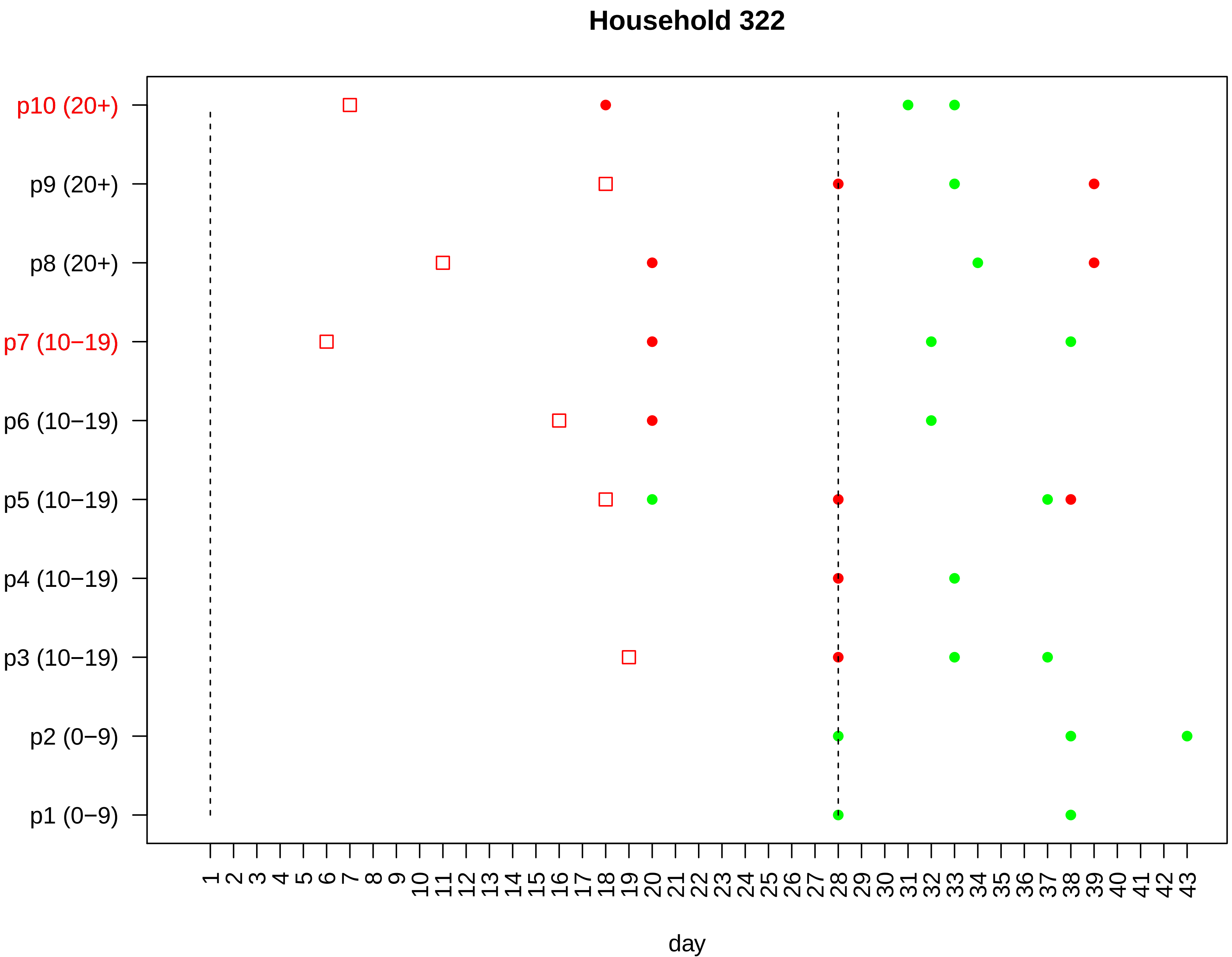

Household 322
p10 (20+)
p9 (20+)
p8 (20+)
p7 (10−19)
p6 (10−19)
p5 (10−19)
p4 (10−19)
p3 (10−19)
p2 (0−9)
p1 (0−9)
1 2 3 4 5 6 7 8 9 10 11 12 13 14 15 16 17 18 19 20 21 22 23 24 25 26 27 28 29 30 31 32 33 34 35 36 37 38 39 40 41 42 43
day

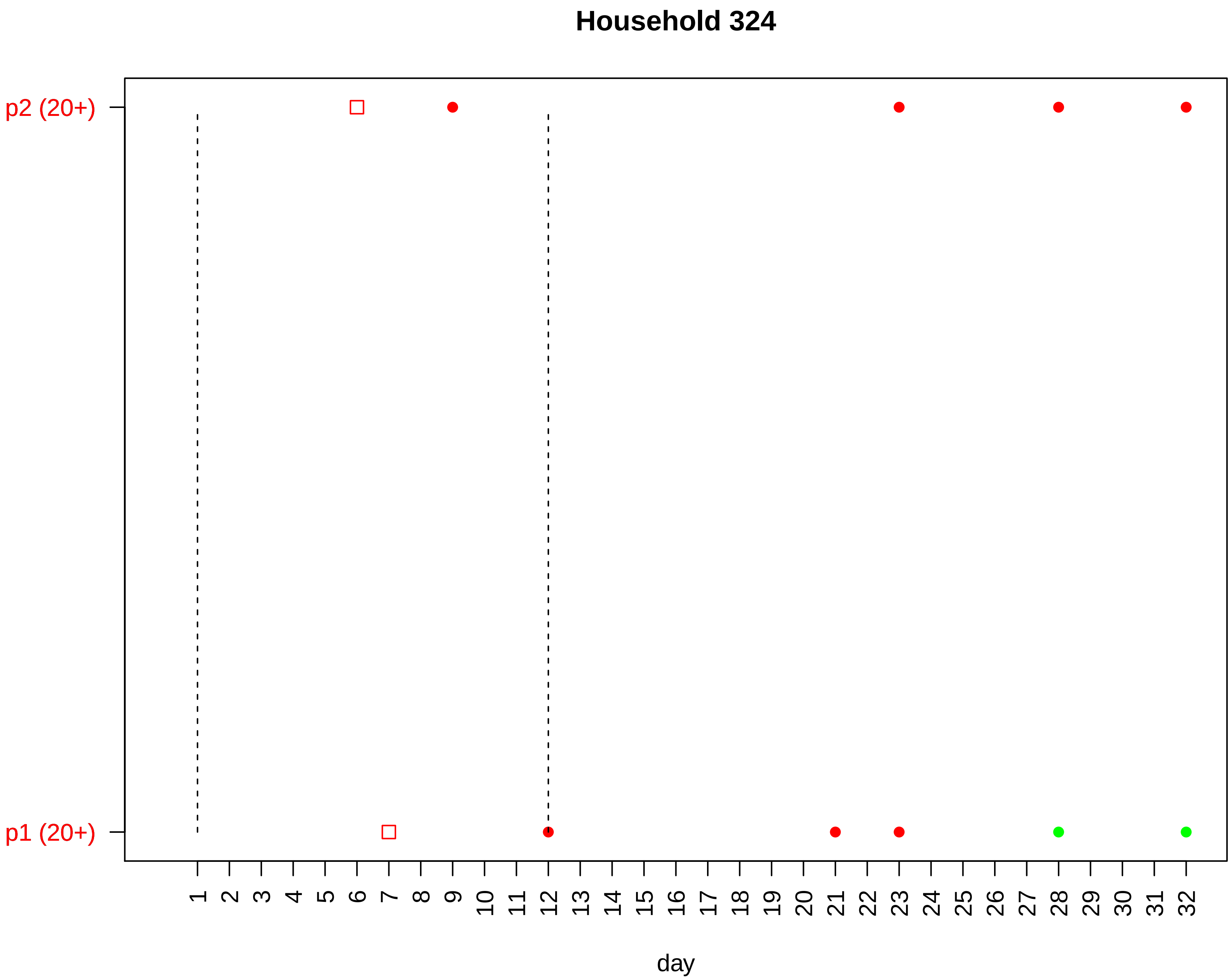

Household 324
p2 (20+)
p1 (20+)
1
2
3
4
5
6
7
8
9
10
11
12
13
14
15
16
17
18
19
20
21
22
23
24
25
26
27
28
29
30
31
32
day

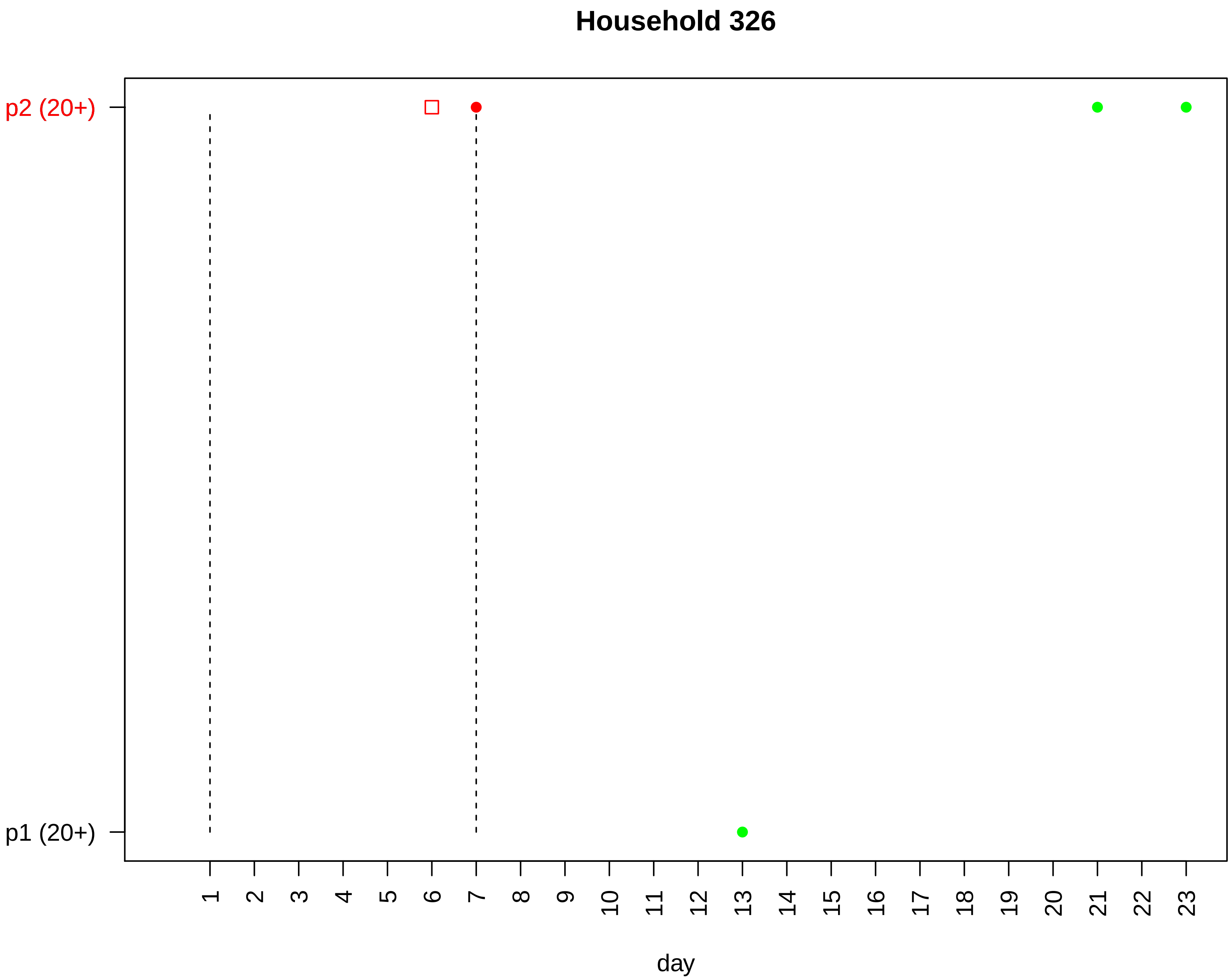
Household 326
p2 (20+)
p1 (20+)
1
2
3
4
5
6
7
8
9
10
11
12
13
14
15
16
17
18
19
20
21
22
23
day

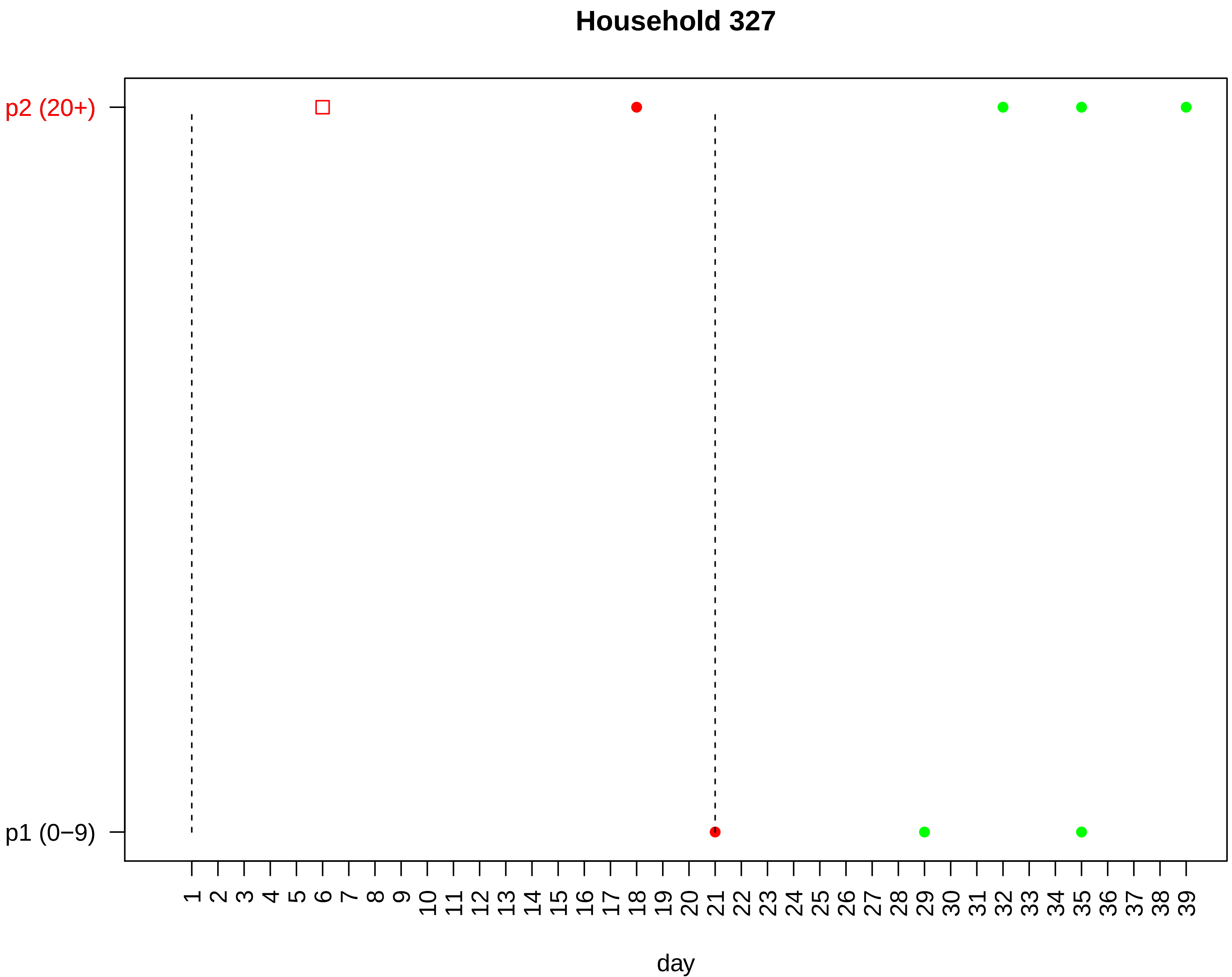
Household 327
p2 (20+)
p1 (0−9)
1 2 3 4 5 6 7 8 9 10 11 12 13 14 15 16 17 18 19 20 21 22 23 24 25 26 27 28 29 30 31 32 33 34 35 36 37 38 39
day

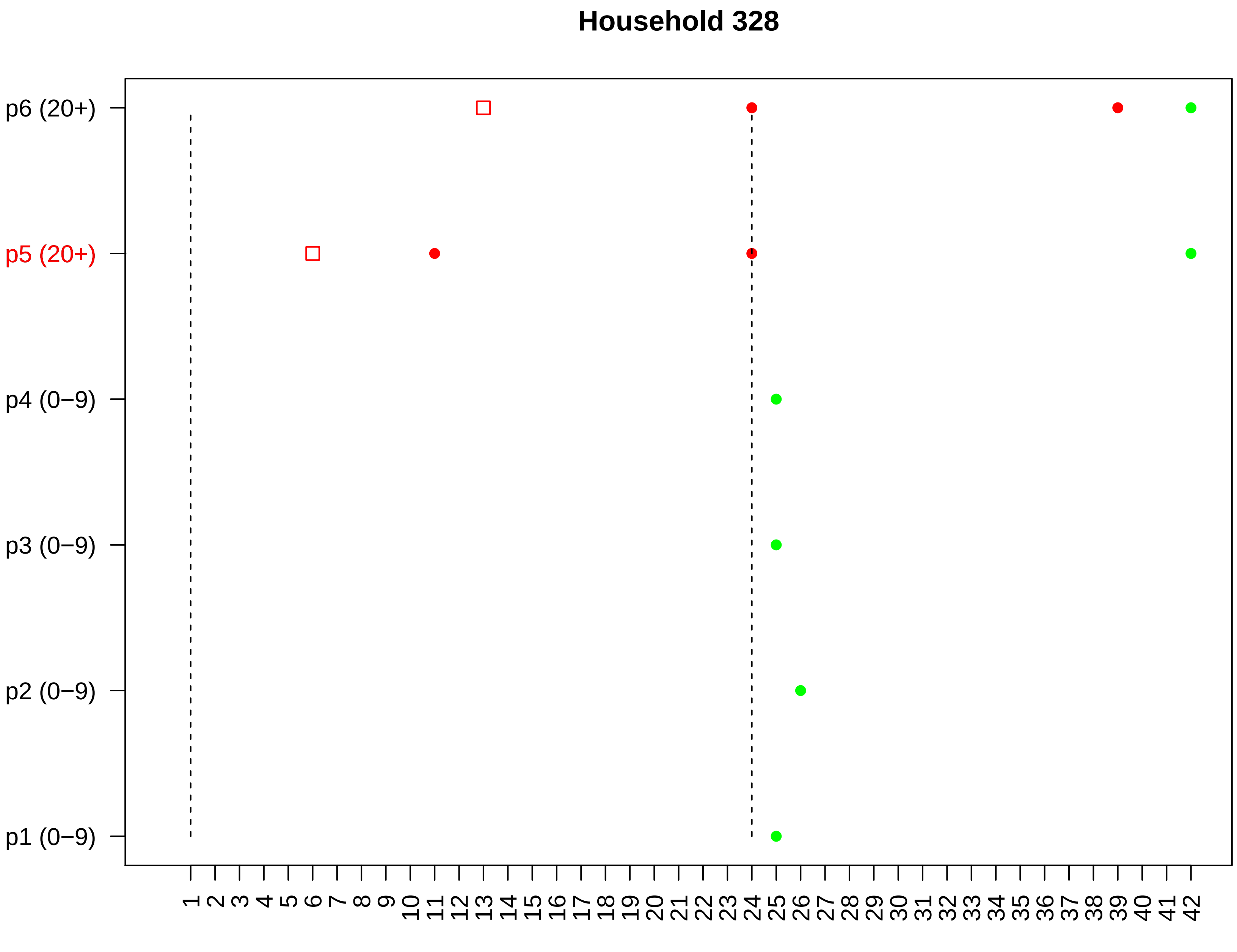

Household 328
p6 (20+)
p5 (20+)
p4 (0−9)
p3 (0−9)
p2 (0−9)
p1 (0−9)
1 2 3 4 5 6 7 8 9 10 11 12 13 14 15 16 17 18 19 20 21 22 23 24 25 26 27 28 29 30 31 32 33 34 35 36 37 38 39 40 41 42
day

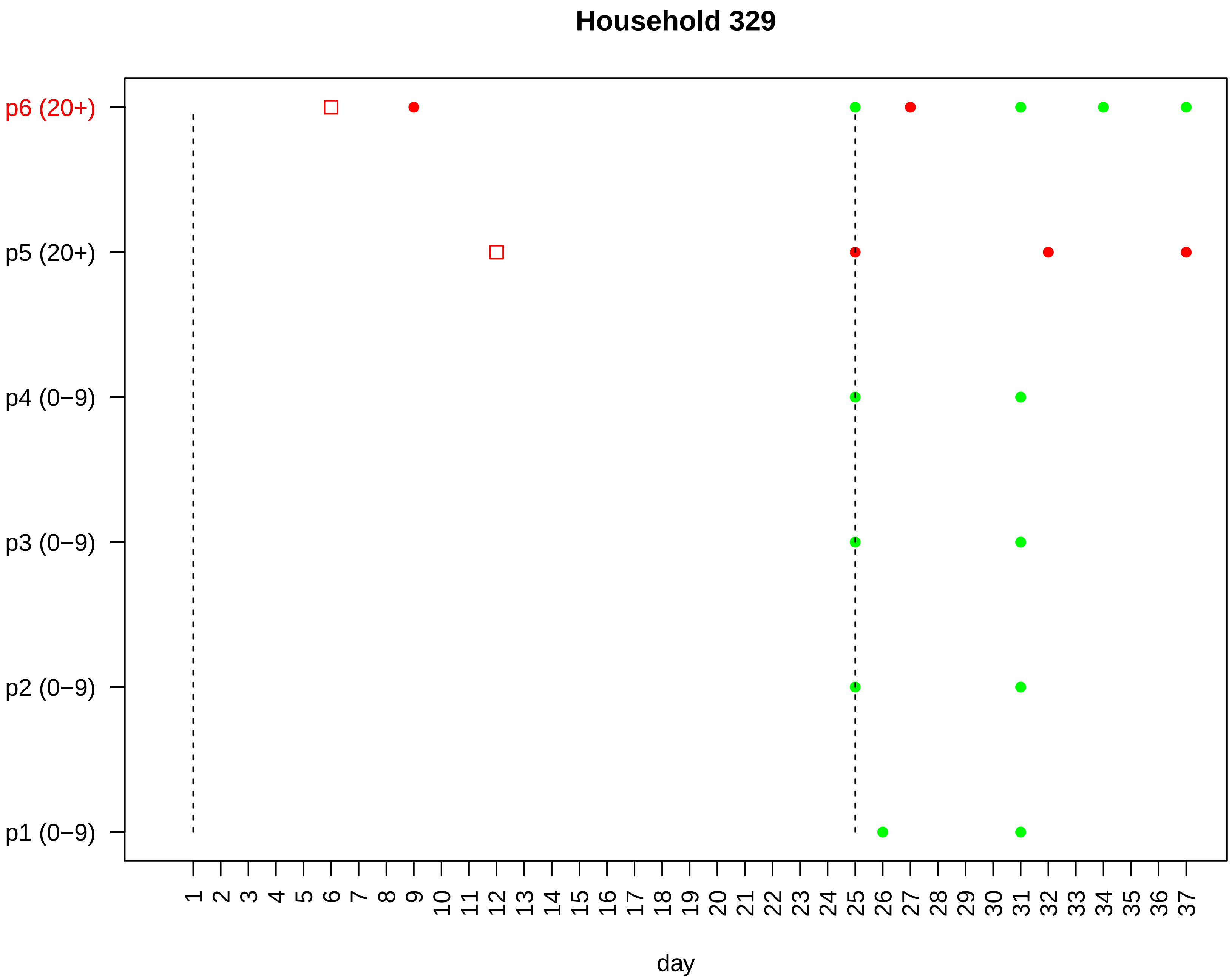

Household 329
p6 (20+)
p5 (20+)
p4 (0−9)
p3 (0−9)
p2 (0−9)
p1 (0−9)
1 2 3 4 5 6 7 8 9 10 11 12 13 14 15 16 17 18 19 20 21 22 23 24 25 26 27 28 29 30 31 32 33 34 35 36 37
day

Household 330

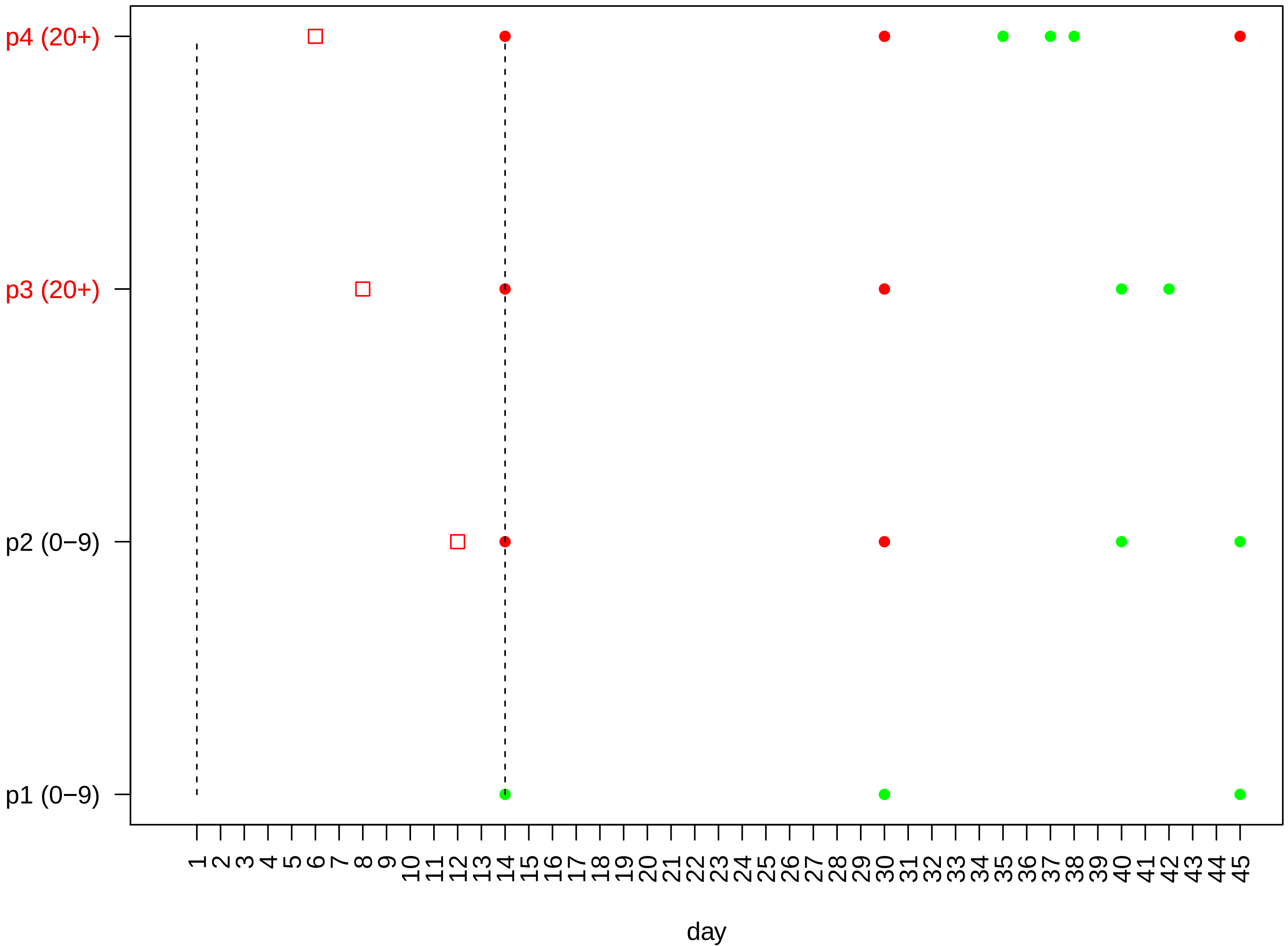

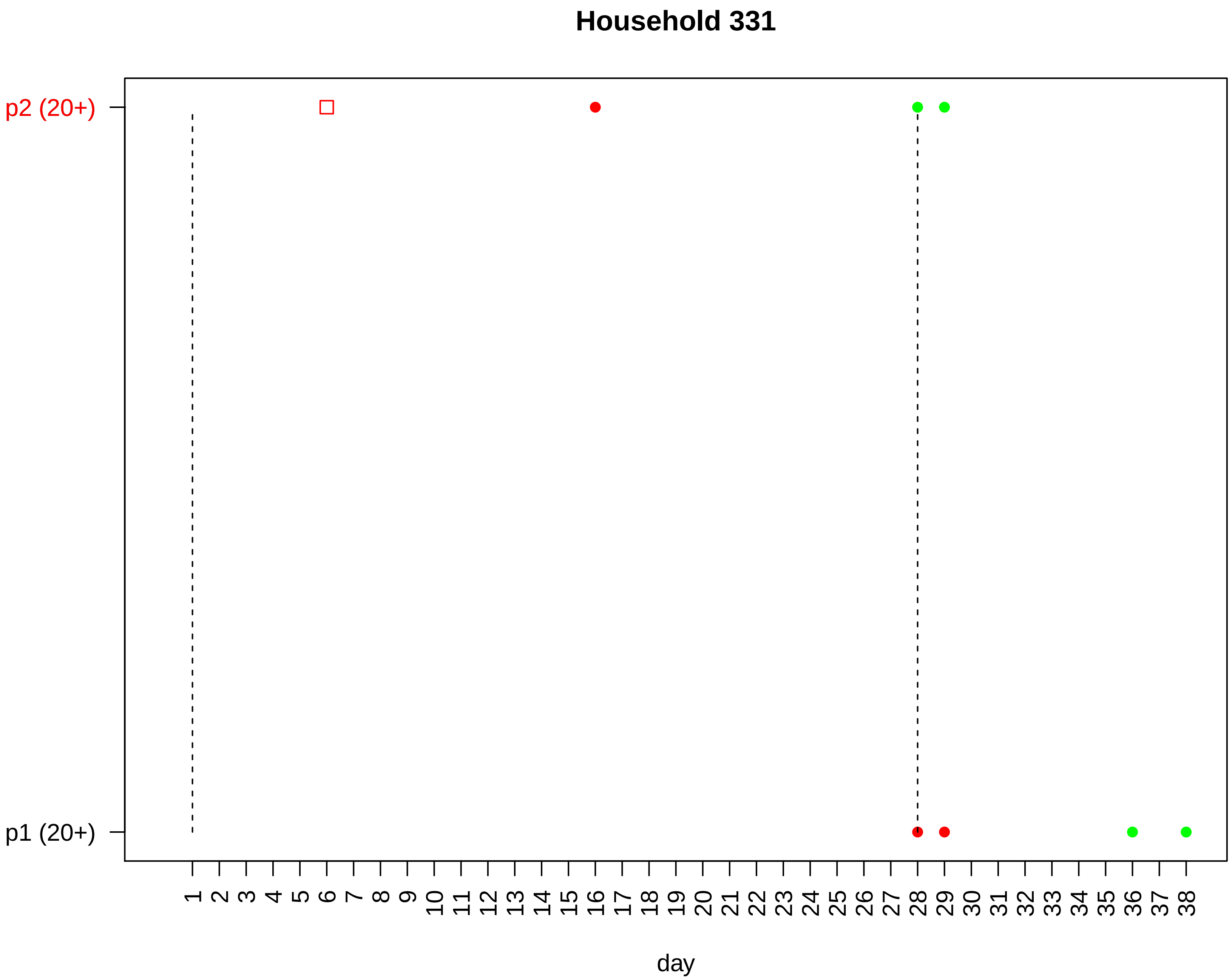

Household 331
p2 (20+)
p1 (20+)
1 2 3 4 5 6 7 8 9 10 11 12 13 14 15 16 17 18 19 20 21 22 23 24 25 26 27 28 29 30 31 32 33 34 35 36 37 38
day

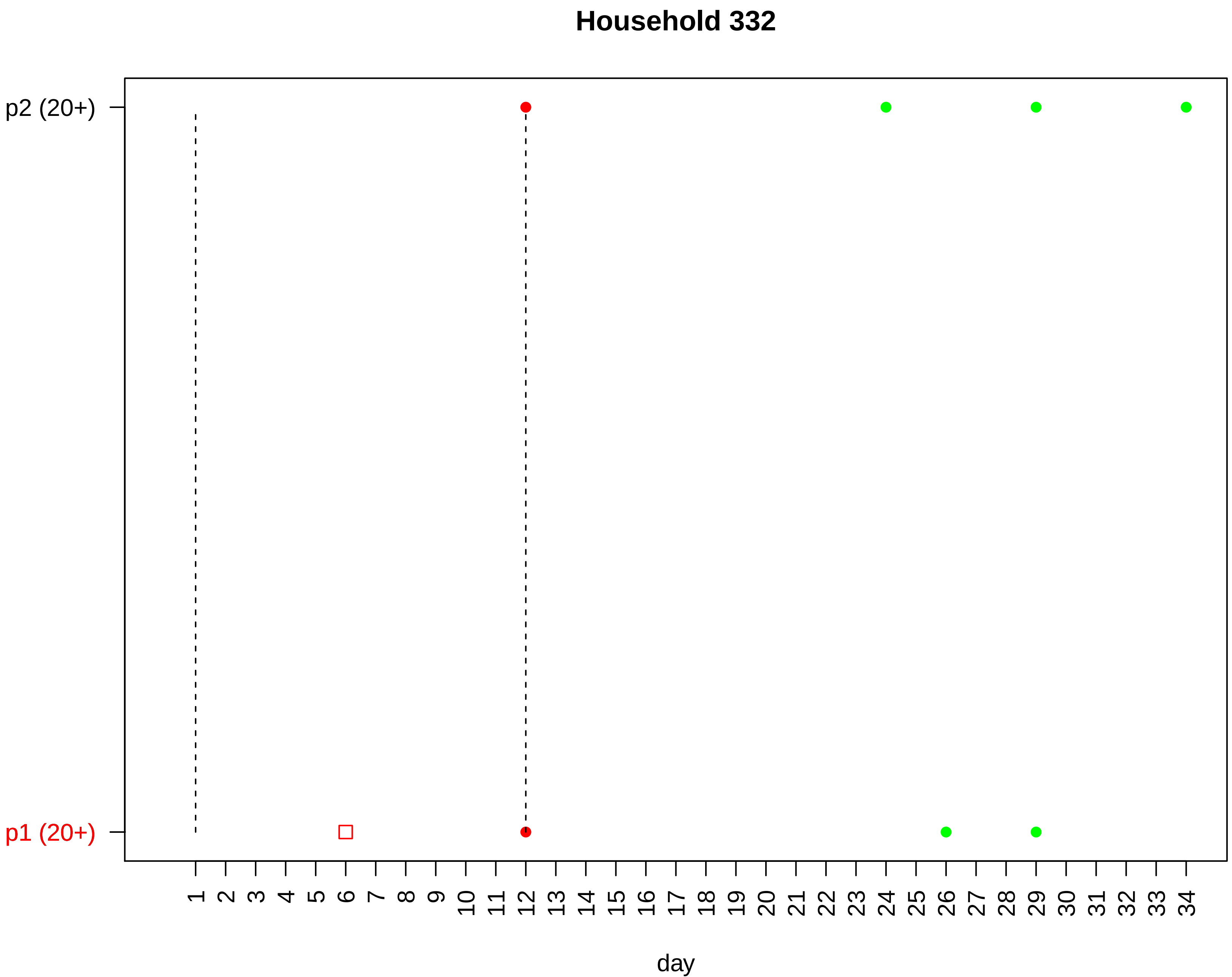
Household 332
p2 (20+)
p1 (20+)
1 2 3 4 5 6 7 8 9 10 11 12 13 14 15 16 17 18 19 20 21 22 23 24 25 26 27 28 29 30 31 32 33 34
day

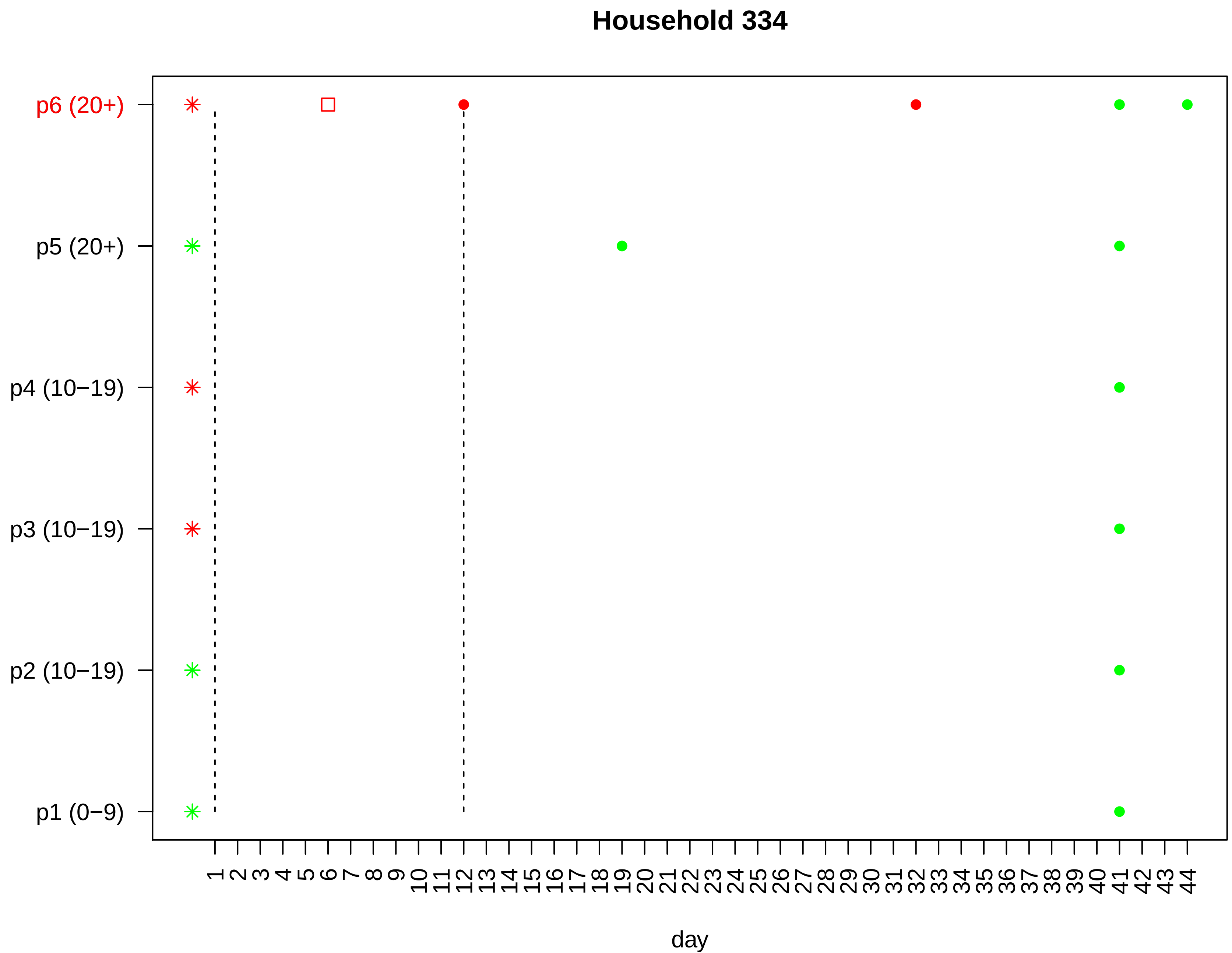

Household 334
p6 (20+)
p5 (20+)
p4 (10−19)
p3 (10−19)
p2 (10−19)
p1 (0−9)
1 2 3 4 5 6 7 8 9 10 11 12 13 14 15 16 17 18 19 20 21 22 23 24 25 26 27 28 29 30 31 32 33 34 35 36 37 38 39 40 41 42 43 44
day

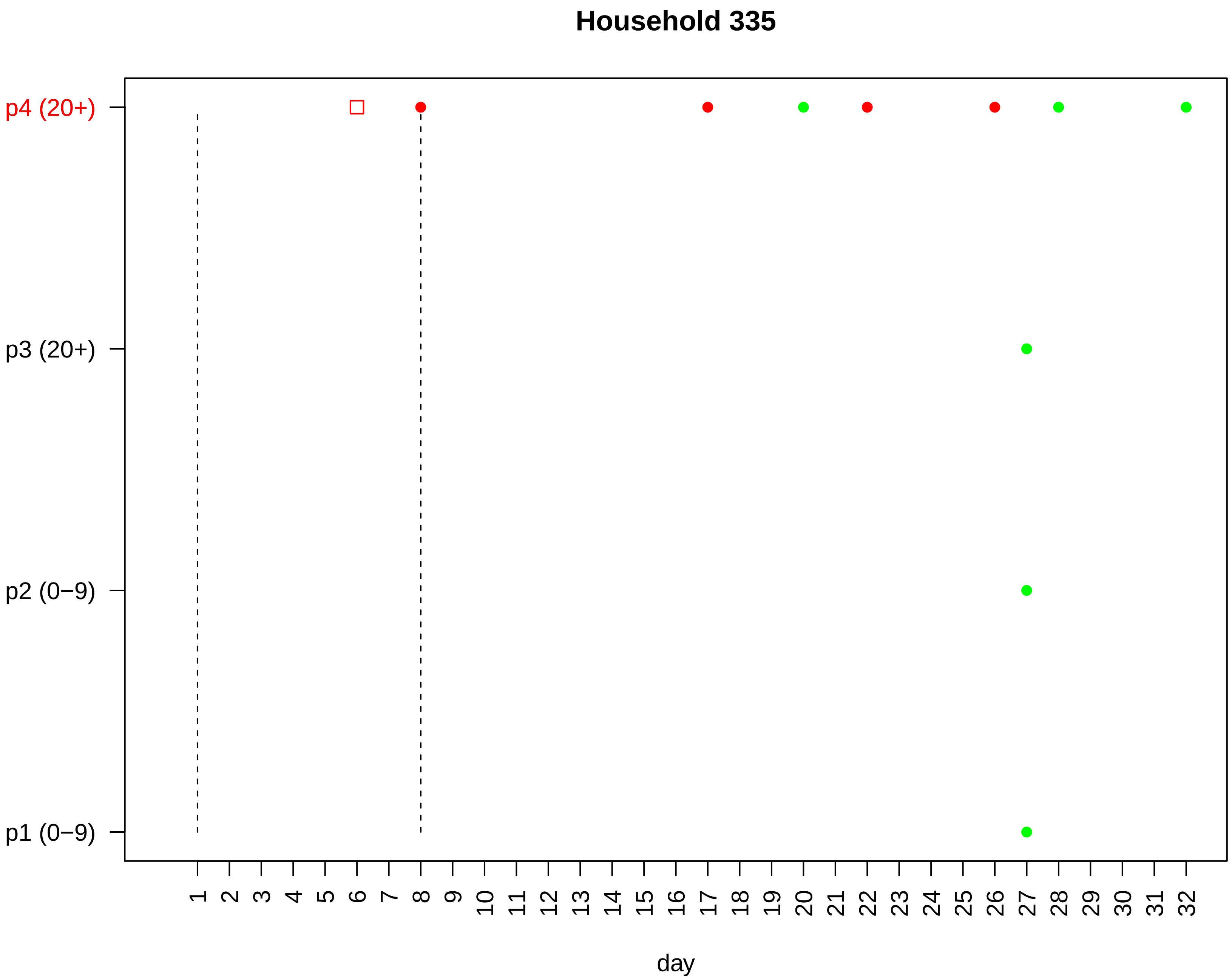
Household 335
p4 (20+)
p3 (20+)
p2 (0−9)
p1 (0−9)
1 2 3 4 5 6 7 8 9 10 11 12 13 14 15 16 17 18 19 20 21 22 23 24 25 26 27 28 29 30 31 32
day

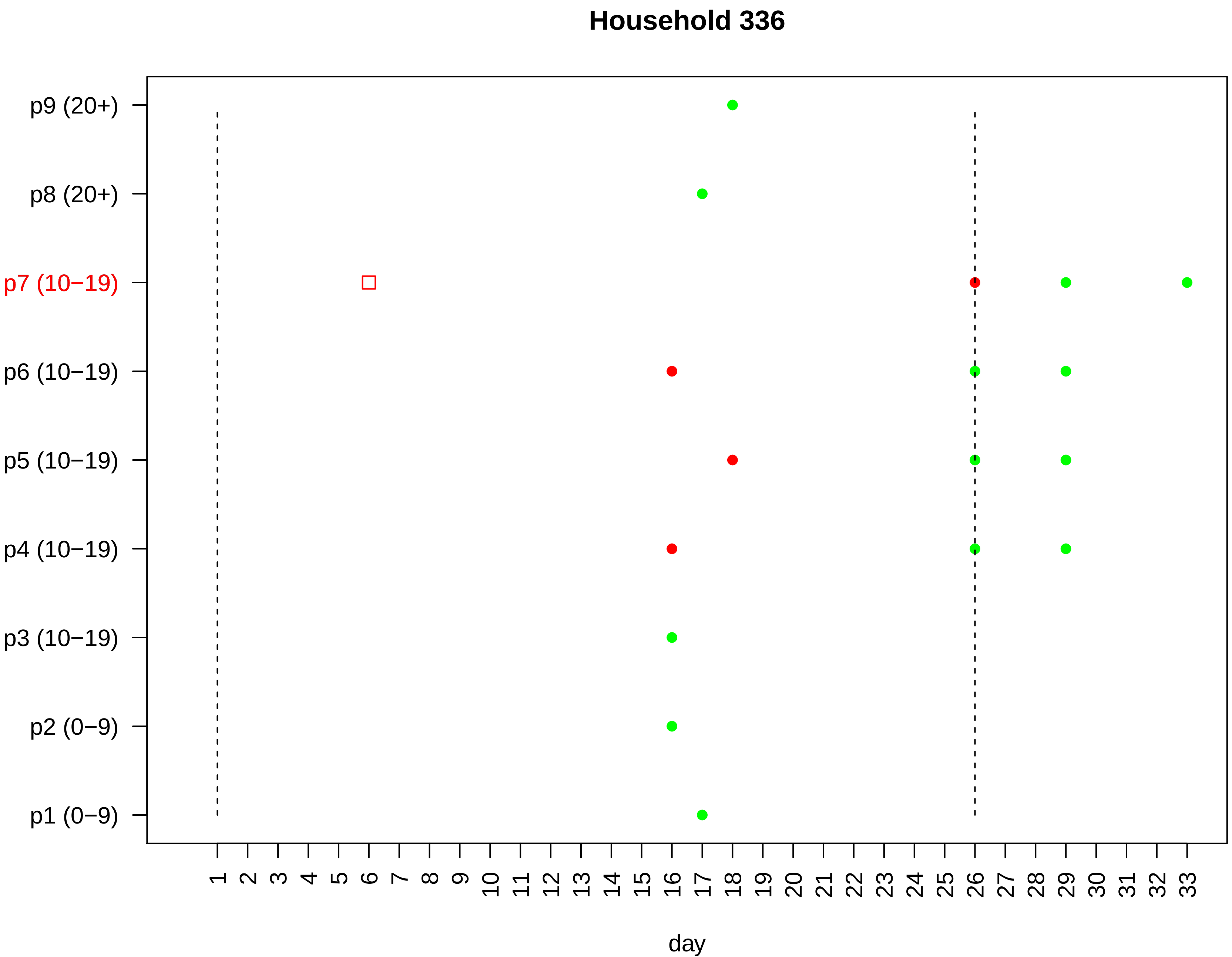

Household 336
p9 (20+)
p8 (20+)
p7 (10−19)
p6 (10−19)
p5 (10−19)
p4 (10−19)
p3 (10−19)
p2 (0−9)
p1 (0−9)
1 2 3 4 5 6 7 8 9 10 11 12 13 14 15 16 17 18 19 20 21 22 23 24 25 26 27 28 29 30 31 32 33
day

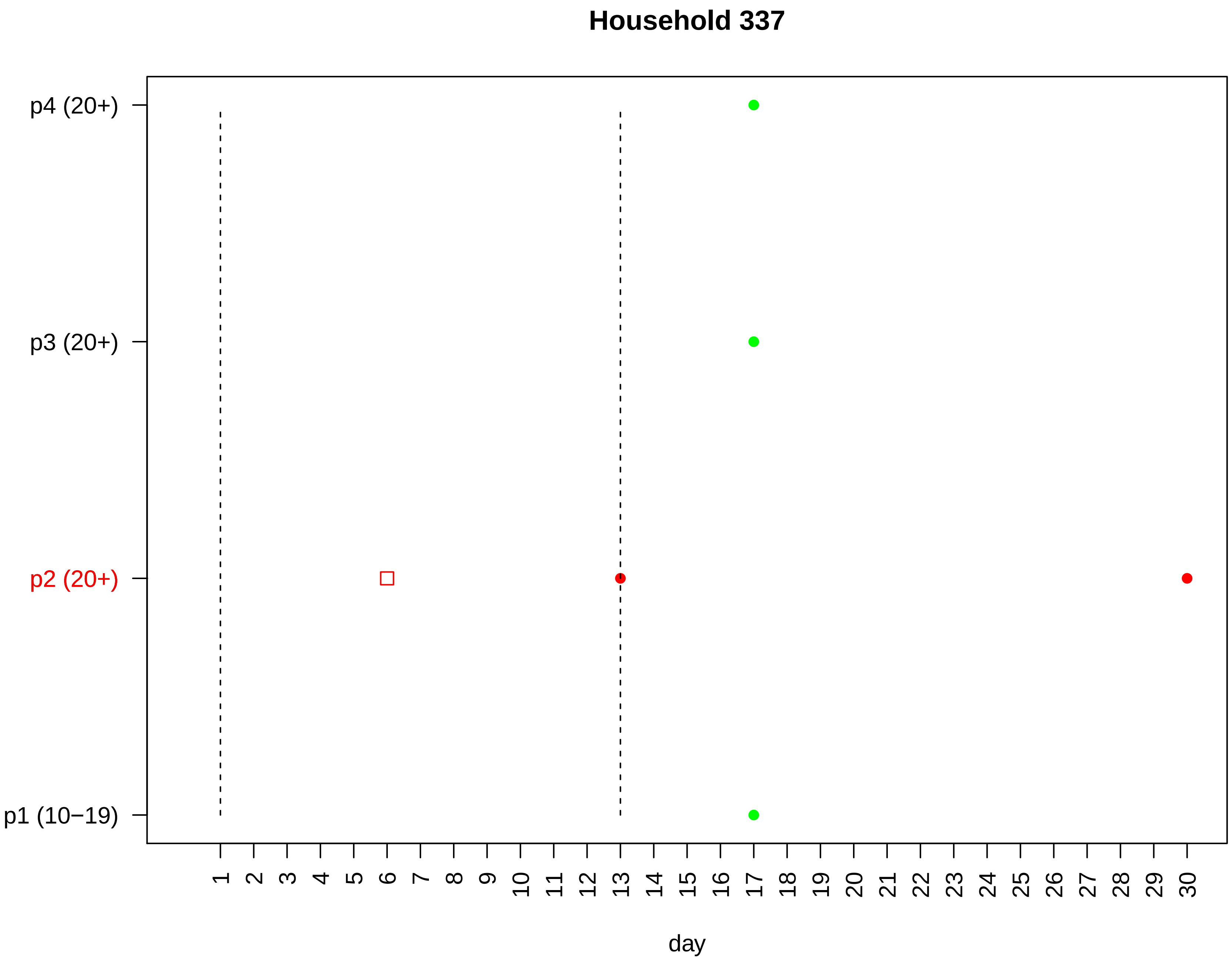
Household 337
p4 (20+)
p3 (20+)
p2 (20+)
p1 (10−19)
1 2 3 4 5 6 7 8 9 10 11 12 13 14 15 16 17 18 19 20 21 22 23 24 25 26 27 28 29 30
day

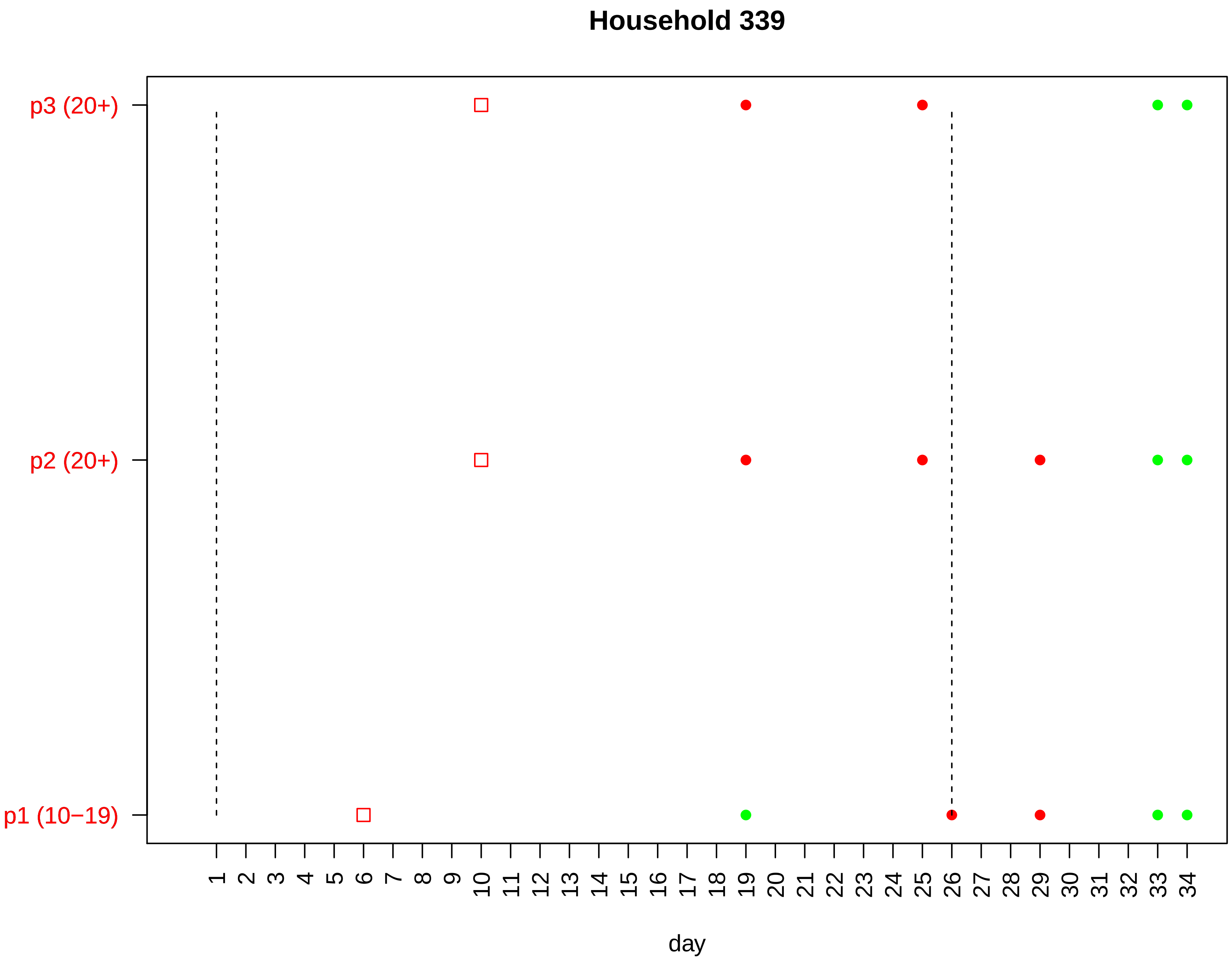

Household 339
p3 (20+)
p2 (20+)
p1 (10−19)
1
2
3
4
5
6
7
8
9
10
11
12
13
14
15
16
17
18
19
20
21
22
23
24
25
26
27
28
29
30
31
32
33
34
day

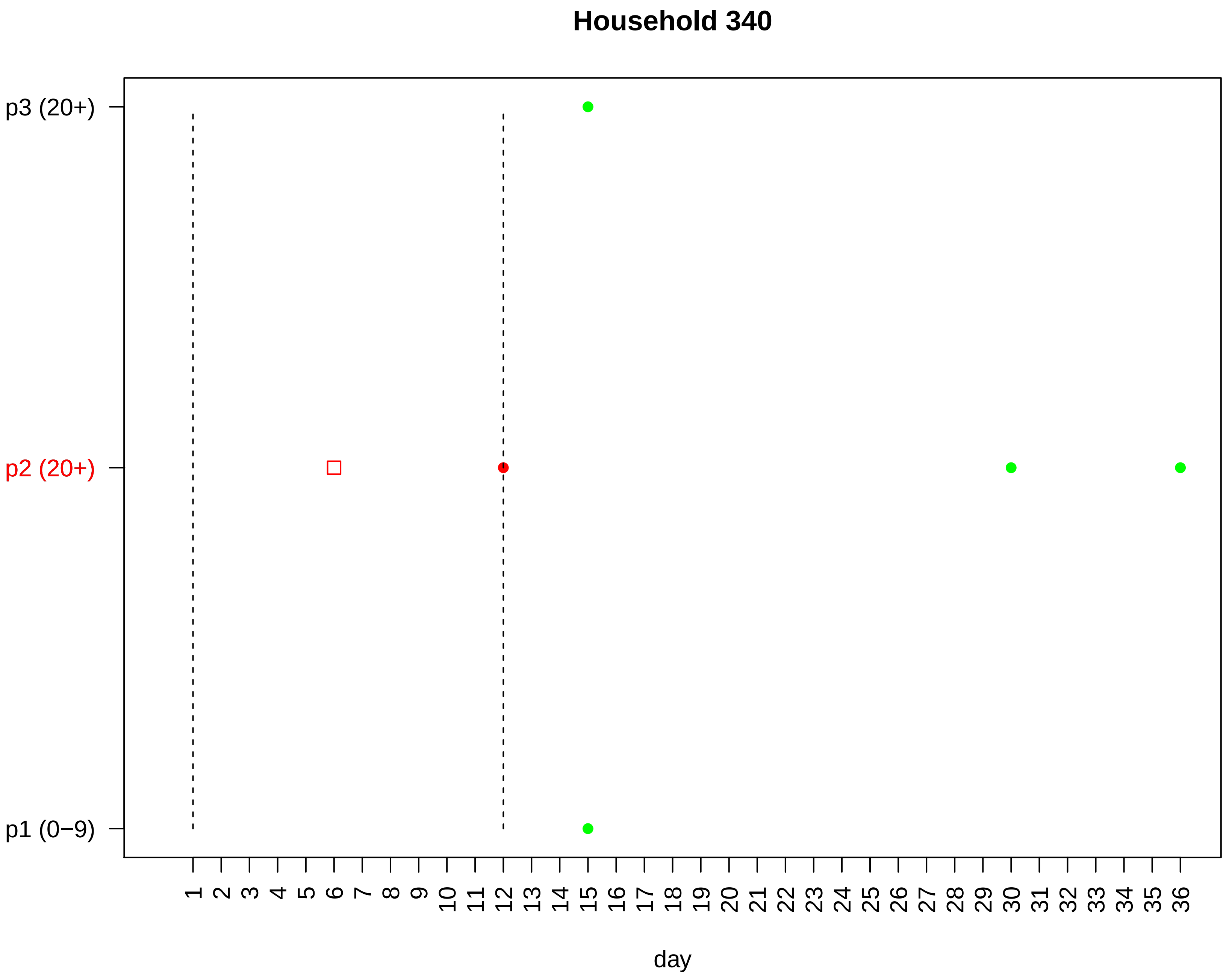
Household 340
p3 (20+)
p2 (20+)
p1 (0−9)
1
2
3
4
5
6
7
8
9
10
11
12
13
14
15
16
17
18
19
20
21
22
23
24
25
26
27
28
29
30
31
32
33
34
35
36
day

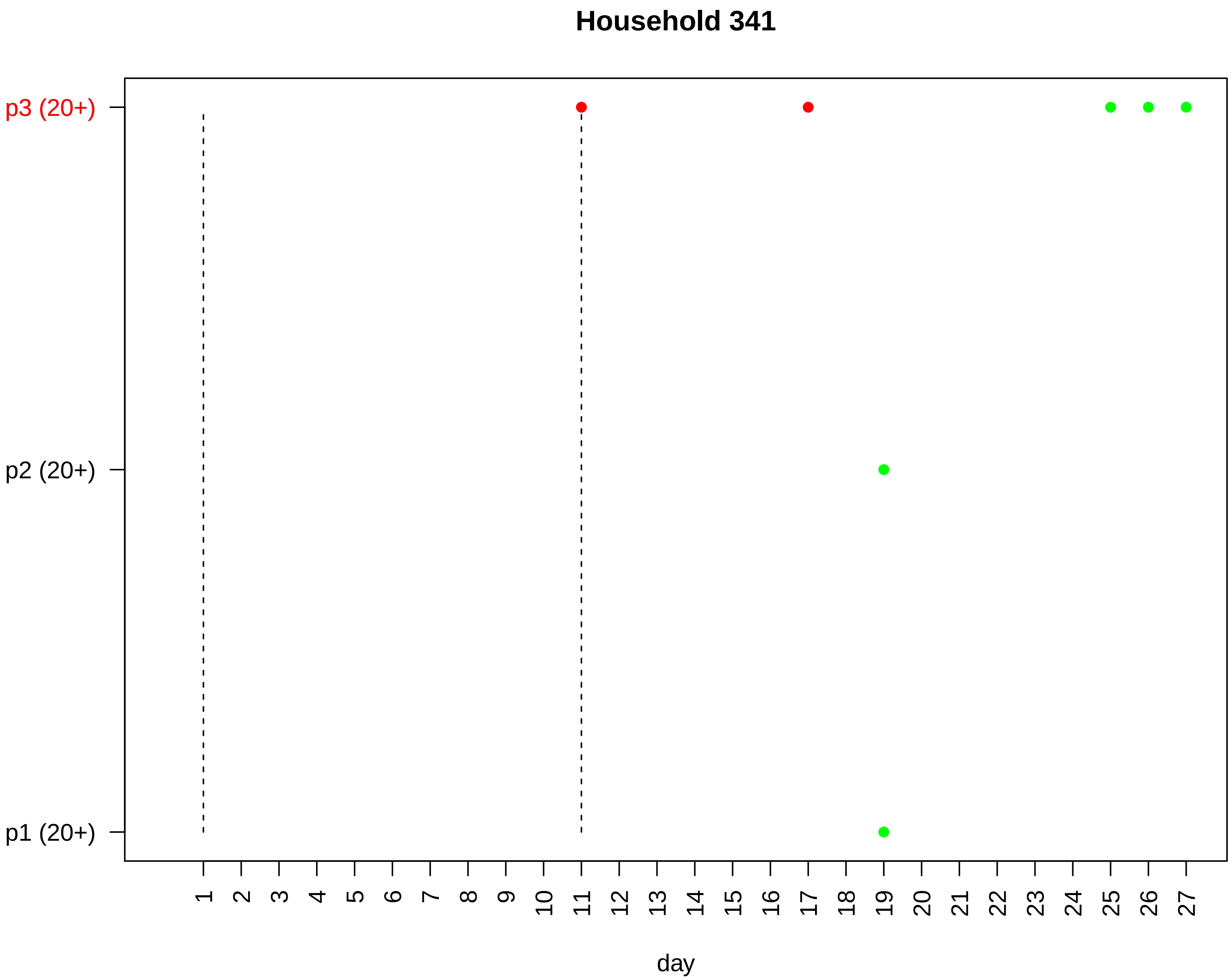

Household 341
p3 (20+)
p2 (20+)
p1 (20+)
1 2 3 4 5 6 7 8 9 10 11 12 13 14 15 16 17 18 19 20 21 22 23 24 25 26 27
day

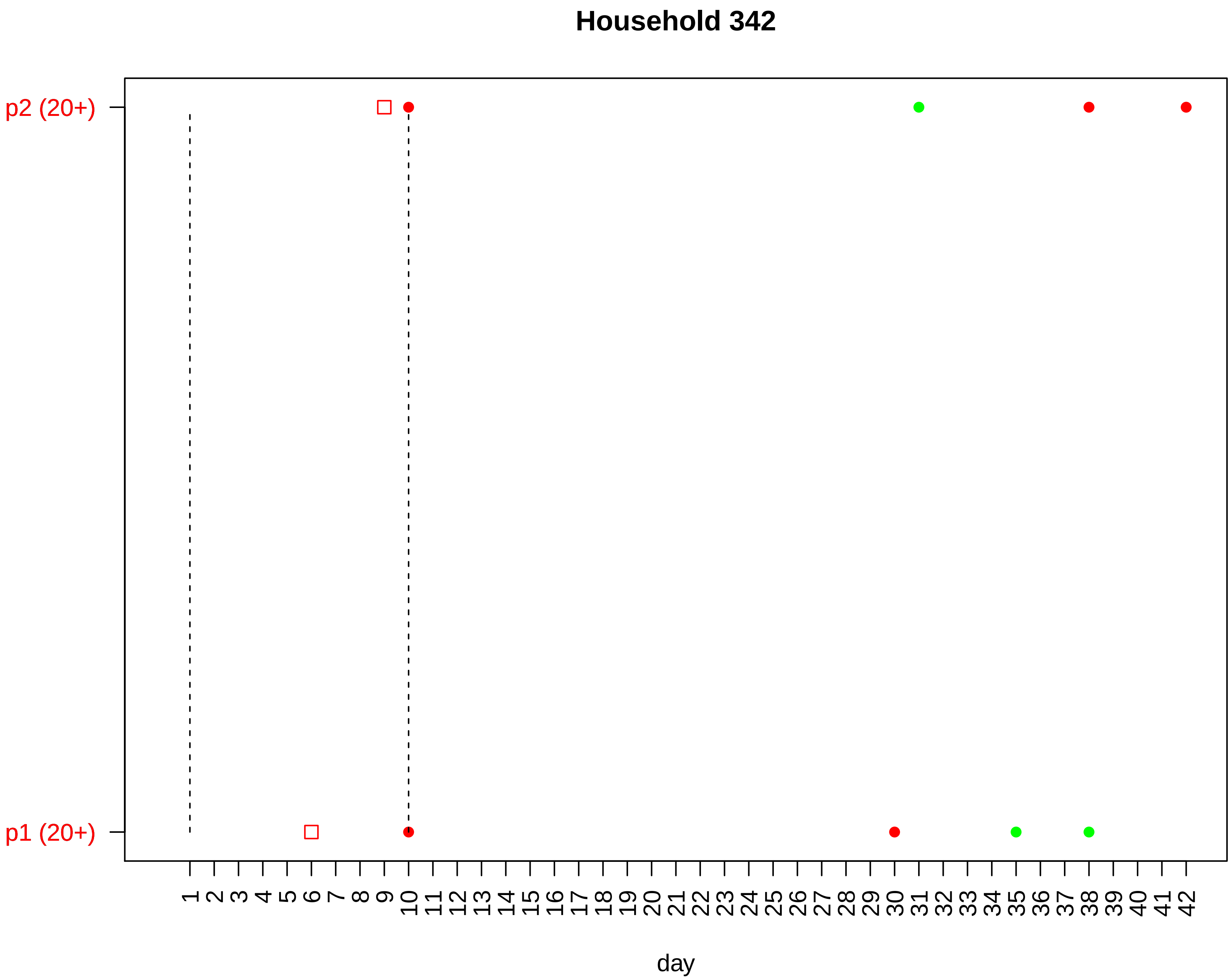

Household 342
p2 (20+)
p1 (20+)
1 2 3 4 5 6 7 8 9 10 11 12 13 14 15 16 17 18 19 20 21 22 23 24 25 26 27 28 29 30 31 32 33 34 35 36 37 38 39 40 41 42
day

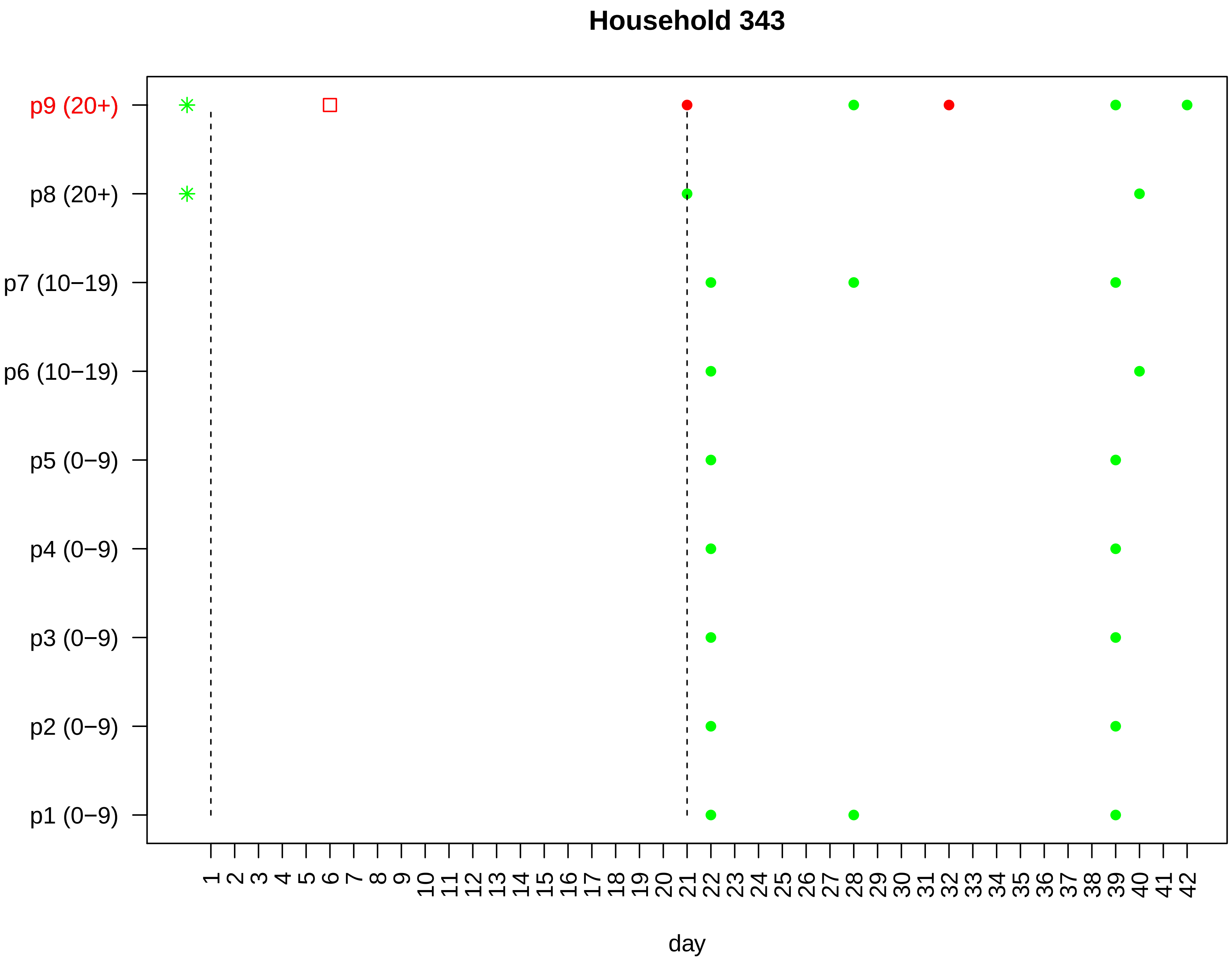

Household 343
p9 (20+)
p8 (20+)
p7 (10−19)
p6 (10−19)
p5 (0−9)
p4 (0−9)
p3 (0−9)
p2 (0−9)
p1 (0−9)
1 2 3 4 5 6 7 8 9 10 11 12 13 14 15 16 17 18 19 20 21 22 23 24 25 26 27 28 29 30 31 32 33 34 35 36 37 38 39 40 41 42
day

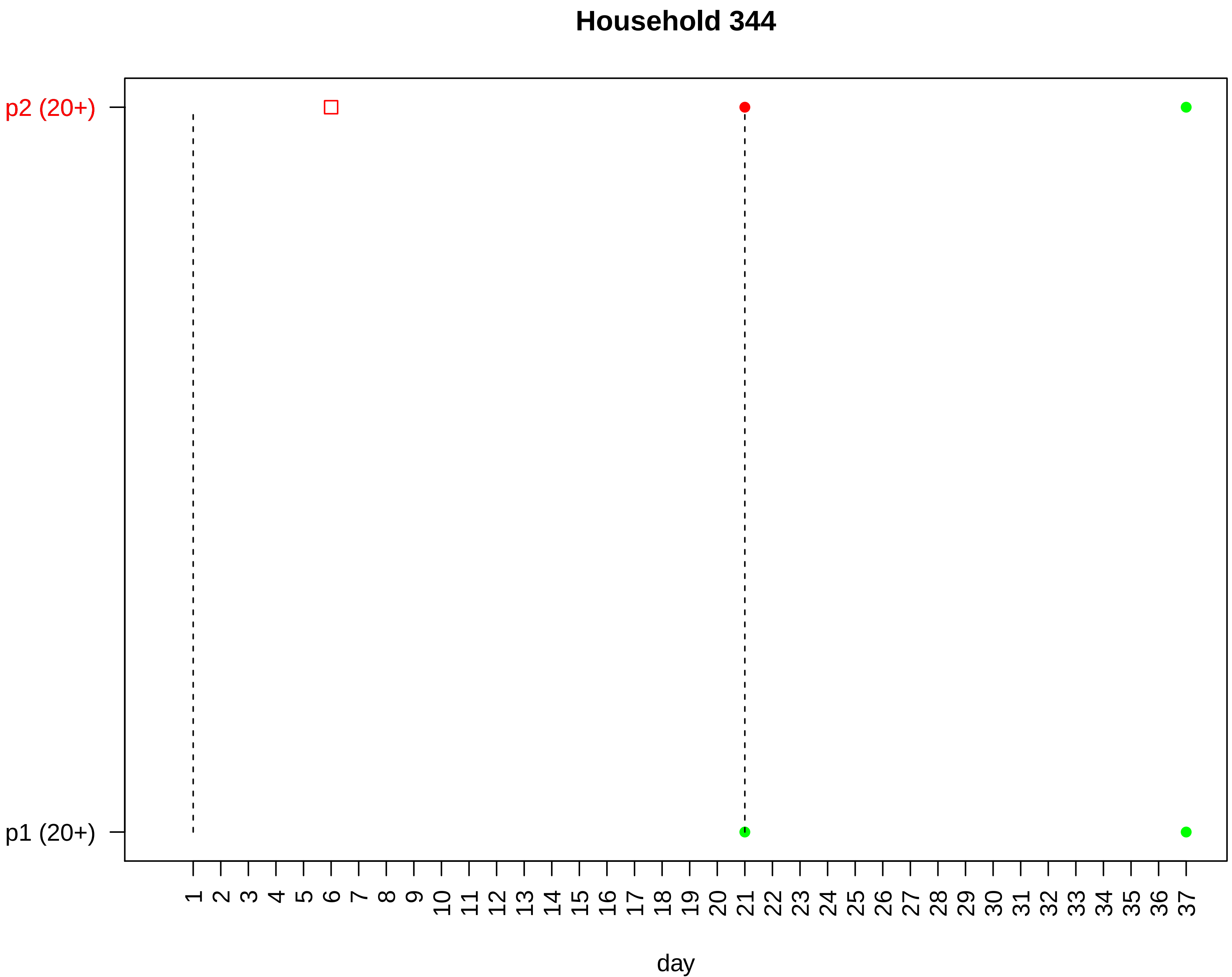
Household 344
p2 (20+)
p1 (20+)
1 2 3 4 5 6 7 8 9 10 11 12 13 14 15 16 17 18 19 20 21 22 23 24 25 26 27 28 29 30 31 32 33 34 35 36 37
day

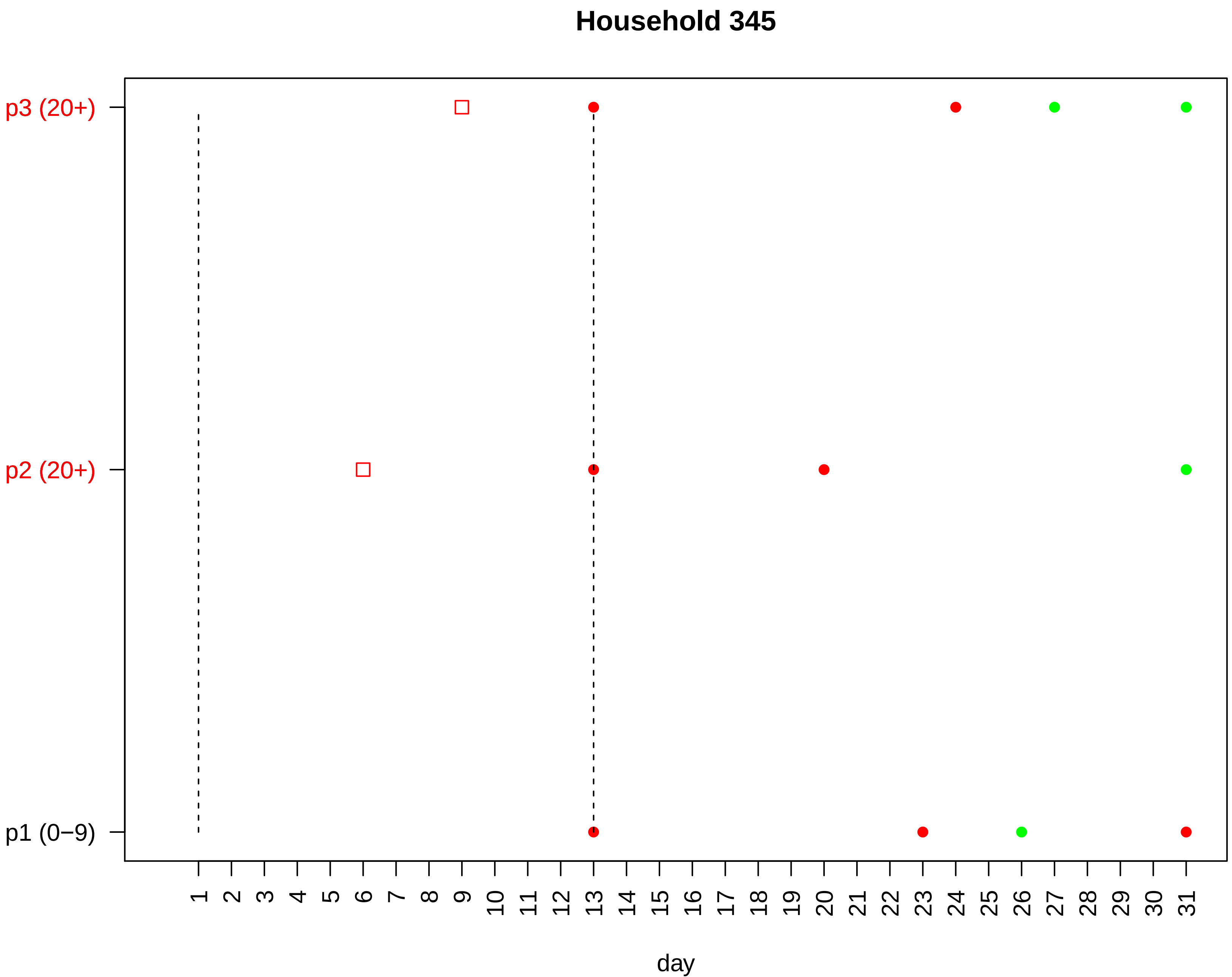
Household 345
p3 (20+)
p2 (20+)
p1 (0−9)
1 2 3 4 5 6 7 8 9 10 11 12 13 14 15 16 17 18 19 20 21 22 23 24 25 26 27 28 29 30 31
day

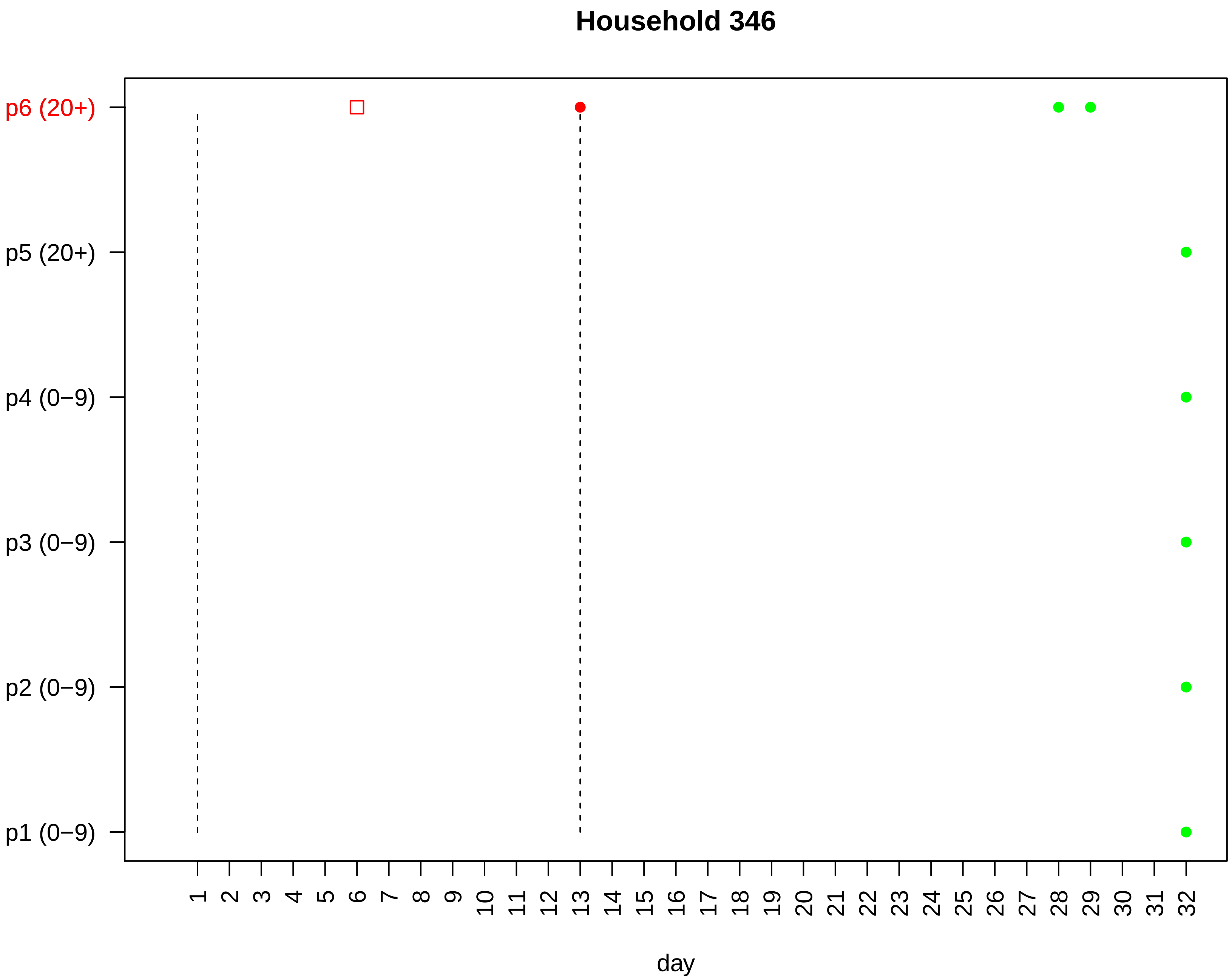

Household 346
p6 (20+)
p5 (20+)
p4 (0−9)
p3 (0−9)
p2 (0−9)
p1 (0−9)
1 2 3 4 5 6 7 8 9 10 11 12 13 14 15 16 17 18 19 20 21 22 23 24 25 26 27 28 29 30 31 32
day

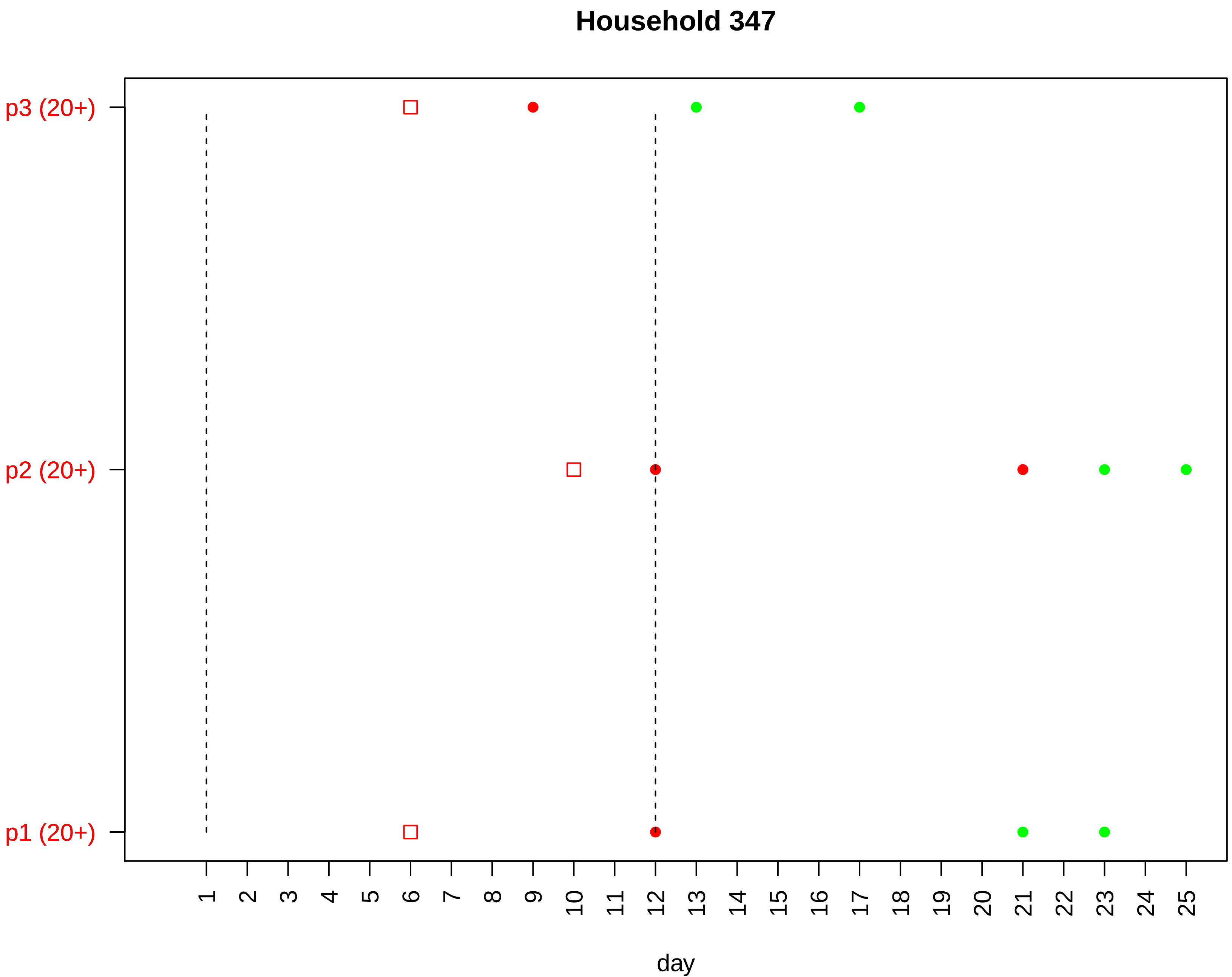

Household 347
p3 (20+)
p2 (20+)
p1 (20+)
1
2
3
4
5
6
7
8
9
10
11
12
13
14
15
16
17
18
19
20
21
22
23
24
25
day

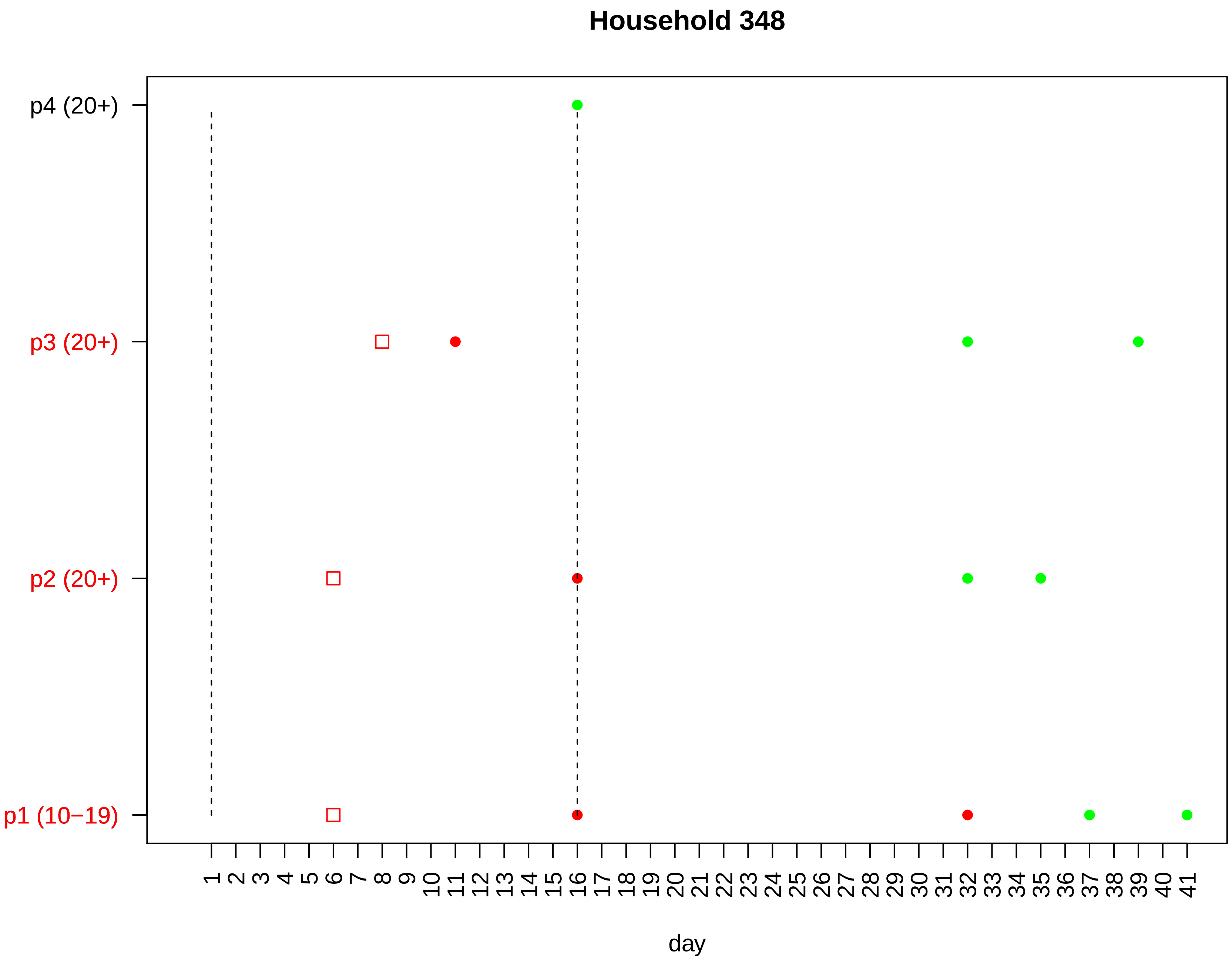

Household 348
p4 (20+)
p3 (20+)
p2 (20+)
p1 (10−19)
1 2 3 4 5 6 7 8 9 10 11 12 13 14 15 16 17 18 19 20 21 22 23 24 25 26 27 28 29 30 31 32 33 34 35 36 37 38 39 40 41
day

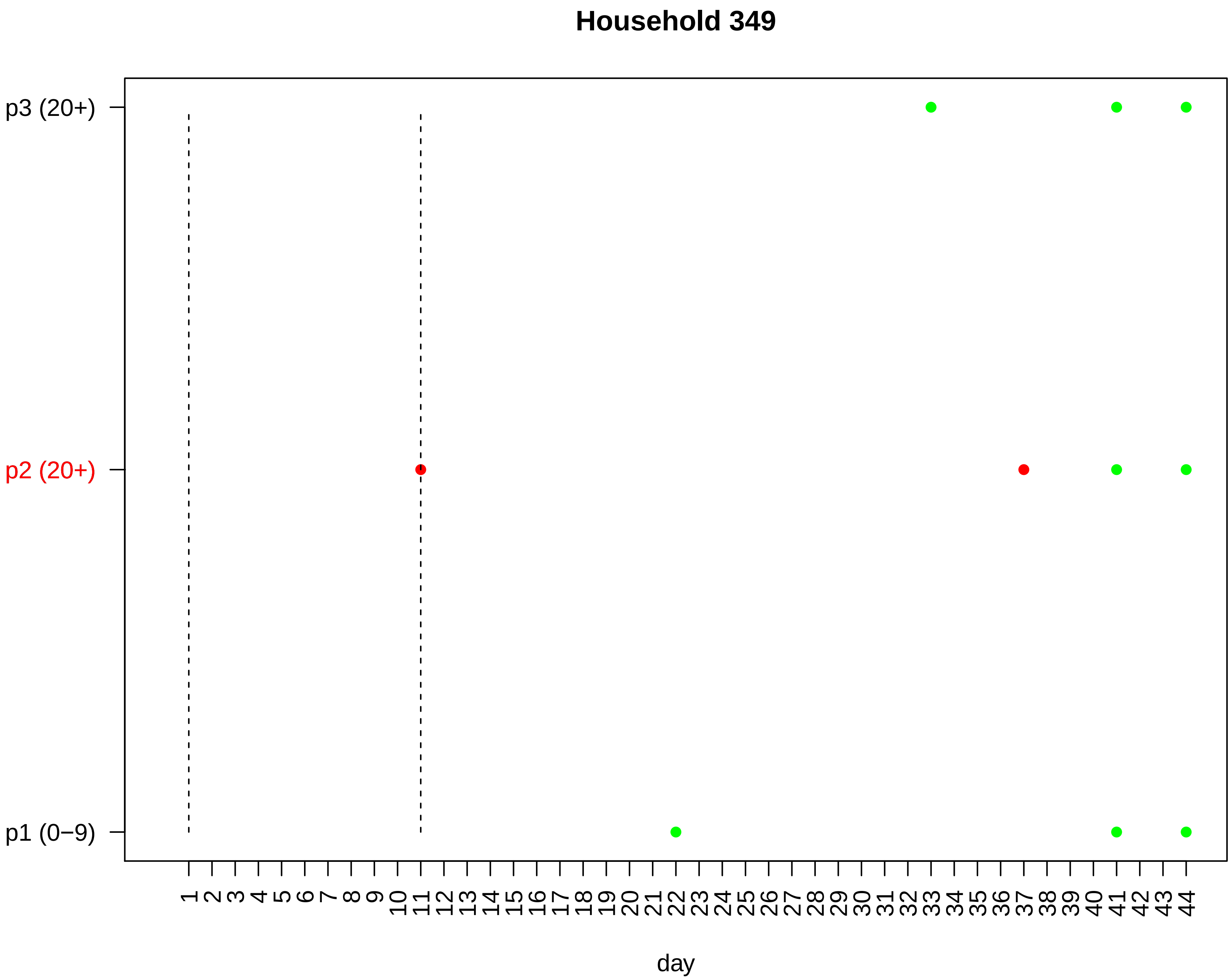

Household 349
p3 (20+)
p2 (20+)
p1 (0−9)
1 2 3 4 5 6 7 8 9 10 11 12 13 14 15 16 17 18 19 20 21 22 23 24 25 26 27 28 29 30 31 32 33 34 35 36 37 38 39 40 41 42 43 44
day

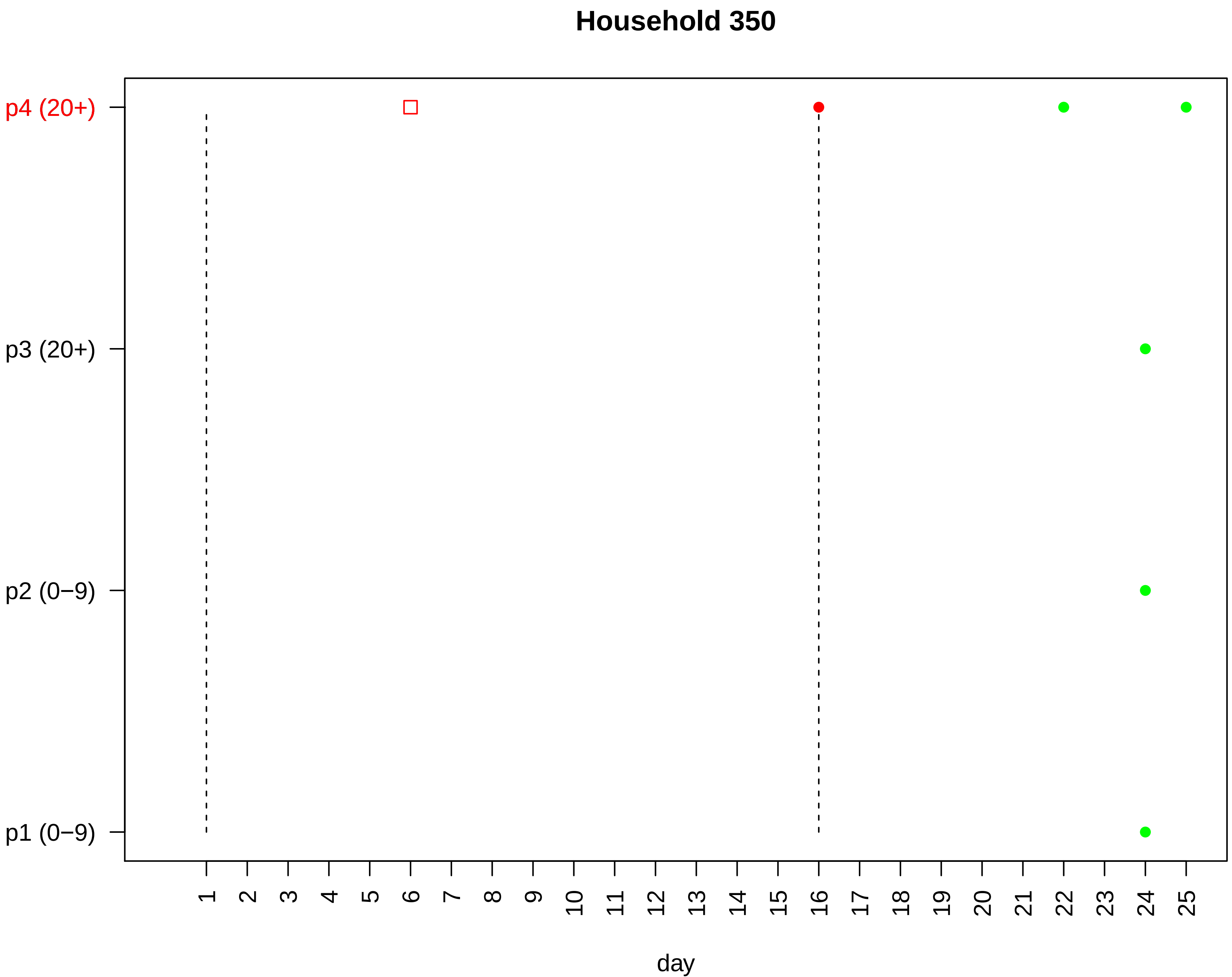
Household 350
p4 (20+)
p3 (20+)
p2 (0−9)
p1 (0−9)
1
2
3
4
5
6
7
8
9
10
11
12
13
14
15
16
17
18
19
20
21
22
23
24
25
day

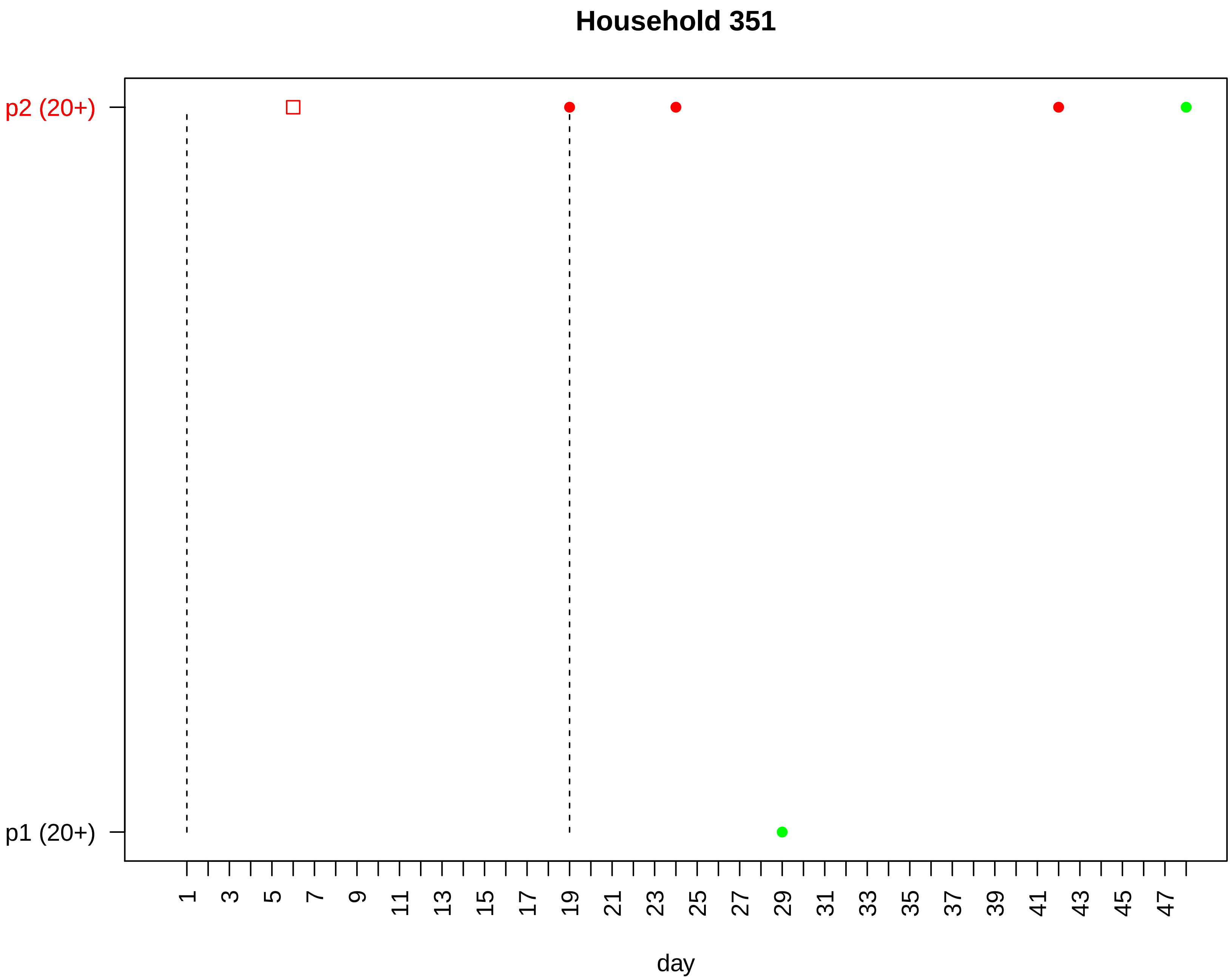

Household 351
p2 (20+)
p1 (20+)
1 3 5 7 9 11 13 15 17 19 21 23 25 27 29 31 33 35 37 39 41 43 45 47
day

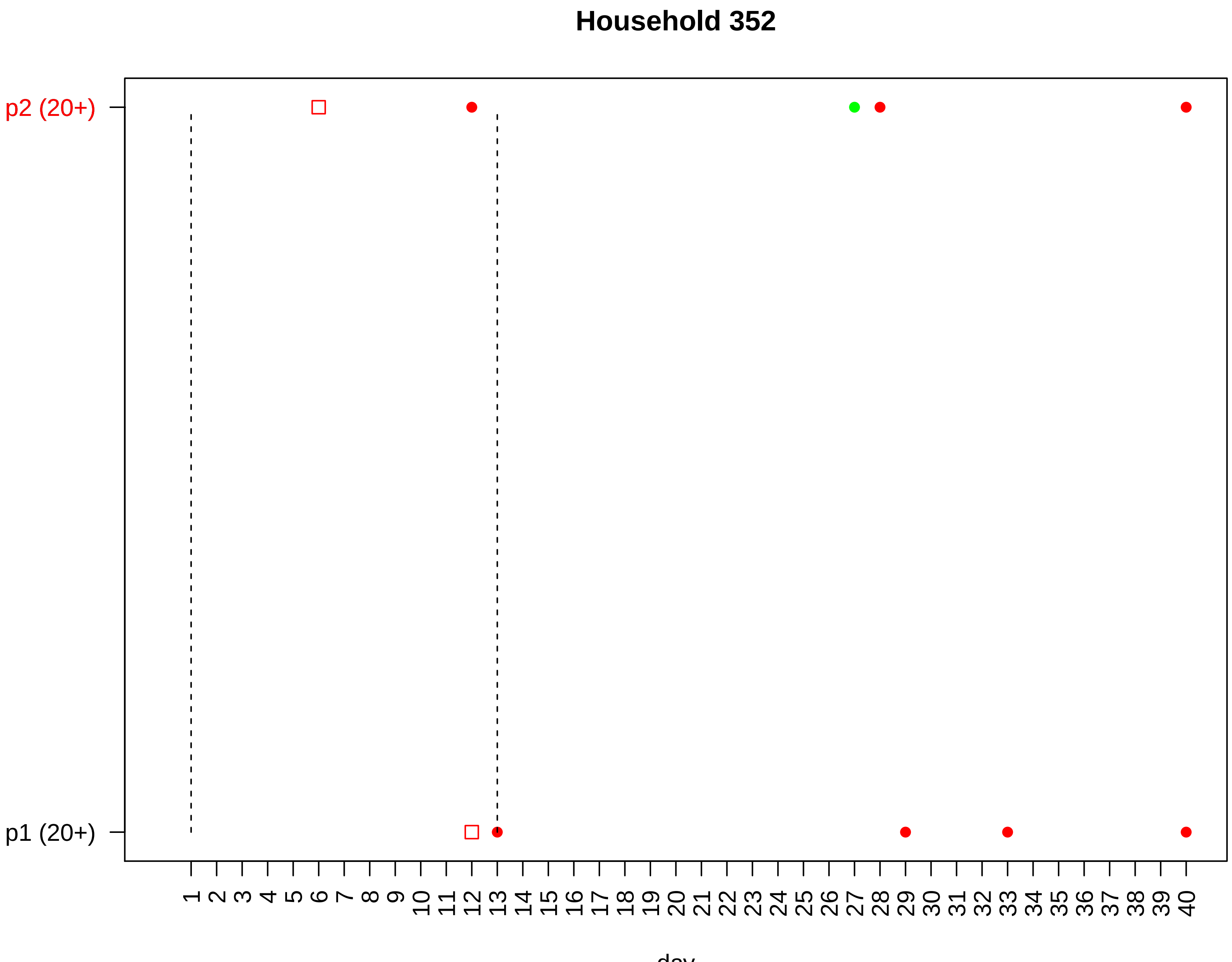
Household 352
p2 (20+)
p1 (20+)
1 2 3 4 5 6 7 8 9 10 11 12 13 14 15 16 17 18 19 20 21 22 23 24 25 26 27 28 29 30 31 32 33 34 35 36 37 38 39 40
day

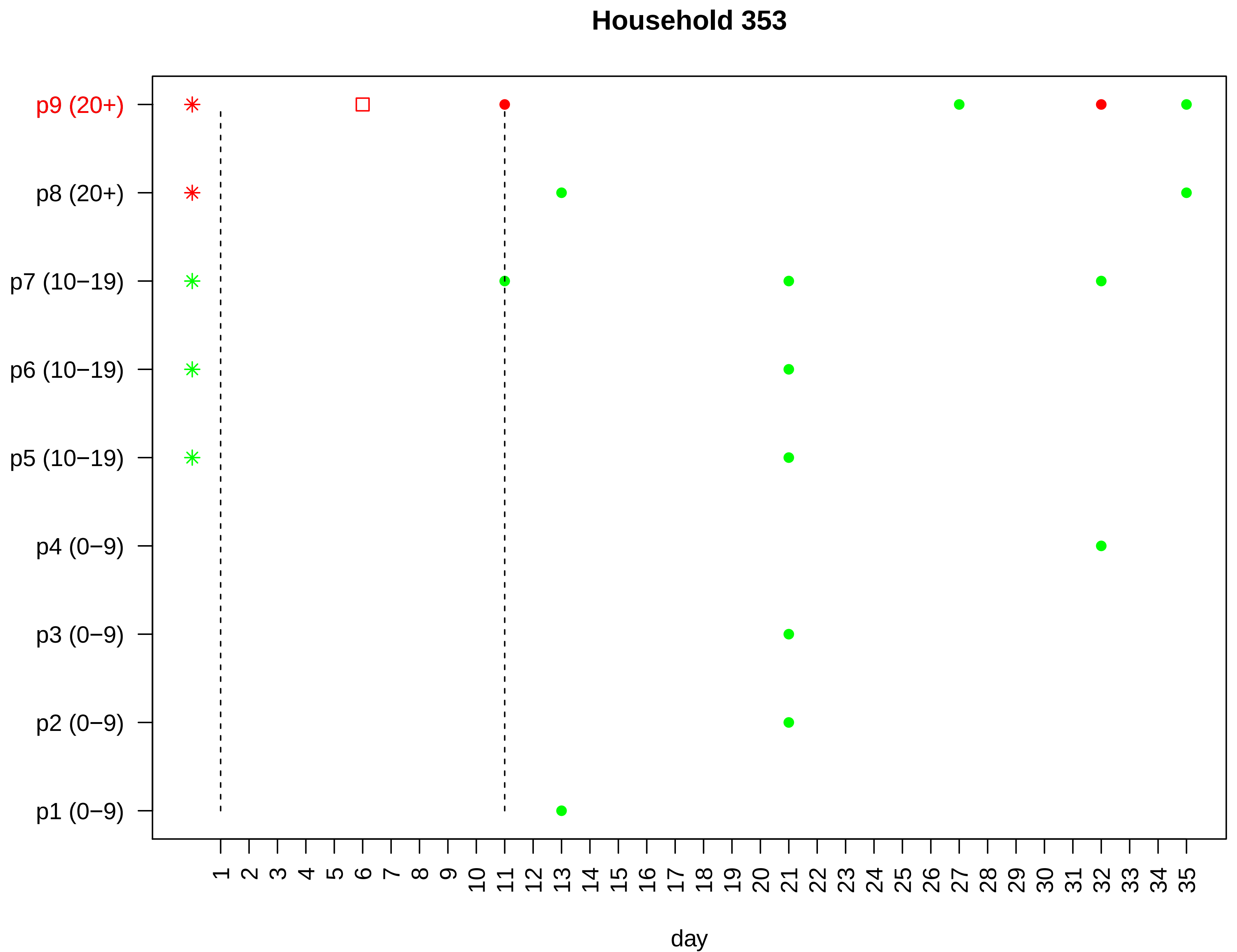

Household 353
p9 (20+)
p8 (20+)
p7 (10−19)
p6 (10−19)
p5 (10−19)
p4 (0−9)
p3 (0−9)
p2 (0−9)
p1 (0−9)
1 2 3 4 5 6 7 8 9 10 11 12 13 14 15 16 17 18 19 20 21 22 23 24 25 26 27 28 29 30 31 32 33 34 35
day

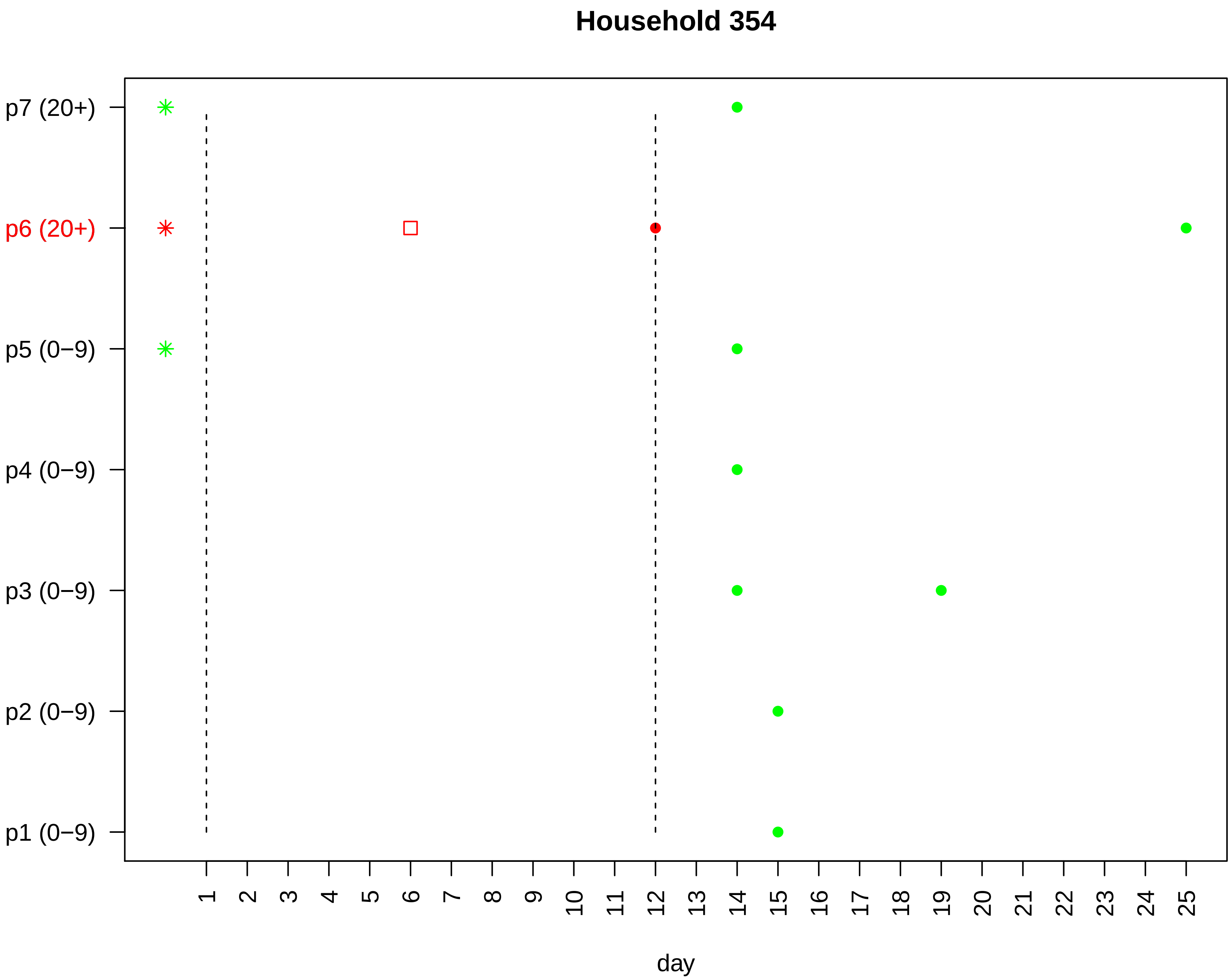
Household 354
p7 (20+)
p6 (20+)
p5 (0−9)
p4 (0−9)
p3 (0−9)
p2 (0−9)
p1 (0−9)
1 2 3 4 5 6 7 8 9 10 11 12 13 14 15 16 17 18 19 20 21 22 23 24 25
day

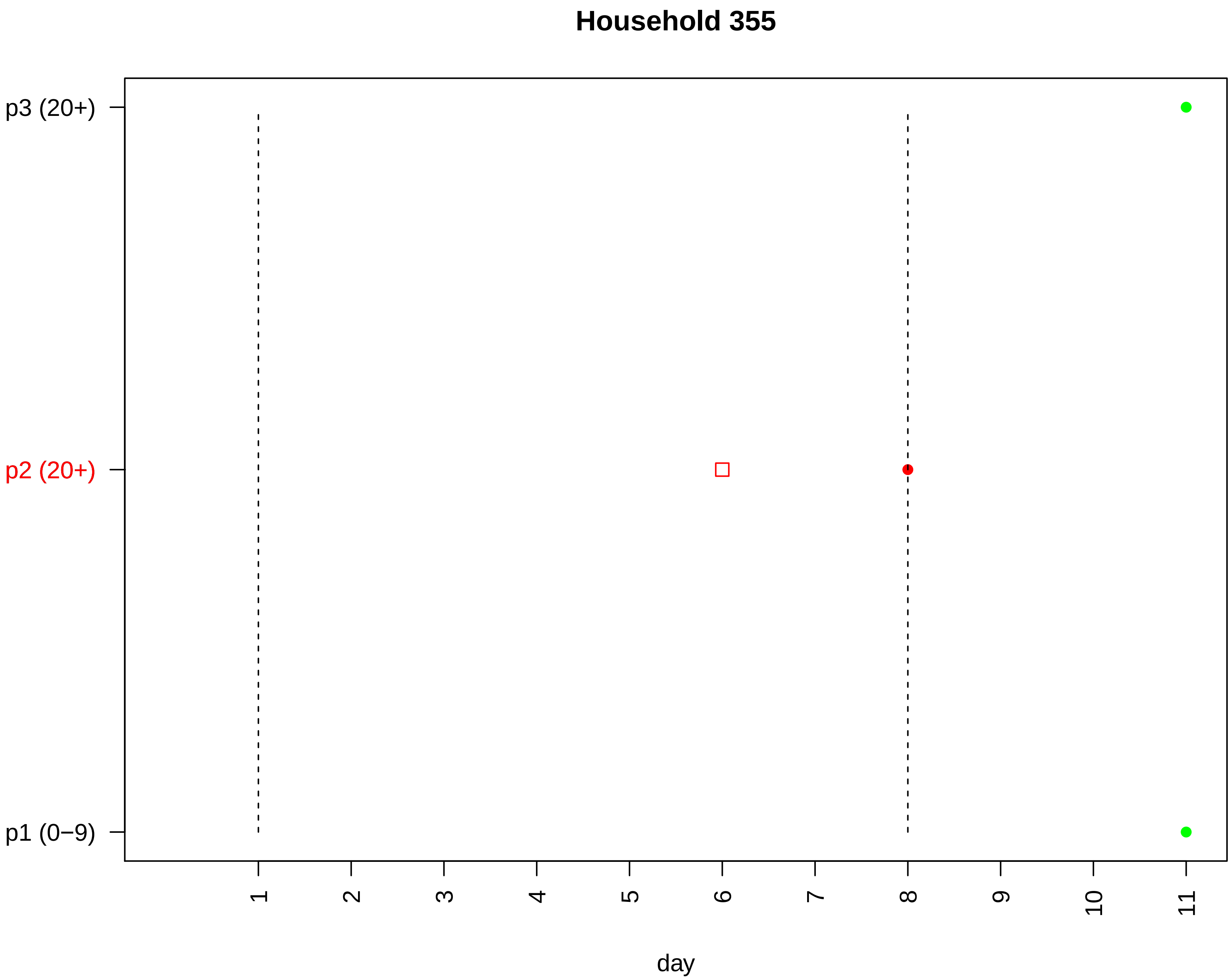

Household 355
p3 (20+)
p2 (20+)
p1 (0−9)
1
2
3
4
5
6
7
8
9
10
11
day

Household 356

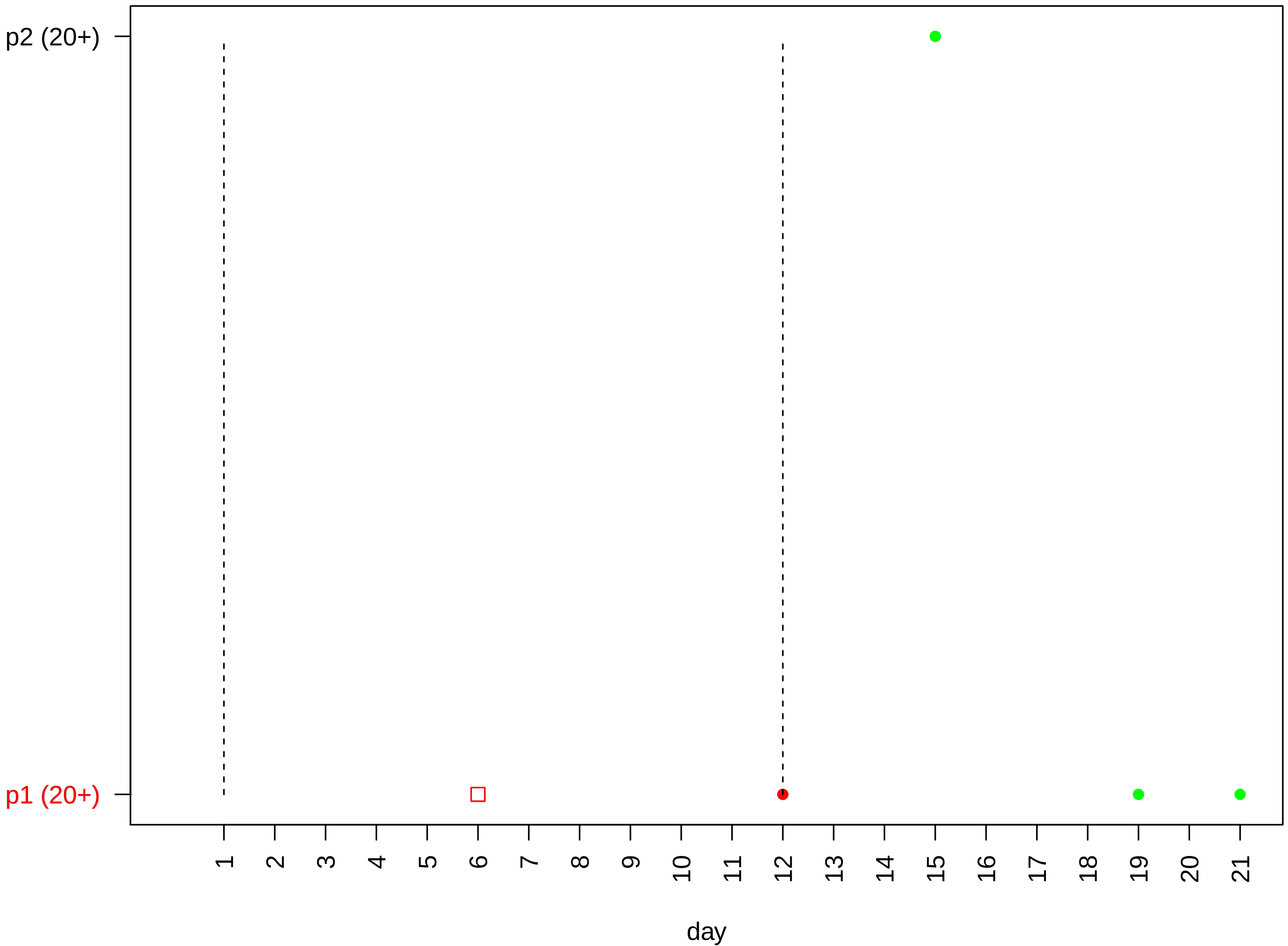

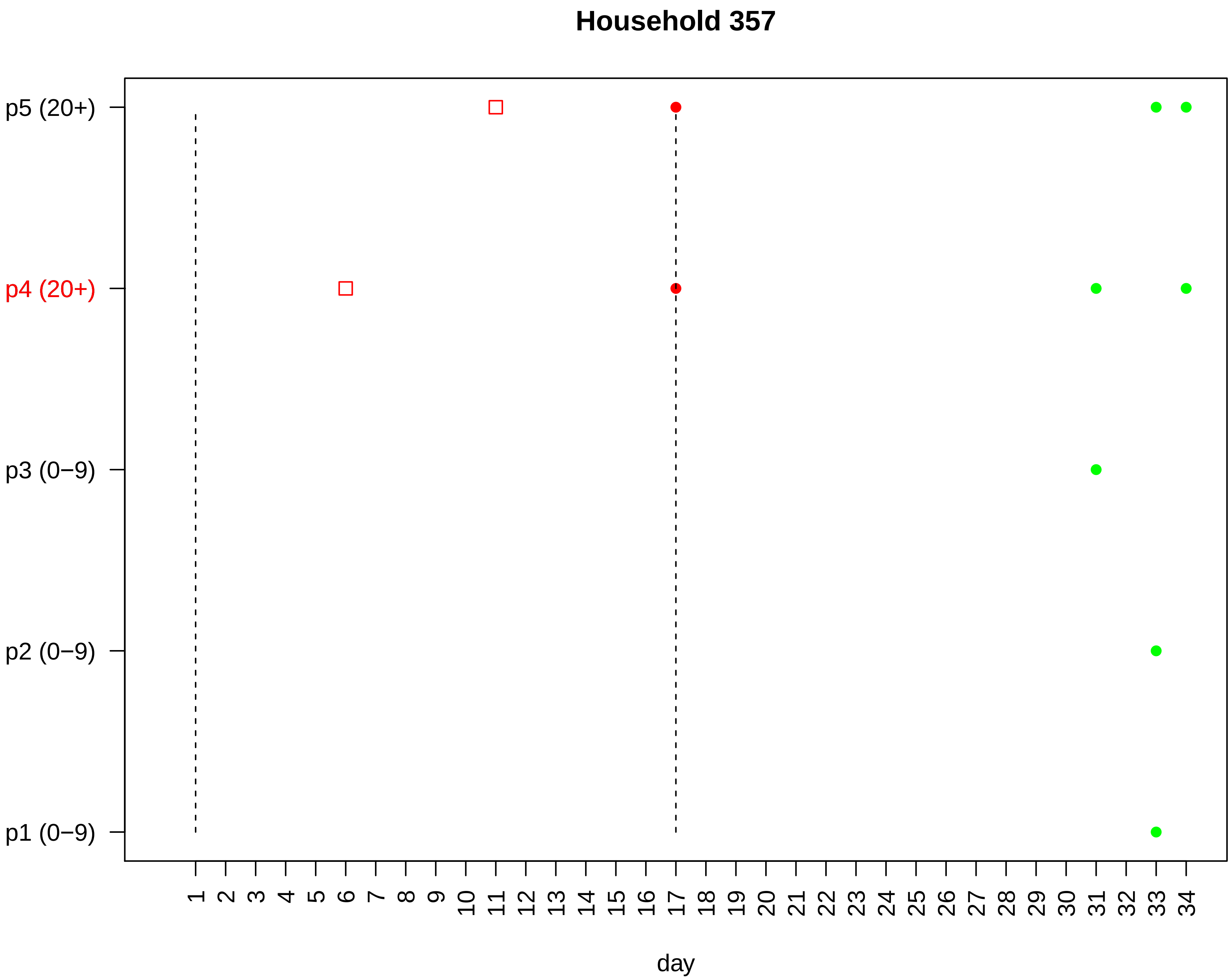

Household 357
p5 (20+)
p4 (20+)
p3 (0−9)
p2 (0−9)
p1 (0−9)
1
2
3
4
5
6
7
8
9
10
11
12
13
14
15
16
17
18
19
20
21
22
23
24
25
26
27
28
29
30
31
32
33
34
day

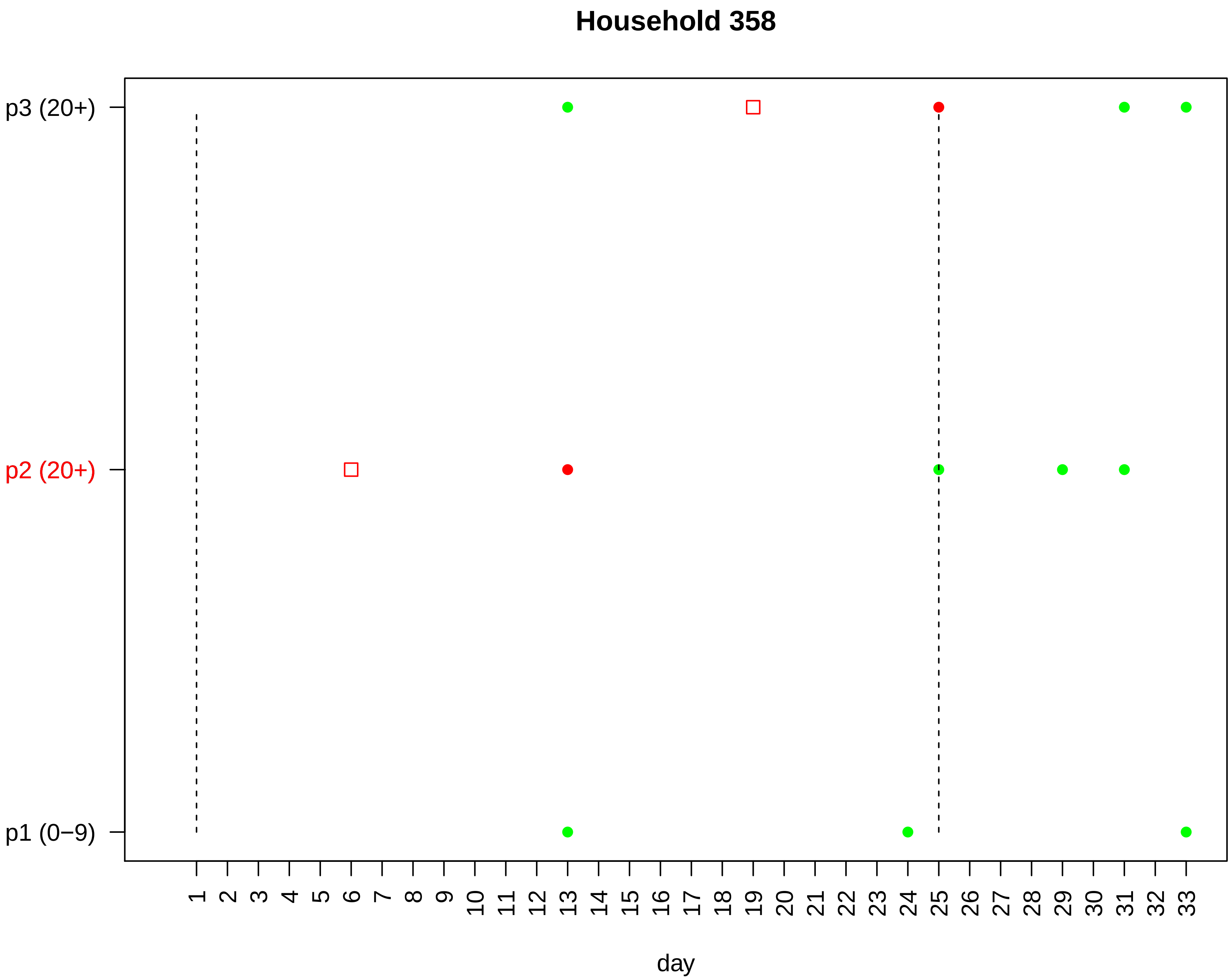
Household 358
p3 (20+)
p2 (20+)
p1 (0−9)
1 2 3 4 5 6 7 8 9 10 11 12 13 14 15 16 17 18 19 20 21 22 23 24 25 26 27 28 29 30 31 32 33
day

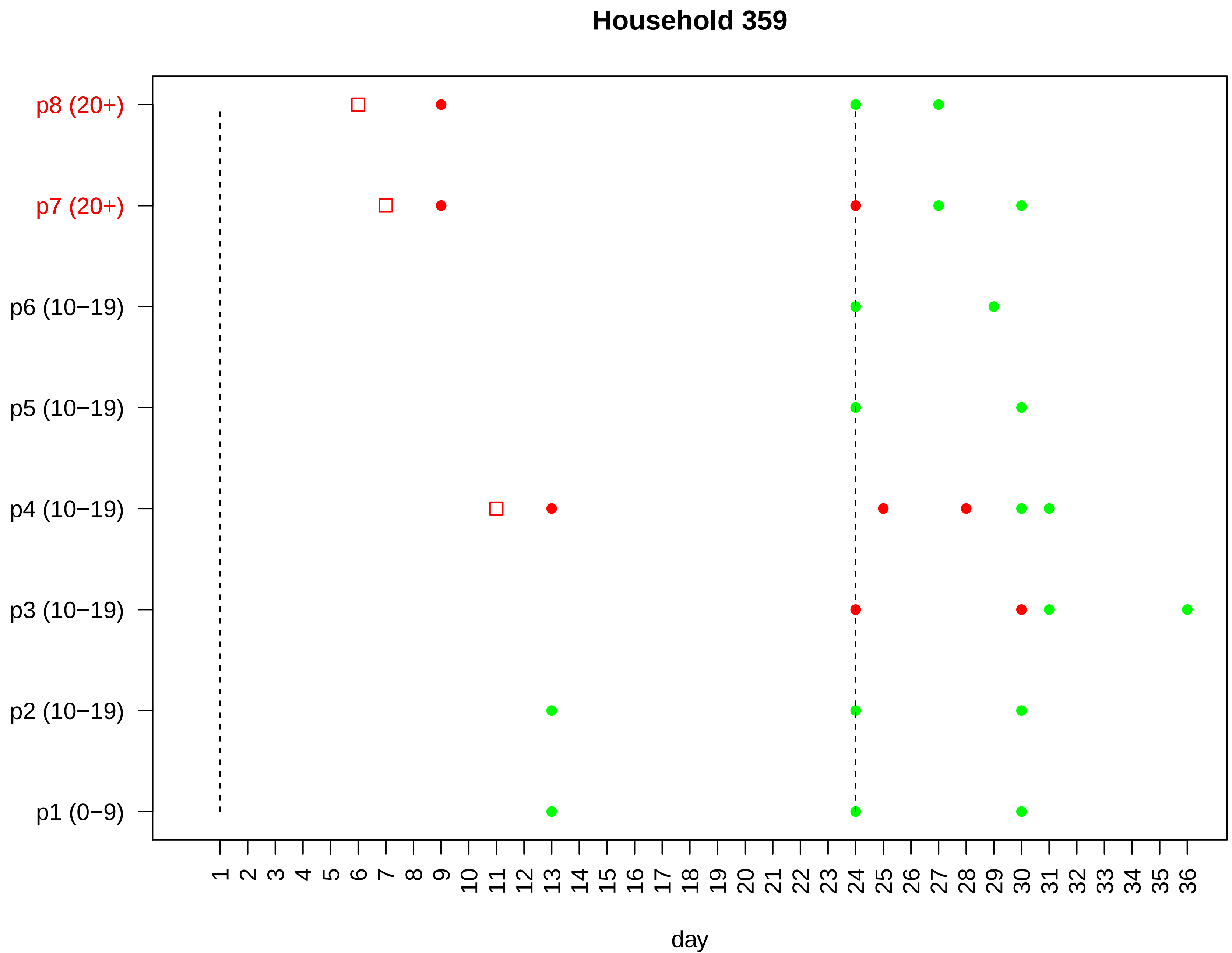

Household 359
p8 (20+)
p7 (20+)
p6 (10−19)
p5 (10−19)
p4 (10−19)
p3 (10−19)
p2 (10−19)
p1 (0−9)
1 2 3 4 5 6 7 8 9 10 11 12 13 14 15 16 17 18 19 20 21 22 23 24 25 26 27 28 29 30 31 32 33 34 35 36
day

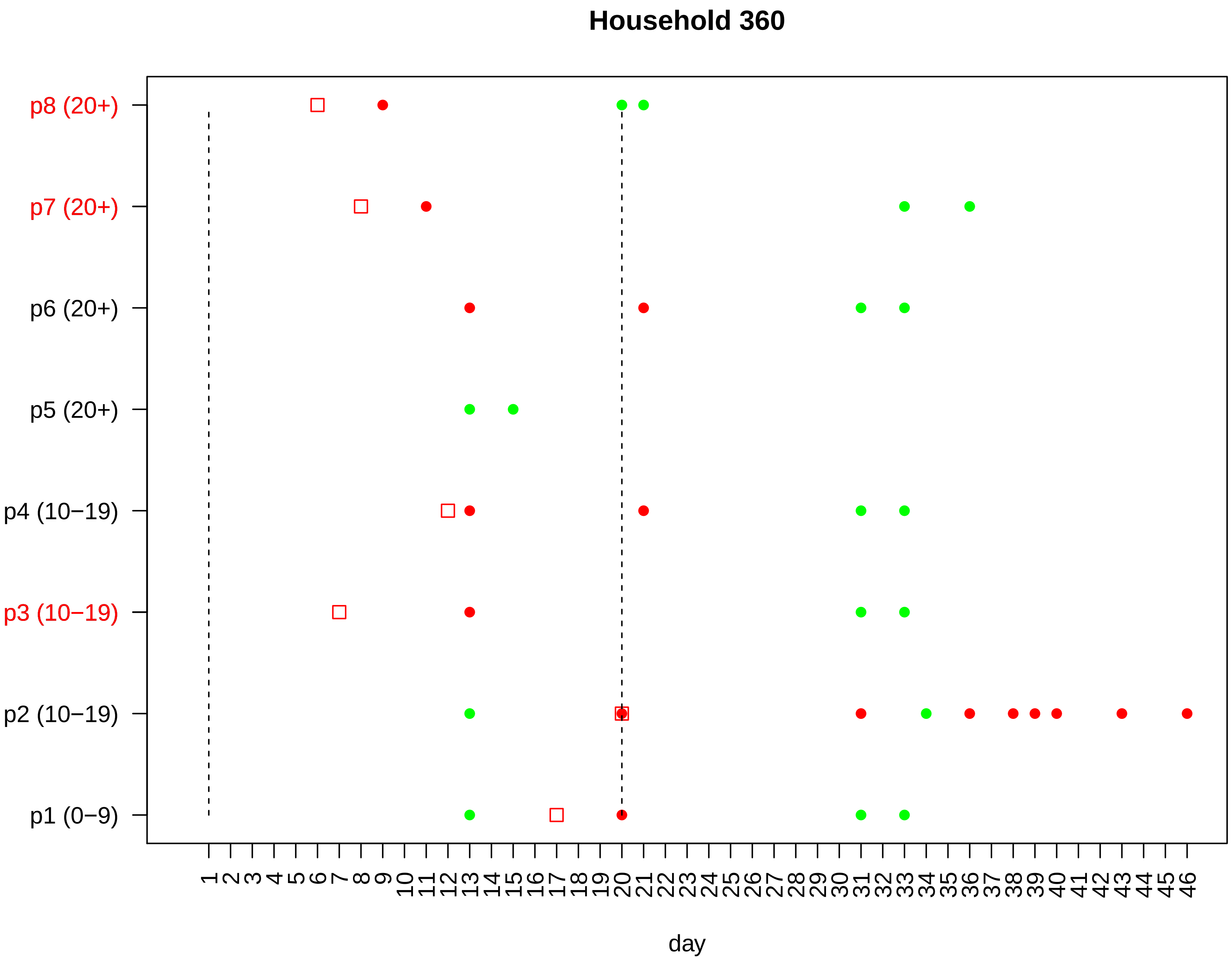

Household 360
p8 (20+)
p7 (20+)
p6 (20+)
p5 (20+)
p4 (10−19)
p3 (10−19)
p2 (10−19)
p1 (0−9)
1 2 3 4 5 6 7 8 9 10 11 12 13 14 15 16 17 18 19 20 21 22 23 24 25 26 27 28 29 30 31 32 33 34 35 36 37 38 39 40 41 42 43 44 45 46
day

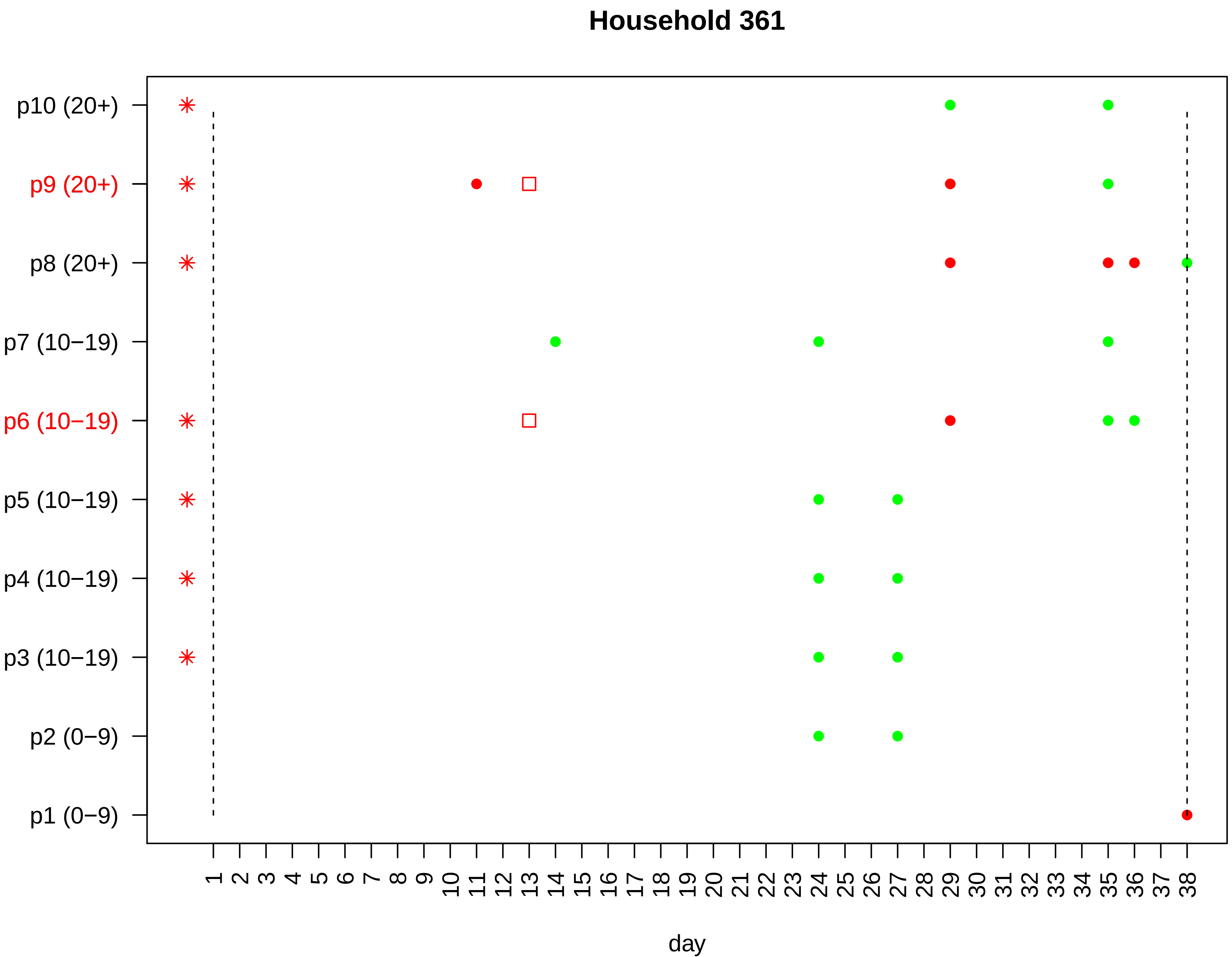

Household 361
p10 (20+)
p9 (20+)
p8 (20+)
p7 (10−19)
p6 (10−19)
p5 (10−19)
p4 (10−19)
p3 (10−19)
p2 (0−9)
p1 (0−9)
1 2 3 4 5 6 7 8 9 10 11 12 13 14 15 16 17 18 19 20 21 22 23 24 25 26 27 28 29 30 31 32 33 34 35 36 37 38
day

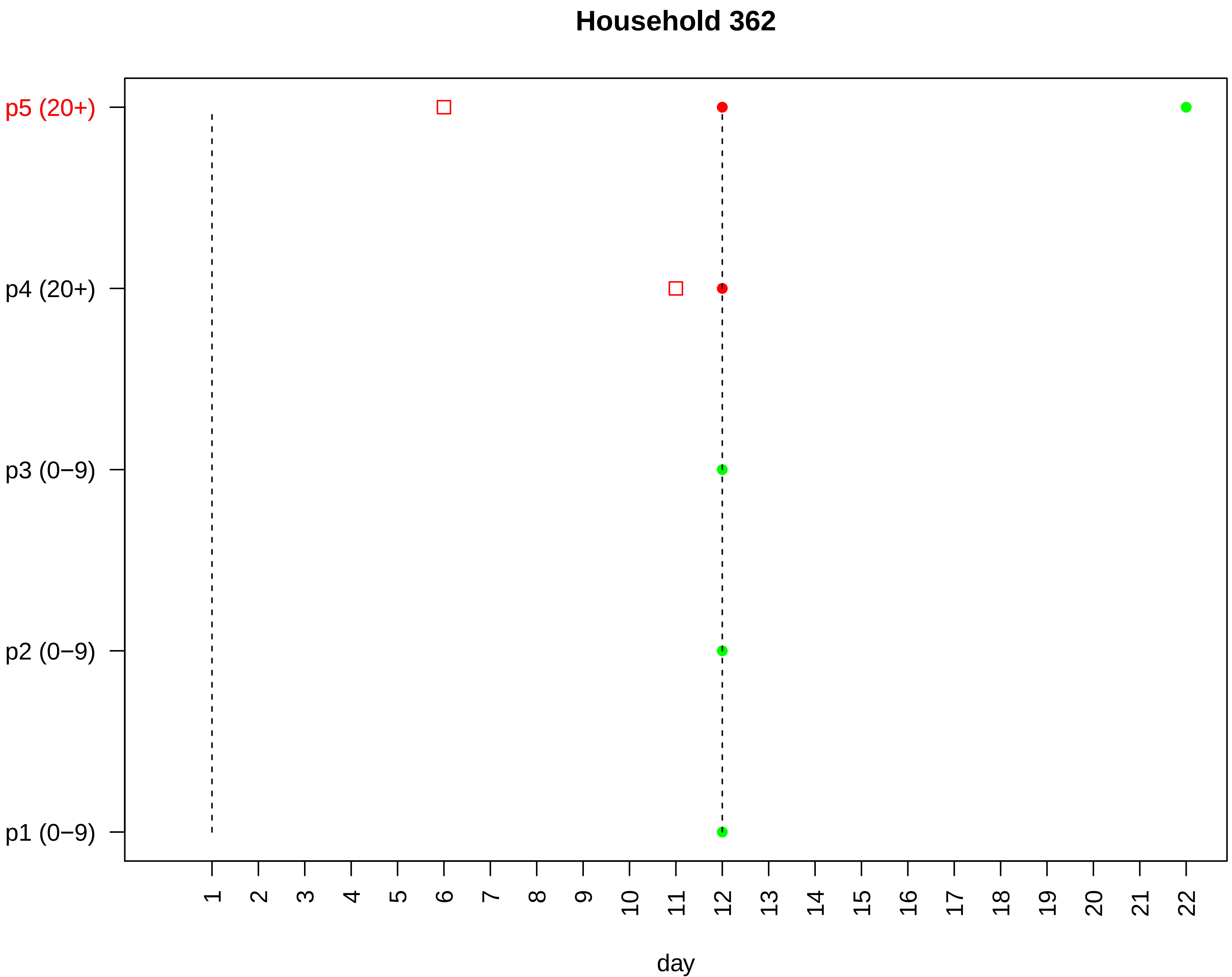

Household 362
p5 (20+)
p4 (20+)
p3 (0−9)
p2 (0−9)
p1 (0−9)
1
2
3
4
5
6
7
8
9
10
11
12
13
14
15
16
17
18
19
20
21
22
day

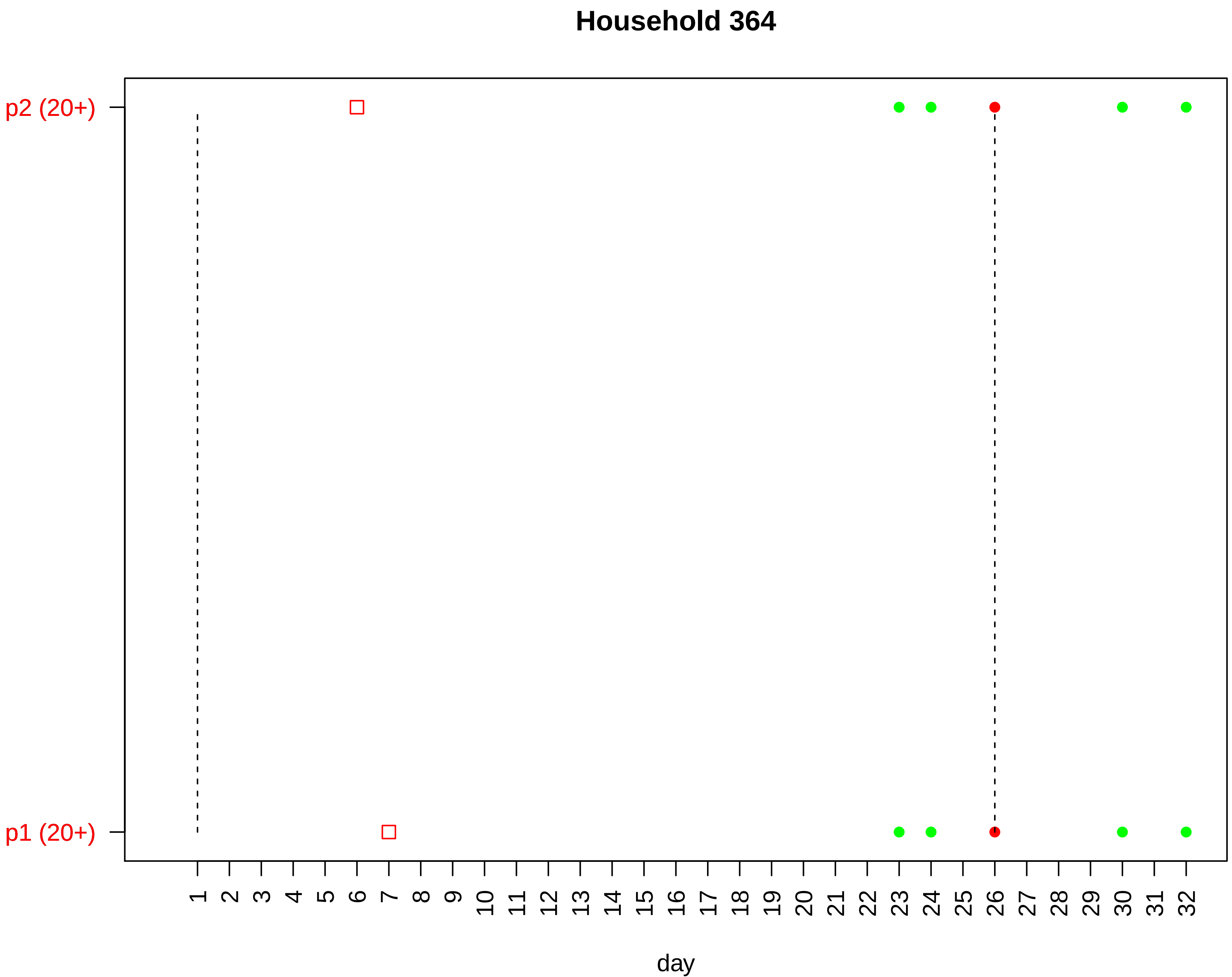

Household 364
p2 (20+)
p1 (20+)
1
2
3
4
5
6
7
8
9
10
11
12
13
14
15
16
17
18
19
20
21
22
23
24
25
26
27
28
29
30
31
32
day

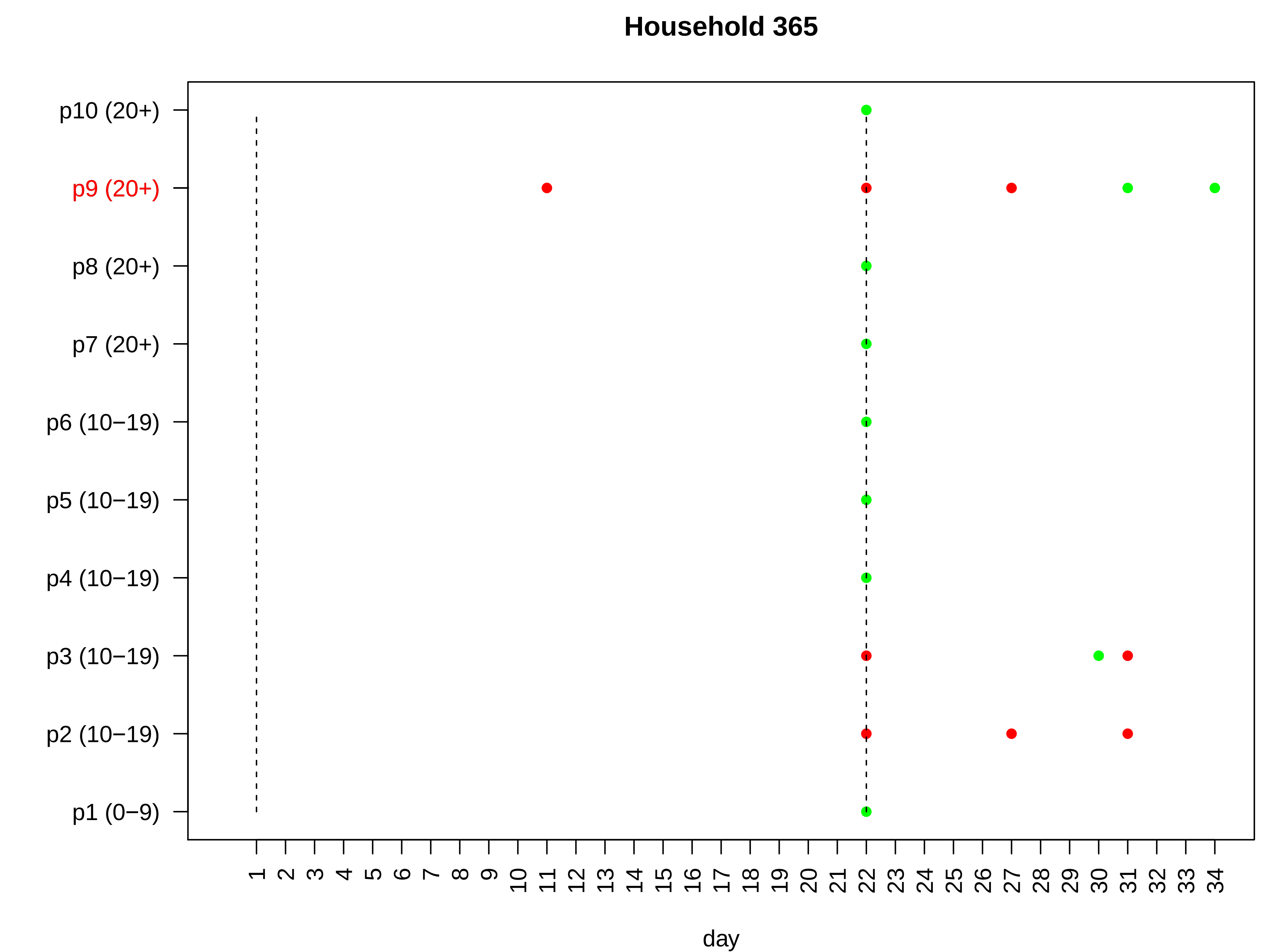

Household 365
p10 (20+)
p9 (20+)
p8 (20+)
p7 (20+)
p6 (10−19)
p5 (10−19)
p4 (10−19)
p3 (10−19)
p2 (10−19)
p1 (0−9)
1 2 3 4 5 6 7 8 9 10 11 12 13 14 15 16 17 18 19 20 21 22 23 24 25 26 27 28 29 30 31 32 33 34
day

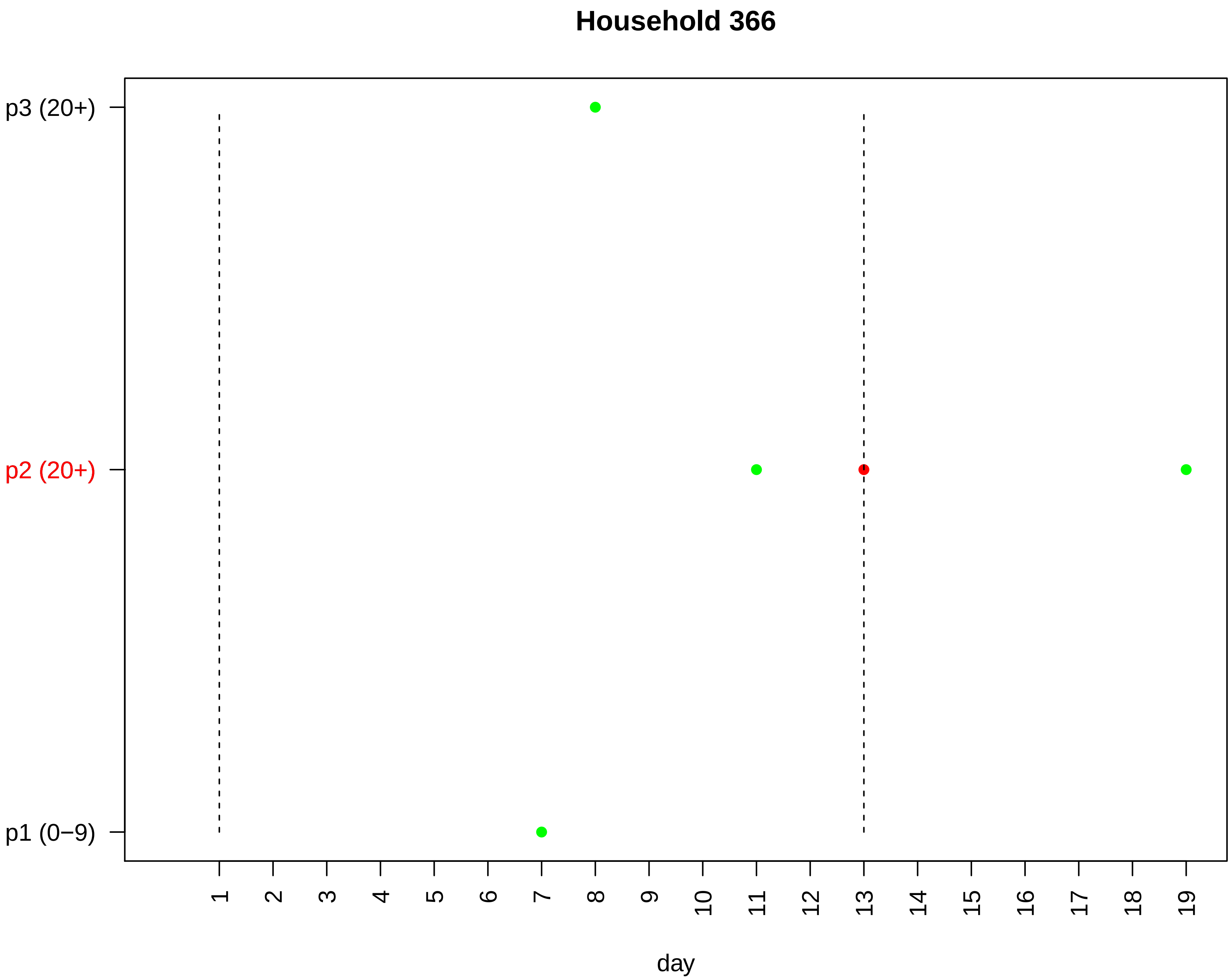
Household 366
p3 (20+)
p2 (20+)
p1 (0−9)
1
2
3
4
5
6
7
8
9
10
11
12
13
14
15
16
17
18
19
day

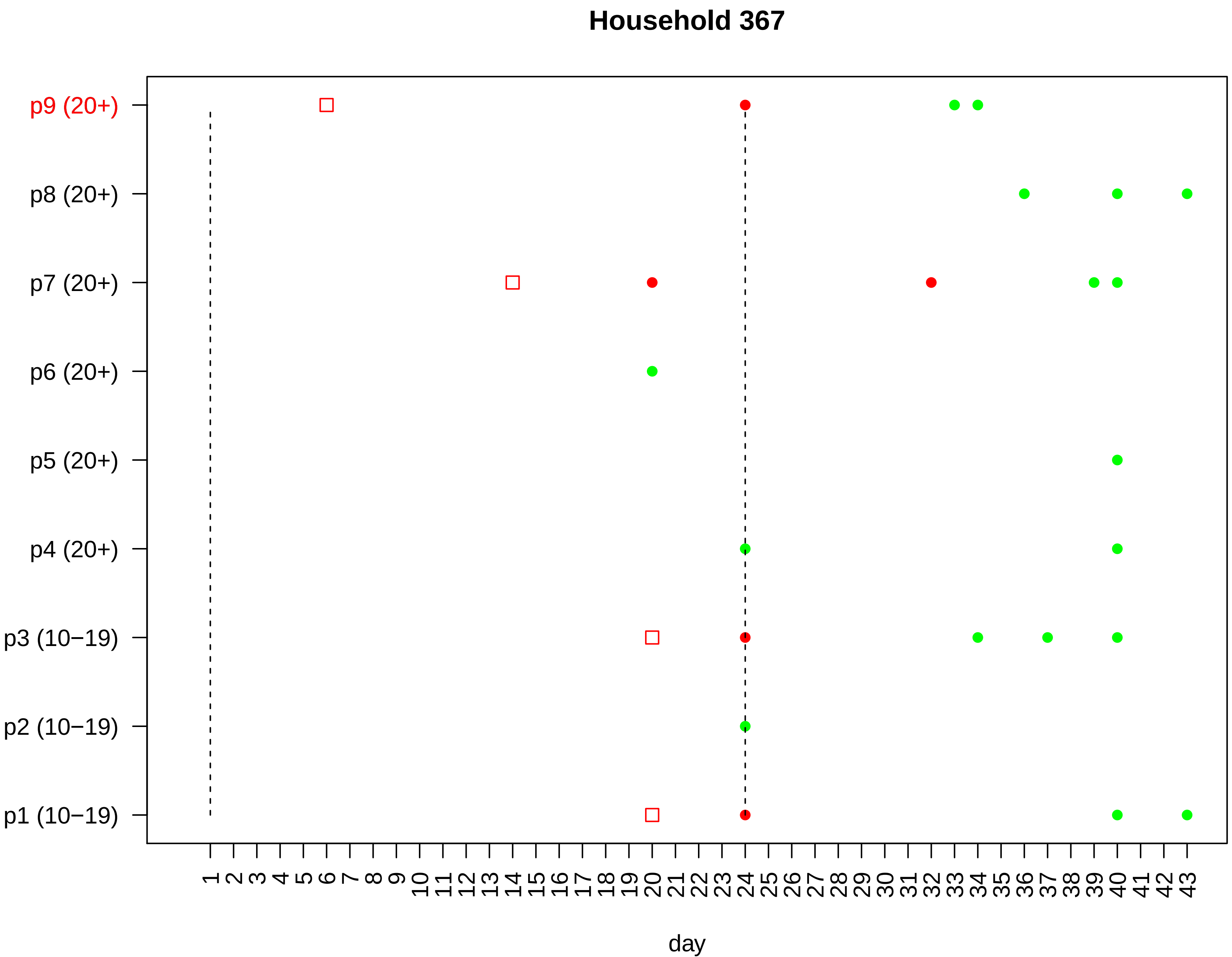

Household 367
p9 (20+)
p8 (20+)
p7 (20+)
p6 (20+)
p5 (20+)
p4 (20+)
p3 (10−19)
p2 (10−19)
p1 (10−19)
1 2 3 4 5 6 7 8 9 10 11 12 13 14 15 16 17 18 19 20 21 22 23 24 25 26 27 28 29 30 31 32 33 34 35 36 37 38 39 40 41 42 43
day

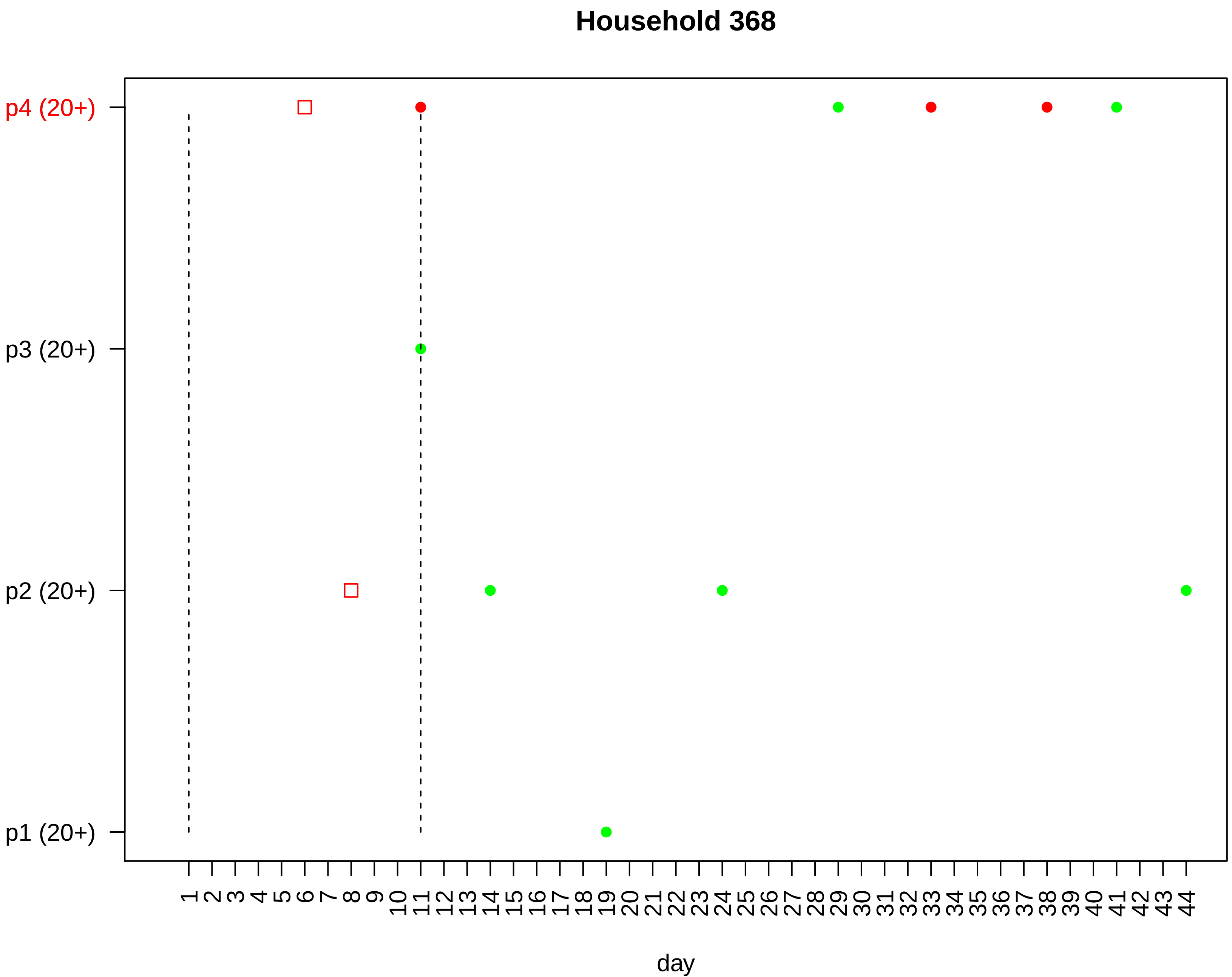

Household 368
p4 (20+)
p3 (20+)
p2 (20+)
p1 (20+)
1 2 3 4 5 6 7 8 9 10 11 12 13 14 15 16 17 18 19 20 21 22 23 24 25 26 27 28 29 30 31 32 33 34 35 36 37 38 39 40 41 42 43 44
day

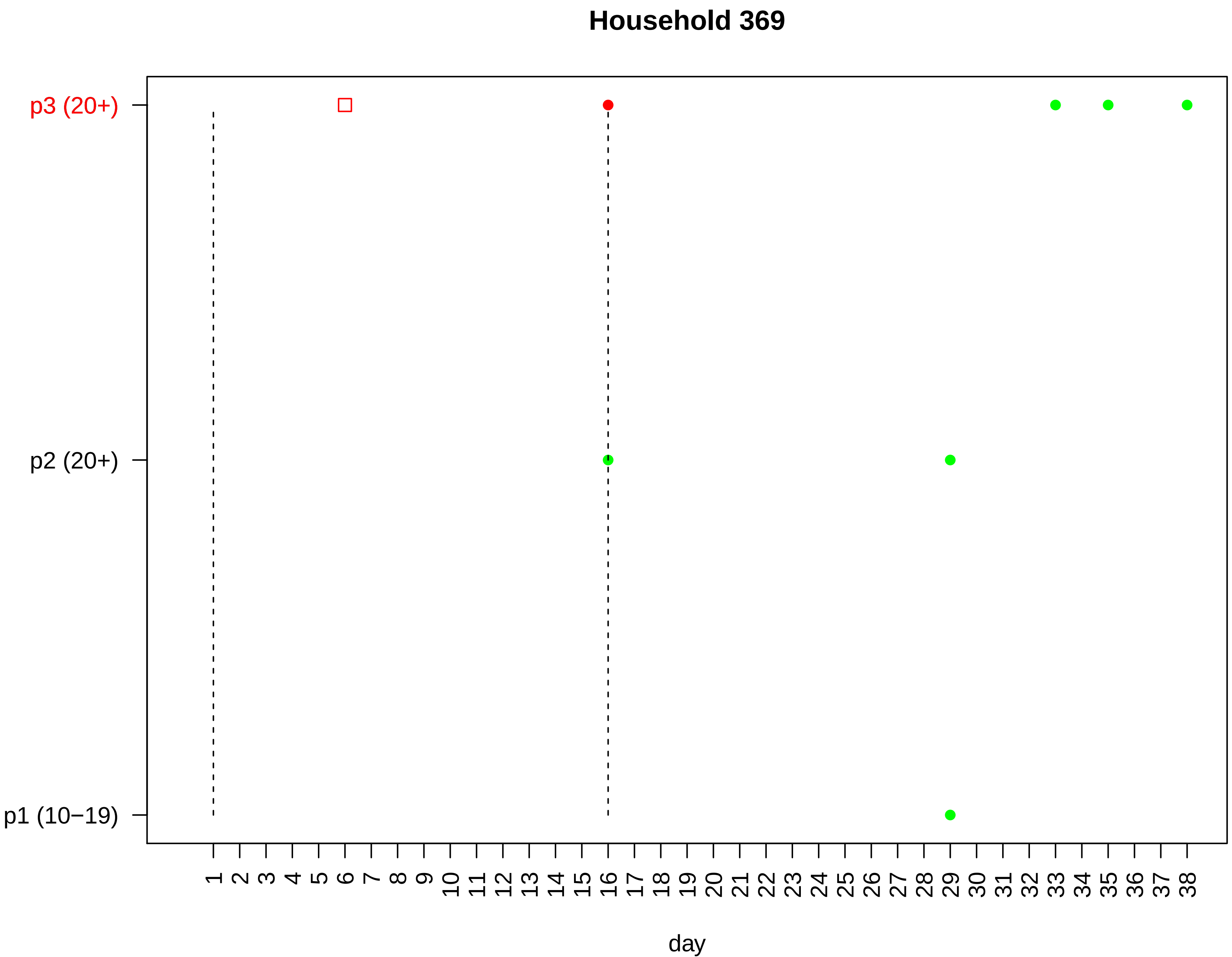

Household 369
p3 (20+)
p2 (20+)
p1 (10−19)
1
2
3
4
5
6
7
8
9
10
11
12
13
14
15
16
17
18
19
20
21
22
23
24
25
26
27
28
29
30
31
32
33
34
35
36
37
38
day

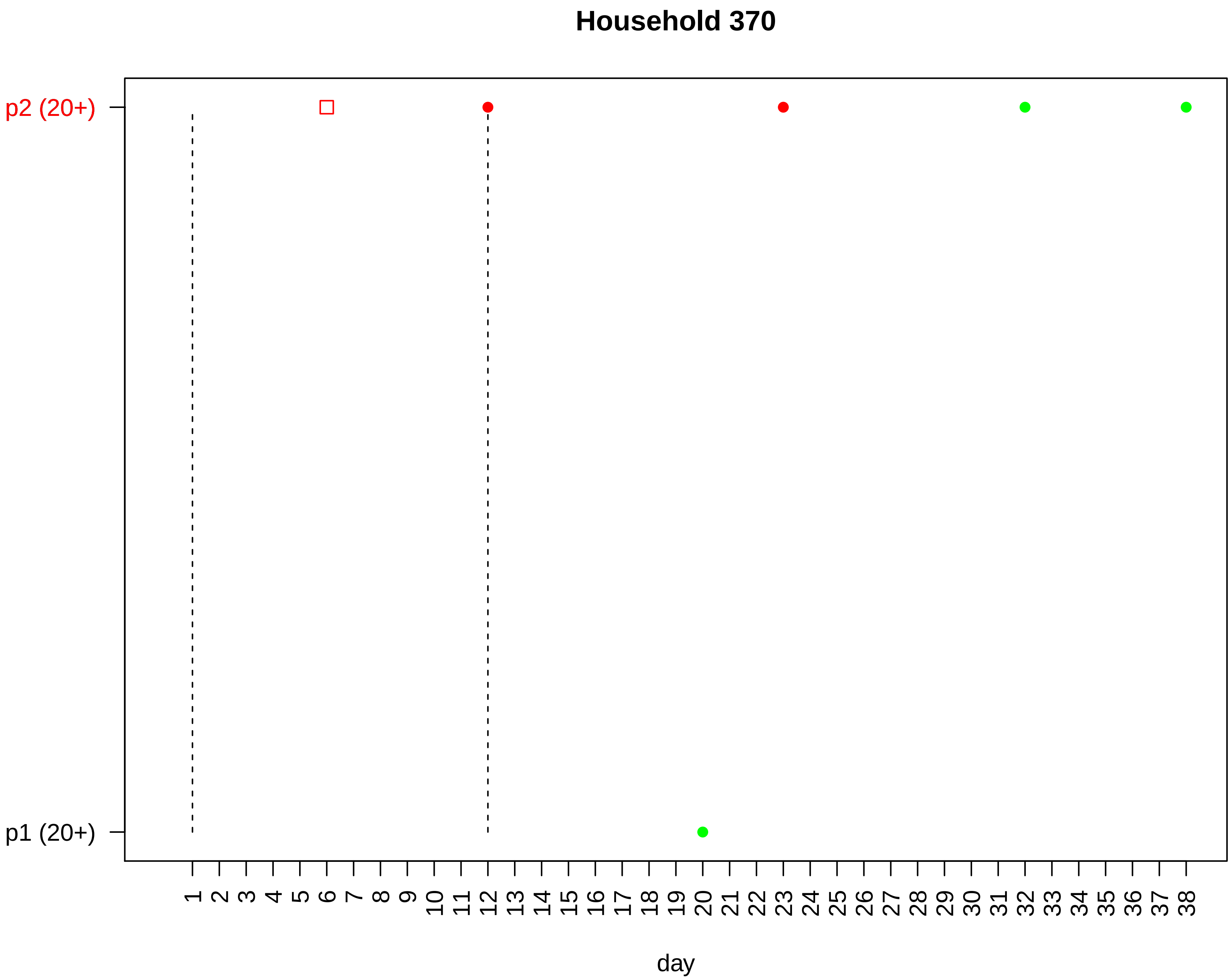

Household 370
p2 (20+)
p1 (20+)
1
2
3
4
5
6
7
8
9
10
11
12
13
14
15
16
17
18
19
20
21
22
23
24
25
26
27
28
29
30
31
32
33
34
35
36
37
38
day

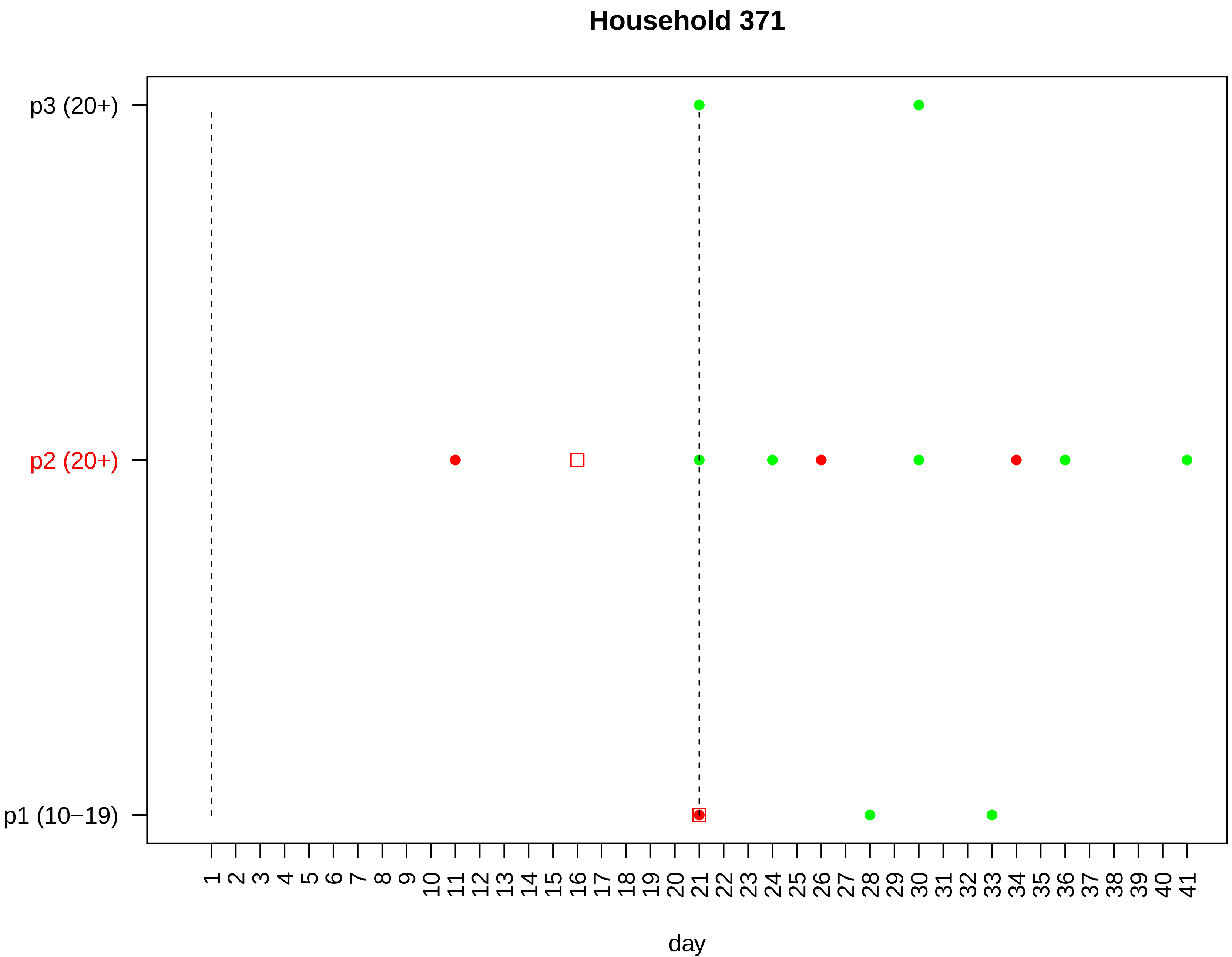

Household 371
p3 (20+)
p2 (20+)
p1 (10−19)
1 2 3 4 5 6 7 8 9 10 11 12 13 14 15 16 17 18 19 20 21 22 23 24 25 26 27 28 29 30 31 32 33 34 35 36 37 38 39 40 41
day

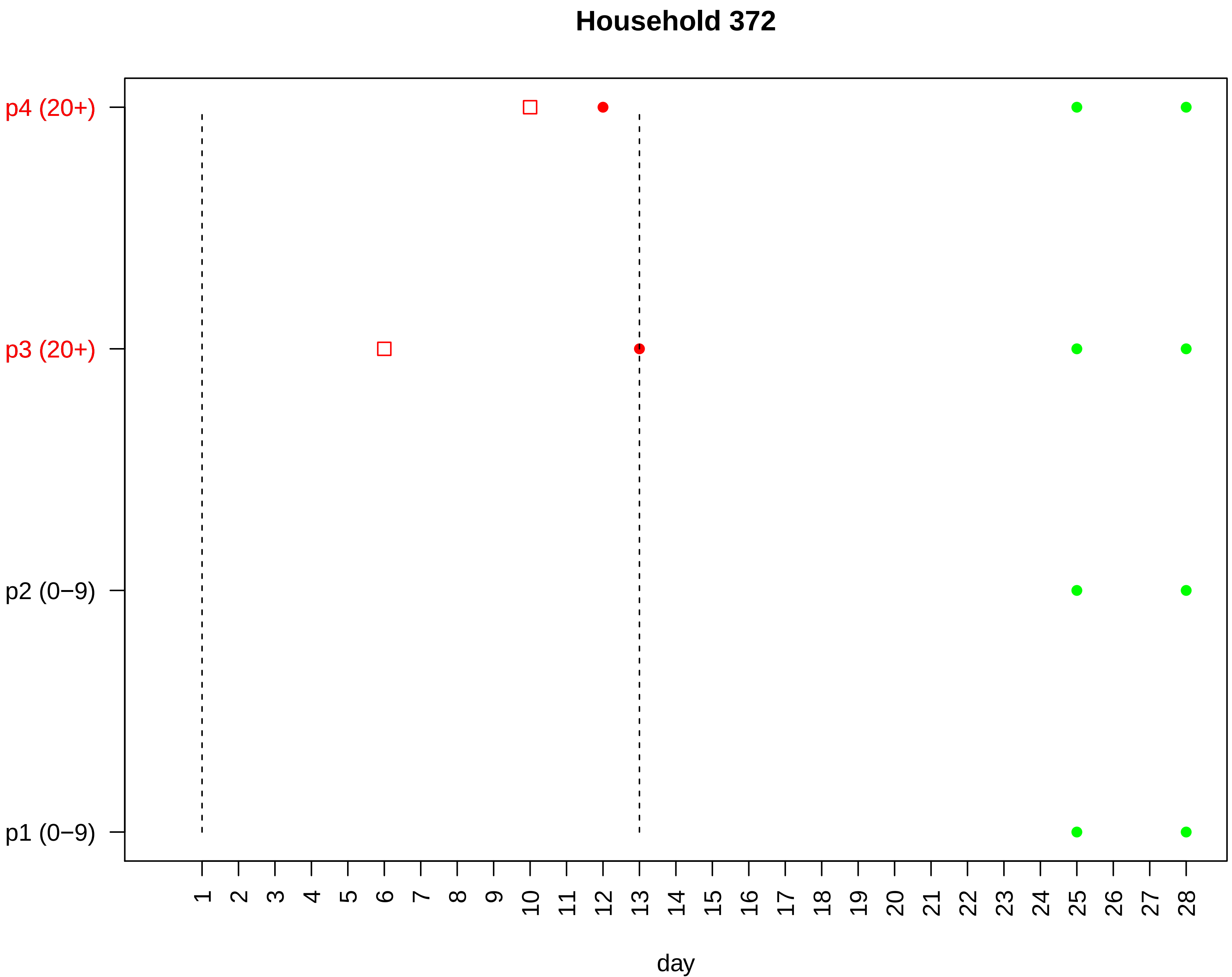

Household 372
p4 (20+)
p3 (20+)
p2 (0−9)
p1 (0−9)
1 2 3 4 5 6 7 8 9 10 11 12 13 14 15 16 17 18 19 20 21 22 23 24 25 26 27 28
day

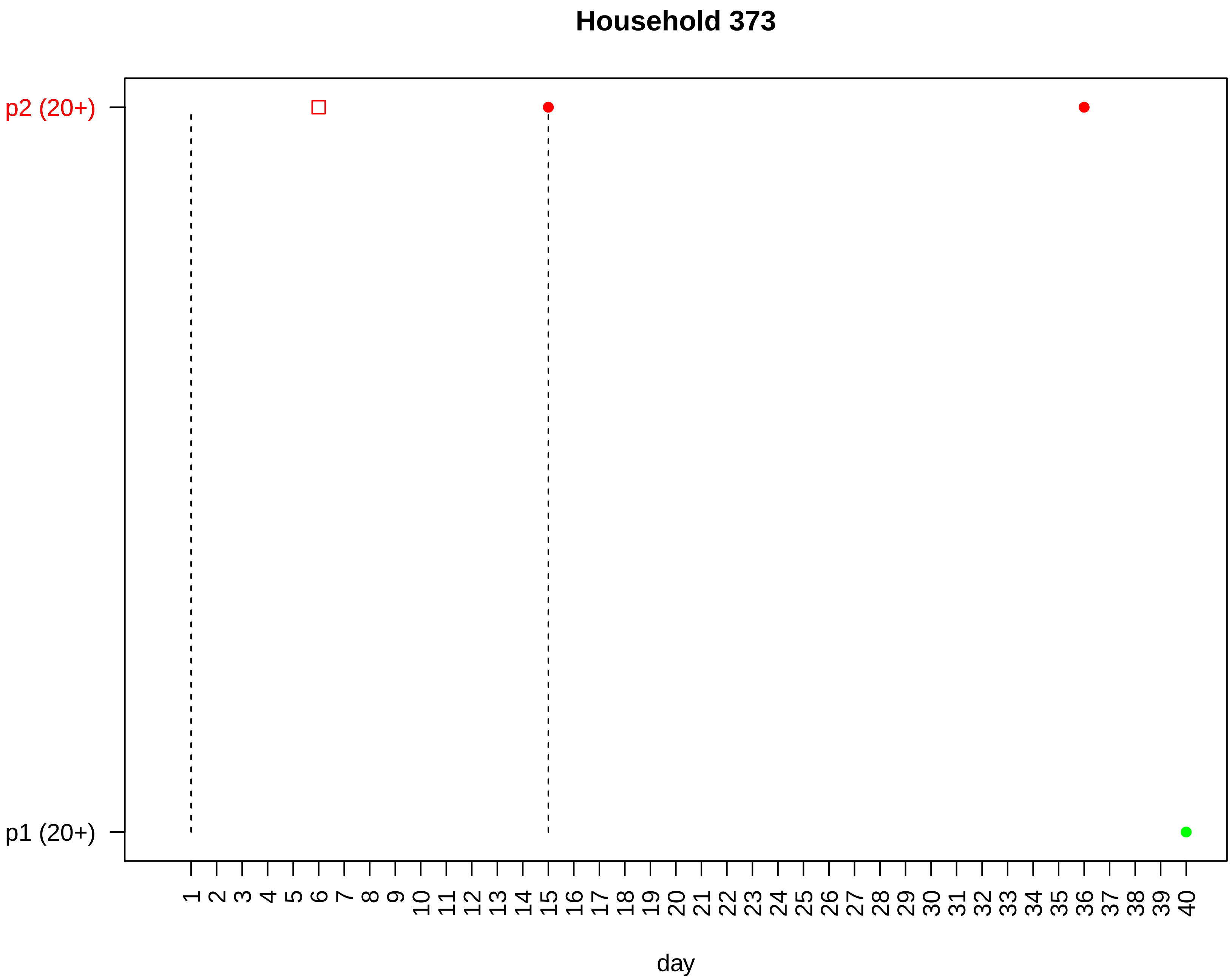

Household 373
p2 (20+)
p1 (20+)
1 2 3 4 5 6 7 8 9 10 11 12 13 14 15 16 17 18 19 20 21 22 23 24 25 26 27 28 29 30 31 32 33 34 35 36 37 38 39 40
day

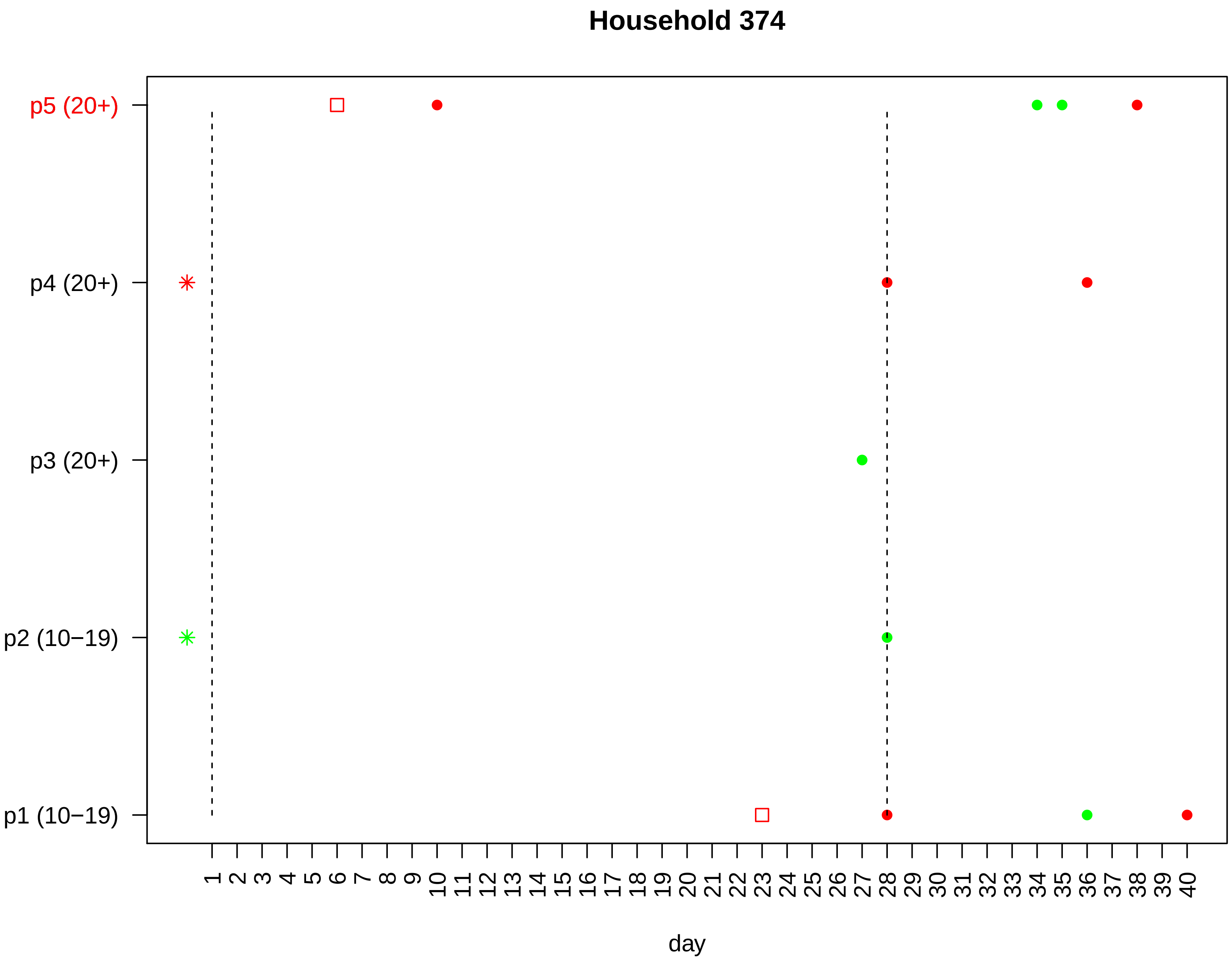

Household 374
p5 (20+)
p4 (20+)
p3 (20+)
p2 (10−19)
p1 (10−19)
1 2 3 4 5 6 7 8 9 10 11 12 13 14 15 16 17 18 19 20 21 22 23 24 25 26 27 28 29 30 31 32 33 34 35 36 37 38 39 40
day

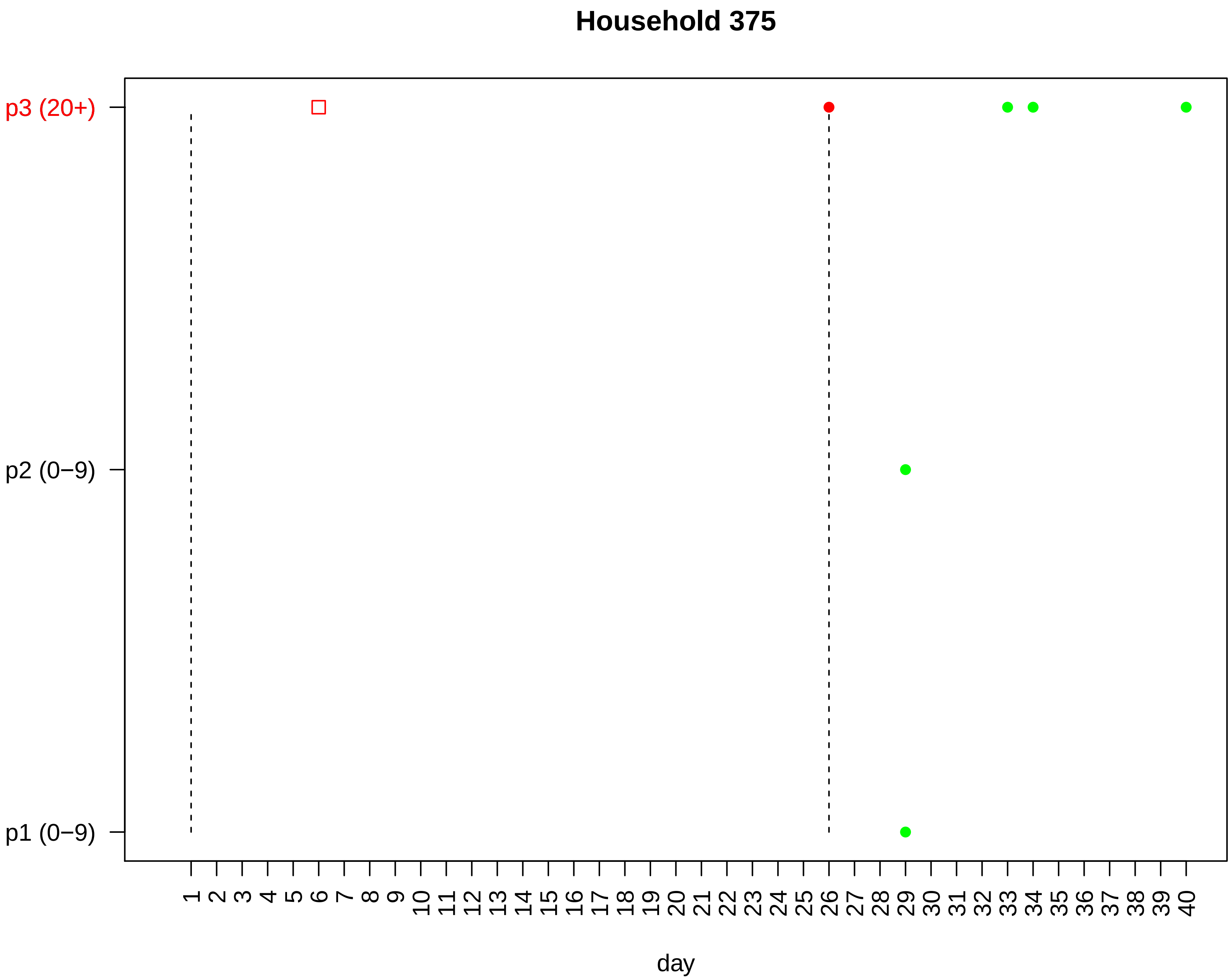
Household 375
p3 (20+)
p2 (0−9)
p1 (0−9)
1 2 3 4 5 6 7 8 9 10 11 12 13 14 15 16 17 18 19 20 21 22 23 24 25 26 27 28 29 30 31 32 33 34 35 36 37 38 39 40
day

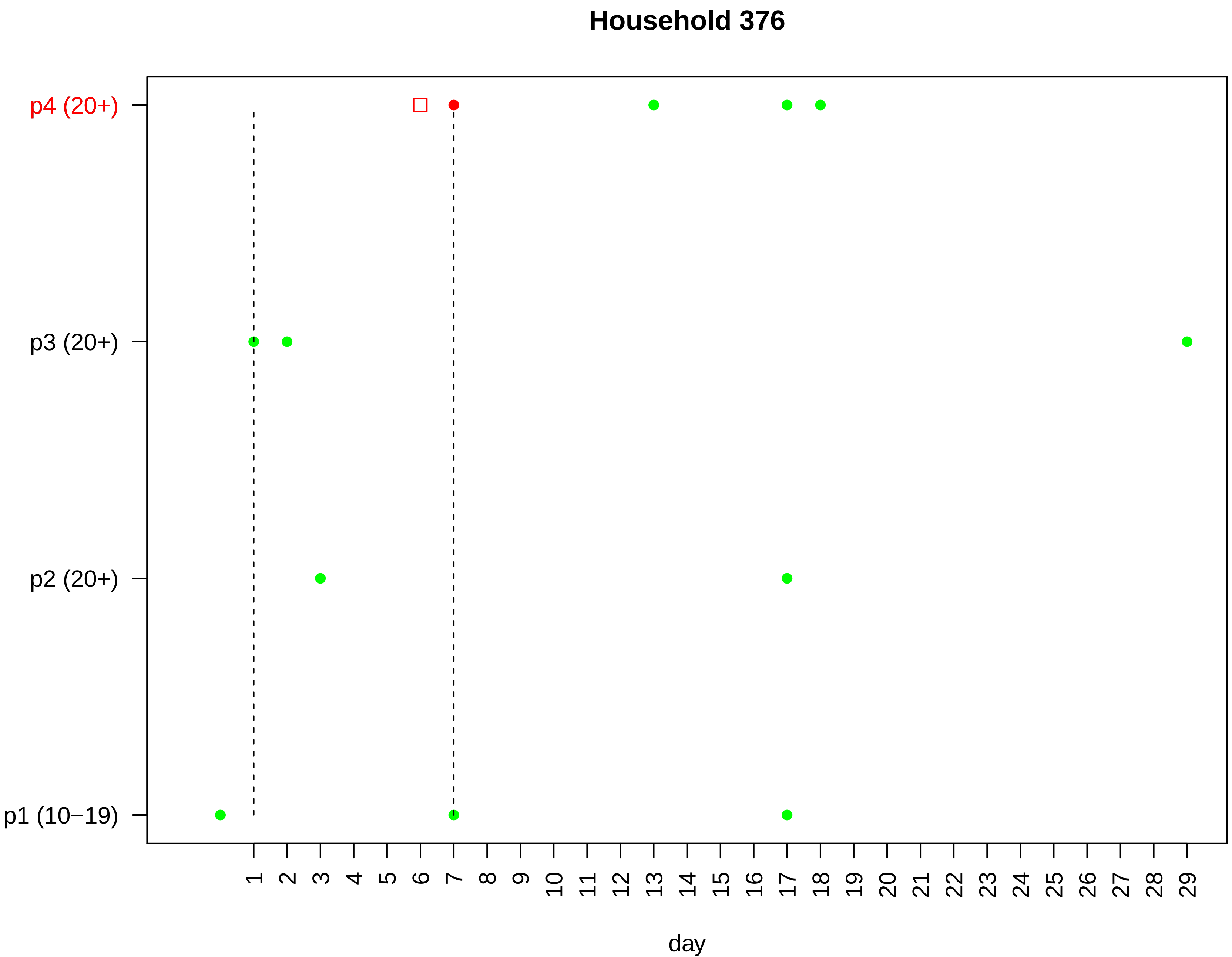

Household 376
p4 (20+)
p3 (20+)
p2 (20+)
p1 (10−19)
1 2 3 4 5 6 7 8 9 10 11 12 13 14 15 16 17 18 19 20 21 22 23 24 25 26 27 28 29
day

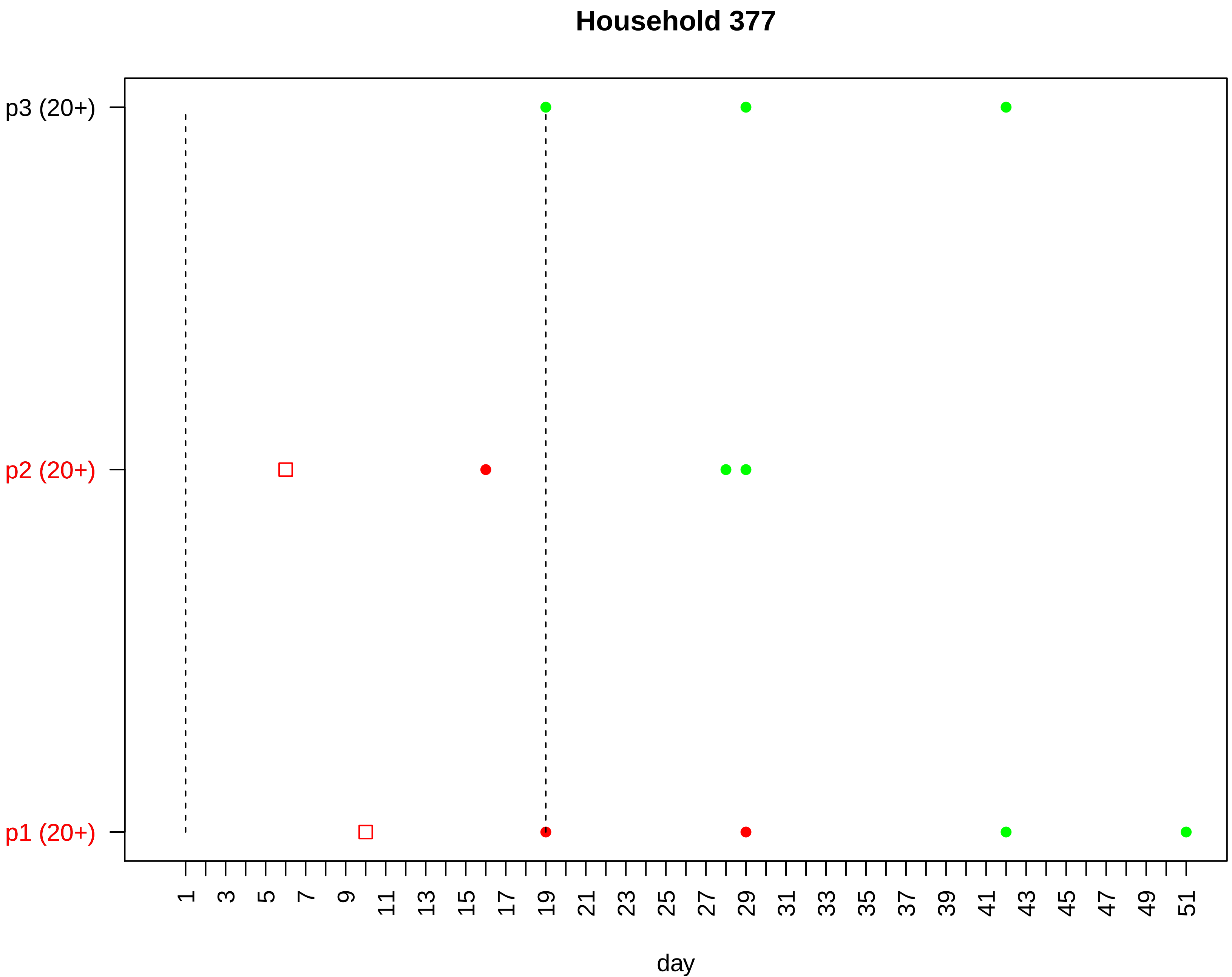

Household 377
p3 (20+)
p2 (20+)
p1 (20+)
1
3
5
7
9
11
13
15
17
19
21
23
25
27
29
31
33
35
37
39
41
43
45
47
49
51
day

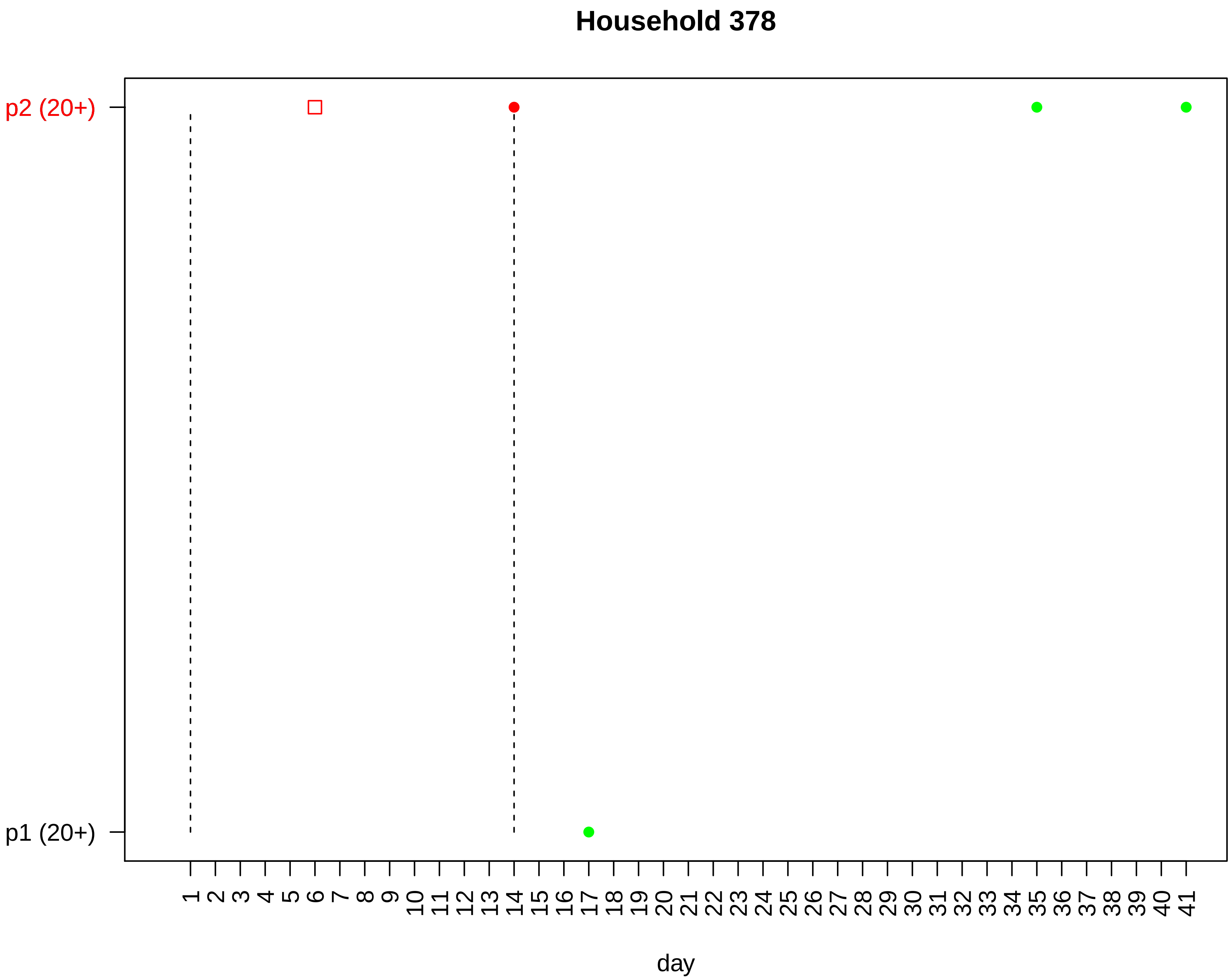

Household 378
p2 (20+)
p1 (20+)
1
2
3
4
5
6
7
8
9
10
11
12
13
14
15
16
17
18
19
20
21
22
23
24
25
26
27
28
29
30
31
32
33
34
35
36
37
38
39
40
41
day

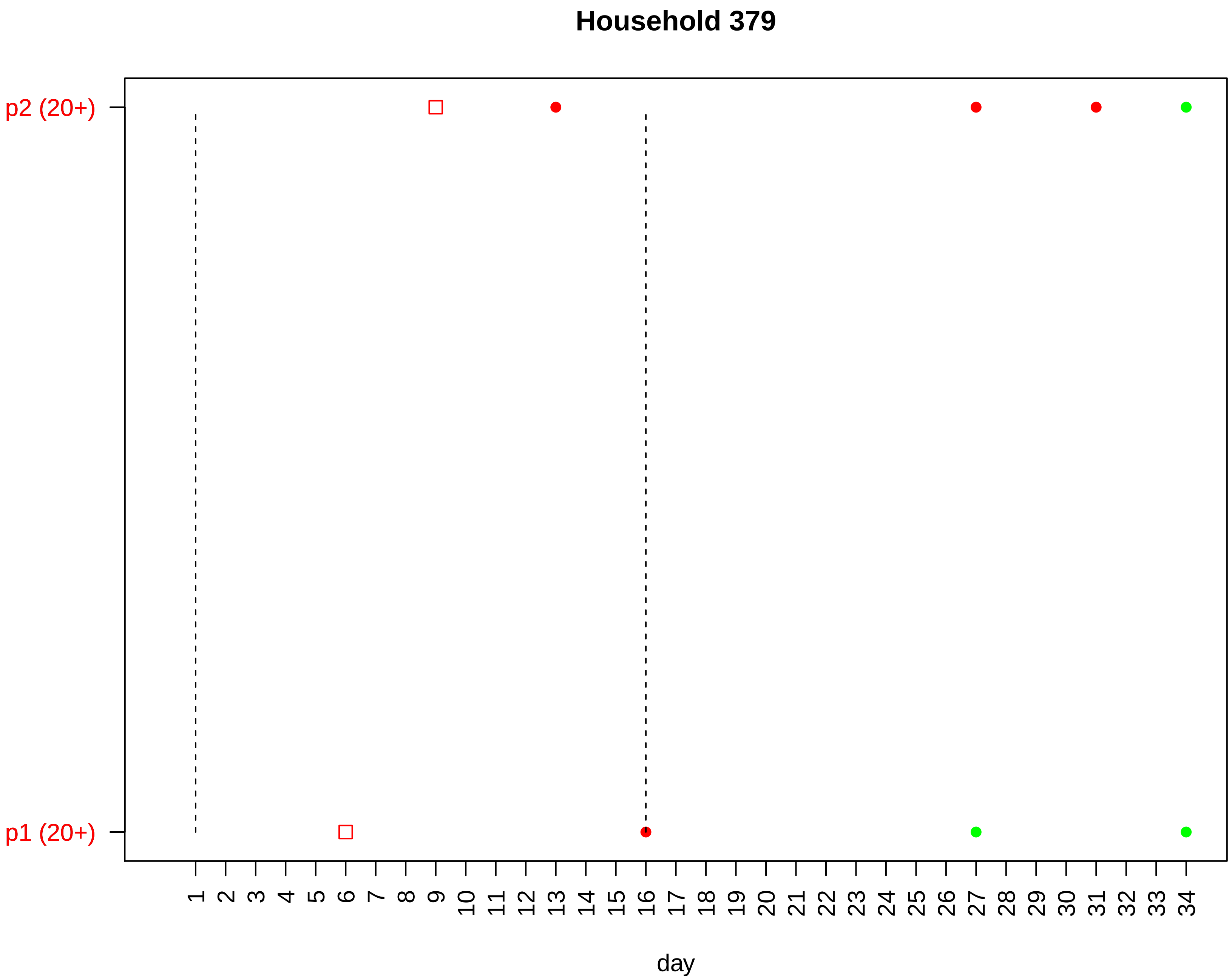
Household 379
p2 (20+)
p1 (20+)
1 2 3 4 5 6 7 8 9 10 11 12 13 14 15 16 17 18 19 20 21 22 23 24 25 26 27 28 29 30 31 32 33 34
day

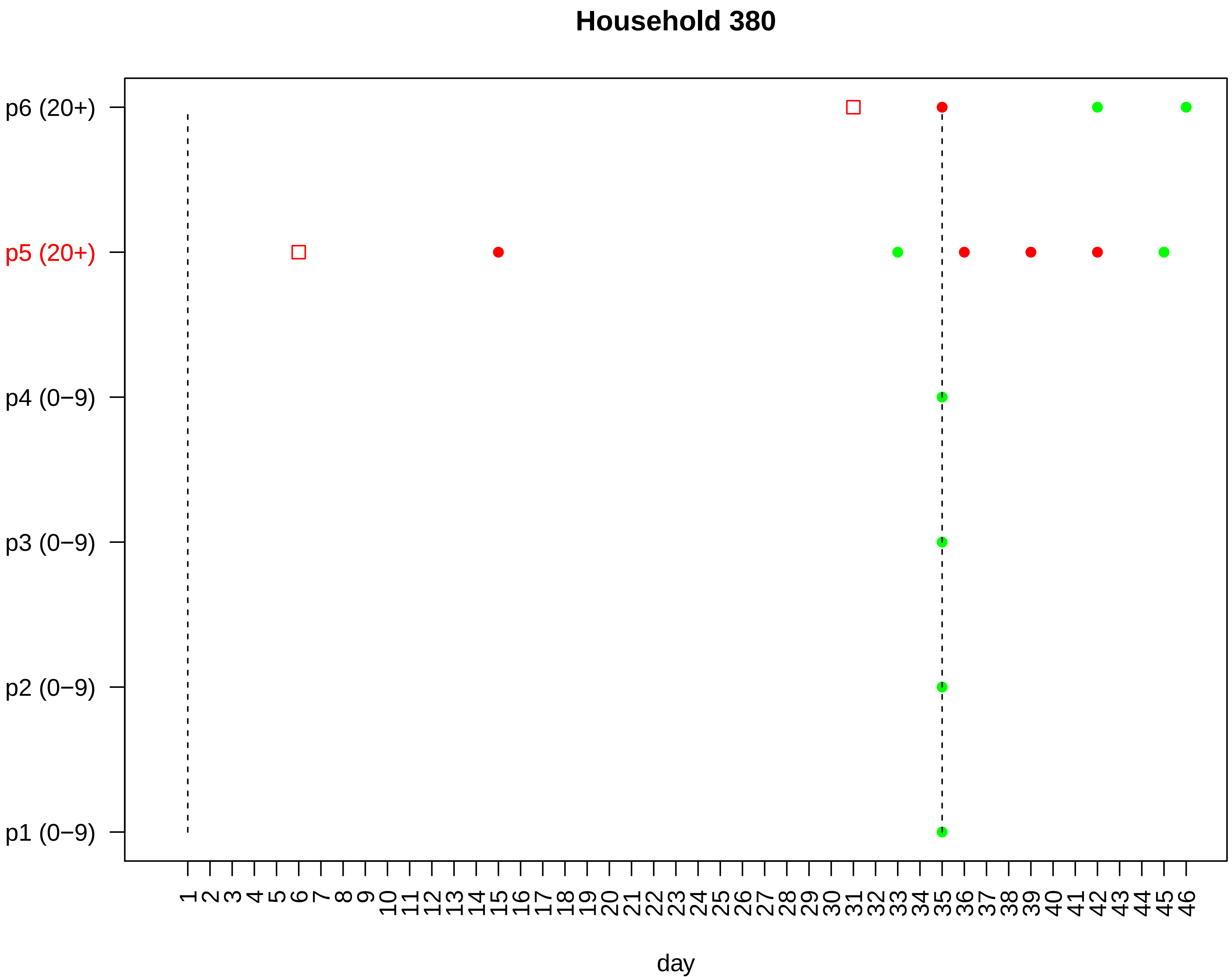

Household 380
p6 (20+)
p5 (20+)
p4 (0−9)
p3 (0−9)
p2 (0−9)
p1 (0−9)
1 2 3 4 5 6 7 8 9 10 11 12 13 14 15 16 17 18 19 20 21 22 23 24 25 26 27 28 29 30 31 32 33 34 35 36 37 38 39 40 41 42 43 44 45 46
day

Household 381

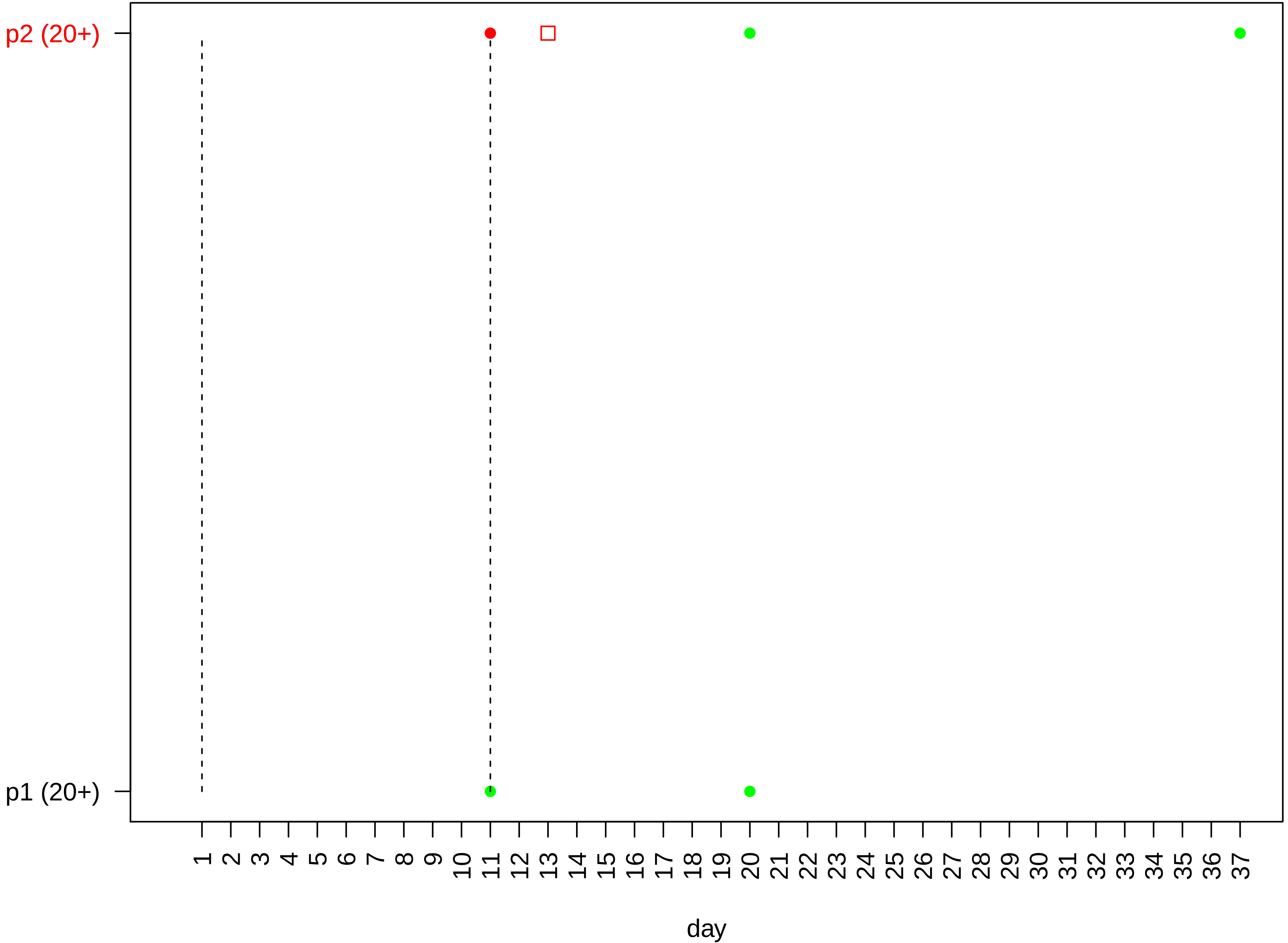

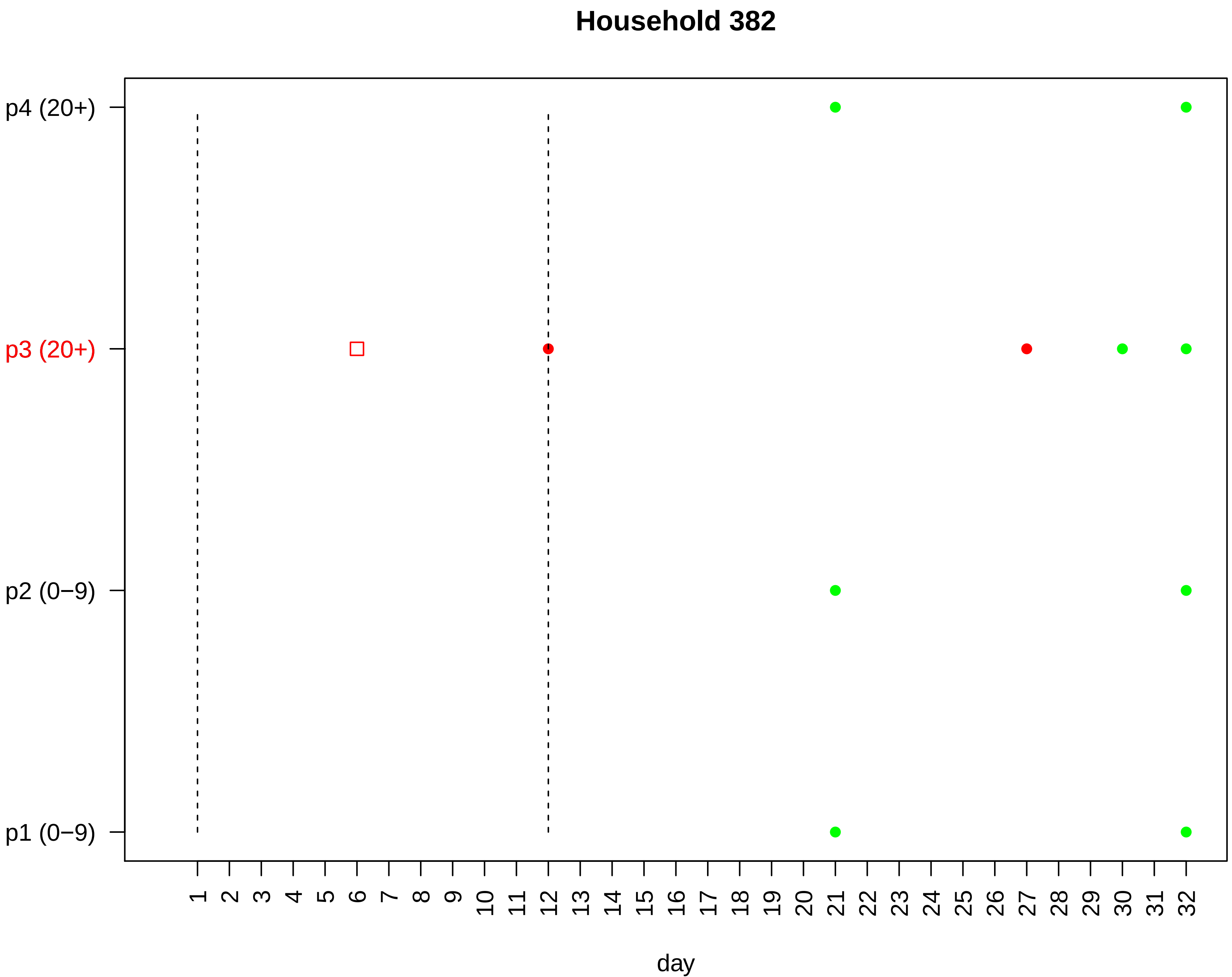

Household 382
p4 (20+)
p3 (20+)
p2 (0−9)
p1 (0−9)
1
2
3
4
5
6
7
8
9
10
11
12
13
14
15
16
17
18
19
20
21
22
23
24
25
26
27
28
29
30
31
32
day

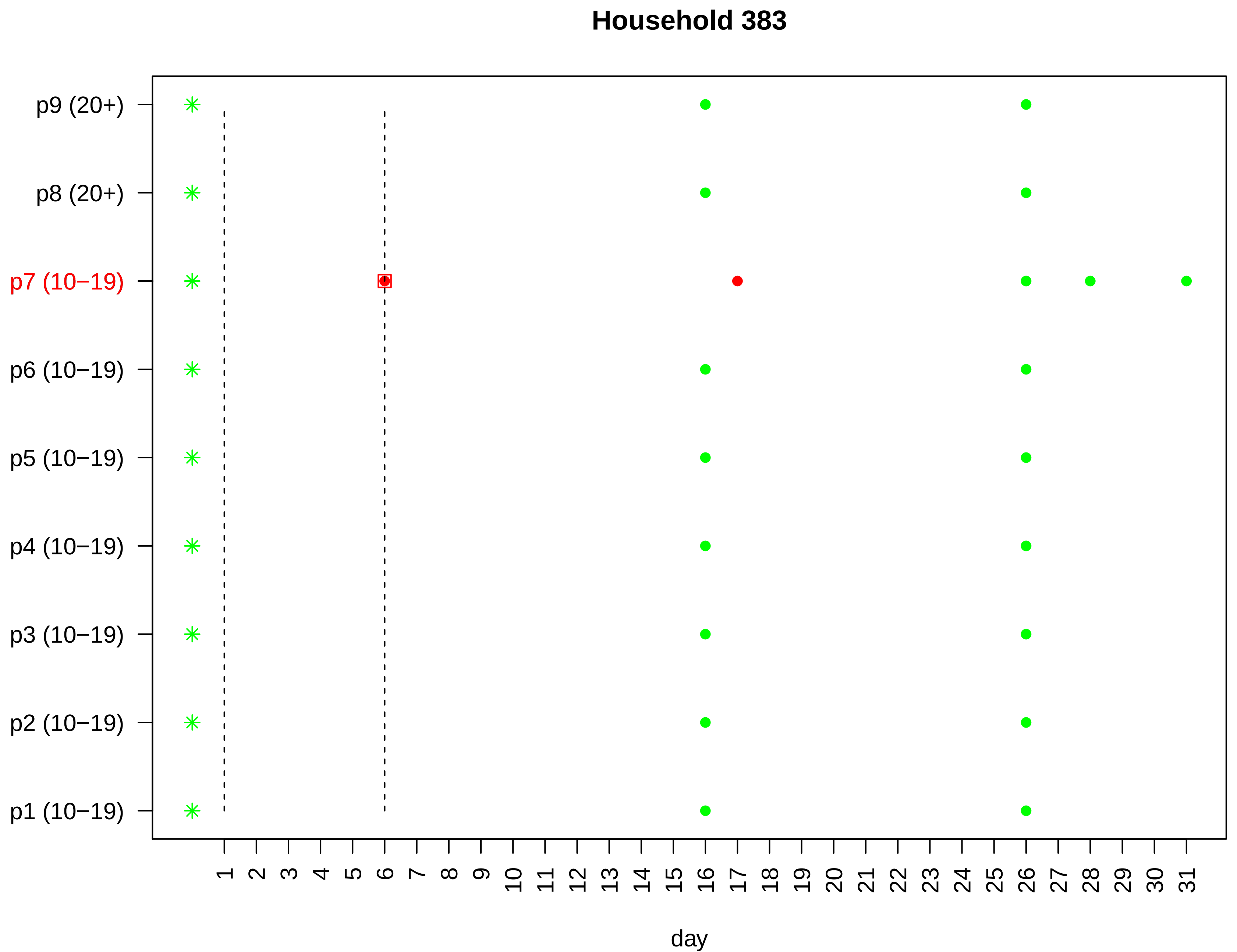

Household 383
p9 (20+)
p8 (20+)
p7 (10−19)
p6 (10−19)
p5 (10−19)
p4 (10−19)
p3 (10−19)
p2 (10−19)
p1 (10−19)
1 2 3 4 5 6 7 8 9 10 11 12 13 14 15 16 17 18 19 20 21 22 23 24 25 26 27 28 29 30 31
day

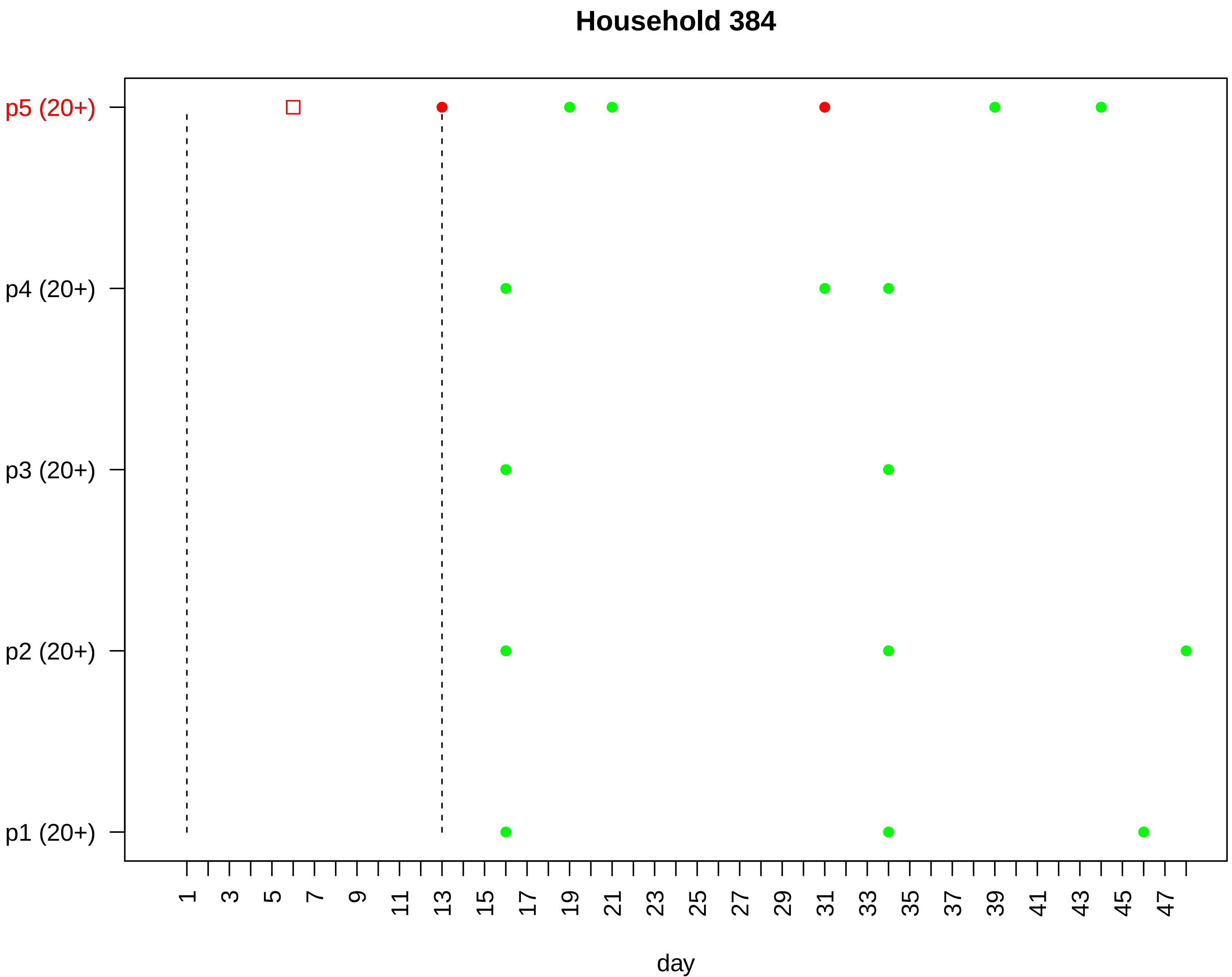

Household 384
p5 (20+)
p4 (20+)
p3 (20+)
p2 (20+)
p1 (20+)
1
3
5
7
9
11
13
15
17
19
21
23
25
27
29
31
33
35
37
39
41
43
45
47
day

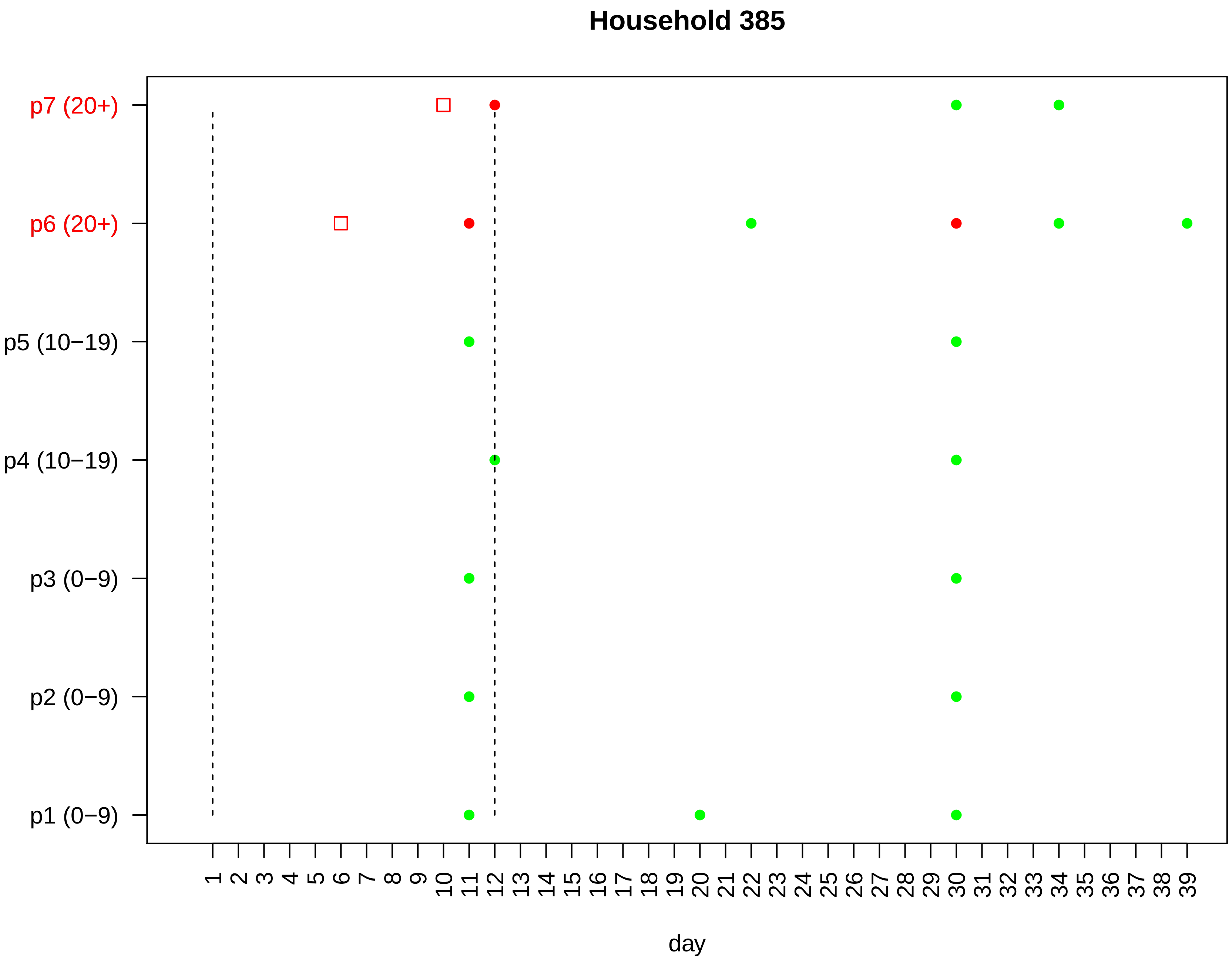
Household 385
p7 (20+)
p6 (20+)
p5 (10−19)
p4 (10−19)
p3 (0−9)
p2 (0−9)
p1 (0−9)
1 2 3 4 5 6 7 8 9 10 11 12 13 14 15 16 17 18 19 20 21 22 23 24 25 26 27 28 29 30 31 32 33 34 35 36 37 38 39
day

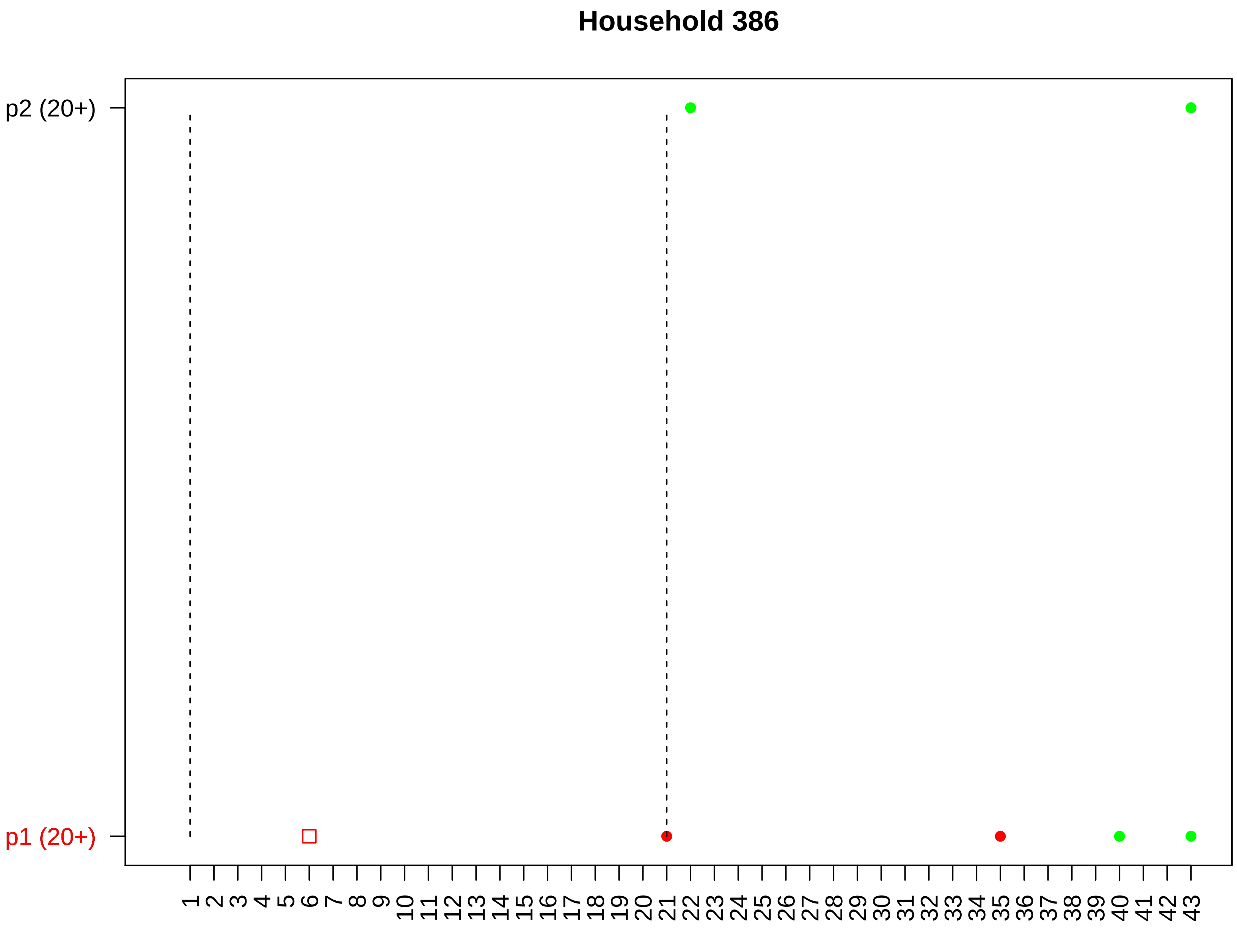

Household 386
p2 (20+)
p1 (20+)
1
2
3
4
5
6
7
8
9
10
11
12
13
14
15
16
17
18
19
20
21
22
23
24
25
26
27
28
29
30
31
32
33
34
35
36
37
38
39
40
41
42
43
day

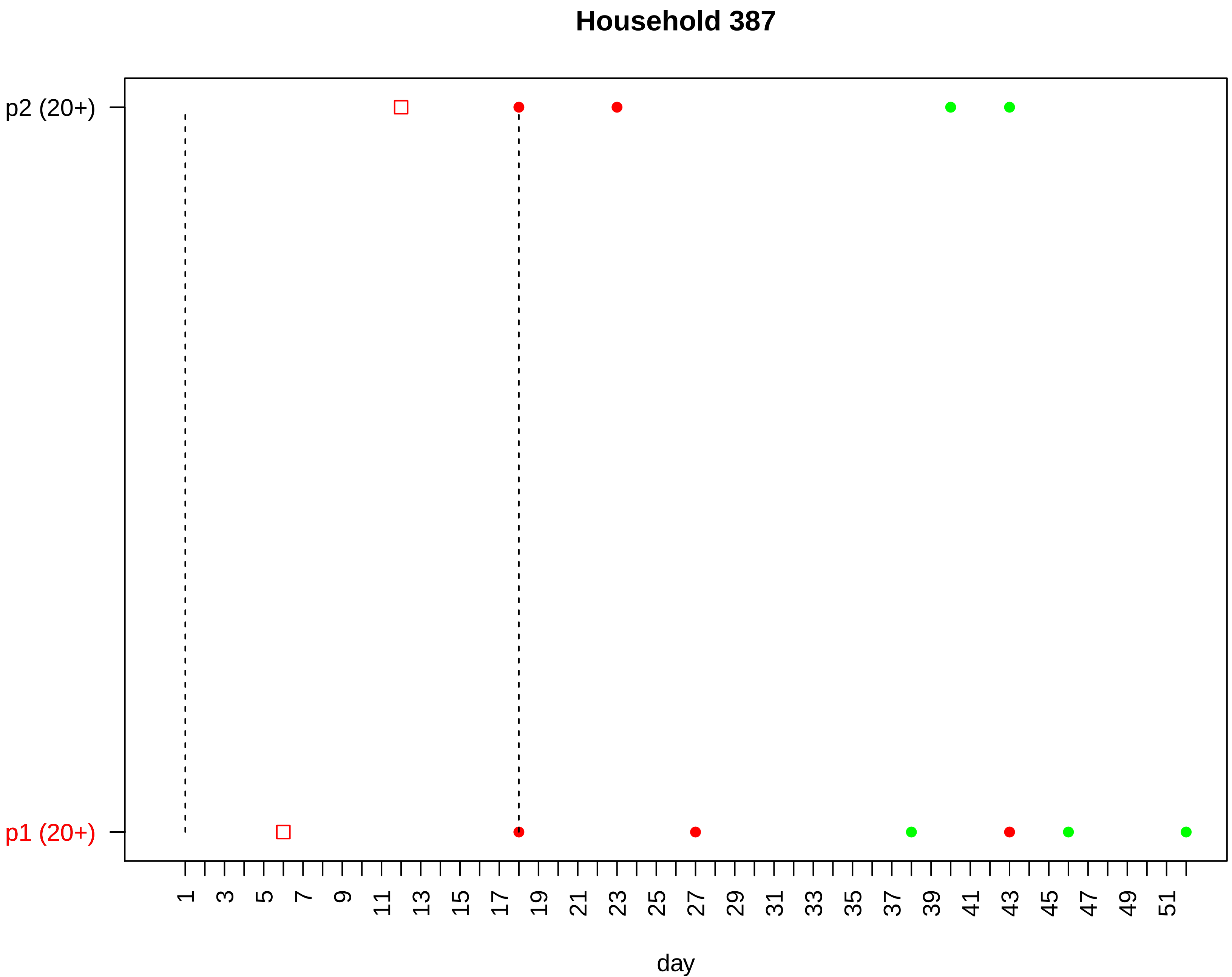

Household 387
p2 (20+)
p1 (20+)
1
3
5
7
9
11
13
15
17
19
21
23
25
27
29
31
33
35
37
39
41
43
45
47
49
51
day

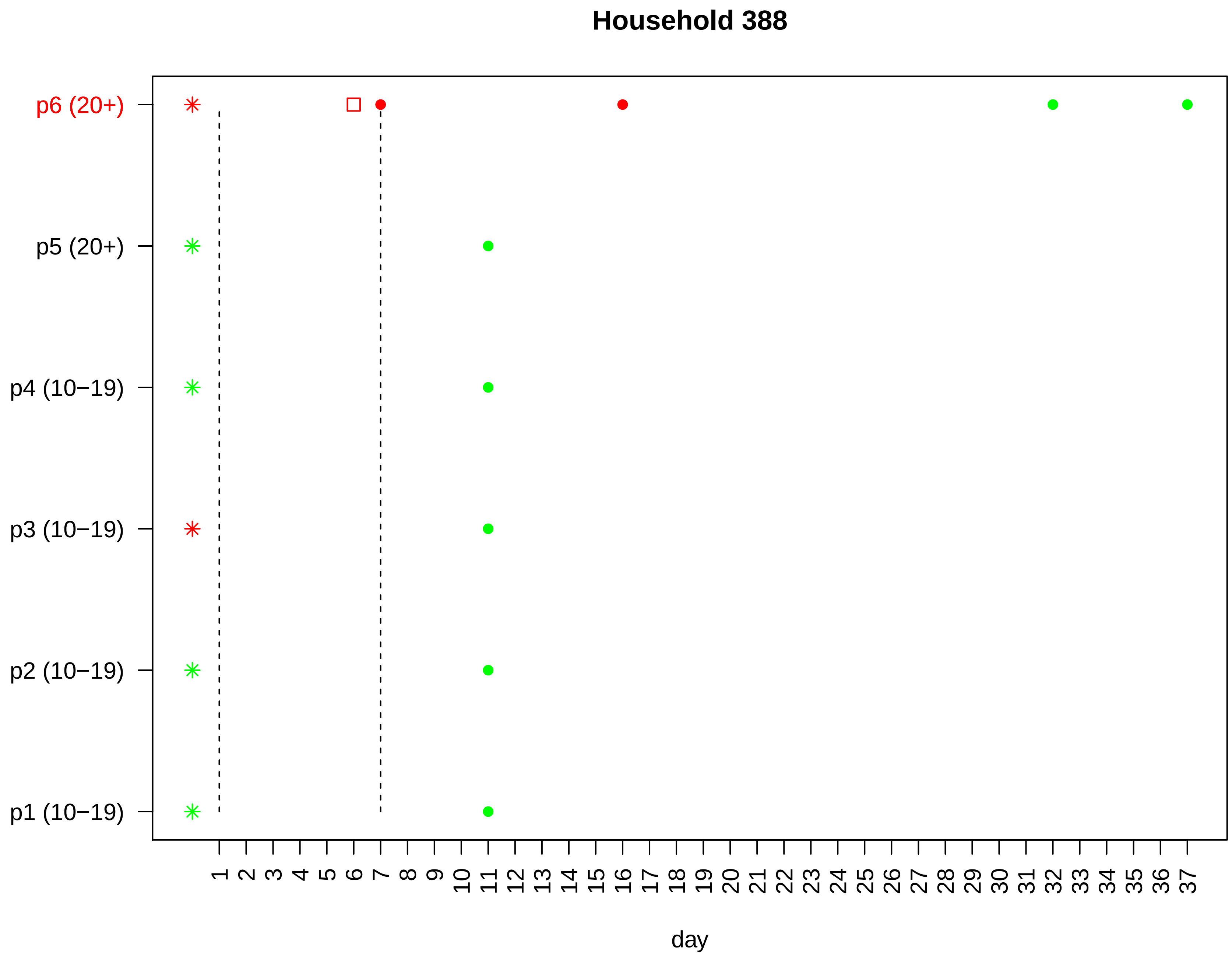

Household 388
p6 (20+)
p5 (20+)
p4 (10−19)
p3 (10−19)
p2 (10−19)
p1 (10−19)
1 2 3 4 5 6 7 8 9 10 11 12 13 14 15 16 17 18 19 20 21 22 23 24 25 26 27 28 29 30 31 32 33 34 35 36 37
day

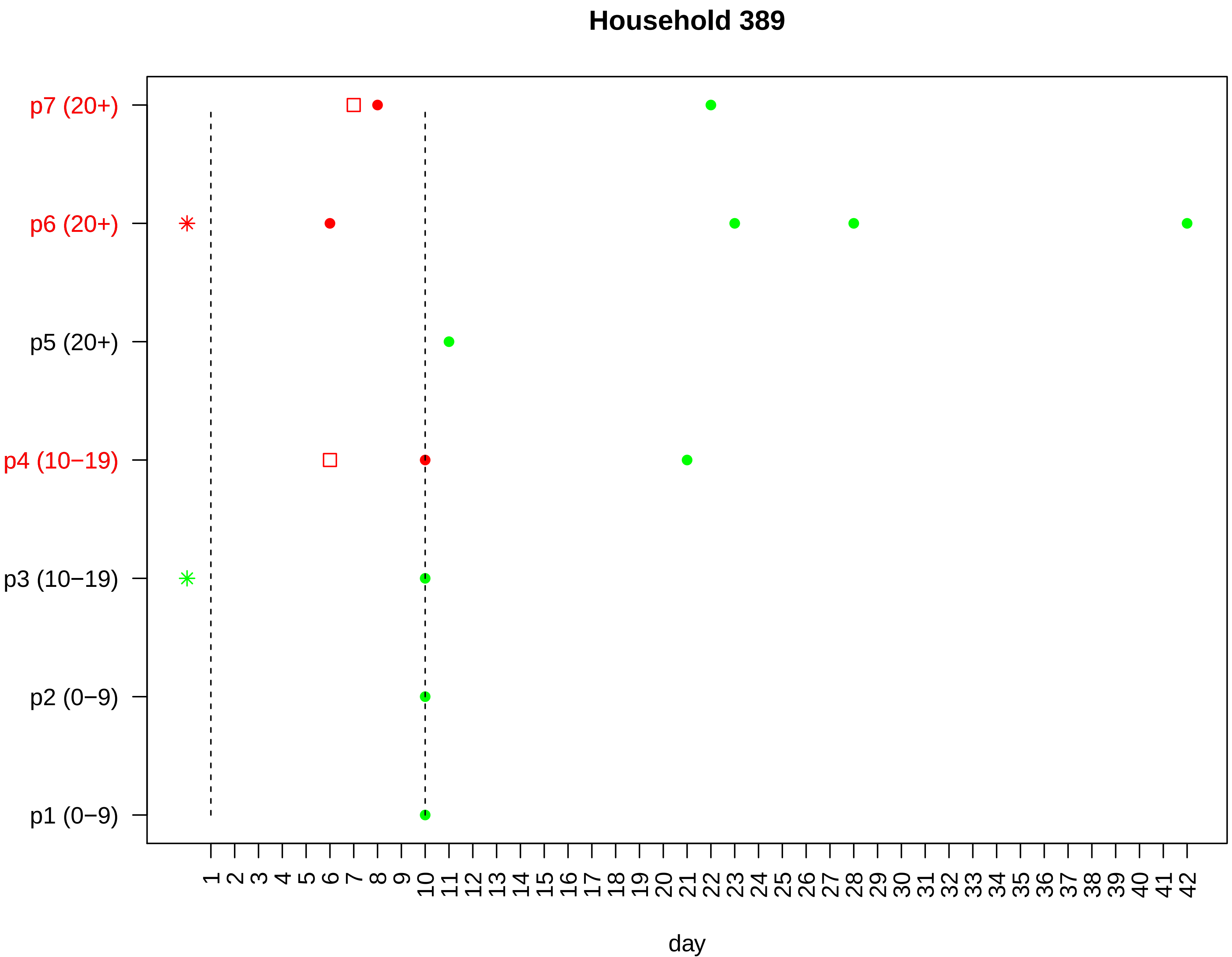
Household 389
p7 (20+)
p6 (20+)
p5 (20+)
p4 (10−19)
p3 (10−19)
p2 (0−9)
p1 (0−9)
1 2 3 4 5 6 7 8 9 10 11 12 13 14 15 16 17 18 19 20 21 22 23 24 25 26 27 28 29 30 31 32 33 34 35 36 37 38 39 40 41 42
day

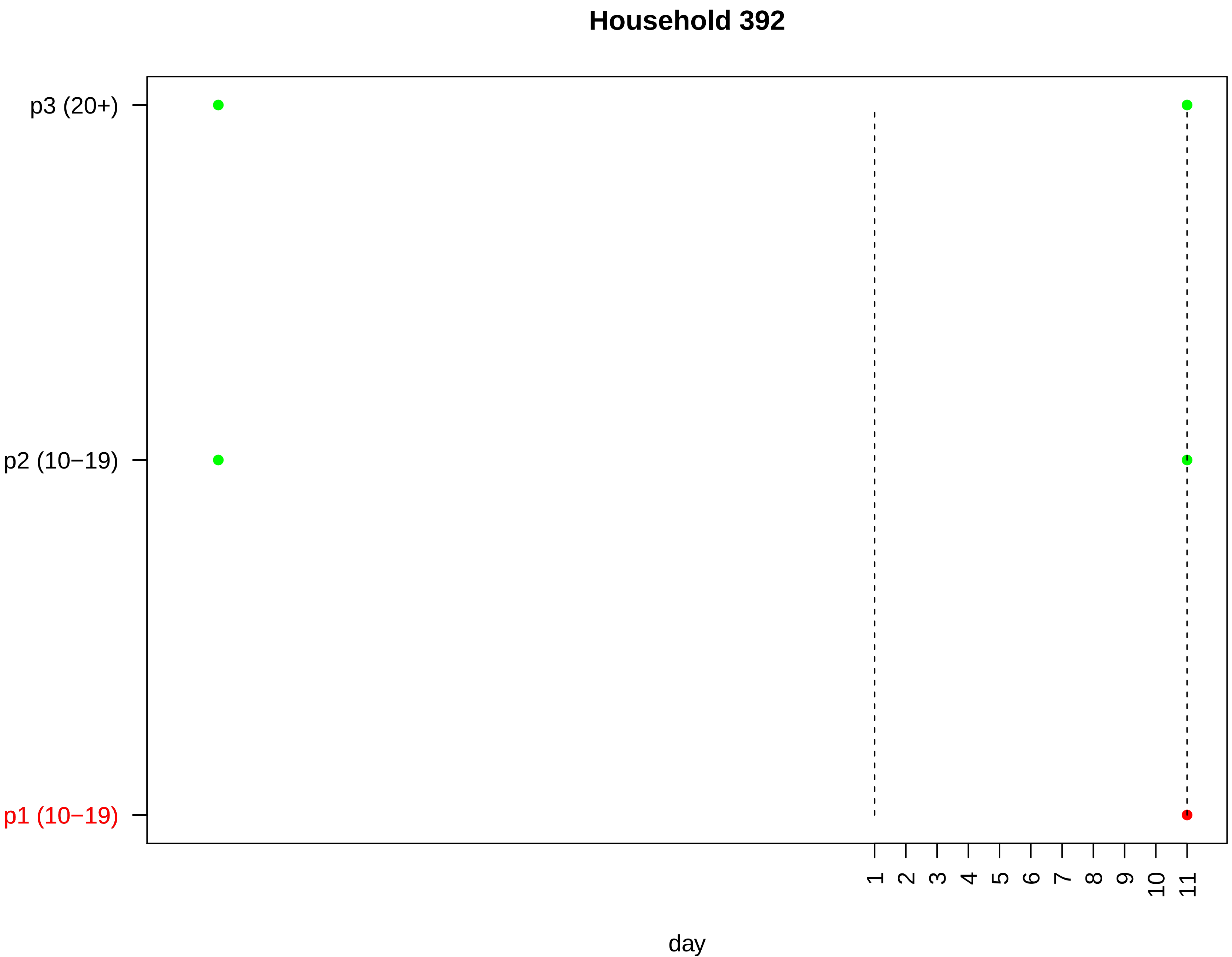

Household 392
p3 (20+)
p2 (10−19)
p1 (10−19)
1
2
3
4
5
6
7
8
9
10
11
day

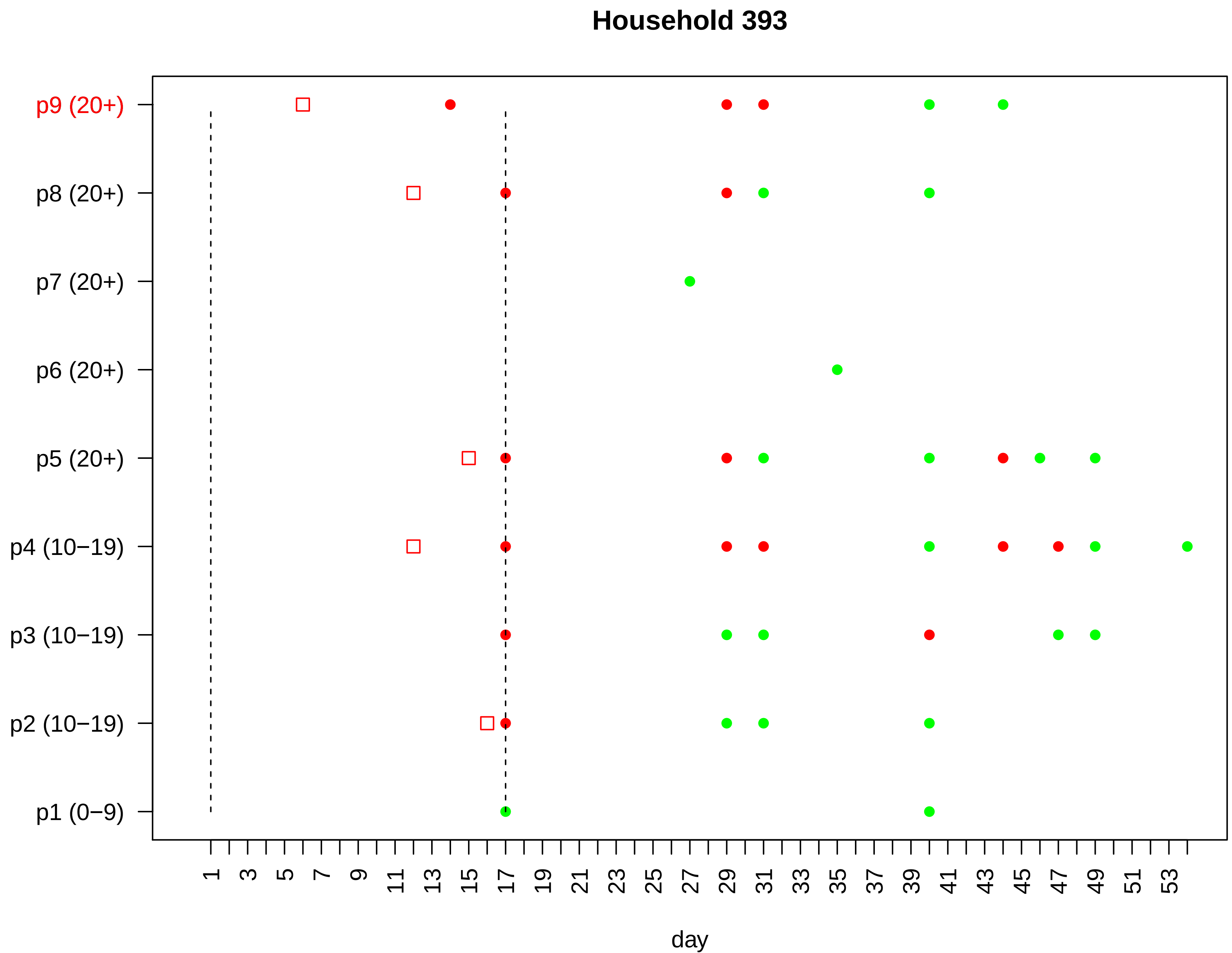
Household 393
p9 (20+)
p8 (20+)
p7 (20+)
p6 (20+)
p5 (20+)
p4 (10−19)
p3 (10−19)
p2 (10−19)
p1 (0−9)
1 3 5 7 9 11 13 15 17 19 21 23 25 27 29 31 33 35 37 39 41 43 45 47 49 51 53
day

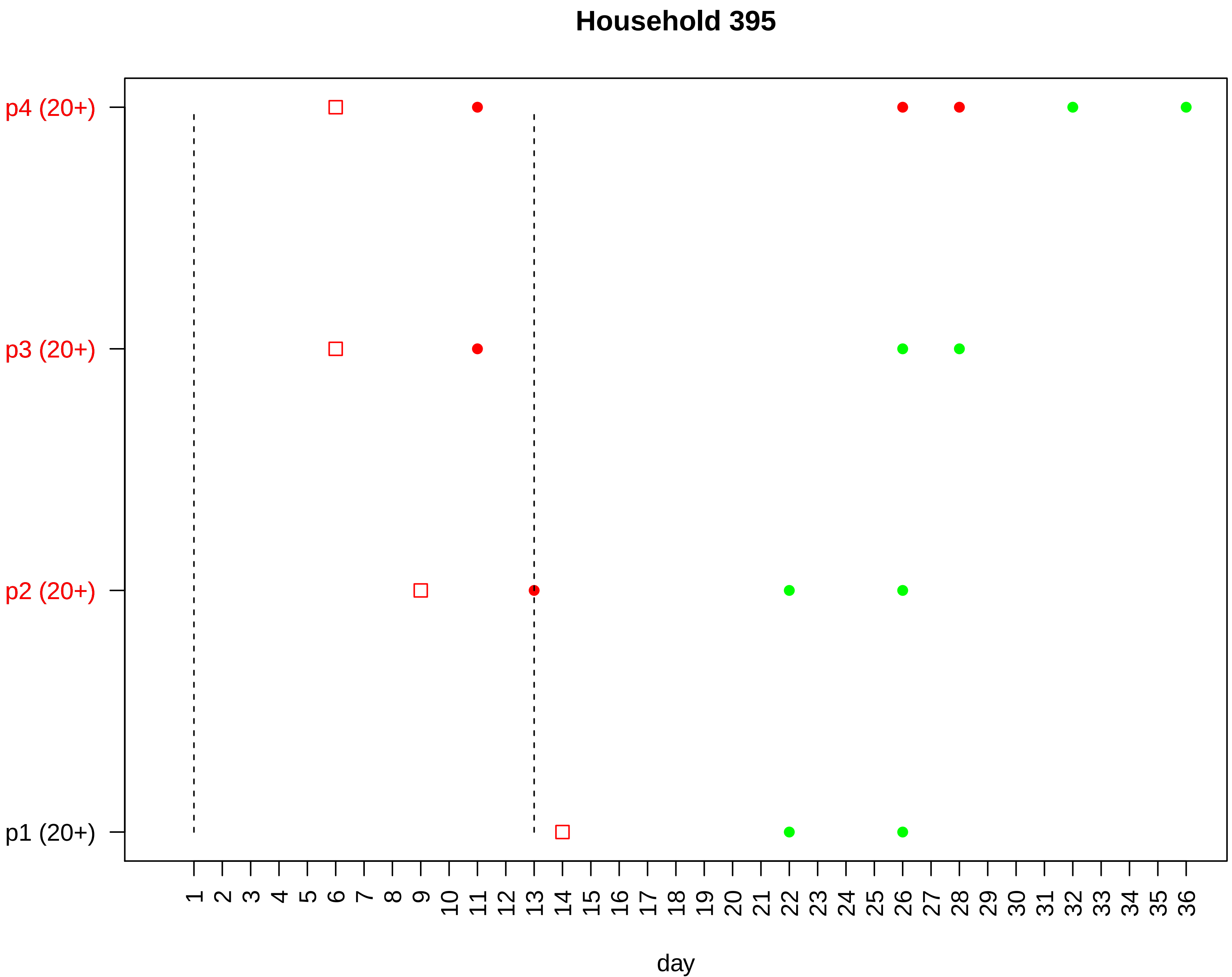

Household 395
p4 (20+)
p3 (20+)
p2 (20+)
p1 (20+)
1 2 3 4 5 6 7 8 9 10 11 12 13 14 15 16 17 18 19 20 21 22 23 24 25 26 27 28 29 30 31 32 33 34 35 36
day

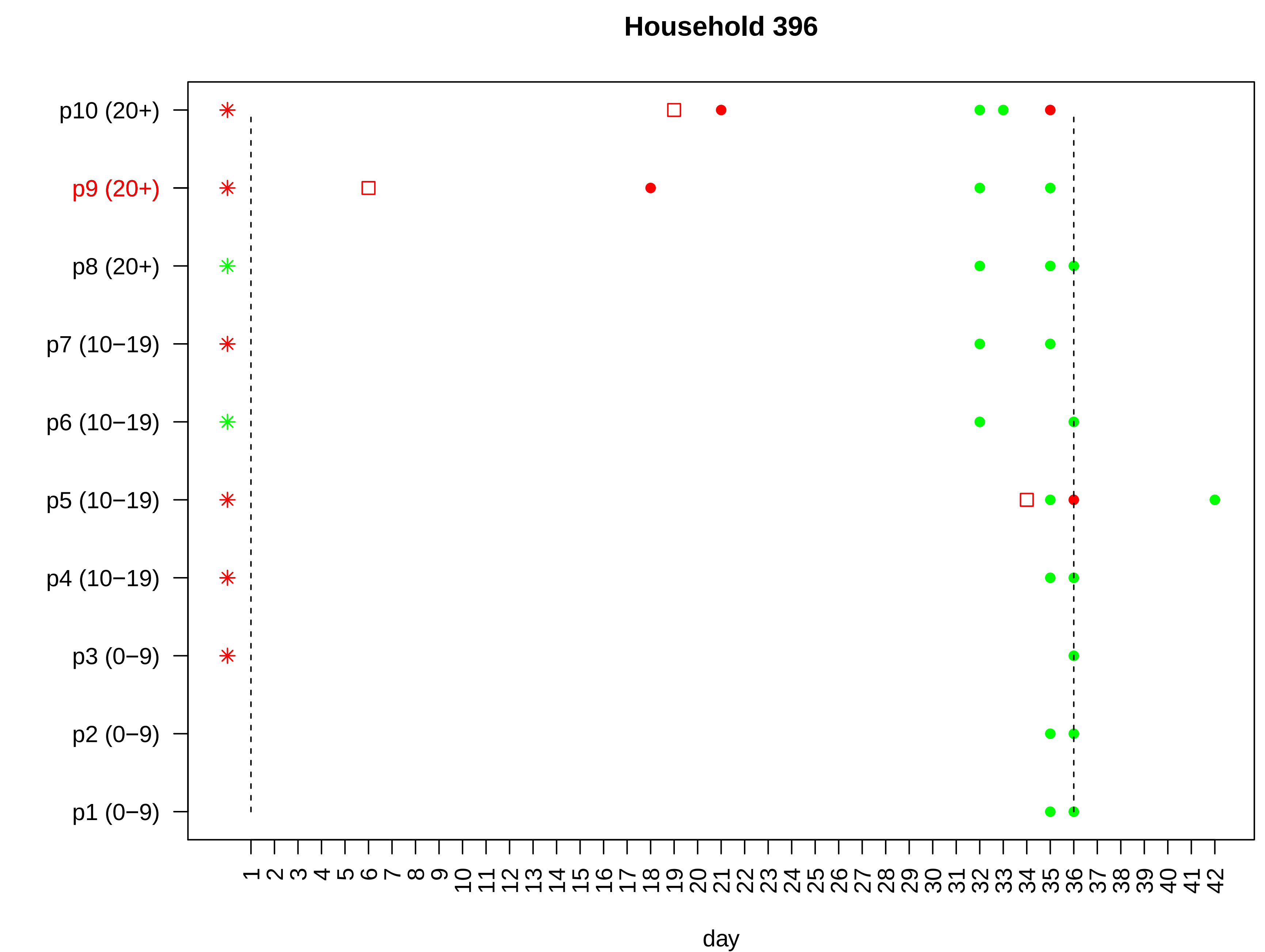

Household 396
p10 (20+)
p9 (20+)
p8 (20+)
p7 (10−19)
p6 (10−19)
p5 (10−19)
p4 (10−19)
p3 (0−9)
p2 (0−9)
p1 (0−9)
1 2 3 4 5 6 7 8 9 10 11 12 13 14 15 16 17 18 19 20 21 22 23 24 25 26 27 28 29 30 31 32 33 34 35 36 37 38 39 40 41 42
day

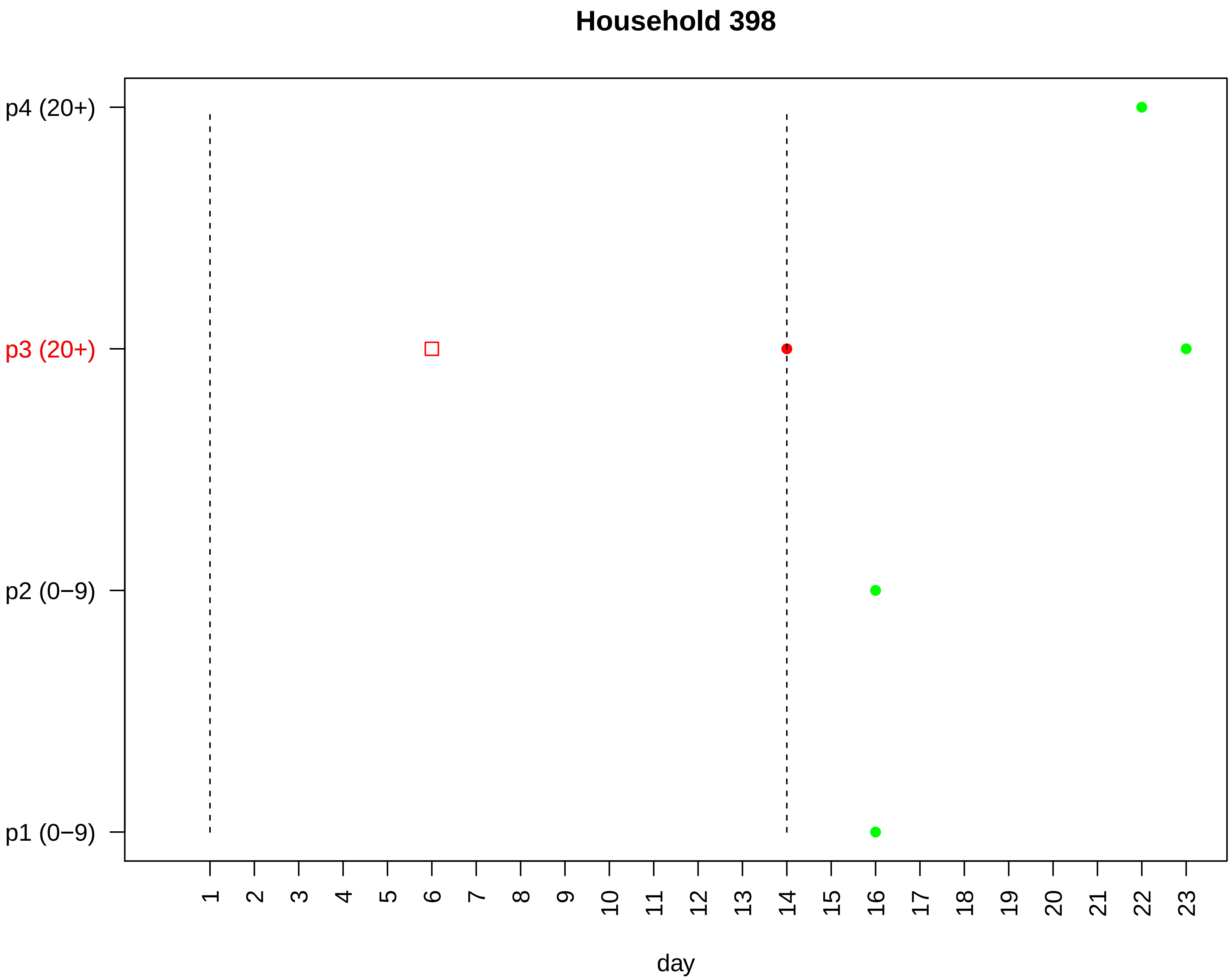

Household 398
p4 (20+)
p3 (20+)
p2 (0−9)
p1 (0−9)
1
2
3
4
5
6
7
8
9
10
11
12
13
14
15
16
17
18
19
20
21
22
23
day

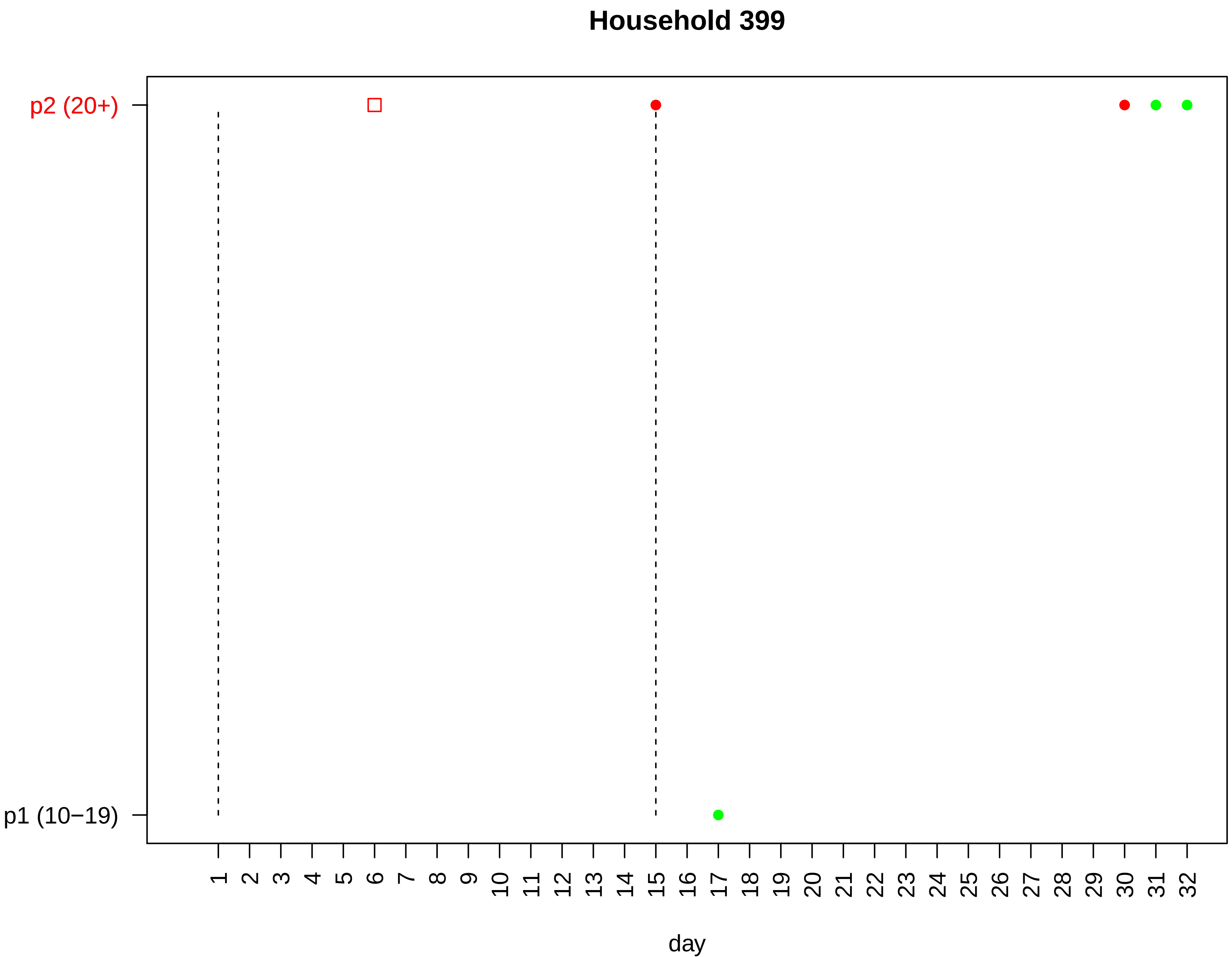
Household 399
p2 (20+)
p1 (10−19)
1 2 3 4 5 6 7 8 9 10 11 12 13 14 15 16 17 18 19 20 21 22 23 24 25 26 27 28 29 30 31 32
day

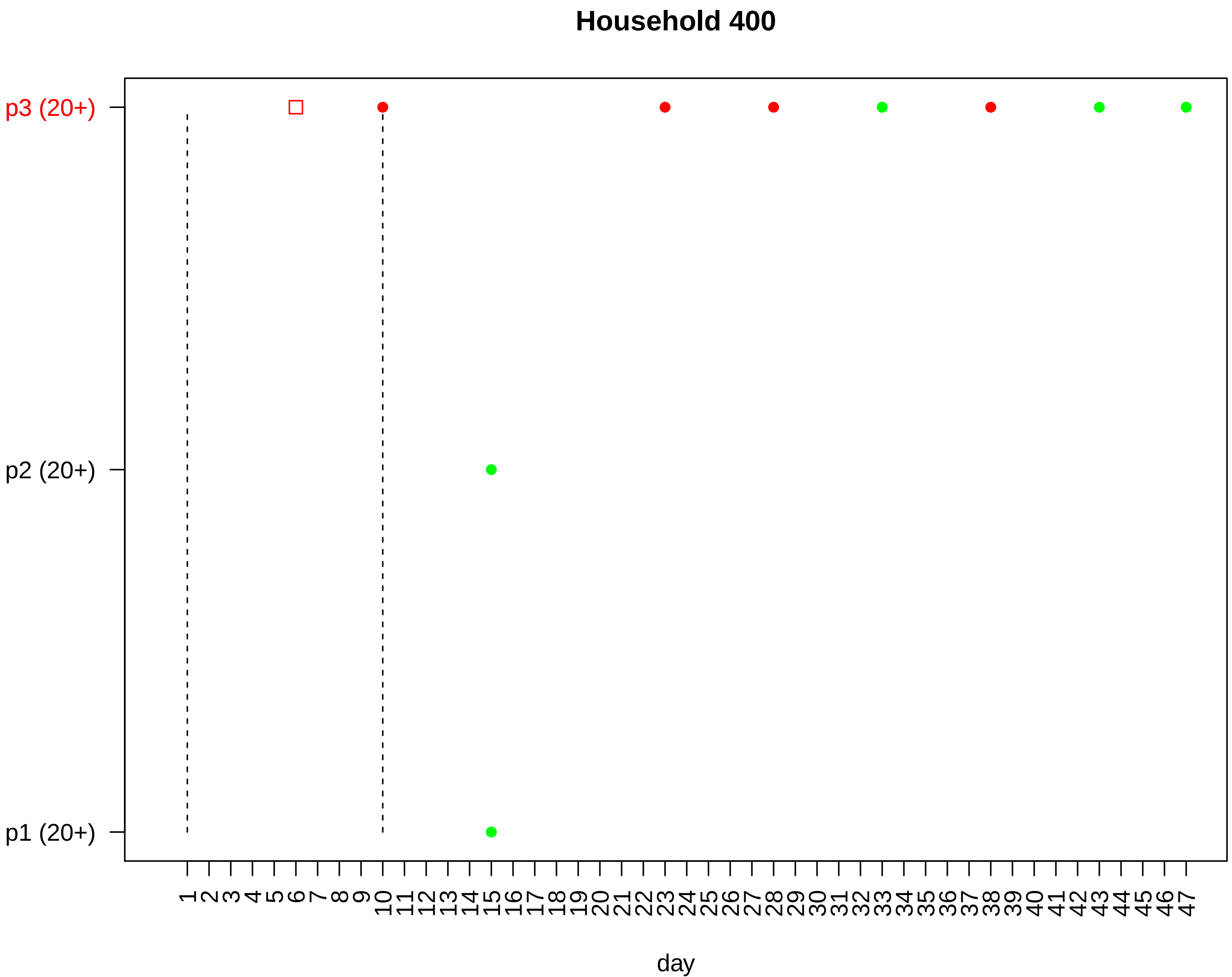
Household 400
p3 (20+)
p2 (20+)
p1 (20+)
1 2 3 4 5 6 7 8 9 10 11 12 13 14 15 16 17 18 19 20 21 22 23 24 25 26 27 28 29 30 31 32 33 34 35 36 37 38 39 40 41 42 43 44 45 46 47
day

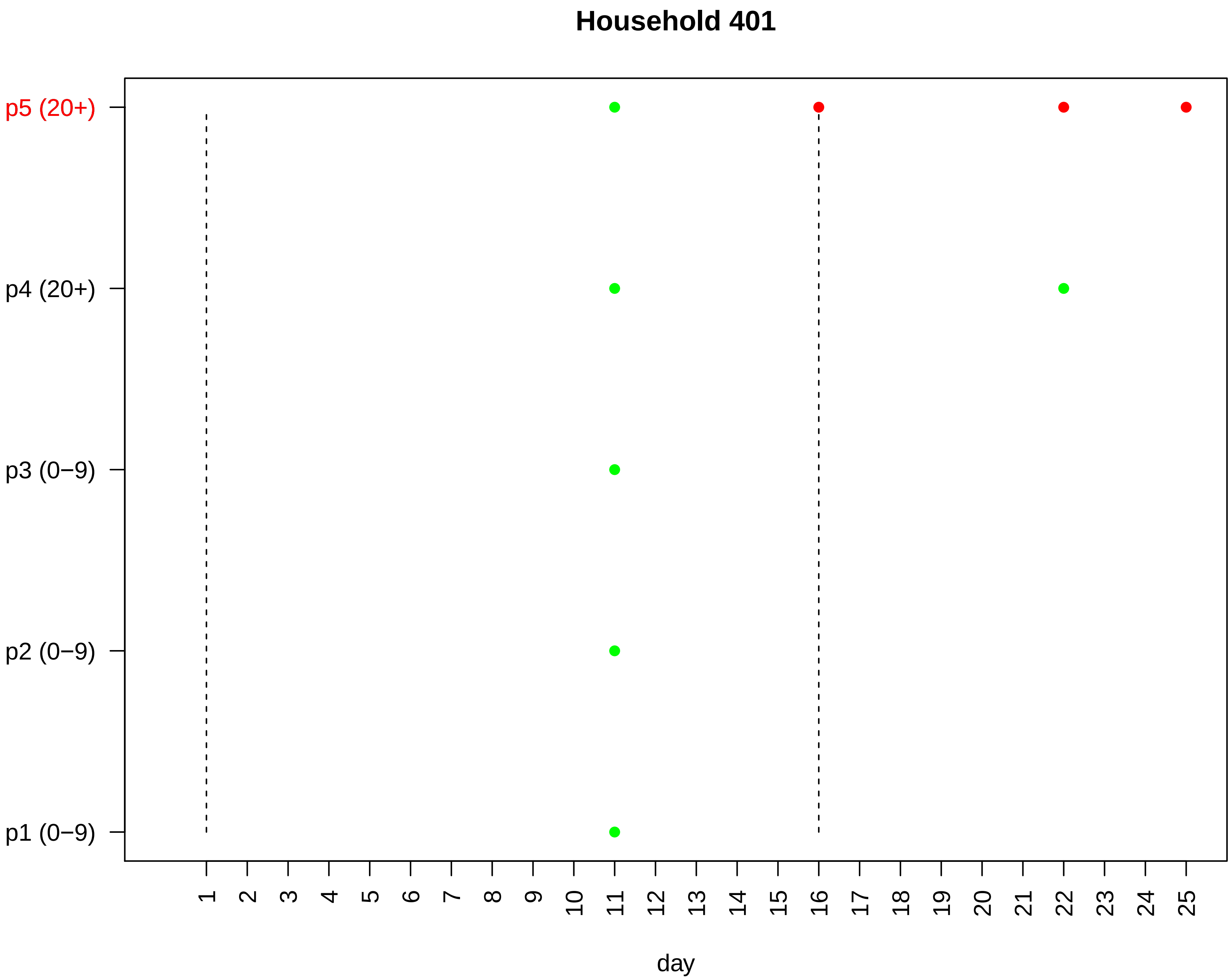
Household 401
p5 (20+)
p4 (20+)
p3 (0−9)
p2 (0−9)
p1 (0−9)
1 2 3 4 5 6 7 8 9 10 11 12 13 14 15 16 17 18 19 20 21 22 23 24 25
day

Household 402

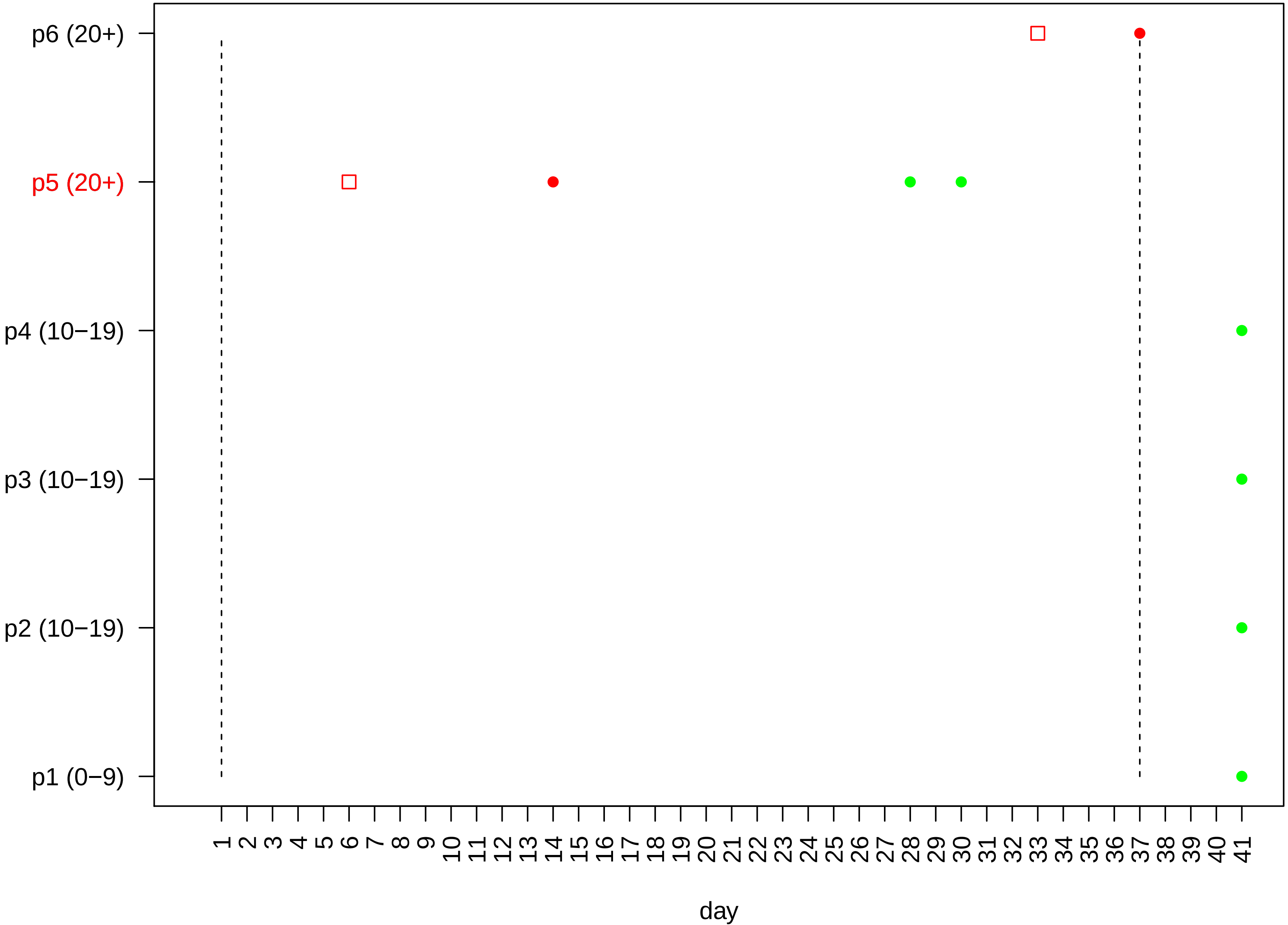

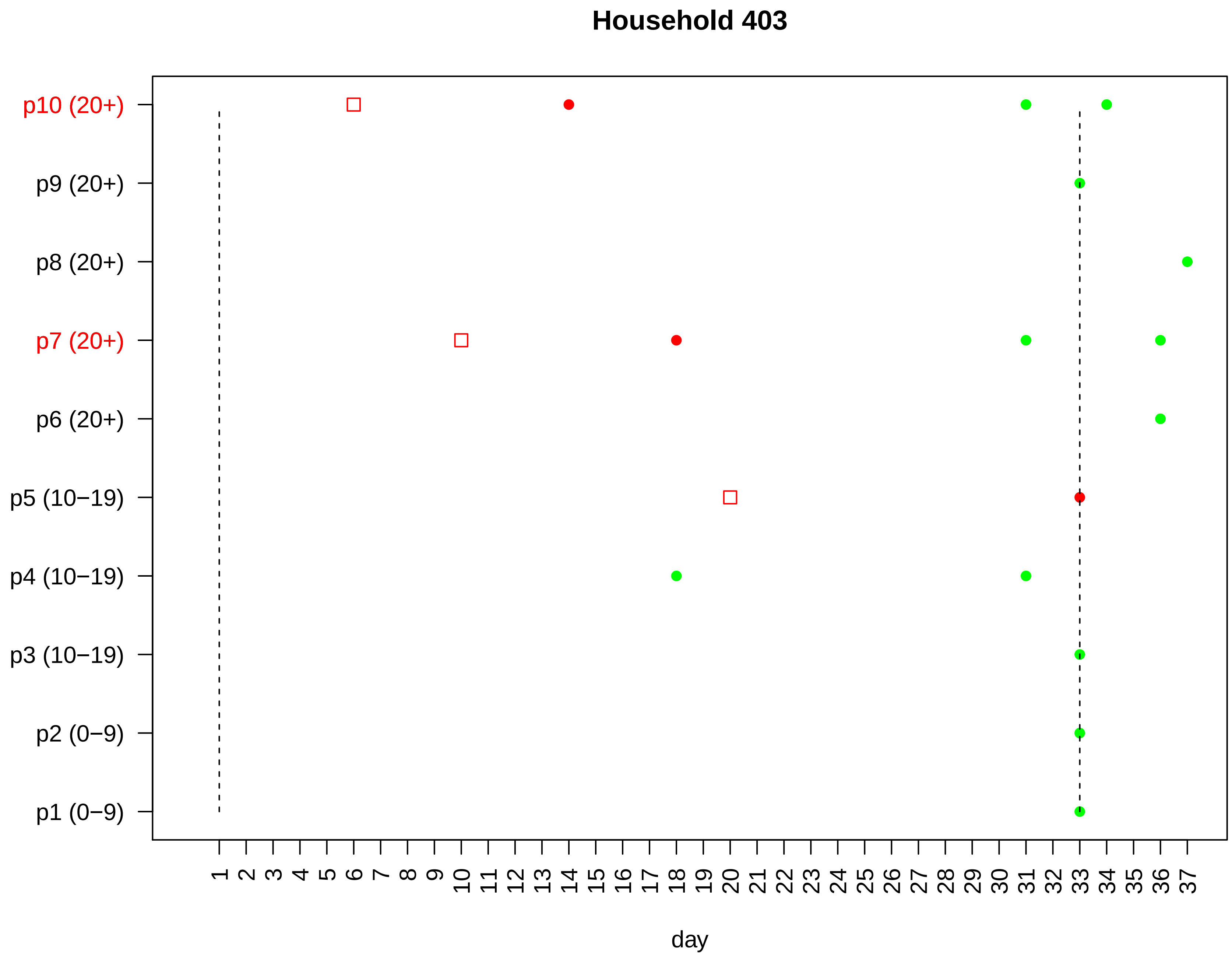
Household 403
p10 (20+)
p9 (20+)
p8 (20+)
p7 (20+)
p6 (20+)
p5 (10−19)
p4 (10−19)
p3 (10−19)
p2 (0−9)
p1 (0−9)
1 2 3 4 5 6 7 8 9 10 11 12 13 14 15 16 17 18 19 20 21 22 23 24 25 26 27 28 29 30 31 32 33 34 35 36 37
day

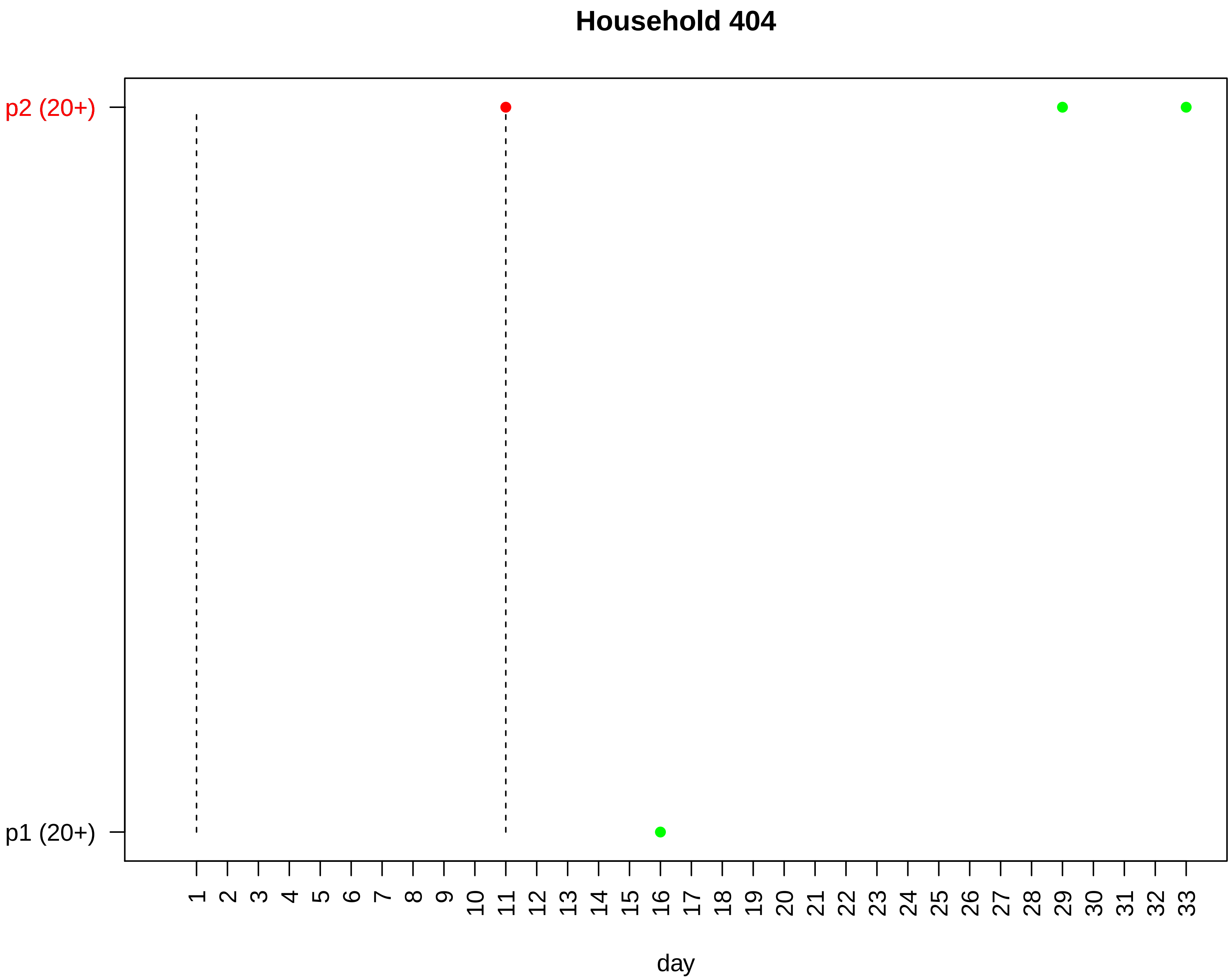
Household 404
p2 (20+)
p1 (20+)
1 2 3 4 5 6 7 8 9 10 11 12 13 14 15 16 17 18 19 20 21 22 23 24 25 26 27 28 29 30 31 32 33
day

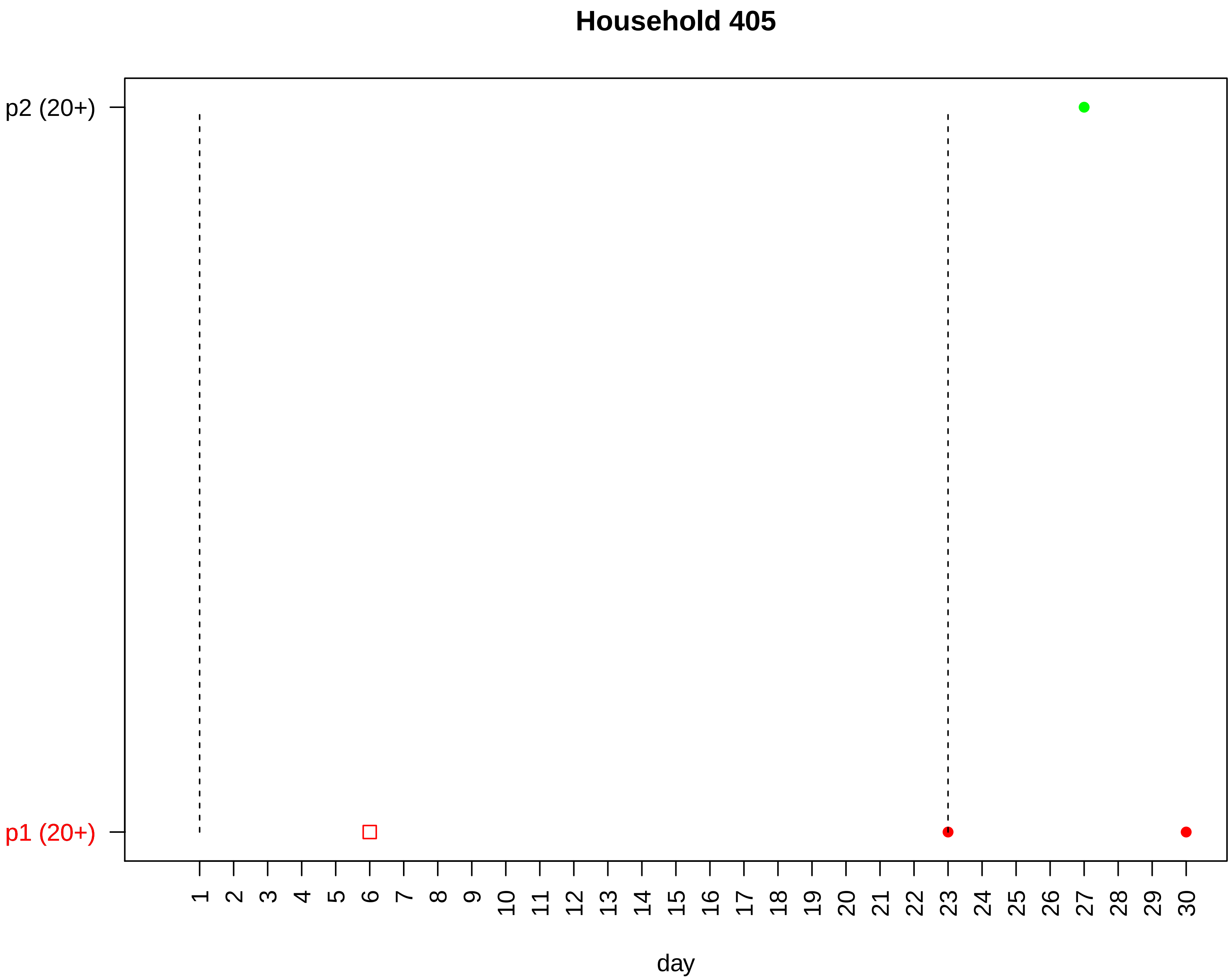

Household 405
p2 (20+)
p1 (20+)
1
2
3
4
5
6
7
8
9
10
11
12
13
14
15
16
17
18
19
20
21
22
23
24
25
26
27
28
29
30
day

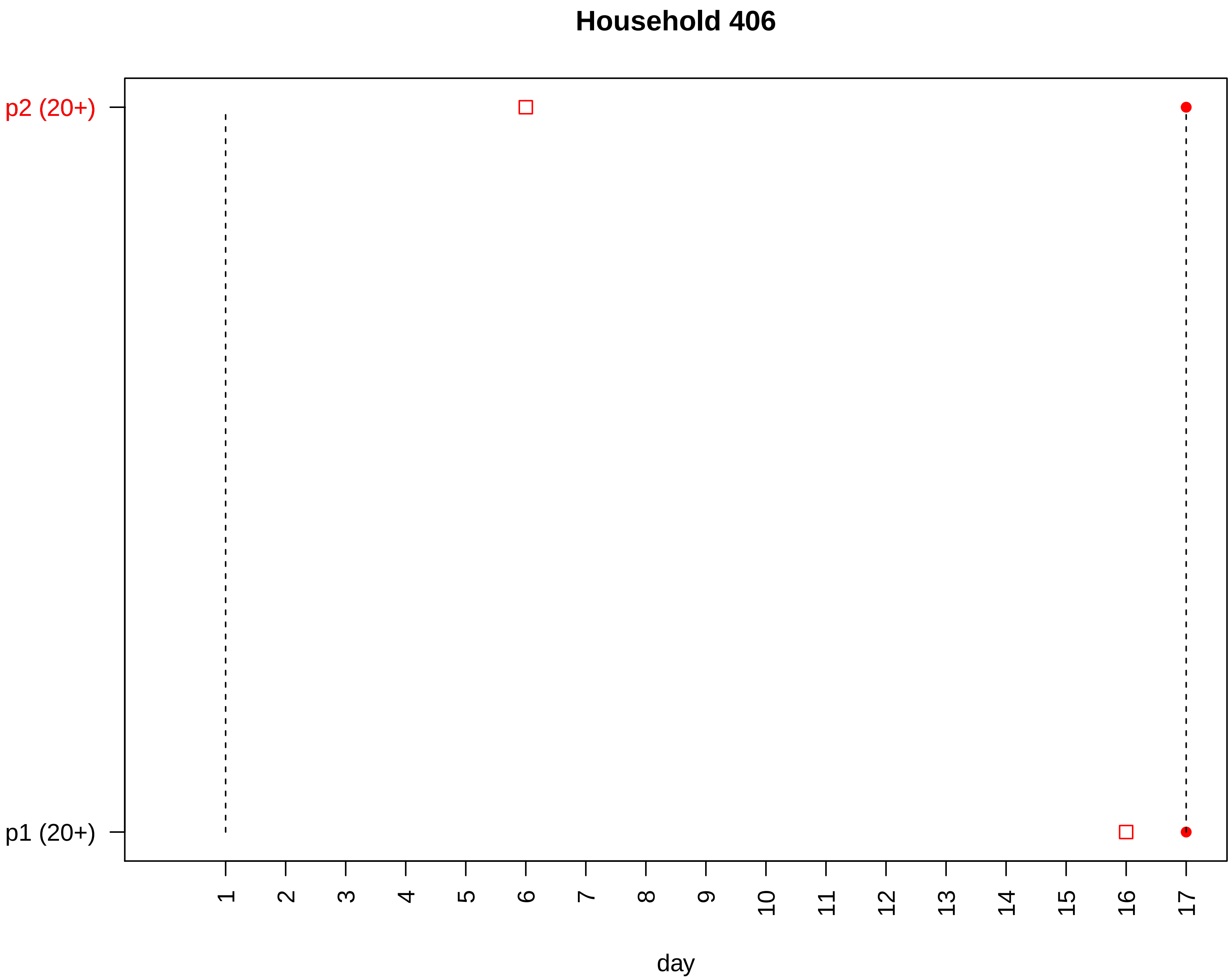

Household 406
p2 (20+)
p1 (20+)
1
2
3
4
5
6
7
8
9
10
11
12
13
14
15
16
17
day

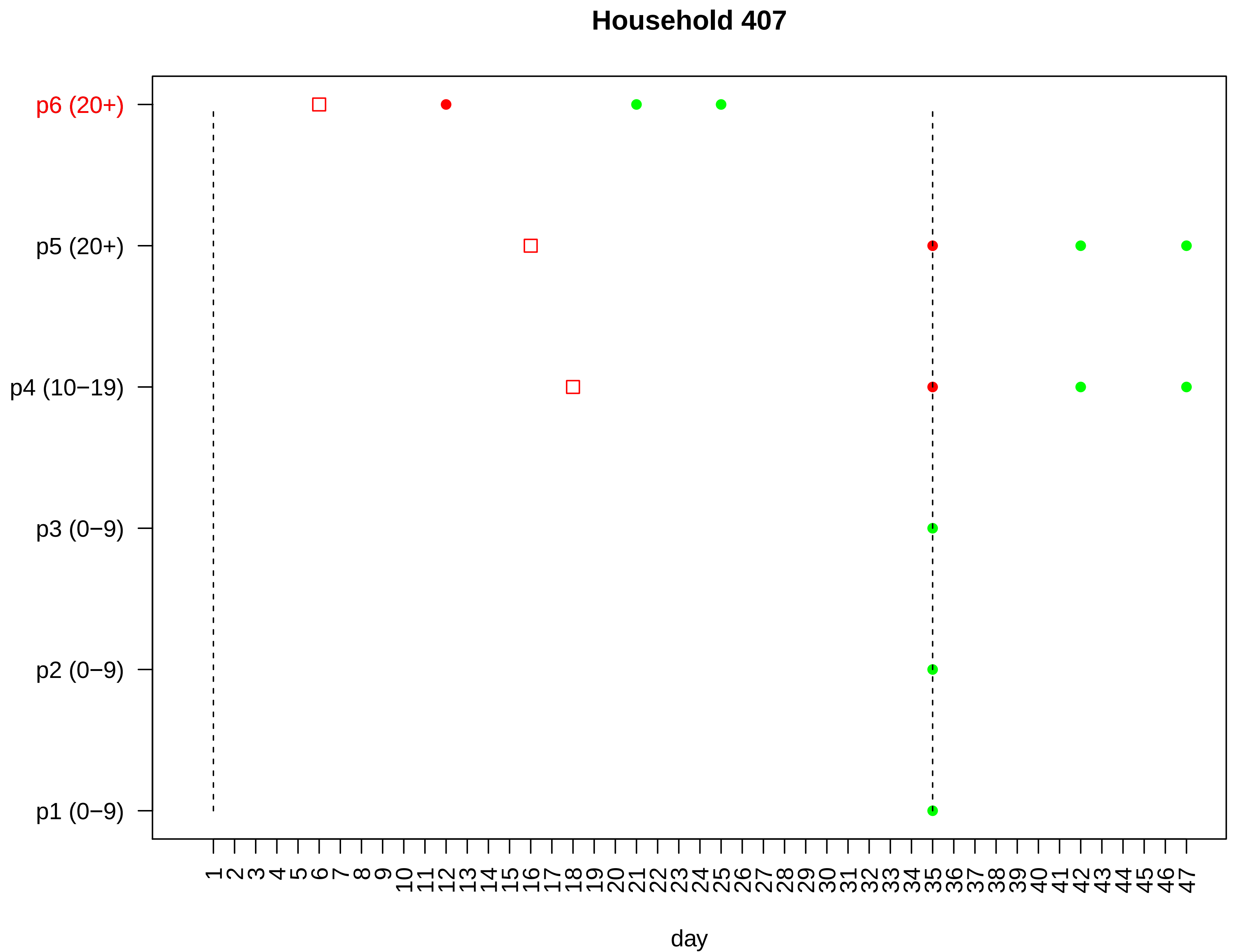

Household 407
p6 (20+)
p5 (20+)
p4 (10−19)
p3 (0−9)
p2 (0−9)
p1 (0−9)
1 2 3 4 5 6 7 8 9 10 11 12 13 14 15 16 17 18 19 20 21 22 23 24 25 26 27 28 29 30 31 32 33 34 35 36 37 38 39 40 41 42 43 44 45 46 47
day

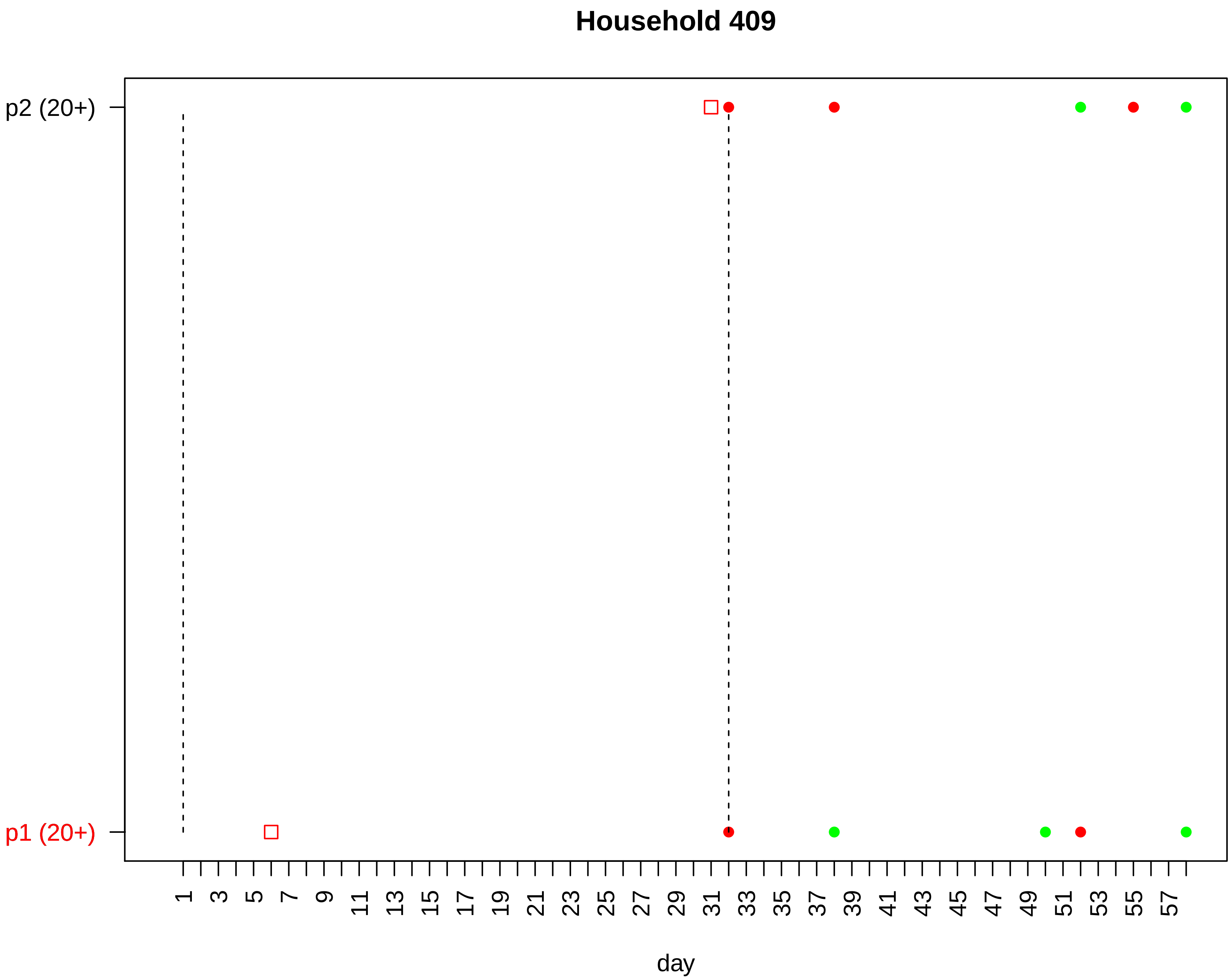

Household 409
p2 (20+)
p1 (20+)
1
3
5
7
9
11
13
15
17
19
21
23
25
27
29
31
33
35
37
39
41
43
45
47
49
51
53
55
57
day

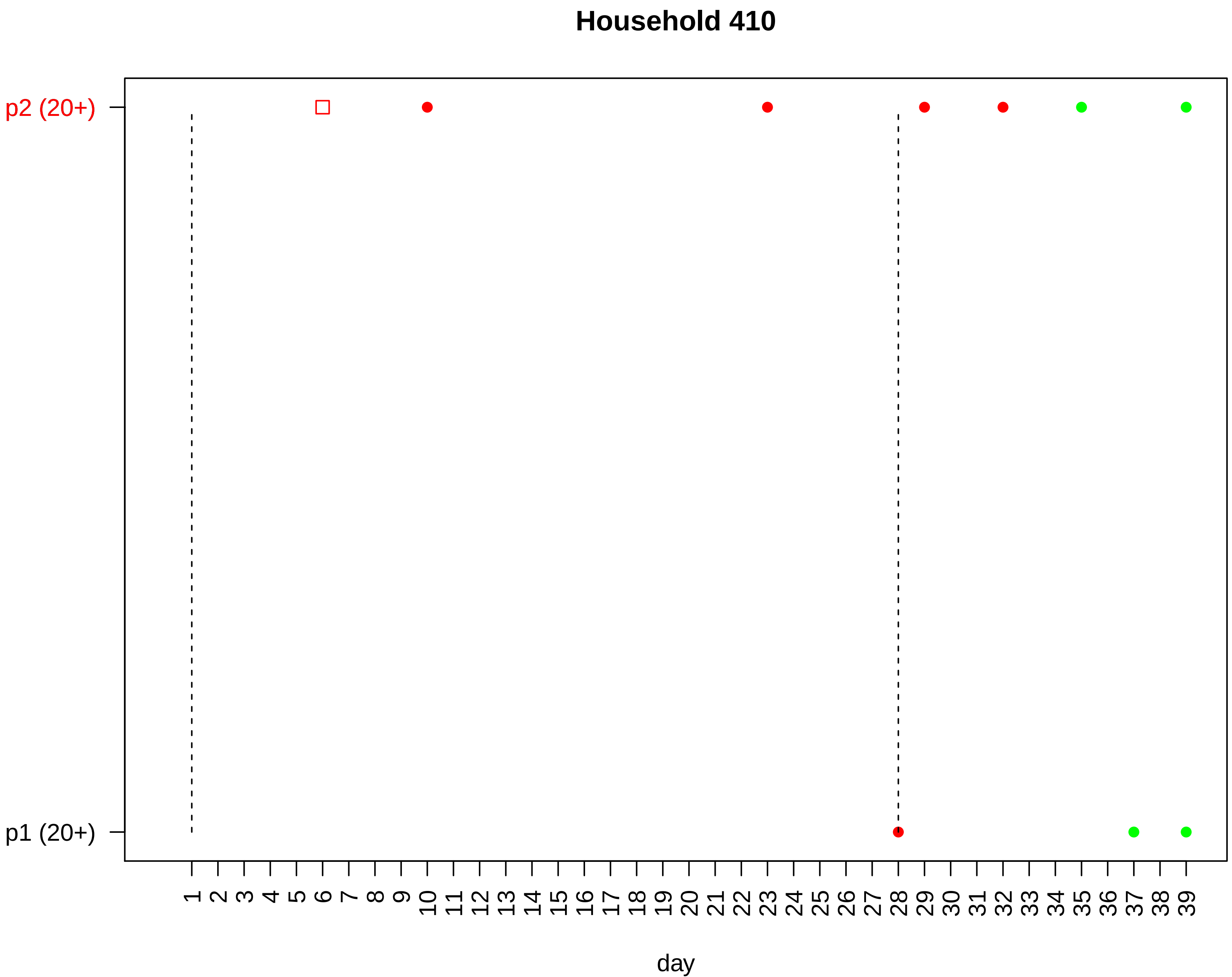
Household 410
p2 (20+)
p1 (20+)
1 2 3 4 5 6 7 8 9 10 11 12 13 14 15 16 17 18 19 20 21 22 23 24 25 26 27 28 29 30 31 32 33 34 35 36 37 38 39
day

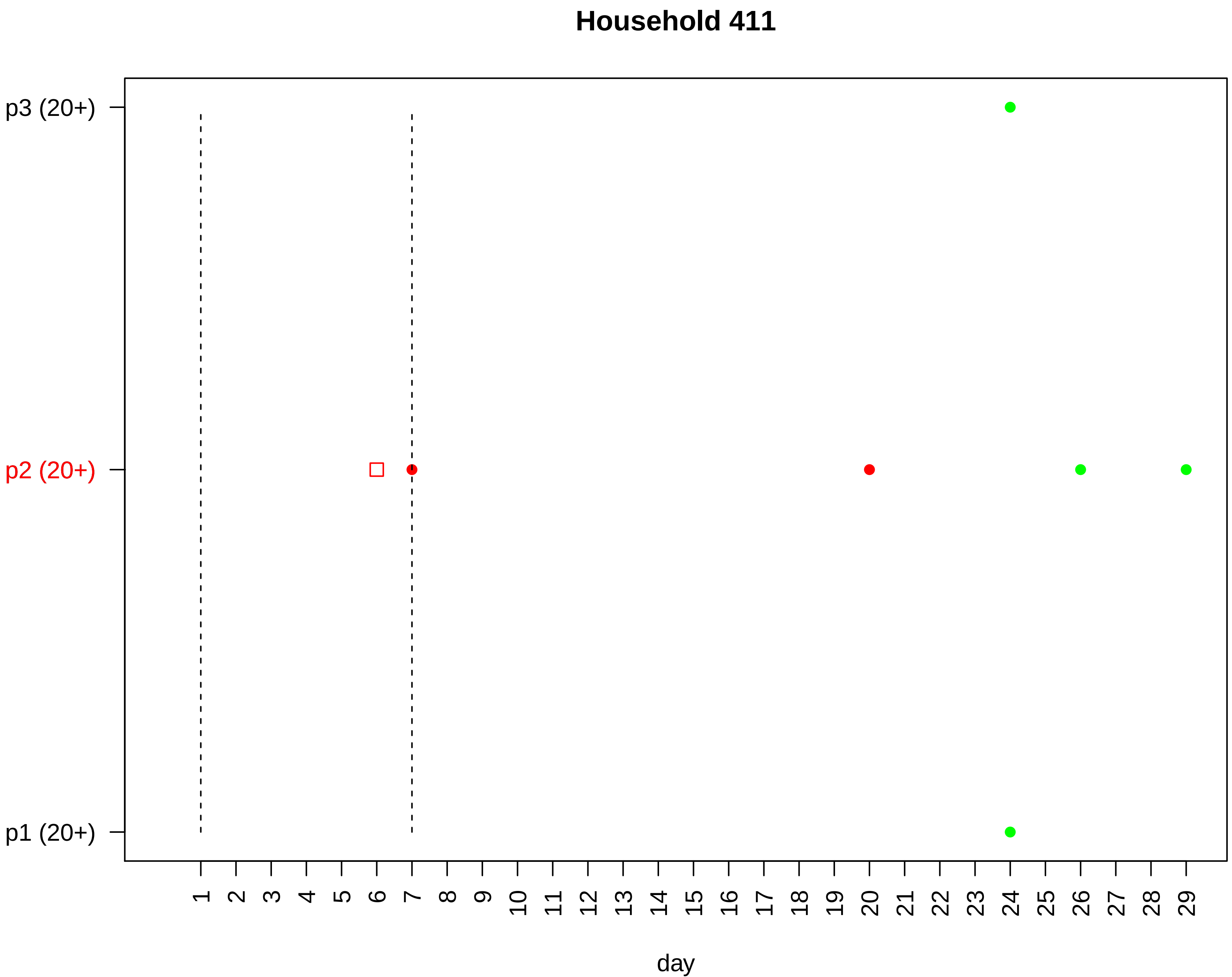

Household 411
p3 (20+)
p2 (20+)
p1 (20+)
1 2 3 4 5 6 7 8 9 10 11 12 13 14 15 16 17 18 19 20 21 22 23 24 25 26 27 28 29
day

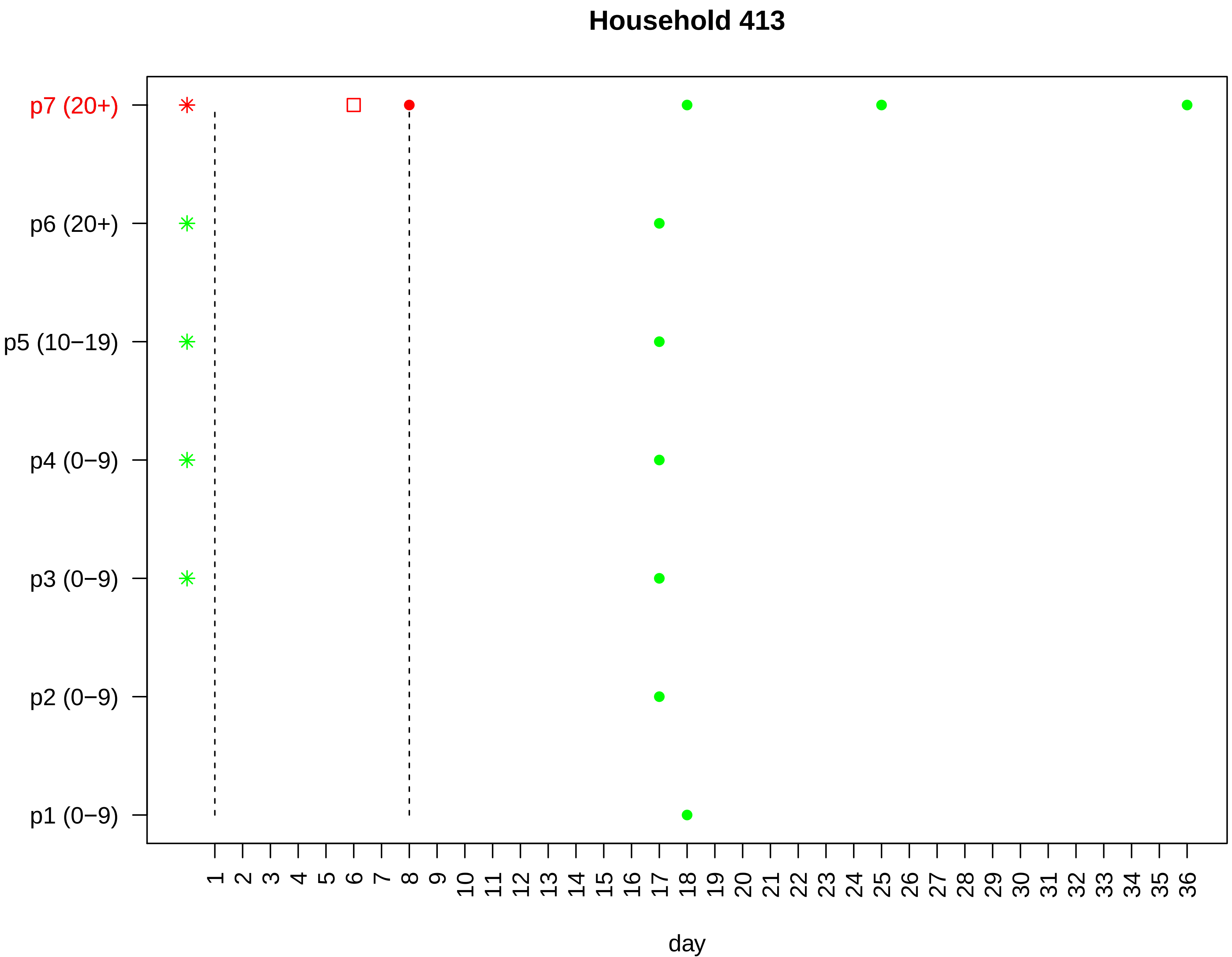
Household 413
p7 (20+)
p6 (20+)
p5 (10−19)
p4 (0−9)
p3 (0−9)
p2 (0−9)
p1 (0−9)
1 2 3 4 5 6 7 8 9 10 11 12 13 14 15 16 17 18 19 20 21 22 23 24 25 26 27 28 29 30 31 32 33 34 35 36
day

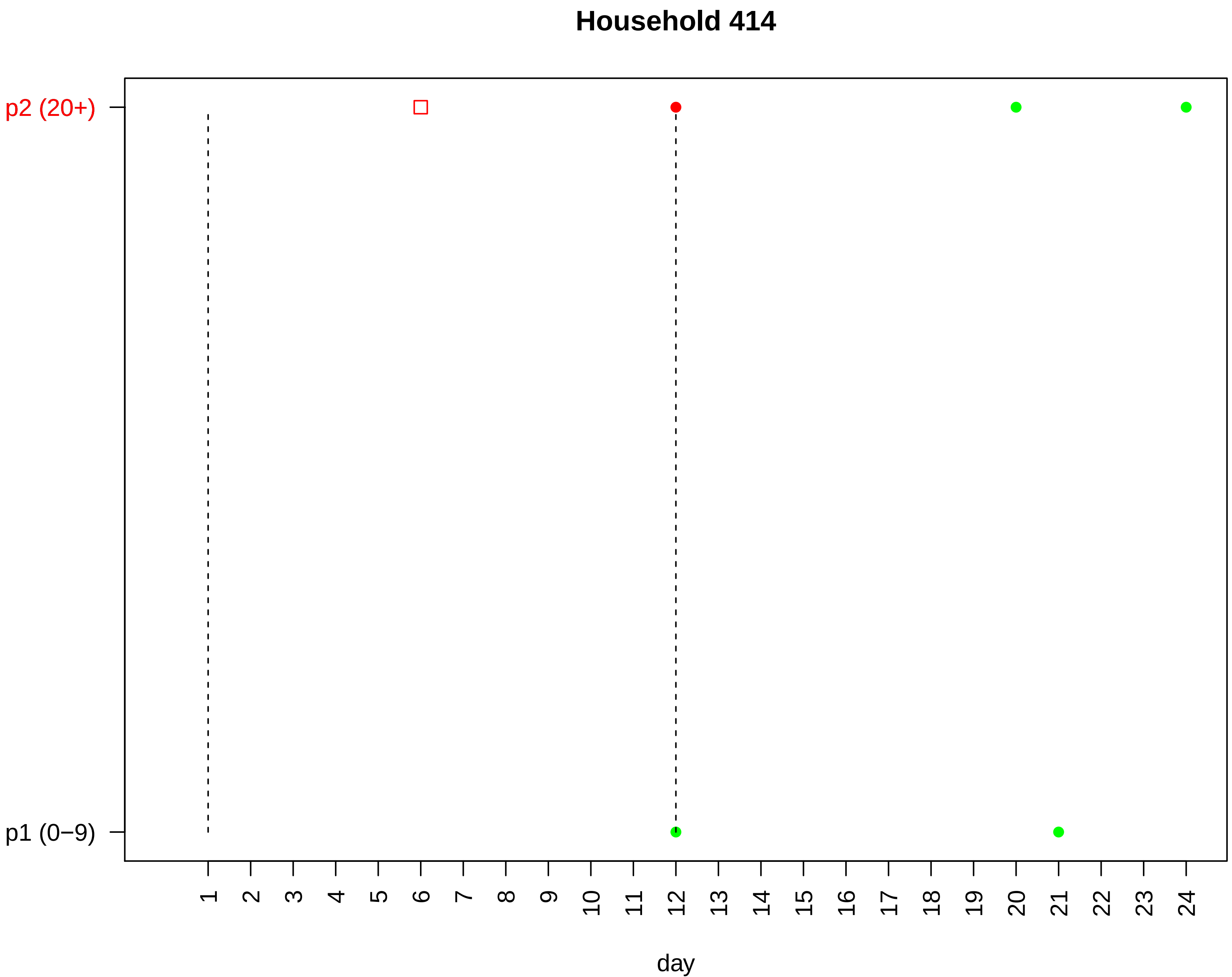
Household 414
p2 (20+)
p1 (0−9)
1
2
3
4
5
6
7
8
9
10
11
12
13
14
15
16
17
18
19
20
21
22
23
24
day

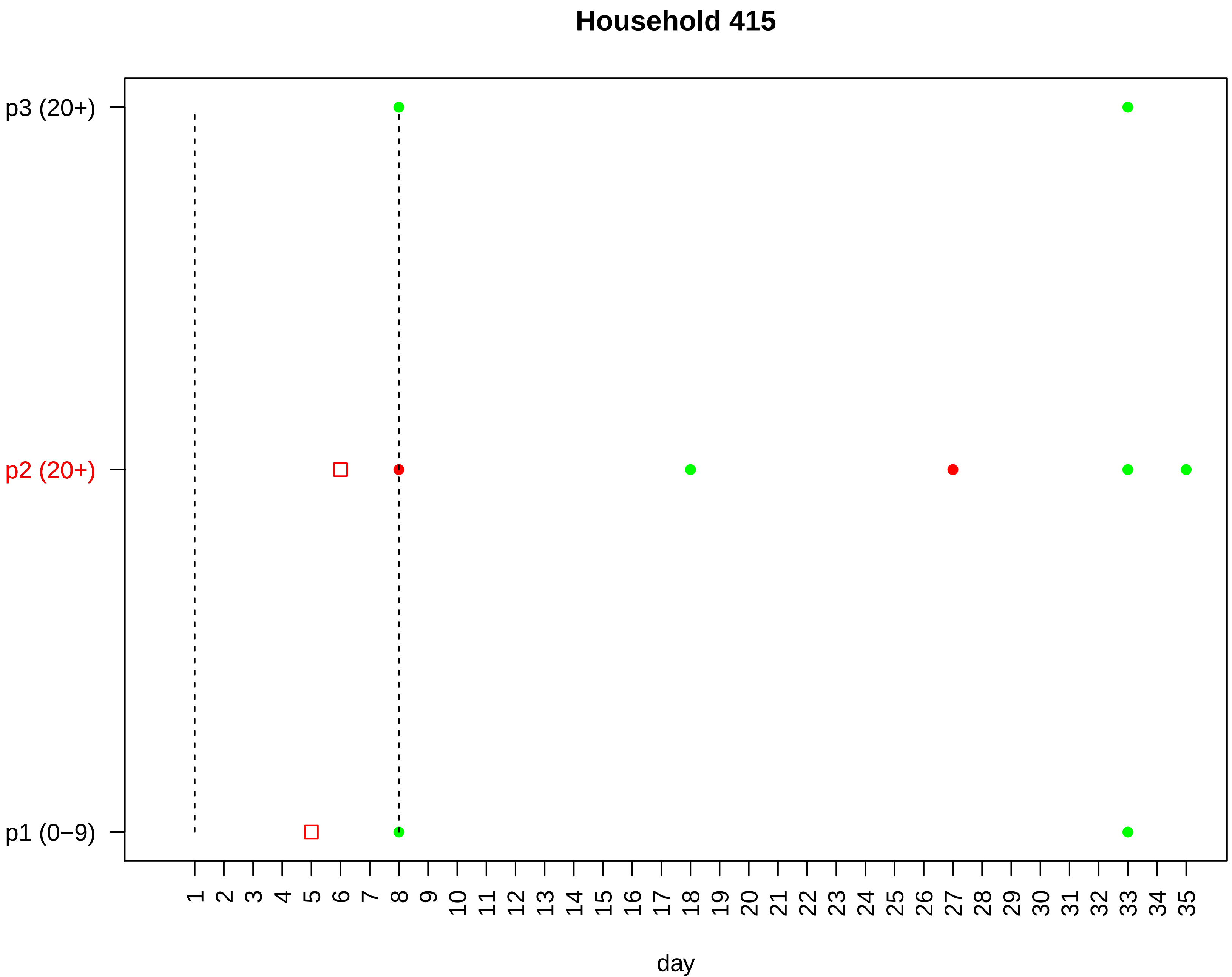

Household 415
p3 (20+)
p2 (20+)
p1 (0−9)
1 2 3 4 5 6 7 8 9 10 11 12 13 14 15 16 17 18 19 20 21 22 23 24 25 26 27 28 29 30 31 32 33 34 35
day

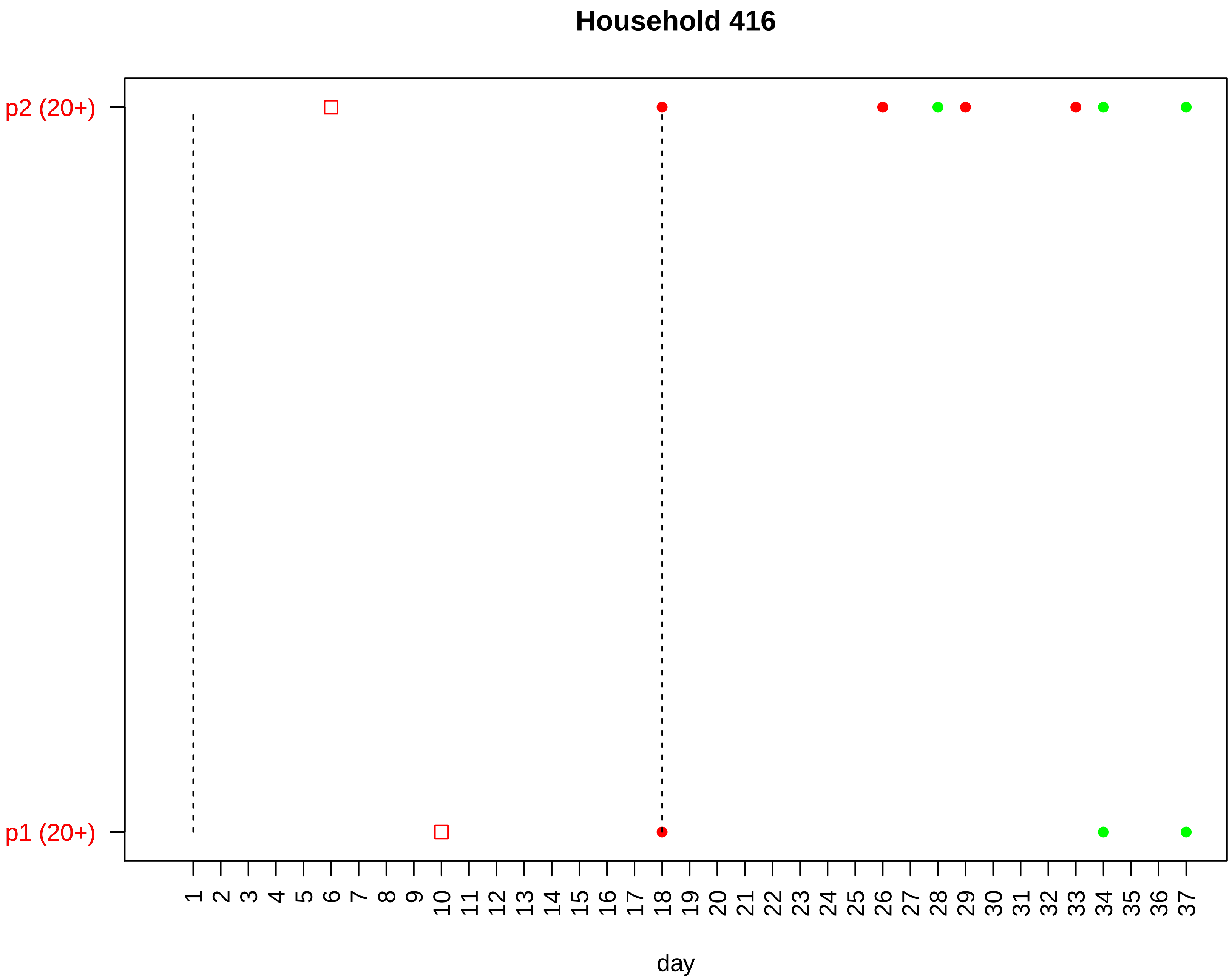
Household 416
p2 (20+)
p1 (20+)
1 2 3 4 5 6 7 8 9 10 11 12 13 14 15 16 17 18 19 20 21 22 23 24 25 26 27 28 29 30 31 32 33 34 35 36 37
day

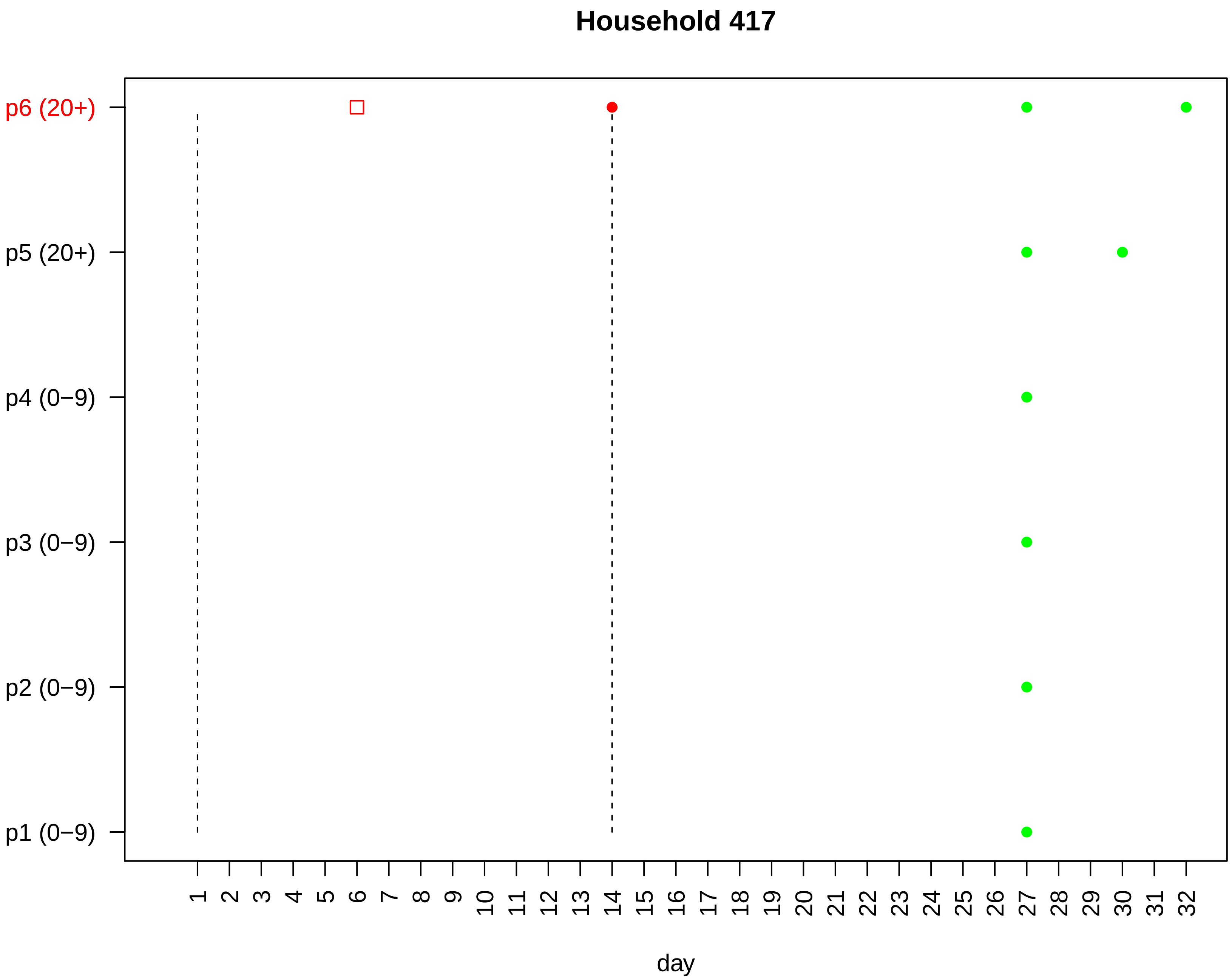

Household 417
p6 (20+)
p5 (20+)
p4 (0−9)
p3 (0−9)
p2 (0−9)
p1 (0−9)
1 2 3 4 5 6 7 8 9 10 11 12 13 14 15 16 17 18 19 20 21 22 23 24 25 26 27 28 29 30 31 32
day

Household 418

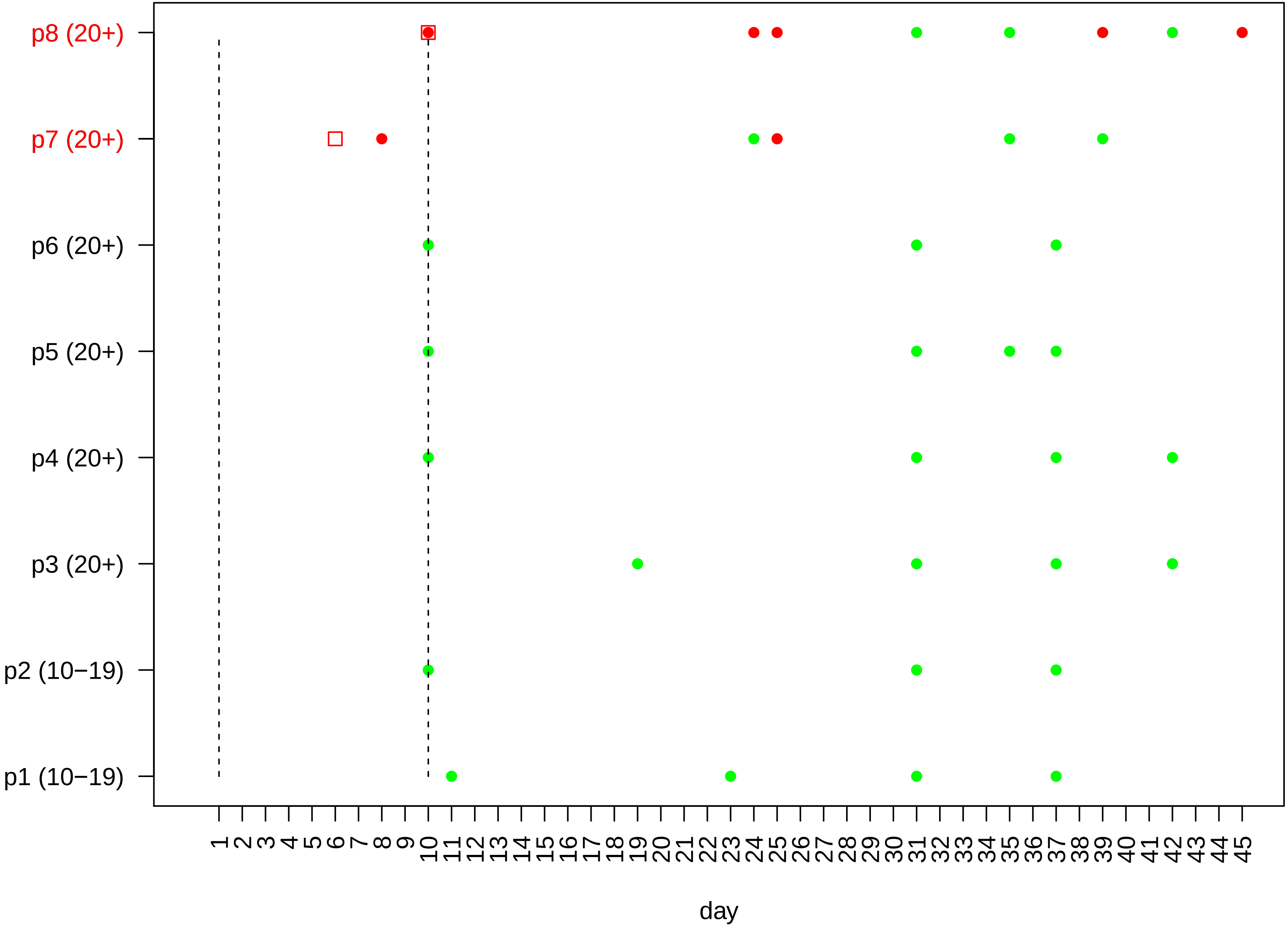

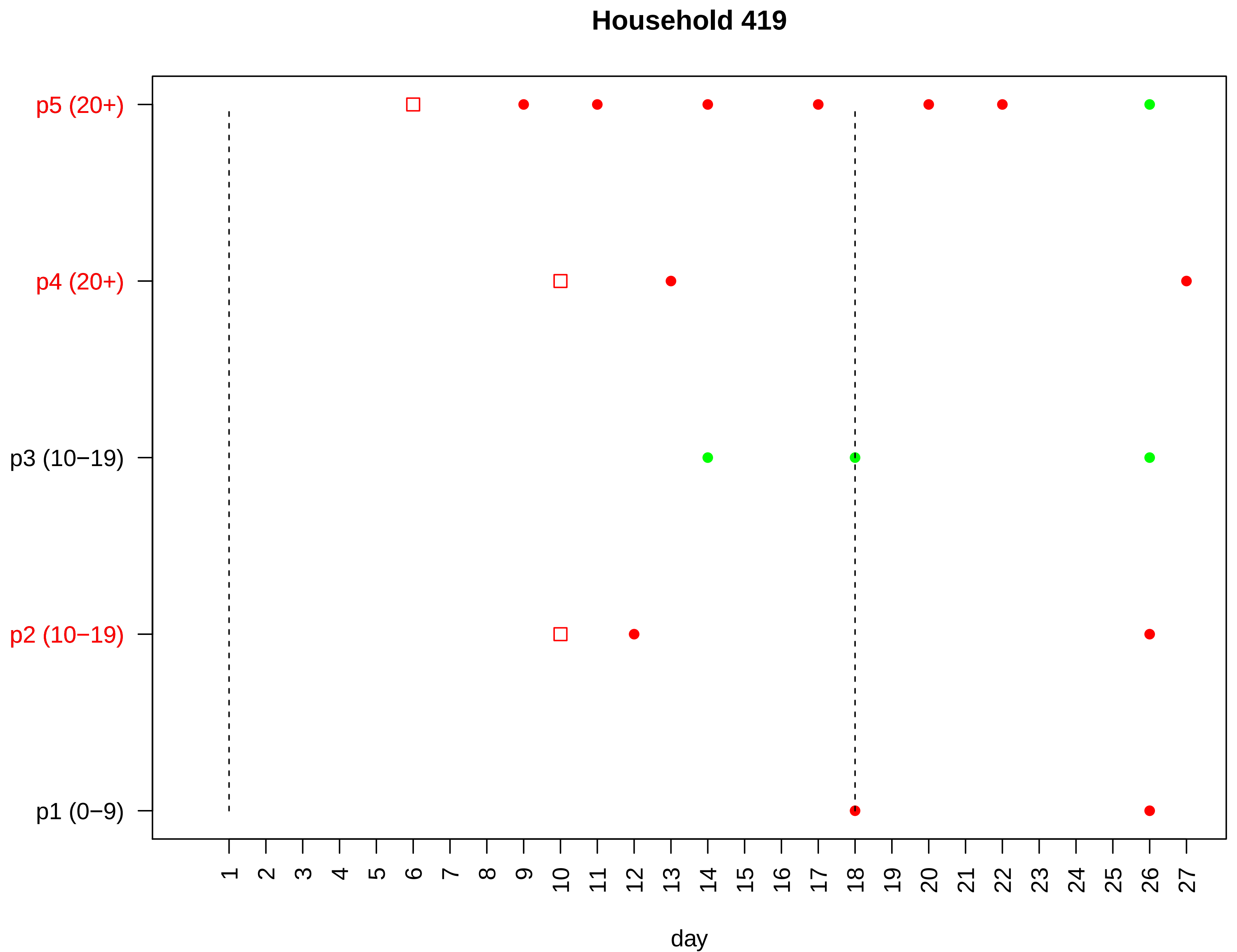

Household 419
p5 (20+)
p4 (20+)
p3 (10−19)
p2 (10−19)
p1 (0−9)
1 2 3 4 5 6 7 8 9 10 11 12 13 14 15 16 17 18 19 20 21 22 23 24 25 26 27
day

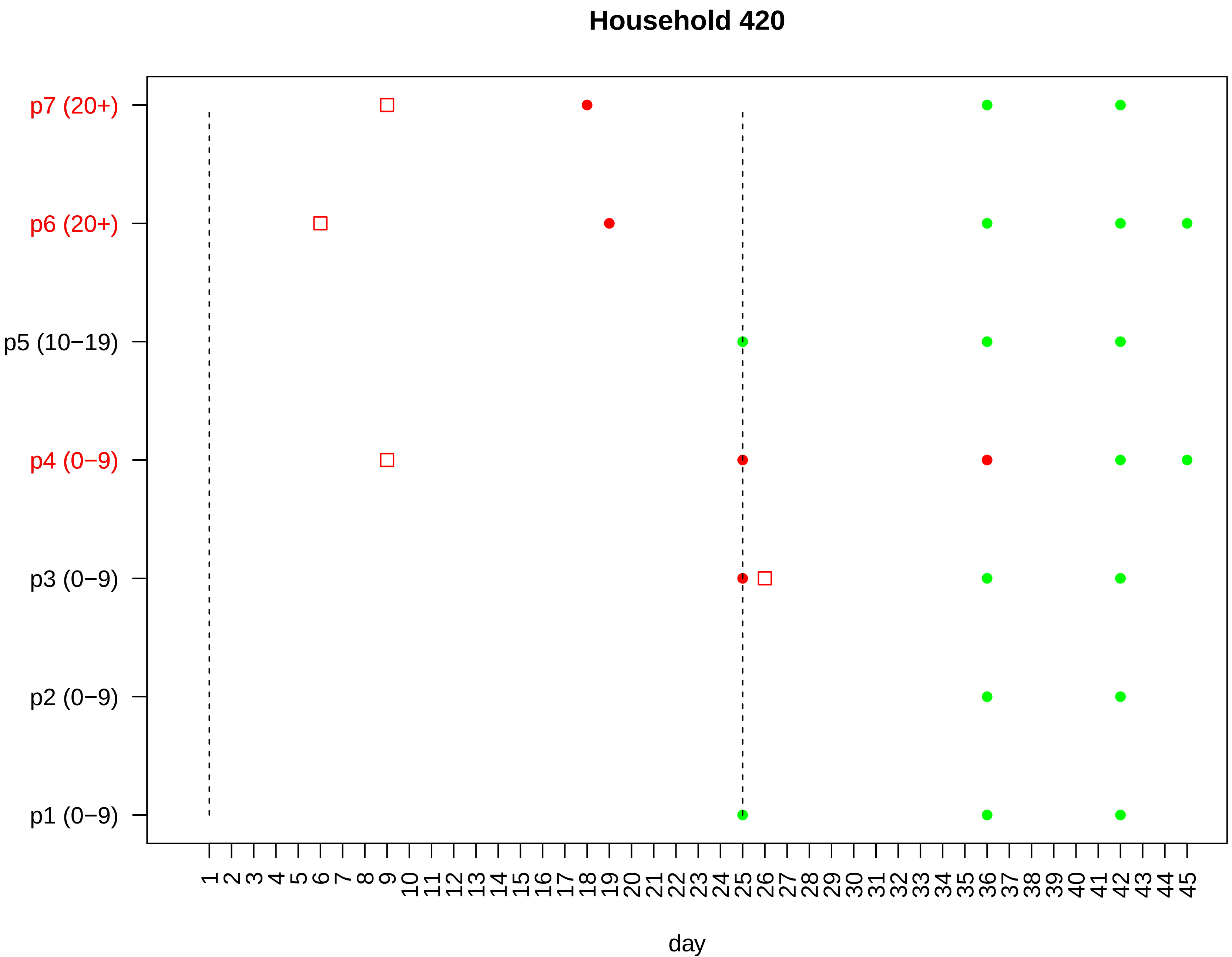

Household 420
p7 (20+)
p6 (20+)
p5 (10−19)
p4 (0−9)
p3 (0−9)
p2 (0−9)
p1 (0−9)
1 2 3 4 5 6 7 8 9 10 11 12 13 14 15 16 17 18 19 20 21 22 23 24 25 26 27 28 29 30 31 32 33 34 35 36 37 38 39 40 41 42 43 44 45
day

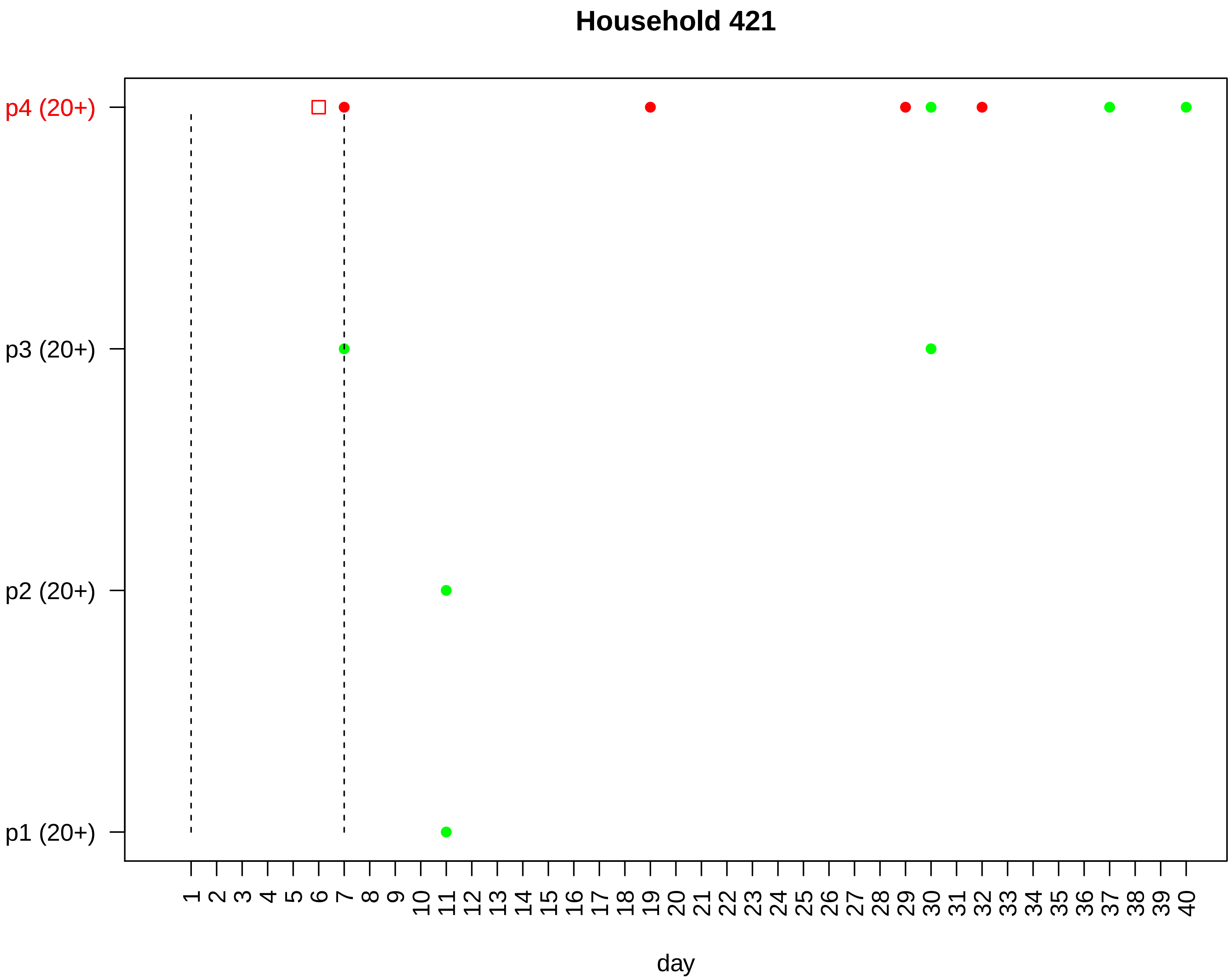

Household 421
p4 (20+)
p3 (20+)
p2 (20+)
p1 (20+)
1 2 3 4 5 6 7 8 9 10 11 12 13 14 15 16 17 18 19 20 21 22 23 24 25 26 27 28 29 30 31 32 33 34 35 36 37 38 39 40
day

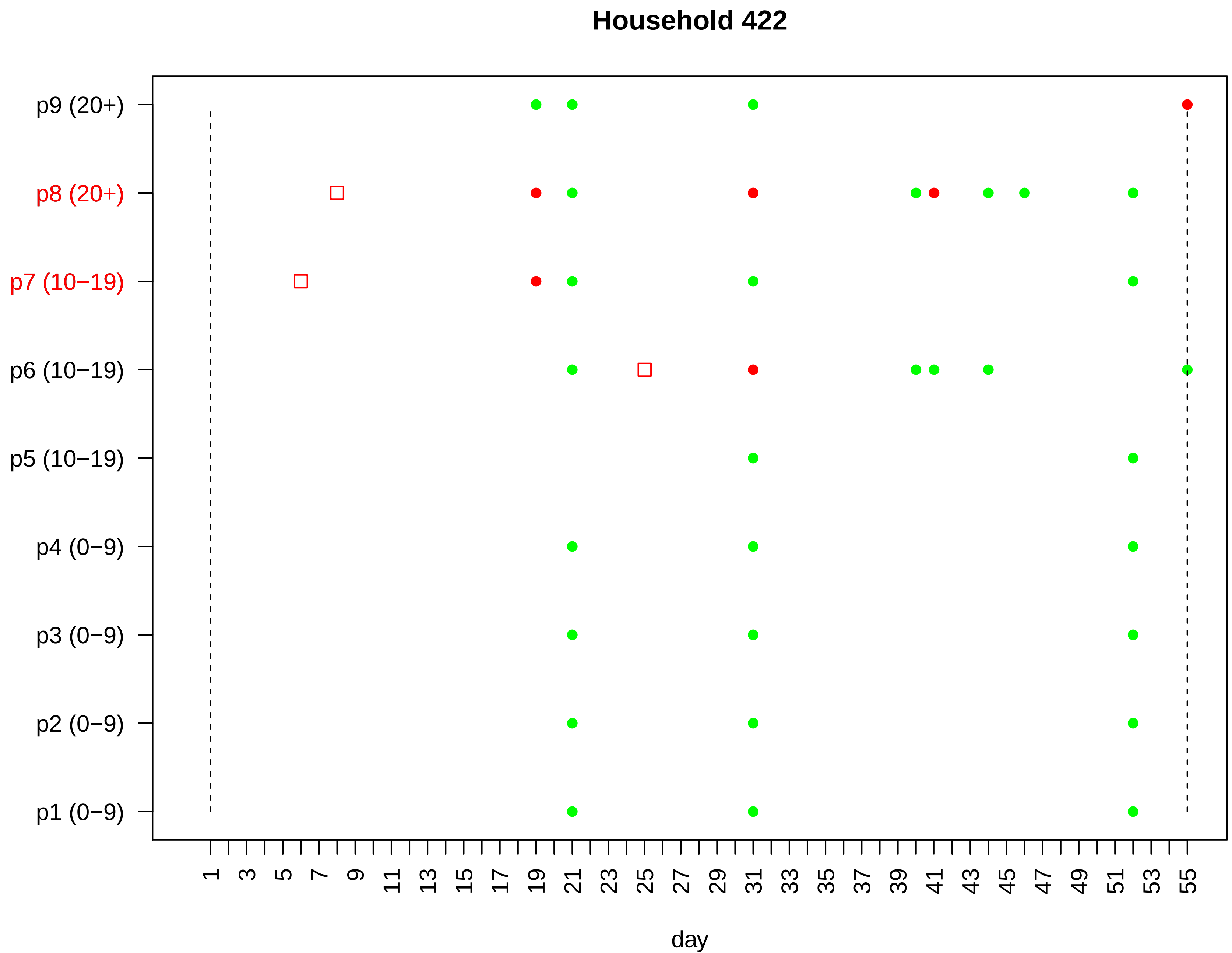

Household 422
p9 (20+)
p8 (20+)
p7 (10−19)
p6 (10−19)
p5 (10−19)
p4 (0−9)
p3 (0−9)
p2 (0−9)
p1 (0−9)
1 3 5 7 9 11 13 15 17 19 21 23 25 27 29 31 33 35 37 39 41 43 45 47 49 51 53 55
day

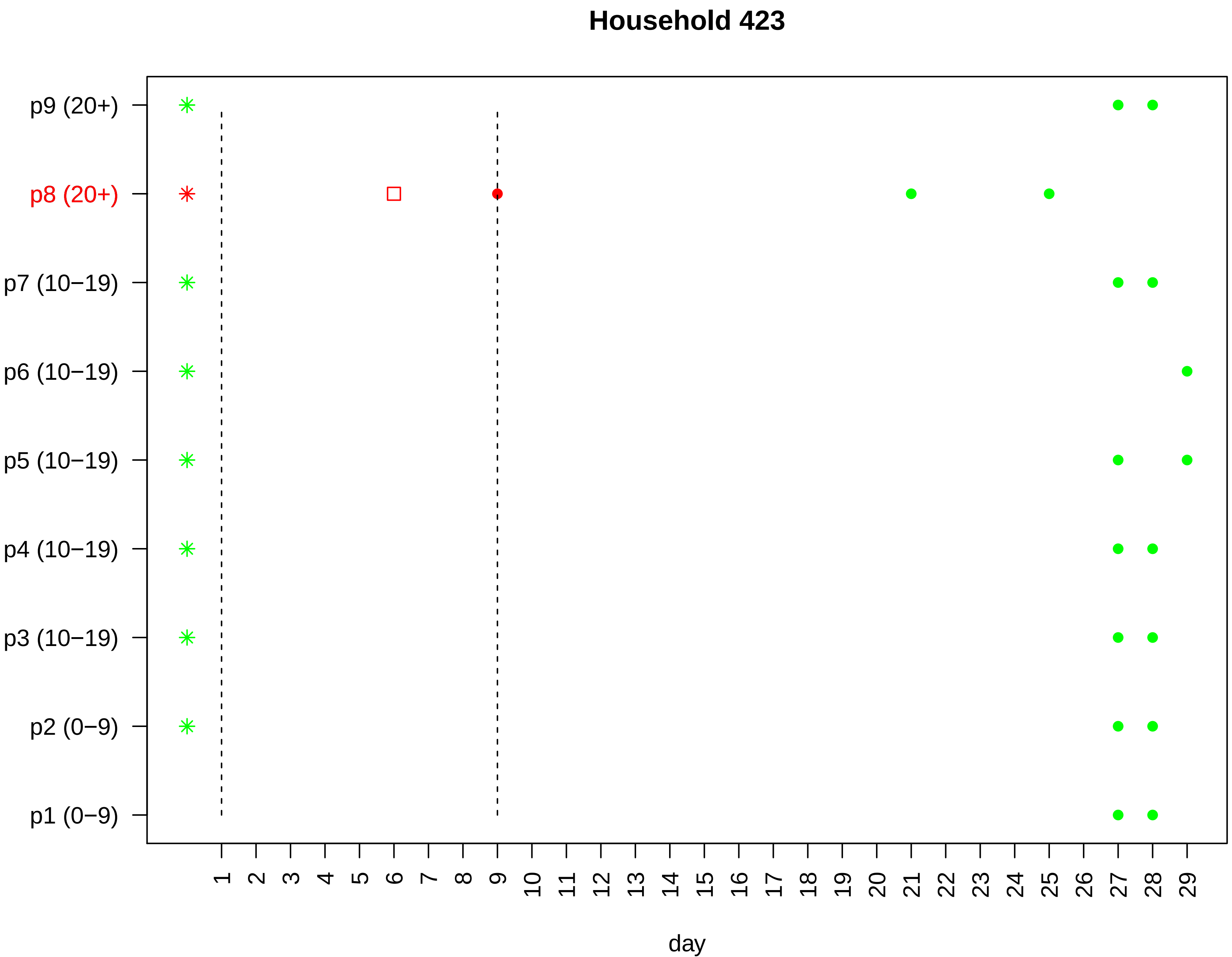

Household 423
p9 (20+)
p8 (20+)
p7 (10−19)
p6 (10−19)
p5 (10−19)
p4 (10−19)
p3 (10−19)
p2 (0−9)
p1 (0−9)
1 2 3 4 5 6 7 8 9 10 11 12 13 14 15 16 17 18 19 20 21 22 23 24 25 26 27 28 29
day

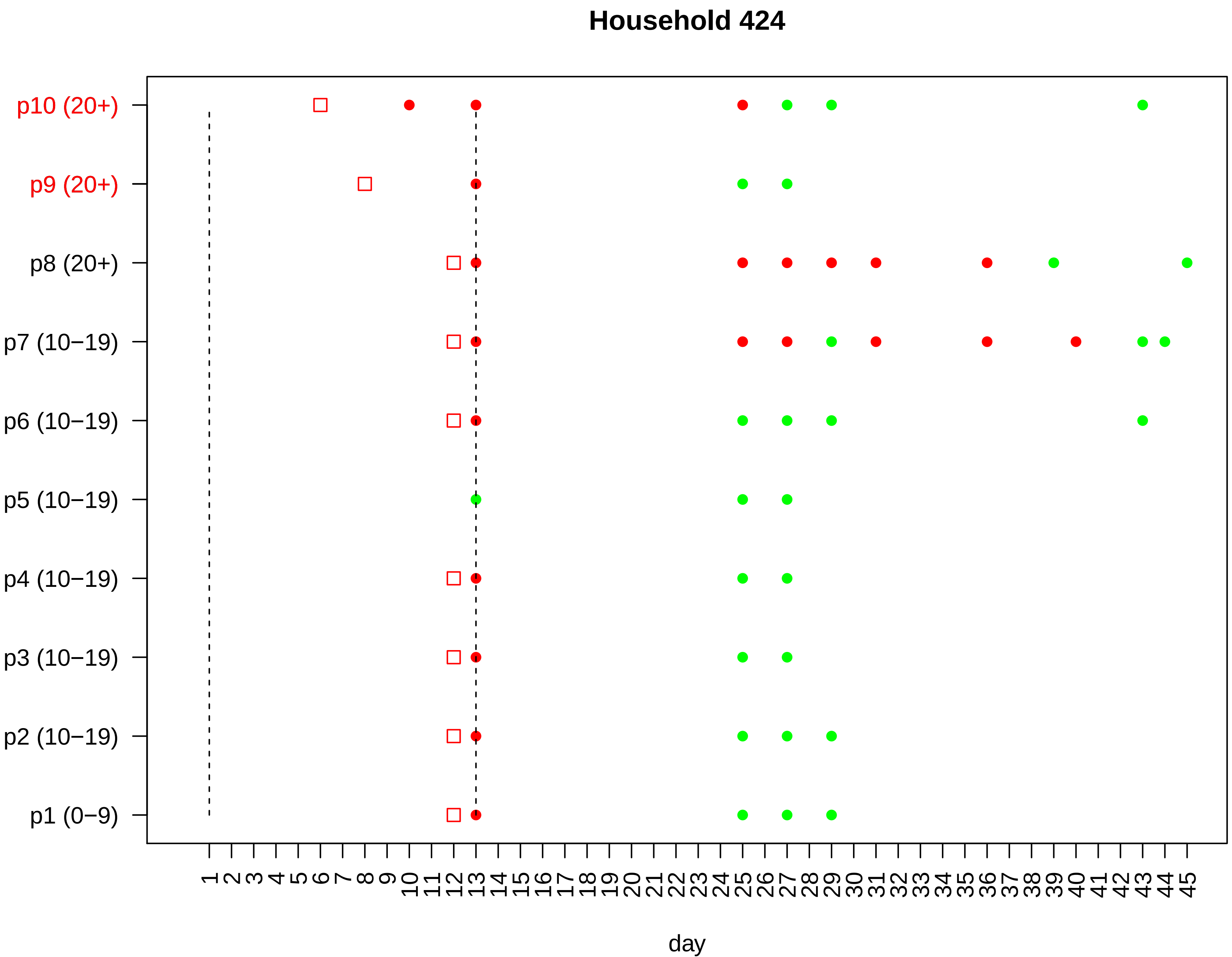

Household 424
p10 (20+)
p9 (20+)
p8 (20+)
p7 (10−19)
p6 (10−19)
p5 (10−19)
p4 (10−19)
p3 (10−19)
p2 (10−19)
p1 (0−9)
1 2 3 4 5 6 7 8 9 10 11 12 13 14 15 16 17 18 19 20 21 22 23 24 25 26 27 28 29 30 31 32 33 34 35 36 37 38 39 40 41 42 43 44 45
day

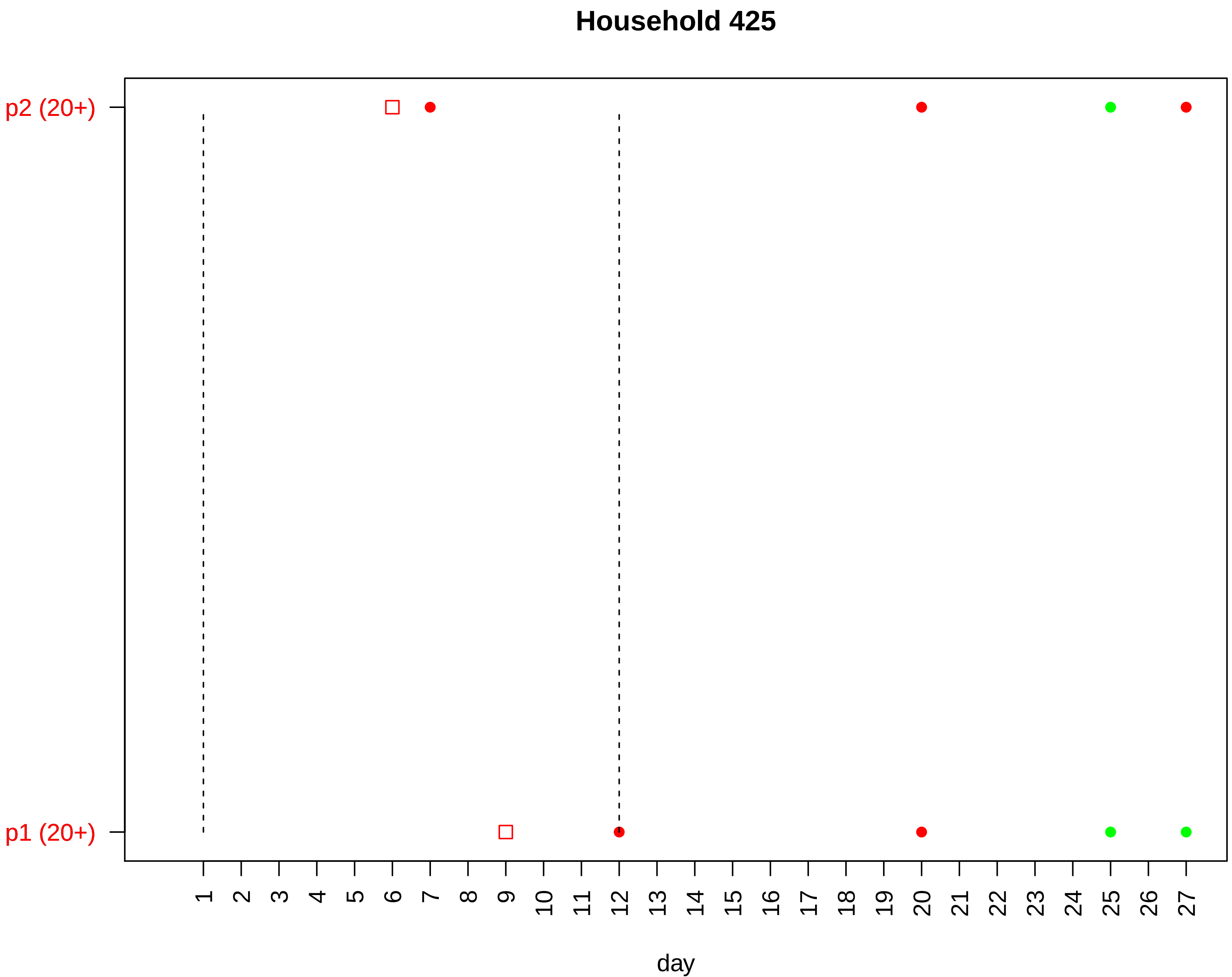

Household 425
p2 (20+)
p1 (20+)
1 2 3 4 5 6 7 8 9 10 11 12 13 14 15 16 17 18 19 20 21 22 23 24 25 26 27
day

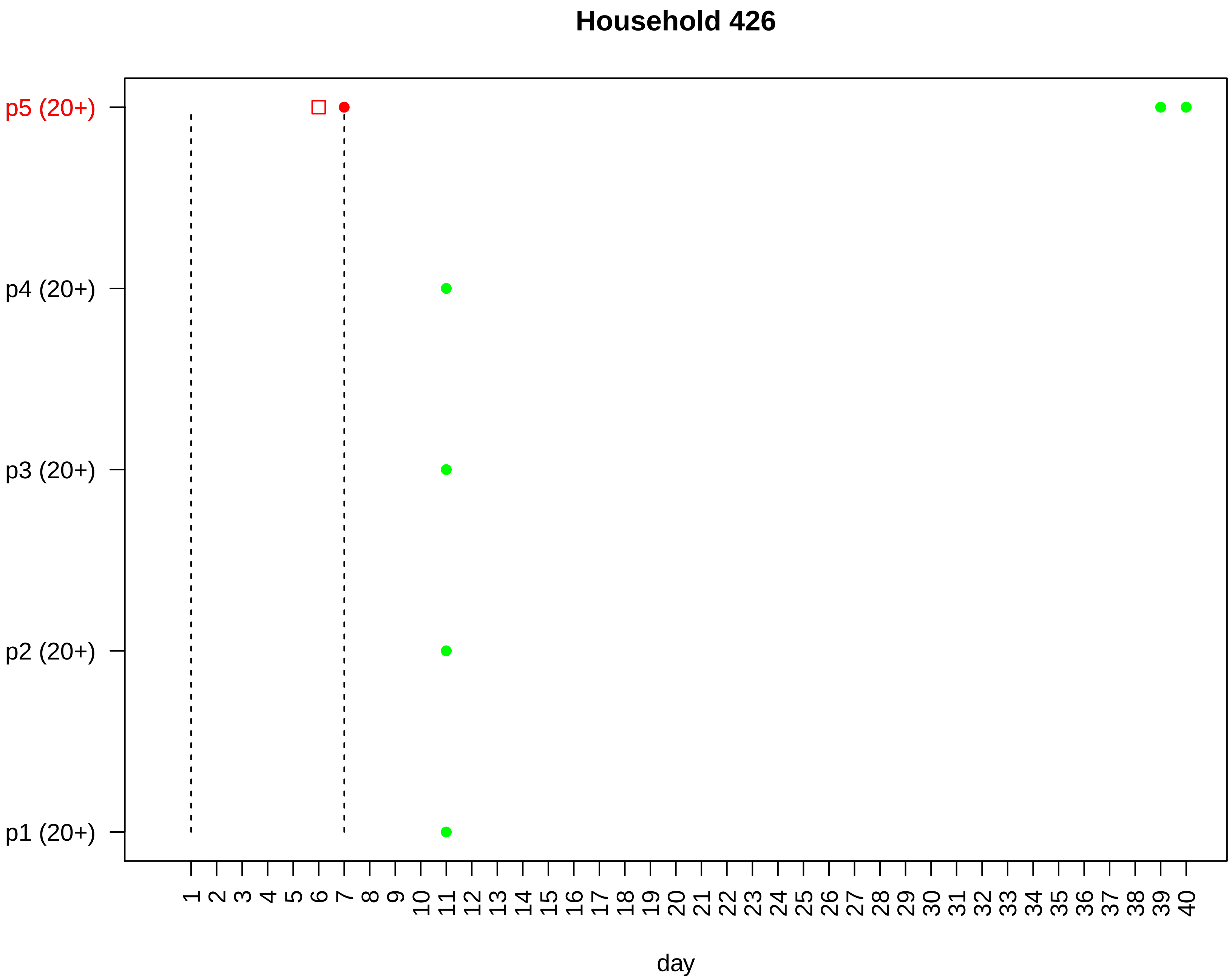
Household 426
p5 (20+)
p4 (20+)
p3 (20+)
p2 (20+)
p1 (20+)
1 2 3 4 5 6 7 8 9 10 11 12 13 14 15 16 17 18 19 20 21 22 23 24 25 26 27 28 29 30 31 32 33 34 35 36 37 38 39 40
day

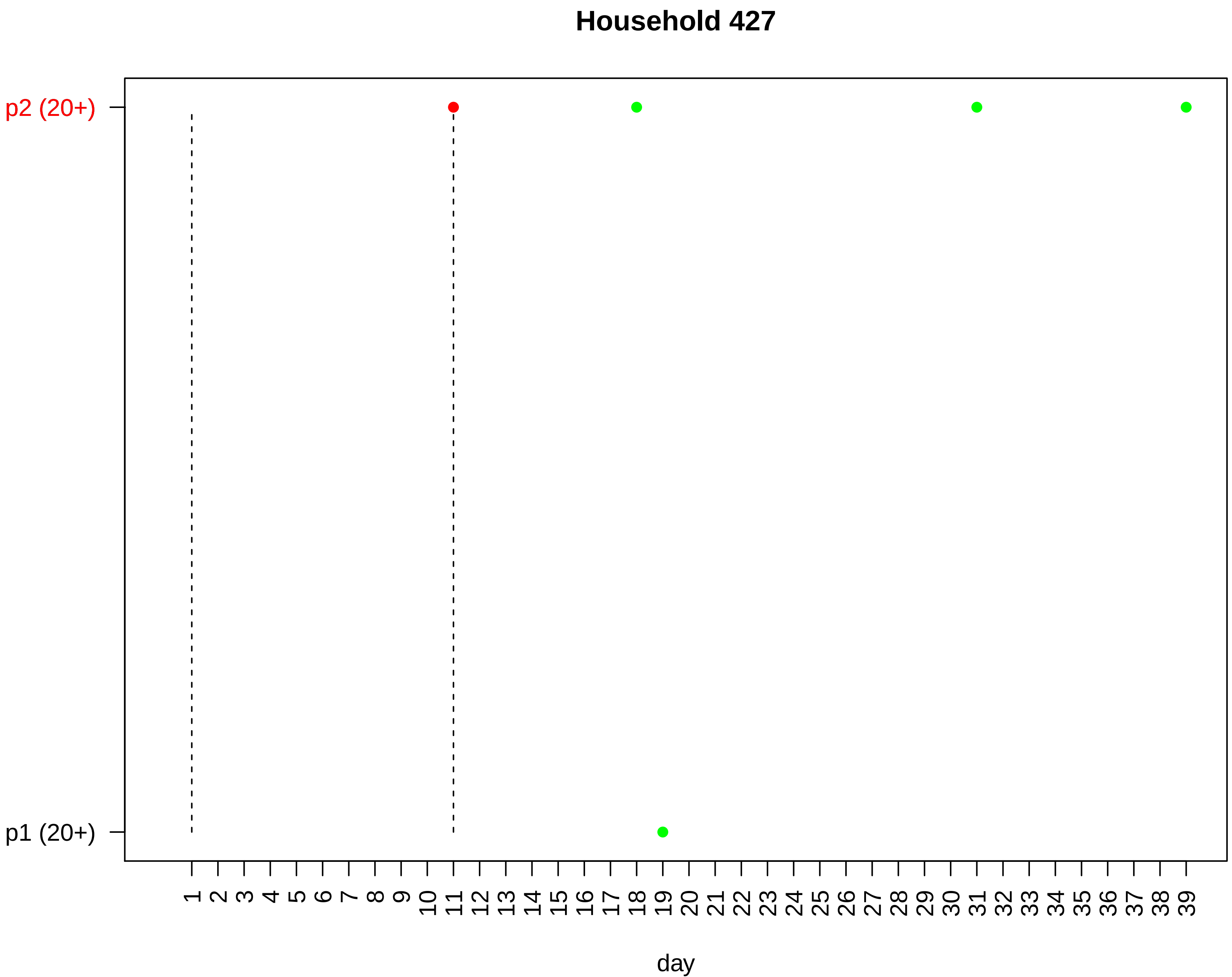

Household 427
p2 (20+)
p1 (20+)
1 2 3 4 5 6 7 8 9 10 11 12 13 14 15 16 17 18 19 20 21 22 23 24 25 26 27 28 29 30 31 32 33 34 35 36 37 38 39
day

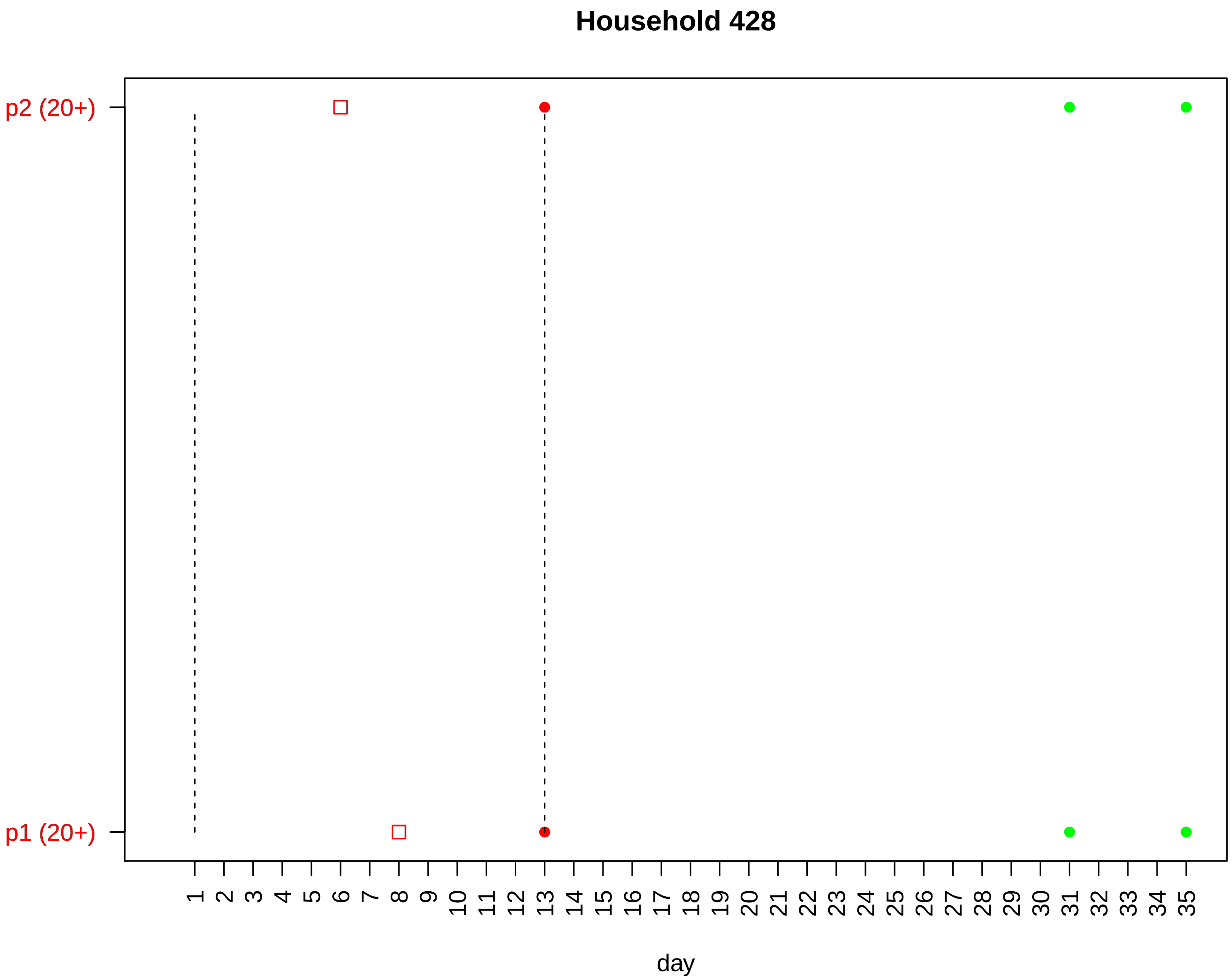

Household 428
p2 (20+)
p1 (20+)
1 2 3 4 5 6 7 8 9 10 11 12 13 14 15 16 17 18 19 20 21 22 23 24 25 26 27 28 29 30 31 32 33 34 35
day

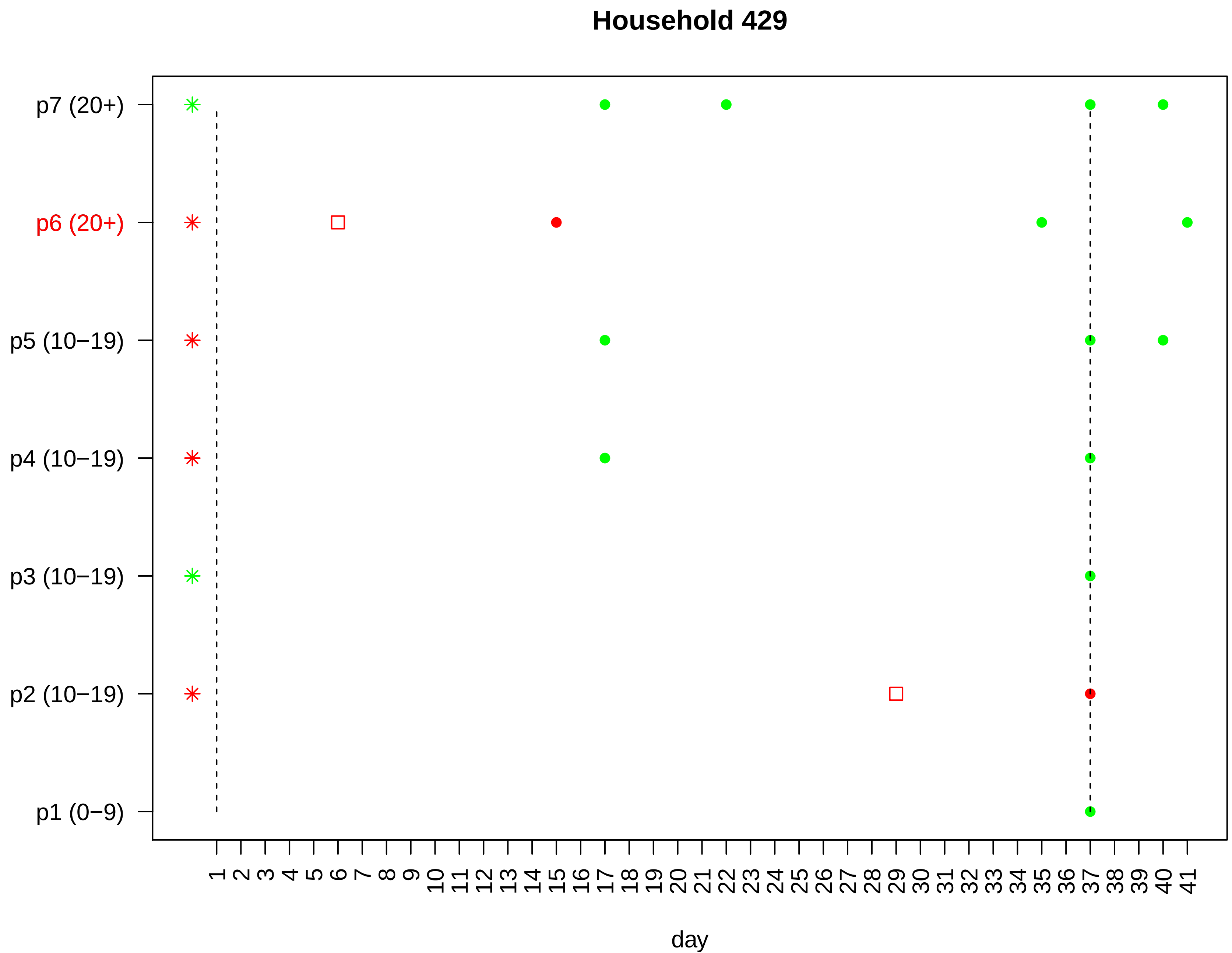

Household 429
p7 (20+)
p6 (20+)
p5 (10−19)
p4 (10−19)
p3 (10−19)
p2 (10−19)
p1 (0−9)
1 2 3 4 5 6 7 8 9 10 11 12 13 14 15 16 17 18 19 20 21 22 23 24 25 26 27 28 29 30 31 32 33 34 35 36 37 38 39 40 41
day

Household 430

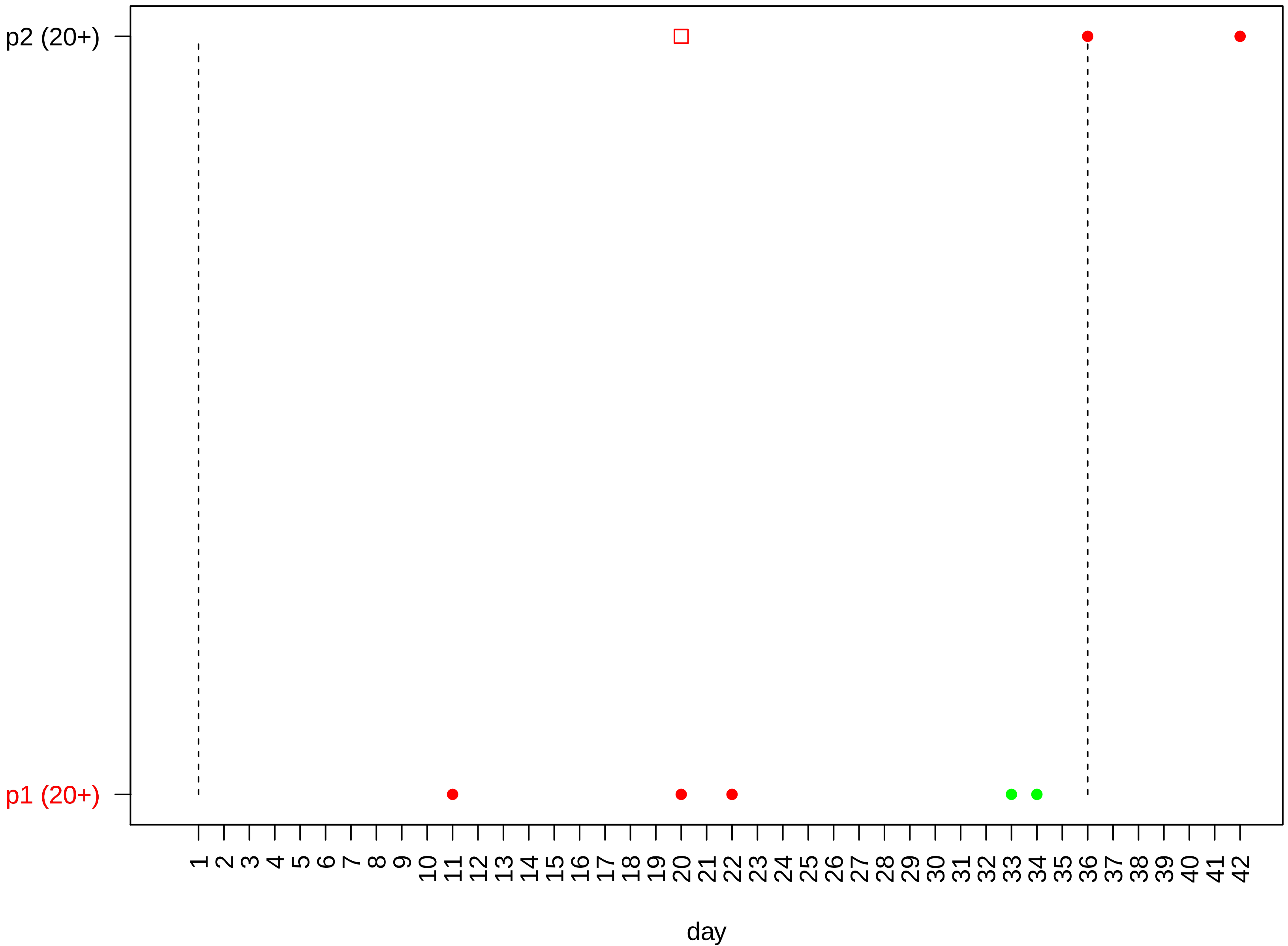

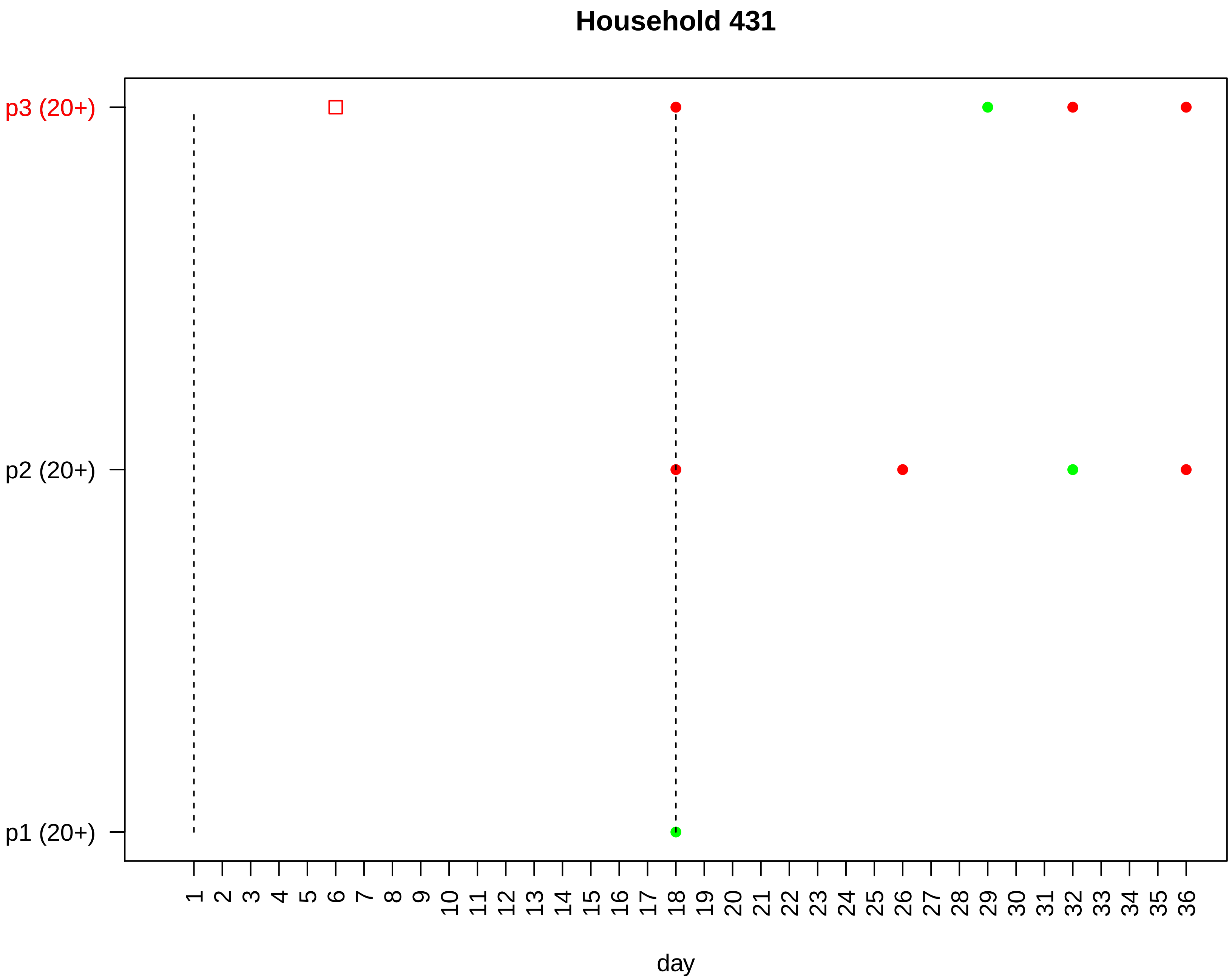

Household 431
p3 (20+)
p2 (20+)
p1 (20+)
1
2
3
4
5
6
7
8
9
10
11
12
13
14
15
16
17
18
19
20
21
22
23
24
25
26
27
28
29
30
31
32
33
34
35
36
day

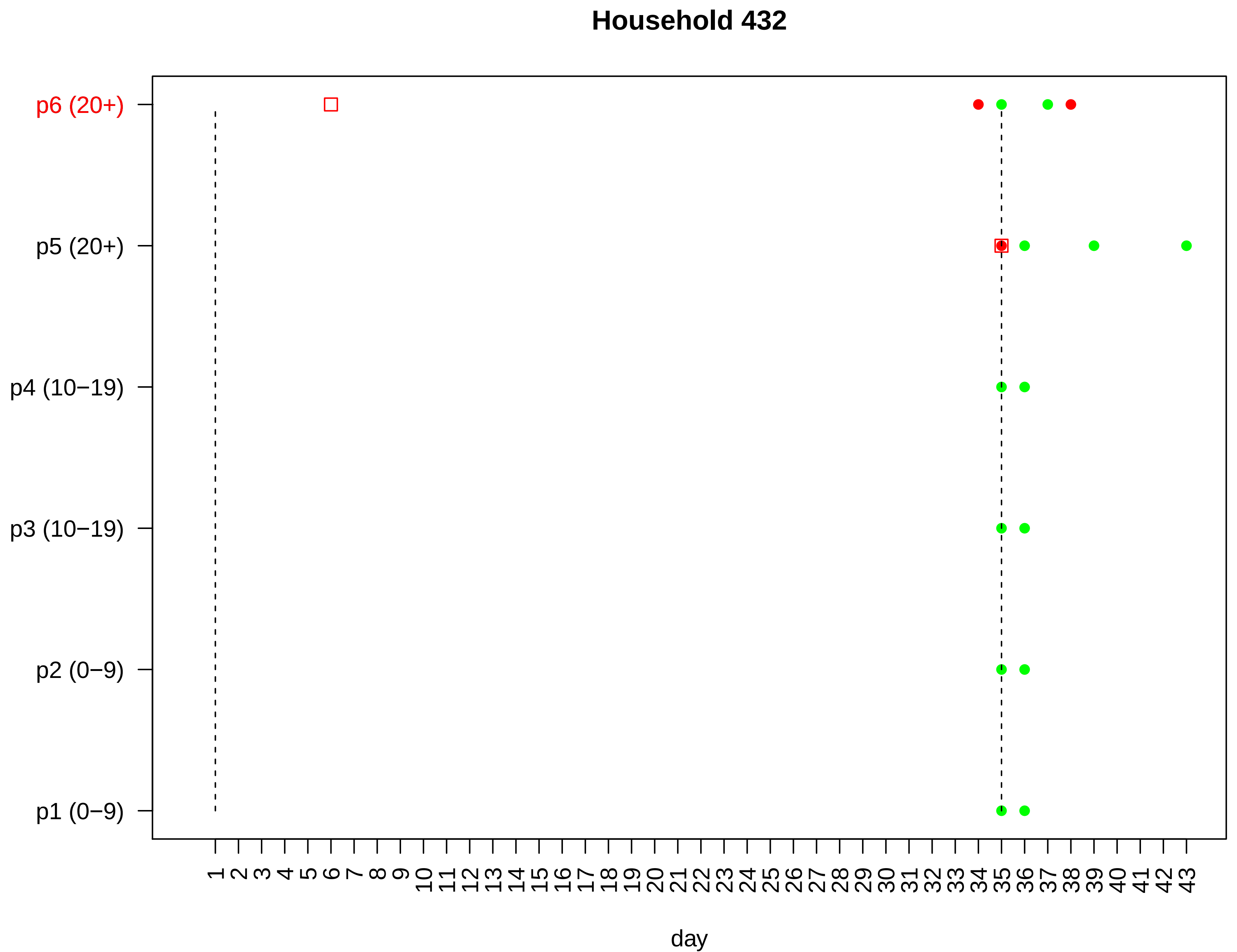

Household 432
p6 (20+)
p5 (20+)
p4 (10−19)
p3 (10−19)
p2 (0−9)
p1 (0−9)
1 2 3 4 5 6 7 8 9 10 11 12 13 14 15 16 17 18 19 20 21 22 23 24 25 26 27 28 29 30 31 32 33 34 35 36 37 38 39 40 41 42 43
day

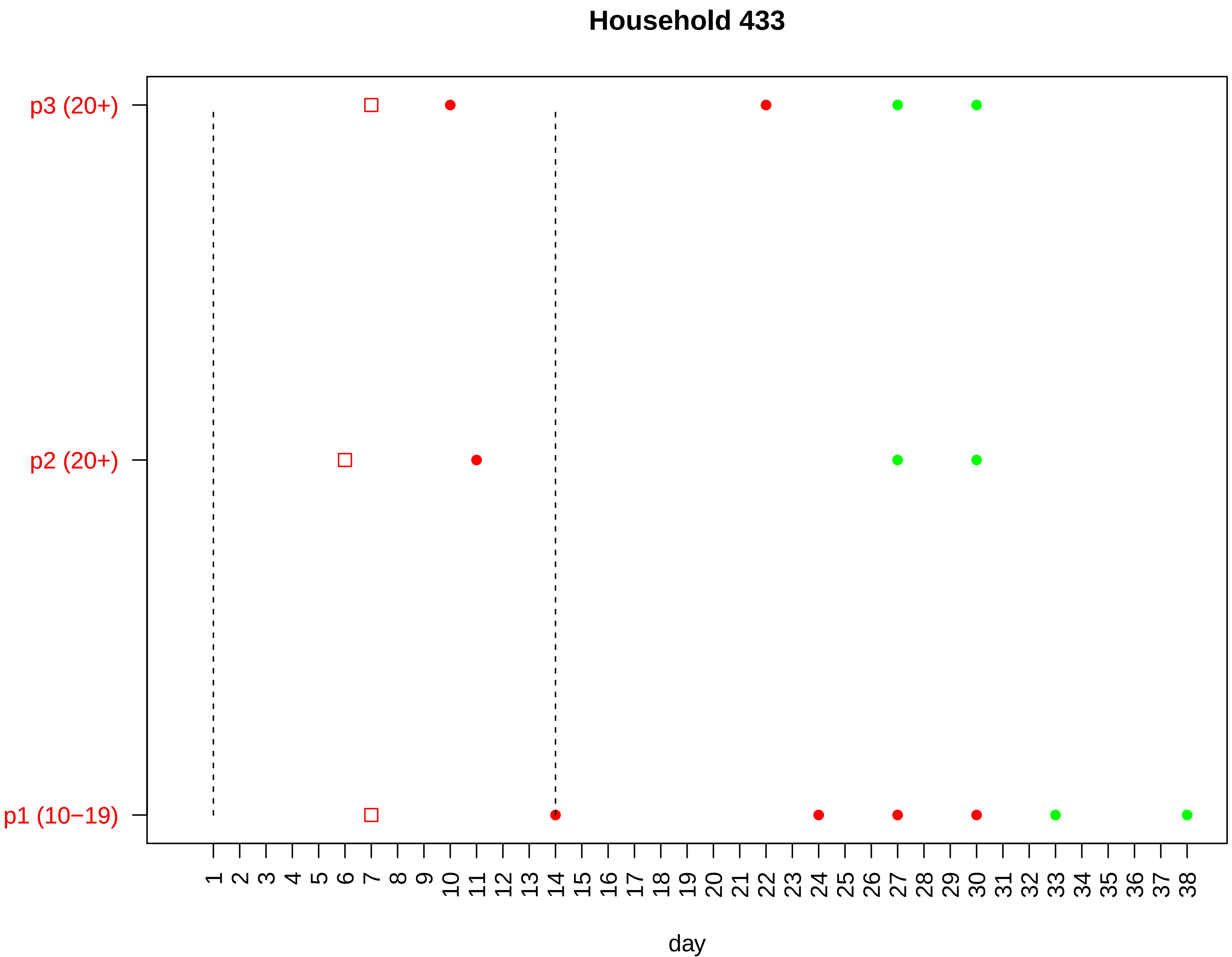

Household 433
p3 (20+)
p2 (20+)
p1 (10−19)
1 2 3 4 5 6 7 8 9 10 11 12 13 14 15 16 17 18 19 20 21 22 23 24 25 26 27 28 29 30 31 32 33 34 35 36 37 38
day

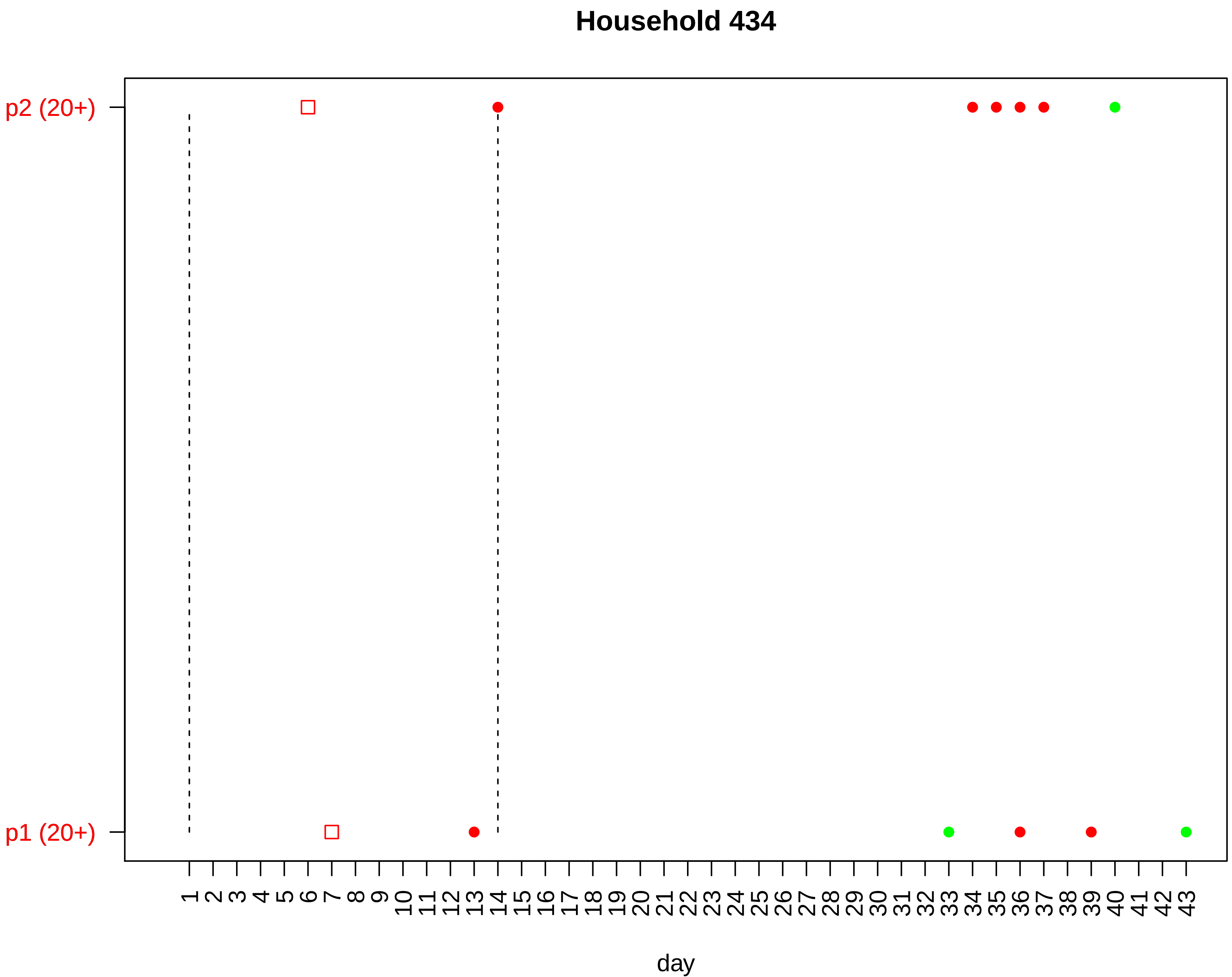
Household 434
p2 (20+)
p1 (20+)
1 2 3 4 5 6 7 8 9 10 11 12 13 14 15 16 17 18 19 20 21 22 23 24 25 26 27 28 29 30 31 32 33 34 35 36 37 38 39 40 41 42 43
day

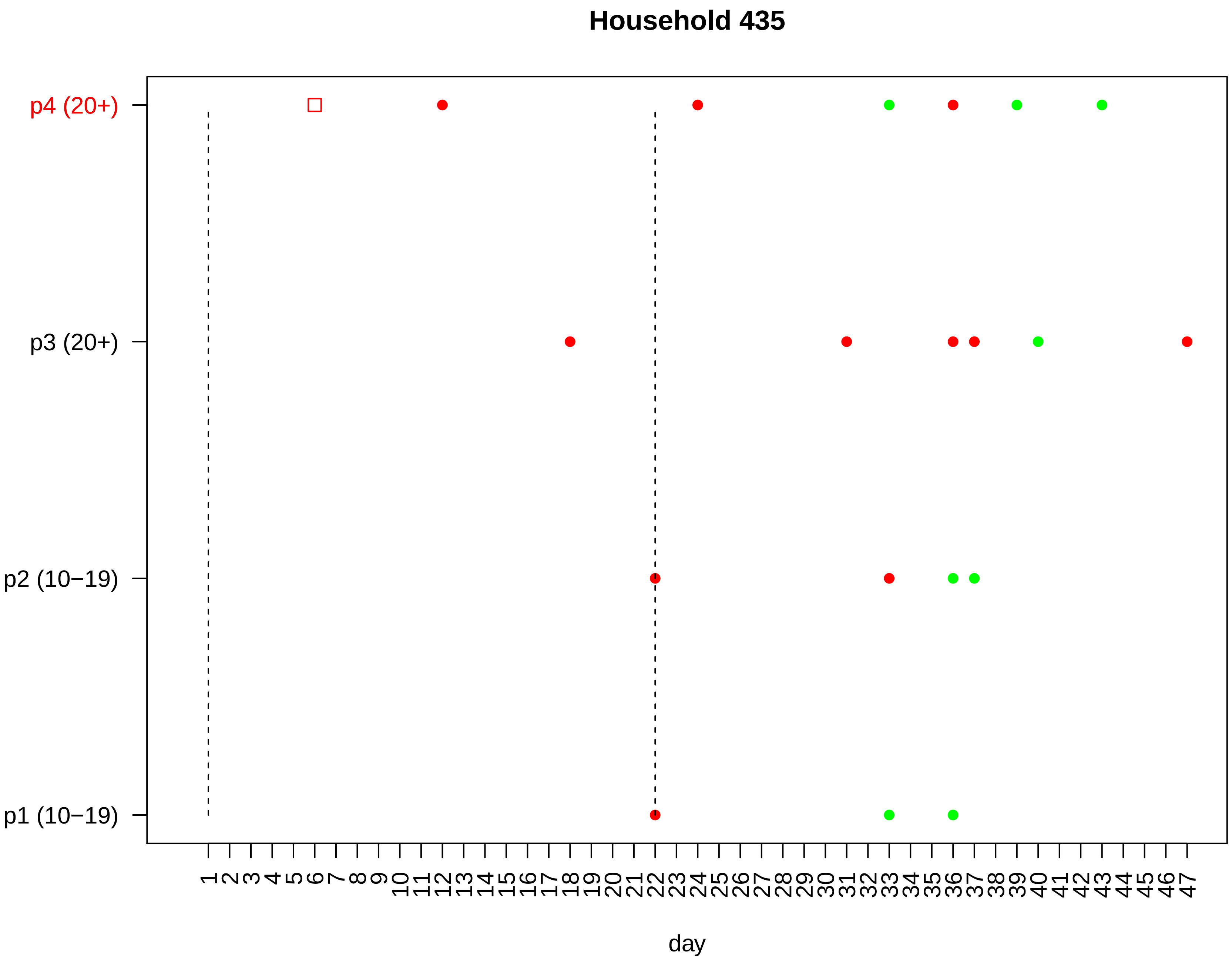

Household 435
p4 (20+)
p3 (20+)
p2 (10−19)
p1 (10−19)
1 2 3 4 5 6 7 8 9 10 11 12 13 14 15 16 17 18 19 20 21 22 23 24 25 26 27 28 29 30 31 32 33 34 35 36 37 38 39 40 41 42 43 44 45 46 47
day

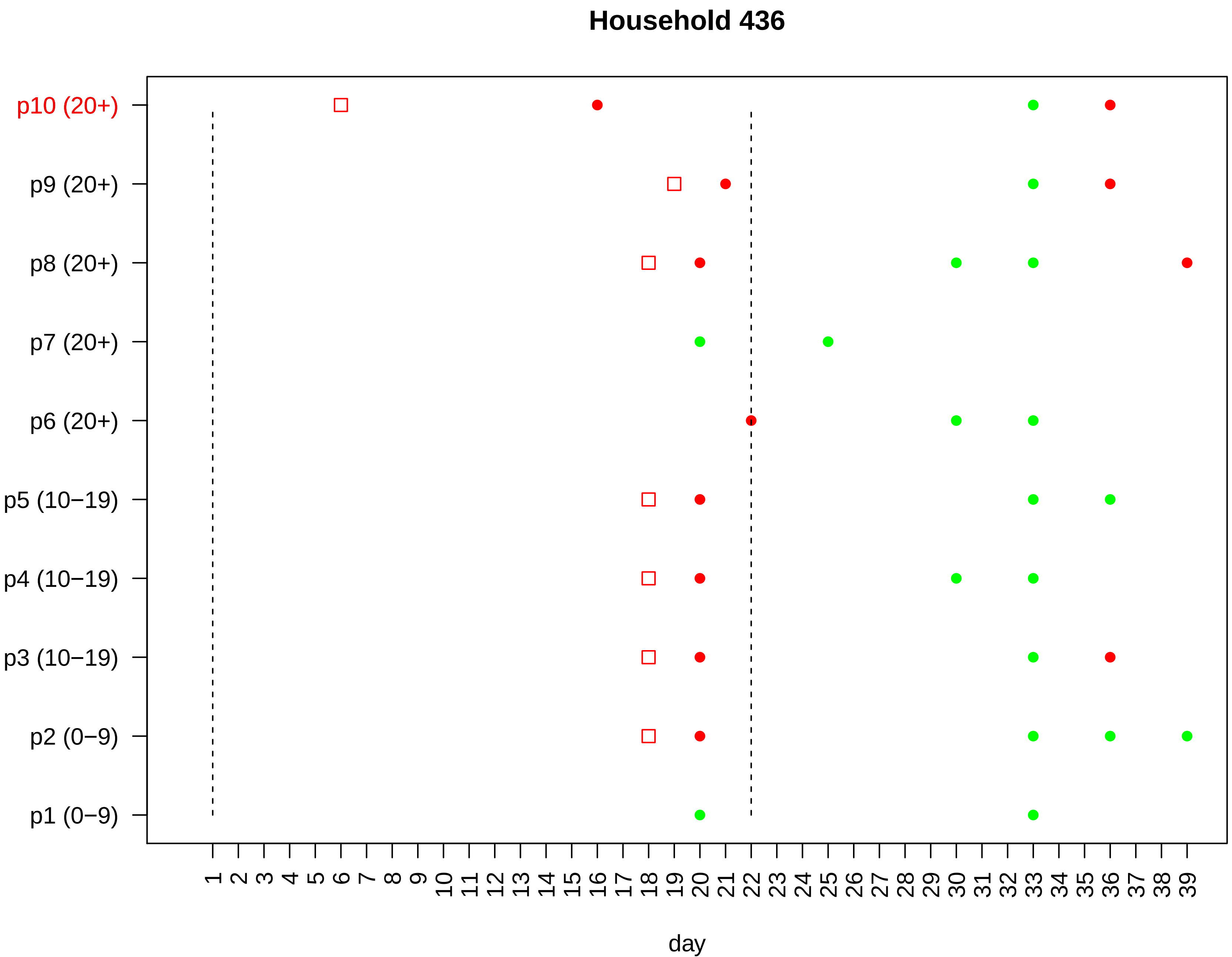
Household 436
p10 (20+)
p9 (20+)
p8 (20+)
p7 (20+)
p6 (20+)
p5 (10−19)
p4 (10−19)
p3 (10−19)
p2 (0−9)
p1 (0−9)
1 2 3 4 5 6 7 8 9 10 11 12 13 14 15 16 17 18 19 20 21 22 23 24 25 26 27 28 29 30 31 32 33 34 35 36 37 38 39
day

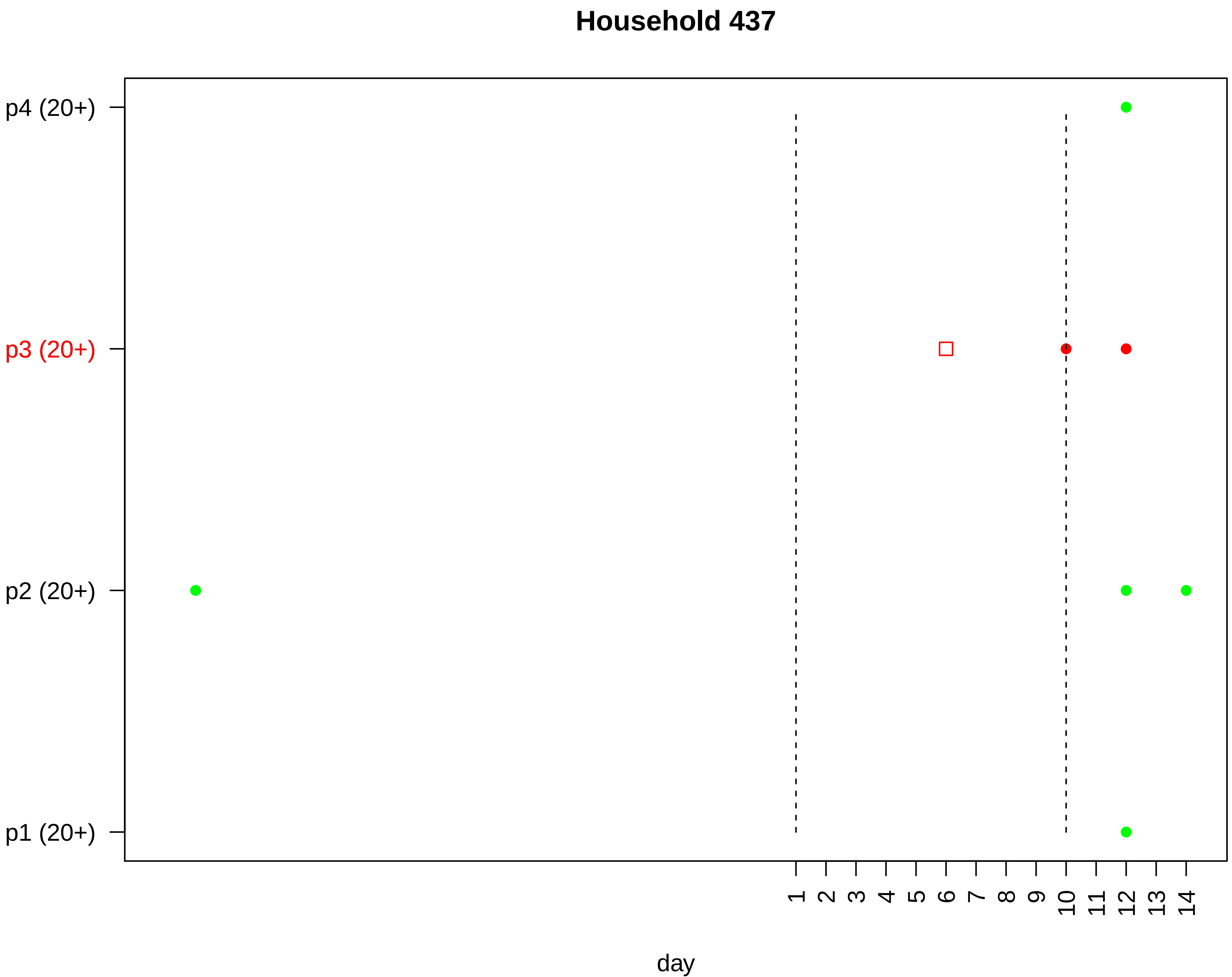
Household 437
p4 (20+)
p3 (20+)
p2 (20+)
p1 (20+)
1
2
3
4
5
6
7
8
9
10
11
12
13
14
day

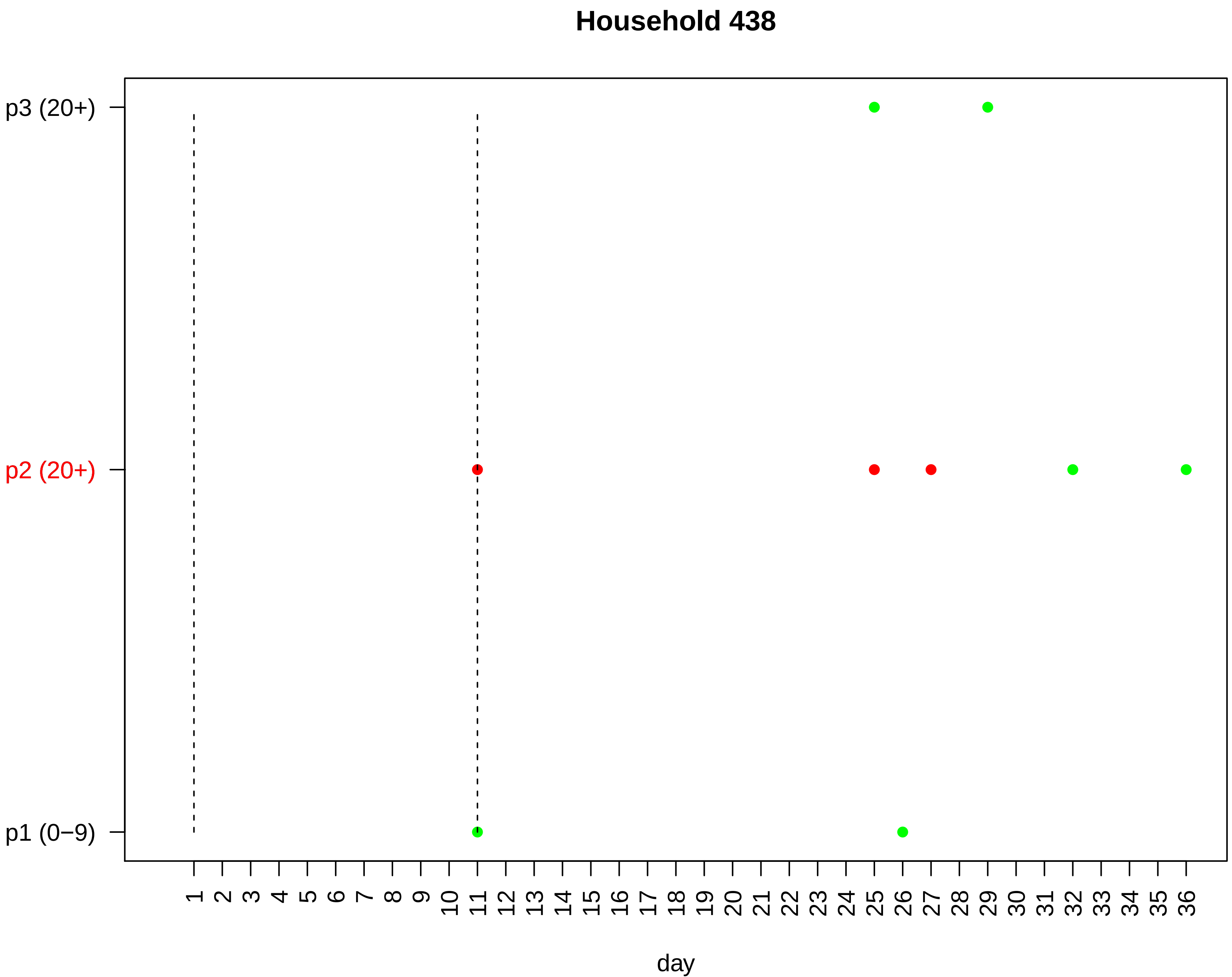

Household 438
p3 (20+)
p2 (20+)
p1 (0−9)
1 2 3 4 5 6 7 8 9 10 11 12 13 14 15 16 17 18 19 20 21 22 23 24 25 26 27 28 29 30 31 32 33 34 35 36
day

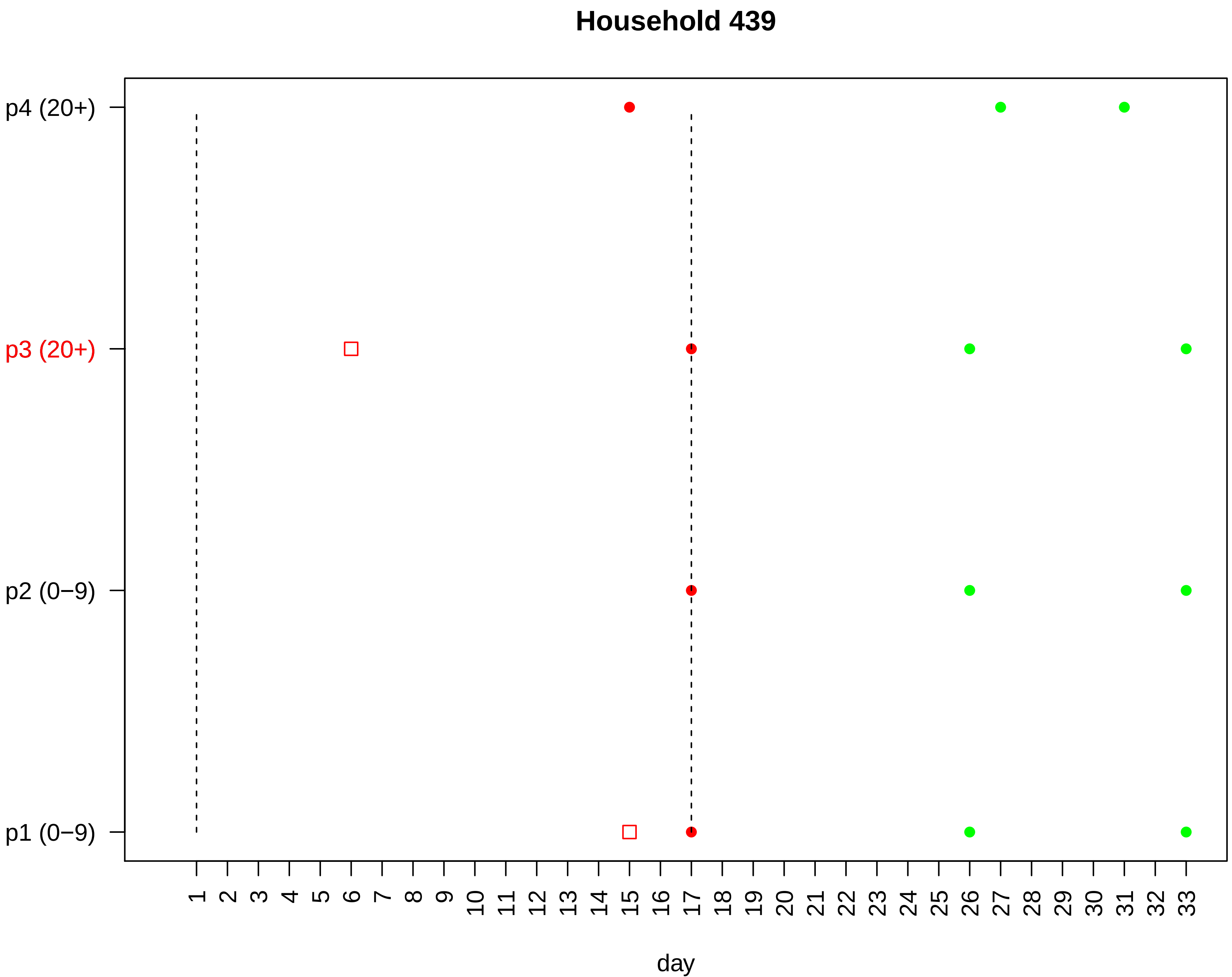

Household 439
p4 (20+)
p3 (20+)
p2 (0−9)
p1 (0−9)
1 2 3 4 5 6 7 8 9 10 11 12 13 14 15 16 17 18 19 20 21 22 23 24 25 26 27 28 29 30 31 32 33
day

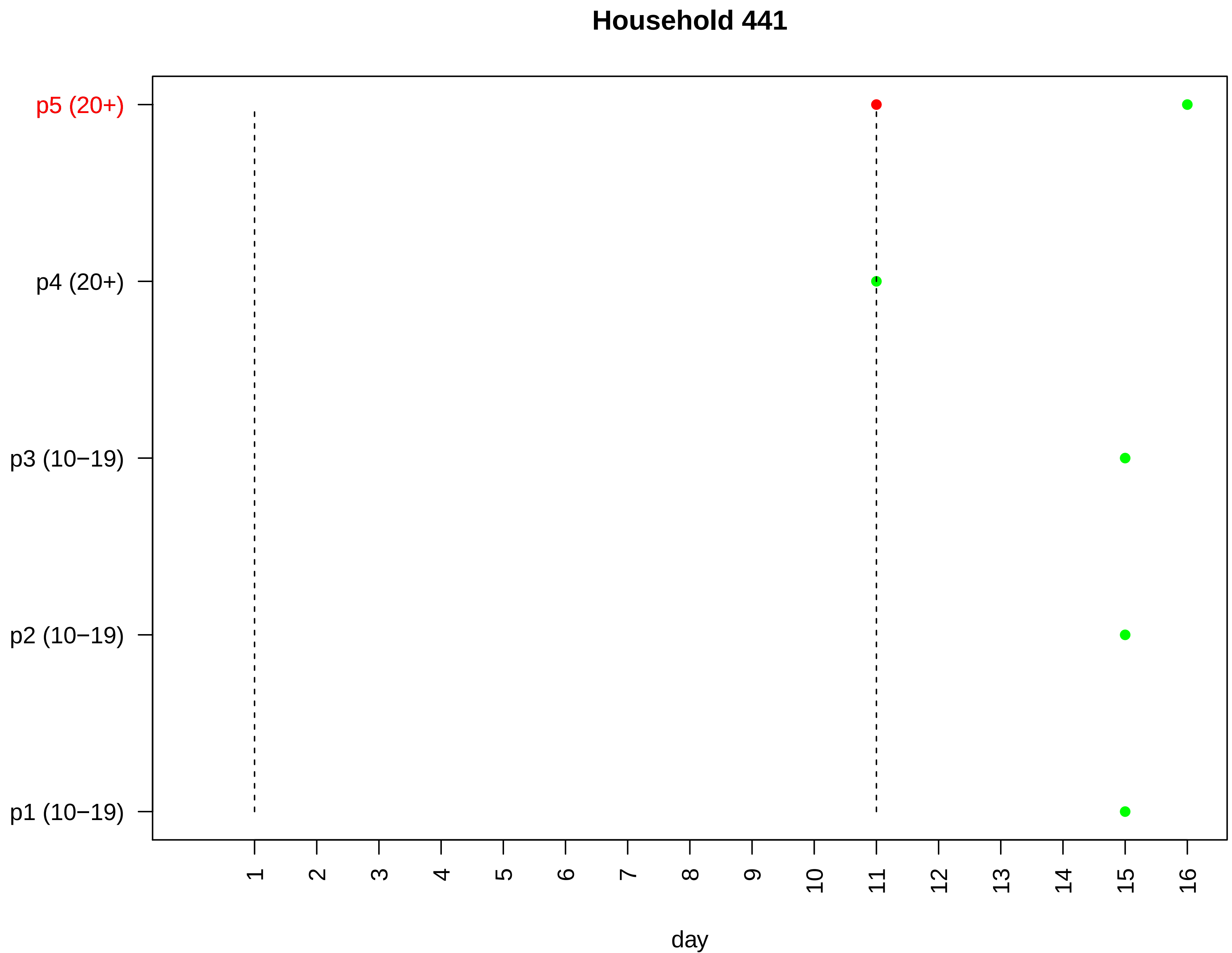

Household 441
p5 (20+)
p4 (20+)
p3 (10−19)
p2 (10−19)
p1 (10−19)
1
2
3
4
5
6
7
8
9
10
11
12
13
14
15
16
day

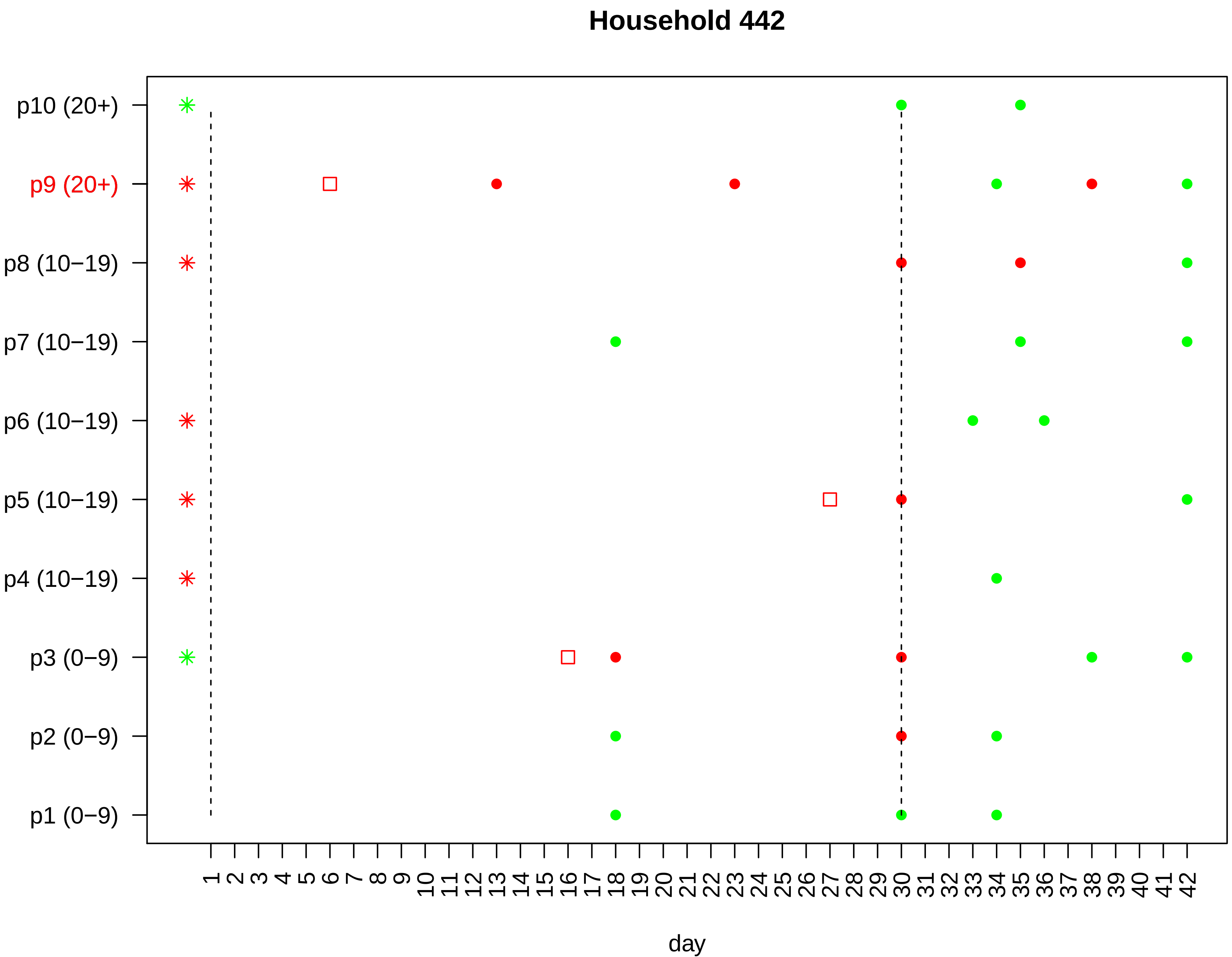

Household 442
p10 (20+)
p9 (20+)
p8 (10−19)
p7 (10−19)
p6 (10−19)
p5 (10−19)
p4 (10−19)
p3 (0−9)
p2 (0−9)
p1 (0−9)
1 2 3 4 5 6 7 8 9 10 11 12 13 14 15 16 17 18 19 20 21 22 23 24 25 26 27 28 29 30 31 32 33 34 35 36 37 38 39 40 41 42
day

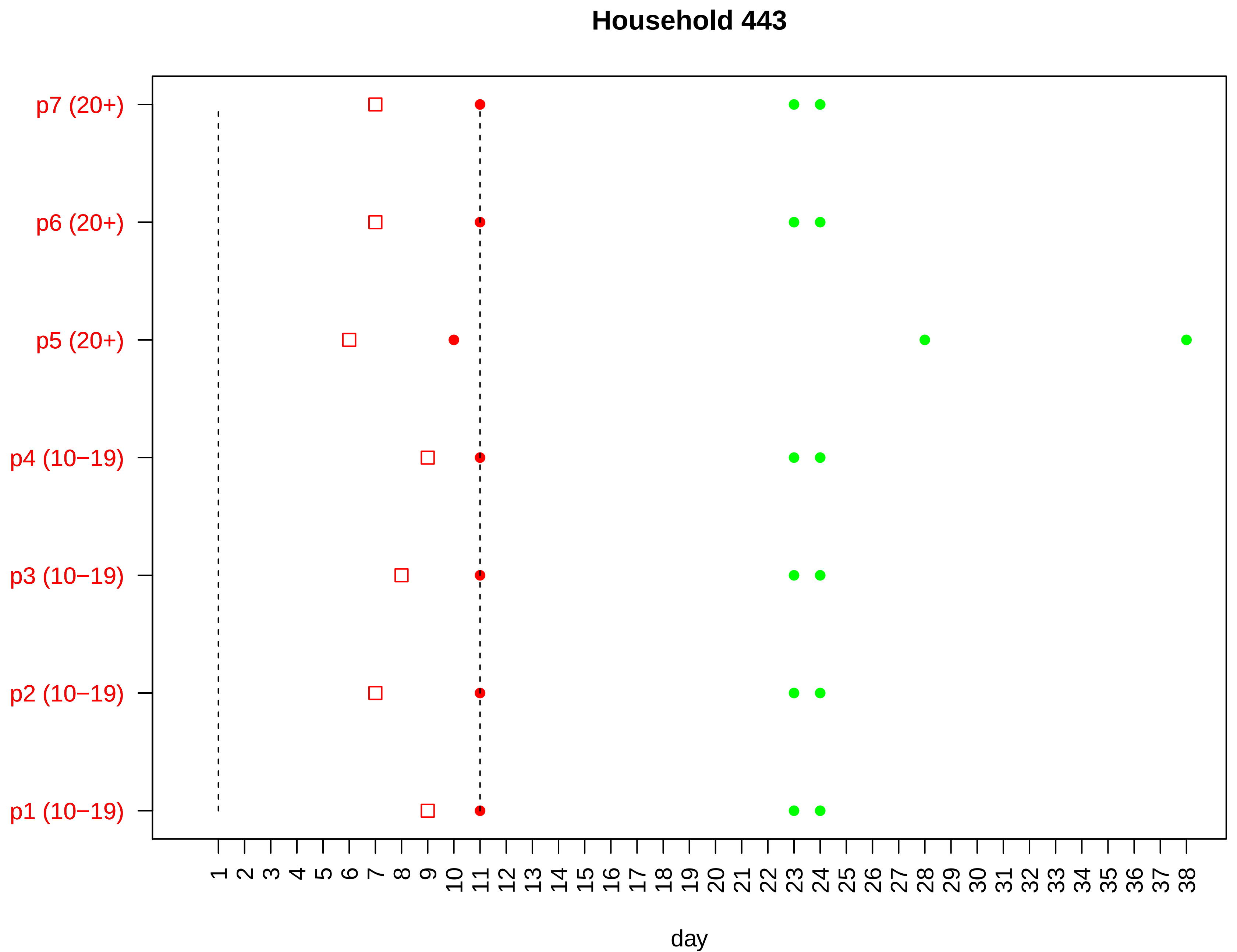
Household 443
p7 (20+)
p6 (20+)
p5 (20+)
p4 (10−19)
p3 (10−19)
p2 (10−19)
p1 (10−19)
1 2 3 4 5 6 7 8 9 10 11 12 13 14 15 16 17 18 19 20 21 22 23 24 25 26 27 28 29 30 31 32 33 34 35 36 37 38
day

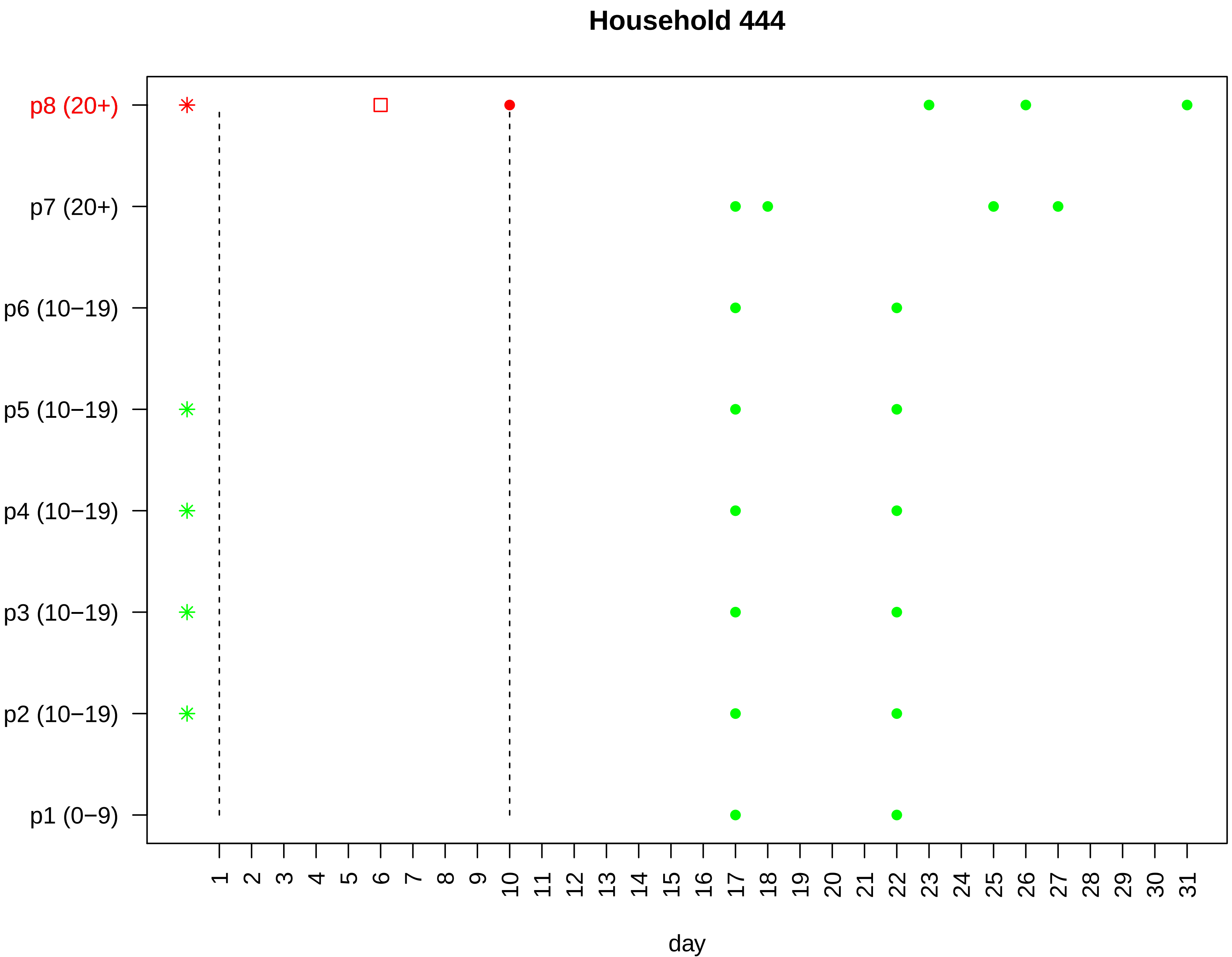

Household 444
p8 (20+)
p7 (20+)
p6 (10−19)
p5 (10−19)
p4 (10−19)
p3 (10−19)
p2 (10−19)
p1 (0−9)
1 2 3 4 5 6 7 8 9 10 11 12 13 14 15 16 17 18 19 20 21 22 23 24 25 26 27 28 29 30 31
day

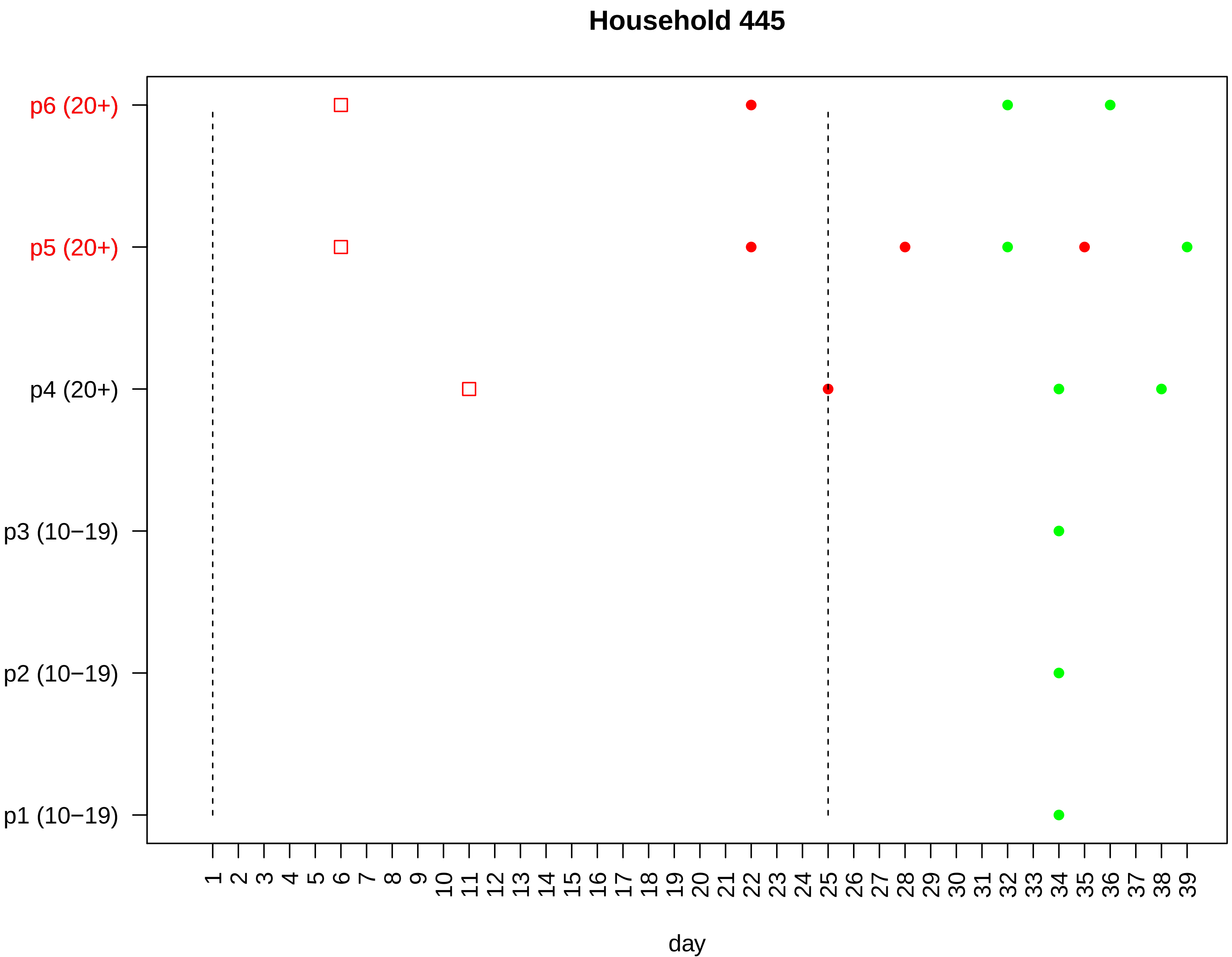

Household 445
p6 (20+)
p5 (20+)
p4 (20+)
p3 (10−19)
p2 (10−19)
p1 (10−19)
1 2 3 4 5 6 7 8 9 10 11 12 13 14 15 16 17 18 19 20 21 22 23 24 25 26 27 28 29 30 31 32 33 34 35 36 37 38 39
day

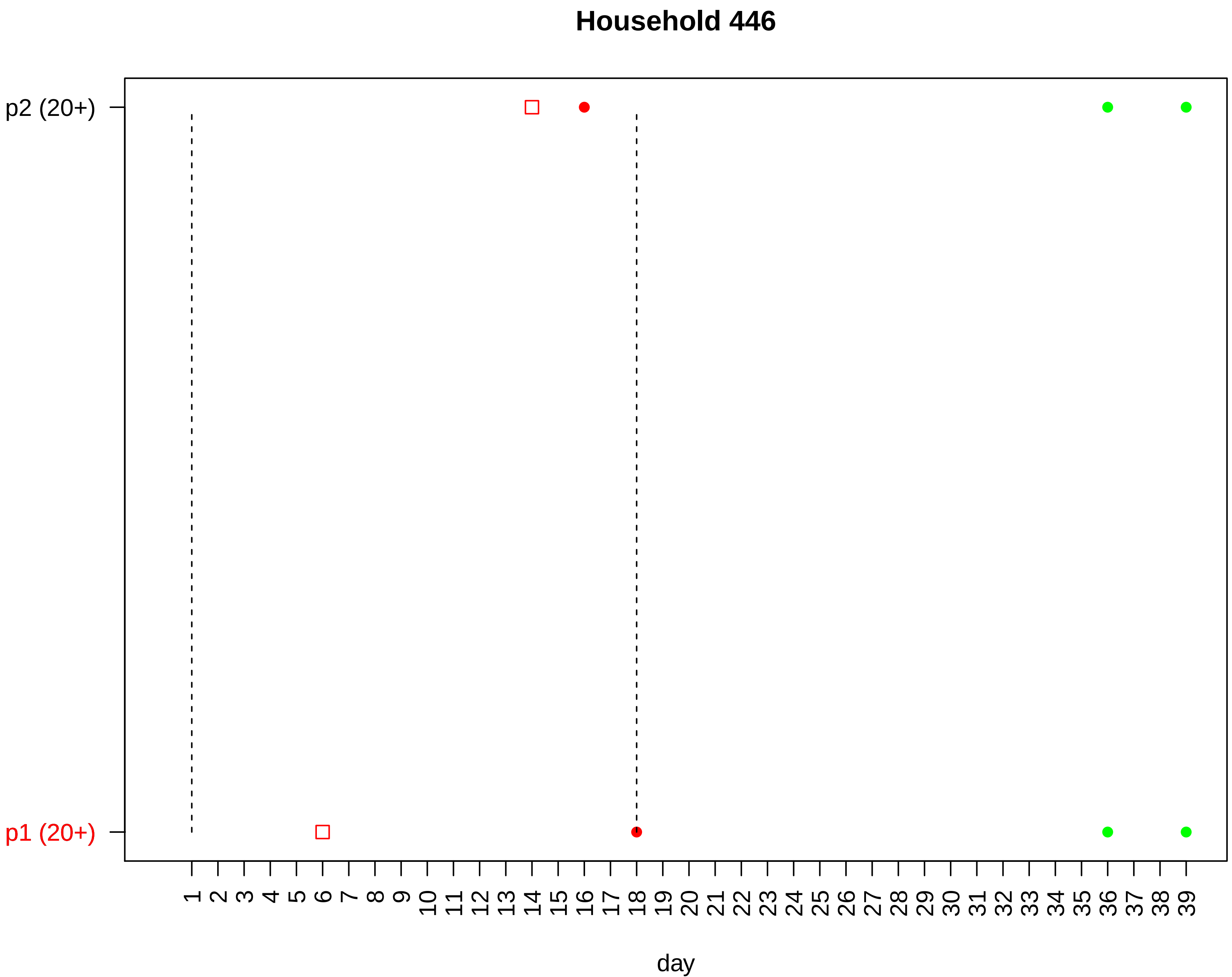

Household 446
p2 (20+)
p1 (20+)
1
2
3
4
5
6
7
8
9
10
11
12
13
14
15
16
17
18
19
20
21
22
23
24
25
26
27
28
29
30
31
32
33
34
35
36
37
38
39
day

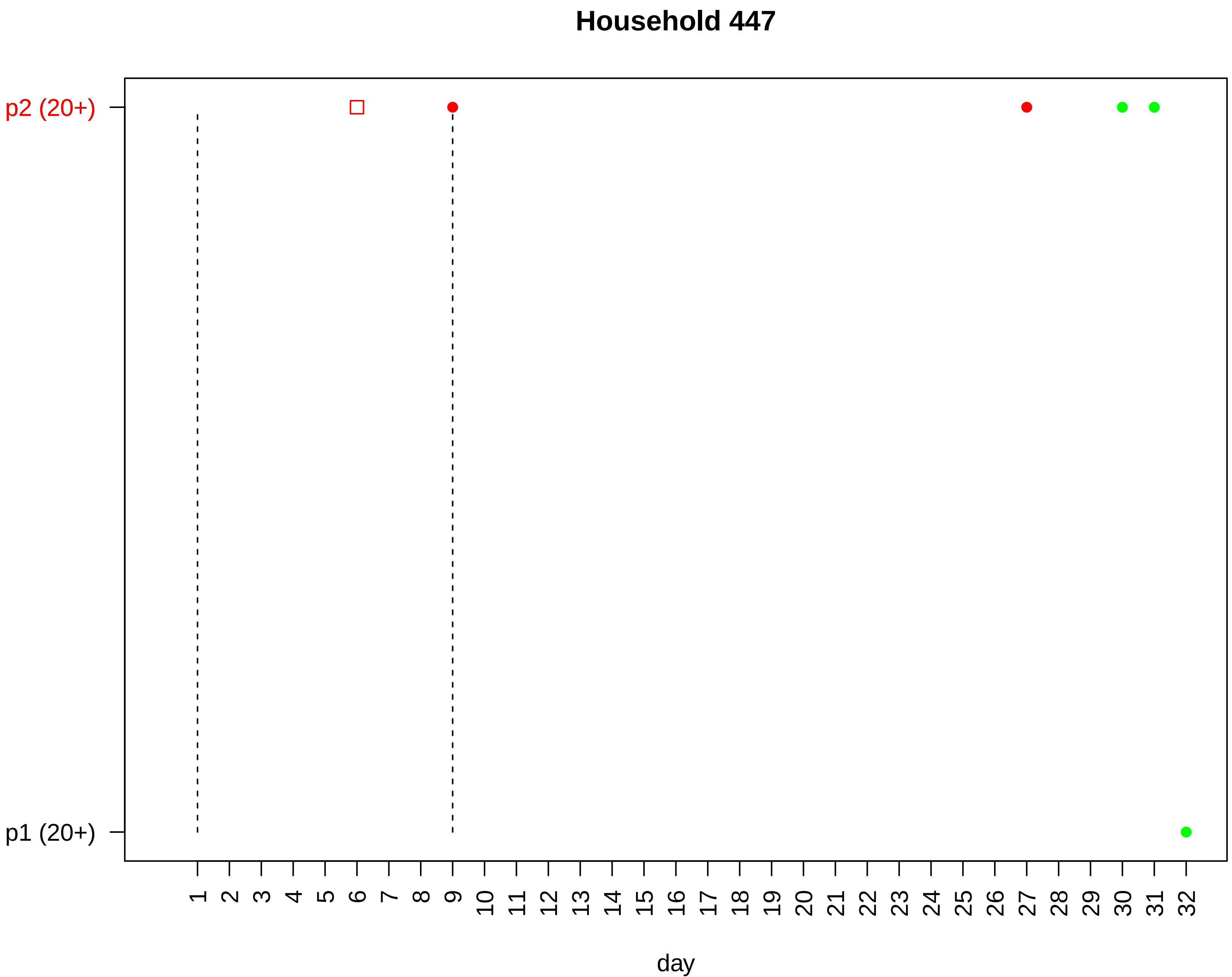

Household 447
p2 (20+)
p1 (20+)
1
2
3
4
5
6
7
8
9
10
11
12
13
14
15
16
17
18
19
20
21
22
23
24
25
26
27
28
29
30
31
32
day

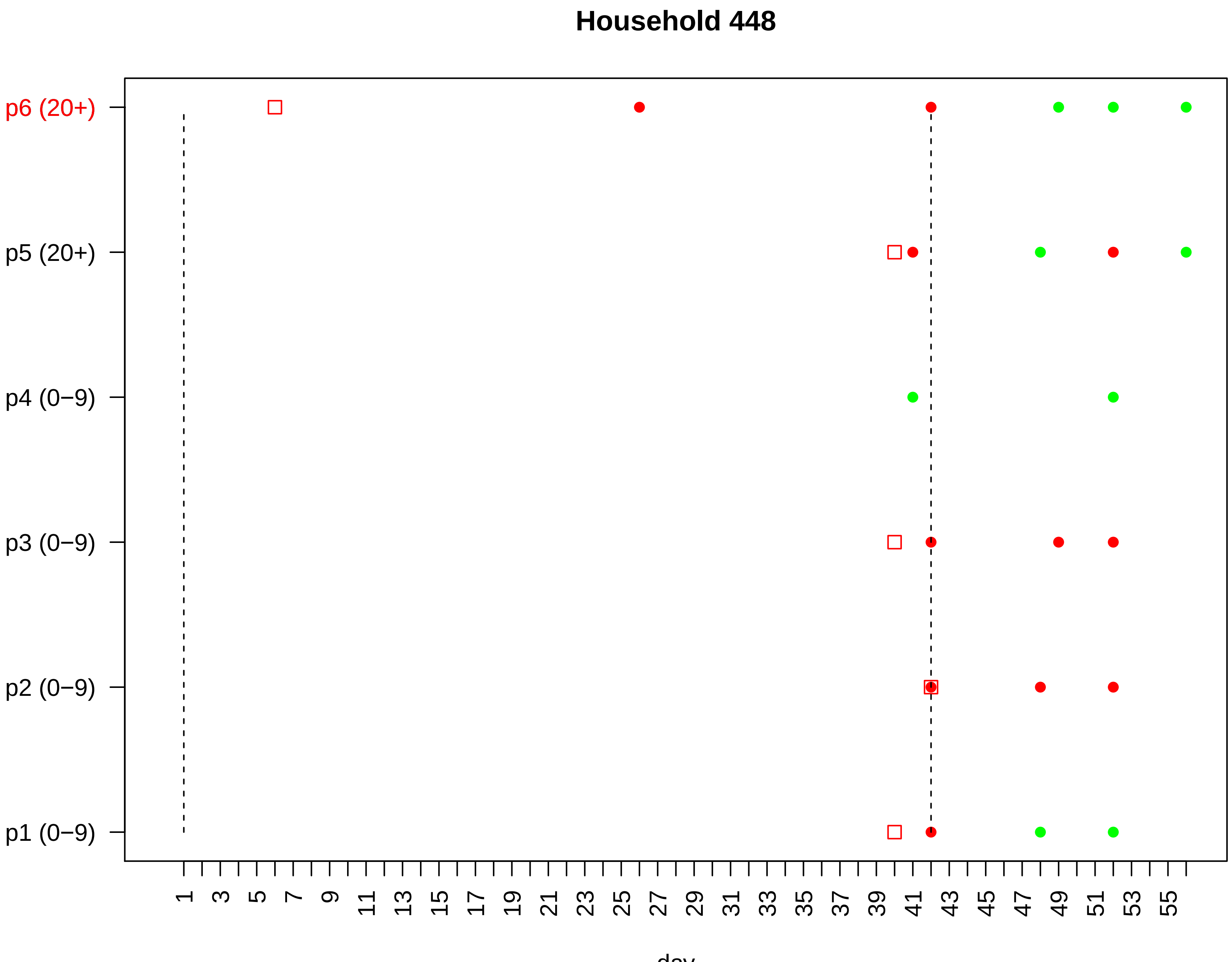

Household 448
p6 (20+)
p5 (20+)
p4 (0−9)
p3 (0−9)
p2 (0−9)
p1 (0−9)
1 3 5 7 9 11 13 15 17 19 21 23 25 27 29 31 33 35 37 39 41 43 45 47 49 51 53 55
day

Household 449

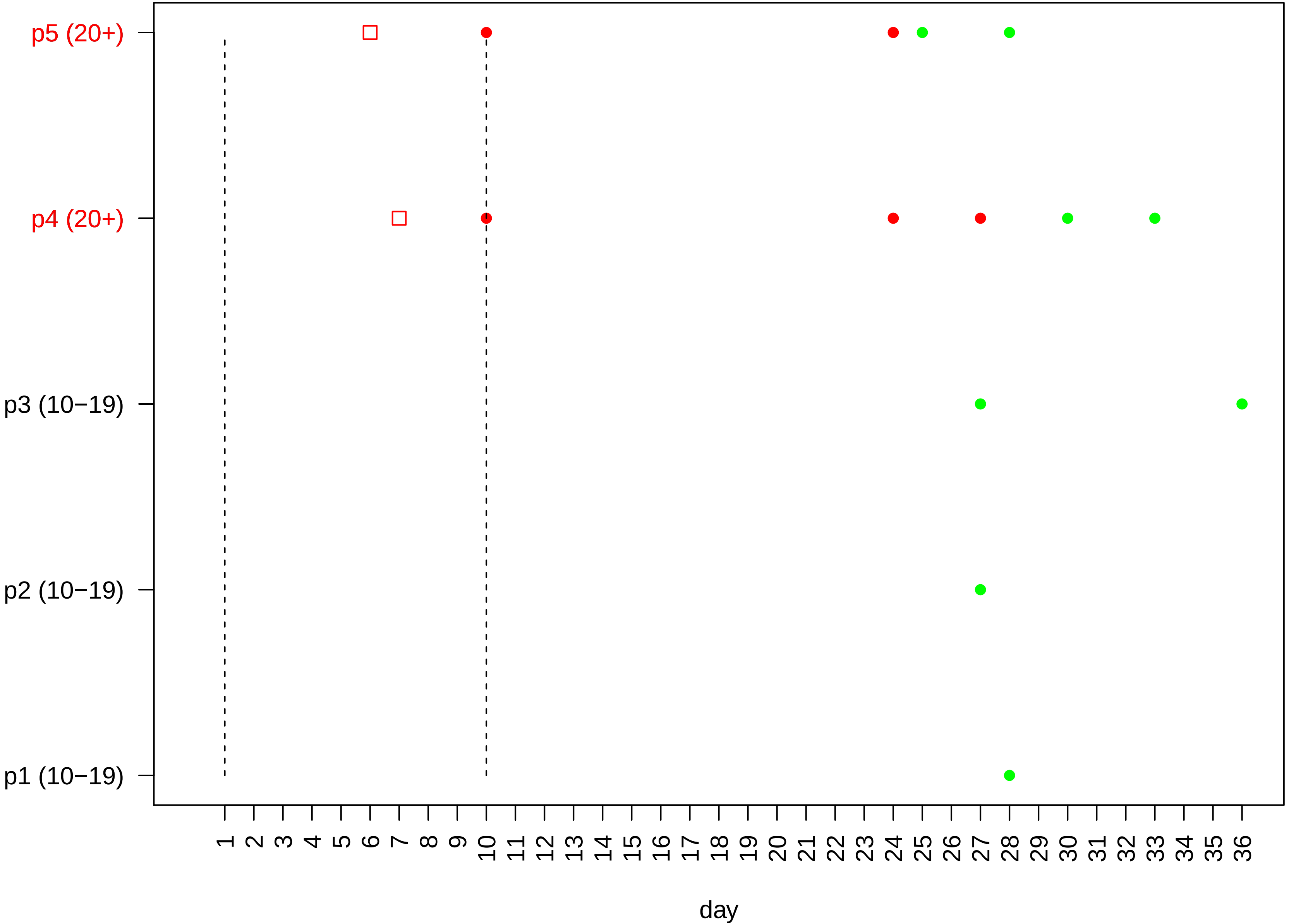

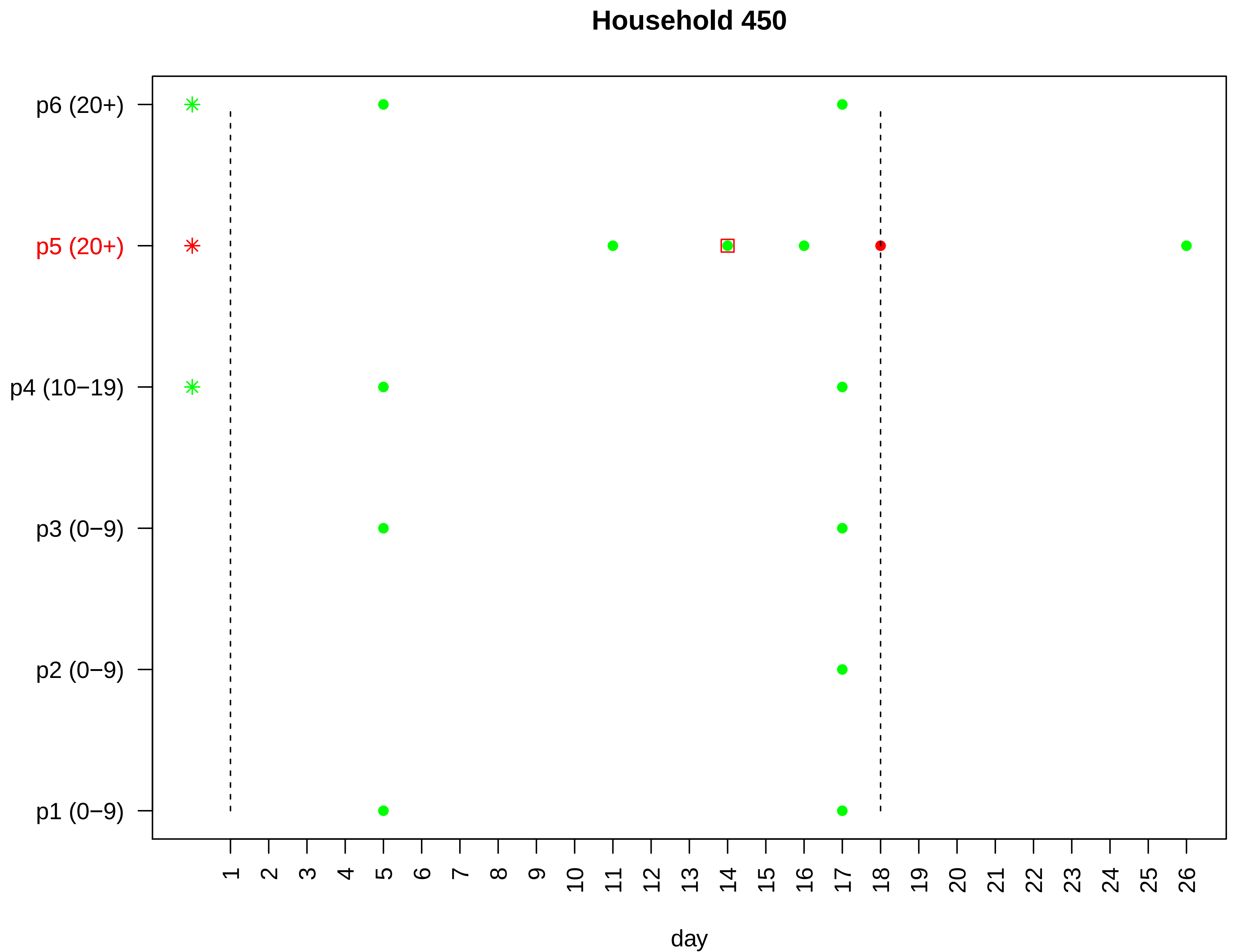

Household 450
p6 (20+)
p5 (20+)
p4 (10−19)
p3 (0−9)
p2 (0−9)
p1 (0−9)
1
2
3
4
5
6
7
8
9
10
11
12
13
14
15
16
17
18
19
20
21
22
23
24
25
26
day

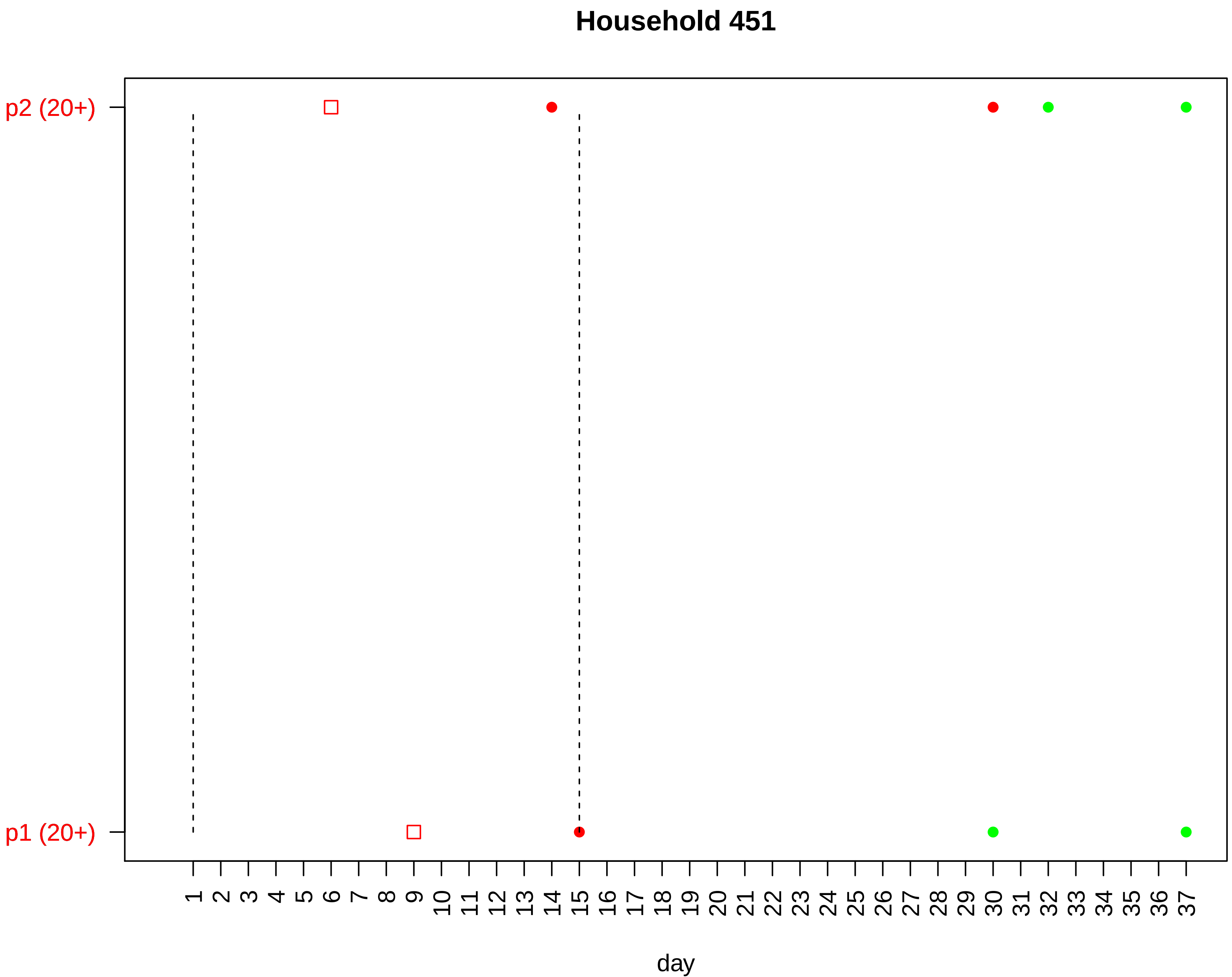
Household 451
p2 (20+)
p1 (20+)
1 2 3 4 5 6 7 8 9 10 11 12 13 14 15 16 17 18 19 20 21 22 23 24 25 26 27 28 29 30 31 32 33 34 35 36 37
day

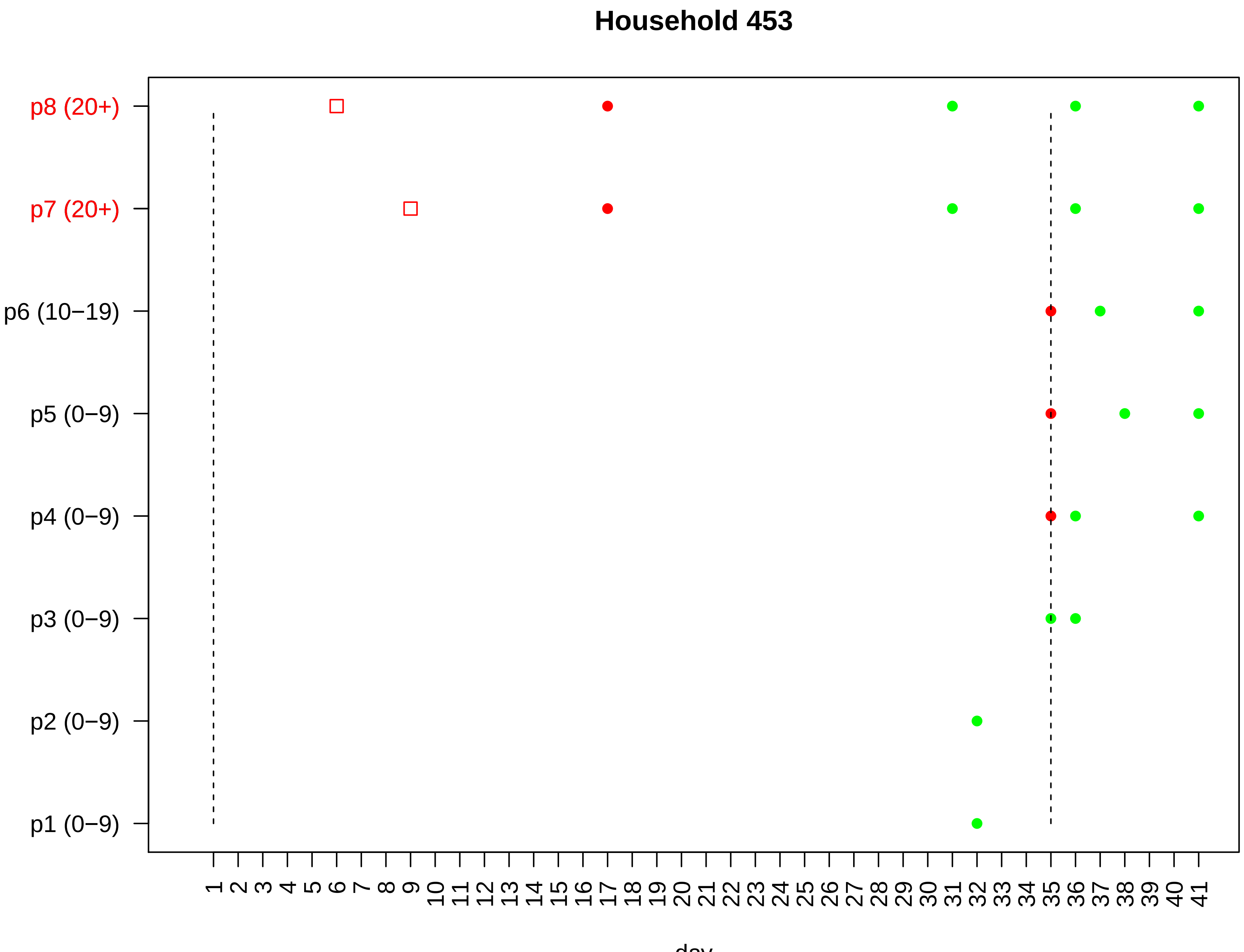

Household 453
p8 (20+)
p7 (20+)
p6 (10−19)
p5 (0−9)
p4 (0−9)
p3 (0−9)
p2 (0−9)
p1 (0−9)
1 2 3 4 5 6 7 8 9 10 11 12 13 14 15 16 17 18 19 20 21 22 23 24 25 26 27 28 29 30 31 32 33 34 35 36 37 38 39 40 41
day

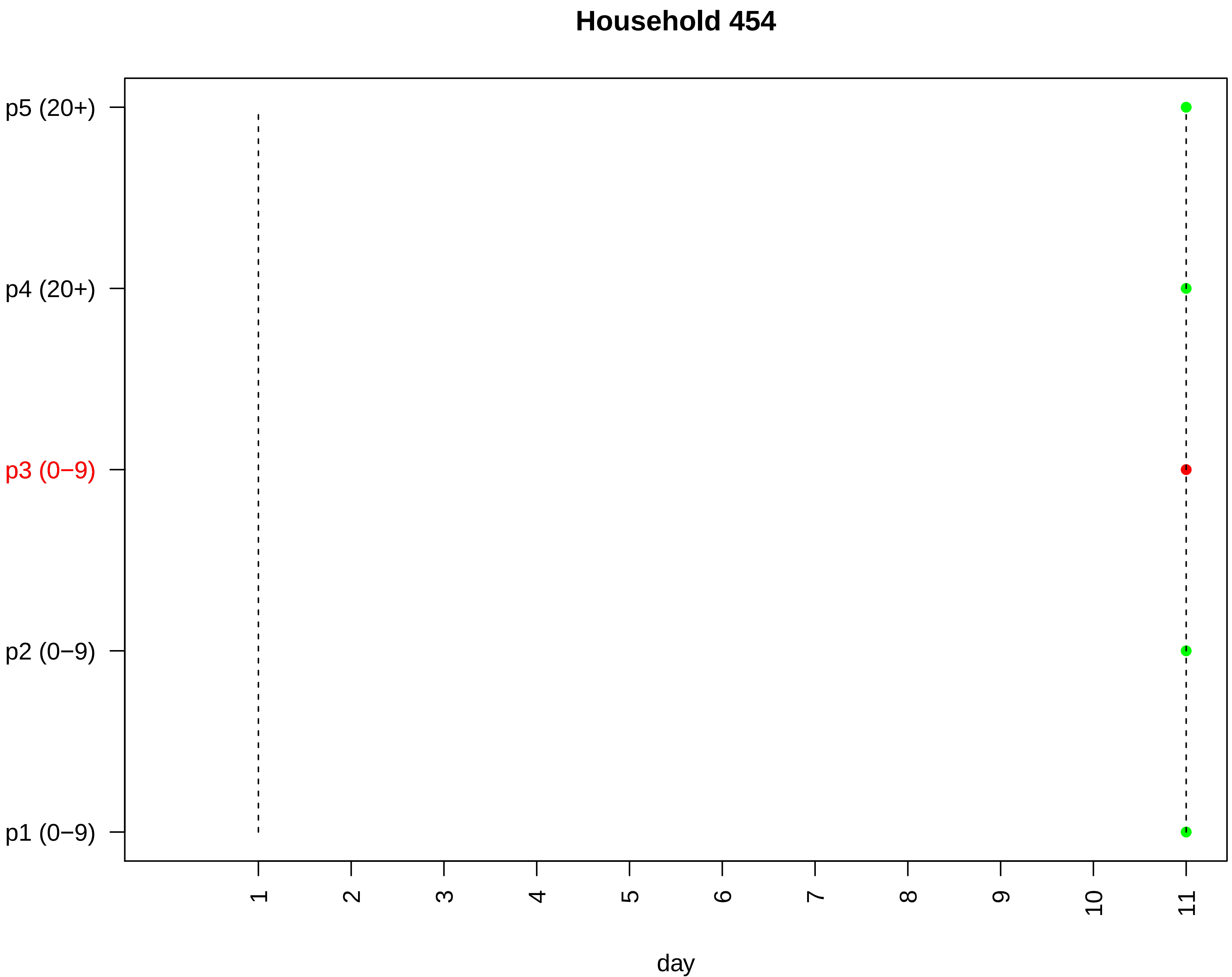

Household 454
p5 (20+)
p4 (20+)
p3 (0−9)
p2 (0−9)
p1 (0−9)
1
2
3
4
5
6
7
8
9
10
11
day

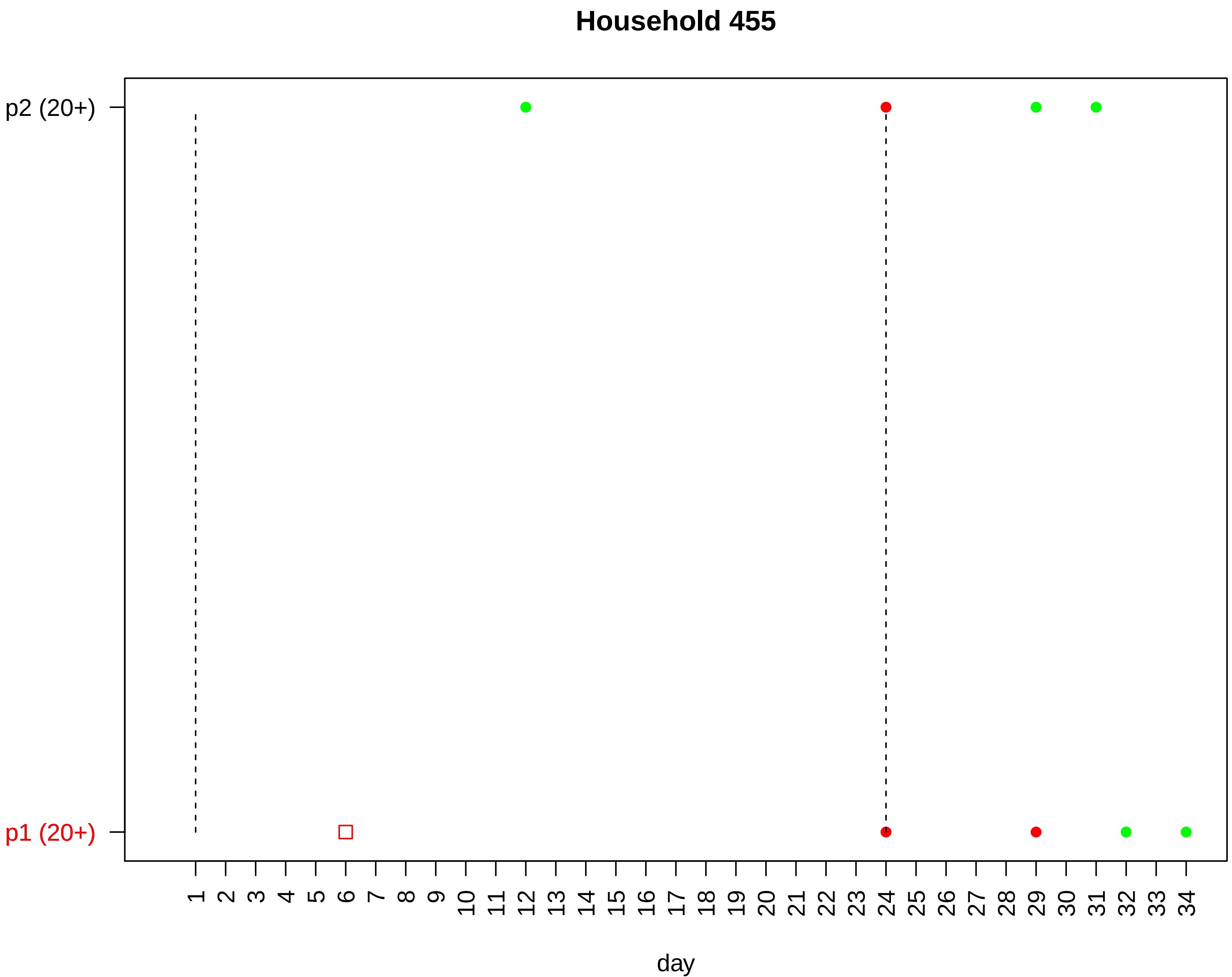

Household 455
p2 (20+)
p1 (20+)
1
2
3
4
5
6
7
8
9
10
11
12
13
14
15
16
17
18
19
20
21
22
23
24
25
26
27
28
29
30
31
32
33
34
day

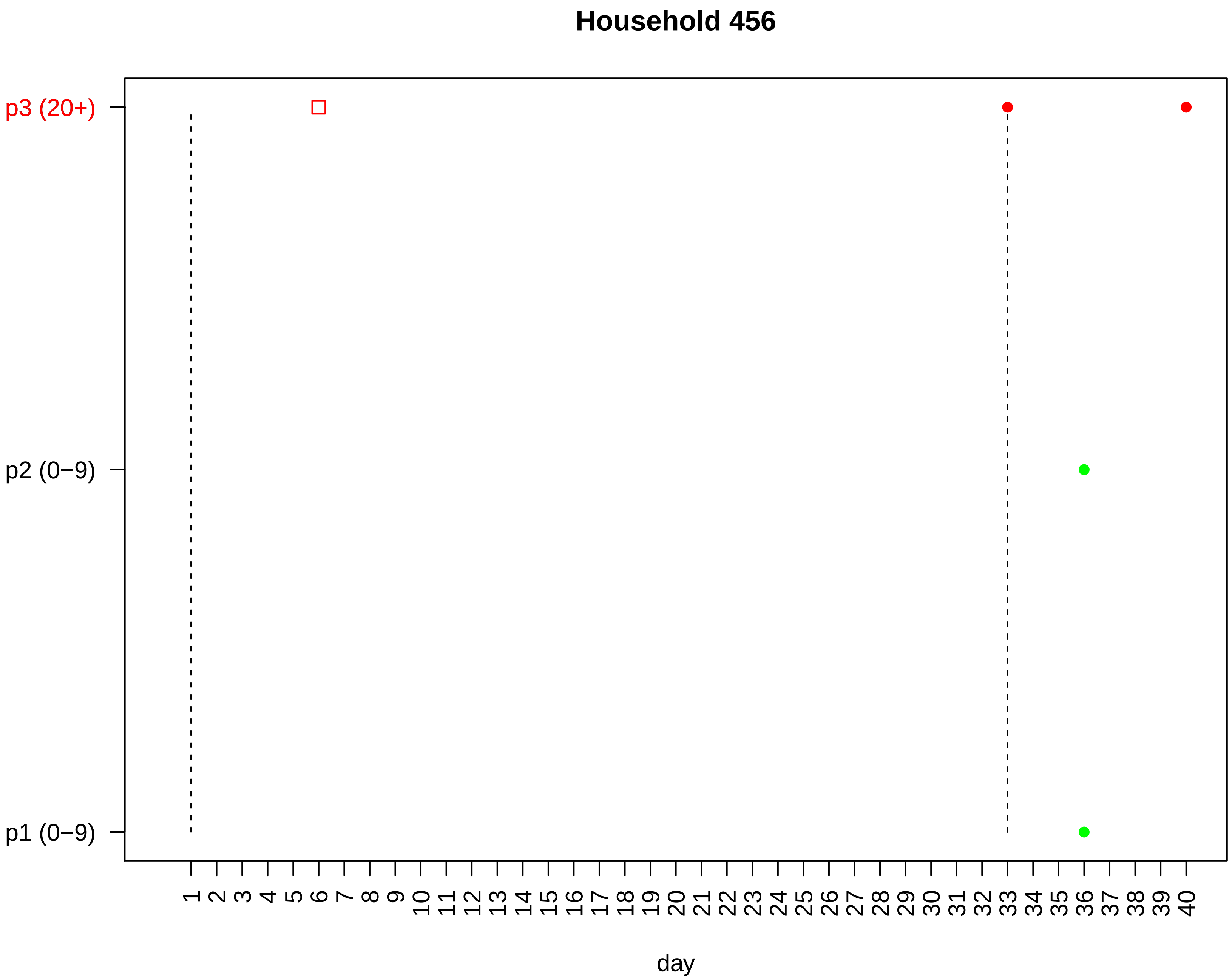

Household 456
p3 (20+)
p2 (0−9)
p1 (0−9)
1 2 3 4 5 6 7 8 9 10 11 12 13 14 15 16 17 18 19 20 21 22 23 24 25 26 27 28 29 30 31 32 33 34 35 36 37 38 39 40
day

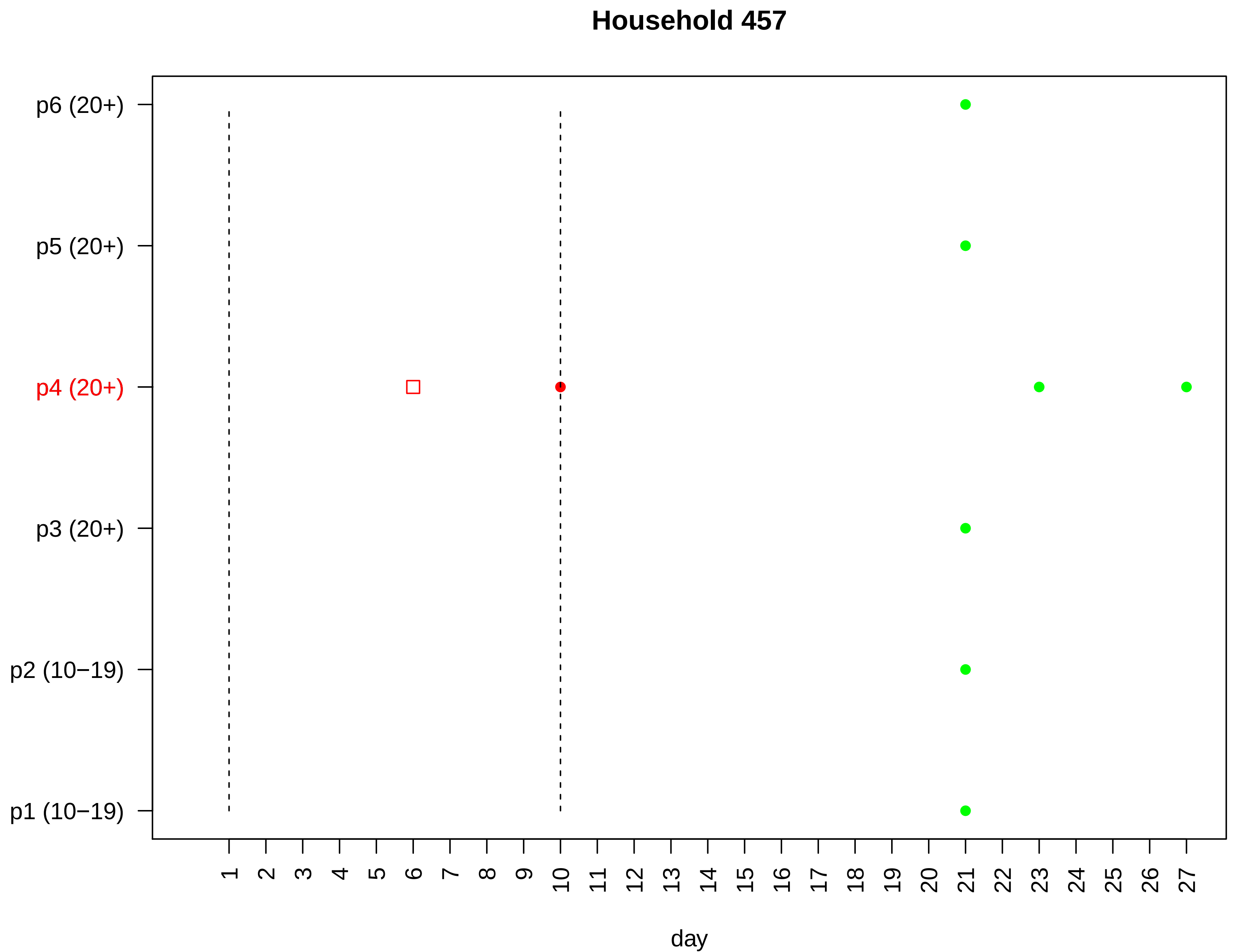

Household 457
p6 (20+)
p5 (20+)
p4 (20+)
p3 (20+)
p2 (10−19)
p1 (10−19)
1 2 3 4 5 6 7 8 9 10 11 12 13 14 15 16 17 18 19 20 21 22 23 24 25 26 27
day

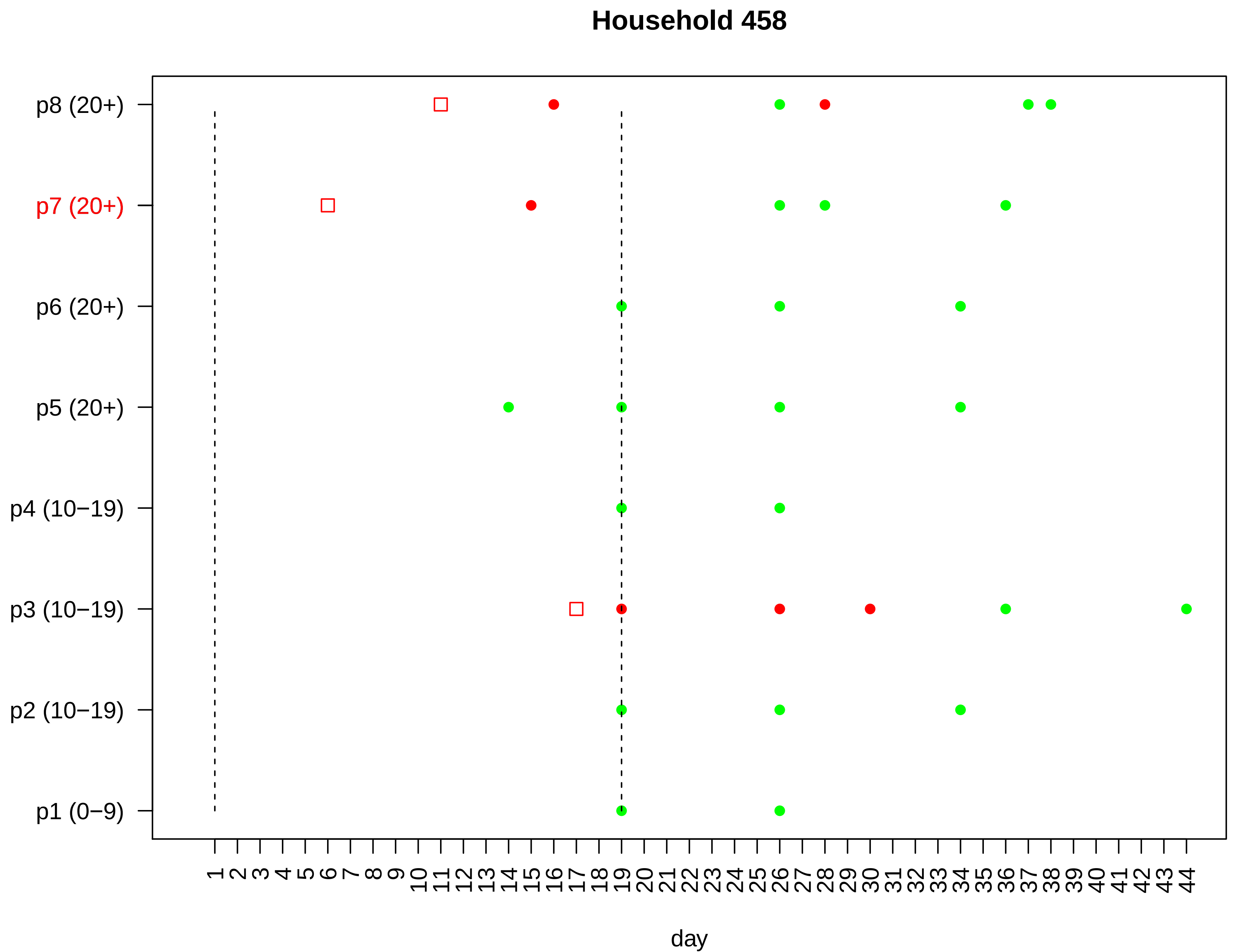
Household 458
p8 (20+)
p7 (20+)
p6 (20+)
p5 (20+)
p4 (10−19)
p3 (10−19)
p2 (10−19)
p1 (0−9)
1 2 3 4 5 6 7 8 9 10 11 12 13 14 15 16 17 18 19 20 21 22 23 24 25 26 27 28 29 30 31 32 33 34 35 36 37 38 39 40 41 42 43 44
day

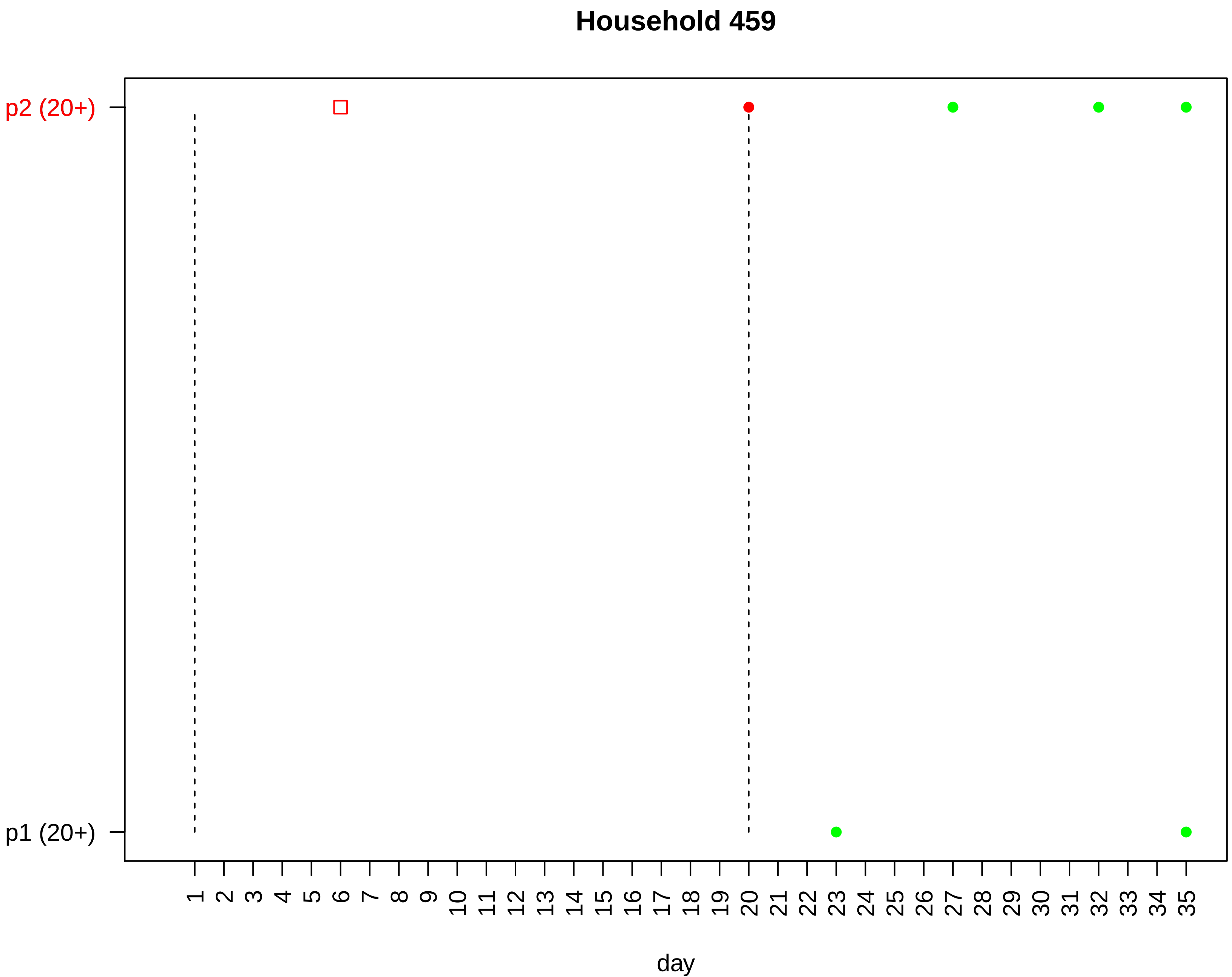

Household 459
p2 (20+)
p1 (20+)
1
2
3
4
5
6
7
8
9
10
11
12
13
14
15
16
17
18
19
20
21
22
23
24
25
26
27
28
29
30
31
32
33
34
35
day

Household 460

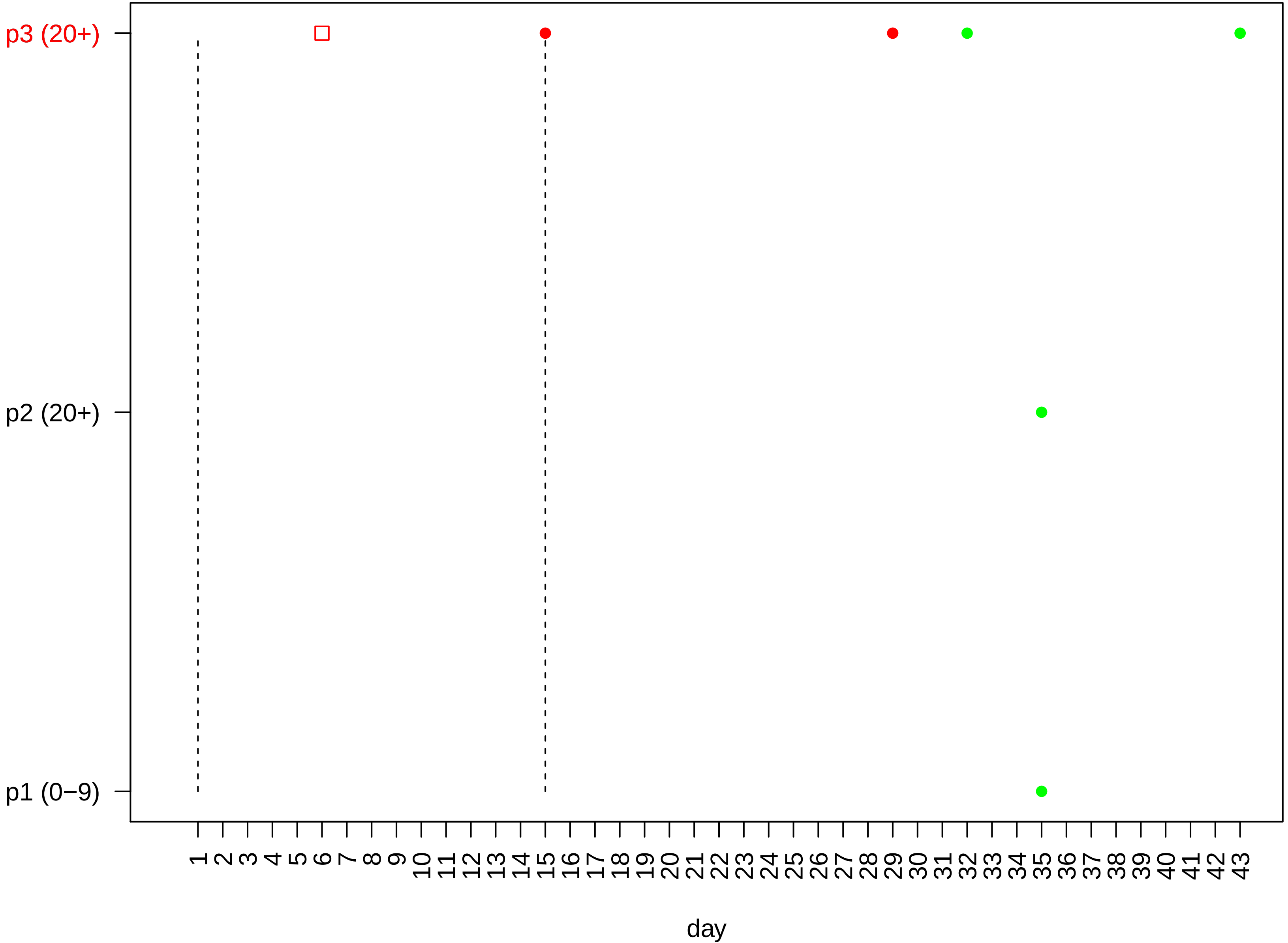

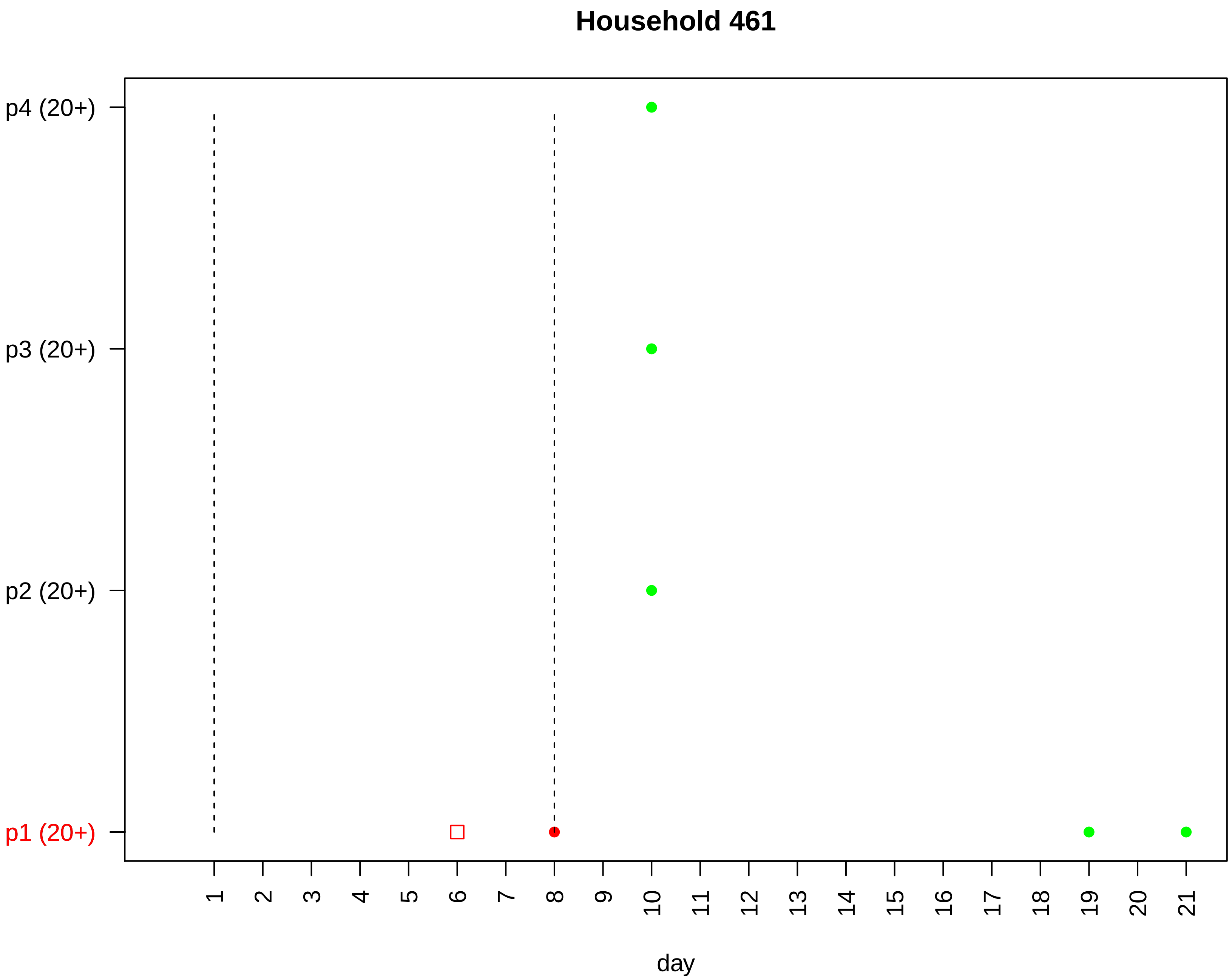

Household 461
p4 (20+)
p3 (20+)
p2 (20+)
p1 (20+)
1
2
3
4
5
6
7
8
9
10
11
12
13
14
15
16
17
18
19
20
21
day

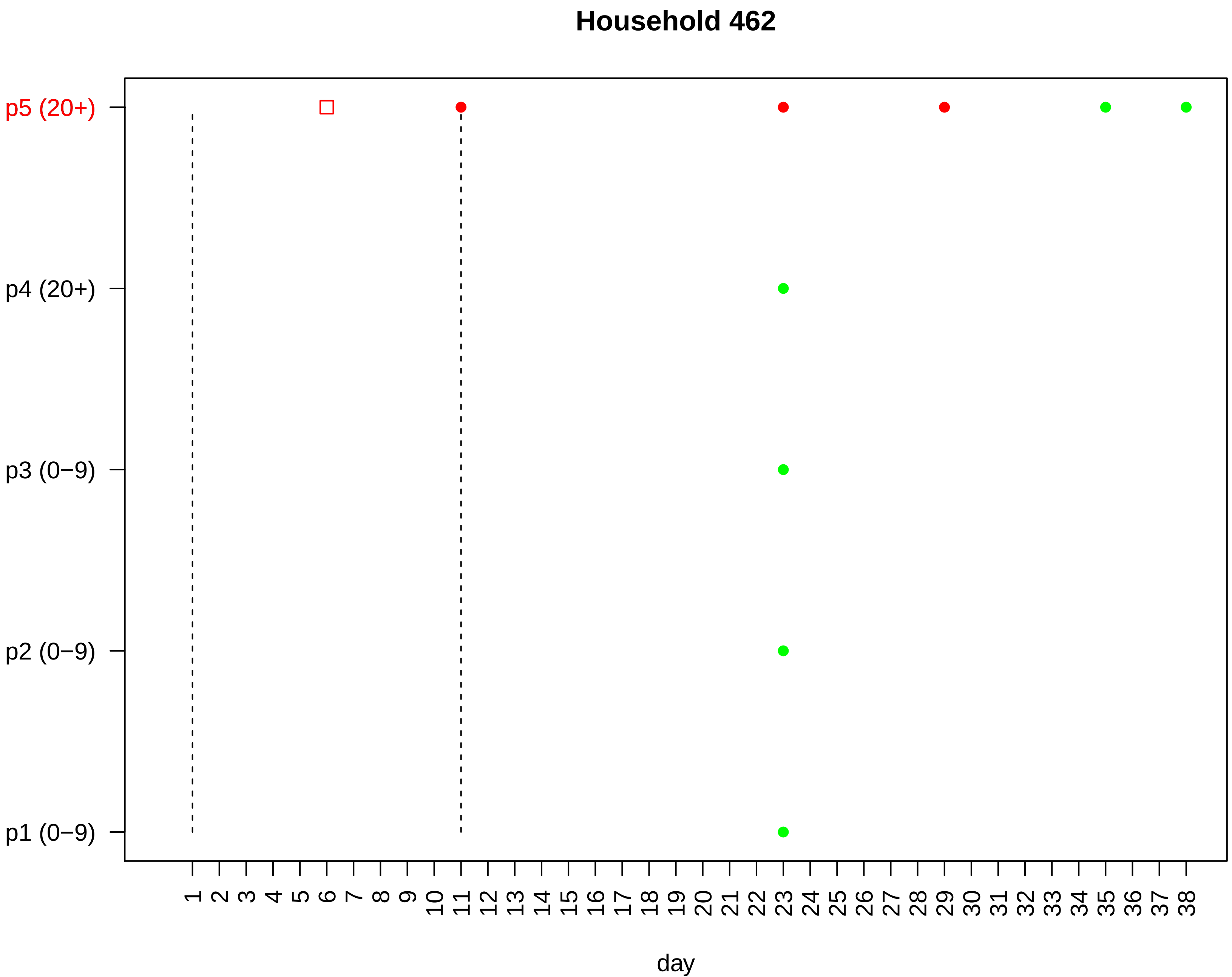

Household 462
p5 (20+)
p4 (20+)
p3 (0−9)
p2 (0−9)
p1 (0−9)
1 2 3 4 5 6 7 8 9 10 11 12 13 14 15 16 17 18 19 20 21 22 23 24 25 26 27 28 29 30 31 32 33 34 35 36 37 38
day

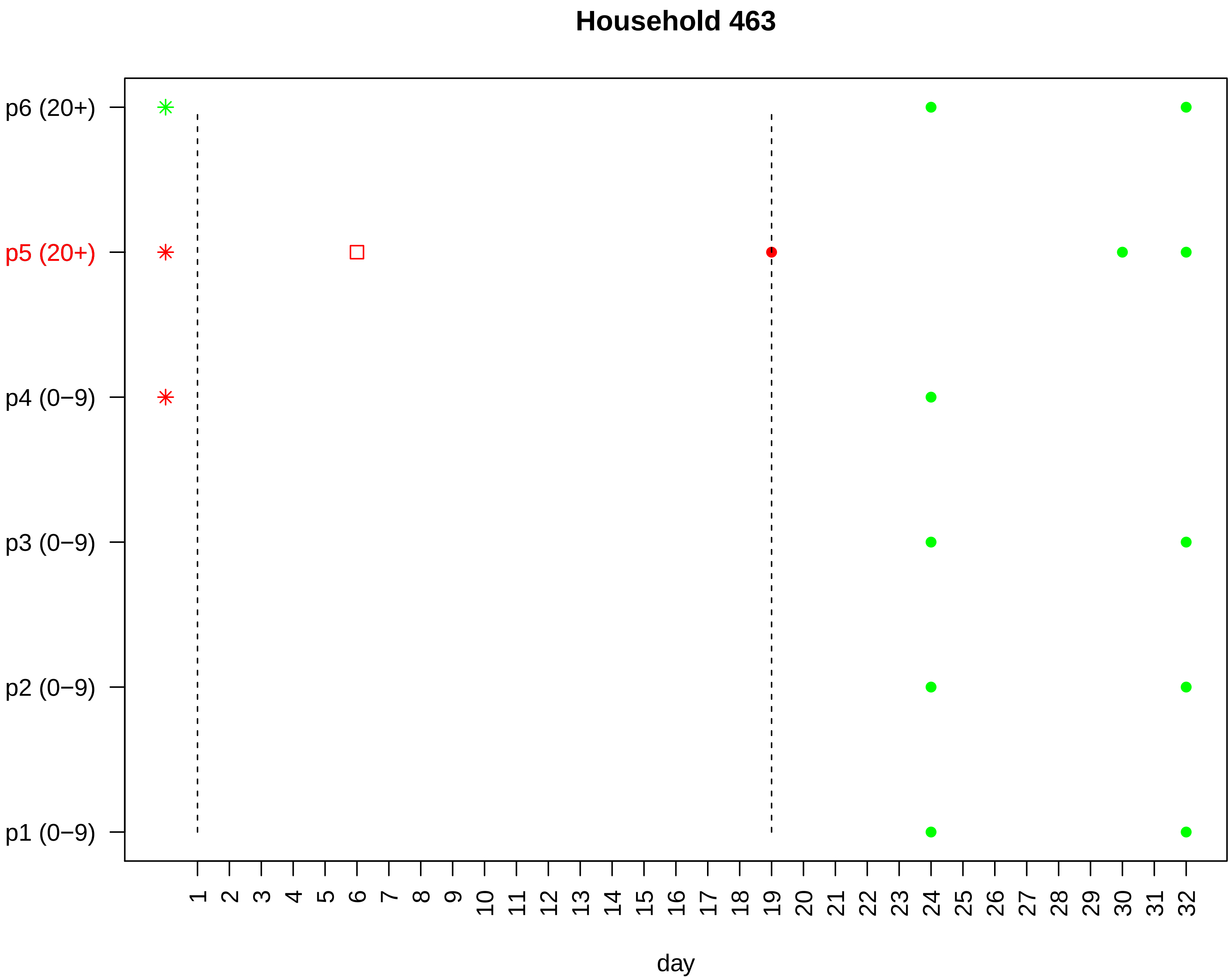

Household 463
p6 (20+)
p5 (20+)
p4 (0−9)
p3 (0−9)
p2 (0−9)
p1 (0−9)
1 2 3 4 5 6 7 8 9 10 11 12 13 14 15 16 17 18 19 20 21 22 23 24 25 26 27 28 29 30 31 32
day

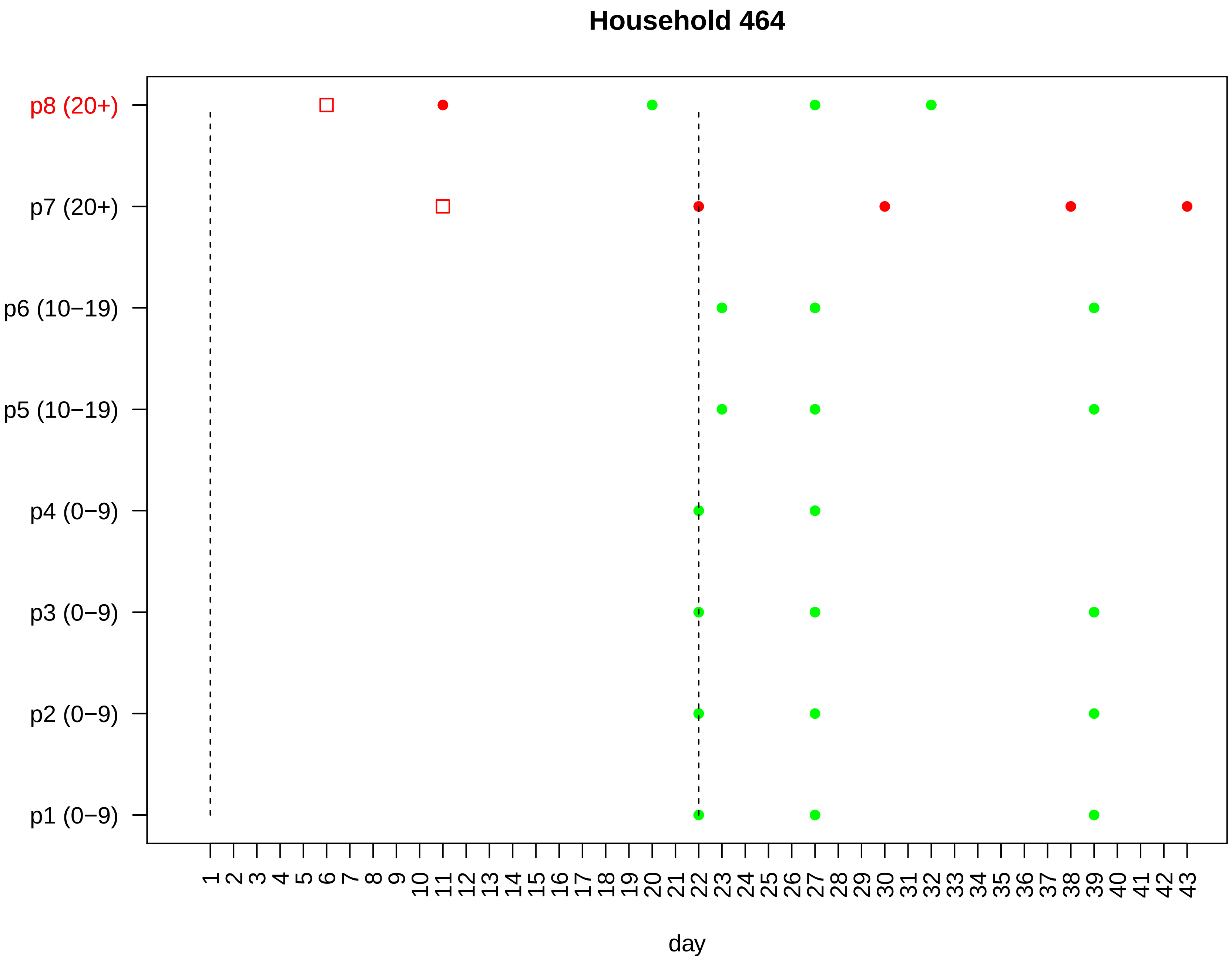

Household 464
p8 (20+)
p7 (20+)
p6 (10−19)
p5 (10−19)
p4 (0−9)
p3 (0−9)
p2 (0−9)
p1 (0−9)
1 2 3 4 5 6 7 8 9 10 11 12 13 14 15 16 17 18 19 20 21 22 23 24 25 26 27 28 29 30 31 32 33 34 35 36 37 38 39 40 41 42 43
day

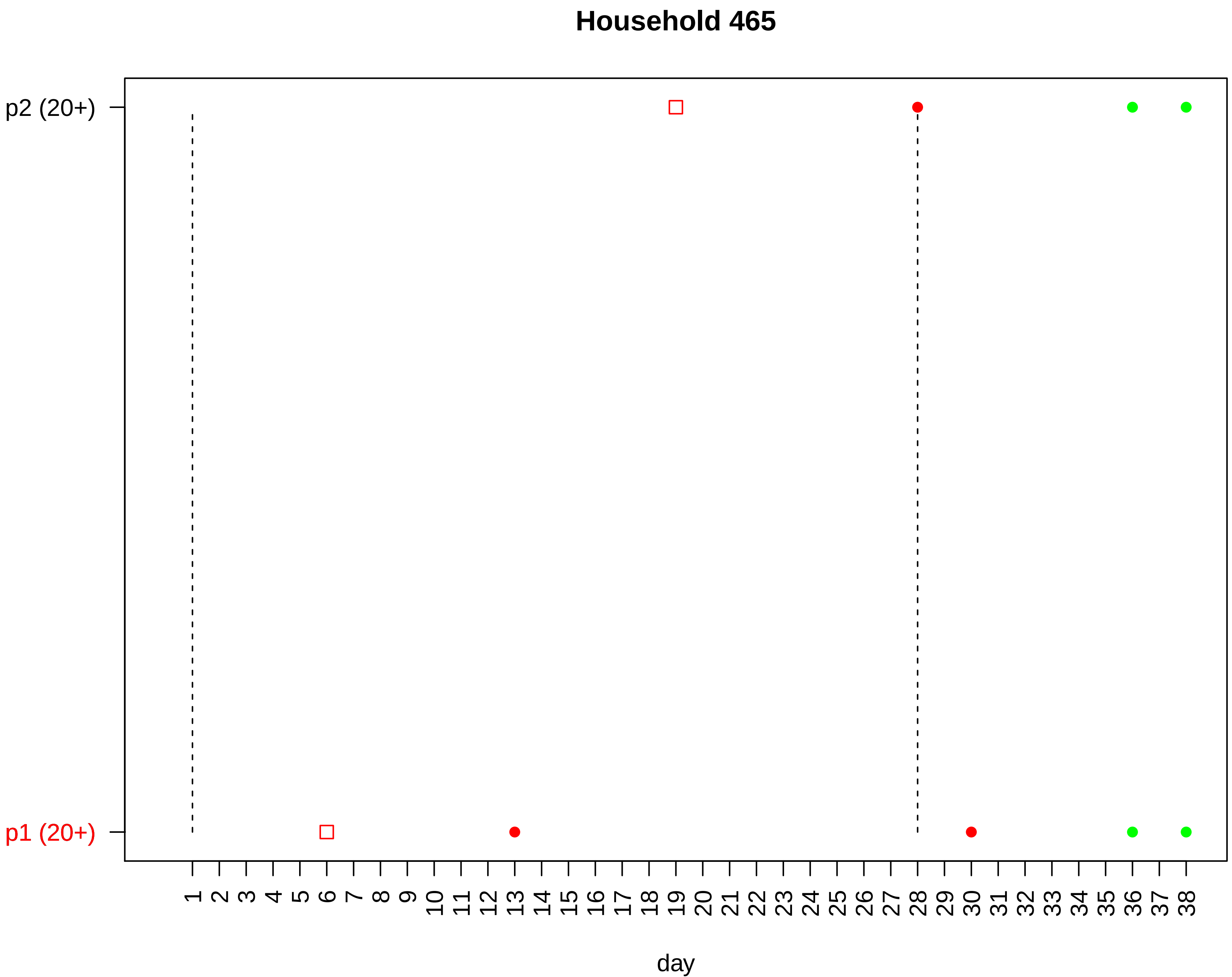
Household 465
p2 (20+)
p1 (20+)
1 2 3 4 5 6 7 8 9 10 11 12 13 14 15 16 17 18 19 20 21 22 23 24 25 26 27 28 29 30 31 32 33 34 35 36 37 38
day

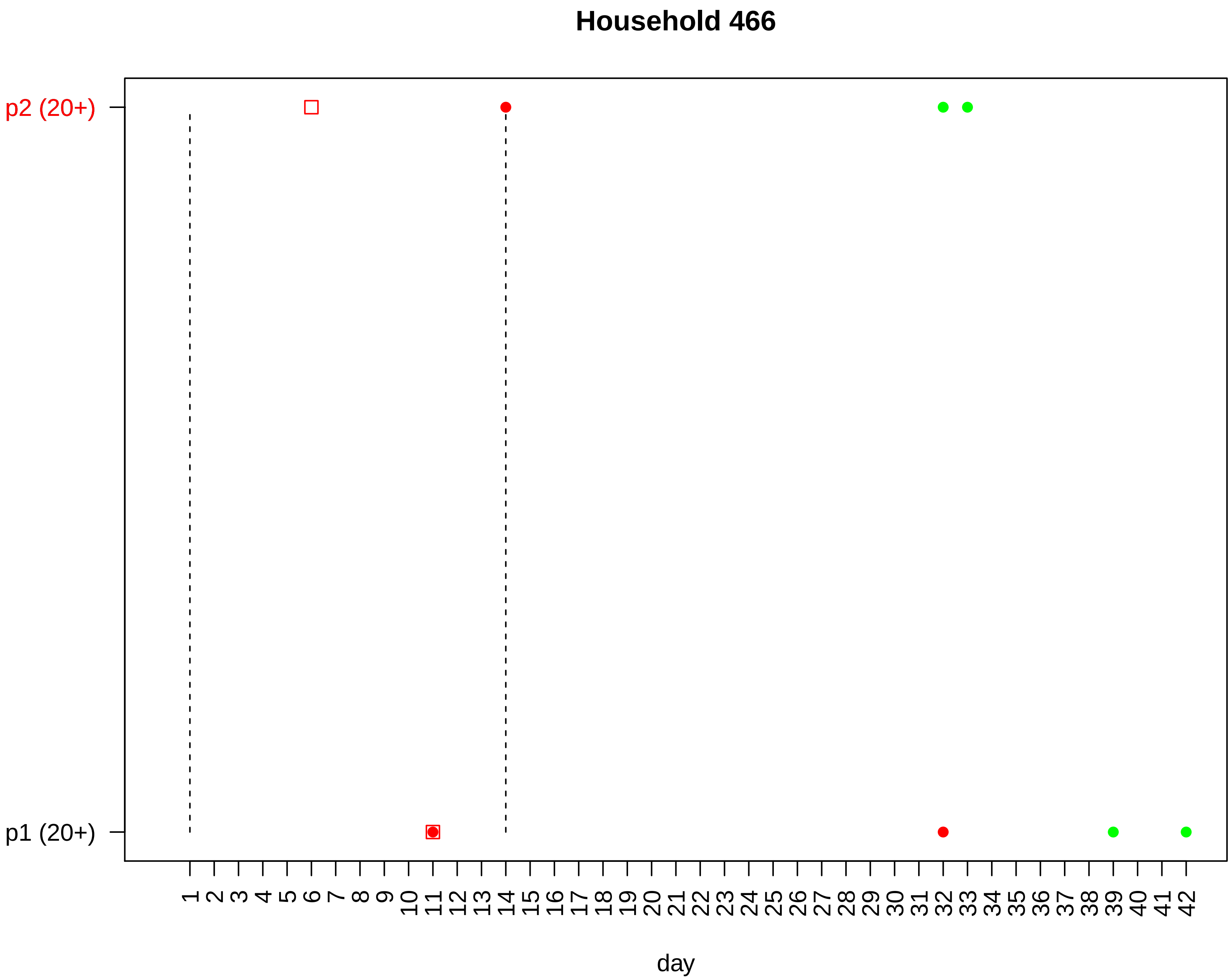

Household 466
p2 (20+)
p1 (20+)
1 2 3 4 5 6 7 8 9 10 11 12 13 14 15 16 17 18 19 20 21 22 23 24 25 26 27 28 29 30 31 32 33 34 35 36 37 38 39 40 41 42
day

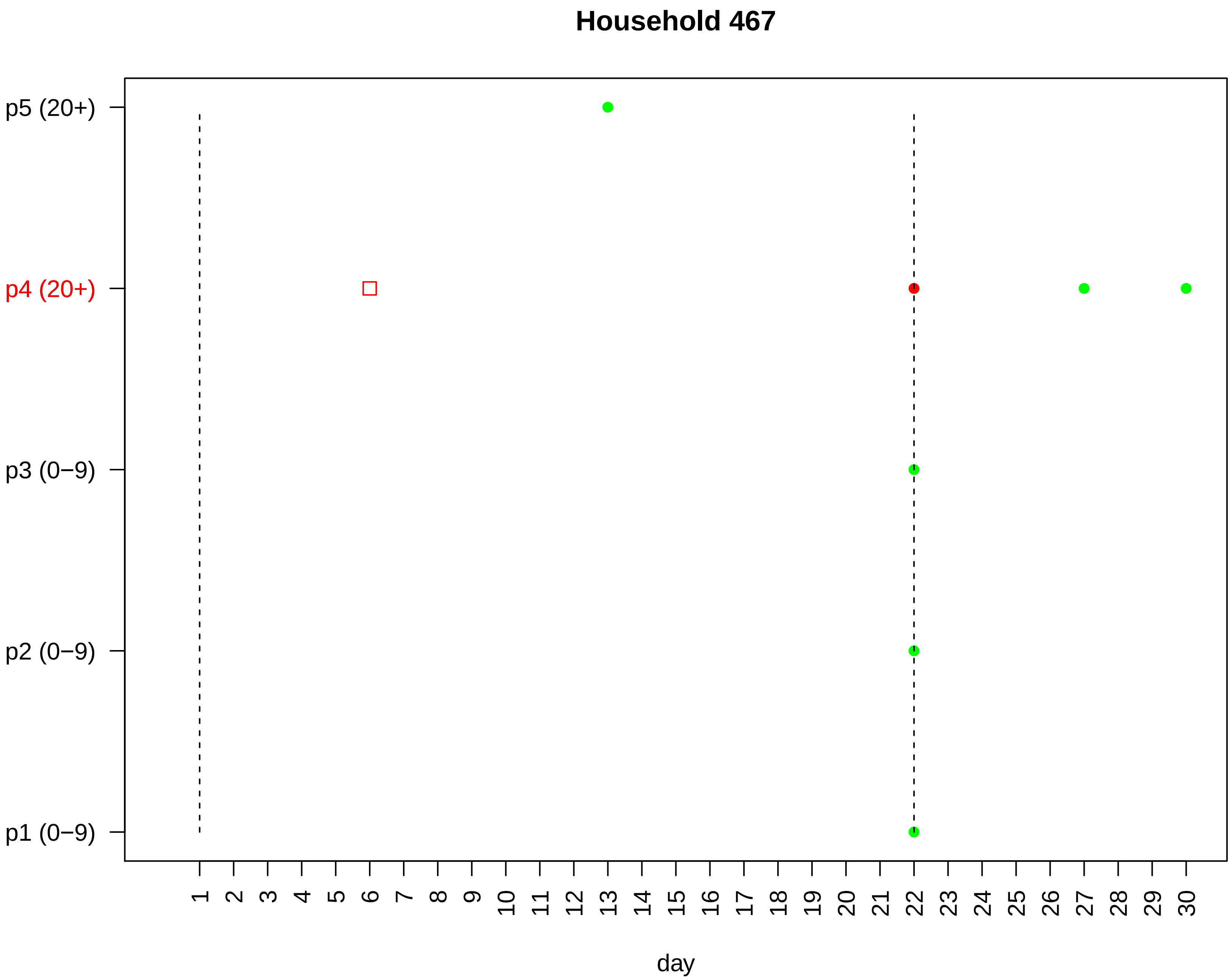
Household 467
p5 (20+)
p4 (20+)
p3 (0−9)
p2 (0−9)
p1 (0−9)
1 2 3 4 5 6 7 8 9 10 11 12 13 14 15 16 17 18 19 20 21 22 23 24 25 26 27 28 29 30
day

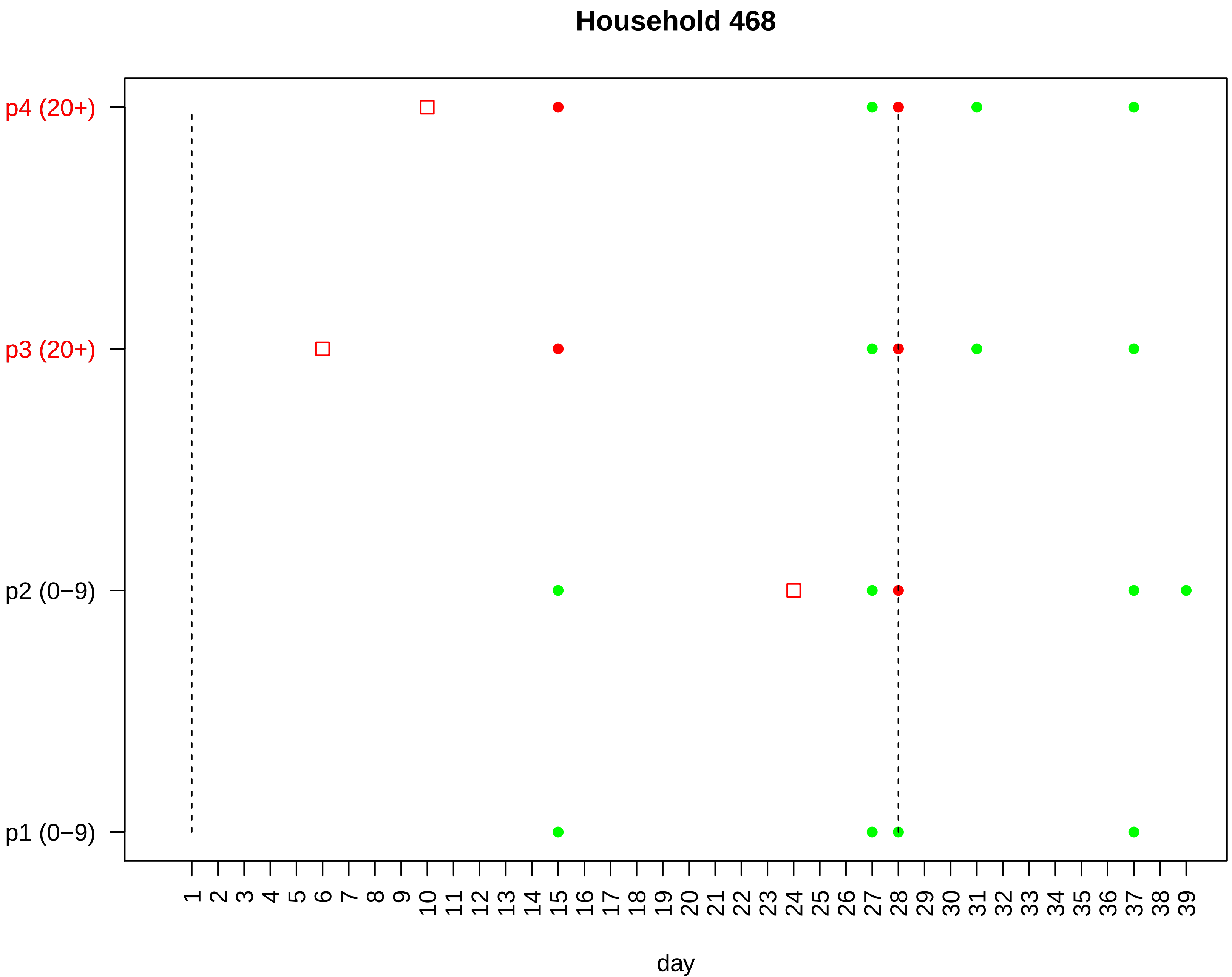

Household 468
p4 (20+)
p3 (20+)
p2 (0−9)
p1 (0−9)
1 2 3 4 5 6 7 8 9 10 11 12 13 14 15 16 17 18 19 20 21 22 23 24 25 26 27 28 29 30 31 32 33 34 35 36 37 38 39
day

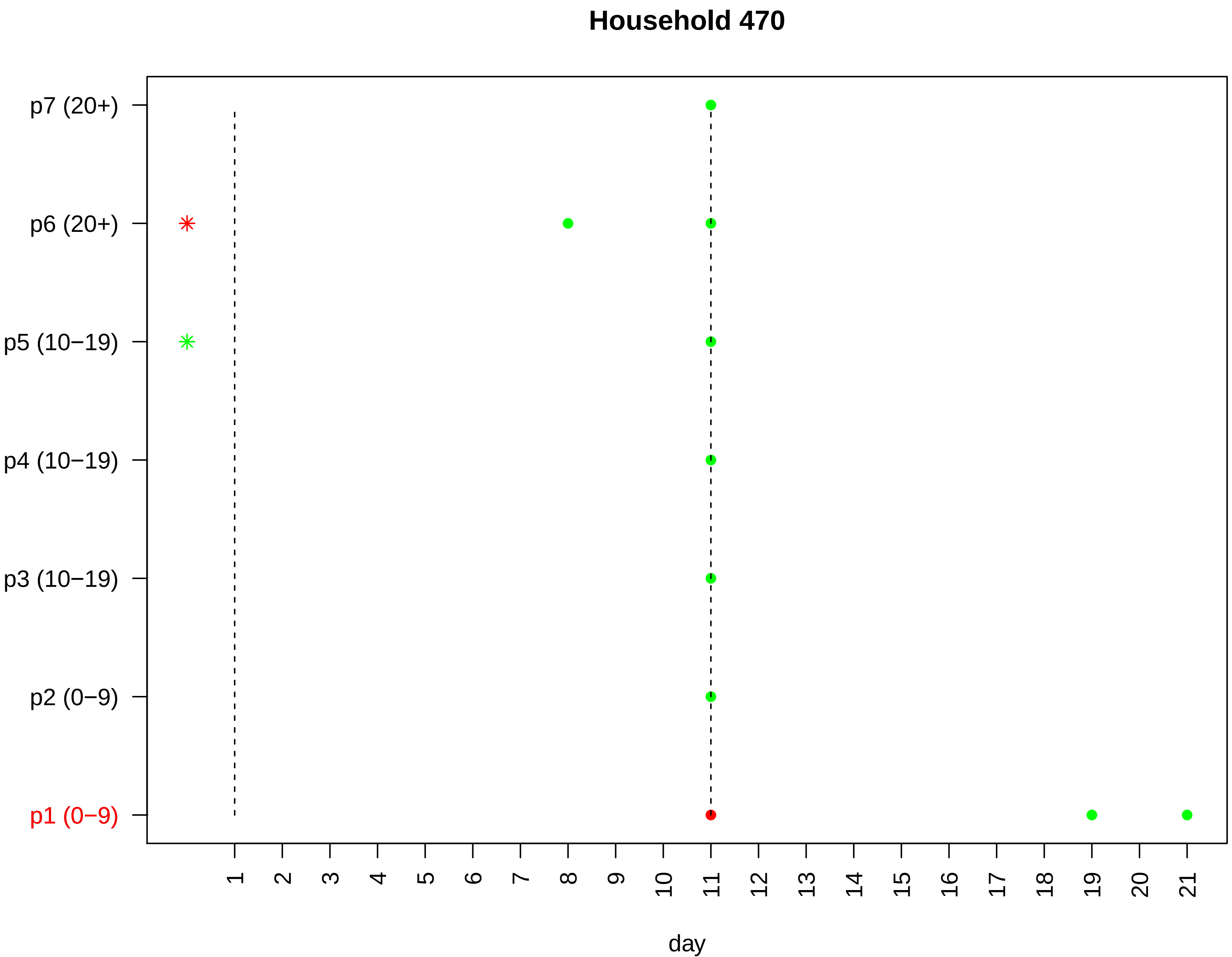
Household 470
p7 (20+)
p6 (20+)
p5 (10−19)
p4 (10−19)
p3 (10−19)
p2 (0−9)
p1 (0−9)
1
2
3
4
5
6
7
8
9
10
11
12
13
14
15
16
17
18
19
20
21
day

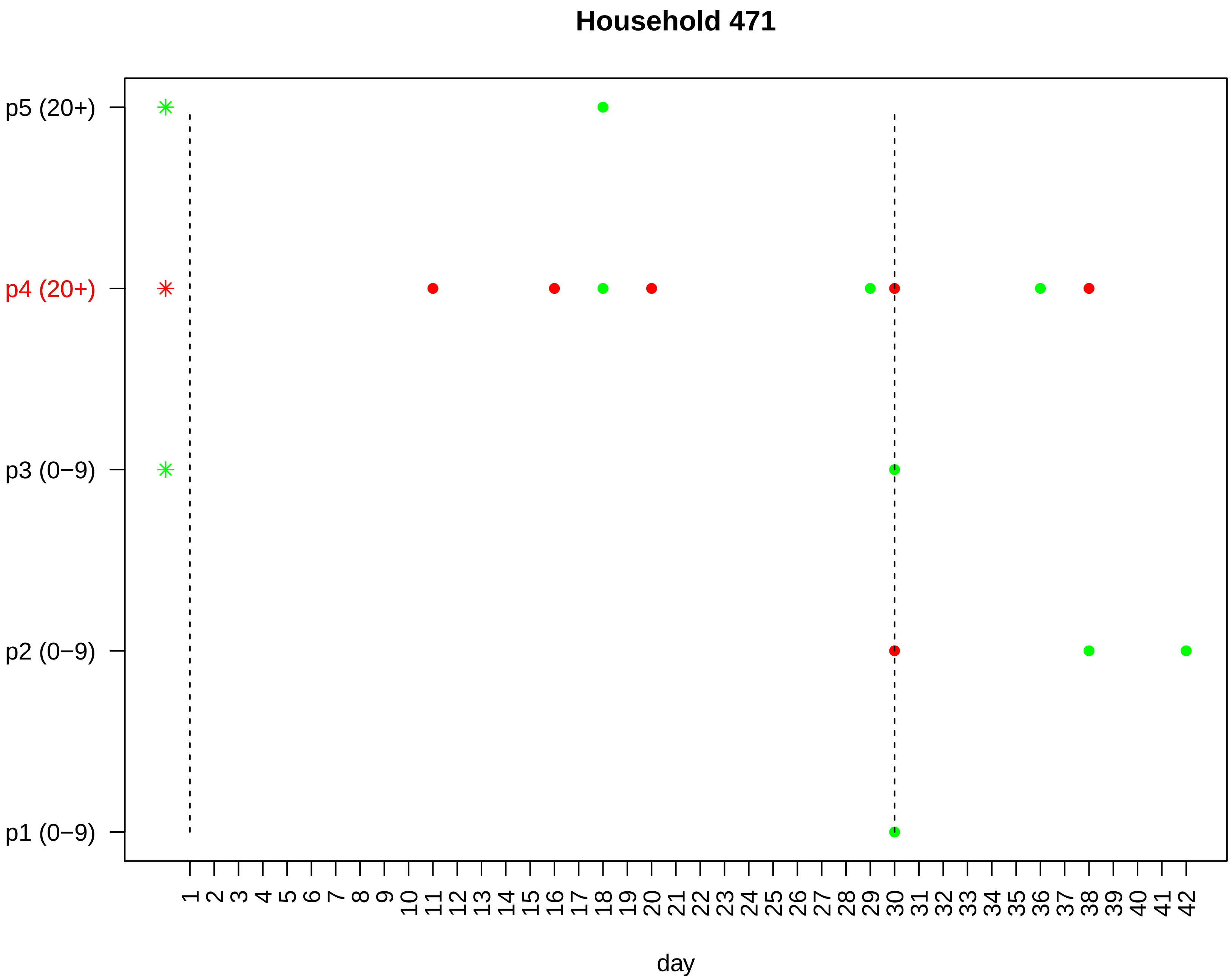

Household 471
p5 (20+)
p4 (20+)
p3 (0−9)
p2 (0−9)
p1 (0−9)
1 2 3 4 5 6 7 8 9 10 11 12 13 14 15 16 17 18 19 20 21 22 23 24 25 26 27 28 29 30 31 32 33 34 35 36 37 38 39 40 41 42
day

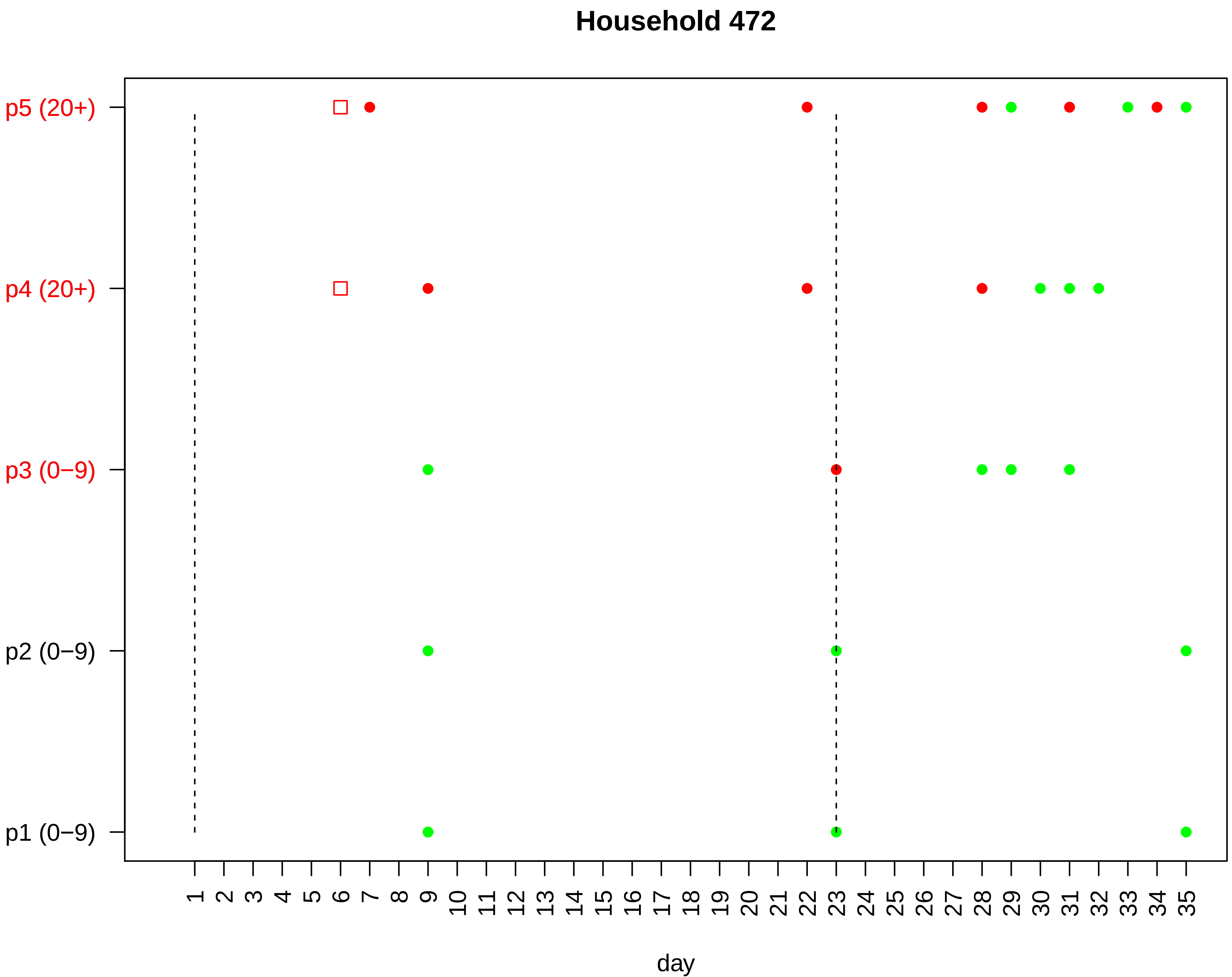

Household 472
p5 (20+)
p4 (20+)
p3 (0−9)
p2 (0−9)
p1 (0−9)
1 2 3 4 5 6 7 8 9 10 11 12 13 14 15 16 17 18 19 20 21 22 23 24 25 26 27 28 29 30 31 32 33 34 35
day

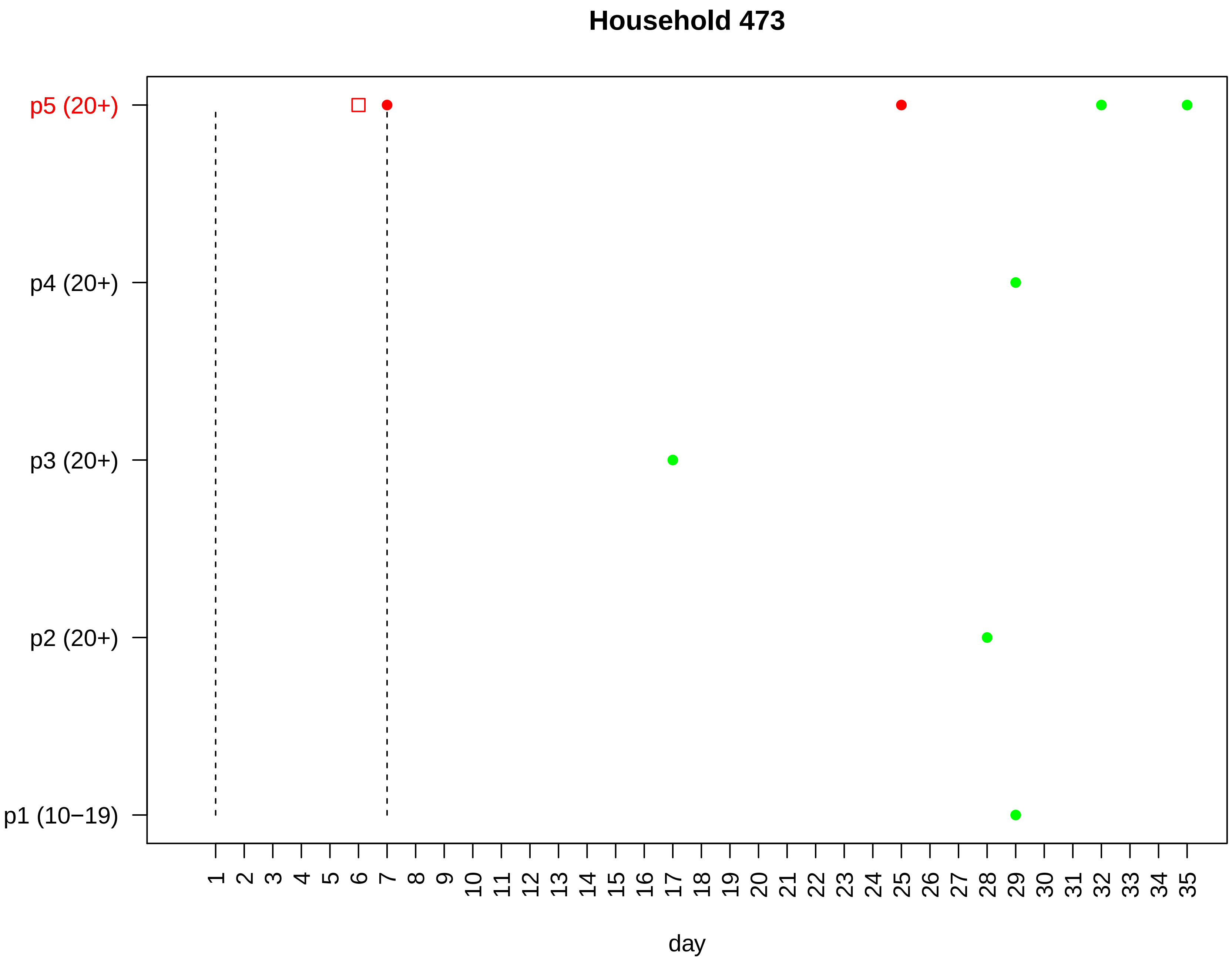

Household 473
p5 (20+)
p4 (20+)
p3 (20+)
p2 (20+)
p1 (10−19)
1 2 3 4 5 6 7 8 9 10 11 12 13 14 15 16 17 18 19 20 21 22 23 24 25 26 27 28 29 30 31 32 33 34 35
day

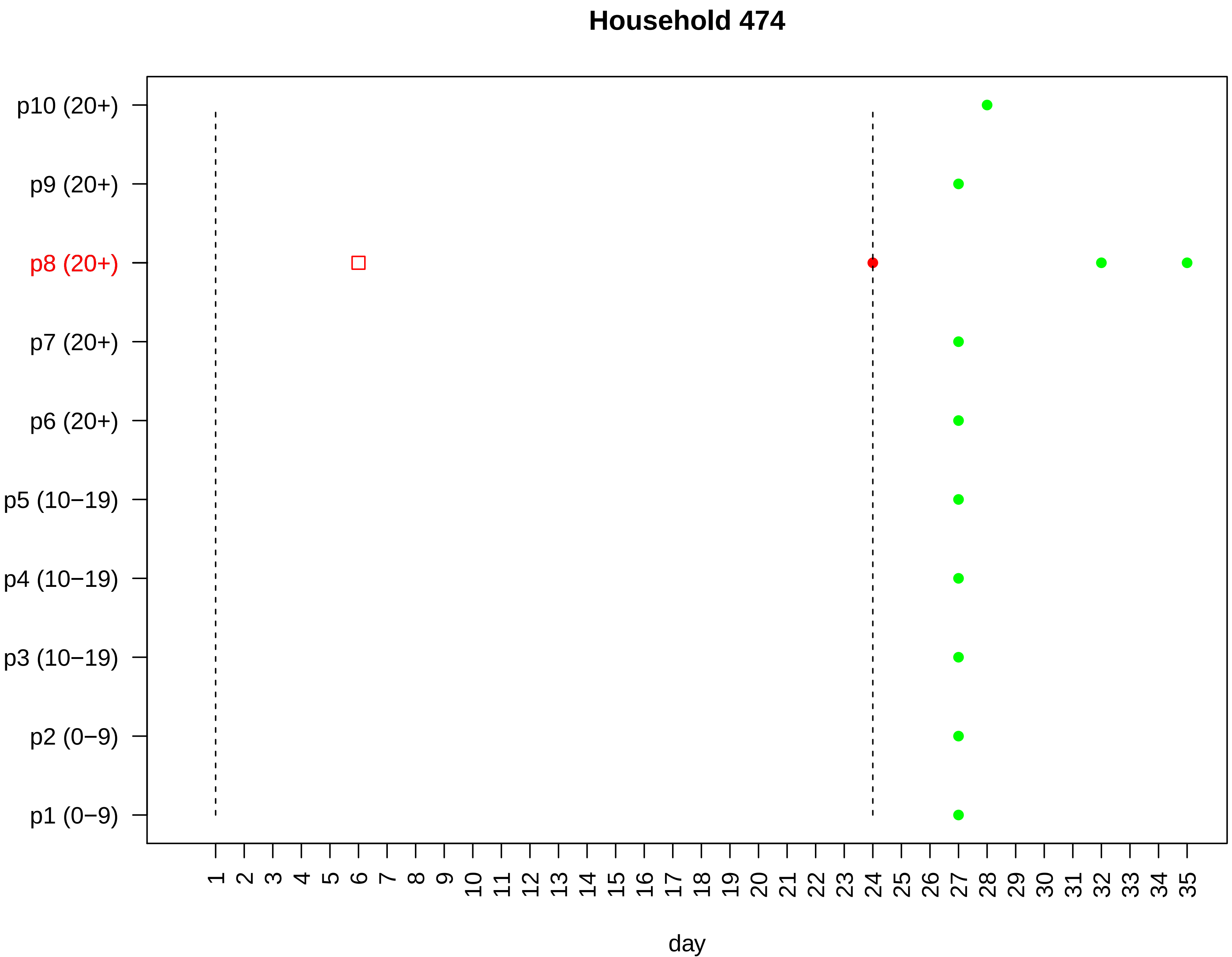

Household 474
p10 (20+)
p9 (20+)
p8 (20+)
p7 (20+)
p6 (20+)
p5 (10−19)
p4 (10−19)
p3 (10−19)
p2 (0−9)
p1 (0−9)
1 2 3 4 5 6 7 8 9 10 11 12 13 14 15 16 17 18 19 20 21 22 23 24 25 26 27 28 29 30 31 32 33 34 35
day

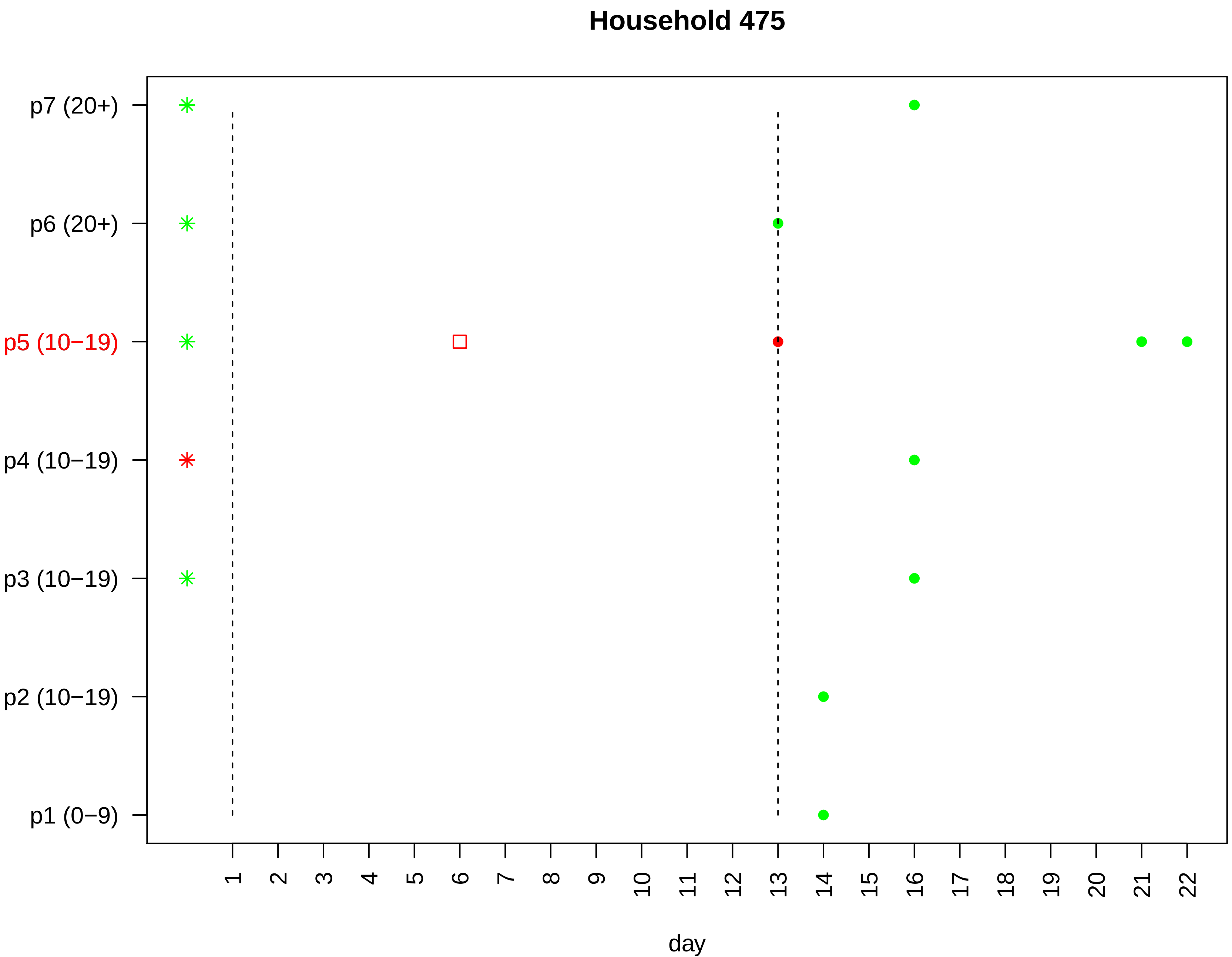
Household 475
p7 (20+)
p6 (20+)
p5 (10−19)
p4 (10−19)
p3 (10−19)
p2 (10−19)
p1 (0−9)
1
2
3
4
5
6
7
8
9
10
11
12
13
14
15
16
17
18
19
20
21
22
day

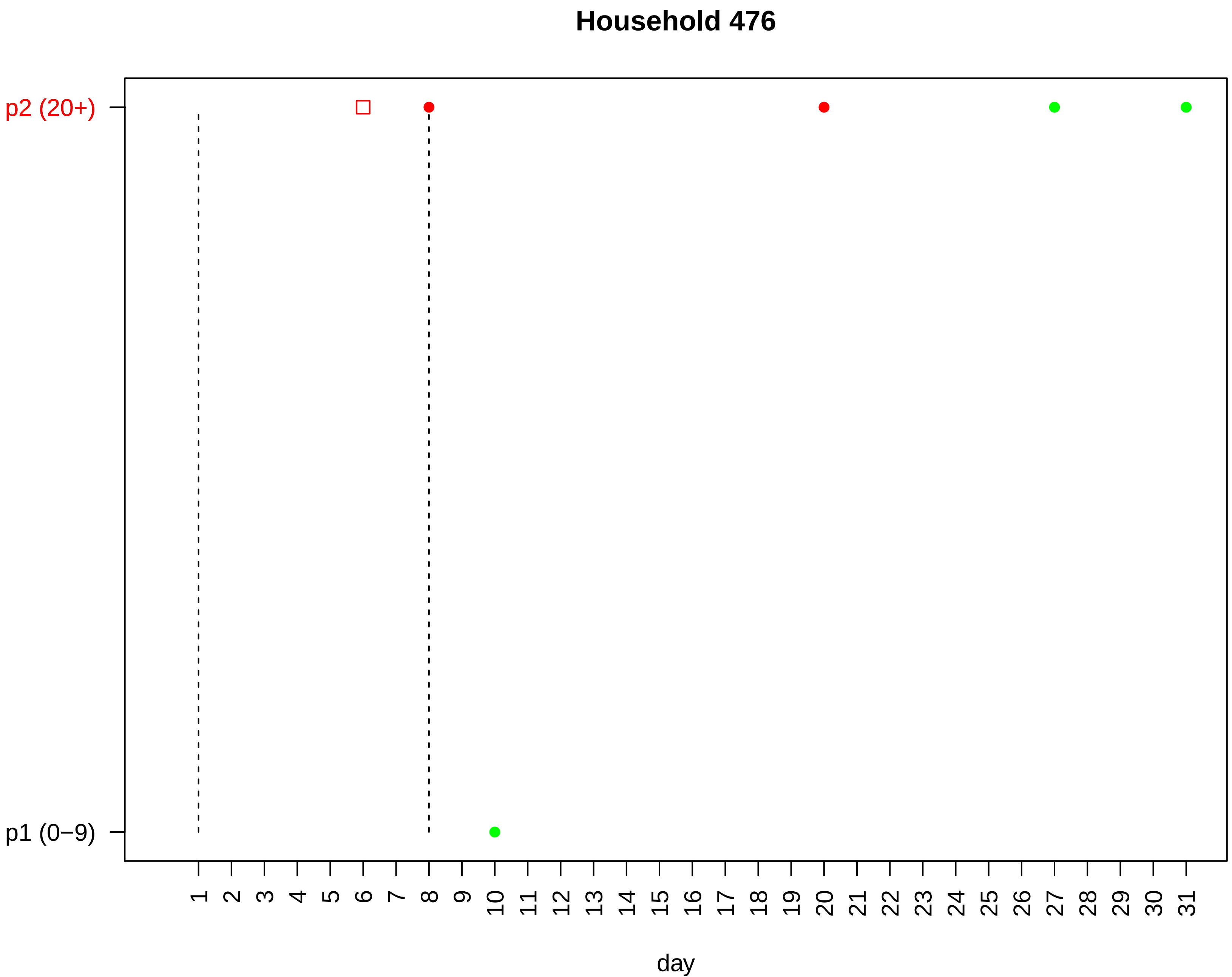

Household 476
p2 (20+)
p1 (0−9)
1
2
3
4
5
6
7
8
9
10
11
12
13
14
15
16
17
18
19
20
21
22
23
24
25
26
27
28
29
30
31
day

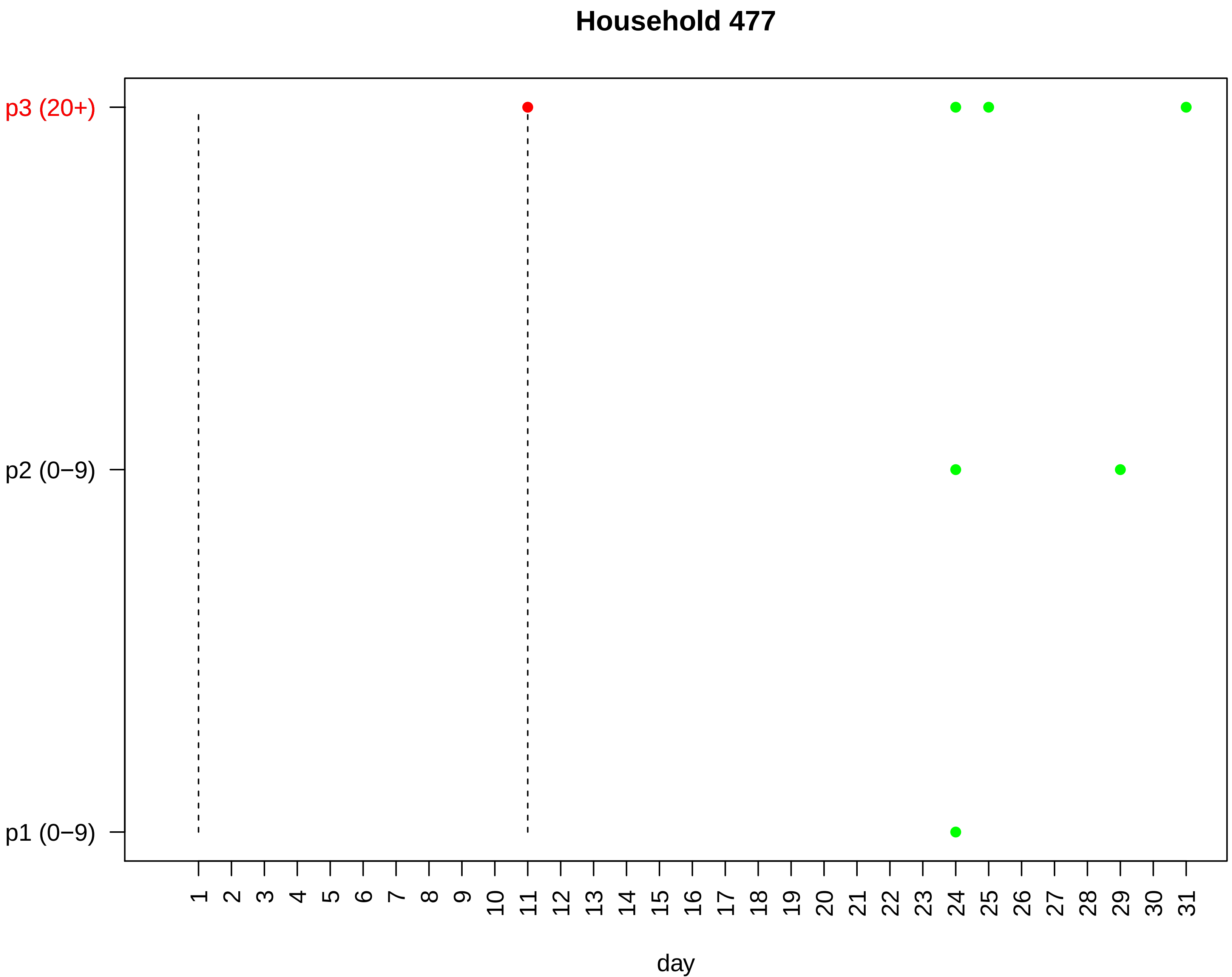

Household 477
p3 (20+)
p2 (0−9)
p1 (0−9)
1 2 3 4 5 6 7 8 9 10 11 12 13 14 15 16 17 18 19 20 21 22 23 24 25 26 27 28 29 30 31
day

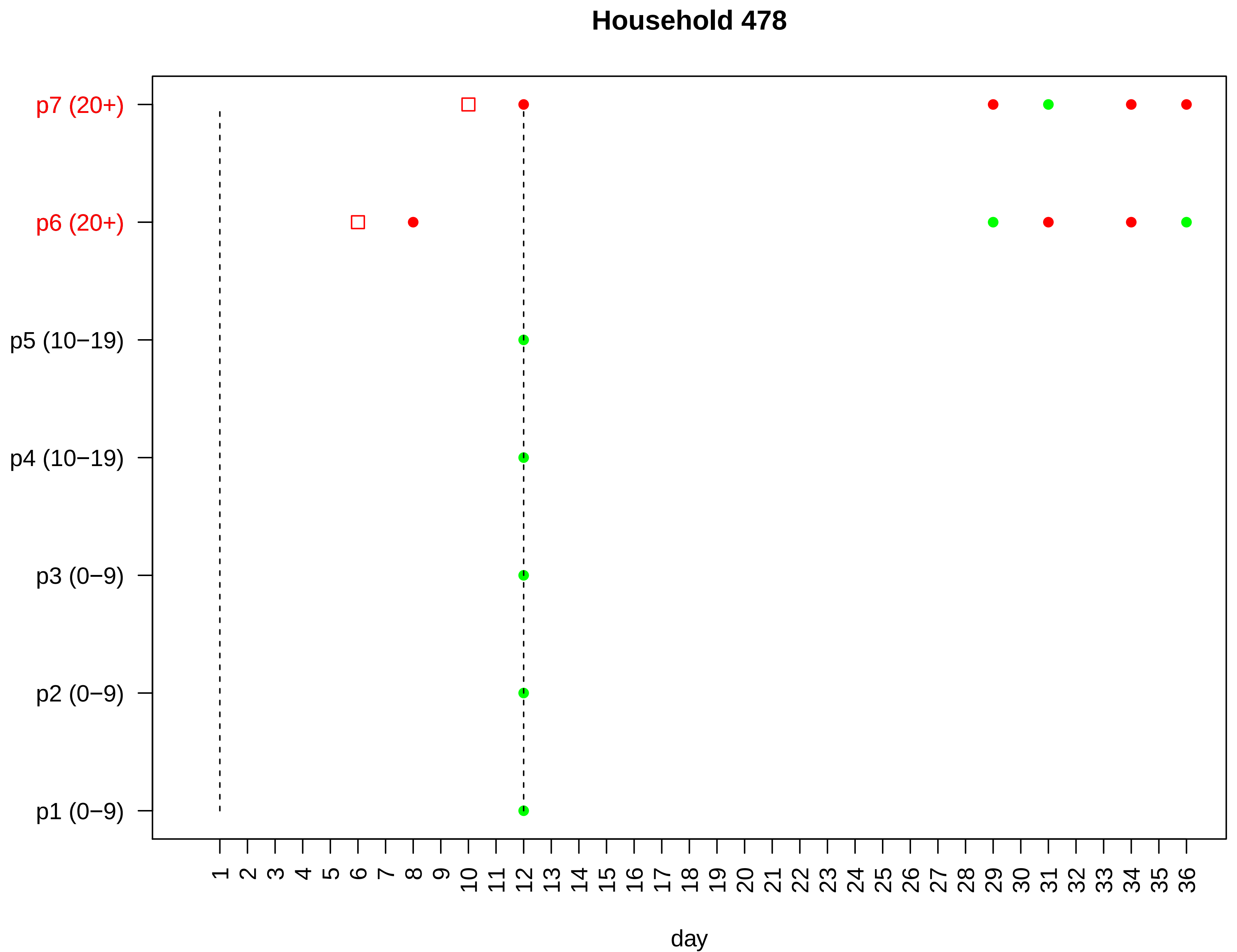

Household 478
p7 (20+)
p6 (20+)
p5 (10−19)
p4 (10−19)
p3 (0−9)
p2 (0−9)
p1 (0−9)
1 2 3 4 5 6 7 8 9 10 11 12 13 14 15 16 17 18 19 20 21 22 23 24 25 26 27 28 29 30 31 32 33 34 35 36
day

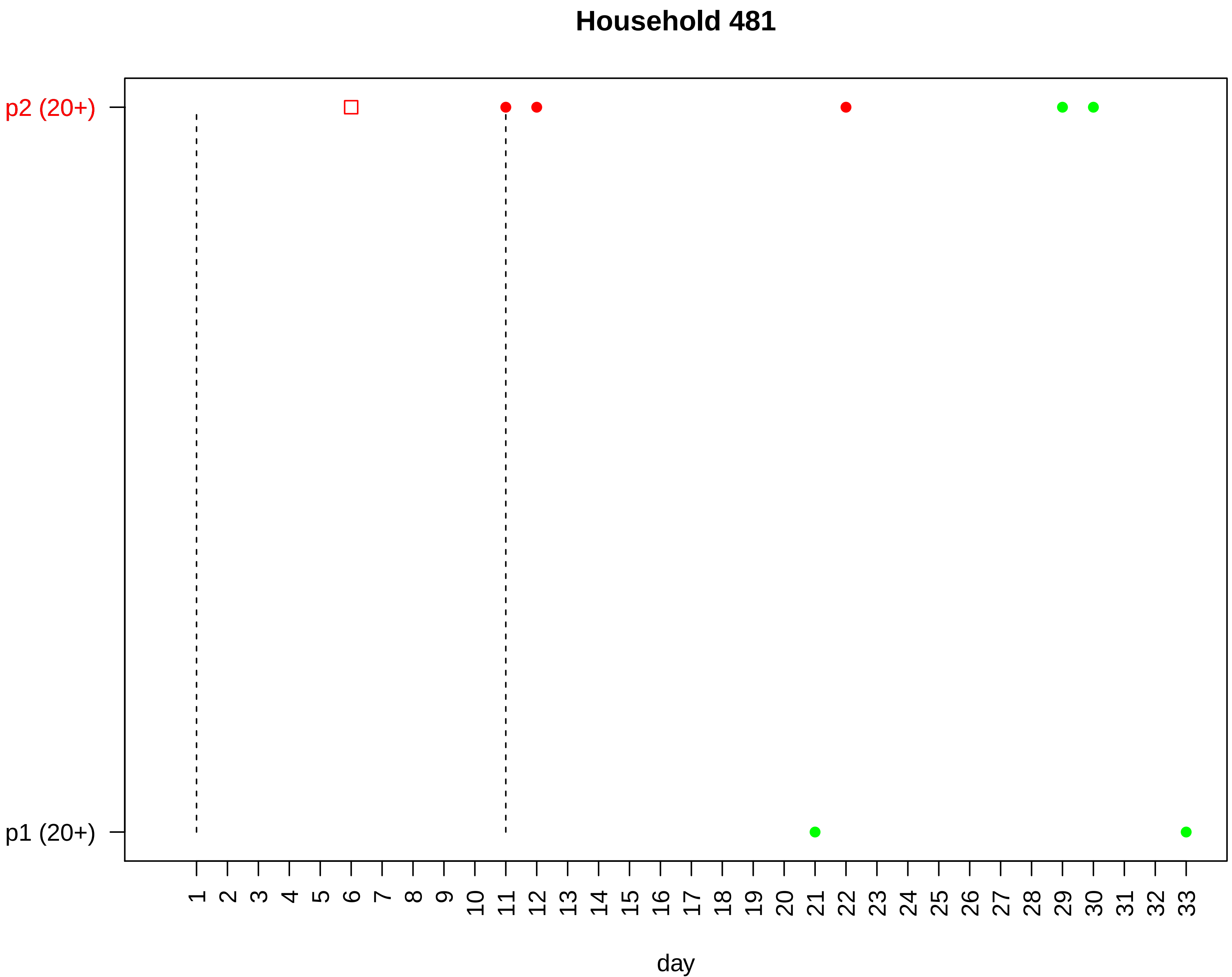

Household 481
p2 (20+)
p1 (20+)
1
2
3
4
5
6
7
8
9
10
11
12
13
14
15
16
17
18
19
20
21
22
23
24
25
26
27
28
29
30
31
32
33
day

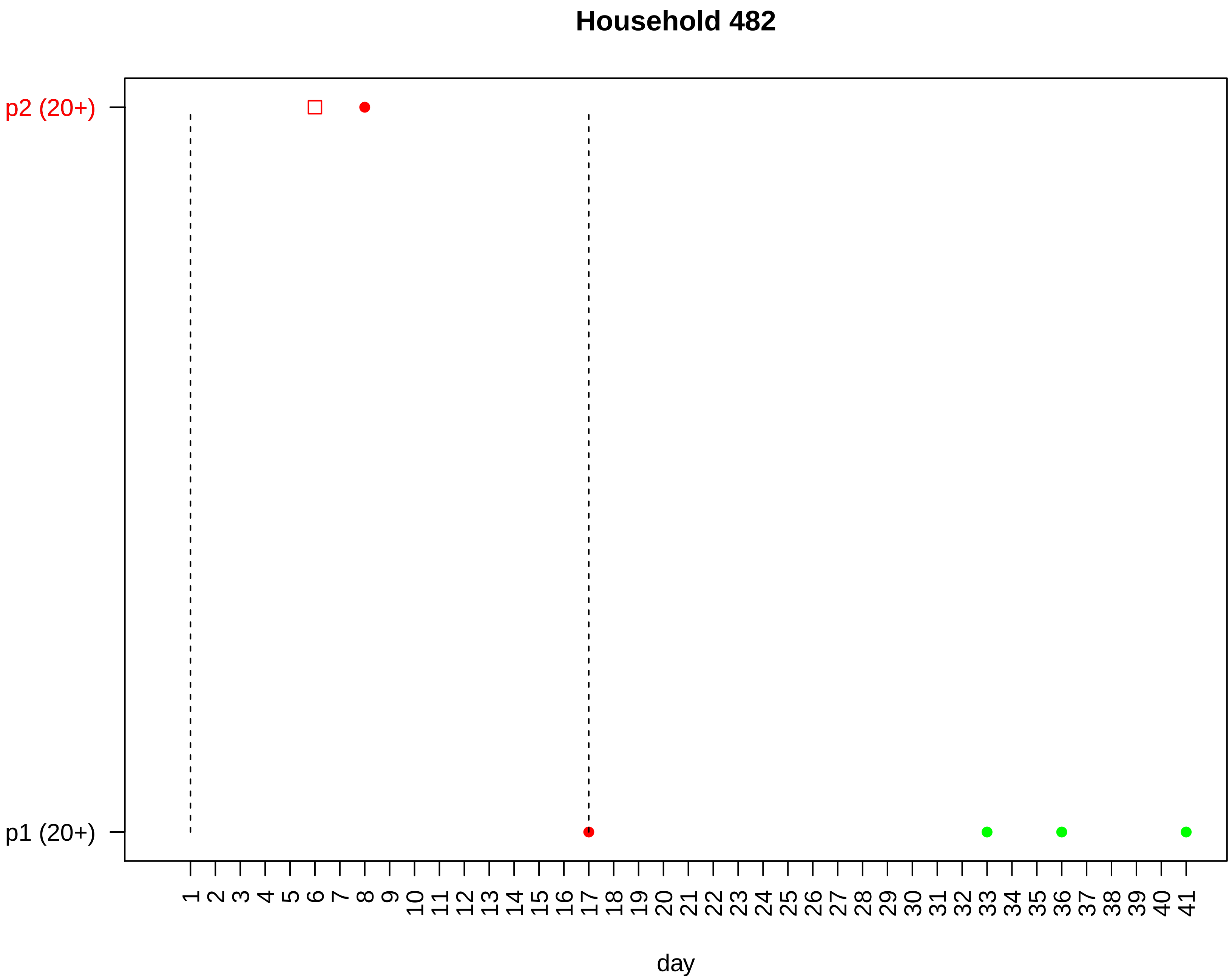
Household 482
p2 (20+)
p1 (20+)
1
2
3
4
5
6
7
8
9
10
11
12
13
14
15
16
17
18
19
20
21
22
23
24
25
26
27
28
29
30
31
32
33
34
35
36
37
38
39
40
41
day

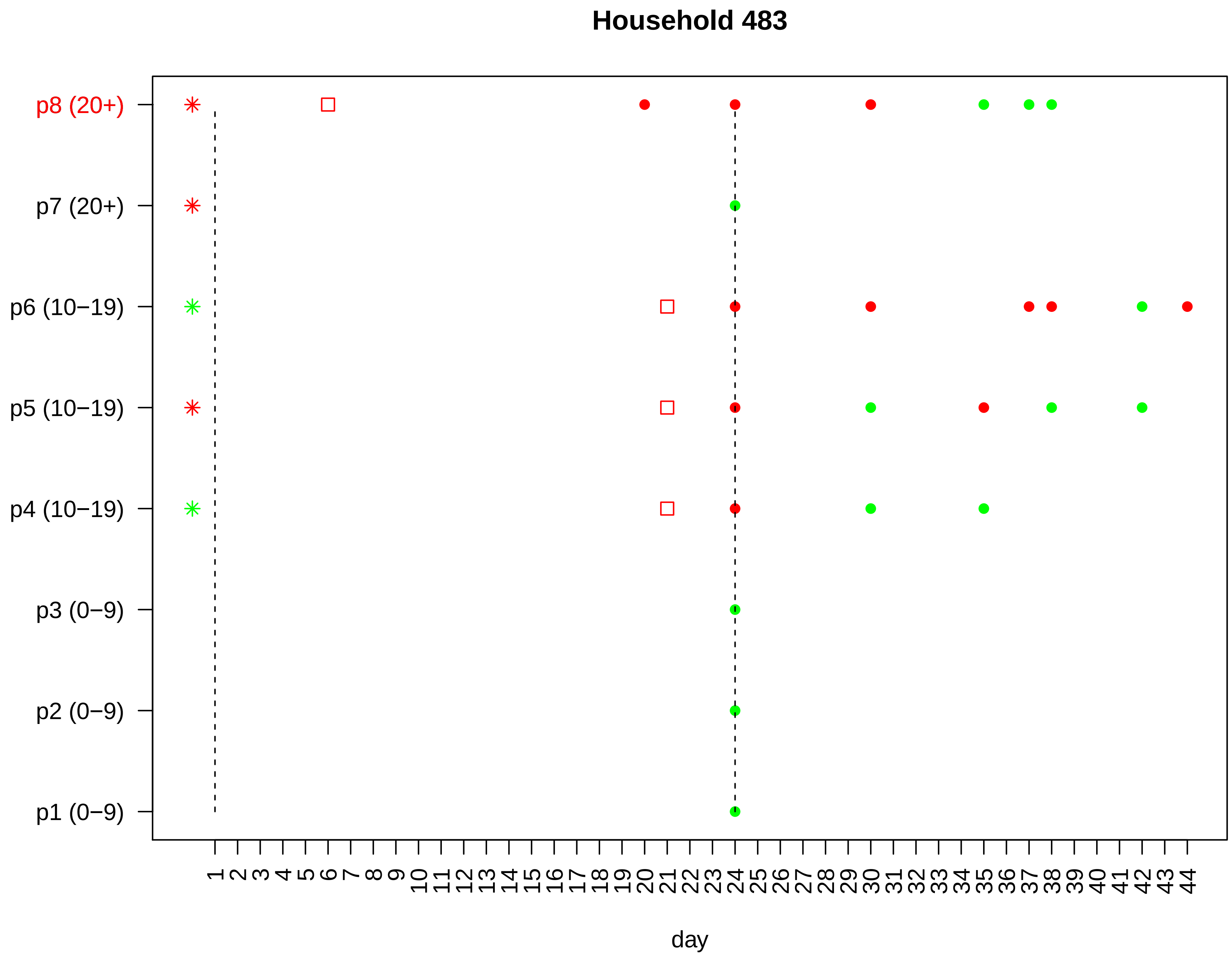

Household 483
p8 (20+)
p7 (20+)
p6 (10−19)
p5 (10−19)
p4 (10−19)
p3 (0−9)
p2 (0−9)
p1 (0−9)
1 2 3 4 5 6 7 8 9 10 11 12 13 14 15 16 17 18 19 20 21 22 23 24 25 26 27 28 29 30 31 32 33 34 35 36 37 38 39 40 41 42 43 44
day

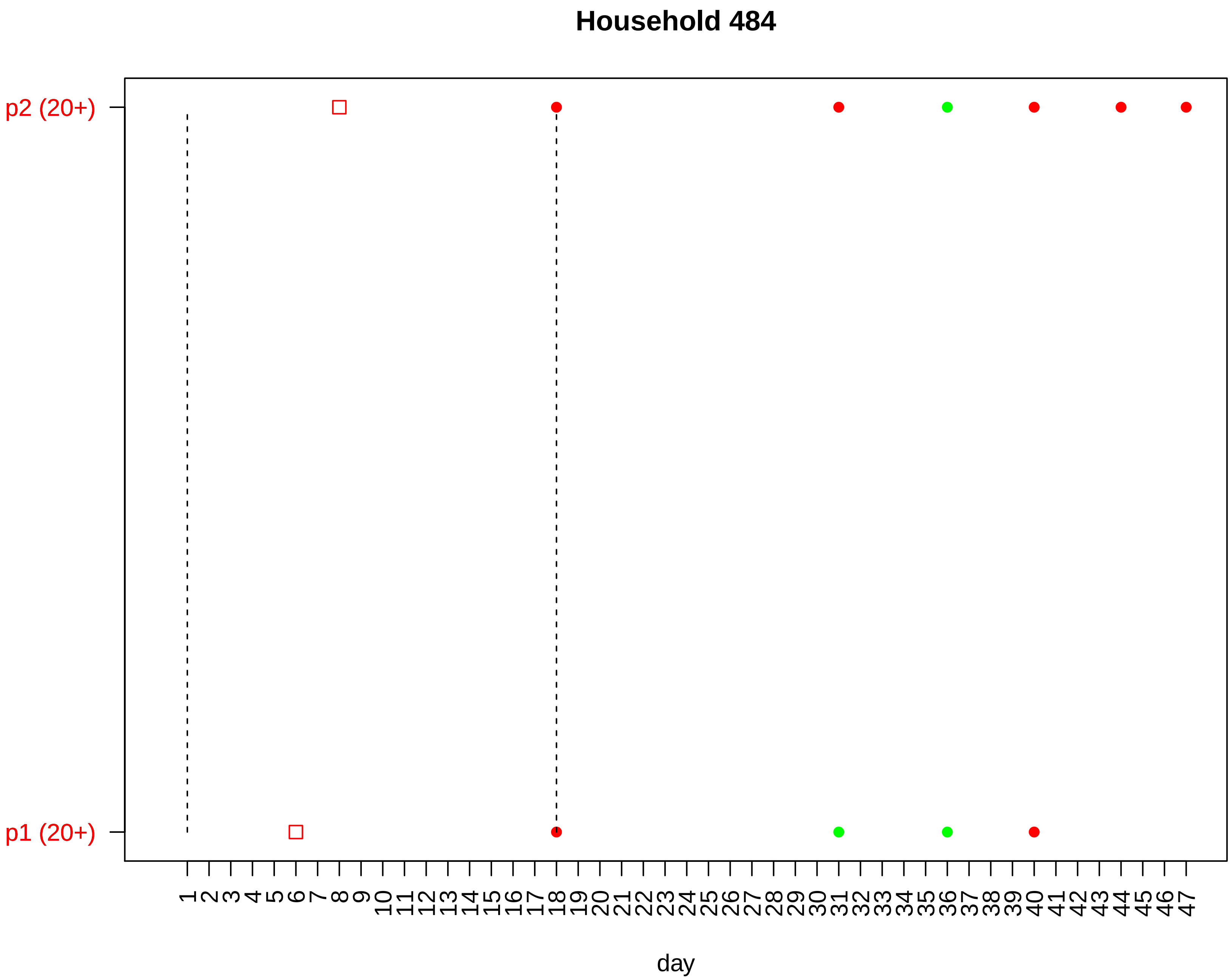

Household 484
p2 (20+)
p1 (20+)
1
2
3
4
5
6
7
8
9
10
11
12
13
14
15
16
17
18
19
20
21
22
23
24
25
26
27
28
29
30
31
32
33
34
35
36
37
38
39
40
41
42
43
44
45
46
47
day

Household 485

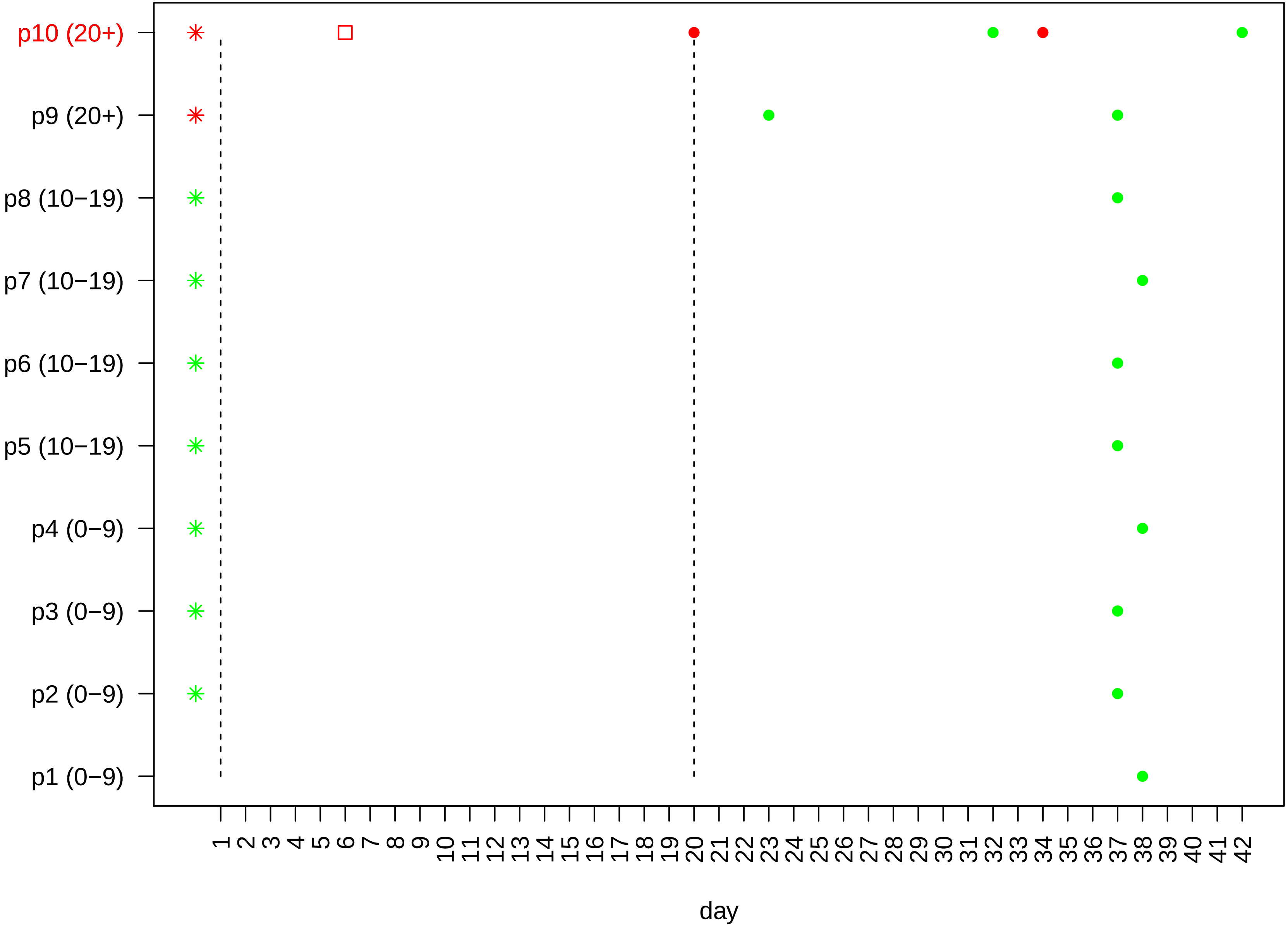

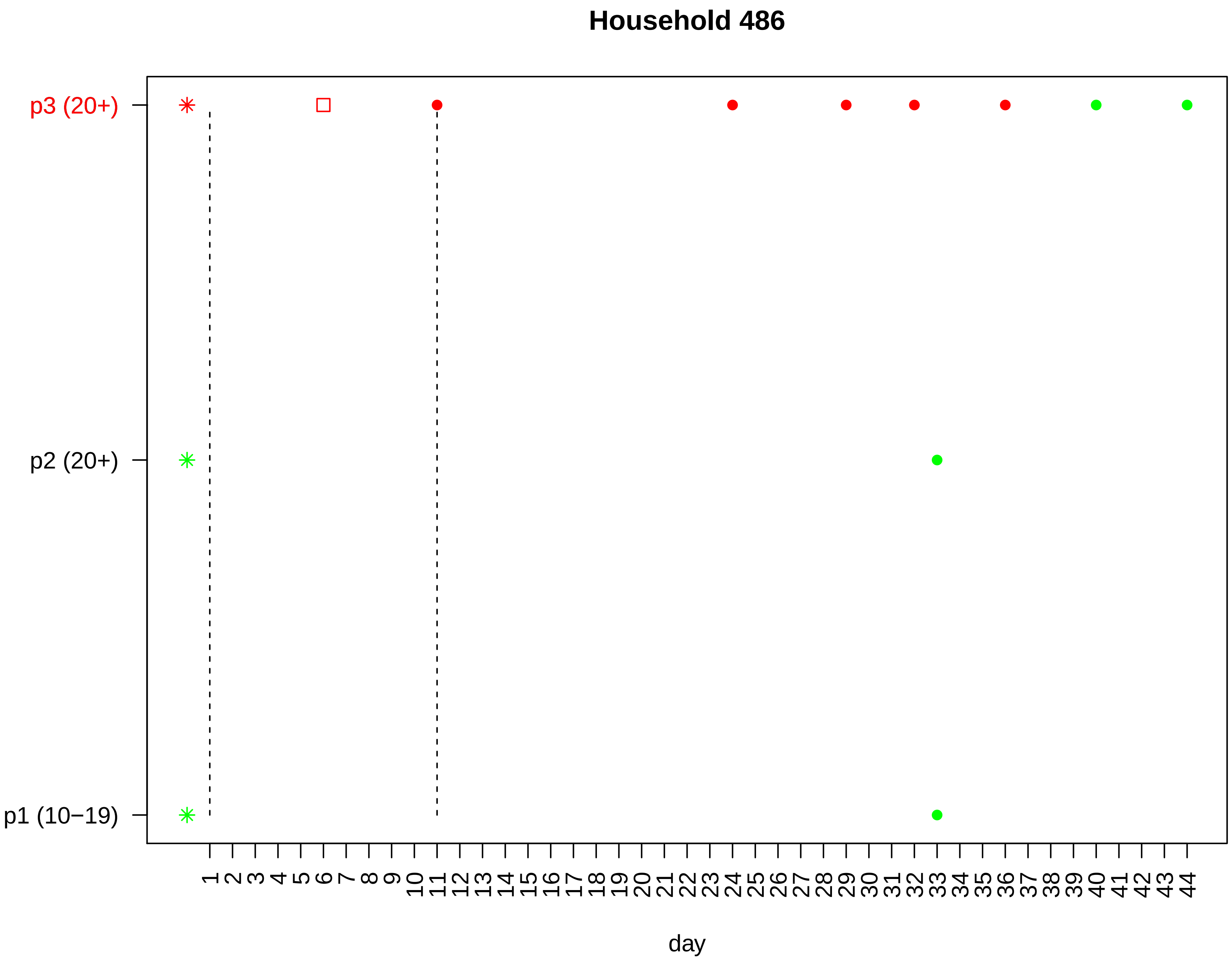

Household 486
p3 (20+)
p2 (20+)
p1 (10−19)
1 2 3 4 5 6 7 8 9 10 11 12 13 14 15 16 17 18 19 20 21 22 23 24 25 26 27 28 29 30 31 32 33 34 35 36 37 38 39 40 41 42 43 44
day

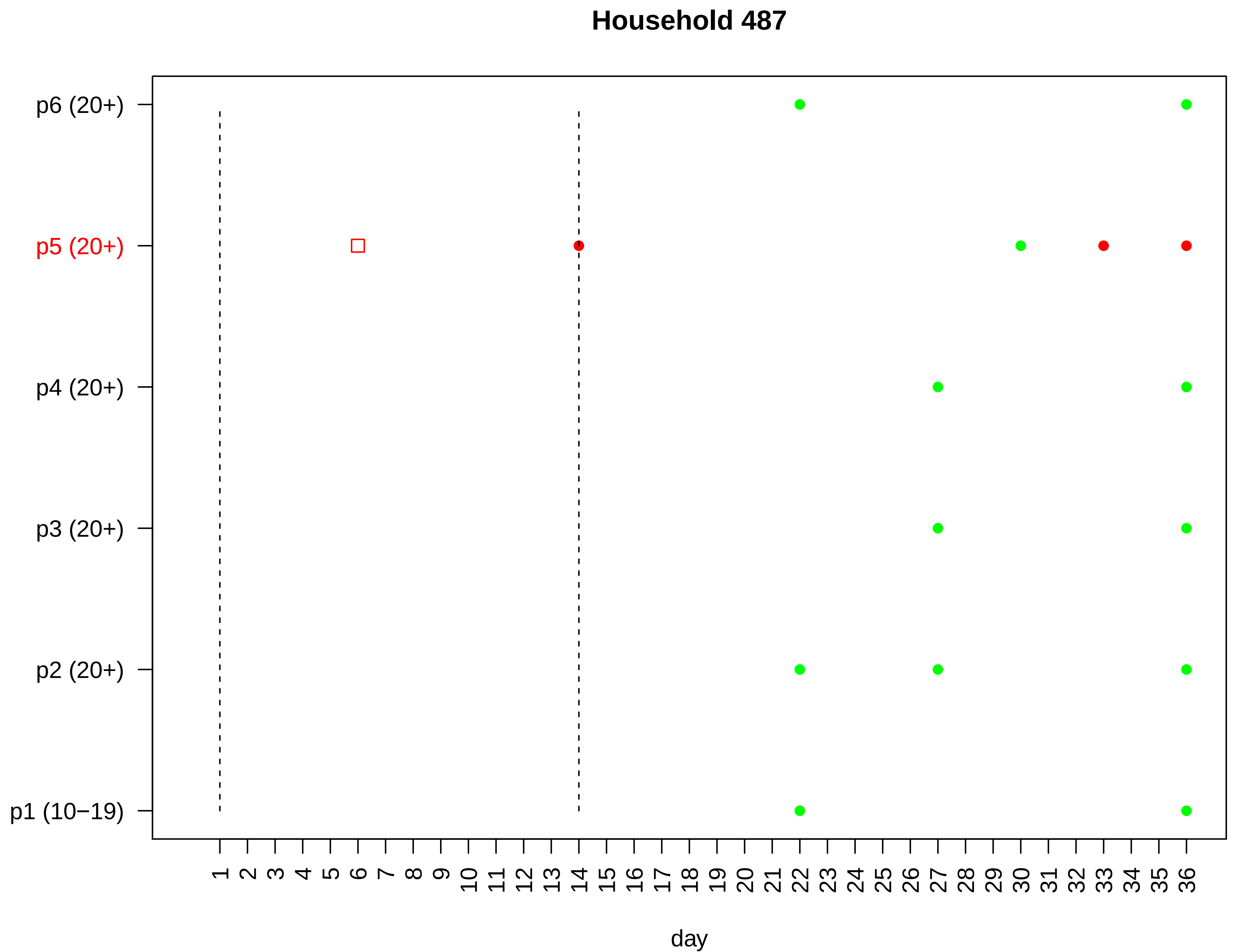

Household 487
p6 (20+)
p5 (20+)
p4 (20+)
p3 (20+)
p2 (20+)
p1 (10−19)
1 2 3 4 5 6 7 8 9 10 11 12 13 14 15 16 17 18 19 20 21 22 23 24 25 26 27 28 29 30 31 32 33 34 35 36
day

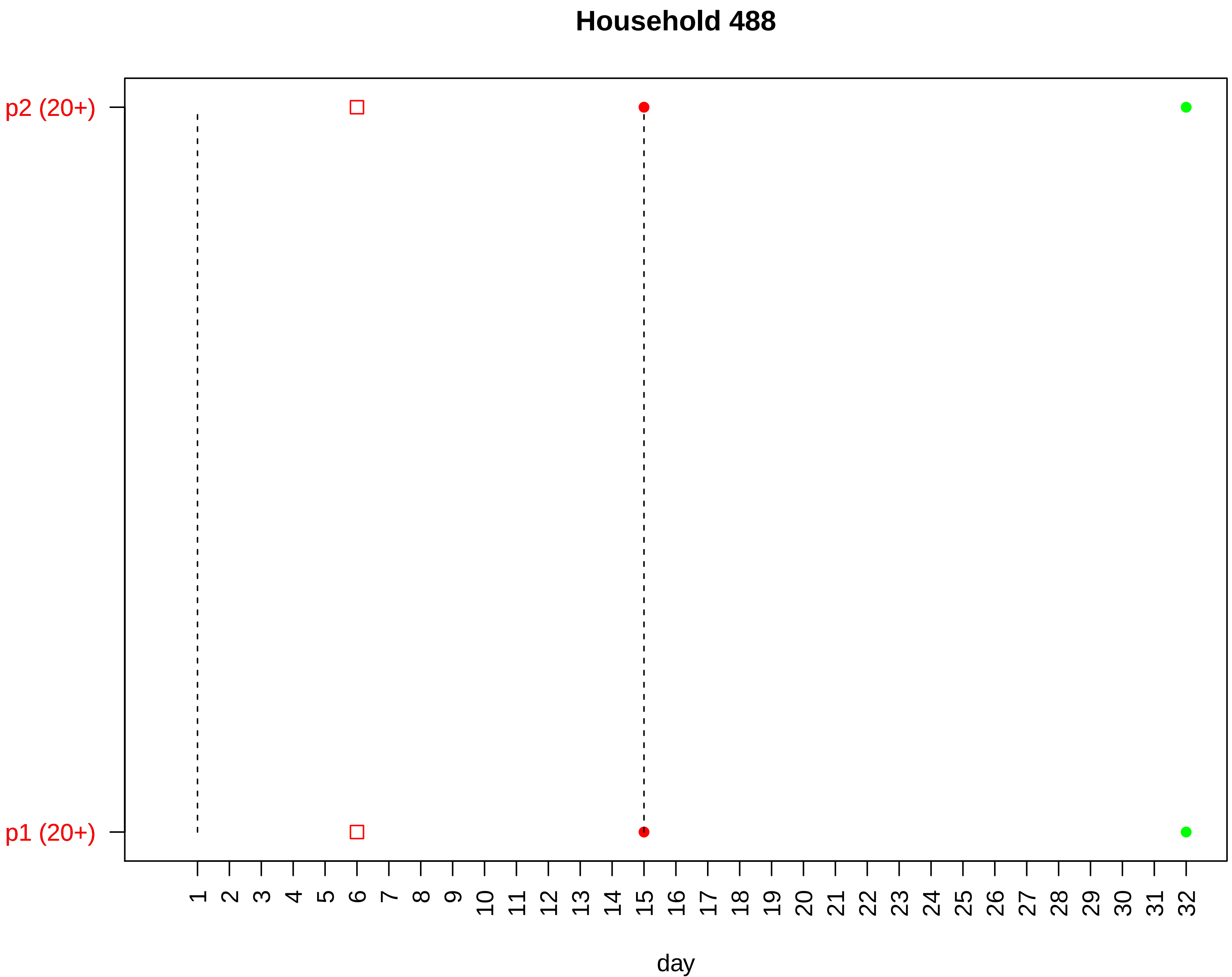
Household 488
p2 (20+)
p1 (20+)
1 2 3 4 5 6 7 8 9 10 11 12 13 14 15 16 17 18 19 20 21 22 23 24 25 26 27 28 29 30 31 32
day

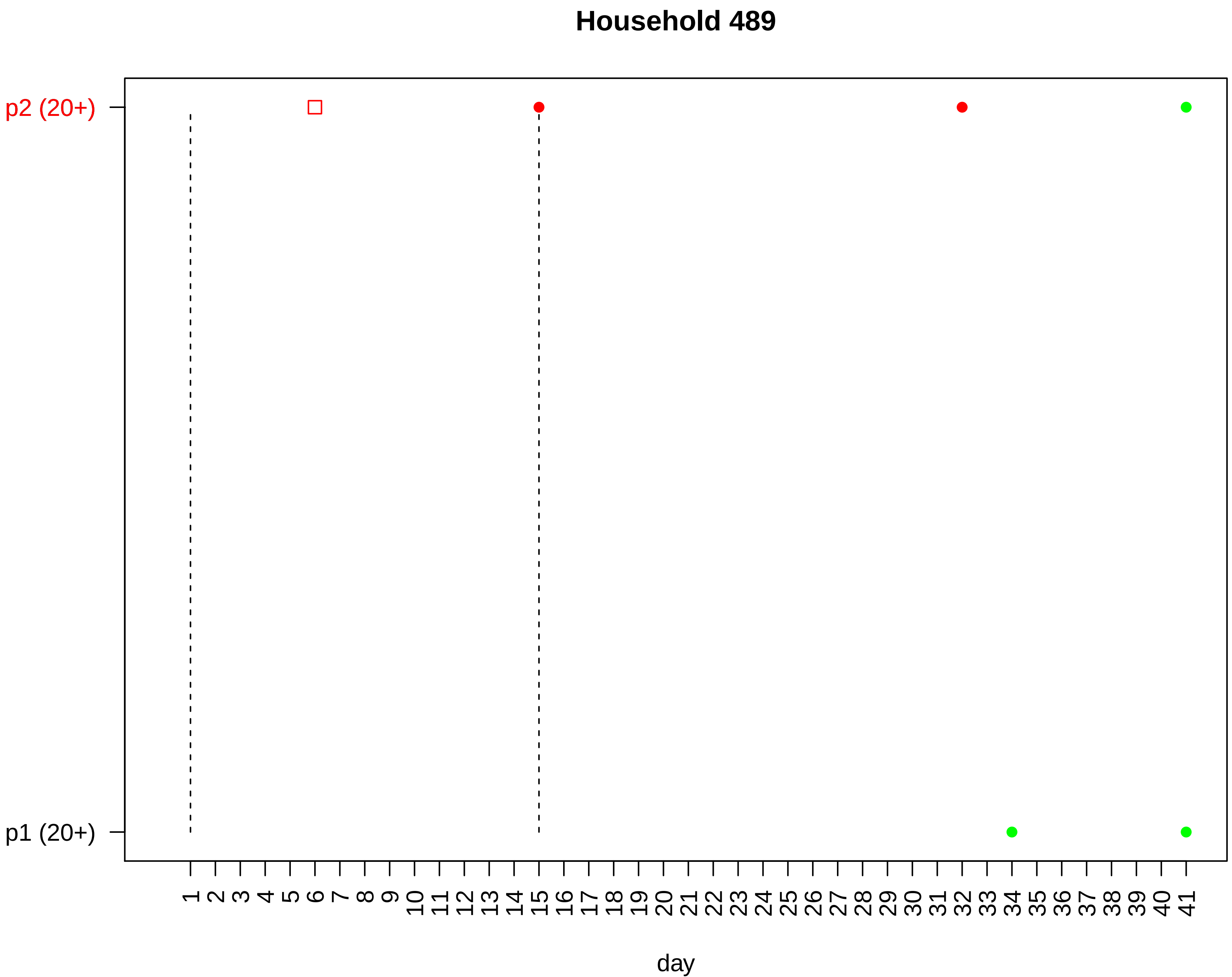

Household 489
p2 (20+)
p1 (20+)
1 2 3 4 5 6 7 8 9 10 11 12 13 14 15 16 17 18 19 20 21 22 23 24 25 26 27 28 29 30 31 32 33 34 35 36 37 38 39 40 41
day

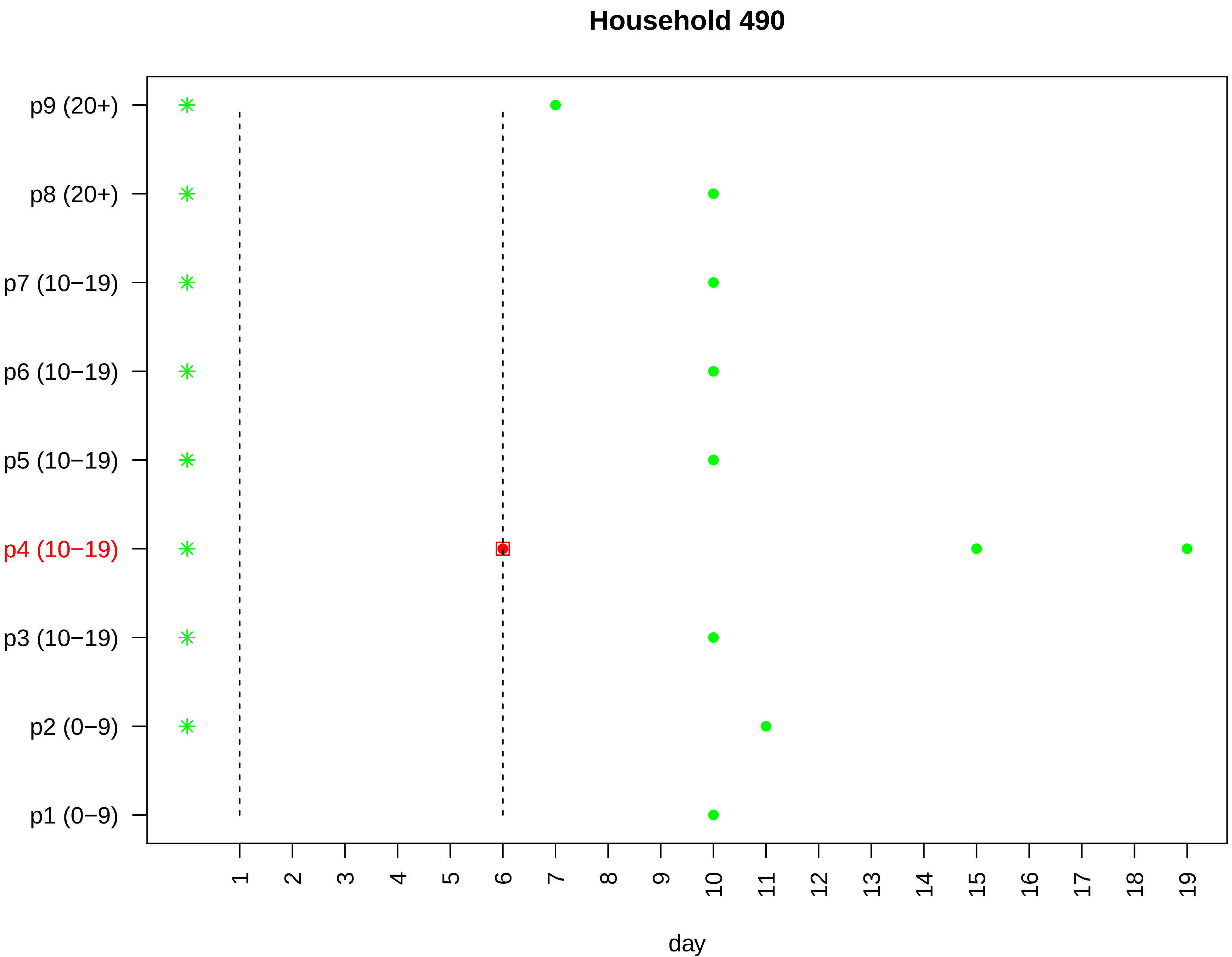

Household 490
p9 (20+)
p8 (20+)
p7 (10−19)
p6 (10−19)
p5 (10−19)
p4 (10−19)
p3 (10−19)
p2 (0−9)
p1 (0−9)
1
2
3
4
5
6
7
8
9
10
11
12
13
14
15
16
17
18
19
day

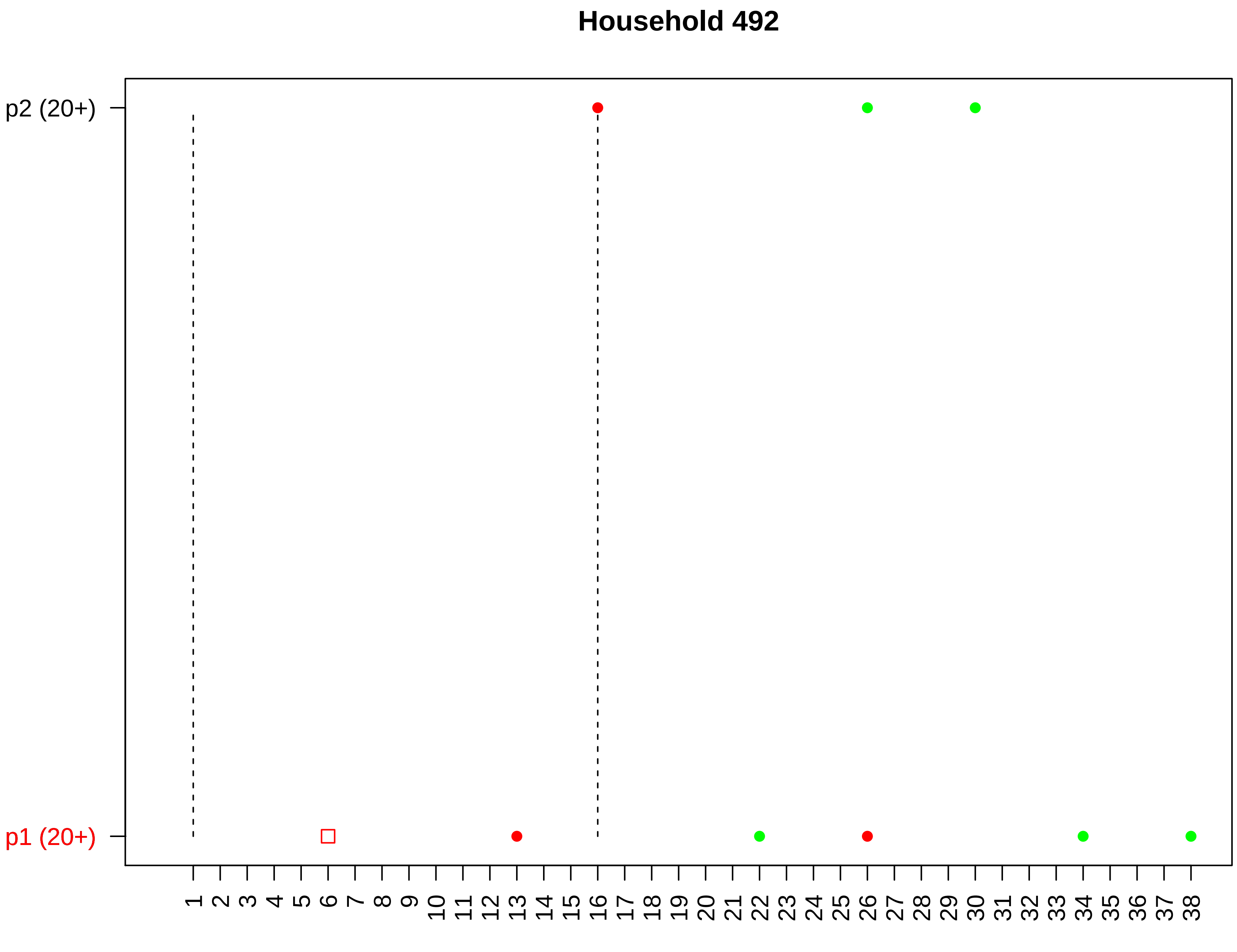

Household 492
p2 (20+)
p1 (20+)
1 2 3 4 5 6 7 8 9 10 11 12 13 14 15 16 17 18 19 20 21 22 23 24 25 26 27 28 29 30 31 32 33 34 35 36 37 38
day

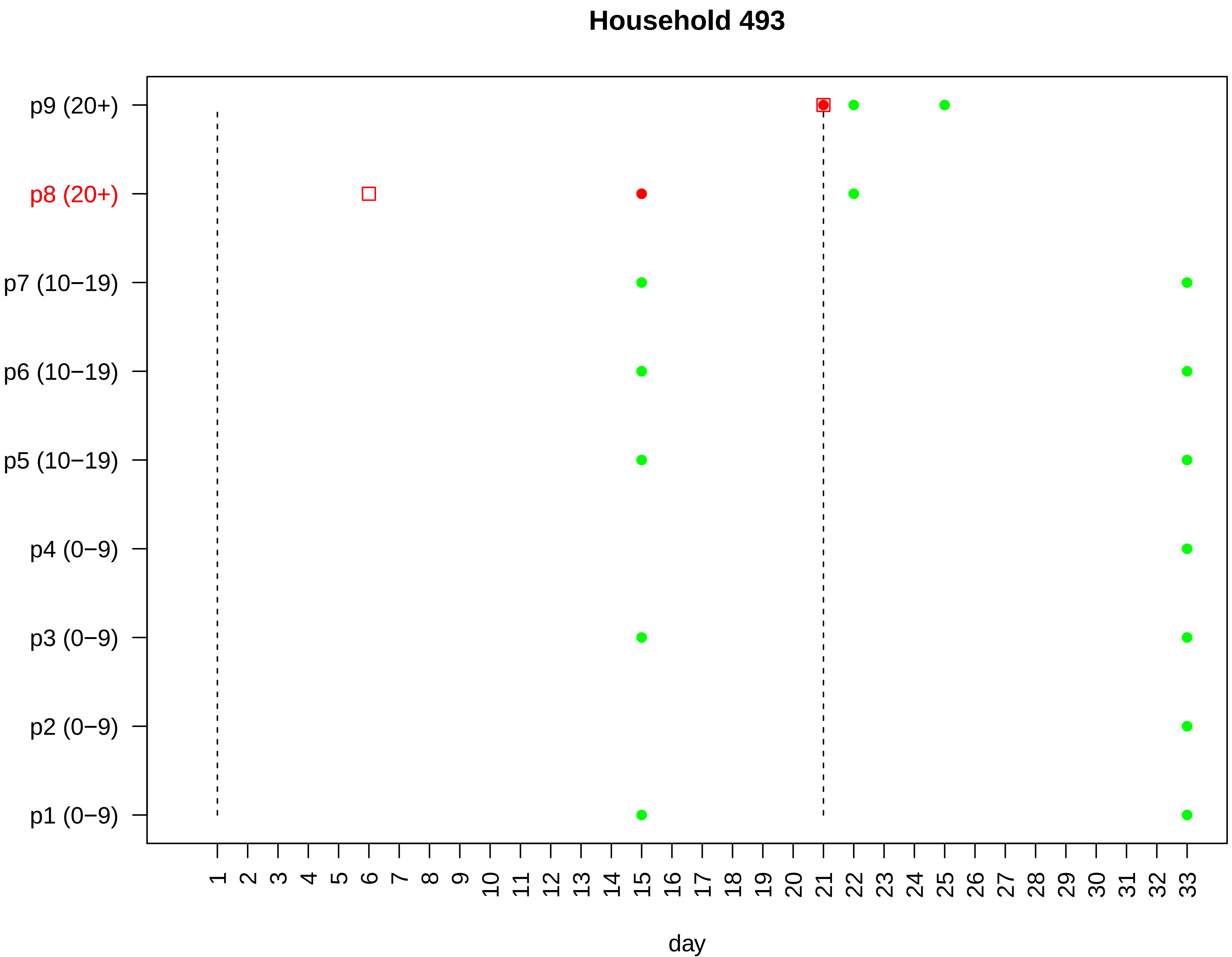
Household 493
p9 (20+)
p8 (20+)
p7 (10−19)
p6 (10−19)
p5 (10−19)
p4 (0−9)
p3 (0−9)
p2 (0−9)
p1 (0−9)
1 2 3 4 5 6 7 8 9 10 11 12 13 14 15 16 17 18 19 20 21 22 23 24 25 26 27 28 29 30 31 32 33
day

Household 494

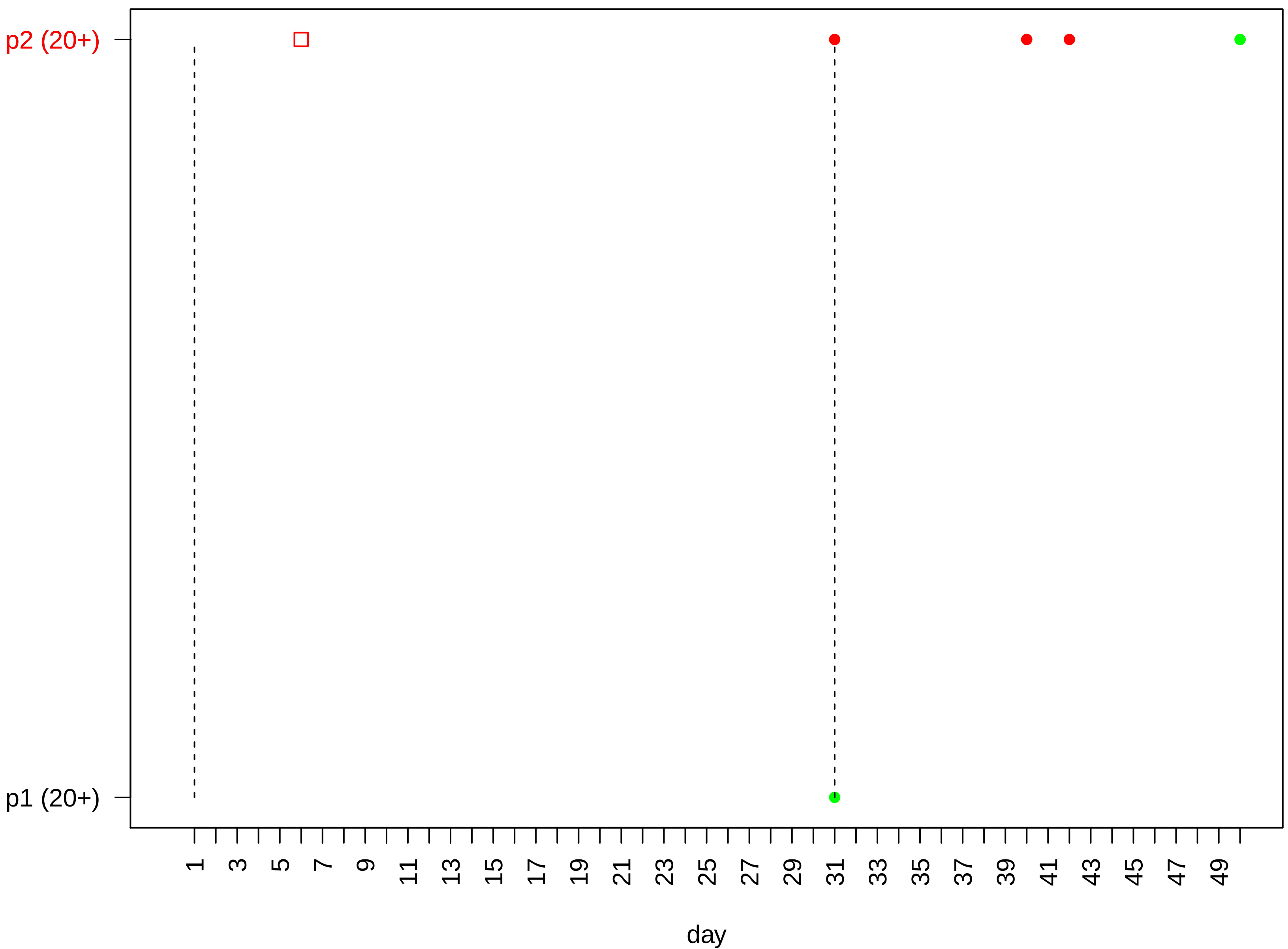

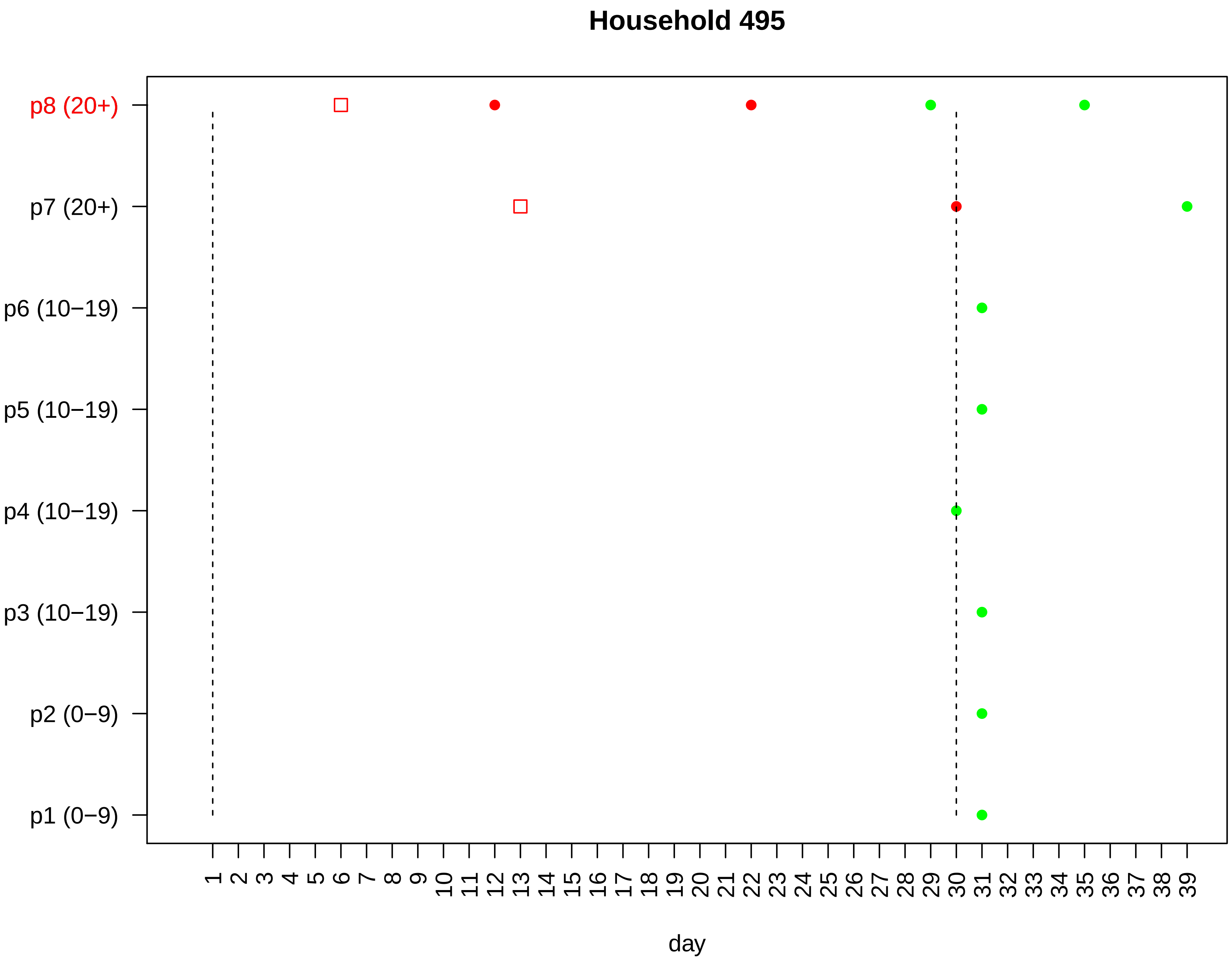

Household 495
p8 (20+)
p7 (20+)
p6 (10−19)
p5 (10−19)
p4 (10−19)
p3 (10−19)
p2 (0−9)
p1 (0−9)
1 2 3 4 5 6 7 8 9 10 11 12 13 14 15 16 17 18 19 20 21 22 23 24 25 26 27 28 29 30 31 32 33 34 35 36 37 38 39
day

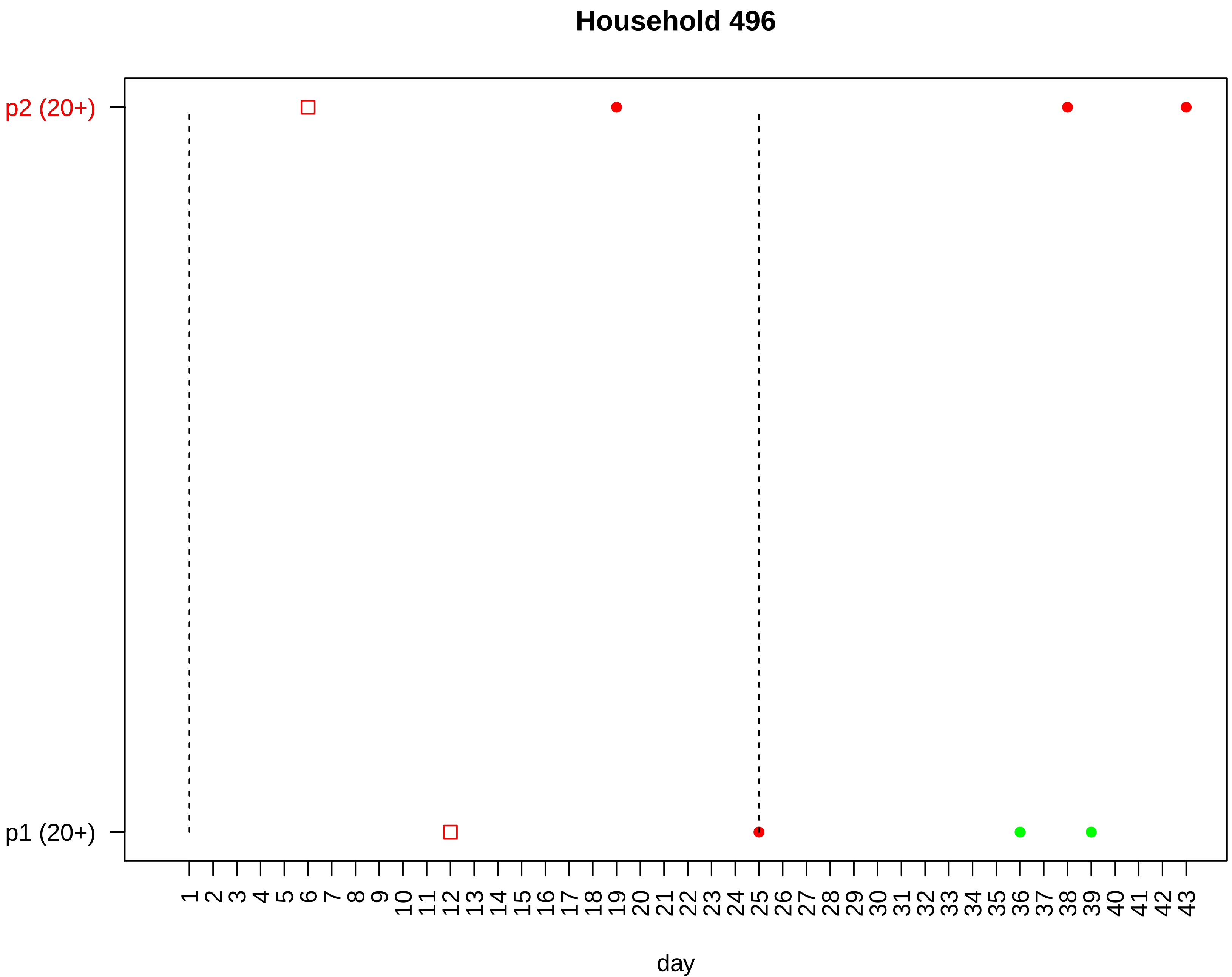

Household 496
p2 (20+)
p1 (20+)
1
2
3
4
5
6
7
8
9
10
11
12
13
14
15
16
17
18
19
20
21
22
23
24
25
26
27
28
29
30
31
32
33
34
35
36
37
38
39
40
41
42
43
day

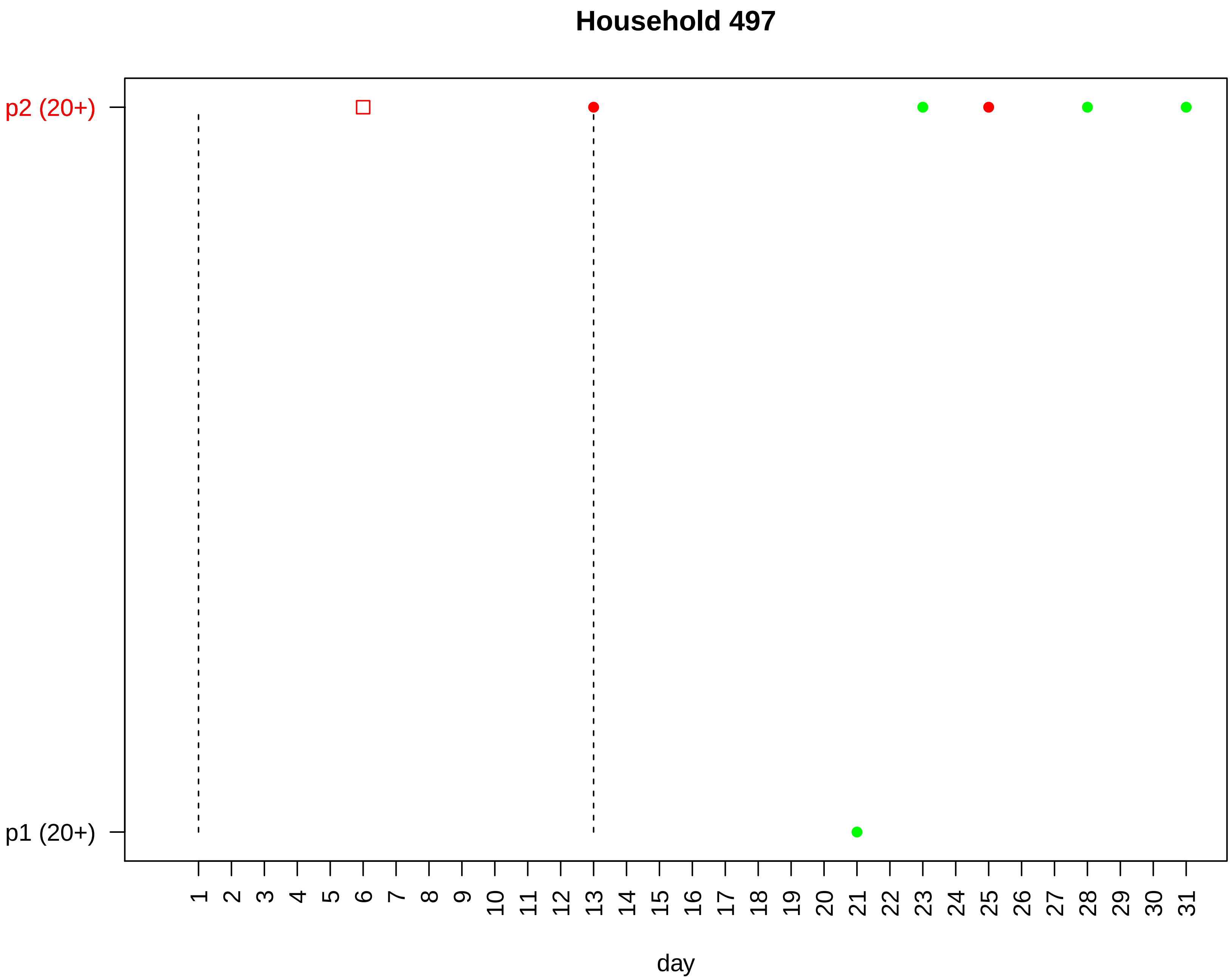

Household 497
p2 (20+)
p1 (20+)
1
2
3
4
5
6
7
8
9
10
11
12
13
14
15
16
17
18
19
20
21
22
23
24
25
26
27
28
29
30
31
day

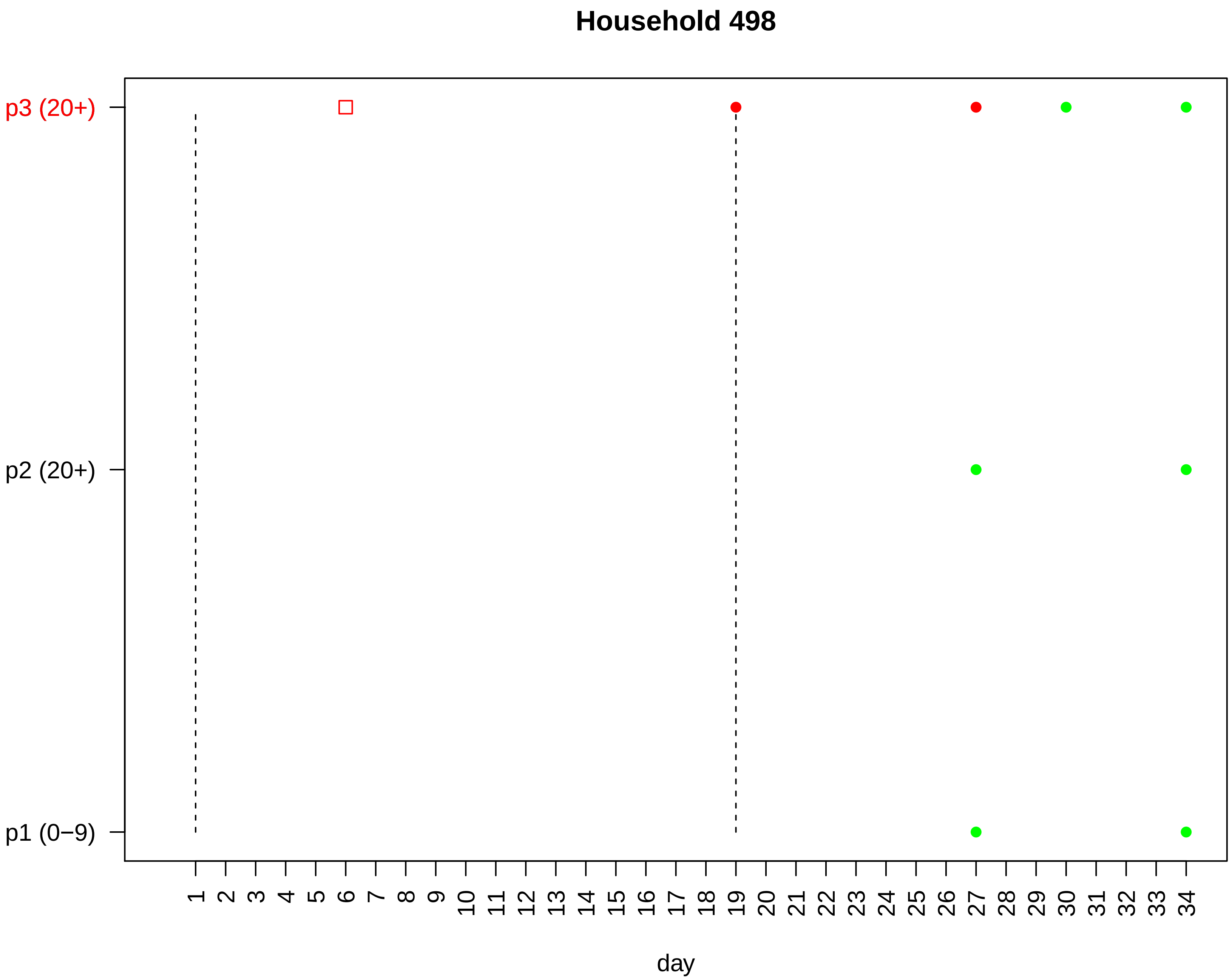
Household 498
p3 (20+)
p2 (20+)
p1 (0−9)
1 2 3 4 5 6 7 8 9 10 11 12 13 14 15 16 17 18 19 20 21 22 23 24 25 26 27 28 29 30 31 32 33 34
day

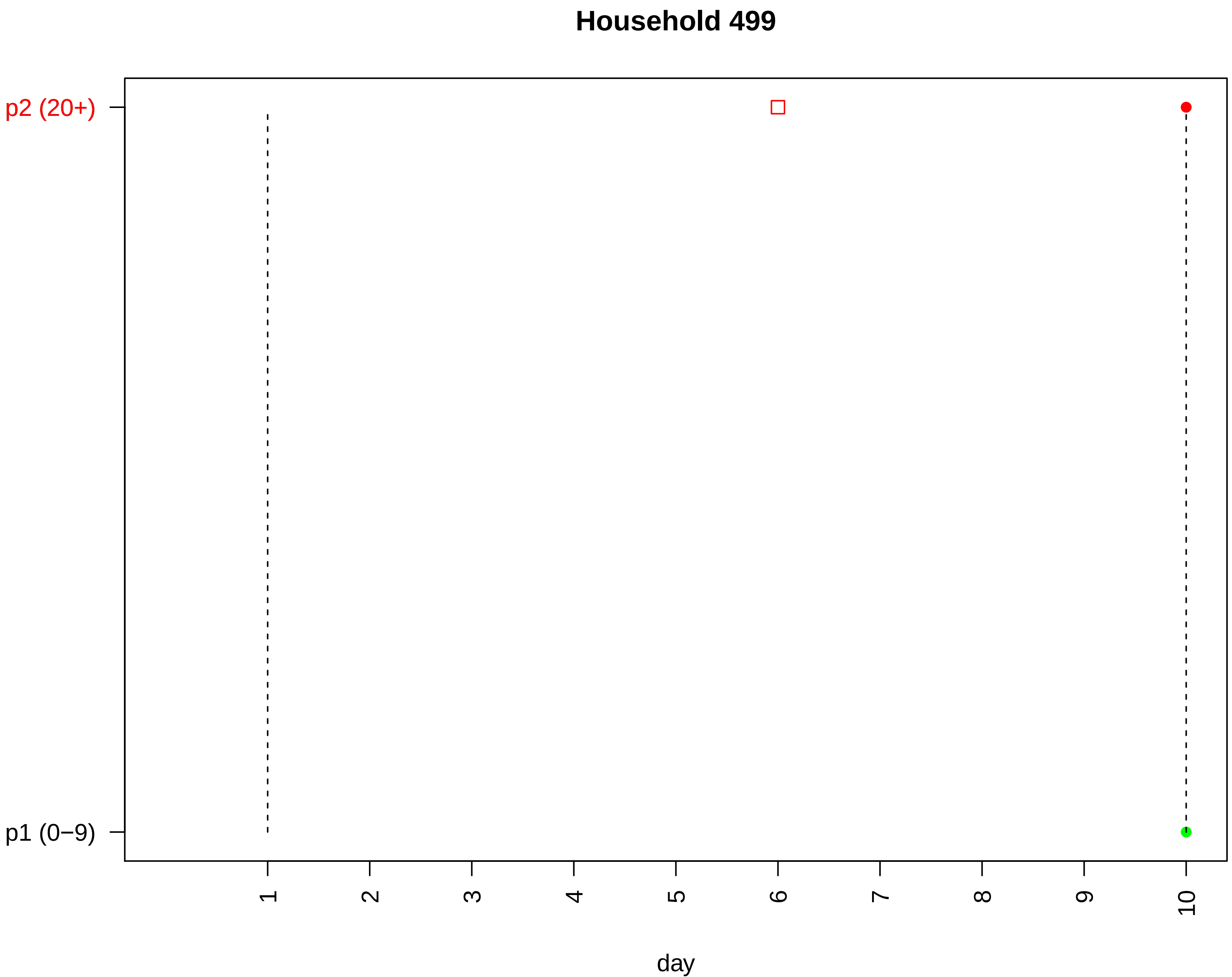
Household 499
p2 (20+)
p1 (0−9)
1
2
3
4
5
6
7
8
9
10
day

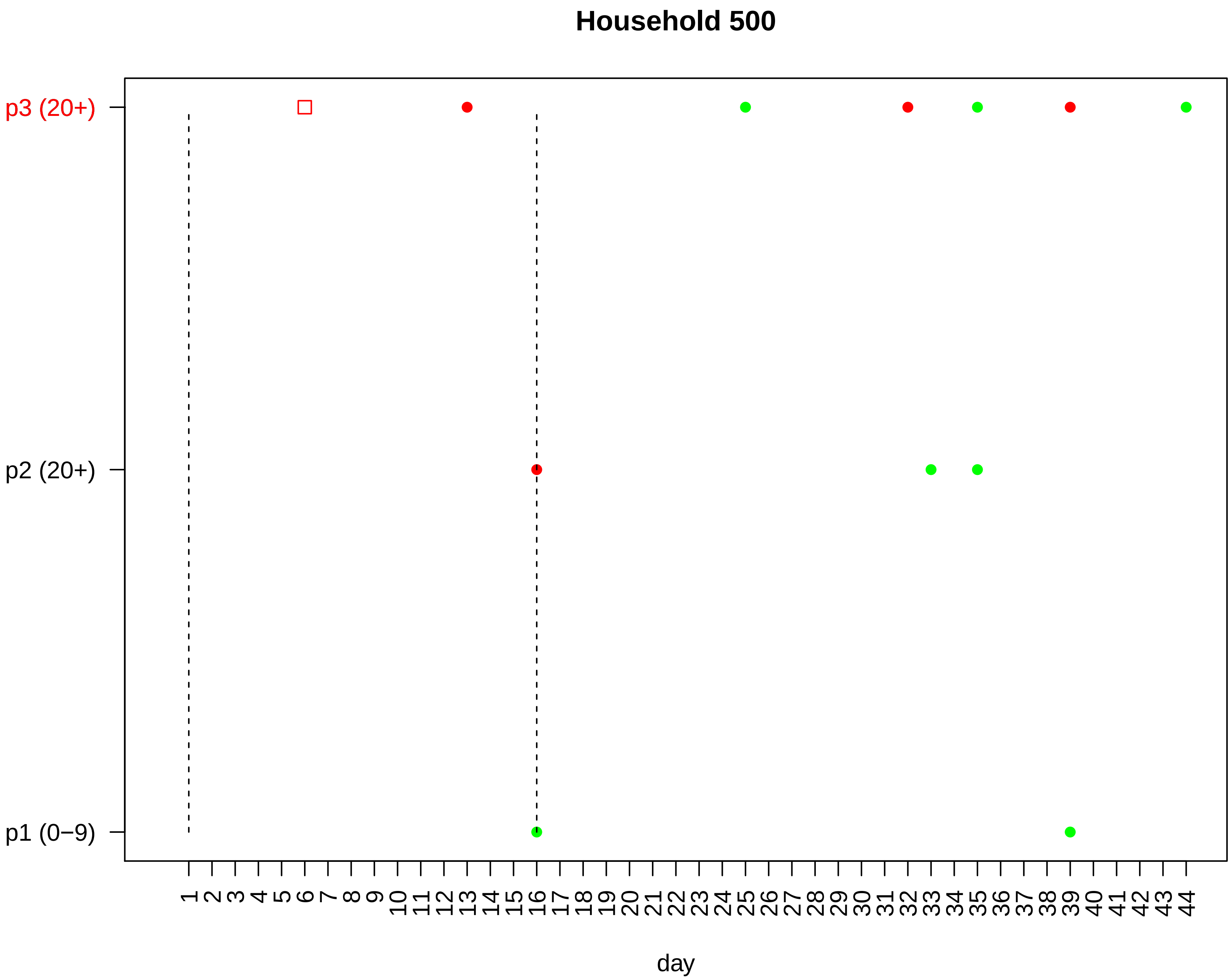

Household 500
p3 (20+)
p2 (20+)
p1 (0−9)
1 2 3 4 5 6 7 8 9 10 11 12 13 14 15 16 17 18 19 20 21 22 23 24 25 26 27 28 29 30 31 32 33 34 35 36 37 38 39 40 41 42 43 44
day

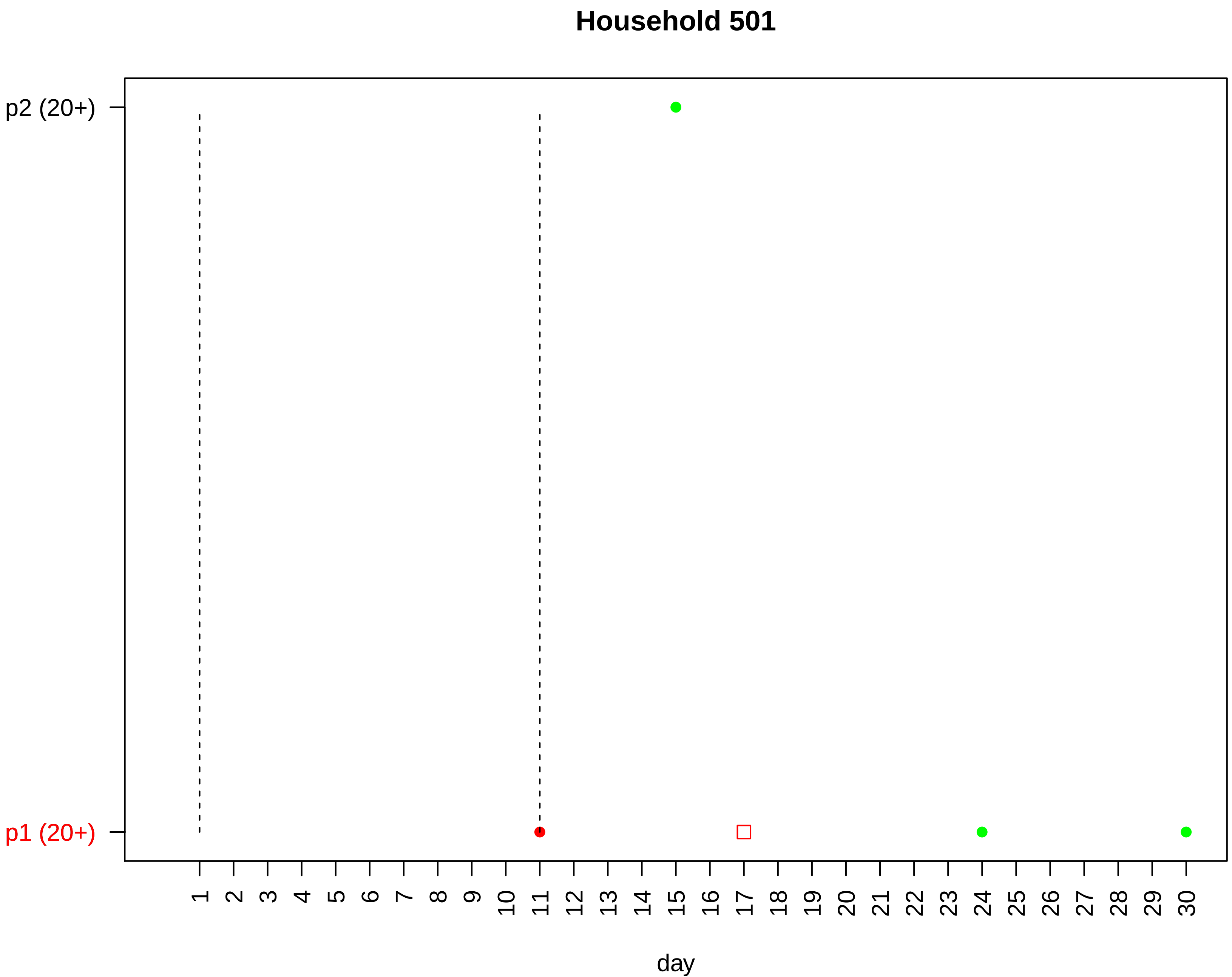

Household 501
p2 (20+)
p1 (20+)
1
2
3
4
5
6
7
8
9
10
11
12
13
14
15
16
17
18
19
20
21
22
23
24
25
26
27
28
29
30
day

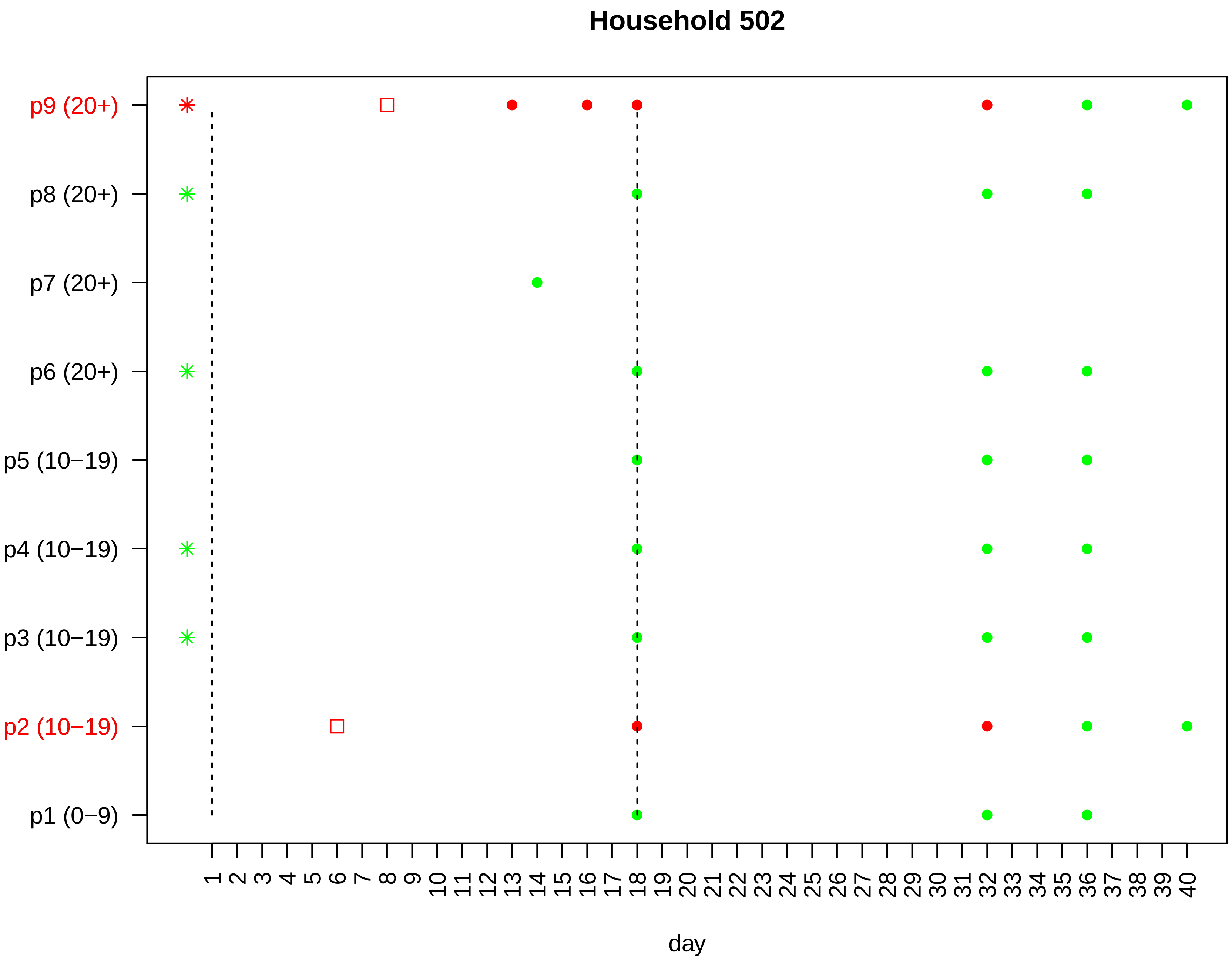

Household 502
p9 (20+)
p8 (20+)
p7 (20+)
p6 (20+)
p5 (10−19)
p4 (10−19)
p3 (10−19)
p2 (10−19)
p1 (0−9)
1 2 3 4 5 6 7 8 9 10 11 12 13 14 15 16 17 18 19 20 21 22 23 24 25 26 27 28 29 30 31 32 33 34 35 36 37 38 39 40
day

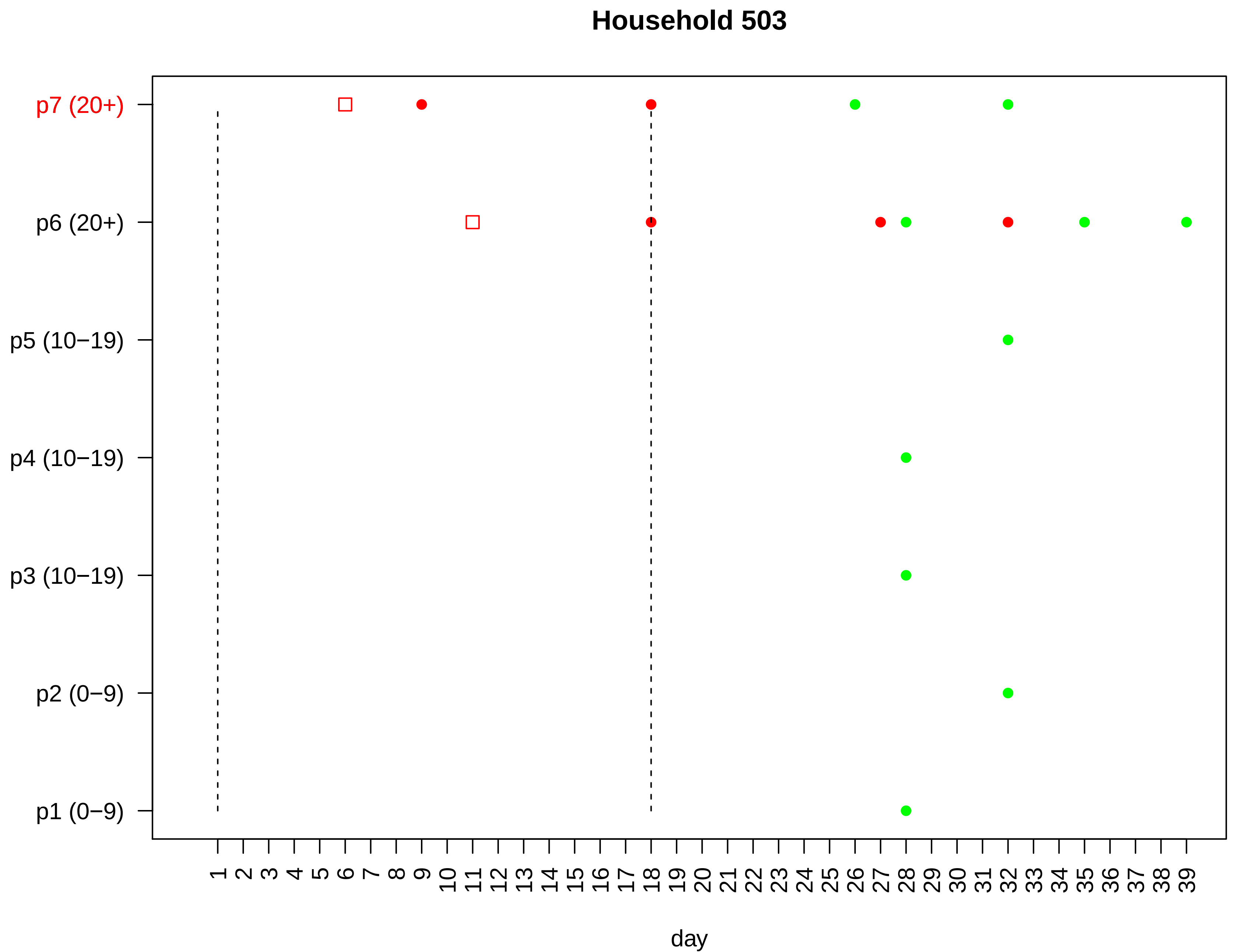
Household 503
p7 (20+)
p6 (20+)
p5 (10−19)
p4 (10−19)
p3 (10−19)
p2 (0−9)
p1 (0−9)
1 2 3 4 5 6 7 8 9 10 11 12 13 14 15 16 17 18 19 20 21 22 23 24 25 26 27 28 29 30 31 32 33 34 35 36 37 38 39
day

Household 504

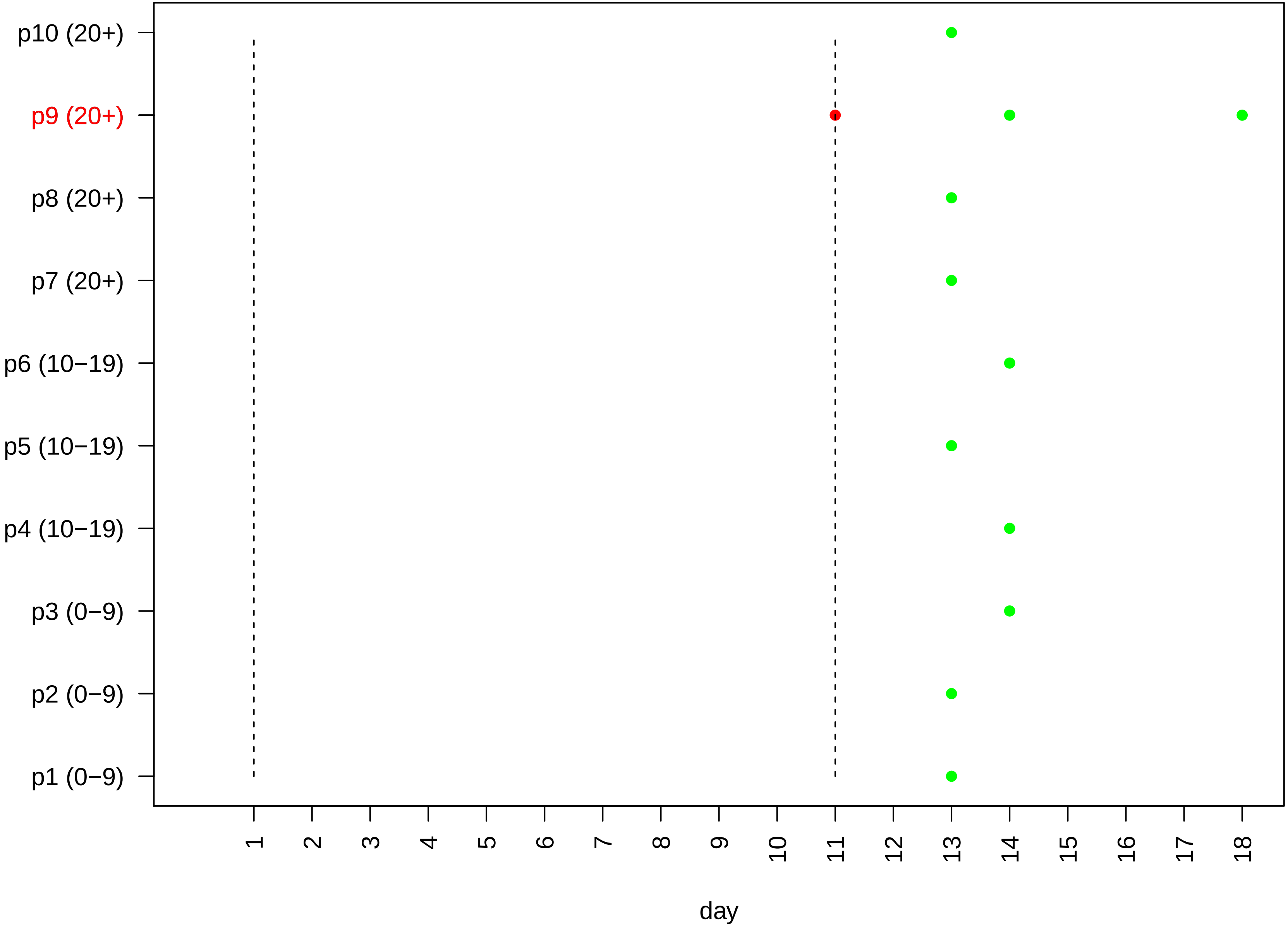

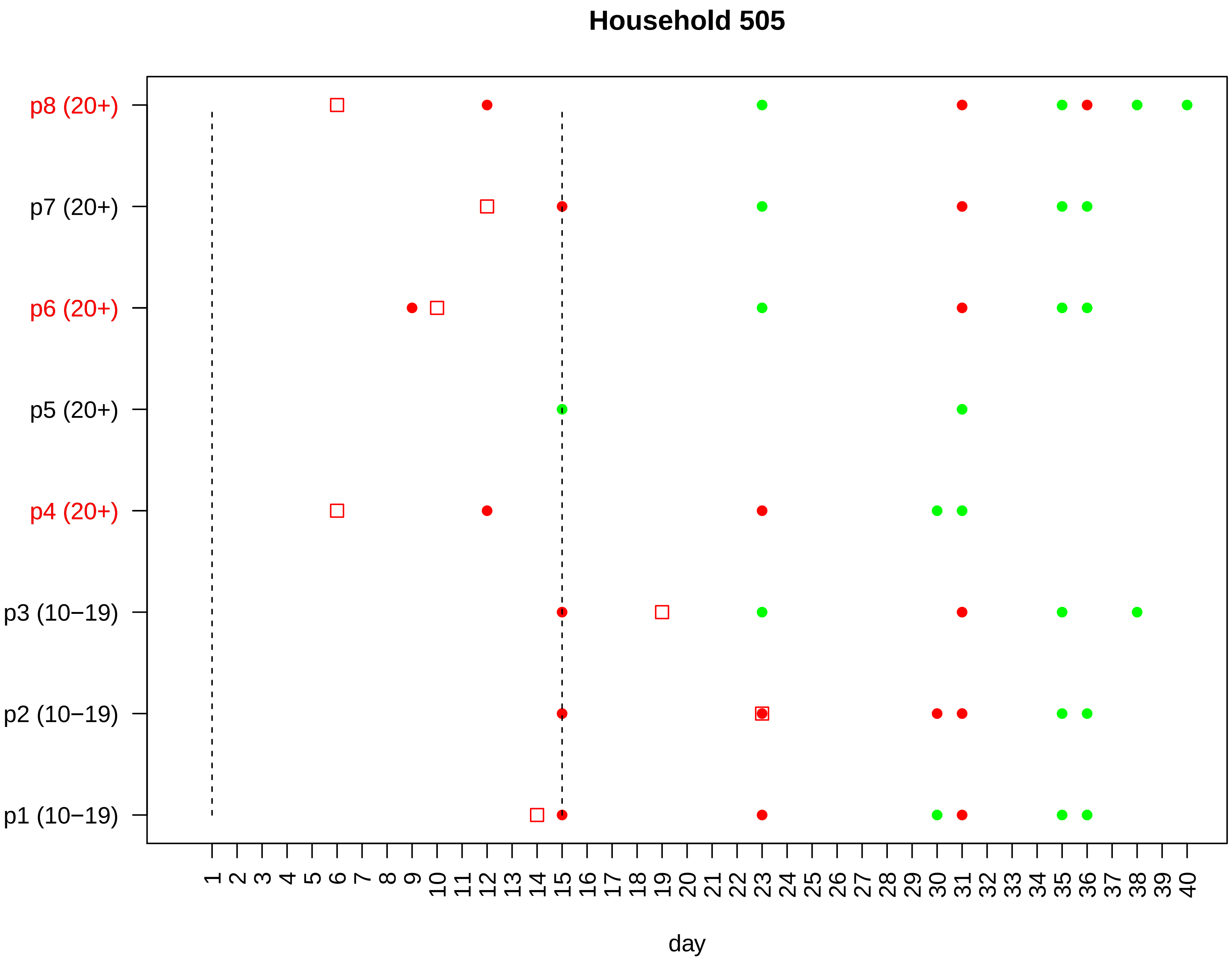

Household 505
p8 (20+)
p7 (20+)
p6 (20+)
p5 (20+)
p4 (20+)
p3 (10−19)
p2 (10−19)
p1 (10−19)
1 2 3 4 5 6 7 8 9 10 11 12 13 14 15 16 17 18 19 20 21 22 23 24 25 26 27 28 29 30 31 32 33 34 35 36 37 38 39 40
day

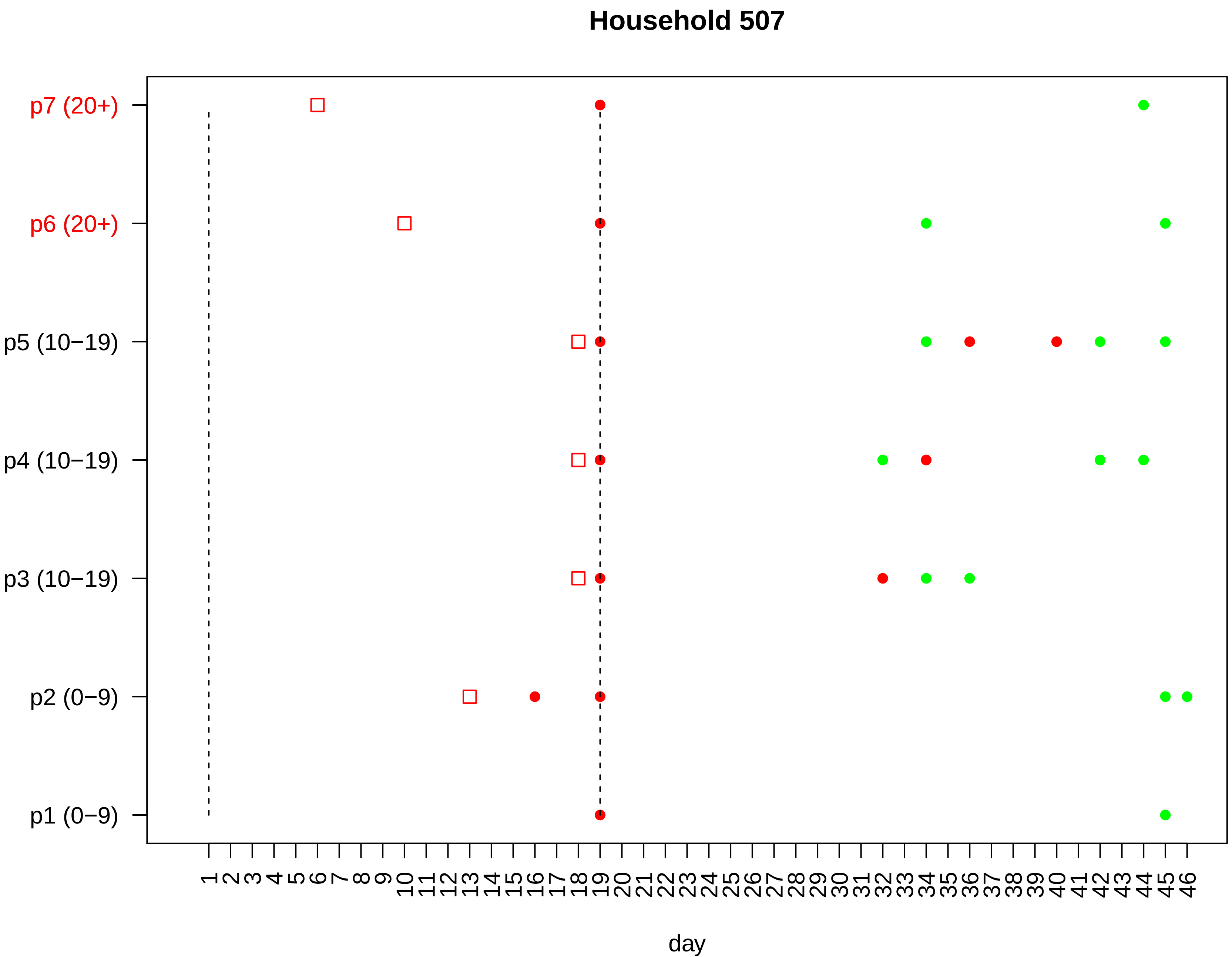

Household 507
p7 (20+)
p6 (20+)
p5 (10−19)
p4 (10−19)
p3 (10−19)
p2 (0−9)
p1 (0−9)
1 2 3 4 5 6 7 8 9 10 11 12 13 14 15 16 17 18 19 20 21 22 23 24 25 26 27 28 29 30 31 32 33 34 35 36 37 38 39 40 41 42 43 44 45 46
day

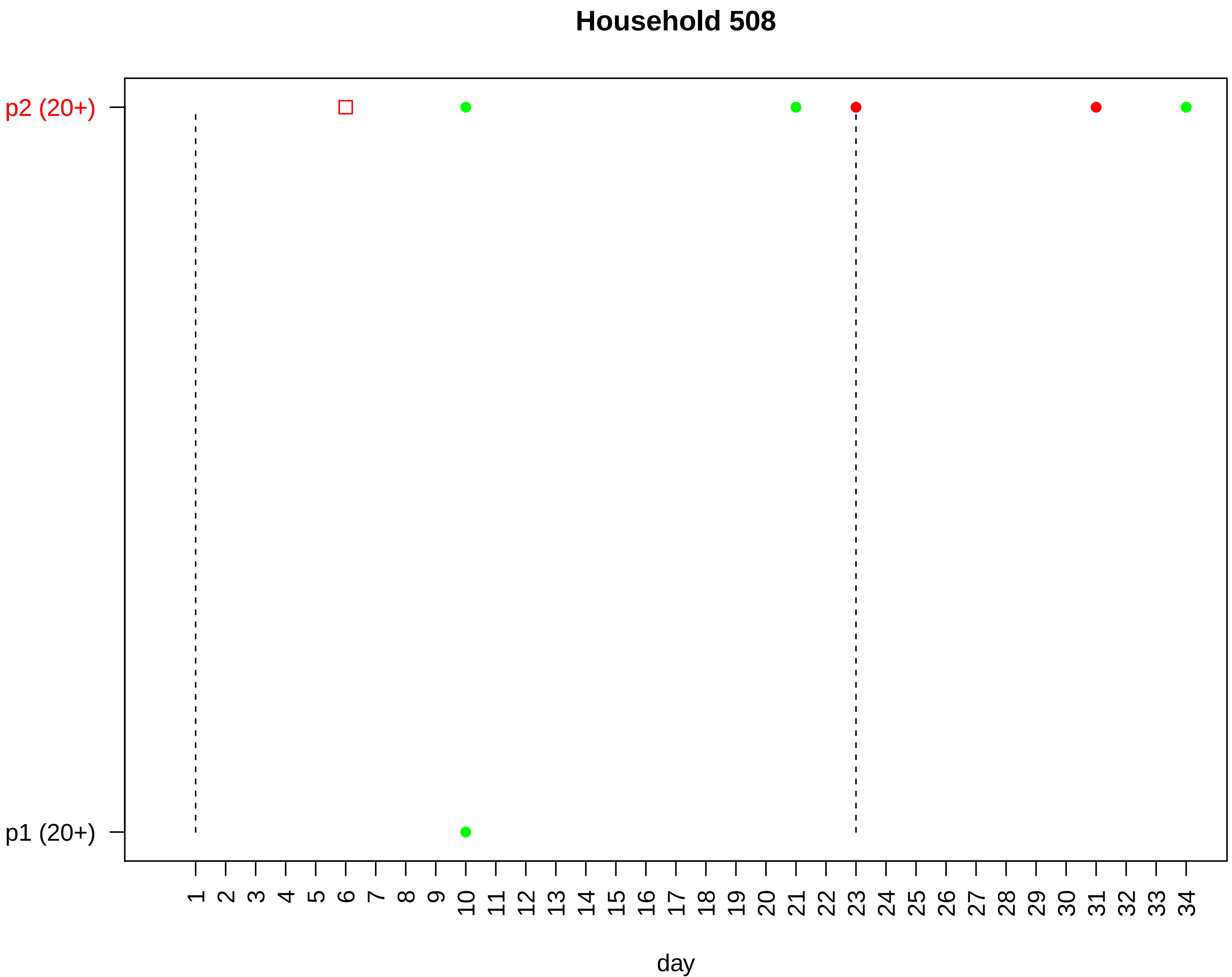

Household 508
p2 (20+)
p1 (20+)
1
2
3
4
5
6
7
8
9
10
11
12
13
14
15
16
17
18
19
20
21
22
23
24
25
26
27
28
29
30
31
32
33
34
day

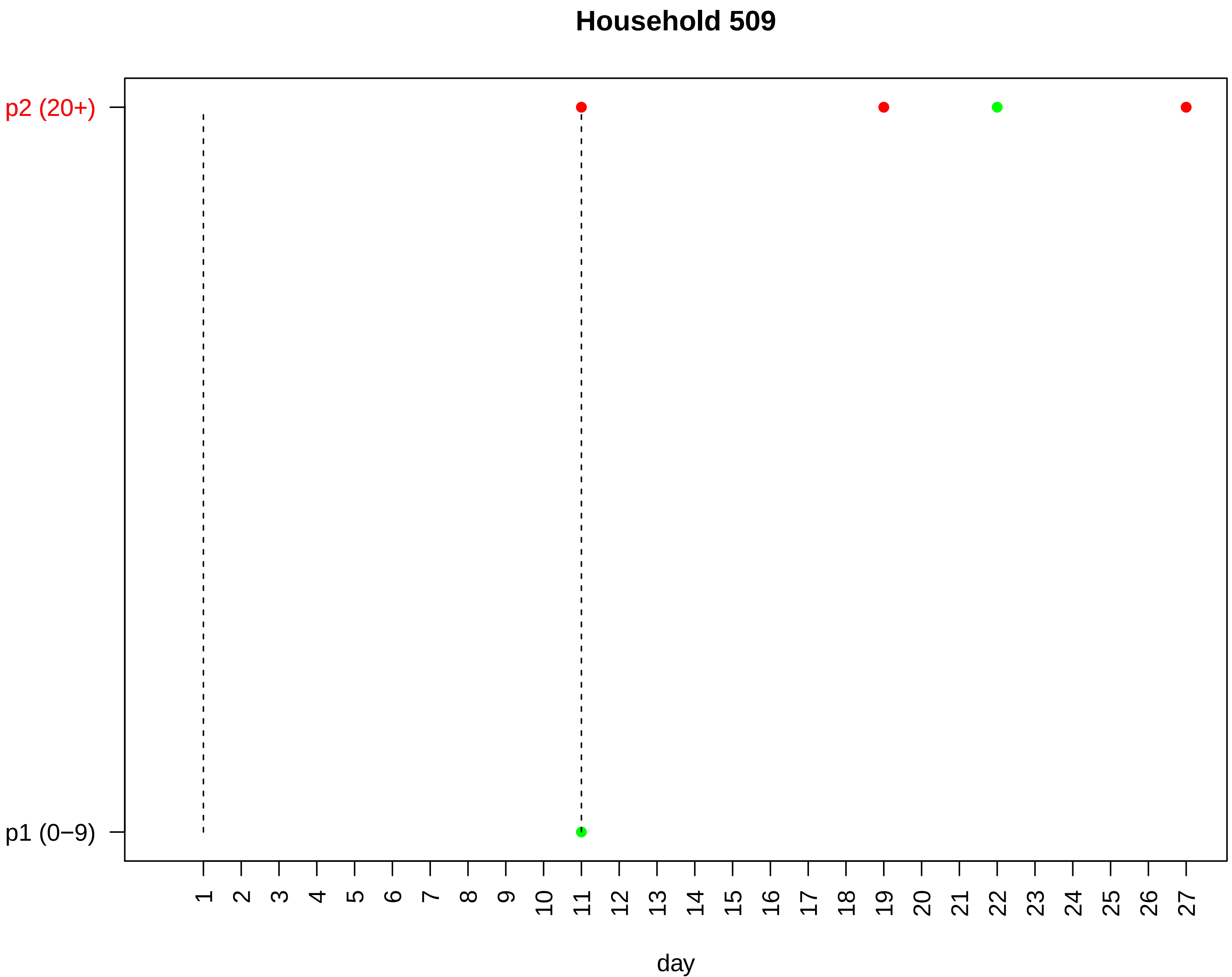

Household 509
p2 (20+)
p1 (0−9)
1
2
3
4
5
6
7
8
9
10
11
12
13
14
15
16
17
18
19
20
21
22
23
24
25
26
27
day

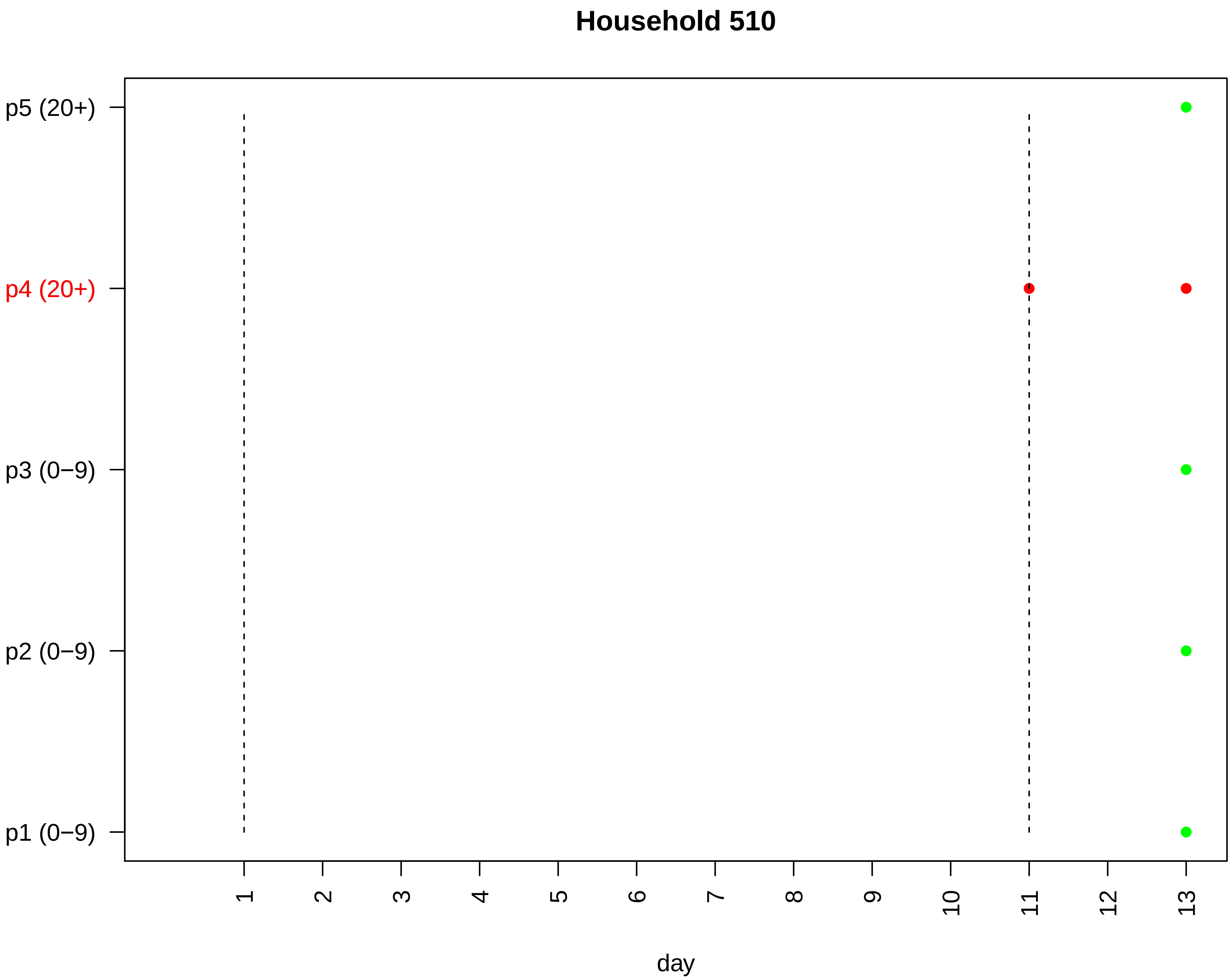
Household 510
p5 (20+)
p4 (20+)
p3 (0−9)
p2 (0−9)
p1 (0−9)
1
2
3
4
5
6
7
8
9
10
11
12
13
day

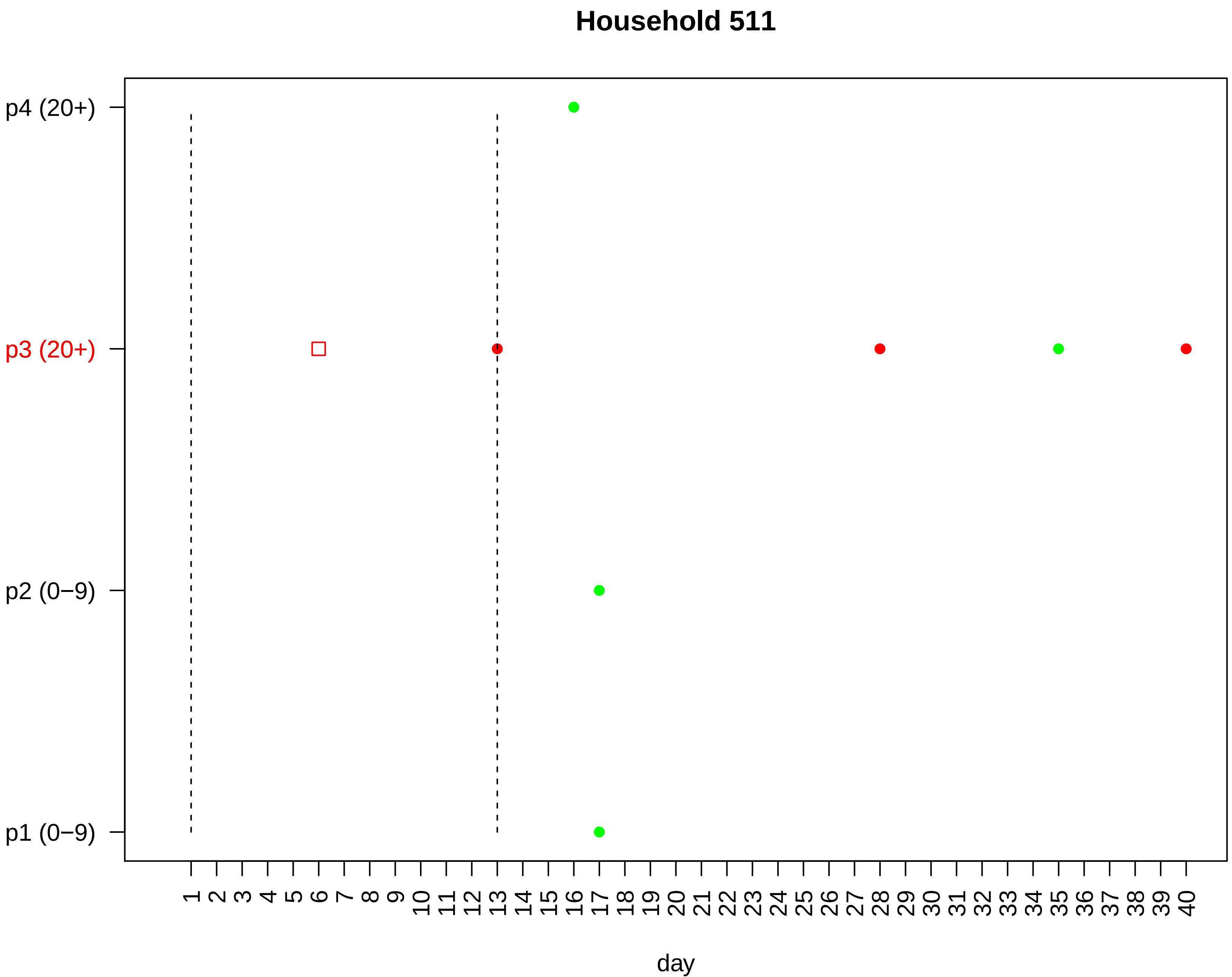
Household 511
p4 (20+)
p3 (20+)
p2 (0−9)
p1 (0−9)
1 2 3 4 5 6 7 8 9 10 11 12 13 14 15 16 17 18 19 20 21 22 23 24 25 26 27 28 29 30 31 32 33 34 35 36 37 38 39 40
day

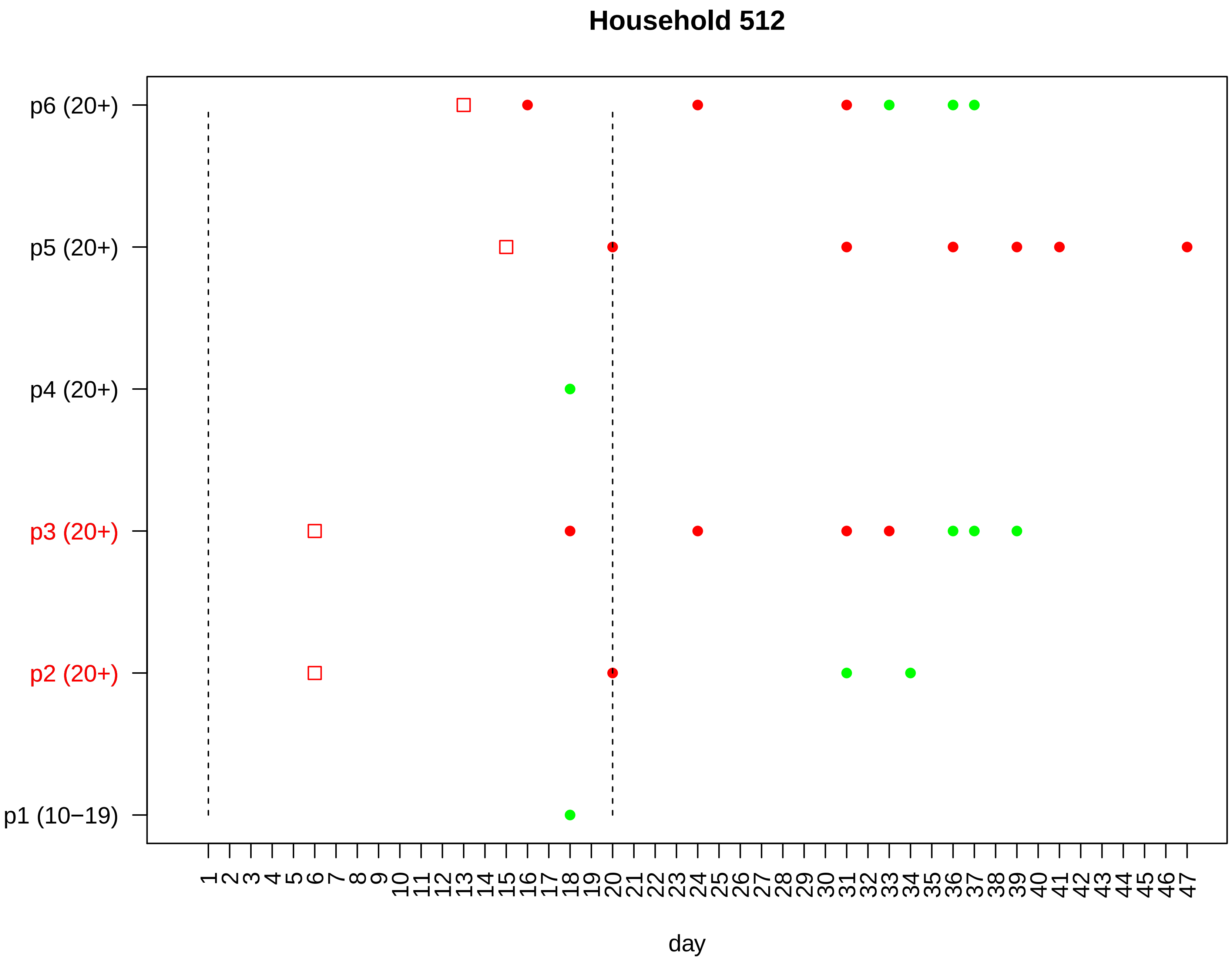
Household 512
p6 (20+)
p5 (20+)
p4 (20+)
p3 (20+)
p2 (20+)
p1 (10−19)
1 2 3 4 5 6 7 8 9 10 11 12 13 14 15 16 17 18 19 20 21 22 23 24 25 26 27 28 29 30 31 32 33 34 35 36 37 38 39 40 41 42 43 44 45 46 47
day

Household 513

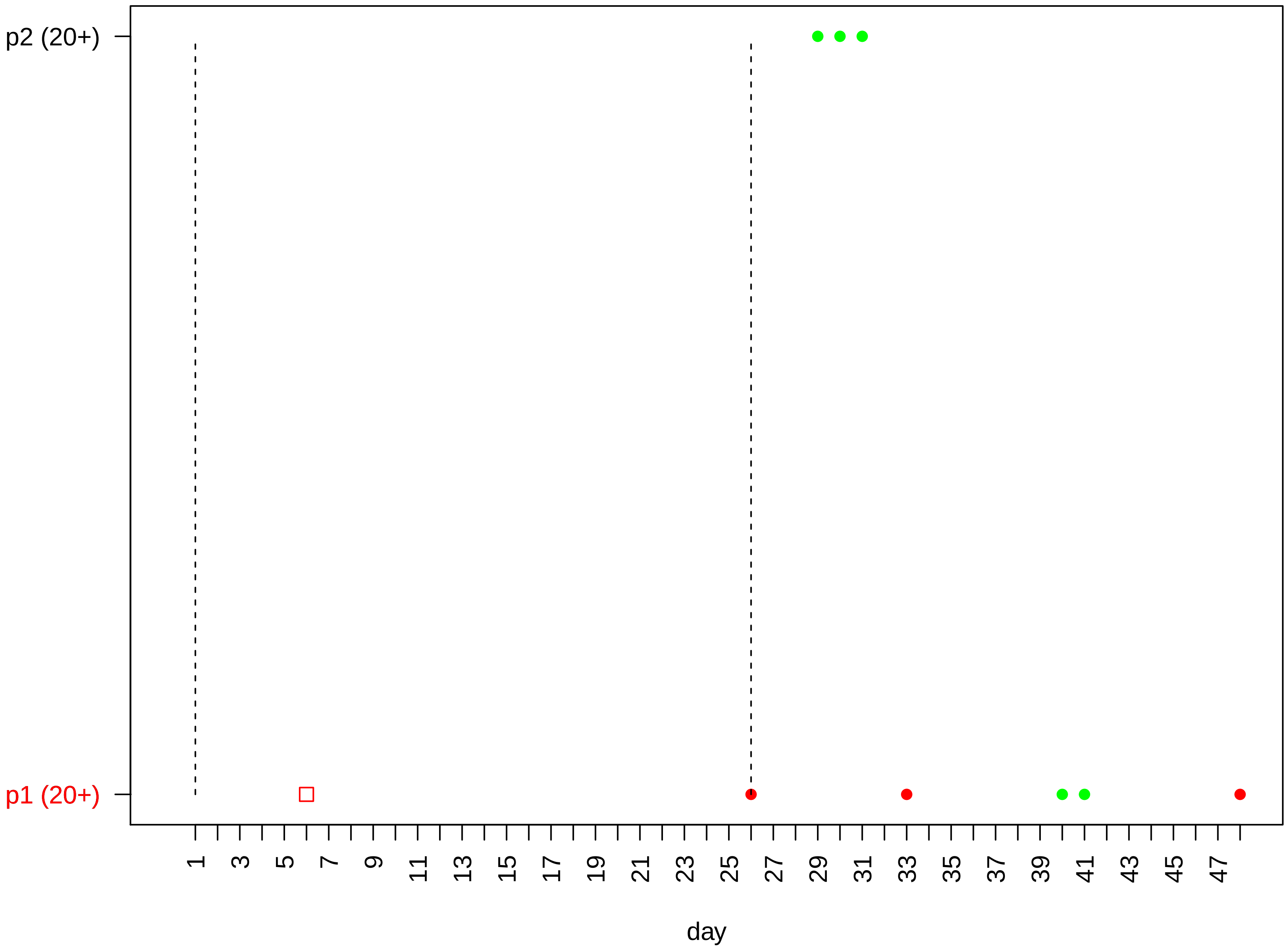

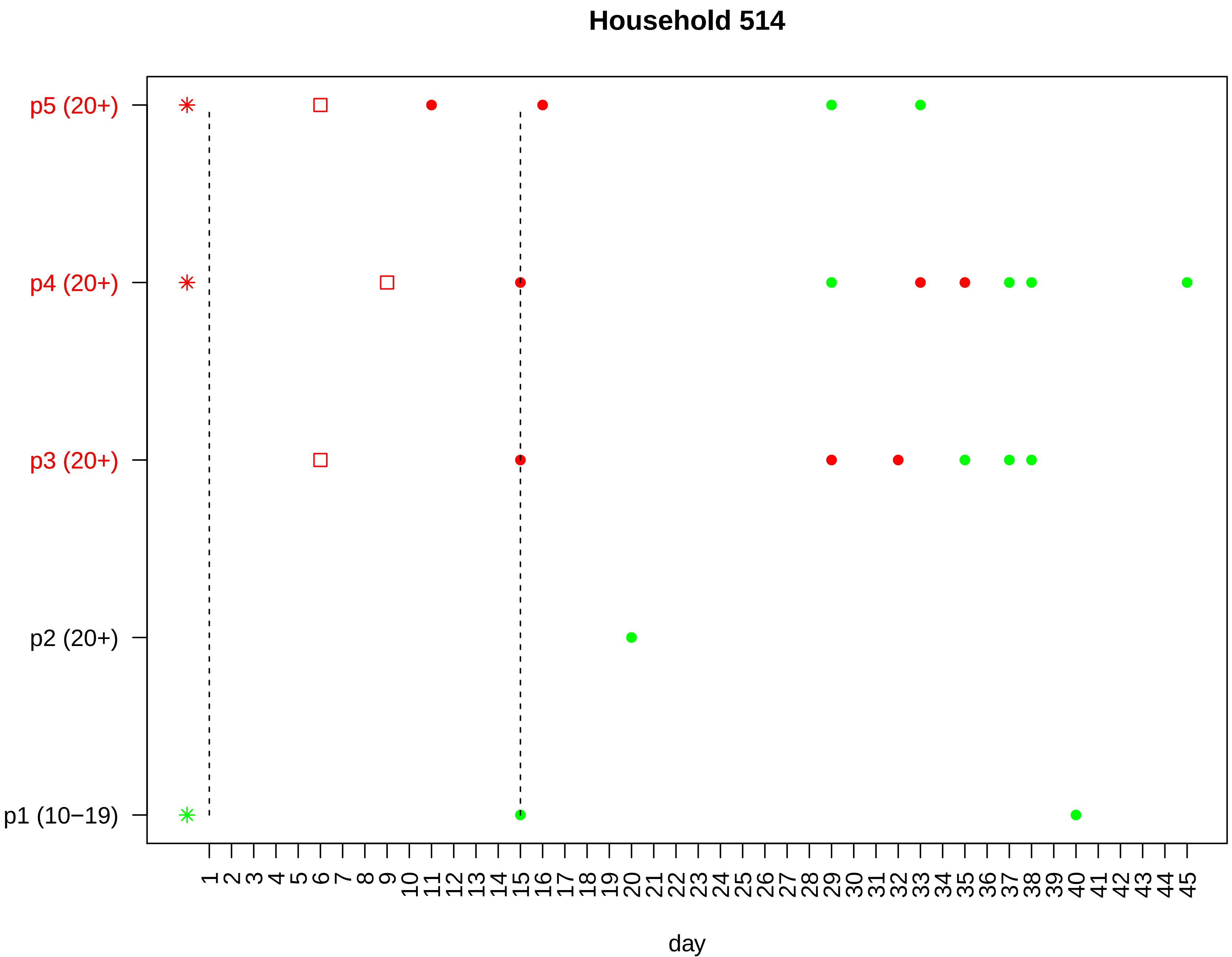

Household 514
p5 (20+)
p4 (20+)
p3 (20+)
p2 (20+)
p1 (10−19)
1 2 3 4 5 6 7 8 9 10 11 12 13 14 15 16 17 18 19 20 21 22 23 24 25 26 27 28 29 30 31 32 33 34 35 36 37 38 39 40 41 42 43 44 45
day

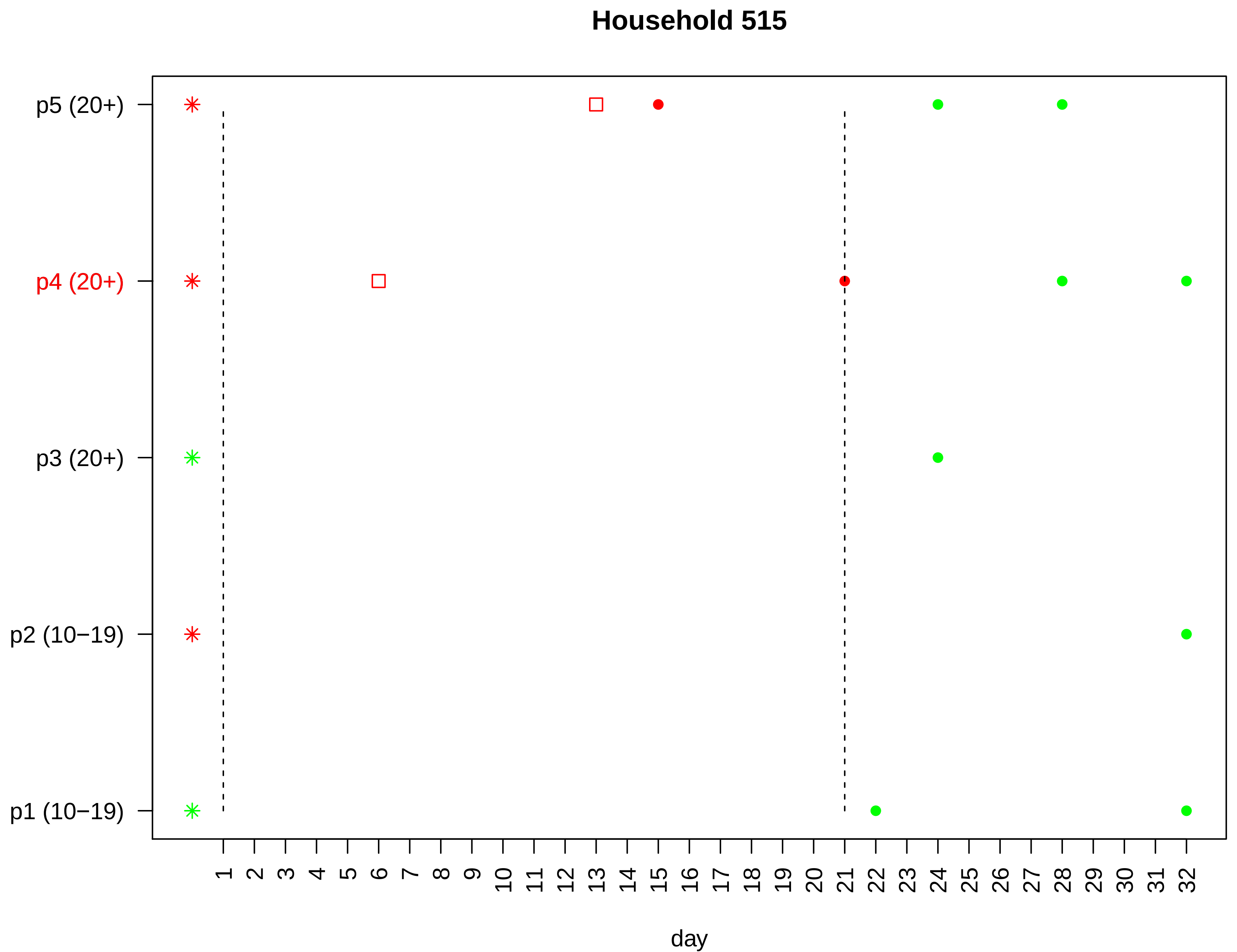

Household 515
p5 (20+)
p4 (20+)
p3 (20+)
p2 (10−19)
p1 (10−19)
1
2
3
4
5
6
7
8
9
10
11
12
13
14
15
16
17
18
19
20
21
22
23
24
25
26
27
28
29
30
31
32
day

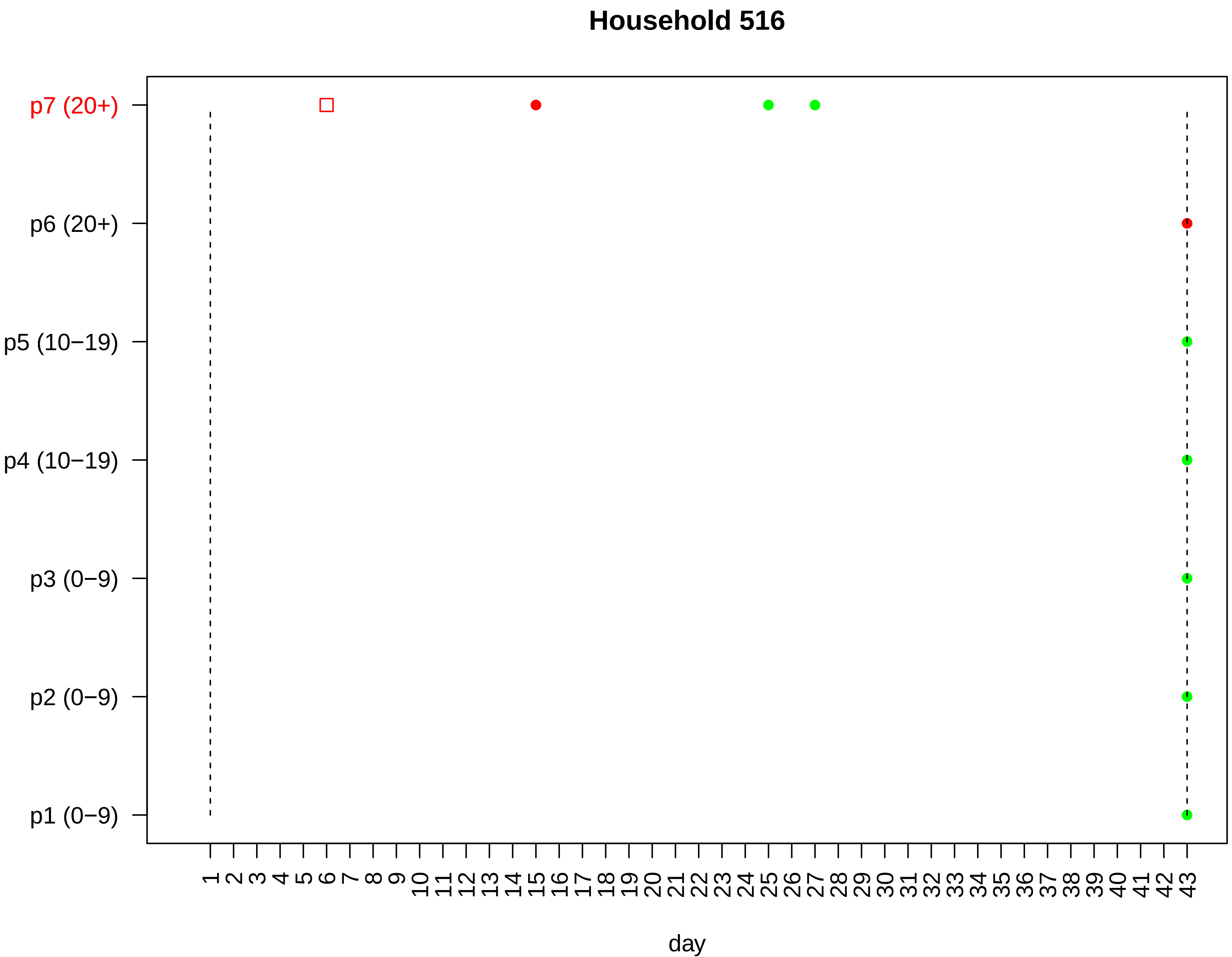

Household 516
p7 (20+)
p6 (20+)
p5 (10−19)
p4 (10−19)
p3 (0−9)
p2 (0−9)
p1 (0−9)
1 2 3 4 5 6 7 8 9 10 11 12 13 14 15 16 17 18 19 20 21 22 23 24 25 26 27 28 29 30 31 32 33 34 35 36 37 38 39 40 41 42 43
day

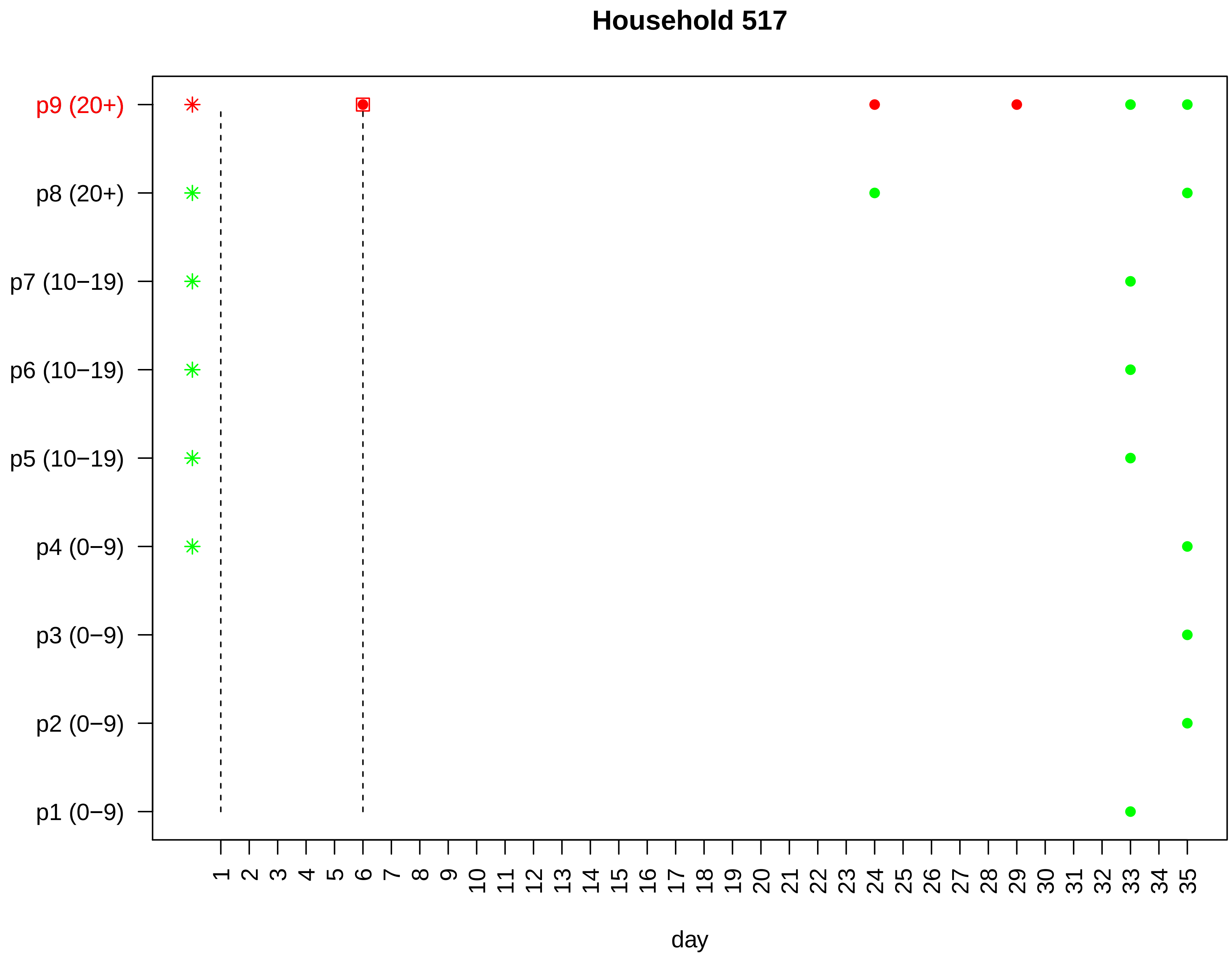

Household 517
p9 (20+)
p8 (20+)
p7 (10−19)
p6 (10−19)
p5 (10−19)
p4 (0−9)
p3 (0−9)
p2 (0−9)
p1 (0−9)
1 2 3 4 5 6 7 8 9 10 11 12 13 14 15 16 17 18 19 20 21 22 23 24 25 26 27 28 29 30 31 32 33 34 35
day

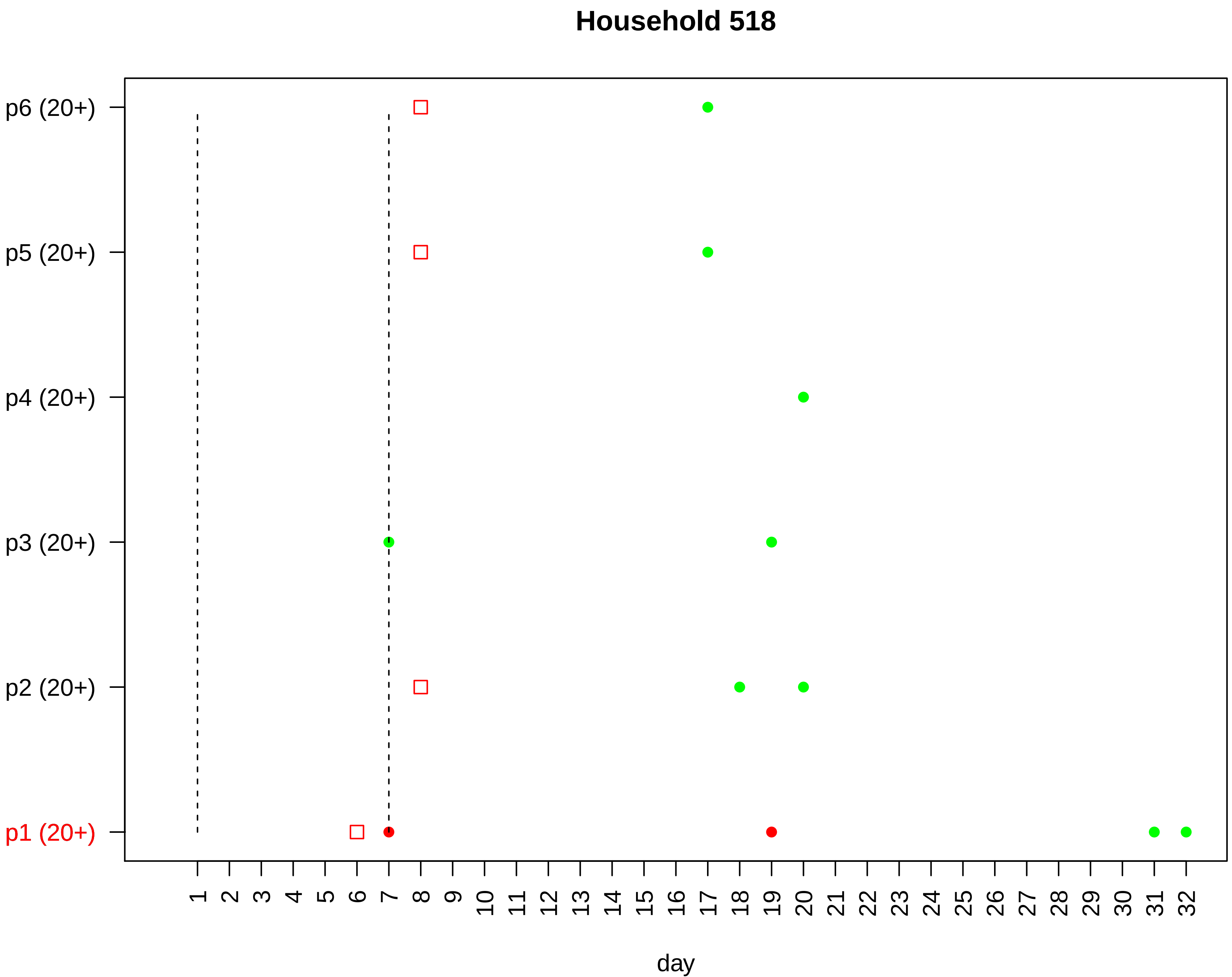

Household 518
p6 (20+)
p5 (20+)
p4 (20+)
p3 (20+)
p2 (20+)
p1 (20+)
1 2 3 4 5 6 7 8 9 10 11 12 13 14 15 16 17 18 19 20 21 22 23 24 25 26 27 28 29 30 31 32
day

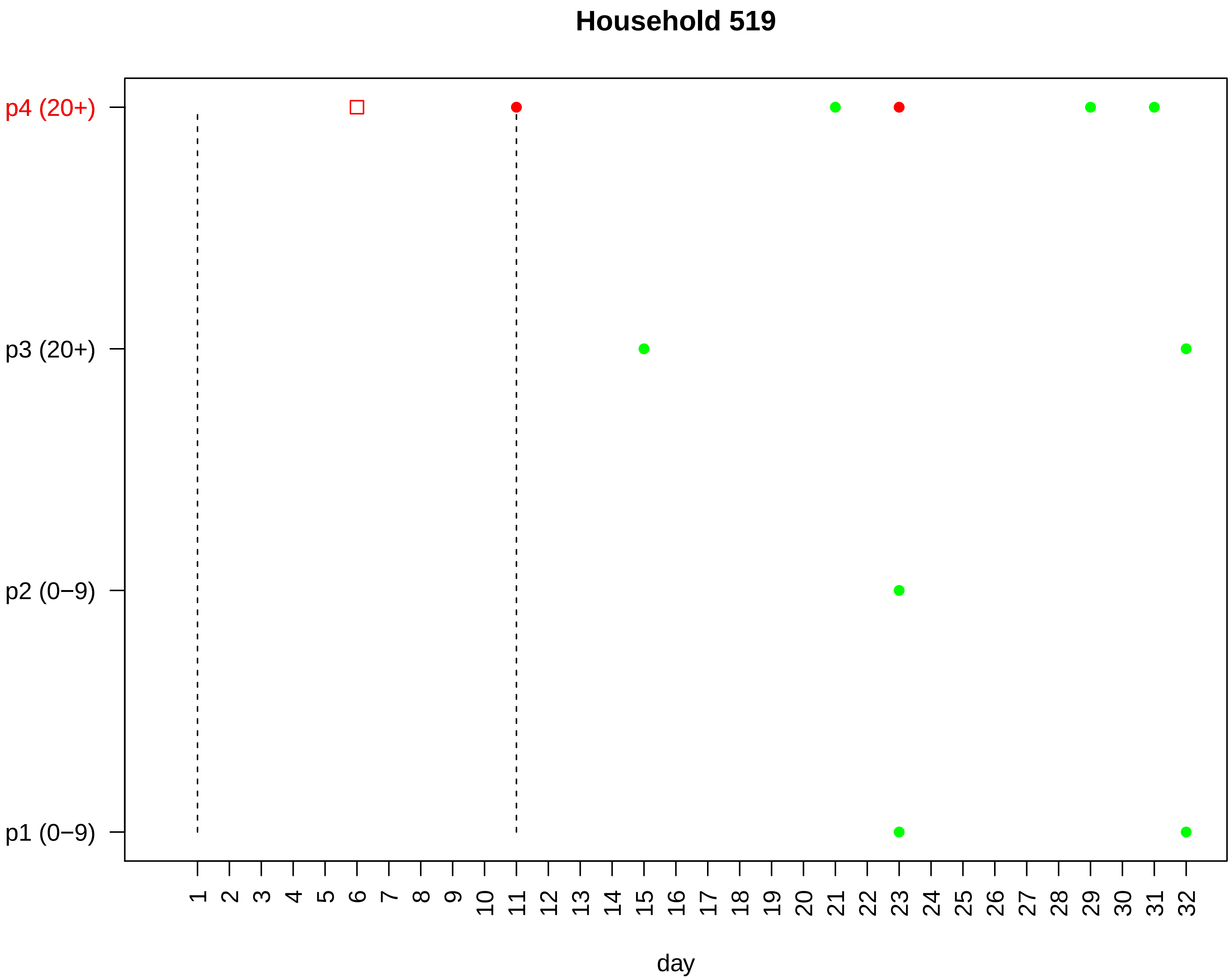

Household 519
p4 (20+)
p3 (20+)
p2 (0−9)
p1 (0−9)
1 2 3 4 5 6 7 8 9 10 11 12 13 14 15 16 17 18 19 20 21 22 23 24 25 26 27 28 29 30 31 32
day

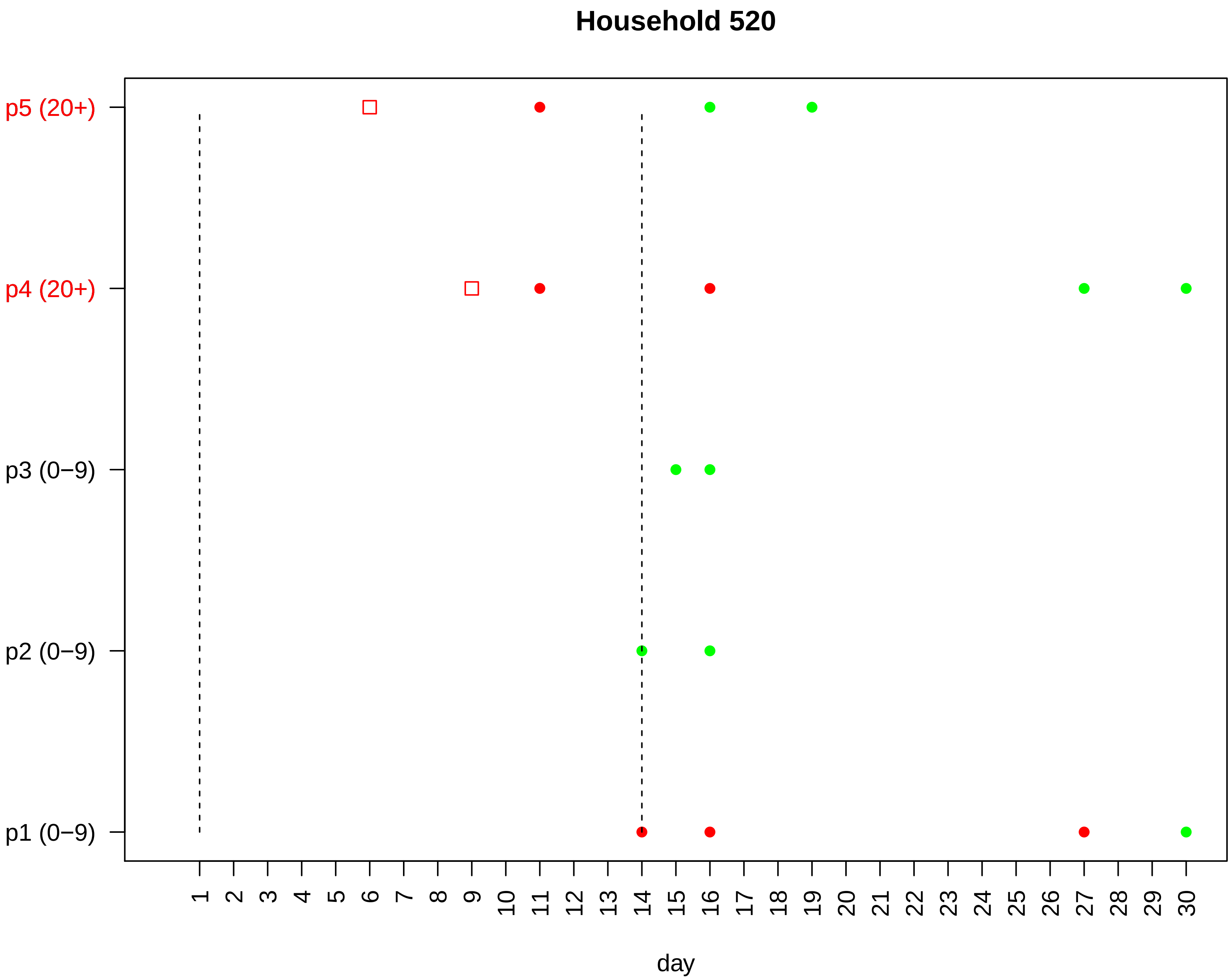

Household 520
p5 (20+)
p4 (20+)
p3 (0−9)
p2 (0−9)
p1 (0−9)
1 2 3 4 5 6 7 8 9 10 11 12 13 14 15 16 17 18 19 20 21 22 23 24 25 26 27 28 29 30
day

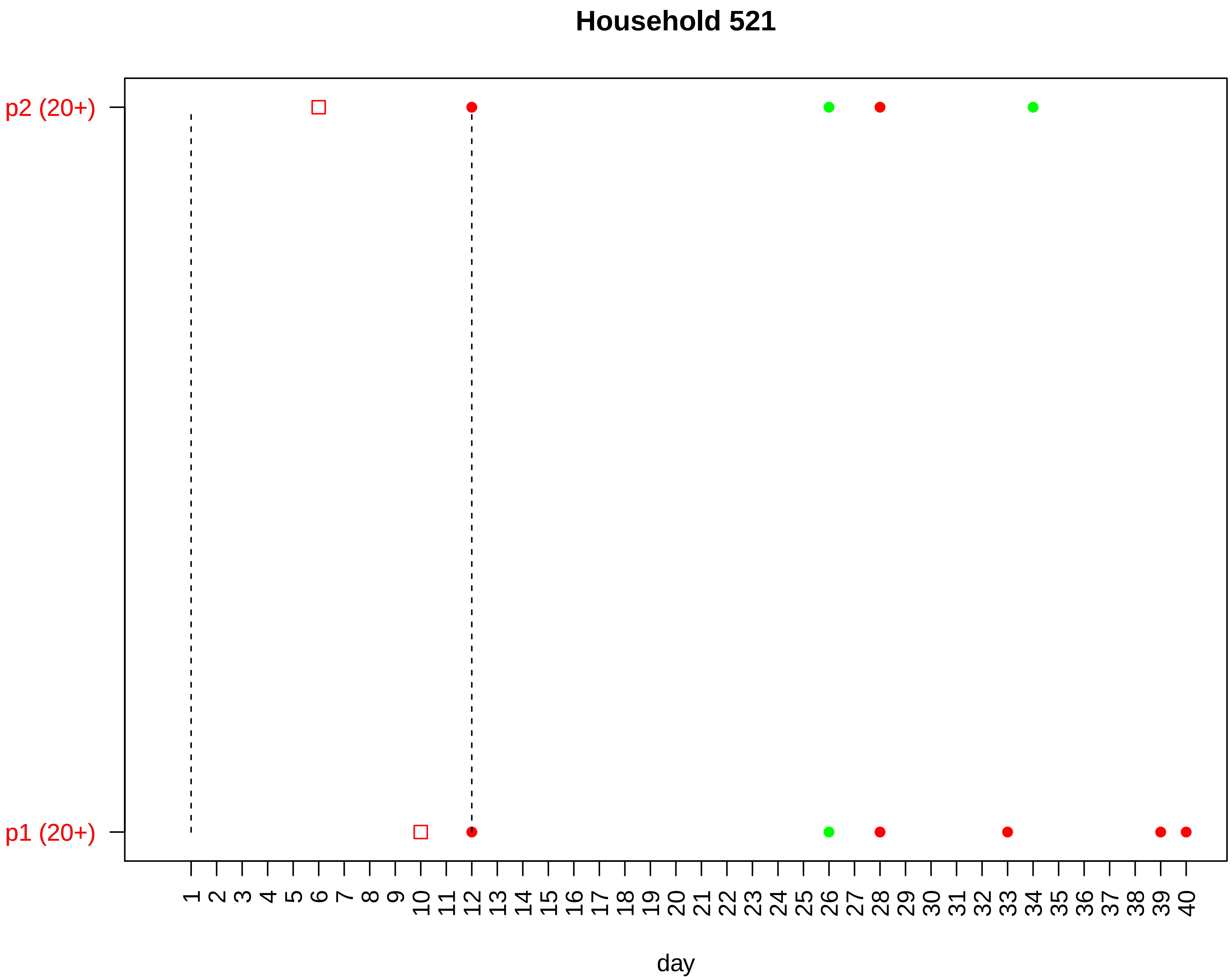

Household 521
p2 (20+)
p1 (20+)
1
2
3
4
5
6
7
8
9
10
11
12
13
14
15
16
17
18
19
20
21
22
23
24
25
26
27
28
29
30
31
32
33
34
35
36
37
38
39
40
day

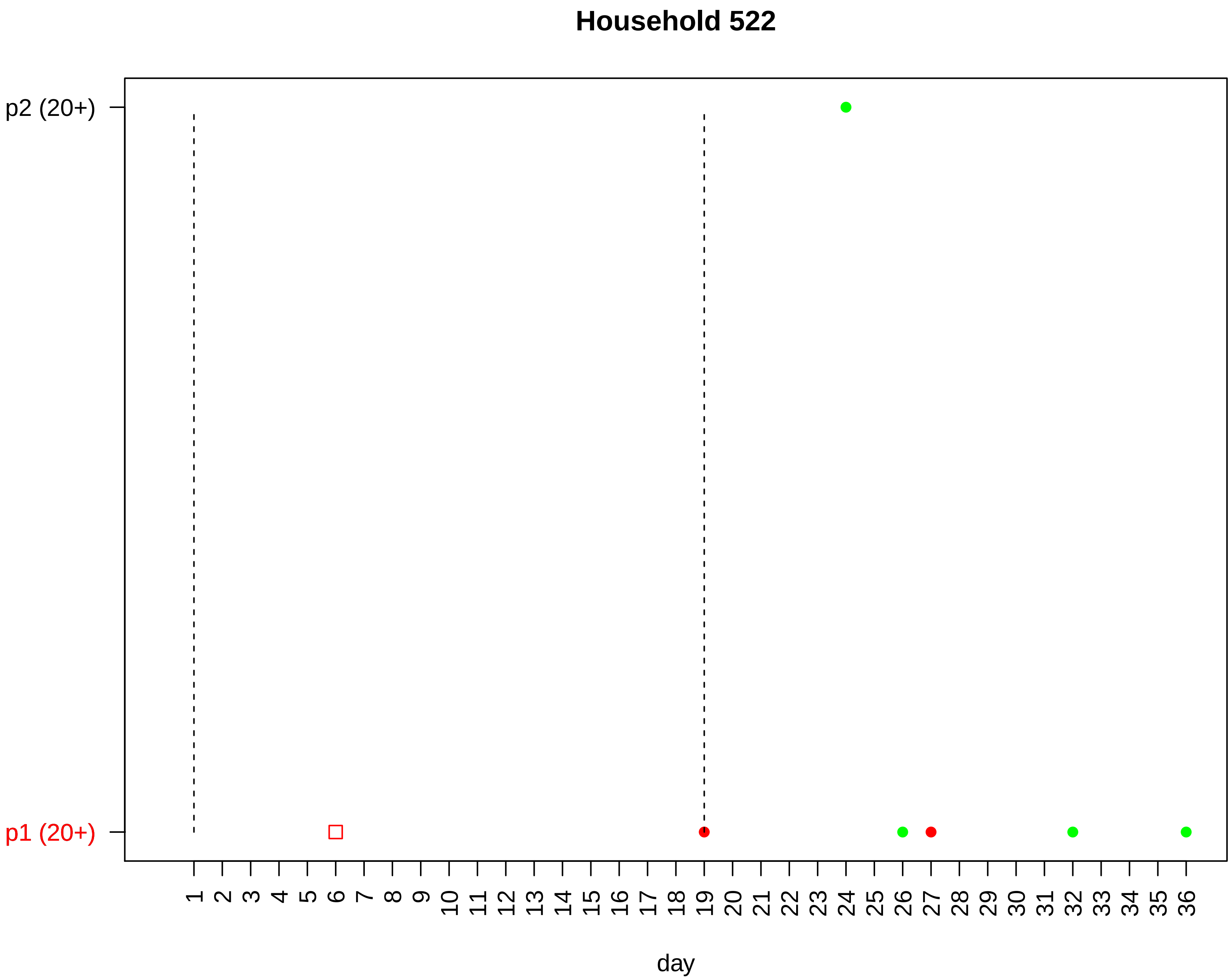

Household 522
p2 (20+)
p1 (20+)
1
2
3
4
5
6
7
8
9
10
11
12
13
14
15
16
17
18
19
20
21
22
23
24
25
26
27
28
29
30
31
32
33
34
35
36
day

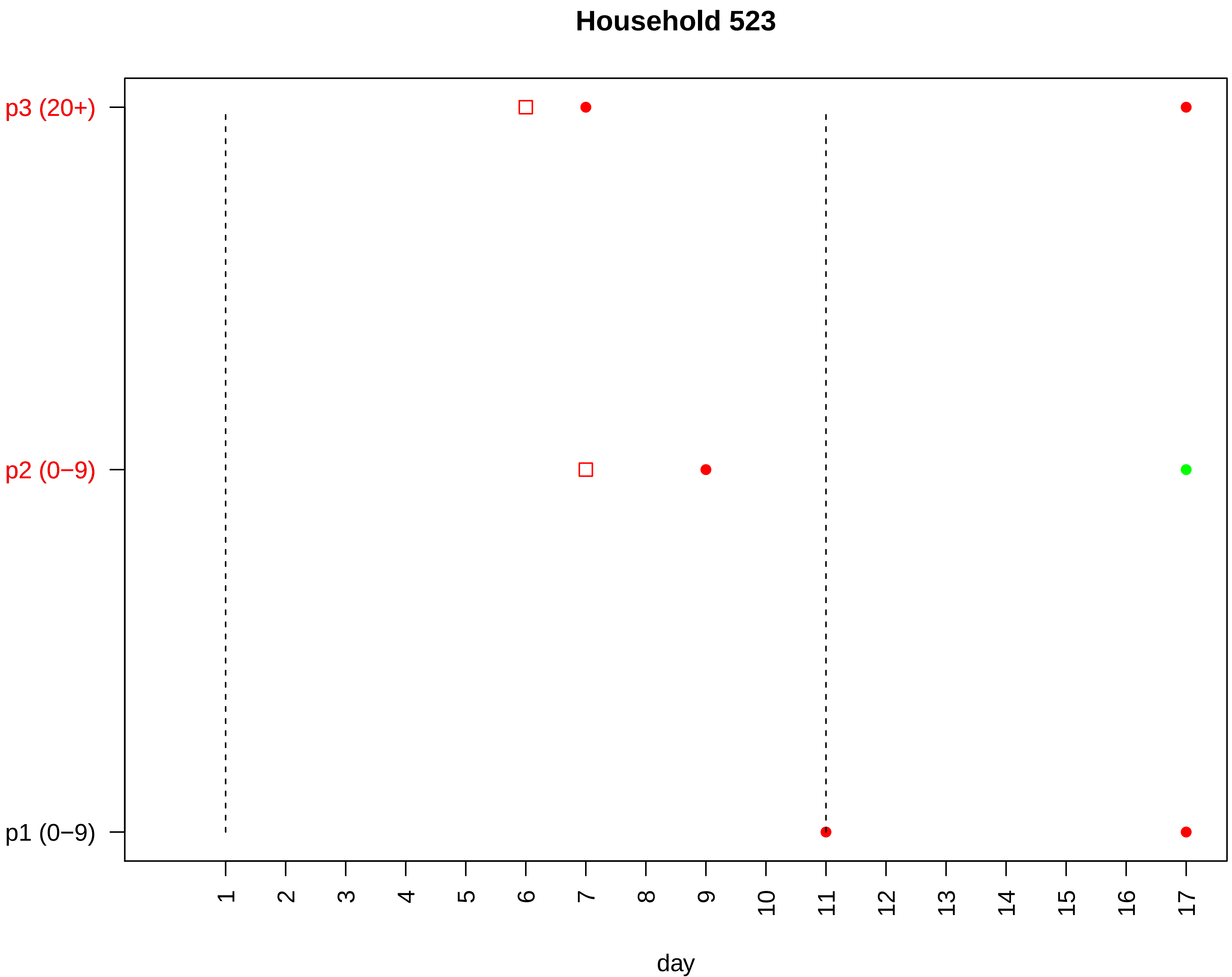

Household 523
p3 (20+)
p2 (0−9)
p1 (0−9)
1
2
3
4
5
6
7
8
9
10
11
12
13
14
15
16
17
day

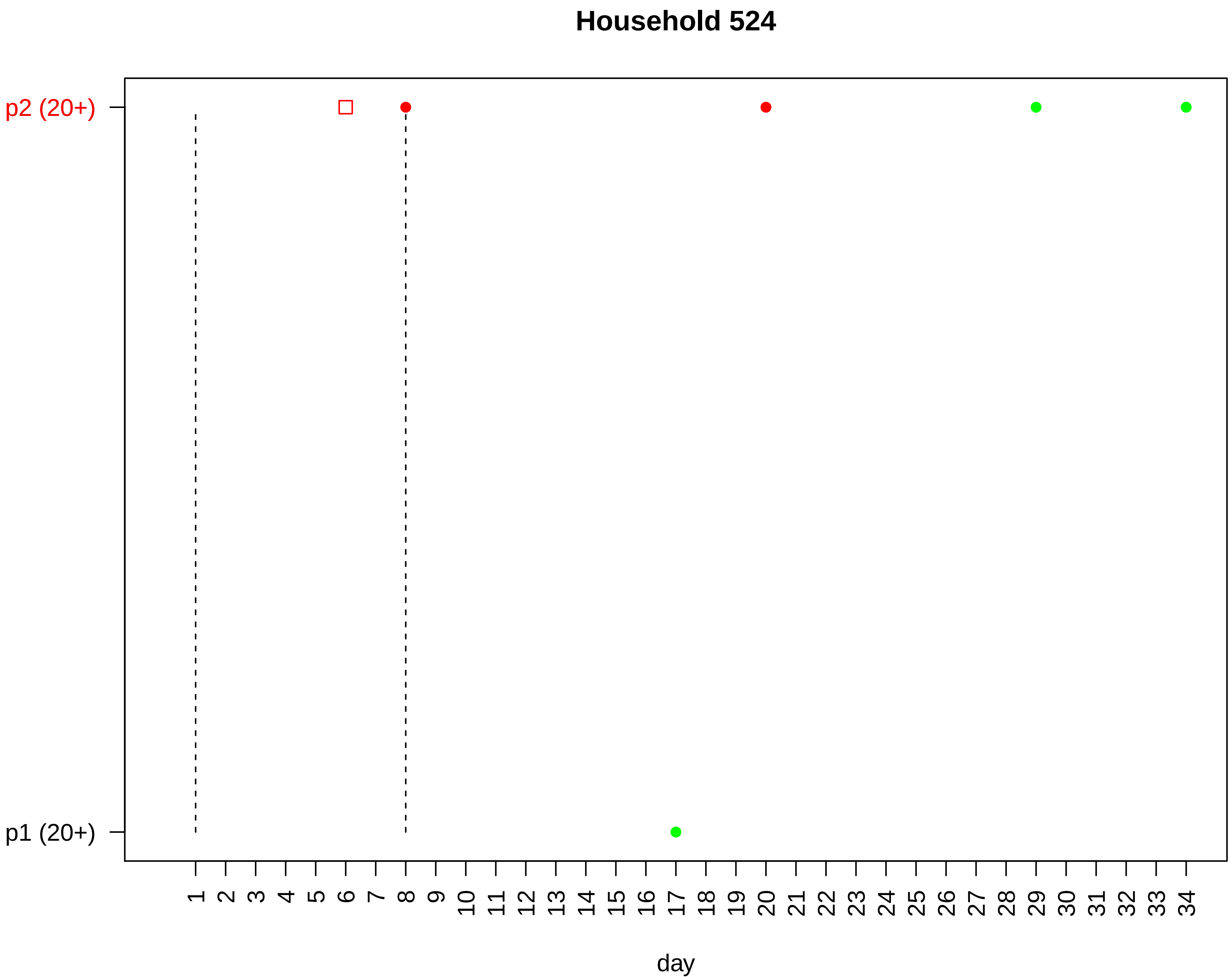

Household 524
p2 (20+)
p1 (20+)
1
2
3
4
5
6
7
8
9
10
11
12
13
14
15
16
17
18
19
20
21
22
23
24
25
26
27
28
29
30
31
32
33
34
day

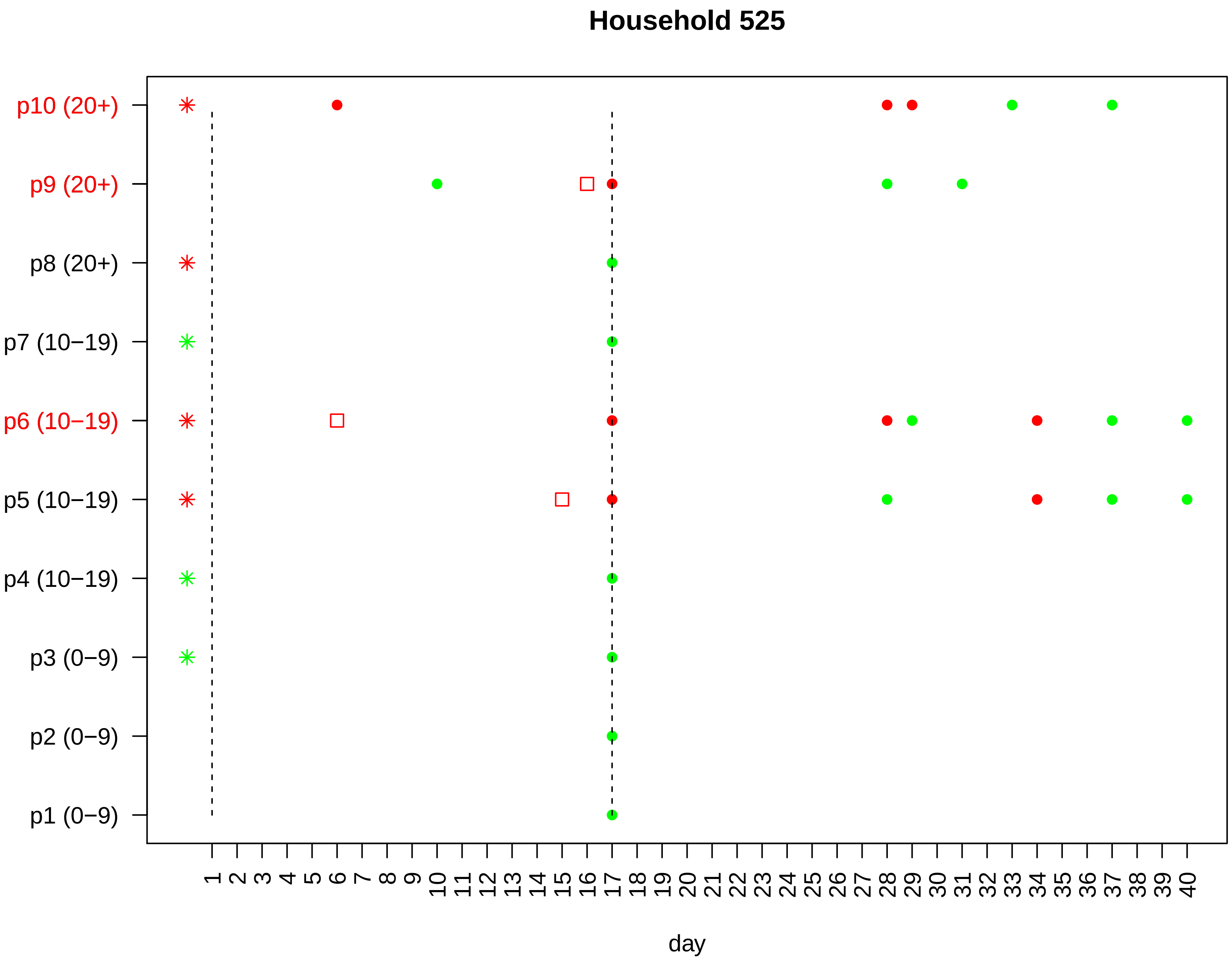

Household 525
p10 (20+)
p9 (20+)
p8 (20+)
p7 (10−19)
p6 (10−19)
p5 (10−19)
p4 (10−19)
p3 (0−9)
p2 (0−9)
p1 (0−9)
1 2 3 4 5 6 7 8 9 10 11 12 13 14 15 16 17 18 19 20 21 22 23 24 25 26 27 28 29 30 31 32 33 34 35 36 37 38 39 40
day

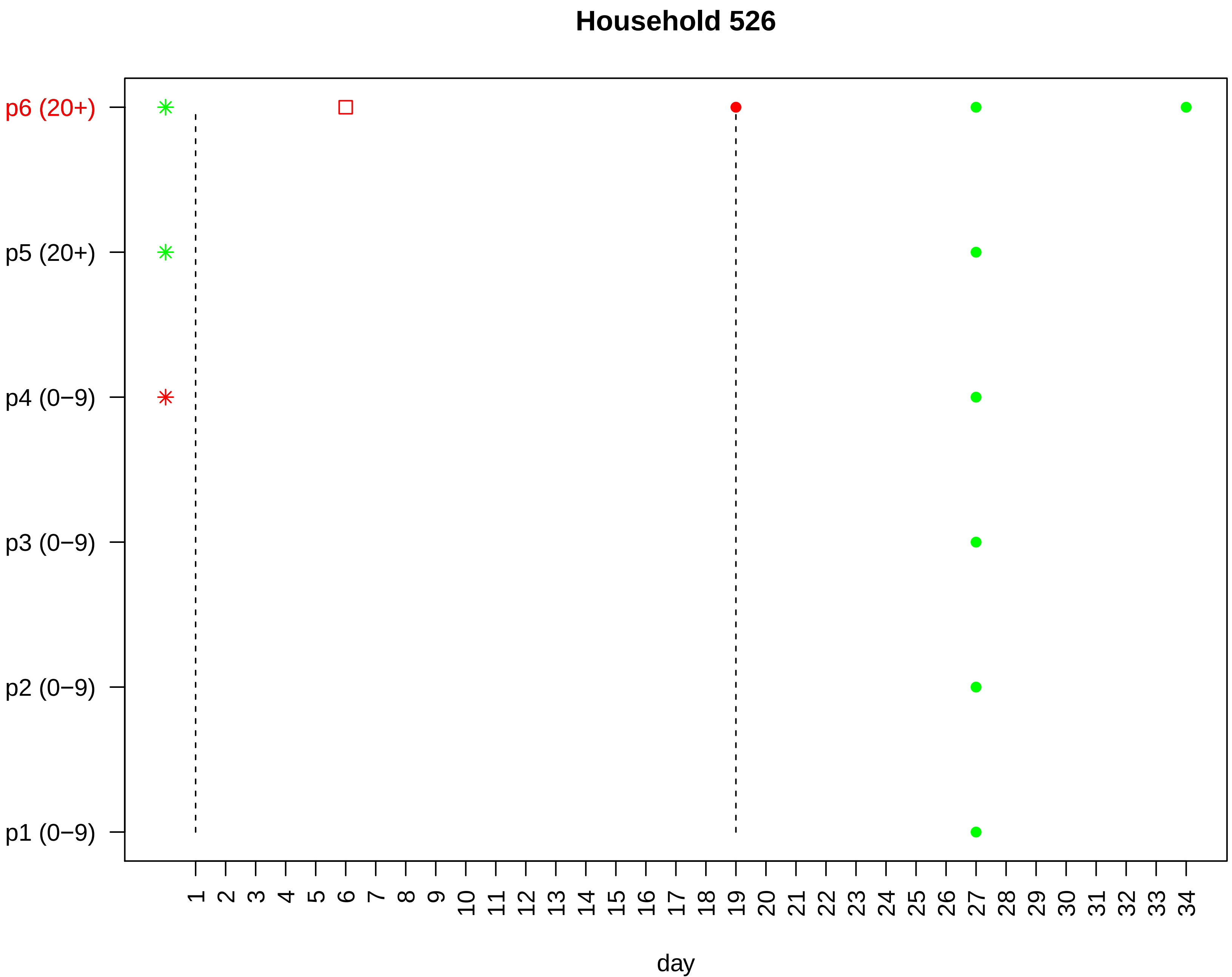
Household 526
p6 (20+)
p5 (20+)
p4 (0−9)
p3 (0−9)
p2 (0−9)
p1 (0−9)
1
2
3
4
5
6
7
8
9
10
11
12
13
14
15
16
17
18
19
20
21
22
23
24
25
26
27
28
29
30
31
32
33
34
day

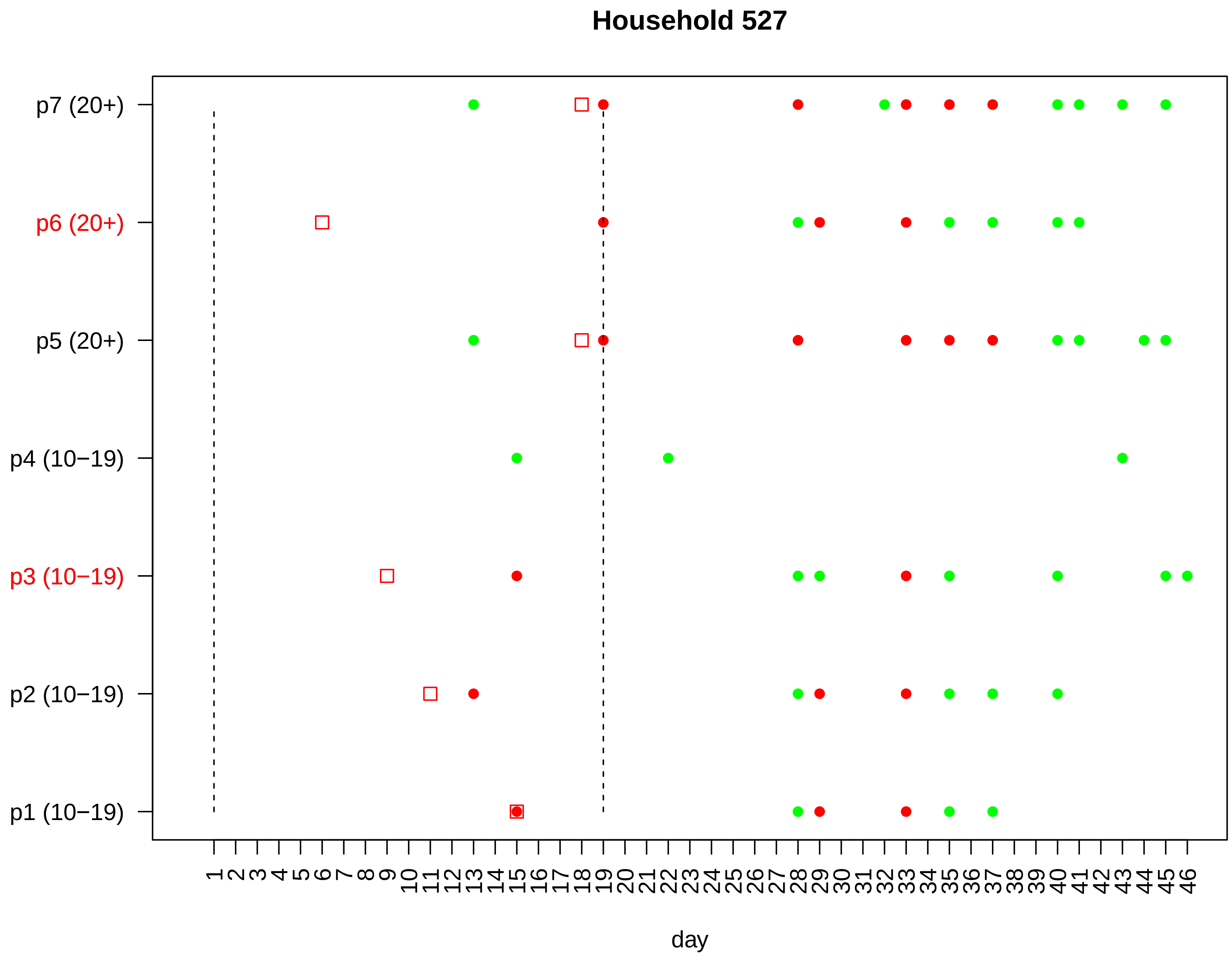
Household 527
p7 (20+)
p6 (20+)
p5 (20+)
p4 (10−19)
p3 (10−19)
p2 (10−19)
p1 (10−19)
1 2 3 4 5 6 7 8 9 10 11 12 13 14 15 16 17 18 19 20 21 22 23 24 25 26 27 28 29 30 31 32 33 34 35 36 37 38 39 40 41 42 43 44 45 46
day

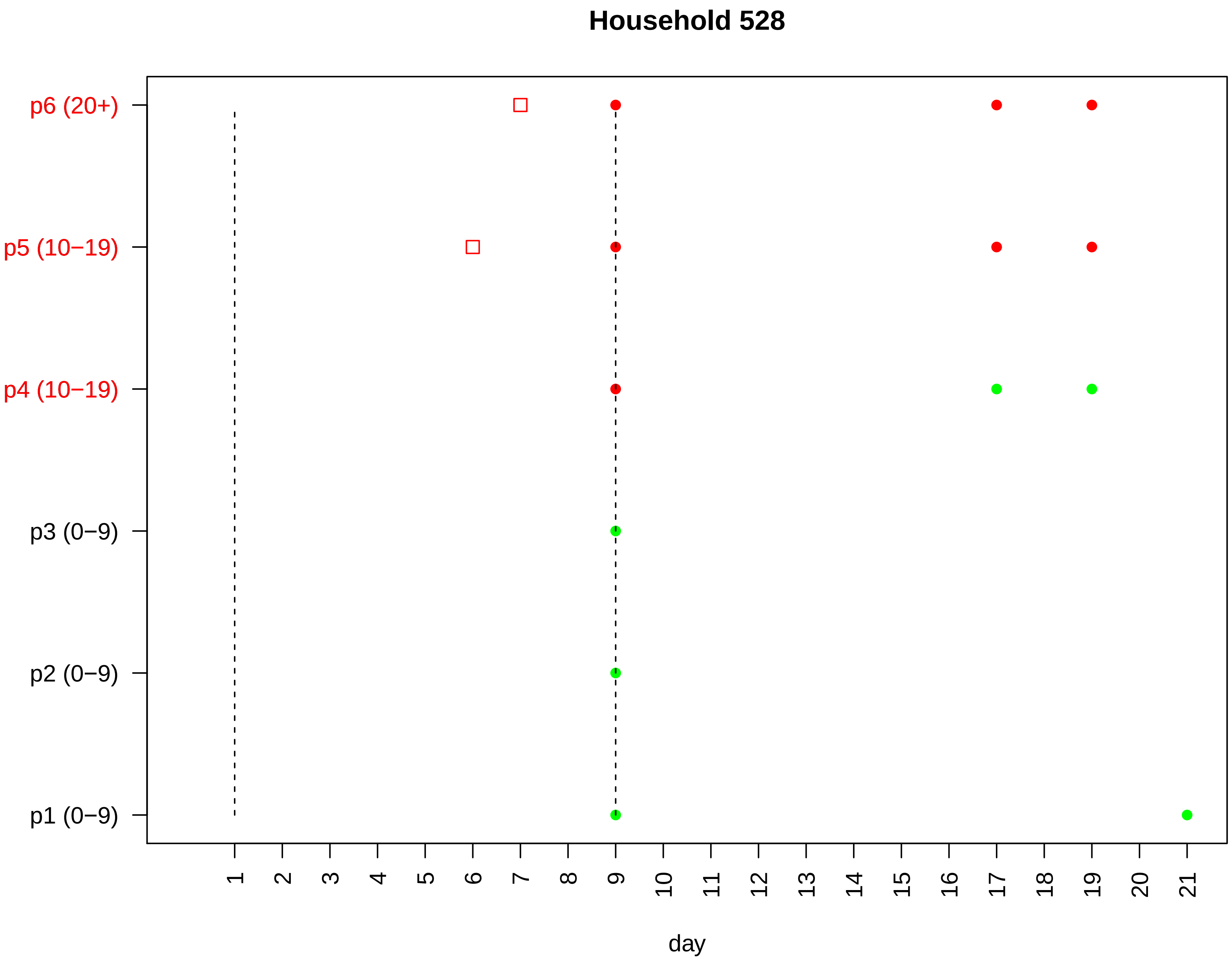

Household 528
p6 (20+)
p5 (10−19)
p4 (10−19)
p3 (0−9)
p2 (0−9)
p1 (0−9)
1
2
3
4
5
6
7
8
9
10
11
12
13
14
15
16
17
18
19
20
21
day

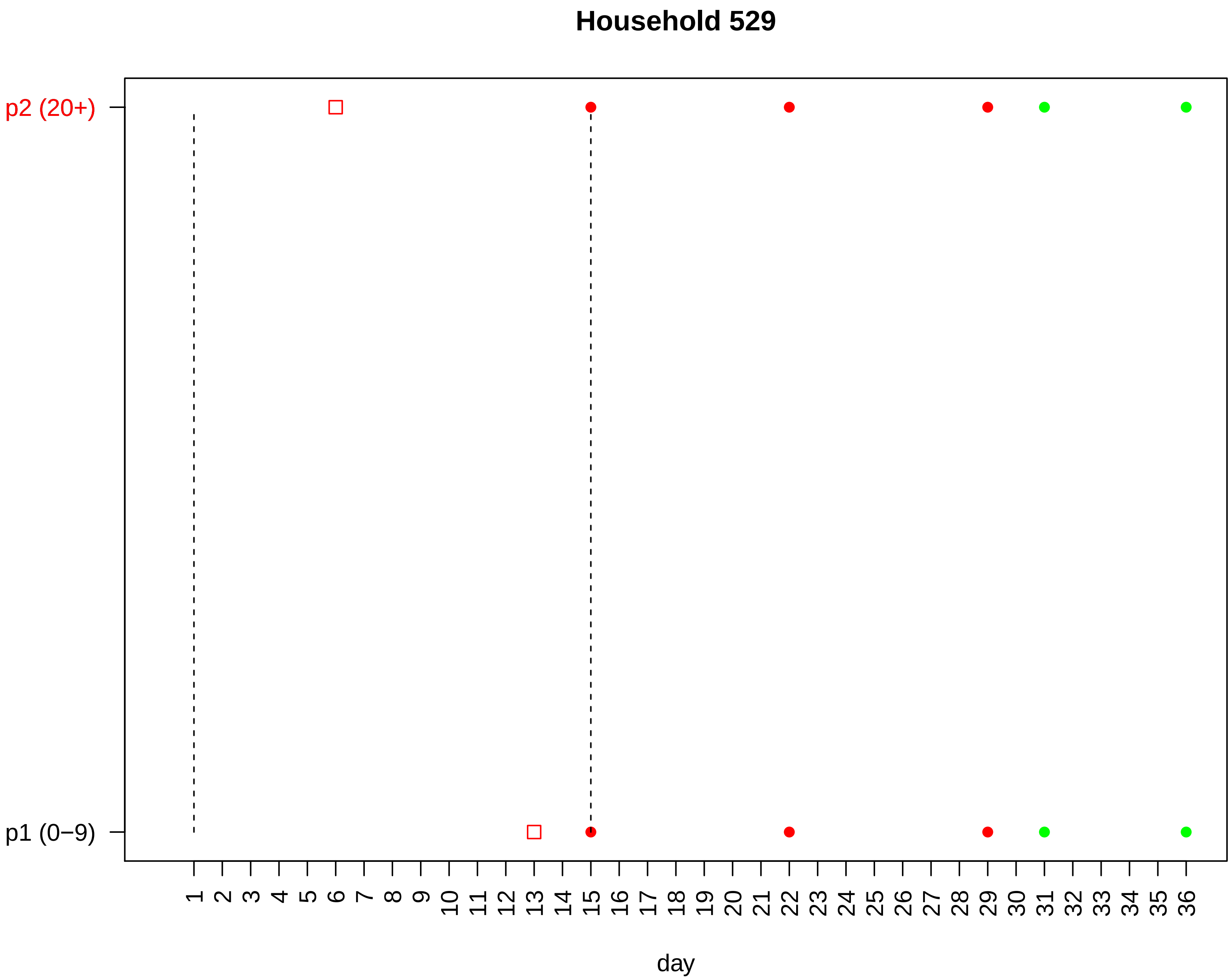

Household 529
p2 (20+)
p1 (0−9)
1 2 3 4 5 6 7 8 9 10 11 12 13 14 15 16 17 18 19 20 21 22 23 24 25 26 27 28 29 30 31 32 33 34 35 36
day

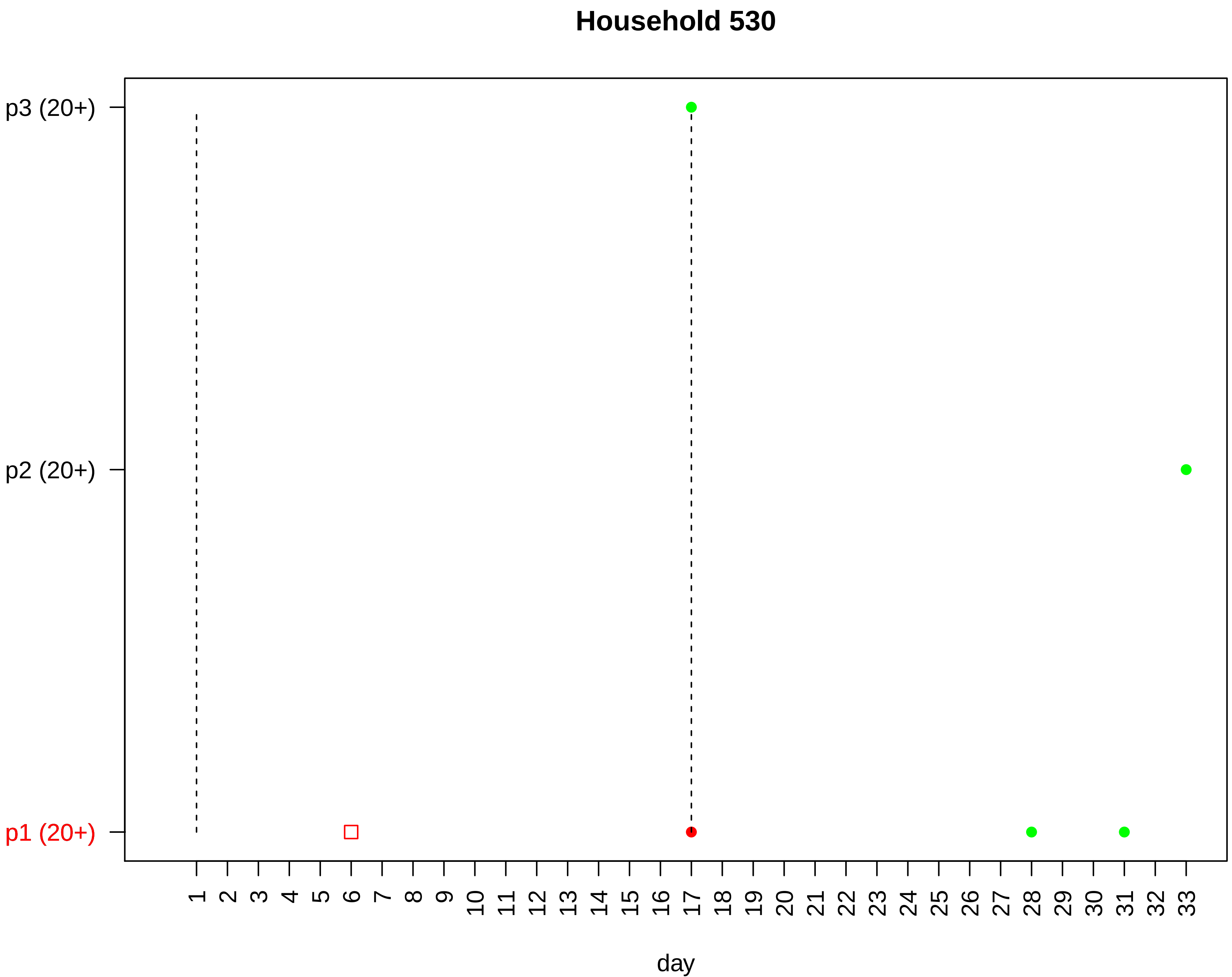
Household 530
p3 (20+)
p2 (20+)
p1 (20+)
1
2
3
4
5
6
7
8
9
10
11
12
13
14
15
16
17
18
19
20
21
22
23
24
25
26
27
28
29
30
31
32
33
day

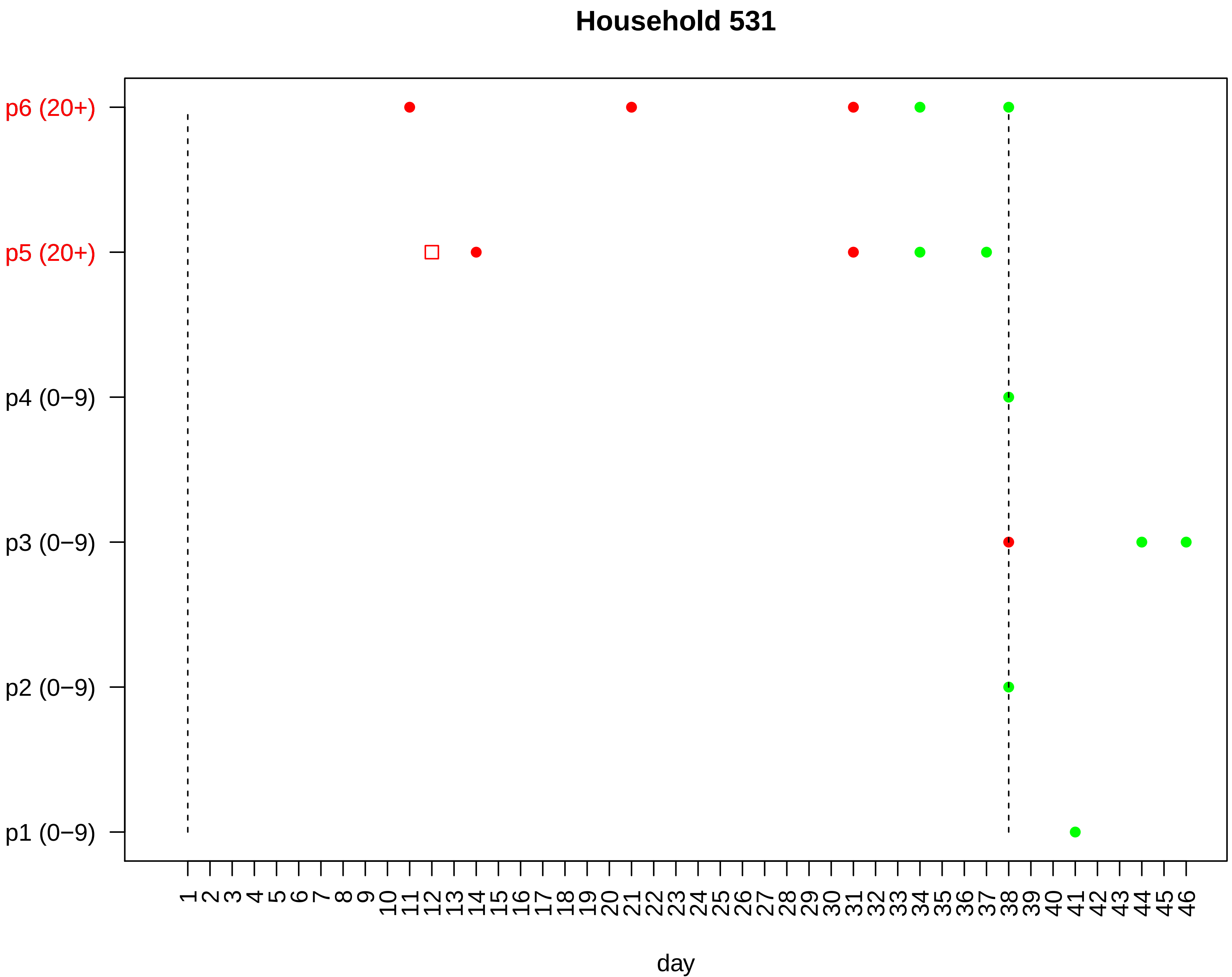
Household 531
p6 (20+)
p5 (20+)
p4 (0−9)
p3 (0−9)
p2 (0−9)
p1 (0−9)
1 2 3 4 5 6 7 8 9 10 11 12 13 14 15 16 17 18 19 20 21 22 23 24 25 26 27 28 29 30 31 32 33 34 35 36 37 38 39 40 41 42 43 44 45 46
day

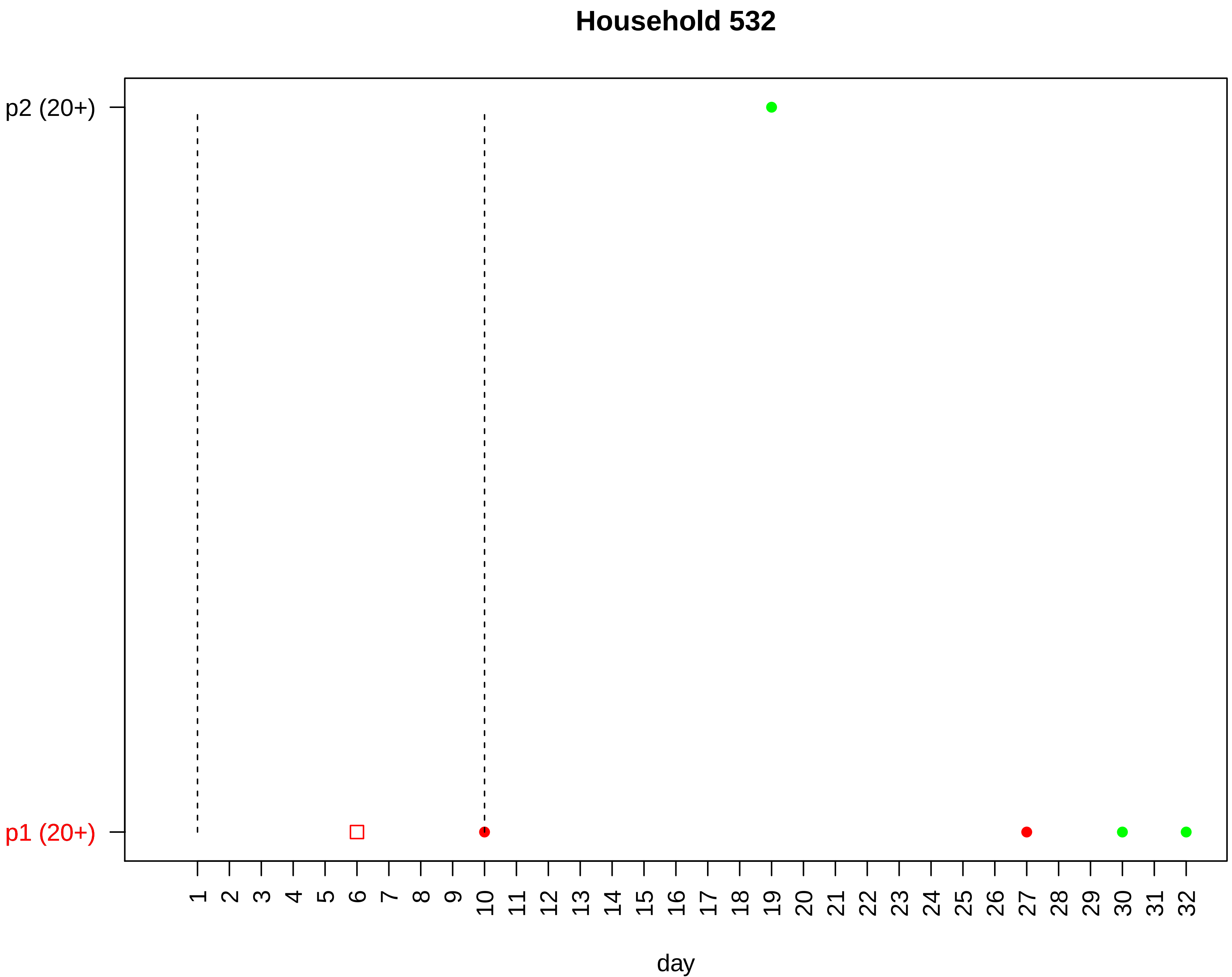

Household 532
p2 (20+)
p1 (20+)
1
2
3
4
5
6
7
8
9
10
11
12
13
14
15
16
17
18
19
20
21
22
23
24
25
26
27
28
29
30
31
32
day

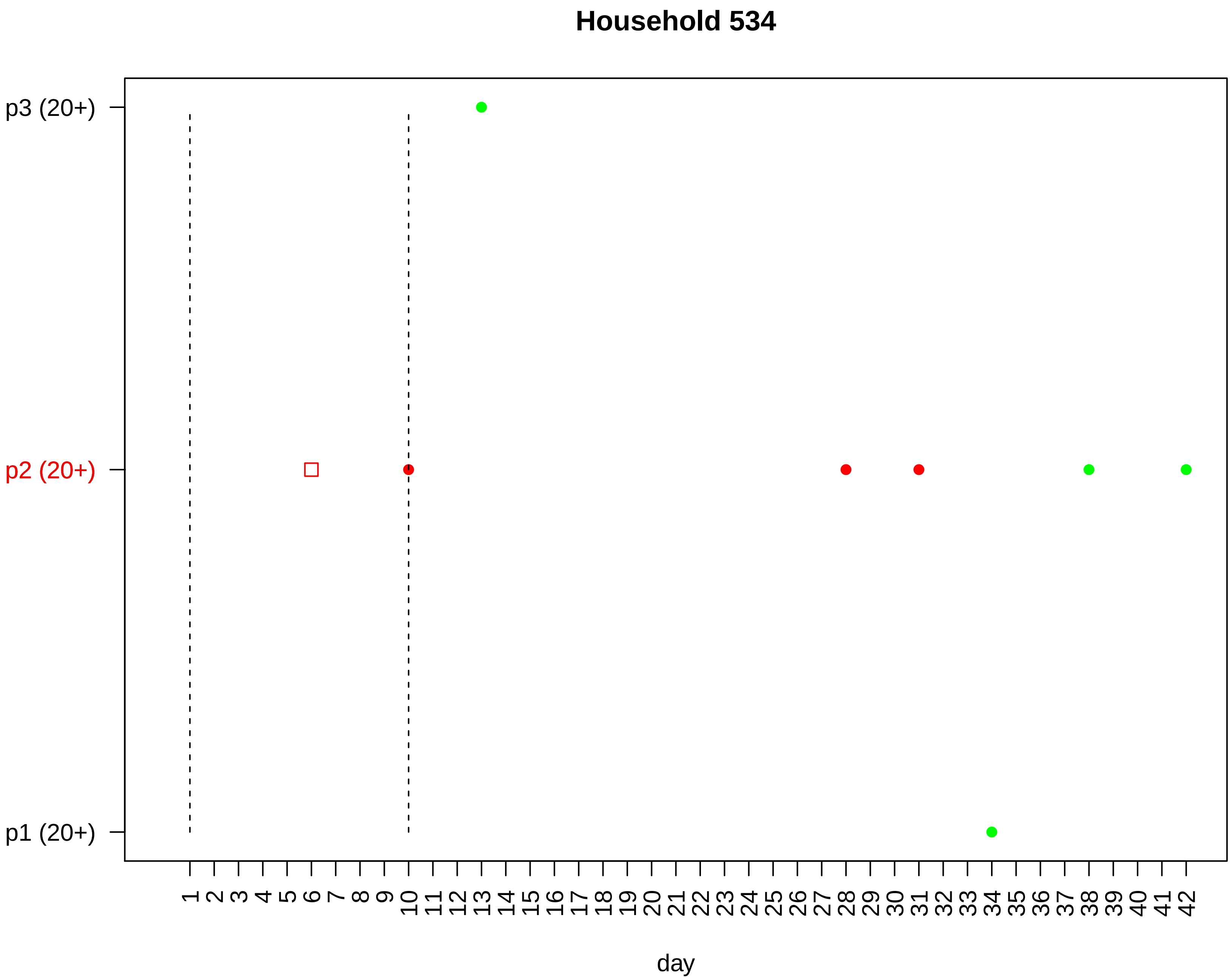

Household 534
p3 (20+)
p2 (20+)
p1 (20+)
1 2 3 4 5 6 7 8 9 10 11 12 13 14 15 16 17 18 19 20 21 22 23 24 25 26 27 28 29 30 31 32 33 34 35 36 37 38 39 40 41 42
day

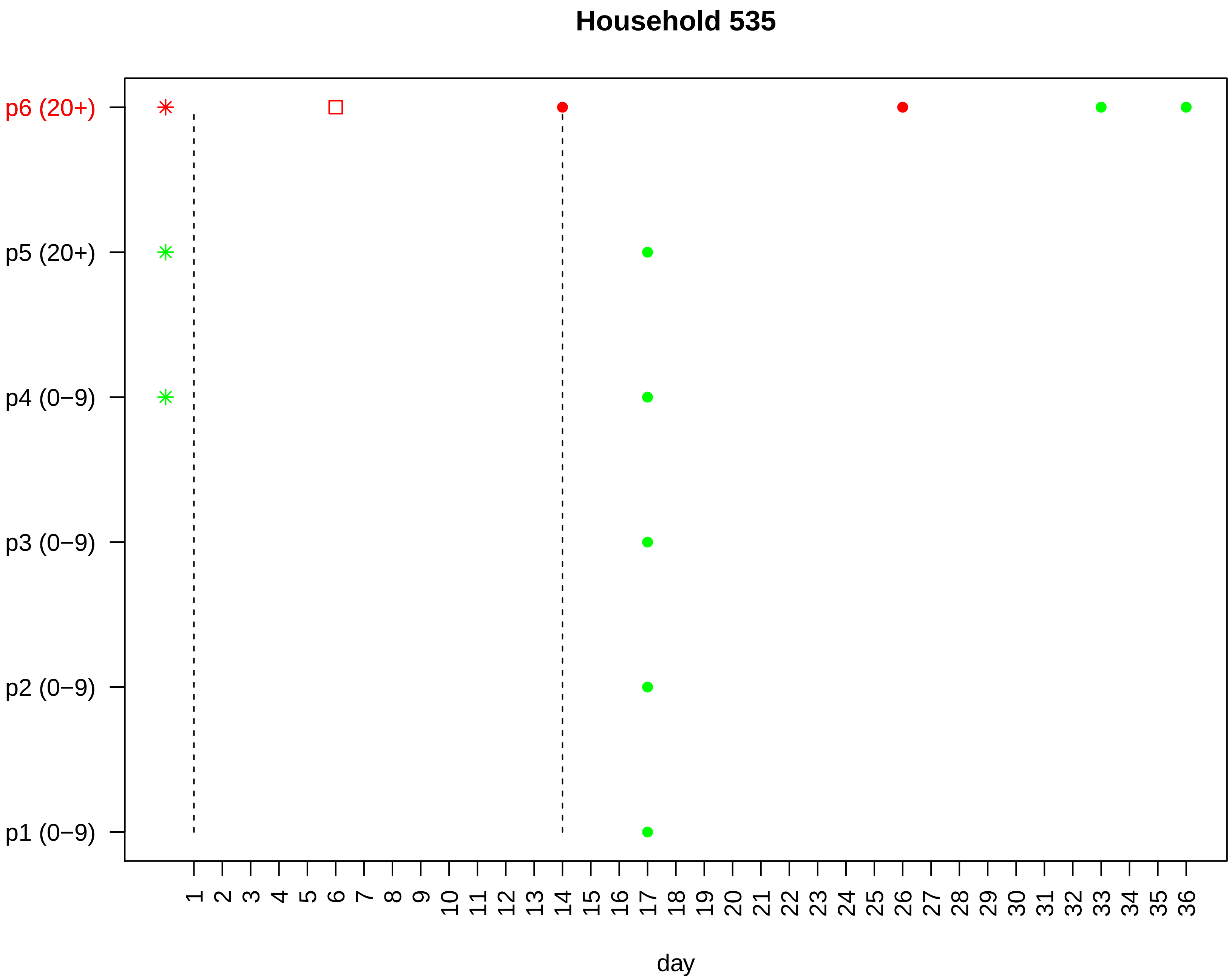

Household 535
p6 (20+)
p5 (20+)
p4 (0−9)
p3 (0−9)
p2 (0−9)
p1 (0−9)
1 2 3 4 5 6 7 8 9 10 11 12 13 14 15 16 17 18 19 20 21 22 23 24 25 26 27 28 29 30 31 32 33 34 35 36
day

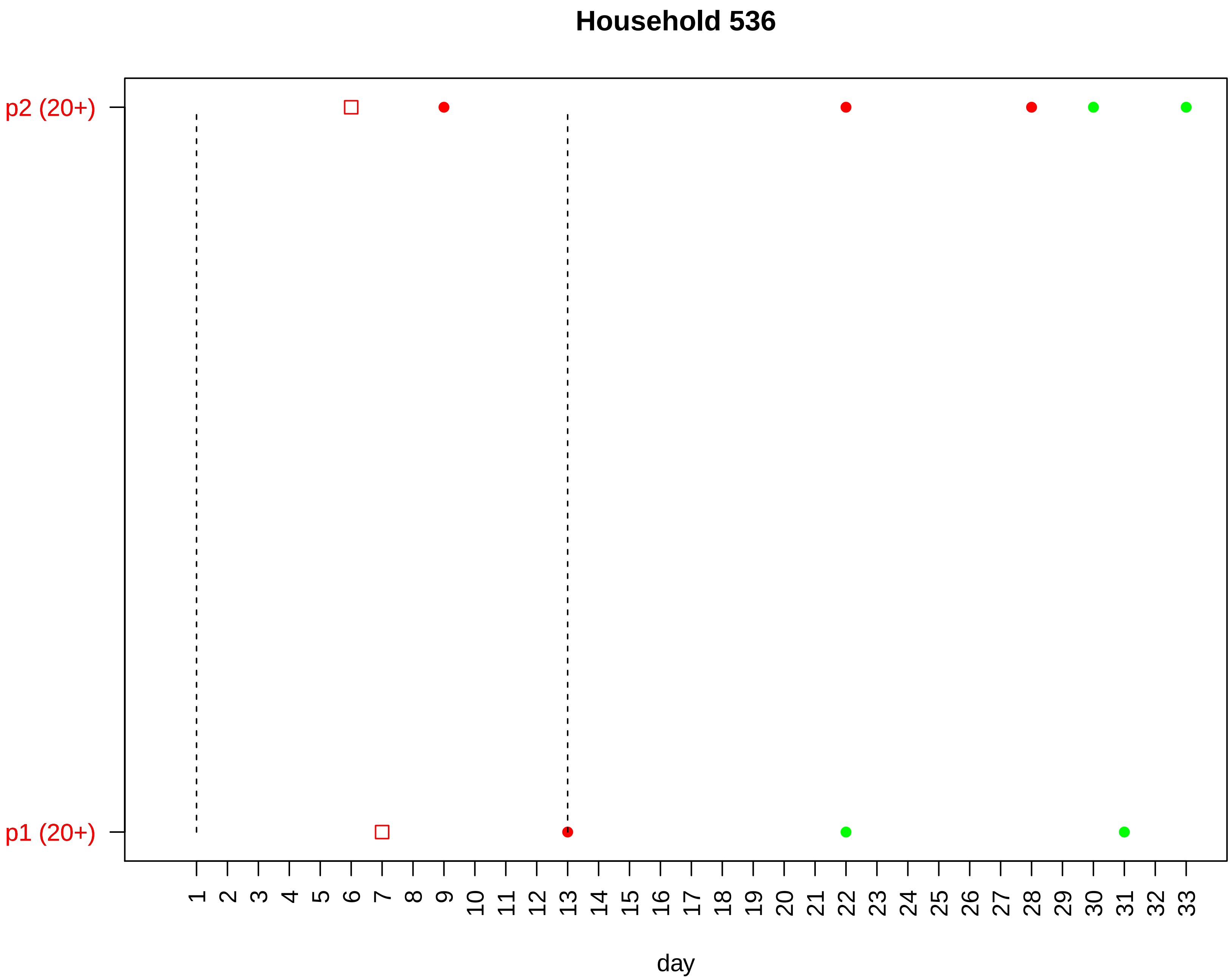

Household 536
p2 (20+)
p1 (20+)
1
2
3
4
5
6
7
8
9
10
11
12
13
14
15
16
17
18
19
20
21
22
23
24
25
26
27
28
29
30
31
32
33
day

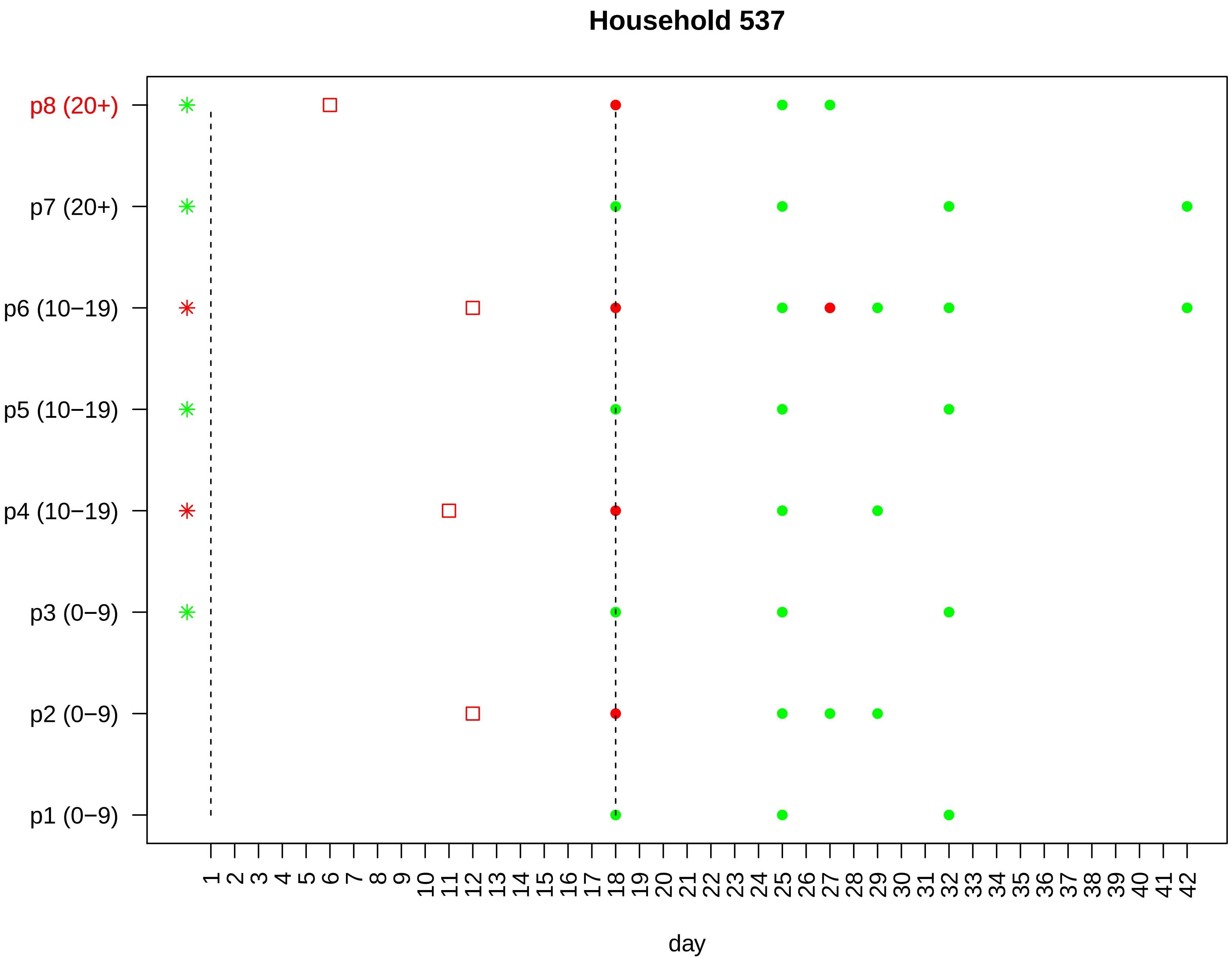

Household 537
p8 (20+)
p7 (20+)
p6 (10−19)
p5 (10−19)
p4 (10−19)
p3 (0−9)
p2 (0−9)
p1 (0−9)
1 2 3 4 5 6 7 8 9 10 11 12 13 14 15 16 17 18 19 20 21 22 23 24 25 26 27 28 29 30 31 32 33 34 35 36 37 38 39 40 41 42
day

Household 538

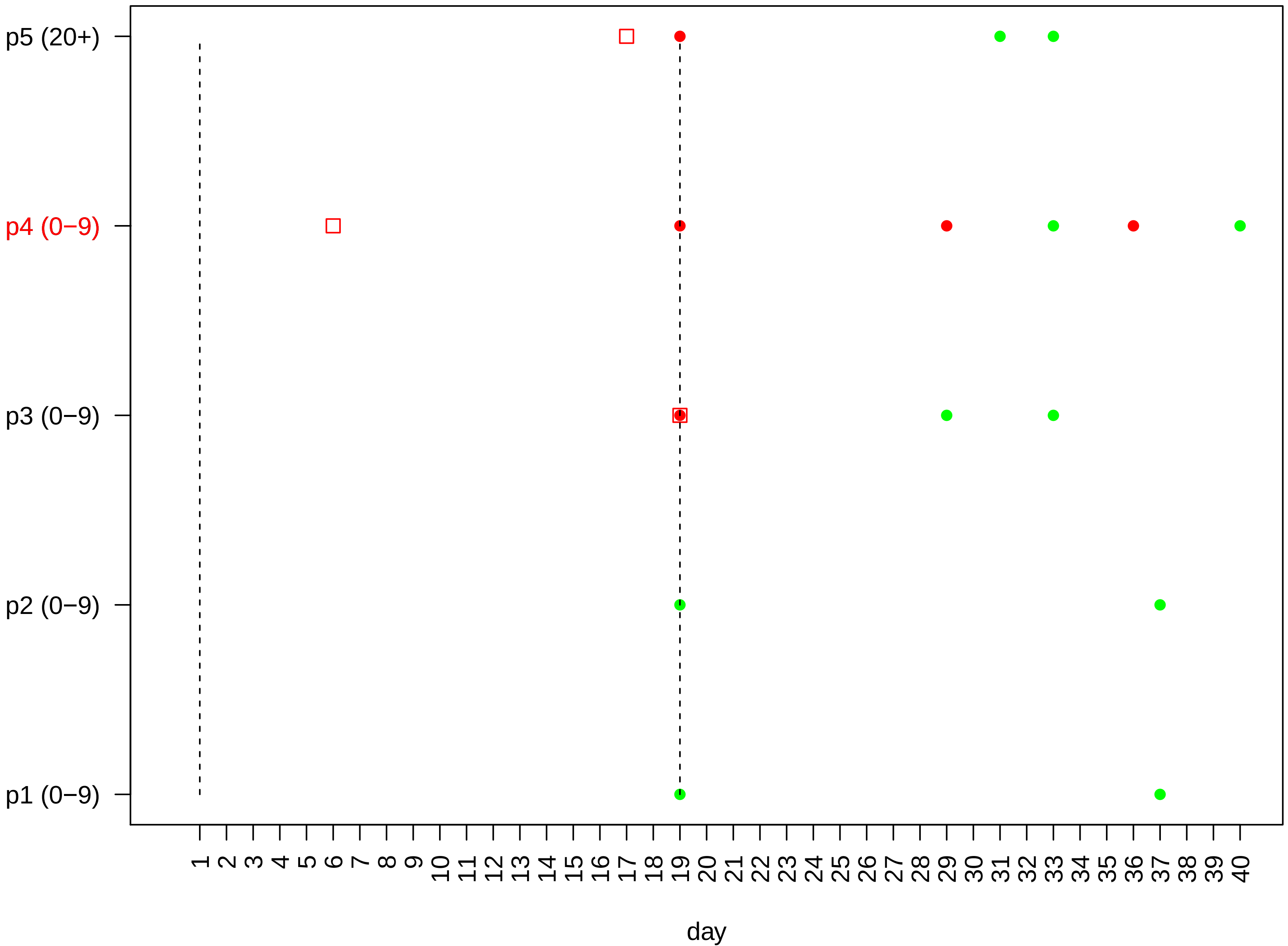

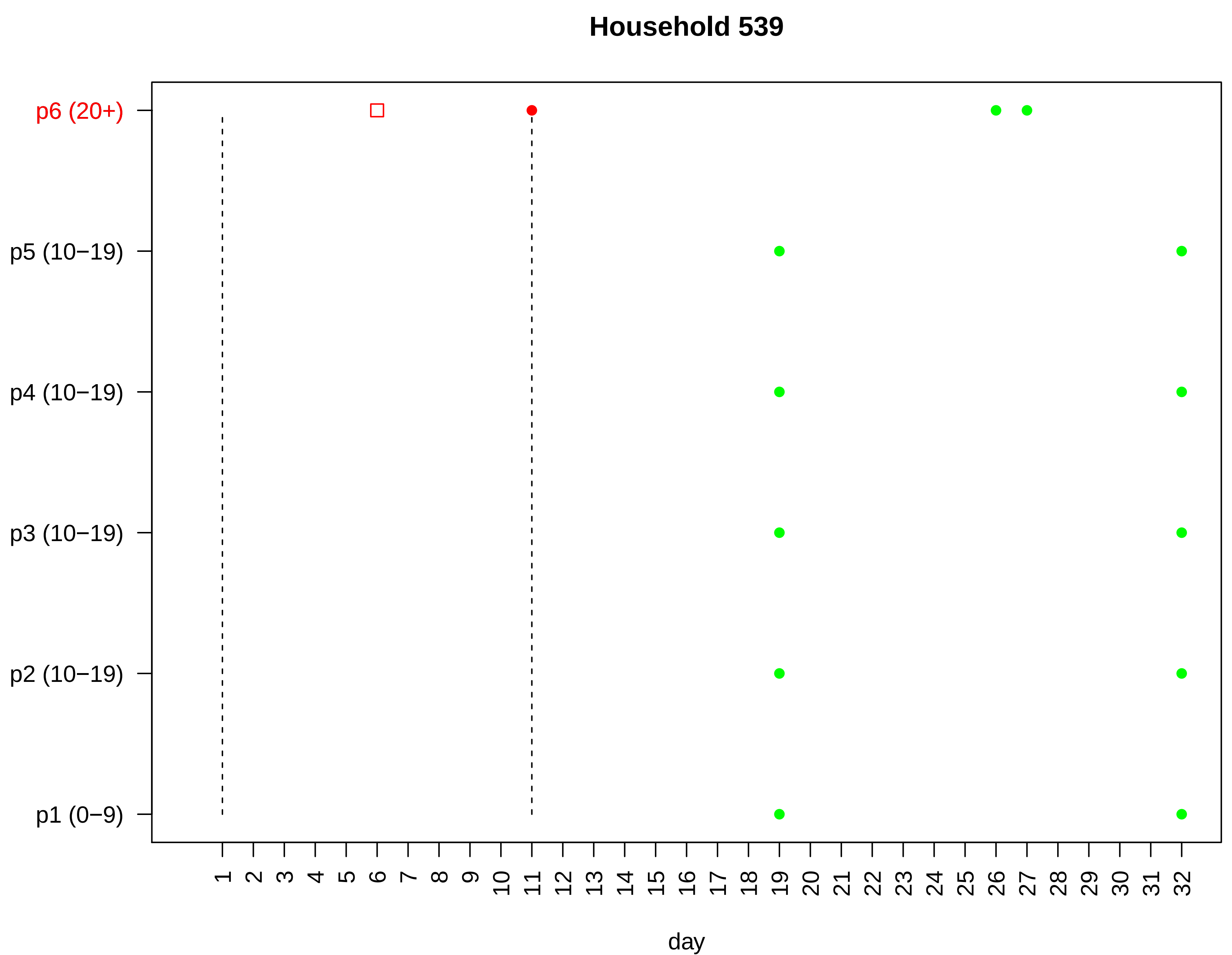

Household 539
p6 (20+)
p5 (10−19)
p4 (10−19)
p3 (10−19)
p2 (10−19)
p1 (0−9)
1 2 3 4 5 6 7 8 9 10 11 12 13 14 15 16 17 18 19 20 21 22 23 24 25 26 27 28 29 30 31 32
day

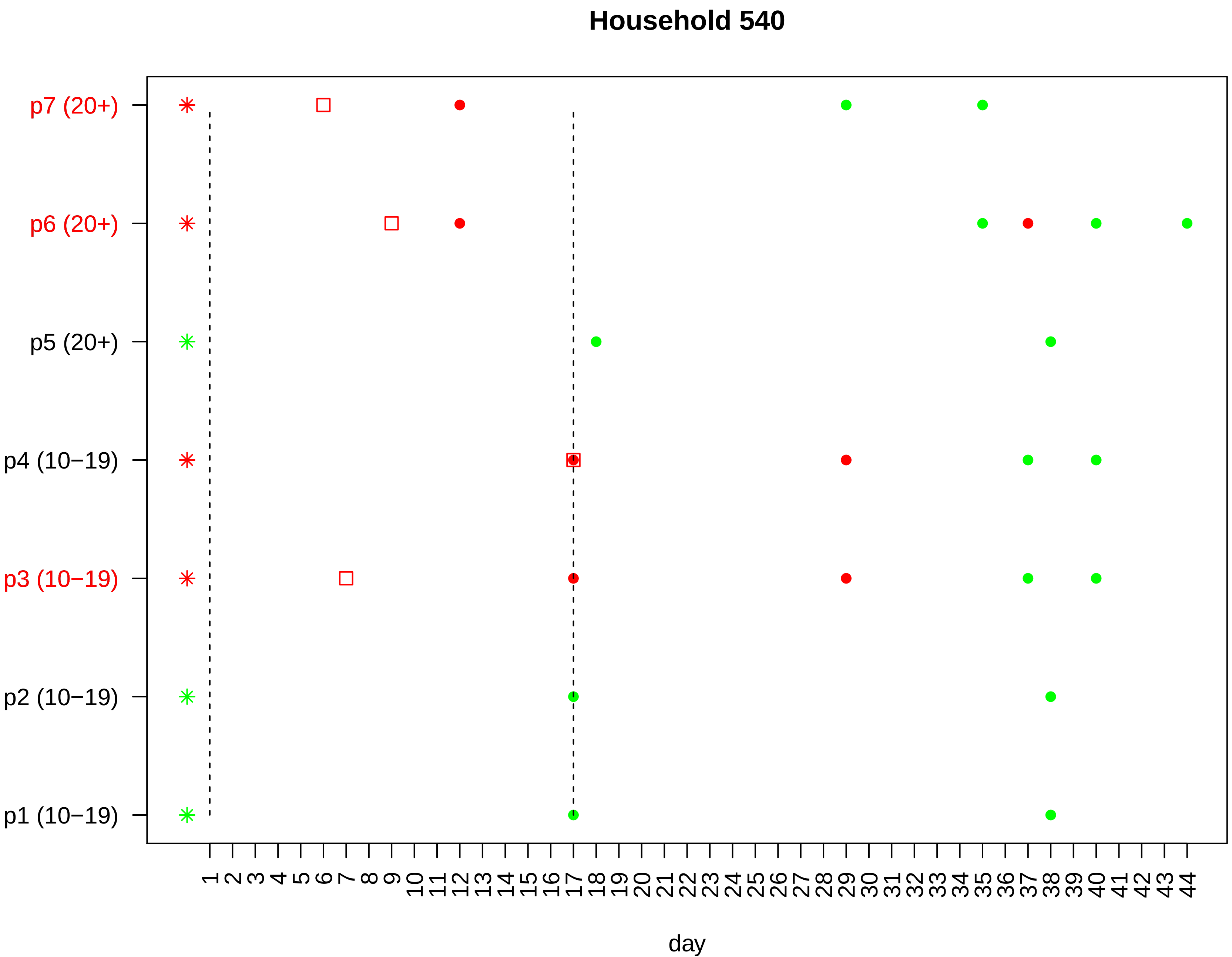

Household 540
p7 (20+)
p6 (20+)
p5 (20+)
p4 (10−19)
p3 (10−19)
p2 (10−19)
p1 (10−19)
1 2 3 4 5 6 7 8 9 10 11 12 13 14 15 16 17 18 19 20 21 22 23 24 25 26 27 28 29 30 31 32 33 34 35 36 37 38 39 40 41 42 43 44
day

Household 541

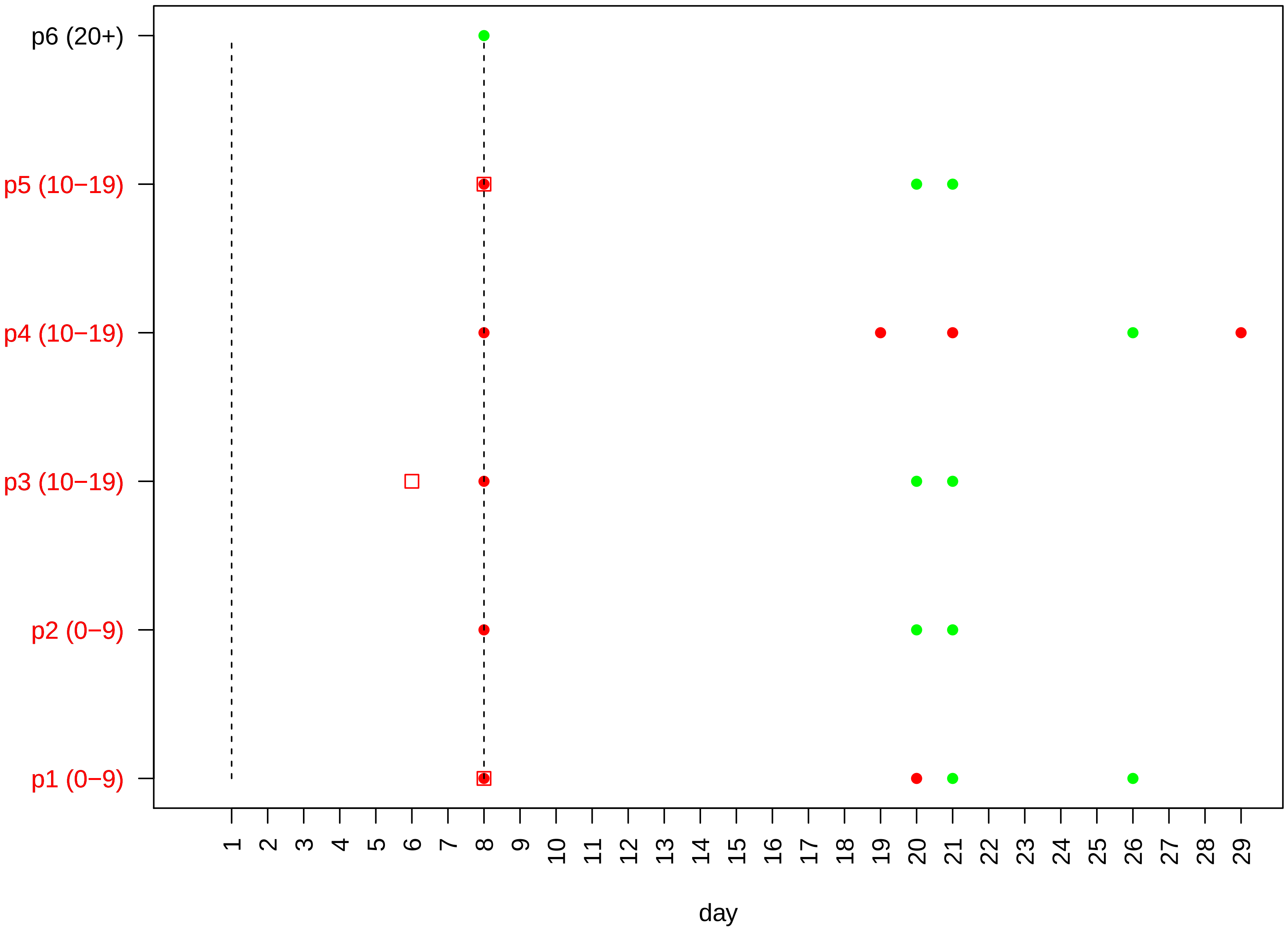

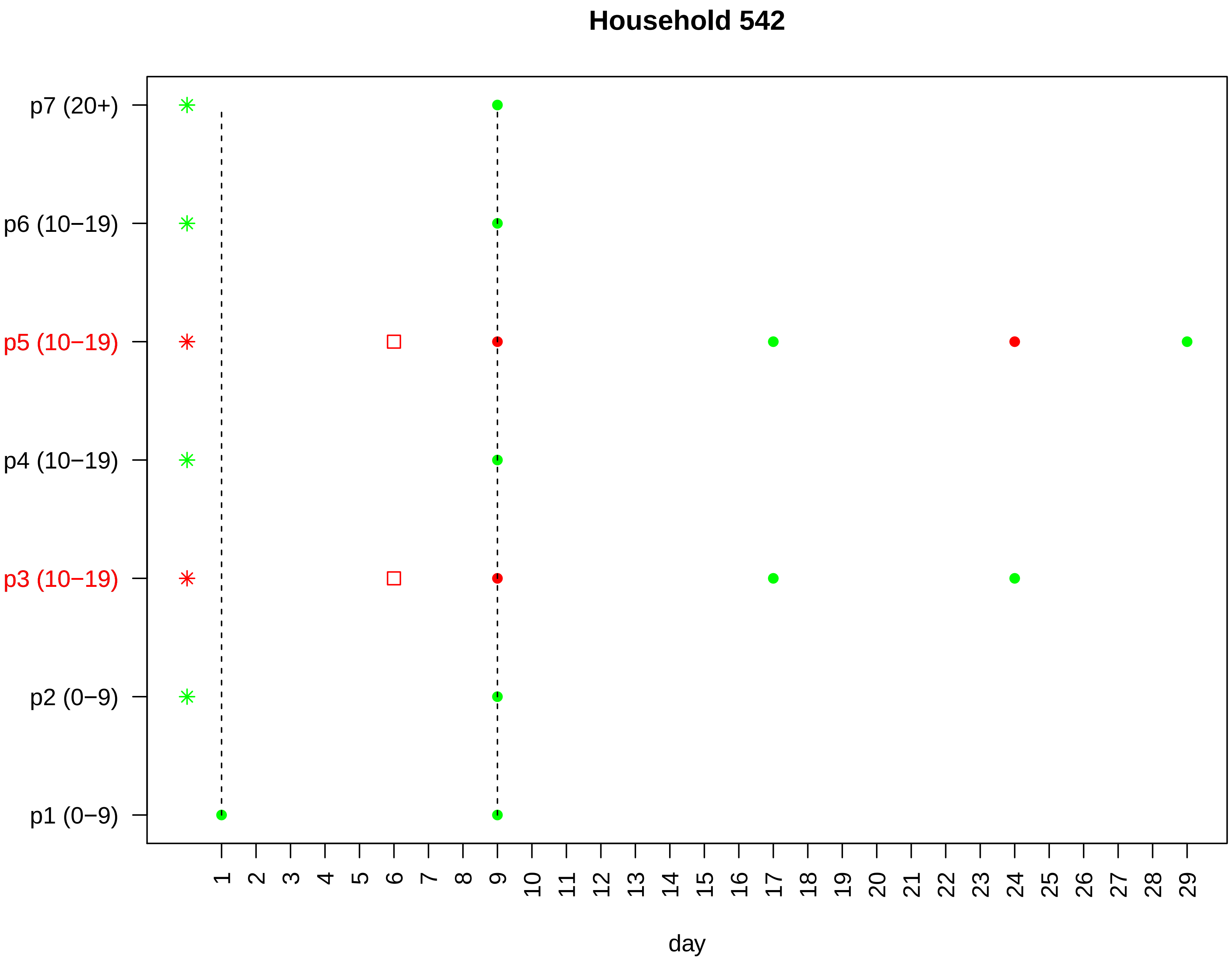

Household 542
p7 (20+)
p6 (10−19)
p5 (10−19)
p4 (10−19)
p3 (10−19)
p2 (0−9)
p1 (0−9)
1 2 3 4 5 6 7 8 9 10 11 12 13 14 15 16 17 18 19 20 21 22 23 24 25 26 27 28 29
day

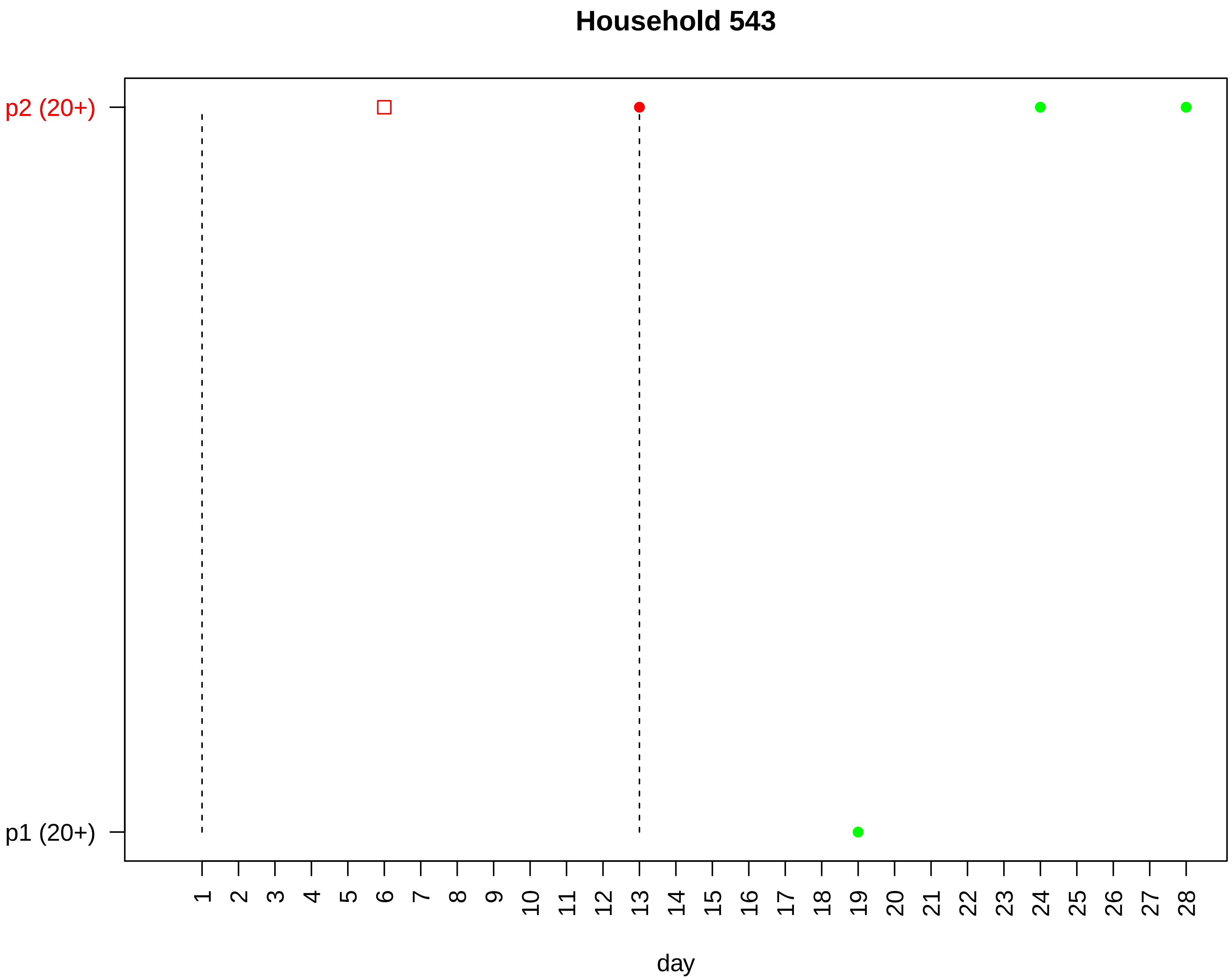

Household 543
p2 (20+)
p1 (20+)
1
2
3
4
5
6
7
8
9
10
11
12
13
14
15
16
17
18
19
20
21
22
23
24
25
26
27
28
day

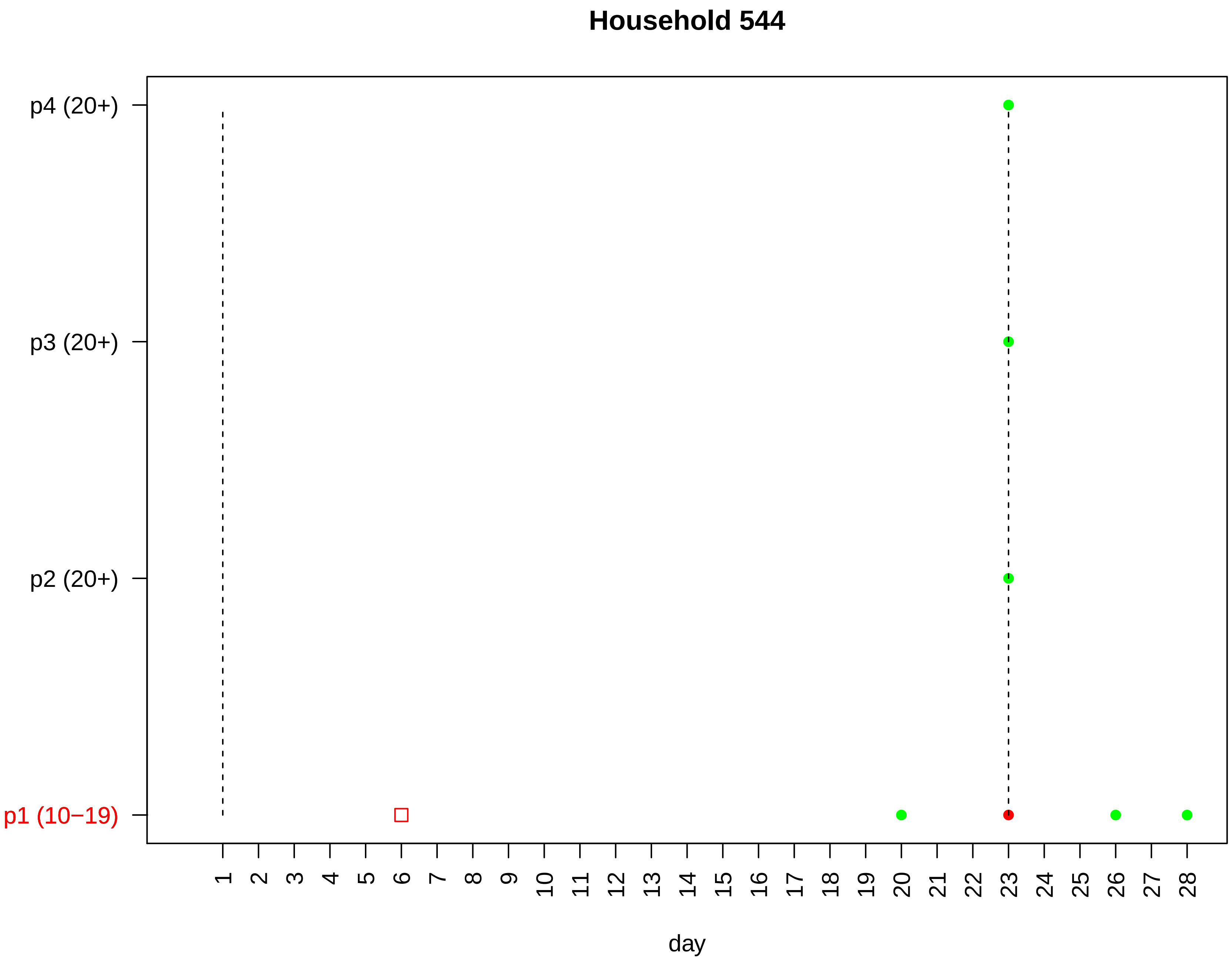

Household 544
p4 (20+)
p3 (20+)
p2 (20+)
p1 (10−19)
1
2
3
4
5
6
7
8
9
10
11
12
13
14
15
16
17
18
19
20
21
22
23
24
25
26
27
28
day

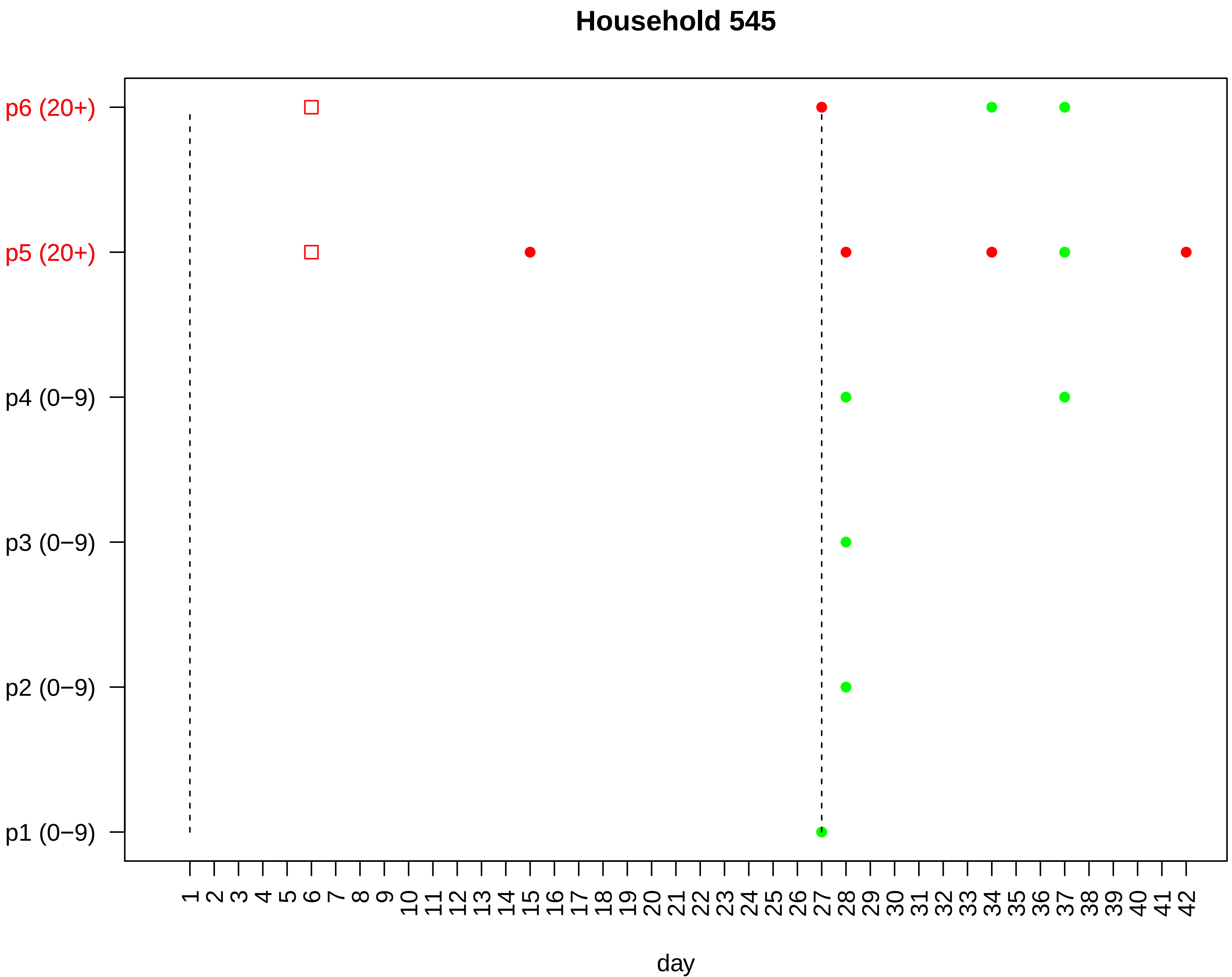

Household 545
p6 (20+)
p5 (20+)
p4 (0−9)
p3 (0−9)
p2 (0−9)
p1 (0−9)
1 2 3 4 5 6 7 8 9 10 11 12 13 14 15 16 17 18 19 20 21 22 23 24 25 26 27 28 29 30 31 32 33 34 35 36 37 38 39 40 41 42
day

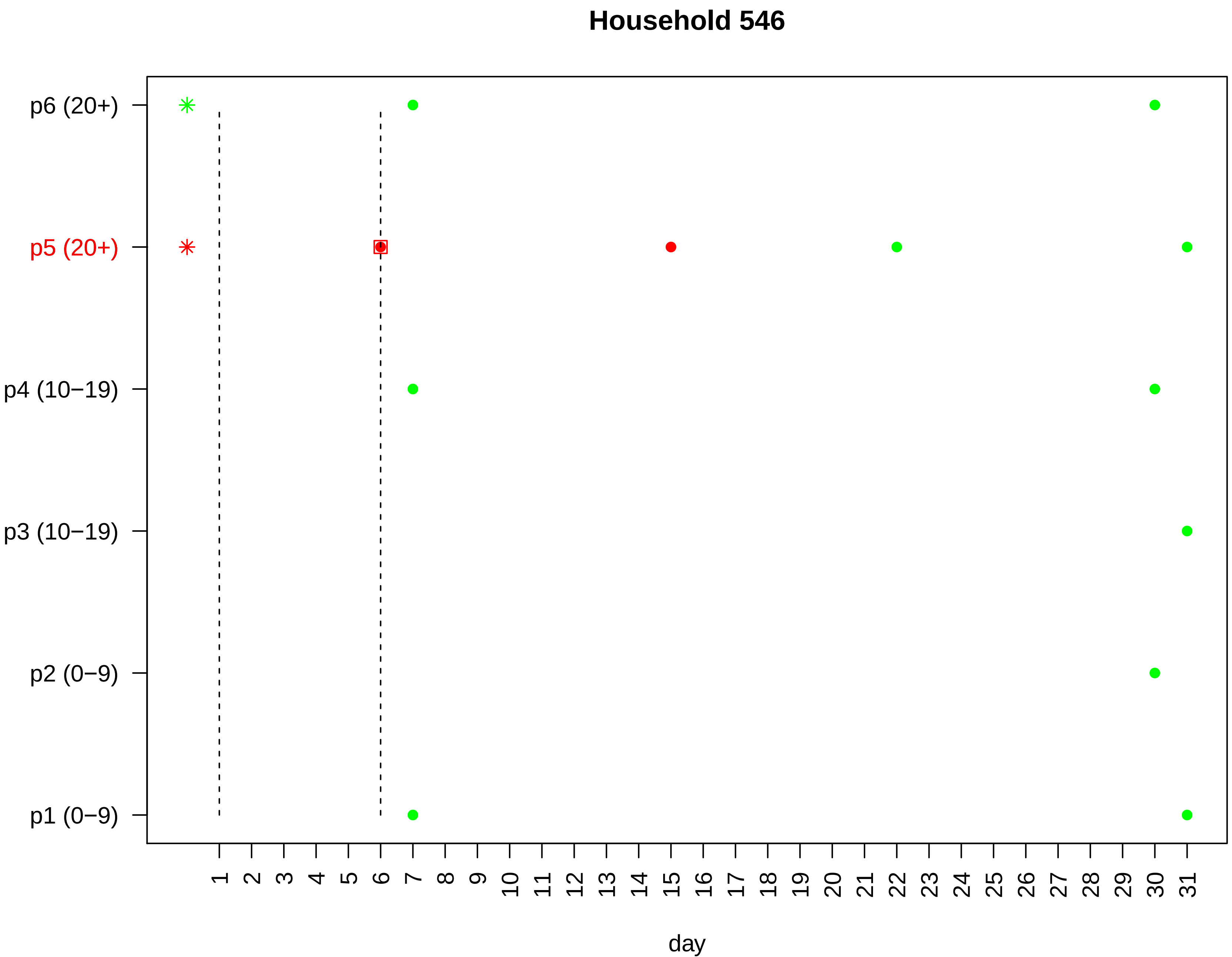

Household 546
p6 (20+)
p5 (20+)
p4 (10−19)
p3 (10−19)
p2 (0−9)
p1 (0−9)
1 2 3 4 5 6 7 8 9 10 11 12 13 14 15 16 17 18 19 20 21 22 23 24 25 26 27 28 29 30 31
day

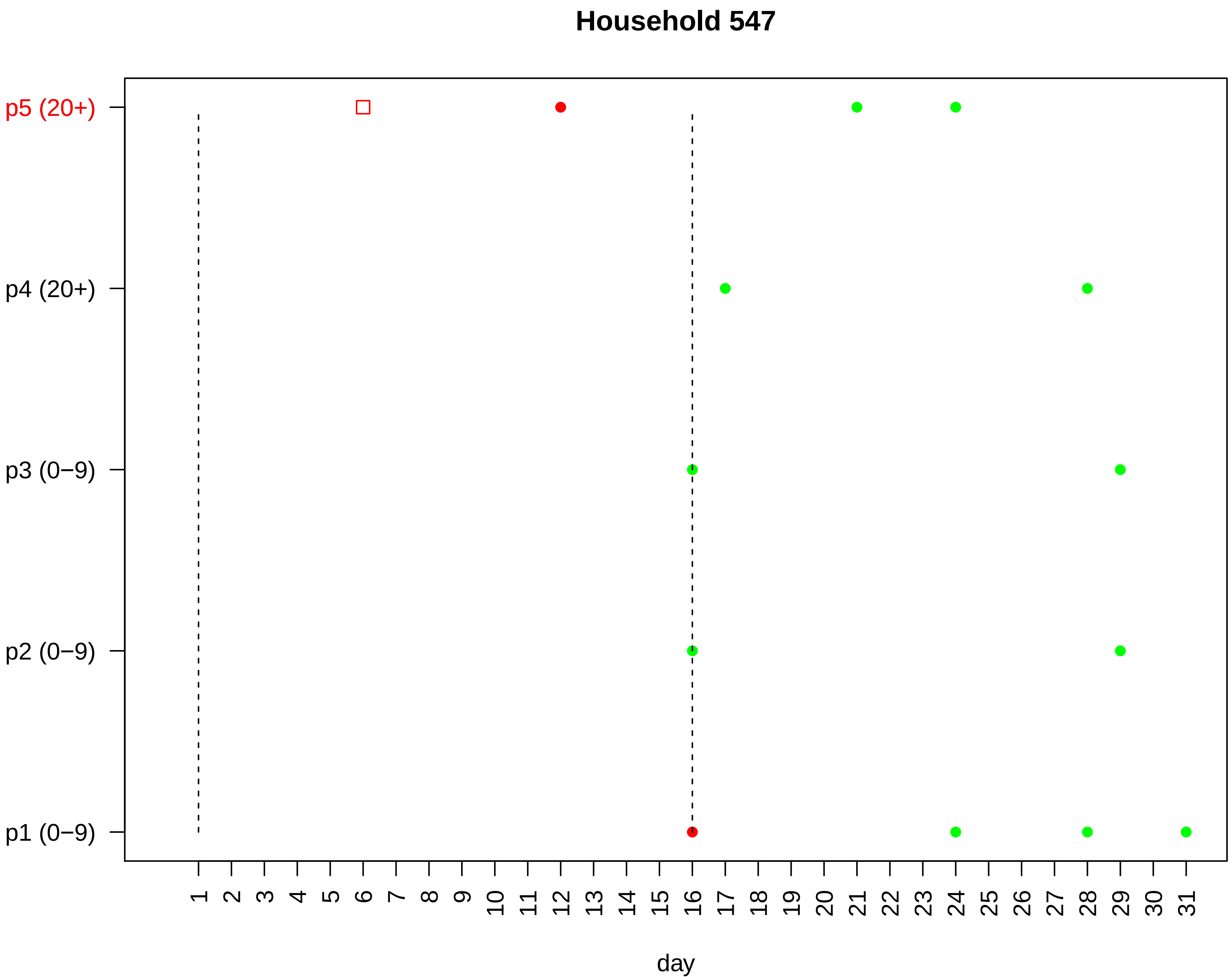

Household 547
p5 (20+)
p4 (20+)
p3 (0−9)
p2 (0−9)
p1 (0−9)
1 2 3 4 5 6 7 8 9 10 11 12 13 14 15 16 17 18 19 20 21 22 23 24 25 26 27 28 29 30 31
day

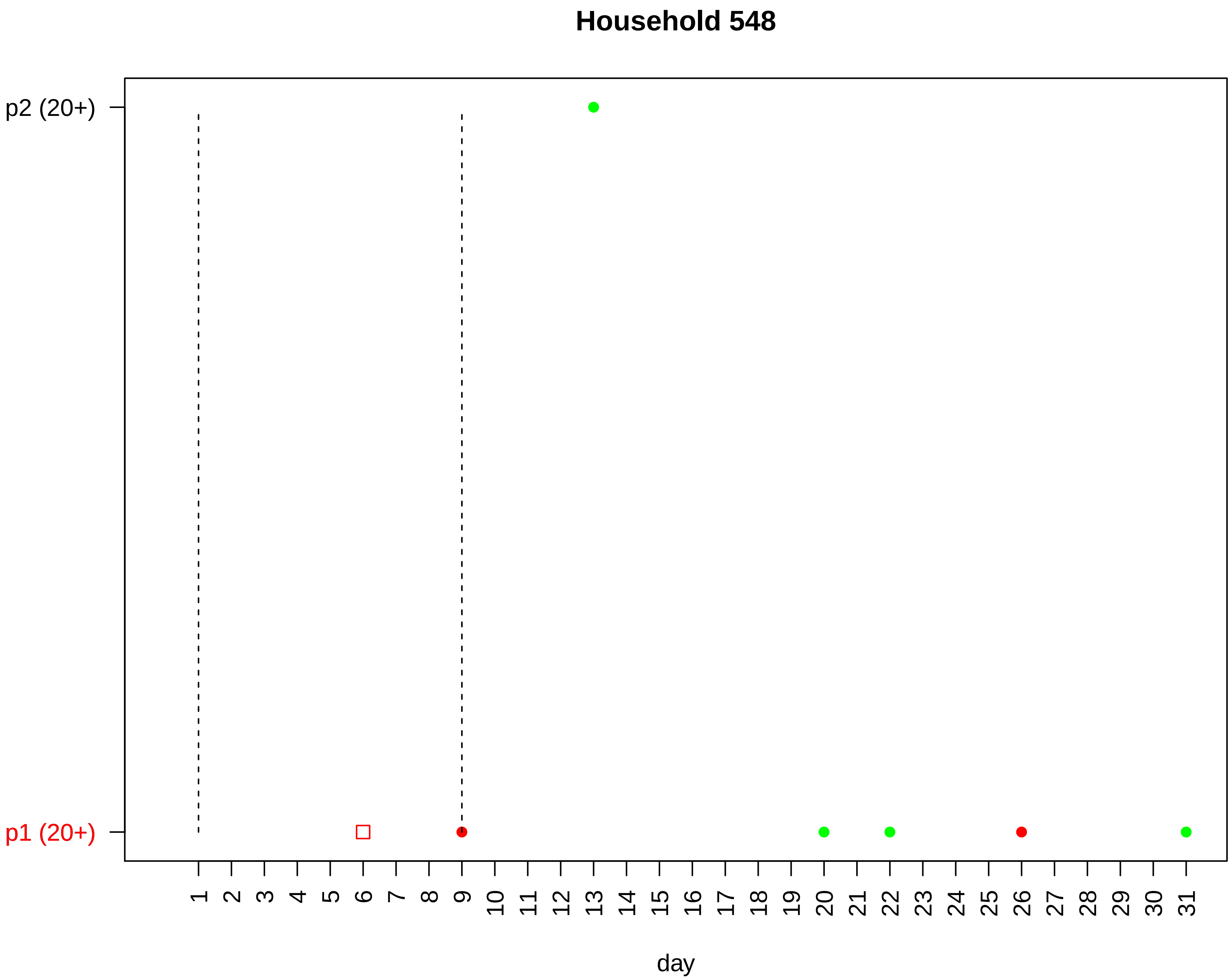

Household 548
p2 (20+)
p1 (20+)
1
2
3
4
5
6
7
8
9
10
11
12
13
14
15
16
17
18
19
20
21
22
23
24
25
26
27
28
29
30
31
day

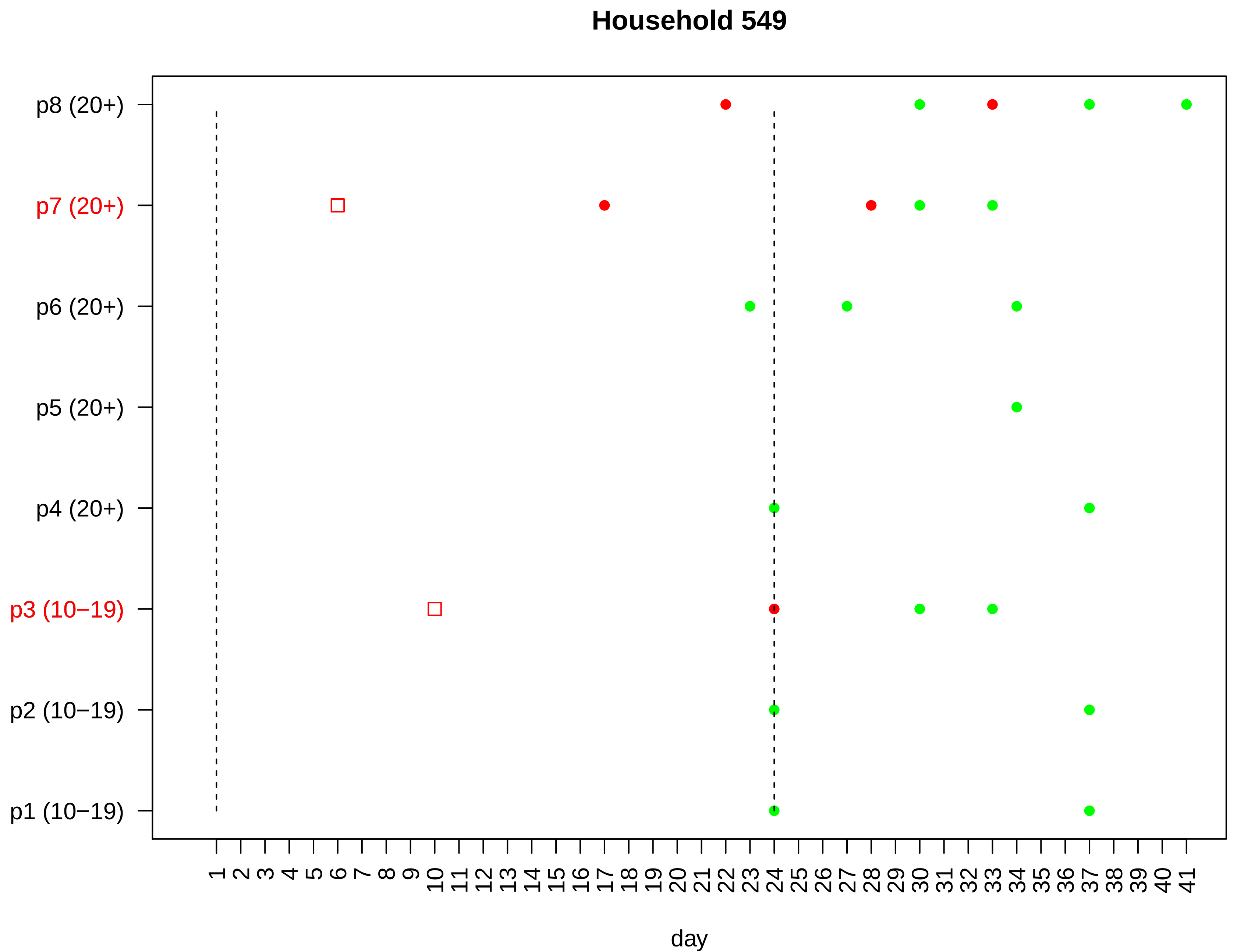

Household 549
p8 (20+)
p7 (20+)
p6 (20+)
p5 (20+)
p4 (20+)
p3 (10−19)
p2 (10−19)
p1 (10−19)
1 2 3 4 5 6 7 8 9 10 11 12 13 14 15 16 17 18 19 20 21 22 23 24 25 26 27 28 29 30 31 32 33 34 35 36 37 38 39 40 41
day

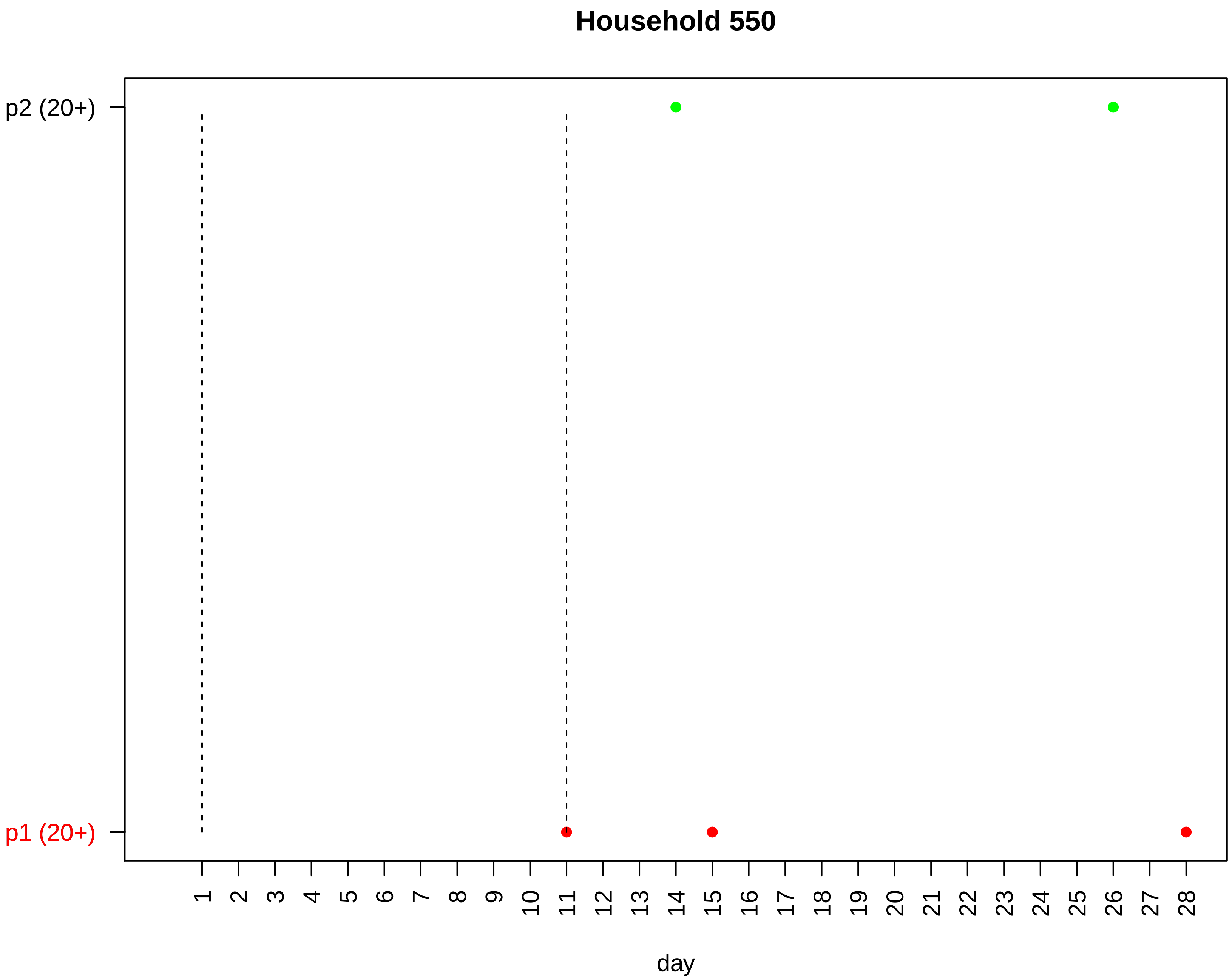
Household 550
p2 (20+)
p1 (20+)
1 2 3 4 5 6 7 8 9 10 11 12 13 14 15 16 17 18 19 20 21 22 23 24 25 26 27 28
day

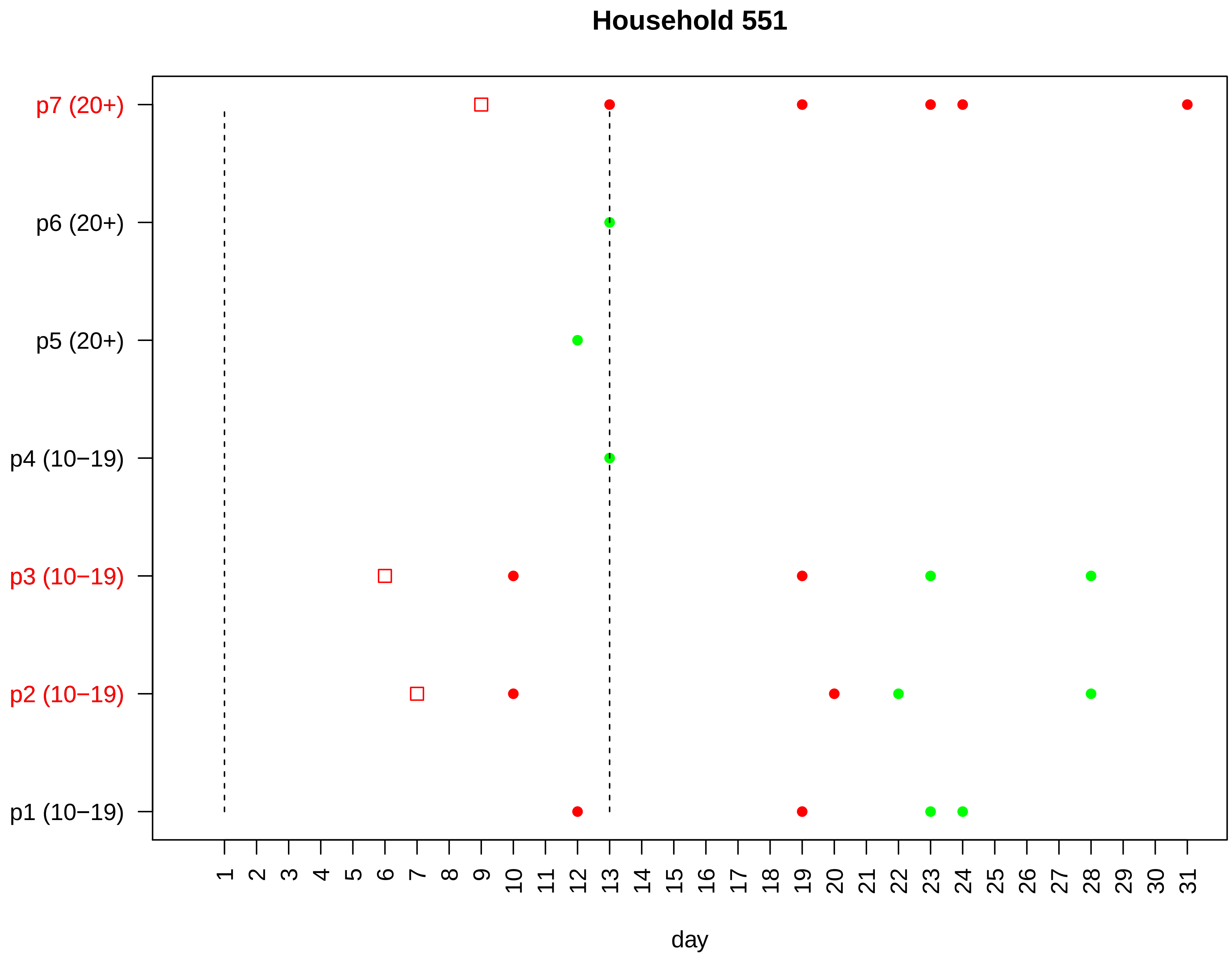

Household 551
p7 (20+)
p6 (20+)
p5 (20+)
p4 (10−19)
p3 (10−19)
p2 (10−19)
p1 (10−19)
1 2 3 4 5 6 7 8 9 10 11 12 13 14 15 16 17 18 19 20 21 22 23 24 25 26 27 28 29 30 31
day

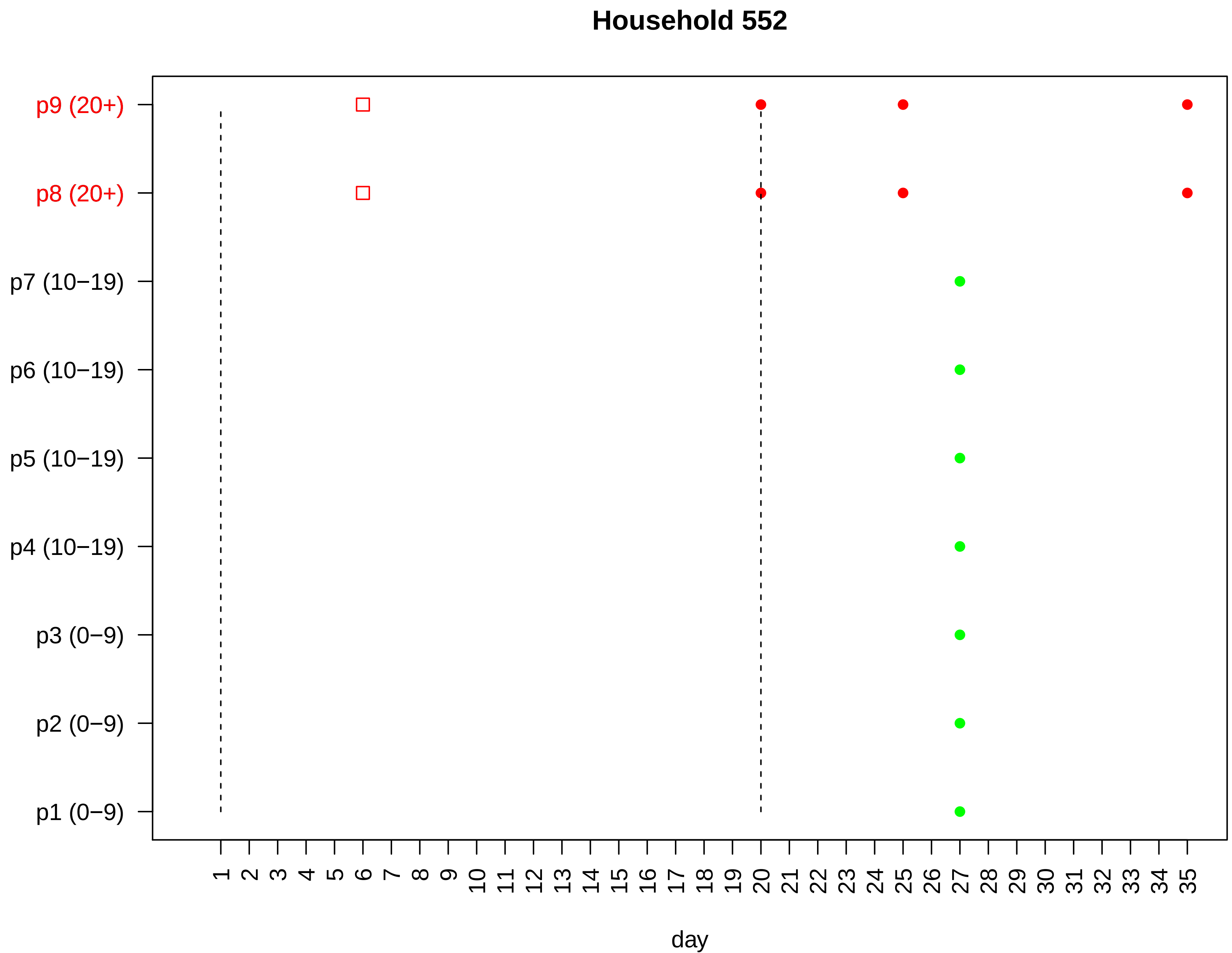

Household 552
p9 (20+)
p8 (20+)
p7 (10−19)
p6 (10−19)
p5 (10−19)
p4 (10−19)
p3 (0−9)
p2 (0−9)
p1 (0−9)
1 2 3 4 5 6 7 8 9 10 11 12 13 14 15 16 17 18 19 20 21 22 23 24 25 26 27 28 29 30 31 32 33 34 35
day

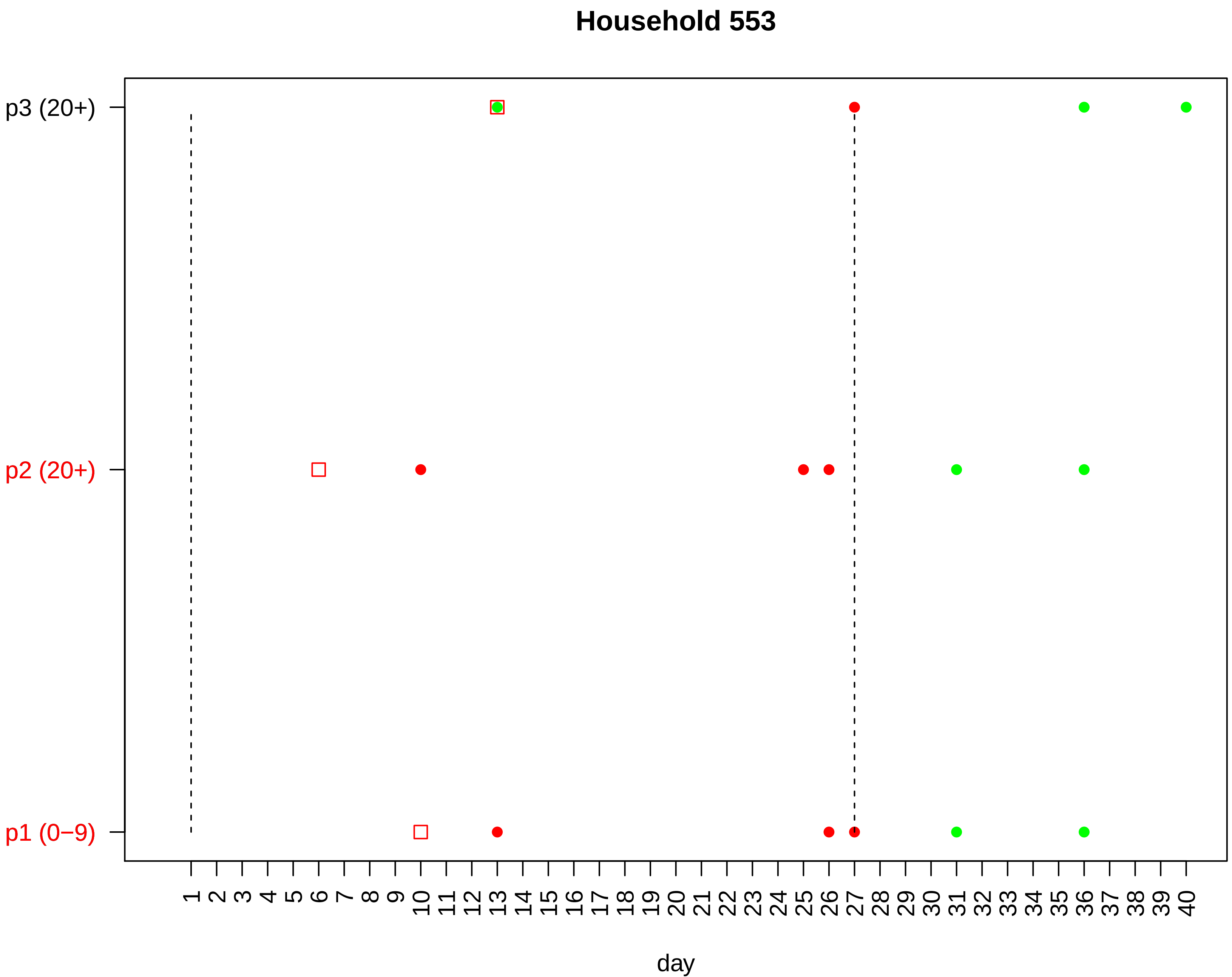

Household 553
p3 (20+)
p2 (20+)
p1 (0−9)
1 2 3 4 5 6 7 8 9 10 11 12 13 14 15 16 17 18 19 20 21 22 23 24 25 26 27 28 29 30 31 32 33 34 35 36 37 38 39 40
day

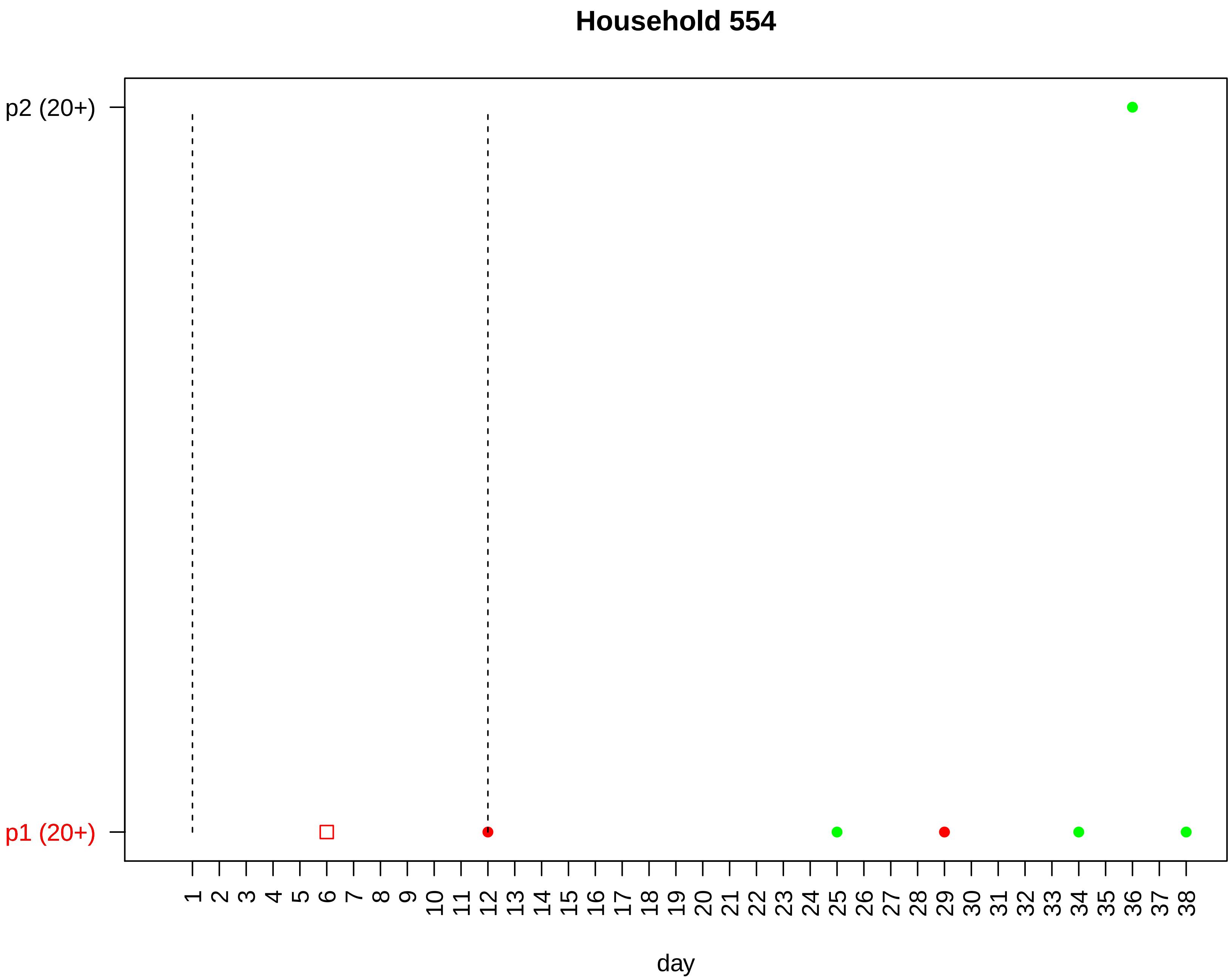

Household 554
p2 (20+)
p1 (20+)
1 2 3 4 5 6 7 8 9 10 11 12 13 14 15 16 17 18 19 20 21 22 23 24 25 26 27 28 29 30 31 32 33 34 35 36 37 38
day

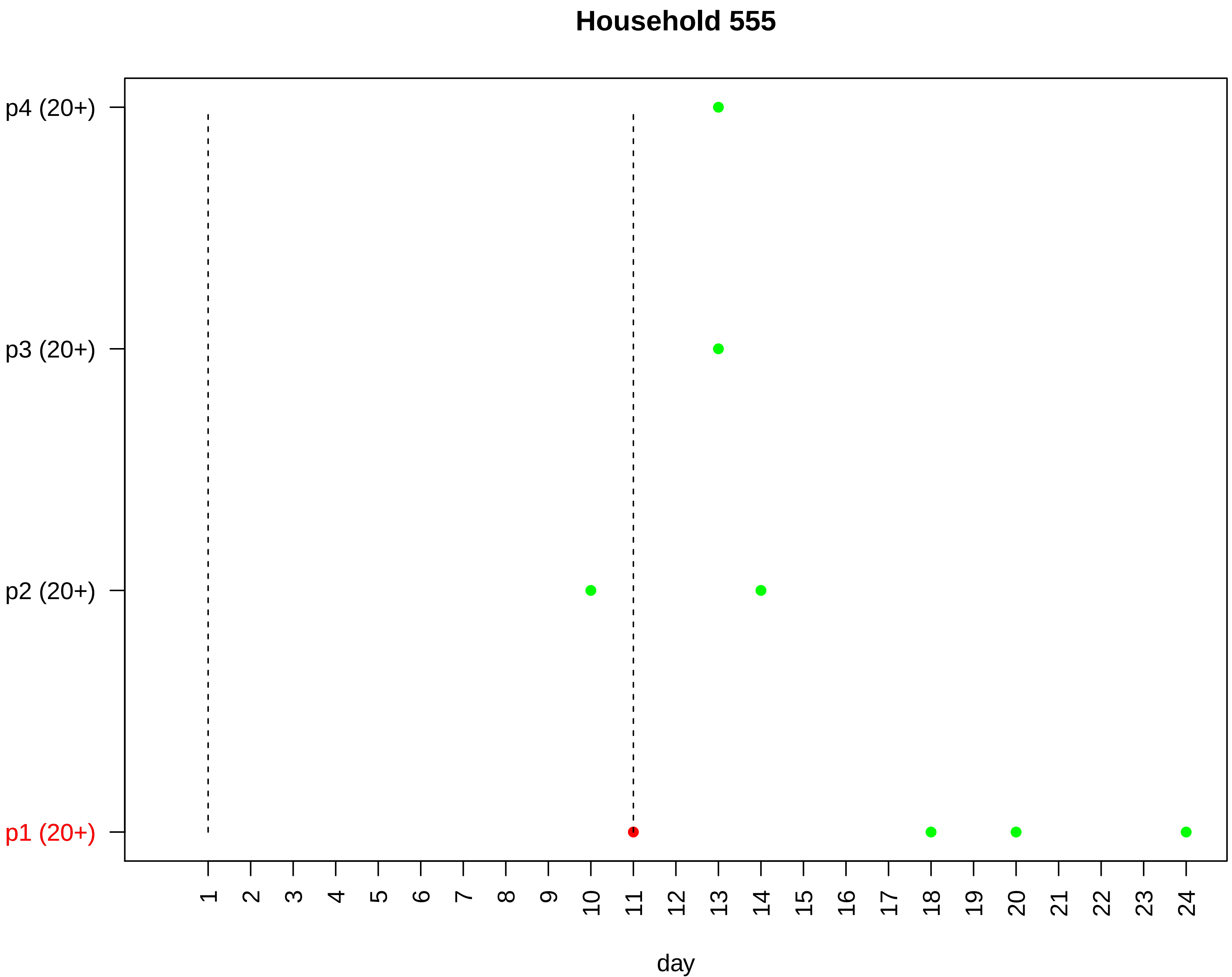

Household 555
p4 (20+)
p3 (20+)
p2 (20+)
p1 (20+)
1
2
3
4
5
6
7
8
9
10
11
12
13
14
15
16
17
18
19
20
21
22
23
24
day

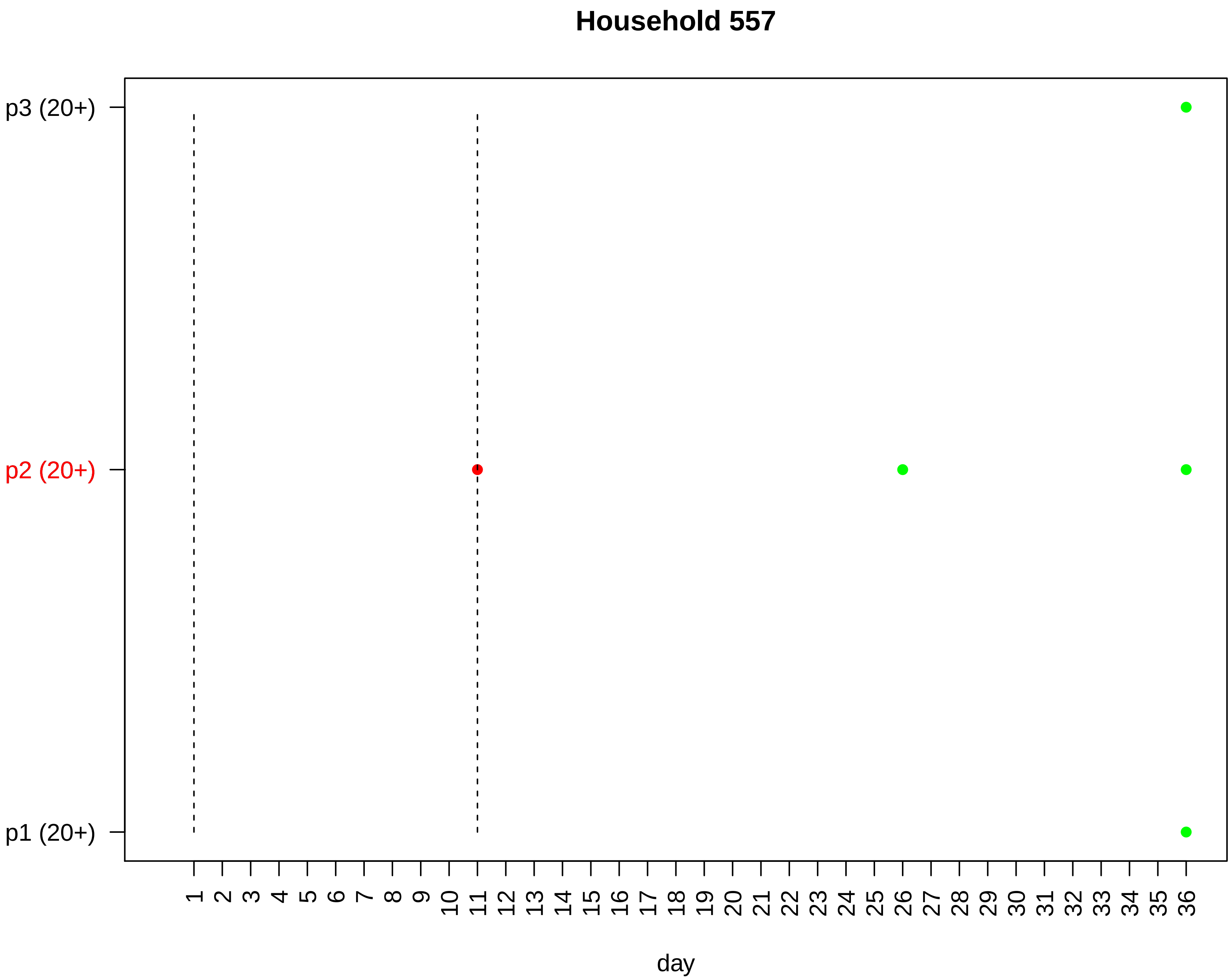
Household 557
p3 (20+)
p2 (20+)
p1 (20+)
1 2 3 4 5 6 7 8 9 10 11 12 13 14 15 16 17 18 19 20 21 22 23 24 25 26 27 28 29 30 31 32 33 34 35 36
day

Household 558

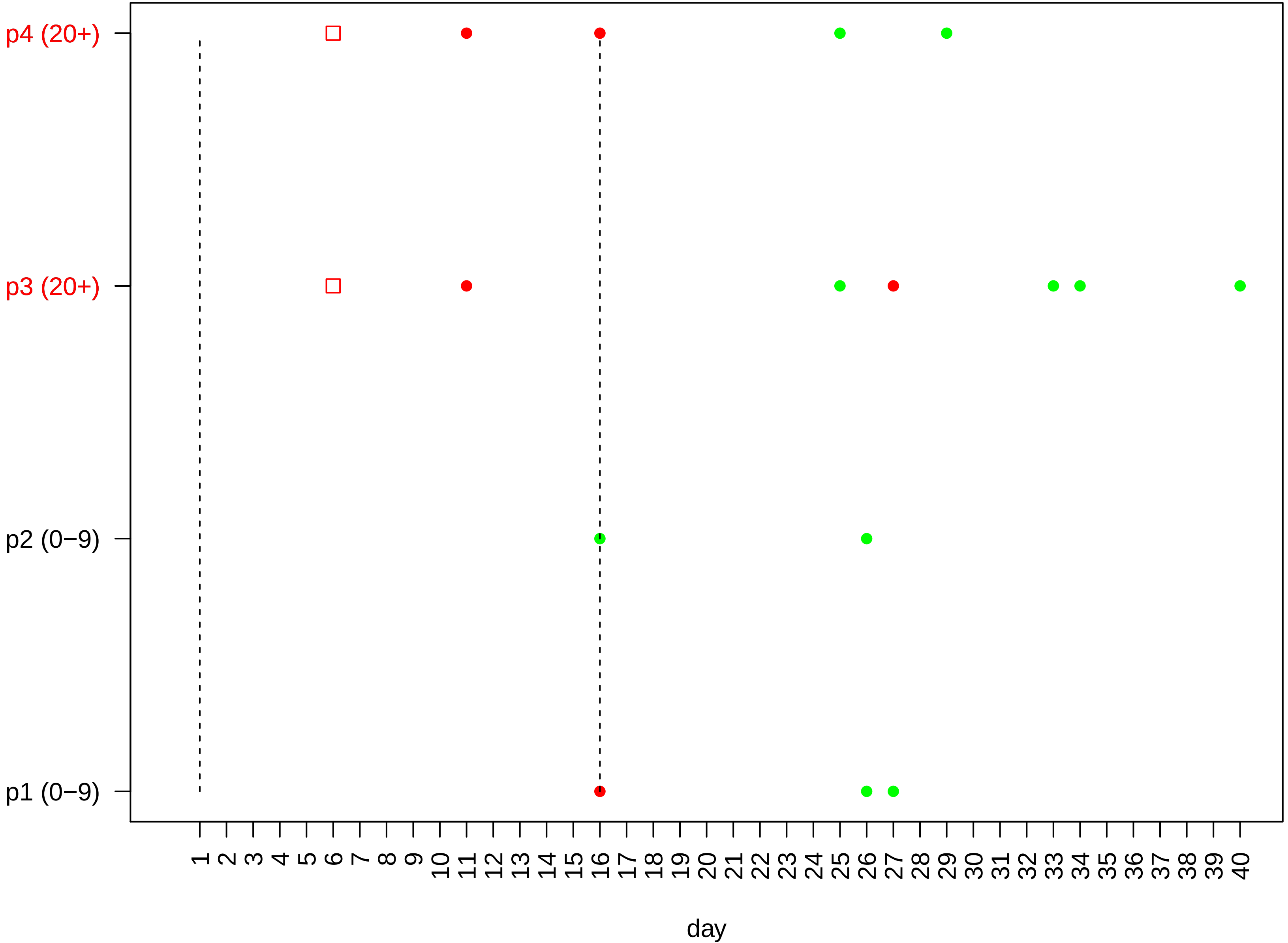

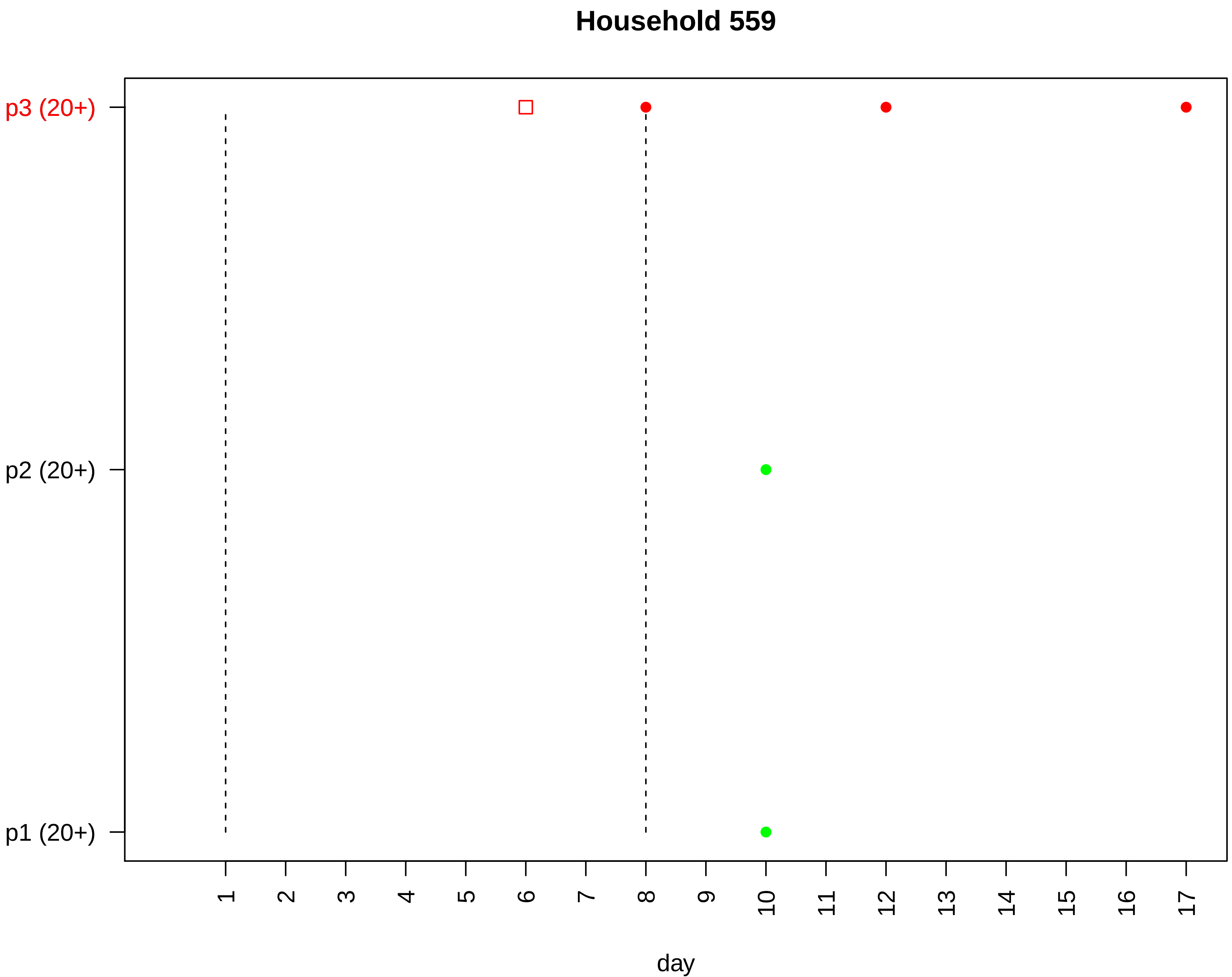

Household 559
p3 (20+)
p2 (20+)
p1 (20+)
1
2
3
4
5
6
7
8
9
10
11
12
13
14
15
16
17
day

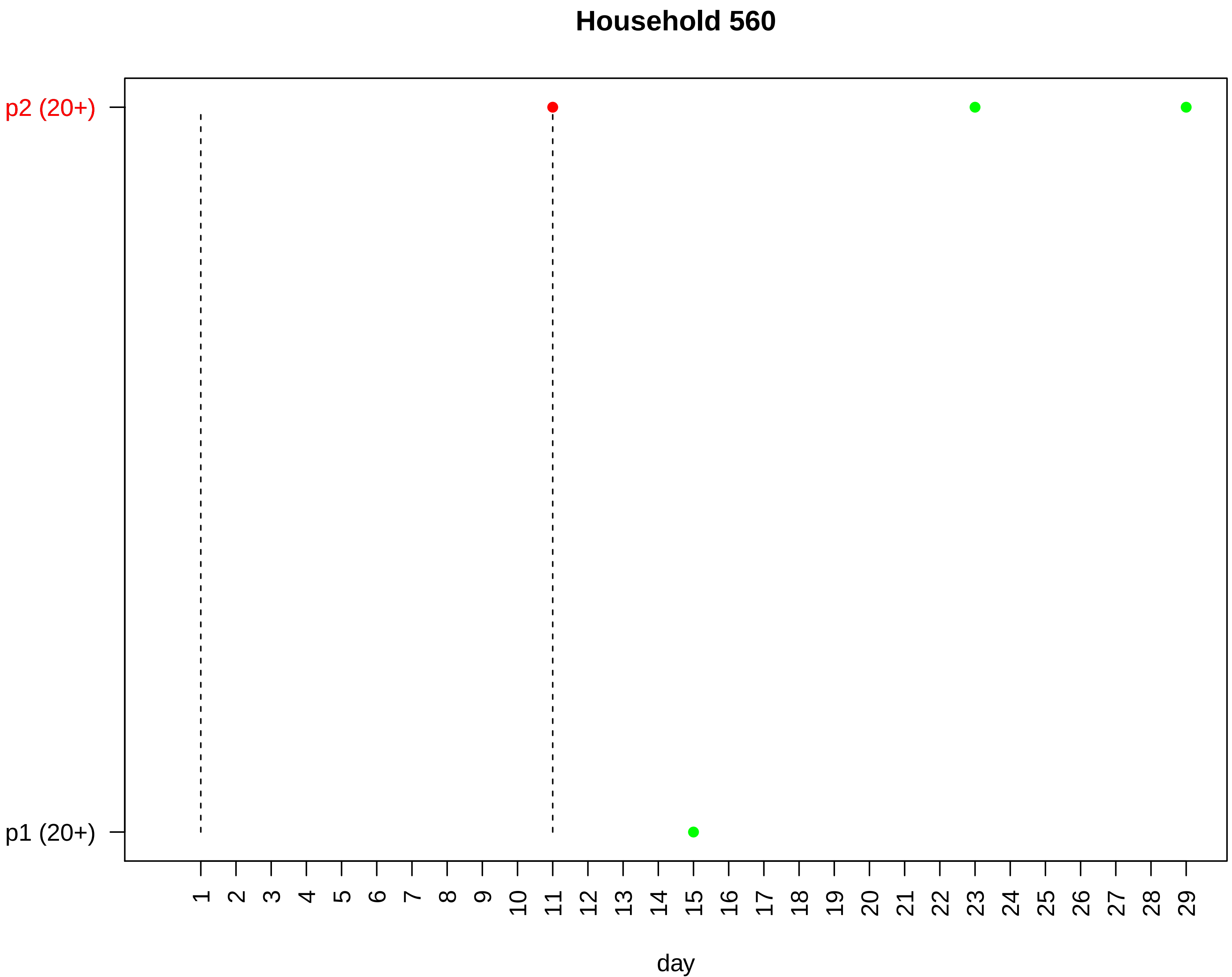

Household 560
p2 (20+)
p1 (20+)
1
2
3
4
5
6
7
8
9
10
11
12
13
14
15
16
17
18
19
20
21
22
23
24
25
26
27
28
29
day

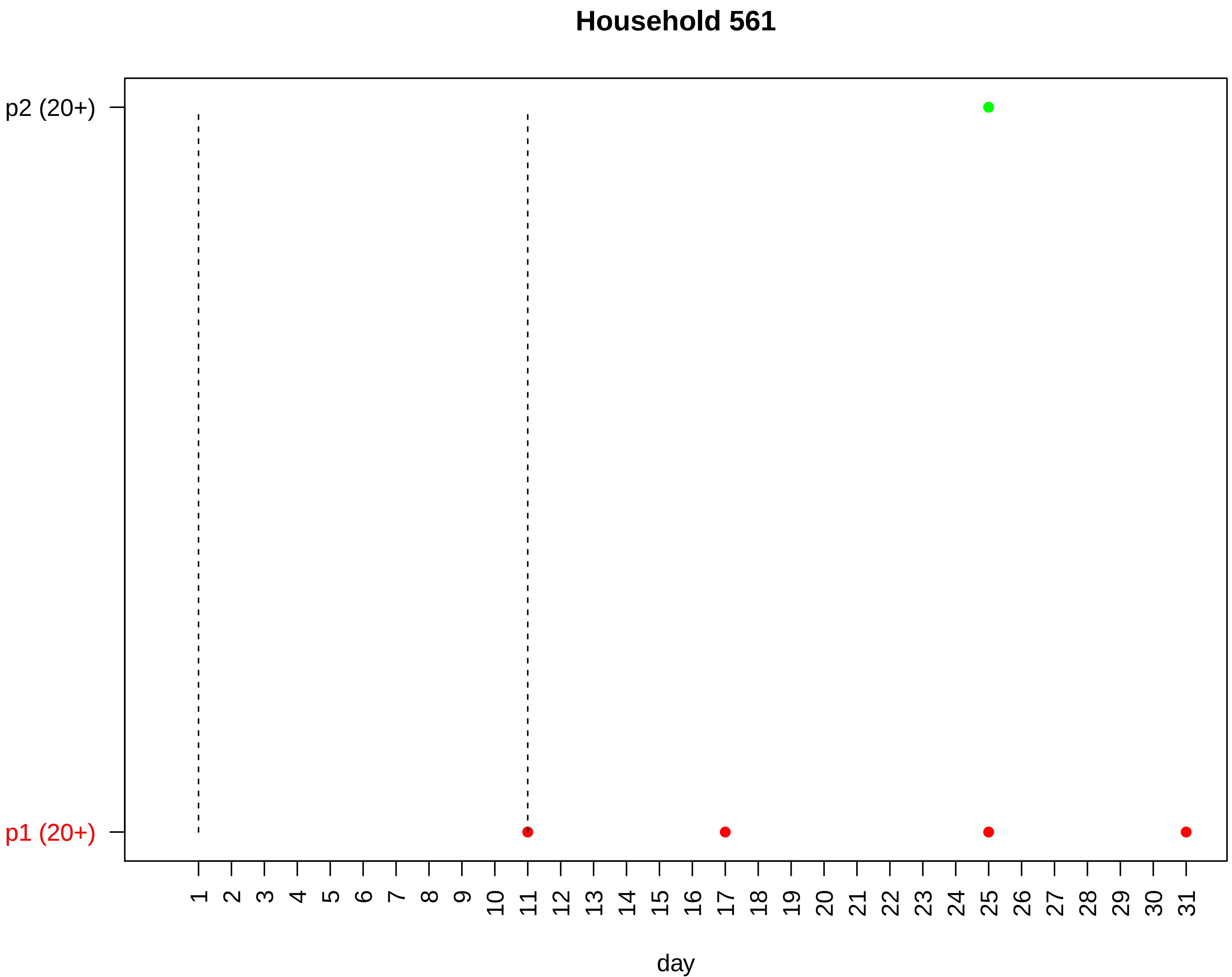

Household 561
p2 (20+)
p1 (20+)
1
2
3
4
5
6
7
8
9
10
11
12
13
14
15
16
17
18
19
20
21
22
23
24
25
26
27
28
29
30
31
day

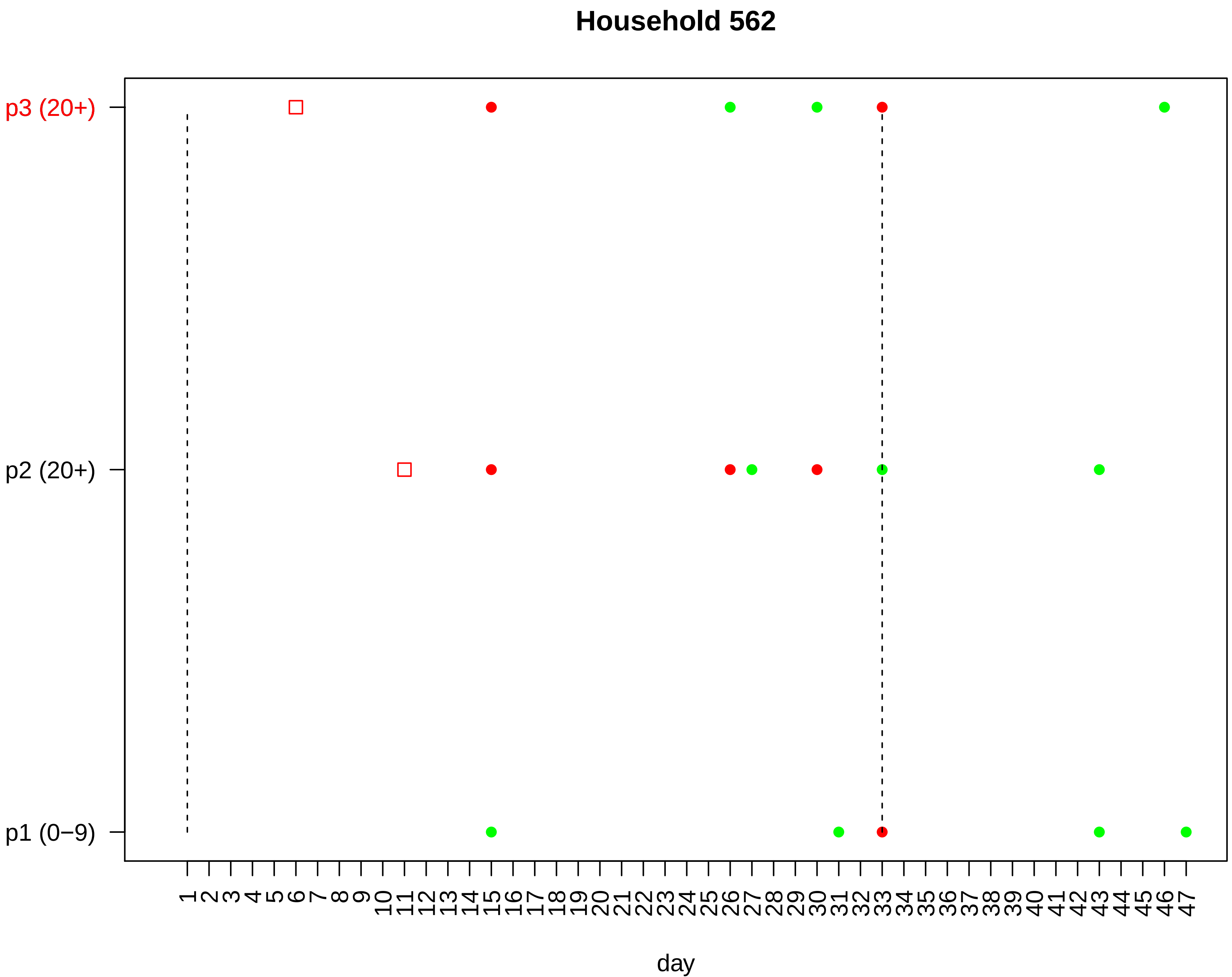

Household 562
p3 (20+)
p2 (20+)
p1 (0−9)
1 2 3 4 5 6 7 8 9 10 11 12 13 14 15 16 17 18 19 20 21 22 23 24 25 26 27 28 29 30 31 32 33 34 35 36 37 38 39 40 41 42 43 44 45 46 47
day

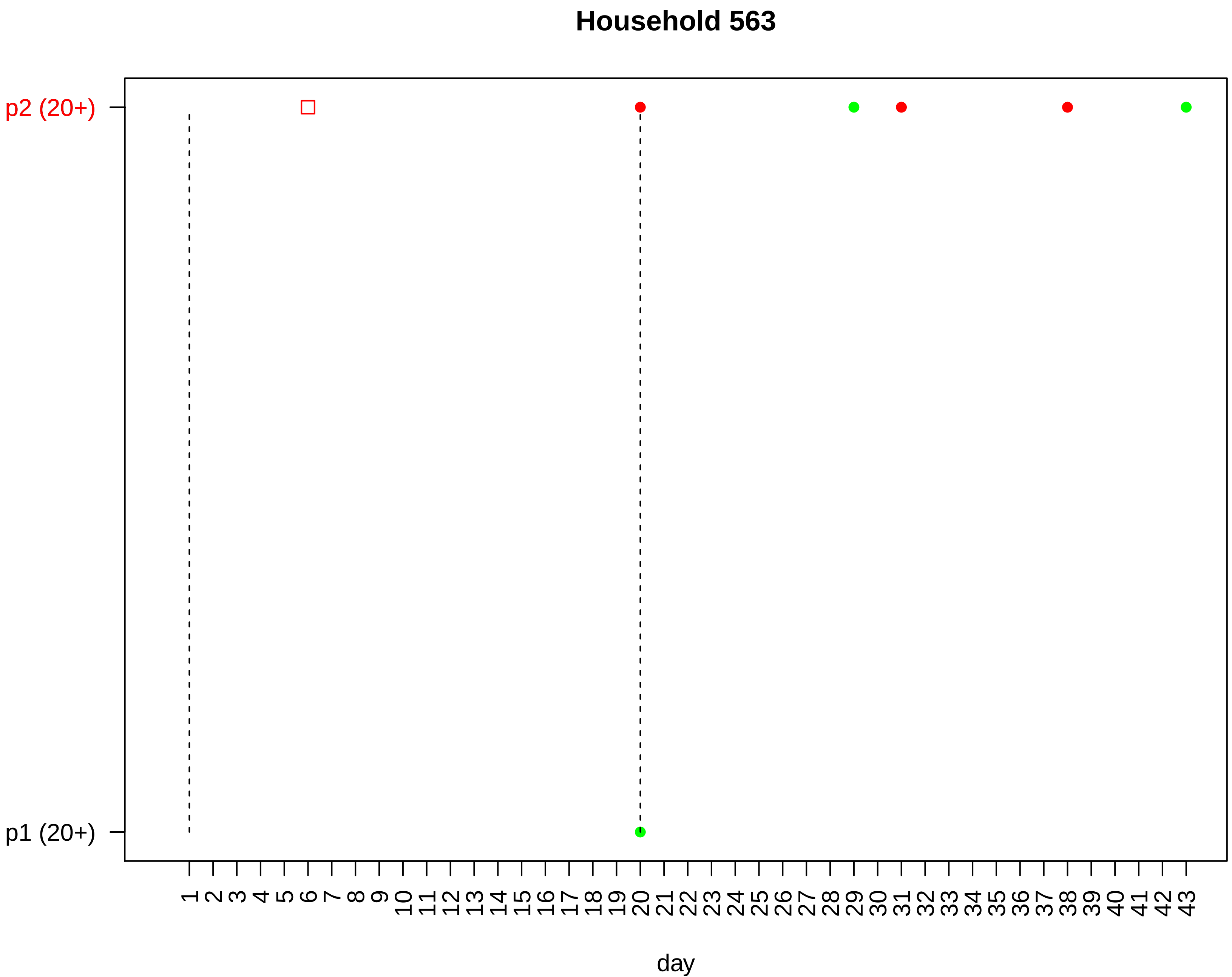
Household 563
p2 (20+)
p1 (20+)
1 2 3 4 5 6 7 8 9 10 11 12 13 14 15 16 17 18 19 20 21 22 23 24 25 26 27 28 29 30 31 32 33 34 35 36 37 38 39 40 41 42 43
day

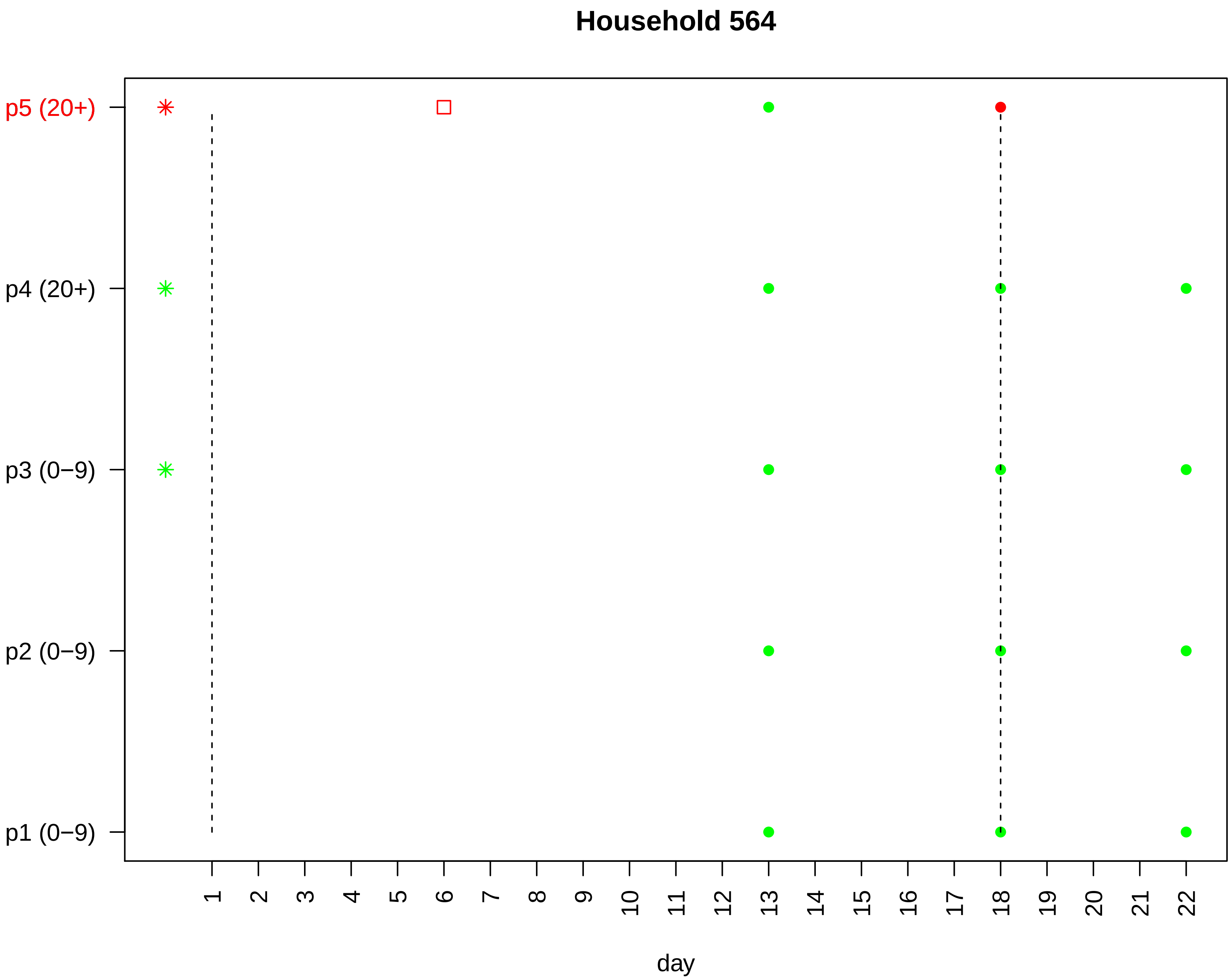

Household 564
p5 (20+)
p4 (20+)
p3 (0−9)
p2 (0−9)
p1 (0−9)
1
2
3
4
5
6
7
8
9
10
11
12
13
14
15
16
17
18
19
20
21
22
day

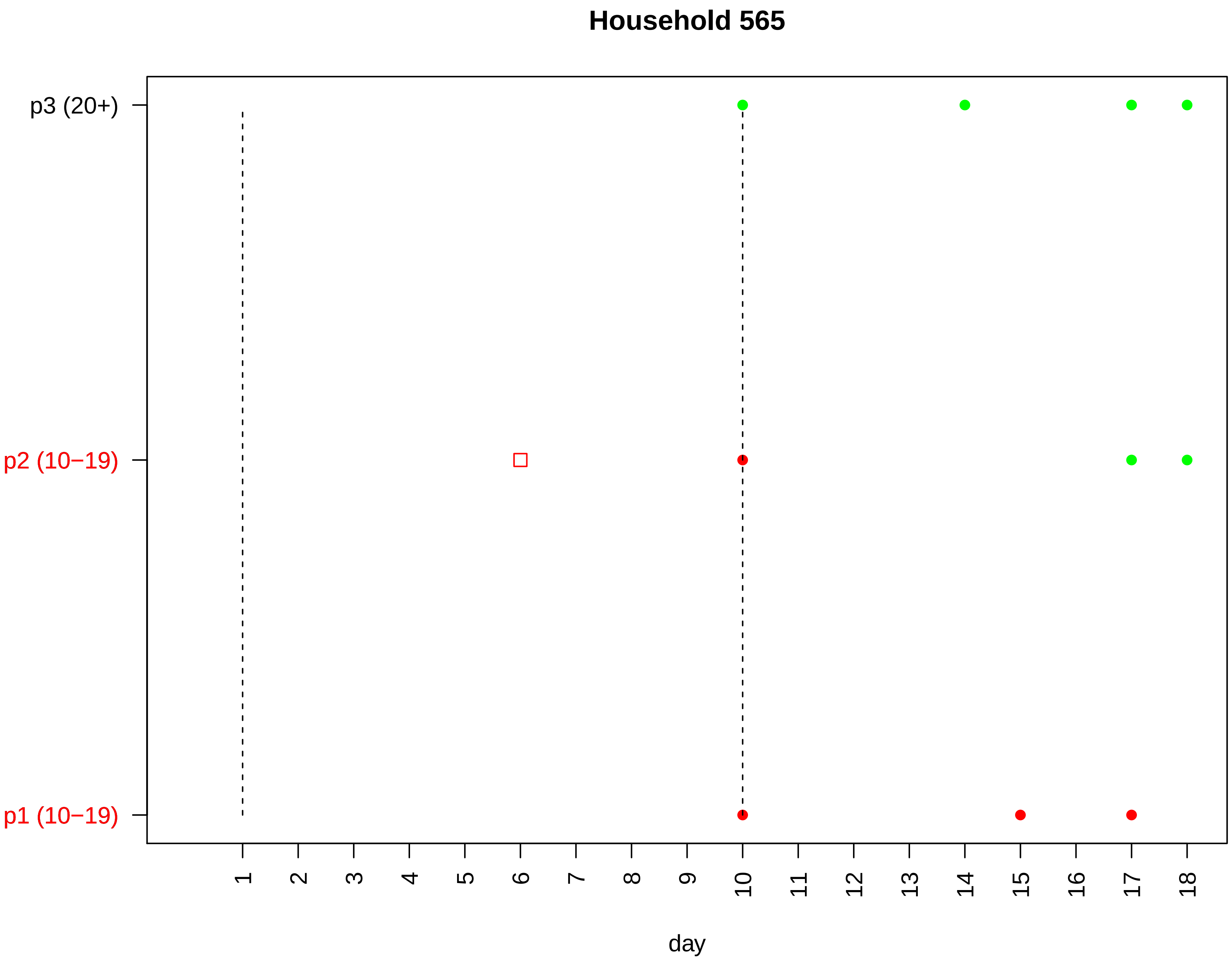

Household 565
p3 (20+)
p2 (10−19)
p1 (10−19)
1
2
3
4
5
6
7
8
9
10
11
12
13
14
15
16
17
18
day

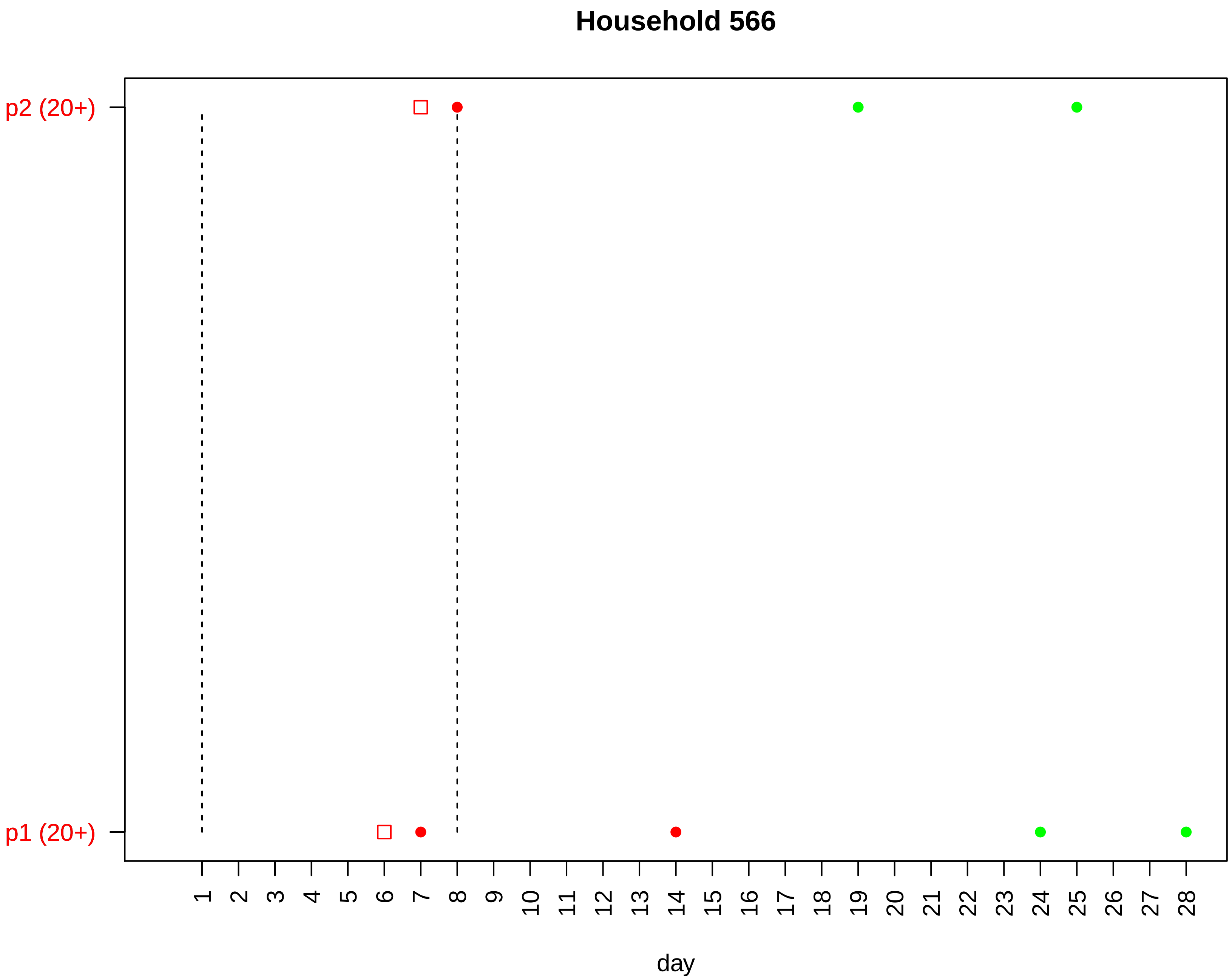
Household 566
p2 (20+)
p1 (20+)
1
2
3
4
5
6
7
8
9
10
11
12
13
14
15
16
17
18
19
20
21
22
23
24
25
26
27
28
day

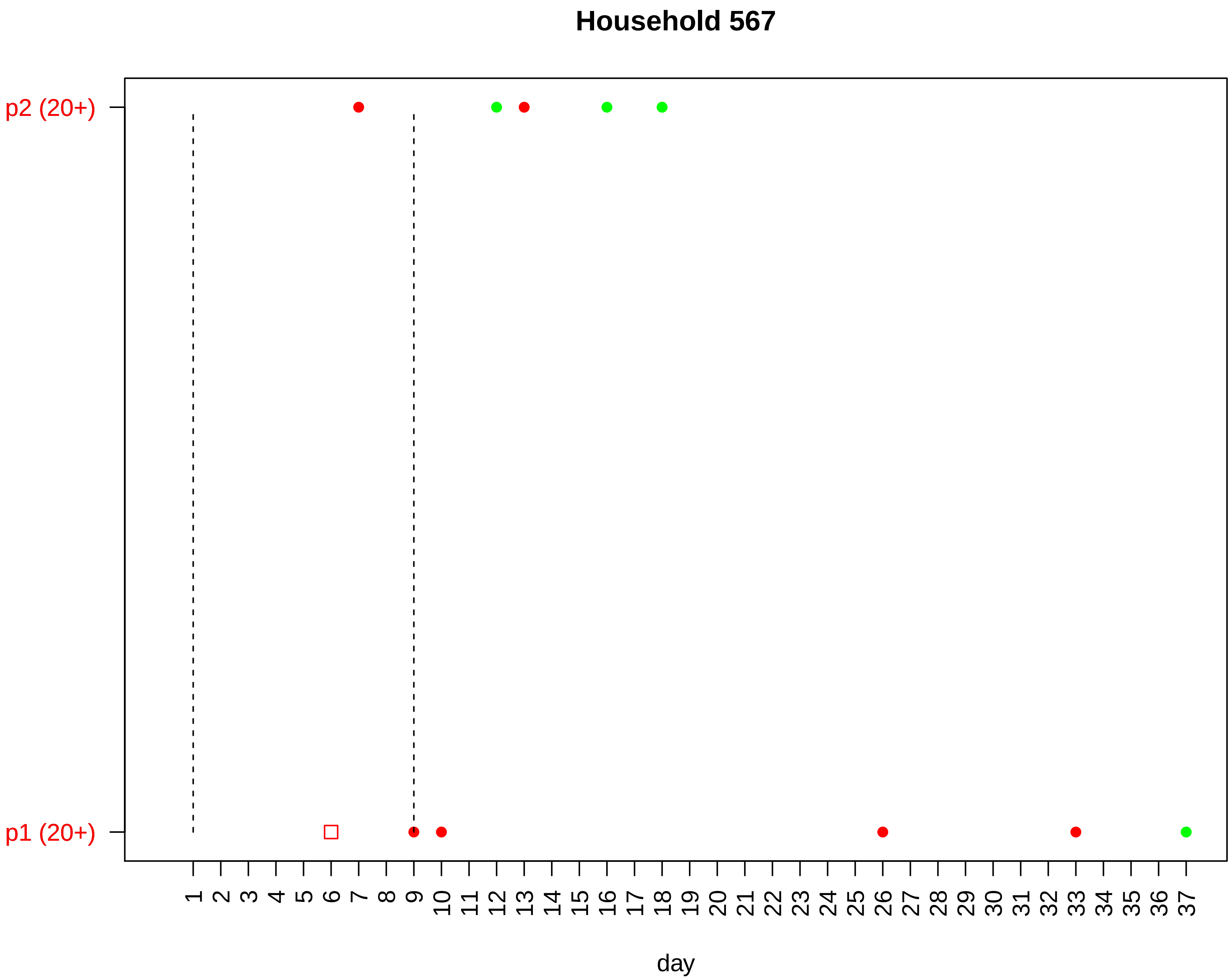

Household 567
p2 (20+)
p1 (20+)
1 2 3 4 5 6 7 8 9 10 11 12 13 14 15 16 17 18 19 20 21 22 23 24 25 26 27 28 29 30 31 32 33 34 35 36 37
day

Household 568

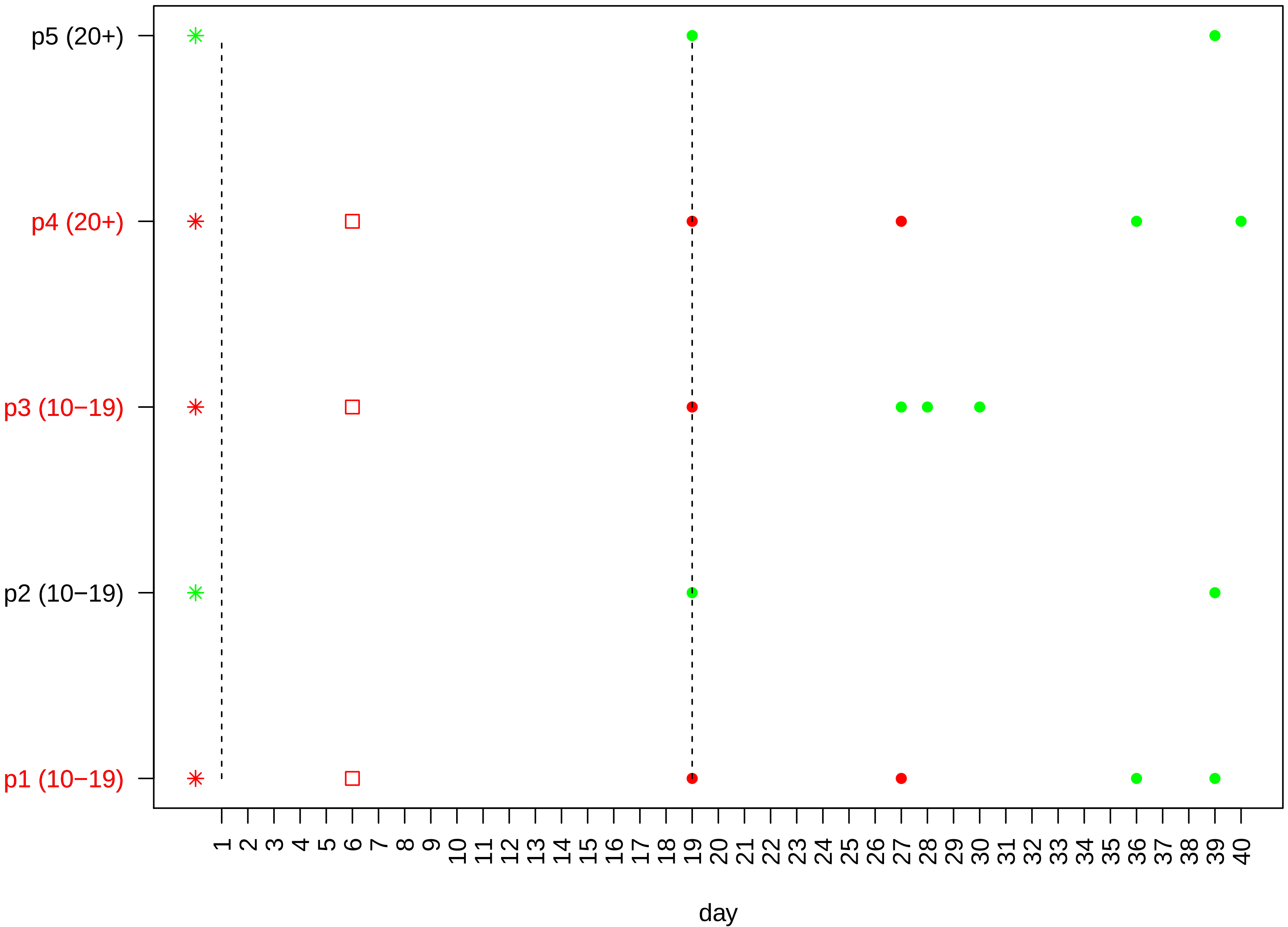

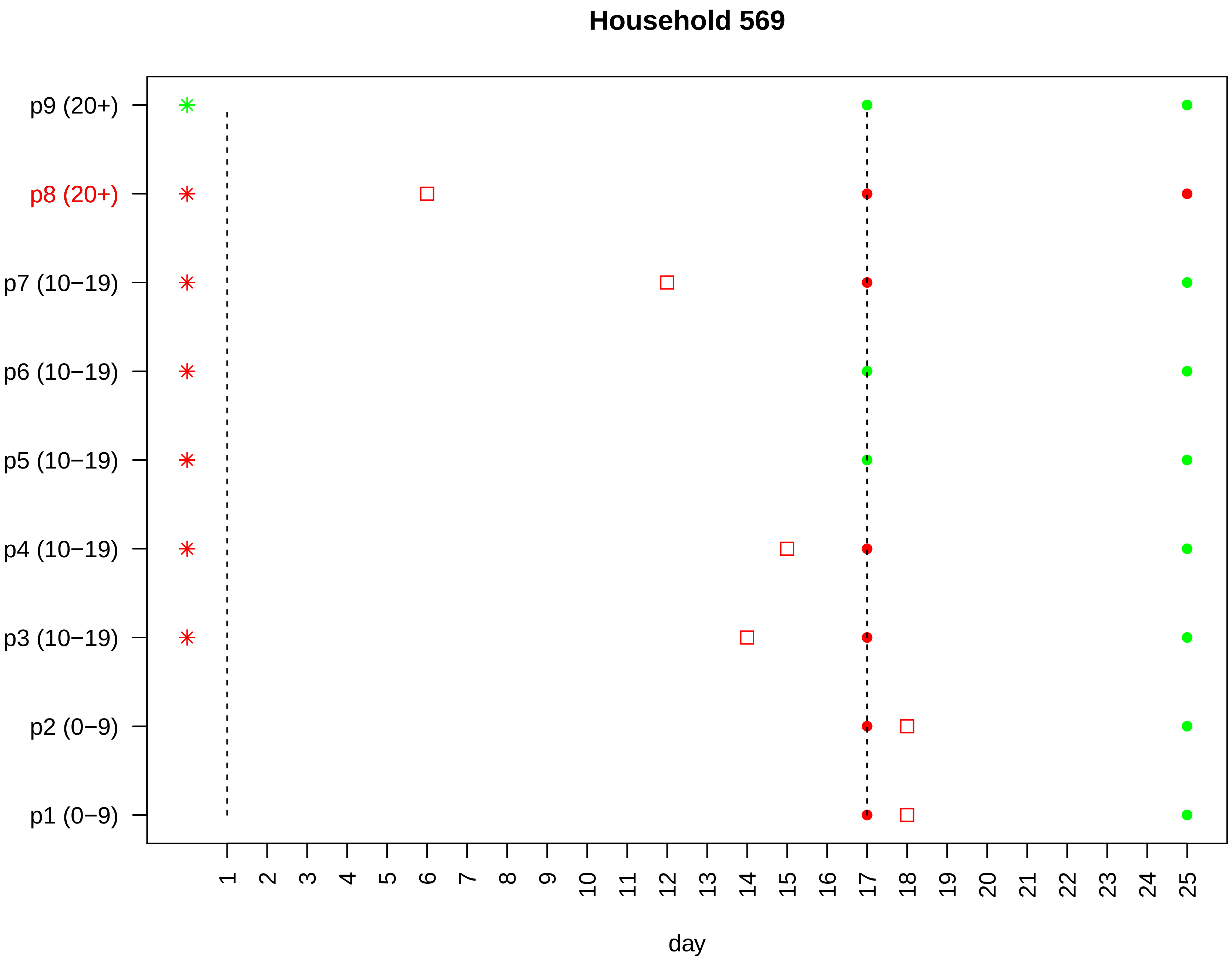

Household 569
p9 (20+)
p8 (20+)
p7 (10−19)
p6 (10−19)
p5 (10−19)
p4 (10−19)
p3 (10−19)
p2 (0−9)
p1 (0−9)
1 2 3 4 5 6 7 8 9 10 11 12 13 14 15 16 17 18 19 20 21 22 23 24 25
day

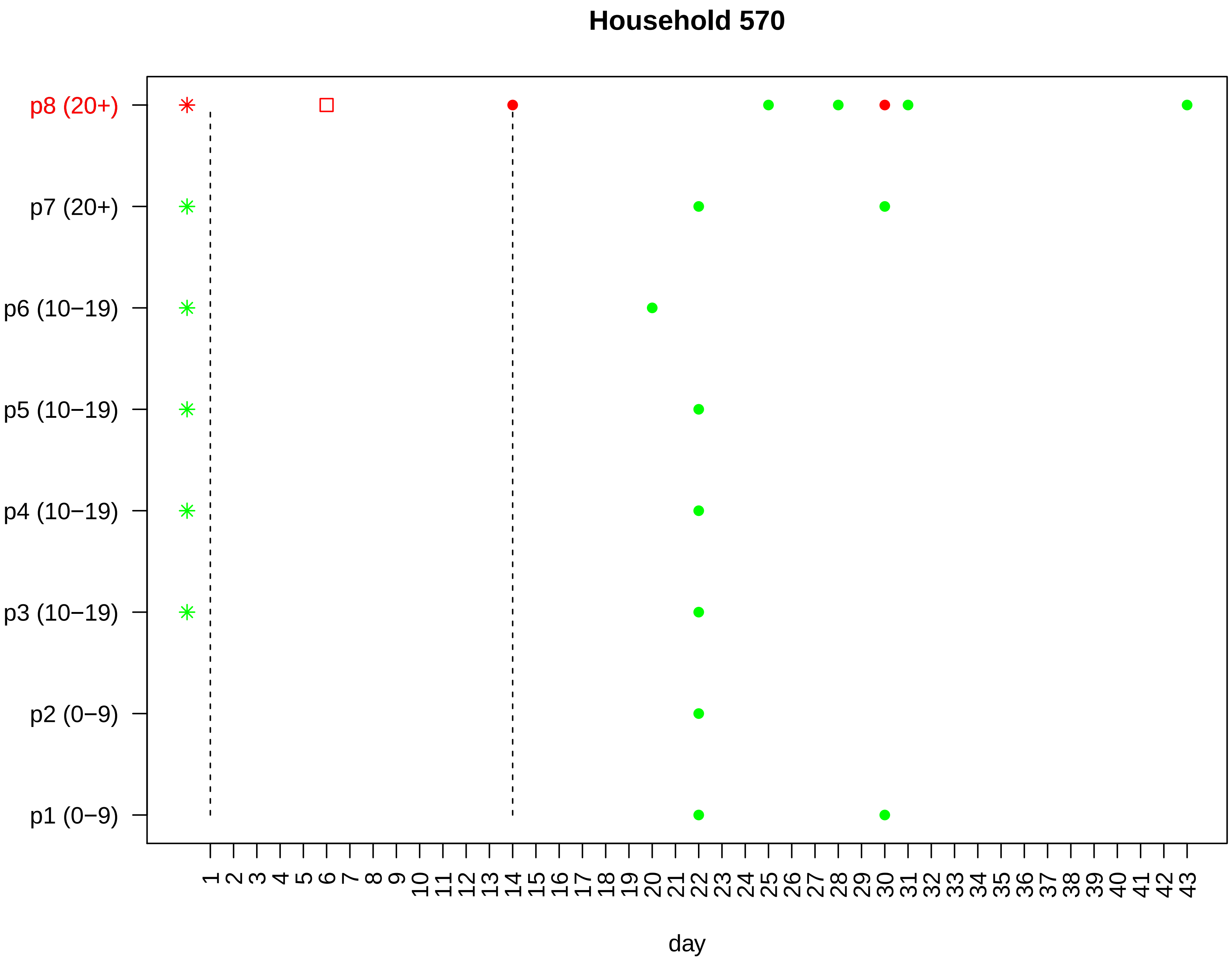

Household 570
p8 (20+)
p7 (20+)
p6 (10−19)
p5 (10−19)
p4 (10−19)
p3 (10−19)
p2 (0−9)
p1 (0−9)
1 2 3 4 5 6 7 8 9 10 11 12 13 14 15 16 17 18 19 20 21 22 23 24 25 26 27 28 29 30 31 32 33 34 35 36 37 38 39 40 41 42 43
day

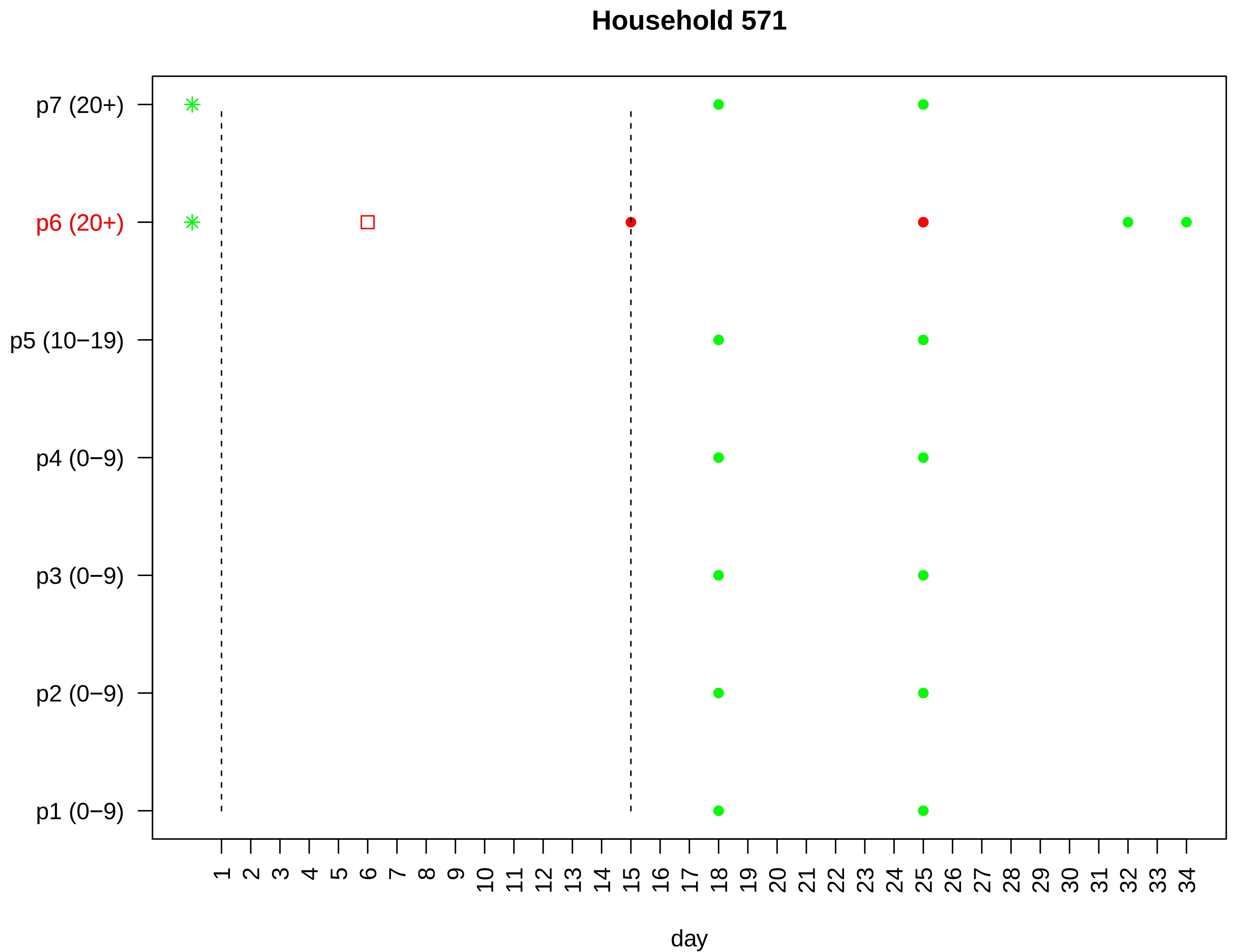

Household 571
p7 (20+)
p6 (20+)
p5 (10−19)
p4 (0−9)
p3 (0−9)
p2 (0−9)
p1 (0−9)
1 2 3 4 5 6 7 8 9 10 11 12 13 14 15 16 17 18 19 20 21 22 23 24 25 26 27 28 29 30 31 32 33 34
day

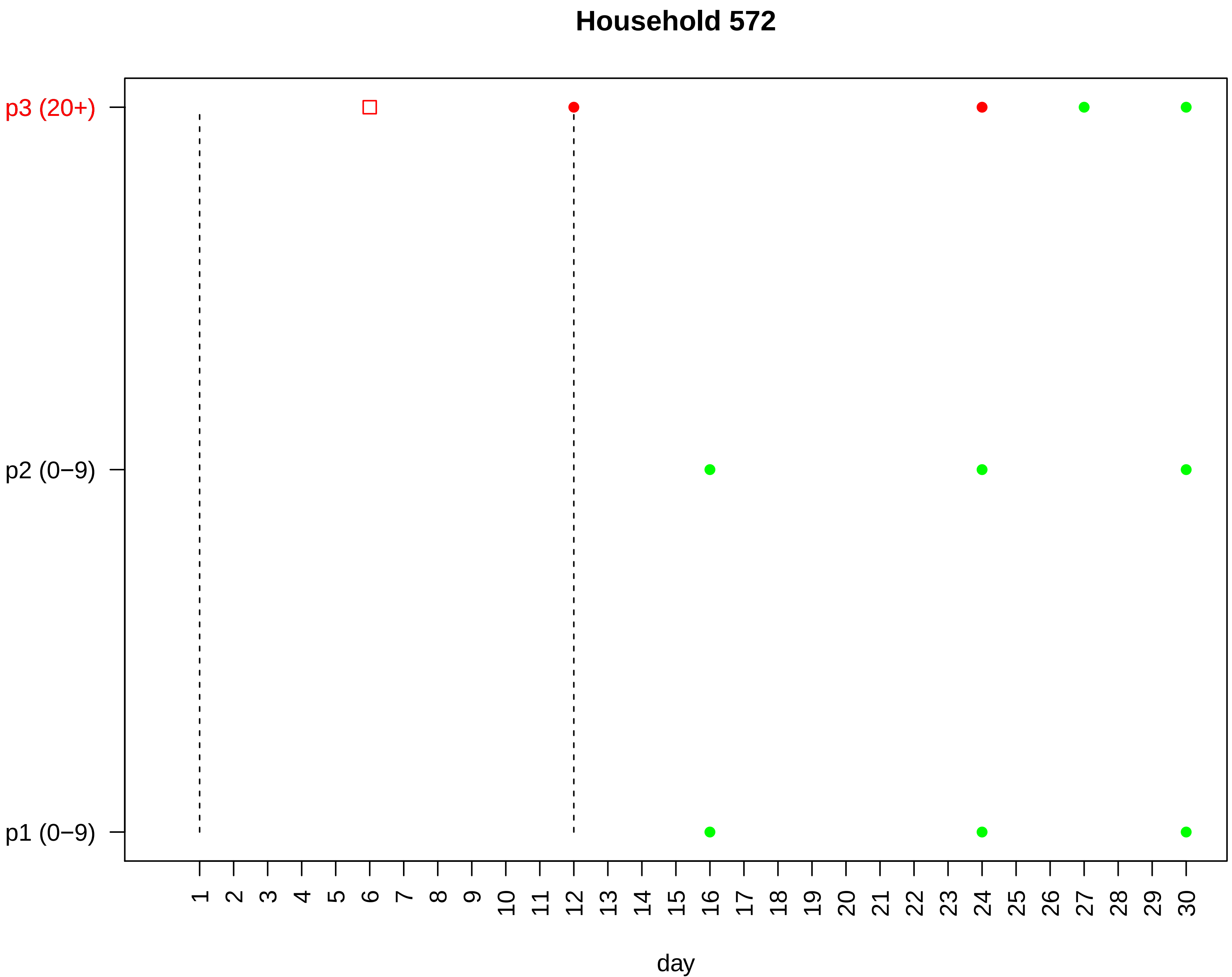

Household 572
p3 (20+)
p2 (0−9)
p1 (0−9)
1
2
3
4
5
6
7
8
9
10
11
12
13
14
15
16
17
18
19
20
21
22
23
24
25
26
27
28
29
30
day

Household 573

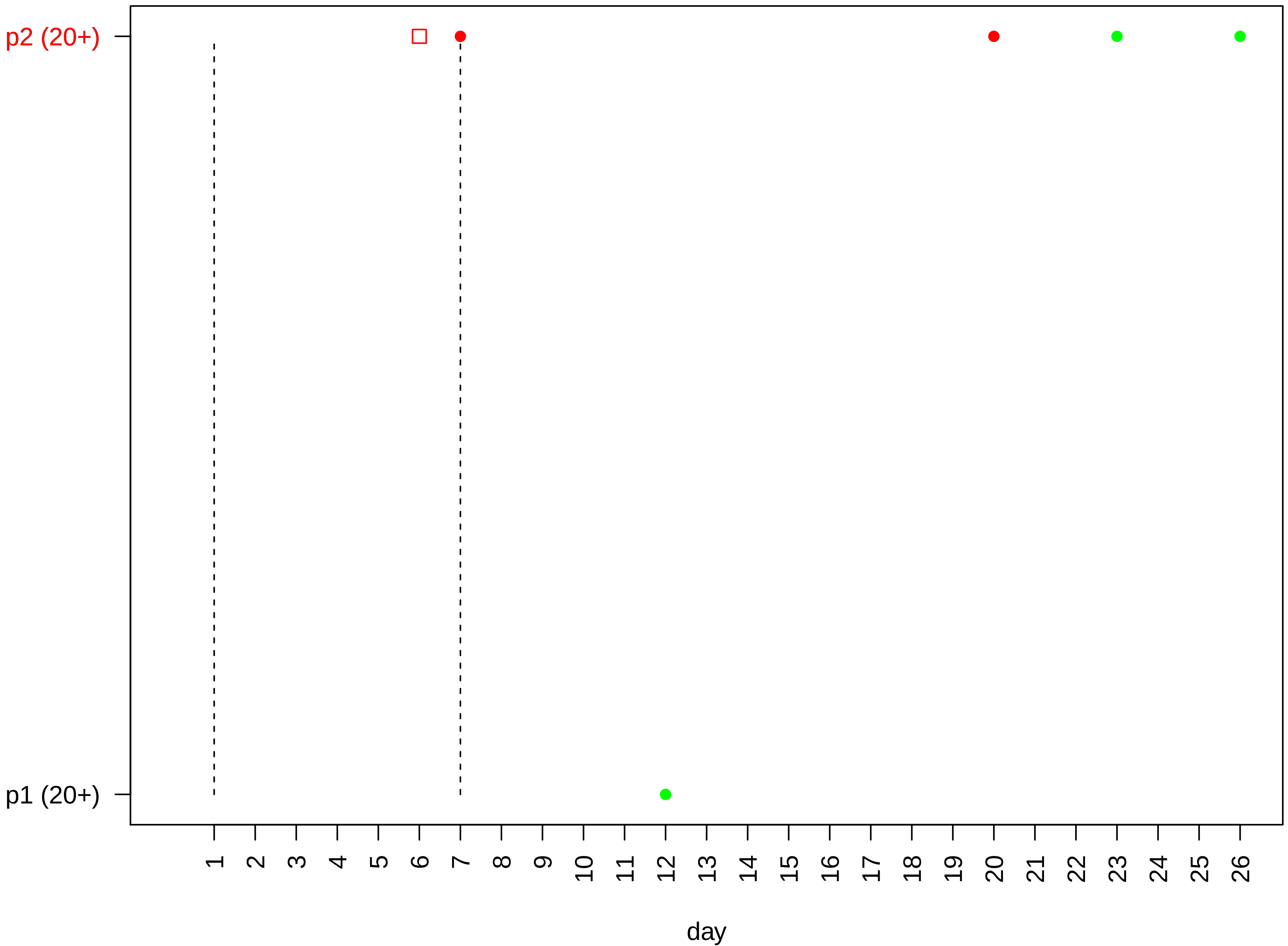

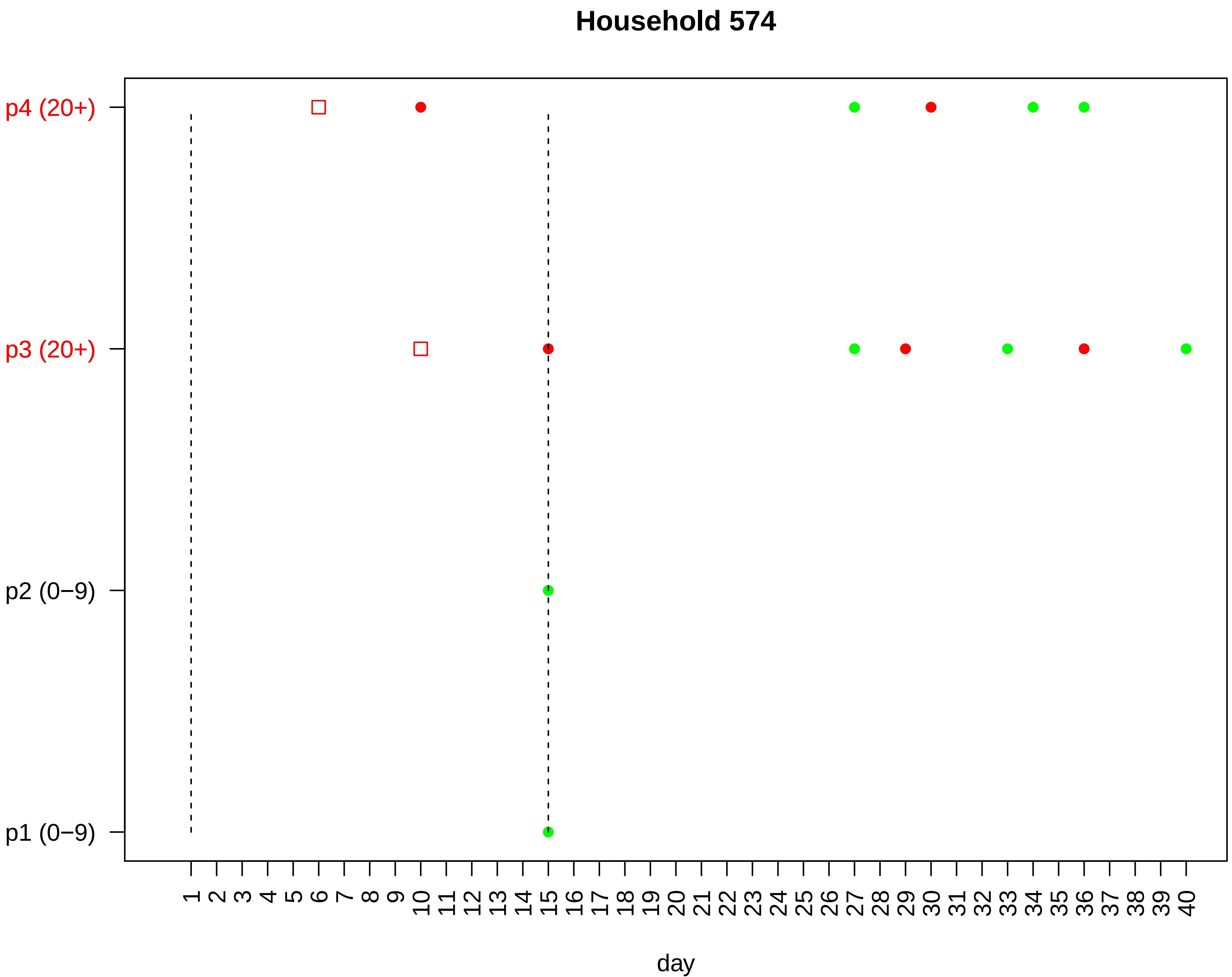

Household 574
p4 (20+)
p3 (20+)
p2 (0−9)
p1 (0−9)
1 2 3 4 5 6 7 8 9 10 11 12 13 14 15 16 17 18 19 20 21 22 23 24 25 26 27 28 29 30 31 32 33 34 35 36 37 38 39 40
day

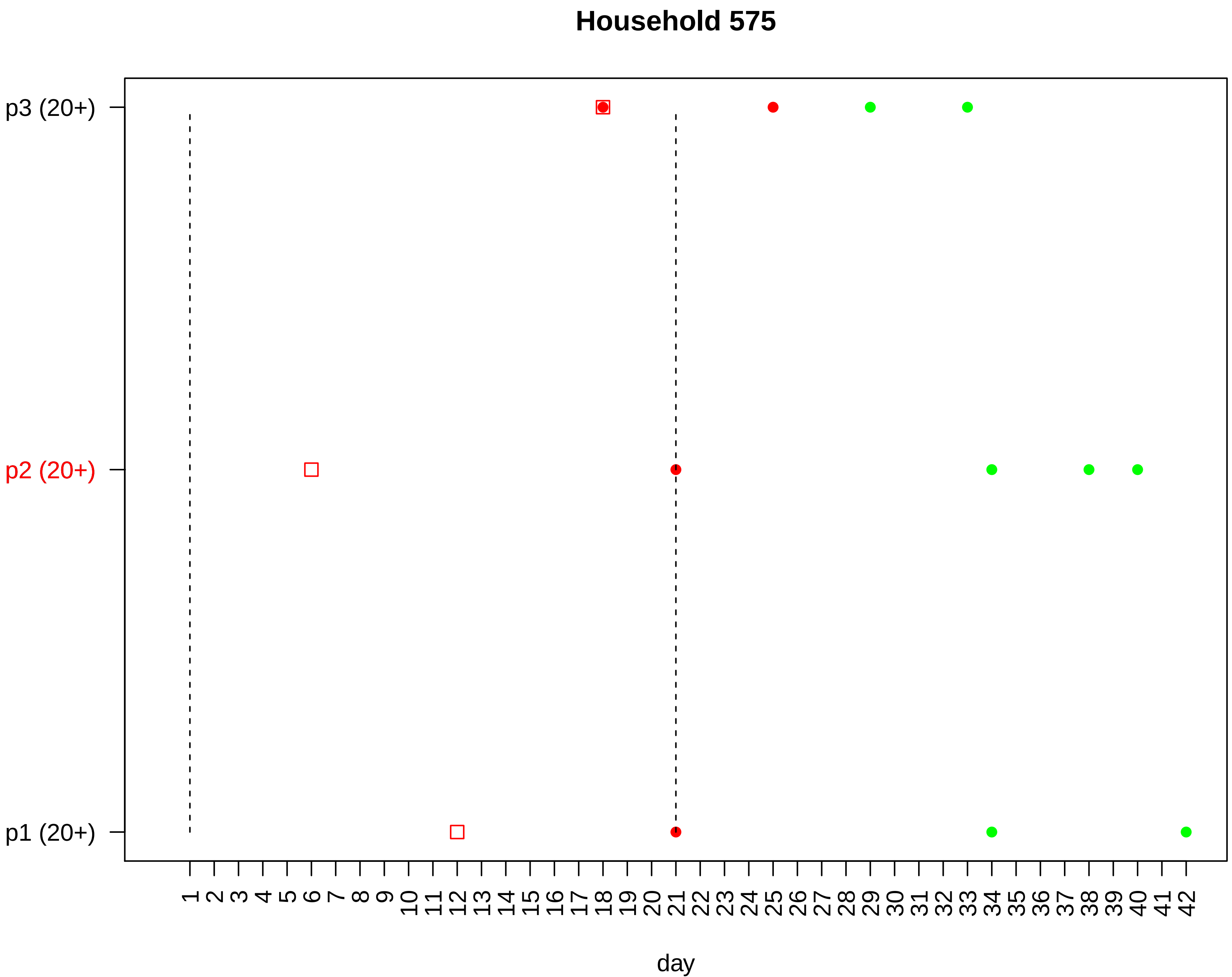
Household 575
p3 (20+)
p2 (20+)
p1 (20+)
1 2 3 4 5 6 7 8 9 10 11 12 13 14 15 16 17 18 19 20 21 22 23 24 25 26 27 28 29 30 31 32 33 34 35 36 37 38 39 40 41 42
day

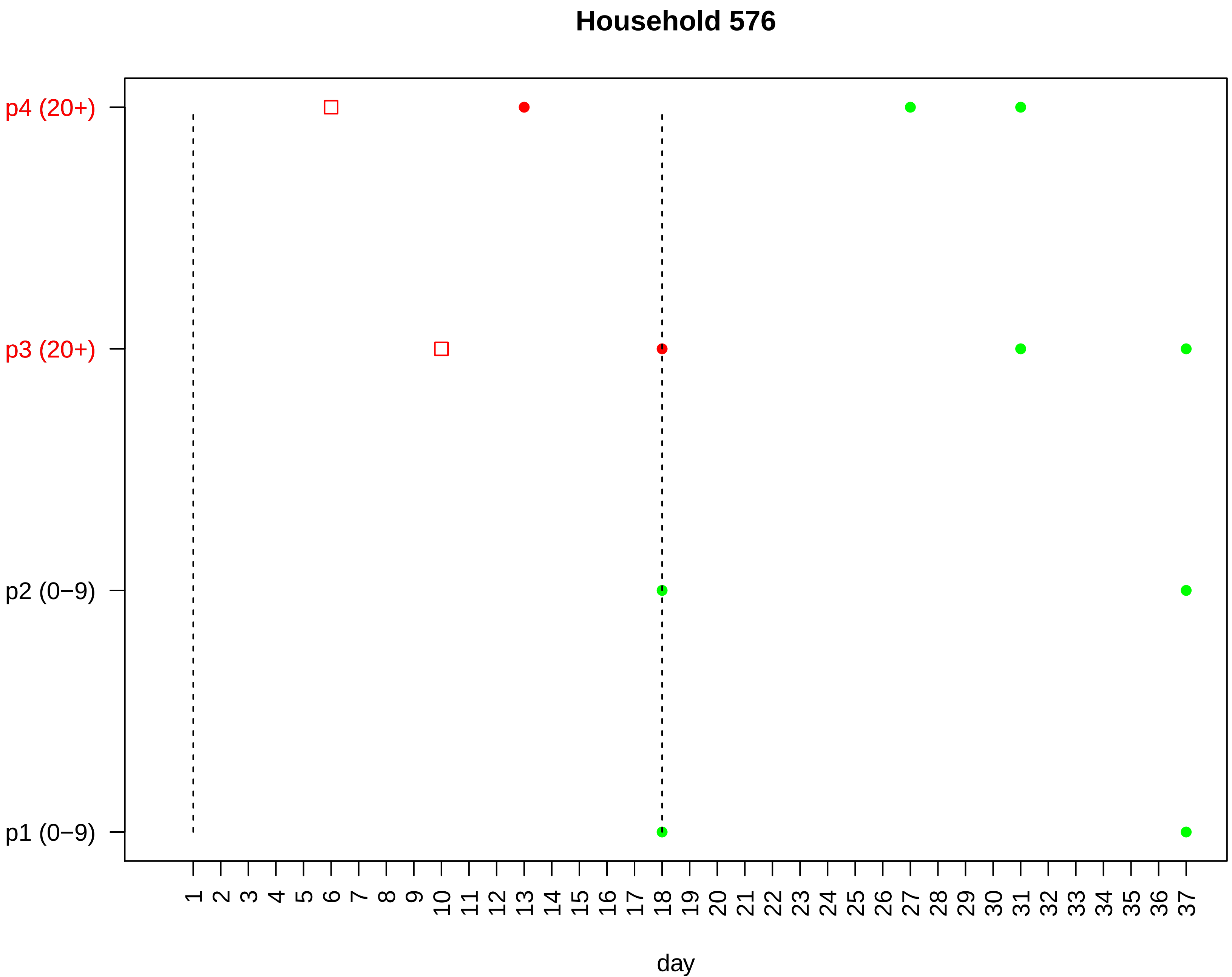

Household 576
p4 (20+)
p3 (20+)
p2 (0−9)
p1 (0−9)
1 2 3 4 5 6 7 8 9 10 11 12 13 14 15 16 17 18 19 20 21 22 23 24 25 26 27 28 29 30 31 32 33 34 35 36 37
day

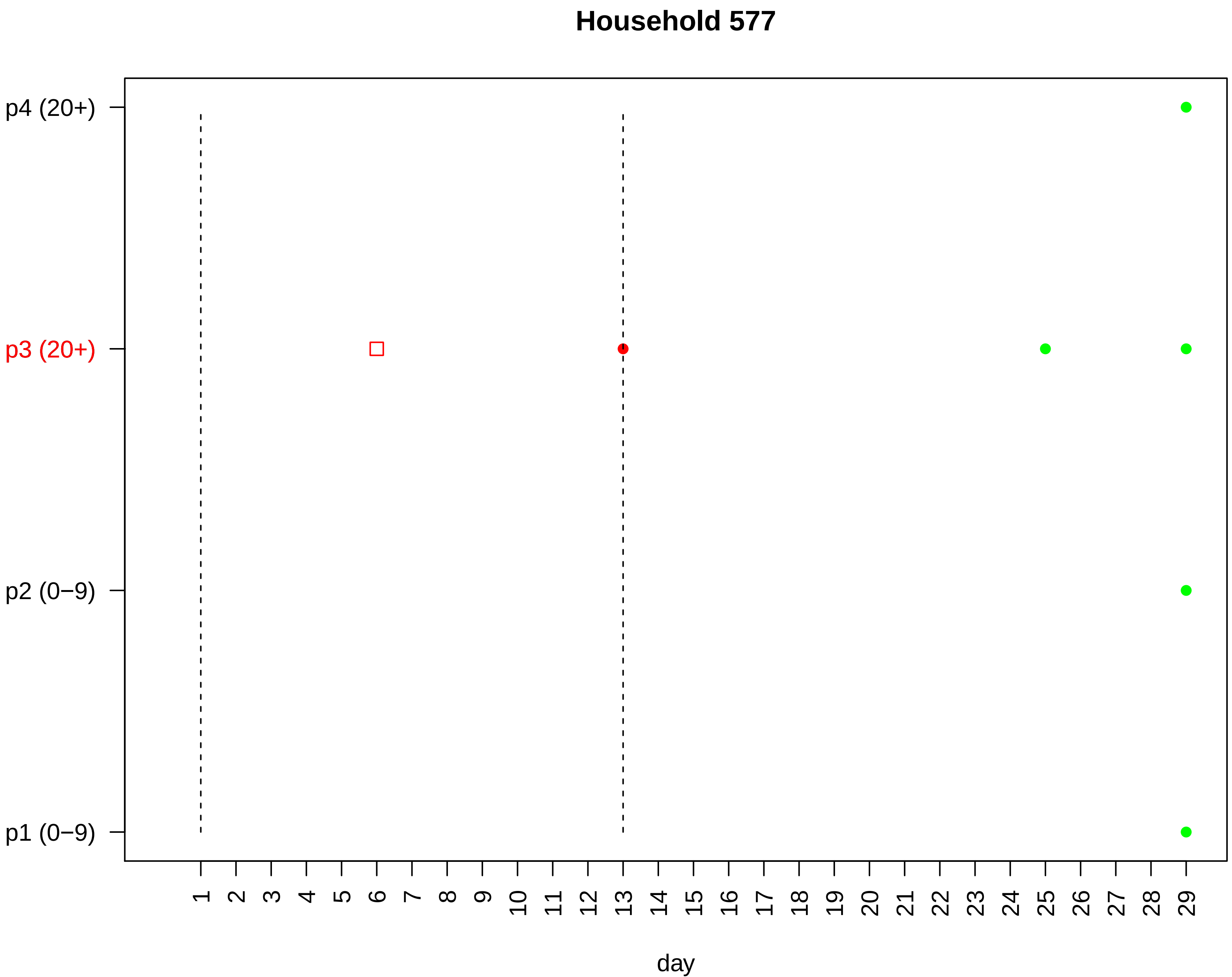

Household 577
p4 (20+)
p3 (20+)
p2 (0−9)
p1 (0−9)
1
2
3
4
5
6
7
8
9
10
11
12
13
14
15
16
17
18
19
20
21
22
23
24
25
26
27
28
29
day

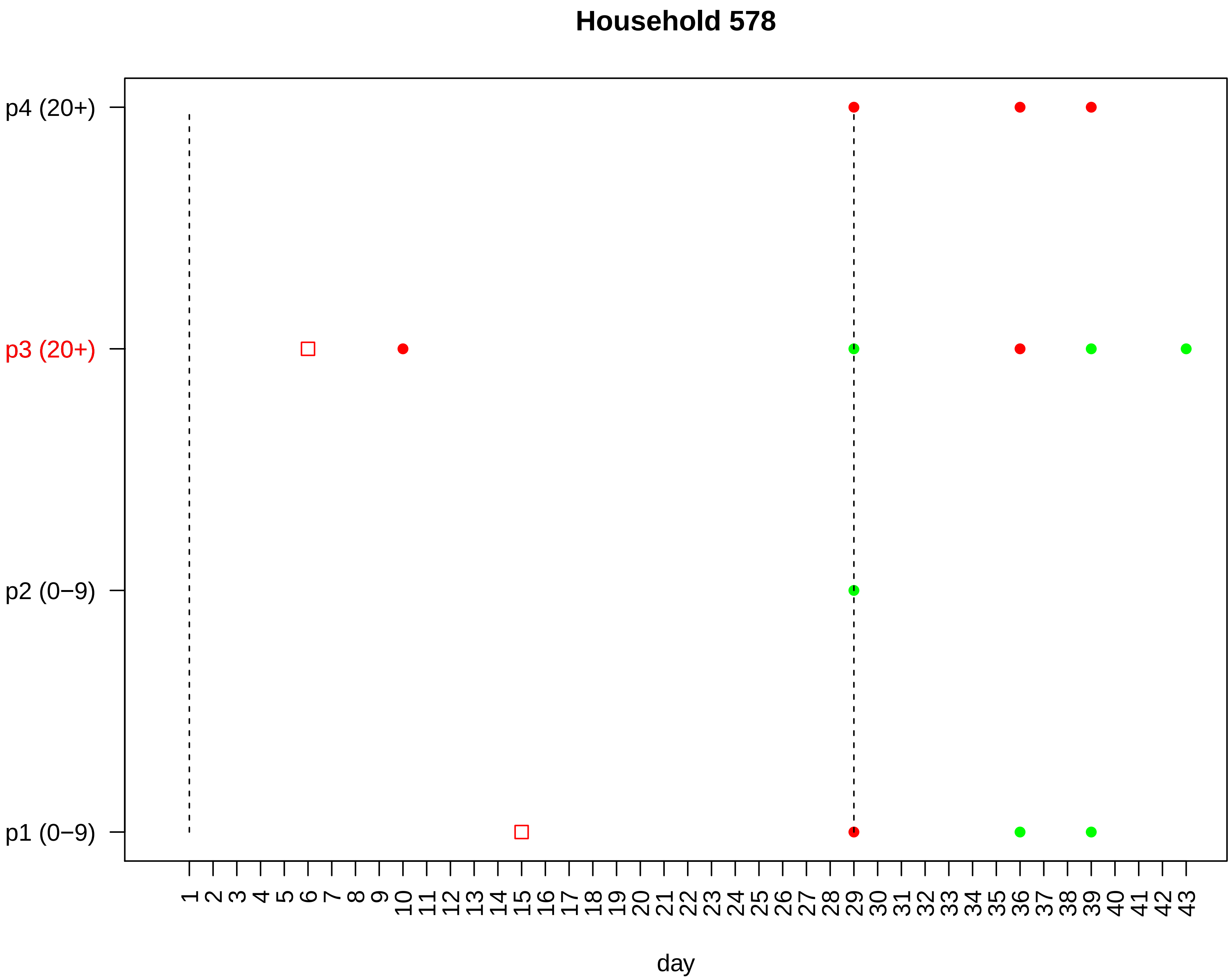

Household 578
p4 (20+)
p3 (20+)
p2 (0−9)
p1 (0−9)
1 2 3 4 5 6 7 8 9 10 11 12 13 14 15 16 17 18 19 20 21 22 23 24 25 26 27 28 29 30 31 32 33 34 35 36 37 38 39 40 41 42 43
day

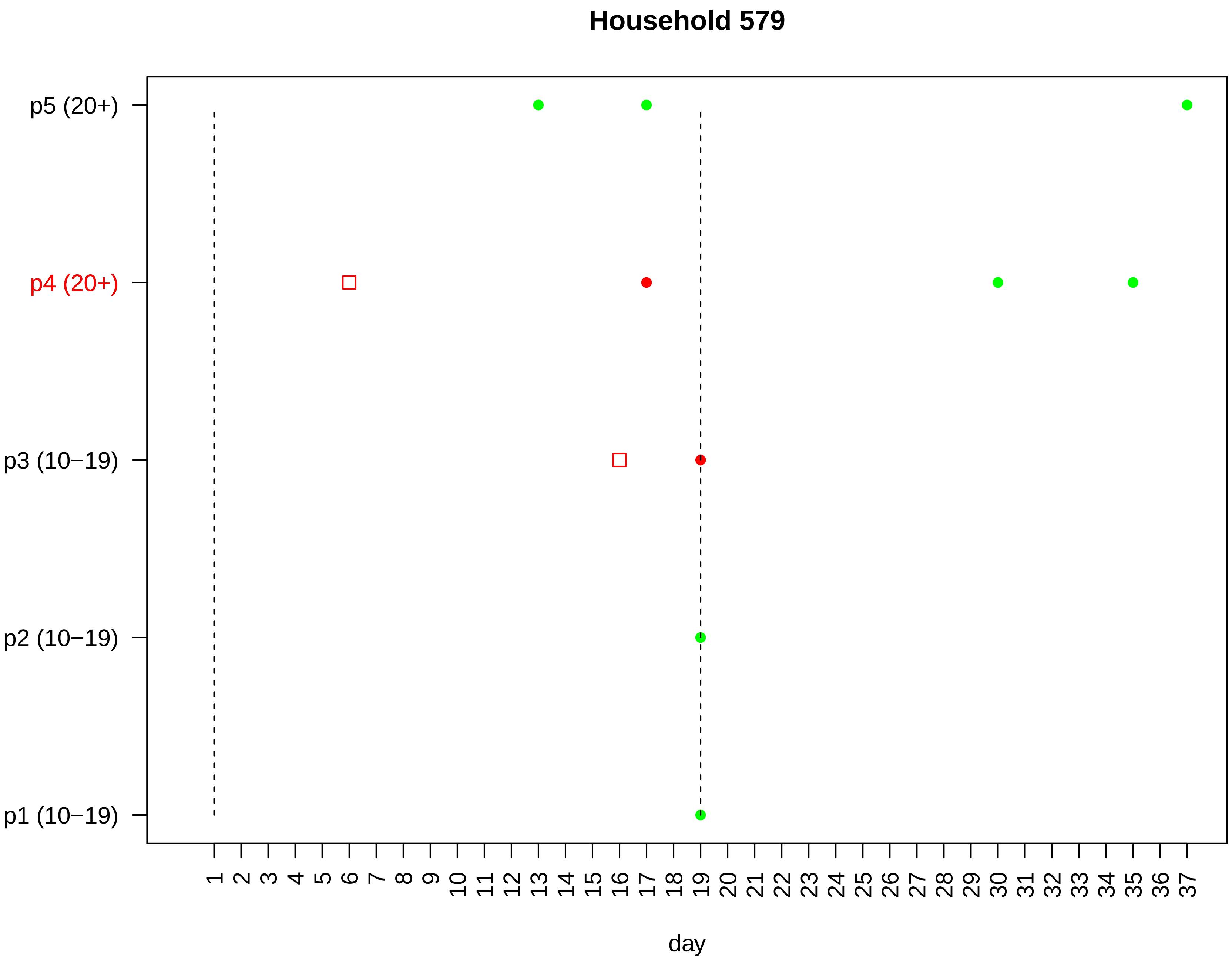

Household 579
p5 (20+)
p4 (20+)
p3 (10−19)
p2 (10−19)
p1 (10−19)
1
2
3
4
5
6
7
8
9
10
11
12
13
14
15
16
17
18
19
20
21
22
23
24
25
26
27
28
29
30
31
32
33
34
35
36
37
day

Household 581

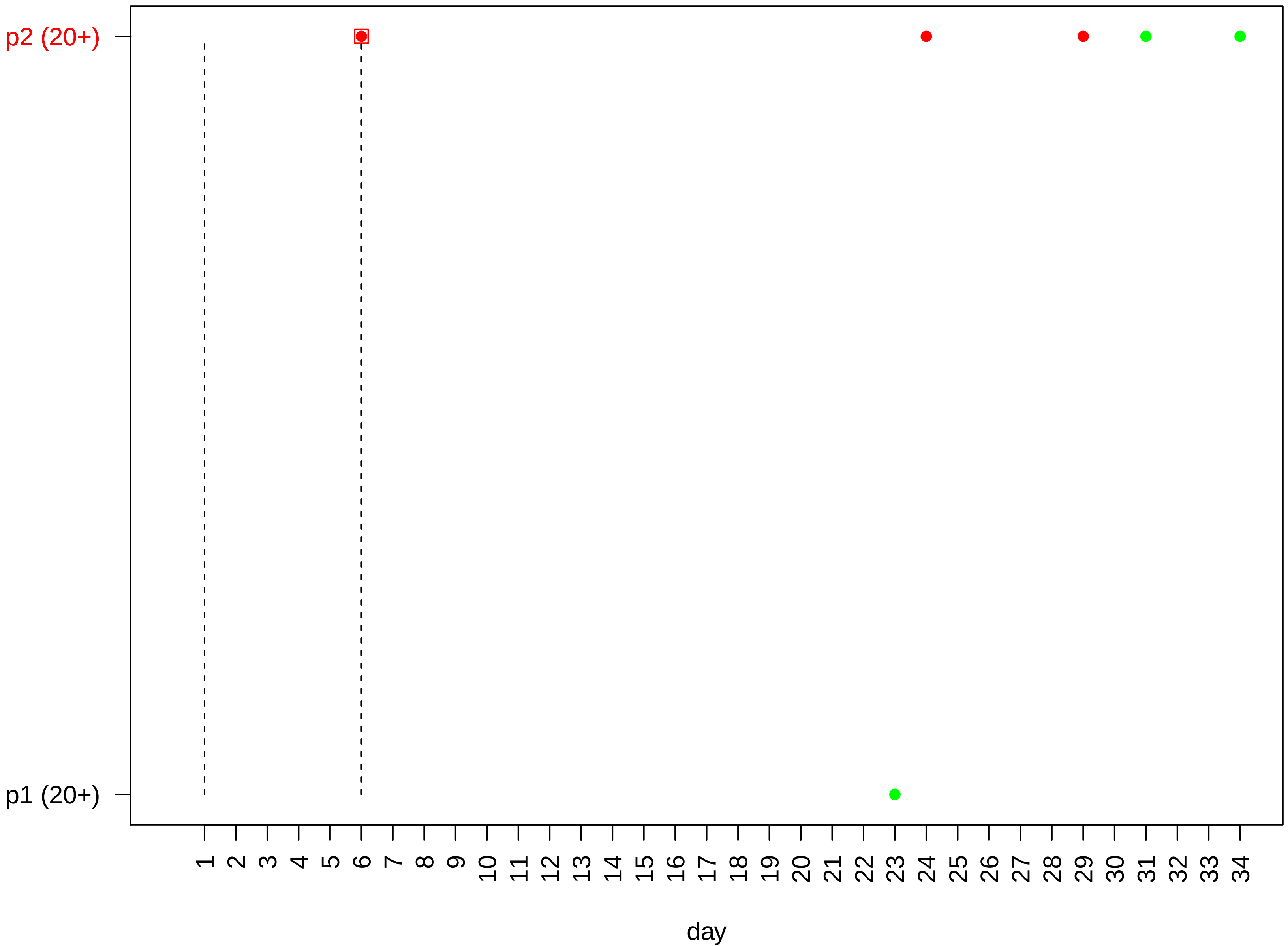

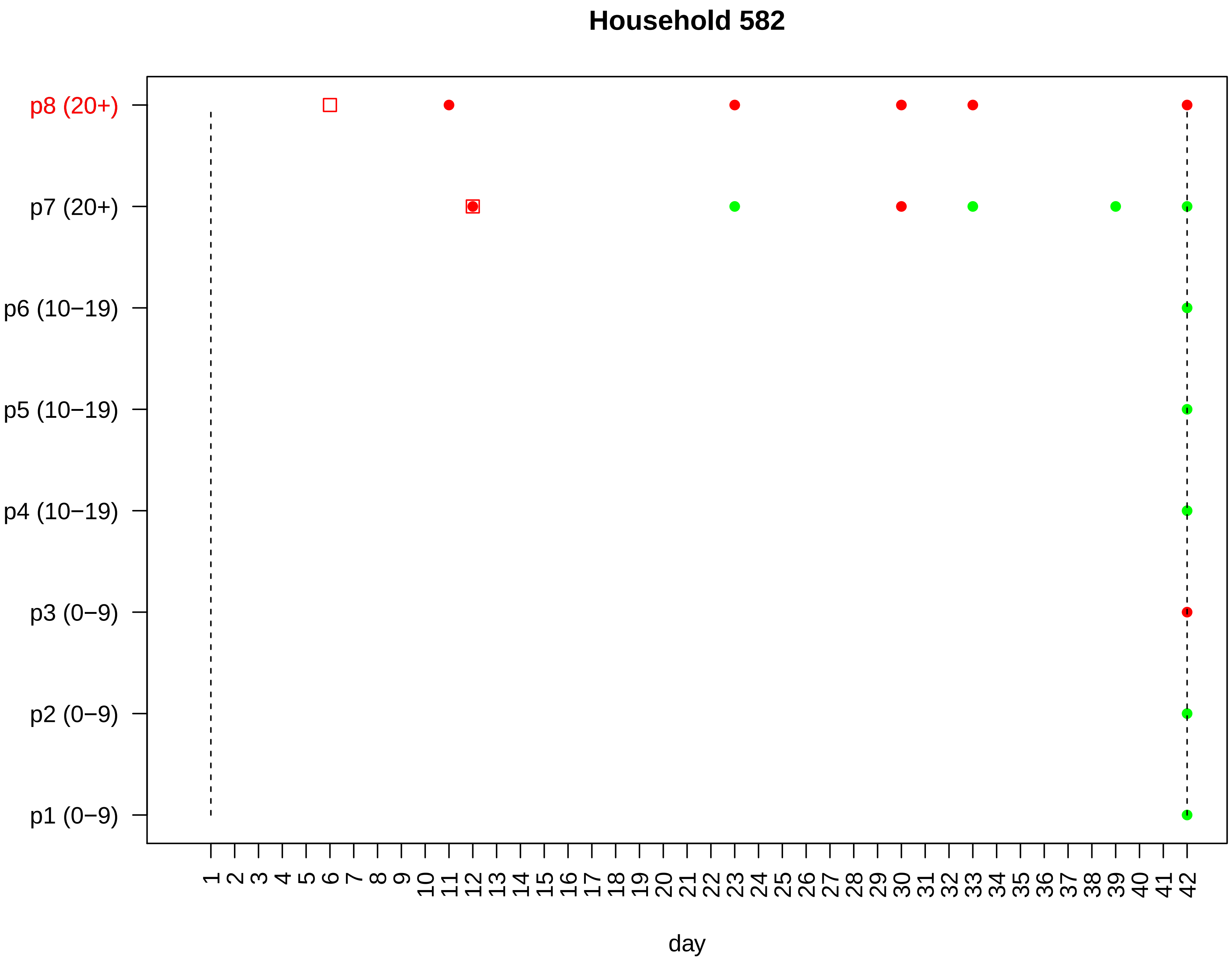
Household 582
p8 (20+)
p7 (20+)
p6 (10−19)
p5 (10−19)
p4 (10−19)
p3 (0−9)
p2 (0−9)
p1 (0−9)
1 2 3 4 5 6 7 8 9 10 11 12 13 14 15 16 17 18 19 20 21 22 23 24 25 26 27 28 29 30 31 32 33 34 35 36 37 38 39 40 41 42
day

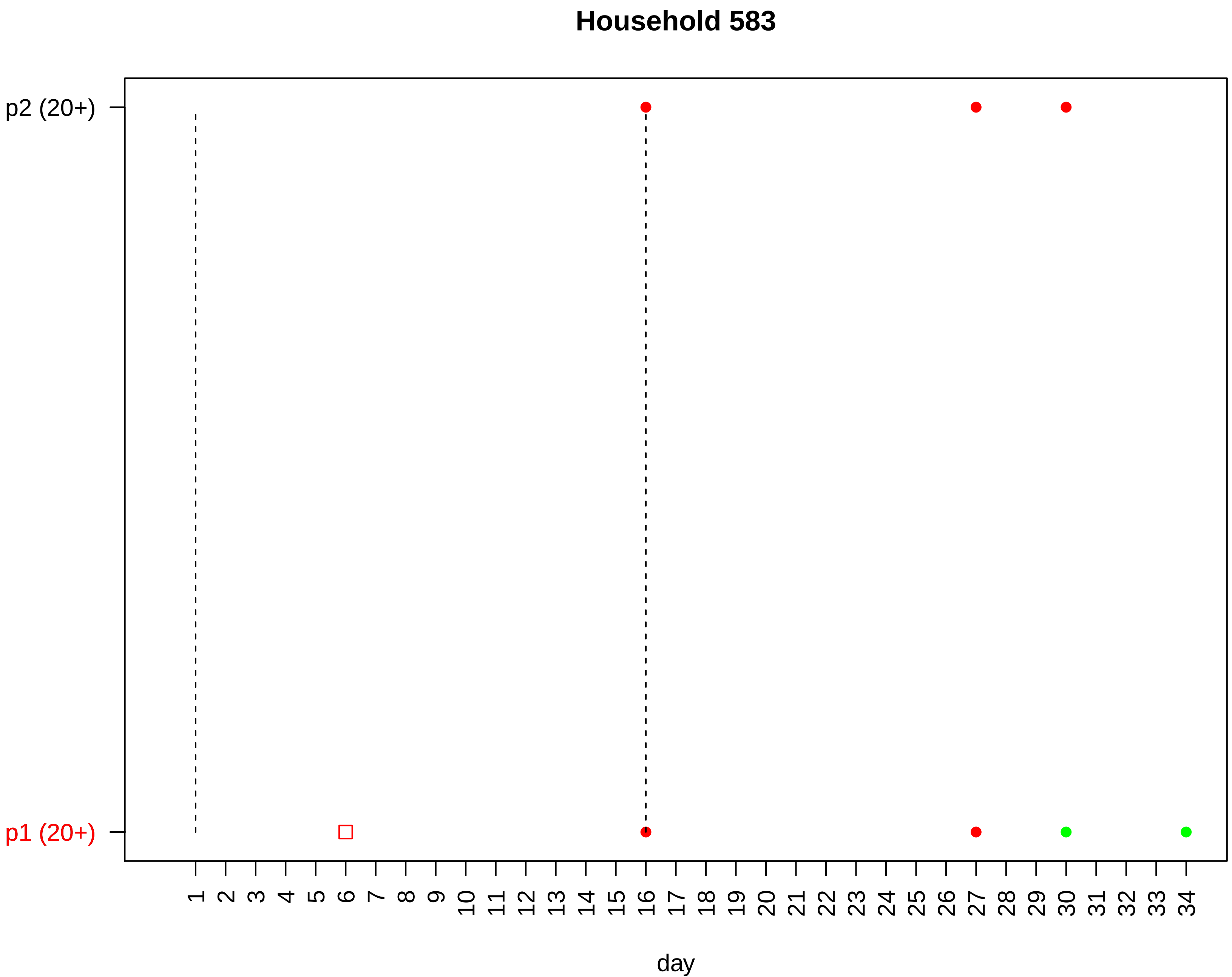

Household 583
p2 (20+)
p1 (20+)
1
2
3
4
5
6
7
8
9
10
11
12
13
14
15
16
17
18
19
20
21
22
23
24
25
26
27
28
29
30
31
32
33
34
day

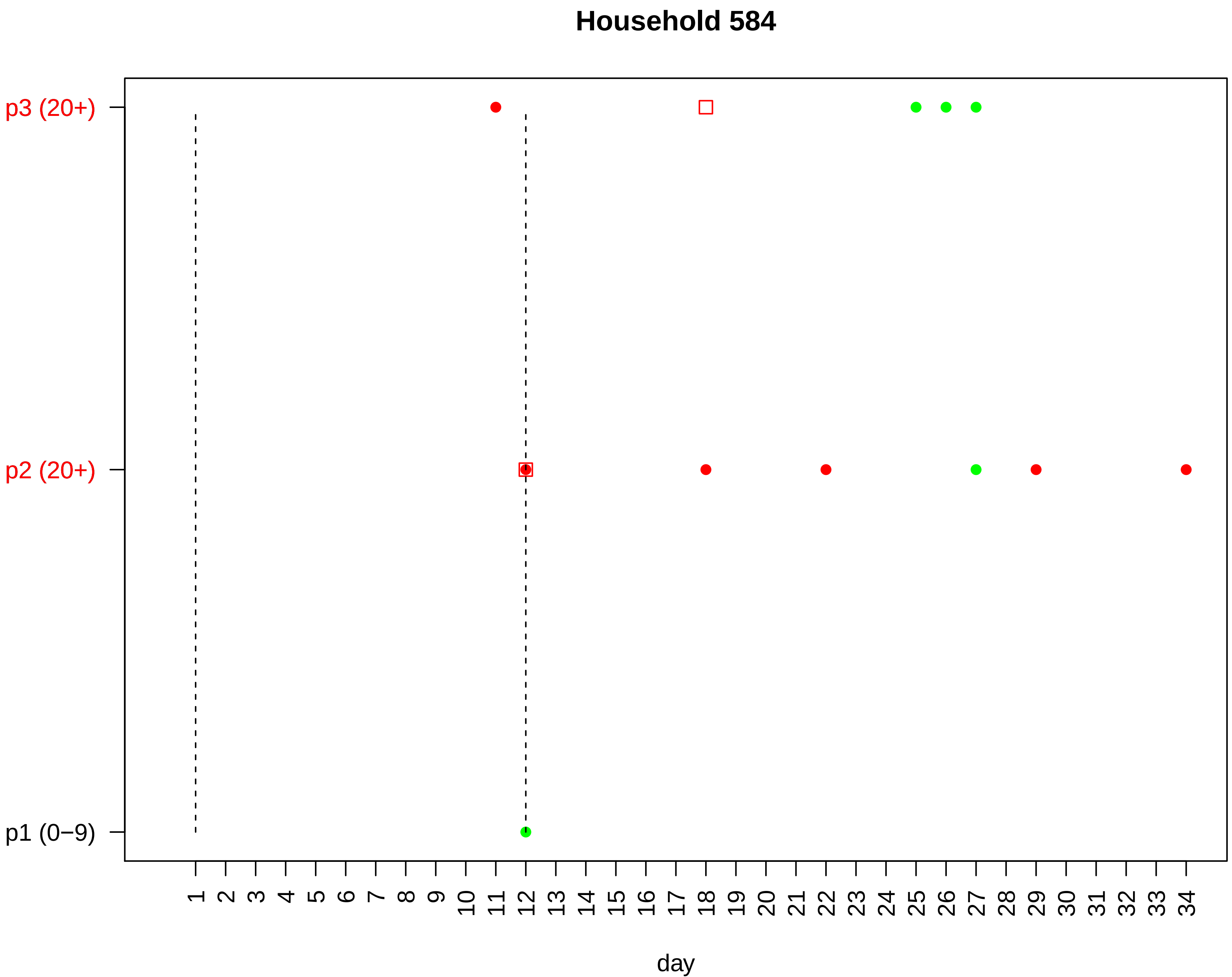
Household 584
p3 (20+)
p2 (20+)
p1 (0−9)
1
2
3
4
5
6
7
8
9
10
11
12
13
14
15
16
17
18
19
20
21
22
23
24
25
26
27
28
29
30
31
32
33
34
day

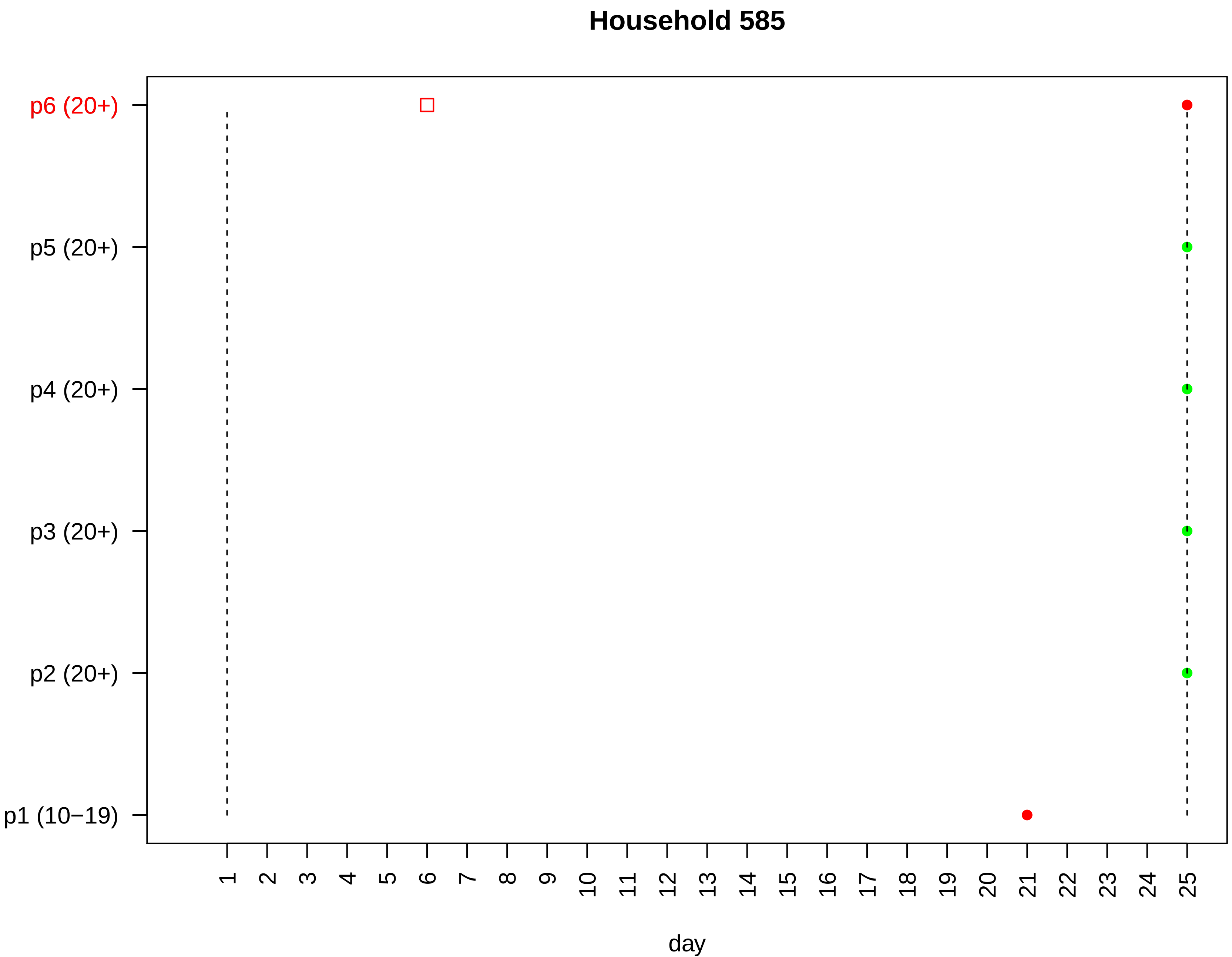

Household 585
p6 (20+)
p5 (20+)
p4 (20+)
p3 (20+)
p2 (20+)
p1 (10−19)
1
2
3
4
5
6
7
8
9
10
11
12
13
14
15
16
17
18
19
20
21
22
23
24
25
day

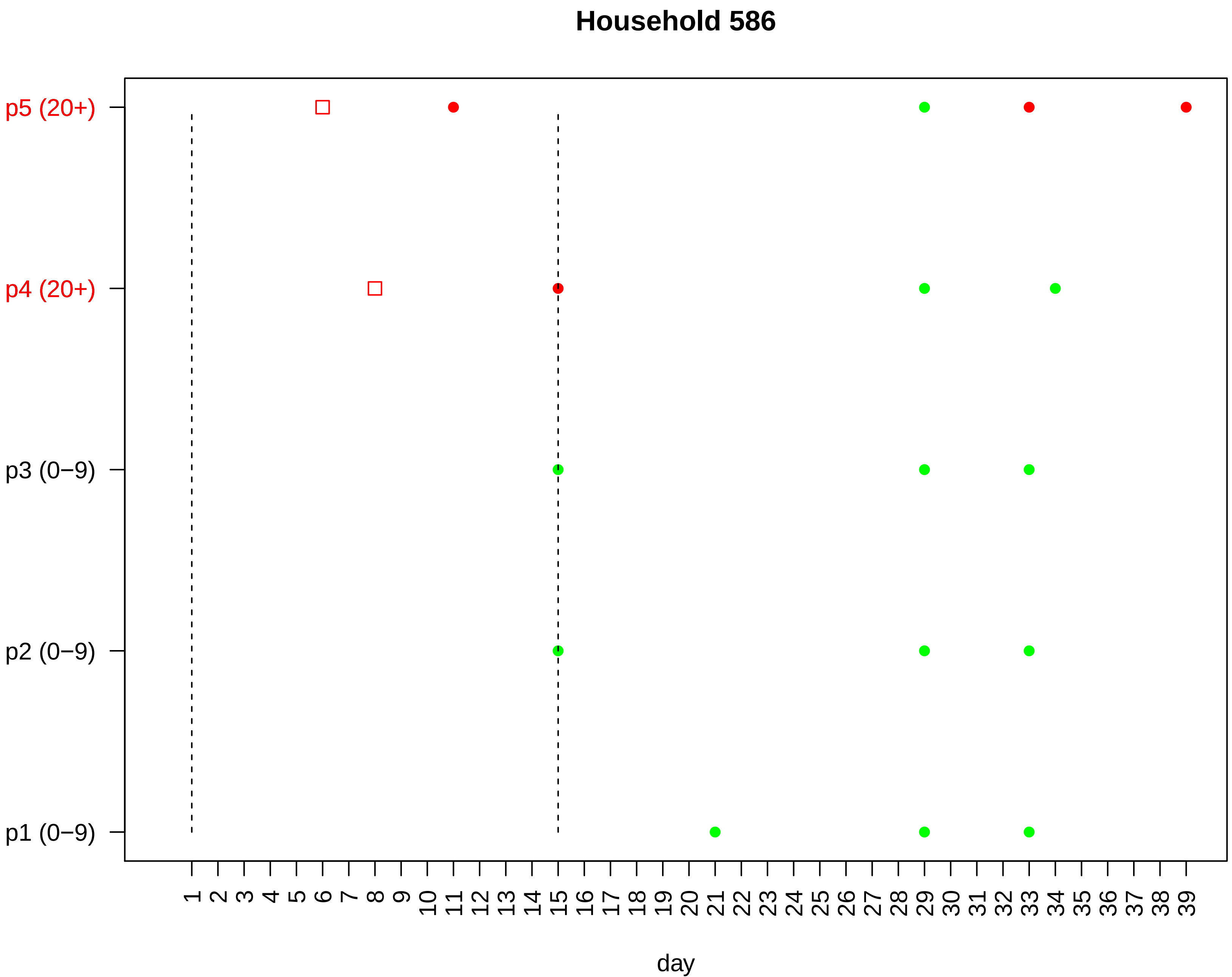
Household 586
p5 (20+)
p4 (20+)
p3 (0−9)
p2 (0−9)
p1 (0−9)
1 2 3 4 5 6 7 8 9 10 11 12 13 14 15 16 17 18 19 20 21 22 23 24 25 26 27 28 29 30 31 32 33 34 35 36 37 38 39
day

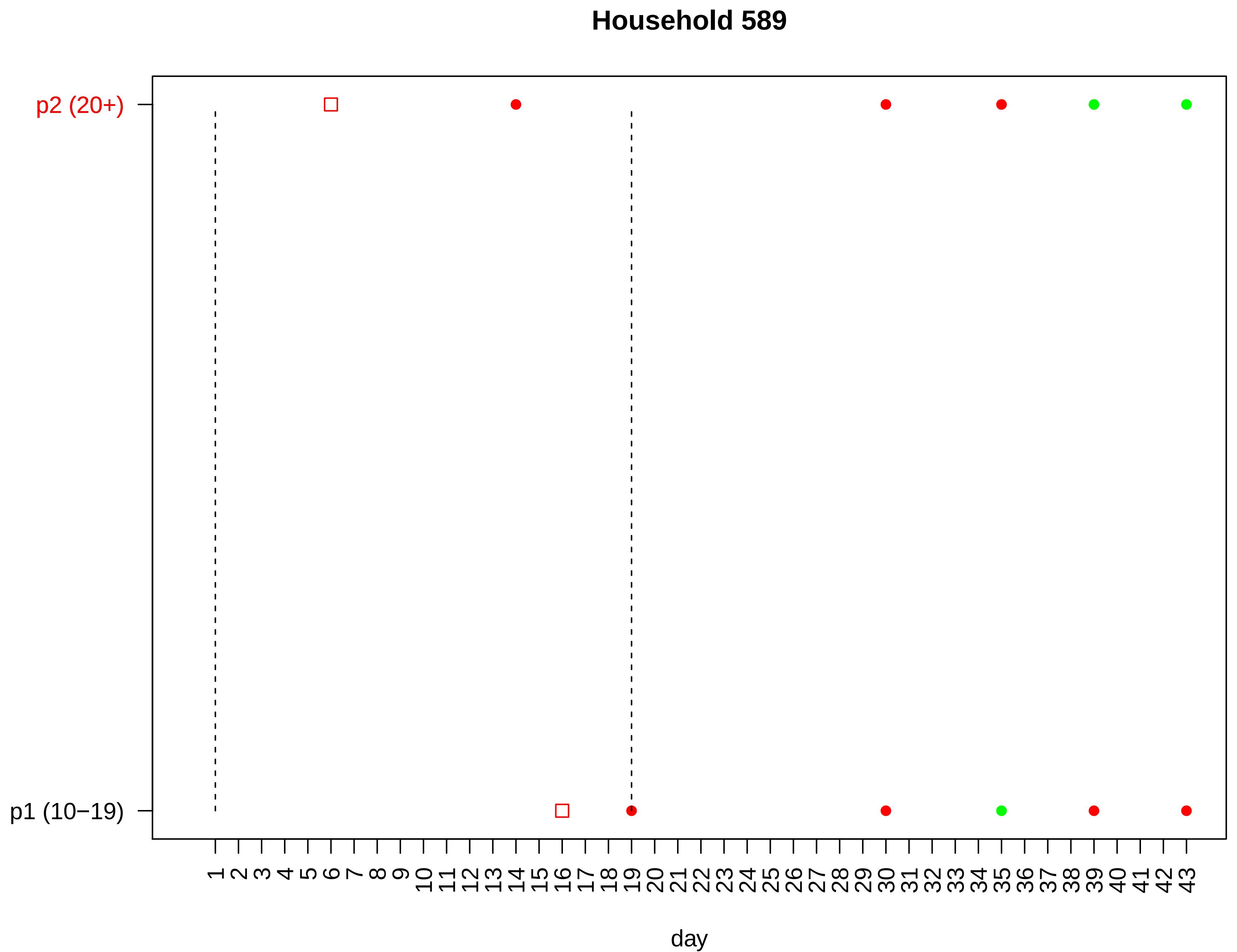

Household 589
p2 (20+)
p1 (10−19)
1 2 3 4 5 6 7 8 9 10 11 12 13 14 15 16 17 18 19 20 21 22 23 24 25 26 27 28 29 30 31 32 33 34 35 36 37 38 39 40 41 42 43
day

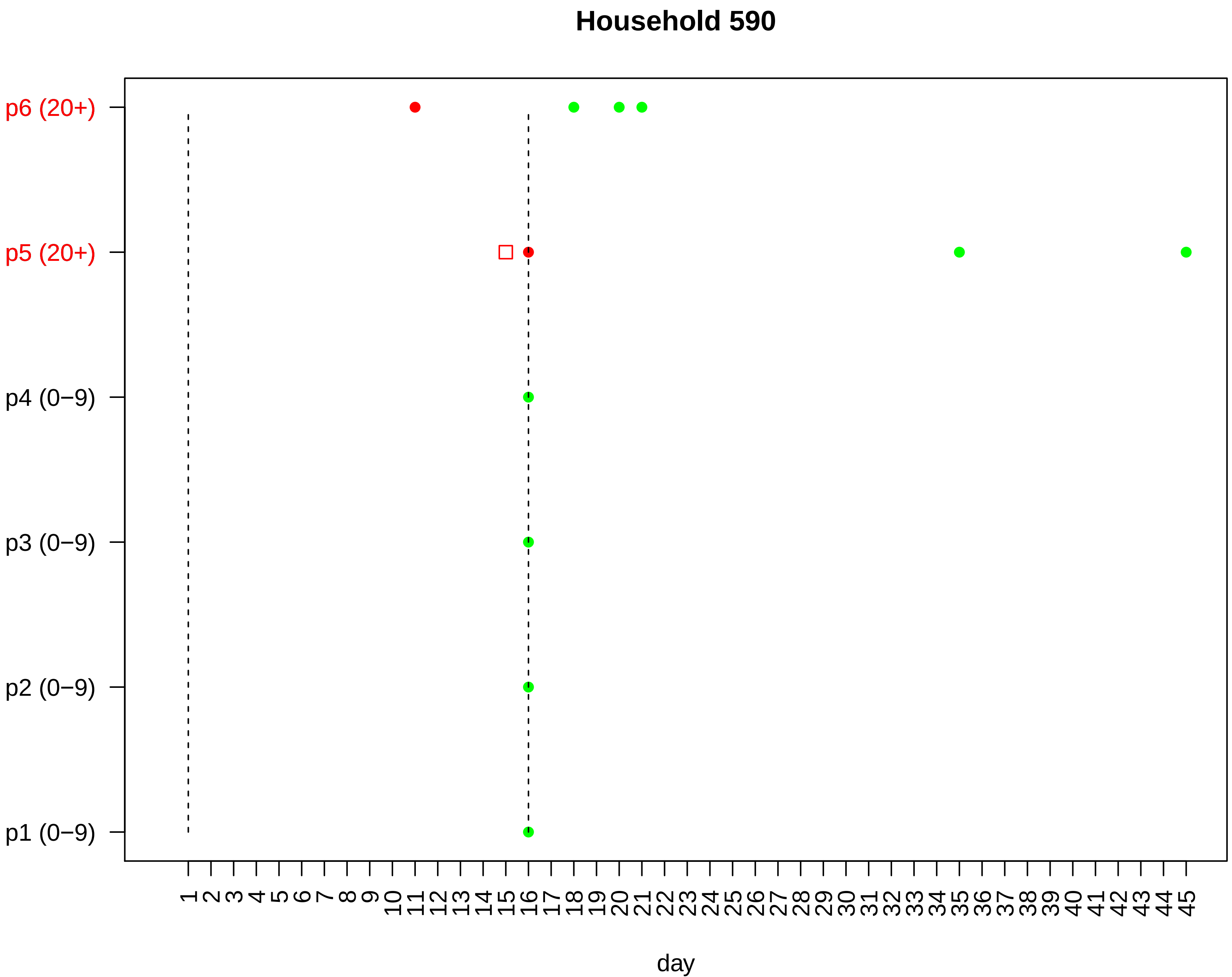

Household 590
p6 (20+)
p5 (20+)
p4 (0−9)
p3 (0−9)
p2 (0−9)
p1 (0−9)
1 2 3 4 5 6 7 8 9 10 11 12 13 14 15 16 17 18 19 20 21 22 23 24 25 26 27 28 29 30 31 32 33 34 35 36 37 38 39 40 41 42 43 44 45
day

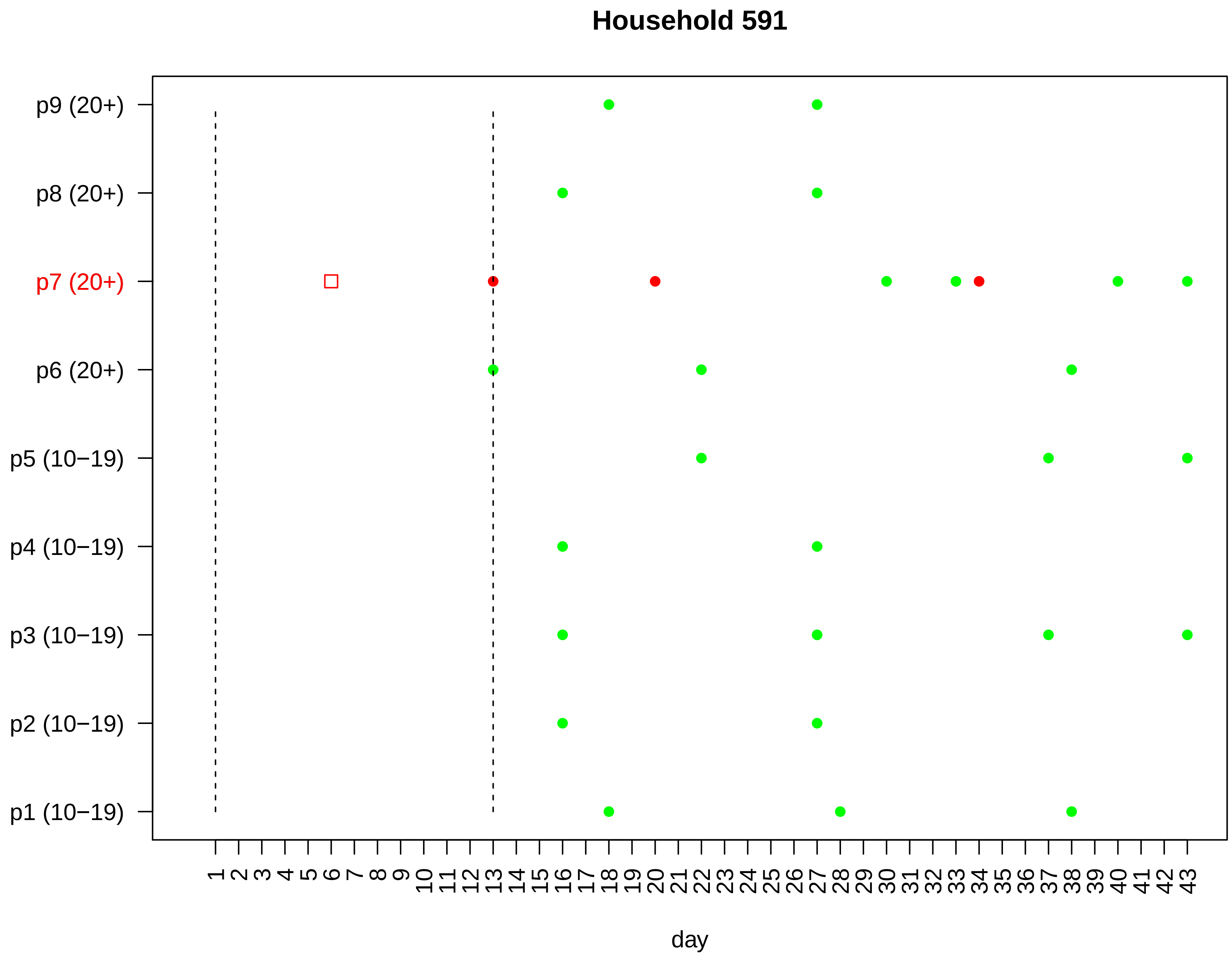

Household 591
p9 (20+)
p8 (20+)
p7 (20+)
p6 (20+)
p5 (10−19)
p4 (10−19)
p3 (10−19)
p2 (10−19)
p1 (10−19)
1 2 3 4 5 6 7 8 9 10 11 12 13 14 15 16 17 18 19 20 21 22 23 24 25 26 27 28 29 30 31 32 33 34 35 36 37 38 39 40 41 42 43
day

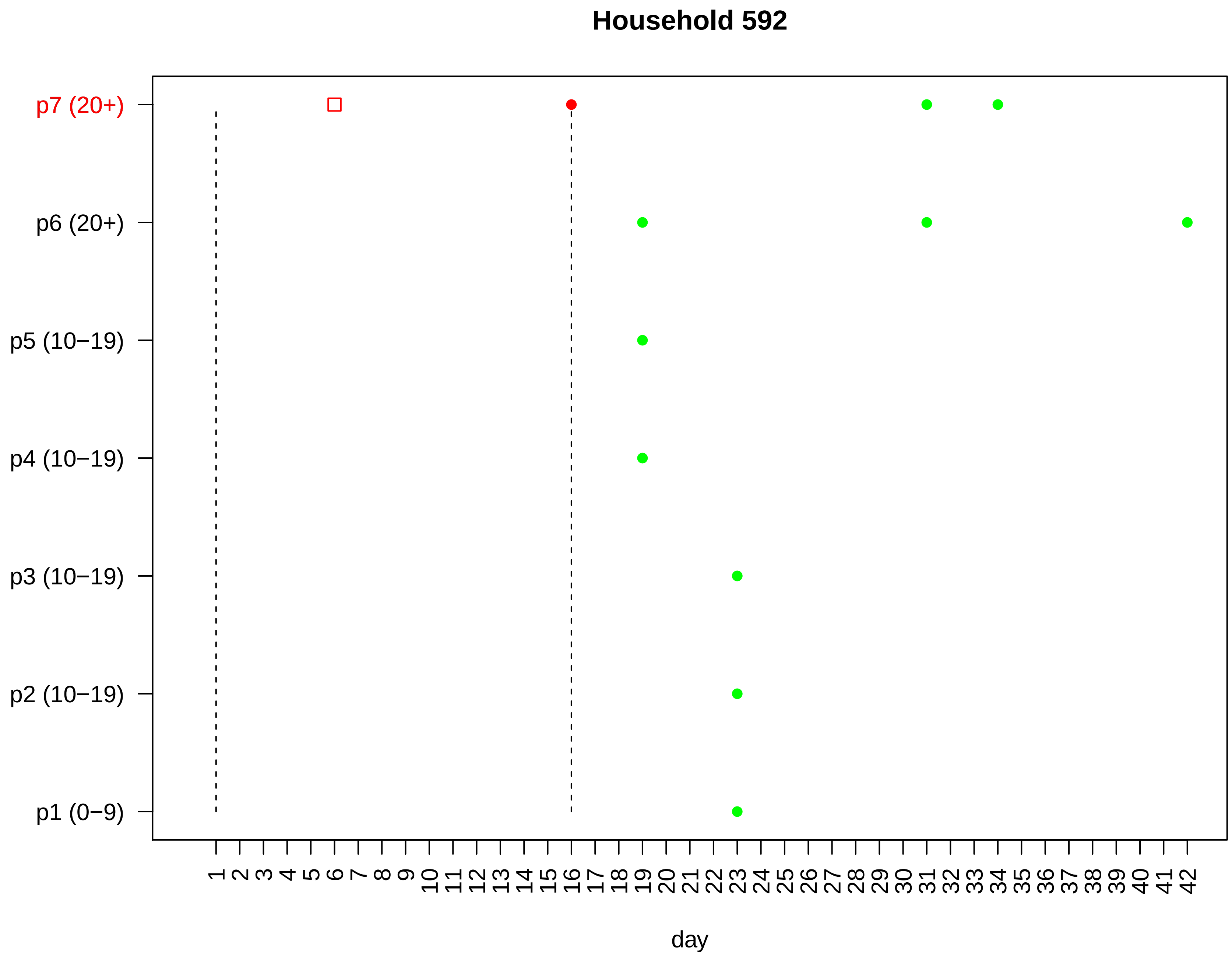

Household 592
p7 (20+)
p6 (20+)
p5 (10−19)
p4 (10−19)
p3 (10−19)
p2 (10−19)
p1 (0−9)
1 2 3 4 5 6 7 8 9 10 11 12 13 14 15 16 17 18 19 20 21 22 23 24 25 26 27 28 29 30 31 32 33 34 35 36 37 38 39 40 41 42
day

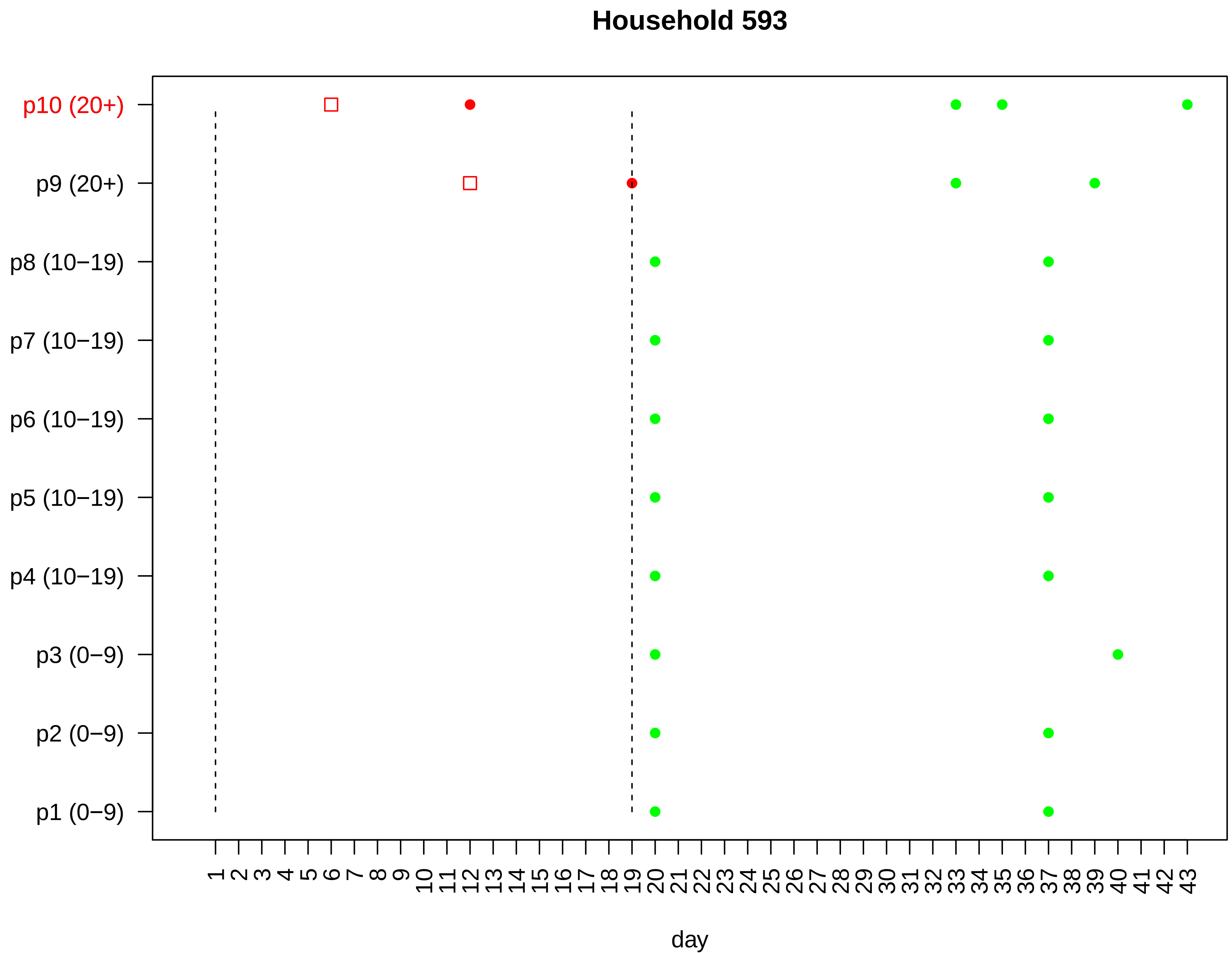

Household 593
p10 (20+)
p9 (20+)
p8 (10−19)
p7 (10−19)
p6 (10−19)
p5 (10−19)
p4 (10−19)
p3 (0−9)
p2 (0−9)
p1 (0−9)
1 2 3 4 5 6 7 8 9 10 11 12 13 14 15 16 17 18 19 20 21 22 23 24 25 26 27 28 29 30 31 32 33 34 35 36 37 38 39 40 41 42 43
day

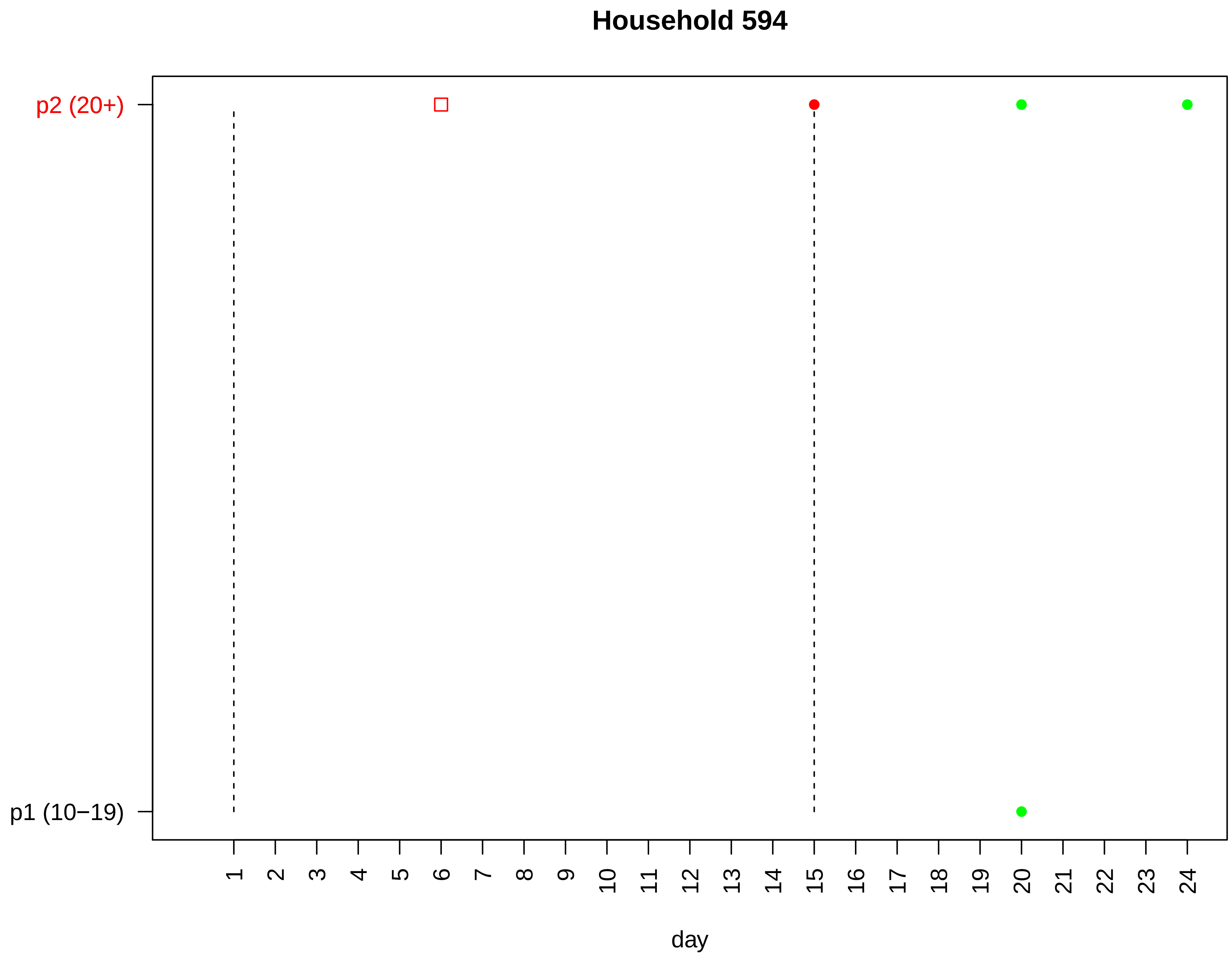

Household 594
p2 (20+)
p1 (10−19)
1
2
3
4
5
6
7
8
9
10
11
12
13
14
15
16
17
18
19
20
21
22
23
24
day

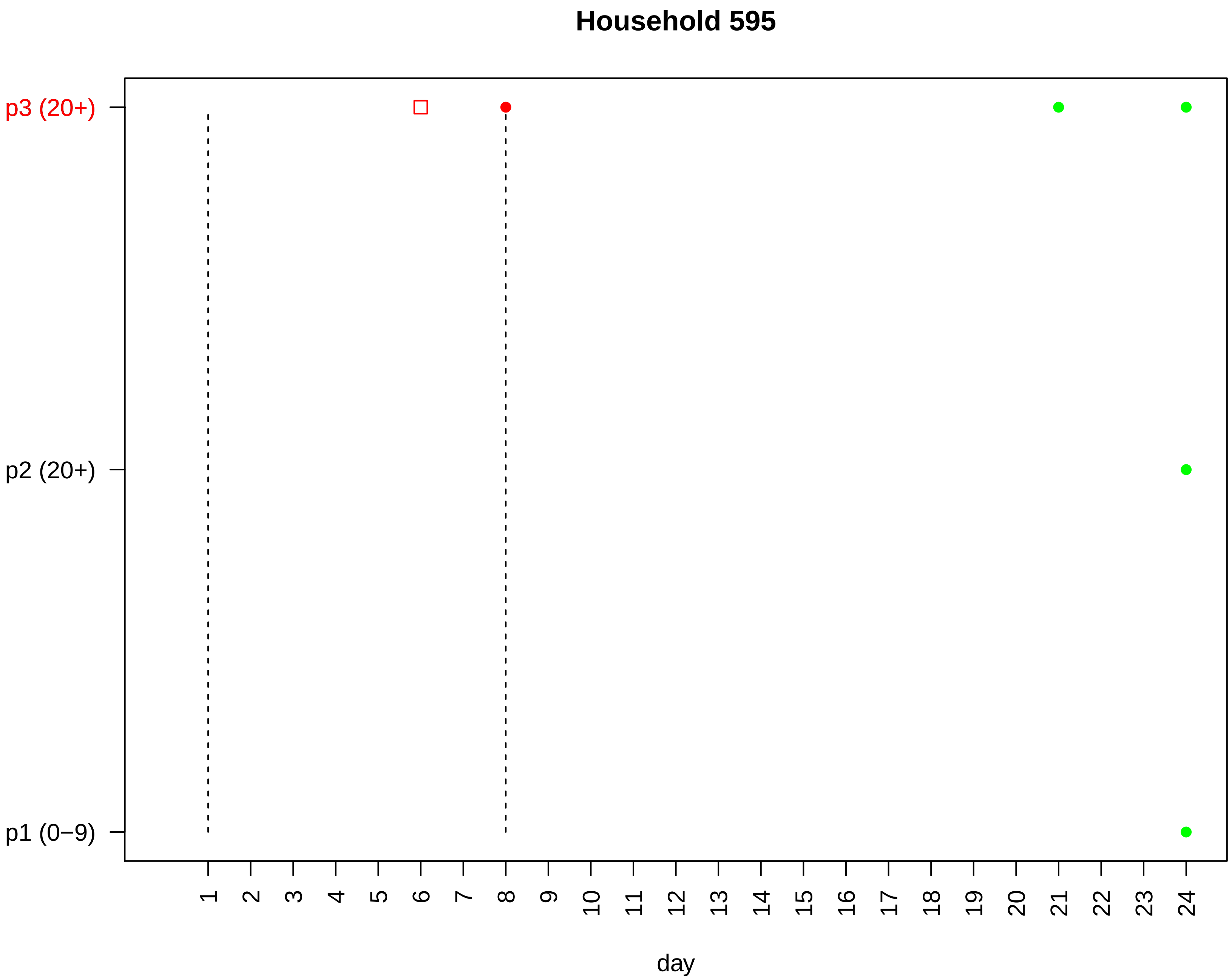
Household 595
p3 (20+)
p2 (20+)
p1 (0−9)
1
2
3
4
5
6
7
8
9
10
11
12
13
14
15
16
17
18
19
20
21
22
23
24
day

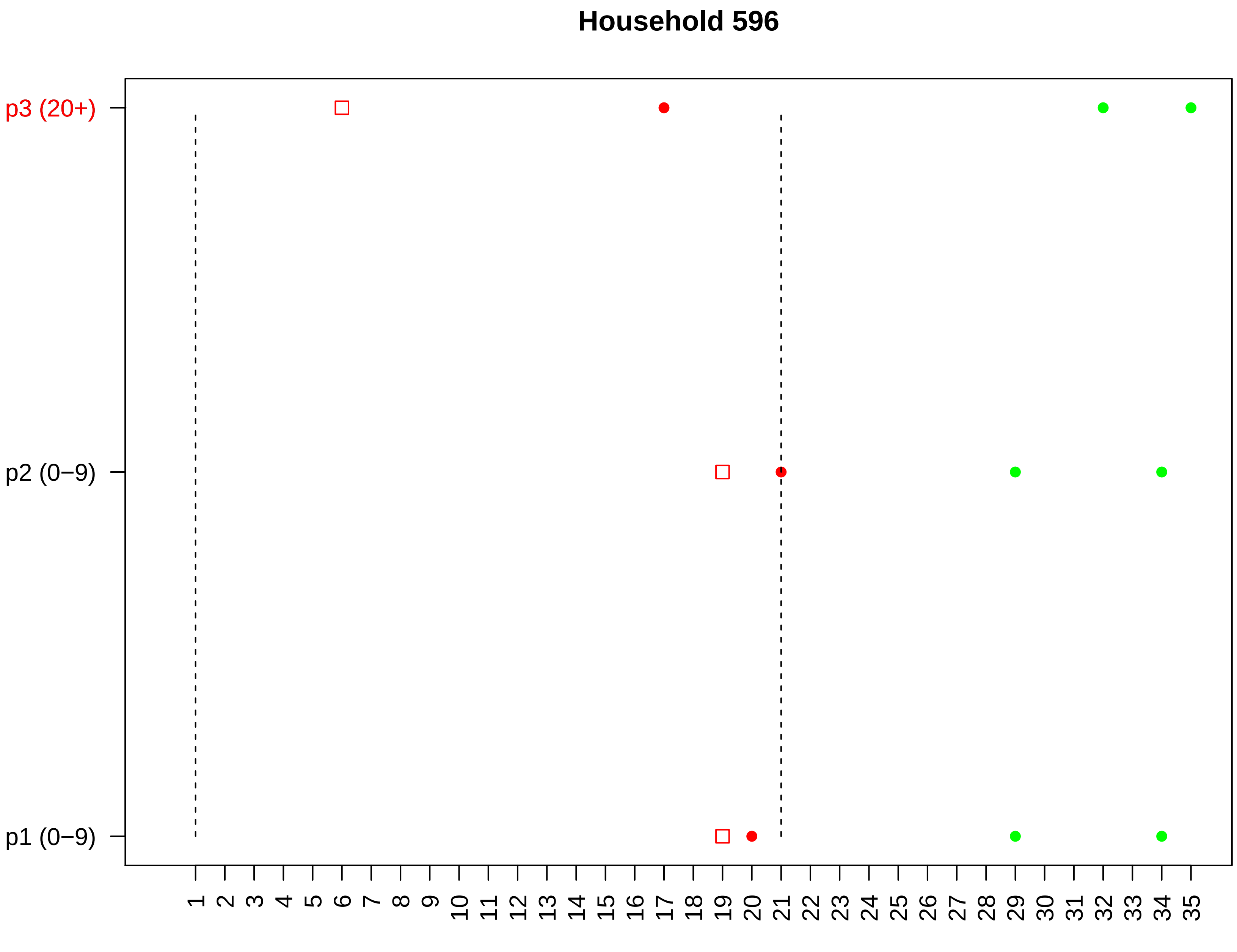

Household 596
p3 (20+)
p2 (0−9)
p1 (0−9)
1 2 3 4 5 6 7 8 9 10 11 12 13 14 15 16 17 18 19 20 21 22 23 24 25 26 27 28 29 30 31 32 33 34 35
day

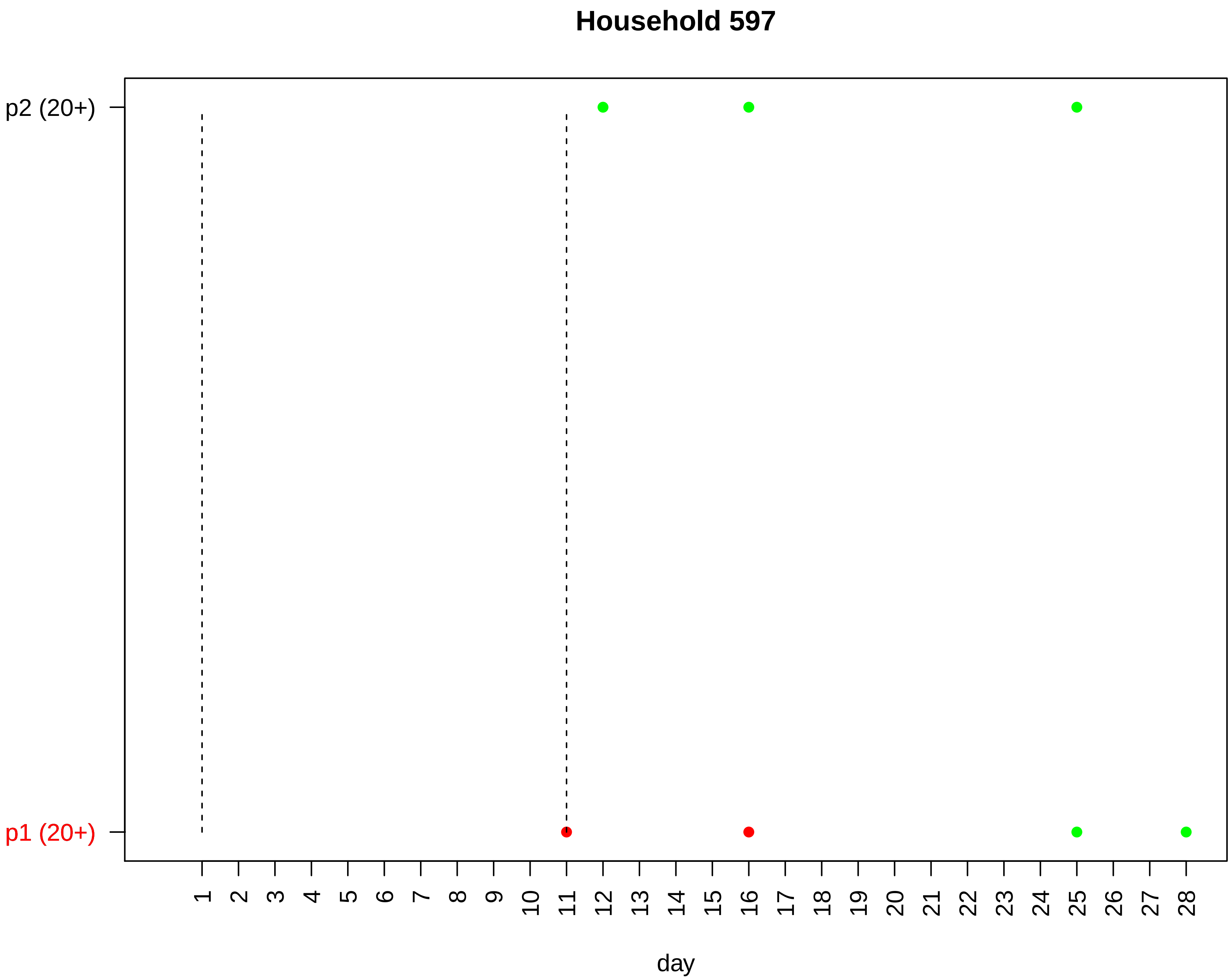
Household 597
p2 (20+)
p1 (20+)
1
2
3
4
5
6
7
8
9
10
11
12
13
14
15
16
17
18
19
20
21
22
23
24
25
26
27
28
day

Household 598

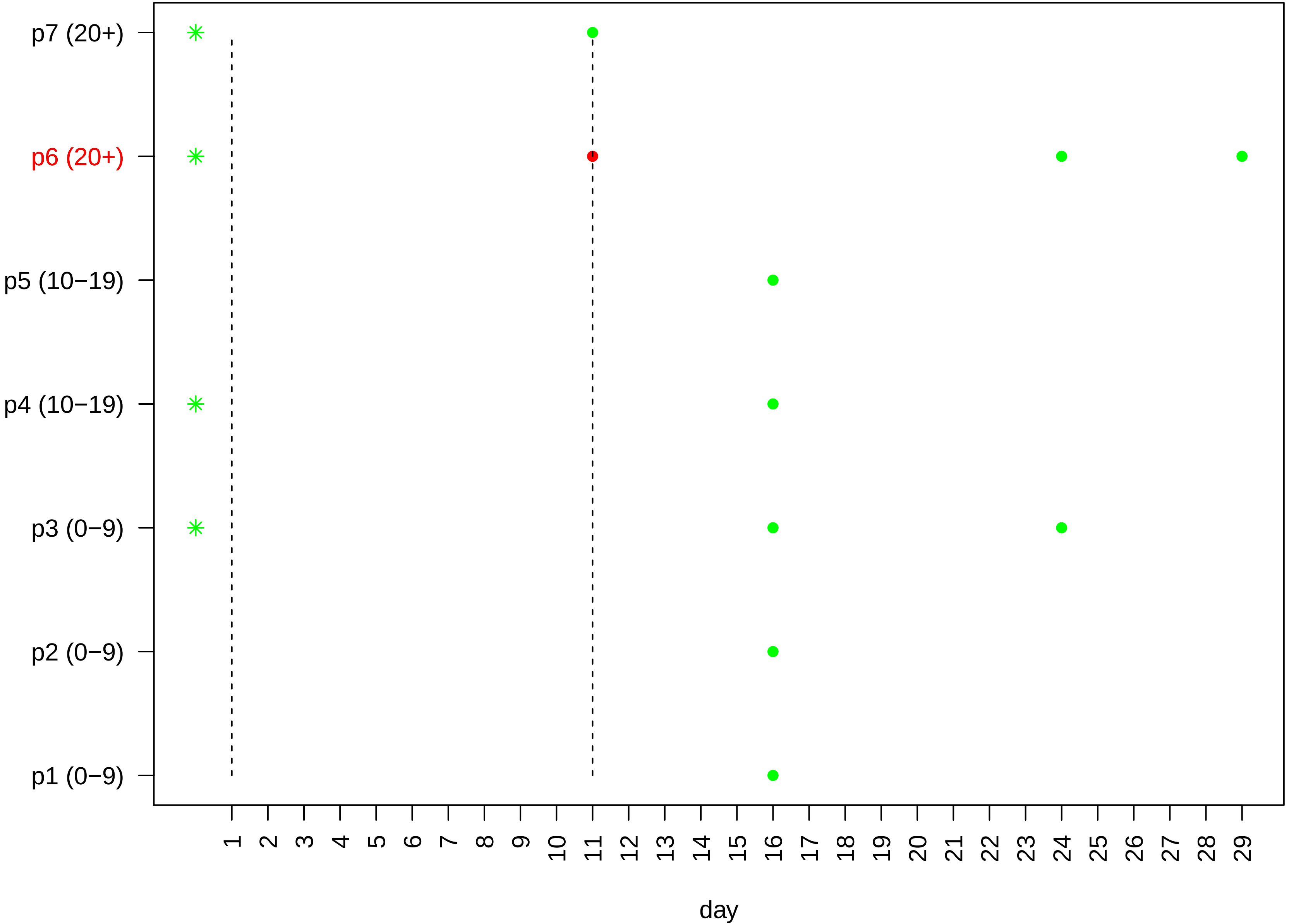

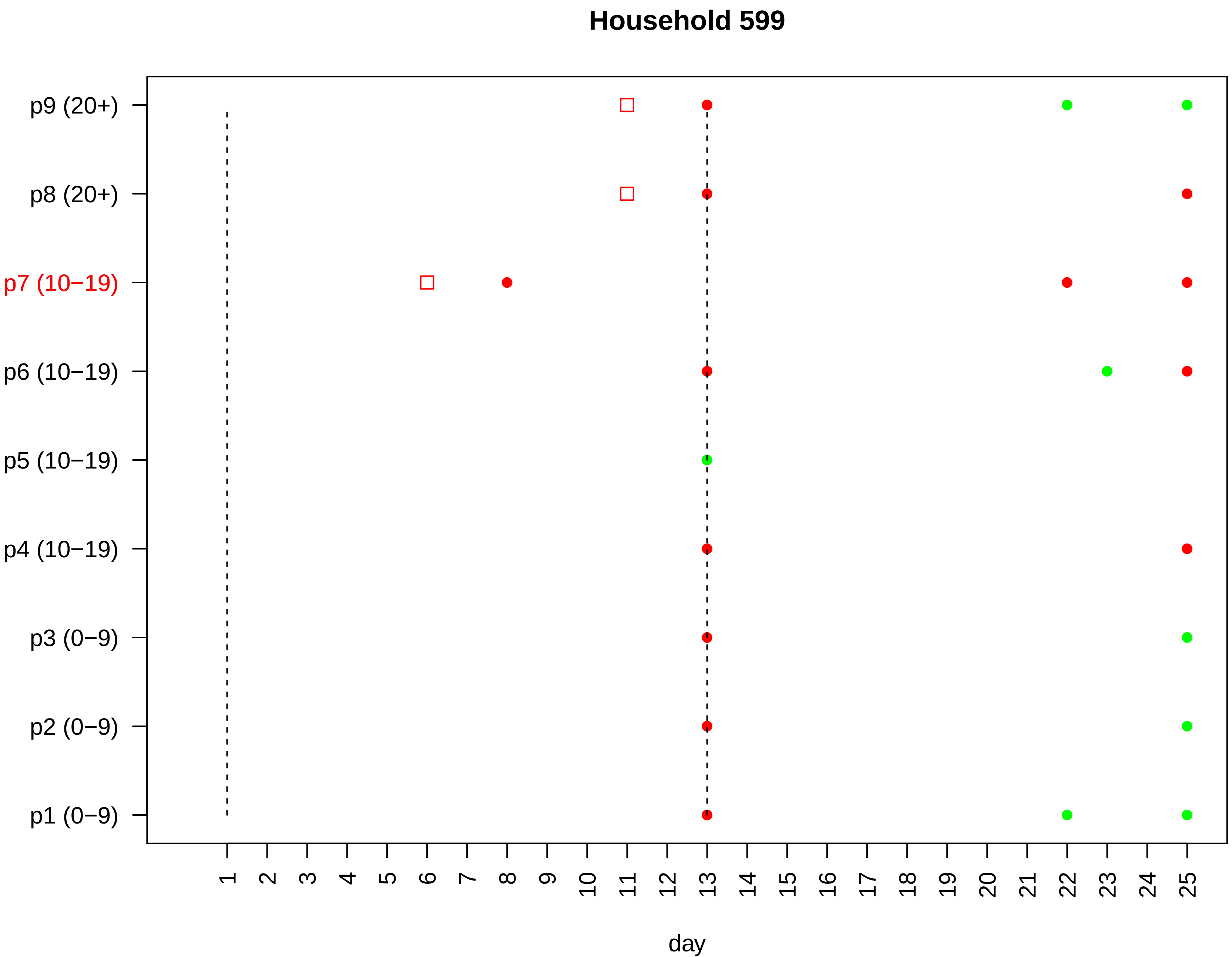

Household 599
p9 (20+)
p8 (20+)
p7 (10−19)
p6 (10−19)
p5 (10−19)
p4 (10−19)
p3 (0−9)
p2 (0−9)
p1 (0−9)
1 2 3 4 5 6 7 8 9 10 11 12 13 14 15 16 17 18 19 20 21 22 23 24 25
day

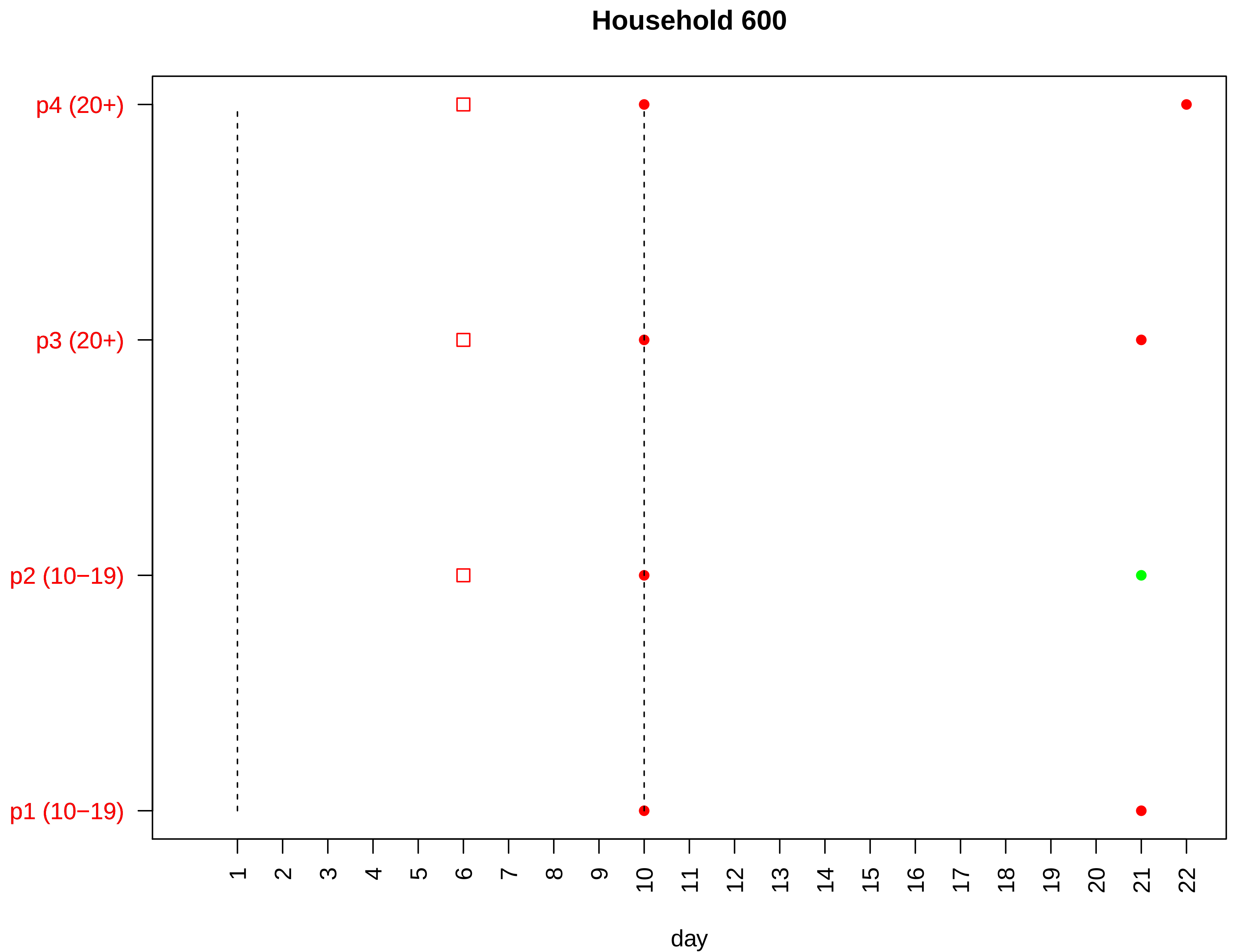

Household 600
p4 (20+)
p3 (20+)
p2 (10−19)
p1 (10−19)
1
2
3
4
5
6
7
8
9
10
11
12
13
14
15
16
17
18
19
20
21
22
day

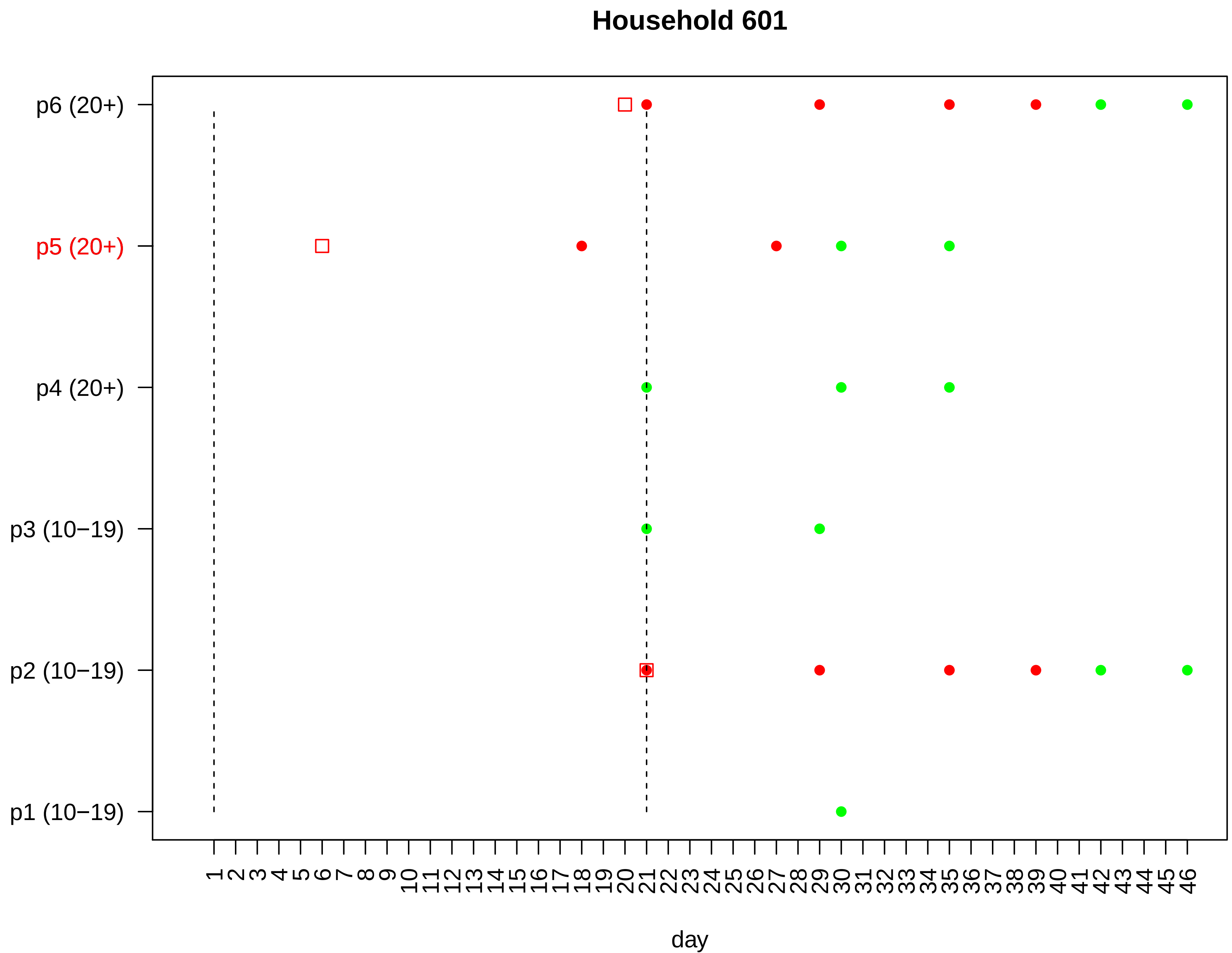

Household 601
p6 (20+)
p5 (20+)
p4 (20+)
p3 (10−19)
p2 (10−19)
p1 (10−19)
1 2 3 4 5 6 7 8 9 10 11 12 13 14 15 16 17 18 19 20 21 22 23 24 25 26 27 28 29 30 31 32 33 34 35 36 37 38 39 40 41 42 43 44 45 46
day

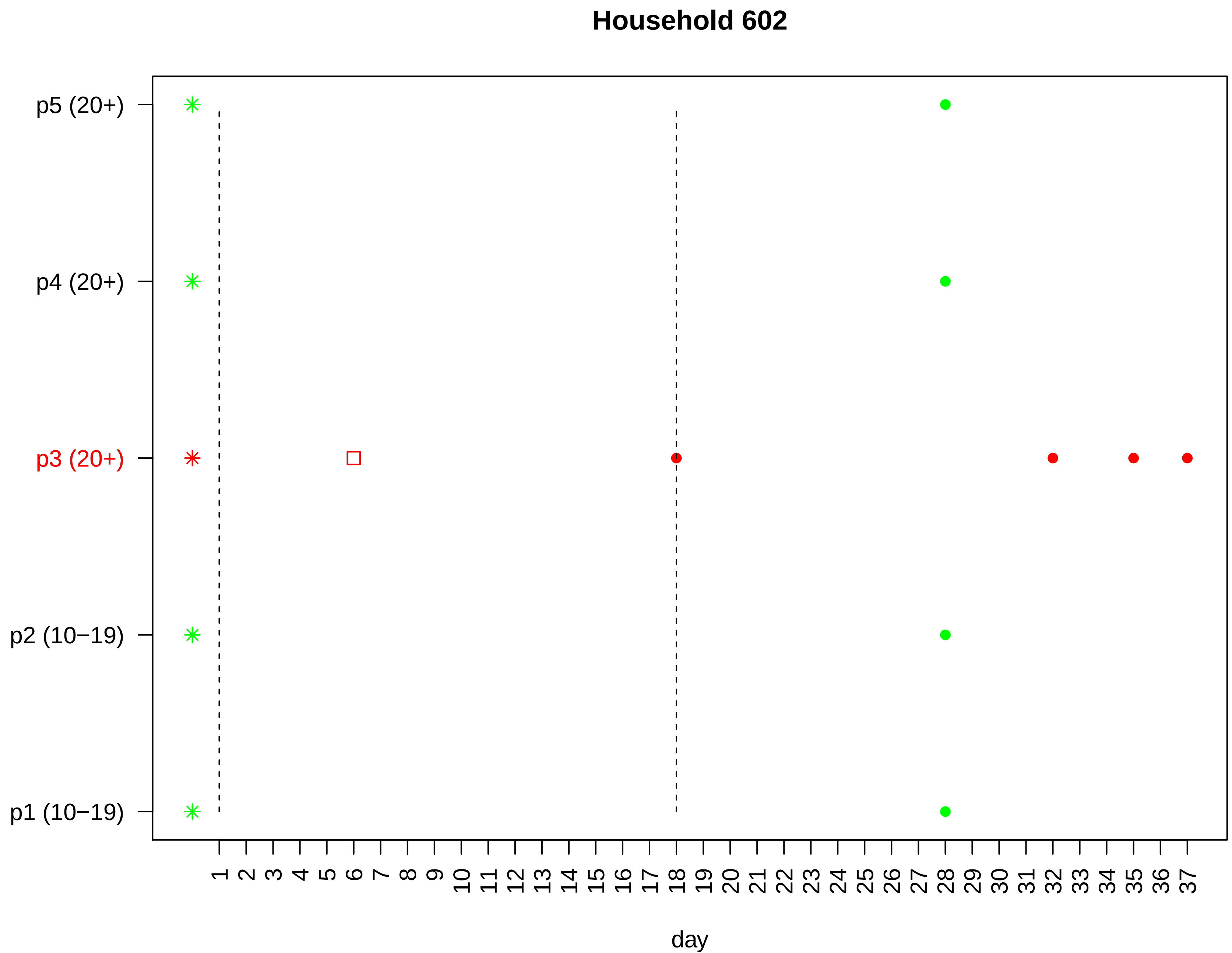
Household 602
p5 (20+)
p4 (20+)
p3 (20+)
p2 (10−19)
p1 (10−19)
1 2 3 4 5 6 7 8 9 10 11 12 13 14 15 16 17 18 19 20 21 22 23 24 25 26 27 28 29 30 31 32 33 34 35 36 37
day

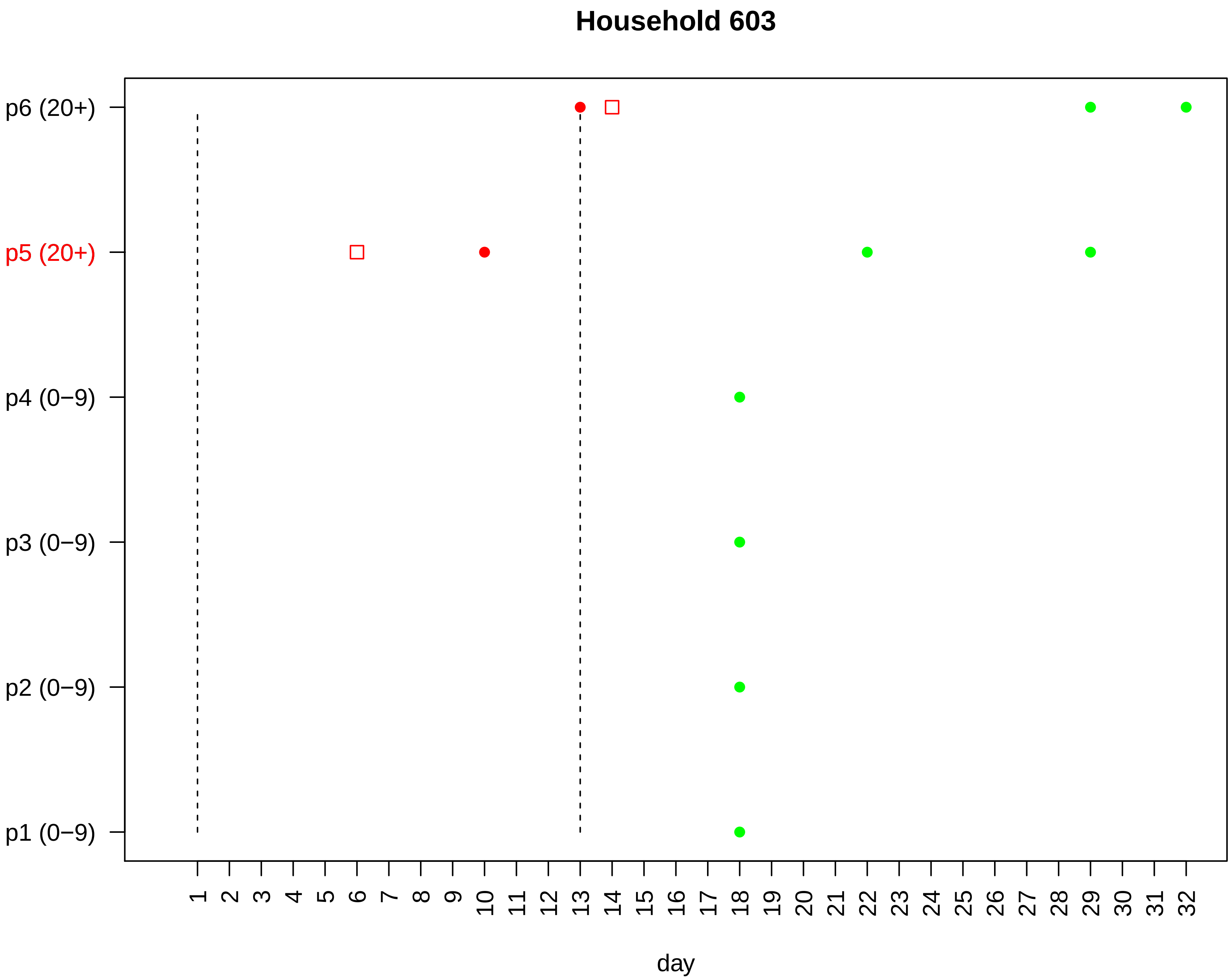

Household 603
p6 (20+)
p5 (20+)
p4 (0−9)
p3 (0−9)
p2 (0−9)
p1 (0−9)
1 2 3 4 5 6 7 8 9 10 11 12 13 14 15 16 17 18 19 20 21 22 23 24 25 26 27 28 29 30 31 32
day

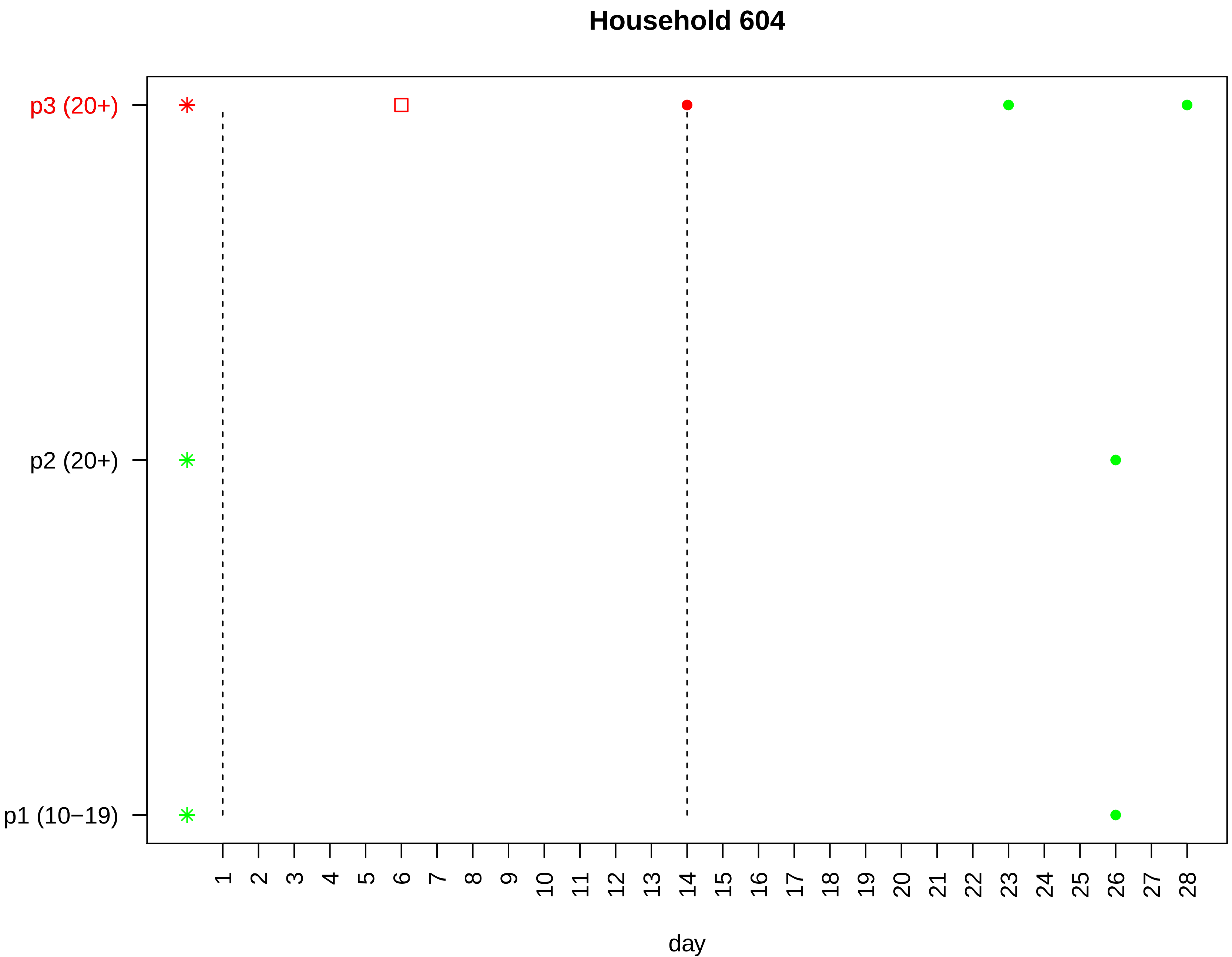

Household 604
p3 (20+)
p2 (20+)
p1 (10−19)
1
2
3
4
5
6
7
8
9
10
11
12
13
14
15
16
17
18
19
20
21
22
23
24
25
26
27
28
day

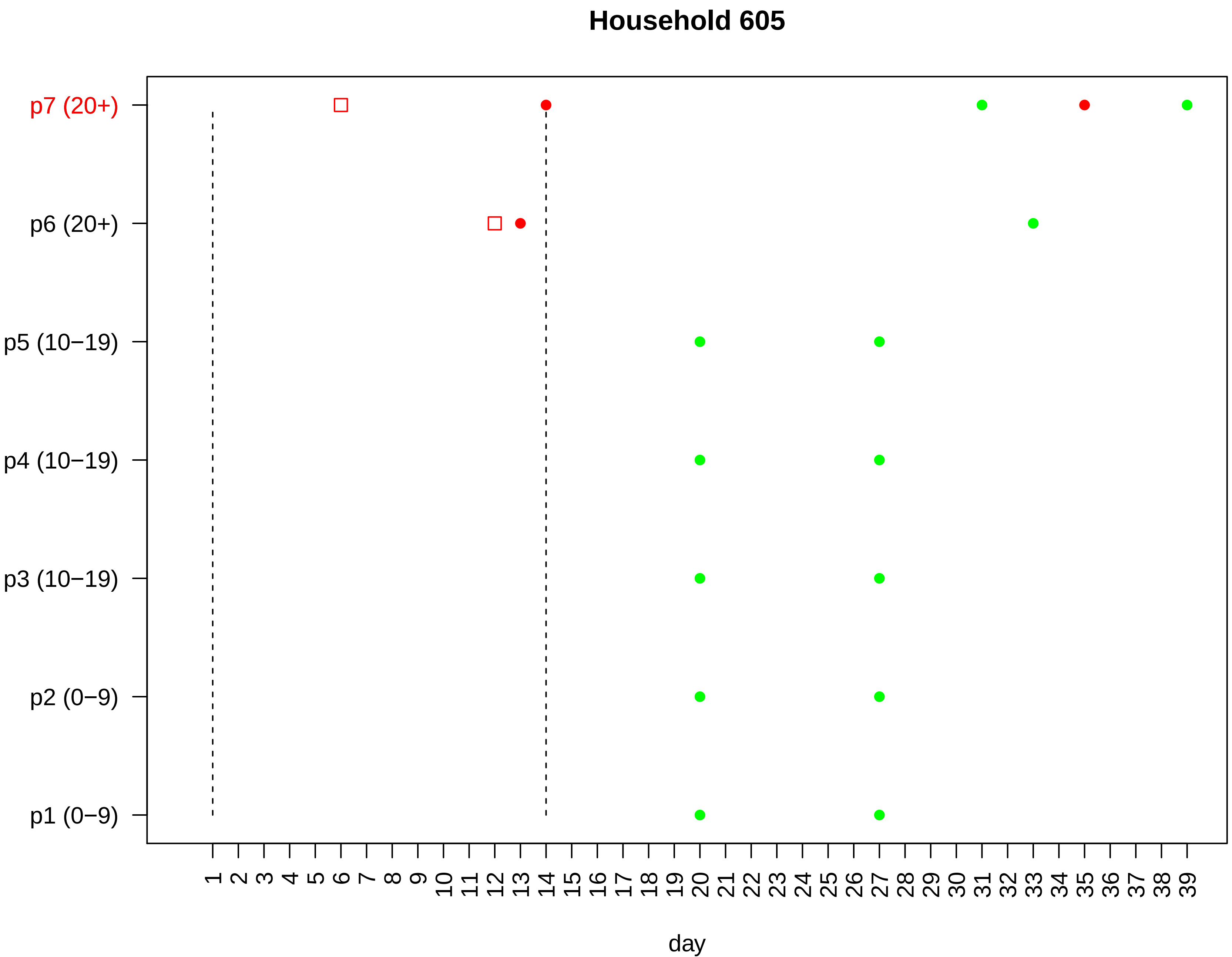

Household 605
p7 (20+)
p6 (20+)
p5 (10−19)
p4 (10−19)
p3 (10−19)
p2 (0−9)
p1 (0−9)
1 2 3 4 5 6 7 8 9 10 11 12 13 14 15 16 17 18 19 20 21 22 23 24 25 26 27 28 29 30 31 32 33 34 35 36 37 38 39
day

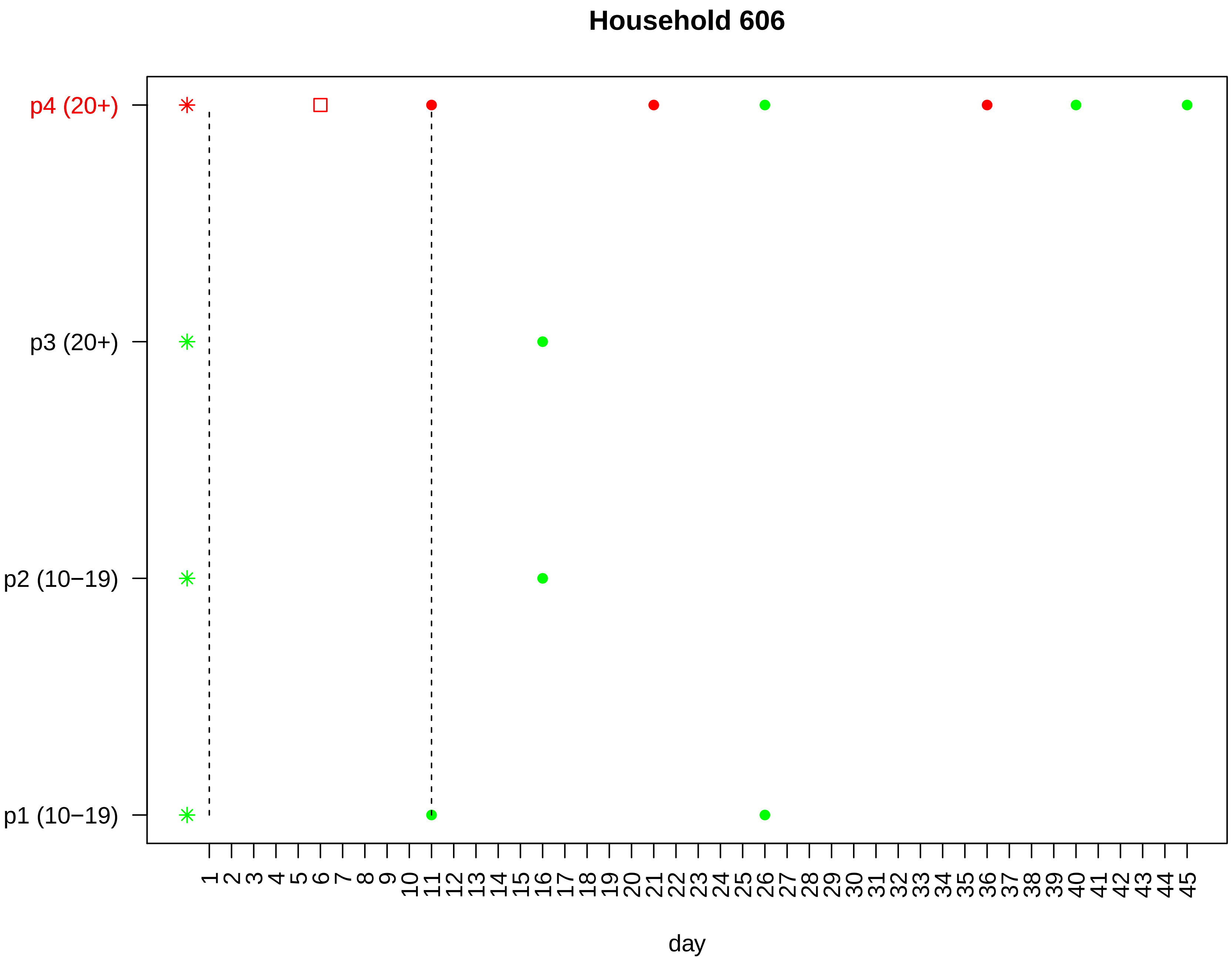
Household 606
p4 (20+)
p3 (20+)
p2 (10−19)
p1 (10−19)
1 2 3 4 5 6 7 8 9 10 11 12 13 14 15 16 17 18 19 20 21 22 23 24 25 26 27 28 29 30 31 32 33 34 35 36 37 38 39 40 41 42 43 44 45
day

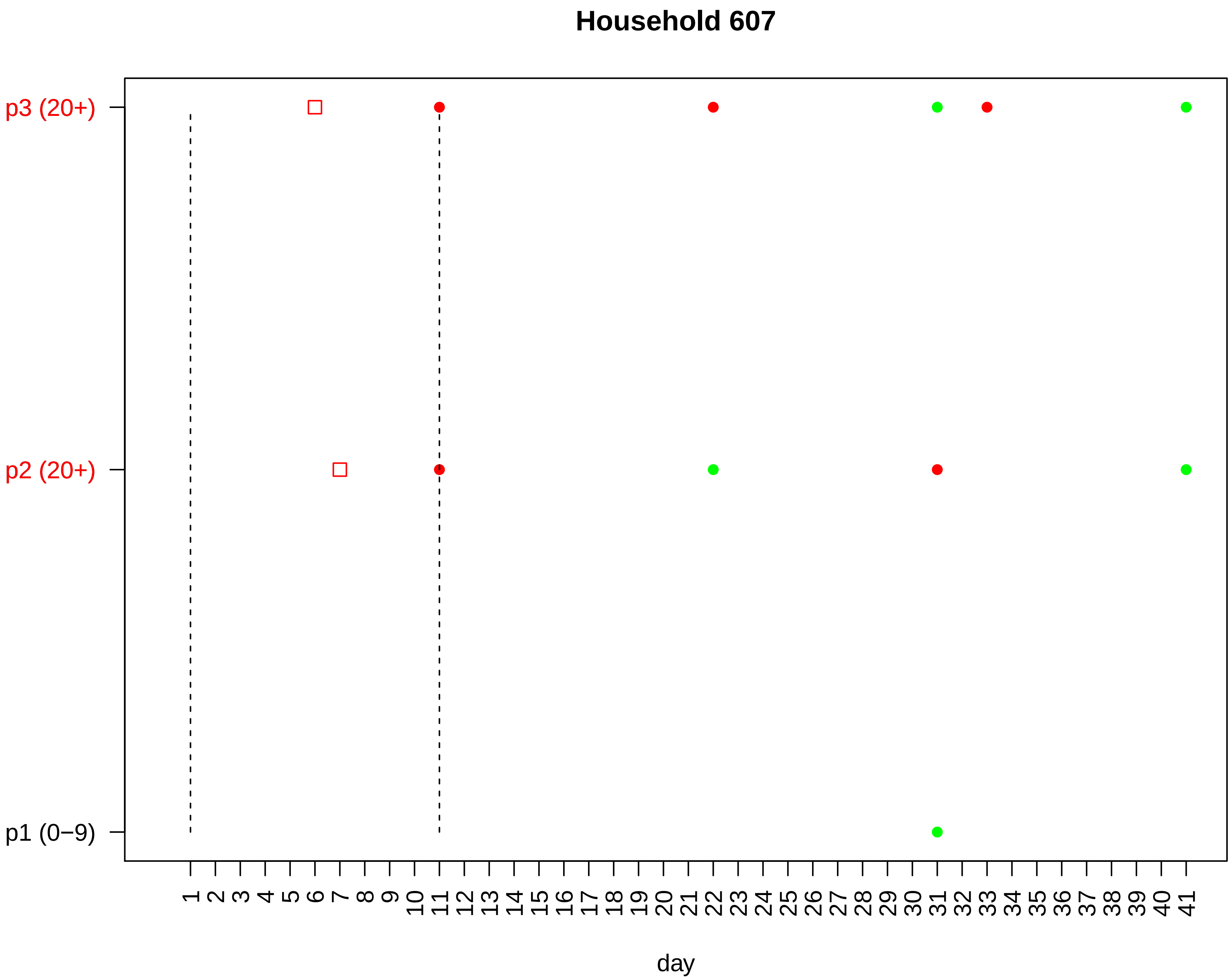

Household 607
p3 (20+)
p2 (20+)
p1 (0−9)
1 2 3 4 5 6 7 8 9 10 11 12 13 14 15 16 17 18 19 20 21 22 23 24 25 26 27 28 29 30 31 32 33 34 35 36 37 38 39 40 41
day

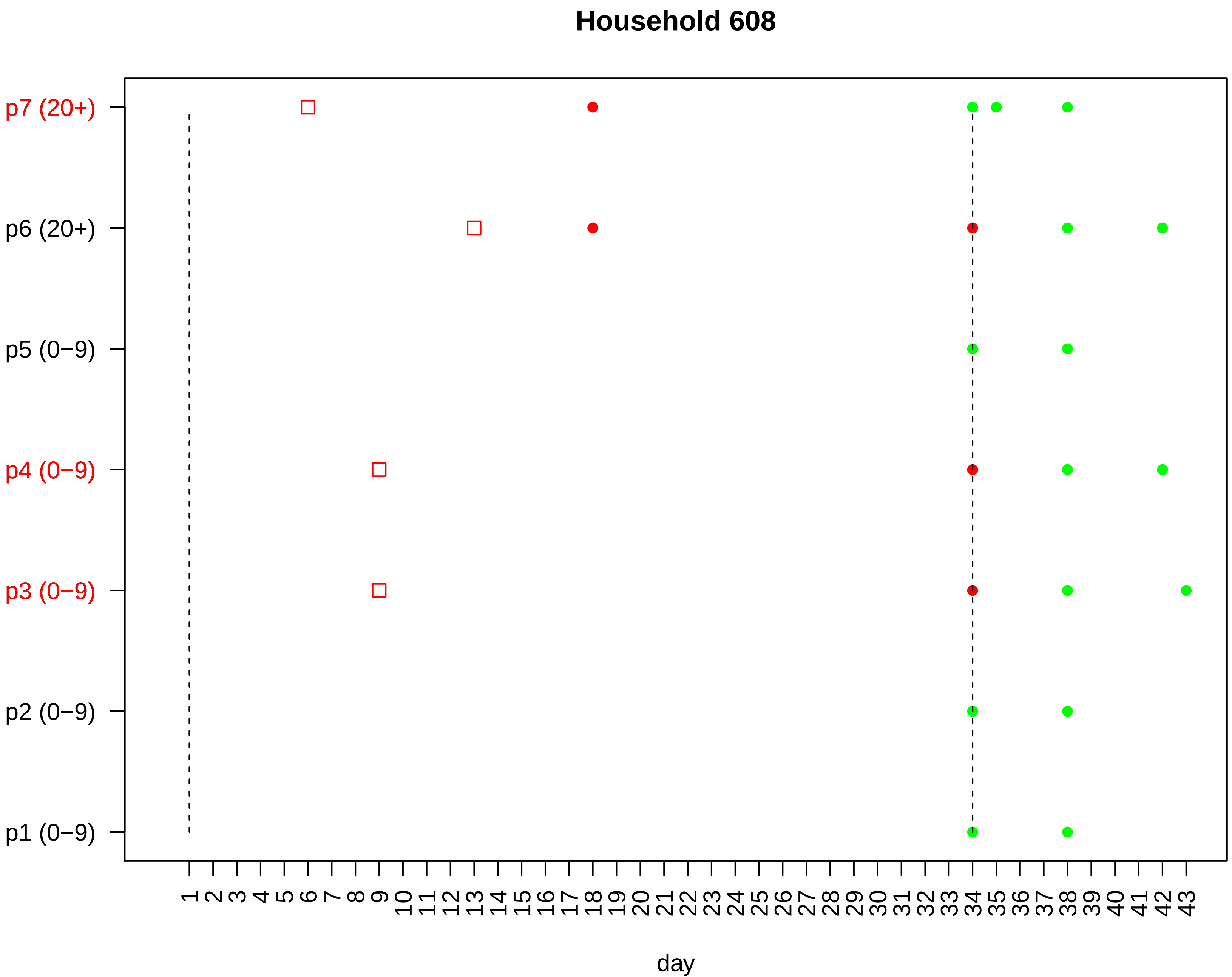

Household 608
p7 (20+)
p6 (20+)
p5 (0−9)
p4 (0−9)
p3 (0−9)
p2 (0−9)
p1 (0−9)
1 2 3 4 5 6 7 8 9 10 11 12 13 14 15 16 17 18 19 20 21 22 23 24 25 26 27 28 29 30 31 32 33 34 35 36 37 38 39 40 41 42 43
day

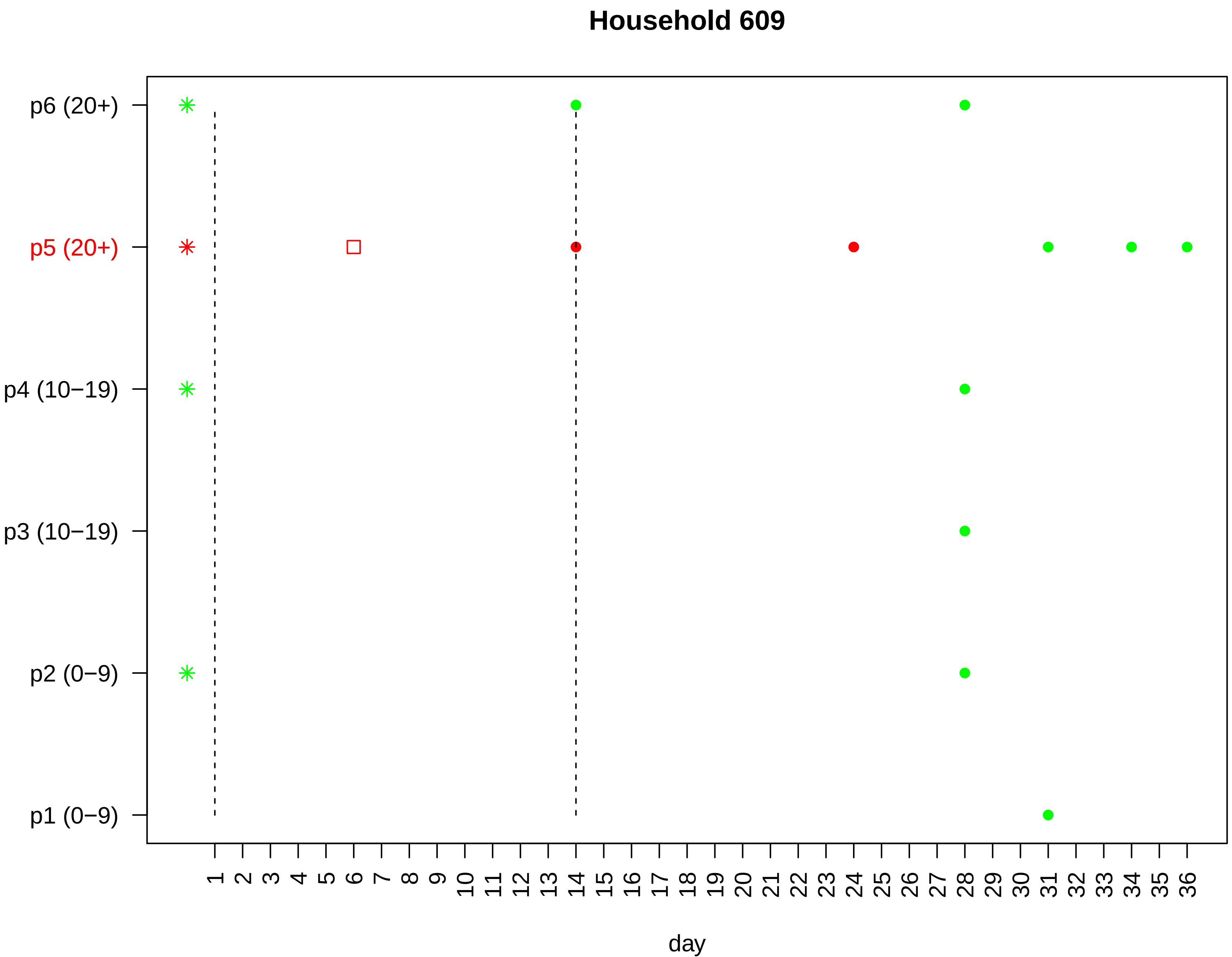

Household 609
p6 (20+)
p5 (20+)
p4 (10−19)
p3 (10−19)
p2 (0−9)
p1 (0−9)
1 2 3 4 5 6 7 8 9 10 11 12 13 14 15 16 17 18 19 20 21 22 23 24 25 26 27 28 29 30 31 32 33 34 35 36
day

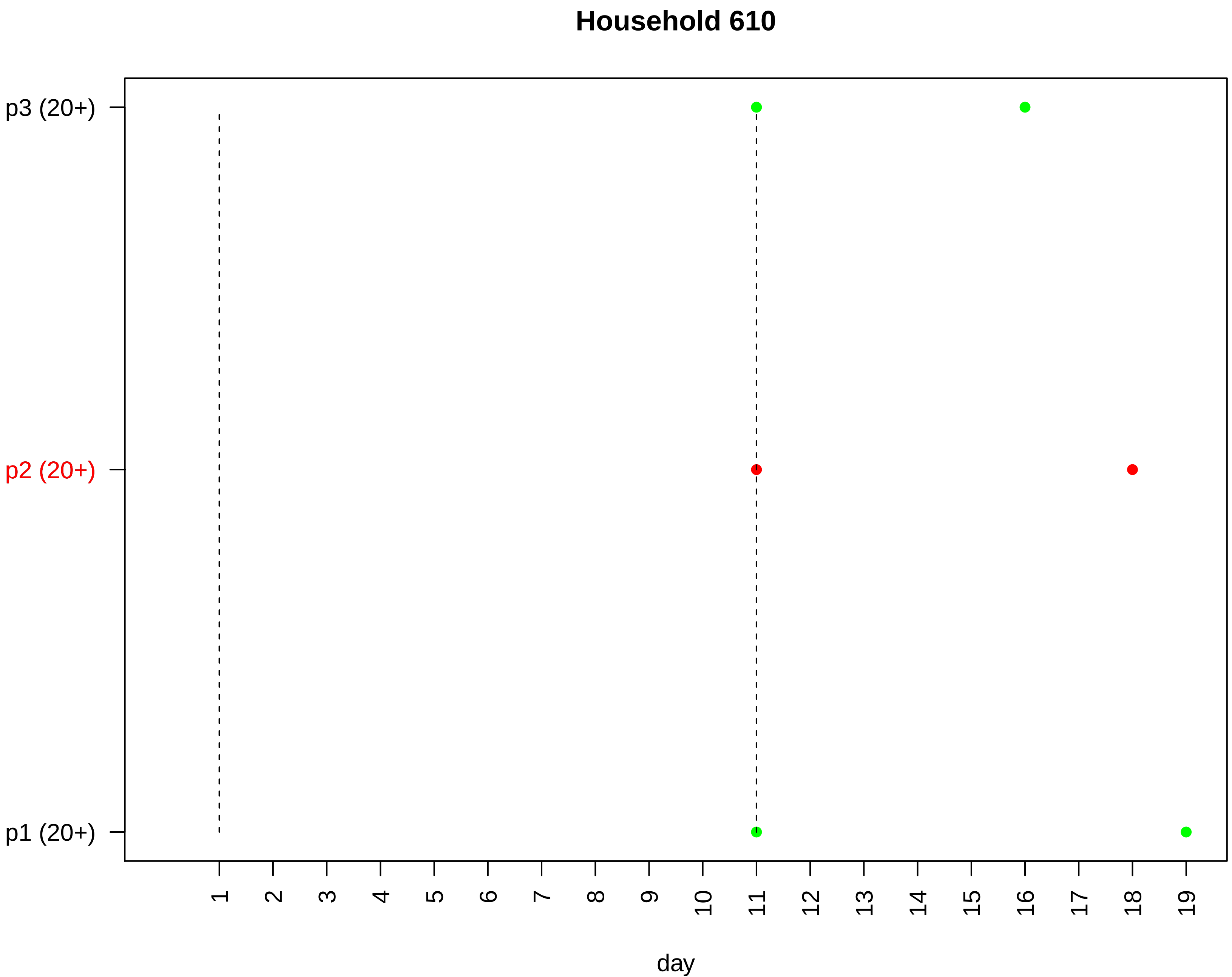

Household 610
p3 (20+)
p2 (20+)
p1 (20+)
1
2
3
4
5
6
7
8
9
10
11
12
13
14
15
16
17
18
19
day

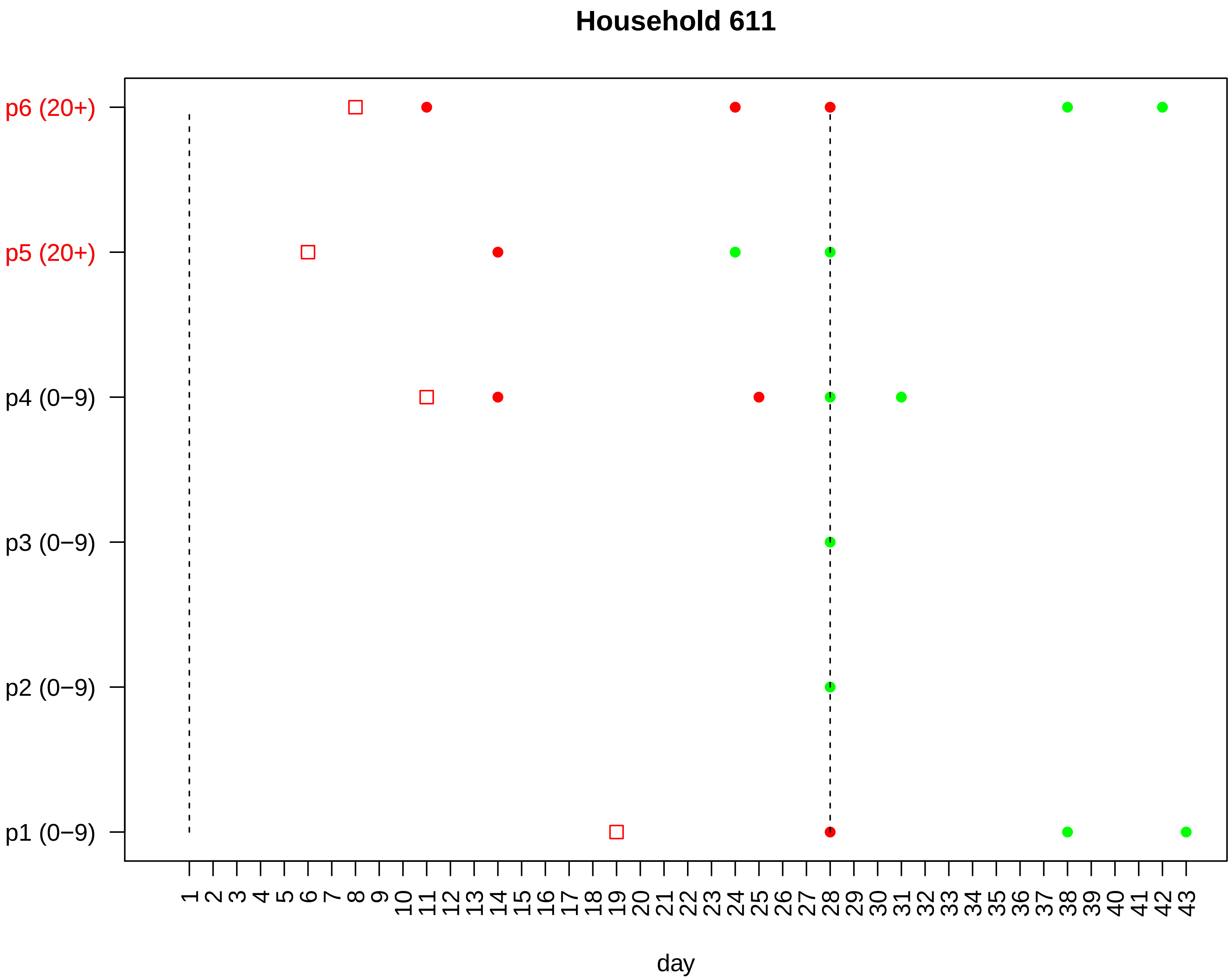

Household 611
p6 (20+)
p5 (20+)
p4 (0−9)
p3 (0−9)
p2 (0−9)
p1 (0−9)
1 2 3 4 5 6 7 8 9 10 11 12 13 14 15 16 17 18 19 20 21 22 23 24 25 26 27 28 29 30 31 32 33 34 35 36 37 38 39 40 41 42 43
day

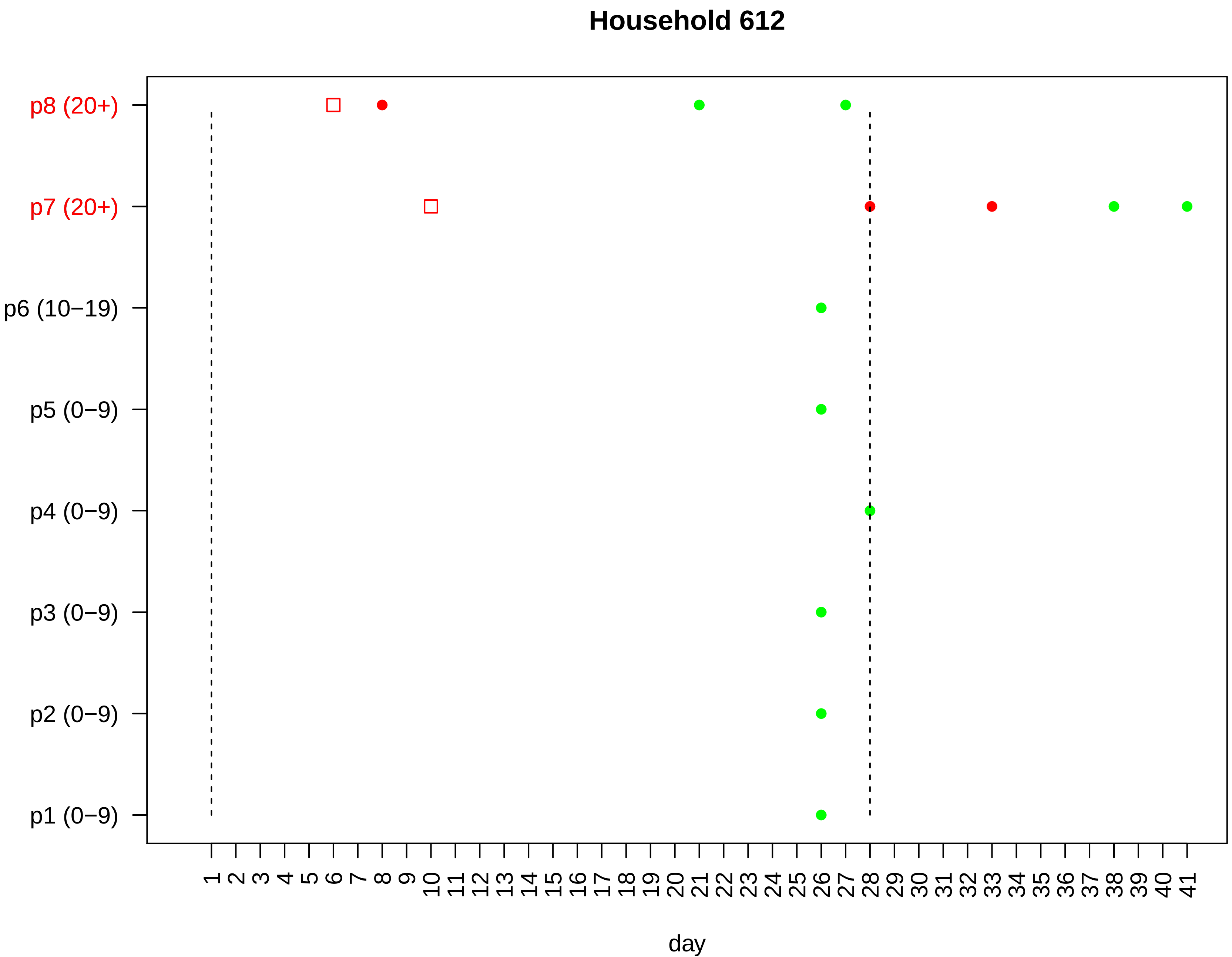

Household 612
p8 (20+)
p7 (20+)
p6 (10−19)
p5 (0−9)
p4 (0−9)
p3 (0−9)
p2 (0−9)
p1 (0−9)
1 2 3 4 5 6 7 8 9 10 11 12 13 14 15 16 17 18 19 20 21 22 23 24 25 26 27 28 29 30 31 32 33 34 35 36 37 38 39 40 41
day

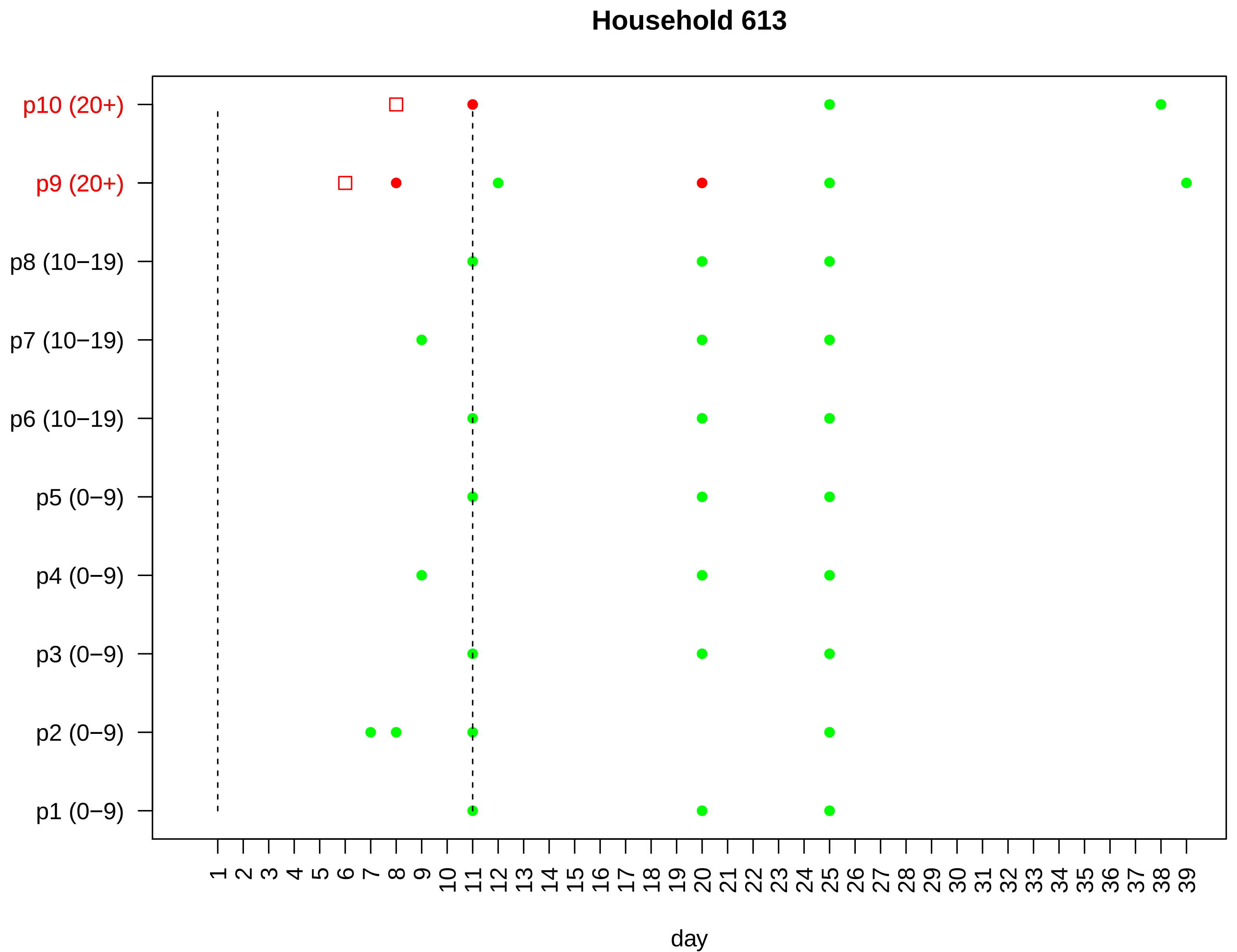
Household 613
p10 (20+)
p9 (20+)
p8 (10−19)
p7 (10−19)
p6 (10−19)
p5 (0−9)
p4 (0−9)
p3 (0−9)
p2 (0−9)
p1 (0−9)
1 2 3 4 5 6 7 8 9 10 11 12 13 14 15 16 17 18 19 20 21 22 23 24 25 26 27 28 29 30 31 32 33 34 35 36 37 38 39
day

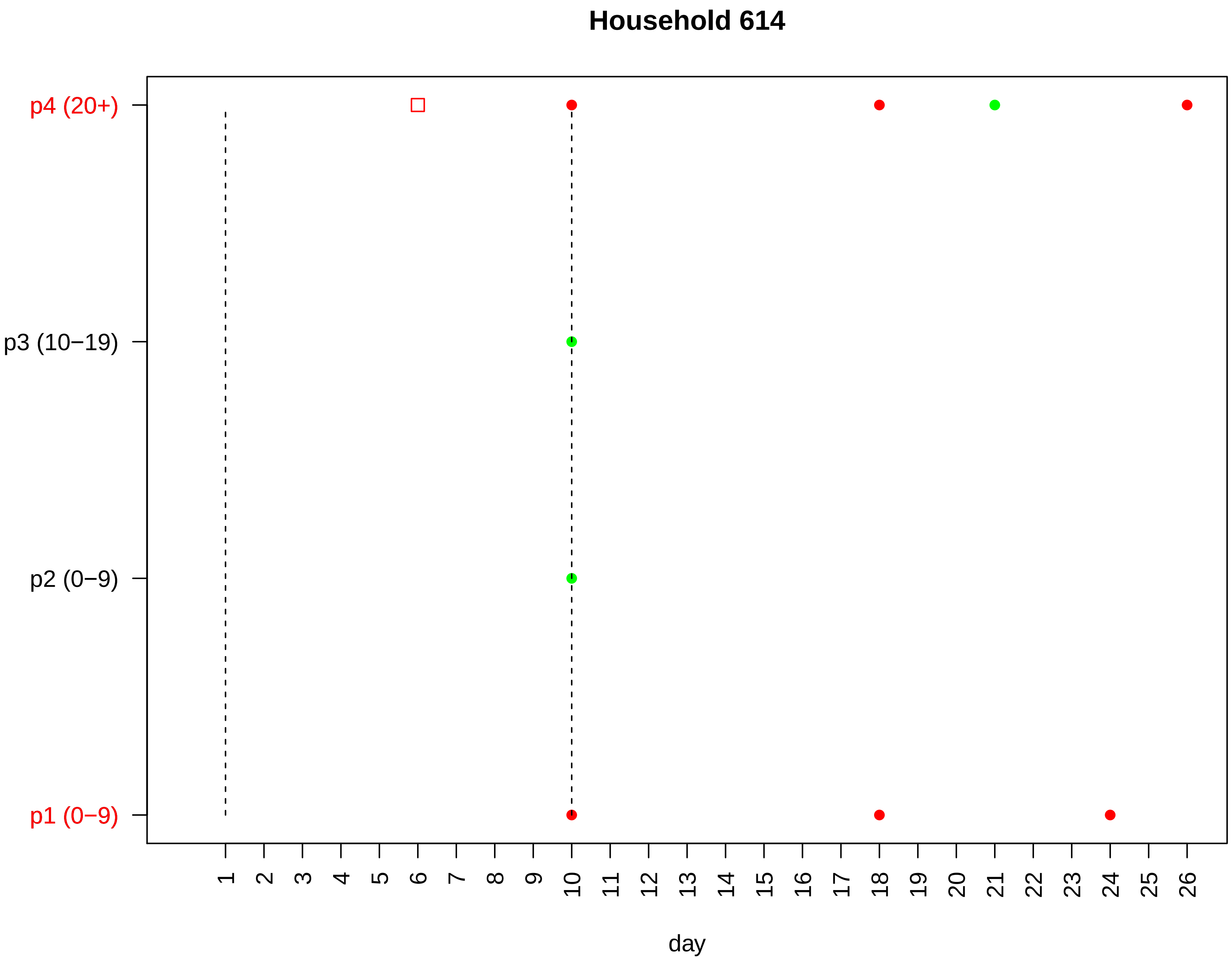

Household 614
p4 (20+)
p3 (10−19)
p2 (0−9)
p1 (0−9)
1
2
3
4
5
6
7
8
9
10
11
12
13
14
15
16
17
18
19
20
21
22
23
24
25
26
day

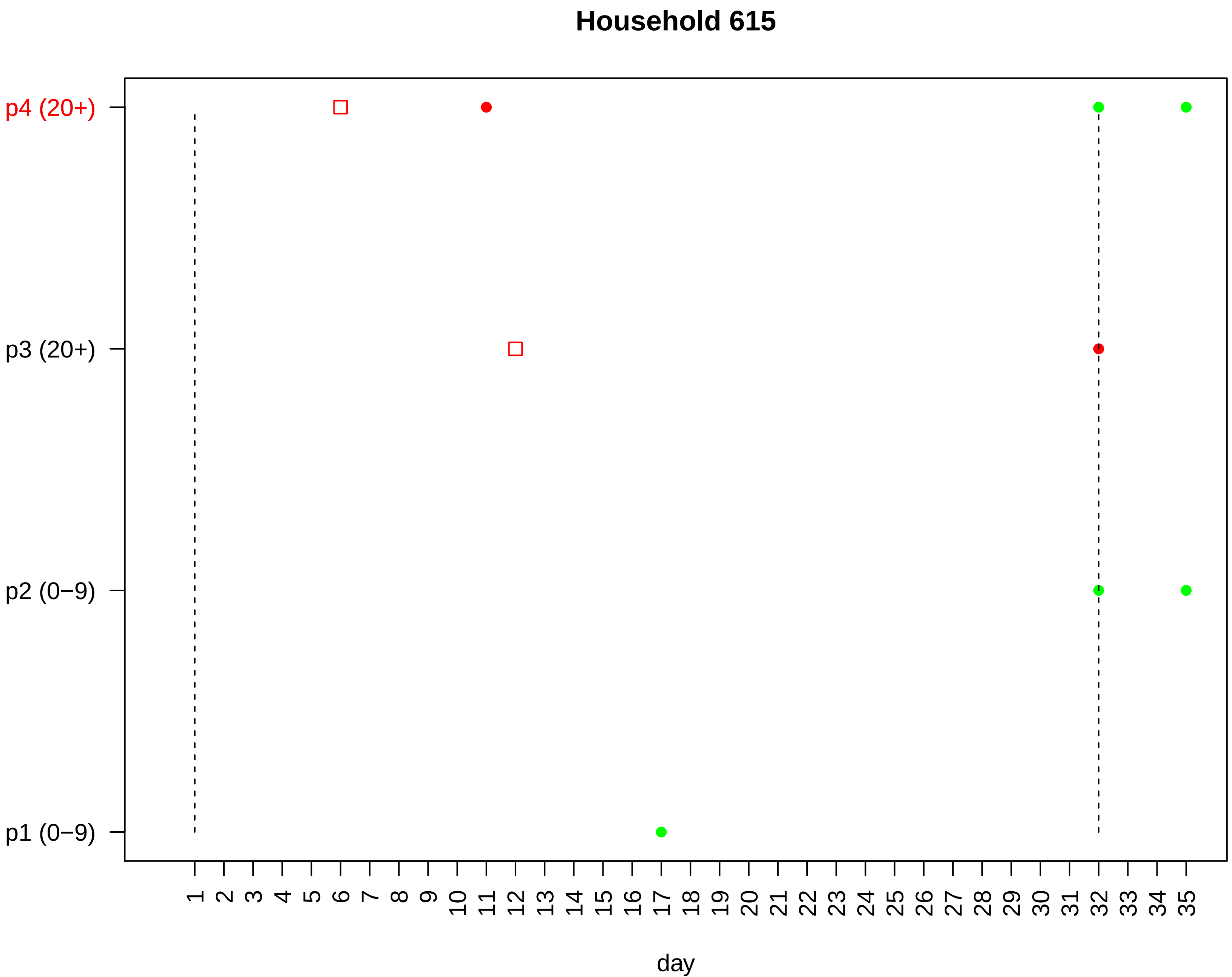
Household 615
p4 (20+)
p3 (20+)
p2 (0−9)
p1 (0−9)
1 2 3 4 5 6 7 8 9 10 11 12 13 14 15 16 17 18 19 20 21 22 23 24 25 26 27 28 29 30 31 32 33 34 35
day

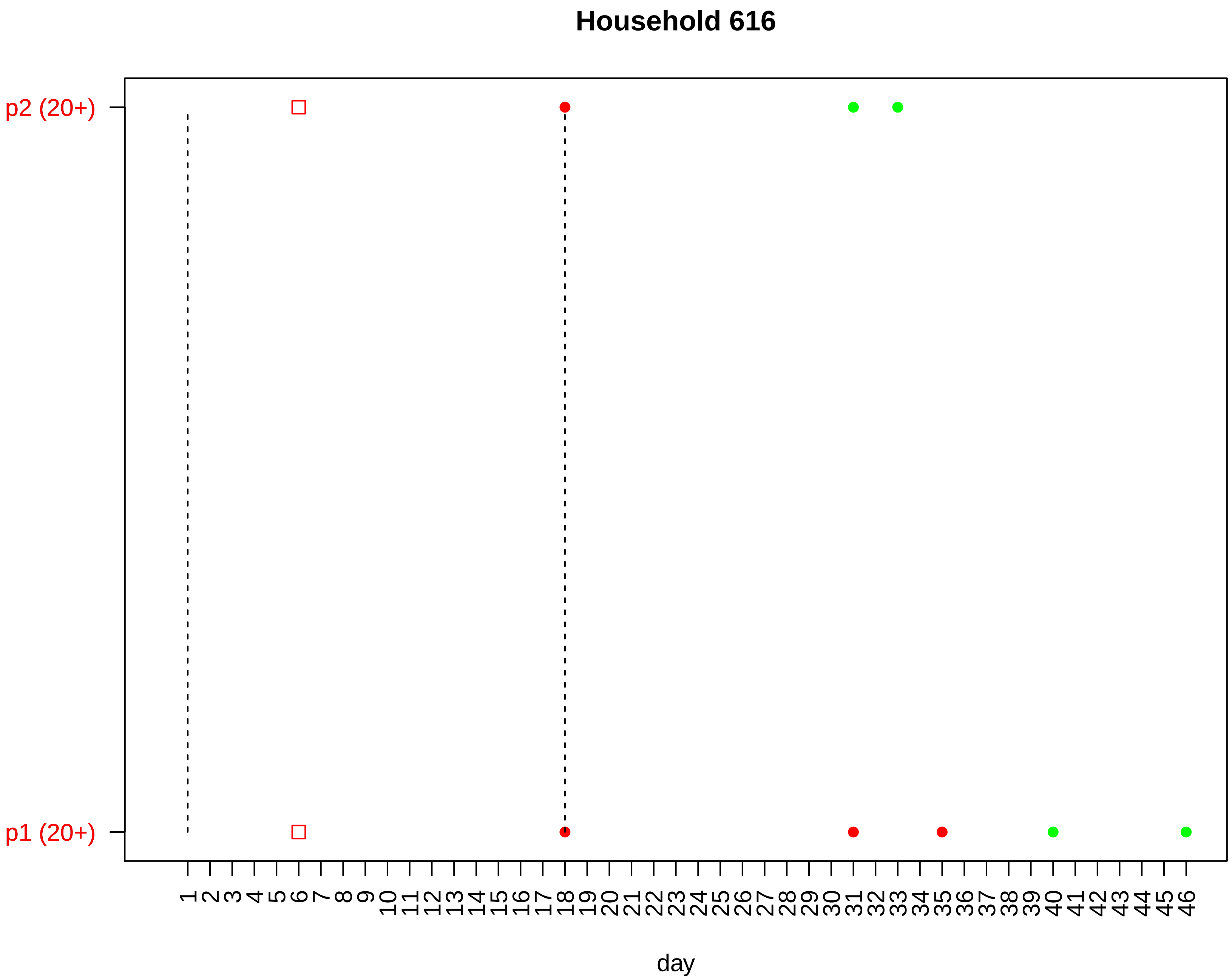
Household 616
p2 (20+)
p1 (20+)
1 2 3 4 5 6 7 8 9 10 11 12 13 14 15 16 17 18 19 20 21 22 23 24 25 26 27 28 29 30 31 32 33 34 35 36 37 38 39 40 41 42 43 44 45 46
day

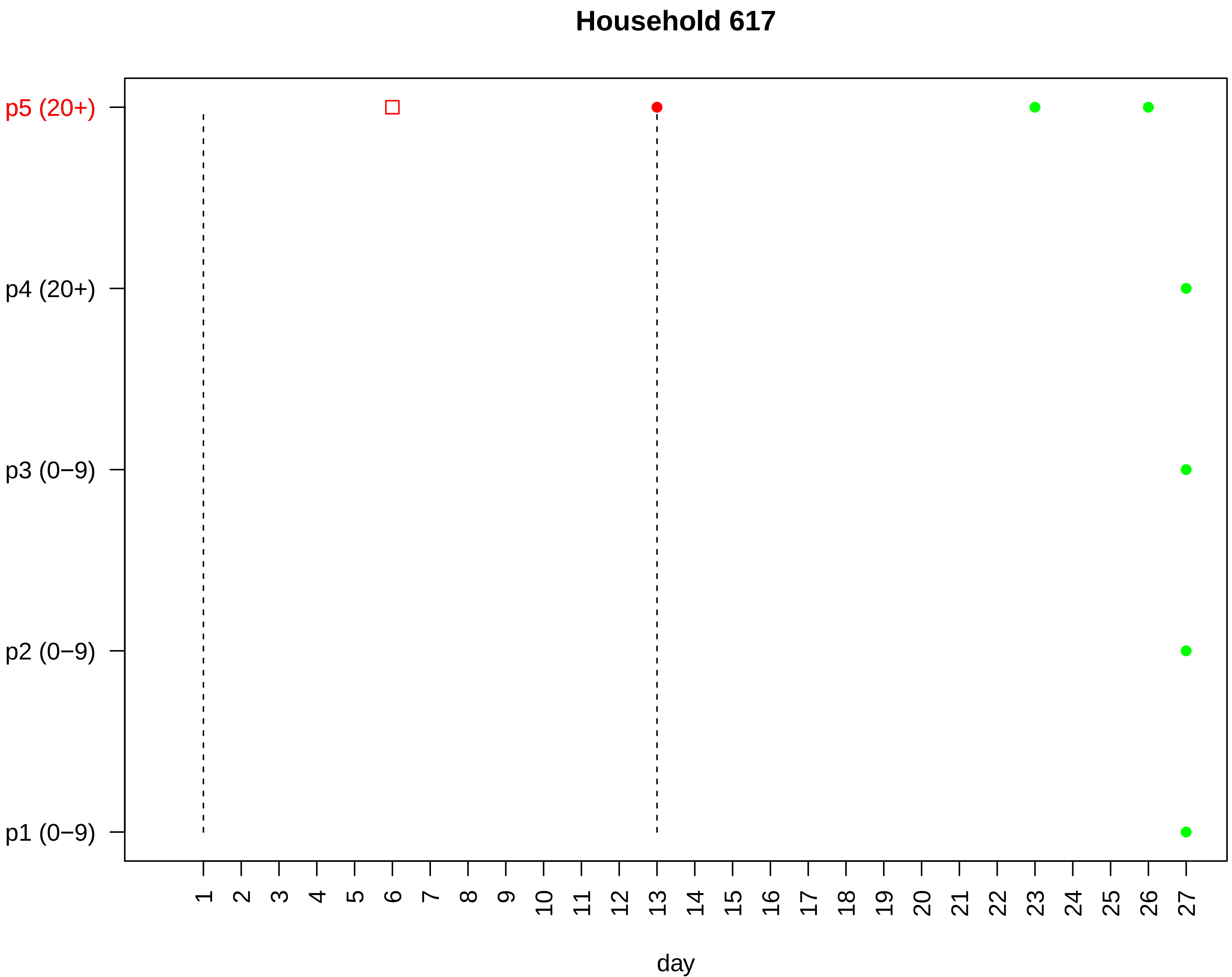
Household 617
p5 (20+)
p4 (20+)
p3 (0−9)
p2 (0−9)
p1 (0−9)
1
2
3
4
5
6
7
8
9
10
11
12
13
14
15
16
17
18
19
20
21
22
23
24
25
26
27
day

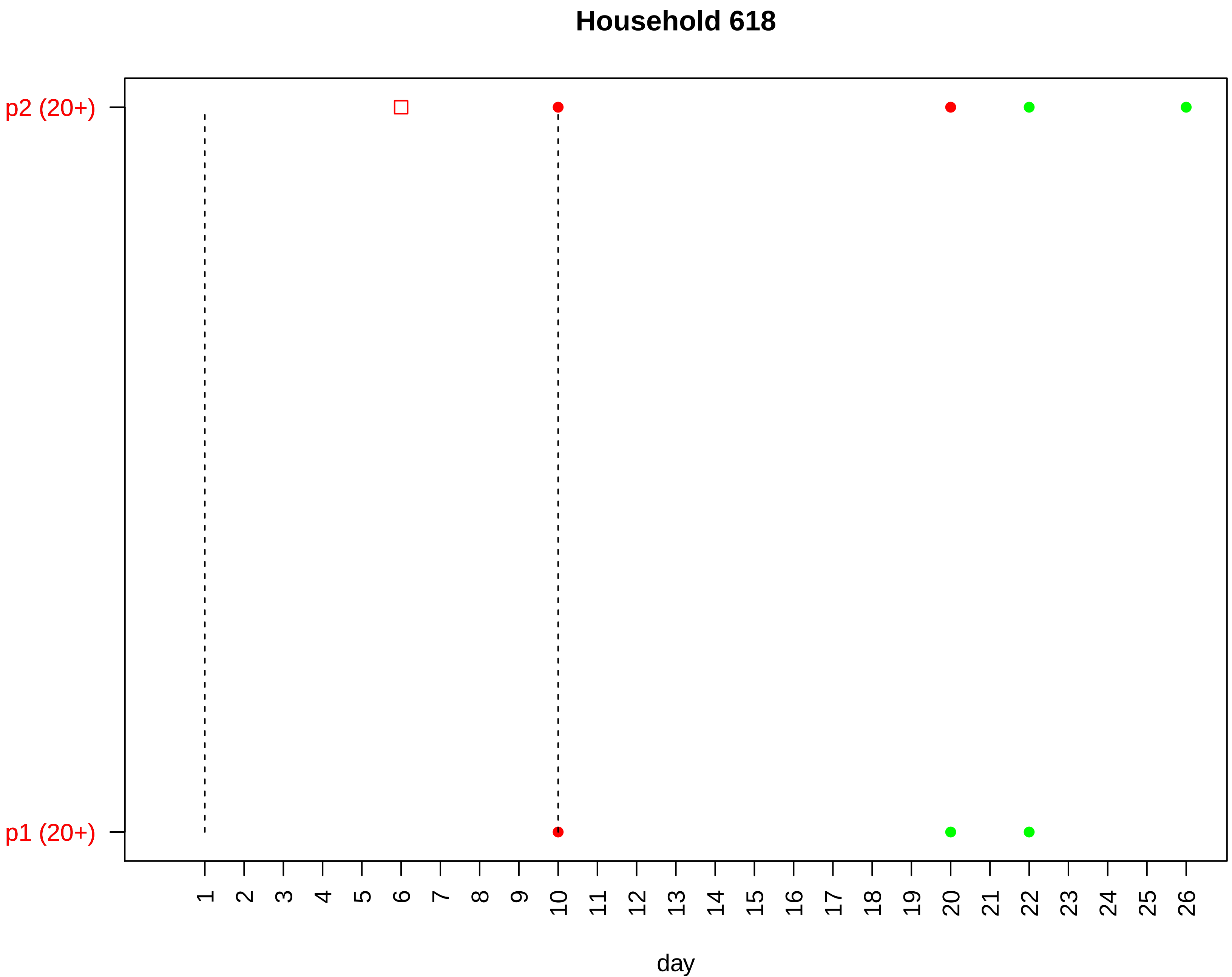

Household 618
p2 (20+)
p1 (20+)
1
2
3
4
5
6
7
8
9
10
11
12
13
14
15
16
17
18
19
20
21
22
23
24
25
26
day

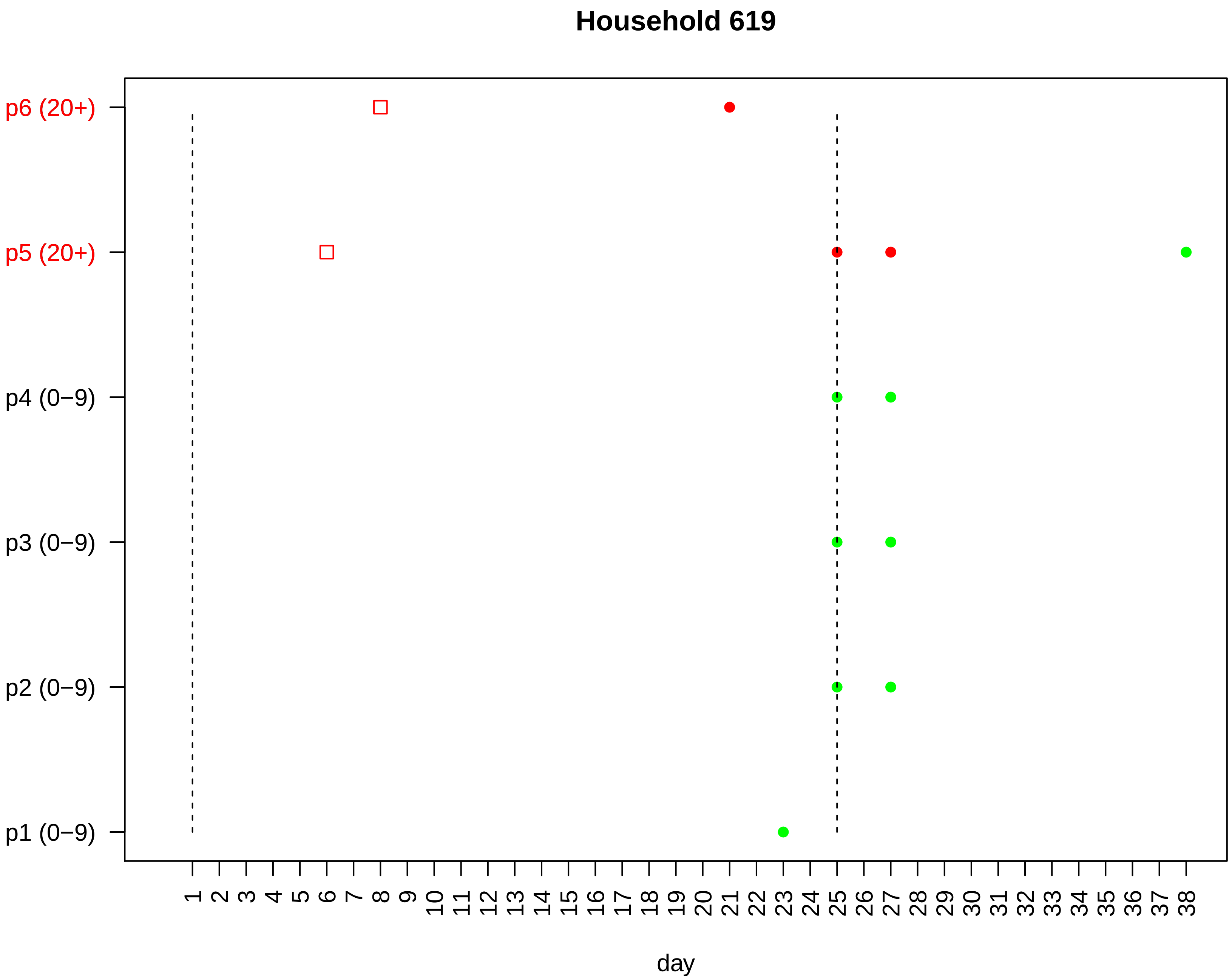

Household 619
p6 (20+)
p5 (20+)
p4 (0−9)
p3 (0−9)
p2 (0−9)
p1 (0−9)
1 2 3 4 5 6 7 8 9 10 11 12 13 14 15 16 17 18 19 20 21 22 23 24 25 26 27 28 29 30 31 32 33 34 35 36 37 38
day

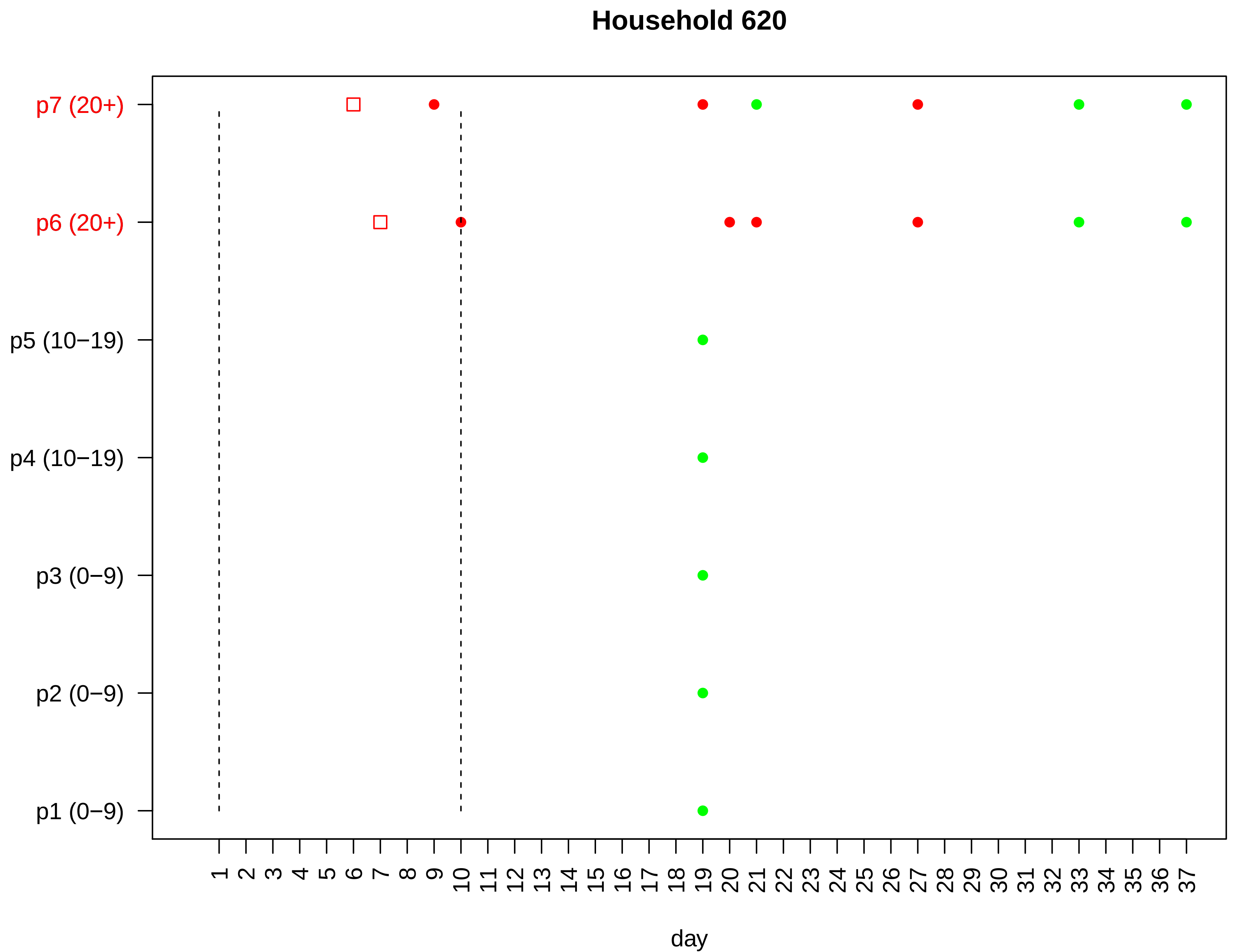

Household 620
p7 (20+)
p6 (20+)
p5 (10−19)
p4 (10−19)
p3 (0−9)
p2 (0−9)
p1 (0−9)
1 2 3 4 5 6 7 8 9 10 11 12 13 14 15 16 17 18 19 20 21 22 23 24 25 26 27 28 29 30 31 32 33 34 35 36 37
day

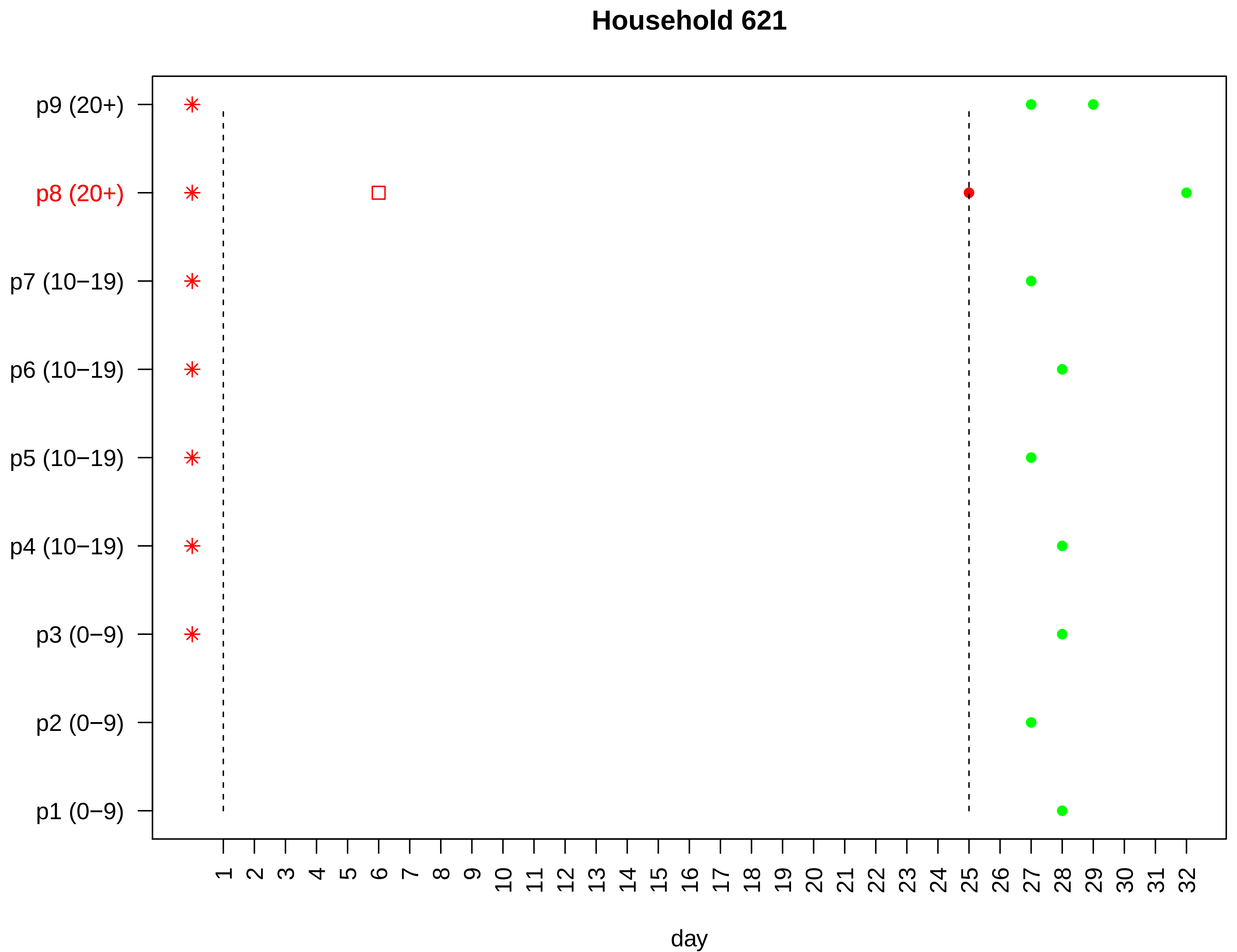

Household 621
p9 (20+)
p8 (20+)
p7 (10−19)
p6 (10−19)
p5 (10−19)
p4 (10−19)
p3 (0−9)
p2 (0−9)
p1 (0−9)
1 2 3 4 5 6 7 8 9 10 11 12 13 14 15 16 17 18 19 20 21 22 23 24 25 26 27 28 29 30 31 32
day

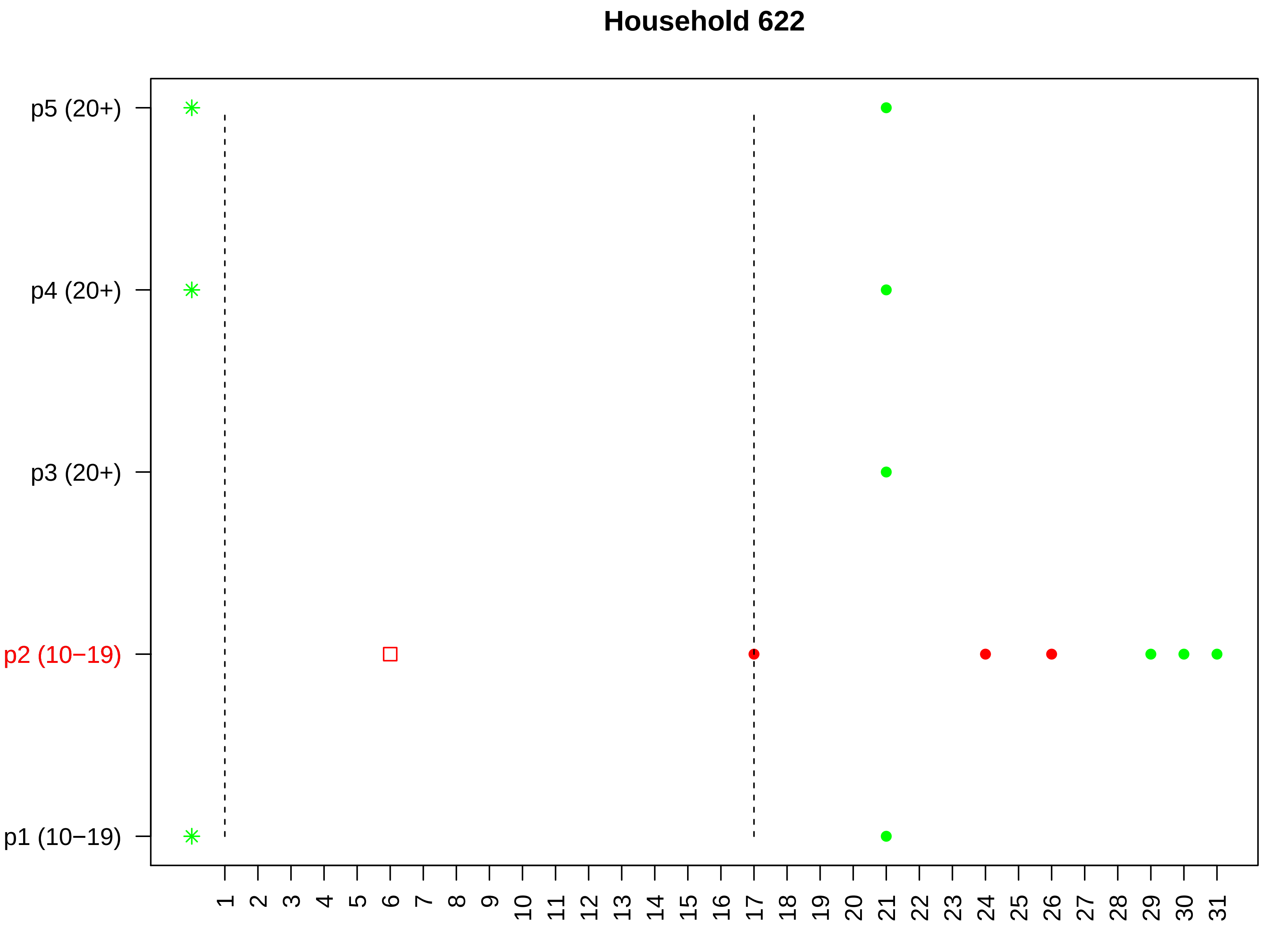
Household 622
p5 (20+)
p4 (20+)
p3 (20+)
p2 (10−19)
p1 (10−19)
1 2 3 4 5 6 7 8 9 10 11 12 13 14 15 16 17 18 19 20 21 22 23 24 25 26 27 28 29 30 31
day

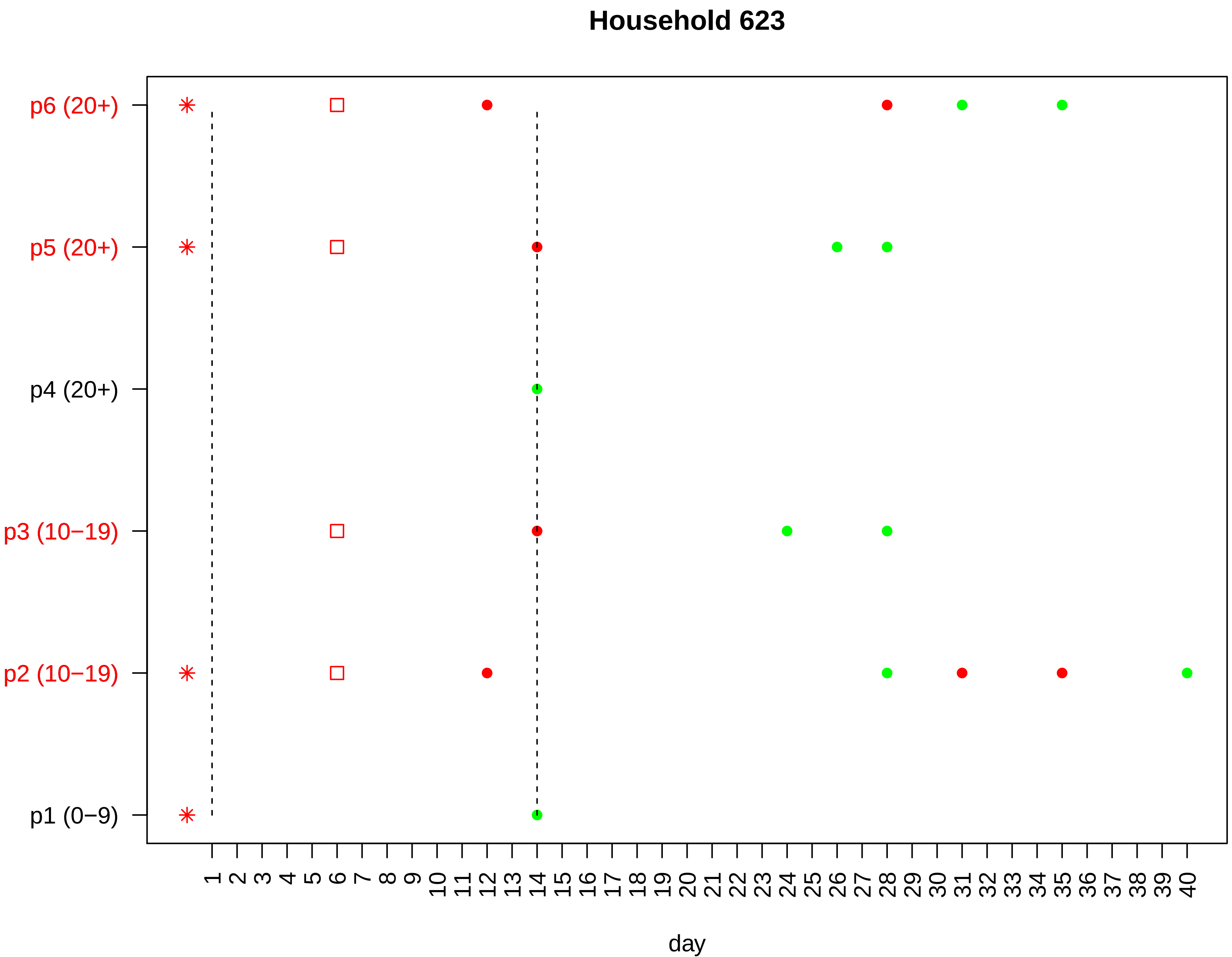

Household 623
p6 (20+)
p5 (20+)
p4 (20+)
p3 (10−19)
p2 (10−19)
p1 (0−9)
1 2 3 4 5 6 7 8 9 10 11 12 13 14 15 16 17 18 19 20 21 22 23 24 25 26 27 28 29 30 31 32 33 34 35 36 37 38 39 40
day

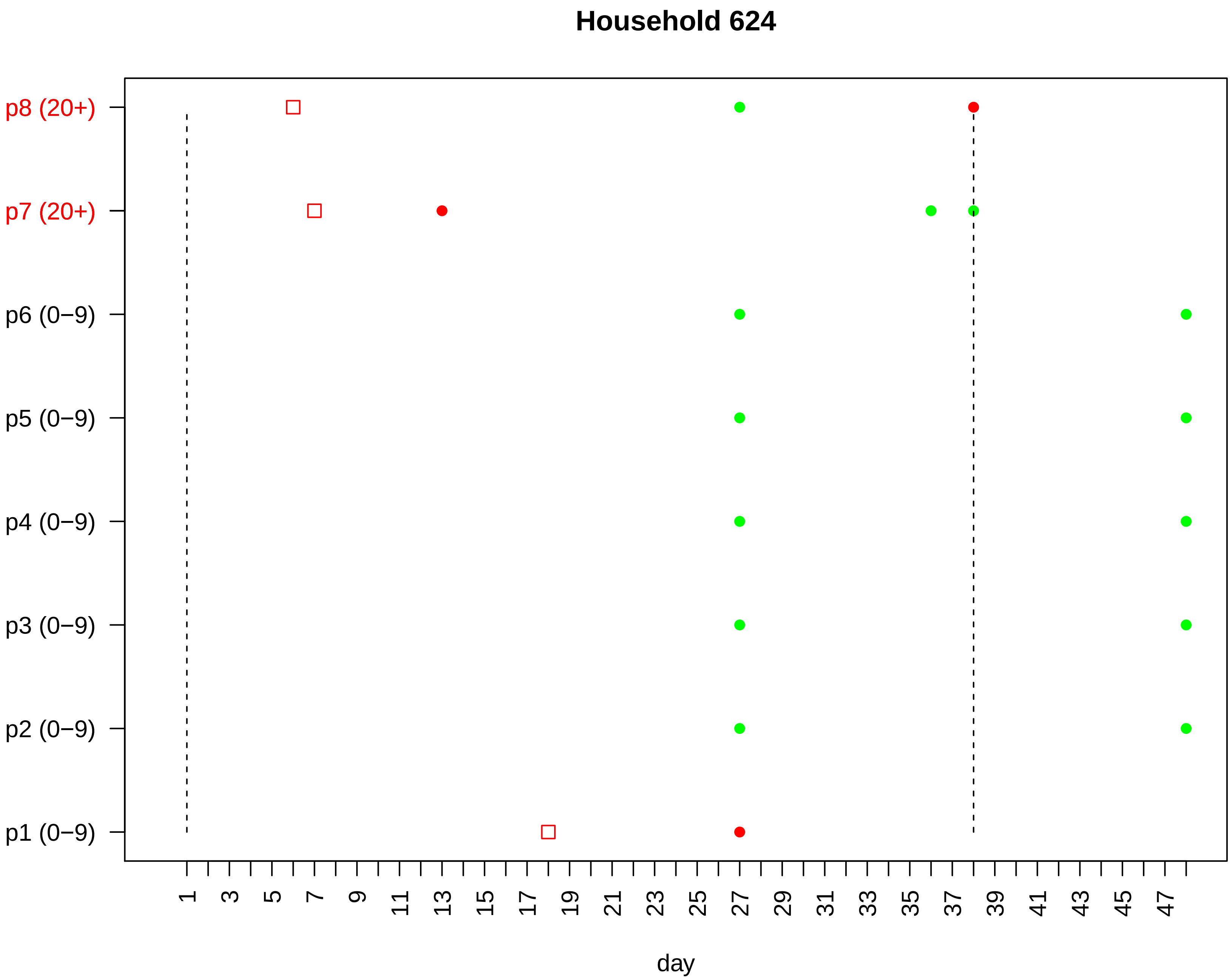

Household 624
p8 (20+)
p7 (20+)
p6 (0−9)
p5 (0−9)
p4 (0−9)
p3 (0−9)
p2 (0−9)
p1 (0−9)
1
3
5
7
9
11
13
15
17
19
21
23
25
27
29
31
33
35
37
39
41
43
45
47
day

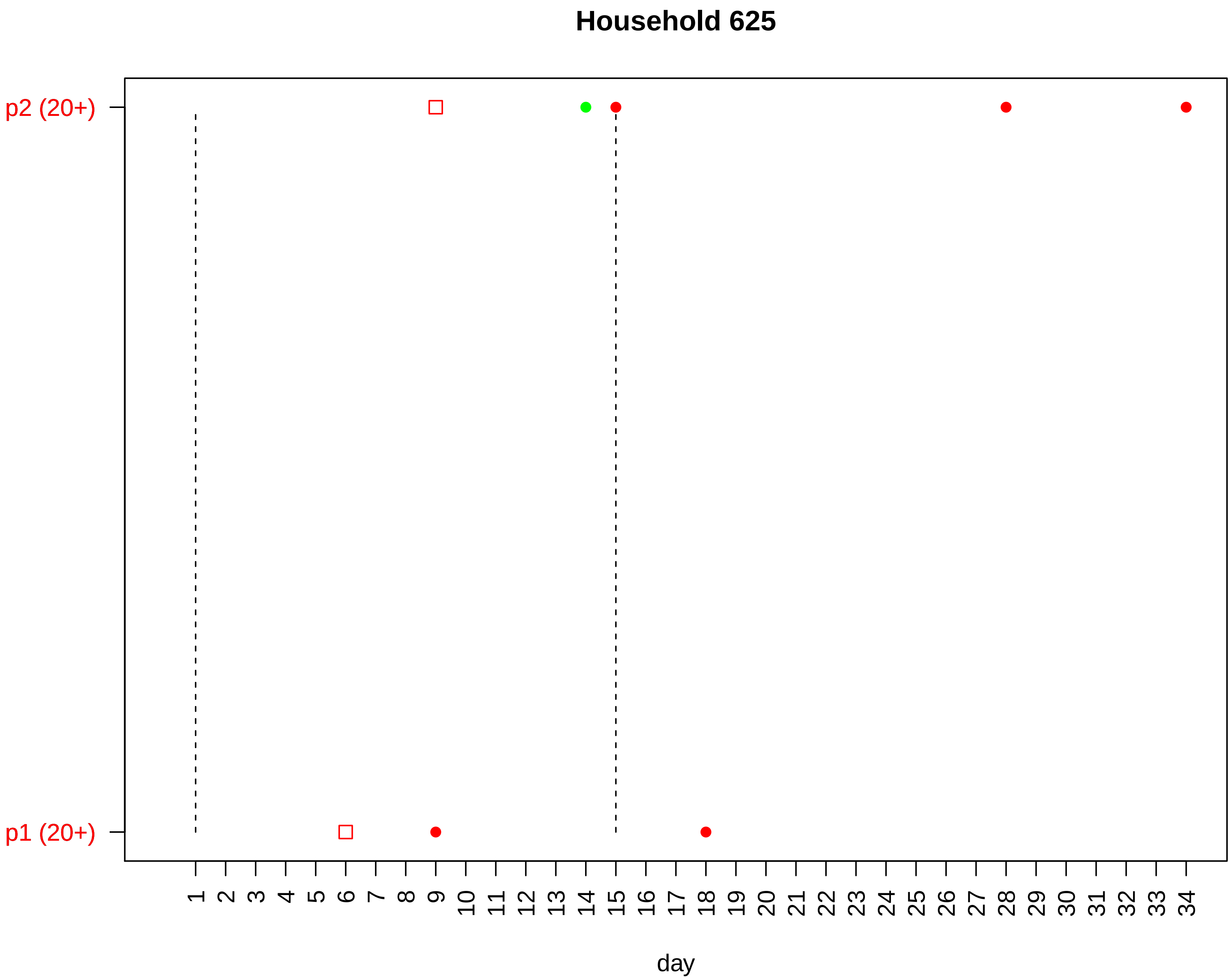
Household 625
p2 (20+)
p1 (20+)
1
2
3
4
5
6
7
8
9
10
11
12
13
14
15
16
17
18
19
20
21
22
23
24
25
26
27
28
29
30
31
32
33
34
day

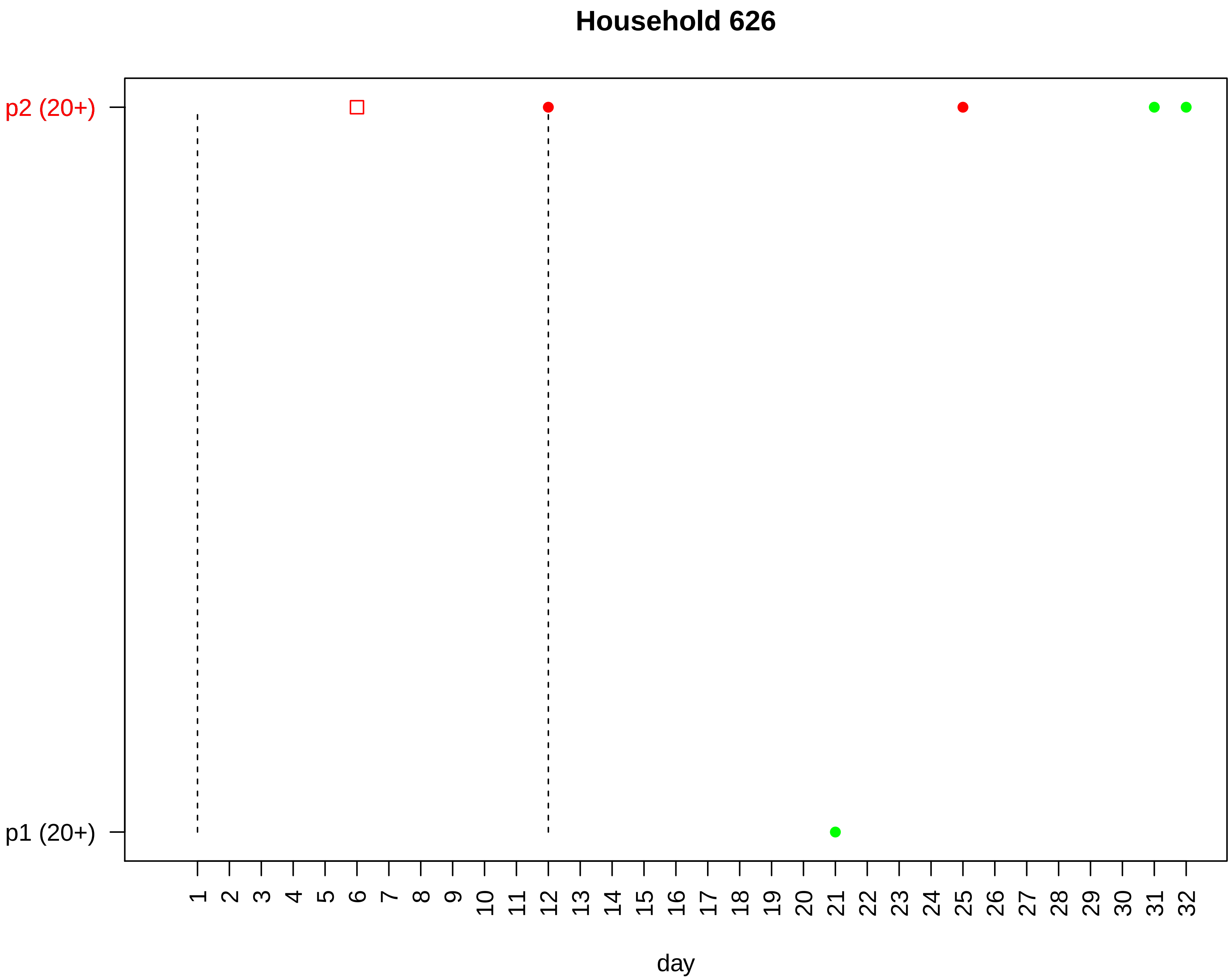

Household 626
p2 (20+)
p1 (20+)
1 2 3 4 5 6 7 8 9 10 11 12 13 14 15 16 17 18 19 20 21 22 23 24 25 26 27 28 29 30 31 32
day

Household 627

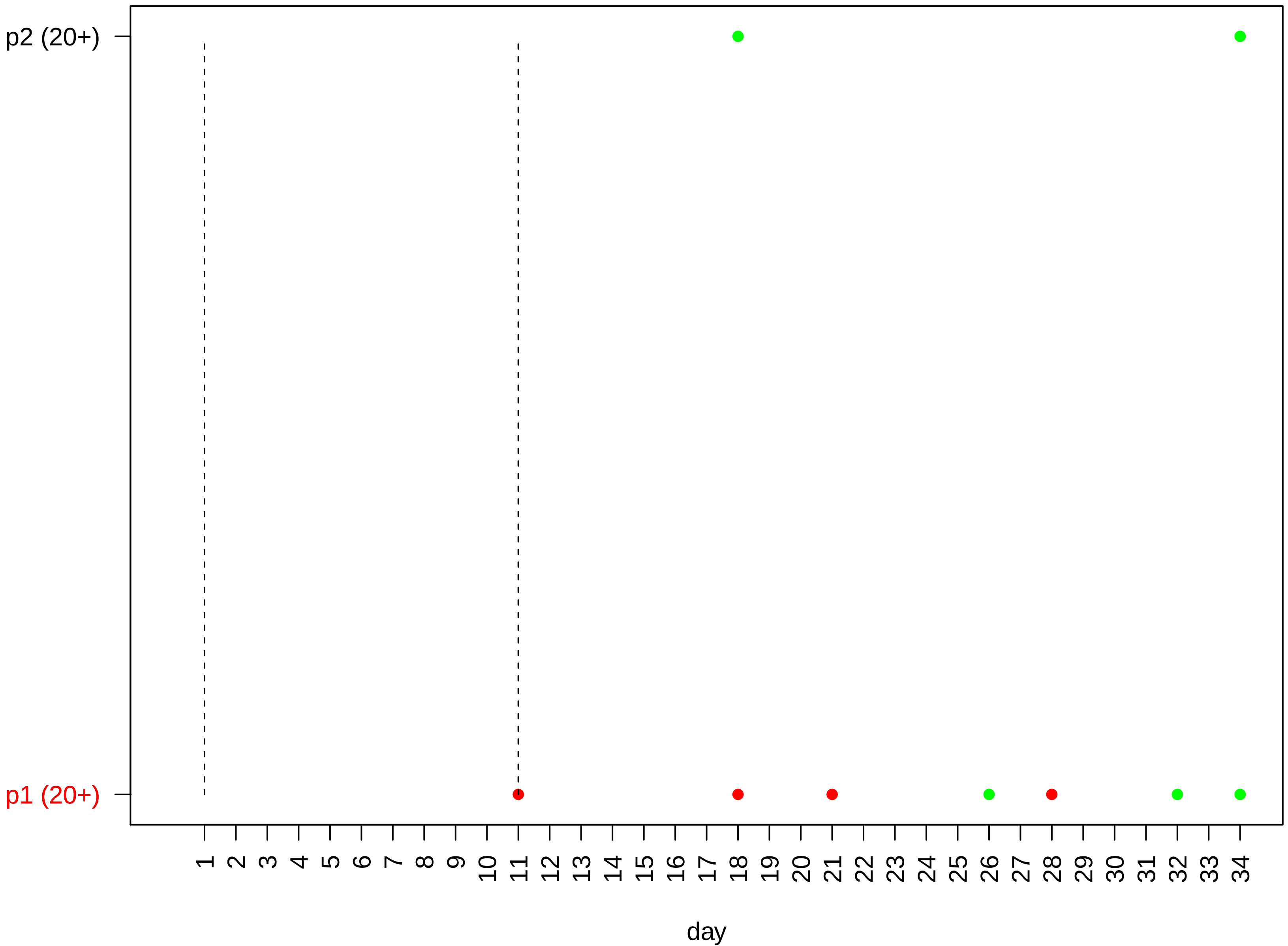

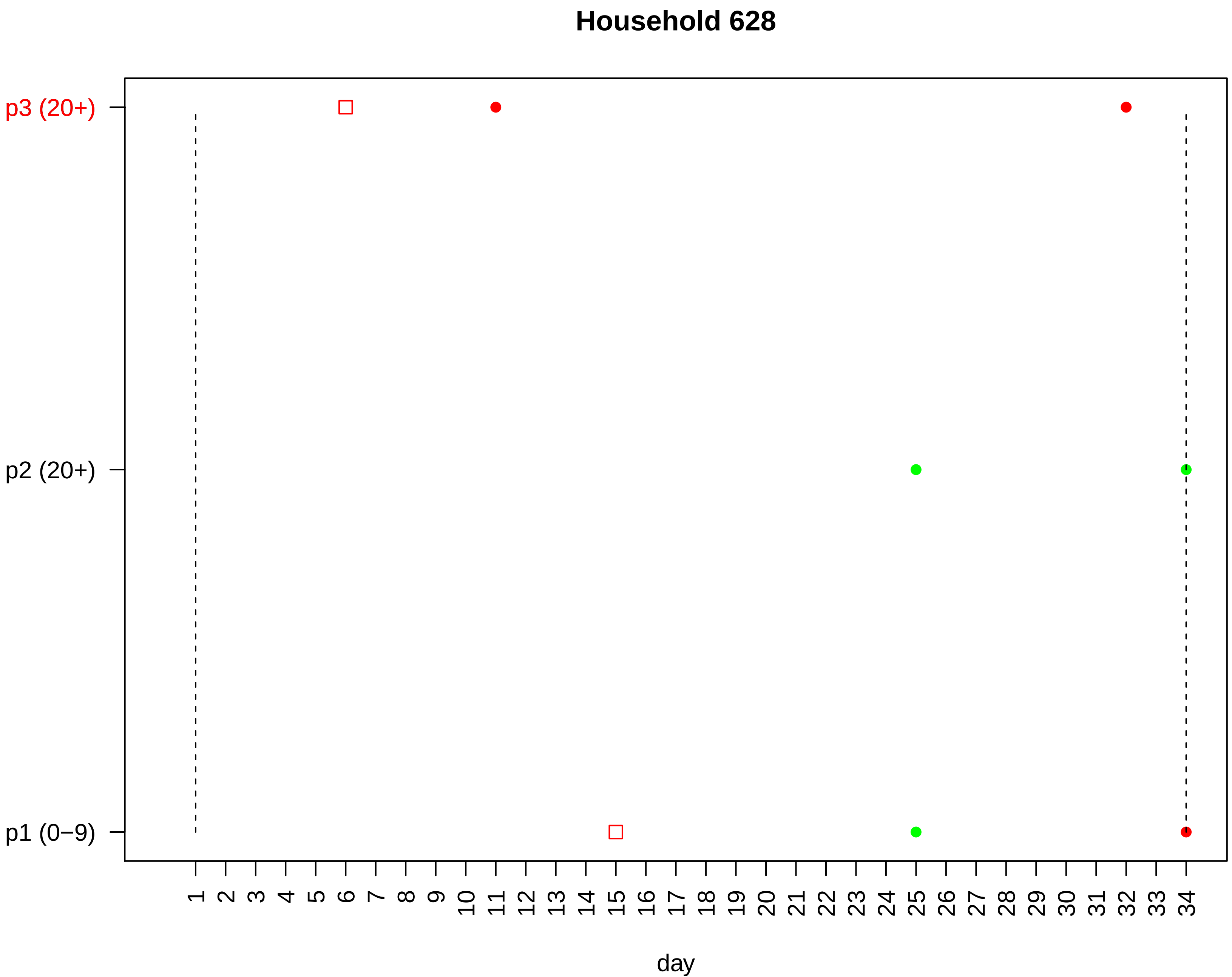
Household 628
p3 (20+)
p2 (20+)
p1 (0−9)
1 2 3 4 5 6 7 8 9 10 11 12 13 14 15 16 17 18 19 20 21 22 23 24 25 26 27 28 29 30 31 32 33 34
day

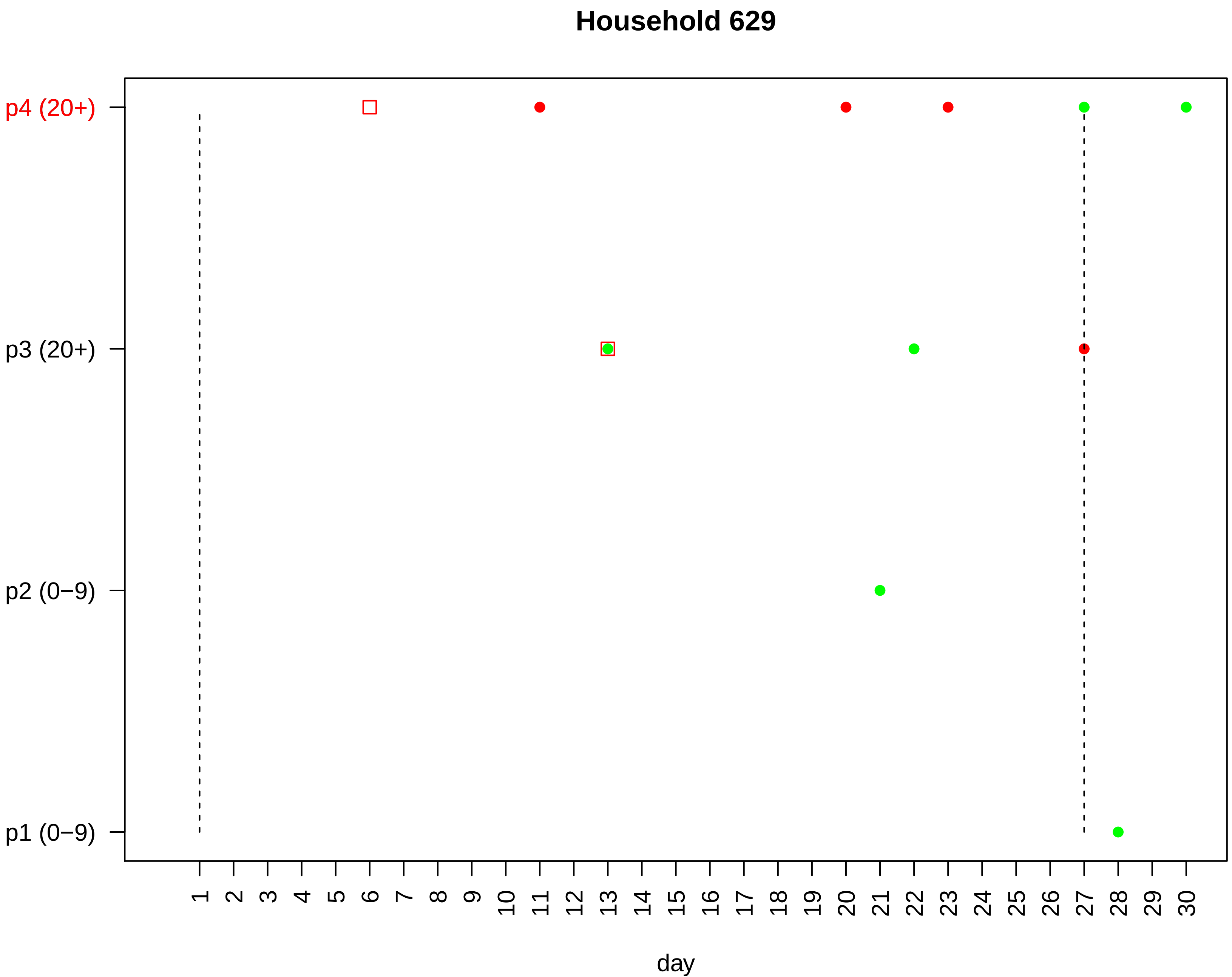

Household 629
p4 (20+)
p3 (20+)
p2 (0−9)
p1 (0−9)
1 2 3 4 5 6 7 8 9 10 11 12 13 14 15 16 17 18 19 20 21 22 23 24 25 26 27 28 29 30
day

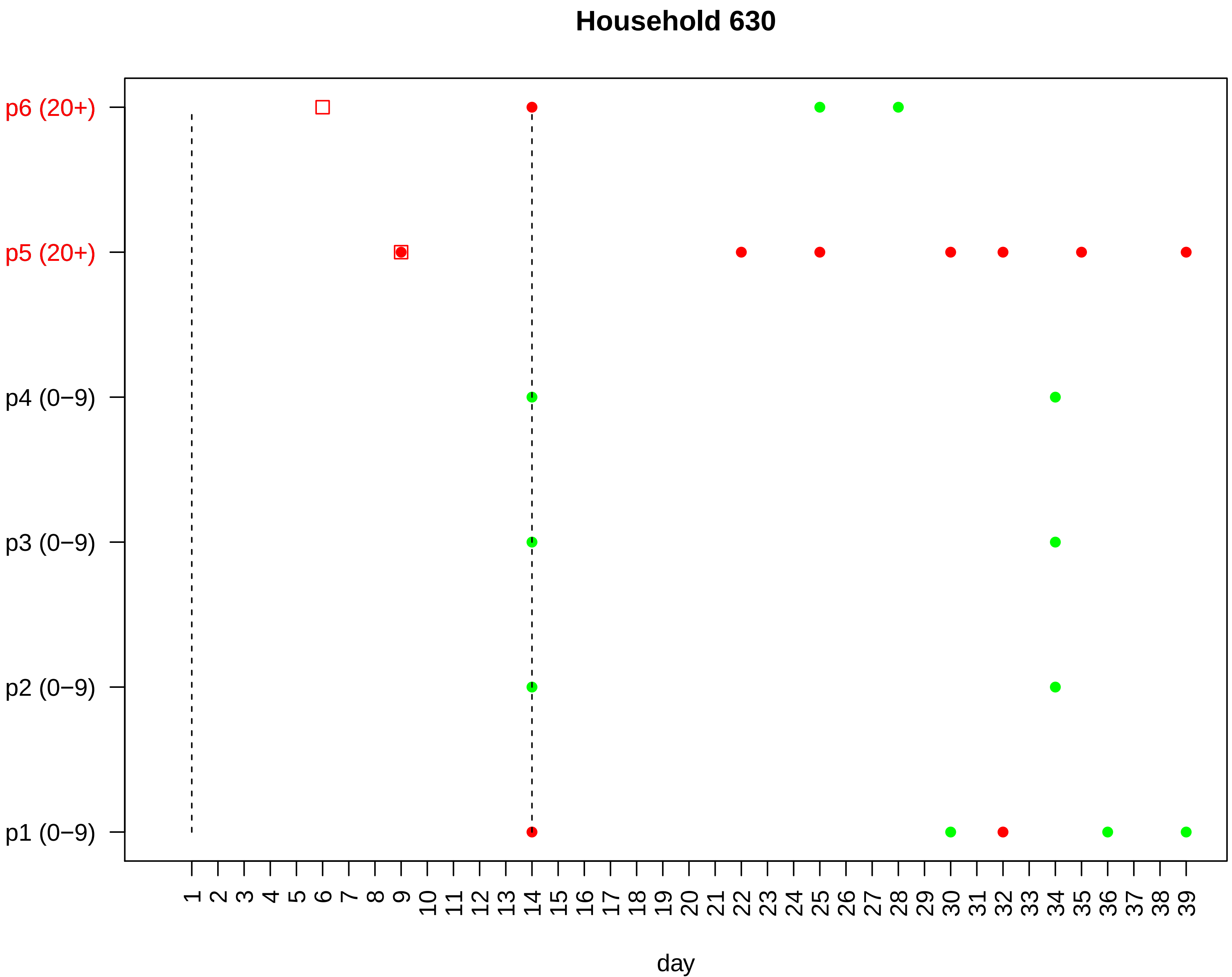

Household 630
p6 (20+)
p5 (20+)
p4 (0−9)
p3 (0−9)
p2 (0−9)
p1 (0−9)
1 2 3 4 5 6 7 8 9 10 11 12 13 14 15 16 17 18 19 20 21 22 23 24 25 26 27 28 29 30 31 32 33 34 35 36 37 38 39
day

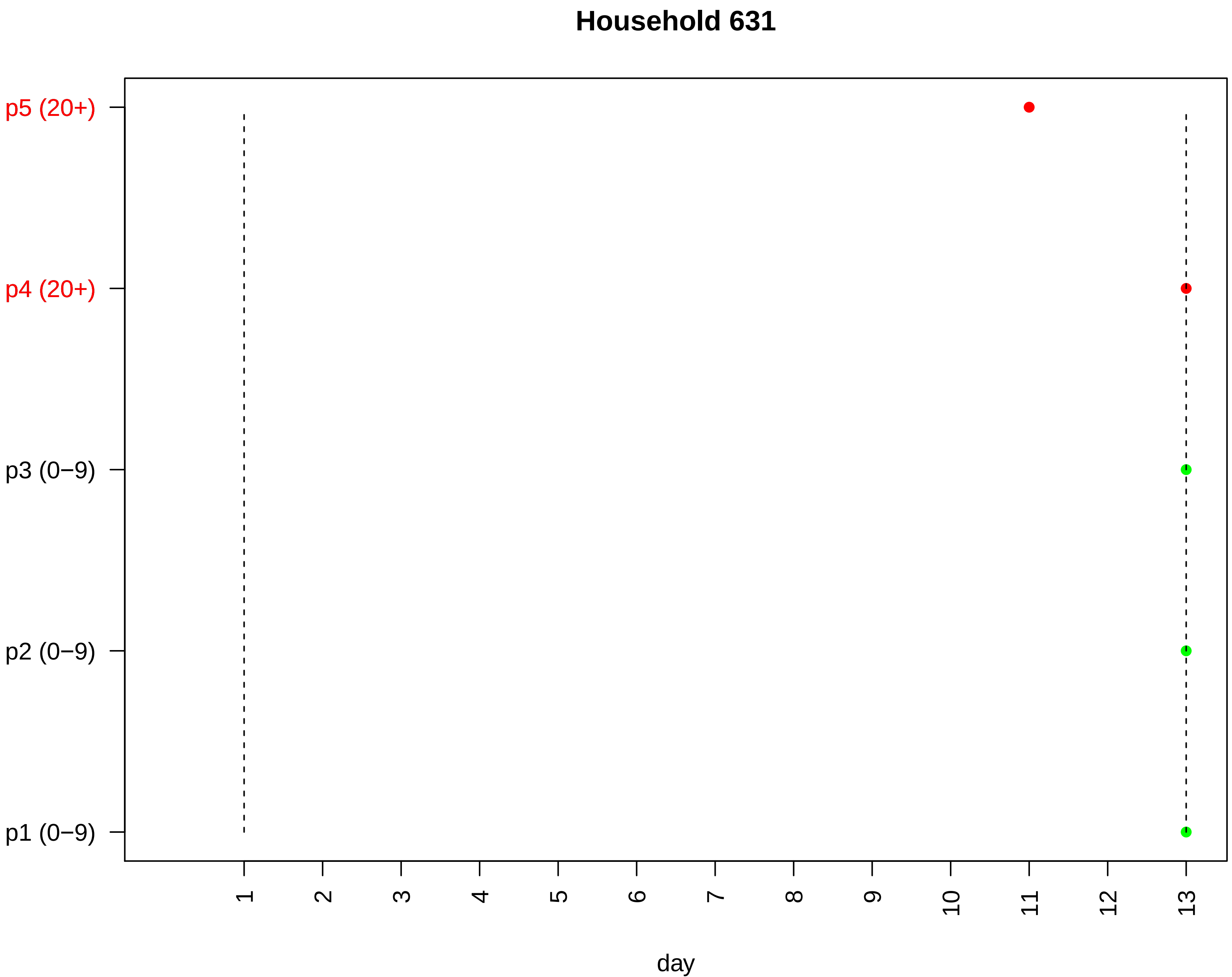
Household 631
p5 (20+)
p4 (20+)
p3 (0−9)
p2 (0−9)
p1 (0−9)
1
2
3
4
5
6
7
8
9
10
11
12
13
day

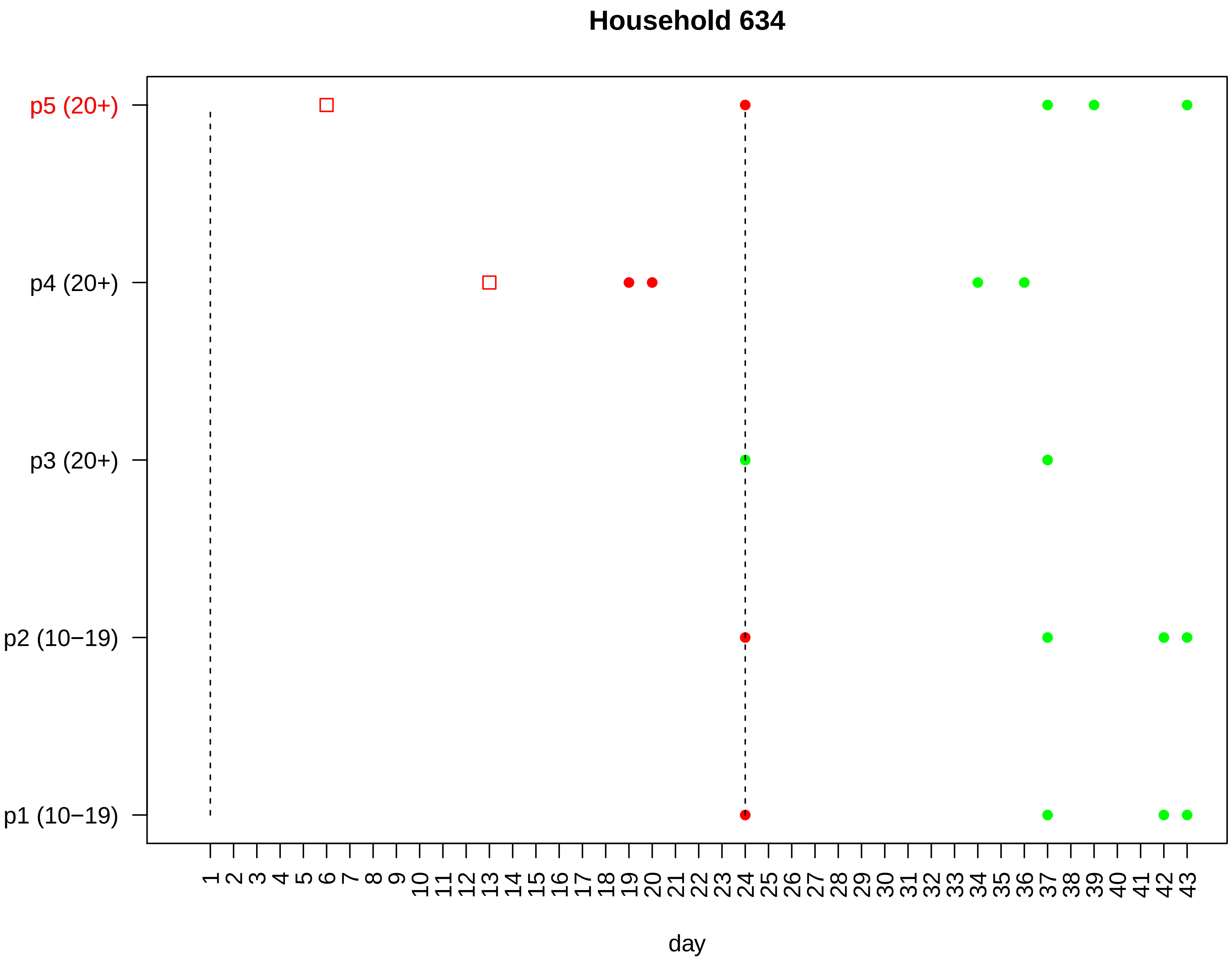

Household 634
p5 (20+)
p4 (20+)
p3 (20+)
p2 (10−19)
p1 (10−19)
1 2 3 4 5 6 7 8 9 10 11 12 13 14 15 16 17 18 19 20 21 22 23 24 25 26 27 28 29 30 31 32 33 34 35 36 37 38 39 40 41 42 43
day

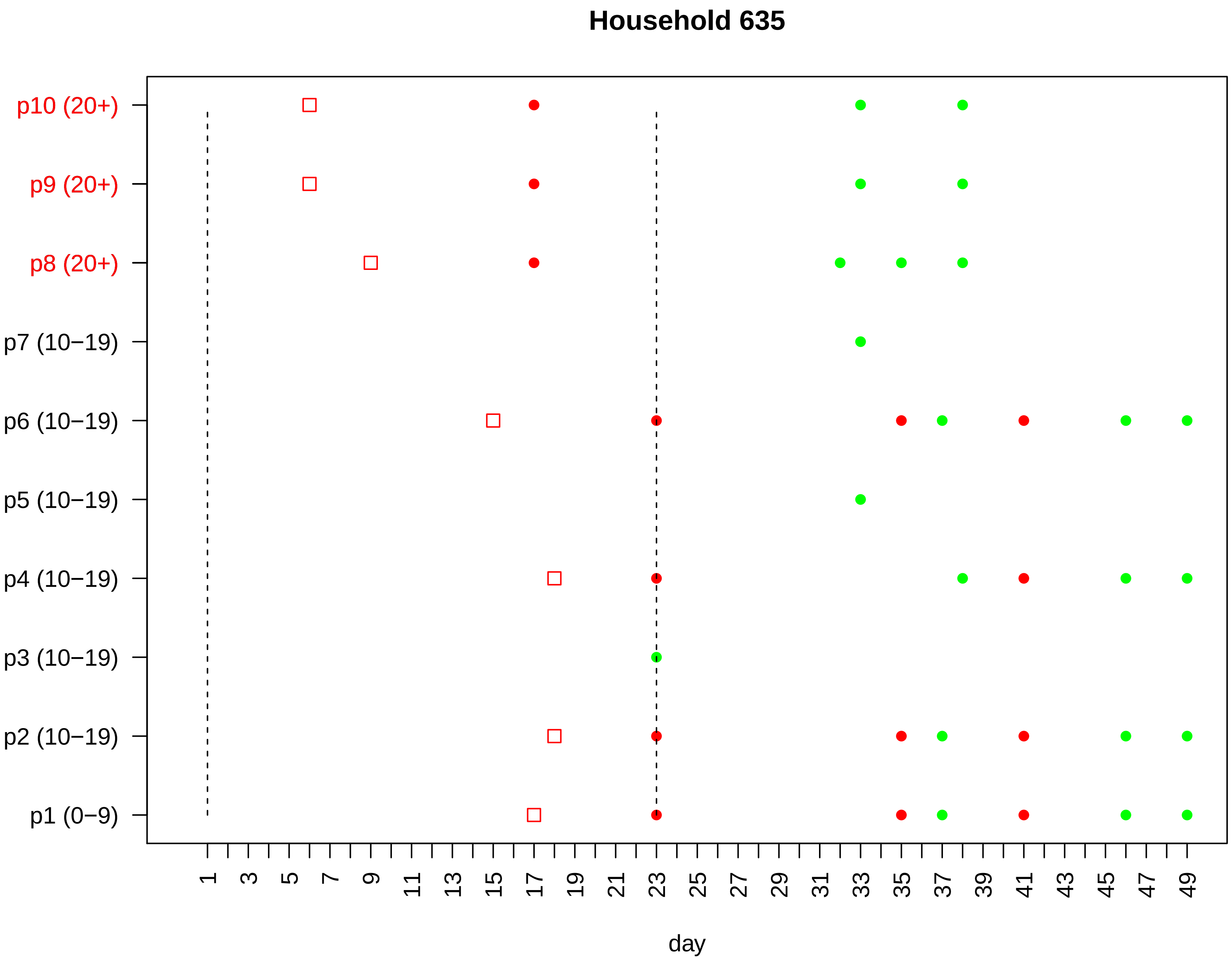
Household 635
p10 (20+)
p9 (20+)
p8 (20+)
p7 (10−19)
p6 (10−19)
p5 (10−19)
p4 (10−19)
p3 (10−19)
p2 (10−19)
p1 (0−9)
1 3 5 7 9 11 13 15 17 19 21 23 25 27 29 31 33 35 37 39 41 43 45 47 49
day

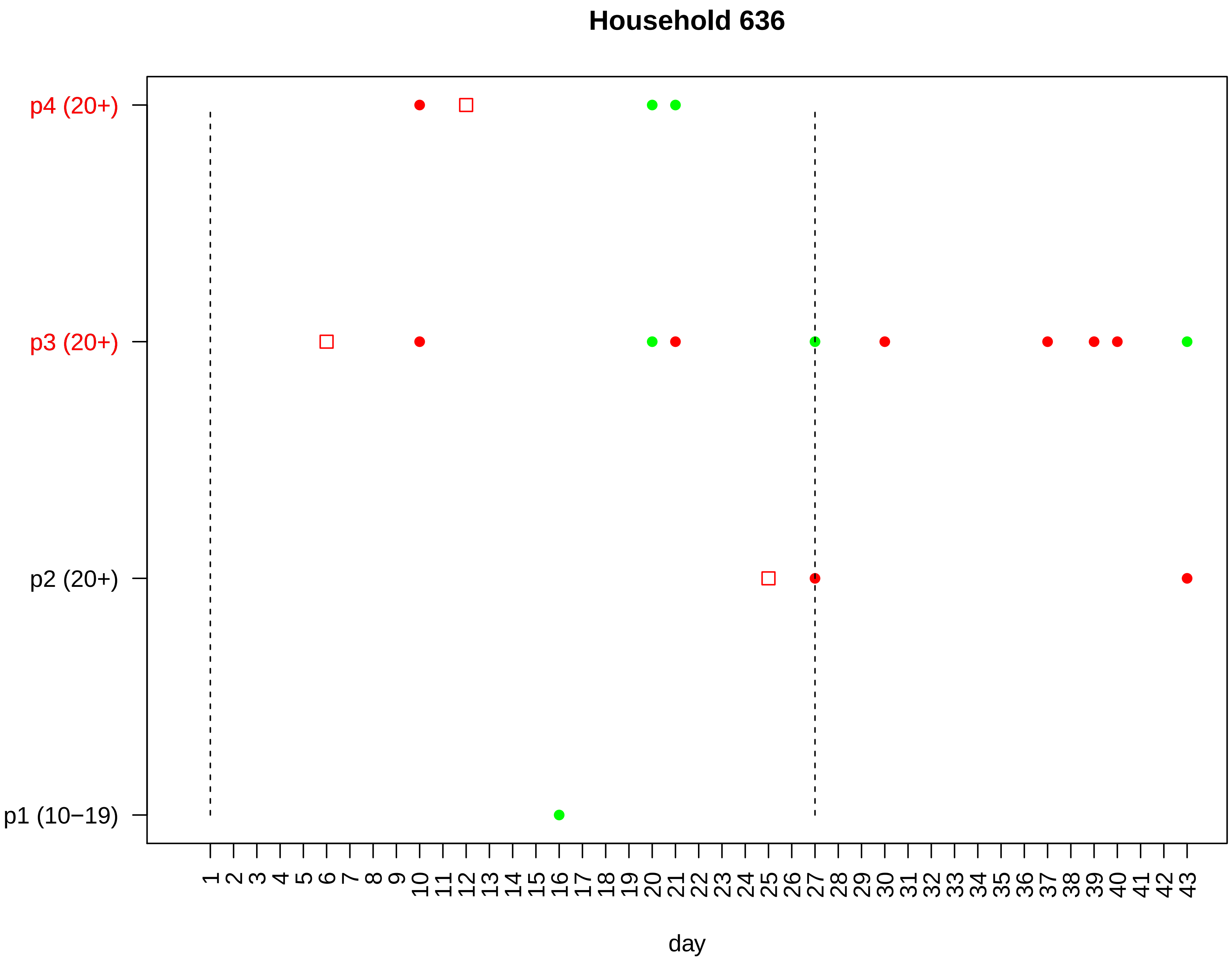
Household 636
p4 (20+)
p3 (20+)
p2 (20+)
p1 (10−19)
1 2 3 4 5 6 7 8 9 10 11 12 13 14 15 16 17 18 19 20 21 22 23 24 25 26 27 28 29 30 31 32 33 34 35 36 37 38 39 40 41 42 43
day

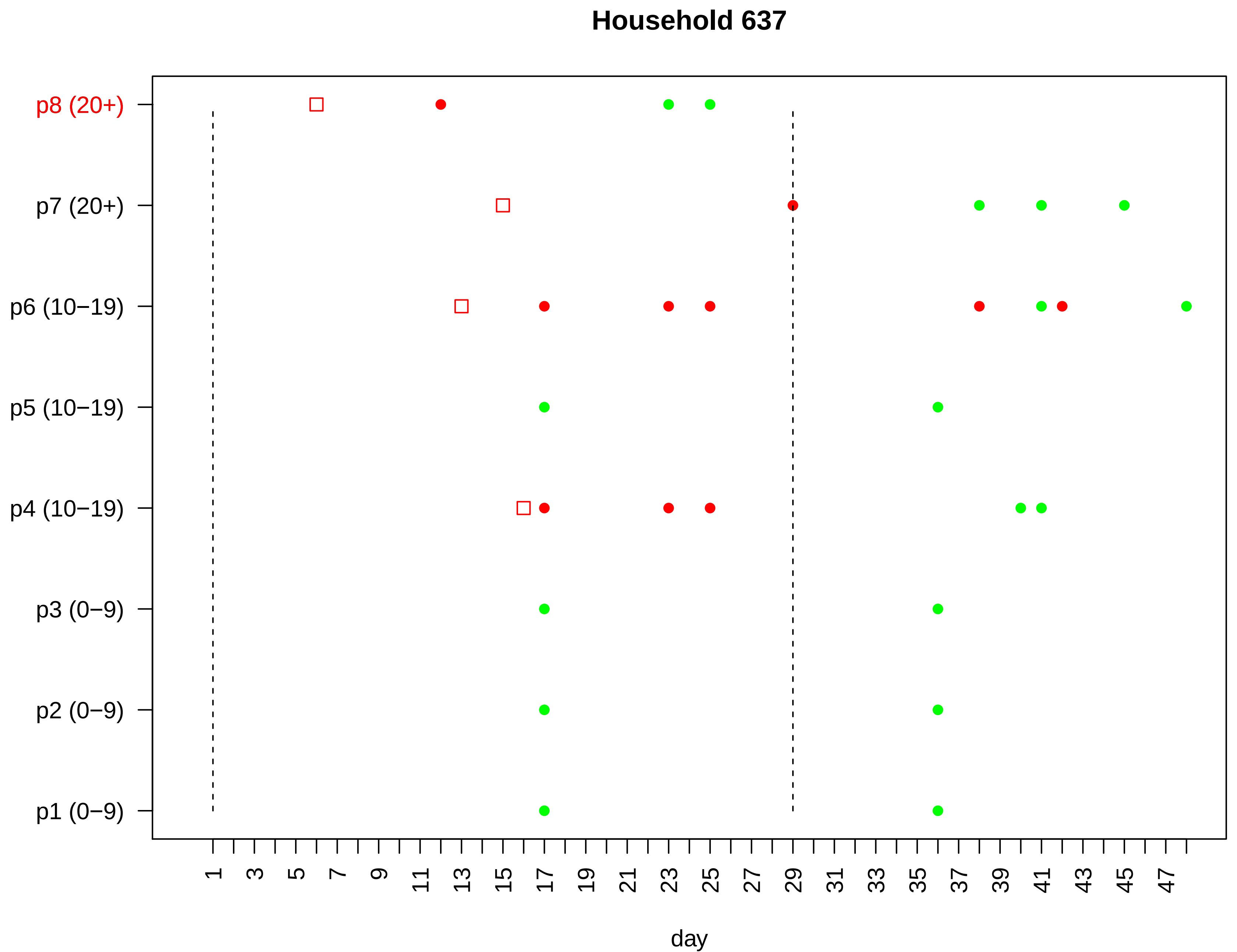

Household 637
p8 (20+)
p7 (20+)
p6 (10−19)
p5 (10−19)
p4 (10−19)
p3 (0−9)
p2 (0−9)
p1 (0−9)
1
3
5
7
9
11
13
15
17
19
21
23
25
27
29
31
33
35
37
39
41
43
45
47
day
