# Supplementary figures and images for: The role of children in the spread of COVID-19: Using household data from Bnei Brak, Israel, to estimate the relative susceptibility and infectivity of children
Source: PLoS Comput Biol. 2021 Feb 11;17(2):e1008559. doi: 10.1371/journal.pcbi.1008559 (PMC7877572; doi:10.1371/journal.pcbi.1008559)

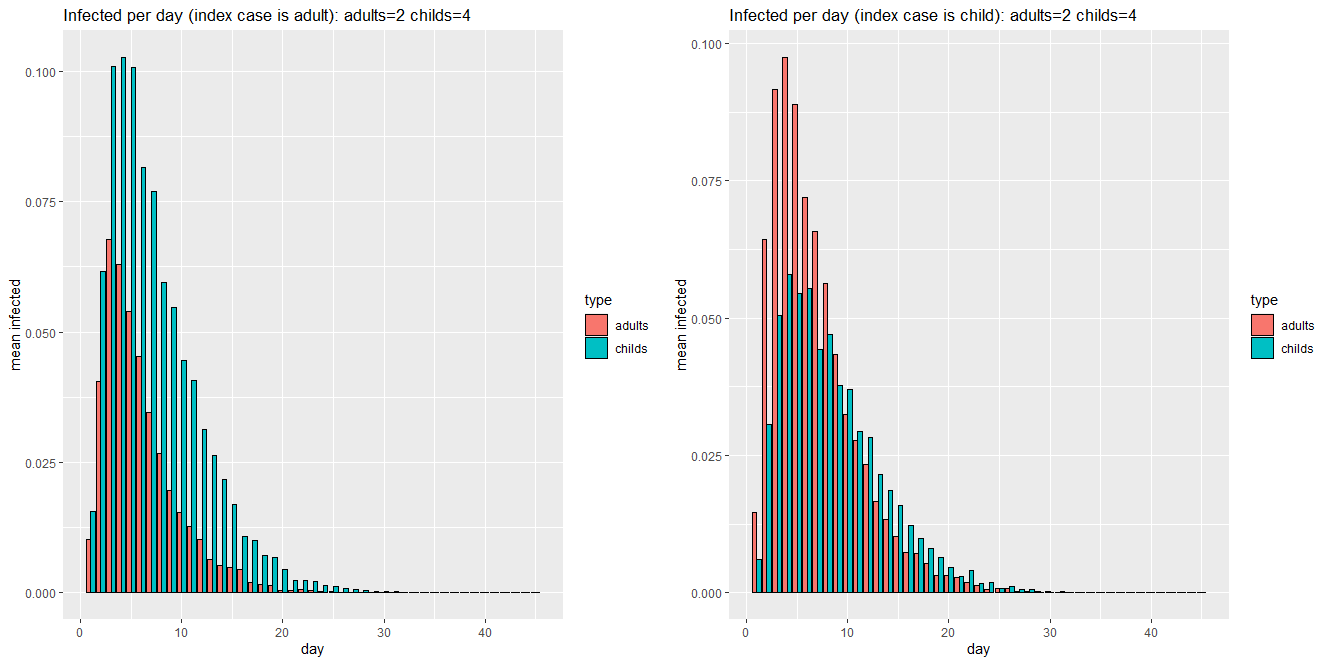

Supplement: S1 Fig — Results of 10,000 simulations of the dynamic model. (TIF) [file pcbi.1008559.s003.tif]

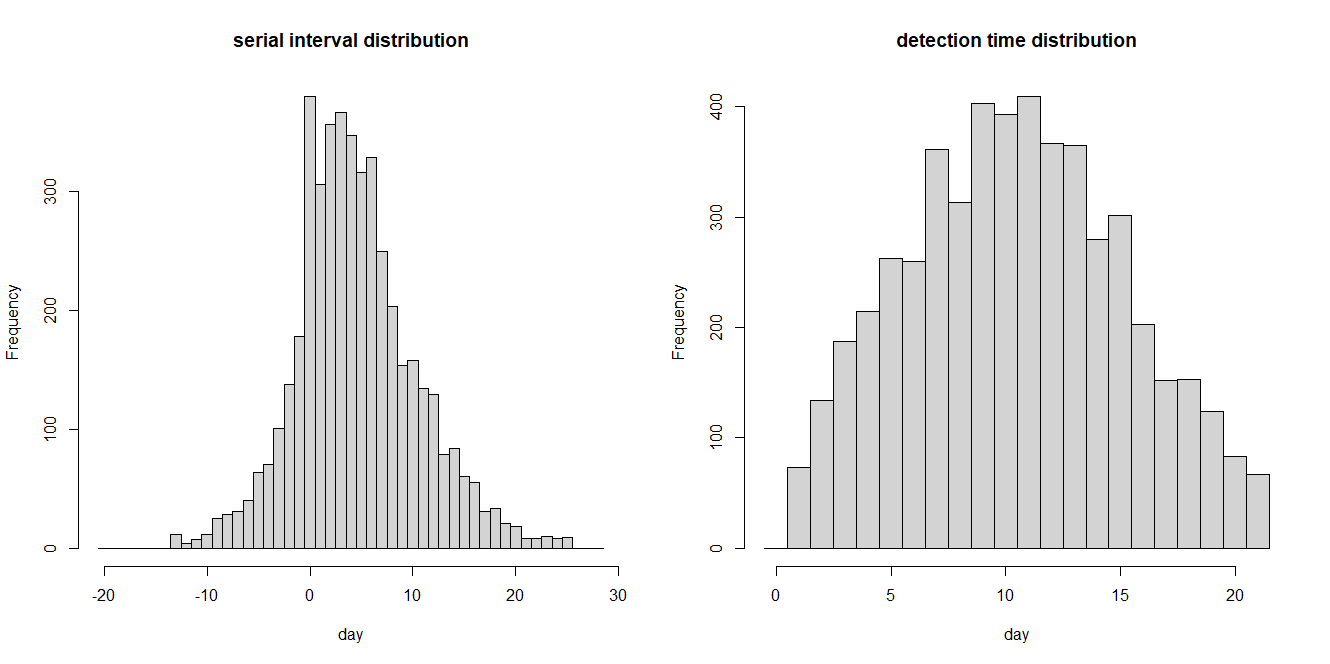

Supplement: S2 Fig — Serial-interval distribution with mean of 4.5 days and Detection-time distribution with mean of ∼10 days. (TIF) [file pcbi.1008559.s004.tif]

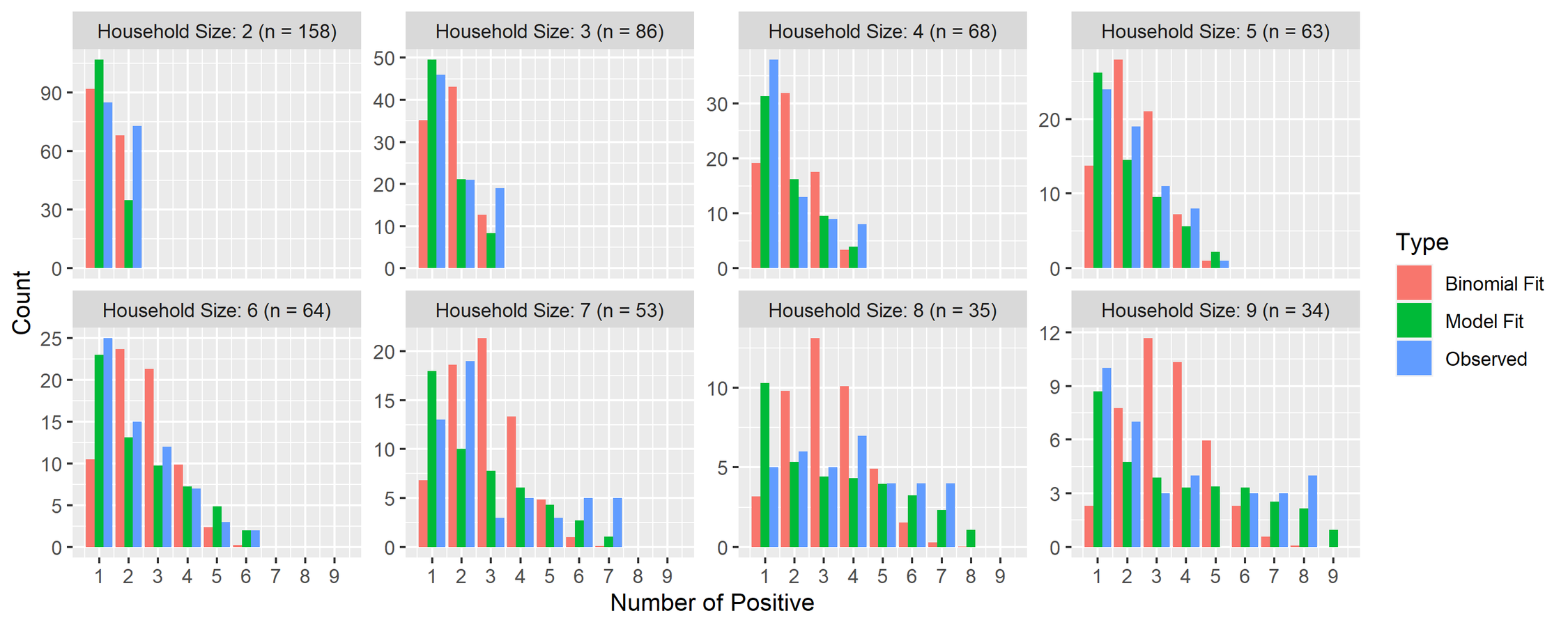

Supplement: S3 Fig — Comparison of the dynamic model and the naive binomial model fit to the data. (TIF) [file pcbi.1008559.s005.tif]

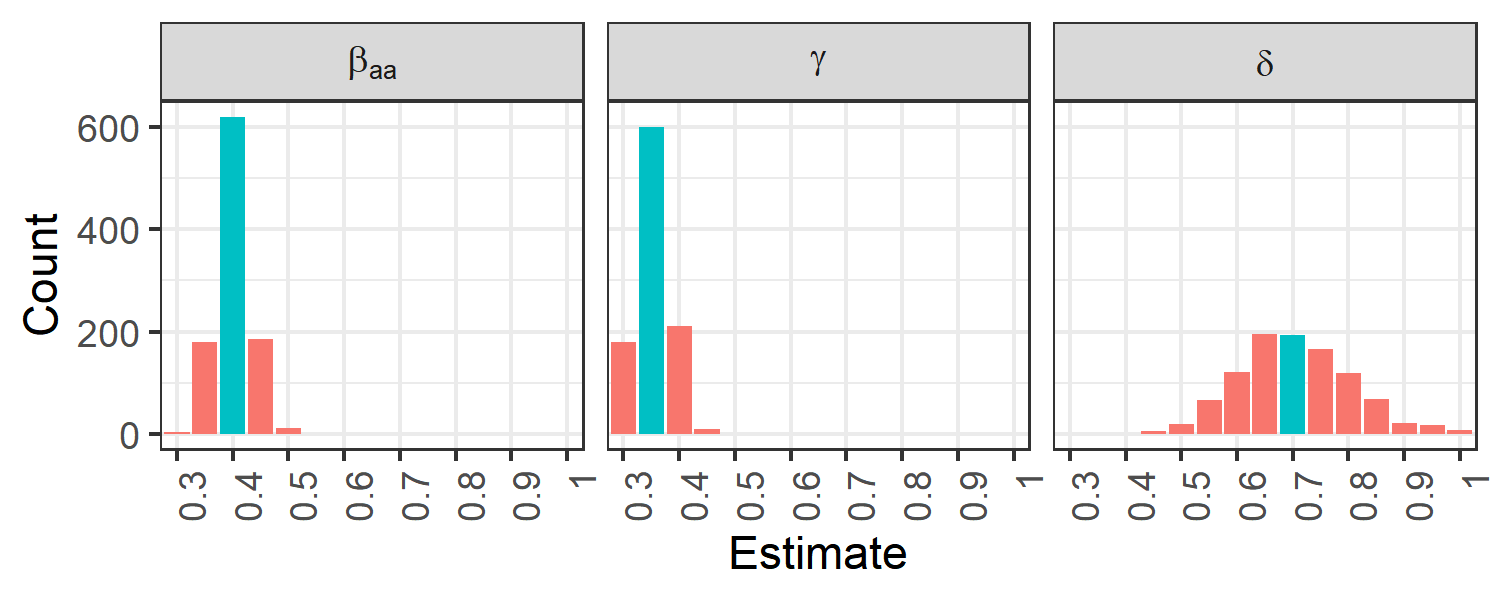

Supplement: S4 Fig — Results of 1000 bootstrap simulations. βaa—the transmission parameter among adults, γ—relative susceptability of children, δ—relative infectivity of children. The blue color indicates the values used to generate the simulated data. (TIF) [file pcbi.1008559.s006.tif]

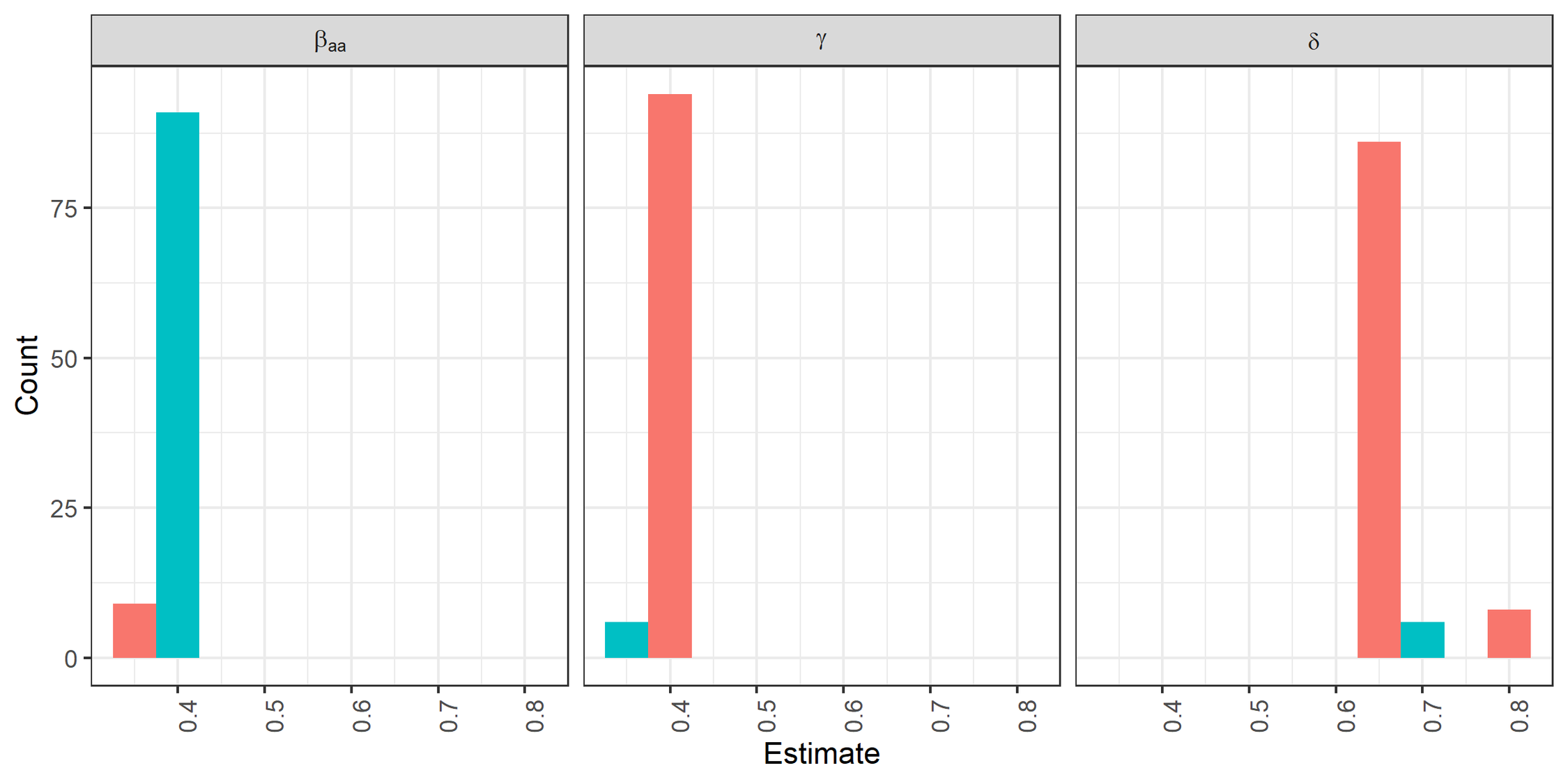

Supplement: S5 Fig — Sensitivity to observed epidemic duration. βaa—the transmission parameter among adults, γ—relative susceptability of children, δ—relative infectivity of children. The blue color indicates the values used to generate the simulated data. (TIF) [file pcbi.1008559.s007.tif]

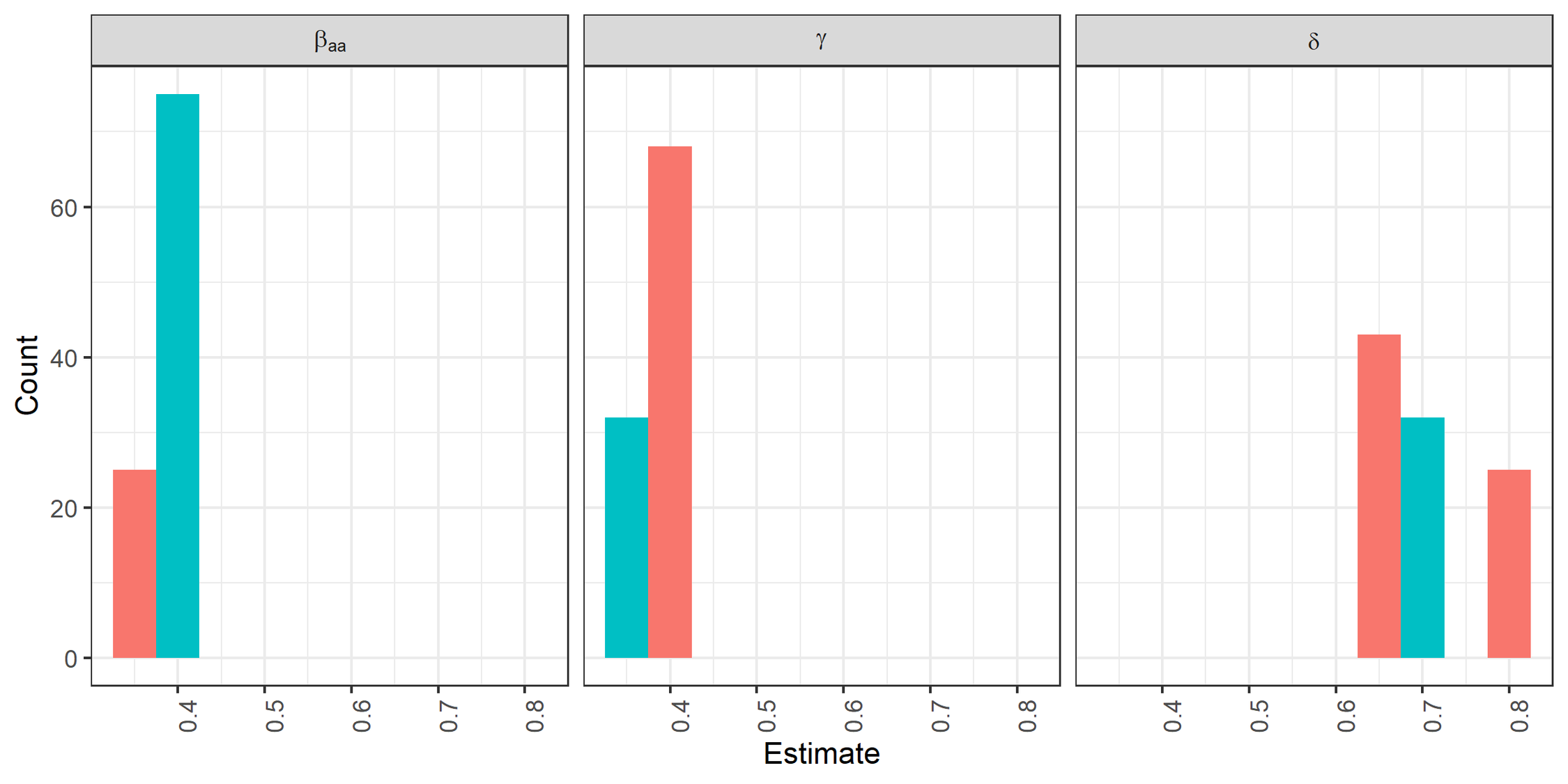

Supplement: S6 Fig — Sensitivity to uncertainty regarding the index case age-group. βaa—the transmission parameter among adults, γ—relative susceptability of children, δ—relative infectivity of children. The blue color indicates the values used to generate the simulated data. (TIF) [file pcbi.1008559.s008.tif]

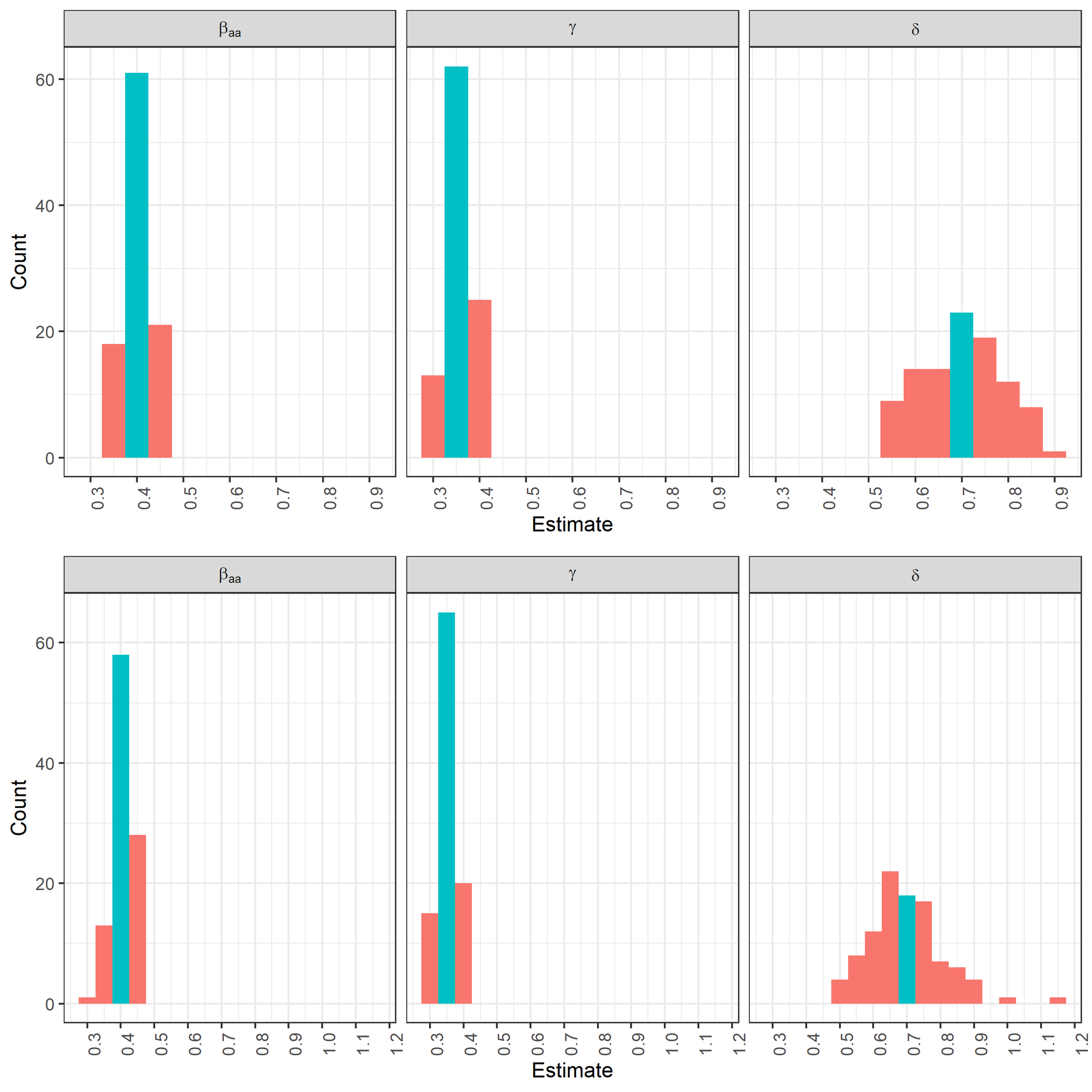

Supplement: S7 Fig — Results for data sets with mean generation-time of 4 days (top row) and mean generation-time of 5 days (bottom row). βaa—the transmission parameter among adults, γ—relative susceptability of children, δ—relative infectivity of children. The blue color indicates the values used to generate the simulated data. (TIF) [file pcbi.1008559.s009.tif]

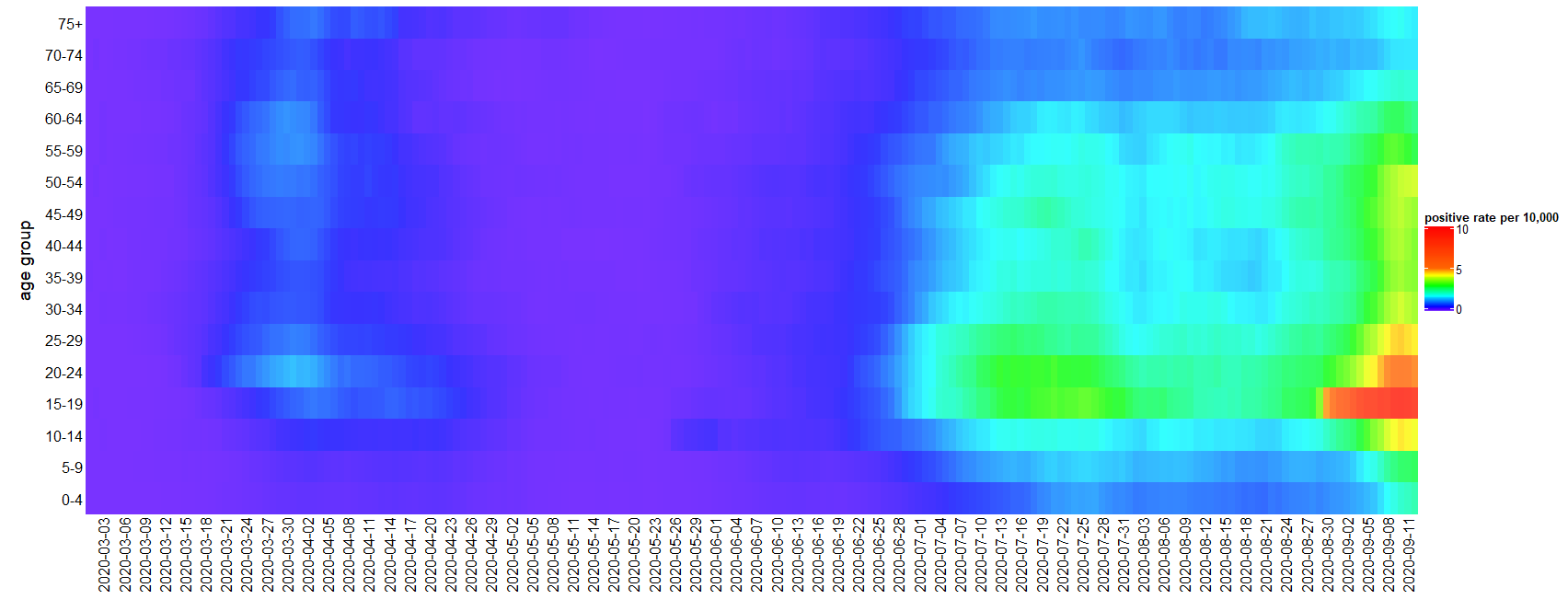

Supplement: S8 Fig — (TIF) [file pcbi.1008559.s010.tif]
